# EspF of Enterohemorrhagic *Escherichia coli* Enhances Apoptosis *via* Endoplasmic Reticulum Stress in Intestinal Epithelial Cells: An Isobaric Tags for Relative and Absolute Quantitation-Based Comparative Proteomic Analysis

**DOI:** 10.3389/fmicb.2022.900919

**Published:** 2022-06-30

**Authors:** Xiangyu Wang, Kaina Yan, Muqing Fu, Song Liang, Haiyi Zhao, Changzhu Fu, Lan Yang, Zhihong Song, Dayong Sun, Chengsong Wan

**Affiliations:** ^1^Department of Gastroenterology, The First Affiliated Hospital of Shenzhen University, Shenzhen Second People’s Hospital, Shenzhen, China; ^2^BSL-3 Laboratory (Guangdong), Guangdong Provincial Key Laboratory of Tropical Disease Research, School of Public Health, Southern Medical University, Guangzhou, China; ^3^Center for Novel Target and Therapeutic Intervention, Institute of Life Sciences, Chongqing Medical University, Chongqing, China; ^4^Genecreate Biological Engineering Co., Ltd., National Bio-industry Base, Wuhan, China; ^5^MRC Toxicology Unit, School of Biological Sciences, University of Cambridge, Cambridge, United Kingdom

**Keywords:** Enterohemorrhagic *Escherichia coli*, EspF, endoplasmic reticulum stress, apoptosis, iTRAQ

## Abstract

There have been large foodborne outbreaks related to Enterohemorrhagic *Escherichia coli* (EHEC) around the world. Among its virulence proteins, the EspF encoded by locus of enterocyte effacement is one of the most known functional effector proteins. In this research, we infected the HT-29 cells with the EHEC wild type strain and EspF-deficient EHEC strain. Via the emerging technique isobaric tags for relative and absolute quantitation (iTRAQ), we explored the pathogenic characteristics of EspF within host cells. Our data showed that the differences regarding cellular responses mainly contained immune regulation, protein synthesis, signal transduction, cellular assembly and organization, endoplasmic reticulum (ER) stress, and apoptosis. Notably, compared with the EspF-deficient strain, the protein processing in the ER and ribosome were upregulated during wild type (WT) infection. Our findings proved that the EspF of Enterohemorrhagic *Escherichia coli* induced ER stress in intestinal epithelial cells; the ER stress-dependent apoptosis pathway was also activated within the host cells. This study provides insight into the virulence mechanism of protein EspF, which will deepen our general understanding of A/E pathogens and their interaction with host proteins.

## Introduction

Enterohemorrhagic *Escherichia coli* (EHEC) is a pathogen of foodborne zoonotic intestinal infectious diseases that spreads through the fecal-oral route (Qadri and Kayali, 1998). EHEC has a variety of serotypes, but only a few of them are related to human diseases. Among these, the serotype O157: H7, first discovered in 1982, has produced outbreaks and epidemics in many countries, especially Northern Europe, Canada (Honish et al., 2017), the United States (Heiman et al., 2015), Argentina, Japan (Kanayama et al., 2015), and China. Symptoms caused by EHEC include abdominal cramps and non-bloody watery diarrhea, which sometimes can develop into bloody diarrhea. The aged people and young children may develop life-threatening diseases, for instance, thrombotic thrombocytopenic purpura and hemolytic uremic syndrome. Currently, there is no effective treatment for EHEC infections. It has strong pathogenicity and lethality, and antibiotic treatment can cause exacerbations. EHEC infection has become a global concern for safety and health issues (Li et al., 2020).

Enterohemorrhagic *Escherichia coli* was classified into attaching and effacing (A/E) pathogens, targeting the mammalian intestines and causing pathological lesions on the apical surface of the host enterocytes (Spears et al., 2006). A/E pathogens consist of EHEC, *Citrobacter rodentium* (CR), and enteropathogenic *Escherichia coli* (EPEC). They all contain the locus of enterocyte effacement (LEE), which carries many toxic genes, including the bacterium’s type III secreted system (T3SS) and effector proteins: EspG, Tir, Map, EspZ, EspH, and EspF (Gaytan et al., 2016). These effector proteins cause significant damage to the host cell, eventually leading to diseases such as diarrhea and inflammation. Through the T3SS, A/E pathogens directly inject the effector proteins into the host cell. These proteins work together to produce characteristic intestinal pathological changes, such as loss of bacterial intestinal microvilli, regulation of actin aggregation, destruction of tight junction barrier, and rearrangement of cell cytoskeleton, ultimately leading to diarrhea diseases (Holmes et al., 2010).

The EspF is one of the most important functional effector proteins encoded by LEE. This protein displays emerging characteristics and becomes a model bacterial effector of multifunctionality. The EspF protein consists of the N-terminal and C-terminal domains. The N-terminus (1–73 aa) is composed of the host cell mitochondrial targeting signal (MTS), the secretion signal and the nucleolar targeting part (NTD); the C-terminus (73–248 aa) contains four proline-rich repeats (PRR) (Mayer, 2001). The EspF protein can inhibit the phagocytosis of macrophages (Tapia et al., 2017), lead to the effacement of host microvilli, remodel the host membrane, and regulate its cytoskeleton (McNamara et al., 2001). EspF can block ribosome synthesis and protein translation by targeting the nucleoli (Dean et al., 2010), regulate DNA mismatch repair (Maddocks et al., 2009), destroy the host cell intermediate fibers, and inhibit sodium-glucose cotransporters, aquaporins, and Na^+^/H^+^ exchanger (Hodges et al., 2008). Our earlier findings showed that the N-terminal portion of the EspF targeted the mitochondria and disrupted its transmembrane potential, destroyed the tight junctions of the epithelial barrier, led to host cell apoptosis (Wang et al., 2017; Xia et al., 2019). Though EspF is multifunctional, the pathogenic pathways and specific regulatory mechanisms that mediate its interaction with the intracellular proteins of host cells are still unclear.

A quantitative proteomic assay combining isobaric tags for relative and absolute quantitation (iTRAQ) and liquid chromatography-tandem mass spectrometry (LC-MS/MS) is developing into a powerful technique to unravel the overall protein changes within host cells (Wiese et al., 2007). At present, the iTRAQ has a wide range of applications in the proteomics (Mertins et al., 2012). At the MS/MS level, it could identify the proteins immediately and conduct relative quantification from peptide fragments and low mass reporter ions.

The superiorities of iTRAQ are the high throughput, high quality, high stability, and little sample limitations compared with other quantitative methods.

In this research, we infected host cells with the EHEC (WT) and EspF-deficient (Δ*espF*) strains. Via iTRAQ, we captured the differentially expressed proteins from HT-29 cells after infecting WT and Δ*espF* strains. These differential proteins were annotated by bioinformatics tools. They were involved in many KEGG pathways. Furthermore, we constructed protein-protein interaction networks after the EHEC WT and Δ*espF* infection. In general, this work compared complete cellular protein alterations induced by EHEC WT and Δ*espF* strain through iTRAQ based proteomic analysis. It will deepen our understanding of the EspF and contribute to the prevention and control of EHEC O157:H7 infection.

## Materials and Methods

### Cell Culture and Bacterial Strains

The EHEC O157:H7 EDL 933 (WT) and its isogenic strains lacking the *espF* gene (Δ*espF*, with kanamycin resistance), complemented strain (Δ*espF*/*pespF*, with kanamycin and chloramphenicol resistance) were constructed in the previous work (Wang et al., 2017). The *espF* gene expression of the complemented strain was induced by L-arabinose. The bacterial strains were grown in Luria Bertani media (Oxiod #LP0137, Basingstoke, United Kingdom) at 37°C at 200 rpm in a constant-temperature, oscillating shaker with appropriate antibiotics: 100 μg/ml kanamycin (Solarbio #K8020, Beijing, China) for Δ*espF* and Δ*espF*/*pespF*; 10 μg/ml chloramphenicol (Solarbio #C8050, Beijing, China) and 2 mg/ml L-arabinose (Solarbio #L8060, Beijing, China) for Δ*espF*/*pespF* strain.

HT-29, Vero, Hela and Caco-2 cells were preserved in our laboratory. Briefly, they were grown in RPMI-1640 (Gibco #C11875500BT, New York, NY, United States) or DMEM medium (Gibco # C11995500BT, New York, NY, United States) containing 10% fetal bovine serum (Gibco #10270-106, New York, NY, United States) and 1% penicillin/streptomycin (Gibco #15140122, New York, NY, United States). The cells were cultured in tissue culture plates at 37°C under humidified 5% CO_2_ prior to infection.

### The Isobaric Tags for Relative and Absolute Quantitation and Liquid Chromatography-Tandem Mass Spectrometry Process

The HT-29 cells were infected with WT, Δ*espF* strains at MOI (multiplicity of infection) of 100:1 for 6 h at 37°C and 5% CO_2_. Then the WT-, Δ*espF*- and mock-infected cell samples were collected via the cell scraper, centrifuged, and washed with PBS (Gibco #10010023, New York, NY, United States) twice. The cell samples were dissolved with 200 μl TEAB dissolution buffer and cracked by the ultrasonic. Then they were centrifuged at 12000 r/min for 20 min. The clear supernatant was transferred into a new tube and added cold acetone containing 10 mM DTT (Thermo #20290, MA, United States) for about 2 h, followed by centrifugation. The precipitate was collected and mixed with 800 μl cold acetone at 56°C. The samples were centrifuged at 12000 r/min for 20 min at 4°C again and dried. Total protein concentration was measured by the BCA protein assay kit (Beyotime # P0012S, Shanghai, China).

Samples were labeled with iTRAQ multiplex kit (AB Sciex, United Kingdom) as follows: iTRAQ 113 and 114 for mock-infected samples; iTRAQ 115 and 116 for WT strain infected samples; iTRAQ 117 and 118 for Δ*espF* strain infected samples. We mixed all the labeled samples in equal quantities. Next, the high-performance liquid chromatography (HPLC) system (DINOEX Ultimate 3000 BioRS, Thermo, United States) was employed to fractionate the labeled samples. The LC-MS/MS analysis was conducted on the Triple TOF 5600 plus system (AB SCIEX, United States).

### Bioinformatics Analysis

The identified protein sequences were annotated by the Gene Ontology Terms (Gene Ontology Consortium, 2021) (GO^1^), Clusters of Orthologous Genes (Galperin et al., 2021) (COG^2^) and Kyoto Encyclopedia of Genes and Genomes (Kanehisa, 1997) (KEGG^3^) to predict the possible function and classify them functionally. We applied hypergeometric tests to perform GO and KEGG enrichment to discover differentially expressed biomarkers in each group. The R language was applied to draw all other figures in the research.

### RNA Extraction and Real-Time PCR Analysis

The HT-29 cells were grown in tissue culture plates (approximately 10^6^ cells per well) overnight. The cells were infected with WT, Δ*espF* or Δ*espF/pespF* strains (at MOI of 100:1) for 6 h. The total RNA of the treated cells was extracted using TRIzol Reagent (Invitrogen #15596026, CA, United States). We used the primescript RT reagent kit (Takara # RR036A, Dalian, China) to remove genomic DNA and perform pre-cDNA synthesis in RT-PCR.

We detected the amplification of PCR products via the TB GreenTM premix Ex (Takara #RR820B, Dalian, China). The primers Bip-F/R, Atf6-F/R, Chop-F/R, Caspase12-F/R, Caspase 9-F/R, Caspase3-F/R, GAPDH-F/R, RT-PCR were designed and synthesized to detect changes in mRNA expression (Table 1). The RT-PCR process was as follows: 95°C 30 s; followed by 40 cycles of 95°C for 5 s, 60°C for 30 s, and 95°C for 5 s, 60°C 1 min; then a final extension step of 72°C for 30 s.

**TABLE 1:** Sequences of the primers used in this study.

Primers	Sequences (5′→3′)
Bip-F	CTGGGTACATTTGATCTGACTGG
Bip-R	GCATCCTGGTGGCTTTCCAGCCATTC
Atf-F	TCCTCGGTCAGTGGACTCTTA
Atf-R	CTTGGGCTGAATTGAAGGTTTTG
Chop-F	CAGAACCAGCAGAGGTCACA
Chop-R	ACCATTCGGTCAATCAGAGC
Caspase12-F	ACCGTAACTGCCAGAGTCTGAA
Caspase12-R	ACCTTGCAAGAGCCGACCAT
Caspase9-F	GATCAGATCGGGAATTGCAA
Caspase9-R	AGGTGAGGAATTGGCTCCTT
Caspase3-F	AACGATATCGCGGGCCCGAA
Caspase3-R	GGAGGTGCCTTGAGCTAATT
GAPDH-F	AGCTCACTGGCATGGCCTTC
GAPDH-R	CGCCTGCTTCACCACCTTCT

### Western Blot Analysis

The HT-29 cells were seeded in 10 cm diameter tissue culture dishes (approximately 1 × 10^7^ cells per dish) overnight. Then the cells were infected with WT, Δ*espF*, and Δ*espF/pespF* strains (at MOI of 100:1) for 6 h. Cells were washed with ice-cold PBS and then lysed in RIPA lysate (Beyotime #P0013B, Shanghai, China), and added PMSF protease inhibitor (Beyotime #ST506-2, Shanghai, China) to collect protein. The cell lysates were centrifugated at 13,000 rpm for 10 min at 4°C and the protein concentration was measured via the BCA protein assay kit (Beyotime #P0012S, Shanghai, China). Equivalent amounts of protein from each sample were separated by SDS-PAGE and electro-transferred to PVDF membrane (Millpore #IPVH00010/ISEQ00010, MA, United States).

The membrane was blocked with 3% bovine serum albumin (Beyotime #ST023, Shanghai, China) and incubated in primary antibody at 4°C overnight. The primary antibody included: Phospho-eIF2α (CST #Ser51, 1:1000) Rabbit mAb (CST #3398S, 1:1000, Boston, MA, United States), eIF2α Rabbit mAb (CST #5324S, 1:1000, Boston, MA, United States), Phospho-JNK (CST #Thr183/Tyr185, 1:1000, Boston, MA, United States) Rabbit mAb (CST #4668S, 1:1000), JNK Antibody (CST #9252S, 1:1000, Boston, MA, United States), Cleaved Caspase-3 (Asp175) (5A1E) Rabbit mAb (CST #9664S, 1:1000, Boston, MA, United States), BiP Antibody (CST #3183S, 1:1000, Boston, MA, United States), Bax Antibody (CST #2772S, 1:1000, Boston, MA, United States), CHOP (CST #L63F7, 1:1000, Boston, MA, United States) Mouse mAb (CST #2895S, 1:1000), IRE1α (CST #14C10, 1:1000, Boston, MA, United States) Rabbit mAb (CST #3294S, 1:1000) from Cell Signaling Technology (United States); ATF6 Rabbit polyclonal Antibody (Abcam #ab37149, 1:1000, Cambridge, United Kingdom), DDIT3 Mouse monoclonal Antibody (Abcam #ab11419, 1:1000, Cambridge, United Kingdom), Caspase-12 Rabbit polyclonal Antibody (Abcam #ab62484, 1:1000, Cambridge, United Kingdom), Cleaved Caspase-9 Rabbit monoclonal Antibody (Abcam #ab2324, 1:1000, Cambridge, United Kingdom) from Abcam (United Kingdom); GAPDH Rabbit polyclonal Antibody (Proteintech #10494-1-AP, 1:10000, Chicago, IL, United States), β-actin Rabbit polyclonal Antibody (Proteintech #20536-1-AP, 1:10000, Chicago, IL, United States), α-tubulin Rabbit polyclonal Antibody (Proteintech #11224-1-AP, 1:10000, Chicago, IL, United States) from Proteintech (United States). The membranes were washed with TBST and then incubated with horseradish peroxidase (HRP) conjugated rabbit (Bioss #bs-0295G, Beijing, China) or mouse (Bioss #bs-0296G, Beijing, China) secondary antibody. The signals were collected via the hypersensitive chemiluminescence (ECL) reagent (Bioworld #BLH01S100CN, MI, United States). Then the target band was photographed by the Tanon MP software.

### The Ca^2+^ Release Experiments

The HT-29 cells were grown in tissue culture dishes (approximately 1 × 10^6^ cells per dish) overnight. Then the cells were infected with WT, Δ*espF* and Δ*espF/pespF* strains (at MOI of 100:1) for 6 h. A Fluo-4 AM kit (Yeasen #HB180709, Shanghai, China) was used to measure the cytosolic Ca^2+^ concentration within host cells. After washing, the Fluo-4 AM working solution was added to each well, followed by incubation at 5% CO^2^ and 37°C for 30 min. Then, cells were gently washed three times by HBSS and stained with Hoechst33342 at room temperature for 10 min. The fluorescent signals were visualized on the laser confocal microscope FV1000 (Olympus, Japan) with the excitation wavelength of 494 nm and emission wavelength of 516 nm.

### Cell Apoptosis Detection

The HT-29 cells were grown in tissue culture dishes (approximately 1 × 10^7^ cells per dish) overnight. Then the cells were infected with WT, Δ*espF*, and Δ*espF/pespF* strains (at MOI of 100:1) for 6 h. The cells were trypsinized, collected, and resuspended in PBS (approximately 1 × 10^6^ cells per tube). We used the annexin V-FITC kit (Keygen Biotech #KGA107-50T, Nanjing, China) to stain apoptotic cells in the dark. The apoptotic cells were detected immediately using the flow cytometry (BD FACScan, United States).

### Immunofluorescence Assay

Vero cells were grown in tissue culture plates (approximately 10^6^ cells per well) overnight. The cells were infected with WT, Δ*espF* or Δ*espF/pespF* strains (at MOI of 100:1) for 6 h. Cells were washed with PBS, fixed with 4% PFA (Leagene #DF0135, Beijing, China), added with 0.1% TritonX-100 (Sigma-Aldrich #T9284, St Louis, MO, United States) and blocked with 10% goat serum (Boster #AR1009, Wuhan, China). Each well was incubated with Bip Rabbit Polyclonal antibody (Proteintech #11587-1-AP, 1: 300, Chicago, IL, United States) overnight at 4°C, then incubated with goat anti-rabbit IgG Alexa 488 (Proteintech #SA00013-2, 1:500, Chicago, IL, United States) for 1 h. The samples were washed with PBS (Gibco, United States) and stained for nucleus using DAPI (Solarbio #C0065, Beijing, China). The fluorescent signals were visualized on the laser confocal microscope FV1000 (Olympus, Japan).

The *espF* gene of EHEC O157: H7 EDL933 was cloned into the eukaryotic expression vector pEGFP-N1 to construct the pEGFP-DH5α-EspF plasmid. Hela cells have good cell morphology and are commonly used in organelle localization experiments. Hela cells were grown in tissue culture plates (approximately 10^6^ cells per well) overnight.

Hela cells were transfected via the pEGFP-DH5α and the pEGFP-DH5α-EspF plasmids. After 24 h, actinomycin (15 μg/ml) was added. After 48 h, the endoplasmic reticulum, nucleoli were stained by ER-tracker red (Beyotime #C1041, Shanghai, China), Hochest33258 (AAT Bioquest #17525, CA, United States). The localization of plasmids within host cells were traced via the FV1000 (Olympus, Japan).

### Transmission Electron Microscopy Analysis

The Caco-2 cells were cultured in tissue culture plates (approximately 10^6^ cells per well) overnight. The cells were infected with WT, Δ*espF*, or Δ*espF/pespF* strains (at MOI of 100:1) for 6 h. Then the infected cells were washed and fixed by 2.5% glutaraldehyde solution (Servicebio #G1102, Wuhan, China) at 4°C for 4 h. Then, the cells were harvested with centrifugation at low-speed centrifugation and coated with 1% agarose. Subsequently, cells were fixed by 1% acetic acid 0.1M phosphate buffer at room temperature for 2 h. The samples were rinsed again, dehydrated with a graded alcohol, and embedded in plates containing pure 812 embedding agent (SPI #90529-77-4, United States). An ultra-thin slicer (Leica UC7, Germany) was used to cut 60–80 nm ultrathin slice. The ultrathin slices were double stained with uranium and lead and visualized on the HT7700 transmission electron microscope (Hitachi, Japan).

### Data Analyses

We applied Protein Pilot Software v4.5 to analyze the original MS/MS file data. The Paragon algorithm (Shilov et al., 2007) was integrated into Protein Pilot to search in the Uniprot Homo sapiens (154724 items) database. The qualified proteins (unique peptide ≥ 1, unused value ≥ 1.3) were considered for further analysis. The results were presented as the mean ± SD of at least three independent experiments performed. Statistical analyses were performed via the SPSS 19.0 software (SPSS, United States). We applied the student’s *t*-test, one-way ANOVA and then performed Bonferroni *post hoc* test for multiple comparisons to compare the values in different groups. The *p*-value < 0.05 was considered significant.

## Results

### Differentially Expressed Proteins Identified in the Study

In this study, 3175 distinct proteins (unused value ≥ 1.3, confidence ≥ 95%) were identified via the iTRAQ (Smith et al., 2015). In total, using a strict cutoff value of 1.5-fold for expressed variation (Deutsch et al., 2014), we detected 145, 230, and 229 proteins differentially expressed in the sample pairs Δ*espF* versus uninfected, Δ*espF* versus WT, and WT versus uninfected (*p* < 0.05), respectively. Supplementary Material reported more details of protein identification (see Supplementary Figures 1a–c).

According to the criteria for defining differentially expressed proteins (fold change ratio ≥ 1.5 and *p* ≤ 0.05), the number of downregulated and upregulated proteins are shown in Table 2. The detailed identification and quantification of each regulated protein was showed in Supplementary Table 1. The COG, KEGG function analysis of differentially expressed proteins within host cells after infection were displayed in the Supplementary Table 2. In our study, two biological replicates among WT-infected, Δ*espF*-infected, and uninfected groups were well-mixed during sampling. And we performed three technical replicates to improve the reliability of our data. The coefficient of variation (CV) analysis was performed to verify the repeatability of technical replicates (see Supplementary Figure 1d).

**TABLE 2:** Differentially expressed proteins identified in the study.

Sample pairs	Δ*espF*/Control	WT/Control	Δ*espF*/ WT
Quantified	3,125	3,128	3,126
Upregulated	80	214	22
Downregulated	65	15	208
Total difference	145	229	230

### Bioinformatics Analysis of the Host Cell Proteome

We adopted several function annotation analyses for identified proteins and function enrichment analyses for differentially expressed proteins to explore more essential proteins and pathways.

#### Gene Ontology Annotation

**FIGURE 1:**
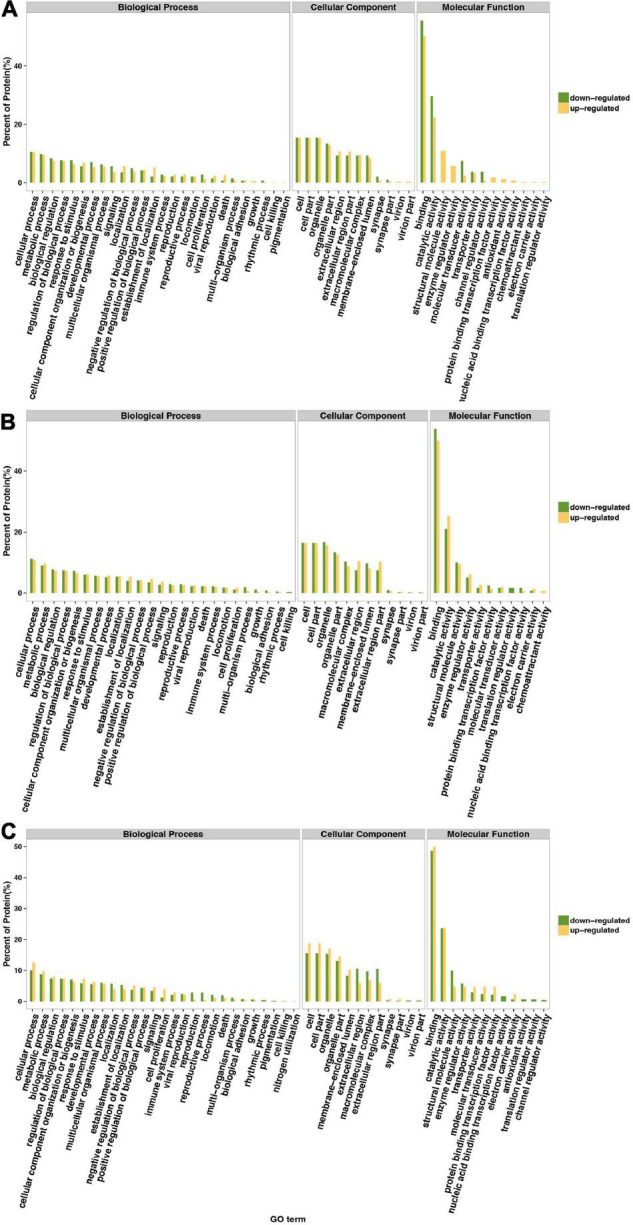
Gene Ontology classification of upregulated and downregulated differentially expressed proteins. The green bar indicates downregulated differentially expressed proteins; the yellow bar indicates upregulated differentially expressed proteins. **(A)** Compared to the control group, the differentially expressed proteins within host cell after WT infection. **(B)** Compared to the control group, the differentially expressed proteins within host cell after Δ*espF* infection. **(C)** Compared to the WT infection, the differentially expressed proteins within host cell after Δ*espF* infection.

Gene Ontology annotation comparison was applied to clarify the features of total altered proteins in HT-29 cells treated by Δ*espF* or WT infection, which might associate with pathogenicity and virulence. The corresponding GO function of each protein is shown in Supplementary Table 3. To analyze the function of differential proteins more clearly, we performed an independent functional annotation analysis of differentially up- and downregulated proteins and compared the altered proteins within WT vs. control, Δ*espF* vs. control and Δ*espF* vs. WT groups respectively. The comparison of the GO term annotations for the differentially expressed proteins in HT-29 cells infected with the WT strain or control group is shown in Figure 1A (Δ*espF* or control group is shown in Figure 1B, and Δ*espF*, or WT group is shown in Figure 1C). GO annotation comparison was conducted to clarify the features of total altered proteins in HT-29 cells induced by WT or Δ*espF* infection (see in Figure 1C), which might be associated with virulence and pathogenicity.

From the Figure 1C, these 229 differentially expressed proteins were categorized into biological processes, cellular components, and molecular functions according to their annotation. The most prevalent biological processes were cellular process, metabolic process, regulation of biological process, cellular component organization or biogenesis and response to stimulus. The most prevalent cellular components were located in the cell, cell part, organelle and organelle part. The most predominant molecular function was binding, catalytic activity, structural molecule activity, enzyme regulator activity.

#### Kyoto Encyclopedia of Genes and Genomes Pathways Analysis

**FIGURE 2:**
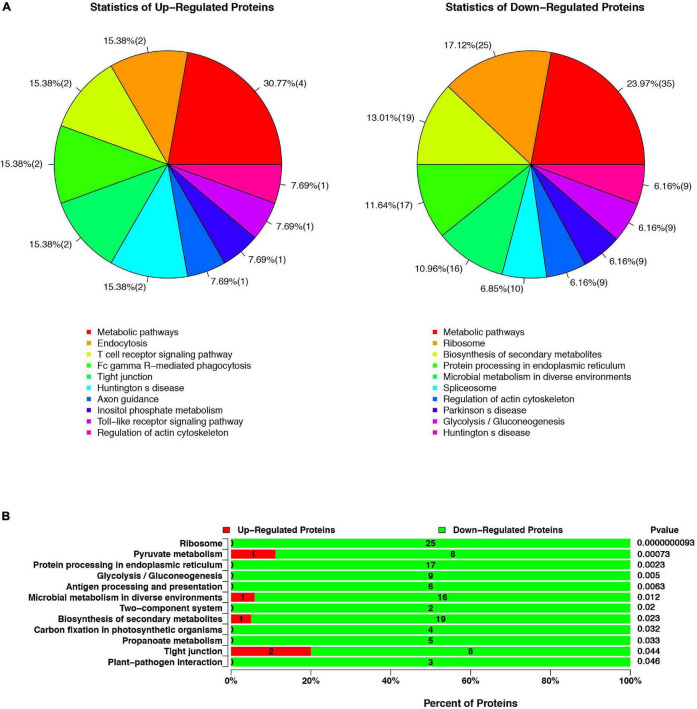
The KEGG pathway analysis of up- or downregulated proteins in cells infected with Δ*espF* respect to cells infected with WT strains. **(A)** Compared with the WT infection, the top ten functions of KEGG pathways from the analysis of up- and downregulated proteins after the Δ*espF* infection. **(B)** Compared with the WT infection, KEGG pathway enrichment analysis after Δ*espF* infection. The green bar indicates downregulated expressed proteins; the red bar indicates upregulated expressed proteins.

All proteins were sub-categorized into 243 KEGG classifications. Figure 2A shows a pie chart of the top 10 functions of KEGG pathways from the analysis of up- and downregulated proteins in cells infected with Δ*espF* respect to cells infected with WT strains. The three pathway’s metabolic function types (metabolic pathways, regulation of actin cytoskeleton, Huntington’s disease) were in all the up and down differential proteins from Figure 2A. It is worth noting that the biological processes involved in endocytosis, T cell receptor signaling pathway, and Fc gamma R-mediated phagocytosis were underscored in Δ*espF* infection, while protein processing in the ER and ribosome were highlighted during WT infection. In order to figure out the function related to altered proteins, we applied KEGG pathway enrichment analysis to reveal the enriched pathways of the considerably altered proteins (Yang et al., 2019). A hypergeometric distribution based on *p*-value was performed to determine the significantly enriched pathways. The KEGG pathway enrichment analysis of up- and downregulated proteins in cells infected with Δ*espF* respect to cells infected with WT strains are shown in Figure 2B. Compared with the Δ*espF* infection, several pathways were more up-regulated after WT infection, such as Ribosome (*p*-value = 9.3 × 10^–9^), Protein processing in the ER (*p*-value = 2.3 × 10^–3^), Antigen processing and presentation (*p*-value = 6.3 × 10^–3^), Biosynthesis of secondary metabolites (*p*-value = 2.3 × 10^–2^), and tight junction (*p*-value = 4.4 × 10^–2^).

#### The Protein–Protein Interaction Analysis

The protein–protein interaction (PPI) analysis is uniformly evaluated based on credibility scores via STRING database (Szklarczyk et al., 2019). This project used the medium credibility of 0.4 (including 0.4) as the threshold to screen the interaction relationship. Supplementary Table 4 shows the detailed results. Cytoscape 3.4 was applied to visualize the network. The node means the protein, and the edge means the interaction relationship between the two nodes. Proteins with higher connectivity than others in the network are called “hubs,” which might play a vital role in the network regulation. The connectivity of each node is evaluated by Degree (Deg), which means the total number of edges connected to this node. The greater the value of Deg, the greater the importance of this node in the network.

**FIGURE 3:**
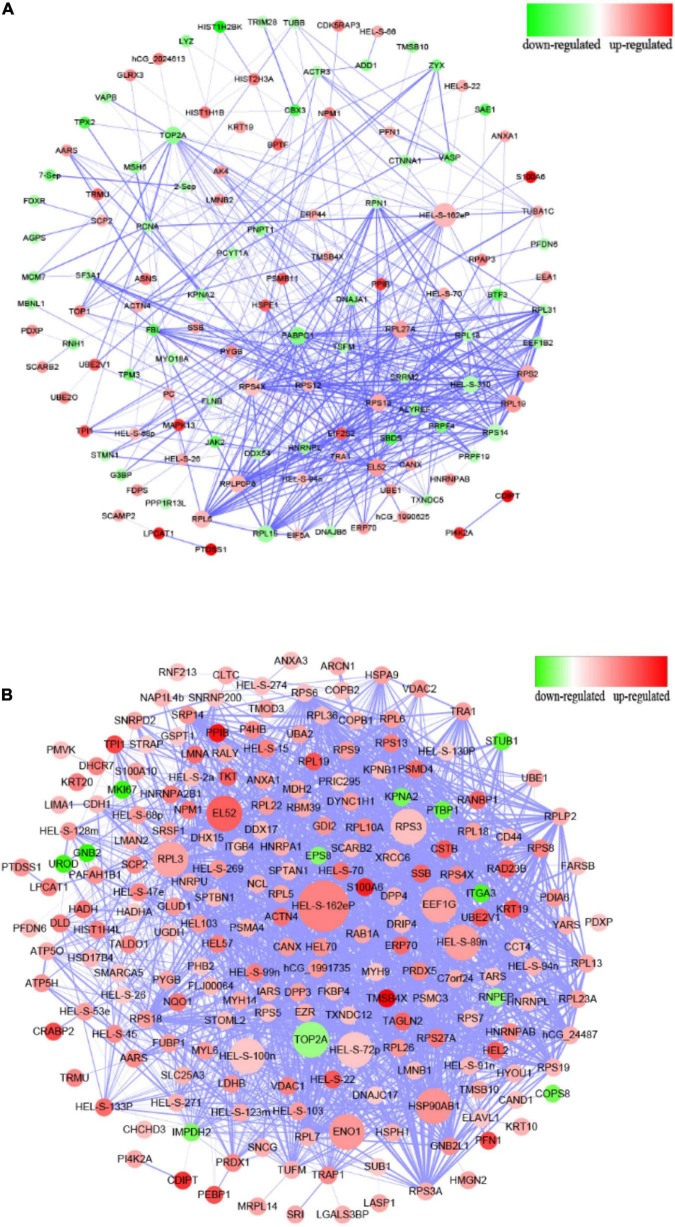
Protein–protein interaction analysis of differentially expressed proteins. **(A)** PPI interaction from the analysis of up- and downregulated proteins comparing the Δ*espF* infected cells against the control cells. **(B)** PPI interaction from the analysis of up- and downregulated proteins comparing the WT infected cells against the control cells. Circular nodes represent the differentially expressed proteins. The red node indicates the upregulation of proteins, while the green node indicates the downregulation. The size of node is proportional to the interaction degree. The interacting proteins are connected by edges. The higher the credibility of the interaction between proteins, the thicker the connection between the nodes.

Compared to the control group, the PPI network diagram was drawn to clarify the features of total altered proteins in HT-29 cells induced by Δ*espF* infection (Figure 3A) and WT infection (Figure 3B). There are 123 nodes in Figure 3A, which form 478 interaction relationships via Δ*espF* infection. PPI analysis revealed the following molecular hubs: Glyceraldehyde-3-phosphate dehydrogenase (Deg:33), ribosomal protein S2 (Deg:27), Epididymis luminal secretory protein 52 (Deg:26), DNA topoisomerase 2-alpha (Deg:26), acidic ribosomal protein P0 (Deg:25), ribosomal protein S14 (Deg:25), ribosomal protein S13 (Deg:24), Polyadenylate-binding protein (Deg:23), ribosomal protein L27a (Deg:22), and ribosomal protein S4 (Deg:22).

There are 213 nodes (Figure 3B), which form 1,882 interaction relationships via WT infection. PPI analysis revealed the following molecular hubs: Glyceraldehyde-3-phosphate dehydrogenase (Deg:77), Epididymis luminal secretory protein 52 (Deg:69), Epididymis luminal protein 33 (Deg:67), Epididymis secretory sperm binding protein Li 89n (Deg:64), Heat shock protein-α (Deg:61), DNA topoisomerase 2-α (Deg:59), Chaperonin containing TCP1 (Deg:55), Enolase 1 (Deg:53), ribosomal protein L3 (Deg:53), and ribosomal protein S3 (Deg:52). As illustrated in Figure 3, the nodes of the WT infection are darker; the Deg value is greater, and the interaction relationship between genes is highly reliable and more complex than the network associated with the Δ*espF* infection.

### EspF Upregulates the Expression of Bip

**FIGURE 4:**
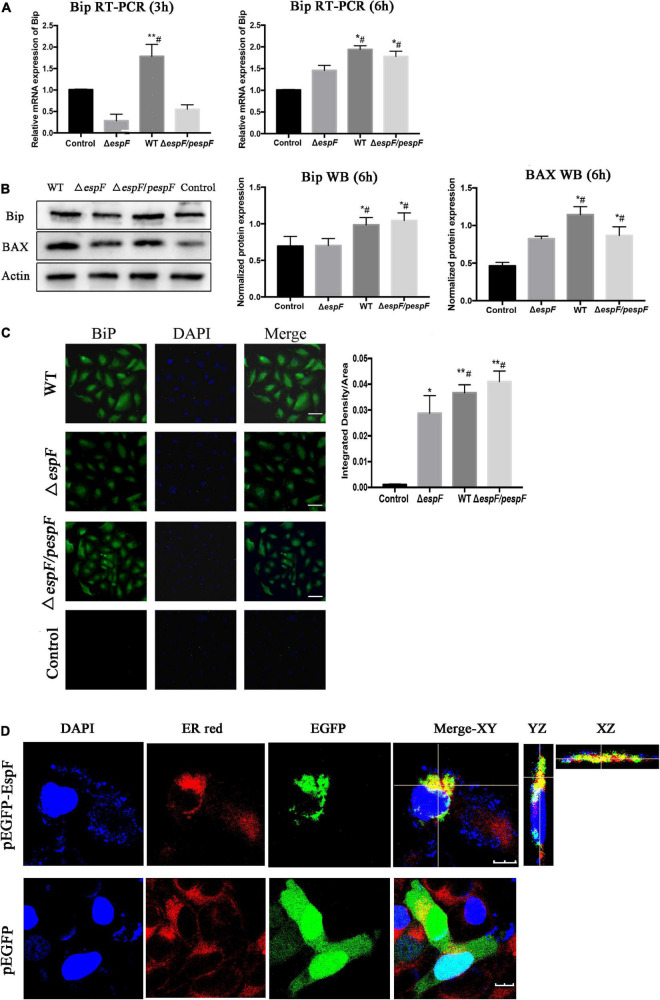
EspF co-localizes with endoplasmic reticulum and upregulates the expression of Bip. **(A,B)** After 6 h of WT, Δ*espF*, and Δ*espF/pespF* infection, the relative RNA expression and protein expression of Bip in HT-29 cells was measured via qPCR and western blot assay. Data were normalized to relative RNA expression, and protein expression is presented as means ± SD from three biological replicates. One-way ANOVA test the *p*-value. *p < 0.05, **p < 0.01 as compared with the control group. #*p* < 0.05 as compared with the Δ*espF* group. **(C)** Immunofluorescent staining assay to visualize the level of Bip expression after the infection of WT, Δ*espF*, and Δ*espF/pespF.* Host cells infected by each group were stained with Bip rabbit polyclonal antibody and goat anti-rabbit IgG Alexa 488 to visualize Bip (green) and DAPI to stain DNA (blue). Representative images from a single experiment are shown. Scale bar, 50 μm. The integrated density of Bip fluorescence signal per field was counted for five randomly selected fields. One-way ANOVA test the *p*-value. *p < 0.05, **p < 0.01 as compared with the control group. #*p* < 0.05 as compared with the Δ*espF* group. **(D)** Immunofluorescent staining assay to visualize the location of EspF and ER after the transfection of plasmids pEGFP-DH5α and pEGFP-DH5α-EspF. Host cells transfected by each group were stained with ER-tracker red to visualize the ER (red) and DAPI to stain DNA (blue). Representative z-stack images were shown. The fluorescence signal in host cells was observed by the confocal microscope FV1000. The Pearson’s correlation was calculated by the NIS-viewer software. Scale bar, 10 μm.

From the ER pathway results of KEGG analysis (Supplementary Figure 1e), several proteins associated with ER stress were significantly upregulated after the WT infection compared with the Δ*espF* infection (*p* < 0.05). Among these, immunoglobulin-binding protein (Bip; Alao known as Grp78) stands out as an ER chaperone that plays a crucial role in protein folding and quality control in the ER lumen (Dana et al., 1990; Evensen et al., 2013; Oka et al., 2013; Cuevas et al., 2017). From the original iTRAQ results, compared with the Δ*espF* infection, the expression of Bip in host cells was significantly upregulated after WT infection with a fold change of 2.845 and *p*-value < 0.001. QPCR and Western blot tests were applied to verify the above results. Compared with Δ*espF* infection, the relative RNA expression (Figure 4A) and protein expression (Figure 4B) of Bip in host cells were significantly upregulated 6 h after WT infection (*p* < 0.05). Immunofluorescent staining assay was applied to visualize the level of Bip expression after the infection of WT versus Δ*espF* in Figure 4C (*p* < 0.05). The integrated density of the Bip fluorescence signal per area was elevated after the WT infection. Furthermore, an expression vector pEGFP-DH5α-EspF was constructed to trace the EspF. The Pearson’s correlation of ER-red and EGFP-green signals were 0.662 (pEGFP-DH5α-EspF) and 0.029 (pEGFP-DH5α group) respectively. By merging the fluorescence signal in host cells after transfection, we demonstrated that EspF localized to the ER (Figure 4D).

### EspF Induces Endoplasmic Reticulum Stress in Host Cells

**FIGURE 5:**
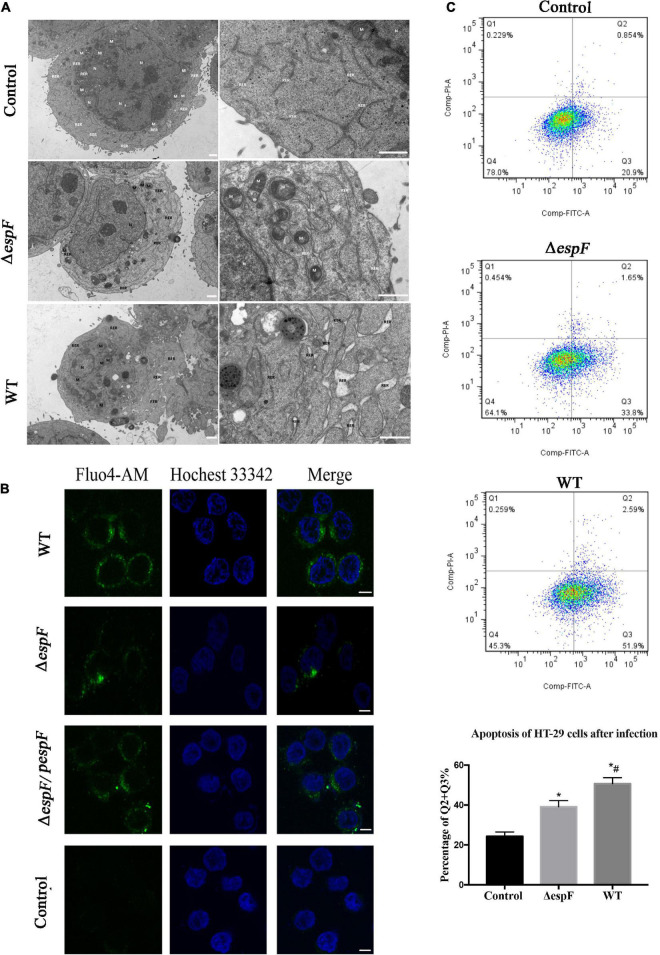
EspF induces ER stress and apoptosis in host cells. **(A)** High-resolution TEM images of Caco-2 cells infected with WT or Δ*espF* for 6 h. Compared to the untreated cells, the TEM images showed the dilatation of the ER after the WT and Δ*espF* infection. RER, rough ER; M, mitochondria; N, nucleus. Scale bar, 1 μm. **(B)** The cytosolic Ca^2+^ concentration within host cells. The HT-29 cells transfected by each group were stained with Fluo-4 probe to visualize Ca^2+^ (green) and Hochest 33342 to stain DNA (blue). Scale bar, 10 μm. **(C)** The effect of WT and Δ*espF* on the viability of HT-29 cells. Detecting HT-29 apoptotic cells after infection via the PI and Annexin V-FITC double-staining. The ratio of late and early apoptotic cells in every group was shown as Q2 + Q3%. Data were expressed as the mean ± SD from three biological replicates. One-way ANOVA test the p value. *p < 0.05 as compared with the control group. #*p* < 0.05 as compared with the Δ*espF* group.

Since Bip ensures the proper folding of proteins and is involved in the degradation of misfolded proteins, it acts as a key repressor of the ERS. The expression of Bip was upregulated within host cells. To further clarify whether ER stress was induced in our cell model, we performed transmission electron microscopy (TEM) to observe the ultrastructure of the infected Caco-2 cells. The rough ER in Caco-2 cells of the Control group did not expand significantly; ribosomes were attaching on the surface (Figure 5A). After Δ*espF* infection, the RER in the cells were abundant, slightly expanded, and some of the surface ribosomes fell off. In the WT infection group, most of the RER expanded, a dilatation change appeared, and the ribosomes on the surface fell off. When ER stress occurs, the Ca^2+^ within the ER will leak into the cytoplasm. We traced the concentration of Ca^2+^ via the Fluo-4 probe (Figure 5B). The cytosolic Ca^2+^ combined the probe and emitted green fluorescence. The fluorescence intensity of the WT-infected group was significantly higher than that of the Δ*espF* infected group and the uninfected group. The Δ*espF/pespF*-infected group regained a high fluorescence intensity.

### EspF Induces the Endoplasmic Reticulum Stress-Dependent Apoptosis Within Host Cells

**FIGURE 6:**
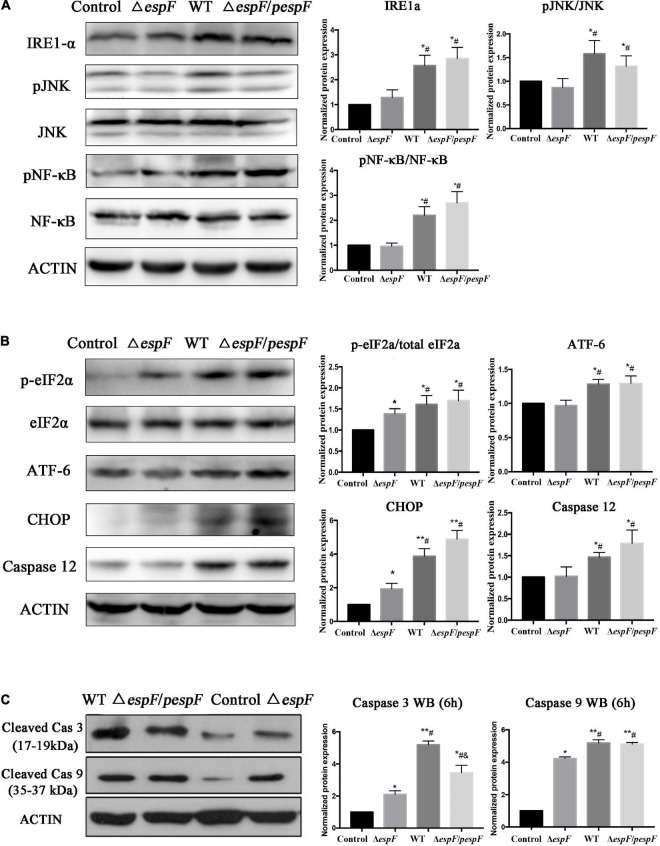
EspF upregulates the proteins associated with ER stress in host cells. After 6 h of WT, Δ*espF*, and Δ*espF/pespF* infection, the expression of proteins associated with ER stress in HT-29 cells was measured via western blot assay. **(A,B)** Protein and phosphor-protein levels of IRE1*a*, JNK, NF-κB, eIF2*a*, ATF-6, CHOP, and Caspase12 in HT-29 cells were assessed by Western blot and quantified. **(C)** Cleaved Caspase 3/9 in HT-29 cells were assessed by Western blot and quantified. Data normalized to protein expression are presented as means ± SD from three biological replicates. One-way ANOVA test the *p*-value. *p < 0.05, **p < 0.01 as compared with the control group. #*p* < 0.05 as compared with the Δ*espF* group. &*p* < 0.05 as compared with the WT group.

Evidence in KEGG pathway showed that the protein processing pathway in the ER was highlighted after WT strain infection compared with the Δ*espF* infection. Several ER stress related proteins were significantly upregulated, which indicated that EspF might induce ER stress. Thus, we explored the expression of downstream signaling molecules. To assess the alternation of ERS after EspF infection, the expression and phosphorylation profiles of IRE1*a*, JNK, NF-κB, eIF2*a*, ATF-6, CHOP, and Caspase3/9/12 were detected by western blot analysis. As shown in Figure 6A, the expression level of IRE1*a* was elevated, followed by increased expression of phosphorylated JNK and NF-κB mediated by WT and Δ*espF/pespF* infection but not by Δ*espF* (*p* < 0.05).

As shown in Figures 6B,C, the phosphorylation levels of eIF2*a* and ATF-6 were elevated, followed by increased expression of CHOP, pro-apoptotic protein Bax (Figure 4B), and activated caspase3/9/12 mediated by WT or Δ*espF/pespF* infection compared with Δ*espF* (*p* < 0.05). To further explore the apoptotic effect of EspF, we also detected the activities of three intrinsic apoptosis markers, caspase-3/caspase-9/caspase-12.

The results were consistent with the proteomic data obtained via the iTRAQ labeled LC-MS/MS method. These protein expression changes were accompanied by significant induction of apoptosis in WT and Δ*espF/pespF*-infected HT-29 cells. The results suggested that the EspF promoted cell death via induction of ERS, which subsequently led to massive DNA strand breaks and the activation of the intrinsic apoptotic pathway. Furthermore, we assessed the apoptosis level of infected HT-29 cells via PI and Annexin V-FITC double staining united with flow cytometry (Figure 5C). The average apoptosis percentage (including late and early apoptosis) of the uninfected, WT, and Δ*espF* infected groups was 24.34, 50.68, and 39.11%, respectively. Compared to the uninfected group (13.1%), the number of apoptotic cells (including late and early apoptosis) significantly increased in the Δ*espF* and WT groups (*p* < 0.05). Compared to the Δ*espF* group, the WT group showed fewer living cells, but apoptotic cells increased significantly (*p* < 0.05).

## Discussion

So far, although proteomic techniques have been widely utilized for bacteria–host interaction research, little information is available on intracellular protein regulation within host cells after EHEC infection.

Here, we applied iTRAQ united with LC-MS/MS for the comparative proteomic analysis of host cells infected with WT and Δ*espF* strains of EHEC, owing to its superior capability in the simultaneous comparison of multi-samples with a vast dynamic protein abundance (Zhao et al., 2016). In this research, based on a fold change > 1.5 or <0.67 and a *p*-value < 0.05229, 145 and 229 differentially regulated proteins were identified in HT-29 cells treated by EHEC WT and Δ*espF*, respectively. Compared with Δ*espF*-infected cells, a total of 230 proteins have been identified in WT-infected group, including 208 up-regulated proteins and 22 down-regulated proteins. The differences in cellular responses mainly included immune regulation, cellular assembly and organization, protein synthesis, signal transduction, ER stress, and apoptosis.

From GO analysis, it indicated that EspF regulated the host metabolic and biological process for its colonization and infection. As for the cellular components part, it demonstrated EspF altered proteins were mostly located in the organelle and organelle part, which was consistent with our previous research that EspF targeted to the mitochondria and nucleus (Zhao et al., 2013; Fu et al., 2021). The most predominant molecular function of EspF infection was binding. As EspF could disrupt the tight junction, degrade the intermediate filament and induce actin polymerization (Holmes et al., 2010).

Several host proteins were once screened and verified to interact with EspF (Holmes et al., 2010; Hua et al., 2018).

From our proteomic analysis, we found three EspF-binding proteins were up-regulated after WT infection (compared with Δ*espF*): profilin (0.2997, *p* = 0.00016), Arp2/3 (0.3679, *p* = 0.02157) and 14-3-3ζ (0.4835, *p* = 0.0089). It meant that the interaction of EspF to these proteins upregulated their expression within host cells significantly. Other proteins were not found to be significantly up- or down- regulated, perhaps because of iTRAQ methodological limitations, insufficient replicates, or inappropriate incubation time.

Using KEGG pathway analysis, several pathways were more up-regulated after WT infection such as Ribosome, Protein processing in the ER, Antigen processing and presentation and tight junction. The ER is a vital membrane organelle for protein synthesis, folding, and secretion in eukaryotic cells. It can also participate in metabolic processes such as Ca^2+^ storage, gluconeogenesis, lipid and cholesterol synthesis, and the formation of autophagic vacuolization (Cao, 2015). When the intracellular oxidation-reduction homeostasis is disturbed, lots of misfolded or unfolded proteins will accumulate in the ER cavity, a phenomenon called ER stress (Cao and Kaufman, 2014). To eliminate the harmful consequences of ER stress, cells have developed the unfolded protein response (UPR) (Hua et al., 2018). Under the ER stress condition, Bip dissociates from sensors and binds to misfolded proteins in the ER, which then activates downstream signals of inositol inositol-requiring protein-1 (IRE1*a*), transcription factor-6 (ATF6), and protein kinase RNA-like ER kinase (PERK) (Kimata et al., 2003).

On the IRE1*a* pathway, the phosphorylated IRE1 interacts with the TRAF2 (tumor necrosis factor receptor-associated factor-2), ultimately activating Jun amino-terminal kinase (JNK) (Urano et al., 2000). Given the connection between sustained JNK activity and cell death, activating JNK may link IRE1-mediated ER stress to cell death (Xia et al., 1995; Ventura et al., 2006). On the PERK pathway, PERK phosphorylates the eIF2 (eukaryotic translation initiation factor 2), which leads to the translation of the ATF4 (activating transcription factor 4). Therefore, the expression of ATF4’s key downstream target, CHOP (C/EBP-homologous protein), is increased when eIF2α is phosphorylated by PERK (Ron and Walter, 2007). Studies involving depleted or overexpressed CHOP have proved that CHOP is involved in ER stress-induced apoptosis (Guo et al., 2012; Shen et al., 2014). CHOP can lead to the activation of NF-κB. The activated NF-κB enhances the production of interleukin-8 (IL-8) and then leads to intestinal dysfunction. In addition, CHOP can promote the macrophage infiltration and induce the production of reactive oxygen species (ROS) and interleukin-1β (IL-1β) (Tabas and Ron, 2011). Furthermore, CHOP can enhance the apoptosis of epithelial cells, goblet cells, Paneth cells and impair their secretion ability. These cells are more sensitive to inflammatory transmitters and bacterial products, which leads to the development of colitis (Ma et al., 2017).

In our following study, we verified that the total expression of Bip, IRE1*a*, ATF-6, CHOP, BAX, caspase 12, cleaved caspase3/9, and cytosolic Ca^2+^ concentration within host cells were elevated after WT and Δ*espF/pespF* infection but not after Δ*espF* infection. The phosphorylation profiles of pJNK/JNK, p-eIF2*a*/eIF2*a*, and pNF-κB/NF-κB were elevated after WT and Δ*espF/pespF* infection. Earlier studies demonstrated that EspF initiated the induction of the intrinsic apoptotic pathway by disrupting the mitochondrial membrane potential (MMP), which released cytochrome *c* into the cytoplasm, thereby leading to caspase 9 cleavage (Nougayrede and Donnenberg, 2004). In the ER stress-induced apoptosis pathway, the excessive accumulation of misfolded proteins in the ER and disturbance in Ca^2+^ homeostasis induced caspase 12-mediated apoptosis, in which activated caspase 12 translocated from the ER into the cytosol to directly cleave caspase 9 and then activate caspase 3 (Chen et al., 2018). Our study proved that caspase 12, cleaved caspase 9, and cleaved caspase 3 levels were elevated in the presence of EspF, which indicated that the ER stress-dependent apoptosis pathway could also be activated within host cells.

Furthermore, we identified that the IRE1*a* pathway was activated after EspF infection.

Prolonged IRE1-mediated activation may promote apoptosis by degrading mRNAs encoding essential cell-survival proteins, including XBP-1. XBP-1 plays a crucial role in the highly secretory cells such as hepatocytes (Reimold et al., 2000), plasma cells (Iwakoshi et al., 2003), plasmacytoid dendritic cells (Lee et al., 2005), and pancreatic acinar cells (Iwakoshi et al., 2007). In addition, ER stress can cause a subsequent series of physiological changes within host cells, including autophagy and autophagic cell death. Hence, ER stress has been increasingly recognized to be both a secondary consequence of inflammation and neoplasia (Zhang and Kaufman, 2008) as well as a primary factor in causing inflammation and potentially cancer (Kaser et al., 2008). Whether the EspF protein participates in these subsequent reactions still needs further experiments to be clarified.

In summary, these results provided novel evidence that EspF activates the ER stress response and enhances cell death of HT-29 cells.

This study shows that the regulation of the UPR signal by EspF protein can promote endoplasmic reticulum stress-related death and enhance cell apoptosis. EHEC has the characteristics of poor prognosis for antibiotic treatment. The findings of this study may be an effective strategy that can be combined with current treatment options to improve the treatment effect of EHEC.

## Conclusion

Based on the iTRAQ results and experiments described herein, we conclude that the EspF of Enterohemorrhagic *Escherichia coli* induced ER stress in intestinal epithelial cells, and the ER stress-dependent apoptosis pathway is activated within host cells.

## Data Availability Statement

The datasets presented in this study can be found in online repositories. The names of the repository/repositories and accession number(s) can be found below: Figshare – doi: 10.6084/m9.figshare.14933373.v1.

## Author Contributions

XW and CW designed the study. KY, MF, SL, and CF engaged in the acquisition and analysis of the data. HZ, LY, ZS, and DS participated in bioinformatics interpretation and following analysis. XW and DS drafted the manuscript. All authors had final approval of the version to be submitted.

## Conflict of Interest

HZ was employed by the Genecreate Biological Engineering Co., Ltd. The remaining authors declare that the research was conducted in the absence of any commercial or financial relationships that could be construed as a potential conflict of interest.

## Publisher’s Note

All claims expressed in this article are solely those of the authors and do not necessarily represent those of their affiliated organizations, or those of the publisher, the editors and the reviewers. Any product that may be evaluated in this article, or claim that may be made by its manufacturer, is not guaranteed or endorsed by the publisher.

## Footnotes

1
http://geneontology.org/

2
http://www.ncbi.nlm.nih.gov/COG/

3
https://www.kegg.jp/
