# Supplementary material for: EspF of Enterohemorrhagic Escherichia coli Enhances Apoptosis via Endoplasmic Reticulum Stress in Intestinal Epithelial Cells: An Isobaric Tags for Relative and Absolute Quantitation-Based Comparative Proteomic Analysis
Source: Front Microbiol. 2022 Jun 30;13:900919. doi: 10.3389/fmicb.2022.900919 (PMC9279134; doi:10.3389/fmicb.2022.900919)
Supplement: Supplementary file 1 [file Table_1.DOCX]

**Table S1. The details of protein identification and quantification.**

| **No.** | **Identification score** | **Accession** | **Name** | **Protein Mass** | **Protein Length** | **Protein**  **Sequence coverage (95% confidence)** | **No. of Peptides** | **No. of Unique Peptides** | **No. of Spectra** | **No. of Unique Spectra** |
| --- | --- | --- | --- | --- | --- | --- | --- | --- | --- | --- |
| 1 | 557.34 | sp|Q15149|PLEC_HUMAN | Plectin OS=Homo sapiens GN=PLEC PE=1 SV=3 | 531785.9 | 4684 | 53.99 | 271 | 239 | 1894 | 1678 |
| 2 | 345.51 | sp|Q09666|AHNK_HUMAN | Neuroblast differentiation-associated protein AHNAK OS=Homo sapiens GN=AHNAK PE=1 SV=2 | 629098.1 | 5890 | 67.1 | 250 | 248 | 1897 | 1890 |
| 3 | 213.91 | sp|P05787|K2C8_HUMAN | Keratin, type II cytoskeletal 8 OS=Homo sapiens GN=KRT8 PE=1 SV=7 | 53703.8 | 483 | 94.62 | 126 | 108 | 4827 | 4300 |
| 4 | 205.61 | sp|Q7Z406|MYH14_HUMAN | Myosin-14 OS=Homo sapiens GN=MYH14 PE=1 SV=2 | 227868.6 | 1995 | 52.08 | 115 | 73 | 947 | 601 |
| 5 | 182.98 | sp|O75369|FLNB_HUMAN | Filamin-B OS=Homo sapiens GN=FLNB PE=1 SV=2 | 278162.4 | 2602 | 46.31 | 101 | 95 | 781 | 753 |
| 6 | 182.52 | tr|A0A024R1N1|A0A024R1N1_HUMAN | Myosin, heavy polypeptide 9, non-muscle, isoform CRA_a OS=Homo sapiens GN=MYH9 PE=3 SV=1 | 226530.5 | 1960 | 49.03 | 114 | 97 | 897 | 756 |
| 7 | 151.18 | tr|A0A024RAY2|A0A024RAY2_HUMAN | Keratin 18, isoform CRA_a OS=Homo sapiens GN=KRT18 PE=3 SV=1 | 48057.4 | 430 | 91.86 | 89 | 86 | 2551 | 2358 |
| 8 | 146.53 | sp|P02545|LMNA_HUMAN | Prelamin-A/C OS=Homo sapiens GN=LMNA PE=1 SV=1 | 74138.8 | 664 | 81.02 | 75 | 73 | 1184 | 1164 |
| 9 | 139.69 | sp|Q13813|SPTN1_HUMAN | Spectrin alpha chain, non-erythrocytic 1 OS=Homo sapiens GN=SPTAN1 PE=1 SV=3 | 284537 | 2472 | 41.22 | 83 | 81 | 341 | 334 |
| 10 | 130.48 | sp|P21333|FLNA_HUMAN | Filamin-A OS=Homo sapiens GN=FLNA PE=1 SV=4 | 280737.6 | 2647 | 39.21 | 83 | 77 | 513 | 485 |
| 11 | 125.15 | tr|A0A0U1RQF0|A0A0U1RQF0_HUMAN | Fatty acid synthase OS=Homo sapiens GN=FASN PE=1 SV=1 | 273195.8 | 2509 | 37.62 | 75 | 75 | 526 | 524 |
| 12 | 117.52 | tr|A0A024R4A0|A0A024R4A0_HUMAN | Nucleolin, isoform CRA_b OS=Homo sapiens GN=NCL PE=4 SV=1 | 76613.9 | 710 | 50.7 | 58 | 56 | 542 | 528 |
| 13 | 112.17 | tr|A0A024R3X4|A0A024R3X4_HUMAN | Heat shock 60kDa protein 1 (Chaperonin), isoform CRA_a OS=Homo sapiens GN=HSPD1 PE=2 SV=1 | 61054.2 | 573 | 86.21 | 59 | 58 | 1293 | 1284 |
| 14 | 111.59 | tr|V9HWB8|V9HWB8_HUMAN | Pyruvate kinase OS=Homo sapiens GN=HEL-S-30 PE=2 SV=1 | 57936.4 | 531 | 82.86 | 56 | 54 | 1632 | 1623 |
| 15 | 109.08 | tr|A0A0S2Z3G9|A0A0S2Z3G9_HUMAN | Actinin alpha 4 isoform 1 (Fragment) OS=Homo sapiens GN=ACTN4 PE=2 SV=1 | 104853.2 | 911 | 62.46 | 57 | 46 | 886 | 638 |
| 16 | 103.84 | tr|A0A024RC65|A0A024RC65_HUMAN | HCG1991735, isoform CRA_a OS=Homo sapiens GN=hCG_1991735 PE=4 SV=1 | 189250.4 | 1657 | 40.74 | 60 | 57 | 342 | 316 |
| 17 | 102.29 | sp|Q14980|NUMA1_HUMAN | Nuclear mitotic apparatus protein 1 OS=Homo sapiens GN=NUMA1 PE=1 SV=2 | 238257.6 | 2115 | 31.73 | 60 | 59 | 226 | 221 |
| 18 | 101.2 | tr|D6W5C0|D6W5C0_HUMAN | Spectrin, beta, non-erythrocytic 1, isoform CRA_b OS=Homo sapiens GN=SPTBN1 PE=4 SV=1 | 264438 | 2278 | 33.67 | 59 | 57 | 309 | 300 |
| 19 | 95.97 | tr|V9HW22|V9HW22_HUMAN | Epididymis luminal protein 33 OS=Homo sapiens GN=HEL-S-72p PE=2 SV=1 | 70897.6 | 646 | 64.24 | 51 | 38 | 1313 | 1022 |
| 21 | 94.99 | tr|A0A024RD80|A0A024RD80_HUMAN | Heat shock protein 90kDa alpha (Cytosolic), class B member 1, isoform CRA_a OS=Homo sapiens GN=HSP90AB1 PE=3 SV=1 | 83263.5 | 724 | 50.97 | 49 | 29 | 1401 | 686 |
| 22 | 94.18 | tr|A0A024R5Z7|A0A024R5Z7_HUMAN | Annexin OS=Homo sapiens GN=ANXA2 PE=3 SV=1 | 38603.6 | 339 | 82.89 | 48 | 48 | 1183 | 1119 |
| 23 | 92.18 | sp|P20700|LMNB1_HUMAN | Lamin-B1 OS=Homo sapiens GN=LMNB1 PE=1 SV=2 | 66407.7 | 586 | 62.46 | 48 | 44 | 234 | 202 |
| 24 | 90.13 | tr|B4E3A4|B4E3A4_HUMAN | cDNA FLJ57283, highly similar to Actin, cytoplasmic 2 OS=Homo sapiens PE=2 SV=1 | 39799.2 | 356 | 87.08 | 53 | 1 | 2068 | 15 |
| 25 | 89.86 | tr|V9HW80|V9HW80_HUMAN | Epididymis luminal protein 220 OS=Homo sapiens GN=HEL-S-70 PE=2 SV=1 | 89320.9 | 806 | 56.08 | 45 | 45 | 378 | 375 |
| 26 | 89.16 | tr|V9HWB4|V9HWB4_HUMAN | Epididymis secretory sperm binding protein Li 89n OS=Homo sapiens GN=HEL-S-89n PE=2 SV=1 | 72332.4 | 654 | 58.1 | 47 | 43 | 1293 | 1142 |
| 27 | 88.15 | tr|V9HVY3|V9HVY3_HUMAN | Protein disulfide-isomerase OS=Homo sapiens GN=HEL-S-269 PE=2 SV=1 | 56781.8 | 505 | 77.82 | 43 | 41 | 810 | 783 |
| 28 | 87.38 | tr|A0A024R4F1|A0A024R4F1_HUMAN | Enolase 1, (Alpha), isoform CRA_a OS=Homo sapiens GN=ENO1 PE=2 SV=1 | 47168.6 | 434 | 79.95 | 46 | 41 | 1071 | 987 |
| 29 | 85.29 | sp|P13639|EF2_HUMAN | Elongation factor 2 OS=Homo sapiens GN=EEF2 PE=1 SV=4 | 95337.4 | 858 | 50.47 | 43 | 42 | 436 | 422 |
| 30 | 84.49 | tr|K7EKI8|K7EKI8_HUMAN | Periplakin OS=Homo sapiens GN=PPL PE=1 SV=1 | 204488.1 | 1754 | 32.1 | 49 | 48 | 147 | 143 |
| 31 | 83.89 | tr|V9HWC0|V9HWC0_HUMAN | Epididymis luminal protein 70 OS=Homo sapiens GN=HEL70 PE=2 SV=1 | 67819.6 | 577 | 57.89 | 40 | 30 | 452 | 339 |
| 32 | 80.67 | tr|V9HWF4|V9HWF4_HUMAN | Phosphoglycerate kinase OS=Homo sapiens GN=HEL-S-68p PE=2 SV=1 | 44614.4 | 417 | 82.97 | 44 | 43 | 631 | 630 |
| 33 | 76.23 | sp|P35900|K1C20_HUMAN | Keratin, type I cytoskeletal 20 OS=Homo sapiens GN=KRT20 PE=1 SV=1 | 48486.3 | 424 | 74.06 | 41 | 37 | 698 | 663 |
| 34 | 74.94 | tr|A0A090N8Y2|A0A090N8Y2_HUMAN | Protein disulfide-isomerase A4 OS=Homo sapiens GN=ERP70 PE=2 SV=1 | 72931.9 | 645 | 48.06 | 41 | 38 | 396 | 372 |
| 35 | 73.5 | sp|P52272|HNRPM_HUMAN | Heterogeneous nuclear ribonucleoprotein M OS=Homo sapiens GN=HNRNPM PE=1 SV=3 | 77515.3 | 730 | 57.12 | 38 | 12 | 317 | 81 |
| 36 | 72.07 | tr|A8K5I0|A8K5I0_HUMAN | Epididymis secretory protein Li 103 OS=Homo sapiens GN=HEL-S-103 PE=2 SV=1 | 70051.6 | 641 | 58.35 | 51 | 39 | 988 | 694 |
| 37 | 71.8 | sp|P07814|SYEP_HUMAN | Bifunctional glutamate/proline--tRNA ligase OS=Homo sapiens GN=EPRS PE=1 SV=5 | 170589.7 | 1512 | 33.33 | 41 | 41 | 181 | 178 |
| 38 | 71.31 | tr|Q5CAQ5|Q5CAQ5_HUMAN | Tumor rejection antigen (Gp96) 1 OS=Homo sapiens GN=TRA1 PE=2 SV=1 | 92338.9 | 802 | 47.01 | 39 | 37 | 483 | 467 |
| 39 | 71.03 | tr|A0A024R8S5|A0A024R8S5_HUMAN | Protein disulfide-isomerase OS=Homo sapiens GN=P4HB PE=2 SV=1 | 57115.8 | 508 | 62.6 | 37 | 35 | 444 | 434 |
| 40 | 70.58 | sp|Q9Y490|TLN1_HUMAN | Talin-1 OS=Homo sapiens GN=TLN1 PE=1 SV=3 | 269764.5 | 2541 | 20.86 | 41 | 39 | 192 | 188 |
| 41 | 70.06 | tr|A7BI36|A7BI36_HUMAN | p180/ribosome receptor OS=Homo sapiens GN=RRBP1 PE=2 SV=2 | 165748.1 | 1540 | 44.87 | 40 | 39 | 184 | 180 |
| 42 | 69 | sp|P11216|PYGB_HUMAN | Glycogen phosphorylase, brain form OS=Homo sapiens GN=PYGB PE=1 SV=5 | 96695.2 | 843 | 40.09 | 35 | 25 | 354 | 284 |
| 43 | 68.74 | tr|A0A0S2Z4G4|A0A0S2Z4G4_HUMAN | Tropomyosin 3 isoform 1 (Fragment) OS=Homo sapiens GN=TPM3 PE=2 SV=1 | 29032.3 | 248 | 77.42 | 35 | 16 | 352 | 186 |
| 44 | 68.71 | sp|P78527|PRKDC_HUMAN | DNA-dependent protein kinase catalytic subunit OS=Homo sapiens GN=PRKDC PE=1 SV=3 | 469084.2 | 4128 | 15.12 | 53 | 53 | 222 | 221 |
| 45 | 68.43 | tr|Q8N1C8|Q8N1C8_HUMAN | HSPA9 protein (Fragment) OS=Homo sapiens GN=HSPA9 PE=2 SV=1 | 73853.1 | 681 | 46.99 | 36 | 34 | 512 | 454 |
| 46 | 68.4 | tr|A0A024R9C1|A0A024R9C1_HUMAN | Polyadenylate-binding protein OS=Homo sapiens GN=PABPC1 PE=3 SV=1 | 70670.4 | 636 | 54.56 | 33 | 24 | 288 | 181 |
| 47 | 68.05 | sp|Q08211|DHX9_HUMAN | ATP-dependent RNA helicase A OS=Homo sapiens GN=DHX9 PE=1 SV=4 | 140957.5 | 1270 | 30.63 | 38 | 38 | 183 | 181 |
| 48 | 67.55 | sp|Q00610|CLH1_HUMAN | Clathrin heavy chain 1 OS=Homo sapiens GN=CLTC PE=1 SV=5 | 191613 | 1675 | 26.09 | 41 | 41 | 272 | 270 |
| 49 | 67.14 | tr|V9HWN7|V9HWN7_HUMAN | Fructose-bisphosphate aldolase OS=Homo sapiens GN=HEL-S-87p PE=2 SV=1 | 39419.7 | 364 | 71.98 | 35 | 30 | 710 | 599 |
| 50 | 65.78 | tr|B4DLR3|B4DLR3_HUMAN | cDNA FLJ54020, highly similar to Heterogeneous nuclear ribonucleoprotein U OS=Homo sapiens PE=2 SV=1 | 86860.3 | 784 | 37.88 | 34 | 34 | 385 | 385 |
| 51 | 65.03 | tr|Q53EM5|Q53EM5_HUMAN | Transketolase (Fragment) OS=Homo sapiens PE=2 SV=1 | 67905 | 623 | 61 | 33 | 33 | 387 | 386 |
| 52 | 64.43 | tr|Q5TZZ9|Q5TZZ9_HUMAN | Annexin OS=Homo sapiens GN=ANXA1 PE=2 SV=1 | 38713.9 | 346 | 68.79 | 31 | 31 | 544 | 541 |
| 53 | 64.04 | sp|P23246|SFPQ_HUMAN | Splicing factor, proline- and glutamine-rich OS=Homo sapiens GN=SFPQ PE=1 SV=2 | 76149.1 | 707 | 35.08 | 31 | 28 | 197 | 184 |
| 54 | 63.46 | tr|A0A024R542|A0A024R542_HUMAN | Tankyrase 1 binding protein 1, 182kDa, isoform CRA_a OS=Homo sapiens GN=TNKS1BP1 PE=2 SV=1 | 181794 | 1729 | 28.92 | 35 | 35 | 167 | 167 |
| 55 | 63.34 | tr|E7EQR4|E7EQR4_HUMAN | Ezrin OS=Homo sapiens GN=EZR PE=1 SV=3 | 69371.2 | 586 | 59.39 | 42 | 31 | 342 | 221 |
| 56 | 63.27 | tr|B7ZLD5|B7ZLD5_HUMAN | Integrin beta OS=Homo sapiens GN=ITGB4 PE=2 SV=1 | 194910.4 | 1752 | 25.17 | 39 | 38 | 161 | 158 |
| 57 | 62.82 | tr|A0A024R1A3|A0A024R1A3_HUMAN | Testicular secretory protein Li 63 OS=Homo sapiens GN=UBE1 PE=2 SV=1 | 117848.1 | 1058 | 36.01 | 32 | 32 | 285 | 285 |
| 58 | 61.72 | tr|V9HVZ4|V9HVZ4_HUMAN | Glyceraldehyde-3-phosphate dehydrogenase OS=Homo sapiens GN=HEL-S-162eP PE=2 SV=1 | 36053 | 335 | 71.04 | 34 | 33 | 980 | 976 |
| 59 | 60.54 | tr|B2R5U1|B2R5U1_HUMAN | cDNA, FLJ92620, highly similar to Homo sapiens staphylococcal nuclease domain containing 1 (SND1),mRNA OS=Homo sapiens PE=2 SV=1 | 99670.5 | 885 | 35.82 | 29 | 29 | 242 | 241 |
| 60 | 60.52 | sp|P35221|CTNA1_HUMAN | Catenin alpha-1 OS=Homo sapiens GN=CTNNA1 PE=1 SV=1 | 100070.3 | 906 | 37.86 | 29 | 29 | 201 | 201 |
| 61 | 59.19 | tr|B2ZZ90|B2ZZ90_HUMAN | Acetyl-Coenzyme A carboxylase alpha OS=Homo sapiens GN=ACACA PE=2 SV=1 | 265551.7 | 2346 | 17.14 | 32 | 32 | 194 | 193 |
| 62 | 58.88 | tr|X5D2M8|X5D2M8_HUMAN | Major vault protein isoform A (Fragment) OS=Homo sapiens GN=MVP PE=2 SV=1 | 99326.2 | 893 | 44.57 | 31 | 31 | 153 | 151 |
| 63 | 58.37 | sp|P60174|TPIS_HUMAN | Triosephosphate isomerase OS=Homo sapiens GN=TPI1 PE=1 SV=3 | 30790.8 | 286 | 81.82 | 29 | 29 | 438 | 437 |
| 64 | 58.29 | tr|Q6FHZ0|Q6FHZ0_HUMAN | Malate dehydrogenase OS=Homo sapiens GN=MDH2 PE=2 SV=1 | 35559.1 | 338 | 72.78 | 30 | 30 | 618 | 615 |
| 65 | 57.78 | tr|V9HW72|V9HW72_HUMAN | Epididymis secretory sperm binding protein Li 94n OS=Homo sapiens GN=HEL-S-94n PE=2 SV=1 | 62638.7 | 543 | 52.67 | 31 | 30 | 238 | 232 |
| 66 | 57.21 | tr|V9HW59|V9HW59_HUMAN | Annexin OS=Homo sapiens GN=HEL-S-274 PE=2 SV=1 | 36056.6 | 321 | 70.4 | 31 | 30 | 278 | 276 |
| 67 | 56.03 | sp|P04843|RPN1_HUMAN | Dolichyl-diphosphooligosaccharide--protein glycosyltransferase subunit 1 OS=Homo sapiens GN=RPN1 PE=1 SV=1 | 68568.8 | 607 | 45.3 | 26 | 26 | 283 | 281 |
| 68 | 55.95 | tr|A0A024R1T9|A0A024R1T9_HUMAN | ATP-citrate synthase OS=Homo sapiens GN=ACLY PE=3 SV=1 | 120838.3 | 1101 | 30.06 | 28 | 28 | 136 | 136 |
| 69 | 55.85 | sp|P09327|VILI_HUMAN | Villin-1 OS=Homo sapiens GN=VIL1 PE=1 SV=4 | 92694.6 | 827 | 39.9 | 29 | 29 | 188 | 187 |
| 70 | 55.42 | tr|E9KL48|E9KL48_HUMAN | Epididymis tissue sperm binding protein Li 18mP OS=Homo sapiens GN=GLUD1 PE=2 SV=1 | 61397.3 | 558 | 51.43 | 27 | 26 | 320 | 319 |
| 71 | 55.12 | tr|A0A087X1U6|A0A087X1U6_HUMAN | Epiplakin OS=Homo sapiens GN=EPPK1 PE=1 SV=1 | 555653.1 | 5088 | 35.12 | 54 | 4 | 195 | 12 |
| 72 | 54.95 | tr|Q53HU0|Q53HU0_HUMAN | Chaperonin containing TCP1, subunit 8 (Theta) variant (Fragment) OS=Homo sapiens PE=2 SV=1 | 59650.1 | 548 | 47.26 | 28 | 28 | 286 | 281 |
| 73 | 54.89 | tr|Q8IZ29|Q8IZ29_HUMAN | Tubulin beta chain OS=Homo sapiens GN=TUBB2C PE=2 SV=1 | 49839.7 | 445 | 65.39 | 29 | 8 | 997 | 308 |
| 74 | 54.85 | tr|B4E0X8|B4E0X8_HUMAN | cDNA FLJ61021, highly similar to Far upstream element-binding protein 1 OS=Homo sapiens PE=2 SV=1 | 66231.7 | 629 | 56.92 | 27 | 21 | 153 | 117 |
| 75 | 54.76 | sp|O00159|MYO1C_HUMAN | Unconventional myosin-Ic OS=Homo sapiens GN=MYO1C PE=1 SV=4 | 121680.6 | 1063 | 30.76 | 30 | 29 | 162 | 160 |
| 76 | 54.4 | sp|O60506|HNRPQ_HUMAN | Heterogeneous nuclear ribonucleoprotein Q OS=Homo sapiens GN=SYNCRIP PE=1 SV=2 | 69602.3 | 623 | 44.78 | 29 | 16 | 185 | 68 |
| 77 | 54.17 | tr|V9HW26|V9HW26_HUMAN | ATP synthase subunit alpha OS=Homo sapiens GN=HEL-S-123m PE=2 SV=1 | 59750.1 | 553 | 49.73 | 29 | 29 | 437 | 435 |
| 78 | 54 | sp|Q92841|DDX17_HUMAN | Probable ATP-dependent RNA helicase DDX17 OS=Homo sapiens GN=DDX17 PE=1 SV=2 | 80271.8 | 729 | 40.74 | 29 | 22 | 214 | 124 |
| 79 | 53.96 | tr|Q53HM9|Q53HM9_HUMAN | Eukaryotic translation elongation factor 1 alpha 1 variant (Fragment) OS=Homo sapiens PE=2 SV=1 | 50141.5 | 462 | 64.07 | 30 | 30 | 1070 | 1067 |
| 80 | 52.72 | tr|A0A024R652|A0A024R652_HUMAN | Methylenetetrahydrofolate dehydrogenase (NADP+ dependent) 1, methenyltetrahydrofolate cyclohydrolase, formyltetrahydrofolate synthetase, isoform CRA_a OS=Homo sapiens GN=MTHFD1 PE=3 SV=1 | 101530.3 | 935 | 29.52 | 28 | 27 | 108 | 102 |
| 81 | 52.68 | tr|E5KNY5|E5KNY5_HUMAN | Leucine-rich PPR-motif containing OS=Homo sapiens GN=LRPPRC PE=4 SV=1 | 157903.4 | 1394 | 22.67 | 29 | 28 | 144 | 138 |
| 82 | 52.46 | sp|P05023|AT1A1_HUMAN | Sodium/potassium-transporting ATPase subunit alpha-1 OS=Homo sapiens GN=ATP1A1 PE=1 SV=1 | 112895 | 1023 | 32.36 | 31 | 30 | 244 | 235 |
| 83 | 51.48 | tr|J9R021|J9R021_HUMAN | Eukaryotic translation initiation factor 3 subunit A OS=Homo sapiens GN=eIF3a PE=2 SV=1 | 166480.6 | 1382 | 19.83 | 25 | 25 | 59 | 58 |
| 84 | 51.45 | sp|P16403|H12_HUMAN | Histone H1.2 OS=Homo sapiens GN=HIST1H1C PE=1 SV=2 | 21364.6 | 213 | 61.97 | 26 | 7 | 404 | 44 |
| 85 | 51.02 | tr|V9HW29|V9HW29_HUMAN | Kinesin-like protein OS=Homo sapiens GN=HEL-S-61 PE=2 SV=1 | 109684.1 | 963 | 32.29 | 27 | 27 | 107 | 101 |
| 86 | 50.99 | sp|Q14204|DYHC1_HUMAN | Cytoplasmic dynein 1 heavy chain 1 OS=Homo sapiens GN=DYNC1H1 PE=1 SV=5 | 532403.4 | 4646 | 11.19 | 44 | 42 | 144 | 142 |
| 87 | 50.86 | tr|B2R5V9|B2R5V9_HUMAN | cDNA, FLJ92652, highly similar to Homo sapiens high density lipoprotein binding protein (vigilin)(HDLBP), mRNA OS=Homo sapiens PE=2 SV=1 | 141378.4 | 1268 | 26.58 | 26 | 26 | 112 | 112 |
| 88 | 50.2 | sp|P53621|COPA_HUMAN | Coatomer subunit alpha OS=Homo sapiens GN=COPA PE=1 SV=2 | 138344.6 | 1224 | 24.02 | 26 | 26 | 197 | 194 |
| 89 | 49.6 | tr|V9HW96|V9HW96_HUMAN | Chaperonin containing TCP1, subunit 2 (Beta), isoform CRA_b OS=Homo sapiens GN=HEL-S-100n PE=2 SV=1 | 57487.6 | 535 | 53.46 | 25 | 25 | 174 | 164 |
| 90 | 49.54 | tr|D0PNI1|D0PNI1_HUMAN | Epididymis luminal protein 4 OS=Homo sapiens GN=YWHAZ PE=2 SV=1 | 27744.8 | 245 | 65.31 | 25 | 16 | 412 | 305 |
| 91 | 49.36 | tr|Q6NXR8|Q6NXR8_HUMAN | 40S ribosomal protein S3a OS=Homo sapiens GN=RPS3A PE=2 SV=1 | 29974.8 | 264 | 62.12 | 25 | 25 | 177 | 176 |
| 92 | 49.09 | sp|Q02790|FKBP4_HUMAN | Peptidyl-prolyl cis-trans isomerase FKBP4 OS=Homo sapiens GN=FKBP4 PE=1 SV=3 | 51804.2 | 459 | 52.51 | 25 | 25 | 149 | 148 |
| 93 | 49.06 | tr|V9HWB9|V9HWB9_HUMAN | L-lactate dehydrogenase OS=Homo sapiens GN=HEL-S-133P PE=2 SV=1 | 36688.5 | 332 | 62.65 | 25 | 22 | 303 | 265 |
| 94 | 48.53 | tr|A0A024RDL1|A0A024RDL1_HUMAN | Chaperonin containing TCP1, subunit 6A (Zeta 1), isoform CRA_a OS=Homo sapiens GN=CCT6A PE=3 SV=1 | 58023.6 | 531 | 44.82 | 23 | 23 | 207 | 207 |
| 95 | 48.5 | tr|A0A0S2Z491|A0A0S2Z491_HUMAN | Nucleophosmin isoform 2 (Fragment) OS=Homo sapiens GN=NPM1 PE=2 SV=1 | 32574.8 | 294 | 61.22 | 25 | 25 | 214 | 213 |
| 96 | 48.26 | tr|B2R659|B2R659_HUMAN | cDNA, FLJ92803, highly similar to Homo sapiens hydroxysteroid (17-beta) dehydrogenase 4 (HSD17B4), mRNA OS=Homo sapiens PE=2 SV=1 | 79611.6 | 736 | 42.12 | 25 | 25 | 154 | 153 |
| 97 | 48.21 | tr|A0A024QZN4|A0A024QZN4_HUMAN | Vinculin, isoform CRA_c OS=Homo sapiens GN=VCL PE=4 SV=1 | 116721.3 | 1066 | 31.05 | 24 | 24 | 112 | 108 |
| 98 | 48.2 | tr|E9KL44|E9KL44_HUMAN | Epididymis tissue sperm binding protein Li 14m OS=Homo sapiens PE=2 SV=1 | 82999 | 763 | 38.66 | 29 | 29 | 129 | 128 |
| 99 | 48.17 | tr|E1NZA1|E1NZA1_HUMAN | Peroxisome proliferator activated receptor interacting complex protein OS=Homo sapiens GN=PRIC295 PE=2 SV=1 | 292740.9 | 2671 | 15.76 | 33 | 31 | 112 | 111 |
| 100 | 47.6 | tr|V9HWJ0|V9HWJ0_HUMAN | Epididymis secretory sperm binding protein Li 164nA OS=Homo sapiens GN=HEL-S-164nA PE=2 SV=1 | 106873.1 | 944 | 32.42 | 28 | 27 | 221 | 211 |
| 101 | 47.57 | tr|Q59ER5|Q59ER5_HUMAN | WD repeat-containing protein 1 isoform 1 variant (Fragment) OS=Homo sapiens PE=2 SV=1 | 68170.3 | 624 | 47.28 | 24 | 24 | 168 | 168 |
| 102 | 47.36 | tr|V9HW31|V9HW31_HUMAN | ATP synthase subunit beta OS=Homo sapiens GN=HEL-S-271 PE=2 SV=1 | 56559.4 | 529 | 61.63 | 27 | 27 | 460 | 459 |
| 103 | 46.96 | tr|A0A024RDQ0|A0A024RDQ0_HUMAN | Heat shock 105kDa/110kDa protein 1, isoform CRA_a OS=Homo sapiens GN=HSPH1 PE=3 SV=1 | 92115.4 | 814 | 29.61 | 24 | 20 | 193 | 150 |
| 104 | 46.78 | tr|Q59H77|Q59H77_HUMAN | T-complex protein 1 subunit gamma (Fragment) OS=Homo sapiens PE=2 SV=1 | 63575.6 | 577 | 44.71 | 22 | 22 | 179 | 175 |
| 105 | 46.77 | sp|Q8TE68|ES8L1_HUMAN | Epidermal growth factor receptor kinase substrate 8-like protein 1 OS=Homo sapiens GN=EPS8L1 PE=1 SV=3 | 80250.3 | 723 | 39.97 | 23 | 23 | 111 | 111 |
| 106 | 46.28 | tr|A0A0S2Z3Y1|A0A0S2Z3Y1_HUMAN | Lectin galactoside-binding soluble 3 binding protein isoform 1 (Fragment) OS=Homo sapiens GN=LGALS3BP PE=2 SV=1 | 65330.3 | 585 | 42.56 | 25 | 25 | 213 | 212 |
| 107 | 46.02 | sp|P11413|G6PD_HUMAN | Glucose-6-phosphate 1-dehydrogenase OS=Homo sapiens GN=G6PD PE=1 SV=4 | 59256.3 | 515 | 46.6 | 24 | 24 | 157 | 156 |
| 108 | 45.86 | tr|F5H5D3|F5H5D3_HUMAN | Tubulin alpha chain OS=Homo sapiens GN=TUBA1C PE=1 SV=1 | 57729.7 | 519 | 48.17 | 26 | 11 | 781 | 249 |
| 109 | 45.8 | tr|A0A0S2Z4Z9|A0A0S2Z4Z9_HUMAN | Non-POU domain containing octamer-binding isoform 1 (Fragment) OS=Homo sapiens GN=NONO PE=2 SV=1 | 54231.3 | 471 | 43.74 | 24 | 21 | 208 | 192 |
| 110 | 45.78 | tr|Q6IBN1|Q6IBN1_HUMAN | HNRPK protein OS=Homo sapiens GN=HNRPK PE=2 SV=1 | 51027.8 | 464 | 46.34 | 25 | 25 | 402 | 402 |
| 111 | 45.58 | tr|A0A0A0MTS2|A0A0A0MTS2_HUMAN | Glucose-6-phosphate isomerase (Fragment) OS=Homo sapiens GN=GPI PE=1 SV=1 | 64824.7 | 573 | 47.29 | 24 | 24 | 205 | 205 |
| 112 | 45.35 | tr|A0A0D9SFK2|A0A0D9SFK2_HUMAN | Unconventional myosin-XVIIIa OS=Homo sapiens GN=MYO18A PE=1 SV=1 | 231104.6 | 2038 | 16.34 | 29 | 26 | 73 | 61 |
| 113 | 44.81 | sp|Q9HC35|EMAL4_HUMAN | Echinoderm microtubule-associated protein-like 4 OS=Homo sapiens GN=EML4 PE=1 SV=3 | 108915.4 | 981 | 22.43 | 24 | 24 | 123 | 122 |
| 114 | 44.41 | tr|Q53HG7|Q53HG7_HUMAN | Cortactin isoform a variant (Fragment) OS=Homo sapiens PE=1 SV=1 | 61557.9 | 550 | 47.45 | 24 | 24 | 156 | 156 |
| 115 | 44.4 | tr|B9A067|B9A067_HUMAN | MICOS complex subunit MIC60 OS=Homo sapiens GN=IMMT PE=1 SV=2 | 78973 | 711 | 33.9 | 22 | 22 | 109 | 109 |
| 116 | 44.39 | tr|Q53GG0|Q53GG0_HUMAN | Epithelial protein lost in neoplasm beta variant (Fragment) OS=Homo sapiens PE=2 SV=1 | 85253 | 759 | 28.33 | 20 | 20 | 107 | 107 |
| 117 | 44.14 | sp|Q99623|PHB2_HUMAN | Prohibitin-2 OS=Homo sapiens GN=PHB2 PE=1 SV=2 | 33296.1 | 299 | 66.89 | 22 | 22 | 161 | 159 |
| 118 | 43.95 | sp|P39023|RL3_HUMAN | 60S ribosomal protein L3 OS=Homo sapiens GN=RPL3 PE=1 SV=2 | 46108.7 | 403 | 50.87 | 21 | 21 | 114 | 113 |
| 119 | 43.85 | sp|P22626|ROA2_HUMAN | Heterogeneous nuclear ribonucleoproteins A2/B1 OS=Homo sapiens GN=HNRNPA2B1 PE=1 SV=2 | 37429.7 | 353 | 65.16 | 23 | 23 | 296 | 294 |
| 120 | 43.61 | tr|V9HW37|V9HW37_HUMAN | Epididymis secretory protein Li 69 OS=Homo sapiens GN=HEL-S-69 PE=2 SV=1 | 59670.5 | 541 | 60.07 | 25 | 25 | 149 | 144 |
| 121 | 43.42 | tr|A0A024R8V0|A0A024R8V0_HUMAN | Septin 9, isoform CRA_a OS=Homo sapiens GN=SEPT9 PE=3 SV=1 | 63632.9 | 568 | 53.7 | 23 | 23 | 112 | 112 |
| 122 | 43.38 | sp|Q15393|SF3B3_HUMAN | Splicing factor 3B subunit 3 OS=Homo sapiens GN=SF3B3 PE=1 SV=4 | 135576.2 | 1217 | 23.17 | 24 | 24 | 154 | 152 |
| 123 | 43.35 | sp|Q99832|TCPH_HUMAN | T-complex protein 1 subunit eta OS=Homo sapiens GN=CCT7 PE=1 SV=2 | 59366.1 | 543 | 45.86 | 22 | 22 | 169 | 168 |
| 124 | 43.21 | tr|V9HW06|V9HW06_HUMAN | Serine hydroxymethyltransferase OS=Homo sapiens GN=HEL-S-51e PE=2 SV=1 | 55992.4 | 504 | 48.41 | 22 | 21 | 135 | 132 |
| 125 | 42.81 | tr|V9HWG3|V9HWG3_HUMAN | Epididymis secretory protein Li 45 OS=Homo sapiens GN=HEL-S-45 PE=2 SV=1 | 77328.2 | 687 | 35.95 | 21 | 21 | 177 | 175 |
| 126 | 42.36 | tr|K9JA46|K9JA46_HUMAN | Epididymis luminal secretory protein 52 OS=Homo sapiens GN=EL52 PE=2 SV=1 | 84659 | 732 | 46.99 | 48 | 29 | 1034 | 303 |
| 127 | 42.25 | sp|P50991|TCPD_HUMAN | T-complex protein 1 subunit delta OS=Homo sapiens GN=CCT4 PE=1 SV=4 | 57923.6 | 539 | 46.38 | 21 | 21 | 169 | 162 |
| 128 | 41.89 | tr|A0A087X054|A0A087X054_HUMAN | Hypoxia up-regulated protein 1 OS=Homo sapiens GN=HYOU1 PE=1 SV=1 | 104777.8 | 937 | 22.73 | 22 | 22 | 119 | 119 |
| 129 | 41.78 | sp|Q1KMD3|HNRL2_HUMAN | Heterogeneous nuclear ribonucleoprotein U-like protein 2 OS=Homo sapiens GN=HNRNPUL2 PE=1 SV=1 | 85104.2 | 747 | 29.85 | 21 | 21 | 137 | 137 |
| 130 | 41.77 | tr|V9HWJ2|V9HWJ2_HUMAN | Isocitrate dehydrogenase [NADP] OS=Homo sapiens GN=HEL-S-26 PE=2 SV=1 | 46659 | 414 | 56.04 | 22 | 22 | 111 | 110 |
| 131 | 41.04 | tr|V9HWF5|V9HWF5_HUMAN | Peptidyl-prolyl cis-trans isomerase OS=Homo sapiens GN=HEL-S-69p PE=2 SV=1 | 18012.4 | 165 | 93.94 | 22 | 21 | 838 | 819 |
| 132 | 41.02 | tr|A0A0S2Z3L2|A0A0S2Z3L2_HUMAN | ATPase Ca++ transporting cardiac muscle slow twitch 2 isoform 1 (Fragment) OS=Homo sapiens GN=ATP2A2 PE=2 SV=1 | 114755.8 | 1042 | 20.44 | 20 | 15 | 117 | 94 |
| 133 | 40.79 | sp|Q9Y678|COPG1_HUMAN | Coatomer subunit gamma-1 OS=Homo sapiens GN=COPG1 PE=1 SV=1 | 97717.3 | 874 | 30.09 | 21 | 19 | 155 | 148 |
| 134 | 40.74 | tr|Q6IPJ9|Q6IPJ9_HUMAN | Ladinin-1 OS=Homo sapiens GN=LAD1 PE=2 SV=1 | 57101.3 | 517 | 39.26 | 19 | 19 | 95 | 95 |
| 135 | 40.51 | sp|P14866|HNRPL_HUMAN | Heterogeneous nuclear ribonucleoprotein L OS=Homo sapiens GN=HNRNPL PE=1 SV=2 | 64132.5 | 589 | 46.35 | 22 | 21 | 235 | 231 |
| 136 | 40.15 | tr|Q53YD7|Q53YD7_HUMAN | EEF1G protein OS=Homo sapiens GN=EEF1G PE=2 SV=1 | 50118.4 | 437 | 35.24 | 20 | 20 | 181 | 180 |
| 137 | 39.74 | sp|O43143|DHX15_HUMAN | Pre-mRNA-splicing factor ATP-dependent RNA helicase DHX15 OS=Homo sapiens GN=DHX15 PE=1 SV=2 | 90932 | 795 | 25.66 | 20 | 20 | 135 | 135 |
| 138 | 39.66 | tr|D3DPU2|D3DPU2_HUMAN | Adenylyl cyclase-associated protein OS=Homo sapiens GN=CAP1 PE=2 SV=1 | 51672.7 | 475 | 54.32 | 19 | 19 | 161 | 161 |
| 139 | 39.59 | tr|V9HWH7|V9HWH7_HUMAN | Epididymis secretory sperm binding protein Li 70p OS=Homo sapiens GN=HEL-S-70p PE=2 SV=1 | 64615.3 | 592 | 51.18 | 22 | 4 | 157 | 36 |
| 140 | 39.39 | sp|P23396|RS3_HUMAN | 40S ribosomal protein S3 OS=Homo sapiens GN=RPS3 PE=1 SV=2 | 26688.1 | 243 | 73.25 | 20 | 20 | 312 | 311 |
| 141 | 39.04 | sp|O75533|SF3B1_HUMAN | Splicing factor 3B subunit 1 OS=Homo sapiens GN=SF3B1 PE=1 SV=3 | 145829.1 | 1304 | 18.71 | 21 | 21 | 52 | 52 |
| 142 | 38.93 | tr|A0A024RCN6|A0A024RCN6_HUMAN | Valyl-tRNA synthetase, isoform CRA_a OS=Homo sapiens GN=VARS PE=3 SV=1 | 140474.8 | 1264 | 21.2 | 21 | 21 | 108 | 104 |
| 143 | 38.92 | sp|Q03252|LMNB2_HUMAN | Lamin-B2 OS=Homo sapiens GN=LMNB2 PE=1 SV=4 | 69947.7 | 620 | 35 | 25 | 21 | 100 | 72 |
| 144 | 38.47 | tr|E7EUU4|E7EUU4_HUMAN | Eukaryotic translation initiation factor 4 gamma 1 OS=Homo sapiens GN=EIF4G1 PE=1 SV=1 | 171640.1 | 1560 | 13.53 | 21 | 20 | 94 | 88 |
| 145 | 38.4 | sp|P13010|XRCC5_HUMAN | X-ray repair cross-complementing protein 5 OS=Homo sapiens GN=XRCC5 PE=1 SV=3 | 82703.8 | 732 | 30.74 | 20 | 20 | 134 | 134 |
| 146 | 38.22 | tr|F4ZW66|F4ZW66_HUMAN | NF110b OS=Homo sapiens PE=2 SV=1 | 95777.4 | 898 | 24.28 | 19 | 18 | 134 | 130 |
| 147 | 38.1 | tr|V9HWE0|V9HWE0_HUMAN | Annexin OS=Homo sapiens GN=HEL-S-7 PE=2 SV=1 | 35936.4 | 320 | 47.5 | 21 | 20 | 226 | 224 |
| 148 | 37.86 | sp|P27816|MAP4_HUMAN | Microtubule-associated protein 4 OS=Homo sapiens GN=MAP4 PE=1 SV=3 | 121003.8 | 1152 | 22.66 | 21 | 14 | 70 | 51 |
| 149 | 37.84 | tr|B2R4R0|B2R4R0_HUMAN | Histone H4 OS=Homo sapiens GN=HIST1H4L PE=2 SV=1 | 11367.3 | 103 | 72.82 | 21 | 21 | 886 | 885 |
| 150 | 37.79 | tr|Q96FS1|Q96FS1_HUMAN | CTNND1 protein (Fragment) OS=Homo sapiens GN=CTNND1 PE=2 SV=2 | 92387 | 830 | 34.1 | 19 | 19 | 105 | 104 |
| 151 | 37.76 | sp|P49411|EFTU_HUMAN | Elongation factor Tu, mitochondrial OS=Homo sapiens GN=TUFM PE=1 SV=2 | 49541.1 | 452 | 41.81 | 19 | 19 | 221 | 217 |
| 152 | 37.72 | tr|B4E0E1|B4E0E1_HUMAN | Poly [ADP-ribose] polymerase OS=Homo sapiens PE=2 SV=1 | 111125.8 | 993 | 24.67 | 18 | 18 | 139 | 139 |
| 153 | 37.62 | sp|Q13263|TIF1B_HUMAN | Transcription intermediary factor 1-beta OS=Homo sapiens GN=TRIM28 PE=1 SV=5 | 88548.8 | 835 | 34.49 | 18 | 18 | 121 | 121 |
| 154 | 37.45 | sp|P49588|SYAC_HUMAN | Alanine--tRNA ligase, cytoplasmic OS=Homo sapiens GN=AARS PE=1 SV=2 | 106809.5 | 968 | 22.11 | 18 | 18 | 137 | 137 |
| 155 | 37.22 | tr|B2R491|B2R491_HUMAN | 40S ribosomal protein S4 OS=Homo sapiens GN=RPS4X PE=2 SV=1 | 29597.5 | 263 | 52.09 | 19 | 19 | 108 | 108 |
| 156 | 37.17 | sp|O75643|U520_HUMAN | U5 small nuclear ribonucleoprotein 200 kDa helicase OS=Homo sapiens GN=SNRNP200 PE=1 SV=2 | 244505.5 | 2136 | 14.84 | 24 | 23 | 98 | 97 |
| 157 | 37.05 | tr|Q53GX7|Q53GX7_HUMAN | Threonyl-tRNA synthetase variant (Fragment) OS=Homo sapiens PE=2 SV=1 | 83444.5 | 723 | 27.52 | 18 | 17 | 97 | 92 |
| 158 | 36.97 | sp|Q15084|PDIA6_HUMAN | Protein disulfide-isomerase A6 OS=Homo sapiens GN=PDIA6 PE=1 SV=1 | 48120.9 | 440 | 44.32 | 19 | 19 | 242 | 242 |
| 159 | 36.84 | sp|Q16531|DDB1_HUMAN | DNA damage-binding protein 1 OS=Homo sapiens GN=DDB1 PE=1 SV=1 | 126966.9 | 1140 | 23.33 | 22 | 22 | 77 | 75 |
| 160 | 36.61 | sp|P23526|SAHH_HUMAN | Adenosylhomocysteinase OS=Homo sapiens GN=AHCY PE=1 SV=4 | 47715.7 | 432 | 35.42 | 19 | 19 | 207 | 206 |
| 161 | 36.5 | sp|Q9NR30|DDX21_HUMAN | Nucleolar RNA helicase 2 OS=Homo sapiens GN=DDX21 PE=1 SV=5 | 87343.9 | 783 | 32.06 | 19 | 17 | 112 | 97 |
| 162 | 36.49 | sp|P26599|PTBP1_HUMAN | Polypyrimidine tract-binding protein 1 OS=Homo sapiens GN=PTBP1 PE=1 SV=1 | 57220.9 | 531 | 45.57 | 20 | 15 | 146 | 111 |
| 163 | 36.44 | sp|O95831|AIFM1_HUMAN | Apoptosis-inducing factor 1, mitochondrial OS=Homo sapiens GN=AIFM1 PE=1 SV=1 | 66900.1 | 613 | 39.15 | 19 | 19 | 103 | 103 |
| 164 | 36.41 | tr|A0A0D9SGC1|A0A0D9SGC1_HUMAN | Unconventional myosin-VI OS=Homo sapiens GN=MYO6 PE=1 SV=1 | 149634.7 | 1294 | 17 | 19 | 3 | 70 | 8 |
| 165 | 36.37 | sp|Q06830|PRDX1_HUMAN | Peroxiredoxin-1 OS=Homo sapiens GN=PRDX1 PE=1 SV=1 | 22110.2 | 199 | 78.89 | 19 | 14 | 632 | 375 |
| 166 | 36.31 | sp|Q15046|SYK_HUMAN | Lysine--tRNA ligase OS=Homo sapiens GN=KARS PE=1 SV=3 | 68047.5 | 597 | 37.35 | 19 | 19 | 69 | 69 |
| 167 | 36.26 | sp|Q9Y230|RUVB2_HUMAN | RuvB-like 2 OS=Homo sapiens GN=RUVBL2 PE=1 SV=3 | 51156.1 | 463 | 51.62 | 20 | 20 | 175 | 175 |
| 168 | 36.25 | tr|A0A140VJW5|A0A140VJW5_HUMAN | Testicular tissue protein Li 192 OS=Homo sapiens PE=2 SV=1 | 57135.8 | 501 | 35.73 | 19 | 19 | 88 | 88 |
| 169 | 36.2 | tr|V9HWI5|V9HWI5_HUMAN | Cofilin 1 (Non-muscle), isoform CRA_b OS=Homo sapiens GN=HEL-S-15 PE=2 SV=1 | 18502.3 | 166 | 95.78 | 21 | 19 | 323 | 309 |
| 170 | 36.15 | tr|Q59EK6|Q59EK6_HUMAN | TNF receptor-associated protein 1 variant (Fragment) OS=Homo sapiens PE=3 SV=1 | 79992 | 703 | 31.29 | 20 | 20 | 134 | 130 |
| 171 | 36.11 | sp|P12429|ANXA3_HUMAN | Annexin A3 OS=Homo sapiens GN=ANXA3 PE=1 SV=3 | 36374.8 | 323 | 57.89 | 20 | 20 | 176 | 176 |
| 172 | 35.97 | tr|A0A024R1N4|A0A024R1N4_HUMAN | X-ray repair complementing defective repair in Chinese hamster cells 6 (Ku autoantigen, 70kDa), isoform CRA_a OS=Homo sapiens GN=XRCC6 PE=4 SV=1 | 69842.4 | 609 | 29.56 | 18 | 18 | 112 | 111 |
| 173 | 35.73 | sp|P38159|RBMX_HUMAN | RNA-binding motif protein, X chromosome OS=Homo sapiens GN=RBMX PE=1 SV=3 | 42331.4 | 391 | 36.57 | 16 | 16 | 103 | 103 |
| 174 | 35.72 | tr|A0A024RCL8|A0A024RCL8_HUMAN | Histone H2B OS=Homo sapiens GN=HIST1H2BK PE=3 SV=1 | 13890 | 126 | 83.33 | 22 | 2 | 880 | 10 |
| 175 | 35.65 | tr|Q5U077|Q5U077_HUMAN | L-lactate dehydrogenase OS=Homo sapiens GN=LDHB PE=2 SV=1 | 36638.2 | 334 | 53.89 | 22 | 19 | 333 | 294 |
| 176 | 35.45 | tr|A0A140VK53|A0A140VK53_HUMAN | Testicular secretory protein Li 53 OS=Homo sapiens PE=2 SV=1 | 299611 | 2752 | 10.25 | 19 | 19 | 110 | 109 |
| 177 | 35.44 | sp|P61604|CH10_HUMAN | 10 kDa heat shock protein, mitochondrial OS=Homo sapiens GN=HSPE1 PE=1 SV=2 | 10931.6 | 102 | 93.14 | 18 | 8 | 529 | 203 |
| 178 | 35.39 | tr|V9HW88|V9HW88_HUMAN | Calreticulin, isoform CRA_b OS=Homo sapiens GN=HEL-S-99n PE=2 SV=1 | 48141.2 | 417 | 46.28 | 18 | 17 | 179 | 178 |
| 179 | 35.21 | sp|P54886|P5CS_HUMAN | Delta-1-pyrroline-5-carboxylate synthase OS=Homo sapiens GN=ALDH18A1 PE=1 SV=2 | 87301.5 | 795 | 27.55 | 19 | 19 | 110 | 108 |
| 180 | 35.15 | sp|P12270|TPR_HUMAN | Nucleoprotein TPR OS=Homo sapiens GN=TPR PE=1 SV=3 | 267291.1 | 2363 | 11.43 | 22 | 21 | 67 | 66 |
| 181 | 35.05 | sp|Q9ULC5|ACSL5_HUMAN | Long-chain-fatty-acid--CoA ligase 5 OS=Homo sapiens GN=ACSL5 PE=1 SV=1 | 75990.1 | 683 | 36.31 | 18 | 17 | 139 | 138 |
| 182 | 34.96 | tr|A0A140VK56|A0A140VK56_HUMAN | Transaldolase OS=Homo sapiens PE=2 SV=1 | 37539.7 | 337 | 37.69 | 17 | 17 | 294 | 293 |
| 183 | 34.81 | sp|Q01813|PFKAP_HUMAN | ATP-dependent 6-phosphofructokinase, platelet type OS=Homo sapiens GN=PFKP PE=1 SV=2 | 85595.4 | 784 | 21.94 | 17 | 13 | 73 | 45 |
| 184 | 34.76 | tr|A2RUM7|A2RUM7_HUMAN | Ribosomal protein L5 OS=Homo sapiens GN=RPL5 PE=2 SV=1 | 34362.4 | 297 | 48.15 | 17 | 17 | 93 | 93 |
| 185 | 34.72 | tr|V9HW38|V9HW38_HUMAN | Epididymis secretory protein Li 106 OS=Homo sapiens GN=HEL-S-106 PE=2 SV=1 | 56048.7 | 519 | 50.1 | 18 | 18 | 142 | 140 |
| 186 | 34.43 | sp|Q9Y265|RUVB1_HUMAN | RuvB-like 1 OS=Homo sapiens GN=RUVBL1 PE=1 SV=1 | 50227.6 | 456 | 40.35 | 17 | 17 | 150 | 150 |
| 187 | 34.3 | sp|Q9NVI7|ATD3A_HUMAN | ATPase family AAA domain-containing protein 3A OS=Homo sapiens GN=ATAD3A PE=1 SV=2 | 71368.6 | 634 | 31.7 | 19 | 19 | 72 | 66 |
| 188 | 34.28 | sp|Q6P2Q9|PRP8_HUMAN | Pre-mRNA-processing-splicing factor 8 OS=Homo sapiens GN=PRPF8 PE=1 SV=2 | 273598.4 | 2335 | 11.86 | 20 | 20 | 77 | 77 |
| 189 | 34.21 | tr|B0YIW6|B0YIW6_HUMAN | Archain 1, isoform CRA_a OS=Homo sapiens GN=ARCN1 PE=1 SV=1 | 61625.8 | 552 | 44.2 | 20 | 20 | 67 | 65 |
| 190 | 34.17 | tr|B2R5W2|B2R5W2_HUMAN | Heterogeneous nuclear ribonucleoproteins C1/C2 OS=Homo sapiens GN=HNRNPC PE=1 SV=1 | 31948 | 290 | 54.83 | 19 | 18 | 188 | 185 |
| 191 | 34.15 | sp|Q9NY33|DPP3_HUMAN | Dipeptidyl peptidase 3 OS=Homo sapiens GN=DPP3 PE=1 SV=2 | 82588.4 | 737 | 36.23 | 17 | 17 | 99 | 99 |
| 192 | 34.13 | tr|A0A140VK93|A0A140VK93_HUMAN | Adenylate kinase 2, mitochondrial OS=Homo sapiens GN=AK2 PE=2 SV=1 | 26477.4 | 239 | 68.2 | 18 | 18 | 175 | 165 |
| 193 | 34.12 | tr|Q6IB91|Q6IB91_HUMAN | PCK2 protein OS=Homo sapiens GN=PCK2 PE=2 SV=1 | 70696.4 | 640 | 32.81 | 19 | 19 | 91 | 90 |
| 194 | 33.93 | tr|V9HWD6|V9HWD6_HUMAN | Epididymis secretory protein Li 1 OS=Homo sapiens GN=HEL-S-1 PE=2 SV=1 | 28082.2 | 246 | 64.23 | 24 | 14 | 365 | 126 |
| 195 | 33.92 | tr|B4DLV7|B4DLV7_HUMAN | cDNA FLJ60299, highly similar to Rab GDP dissociation inhibitor beta OS=Homo sapiens PE=2 SV=1 | 51153.5 | 449 | 47.22 | 16 | 10 | 148 | 115 |
| 196 | 33.71 | tr|V9HW69|V9HW69_HUMAN | Epididymis secretory protein Li 66 OS=Homo sapiens GN=HEL-S-66 PE=2 SV=1 | 38498.3 | 348 | 47.99 | 17 | 17 | 159 | 159 |
| 197 | 33.69 | tr|E9PMS6|E9PMS6_HUMAN | LIM domain only protein 7 OS=Homo sapiens GN=LMO7 PE=1 SV=1 | 145422.1 | 1275 | 17.57 | 18 | 18 | 45 | 45 |
| 198 | 33.58 | sp|P35606|COPB2_HUMAN | Coatomer subunit beta' OS=Homo sapiens GN=COPB2 PE=1 SV=2 | 102486.4 | 906 | 27.04 | 18 | 18 | 86 | 86 |
| 199 | 33.54 | tr|B3KNP8|B3KNP8_HUMAN | cDNA FLJ30111 fis, clone BNGH42000360, highly similar to 3-ketoacyl-CoA thiolase, mitochondrial (EC 2.3.1.16) OS=Homo sapiens PE=2 SV=1 | 41895.8 | 397 | 49.87 | 18 | 18 | 83 | 83 |
| 200 | 33.43 | sp|Q15019|SEPT2_HUMAN | Septin-2 OS=Homo sapiens GN=SEPT2 PE=1 SV=1 | 41487.2 | 361 | 57.34 | 17 | 17 | 145 | 145 |
| 201 | 33.41 | sp|Q13011|ECH1_HUMAN | Delta(3,5)-Delta(2,4)-dienoyl-CoA isomerase, mitochondrial OS=Homo sapiens GN=ECH1 PE=1 SV=2 | 35815.8 | 328 | 56.71 | 17 | 17 | 140 | 140 |
| 202 | 33.39 | tr|Q8N5Z7|Q8N5Z7_HUMAN | 60S ribosomal protein L6 OS=Homo sapiens GN=RPL6 PE=2 SV=1 | 32725.8 | 288 | 46.18 | 17 | 17 | 149 | 142 |
| 203 | 33.38 | tr|Q6FHG5|Q6FHG5_HUMAN | Gamma-synuclein OS=Homo sapiens GN=SNCG PE=2 SV=1 | 13330.8 | 127 | 95.28 | 18 | 18 | 367 | 364 |
| 204 | 33.34 | tr|B2RDW1|B2RDW1_HUMAN | Epididymis luminal protein 112 OS=Homo sapiens GN=RPS27A PE=2 SV=1 | 17964.8 | 156 | 73.08 | 18 | 7 | 504 | 186 |
| 205 | 33.19 | tr|Q3B7A7|Q3B7A7_HUMAN | Trifunctional purine biosynthetic protein adenosine-3 OS=Homo sapiens GN=GART PE=2 SV=1 | 107722.3 | 1010 | 25.05 | 18 | 18 | 71 | 71 |
| 206 | 33.07 | tr|A8K088|A8K088_HUMAN | cDNA FLJ78614, highly similar to Homo sapiens eukaryotic translation initiation factor 4A, isoform 1 (EIF4A1), mRNA OS=Homo sapiens PE=2 SV=1 | 46125.5 | 406 | 38.18 | 15 | 9 | 159 | 46 |
| 207 | 32.9 | tr|A0A024RB85|A0A024RB85_HUMAN | Proliferation-associated 2G4, 38kDa, isoform CRA_a OS=Homo sapiens GN=PA2G4 PE=4 SV=1 | 43786.6 | 394 | 41.88 | 17 | 17 | 119 | 119 |
| 208 | 32.8 | tr|G8JLB6|G8JLB6_HUMAN | Heterogeneous nuclear ribonucleoprotein H OS=Homo sapiens GN=HNRNPH1 PE=1 SV=1 | 51229.6 | 472 | 42.37 | 16 | 8 | 186 | 87 |
| 209 | 32.51 | tr|A0A0S2Z3L0|A0A0S2Z3L0_HUMAN | Electron-transfer-flavoprotein alpha polypeptide isoform 1 (Fragment) OS=Homo sapiens GN=ETFA PE=2 SV=1 | 35079.2 | 333 | 68.17 | 16 | 16 | 255 | 253 |
| 210 | 32.45 | tr|Q6P6D7|Q6P6D7_HUMAN | Phosphoglycerate mutase OS=Homo sapiens GN=PGAM1 PE=2 SV=1 | 28819.7 | 254 | 57.87 | 16 | 16 | 211 | 211 |
| 211 | 32.13 | sp|Q27J81|INF2_HUMAN | Inverted formin-2 OS=Homo sapiens GN=INF2 PE=1 SV=2 | 135622.7 | 1249 | 23.22 | 17 | 17 | 55 | 54 |
| 212 | 32.09 | sp|P41250|SYG_HUMAN | Glycine--tRNA ligase OS=Homo sapiens GN=GARS PE=1 SV=3 | 83164.8 | 739 | 26.12 | 19 | 19 | 67 | 66 |
| 213 | 32.07 | sp|P17844|DDX5_HUMAN | Probable ATP-dependent RNA helicase DDX5 OS=Homo sapiens GN=DDX5 PE=1 SV=1 | 69147.6 | 614 | 39.41 | 24 | 17 | 173 | 77 |
| 214 | 32.07 | tr|A0A024RAZ7|A0A024RAZ7_HUMAN | Heterogeneous nuclear ribonucleoprotein A1, isoform CRA_b OS=Homo sapiens GN=HNRPA1 PE=4 SV=1 | 38746.7 | 372 | 48.92 | 17 | 17 | 175 | 159 |
| 215 | 32.03 | sp|P23284|PPIB_HUMAN | Peptidyl-prolyl cis-trans isomerase B OS=Homo sapiens GN=PPIB PE=1 SV=2 | 23742.4 | 216 | 68.98 | 18 | 18 | 174 | 173 |
| 216 | 31.87 | tr|V9HW83|V9HW83_HUMAN | Aldehyde dehydrogenase 1 family, member A1, isoform CRA_a OS=Homo sapiens GN=HEL-S-53e PE=2 SV=1 | 54861.4 | 501 | 36.73 | 17 | 16 | 248 | 232 |
| 217 | 31.65 | sp|P38117|ETFB_HUMAN | Electron transfer flavoprotein subunit beta OS=Homo sapiens GN=ETFB PE=1 SV=3 | 27843.4 | 255 | 60 | 15 | 15 | 244 | 244 |
| 218 | 31.64 | tr|A0A024RBH2|A0A024RBH2_HUMAN | Cytoskeleton-associated protein 4, isoform CRA_c OS=Homo sapiens GN=CKAP4 PE=4 SV=1 | 66022 | 602 | 35.71 | 17 | 16 | 80 | 75 |
| 219 | 31.63 | sp|P30084|ECHM_HUMAN | Enoyl-CoA hydratase, mitochondrial OS=Homo sapiens GN=ECHS1 PE=1 SV=4 | 31387.1 | 290 | 55.52 | 16 | 16 | 202 | 201 |
| 221 | 31.25 | sp|P17987|TCPA_HUMAN | T-complex protein 1 subunit alpha OS=Homo sapiens GN=TCP1 PE=1 SV=1 | 60342.9 | 556 | 34.71 | 17 | 16 | 219 | 207 |
| 222 | 31.21 | tr|B4E266|B4E266_HUMAN | cDNA FLJ58466, highly similar to Leucyl-tRNA synthetase, cytoplasmic (EC 6.1.1.4) OS=Homo sapiens PE=2 SV=1 | 129187.3 | 1130 | 18.41 | 17 | 17 | 74 | 74 |
| 223 | 31.17 | tr|A8KAK1|A8KAK1_HUMAN | cDNA FLJ77398, highly similar to Homo sapiens UDP-glucose ceramide glucosyltransferase-like 1, transcript variant 2, mRNA OS=Homo sapiens PE=2 SV=1 | 175005.4 | 1531 | 13.26 | 16 | 16 | 57 | 57 |
| 224 | 31.08 | tr|V9HWH1|V9HWH1_HUMAN | Epididymis luminal protein 57 OS=Homo sapiens GN=HEL57 PE=2 SV=1 | 42741.4 | 379 | 43.27 | 16 | 16 | 132 | 131 |
| 225 | 31.07 | tr|V9HW98|V9HW98_HUMAN | Epididymis luminal protein 2 OS=Homo sapiens GN=HEL2 PE=2 SV=1 | 29173.6 | 255 | 69.02 | 19 | 16 | 274 | 191 |
| 226 | 31.05 | tr|Q53G72|Q53G72_HUMAN | B-cell receptor-associated protein 31 variant (Fragment) OS=Homo sapiens PE=2 SV=1 | 27931.3 | 246 | 50 | 14 | 14 | 104 | 104 |
| 227 | 30.99 | tr|E9PPJ0|E9PPJ0_HUMAN | Splicing factor 3B subunit 2 OS=Homo sapiens GN=SF3B2 PE=1 SV=1 | 98169.7 | 878 | 29.38 | 19 | 18 | 49 | 47 |
| 228 | 30.93 | tr|Q6NVC0|Q6NVC0_HUMAN | SLC25A5 protein (Fragment) OS=Homo sapiens GN=SLC25A5 PE=2 SV=1 | 35293.6 | 323 | 38.39 | 16 | 8 | 194 | 150 |
| 229 | 30.8 | tr|B4DEA8|B4DEA8_HUMAN | cDNA FLJ56425, highly similar to Very-long-chain specific acyl-CoAdehydrogenase, mitochondrial (EC 1.3.99.-) OS=Homo sapiens PE=2 SV=1 | 75209.8 | 701 | 30.24 | 15 | 15 | 88 | 88 |
| 230 | 30.77 | sp|P21796|VDAC1_HUMAN | Voltage-dependent anion-selective channel protein 1 OS=Homo sapiens GN=VDAC1 PE=1 SV=2 | 30772.4 | 283 | 67.84 | 17 | 16 | 145 | 133 |
| 231 | 30.52 | sp|Q9NZB2|F120A_HUMAN | Constitutive coactivator of PPAR-gamma-like protein 1 OS=Homo sapiens GN=FAM120A PE=1 SV=2 | 121886.7 | 1118 | 20.48 | 15 | 15 | 67 | 66 |
| 232 | 30.44 | sp|P62081|RS7_HUMAN | 40S ribosomal protein S7 OS=Homo sapiens GN=RPS7 PE=1 SV=1 | 22126.7 | 194 | 63.92 | 15 | 15 | 112 | 112 |
| 233 | 30.39 | tr|A0A0D9SF53|A0A0D9SF53_HUMAN | ATP-dependent RNA helicase DDX3X OS=Homo sapiens GN=DDX3X PE=1 SV=1 | 81476.3 | 733 | 25.24 | 20 | 18 | 105 | 56 |
| 234 | 30.33 | tr|Q5U0I6|Q5U0I6_HUMAN | H.sapiens ras-related Hrab1A protein OS=Homo sapiens GN=RAB1A PE=2 SV=1 | 22677.6 | 205 | 70.73 | 18 | 8 | 221 | 58 |
| 235 | 30.29 | tr|A0A024R233|A0A024R233_HUMAN | Tight junction protein 2 (Zona occludens 2), isoform CRA_a OS=Homo sapiens GN=TJP2 PE=4 SV=1 | 133970.9 | 1190 | 20.34 | 19 | 19 | 67 | 66 |
| 236 | 30.15 | tr|A0A024RD93|A0A024RD93_HUMAN | Phosphoribosylaminoimidazole carboxylase, phosphoribosylaminoimidazole succinocarboxamide synthetase, isoform CRA_c OS=Homo sapiens GN=PAICS PE=3 SV=1 | 47078.8 | 425 | 28.94 | 15 | 15 | 164 | 164 |
| 237 | 29.94 | tr|B5BUB5|B5BUB5_HUMAN | Autoantigen La (Fragment) OS=Homo sapiens GN=SSB PE=2 SV=1 | 46866.8 | 408 | 34.31 | 16 | 16 | 68 | 68 |
| 238 | 29.81 | tr|Q53SY7|Q53SY7_HUMAN | Putative uncharacterized protein CAD (Fragment) OS=Homo sapiens GN=CAD PE=3 SV=1 | 235175.8 | 2151 | 8.787 | 17 | 17 | 57 | 57 |
| 239 | 29.7 | tr|A0A140VK29|A0A140VK29_HUMAN | Testicular secretory protein Li 29 OS=Homo sapiens PE=2 SV=1 | 61332.7 | 563 | 34.28 | 15 | 15 | 51 | 51 |
| 240 | 29.64 | sp|P41091|IF2G_HUMAN | Eukaryotic translation initiation factor 2 subunit 3 OS=Homo sapiens GN=EIF2S3 PE=1 SV=3 | 51109.1 | 472 | 32.42 | 15 | 15 | 76 | 76 |
| 241 | 29.62 | tr|A0A024R814|A0A024R814_HUMAN | Ribosomal protein L7, isoform CRA_a OS=Homo sapiens GN=RPL7 PE=4 SV=1 | 30438 | 259 | 46.72 | 16 | 16 | 146 | 145 |
| 242 | 29.57 | tr|A0A140VK27|A0A140VK27_HUMAN | Leukotriene A(4) hydrolase OS=Homo sapiens PE=2 SV=1 | 69284.6 | 611 | 30.61 | 16 | 16 | 80 | 80 |
| 243 | 29.57 | tr|B4DV79|B4DV79_HUMAN | Eukaryotic translation initiation factor 3 subunit B OS=Homo sapiens GN=EIF3B PE=2 SV=1 | 85134.2 | 738 | 25.75 | 15 | 15 | 74 | 74 |
| 244 | 29.56 | sp|Q8NBS9|TXND5_HUMAN | Thioredoxin domain-containing protein 5 OS=Homo sapiens GN=TXNDC5 PE=1 SV=2 | 47628.5 | 432 | 46.76 | 18 | 15 | 99 | 88 |
| 245 | 29.51 | sp|Q9H0A0|NAT10_HUMAN | RNA cytidine acetyltransferase OS=Homo sapiens GN=NAT10 PE=1 SV=2 | 115728.8 | 1025 | 16.39 | 15 | 14 | 53 | 51 |
| 246 | 29.48 | tr|Q53ZR1|Q53ZR1_HUMAN | Bumetanide-sensitive Na-K-Cl cotransporter OS=Homo sapiens GN=SLC12A2 PE=2 SV=1 | 131445.8 | 1212 | 14.19 | 15 | 15 | 122 | 121 |
| 247 | 29.46 | tr|A0A024RAC5|A0A024RAC5_HUMAN | Regulator of chromosome condensation 2, isoform CRA_a OS=Homo sapiens GN=RCC2 PE=4 SV=1 | 56084.1 | 522 | 36.02 | 14 | 14 | 77 | 77 |
| 248 | 29.43 | sp|Q16881|TRXR1_HUMAN | Thioredoxin reductase 1, cytoplasmic OS=Homo sapiens GN=TXNRD1 PE=1 SV=3 | 70905.6 | 649 | 34.05 | 15 | 15 | 80 | 80 |
| 249 | 29.38 | sp|P34932|HSP74_HUMAN | Heat shock 70 kDa protein 4 OS=Homo sapiens GN=HSPA4 PE=1 SV=4 | 94330.2 | 840 | 27.98 | 20 | 17 | 155 | 117 |
| 250 | 29.32 | sp|Q14683|SMC1A_HUMAN | Structural maintenance of chromosomes protein 1A OS=Homo sapiens GN=SMC1A PE=1 SV=2 | 143231.9 | 1233 | 17.03 | 18 | 18 | 37 | 36 |
| 251 | 29.27 | sp|O43175|SERA_HUMAN | D-3-phosphoglycerate dehydrogenase OS=Homo sapiens GN=PHGDH PE=1 SV=4 | 56650 | 533 | 33.58 | 19 | 19 | 88 | 83 |
| 252 | 29.23 | sp|Q13177|PAK2_HUMAN | Serine/threonine-protein kinase PAK 2 OS=Homo sapiens GN=PAK2 PE=1 SV=3 | 58042.1 | 524 | 37.6 | 15 | 12 | 85 | 62 |
| 253 | 29.14 | sp|O60832|DKC1_HUMAN | H/ACA ribonucleoprotein complex subunit 4 OS=Homo sapiens GN=DKC1 PE=1 SV=3 | 57673.7 | 514 | 26.26 | 16 | 16 | 52 | 52 |
| 254 | 29.13 | tr|Q8N9M2|Q8N9M2_HUMAN | cDNA FLJ36887 fis, clone BNGH42005504, highly similar to 26S PROTEASOME REGULATORY SUBUNIT S3 OS=Homo sapiens PE=2 SV=1 | 59716.6 | 521 | 31.67 | 15 | 15 | 57 | 57 |
| 255 | 29.11 | tr|A0A140VJZ1|A0A140VJZ1_HUMAN | Testicular tissue protein Li 218 OS=Homo sapiens PE=2 SV=1 | 95785.4 | 858 | 18.41 | 15 | 15 | 58 | 57 |
| 256 | 29.09 | tr|B5MDF5|B5MDF5_HUMAN | GTP-binding nuclear protein Ran OS=Homo sapiens GN=RAN PE=1 SV=1 | 26224 | 233 | 47.21 | 15 | 15 | 149 | 148 |
| 257 | 28.95 | sp|P07384|CAN1_HUMAN | Calpain-1 catalytic subunit OS=Homo sapiens GN=CAPN1 PE=1 SV=1 | 81889.3 | 714 | 25.49 | 16 | 16 | 127 | 127 |
| 258 | 28.91 | tr|V9HWE9|V9HWE9_HUMAN | Epididymis secretory protein Li 22 OS=Homo sapiens GN=HEL-S-22 PE=2 SV=1 | 23355.6 | 210 | 72.86 | 15 | 11 | 623 | 507 |
| 259 | 28.81 | tr|Q8IXJ3|Q8IXJ3_HUMAN | Small nuclear ribonucleoprotein component OS=Homo sapiens GN=SNRP116 PE=2 SV=1 | 95370.9 | 850 | 28.24 | 18 | 17 | 72 | 58 |
| 260 | 28.8 | sp|P50914|RL14_HUMAN | 60S ribosomal protein L14 OS=Homo sapiens GN=RPL14 PE=1 SV=4 | 23431.7 | 215 | 48.84 | 16 | 5 | 109 | 28 |
| 261 | 28.77 | tr|A0A024QZ30|A0A024QZ30_HUMAN | Succinate dehydrogenase [ubiquinone] flavoprotein subunit, mitochondrial OS=Homo sapiens GN=SDHA PE=3 SV=1 | 72691 | 664 | 32.08 | 15 | 15 | 39 | 39 |
| 262 | 28.62 | sp|Q14789|GOGB1_HUMAN | Golgin subfamily B member 1 OS=Homo sapiens GN=GOLGB1 PE=1 SV=2 | 376016.6 | 3259 | 7.855 | 21 | 18 | 59 | 53 |
| 263 | 28.59 | tr|B7Z4B2|B7Z4B2_HUMAN | cDNA FLJ56108, highly similar to Puromycin-sensitive aminopeptidase (EC 3.4.11.-) OS=Homo sapiens PE=2 SV=1 | 92865.2 | 825 | 16 | 15 | 15 | 44 | 44 |
| 264 | 28.58 | tr|G3V5Z7|G3V5Z7_HUMAN | Proteasome subunit alpha type OS=Homo sapiens GN=PSMA6 PE=1 SV=1 | 28147.1 | 252 | 52.78 | 14 | 14 | 106 | 106 |
| 265 | 28.56 | sp|P37802|TAGL2_HUMAN | Transgelin-2 OS=Homo sapiens GN=TAGLN2 PE=1 SV=3 | 22391.4 | 199 | 66.83 | 14 | 14 | 330 | 329 |
| 266 | 28.5 | tr|B2RAU8|B2RAU8_HUMAN | cDNA, FLJ95131, highly similar to Homo sapiens nucleolar and coiled-body phosphoprotein 1 (NOLC1), mRNA OS=Homo sapiens PE=2 SV=1 | 73603.4 | 699 | 21.75 | 15 | 15 | 56 | 56 |
| 267 | 28.4 | tr|V9HWB5|V9HWB5_HUMAN | Epididymis secretory sperm binding protein Li 66p OS=Homo sapiens GN=HEL-S-66p PE=2 SV=1 | 32659.8 | 289 | 59.86 | 14 | 14 | 102 | 98 |
| 268 | 28.28 | tr|Q4W4Y1|Q4W4Y1_HUMAN | Dopamine receptor interacting protein 4 OS=Homo sapiens GN=DRIP4 PE=2 SV=1 | 96078.4 | 868 | 18.43 | 15 | 15 | 65 | 65 |
| 269 | 28.22 | sp|P13645|K1C10_HUMAN | Keratin, type I cytoskeletal 10 OS=Homo sapiens GN=KRT10 PE=1 SV=6 | 58826.9 | 584 | 26.37 | 17 | 12 | 99 | 42 |
| 270 | 28.19 | tr|Q53SS8|Q53SS8_HUMAN | Epididymis secretory protein Li 85 OS=Homo sapiens GN=PCBP1 PE=2 SV=1 | 37497.5 | 356 | 53.65 | 14 | 9 | 211 | 104 |
| 271 | 28.19 | sp|P02786|TFR1_HUMAN | Transferrin receptor protein 1 OS=Homo sapiens GN=TFRC PE=1 SV=2 | 84870.7 | 760 | 22.63 | 15 | 15 | 113 | 111 |
| 272 | 27.97 | sp|Q8WVV4|POF1B_HUMAN | Protein POF1B OS=Homo sapiens GN=POF1B PE=1 SV=3 | 68064.6 | 589 | 25.47 | 14 | 14 | 69 | 69 |
| 273 | 27.89 | tr|Q8TC62|Q8TC62_HUMAN | Septin 7 OS=Homo sapiens GN=SEPT7 PE=2 SV=3 | 48658.6 | 417 | 37.89 | 14 | 14 | 69 | 62 |
| 274 | 27.89 | sp|P55084|ECHB_HUMAN | Trifunctional enzyme subunit beta, mitochondrial OS=Homo sapiens GN=HADHB PE=1 SV=3 | 51294 | 474 | 32.07 | 16 | 16 | 61 | 53 |
| 275 | 27.8 | tr|Q6PUJ7|Q6PUJ7_HUMAN | Epididymis luminal protein 215 OS=Homo sapiens GN=HEL-215 PE=2 SV=1 | 29819.8 | 272 | 59.19 | 15 | 15 | 162 | 159 |
| 276 | 27.76 | sp|Q07955|SRSF1_HUMAN | Serine/arginine-rich splicing factor 1 OS=Homo sapiens GN=SRSF1 PE=1 SV=2 | 27744.3 | 248 | 50 | 14 | 14 | 162 | 162 |
| 277 | 27.73 | sp|Q92945|FUBP2_HUMAN | Far upstream element-binding protein 2 OS=Homo sapiens GN=KHSRP PE=1 SV=4 | 73115.2 | 711 | 42.19 | 23 | 18 | 98 | 76 |
| 278 | 27.61 | sp|Q06210|GFPT1_HUMAN | Glutamine--fructose-6-phosphate aminotransferase [isomerizing] 1 OS=Homo sapiens GN=GFPT1 PE=1 SV=3 | 78805.8 | 699 | 30.04 | 15 | 15 | 95 | 95 |
| 279 | 27.51 | tr|Q59EC0|Q59EC0_HUMAN | Adenosine deaminase, RNA-specific isoform ADAR-a variant (Fragment) OS=Homo sapiens PE=2 SV=1 | 137832.1 | 1244 | 13.67 | 14 | 14 | 61 | 61 |
| 280 | 27.44 | tr|A0A024RBE7|A0A024RBE7_HUMAN | Thymopoietin, isoform CRA_c OS=Homo sapiens GN=TMPO PE=4 SV=1 | 50669.8 | 454 | 31.72 | 13 | 5 | 113 | 41 |
| 281 | 27.3 | tr|A0A024R713|A0A024R713_HUMAN | Dihydrolipoyl dehydrogenase OS=Homo sapiens GN=DLD PE=4 SV=1 | 54176.9 | 509 | 32.22 | 14 | 14 | 84 | 83 |
| 282 | 27.21 | tr|Q71RH4|Q71RH4_HUMAN | FP1047 OS=Homo sapiens PE=2 SV=1 | 69394.9 | 632 | 21.36 | 14 | 3 | 199 | 28 |
| 283 | 27.18 | tr|A8K7B7|A8K7B7_HUMAN | Protein phosphatase 2 (Formerly 2A), regulatory subunit A (PR 65), alpha isoform OS=Homo sapiens GN=PPP2R1A PE=1 SV=1 | 65307.8 | 589 | 28.01 | 14 | 11 | 49 | 43 |
| 284 | 27.16 | tr|Q5JR94|Q5JR94_HUMAN | 40S ribosomal protein S8 OS=Homo sapiens GN=RPS8 PE=2 SV=1 | 24205 | 208 | 48.56 | 15 | 15 | 216 | 216 |
| 285 | 27.15 | tr|A0A0S2Z471|A0A0S2Z471_HUMAN | Creatine kinase brain isoform 2 (Fragment) OS=Homo sapiens GN=CKB PE=2 SV=1 | 44921.4 | 405 | 43.95 | 13 | 12 | 93 | 89 |
| 286 | 27.14 | tr|A0A0S2Z4A5|A0A0S2Z4A5_HUMAN | DNA helicase (Fragment) OS=Homo sapiens GN=MCM7 PE=2 SV=1 | 81307.2 | 719 | 25.87 | 15 | 15 | 68 | 68 |
| 287 | 27.1 | tr|E9KL35|E9KL35_HUMAN | Epididymis tissue sperm binding protein Li 3a OS=Homo sapiens PE=1 SV=1 | 35076.5 | 317 | 42.59 | 13 | 13 | 136 | 134 |
| 288 | 27.03 | tr|Q7RU04|Q7RU04_HUMAN | Aminopeptidase B OS=Homo sapiens GN=RNPEP PE=4 SV=1 | 73497.2 | 658 | 24.77 | 14 | 14 | 63 | 63 |
| 289 | 26.91 | tr|A0A0S2Z4R1|A0A0S2Z4R1_HUMAN | Tyrosine--tRNA ligase (Fragment) OS=Homo sapiens GN=YARS PE=2 SV=1 | 59143 | 528 | 31.44 | 17 | 17 | 60 | 58 |
| 290 | 26.86 | sp|Q7Z2K6|ERMP1_HUMAN | Endoplasmic reticulum metallopeptidase 1 OS=Homo sapiens GN=ERMP1 PE=1 SV=2 | 100230.4 | 904 | 14.05 | 14 | 13 | 50 | 49 |
| 291 | 26.67 | tr|G0TQY6|G0TQY6_HUMAN | Lutheran blood group OS=Homo sapiens GN=LU PE=2 SV=1 | 67388.2 | 628 | 33.28 | 15 | 15 | 74 | 73 |
| 292 | 26.66 | tr|C9JIF9|C9JIF9_HUMAN | Acylamino-acid-releasing enzyme OS=Homo sapiens GN=APEH PE=1 SV=1 | 81673.5 | 737 | 22.39 | 14 | 14 | 69 | 69 |
| 293 | 26.65 | tr|V9HWI3|V9HWI3_HUMAN | Cathepsin D (Lysosomal aspartyl peptidase), isoform CRA_a OS=Homo sapiens GN=HEL-S-130P PE=2 SV=1 | 44551.8 | 412 | 36.41 | 14 | 14 | 135 | 131 |
| 294 | 26.62 | sp|P62917|RL8_HUMAN | 60S ribosomal protein L8 OS=Homo sapiens GN=RPL8 PE=1 SV=2 | 28024.5 | 257 | 45.14 | 13 | 13 | 73 | 73 |
| 295 | 26.59 | tr|A0A0A0MS51|A0A0A0MS51_HUMAN | Gelsolin OS=Homo sapiens GN=GSN PE=1 SV=1 | 82525.1 | 748 | 19.25 | 13 | 13 | 82 | 82 |
| 296 | 26.54 | tr|Q86VX4|Q86VX4_HUMAN | Structural maintenance of chromosomes protein OS=Homo sapiens GN=SMC3 PE=2 SV=1 | 141506.6 | 1217 | 13.06 | 13 | 13 | 46 | 46 |
| 297 | 26.45 | tr|A0A024R7T3|A0A024R7T3_HUMAN | Heterogeneous nuclear ribonucleoprotein F, isoform CRA_a OS=Homo sapiens GN=HNRPF PE=4 SV=1 | 45671.6 | 415 | 46.02 | 17 | 14 | 194 | 169 |
| 298 | 26.44 | tr|V9HW12|V9HW12_HUMAN | Epididymis secretory sperm binding protein Li 2a OS=Homo sapiens GN=HEL-S-2a PE=2 SV=1 | 21891.7 | 198 | 64.14 | 16 | 14 | 201 | 110 |
| 299 | 26.38 | tr|A0A0R4J2E8|A0A0R4J2E8_HUMAN | Matrin-3 OS=Homo sapiens GN=MATR3 PE=1 SV=1 | 94622.4 | 847 | 22.31 | 13 | 13 | 84 | 83 |
| 300 | 26.26 | sp|P07737|PROF1_HUMAN | Profilin-1 OS=Homo sapiens GN=PFN1 PE=1 SV=2 | 15054.1 | 140 | 78.57 | 16 | 16 | 615 | 613 |
| 301 | 26.23 | tr|A0A024R1K8|A0A024R1K8_HUMAN | Splicing factor 3a, subunit 1, 120kDa, isoform CRA_a OS=Homo sapiens GN=SF3A1 PE=4 SV=1 | 88885.6 | 793 | 22.45 | 15 | 15 | 44 | 43 |
| 302 | 26.21 | tr|Q5T0G8|Q5T0G8_HUMAN | Annexin OS=Homo sapiens GN=ANXA11 PE=2 SV=1 | 54389.3 | 505 | 29.11 | 13 | 13 | 112 | 111 |
| 303 | 26.16 | tr|A0A0S2Z4N8|A0A0S2Z4N8_HUMAN | Vasodilator-stimulated phosphoprotein isoform 2 (Fragment) OS=Homo sapiens GN=VASP PE=2 SV=1 | 35427.5 | 328 | 53.05 | 16 | 15 | 72 | 71 |
| 304 | 25.97 | sp|P16401|H15_HUMAN | Histone H1.5 OS=Homo sapiens GN=HIST1H1B PE=1 SV=3 | 22579.9 | 226 | 55.31 | 24 | 14 | 294 | 218 |
| 305 | 25.91 | tr|A0A140VJK2|A0A140VJK2_HUMAN | Glycerol-3-phosphate dehydrogenase OS=Homo sapiens PE=2 SV=1 | 80832.9 | 727 | 31.91 | 16 | 16 | 33 | 32 |
| 306 | 25.9 | tr|B2RBE0|B2RBE0_HUMAN | cDNA, FLJ95462, highly similar to Homo sapiens fatty-acid-Coenzyme A ligase, long-chain 3 (FACL3),mRNA OS=Homo sapiens PE=2 SV=1 | 80347.4 | 720 | 24.86 | 14 | 13 | 63 | 57 |
| 307 | 25.89 | tr|A0A0S2Z4Z0|A0A0S2Z4Z0_HUMAN | RNA binding motif protein 14 isoform 1 (Fragment) OS=Homo sapiens GN=RBM14 PE=2 SV=1 | 69490.9 | 669 | 23.62 | 13 | 13 | 63 | 63 |
| 308 | 25.88 | sp|P61981|1433G_HUMAN | 14-3-3 protein gamma OS=Homo sapiens GN=YWHAG PE=1 SV=2 | 28302.3 | 247 | 63.56 | 21 | 13 | 384 | 147 |
| 309 | 25.88 | tr|A0A024R1S8|A0A024R1S8_HUMAN | LIM and SH3 protein 1, isoform CRA_b OS=Homo sapiens GN=LASP1 PE=4 SV=1 | 29717.1 | 261 | 45.21 | 13 | 13 | 152 | 151 |
| 310 | 25.87 | tr|A0A140VK69|A0A140VK69_HUMAN | Aspartate aminotransferase OS=Homo sapiens PE=2 SV=1 | 46247.1 | 413 | 44.79 | 12 | 12 | 79 | 78 |
| 311 | 25.82 | tr|A0A024R8A7|A0A024R8A7_HUMAN | HCG31253, isoform CRA_a OS=Homo sapiens GN=hCG_31253 PE=4 SV=1 | 61640.1 | 572 | 40.56 | 19 | 16 | 54 | 33 |
| 312 | 25.74 | sp|P25786|PSA1_HUMAN | Proteasome subunit alpha type-1 OS=Homo sapiens GN=PSMA1 PE=1 SV=1 | 29555.3 | 263 | 51.71 | 14 | 14 | 94 | 94 |
| 313 | 25.7 | tr|A0A140VJT8|A0A140VJT8_HUMAN | Testicular tissue protein Li 164 OS=Homo sapiens PE=2 SV=1 | 49972.7 | 461 | 28.85 | 13 | 13 | 74 | 74 |
| 314 | 25.69 | tr|Q5H924|Q5H924_HUMAN | HECT, UBA and WWE domain containing 1 (Fragment) OS=Homo sapiens GN=HUWE1 PE=4 SV=1 | 374191.7 | 3407 | 6.868 | 15 | 13 | 48 | 38 |
| 315 | 25.68 | sp|P36578|RL4_HUMAN | 60S ribosomal protein L4 OS=Homo sapiens GN=RPL4 PE=1 SV=5 | 47696.9 | 427 | 36.3 | 14 | 14 | 115 | 115 |
| 316 | 25.66 | tr|A0A087X208|A0A087X208_HUMAN | Agrin OS=Homo sapiens GN=AGRN PE=1 SV=1 | 202291.1 | 1930 | 10.05 | 15 | 15 | 53 | 51 |
| 317 | 25.61 | tr|V9HW43|V9HW43_HUMAN | Epididymis secretory protein Li 102 OS=Homo sapiens GN=HEL-S-102 PE=2 SV=1 | 22782.3 | 205 | 67.32 | 13 | 13 | 207 | 207 |
| 318 | 25.53 | tr|A8K7J7|A8K7J7_HUMAN | cDNA FLJ78173, highly similar to Homo sapiens hexokinase 1 (HK1) mRNA OS=Homo sapiens PE=2 SV=1 | 102386 | 917 | 17.45 | 14 | 13 | 53 | 51 |
| 319 | 25.52 | sp|P53618|COPB_HUMAN | Coatomer subunit beta OS=Homo sapiens GN=COPB1 PE=1 SV=3 | 107141.1 | 953 | 18.89 | 12 | 12 | 53 | 53 |
| 320 | 25.5 | sp|P09012|SNRPA_HUMAN | U1 small nuclear ribonucleoprotein A OS=Homo sapiens GN=SNRPA PE=1 SV=3 | 31279.4 | 282 | 47.52 | 13 | 11 | 48 | 31 |
| 321 | 25.5 | sp|P22695|QCR2_HUMAN | Cytochrome b-c1 complex subunit 2, mitochondrial OS=Homo sapiens GN=UQCRC2 PE=1 SV=3 | 48442.6 | 453 | 43.71 | 16 | 16 | 82 | 82 |
| 322 | 25.44 | tr|A0A024R5K1|A0A024R5K1_HUMAN | Coronin OS=Homo sapiens GN=CORO1B PE=3 SV=1 | 54234.1 | 489 | 33.54 | 13 | 13 | 100 | 100 |
| 323 | 25.42 | tr|X5D2J9|X5D2J9_HUMAN | General transcription factor IIi isoform D (Fragment) OS=Homo sapiens GN=GTF2I PE=2 SV=1 | 107968.9 | 957 | 15.26 | 15 | 14 | 58 | 57 |
| 324 | 25.38 | tr|A0A024R8L7|A0A024R8L7_HUMAN | Acyl-coenzyme A oxidase OS=Homo sapiens GN=ACOX1 PE=3 SV=1 | 74685.2 | 660 | 19.85 | 12 | 12 | 30 | 30 |
| 325 | 25.33 | tr|Q8WVX7|Q8WVX7_HUMAN | Ribosomal protein S19 (Fragment) OS=Homo sapiens PE=2 SV=1 | 17281.8 | 157 | 66.88 | 14 | 14 | 93 | 93 |
| 326 | 25.32 | sp|Q15717|ELAV1_HUMAN | ELAV-like protein 1 OS=Homo sapiens GN=ELAVL1 PE=1 SV=2 | 36091.6 | 326 | 43.25 | 13 | 13 | 64 | 63 |
| 327 | 25.28 | tr|G8JLA2|G8JLA2_HUMAN | Myosin light polypeptide 6 OS=Homo sapiens GN=MYL6 PE=1 SV=1 | 17089.2 | 152 | 69.74 | 13 | 13 | 215 | 215 |
| 328 | 25.27 | sp|P27824|CALX_HUMAN | Calnexin OS=Homo sapiens GN=CANX PE=1 SV=2 | 67567.7 | 592 | 21.62 | 14 | 14 | 140 | 139 |
| 329 | 25.26 | tr|A0A024RCM3|A0A024RCM3_HUMAN | HCG2005638, isoform CRA_a OS=Homo sapiens GN=hCG_2005638 PE=4 SV=1 | 48990.9 | 428 | 25.93 | 12 | 3 | 96 | 47 |
| 330 | 25.23 | tr|A0A024RDR0|A0A024RDR0_HUMAN | High-mobility group box 1, isoform CRA_a OS=Homo sapiens GN=HMGB1 PE=4 SV=1 | 24893.6 | 215 | 48.37 | 14 | 11 | 54 | 42 |
| 331 | 25.18 | sp|O60568|PLOD3_HUMAN | Procollagen-lysine,2-oxoglutarate 5-dioxygenase 3 OS=Homo sapiens GN=PLOD3 PE=1 SV=1 | 84784.5 | 738 | 21.41 | 14 | 14 | 55 | 55 |
| 332 | 25.13 | sp|P15924|DESP_HUMAN | Desmoplakin OS=Homo sapiens GN=DSP PE=1 SV=3 | 331771.2 | 2871 | 8.15 | 20 | 17 | 49 | 39 |
| 333 | 25.06 | sp|Q9Y266|NUDC_HUMAN | Nuclear migration protein nudC OS=Homo sapiens GN=NUDC PE=1 SV=1 | 38242.7 | 331 | 36.56 | 14 | 14 | 53 | 53 |
| 334 | 25.06 | sp|Q15155|NOMO1_HUMAN | Nodal modulator 1 OS=Homo sapiens GN=NOMO1 PE=1 SV=5 | 134323 | 1222 | 14.57 | 13 | 12 | 44 | 43 |
| 335 | 25.02 | tr|A0A024R5H8|A0A024R5H8_HUMAN | RAB6A, member RAS oncogene family, isoform CRA_b OS=Homo sapiens GN=RAB6A PE=4 SV=1 | 23548.5 | 208 | 63.94 | 13 | 12 | 117 | 68 |
| 336 | 24.98 | sp|P11387|TOP1_HUMAN | DNA topoisomerase 1 OS=Homo sapiens GN=TOP1 PE=1 SV=2 | 90725.2 | 765 | 18.04 | 13 | 13 | 52 | 52 |
| 337 | 24.93 | tr|A0A0A0MSS8|A0A0A0MSS8_HUMAN | Aldo-keto reductase family 1 member C3 OS=Homo sapiens GN=AKR1C3 PE=1 SV=1 | 36866.9 | 323 | 53.25 | 14 | 6 | 136 | 60 |
| 338 | 24.89 | tr|B7ZAX9|B7ZAX9_HUMAN | cDNA, FLJ79343, highly similar to SWI/SNF-related matrix-associated actin-dependent regulator of chromatin subfamily A member 5 (EC 3.6.1.-) OS=Homo sapiens PE=2 SV=1 | 116770.9 | 995 | 13.87 | 14 | 14 | 53 | 53 |
| 339 | 24.85 | sp|P12004|PCNA_HUMAN | Proliferating cell nuclear antigen OS=Homo sapiens GN=PCNA PE=1 SV=1 | 28768.5 | 261 | 54.79 | 13 | 13 | 126 | 125 |
| 340 | 24.82 | sp|P31930|QCR1_HUMAN | Cytochrome b-c1 complex subunit 1, mitochondrial OS=Homo sapiens GN=UQCRC1 PE=1 SV=3 | 52645.3 | 480 | 32.08 | 12 | 11 | 70 | 69 |
| 341 | 24.8 | sp|P04040|CATA_HUMAN | Catalase OS=Homo sapiens GN=CAT PE=1 SV=3 | 59755.8 | 527 | 30.17 | 12 | 12 | 61 | 61 |
| 342 | 24.76 | sp|P07305|H10_HUMAN | Histone H1.0 OS=Homo sapiens GN=H1F0 PE=1 SV=3 | 20862.8 | 194 | 38.14 | 13 | 13 | 71 | 71 |
| 343 | 24.72 | tr|A0A0J9YXX5|A0A0J9YXX5_HUMAN | Poly(U)-binding-splicing factor PUF60 (Fragment) OS=Homo sapiens GN=PUF60 PE=1 SV=1 | 59102.1 | 551 | 31.03 | 14 | 14 | 45 | 45 |
| 344 | 24.61 | sp|P52209|6PGD_HUMAN | 6-phosphogluconate dehydrogenase, decarboxylating OS=Homo sapiens GN=PGD PE=1 SV=3 | 53139.6 | 483 | 32.51 | 14 | 13 | 118 | 114 |
| 345 | 24.56 | tr|D3DUJ0|D3DUJ0_HUMAN | AFG3 ATPase family gene 3-like 2 (Yeast), isoform CRA_a (Fragment) OS=Homo sapiens GN=AFG3L2 PE=3 SV=1 | 84444.1 | 759 | 19.76 | 13 | 13 | 59 | 58 |
| 346 | 24.54 | tr|Q567R6|Q567R6_HUMAN | Single-stranded DNA-binding protein OS=Homo sapiens GN=SSBP1 PE=2 SV=1 | 17358.7 | 148 | 73.65 | 15 | 15 | 129 | 129 |
| 347 | 24.51 | tr|B5BU28|B5BU28_HUMAN | Catenin beta-1 OS=Homo sapiens GN=CTNNB1 PE=2 SV=1 | 85514 | 781 | 27.27 | 15 | 14 | 62 | 53 |
| 348 | 24.42 | tr|K7ELL7|K7ELL7_HUMAN | Glucosidase 2 subunit beta OS=Homo sapiens GN=PRKCSH PE=1 SV=1 | 60191.7 | 535 | 22.06 | 13 | 13 | 98 | 98 |
| 349 | 24.35 | sp|P04844|RPN2_HUMAN | Dolichyl-diphosphooligosaccharide--protein glycosyltransferase subunit 2 OS=Homo sapiens GN=RPN2 PE=1 SV=3 | 69283.3 | 631 | 27.89 | 12 | 12 | 75 | 75 |
| 350 | 24.35 | sp|P62277|RS13_HUMAN | 40S ribosomal protein S13 OS=Homo sapiens GN=RPS13 PE=1 SV=2 | 17222.1 | 151 | 60.93 | 12 | 12 | 78 | 65 |
| 351 | 24.11 | tr|A0A024R872|A0A024R872_HUMAN | Chromosome 9 open reading frame 88, isoform CRA_a OS=Homo sapiens GN=C9orf88 PE=4 SV=1 | 82682.7 | 733 | 22.37 | 12 | 12 | 99 | 99 |
| 352 | 24.02 | tr|D3DQV9|D3DQV9_HUMAN | Eukaryotic translation initiation factor 4 gamma 2 (Fragment) OS=Homo sapiens GN=EIF4G2 PE=1 SV=1 | 102329 | 907 | 17.86 | 12 | 12 | 58 | 57 |
| 353 | 24.02 | tr|A0A024R7L5|A0A024R7L5_HUMAN | UPF1 regulator of nonsense transcripts homolog (Yeast), isoform CRA_b OS=Homo sapiens GN=UPF1 PE=4 SV=1 | 123034.9 | 1118 | 14.22 | 13 | 13 | 56 | 56 |
| 354 | 24.01 | tr|A0A024R845|A0A024R845_HUMAN | RAB14, member RAS oncogene family, isoform CRA_a OS=Homo sapiens GN=RAB14 PE=4 SV=1 | 23896.7 | 215 | 72.56 | 14 | 12 | 166 | 112 |
| 355 | 23.93 | tr|F1T0I1|F1T0I1_HUMAN | Protein transport protein Sec16A OS=Homo sapiens GN=SEC16A PE=1 SV=1 | 249479.9 | 2334 | 10.28 | 16 | 16 | 44 | 44 |
| 356 | 23.92 | sp|O15371|EIF3D_HUMAN | Eukaryotic translation initiation factor 3 subunit D OS=Homo sapiens GN=EIF3D PE=1 SV=1 | 63972.3 | 548 | 34.12 | 13 | 13 | 49 | 49 |
| 357 | 23.9 | tr|A0A0F7NGI8|A0A0F7NGI8_HUMAN | Leucine rich repeat (In FLII) interacting protein 1, isoform CRA_c OS=Homo sapiens GN=LRRFIP1 PE=2 SV=1 | 82688.3 | 752 | 21.81 | 12 | 12 | 29 | 28 |
| 358 | 23.84 | sp|Q9P2M7|CING_HUMAN | Cingulin OS=Homo sapiens GN=CGN PE=1 SV=2 | 136384.9 | 1197 | 15.79 | 15 | 15 | 23 | 22 |
| 359 | 23.76 | sp|O00425|IF2B3_HUMAN | Insulin-like growth factor 2 mRNA-binding protein 3 OS=Homo sapiens GN=IGF2BP3 PE=1 SV=2 | 63704.6 | 579 | 32.12 | 13 | 12 | 104 | 79 |
| 360 | 23.73 | tr|A0A024RB75|A0A024RB75_HUMAN | Citrate synthase OS=Homo sapiens GN=CS PE=3 SV=1 | 51712 | 466 | 30.9 | 12 | 10 | 109 | 94 |
| 361 | 23.69 | sp|P62249|RS16_HUMAN | 40S ribosomal protein S16 OS=Homo sapiens GN=RPS16 PE=1 SV=2 | 16445.2 | 146 | 54.11 | 12 | 12 | 132 | 132 |
| 362 | 23.67 | tr|A0A140VK42|A0A140VK42_HUMAN | Testicular secretory protein Li 42 OS=Homo sapiens PE=2 SV=1 | 49203.1 | 439 | 45.33 | 13 | 13 | 29 | 29 |
| 363 | 23.66 | sp|Q12929|EPS8_HUMAN | Epidermal growth factor receptor kinase substrate 8 OS=Homo sapiens GN=EPS8 PE=1 SV=1 | 91880.8 | 822 | 20.92 | 12 | 12 | 46 | 46 |
| 364 | 23.62 | tr|A0A158RFU6|A0A158RFU6_HUMAN | RAB7, member RAS oncogene family, isoform CRA_a OS=Homo sapiens GN=RAB7A PE=2 SV=1 | 23489.5 | 207 | 68.6 | 12 | 12 | 133 | 133 |
| 365 | 23.59 | tr|B4DMN1|B4DMN1_HUMAN | cDNA FLJ61136, highly similar to Ras-related protein Rab-11A OS=Homo sapiens PE=2 SV=1 | 28861.4 | 261 | 36.78 | 12 | 11 | 155 | 144 |
| 366 | 23.51 | sp|P46109|CRKL_HUMAN | Crk-like protein OS=Homo sapiens GN=CRKL PE=1 SV=1 | 33776.7 | 303 | 48.84 | 12 | 11 | 56 | 53 |
| 367 | 23.42 | tr|B9EKV4|B9EKV4_HUMAN | Aldehyde dehydrogenase 9 family, member A1 OS=Homo sapiens GN=ALDH9A1 PE=2 SV=1 | 56291.5 | 518 | 20.27 | 13 | 12 | 56 | 46 |
| 368 | 23.42 | sp|O60701|UGDH_HUMAN | UDP-glucose 6-dehydrogenase OS=Homo sapiens GN=UGDH PE=1 SV=1 | 55023.5 | 494 | 31.38 | 12 | 12 | 86 | 84 |
| 369 | 23.41 | tr|C9J9K3|C9J9K3_HUMAN | 40S ribosomal protein SA (Fragment) OS=Homo sapiens GN=RPSA PE=1 SV=7 | 29505.4 | 264 | 65.53 | 13 | 12 | 157 | 151 |
| 370 | 23.38 | sp|P51610|HCFC1_HUMAN | Host cell factor 1 OS=Homo sapiens GN=HCFC1 PE=1 SV=2 | 208731 | 2035 | 9.877 | 15 | 15 | 42 | 41 |
| 371 | 23.35 | sp|P55060|XPO2_HUMAN | Exportin-2 OS=Homo sapiens GN=CSE1L PE=1 SV=3 | 110415.5 | 971 | 13.7 | 12 | 12 | 78 | 77 |
| 372 | 23.28 | sp|P40429|RL13A_HUMAN | 60S ribosomal protein L13a OS=Homo sapiens GN=RPL13A PE=1 SV=2 | 23577.1 | 203 | 33.5 | 13 | 12 | 116 | 115 |
| 373 | 23.13 | sp|Q9Y5K6|CD2AP_HUMAN | CD2-associated protein OS=Homo sapiens GN=CD2AP PE=1 SV=1 | 71450.5 | 639 | 20.97 | 12 | 11 | 61 | 60 |
| 374 | 23.13 | tr|V9HWC7|V9HWC7_HUMAN | Epididymis secretory sperm binding protein Li 128m OS=Homo sapiens GN=HEL-S-128m PE=2 SV=1 | 25034.7 | 224 | 69.64 | 13 | 13 | 119 | 119 |
| 375 | 23.13 | tr|Q59GB4|Q59GB4_HUMAN | Dihydropyrimidinase-like 2 variant (Fragment) OS=Homo sapiens PE=2 SV=1 | 68183.4 | 628 | 28.98 | 12 | 12 | 31 | 31 |
| 376 | 23.12 | sp|Q14157|UBP2L_HUMAN | Ubiquitin-associated protein 2-like OS=Homo sapiens GN=UBAP2L PE=1 SV=2 | 114533.8 | 1087 | 19.6 | 12 | 12 | 37 | 36 |
| 377 | 23.05 | sp|Q9H9B4|SFXN1_HUMAN | Sideroflexin-1 OS=Homo sapiens GN=SFXN1 PE=1 SV=4 | 35619.1 | 322 | 48.45 | 12 | 12 | 121 | 112 |
| 378 | 23 | sp|Q8TCS8|PNPT1_HUMAN | Polyribonucleotide nucleotidyltransferase 1, mitochondrial OS=Homo sapiens GN=PNPT1 PE=1 SV=2 | 85949.8 | 783 | 17.75 | 13 | 12 | 44 | 42 |
| 379 | 22.98 | sp|Q13428|TCOF_HUMAN | Treacle protein OS=Homo sapiens GN=TCOF1 PE=1 SV=3 | 152104.7 | 1488 | 13.04 | 16 | 16 | 30 | 30 |
| 380 | 22.96 | tr|A0A024R4G1|A0A024R4G1_HUMAN | Leucine rich repeat containing 47, isoform CRA_a OS=Homo sapiens GN=LRRC47 PE=4 SV=1 | 63472.2 | 583 | 31.39 | 14 | 14 | 70 | 68 |
| 381 | 22.95 | tr|A0A024RAI1|A0A024RAI1_HUMAN | ARP3 actin-related protein 3 homolog (Yeast), isoform CRA_a OS=Homo sapiens GN=ACTR3 PE=3 SV=1 | 47370.8 | 418 | 29.9 | 13 | 13 | 34 | 34 |
| 382 | 22.94 | tr|B3KUB6|B3KUB6_HUMAN | cDNA FLJ39529 fis, clone PUAEN2004067, highly similar to Band 4.1-like protein 1 OS=Homo sapiens PE=2 SV=1 | 86472.9 | 772 | 20.08 | 11 | 11 | 38 | 38 |
| 383 | 22.92 | sp|Q9Y3I0|RTCB_HUMAN | tRNA-splicing ligase RtcB homolog OS=Homo sapiens GN=RTCB PE=1 SV=1 | 55209.9 | 505 | 29.7 | 12 | 11 | 70 | 69 |
| 384 | 22.86 | sp|Q14444|CAPR1_HUMAN | Caprin-1 OS=Homo sapiens GN=CAPRIN1 PE=1 SV=2 | 78366 | 709 | 18.9 | 12 | 12 | 101 | 101 |
| 385 | 22.75 | sp|P41252|SYIC_HUMAN | Isoleucine--tRNA ligase, cytoplasmic OS=Homo sapiens GN=IARS PE=1 SV=2 | 144496.9 | 1262 | 11.97 | 13 | 13 | 55 | 55 |
| 386 | 22.74 | sp|Q6XQN6|PNCB_HUMAN | Nicotinate phosphoribosyltransferase OS=Homo sapiens GN=NAPRT PE=1 SV=2 | 57577.6 | 538 | 21.56 | 11 | 11 | 52 | 52 |
| 387 | 22.72 | tr|B3KXZ4|B3KXZ4_HUMAN | DNA helicase OS=Homo sapiens PE=2 SV=1 | 91271 | 808 | 17.57 | 11 | 11 | 58 | 57 |
| 388 | 22.7 | sp|P62280|RS11_HUMAN | 40S ribosomal protein S11 OS=Homo sapiens GN=RPS11 PE=1 SV=3 | 18430.6 | 158 | 59.49 | 12 | 12 | 105 | 105 |
| 389 | 22.58 | tr|Q6NZ55|Q6NZ55_HUMAN | 60S ribosomal protein L13 OS=Homo sapiens GN=RPL13 PE=2 SV=1 | 24265.2 | 211 | 41.23 | 12 | 12 | 138 | 134 |
| 390 | 22.57 | tr|A0A140VJP5|A0A140VJP5_HUMAN | S-adenosylmethionine synthase OS=Homo sapiens PE=2 SV=1 | 43660.4 | 395 | 27.34 | 12 | 12 | 91 | 91 |
| 391 | 22.55 | sp|P00505|AATM_HUMAN | Aspartate aminotransferase, mitochondrial OS=Homo sapiens GN=GOT2 PE=1 SV=3 | 47517.3 | 430 | 45.81 | 14 | 14 | 125 | 125 |
| 392 | 22.55 | tr|B4DSH1|B4DSH1_HUMAN | cDNA FLJ51295, highly similar to Cell division cycle 5-like protein OS=Homo sapiens PE=2 SV=1 | 89225.6 | 775 | 20.26 | 14 | 14 | 31 | 31 |
| 393 | 22.55 | tr|V9HWC2|V9HWC2_HUMAN | Epididymis secretory sperm binding protein Li 67p OS=Homo sapiens GN=HEL-S-67p PE=2 SV=1 | 19890.9 | 189 | 80.95 | 12 | 12 | 215 | 214 |
| 394 | 22.51 | sp|P61254|RL26_HUMAN | 60S ribosomal protein L26 OS=Homo sapiens GN=RPL26 PE=1 SV=1 | 17258.2 | 145 | 42.76 | 11 | 10 | 91 | 89 |
| 395 | 22.48 | tr|A8K9K6|A8K9K6_HUMAN | cDNA FLJ76962, highly similar to Homo sapiens nucleolar protein 5A (56kDa with KKE/D repeat) (NOL5A), mRNA OS=Homo sapiens PE=2 SV=1 | 65977.2 | 594 | 21.72 | 11 | 11 | 46 | 46 |
| 396 | 22.43 | sp|Q9NSE4|SYIM_HUMAN | Isoleucine--tRNA ligase, mitochondrial OS=Homo sapiens GN=IARS2 PE=1 SV=2 | 113790.6 | 1012 | 14.62 | 12 | 12 | 55 | 55 |
| 397 | 22.37 | sp|P22059|OSBP1_HUMAN | Oxysterol-binding protein 1 OS=Homo sapiens GN=OSBP PE=1 SV=1 | 89419.9 | 807 | 18.59 | 12 | 12 | 46 | 45 |
| 398 | 22.36 | sp|Q13045|FLII_HUMAN | Protein flightless-1 homolog OS=Homo sapiens GN=FLII PE=1 SV=2 | 144749.9 | 1269 | 12.06 | 13 | 12 | 39 | 38 |
| 399 | 22.34 | sp|Q7Z2W4|ZCCHV_HUMAN | Zinc finger CCCH-type antiviral protein 1 OS=Homo sapiens GN=ZC3HAV1 PE=1 SV=3 | 101430.5 | 902 | 15.96 | 12 | 12 | 37 | 37 |
| 400 | 22.33 | sp|P15880|RS2_HUMAN | 40S ribosomal protein S2 OS=Homo sapiens GN=RPS2 PE=1 SV=2 | 31324.2 | 293 | 39.93 | 14 | 12 | 137 | 135 |
| 401 | 22.32 | tr|Q53Z07|Q53Z07_HUMAN | NPC-A-16 OS=Homo sapiens GN=RPL9 PE=2 SV=1 | 21863.3 | 192 | 39.06 | 11 | 11 | 70 | 70 |
| 402 | 22.29 | sp|Q5JRX3|PREP_HUMAN | Presequence protease, mitochondrial OS=Homo sapiens GN=PITRM1 PE=1 SV=3 | 117411.8 | 1037 | 16.01 | 12 | 12 | 46 | 46 |
| 403 | 22.18 | sp|O75116|ROCK2_HUMAN | Rho-associated protein kinase 2 OS=Homo sapiens GN=ROCK2 PE=1 SV=4 | 160898.6 | 1388 | 12.1 | 14 | 12 | 34 | 25 |
| 404 | 22.18 | tr|A0A024R5K8|A0A024R5K8_HUMAN | Serpin peptidase inhibitor, clade H (Heat shock protein 47), member 1, (Collagen binding protein 1), isoform CRA_a OS=Homo sapiens GN=SERPINH1 PE=3 SV=1 | 46440.1 | 418 | 36.84 | 11 | 11 | 79 | 79 |
| 405 | 22.16 | tr|A0A024RB17|A0A024RB17_HUMAN | Family with sequence similarity 62 (C2 domain containing), member A, isoform CRA_b OS=Homo sapiens GN=FAM62A PE=4 SV=1 | 122855.3 | 1104 | 15.85 | 13 | 13 | 57 | 57 |
| 406 | 22.13 | tr|A0A140VJX1|A0A140VJX1_HUMAN | Testicular tissue protein Li 198 OS=Homo sapiens PE=2 SV=1 | 45199.2 | 427 | 38.17 | 13 | 13 | 56 | 55 |
| 407 | 22.07 | sp|P16152|CBR1_HUMAN | Carbonyl reductase [NADPH] 1 OS=Homo sapiens GN=CBR1 PE=1 SV=3 | 30374.7 | 277 | 46.21 | 11 | 11 | 65 | 65 |
| 408 | 22.06 | sp|P62424|RL7A_HUMAN | 60S ribosomal protein L7a OS=Homo sapiens GN=RPL7A PE=1 SV=2 | 29995.4 | 266 | 30.83 | 11 | 11 | 93 | 92 |
| 409 | 22.03 | tr|A0A024QZN9|A0A024QZN9_HUMAN | Voltage-dependent anion channel 2, isoform CRA_a OS=Homo sapiens GN=VDAC2 PE=4 SV=1 | 34480.6 | 319 | 45.14 | 13 | 12 | 173 | 161 |
| 410 | 21.98 | sp|Q10567|AP1B1_HUMAN | AP-1 complex subunit beta-1 OS=Homo sapiens GN=AP1B1 PE=1 SV=2 | 104635.6 | 949 | 13.91 | 11 | 9 | 56 | 39 |
| 411 | 21.97 | tr|B3KY63|B3KY63_HUMAN | cDNA FLJ16830 fis, clone UTERU3022536, highly similar to Chromodomain helicase-DNA-binding protein 4 (EC 3.6.1.-) OS=Homo sapiens PE=2 SV=1 | 215282.9 | 1886 | 10.23 | 16 | 15 | 37 | 26 |
| 412 | 21.78 | sp|P54136|SYRC_HUMAN | Arginine--tRNA ligase, cytoplasmic OS=Homo sapiens GN=RARS PE=1 SV=2 | 75378.3 | 660 | 19.7 | 12 | 11 | 84 | 79 |
| 413 | 21.74 | sp|Q8TEM1|PO210_HUMAN | Nuclear pore membrane glycoprotein 210 OS=Homo sapiens GN=NUP210 PE=1 SV=3 | 205109.5 | 1887 | 11.29 | 17 | 17 | 44 | 43 |
| 414 | 21.73 | sp|Q9UJU6|DBNL_HUMAN | Drebrin-like protein OS=Homo sapiens GN=DBNL PE=1 SV=1 | 48207 | 430 | 36.05 | 13 | 3 | 61 | 10 |
| 415 | 21.72 | tr|Q6ZSA3|Q6ZSA3_HUMAN | cDNA FLJ45695 fis, clone FEBRA2013570, highly similar to 2-oxoisovalerate dehydrogenase alpha subunit, mitochondrial (EC 1.2.4.4) OS=Homo sapiens PE=2 SV=1 | 54130.4 | 479 | 32.15 | 11 | 11 | 39 | 39 |
| 416 | 21.68 | tr|D6RD18|D6RD18_HUMAN | Heterogeneous nuclear ribonucleoprotein A/B OS=Homo sapiens GN=HNRNPAB PE=1 SV=1 | 30473.8 | 283 | 34.98 | 11 | 10 | 74 | 63 |
| 417 | 21.65 | tr|Q53FS4|Q53FS4_HUMAN | Lectin, mannose-binding, 1 variant (Fragment) OS=Homo sapiens PE=2 SV=1 | 57557.7 | 510 | 23.92 | 10 | 10 | 47 | 46 |
| 418 | 21.63 | sp|Q14573|ITPR3_HUMAN | Inositol 1,4,5-trisphosphate receptor type 3 OS=Homo sapiens GN=ITPR3 PE=1 SV=2 | 304104 | 2671 | 7.787 | 15 | 15 | 37 | 35 |
| 419 | 21.6 | sp|P08727|K1C19_HUMAN | Keratin, type I cytoskeletal 19 OS=Homo sapiens GN=KRT19 PE=1 SV=4 | 44105.6 | 400 | 91 | 52 | 11 | 1209 | 179 |
| 420 | 21.58 | sp|P50570|DYN2_HUMAN | Dynamin-2 OS=Homo sapiens GN=DNM2 PE=1 SV=2 | 98063.3 | 870 | 15.17 | 13 | 6 | 41 | 20 |
| 421 | 21.58 | sp|Q02543|RL18A_HUMAN | 60S ribosomal protein L18a OS=Homo sapiens GN=RPL18A PE=1 SV=2 | 20762.2 | 176 | 43.75 | 11 | 11 | 96 | 96 |
| 422 | 21.56 | tr|A0A024R6C9|A0A024R6C9_HUMAN | Dihydrolipoamide S-succinyltransferase (E2 component of 2-oxo-glutarate complex), isoform CRA_a OS=Homo sapiens GN=DLST PE=4 SV=1 | 48754.9 | 453 | 33.33 | 11 | 11 | 54 | 54 |
| 423 | 21.53 | tr|Q6FHX6|Q6FHX6_HUMAN | Flap endonuclease 1 OS=Homo sapiens GN=FEN1 PE=2 SV=1 | 42592.6 | 380 | 31.84 | 13 | 11 | 38 | 35 |
| 424 | 21.51 | tr|A0A140VJF3|A0A140VJF3_HUMAN | Testicular tissue protein Li 27 OS=Homo sapiens PE=2 SV=1 | 36954.3 | 326 | 32.52 | 11 | 11 | 70 | 70 |
| 425 | 21.5 | tr|A0A024R4M0|A0A024R4M0_HUMAN | 40S ribosomal protein S9 OS=Homo sapiens GN=RPS9 PE=1 SV=1 | 22591.2 | 194 | 43.81 | 10 | 10 | 69 | 69 |
| 426 | 21.46 | sp|Q9NR45|SIAS_HUMAN | Sialic acid synthase OS=Homo sapiens GN=NANS PE=1 SV=2 | 40307.3 | 359 | 43.18 | 14 | 14 | 74 | 73 |
| 427 | 21.38 | tr|Q6FHM2|Q6FHM2_HUMAN | GNB2 protein OS=Homo sapiens GN=GNB2 PE=2 SV=1 | 37330.6 | 340 | 30.59 | 12 | 7 | 46 | 30 |
| 428 | 21.37 | sp|Q15075|EEA1_HUMAN | Early endosome antigen 1 OS=Homo sapiens GN=EEA1 PE=1 SV=2 | 162464.9 | 1411 | 10.63 | 13 | 13 | 21 | 21 |
| 429 | 21.32 | sp|Q9BXP5|SRRT_HUMAN | Serrate RNA effector molecule homolog OS=Homo sapiens GN=SRRT PE=1 SV=1 | 100665.6 | 876 | 14.73 | 11 | 11 | 20 | 20 |
| 430 | 21.3 | tr|Q59F99|Q59F99_HUMAN | Staufen isoform b variant (Fragment) OS=Homo sapiens PE=2 SV=1 | 64745.9 | 591 | 28.43 | 12 | 9 | 41 | 35 |
| 431 | 21.29 | tr|A4D275|A4D275_HUMAN | Actin related protein 2/3 complex, subunit 1B, 41kDa OS=Homo sapiens GN=ARPC1B PE=2 SV=1 | 40949.4 | 372 | 31.18 | 11 | 10 | 54 | 51 |
| 432 | 21.19 | tr|Q9GZV0|Q9GZV0_HUMAN | cDNA FLJ12454 fis, clone NT2RM1000555, highly similar to UNR PROTEIN OS=Homo sapiens PE=2 SV=1 | 85729.7 | 767 | 17.99 | 13 | 12 | 43 | 40 |
| 433 | 21.18 | sp|O43390|HNRPR_HUMAN | Heterogeneous nuclear ribonucleoprotein R OS=Homo sapiens GN=HNRNPR PE=1 SV=1 | 70942.8 | 633 | 29.7 | 21 | 11 | 144 | 59 |
| 434 | 21.15 | sp|Q12792|TWF1_HUMAN | Twinfilin-1 OS=Homo sapiens GN=TWF1 PE=1 SV=3 | 40282.4 | 350 | 31.14 | 11 | 10 | 79 | 77 |
| 435 | 21.14 | sp|Q9H6S3|ES8L2_HUMAN | Epidermal growth factor receptor kinase substrate 8-like protein 2 OS=Homo sapiens GN=EPS8L2 PE=1 SV=2 | 80620 | 715 | 19.58 | 13 | 13 | 48 | 48 |
| 436 | 21.08 | tr|A8K4T9|A8K4T9_HUMAN | cDNA FLJ77421, highly similar to Homo sapiens autoantigen p542 mRNA OS=Homo sapiens PE=2 SV=1 | 30397.8 | 291 | 40.89 | 13 | 11 | 107 | 103 |
| 437 | 21.02 | sp|P16104|H2AX_HUMAN | Histone H2AX OS=Homo sapiens GN=H2AFX PE=1 SV=2 | 15144.4 | 143 | 44.06 | 12 | 6 | 132 | 10 |
| 438 | 20.99 | tr|B5BU83|B5BU83_HUMAN | Stathmin OS=Homo sapiens GN=STMN1 PE=2 SV=1 | 17330.4 | 149 | 61.07 | 13 | 13 | 108 | 107 |
| 439 | 20.98 | tr|Q5U0F4|Q5U0F4_HUMAN | Eukaryotic translation initiation factor 3 subunit I OS=Homo sapiens GN=EIF3S2 PE=2 SV=1 | 36501.6 | 325 | 39.69 | 10 | 10 | 39 | 39 |
| 440 | 20.94 | tr|B4DS24|B4DS24_HUMAN | cDNA FLJ50635, highly similar to ATP-dependent RNA helicase DDX19A (EC 3.6.1.-) OS=Homo sapiens PE=2 SV=1 | 50433.4 | 447 | 31.54 | 12 | 12 | 44 | 44 |
| 441 | 20.81 | sp|P15559|NQO1_HUMAN | NAD(P)H dehydrogenase [quinone] 1 OS=Homo sapiens GN=NQO1 PE=1 SV=1 | 30867.4 | 274 | 36.13 | 11 | 11 | 151 | 151 |
| 442 | 20.77 | sp|Q6P996|PDXD1_HUMAN | Pyridoxal-dependent decarboxylase domain-containing protein 1 OS=Homo sapiens GN=PDXDC1 PE=1 SV=2 | 86706.1 | 788 | 20.94 | 11 | 11 | 31 | 30 |
| 443 | 20.76 | tr|V9HW01|V9HW01_HUMAN | Epididymis secretory protein Li 310 OS=Homo sapiens GN=HEL-S-310 PE=2 SV=1 | 17778.8 | 157 | 44.59 | 10 | 10 | 181 | 180 |
| 444 | 20.75 | tr|Q53G41|Q53G41_HUMAN | Tripartite motif protein TRIM29 isoform alpha variant (Fragment) OS=Homo sapiens PE=2 SV=1 | 65816.2 | 588 | 19.56 | 12 | 12 | 26 | 26 |
| 445 | 20.67 | tr|V9HWE8|V9HWE8_HUMAN | Epididymis secretory sperm binding protein Li 47e OS=Homo sapiens GN=HEL-S-47e PE=2 SV=1 | 23206.9 | 204 | 50.49 | 10 | 10 | 140 | 137 |
| 446 | 20.64 | sp|P46779|RL28_HUMAN | 60S ribosomal protein L28 OS=Homo sapiens GN=RPL28 PE=1 SV=3 | 15747.4 | 137 | 53.28 | 11 | 11 | 90 | 90 |
| 447 | 20.63 | tr|V9HWJ1|V9HWJ1_HUMAN | Glutathione synthetase OS=Homo sapiens GN=HEL-S-64p PE=2 SV=1 | 52384.3 | 474 | 27 | 11 | 11 | 70 | 69 |
| 448 | 20.62 | sp|Q15056|IF4H_HUMAN | Eukaryotic translation initiation factor 4H OS=Homo sapiens GN=EIF4H PE=1 SV=5 | 27384.8 | 248 | 40.73 | 11 | 11 | 38 | 38 |
| 449 | 20.55 | tr|B3KMX0|B3KMX0_HUMAN | DNA helicase OS=Homo sapiens PE=2 SV=1 | 96603 | 863 | 16.57 | 11 | 11 | 43 | 43 |
| 450 | 20.55 | tr|Q8NHX6|Q8NHX6_HUMAN | DNA helicase OS=Homo sapiens GN=HCC5 PE=2 SV=1 | 75786.4 | 676 | 23.96 | 12 | 12 | 38 | 38 |
| 451 | 20.55 | tr|G5EA31|G5EA31_HUMAN | Protein transport protein Sec24C OS=Homo sapiens GN=SEC24C PE=1 SV=1 | 111984.2 | 1042 | 13.82 | 10 | 9 | 51 | 50 |
| 452 | 20.51 | tr|A0A140VK70|A0A140VK70_HUMAN | Testis secretory sperm-binding protein Li 197a OS=Homo sapiens PE=2 SV=1 | 48633.4 | 433 | 25.87 | 10 | 10 | 65 | 65 |
| 453 | 20.51 | tr|B2RDE8|B2RDE8_HUMAN | cDNA, FLJ96580, highly similar to Homo sapiens hepatoma-derived growth factor (high-mobility group protein 1-like) (HDGF), mRNA OS=Homo sapiens PE=2 SV=1 | 26805.1 | 240 | 47.92 | 10 | 10 | 125 | 123 |
| 454 | 20.49 | tr|A0A024R0R1|A0A024R0R1_HUMAN | Rho guanine nucleotide exchange factor (GEF) 1, isoform CRA_e OS=Homo sapiens GN=ARHGEF1 PE=4 SV=1 | 102434.3 | 912 | 13.05 | 10 | 10 | 46 | 46 |
| 455 | 20.43 | tr|A8K6X3|A8K6X3_HUMAN | cDNA FLJ78679, highly similar to Homo sapiens DEAD (Asp-Glu-Ala-Asp) box polypeptide 46 (DDX46), mRNA OS=Homo sapiens PE=2 SV=1 | 117470.3 | 1032 | 14.15 | 13 | 13 | 33 | 33 |
| 456 | 20.36 | tr|A0A024R534|A0A024R534_HUMAN | Metastasis associated 1 family, member 2, isoform CRA_a OS=Homo sapiens GN=MTA2 PE=4 SV=1 | 75022.4 | 668 | 18.71 | 11 | 11 | 32 | 32 |
| 457 | 20.35 | sp|P38606|VATA_HUMAN | V-type proton ATPase catalytic subunit A OS=Homo sapiens GN=ATP6V1A PE=1 SV=2 | 68303.5 | 617 | 23.18 | 12 | 11 | 48 | 47 |
| 458 | 20.3 | sp|P27694|RFA1_HUMAN | Replication protein A 70 kDa DNA-binding subunit OS=Homo sapiens GN=RPA1 PE=1 SV=2 | 68137.7 | 616 | 24.84 | 11 | 11 | 36 | 36 |
| 459 | 20.3 | tr|Q53XM7|Q53XM7_HUMAN | VAMP (Vesicle-associated membrane protein)-associated protein B and C OS=Homo sapiens GN=VAPB PE=1 SV=1 | 27228.2 | 243 | 39.51 | 11 | 10 | 46 | 37 |
| 460 | 20.26 | sp|O75367|H2AY_HUMAN | Core histone macro-H2A.1 OS=Homo sapiens GN=H2AFY PE=1 SV=4 | 39616.7 | 372 | 38.44 | 12 | 12 | 216 | 216 |
| 461 | 20.22 | tr|Q6MZU1|Q6MZU1_HUMAN | Putative uncharacterized protein DKFZp686A1195 OS=Homo sapiens GN=DKFZp686A1195 PE=2 SV=1 | 189616.1 | 1692 | 10.99 | 15 | 15 | 50 | 50 |
| 462 | 20.18 | tr|A8K8U1|A8K8U1_HUMAN | cDNA FLJ77762, highly similar to Homo sapiens cullin-associated and neddylation-dissociated 1 (CAND1), mRNA OS=Homo sapiens PE=2 SV=1 | 136316 | 1230 | 11.14 | 11 | 11 | 68 | 68 |
| 463 | 20.17 | tr|A0A140VKE7|A0A140VKE7_HUMAN | Testis tissue sperm-binding protein Li 64n OS=Homo sapiens PE=2 SV=1 | 94511.7 | 839 | 18.95 | 14 | 11 | 82 | 35 |
| 464 | 20.15 | tr|A0A024R0J9|A0A024R0J9_HUMAN | Heterogeneous nuclear ribonucleoprotein U-like 1, isoform CRA_a OS=Homo sapiens GN=HNRPUL1 PE=4 SV=1 | 84793.3 | 756 | 15.61 | 10 | 10 | 45 | 43 |
| 465 | 20.13 | sp|O43776|SYNC_HUMAN | Asparagine--tRNA ligase, cytoplasmic OS=Homo sapiens GN=NARS PE=1 SV=1 | 62942.4 | 548 | 20.8 | 12 | 12 | 72 | 71 |
| 466 | 20.12 | tr|B3KSL5|B3KSL5_HUMAN | cDNA FLJ36545 fis, clone TRACH2006670, highly similar to RNA-binding protein Luc7-like 2 OS=Homo sapiens PE=2 SV=1 | 46483.4 | 392 | 22.96 | 10 | 7 | 64 | 54 |
| 467 | 20.08 | sp|P29373|RABP2_HUMAN | Cellular retinoic acid-binding protein 2 OS=Homo sapiens GN=CRABP2 PE=1 SV=2 | 15692.9 | 138 | 63.77 | 10 | 10 | 73 | 73 |
| 468 | 20.06 | tr|V9HW24|V9HW24_HUMAN | Epididymis secretory protein Li 73 OS=Homo sapiens GN=HEL-S-73 PE=2 SV=1 | 44160.5 | 389 | 30.59 | 10 | 10 | 44 | 44 |
| 469 | 20.06 | sp|Q92878|RAD50_HUMAN | DNA repair protein RAD50 OS=Homo sapiens GN=RAD50 PE=1 SV=1 | 153891.1 | 1312 | 11.2 | 13 | 13 | 28 | 27 |
| 470 | 20.05 | tr|A0A024R5C5|A0A024R5C5_HUMAN | Pyruvate carboxylase OS=Homo sapiens GN=PC PE=4 SV=1 | 129632.6 | 1178 | 12.39 | 12 | 12 | 28 | 28 |
| 471 | 19.98 | sp|Q8WWM7|ATX2L_HUMAN | Ataxin-2-like protein OS=Homo sapiens GN=ATXN2L PE=1 SV=2 | 113372.9 | 1075 | 15.72 | 13 | 12 | 37 | 33 |
| 472 | 19.85 | tr|A2A3R6|A2A3R6_HUMAN | 40S ribosomal protein S6 OS=Homo sapiens GN=RPS6 PE=2 SV=1 | 28680.4 | 249 | 37.35 | 12 | 12 | 137 | 134 |
| 473 | 19.81 | sp|Q6PKG0|LARP1_HUMAN | La-related protein 1 OS=Homo sapiens GN=LARP1 PE=1 SV=2 | 123509.5 | 1096 | 16.88 | 15 | 13 | 33 | 31 |
| 474 | 19.79 | tr|A0A024R261|A0A024R261_HUMAN | HCG24487, isoform CRA_c OS=Homo sapiens GN=hCG_24487 PE=3 SV=1 | 21396.9 | 184 | 47.83 | 11 | 10 | 67 | 66 |
| 475 | 19.79 | sp|Q08J23|NSUN2_HUMAN | tRNA (cytosine(34)-C(5))-methyltransferase OS=Homo sapiens GN=NSUN2 PE=1 SV=2 | 86470.1 | 767 | 16.17 | 11 | 11 | 33 | 33 |
| 476 | 19.77 | tr|A0A0G2JNZ2|A0A0G2JNZ2_HUMAN | Protein scribble homolog OS=Homo sapiens GN=SCRIB PE=1 SV=1 | 174913 | 1630 | 8.466 | 12 | 12 | 22 | 21 |
| 477 | 19.74 | tr|B4DUT7|B4DUT7_HUMAN | cDNA FLJ57604, highly similar to GMP synthase (glutamine-hydrolyzing) (EC 6.3.5.2) OS=Homo sapiens PE=2 SV=1 | 71240.7 | 642 | 18.85 | 11 | 11 | 51 | 51 |
| 478 | 19.71 | sp|Q9Y3F4|STRAP_HUMAN | Serine-threonine kinase receptor-associated protein OS=Homo sapiens GN=STRAP PE=1 SV=1 | 38438 | 350 | 42.57 | 12 | 11 | 65 | 63 |
| 479 | 19.69 | tr|Q2L6I2|Q2L6I2_HUMAN | ABC50 protein OS=Homo sapiens GN=ABCF1 PE=4 SV=1 | 91679.7 | 807 | 18.46 | 11 | 11 | 42 | 42 |
| 480 | 19.67 | tr|V9HW91|V9HW91_HUMAN | Epididymis secretory sperm binding protein Li 8a OS=Homo sapiens GN=HEL-S-8a PE=2 SV=1 | 30607.6 | 276 | 47.1 | 11 | 11 | 40 | 40 |
| 481 | 19.62 | sp|Q9HDC9|APMAP_HUMAN | Adipocyte plasma membrane-associated protein OS=Homo sapiens GN=APMAP PE=1 SV=2 | 46479.9 | 416 | 25.96 | 10 | 10 | 47 | 43 |
| 482 | 19.59 | tr|A0A024R8W0|A0A024R8W0_HUMAN | DEAD (Asp-Glu-Ala-Asp) box polypeptide 48, isoform CRA_a OS=Homo sapiens GN=DDX48 PE=3 SV=1 | 46870.6 | 411 | 27.98 | 13 | 11 | 75 | 25 |
| 483 | 19.59 | tr|Q59ED7|Q59ED7_HUMAN | Putative uncharacterized protein (Fragment) OS=Homo sapiens PE=2 SV=1 | 77736.4 | 686 | 20.26 | 11 | 11 | 41 | 41 |
| 484 | 19.58 | tr|B4DV28|B4DV28_HUMAN | cDNA FLJ54170, highly similar to Cytosolic nonspecific dipeptidase OS=Homo sapiens PE=2 SV=1 | 51501.7 | 463 | 32.4 | 11 | 11 | 78 | 78 |
| 485 | 19.56 | tr|Q6FHQ0|Q6FHQ0_HUMAN | RBBP7 protein (Fragment) OS=Homo sapiens GN=RBBP7 PE=2 SV=1 | 47819.7 | 425 | 22.59 | 10 | 4 | 65 | 37 |
| 486 | 19.44 | tr|E5KRK5|E5KRK5_HUMAN | Mitochondrial NADH-ubiquinone oxidoreductase 75 kDa subunit OS=Homo sapiens GN=NDUFS1 PE=3 SV=1 | 79466.8 | 727 | 22.7 | 11 | 11 | 30 | 30 |
| 487 | 19.44 | sp|Q71DI3|H32_HUMAN | Histone H3.2 OS=Homo sapiens GN=HIST2H3A PE=1 SV=3 | 15387.9 | 136 | 63.97 | 12 | 12 | 213 | 204 |
| 488 | 19.42 | tr|V9HWF2|V9HWF2_HUMAN | Malate dehydrogenase OS=Homo sapiens GN=HEL-S-32 PE=2 SV=1 | 36425.8 | 334 | 38.32 | 12 | 12 | 77 | 77 |
| 489 | 19.41 | sp|P22087|FBRL_HUMAN | rRNA 2'-O-methyltransferase fibrillarin OS=Homo sapiens GN=FBL PE=1 SV=2 | 33784.1 | 321 | 39.56 | 12 | 12 | 67 | 67 |
| 490 | 19.39 | sp|Q8IVT2|MISP_HUMAN | Mitotic interactor and substrate of PLK1 OS=Homo sapiens GN=MISP PE=1 SV=1 | 75355.9 | 679 | 24.59 | 10 | 10 | 24 | 24 |
| 491 | 19.36 | tr|Q6FHZ4|Q6FHZ4_HUMAN | Galectin OS=Homo sapiens GN=LGALS4 PE=2 SV=1 | 35941 | 323 | 36.53 | 11 | 10 | 227 | 225 |
| 492 | 19.34 | tr|A0A024R7V6|A0A024R7V6_HUMAN | RAB2, member RAS oncogene family, isoform CRA_a OS=Homo sapiens GN=RAB2 PE=4 SV=1 | 23545.4 | 212 | 59.91 | 13 | 12 | 87 | 85 |
| 493 | 19.33 | tr|A8K3M9|A8K3M9_HUMAN | cDNA FLJ76387, highly similar to Homo sapiens splicing factor, arginine/serine-rich 9 (SFRS9), mRNA OS=Homo sapiens PE=2 SV=1 | 25514 | 221 | 44.34 | 9 | 9 | 23 | 23 |
| 494 | 19.32 | tr|A4D0U5|A4D0U5_HUMAN | Testis derived transcript (3 LIM domains) OS=Homo sapiens GN=TES PE=4 SV=1 | 47996.1 | 421 | 23.99 | 11 | 11 | 53 | 53 |
| 495 | 19.3 | tr|Q2TB59|Q2TB59_HUMAN | Nicotinamide nucleotide transhydrogenase OS=Homo sapiens GN=NNT PE=2 SV=1 | 113882.5 | 1086 | 11.88 | 11 | 11 | 28 | 28 |
| 496 | 19.27 | tr|B4DQI7|B4DQI7_HUMAN | cDNA FLJ58042, highly similar to Protein NipSnap1 OS=Homo sapiens PE=2 SV=1 | 31406.6 | 264 | 34.85 | 10 | 9 | 68 | 64 |
| 497 | 19.26 | tr|A0A024R231|A0A024R231_HUMAN | Guanine deaminase, isoform CRA_b OS=Homo sapiens GN=GDA PE=4 SV=1 | 51002.6 | 454 | 25.55 | 10 | 10 | 57 | 57 |
| 498 | 19.25 | tr|I3L2B0|I3L2B0_HUMAN | Clustered mitochondria protein homolog (Fragment) OS=Homo sapiens GN=CLUH PE=1 SV=2 | 138227.9 | 1236 | 11.57 | 10 | 10 | 27 | 27 |
| 499 | 19.23 | tr|A0A024QZD5|A0A024QZD5_HUMAN | Small nuclear ribonucleoprotein 70kDa polypeptide (RNP antigen), isoform CRA_b OS=Homo sapiens GN=SNRP70 PE=4 SV=1 | 51556.2 | 437 | 22.88 | 10 | 10 | 78 | 77 |
| 500 | 19.22 | sp|Q8N1F7|NUP93_HUMAN | Nuclear pore complex protein Nup93 OS=Homo sapiens GN=NUP93 PE=1 SV=2 | 93487.4 | 819 | 12.09 | 10 | 10 | 35 | 35 |
| 501 | 19.2 | sp|P23229|ITA6_HUMAN | Integrin alpha-6 OS=Homo sapiens GN=ITGA6 PE=1 SV=5 | 126604.4 | 1130 | 12.48 | 11 | 11 | 35 | 35 |
| 502 | 19.17 | sp|P35527|K1C9_HUMAN | Keratin, type I cytoskeletal 9 OS=Homo sapiens GN=KRT9 PE=1 SV=3 | 62064.3 | 623 | 22.95 | 11 | 11 | 63 | 63 |
| 503 | 19.16 | tr|M0QXB4|M0QXB4_HUMAN | Coatomer protein complex, subunit epsilon, isoform CRA_g OS=Homo sapiens GN=COPE PE=1 SV=1 | 36923.3 | 331 | 29.61 | 9 | 9 | 52 | 52 |
| 504 | 19.15 | tr|A0A024R7B7|A0A024R7B7_HUMAN | CDC37 cell division cycle 37 homolog (S. cerevisiae), isoform CRA_a OS=Homo sapiens GN=CDC37 PE=4 SV=1 | 44468 | 378 | 29.89 | 10 | 10 | 44 | 44 |
| 505 | 19.14 | tr|A0A0S2Z4C3|A0A0S2Z4C3_HUMAN | Fumarate hydratase isoform 1 (Fragment) OS=Homo sapiens GN=FH PE=2 SV=1 | 54636.6 | 510 | 26.27 | 12 | 12 | 45 | 45 |
| 506 | 19.12 | tr|A8K0T9|A8K0T9_HUMAN | cDNA FLJ75422, highly similar to Homo sapiens capping protein (actin filament) muscle Z-line, alpha 1, mRNA OS=Homo sapiens PE=2 SV=1 | 32908.5 | 286 | 44.41 | 10 | 8 | 108 | 78 |
| 507 | 19.11 | tr|A8KAH7|A8KAH7_HUMAN | cDNA FLJ75444, highly similar to Homo sapiens protein kinase, cAMP-dependent, regulatory, type II, alpha (PRKAR2A), mRNA OS=Homo sapiens PE=2 SV=1 | 45504 | 404 | 32.43 | 11 | 11 | 39 | 39 |
| 508 | 19.1 | tr|A0A024R6K8|A0A024R6K8_HUMAN | Tryptophanyl-tRNA synthetase, isoform CRA_a OS=Homo sapiens GN=WARS PE=3 SV=1 | 53164.9 | 471 | 30.36 | 10 | 10 | 69 | 68 |
| 509 | 19.06 | sp|Q08257|QOR_HUMAN | Quinone oxidoreductase OS=Homo sapiens GN=CRYZ PE=1 SV=1 | 35206.4 | 329 | 34.04 | 10 | 10 | 61 | 61 |
| 510 | 19.03 | tr|Q6IAX2|Q6IAX2_HUMAN | RPL21 protein OS=Homo sapiens GN=RPL21 PE=2 SV=1 | 18564.8 | 160 | 45 | 10 | 10 | 96 | 95 |
| 511 | 18.96 | sp|Q12797|ASPH_HUMAN | Aspartyl/asparaginyl beta-hydroxylase OS=Homo sapiens GN=ASPH PE=1 SV=3 | 85862.1 | 758 | 16.49 | 11 | 10 | 31 | 30 |
| 512 | 18.95 | tr|B4DHQ3|B4DHQ3_HUMAN | Phosphoserine aminotransferase OS=Homo sapiens PE=2 SV=1 | 45355 | 415 | 25.78 | 10 | 10 | 58 | 58 |
| 513 | 18.93 | sp|Q13151|ROA0_HUMAN | Heterogeneous nuclear ribonucleoprotein A0 OS=Homo sapiens GN=HNRNPA0 PE=1 SV=1 | 30840.6 | 305 | 41.64 | 10 | 10 | 42 | 42 |
| 514 | 18.87 | tr|A0A090N7V5|A0A090N7V5_HUMAN | Chromosome 7 open reading frame 24 OS=Homo sapiens GN=C7orf24 PE=4 SV=1 | 21007.6 | 188 | 56.38 | 10 | 10 | 64 | 64 |
| 515 | 18.86 | tr|A0A024RDG1|A0A024RDG1_HUMAN | Vesicle docking protein p115, isoform CRA_a OS=Homo sapiens GN=VDP PE=4 SV=1 | 107894.4 | 962 | 14.66 | 12 | 11 | 57 | 53 |
| 516 | 18.83 | tr|A8K4Z4|A8K4Z4_HUMAN | 60S acidic ribosomal protein P0 OS=Homo sapiens PE=2 SV=1 | 34239.1 | 317 | 40.69 | 11 | 11 | 83 | 81 |
| 517 | 18.82 | tr|A0A024R904|A0A024R904_HUMAN | Calcyclin binding protein, isoform CRA_a OS=Homo sapiens GN=CACYBP PE=4 SV=1 | 26209.8 | 228 | 46.49 | 12 | 11 | 42 | 39 |
| 518 | 18.81 | sp|O14980|XPO1_HUMAN | Exportin-1 OS=Homo sapiens GN=XPO1 PE=1 SV=1 | 123385 | 1071 | 14.01 | 12 | 12 | 31 | 31 |
| 519 | 18.7 | tr|A0A024R4Q8|A0A024R4Q8_HUMAN | Ribosomal protein S5, isoform CRA_a OS=Homo sapiens GN=RPS5 PE=3 SV=1 | 22876.2 | 204 | 52.94 | 11 | 11 | 75 | 75 |
| 520 | 18.68 | sp|Q9Y446|PKP3_HUMAN | Plakophilin-3 OS=Homo sapiens GN=PKP3 PE=1 SV=1 | 87081.1 | 797 | 20.2 | 12 | 12 | 61 | 61 |
| 521 | 18.65 | tr|A3RJH1|A3RJH1_HUMAN | ATP-dependent RNA helicase DDX1 OS=Homo sapiens GN=DDX1 PE=2 SV=1 | 82431.7 | 740 | 16.89 | 11 | 11 | 36 | 36 |
| 522 | 18.57 | sp|O94906|PRP6_HUMAN | Pre-mRNA-processing factor 6 OS=Homo sapiens GN=PRPF6 PE=1 SV=1 | 106924 | 941 | 10.63 | 9 | 9 | 33 | 32 |
| 523 | 18.56 | sp|P62269|RS18_HUMAN | 40S ribosomal protein S18 OS=Homo sapiens GN=RPS18 PE=1 SV=3 | 17718.6 | 152 | 62.5 | 11 | 11 | 58 | 58 |
| 524 | 18.55 | tr|V9HW89|V9HW89_HUMAN | Epididymis secretory sperm binding protein Li 95n OS=Homo sapiens GN=HEL-S-95n PE=2 SV=1 | 38311.2 | 357 | 27.73 | 9 | 9 | 47 | 47 |
| 525 | 18.54 | sp|P61160|ARP2_HUMAN | Actin-related protein 2 OS=Homo sapiens GN=ACTR2 PE=1 SV=1 | 44760.5 | 394 | 23.86 | 9 | 9 | 97 | 97 |
| 526 | 18.51 | sp|O60547|GMDS_HUMAN | GDP-mannose 4,6 dehydratase OS=Homo sapiens GN=GMDS PE=1 SV=1 | 41949.4 | 372 | 33.87 | 9 | 9 | 37 | 37 |
| 527 | 18.5 | tr|A0A024QZ77|A0A024QZ77_HUMAN | EF-hand domain family, member D2, isoform CRA_a OS=Homo sapiens GN=EFHD2 PE=2 SV=1 | 26697 | 240 | 42.5 | 10 | 9 | 39 | 34 |
| 528 | 18.47 | tr|B3KTJ9|B3KTJ9_HUMAN | cDNA FLJ38393 fis, clone FEBRA2007212 OS=Homo sapiens PE=2 SV=1 | 102928.6 | 923 | 14.52 | 9 | 9 | 27 | 27 |
| 529 | 18.43 | tr|Q6IB58|Q6IB58_HUMAN | FLOT1 protein OS=Homo sapiens GN=FLOT1 PE=2 SV=1 | 47336.9 | 427 | 27.87 | 10 | 10 | 40 | 40 |
| 530 | 18.41 | sp|O00231|PSD11_HUMAN | 26S proteasome non-ATPase regulatory subunit 11 OS=Homo sapiens GN=PSMD11 PE=1 SV=3 | 47463.2 | 422 | 23.93 | 9 | 9 | 35 | 35 |
| 531 | 18.4 | tr|B4DRS6|B4DRS6_HUMAN | Sideroflexin OS=Homo sapiens PE=2 SV=1 | 36349.8 | 329 | 36.78 | 11 | 11 | 53 | 46 |
| 532 | 18.4 | sp|P46087|NOP2_HUMAN | Probable 28S rRNA (cytosine(4447)-C(5))-methyltransferase OS=Homo sapiens GN=NOP2 PE=1 SV=2 | 89301.1 | 812 | 16.38 | 11 | 11 | 38 | 38 |
| 533 | 18.38 | tr|A0A024R1U4|A0A024R1U4_HUMAN | RAB5C, member RAS oncogene family, isoform CRA_a OS=Homo sapiens GN=RAB5C PE=4 SV=1 | 23482.4 | 216 | 56.02 | 11 | 8 | 46 | 26 |
| 534 | 18.34 | tr|Q5SRT3|Q5SRT3_HUMAN | Chloride intracellular channel protein OS=Homo sapiens GN=CLIC1 PE=2 SV=2 | 26922.5 | 241 | 63.49 | 12 | 11 | 64 | 61 |
| 535 | 18.32 | tr|S4R3H4|S4R3H4_HUMAN | Apoptotic chromatin condensation inducer in the nucleus OS=Homo sapiens GN=ACIN1 PE=1 SV=1 | 145440.9 | 1283 | 11.3 | 10 | 9 | 27 | 25 |
| 536 | 18.29 | sp|Q9UMS4|PRP19_HUMAN | Pre-mRNA-processing factor 19 OS=Homo sapiens GN=PRPF19 PE=1 SV=1 | 55180.3 | 504 | 28.97 | 12 | 11 | 51 | 49 |
| 537 | 18.25 | tr|A0A024R588|A0A024R588_HUMAN | Splicing factor 1, isoform CRA_d OS=Homo sapiens GN=SF1 PE=4 SV=1 | 59711.7 | 548 | 20.44 | 9 | 9 | 76 | 76 |
| 538 | 18.17 | sp|O60664|PLIN3_HUMAN | Perilipin-3 OS=Homo sapiens GN=PLIN3 PE=1 SV=3 | 47074.7 | 434 | 28.57 | 9 | 9 | 51 | 50 |
| 539 | 18.14 | sp|P06703|S10A6_HUMAN | Protein S100-A6 OS=Homo sapiens GN=S100A6 PE=1 SV=1 | 10179.6 | 90 | 35.56 | 9 | 9 | 137 | 137 |
| 540 | 18.11 | sp|O43290|SNUT1_HUMAN | U4/U6.U5 tri-snRNP-associated protein 1 OS=Homo sapiens GN=SART1 PE=1 SV=1 | 90254.1 | 800 | 18.75 | 10 | 9 | 27 | 24 |
| 541 | 18.1 | sp|Q9H444|CHM4B_HUMAN | Charged multivesicular body protein 4b OS=Homo sapiens GN=CHMP4B PE=1 SV=1 | 24950 | 224 | 39.73 | 10 | 10 | 66 | 64 |
| 542 | 18.09 | tr|B4DUD5|B4DUD5_HUMAN | cDNA FLJ58787, highly similar to Cleavage stimulation factor 64 kDa subunit OS=Homo sapiens PE=2 SV=1 | 58617.7 | 553 | 25.68 | 9 | 5 | 40 | 24 |
| 543 | 17.97 | tr|Q9BR63|Q9BR63_HUMAN | FARSB protein (Fragment) OS=Homo sapiens GN=FARSB PE=2 SV=2 | 65700.4 | 585 | 17.26 | 9 | 9 | 57 | 57 |
| 544 | 17.97 | tr|S4R3V8|S4R3V8_HUMAN | Lipolysis-stimulated lipoprotein receptor OS=Homo sapiens GN=LSR PE=1 SV=2 | 66175.2 | 601 | 20.3 | 9 | 9 | 35 | 35 |
| 545 | 17.97 | tr|B4DS05|B4DS05_HUMAN | cDNA FLJ59403, highly similar to Nucleosome assembly protein 1-like 4 OS=Homo sapiens PE=2 SV=1 | 44094.5 | 386 | 26.68 | 10 | 8 | 54 | 39 |
| 546 | 17.95 | tr|Q6IAU5|Q6IAU5_HUMAN | PPM1G protein OS=Homo sapiens GN=PPM1G PE=2 SV=1 | 59271.1 | 546 | 20.88 | 10 | 10 | 31 | 31 |
| 547 | 17.92 | tr|Q14TF0|Q14TF0_HUMAN | Glutamate-cysteine ligase OS=Homo sapiens GN=GCLC PE=2 SV=1 | 72765.1 | 637 | 20.88 | 10 | 10 | 27 | 27 |
| 548 | 17.91 | tr|V9HVZ0|V9HVZ0_HUMAN | Epididymis secretory protein Li 91 OS=Homo sapiens GN=HEL-S-91 PE=2 SV=1 | 39567.3 | 344 | 23.26 | 10 | 7 | 51 | 33 |
| 549 | 17.9 | tr|A0A024R5Q7|A0A024R5Q7_HUMAN | Adenylosuccinate synthetase isozyme 2 OS=Homo sapiens GN=ADSS PE=3 SV=1 | 50097.1 | 456 | 25.66 | 10 | 10 | 49 | 49 |
| 550 | 17.83 | tr|B2R6S5|B2R6S5_HUMAN | UMP-CMP kinase OS=Homo sapiens GN=CMPK PE=2 SV=1 | 25854.6 | 228 | 53.51 | 10 | 10 | 66 | 56 |
| 551 | 17.8 | tr|A0A024R9N6|A0A024R9N6_HUMAN | EH-domain containing 4, isoform CRA_a OS=Homo sapiens GN=EHD4 PE=3 SV=1 | 61174.6 | 541 | 23.29 | 10 | 9 | 38 | 30 |
| 552 | 17.73 | tr|B4DWA6|B4DWA6_HUMAN | cDNA FLJ60094, highly similar to F-actin capping protein subunit beta OS=Homo sapiens PE=2 SV=1 | 37482 | 335 | 34.63 | 9 | 9 | 61 | 61 |
| 553 | 17.71 | tr|A0A024R4Z6|A0A024R4Z6_HUMAN | Structure specific recognition protein 1, isoform CRA_a OS=Homo sapiens GN=SSRP1 PE=4 SV=1 | 81074.2 | 709 | 15.8 | 9 | 9 | 36 | 36 |
| 554 | 17.68 | tr|A0A024R5X2|A0A024R5X2_HUMAN | HCG2001986, isoform CRA_a OS=Homo sapiens GN=hCG_2001986 PE=4 SV=1 | 49960.3 | 450 | 24.22 | 9 | 9 | 33 | 33 |
| 555 | 17.67 | sp|P28074|PSB5_HUMAN | Proteasome subunit beta type-5 OS=Homo sapiens GN=PSMB5 PE=1 SV=3 | 28480 | 263 | 32.32 | 10 | 9 | 42 | 36 |
| 556 | 17.65 | tr|Q53FF5|Q53FF5_HUMAN | p47 protein isoform a variant (Fragment) OS=Homo sapiens PE=2 SV=1 | 40499.4 | 370 | 31.62 | 9 | 9 | 47 | 47 |
| 557 | 17.6 | tr|Q59FR8|Q59FR8_HUMAN | Galectin (Fragment) OS=Homo sapiens PE=2 SV=1 | 27117.3 | 258 | 33.33 | 10 | 10 | 75 | 75 |
| 558 | 17.59 | sp|P18583|SON_HUMAN | Protein SON OS=Homo sapiens GN=SON PE=1 SV=4 | 263827.3 | 2426 | 5.936 | 10 | 10 | 19 | 19 |
| 559 | 17.56 | tr|H7C2I1|H7C2I1_HUMAN | Protein arginine N-methyltransferase 1 OS=Homo sapiens GN=PRMT1 PE=1 SV=1 | 42461.3 | 371 | 31.27 | 9 | 9 | 38 | 38 |
| 560 | 17.49 | tr|Q53HS0|Q53HS0_HUMAN | Glutaminyl-tRNA synthetase variant (Fragment) OS=Homo sapiens PE=2 SV=1 | 87709.9 | 775 | 16.77 | 10 | 10 | 39 | 38 |
| 561 | 17.47 | sp|Q92896|GSLG1_HUMAN | Golgi apparatus protein 1 OS=Homo sapiens GN=GLG1 PE=1 SV=2 | 134550.6 | 1179 | 8.227 | 9 | 9 | 25 | 25 |
| 562 | 17.47 | tr|Q5VVD0|Q5VVD0_HUMAN | Ribosomal protein L11, isoform CRA_b OS=Homo sapiens GN=RPL11 PE=2 SV=1 | 20252.2 | 178 | 35.96 | 9 | 2 | 77 | 13 |
| 563 | 17.46 | sp|P62263|RS14_HUMAN | 40S ribosomal protein S14 OS=Homo sapiens GN=RPS14 PE=1 SV=3 | 16272.6 | 151 | 45.7 | 9 | 9 | 110 | 110 |
| 564 | 17.4 | tr|J3KPF3|J3KPF3_HUMAN | 4F2 cell-surface antigen heavy chain OS=Homo sapiens GN=SLC3A2 PE=1 SV=1 | 68100.3 | 631 | 19.02 | 10 | 10 | 34 | 34 |
| 565 | 17.35 | sp|P42224|STAT1_HUMAN | Signal transducer and activator of transcription 1-alpha/beta OS=Homo sapiens GN=STAT1 PE=1 SV=2 | 87334.2 | 750 | 15.87 | 9 | 9 | 46 | 46 |
| 566 | 17.35 | sp|Q6ZRV2|FA83H_HUMAN | Protein FAM83H OS=Homo sapiens GN=FAM83H PE=1 SV=3 | 127121 | 1179 | 14.93 | 11 | 11 | 31 | 31 |
| 567 | 17.27 | tr|A0A024R395|A0A024R395_HUMAN | MRE11 meiotic recombination 11 homolog A (S. cerevisiae), isoform CRA_a OS=Homo sapiens GN=MRE11A PE=3 SV=1 | 80592.6 | 708 | 14.55 | 9 | 9 | 18 | 18 |
| 568 | 17.25 | tr|Q59GE4|Q59GE4_HUMAN | Ribosomal protein S10 variant (Fragment) OS=Homo sapiens PE=4 SV=1 | 19859.8 | 174 | 31.61 | 9 | 8 | 77 | 75 |
| 569 | 17.19 | tr|B3KWX7|B3KWX7_HUMAN | cDNA FLJ44170 fis, clone THYMU2035319, highly similar to RNA-binding region-containing protein 2 OS=Homo sapiens PE=2 SV=1 | 56743.3 | 506 | 17.98 | 9 | 9 | 62 | 62 |
| 570 | 17.15 | tr|B4DF00|B4DF00_HUMAN | cDNA FLJ53308, highly similar to 2-oxoglutarate dehydrogenase E1 component, mitochondrial (EC 1.2.4.2) OS=Homo sapiens PE=2 SV=1 | 110525.5 | 974 | 13.66 | 9 | 9 | 26 | 26 |
| 571 | 17.15 | sp|P67809|YBOX1_HUMAN | Nuclease-sensitive element-binding protein 1 OS=Homo sapiens GN=YBX1 PE=1 SV=3 | 35924.1 | 324 | 54.01 | 10 | 7 | 50 | 36 |
| 572 | 17.12 | tr|V9HW56|V9HW56_HUMAN | Epididymis secretory protein Li 108 OS=Homo sapiens GN=HEL-S-108 PE=2 SV=1 | 28521.5 | 248 | 59.27 | 24 | 10 | 163 | 33 |
| 573 | 17.11 | sp|P30050|RL12_HUMAN | 60S ribosomal protein L12 OS=Homo sapiens GN=RPL12 PE=1 SV=1 | 17818.4 | 165 | 60.61 | 7 | 7 | 187 | 187 |
| 574 | 17.08 | sp|Q08378|GOGA3_HUMAN | Golgin subfamily A member 3 OS=Homo sapiens GN=GOLGA3 PE=1 SV=2 | 167353.4 | 1498 | 9.68 | 11 | 11 | 20 | 18 |
| 575 | 17.05 | sp|P62906|RL10A_HUMAN | 60S ribosomal protein L10a OS=Homo sapiens GN=RPL10A PE=1 SV=2 | 24831.1 | 217 | 40.09 | 10 | 10 | 96 | 93 |
| 576 | 17.04 | tr|A6NFX8|A6NFX8_HUMAN | ADP-sugar pyrophosphatase OS=Homo sapiens GN=NUDT5 PE=1 SV=1 | 25895.3 | 232 | 43.53 | 10 | 10 | 52 | 52 |
| 577 | 17.03 | tr|H0Y4R1|H0Y4R1_HUMAN | Inosine-5'-monophosphate dehydrogenase 2 (Fragment) OS=Homo sapiens GN=IMPDH2 PE=1 SV=1 | 51014.3 | 470 | 22.98 | 12 | 11 | 27 | 17 |
| 578 | 17.02 | sp|O94832|MYO1D_HUMAN | Unconventional myosin-Id OS=Homo sapiens GN=MYO1D PE=1 SV=2 | 116201.3 | 1006 | 9.642 | 9 | 9 | 32 | 32 |
| 579 | 17.02 | tr|Q2TU34|Q2TU34_HUMAN | Fructose-1,6-bisphosphatase 1 OS=Homo sapiens GN=FBP1 PE=2 SV=1 | 36814.1 | 338 | 32.25 | 11 | 11 | 48 | 48 |
| 580 | 17 | tr|A0A0S2Z3S5|A0A0S2Z3S5_HUMAN | GNAS complex locus isoform 2 (Fragment) OS=Homo sapiens GN=GNAS PE=2 SV=1 | 44265.8 | 380 | 23.95 | 9 | 7 | 83 | 39 |
| 581 | 16.99 | tr|B4DPV7|B4DPV7_HUMAN | cDNA FLJ54534, highly similar to Homo sapiens cysteinyl-tRNA synthetase (CARS), transcript variant 3, mRNA OS=Homo sapiens PE=2 SV=1 | 92017.8 | 809 | 11.12 | 9 | 9 | 24 | 24 |
| 582 | 16.99 | tr|A8K6I4|A8K6I4_HUMAN | cDNA FLJ76877, highly similar to Homo sapiens superkiller viralicidic activity 2-like 2 (SKIV2L2), mRNA OS=Homo sapiens PE=2 SV=1 | 117897.8 | 1042 | 13.15 | 12 | 12 | 24 | 24 |
| 583 | 16.98 | tr|A0A024R8L8|A0A024R8L8_HUMAN | Envoplakin, isoform CRA_a OS=Homo sapiens GN=EVPL PE=4 SV=1 | 231630.3 | 2033 | 7.673 | 12 | 12 | 23 | 23 |
| 584 | 16.97 | sp|P20290|BTF3_HUMAN | Transcription factor BTF3 OS=Homo sapiens GN=BTF3 PE=1 SV=1 | 22167.8 | 206 | 59.71 | 8 | 8 | 24 | 23 |
| 585 | 16.96 | tr|A0A0S2Z410|A0A0S2Z410_HUMAN | Hydroxysteroid dehydrogenase 10 isoform 1 (Fragment) OS=Homo sapiens GN=HSD17B10 PE=2 SV=1 | 26922.9 | 261 | 46.74 | 8 | 8 | 100 | 100 |
| 586 | 16.94 | tr|B4DDB6|B4DDB6_HUMAN | Heterogeneous nuclear ribonucleoprotein A3, isoform CRA_a OS=Homo sapiens GN=HNRPA3 PE=2 SV=1 | 37029.1 | 356 | 36.24 | 10 | 10 | 87 | 72 |
| 587 | 16.91 | tr|B3KSH1|B3KSH1_HUMAN | Eukaryotic translation initiation factor 3 subunit F OS=Homo sapiens GN=EIF3F PE=2 SV=1 | 39146.3 | 372 | 32.26 | 8 | 8 | 78 | 77 |
| 588 | 16.9 | sp|P35270|SPRE_HUMAN | Sepiapterin reductase OS=Homo sapiens GN=SPR PE=1 SV=1 | 28048.1 | 261 | 46.36 | 10 | 9 | 56 | 55 |
| 589 | 16.89 | tr|B2R4W8|B2R4W8_HUMAN | HCG1994130, isoform CRA_a OS=Homo sapiens GN=hCG_1994130 PE=2 SV=1 | 14839.4 | 130 | 61.54 | 8 | 8 | 101 | 101 |
| 590 | 16.86 | tr|A0A024RDH6|A0A024RDH6_HUMAN | SEC31-like 1 (S. cerevisiae), isoform CRA_b OS=Homo sapiens GN=SEC31L1 PE=4 SV=1 | 129035.4 | 1181 | 8.637 | 9 | 9 | 35 | 35 |
| 591 | 16.86 | sp|O15160|RPAC1_HUMAN | DNA-directed RNA polymerases I and III subunit RPAC1 OS=Homo sapiens GN=POLR1C PE=1 SV=1 | 39249.4 | 346 | 34.97 | 9 | 9 | 30 | 30 |
| 592 | 16.83 | sp|P17301|ITA2_HUMAN | Integrin alpha-2 OS=Homo sapiens GN=ITGA2 PE=1 SV=1 | 129294.4 | 1181 | 9.822 | 9 | 2 | 22 | 5 |
| 593 | 16.83 | tr|V9HWC9|V9HWC9_HUMAN | Superoxide dismutase [Cu-Zn] OS=Homo sapiens GN=HEL-S-44 PE=2 SV=1 | 15935.7 | 154 | 88.96 | 9 | 9 | 38 | 38 |
| 594 | 16.83 | tr|D6W5K2|D6W5K2_HUMAN | Thymosin, beta 10, isoform CRA_a (Fragment) OS=Homo sapiens GN=TMSB10 PE=4 SV=1 | 17941.2 | 169 | 23.67 | 10 | 9 | 190 | 188 |
| 595 | 16.81 | tr|B0QZ18|B0QZ18_HUMAN | Copine-1 OS=Homo sapiens GN=CPNE1 PE=1 SV=1 | 59717.2 | 542 | 16.97 | 8 | 8 | 33 | 31 |
| 596 | 16.8 | tr|A0A087X1N8|A0A087X1N8_HUMAN | Serpin B6 OS=Homo sapiens GN=SERPINB6 PE=1 SV=1 | 44822.1 | 395 | 30.63 | 9 | 9 | 54 | 54 |
| 597 | 16.79 | tr|Q53FB6|Q53FB6_HUMAN | Mitochondrial aldehyde dehydrogenase 2 variant (Fragment) OS=Homo sapiens PE=2 SV=1 | 56366.9 | 517 | 25.92 | 11 | 10 | 76 | 60 |
| 598 | 16.77 | tr|B2R4R9|B2R4R9_HUMAN | HCG26477 OS=Homo sapiens GN=RPS28 PE=2 SV=1 | 7841 | 69 | 60.87 | 8 | 8 | 85 | 85 |
| 599 | 16.75 | sp|O76021|RL1D1_HUMAN | Ribosomal L1 domain-containing protein 1 OS=Homo sapiens GN=RSL1D1 PE=1 SV=3 | 54972 | 490 | 19.59 | 9 | 9 | 46 | 45 |
| 600 | 16.74 | tr|Q9BTQ7|Q9BTQ7_HUMAN | Similar to ribosomal protein L23 (Fragment) OS=Homo sapiens PE=2 SV=1 | 14149.5 | 134 | 71.64 | 9 | 9 | 95 | 94 |
| 601 | 16.72 | tr|A0A024R8U5|A0A024R8U5_HUMAN | Splicing factor, arginine/serine-rich 2, isoform CRA_a OS=Homo sapiens GN=SFRS2 PE=4 SV=1 | 25476 | 221 | 28.96 | 9 | 6 | 98 | 72 |
| 602 | 16.7 | tr|G3V1C3|G3V1C3_HUMAN | Apoptosis inhibitor 5 OS=Homo sapiens GN=API5 PE=1 SV=1 | 57560.6 | 510 | 17.25 | 9 | 5 | 33 | 13 |
| 603 | 16.64 | tr|B3KTS4|B3KTS4_HUMAN | cDNA FLJ38665 fis, clone HLUNG2003378, highly similar to Rho guanine nucleotide exchange factor 16 OS=Homo sapiens PE=2 SV=1 | 80183.4 | 709 | 20.03 | 9 | 9 | 23 | 22 |
| 604 | 16.64 | sp|Q15785|TOM34_HUMAN | Mitochondrial import receptor subunit TOM34 OS=Homo sapiens GN=TOMM34 PE=1 SV=2 | 34559 | 309 | 31.39 | 9 | 9 | 29 | 29 |
| 605 | 16.63 | sp|Q5SSJ5|HP1B3_HUMAN | Heterochromatin protein 1-binding protein 3 OS=Homo sapiens GN=HP1BP3 PE=1 SV=1 | 61206.5 | 553 | 16.27 | 9 | 9 | 41 | 41 |
| 606 | 16.61 | sp|P39656|OST48_HUMAN | Dolichyl-diphosphooligosaccharide--protein glycosyltransferase 48 kDa subunit OS=Homo sapiens GN=DDOST PE=1 SV=4 | 50800.3 | 456 | 26.54 | 9 | 9 | 83 | 83 |
| 607 | 16.59 | tr|A0A0S2Z4W4|A0A0S2Z4W4_HUMAN | RNA binding motif protein 10 isoform 2 (Fragment) OS=Homo sapiens GN=RBM10 PE=2 SV=1 | 103531.9 | 930 | 16.67 | 8 | 8 | 20 | 20 |
| 608 | 16.58 | sp|Q13442|HAP28_HUMAN | 28 kDa heat- and acid-stable phosphoprotein OS=Homo sapiens GN=PDAP1 PE=1 SV=1 | 20629.9 | 181 | 31.49 | 8 | 8 | 73 | 73 |
| 609 | 16.53 | sp|Q12904|AIMP1_HUMAN | Aminoacyl tRNA synthase complex-interacting multifunctional protein 1 OS=Homo sapiens GN=AIMP1 PE=1 SV=2 | 34352.4 | 312 | 39.1 | 8 | 8 | 43 | 43 |
| 610 | 16.48 | tr|Q53HM8|Q53HM8_HUMAN | Annexin (Fragment) OS=Homo sapiens PE=2 SV=1 | 50227.3 | 466 | 20.17 | 9 | 9 | 26 | 25 |
| 611 | 16.43 | tr|A0A024RDG6|A0A024RDG6_HUMAN | Scavenger receptor class B, member 2, isoform CRA_a OS=Homo sapiens GN=SCARB2 PE=3 SV=1 | 54289.7 | 478 | 14.85 | 8 | 8 | 56 | 56 |
| 612 | 16.38 | tr|B2RD79|B2RD79_HUMAN | cDNA, FLJ96494, highly similar to Homo sapiens ubiquitin specific peptidase 14 (tRNA-guanine transglycosylase) (USP14), mRNA OS=Homo sapiens PE=2 SV=1 | 56041.6 | 494 | 21.05 | 9 | 9 | 68 | 68 |
| 613 | 16.38 | tr|Q53GF9|Q53GF9_HUMAN | Full-length cDNA 5-PRIME end of clone CS0DF013YM24 of Fetal brain of Homo sapiens (Human) variant (Fragment) OS=Homo sapiens PE=2 SV=1 | 25636.3 | 225 | 30.22 | 8 | 8 | 67 | 67 |
| 614 | 16.36 | sp|O95834|EMAL2_HUMAN | Echinoderm microtubule-associated protein-like 2 OS=Homo sapiens GN=EML2 PE=1 SV=1 | 70678.3 | 649 | 18.49 | 9 | 9 | 37 | 37 |
| 615 | 16.3 | tr|A0A0S2Z422|A0A0S2Z422_HUMAN | Isovaleryl-CoA dehydrogenase isoform 1 (Fragment) OS=Homo sapiens GN=IVD PE=2 SV=1 | 46319 | 423 | 23.17 | 8 | 8 | 62 | 62 |
| 616 | 16.28 | tr|B2R761|B2R761_HUMAN | cDNA, FLJ93299, highly similar to Homo sapiens sterol carrier protein 2 (SCP2), mRNA OS=Homo sapiens PE=2 SV=1 | 59021.3 | 547 | 13.89 | 9 | 9 | 44 | 44 |
| 617 | 16.28 | sp|O75396|SC22B_HUMAN | Vesicle-trafficking protein SEC22b OS=Homo sapiens GN=SEC22B PE=1 SV=4 | 24593.1 | 215 | 47.44 | 9 | 9 | 68 | 68 |
| 618 | 16.27 | tr|B3GQE6|B3GQE6_HUMAN | DEAD box polypeptide 27 OS=Homo sapiens GN=DDX27 PE=2 SV=1 | 86633.8 | 765 | 12.03 | 9 | 9 | 42 | 41 |
| 619 | 16.24 | tr|B4E327|B4E327_HUMAN | cDNA FLJ58830, highly similar to Homo sapiens zinc finger protein 289, ID1 regulated (ZNF289), mRNA OS=Homo sapiens PE=2 SV=1 | 41176.7 | 382 | 29.58 | 9 | 9 | 44 | 43 |
| 620 | 16.23 | sp|P11177|ODPB_HUMAN | Pyruvate dehydrogenase E1 component subunit beta, mitochondrial OS=Homo sapiens GN=PDHB PE=1 SV=3 | 39233.1 | 359 | 28.97 | 9 | 9 | 56 | 55 |
| 621 | 16.22 | tr|A0A024R325|A0A024R325_HUMAN | Succinate-CoA ligase subunit beta OS=Homo sapiens GN=SUCLG2 PE=3 SV=1 | 46510.2 | 432 | 25.23 | 9 | 9 | 36 | 36 |
| 622 | 16.17 | tr|H9KV75|H9KV75_HUMAN | Alpha-actinin-1 OS=Homo sapiens GN=ACTN1 PE=1 SV=1 | 94825 | 822 | 30.54 | 21 | 10 | 286 | 41 |
| 623 | 16.17 | tr|B7Z3K9|B7Z3K9_HUMAN | Fructose-bisphosphate aldolase OS=Homo sapiens PE=2 SV=1 | 48407.8 | 451 | 29.71 | 14 | 9 | 148 | 39 |
| 624 | 16.17 | tr|A0A024R2M6|A0A024R2M6_HUMAN | Acetyl-Coenzyme A acyltransferase 1 (Peroxisomal 3-oxoacyl-Coenzyme A thiolase), isoform CRA_a OS=Homo sapiens GN=ACAA1 PE=3 SV=1 | 44291.6 | 424 | 29.72 | 9 | 9 | 29 | 29 |
| 625 | 16.17 | tr|C9JJX6|C9JJX6_HUMAN | Armadillo repeat protein deleted in velo-cardio-facial syndrome OS=Homo sapiens GN=ARVCF PE=1 SV=1 | 96827.1 | 893 | 14.11 | 8 | 8 | 13 | 13 |
| 626 | 16.17 | sp|Q10713|MPPA_HUMAN | Mitochondrial-processing peptidase subunit alpha OS=Homo sapiens GN=PMPCA PE=1 SV=2 | 58252.4 | 525 | 19.62 | 9 | 9 | 39 | 39 |
| 627 | 16.14 | tr|B0QY89|B0QY89_HUMAN | Eukaryotic translation initiation factor 3 subunit L OS=Homo sapiens GN=EIF3L PE=1 SV=1 | 70901.3 | 607 | 14.5 | 8 | 8 | 21 | 21 |
| 628 | 16.09 | tr|A0A0K0K1K7|A0A0K0K1K7_HUMAN | 6-phosphogluconolactonase, isoform CRA_b OS=Homo sapiens GN=HEL-S-304 PE=2 SV=1 | 27546.5 | 258 | 52.33 | 9 | 9 | 66 | 66 |
| 629 | 16.07 | tr|A0A024R0R9|A0A024R0R9_HUMAN | N-ethylmaleimide-sensitive factor attachment protein, alpha, isoform CRA_c OS=Homo sapiens GN=NAPA PE=4 SV=1 | 33232.3 | 295 | 40 | 10 | 10 | 40 | 40 |
| 630 | 16.02 | tr|G1UI22|G1UI22_HUMAN | Afadin OS=Homo sapiens GN=MLLT4 PE=2 SV=1 | 201817.9 | 1781 | 7.524 | 12 | 11 | 27 | 26 |
| 631 | 16.02 | tr|A0A024RAD8|A0A024RAD8_HUMAN | Aldehyde dehydrogenase 4 family, member A1, isoform CRA_a OS=Homo sapiens GN=ALDH4A1 PE=3 SV=1 | 61718.9 | 563 | 17.41 | 8 | 8 | 43 | 43 |
| 632 | 16.02 | sp|P56537|IF6_HUMAN | Eukaryotic translation initiation factor 6 OS=Homo sapiens GN=EIF6 PE=1 SV=1 | 26598.8 | 245 | 46.12 | 8 | 8 | 62 | 62 |
| 633 | 16.02 | tr|A0A087WV66|A0A087WV66_HUMAN | Antigen KI-67 OS=Homo sapiens GN=MKI67 PE=1 SV=1 | 358623.9 | 3255 | 6.82 | 14 | 14 | 21 | 21 |
| 634 | 15.97 | sp|Q9Y6C9|MTCH2_HUMAN | Mitochondrial carrier homolog 2 OS=Homo sapiens GN=MTCH2 PE=1 SV=1 | 33330.6 | 303 | 34.32 | 8 | 8 | 42 | 39 |
| 635 | 15.96 | tr|B4E2Z6|B4E2Z6_HUMAN | Eukaryotic translation initiation factor 3 subunit C OS=Homo sapiens GN=EIF3C PE=2 SV=1 | 90536.3 | 784 | 13.9 | 9 | 9 | 49 | 49 |
| 636 | 15.95 | sp|P49321|NASP_HUMAN | Nuclear autoantigenic sperm protein OS=Homo sapiens GN=NASP PE=1 SV=2 | 85237.2 | 788 | 15.23 | 8 | 8 | 43 | 43 |
| 637 | 15.93 | sp|O95433|AHSA1_HUMAN | Activator of 90 kDa heat shock protein ATPase homolog 1 OS=Homo sapiens GN=AHSA1 PE=1 SV=1 | 38274.1 | 338 | 21.01 | 9 | 9 | 19 | 19 |
| 638 | 15.87 | tr|V9HW71|V9HW71_HUMAN | Endoplasmic reticulum resident protein 29 OS=Homo sapiens GN=HEL-S-107 PE=2 SV=1 | 28993.2 | 261 | 35.25 | 10 | 10 | 143 | 142 |
| 639 | 15.83 | tr|Q53XC0|Q53XC0_HUMAN | Eukaryotic translation initiation factor 2, subunit 1 alpha, 35kDa, isoform CRA_a OS=Homo sapiens GN=EIF2S1 PE=2 SV=1 | 36111.8 | 315 | 34.92 | 8 | 8 | 34 | 34 |
| 640 | 15.82 | tr|Q8NCF7|Q8NCF7_HUMAN | cDNA FLJ90278 fis, clone NT2RP1000325, highly similar to Phosphate carrier protein, mitochondrialprecursor OS=Homo sapiens PE=2 SV=1 | 39932.3 | 361 | 19.67 | 7 | 7 | 60 | 60 |
| 641 | 15.79 | tr|J3QQ67|J3QQ67_HUMAN | 60S ribosomal protein L18 (Fragment) OS=Homo sapiens GN=RPL18 PE=1 SV=1 | 21785.4 | 190 | 30.53 | 8 | 8 | 116 | 115 |
| 642 | 15.78 | sp|P35658|NU214_HUMAN | Nuclear pore complex protein Nup214 OS=Homo sapiens GN=NUP214 PE=1 SV=2 | 213617.6 | 2090 | 6.029 | 9 | 8 | 29 | 28 |
| 643 | 15.76 | sp|P30044|PRDX5_HUMAN | Peroxiredoxin-5, mitochondrial OS=Homo sapiens GN=PRDX5 PE=1 SV=4 | 22086.2 | 214 | 46.26 | 8 | 8 | 105 | 103 |
| 644 | 15.76 | tr|Q86YI5|Q86YI5_HUMAN | Acetyltransferase component of pyruvate dehydrogenase complex OS=Homo sapiens GN=DLAT PE=2 SV=1 | 68996 | 647 | 11.9 | 8 | 7 | 59 | 58 |
| 645 | 15.73 | sp|P18077|RL35A_HUMAN | 60S ribosomal protein L35a OS=Homo sapiens GN=RPL35A PE=1 SV=2 | 12537.6 | 110 | 52.73 | 9 | 9 | 20 | 20 |
| 646 | 15.71 | sp|Q16851|UGPA_HUMAN | UTP--glucose-1-phosphate uridylyltransferase OS=Homo sapiens GN=UGP2 PE=1 SV=5 | 56939.8 | 508 | 21.46 | 8 | 8 | 34 | 34 |
| 647 | 15.7 | tr|B4DDI9|B4DDI9_HUMAN | NADPH:adrenodoxin oxidoreductase, mitochondrial OS=Homo sapiens PE=2 SV=1 | 53161.6 | 489 | 19.22 | 9 | 9 | 14 | 14 |
| 648 | 15.69 | tr|D9IAI1|D9IAI1_HUMAN | Epididymis secretory protein Li 34 OS=Homo sapiens GN=PEBP1 PE=2 SV=1 | 21056.6 | 187 | 61.5 | 7 | 7 | 92 | 92 |
| 649 | 15.64 | sp|Q9Y2X3|NOP58_HUMAN | Nucleolar protein 58 OS=Homo sapiens GN=NOP58 PE=1 SV=1 | 59577.7 | 529 | 19.47 | 8 | 8 | 50 | 50 |
| 650 | 15.64 | tr|Q8WVC2|Q8WVC2_HUMAN | 40S ribosomal protein S21 OS=Homo sapiens GN=RPS21 PE=1 SV=1 | 8850 | 81 | 75.31 | 7 | 7 | 82 | 82 |
| 651 | 15.61 | tr|B2RA56|B2RA56_HUMAN | Nicalin OS=Homo sapiens PE=2 SV=1 | 61758.5 | 552 | 22.1 | 9 | 9 | 27 | 27 |
| 652 | 15.61 | tr|B2R6F3|B2R6F3_HUMAN | Splicing factor arginine/serine-rich 3 OS=Homo sapiens GN=SFRS3 PE=2 SV=1 | 19329.4 | 164 | 45.73 | 9 | 8 | 62 | 60 |
| 653 | 15.6 | tr|E9PF82|E9PF82_HUMAN | Calcium/calmodulin-dependent protein kinase type II subunit delta OS=Homo sapiens GN=CAMK2D PE=1 SV=1 | 60004.1 | 533 | 15.57 | 8 | 8 | 34 | 34 |
| 654 | 15.58 | tr|A0A024R9D3|A0A024R9D3_HUMAN | Ribosomal protein L30, isoform CRA_b OS=Homo sapiens GN=RPL30 PE=3 SV=1 | 12783.9 | 115 | 62.61 | 10 | 10 | 85 | 71 |
| 655 | 15.56 | tr|Q6FHU3|Q6FHU3_HUMAN | PSME1 protein (Fragment) OS=Homo sapiens GN=PSME1 PE=2 SV=1 | 28708.9 | 249 | 33.33 | 8 | 8 | 35 | 35 |
| 656 | 15.55 | tr|V9HW92|V9HW92_HUMAN | Epididymis secretory protein Li 112 OS=Homo sapiens GN=HEL-S-112 PE=2 SV=1 | 36071.5 | 329 | 41.95 | 9 | 9 | 59 | 58 |
| 657 | 15.49 | tr|V9HWI0|V9HWI0_HUMAN | Epididymis secretory protein Li 6 OS=Homo sapiens GN=HEL-S-165mP PE=2 SV=1 | 36572.7 | 325 | 39.08 | 8 | 8 | 62 | 62 |
| 658 | 15.46 | tr|A8K517|A8K517_HUMAN | Ribosomal protein S23, isoform CRA_a OS=Homo sapiens GN=RPS23 PE=2 SV=1 | 15807.5 | 143 | 38.46 | 8 | 8 | 62 | 62 |
| 659 | 15.44 | tr|Q59GA1|Q59GA1_HUMAN | Splicing factor, arginine/serine-rich 10 (Transformer 2 homolog, Drosophila) variant (Fragment) OS=Homo sapiens PE=2 SV=1 | 32273.9 | 278 | 25.18 | 8 | 6 | 32 | 26 |
| 660 | 15.44 | tr|Q53F48|Q53F48_HUMAN | Heterogeneous nuclear ribonucleoprotein H3 isoform a variant (Fragment) OS=Homo sapiens PE=2 SV=1 | 36925.5 | 346 | 29.48 | 8 | 8 | 81 | 78 |
| 661 | 15.43 | tr|B2RB06|B2RB06_HUMAN | cDNA, FLJ95242, highly similar to Homo sapiens L-3-hydroxyacyl-Coenzyme A dehydrogenase, short chain (HADHSC), mRNA OS=Homo sapiens PE=2 SV=1 | 34261.2 | 314 | 42.04 | 9 | 9 | 41 | 41 |
| 662 | 15.42 | tr|Q5H9N4|Q5H9N4_HUMAN | Putative uncharacterized protein DKFZp686L20222 OS=Homo sapiens GN=DKFZp686L20222 PE=3 SV=1 | 34778 | 303 | 29.37 | 9 | 7 | 48 | 20 |
| 663 | 15.38 | tr|E7EMK3|E7EMK3_HUMAN | Flotillin-2 OS=Homo sapiens GN=FLOT2 PE=1 SV=1 | 53137 | 483 | 17.18 | 9 | 9 | 22 | 21 |
| 664 | 15.37 | sp|P17858|PFKAL_HUMAN | ATP-dependent 6-phosphofructokinase, liver type OS=Homo sapiens GN=PFKL PE=1 SV=6 | 85017.8 | 780 | 23.59 | 15 | 10 | 67 | 37 |
| 665 | 15.35 | tr|A0A087WW66|A0A087WW66_HUMAN | 26S proteasome non-ATPase regulatory subunit 1 OS=Homo sapiens GN=PSMD1 PE=1 SV=1 | 105850.4 | 953 | 11.23 | 9 | 9 | 24 | 24 |
| 666 | 15.35 | tr|E7D7Y0|E7D7Y0_HUMAN | Pyrroline-5-carboxylate reductase OS=Homo sapiens PE=2 SV=1 | 33432.3 | 319 | 31.97 | 7 | 6 | 29 | 27 |
| 667 | 15.34 | tr|Q6URC4|Q6URC4_HUMAN | Diaphanous 1 OS=Homo sapiens PE=2 SV=1 | 141302 | 1272 | 8.648 | 10 | 10 | 37 | 35 |
| 668 | 15.34 | sp|P31689|DNJA1_HUMAN | DnaJ homolog subfamily A member 1 OS=Homo sapiens GN=DNAJA1 PE=1 SV=2 | 44868 | 397 | 20.4 | 7 | 7 | 15 | 15 |
| 669 | 15.33 | tr|H9ZYJ2|H9ZYJ2_HUMAN | Thioredoxin OS=Homo sapiens GN=TXN PE=2 SV=1 | 11737.4 | 105 | 51.43 | 8 | 8 | 100 | 100 |
| 670 | 15.29 | sp|Q15020|SART3_HUMAN | Squamous cell carcinoma antigen recognized by T-cells 3 OS=Homo sapiens GN=SART3 PE=1 SV=1 | 109933.5 | 963 | 12.56 | 11 | 10 | 19 | 16 |
| 671 | 15.29 | tr|B3KML1|B3KML1_HUMAN | cDNA FLJ11308 fis, clone PLACE1010074, highly similar to Sorting nexin-2 OS=Homo sapiens PE=2 SV=1 | 58471.5 | 519 | 15.41 | 7 | 6 | 20 | 17 |
| 672 | 15.27 | tr|Q4JM47|Q4JM47_HUMAN | AGR2 OS=Homo sapiens GN=AGR2 PE=4 SV=1 | 22237.4 | 195 | 48.21 | 9 | 9 | 139 | 136 |
| 673 | 15.25 | sp|P12532|KCRU_HUMAN | Creatine kinase U-type, mitochondrial OS=Homo sapiens GN=CKMT1A PE=1 SV=1 | 47036.3 | 417 | 27.58 | 10 | 8 | 33 | 28 |
| 674 | 15.25 | sp|P11388|TOP2A_HUMAN | DNA topoisomerase 2-alpha OS=Homo sapiens GN=TOP2A PE=1 SV=3 | 174383.9 | 1531 | 7.642 | 9 | 8 | 22 | 20 |
| 675 | 15.23 | tr|A0A024RDF4|A0A024RDF4_HUMAN | Heterogeneous nuclear ribonucleoprotein D (AU-rich element RNA binding protein 1, 37kDa), isoform CRA_e OS=Homo sapiens GN=HNRPD PE=4 SV=1 | 32834.4 | 306 | 32.03 | 10 | 9 | 78 | 48 |
| 676 | 15.22 | tr|H7BYY1|H7BYY1_HUMAN | Tropomyosin 1 (Alpha), isoform CRA_m OS=Homo sapiens GN=TPM1 PE=1 SV=1 | 28746.8 | 248 | 58.87 | 24 | 11 | 164 | 32 |
| 677 | 15.2 | tr|A0A024RDH8|A0A024RDH8_HUMAN | Ribosomal protein L34, isoform CRA_a OS=Homo sapiens GN=RPL34 PE=4 SV=1 | 13292.9 | 117 | 48.72 | 8 | 8 | 54 | 54 |
| 678 | 15.17 | tr|Q6FGD7|Q6FGD7_HUMAN | TBCA protein OS=Homo sapiens GN=TBCA PE=2 SV=1 | 12854.7 | 108 | 62.04 | 8 | 8 | 62 | 61 |
| 679 | 15.16 | tr|Q6FGS1|Q6FGS1_HUMAN | TPD52L2 protein OS=Homo sapiens GN=TPD52L2 PE=1 SV=1 | 22237.5 | 206 | 63.59 | 9 | 8 | 67 | 64 |
| 680 | 15.13 | sp|Q9Y2W1|TR150_HUMAN | Thyroid hormone receptor-associated protein 3 OS=Homo sapiens GN=THRAP3 PE=1 SV=2 | 108665 | 955 | 9.843 | 8 | 8 | 45 | 43 |
| 681 | 15.13 | tr|A0A087WXR2|A0A087WXR2_HUMAN | PCTP-like protein OS=Homo sapiens GN=STARD10 PE=1 SV=1 | 40516.9 | 359 | 21.73 | 8 | 8 | 36 | 36 |
| 682 | 15.1 | tr|A0A024RA52|A0A024RA52_HUMAN | Proteasome subunit alpha type OS=Homo sapiens GN=PSMA2 PE=1 SV=1 | 25898.4 | 234 | 43.59 | 9 | 9 | 70 | 70 |
| 683 | 15.08 | tr|A0A140VKA6|A0A140VKA6_HUMAN | Testis secretory sperm-binding protein Li 233m OS=Homo sapiens PE=2 SV=1 | 41331.4 | 369 | 18.43 | 8 | 8 | 62 | 62 |
| 684 | 15.04 | sp|O60925|PFD1_HUMAN | Prefoldin subunit 1 OS=Homo sapiens GN=PFDN1 PE=1 SV=2 | 14210.4 | 122 | 63.11 | 8 | 8 | 31 | 31 |
| 685 | 15.03 | tr|B4DZC3|B4DZC3_HUMAN | cDNA FLJ55645, highly similar to 5'-3' exoribonuclease 2 (EC 3.1.11.-) OS=Homo sapiens PE=2 SV=1 | 102415.5 | 896 | 10.49 | 8 | 8 | 27 | 27 |
| 686 | 15.01 | tr|C9J2Y9|C9J2Y9_HUMAN | DNA-directed RNA polymerase subunit beta OS=Homo sapiens GN=POLR2B PE=1 SV=2 | 133055.6 | 1167 | 10.28 | 10 | 9 | 20 | 19 |
| 687 | 15.01 | tr|B4DRA5|B4DRA5_HUMAN | cDNA FLJ61346, highly similar to Protein transport protein Sec23B OS=Homo sapiens PE=2 SV=1 | 83809.4 | 742 | 13.07 | 7 | 5 | 33 | 17 |
| 688 | 15.01 | sp|Q9UJZ1|STML2_HUMAN | Stomatin-like protein 2, mitochondrial OS=Homo sapiens GN=STOML2 PE=1 SV=1 | 38533.6 | 356 | 35.67 | 9 | 8 | 51 | 50 |
| 689 | 15 | sp|Q9BW04|SARG_HUMAN | Specifically androgen-regulated gene protein OS=Homo sapiens GN=SARG PE=1 SV=2 | 63963.6 | 601 | 27.29 | 9 | 9 | 27 | 27 |
| 690 | 14.98 | tr|A1A4E9|A1A4E9_HUMAN | Keratin 13 OS=Homo sapiens GN=KRT13 PE=2 SV=1 | 49585.9 | 458 | 30.13 | 14 | 8 | 100 | 18 |
| 691 | 14.98 | tr|E9PB61|E9PB61_HUMAN | THO complex subunit 4 OS=Homo sapiens GN=ALYREF PE=1 SV=1 | 27557.5 | 264 | 40.15 | 9 | 9 | 127 | 123 |
| 692 | 14.98 | sp|Q9BRP8|PYM1_HUMAN | Partner of Y14 and mago OS=Homo sapiens GN=PYM1 PE=1 SV=1 | 22655.4 | 204 | 58.82 | 8 | 8 | 24 | 24 |
| 693 | 14.91 | tr|X5D2T3|X5D2T3_HUMAN | Ribosomal protein L10 isoform A (Fragment) OS=Homo sapiens GN=RPL10 PE=2 SV=1 | 24576.7 | 214 | 39.25 | 8 | 8 | 66 | 66 |
| 694 | 14.89 | tr|A0A0K0K1K4|A0A0K0K1K4_HUMAN | Proteasome subunit alpha type OS=Homo sapiens GN=HEL-S-276 PE=2 SV=1 | 27886.6 | 248 | 31.85 | 7 | 7 | 47 | 47 |
| 695 | 14.88 | sp|Q96HS1|PGAM5_HUMAN | Serine/threonine-protein phosphatase PGAM5, mitochondrial OS=Homo sapiens GN=PGAM5 PE=1 SV=2 | 32004.1 | 289 | 31.14 | 8 | 8 | 53 | 51 |
| 696 | 14.88 | sp|Q15942|ZYX_HUMAN | Zyxin OS=Homo sapiens GN=ZYX PE=1 SV=1 | 61276.9 | 572 | 25.35 | 9 | 9 | 32 | 32 |
| 697 | 14.83 | tr|A0A024RBR4|A0A024RBR4_HUMAN | Huntingtin interacting protein 1 related, isoform CRA_a OS=Homo sapiens GN=HIP1R PE=4 SV=1 | 99089.1 | 890 | 11.12 | 9 | 8 | 11 | 9 |
| 698 | 14.82 | sp|Q9NTZ6|RBM12_HUMAN | RNA-binding protein 12 OS=Homo sapiens GN=RBM12 PE=1 SV=1 | 97394.9 | 932 | 12.34 | 9 | 9 | 22 | 22 |
| 699 | 14.81 | tr|A0A140VJS3|A0A140VJS3_HUMAN | Testicular tissue protein Li 149 OS=Homo sapiens PE=2 SV=1 | 45625.7 | 406 | 33 | 11 | 10 | 47 | 31 |
| 700 | 14.8 | tr|D3DND1|D3DND1_HUMAN | CDV3 homolog (Mouse), isoform CRA_a OS=Homo sapiens GN=CDV3 PE=4 SV=1 | 28598.6 | 258 | 36.05 | 7 | 7 | 35 | 35 |
| 701 | 14.8 | tr|D3DQ70|D3DQ70_HUMAN | SERPINE1 mRNA binding protein 1, isoform CRA_d OS=Homo sapiens GN=SERBP1 PE=4 SV=1 | 50920.1 | 465 | 18.92 | 7 | 7 | 69 | 69 |
| 702 | 14.78 | sp|O60218|AK1BA_HUMAN | Aldo-keto reductase family 1 member B10 OS=Homo sapiens GN=AKR1B10 PE=1 SV=2 | 36019.3 | 316 | 28.48 | 8 | 8 | 33 | 33 |
| 703 | 14.77 | tr|E5KND5|E5KND5_HUMAN | Elongation factor G, mitochondrial OS=Homo sapiens GN=GFM1 PE=2 SV=1 | 83470.9 | 751 | 10.52 | 7 | 7 | 37 | 36 |
| 704 | 14.74 | tr|Q05CP8|Q05CP8_HUMAN | CCDC6 protein (Fragment) OS=Homo sapiens GN=CCDC6 PE=2 SV=1 | 38163.3 | 334 | 32.93 | 9 | 9 | 28 | 28 |
| 705 | 14.74 | sp|P12931|SRC_HUMAN | Proto-oncogene tyrosine-protein kinase Src OS=Homo sapiens GN=SRC PE=1 SV=3 | 59834.3 | 536 | 15.86 | 8 | 5 | 38 | 23 |
| 706 | 14.74 | sp|Q9UII2|ATIF1_HUMAN | ATPase inhibitor, mitochondrial OS=Homo sapiens GN=ATPIF1 PE=1 SV=1 | 12248.8 | 106 | 41.51 | 7 | 7 | 30 | 30 |
| 707 | 14.71 | tr|B4DWH7|B4DWH7_HUMAN | cDNA FLJ57632, highly similar to Vacuolar ATP synthase subunit B, kidney isoform (EC 3.6.3.14) OS=Homo sapiens PE=2 SV=1 | 54293.7 | 488 | 19.88 | 7 | 4 | 46 | 20 |
| 708 | 14.7 | sp|P26368|U2AF2_HUMAN | Splicing factor U2AF 65 kDa subunit OS=Homo sapiens GN=U2AF2 PE=1 SV=4 | 53500.6 | 475 | 28.63 | 9 | 9 | 35 | 35 |
| 709 | 14.69 | sp|P23193|TCEA1_HUMAN | Transcription elongation factor A protein 1 OS=Homo sapiens GN=TCEA1 PE=1 SV=2 | 33969.3 | 301 | 27.57 | 8 | 8 | 33 | 33 |
| 710 | 14.67 | tr|A0A024R1K7|A0A024R1K7_HUMAN | Tyrosine 3-monooxygenase/tryptophan 5-monooxygenase activation protein, eta polypeptide, isoform CRA_b OS=Homo sapiens GN=YWHAH PE=3 SV=1 | 28218.4 | 246 | 47.15 | 15 | 8 | 220 | 51 |
| 711 | 14.67 | tr|Q53H88|Q53H88_HUMAN | Dynactin 2 variant (Fragment) OS=Homo sapiens PE=2 SV=1 | 44820.3 | 406 | 25.37 | 8 | 8 | 18 | 17 |
| 712 | 14.65 | tr|B1AKK2|B1AKK2_HUMAN | Dimethylarginine dimethylaminohydrolase 1, isoform CRA_b OS=Homo sapiens GN=DDAH1 PE=2 SV=1 | 31121.5 | 285 | 36.14 | 7 | 7 | 60 | 60 |
| 713 | 14.65 | tr|B2R6A3|B2R6A3_HUMAN | Na(+)/H(+) exchange regulatory cofactor NHE-RF OS=Homo sapiens PE=2 SV=1 | 38898.1 | 358 | 27.65 | 7 | 7 | 43 | 43 |
| 714 | 14.62 | sp|Q9NYL9|TMOD3_HUMAN | Tropomodulin-3 OS=Homo sapiens GN=TMOD3 PE=1 SV=1 | 39594.4 | 352 | 27.27 | 8 | 8 | 49 | 49 |
| 715 | 14.58 | tr|Q6NZX3|Q6NZX3_HUMAN | 5'-nucleotidase, ecto (CD73) OS=Homo sapiens GN=NT5E PE=2 SV=1 | 63307.1 | 574 | 17.94 | 7 | 7 | 9 | 9 |
| 716 | 14.52 | tr|A0A024R663|A0A024R663_HUMAN | Kinectin 1 (Kinesin receptor), isoform CRA_a OS=Homo sapiens GN=KTN1 PE=4 SV=1 | 156274.5 | 1357 | 9.948 | 11 | 10 | 21 | 19 |
| 717 | 14.51 | tr|E7EX17|E7EX17_HUMAN | Eukaryotic translation initiation factor 4B OS=Homo sapiens GN=EIF4B PE=1 SV=1 | 69697 | 616 | 18.51 | 9 | 8 | 38 | 37 |
| 718 | 14.48 | tr|Q6IB71|Q6IB71_HUMAN | Proteasome subunit alpha type OS=Homo sapiens GN=PSMA3 PE=2 SV=1 | 28414.9 | 255 | 34.51 | 8 | 8 | 71 | 70 |
| 719 | 14.47 | tr|Q6NVW7|Q6NVW7_HUMAN | Importin subunit alpha OS=Homo sapiens GN=KPNA2 PE=2 SV=1 | 57964.5 | 529 | 17.58 | 7 | 7 | 57 | 57 |
| 720 | 14.45 | tr|A8K2M0|A8K2M0_HUMAN | Proteasome (Prosome, macropain) 26S subunit, ATPase, 4, isoform CRA_b OS=Homo sapiens GN=PSMC4 PE=2 SV=1 | 47365.8 | 418 | 22.25 | 11 | 10 | 33 | 29 |
| 721 | 14.42 | sp|Q14974|IMB1_HUMAN | Importin subunit beta-1 OS=Homo sapiens GN=KPNB1 PE=1 SV=2 | 97169.2 | 876 | 14.61 | 11 | 11 | 58 | 57 |
| 722 | 14.38 | tr|Q6IBR2|Q6IBR2_HUMAN | FARSLA protein OS=Homo sapiens GN=FARSLA PE=2 SV=1 | 57563.2 | 508 | 19.69 | 8 | 8 | 70 | 70 |
| 723 | 14.38 | sp|P10606|COX5B_HUMAN | Cytochrome c oxidase subunit 5B, mitochondrial OS=Homo sapiens GN=COX5B PE=1 SV=2 | 13695.6 | 129 | 46.51 | 8 | 8 | 66 | 66 |
| 724 | 14.35 | sp|P68366|TBA4A_HUMAN | Tubulin alpha-4A chain OS=Homo sapiens GN=TUBA4A PE=1 SV=1 | 49924 | 448 | 50.67 | 22 | 7 | 659 | 128 |
| 725 | 14.34 | tr|Q59G24|Q59G24_HUMAN | Activated RNA polymerase II transcription cofactor 4 variant (Fragment) OS=Homo sapiens PE=2 SV=1 | 15135 | 134 | 38.81 | 8 | 8 | 58 | 58 |
| 726 | 14.3 | tr|Q53G61|Q53G61_HUMAN | Small nuclear ribonucleoprotein polypeptide A' variant (Fragment) OS=Homo sapiens PE=2 SV=1 | 28401.2 | 255 | 37.25 | 10 | 9 | 20 | 16 |
| 727 | 14.3 | tr|Q6IAX5|Q6IAX5_HUMAN | Eukaryotic translation initiation factor 3 subunit E OS=Homo sapiens GN=EIF3E PE=2 SV=1 | 52205.4 | 445 | 20.67 | 7 | 7 | 59 | 59 |
| 728 | 14.29 | sp|O75947|ATP5H_HUMAN | ATP synthase subunit d, mitochondrial OS=Homo sapiens GN=ATP5H PE=1 SV=3 | 18491 | 161 | 55.28 | 8 | 8 | 37 | 36 |
| 729 | 14.28 | tr|Q6IQ30|Q6IQ30_HUMAN | Polyadenylate-binding protein OS=Homo sapiens GN=PABPC4 PE=2 SV=1 | 72360.1 | 660 | 24.09 | 16 | 7 | 141 | 39 |
| 730 | 14.28 | tr|A8K7T4|A8K7T4_HUMAN | cDNA FLJ75774, highly similar to Homo sapiens lectin, mannose-binding 2 (LMAN2), mRNA OS=Homo sapiens PE=2 SV=1 | 40242.4 | 356 | 28.93 | 8 | 8 | 30 | 30 |
| 731 | 14.27 | sp|Q13126|MTAP_HUMAN | S-methyl-5'-thioadenosine phosphorylase OS=Homo sapiens GN=MTAP PE=1 SV=2 | 31235.8 | 283 | 32.86 | 8 | 8 | 49 | 49 |
| 732 | 14.27 | sp|Q9UJS0|CMC2_HUMAN | Calcium-binding mitochondrial carrier protein Aralar2 OS=Homo sapiens GN=SLC25A13 PE=1 SV=2 | 74175 | 675 | 15.41 | 7 | 6 | 35 | 30 |
| 733 | 14.26 | tr|Q5T9B7|Q5T9B7_HUMAN | Adenylate kinase isoenzyme 1 OS=Homo sapiens GN=AK1 PE=1 SV=1 | 23410.6 | 210 | 27.14 | 7 | 7 | 30 | 30 |
| 734 | 14.25 | tr|A8KA82|A8KA82_HUMAN | DnaJ (Hsp40) homolog, subfamily C, member 3 OS=Homo sapiens GN=DNAJC3 PE=2 SV=1 | 57579.6 | 504 | 15.67 | 9 | 9 | 25 | 23 |
| 735 | 14.25 | sp|P56192|SYMC_HUMAN | Methionine--tRNA ligase, cytoplasmic OS=Homo sapiens GN=MARS PE=1 SV=2 | 101114.9 | 900 | 10.89 | 8 | 8 | 22 | 22 |
| 736 | 14.25 | tr|A0A0C4DGB5|A0A0C4DGB5_HUMAN | Calpastatin OS=Homo sapiens GN=CAST PE=1 SV=1 | 80998.5 | 754 | 13.53 | 8 | 8 | 26 | 26 |
| 737 | 14.23 | tr|B2RBM7|B2RBM7_HUMAN | cDNA, FLJ95595, highly similar to Homo sapiens proteasome (prosome, macropain) 26S subunit, non-ATPase, 13 (PSMD13), mRNA OS=Homo sapiens PE=2 SV=1 | 42873.1 | 376 | 21.01 | 7 | 7 | 29 | 25 |
| 738 | 14.19 | sp|O14737|PDCD5_HUMAN | Programmed cell death protein 5 OS=Homo sapiens GN=PDCD5 PE=1 SV=3 | 14285 | 125 | 55.2 | 8 | 8 | 61 | 61 |
| 739 | 14.17 | tr|B3KY11|B3KY11_HUMAN | cDNA FLJ46571 fis, clone THYMU3041428, highly similar to Probable ATP-dependent RNA helicase DDX23 (EC 3.6.1.-) OS=Homo sapiens PE=2 SV=1 | 93233.1 | 800 | 9.875 | 8 | 8 | 15 | 14 |
| 740 | 14.14 | sp|A0MZ66|SHOT1_HUMAN | Shootin-1 OS=Homo sapiens GN=SHTN1 PE=1 SV=4 | 71639.3 | 631 | 15.53 | 8 | 8 | 20 | 20 |
| 741 | 14.13 | sp|Q9NZM1|MYOF_HUMAN | Myoferlin OS=Homo sapiens GN=MYOF PE=1 SV=1 | 234706.8 | 2061 | 6.453 | 11 | 9 | 16 | 10 |
| 742 | 14.1 | tr|B7Z3E7|B7Z3E7_HUMAN | cDNA FLJ56510, highly similar to Tumor suppressor p53-binding protein 1 OS=Homo sapiens PE=2 SV=1 | 189917.3 | 1760 | 7.614 | 9 | 9 | 21 | 21 |
| 743 | 14.03 | tr|A0A024R0P9|A0A024R0P9_HUMAN | Translocase of outer mitochondrial membrane 40 homolog (Yeast), isoform CRA_c OS=Homo sapiens GN=TOMM40 PE=4 SV=1 | 37892.9 | 361 | 28.25 | 7 | 7 | 45 | 45 |
| 744 | 14.02 | tr|A0A024R4U3|A0A024R4U3_HUMAN | Tubulin tyrosine ligase-like family, member 12, isoform CRA_a OS=Homo sapiens GN=TTLL12 PE=4 SV=1 | 74403 | 644 | 16.15 | 8 | 8 | 22 | 22 |
| 745 | 14.02 | tr|X5DNI9|X5DNI9_HUMAN | 7-dehydrocholesterol reductase isoform A (Fragment) OS=Homo sapiens GN=DHCR7 PE=2 SV=1 | 54460.9 | 475 | 12.84 | 7 | 7 | 27 | 27 |
| 746 | 14.01 | tr|Q5TB52|Q5TB52_HUMAN | 3'-phosphoadenosine 5'-phosphosulfate synthase 2, isoform CRA_b OS=Homo sapiens GN=PAPSS2 PE=2 SV=1 | 69500.2 | 614 | 15.96 | 7 | 6 | 23 | 21 |
| 747 | 14 | sp|Q96S55|WRIP1_HUMAN | ATPase WRNIP1 OS=Homo sapiens GN=WRNIP1 PE=1 SV=2 | 72132.2 | 665 | 15.19 | 9 | 9 | 25 | 25 |
| 748 | 14 | tr|Q6FHM6|Q6FHM6_HUMAN | NHP2 non-histone chromosome protein 2-like 1 (S. cerevisiae) OS=Homo sapiens GN=NHP2L1 PE=2 SV=1 | 14173.4 | 128 | 39.84 | 7 | 7 | 41 | 40 |
| 749 | 13.97 | tr|F5H7S7|F5H7S7_HUMAN | Ras GTPase-activating-like protein IQGAP2 OS=Homo sapiens GN=IQGAP2 PE=1 SV=2 | 174447.5 | 1525 | 9.508 | 13 | 5 | 32 | 7 |
| 750 | 13.93 | tr|J3KS22|J3KS22_HUMAN | L-xylulose reductase (Fragment) OS=Homo sapiens GN=DCXR PE=1 SV=7 | 23959.7 | 224 | 33.04 | 8 | 7 | 18 | 17 |
| 751 | 13.9 | sp|O60869|EDF1_HUMAN | Endothelial differentiation-related factor 1 OS=Homo sapiens GN=EDF1 PE=1 SV=1 | 16368.6 | 148 | 39.19 | 7 | 7 | 34 | 34 |
| 752 | 13.86 | tr|I3L0N3|I3L0N3_HUMAN | Vesicle-fusing ATPase OS=Homo sapiens GN=NSF PE=1 SV=1 | 82091.1 | 739 | 10.55 | 7 | 7 | 46 | 46 |
| 753 | 13.84 | tr|A4D2P1|A4D2P1_HUMAN | Ras-related C3 botulinum toxin substrate 1 (Rho family, small GTP binding protein Rac1) OS=Homo sapiens GN=RAC1 PE=2 SV=1 | 21449.9 | 192 | 27.6 | 7 | 6 | 54 | 51 |
| 754 | 13.83 | tr|A0A024R0Q7|A0A024R0Q7_HUMAN | Serine/threonine-protein phosphatase OS=Homo sapiens GN=PPP5C PE=3 SV=1 | 56878.2 | 499 | 21.44 | 8 | 8 | 37 | 37 |
| 755 | 13.81 | sp|P27487|DPP4_HUMAN | Dipeptidyl peptidase 4 OS=Homo sapiens GN=DPP4 PE=1 SV=2 | 88277.9 | 766 | 11.88 | 8 | 8 | 47 | 47 |
| 756 | 13.8 | sp|Q9BS26|ERP44_HUMAN | Endoplasmic reticulum resident protein 44 OS=Homo sapiens GN=ERP44 PE=1 SV=1 | 46970.7 | 406 | 16.75 | 8 | 8 | 44 | 29 |
| 757 | 13.79 | sp|Q9P0L0|VAPA_HUMAN | Vesicle-associated membrane protein-associated protein A OS=Homo sapiens GN=VAPA PE=1 SV=3 | 27893 | 249 | 38.55 | 10 | 9 | 59 | 49 |
| 758 | 13.79 | sp|P54727|RD23B_HUMAN | UV excision repair protein RAD23 homolog B OS=Homo sapiens GN=RAD23B PE=1 SV=1 | 43170.9 | 409 | 14.43 | 8 | 8 | 25 | 25 |
| 759 | 13.77 | sp|P35611|ADDA_HUMAN | Alpha-adducin OS=Homo sapiens GN=ADD1 PE=1 SV=2 | 80954.5 | 737 | 17.37 | 10 | 10 | 34 | 34 |
| 760 | 13.77 | tr|A0A0A0MRW6|A0A0A0MRW6_HUMAN | Nucleolar protein 6 OS=Homo sapiens GN=NOL6 PE=1 SV=1 | 112156.2 | 1008 | 13.49 | 10 | 9 | 15 | 14 |
| 761 | 13.76 | sp|P27348|1433T_HUMAN | 14-3-3 protein theta OS=Homo sapiens GN=YWHAQ PE=1 SV=1 | 27763.9 | 245 | 45.31 | 13 | 8 | 244 | 94 |
| 762 | 13.73 | sp|P07741|APT_HUMAN | Adenine phosphoribosyltransferase OS=Homo sapiens GN=APRT PE=1 SV=2 | 19607.5 | 180 | 45.56 | 7 | 7 | 33 | 33 |
| 763 | 13.62 | sp|Q8IX12|CCAR1_HUMAN | Cell division cycle and apoptosis regulator protein 1 OS=Homo sapiens GN=CCAR1 PE=1 SV=2 | 132820 | 1150 | 8.087 | 9 | 8 | 16 | 13 |
| 764 | 13.61 | sp|P12081|SYHC_HUMAN | Histidine--tRNA ligase, cytoplasmic OS=Homo sapiens GN=HARS PE=1 SV=2 | 57410 | 509 | 13.16 | 7 | 3 | 41 | 28 |
| 765 | 13.6 | tr|A0A0S2Z3X8|A0A0S2Z3X8_HUMAN | GDP dissociation inhibitor 1 isoform 1 (Fragment) OS=Homo sapiens GN=GDI1 PE=2 SV=1 | 50582.3 | 447 | 34.23 | 14 | 8 | 76 | 45 |
| 766 | 13.58 | tr|A0A024R4A5|A0A024R4A5_HUMAN | Trinucleotide repeat containing 15, isoform CRA_a OS=Homo sapiens GN=TNRC15 PE=4 SV=1 | 149941.2 | 1298 | 9.091 | 9 | 8 | 18 | 17 |
| 767 | 13.54 | tr|B2RDF5|B2RDF5_HUMAN | cDNA, FLJ96587, highly similar to Homo sapiens SUMO-1 activating enzyme subunit 2 (UBA2), mRNA OS=Homo sapiens PE=2 SV=1 | 71178.9 | 640 | 15.16 | 8 | 8 | 53 | 51 |
| 768 | 13.51 | tr|A0A024R3P9|A0A024R3P9_HUMAN | Acyl-Coenzyme A binding domain containing 3, isoform CRA_a OS=Homo sapiens GN=ACBD3 PE=4 SV=1 | 60593 | 528 | 21.4 | 7 | 7 | 29 | 29 |
| 769 | 13.5 | sp|Q96G03|PGM2_HUMAN | Phosphoglucomutase-2 OS=Homo sapiens GN=PGM2 PE=1 SV=4 | 68282.8 | 612 | 11.6 | 7 | 6 | 12 | 11 |
| 770 | 13.47 | tr|Q0P5N8|Q0P5N8_HUMAN | TMSB4X protein (Fragment) OS=Homo sapiens GN=TMSB4X PE=2 SV=1 | 7768.6 | 70 | 57.14 | 8 | 7 | 98 | 97 |
| 771 | 13.46 | tr|A0A024R9G4|A0A024R9G4_HUMAN | Family with sequence similarity 49, member B, isoform CRA_a OS=Homo sapiens GN=FAM49B PE=4 SV=1 | 36747.7 | 324 | 26.54 | 8 | 8 | 48 | 48 |
| 772 | 13.46 | tr|A0A024R4E2|A0A024R4E2_HUMAN | TAR DNA binding protein, isoform CRA_b OS=Homo sapiens GN=TARDBP PE=4 SV=1 | 44739.6 | 414 | 15.7 | 7 | 7 | 21 | 21 |
| 773 | 13.44 | tr|Q49AG4|Q49AG4_HUMAN | DIS3 protein OS=Homo sapiens GN=DIS3 PE=2 SV=1 | 90598.1 | 796 | 11.43 | 8 | 7 | 21 | 20 |
| 774 | 13.43 | sp|Q9Y5X3|SNX5_HUMAN | Sorting nexin-5 OS=Homo sapiens GN=SNX5 PE=1 SV=1 | 46816.1 | 404 | 22.03 | 7 | 7 | 25 | 25 |
| 775 | 13.36 | tr|A0A087WZN1|A0A087WZN1_HUMAN | Isocitrate dehydrogenase [NAD] subunit, mitochondrial OS=Homo sapiens GN=IDH3B PE=1 SV=1 | 42410.7 | 387 | 18.86 | 9 | 8 | 31 | 30 |
| 776 | 13.35 | tr|A4D0V4|A4D0V4_HUMAN | Capping protein (Actin filament) muscle Z-line, alpha 2 OS=Homo sapiens GN=CAPZA2 PE=2 SV=1 | 32948.9 | 286 | 39.51 | 9 | 7 | 73 | 44 |
| 777 | 13.31 | tr|H0YHX9|H0YHX9_HUMAN | Nascent polypeptide-associated complex subunit alpha (Fragment) OS=Homo sapiens GN=NACA PE=1 SV=1 | 22943.3 | 213 | 36.15 | 8 | 8 | 66 | 66 |
| 778 | 13.29 | sp|Q8TCJ2|STT3B_HUMAN | Dolichyl-diphosphooligosaccharide--protein glycosyltransferase subunit STT3B OS=Homo sapiens GN=STT3B PE=1 SV=1 | 93673.5 | 826 | 7.385 | 7 | 6 | 16 | 15 |
| 779 | 13.28 | tr|A0A087WY71|A0A087WY71_HUMAN | AP-2 complex subunit mu OS=Homo sapiens GN=AP2M1 PE=1 SV=1 | 49526.2 | 434 | 15.67 | 7 | 7 | 12 | 12 |
| 780 | 13.26 | tr|Q4ZG57|Q4ZG57_HUMAN | DNA helicase (Fragment) OS=Homo sapiens GN=MCM6 PE=3 SV=1 | 88945 | 785 | 13.12 | 10 | 10 | 50 | 50 |
| 781 | 13.24 | tr|A0A024RAJ6|A0A024RAJ6_HUMAN | Beta-hexosaminidase OS=Homo sapiens GN=HEXB PE=3 SV=1 | 63110.7 | 556 | 14.57 | 7 | 7 | 20 | 20 |
| 782 | 13.24 | sp|O75400|PR40A_HUMAN | Pre-mRNA-processing factor 40 homolog A OS=Homo sapiens GN=PRPF40A PE=1 SV=2 | 108804.2 | 957 | 9.091 | 7 | 7 | 18 | 18 |
| 783 | 13.22 | sp|Q13404|UB2V1_HUMAN | Ubiquitin-conjugating enzyme E2 variant 1 OS=Homo sapiens GN=UBE2V1 PE=1 SV=2 | 16494.9 | 147 | 47.62 | 8 | 6 | 78 | 44 |
| 784 | 13.21 | tr|Q6FGU2|Q6FGU2_HUMAN | DTYMK protein (Fragment) OS=Homo sapiens GN=DTYMK PE=2 SV=1 | 23819.1 | 212 | 23.58 | 6 | 6 | 30 | 30 |
| 785 | 13.19 | sp|P18283|GPX2_HUMAN | Glutathione peroxidase 2 OS=Homo sapiens GN=GPX2 PE=1 SV=3 | 21953.8 | 190 | 32.11 | 6 | 6 | 39 | 39 |
| 786 | 13.18 | tr|E9PQ61|E9PQ61_HUMAN | Zinc finger CCCH domain-containing protein 11A OS=Homo sapiens GN=ZC3H11A PE=1 SV=1 | 72531.7 | 652 | 16.41 | 7 | 7 | 13 | 13 |
| 787 | 13.18 | sp|Q5JTV8|TOIP1_HUMAN | Torsin-1A-interacting protein 1 OS=Homo sapiens GN=TOR1AIP1 PE=1 SV=2 | 66247.9 | 583 | 13.72 | 7 | 7 | 31 | 31 |
| 788 | 13.18 | tr|Q53FT8|Q53FT8_HUMAN | Proteasome subunit beta type (Fragment) OS=Homo sapiens PE=2 SV=1 | 26491.1 | 241 | 40.25 | 7 | 7 | 26 | 26 |
| 789 | 13.15 | sp|P08397|HEM3_HUMAN | Porphobilinogen deaminase OS=Homo sapiens GN=HMBS PE=1 SV=2 | 39329.7 | 361 | 28.53 | 7 | 7 | 14 | 14 |
| 790 | 13.14 | tr|A0A0S2Z489|A0A0S2Z489_HUMAN | Proteasome (Prosome, macropain) 26S subunit, non-ATPase, 12, isoform CRA_a (Fragment) OS=Homo sapiens GN=PSMD12 PE=2 SV=1 | 52903.9 | 456 | 20.83 | 7 | 7 | 16 | 16 |
| 791 | 13.13 | tr|G3V3A4|G3V3A4_HUMAN | SNW domain-containing protein 1 OS=Homo sapiens GN=SNW1 PE=1 SV=1 | 65390.8 | 571 | 22.07 | 9 | 9 | 17 | 17 |
| 792 | 13.12 | tr|C9JAB2|C9JAB2_HUMAN | Serine/arginine-rich-splicing factor 7 OS=Homo sapiens GN=SRSF7 PE=1 SV=1 | 26927.7 | 235 | 24.68 | 7 | 6 | 54 | 51 |
| 793 | 13.11 | tr|A6NGP5|A6NGP5_HUMAN | Hematological and neurological-expressed 1-like protein OS=Homo sapiens GN=HN1L PE=1 SV=2 | 18906 | 178 | 41.57 | 6 | 6 | 46 | 46 |
| 794 | 13.1 | tr|A0A024R277|A0A024R277_HUMAN | Serine palmitoyltransferase, long chain base subunit 1, isoform CRA_a OS=Homo sapiens GN=SPTLC1 PE=4 SV=1 | 52743.4 | 473 | 15.64 | 6 | 6 | 17 | 17 |
| 795 | 13.08 | tr|E5KS55|E5KS55_HUMAN | Succinate-CoA ligase subunit beta OS=Homo sapiens PE=3 SV=1 | 50330.9 | 463 | 14.9 | 7 | 7 | 36 | 36 |
| 796 | 13.07 | tr|B3KM90|B3KM90_HUMAN | cDNA FLJ10529 fis, clone NT2RP2000965, highly similar to Targeting protein for Xklp2 OS=Homo sapiens PE=2 SV=1 | 69757.9 | 605 | 19.83 | 10 | 9 | 20 | 14 |
| 797 | 13.07 | tr|B7Z959|B7Z959_HUMAN | cDNA FLJ54736, highly similar to Scaffold attachment factor B OS=Homo sapiens PE=2 SV=1 | 75755.7 | 666 | 11.71 | 8 | 7 | 17 | 16 |
| 798 | 13.04 | tr|E7ERY9|E7ERY9_HUMAN | Calcium-transporting ATPase OS=Homo sapiens GN=ATP2B1 PE=1 SV=2 | 106855.4 | 963 | 11.42 | 8 | 8 | 13 | 13 |
| 799 | 13.04 | sp|Q6YN16|HSDL2_HUMAN | Hydroxysteroid dehydrogenase-like protein 2 OS=Homo sapiens GN=HSDL2 PE=1 SV=1 | 45394.3 | 418 | 24.88 | 8 | 8 | 38 | 38 |
| 800 | 13.03 | sp|Q8WW12|PCNP_HUMAN | PEST proteolytic signal-containing nuclear protein OS=Homo sapiens GN=PCNP PE=1 SV=2 | 18924.7 | 178 | 46.07 | 7 | 7 | 39 | 39 |
| 801 | 13 | tr|R4SBI6|R4SBI6_HUMAN | EPHX1 OS=Homo sapiens GN=EPHX1 PE=2 SV=1 | 52948.5 | 455 | 14.95 | 7 | 7 | 69 | 68 |
| 802 | 12.96 | tr|X5D2F4|X5D2F4_HUMAN | Cytoplasmic FMR1 interacting protein 1 isoform A (Fragment) OS=Homo sapiens GN=CYFIP1 PE=2 SV=1 | 145167.2 | 1253 | 6.864 | 8 | 8 | 46 | 46 |
| 803 | 12.94 | tr|Q59E88|Q59E88_HUMAN | DnaJ (Hsp40) homolog, subfamily A, member 3 variant (Fragment) OS=Homo sapiens PE=2 SV=1 | 52286 | 478 | 18.2 | 7 | 7 | 37 | 37 |
| 804 | 12.92 | sp|Q86XP3|DDX42_HUMAN | ATP-dependent RNA helicase DDX42 OS=Homo sapiens GN=DDX42 PE=1 SV=1 | 102974.5 | 938 | 13.33 | 10 | 10 | 29 | 29 |
| 805 | 12.9 | sp|P50851|LRBA_HUMAN | Lipopolysaccharide-responsive and beige-like anchor protein OS=Homo sapiens GN=LRBA PE=1 SV=4 | 319105 | 2863 | 3.633 | 8 | 2 | 14 | 4 |
| 806 | 12.88 | tr|A8K5J1|A8K5J1_HUMAN | Uridine monophosphate synthetase (Orotate phosphoribosyl transferase and orotidine-5'-decarboxylase), isoform CRA_b OS=Homo sapiens GN=UMPS PE=2 SV=1 | 52221.1 | 480 | 17.08 | 8 | 8 | 35 | 35 |
| 807 | 12.83 | tr|D6RGI3|D6RGI3_HUMAN | Septin 11, isoform CRA_b OS=Homo sapiens GN=SEPT11 PE=1 SV=1 | 49005.6 | 425 | 21.41 | 7 | 4 | 31 | 17 |
| 808 | 12.82 | tr|F6WQW2|F6WQW2_HUMAN | Ran-specific GTPase-activating protein OS=Homo sapiens GN=RANBP1 PE=1 SV=1 | 31904 | 278 | 28.06 | 7 | 7 | 93 | 93 |
| 809 | 12.81 | tr|A0A024R1V4|A0A024R1V4_HUMAN | 60S ribosomal protein L27 OS=Homo sapiens GN=RPL27 PE=3 SV=1 | 15797.6 | 136 | 48.53 | 7 | 7 | 32 | 32 |
| 810 | 12.79 | tr|A0A024R0L6|A0A024R0L6_HUMAN | Platelet-activating factor acetylhydrolase, isoform Ib, gamma subunit 29kDa, isoform CRA_a OS=Homo sapiens GN=PAFAH1B3 PE=4 SV=1 | 25734.1 | 231 | 43.72 | 7 | 7 | 27 | 27 |
| 811 | 12.74 | tr|R4RWV3|R4RWV3_HUMAN | MHC class I antigen (Fragment) OS=Homo sapiens GN=HLA-C PE=3 SV=1 | 31465.5 | 273 | 32.97 | 6 | 3 | 64 | 9 |
| 812 | 12.69 | tr|Q6FI03|Q6FI03_HUMAN | G3BP protein OS=Homo sapiens GN=G3BP PE=2 SV=1 | 52164.1 | 466 | 20.17 | 7 | 6 | 62 | 59 |
| 813 | 12.66 | tr|A0A024RDE8|A0A024RDE8_HUMAN | PDZ and LIM domain 5, isoform CRA_c OS=Homo sapiens GN=PDLIM5 PE=4 SV=1 | 63974.6 | 596 | 21.64 | 7 | 7 | 26 | 26 |
| 814 | 12.65 | sp|O75439|MPPB_HUMAN | Mitochondrial-processing peptidase subunit beta OS=Homo sapiens GN=PMPCB PE=1 SV=2 | 54365.6 | 489 | 19.02 | 8 | 7 | 32 | 31 |
| 815 | 12.65 | sp|Q8N766|EMC1_HUMAN | ER membrane protein complex subunit 1 OS=Homo sapiens GN=EMC1 PE=1 SV=1 | 111758.5 | 993 | 12.08 | 8 | 7 | 24 | 23 |
| 816 | 12.64 | tr|A0A024R3W7|A0A024R3W7_HUMAN | Eukaryotic translation elongation factor 1 beta 2, isoform CRA_a OS=Homo sapiens GN=EEF1B2 PE=3 SV=1 | 24763.5 | 225 | 37.78 | 9 | 7 | 120 | 117 |
| 817 | 12.64 | sp|P59998|ARPC4_HUMAN | Actin-related protein 2/3 complex subunit 4 OS=Homo sapiens GN=ARPC4 PE=1 SV=3 | 19666.8 | 168 | 25.6 | 6 | 6 | 67 | 67 |
| 818 | 12.63 | sp|Q13123|RED_HUMAN | Protein Red OS=Homo sapiens GN=IK PE=1 SV=3 | 65601.7 | 557 | 14 | 6 | 6 | 13 | 13 |
| 819 | 12.6 | tr|B4DKU3|B4DKU3_HUMAN | cDNA FLJ53449, highly similar to rRNA methyltransferase 3 (EC 2.1.1.-) OS=Homo sapiens PE=2 SV=1 | 82578.3 | 726 | 14.33 | 8 | 8 | 28 | 27 |
| 820 | 12.59 | tr|A0A024RDL8|A0A024RDL8_HUMAN | Argininosuccinate lyase isoform 1 OS=Homo sapiens GN=ASL PE=2 SV=1 | 51657.5 | 464 | 14.44 | 7 | 7 | 24 | 24 |
| 821 | 12.58 | sp|Q6P2E9|EDC4_HUMAN | Enhancer of mRNA-decapping protein 4 OS=Homo sapiens GN=EDC4 PE=1 SV=1 | 151659.6 | 1401 | 6.924 | 7 | 7 | 12 | 12 |
| 822 | 12.58 | tr|A0A024RDB0|A0A024RDB0_HUMAN | Ubiquitin-activating enzyme E1-like 2, isoform CRA_a OS=Homo sapiens GN=UBE1L2 PE=4 SV=1 | 117968.9 | 1052 | 6.369 | 7 | 7 | 26 | 26 |
| 823 | 12.56 | tr|B3KNJ4|B3KNJ4_HUMAN | SUMO-1 activating enzyme subunit 1, isoform CRA_a OS=Homo sapiens GN=SAE1 PE=1 SV=1 | 33384.1 | 299 | 28.09 | 7 | 7 | 22 | 22 |
| 824 | 12.56 | tr|V9HW62|V9HW62_HUMAN | Lactoylglutathione lyase OS=Homo sapiens GN=HEL-S-74 PE=2 SV=1 | 20719.5 | 184 | 39.13 | 7 | 7 | 18 | 17 |
| 825 | 12.54 | tr|A0A140VKC8|A0A140VKC8_HUMAN | Testis tissue sperm-binding protein Li 45a OS=Homo sapiens PE=2 SV=1 | 35892.5 | 321 | 19.94 | 6 | 6 | 36 | 35 |
| 826 | 12.54 | sp|Q9Y295|DRG1_HUMAN | Developmentally-regulated GTP-binding protein 1 OS=Homo sapiens GN=DRG1 PE=1 SV=1 | 40541.8 | 367 | 25.61 | 7 | 7 | 47 | 47 |
| 827 | 12.53 | tr|Q53EW8|Q53EW8_HUMAN | Sulfurtransferase (Fragment) OS=Homo sapiens PE=2 SV=1 | 33452.7 | 297 | 31.65 | 8 | 8 | 47 | 47 |
| 828 | 12.52 | tr|Q6IAX9|Q6IAX9_HUMAN | SRPR protein OS=Homo sapiens GN=SRPR PE=2 SV=1 | 69893.7 | 638 | 12.23 | 6 | 6 | 30 | 30 |
| 829 | 12.52 | tr|Q54A51|Q54A51_HUMAN | Basigin (Ok blood group), isoform CRA_a OS=Homo sapiens GN=hEMMPRIN PE=2 SV=1 | 29220.7 | 269 | 32.34 | 6 | 6 | 74 | 74 |
| 830 | 12.5 | sp|P55036|PSMD4_HUMAN | 26S proteasome non-ATPase regulatory subunit 4 OS=Homo sapiens GN=PSMD4 PE=1 SV=1 | 40736.3 | 377 | 25.73 | 7 | 7 | 27 | 26 |
| 831 | 12.5 | sp|Q5JTZ9|SYAM_HUMAN | Alanine--tRNA ligase, mitochondrial OS=Homo sapiens GN=AARS2 PE=1 SV=1 | 107339.5 | 985 | 9.746 | 8 | 8 | 18 | 17 |
| 832 | 12.48 | sp|Q12849|GRSF1_HUMAN | G-rich sequence factor 1 OS=Homo sapiens GN=GRSF1 PE=1 SV=3 | 53125.8 | 480 | 20 | 7 | 7 | 23 | 23 |
| 833 | 12.46 | tr|Q5STK2|Q5STK2_HUMAN | Prefoldin subunit 6, isoform CRA_b OS=Homo sapiens GN=PFDN6 PE=2 SV=1 | 14582.6 | 129 | 62.79 | 7 | 7 | 36 | 35 |
| 834 | 12.45 | tr|A0A024RBV2|A0A024RBV2_HUMAN | Acetoacetyl-CoA synthetase, isoform CRA_b OS=Homo sapiens GN=AACS PE=4 SV=1 | 75143.6 | 672 | 11.61 | 8 | 8 | 13 | 13 |
| 835 | 12.44 | tr|E7EX90|E7EX90_HUMAN | Dynactin subunit 1 OS=Homo sapiens GN=DCTN1 PE=1 SV=1 | 139092.7 | 1256 | 7.245 | 7 | 7 | 17 | 16 |
| 836 | 12.44 | tr|Q5JPT6|Q5JPT6_HUMAN | GIG10 OS=Homo sapiens PE=1 SV=1 | 73125.6 | 665 | 13.53 | 7 | 7 | 12 | 12 |
| 837 | 12.42 | sp|P30048|PRDX3_HUMAN | Thioredoxin-dependent peroxide reductase, mitochondrial OS=Homo sapiens GN=PRDX3 PE=1 SV=3 | 27692.4 | 256 | 35.94 | 8 | 8 | 57 | 57 |
| 838 | 12.41 | tr|B4DEF8|B4DEF8_HUMAN | cDNA FLJ61100, highly similar to 39S ribosomal protein L45, mitochondrial OS=Homo sapiens PE=2 SV=1 | 37775.5 | 329 | 32.52 | 7 | 7 | 20 | 20 |
| 839 | 12.41 | tr|A0A024R394|A0A024R394_HUMAN | Cysteine and histidine-rich domain (CHORD)-containing 1, isoform CRA_c OS=Homo sapiens GN=CHORDC1 PE=4 SV=1 | 37533.5 | 332 | 27.11 | 6 | 6 | 16 | 16 |
| 840 | 12.39 | tr|B4E273|B4E273_HUMAN | ADP-ribosylation factor interacting protein 1 (Arfaptin 1), isoform CRA_b OS=Homo sapiens GN=ARFIP1 PE=2 SV=1 | 41737.9 | 373 | 20.91 | 7 | 7 | 47 | 47 |
| 841 | 12.39 | tr|Q6IAL5|Q6IAL5_HUMAN | Putative uncharacterized protein tmp_locus_1 OS=Homo sapiens GN=SUCLG1 PE=2 SV=1 | 35047.1 | 333 | 24.02 | 7 | 7 | 29 | 29 |
| 842 | 12.37 | sp|Q9NYK5|RM39_HUMAN | 39S ribosomal protein L39, mitochondrial OS=Homo sapiens GN=MRPL39 PE=1 SV=3 | 38711.4 | 338 | 27.51 | 8 | 8 | 22 | 22 |
| 843 | 12.37 | sp|P00167|CYB5_HUMAN | Cytochrome b5 OS=Homo sapiens GN=CYB5A PE=1 SV=2 | 15330 | 134 | 60.45 | 7 | 7 | 39 | 38 |
| 844 | 12.34 | tr|A0A087WUT6|A0A087WUT6_HUMAN | Eukaryotic translation initiation factor 5B OS=Homo sapiens GN=EIF5B PE=1 SV=1 | 138680.8 | 1220 | 8.525 | 7 | 7 | 37 | 37 |
| 845 | 12.32 | tr|A0A0S2Z487|A0A0S2Z487_HUMAN | Junction plakoglobin isoform 1 (Fragment) OS=Homo sapiens GN=JUP PE=2 SV=1 | 81744 | 745 | 28.46 | 19 | 7 | 130 | 29 |
| 846 | 12.32 | tr|A4FVC0|A4FVC0_HUMAN | EIF2C2 protein (Fragment) OS=Homo sapiens GN=EIF2C2 PE=2 SV=1 | 91496 | 808 | 11.14 | 7 | 7 | 20 | 20 |
| 847 | 12.31 | tr|A0A024R702|A0A024R702_HUMAN | Brain specific protein, isoform CRA_a OS=Homo sapiens GN=CGI-38 PE=4 SV=1 | 18985.3 | 176 | 35.8 | 6 | 6 | 46 | 46 |
| 848 | 12.3 | sp|Q13724|MOGS_HUMAN | Mannosyl-oligosaccharide glucosidase OS=Homo sapiens GN=MOGS PE=1 SV=5 | 91916.9 | 837 | 9.319 | 7 | 7 | 22 | 22 |
| 849 | 12.27 | sp|Q9NQW7|XPP1_HUMAN | Xaa-Pro aminopeptidase 1 OS=Homo sapiens GN=XPNPEP1 PE=1 SV=3 | 69917.2 | 623 | 15.25 | 9 | 9 | 27 | 27 |
| 850 | 12.27 | tr|C9JRZ6|C9JRZ6_HUMAN | MICOS complex subunit OS=Homo sapiens GN=CHCHD3 PE=1 SV=1 | 26693.8 | 232 | 34.05 | 8 | 8 | 32 | 30 |
| 851 | 12.24 | tr|M0QWZ7|M0QWZ7_HUMAN | Serine--tRNA ligase, mitochondrial OS=Homo sapiens GN=SARS2 PE=1 SV=1 | 58181.9 | 518 | 20.27 | 7 | 7 | 16 | 12 |
| 852 | 12.23 | sp|P61513|RL37A_HUMAN | 60S ribosomal protein L37a OS=Homo sapiens GN=RPL37A PE=1 SV=2 | 10275.2 | 92 | 58.7 | 6 | 6 | 52 | 52 |
| 853 | 12.22 | tr|V9HWG1|V9HWG1_HUMAN | Epididymis secretory sperm binding protein Li 134P OS=Homo sapiens GN=HEL-S-134P PE=2 SV=1 | 52390.6 | 472 | 23.94 | 7 | 7 | 35 | 35 |
| 854 | 12.21 | tr|Q53HB9|Q53HB9_HUMAN | DEAD (Asp-Glu-Ala-Asp) box polypeptide 56 variant (Fragment) OS=Homo sapiens PE=2 SV=1 | 61564.8 | 547 | 11.88 | 6 | 6 | 18 | 18 |
| 855 | 12.21 | tr|Q6FHN3|Q6FHN3_HUMAN | Nucleoside diphosphate kinase OS=Homo sapiens GN=NME2 PE=2 SV=1 | 17297.9 | 152 | 50 | 6 | 6 | 94 | 93 |
| 856 | 12.19 | sp|P48047|ATPO_HUMAN | ATP synthase subunit O, mitochondrial OS=Homo sapiens GN=ATP5O PE=1 SV=1 | 23277.1 | 213 | 29.58 | 6 | 6 | 55 | 55 |
| 857 | 12.19 | tr|B7Z700|B7Z700_HUMAN | cDNA FLJ54557, highly similar to helicase MOV-10 (EC 3.6.1.-) OS=Homo sapiens PE=2 SV=1 | 106518.1 | 941 | 8.183 | 7 | 7 | 14 | 14 |
| 858 | 12.19 | tr|B4E074|B4E074_HUMAN | cDNA FLJ58655, highly similar to Notchless homolog 1 OS=Homo sapiens PE=2 SV=1 | 50728.8 | 461 | 24.95 | 7 | 7 | 17 | 16 |
| 859 | 12.18 | tr|Q53HN4|Q53HN4_HUMAN | DNAation factor, 45kDa, alpha polypeptide isoform 1 variant (Fragment) OS=Homo sapiens PE=2 SV=1 | 36593.5 | 331 | 25.98 | 6 | 6 | 17 | 17 |
| 860 | 12.18 | sp|P18615|NELFE_HUMAN | Negative elongation factor E OS=Homo sapiens GN=NELFE PE=1 SV=3 | 43239.3 | 380 | 21.05 | 6 | 6 | 18 | 18 |
| 861 | 12.15 | tr|A0A024R172|A0A024R172_HUMAN | Leukotriene B4 12-hydroxydehydrogenase, isoform CRA_a OS=Homo sapiens GN=LTB4DH PE=4 SV=1 | 35885.6 | 329 | 24.62 | 6 | 6 | 10 | 10 |
| 862 | 12.14 | tr|A0A024R9D2|A0A024R9D2_HUMAN | Metadherin, isoform CRA_a OS=Homo sapiens GN=MTDH PE=4 SV=1 | 63836.5 | 582 | 15.81 | 6 | 6 | 28 | 28 |
| 863 | 12.14 | tr|Q53FV3|Q53FV3_HUMAN | COP9 signalosome subunit 4 variant (Fragment) OS=Homo sapiens PE=2 SV=1 | 46267.4 | 406 | 20.94 | 6 | 6 | 18 | 18 |
| 864 | 12.14 | tr|Q6FIA3|Q6FIA3_HUMAN | PACSIN2 protein OS=Homo sapiens GN=PACSIN2 PE=2 SV=1 | 51352.8 | 445 | 22.92 | 7 | 7 | 15 | 15 |
| 865 | 12.14 | tr|F5H4B6|F5H4B6_HUMAN | Aldehyde dehydrogenase family 16 member A1 OS=Homo sapiens GN=ALDH16A1 PE=1 SV=1 | 67152.4 | 639 | 11.89 | 6 | 6 | 21 | 21 |
| 866 | 12.13 | sp|P62851|RS25_HUMAN | 40S ribosomal protein S25 OS=Homo sapiens GN=RPS25 PE=1 SV=1 | 13742 | 125 | 36.8 | 7 | 7 | 47 | 45 |
| 867 | 12.12 | tr|B4DWI8|B4DWI8_HUMAN | cDNA FLJ57805, highly similar to Homo sapiens paraspeckle component 1 (PSPC1), transcript variant alpha, mRNA OS=Homo sapiens PE=2 SV=1 | 52579.3 | 463 | 19.87 | 7 | 6 | 17 | 14 |
| 868 | 12.11 | tr|Q53GL5|Q53GL5_HUMAN | Isocitrate dehydrogenase 2 (NADP+), mitochondrial variant (Fragment) OS=Homo sapiens PE=2 SV=1 | 50909.9 | 452 | 17.04 | 6 | 6 | 28 | 28 |
| 869 | 12.1 | tr|B2RAQ8|B2RAQ8_HUMAN | cDNA, FLJ95058, highly similar to Homo sapiens carnitine palmitoyltransferase 1A (liver) (CPT1A),nuclear gene encoding mitochondrial protein, mRNA OS=Homo sapiens PE=2 SV=1 | 88338.9 | 773 | 9.314 | 6 | 6 | 20 | 20 |
| 870 | 12.09 | tr|J3KNP2|J3KNP2_HUMAN | Transducin beta-like protein 3 (Fragment) OS=Homo sapiens GN=TBL3 PE=1 SV=1 | 77036.4 | 697 | 13.49 | 7 | 7 | 31 | 26 |
| 871 | 12.08 | tr|B2R7M3|B2R7M3_HUMAN | cDNA, FLJ93510, highly similar to Homo sapiens JTV1 gene (JTV1), mRNA OS=Homo sapiens PE=2 SV=1 | 35447.6 | 320 | 21.56 | 6 | 6 | 26 | 26 |
| 872 | 12.06 | tr|Q59EA2|Q59EA2_HUMAN | Coronin (Fragment) OS=Homo sapiens PE=2 SV=1 | 56369.1 | 501 | 15.17 | 6 | 6 | 20 | 20 |
| 873 | 12.04 | tr|B4DWA0|B4DWA0_HUMAN | cDNA FLJ54188, moderately similar to High mobility group protein HMG-I/HMG-Y OS=Homo sapiens PE=2 SV=1 | 34301.1 | 328 | 12.8 | 6 | 6 | 41 | 41 |
| 874 | 12.03 | sp|P35659|DEK_HUMAN | Protein DEK OS=Homo sapiens GN=DEK PE=1 SV=1 | 42673.9 | 375 | 13.6 | 6 | 6 | 14 | 14 |
| 875 | 12.02 | tr|B2RCX0|B2RCX0_HUMAN | cDNA, FLJ96345, Homo sapiens SET translocation (myeloid leukemia-associated) (SET),mRNA OS=Homo sapiens PE=2 SV=1 | 32133.8 | 277 | 28.52 | 6 | 6 | 66 | 65 |
| 876 | 12.01 | sp|Q8N684|CPSF7_HUMAN | Cleavage and polyadenylation specificity factor subunit 7 OS=Homo sapiens GN=CPSF7 PE=1 SV=1 | 52049.5 | 471 | 17.2 | 7 | 6 | 21 | 20 |
| 877 | 12.01 | tr|G5EA30|G5EA30_HUMAN | CUG triplet repeat, RNA binding protein 1, isoform CRA_c OS=Homo sapiens GN=CELF1 PE=1 SV=1 | 55142.4 | 514 | 15.37 | 7 | 7 | 17 | 17 |
| 878 | 12 | sp|Q04695|K1C17_HUMAN | Keratin, type I cytoskeletal 17 OS=Homo sapiens GN=KRT17 PE=1 SV=2 | 48105.3 | 432 | 25 | 15 | 6 | 199 | 21 |
| 879 | 12 | sp|Q07812|BAX_HUMAN | Apoptosis regulator BAX OS=Homo sapiens GN=BAX PE=1 SV=1 | 21184.2 | 192 | 36.46 | 6 | 6 | 55 | 55 |
| 880 | 11.95 | tr|B2R4C1|B2R4C1_HUMAN | cDNA, FLJ92036, highly similar to Homo sapiens ribosomal protein L31 (RPL31), mRNA OS=Homo sapiens PE=2 SV=1 | 14494.8 | 125 | 48.8 | 5 | 5 | 50 | 50 |
| 881 | 11.94 | tr|Q59EH3|Q59EH3_HUMAN | Acid phosphatase 1 isoform c variant (Fragment) OS=Homo sapiens PE=2 SV=1 | 18698.1 | 165 | 39.39 | 7 | 7 | 29 | 29 |
| 882 | 11.93 | tr|Q96QA9|Q96QA9_HUMAN | Multidrug resistance associated protein OS=Homo sapiens GN=MRP3 PE=4 SV=1 | 167825.4 | 1514 | 7.53 | 10 | 7 | 21 | 16 |
| 883 | 11.89 | sp|Q92974|ARHG2_HUMAN | Rho guanine nucleotide exchange factor 2 OS=Homo sapiens GN=ARHGEF2 PE=1 SV=4 | 111541.3 | 986 | 12.07 | 9 | 8 | 17 | 13 |
| 884 | 11.88 | sp|Q9UHR4|BI2L1_HUMAN | Brain-specific angiogenesis inhibitor 1-associated protein 2-like protein 1 OS=Homo sapiens GN=BAIAP2L1 PE=1 SV=2 | 56882.1 | 511 | 16.63 | 6 | 6 | 25 | 25 |
| 885 | 11.88 | sp|P16422|EPCAM_HUMAN | Epithelial cell adhesion molecule OS=Homo sapiens GN=EPCAM PE=1 SV=2 | 34932 | 314 | 30.57 | 7 | 7 | 16 | 16 |
| 886 | 11.87 | tr|D3XNU5|D3XNU5_HUMAN | E-cadherin 1 OS=Homo sapiens GN=CDH1 PE=4 SV=1 | 97485.3 | 882 | 8.277 | 13 | 11 | 75 | 64 |
| 887 | 11.87 | sp|Q9BY44|EIF2A_HUMAN | Eukaryotic translation initiation factor 2A OS=Homo sapiens GN=EIF2A PE=1 SV=3 | 64989.7 | 585 | 13.5 | 6 | 6 | 27 | 27 |
| 888 | 11.87 | sp|Q9BTC0|DIDO1_HUMAN | Death-inducer obliterator 1 OS=Homo sapiens GN=DIDO1 PE=1 SV=5 | 243870.3 | 2240 | 4.643 | 8 | 8 | 12 | 12 |
| 889 | 11.86 | sp|P49773|HINT1_HUMAN | Histidine triad nucleotide-binding protein 1 OS=Homo sapiens GN=HINT1 PE=1 SV=2 | 13801.8 | 126 | 61.11 | 7 | 7 | 29 | 29 |
| 890 | 11.85 | tr|A0A024R0Q5|A0A024R0Q5_HUMAN | Protein phosphatase 1, regulatory (Inhibitor) subunit 13 like, isoform CRA_a OS=Homo sapiens GN=PPP1R13L PE=4 SV=1 | 89090.1 | 828 | 11.11 | 6 | 6 | 11 | 11 |
| 891 | 11.83 | tr|A0A024R4S0|A0A024R4S0_HUMAN | Chromatin modifying protein 2A, isoform CRA_b OS=Homo sapiens GN=CHMP2A PE=3 SV=1 | 25103.7 | 222 | 31.08 | 8 | 8 | 28 | 28 |
| 892 | 11.82 | sp|P25398|RS12_HUMAN | 40S ribosomal protein S12 OS=Homo sapiens GN=RPS12 PE=1 SV=3 | 14514.8 | 132 | 47.73 | 7 | 7 | 53 | 53 |
| 893 | 11.82 | tr|G4XXL9|G4XXL9_HUMAN | Cytochrome c OS=Homo sapiens GN=CYCS PE=3 SV=1 | 11748.7 | 105 | 58.1 | 7 | 7 | 34 | 34 |
| 894 | 11.79 | sp|P17812|PYRG1_HUMAN | CTP synthase 1 OS=Homo sapiens GN=CTPS1 PE=1 SV=2 | 66689.9 | 591 | 11.34 | 6 | 6 | 21 | 21 |
| 895 | 11.77 | tr|Q5JR08|Q5JR08_HUMAN | Rho-related GTP-binding protein RhoC (Fragment) OS=Homo sapiens GN=RHOC PE=1 SV=7 | 21579.6 | 189 | 36.51 | 7 | 3 | 35 | 14 |
| 896 | 11.76 | tr|A0A0S2Z404|A0A0S2Z404_HUMAN | Regulator of chromosome condensation 1 isoform 2 (Fragment) OS=Homo sapiens GN=RCC1 PE=2 SV=1 | 48145.4 | 452 | 19.25 | 6 | 6 | 51 | 51 |
| 897 | 11.74 | tr|B4DEN9|B4DEN9_HUMAN | Ribosome biogenesis protein BOP1 OS=Homo sapiens GN=BOP1 PE=2 SV=1 | 71917.7 | 634 | 13.41 | 7 | 7 | 15 | 15 |
| 898 | 11.73 | tr|K7EJE8|K7EJE8_HUMAN | Lon protease homolog, mitochondrial OS=Homo sapiens GN=LONP1 PE=1 SV=1 | 93295.8 | 829 | 9.168 | 7 | 7 | 15 | 14 |
| 899 | 11.7 | tr|A0A024RCZ8|A0A024RCZ8_HUMAN | Kinesin light chain 4, isoform CRA_a OS=Homo sapiens GN=KLC4 PE=4 SV=1 | 68639 | 619 | 12.28 | 6 | 6 | 26 | 25 |
| 900 | 11.68 | tr|B7Z6I3|B7Z6I3_HUMAN | DNA ligase OS=Homo sapiens PE=2 SV=1 | 82321.8 | 738 | 8.808 | 6 | 6 | 20 | 20 |
| 901 | 11.63 | sp|P31947|1433S_HUMAN | 14-3-3 protein sigma OS=Homo sapiens GN=SFN PE=1 SV=1 | 27773.8 | 248 | 44.76 | 12 | 8 | 185 | 36 |
| 902 | 11.62 | sp|Q9NQP4|PFD4_HUMAN | Prefoldin subunit 4 OS=Homo sapiens GN=PFDN4 PE=1 SV=1 | 15314.1 | 134 | 51.49 | 8 | 8 | 37 | 36 |
| 903 | 11.61 | tr|A0A024R718|A0A024R718_HUMAN | Pre-B-cell colony enhancing factor 1, isoform CRA_a OS=Homo sapiens GN=PBEF1 PE=4 SV=1 | 55520.8 | 491 | 15.89 | 7 | 7 | 36 | 36 |
| 904 | 11.6 | tr|B4DNW0|B4DNW0_HUMAN | cDNA FLJ60317, highly similar to Aminoacylase-1 (EC 3.5.1.14) OS=Homo sapiens PE=2 SV=1 | 55994.2 | 498 | 20.88 | 7 | 7 | 23 | 23 |
| 905 | 11.58 | tr|A0A024R565|A0A024R565_HUMAN | Uncharacterized protein OS=Homo sapiens GN=HSPC152 PE=4 SV=1 | 14199.3 | 125 | 54.4 | 6 | 6 | 35 | 35 |
| 906 | 11.57 | sp|Q9HB07|MYG1_HUMAN | UPF0160 protein MYG1, mitochondrial OS=Homo sapiens GN=C12orf10 PE=1 SV=2 | 42448.9 | 376 | 22.34 | 7 | 7 | 22 | 22 |
| 907 | 11.56 | tr|A0A140VJH7|A0A140VJH7_HUMAN | Testis tissue sperm-binding protein Li 84P OS=Homo sapiens PE=2 SV=1 | 58946.5 | 575 | 15.13 | 6 | 6 | 21 | 20 |
| 908 | 11.54 | tr|A8KAN3|A8KAN3_HUMAN | cDNA FLJ77226, highly similar to Homo sapiens p21 activated kinase 1B (PAK1B) mRNA OS=Homo sapiens PE=2 SV=1 | 61591.3 | 553 | 20.61 | 10 | 7 | 37 | 14 |
| 909 | 11.53 | tr|A0A0S2Z5U7|A0A0S2Z5U7_HUMAN | Diablo-like protein isoform 1 (Fragment) OS=Homo sapiens GN=DIABLO PE=2 SV=1 | 27130.6 | 239 | 26.78 | 7 | 6 | 23 | 17 |
| 910 | 11.53 | sp|Q13895|BYST_HUMAN | Bystin OS=Homo sapiens GN=BYSL PE=1 SV=3 | 49600.9 | 437 | 15.1 | 6 | 6 | 21 | 21 |
| 911 | 11.52 | sp|O95202|LETM1_HUMAN | LETM1 and EF-hand domain-containing protein 1, mitochondrial OS=Homo sapiens GN=LETM1 PE=1 SV=1 | 83353.4 | 739 | 11.1 | 6 | 6 | 30 | 29 |
| 912 | 11.49 | sp|Q01970|PLCB3_HUMAN | 1-phosphatidylinositol 4,5-bisphosphate phosphodiesterase beta-3 OS=Homo sapiens GN=PLCB3 PE=1 SV=2 | 138797.7 | 1234 | 6.969 | 7 | 7 | 22 | 22 |
| 913 | 11.46 | tr|Q53FN1|Q53FN1_HUMAN | Cysteine-rich protein 2 variant (Fragment) OS=Homo sapiens PE=2 SV=1 | 22493.5 | 208 | 39.9 | 6 | 5 | 68 | 67 |
| 914 | 11.44 | tr|Q8N9D7|Q8N9D7_HUMAN | cDNA FLJ37680 fis, clone BRHIP2012923, highly similar to FOCAL ADHESION KINASE 1 (EC 2.7.1.112) OS=Homo sapiens PE=2 SV=1 | 81040.6 | 720 | 10 | 7 | 7 | 13 | 13 |
| 915 | 11.44 | sp|A6NHR9|SMHD1_HUMAN | Structural maintenance of chromosomes flexible hinge domain-containing protein 1 OS=Homo sapiens GN=SMCHD1 PE=1 SV=2 | 226371.7 | 2005 | 4.489 | 6 | 6 | 8 | 8 |
| 916 | 11.42 | tr|Q5T5C7|Q5T5C7_HUMAN | Serine--tRNA ligase, cytoplasmic OS=Homo sapiens GN=SARS PE=1 SV=1 | 61312.7 | 536 | 9.888 | 8 | 8 | 18 | 17 |
| 917 | 11.41 | sp|P13073|COX41_HUMAN | Cytochrome c oxidase subunit 4 isoform 1, mitochondrial OS=Homo sapiens GN=COX4I1 PE=1 SV=1 | 19576.6 | 169 | 34.32 | 6 | 6 | 25 | 25 |
| 918 | 11.4 | sp|P25789|PSA4_HUMAN | Proteasome subunit alpha type-4 OS=Homo sapiens GN=PSMA4 PE=1 SV=1 | 29483.6 | 261 | 15.71 | 6 | 6 | 32 | 32 |
| 919 | 11.37 | sp|P82979|SARNP_HUMAN | SAP domain-containing ribonucleoprotein OS=Homo sapiens GN=SARNP PE=1 SV=3 | 23670.6 | 210 | 32.86 | 7 | 7 | 37 | 37 |
| 920 | 11.36 | tr|B4DFR2|B4DFR2_HUMAN | cDNA FLJ59194, moderately similar to Dynein light chain 2A, cytoplasmic OS=Homo sapiens PE=2 SV=1 | 13362.1 | 121 | 51.24 | 6 | 6 | 24 | 24 |
| 921 | 11.36 | tr|Q6LEU2|Q6LEU2_HUMAN | Sodium/potassium-transporting ATPase subunit beta (Fragment) OS=Homo sapiens GN=ATP1B1 PE=2 SV=1 | 35049 | 303 | 18.15 | 6 | 6 | 47 | 47 |
| 922 | 11.34 | tr|A0A024RAH8|A0A024RAH8_HUMAN | DEAD (Asp-Glu-Ala-Asp) box polypeptide 18, isoform CRA_b OS=Homo sapiens GN=DDX18 PE=3 SV=1 | 75406.3 | 670 | 13.73 | 7 | 7 | 16 | 12 |
| 923 | 11.3 | sp|O00429|DNM1L_HUMAN | Dynamin-1-like protein OS=Homo sapiens GN=DNM1L PE=1 SV=2 | 81876.4 | 736 | 11.68 | 6 | 6 | 13 | 13 |
| 924 | 11.29 | sp|Q9NQG5|RPR1B_HUMAN | Regulation of nuclear pre-mRNA domain-containing protein 1B OS=Homo sapiens GN=RPRD1B PE=1 SV=1 | 36899.4 | 326 | 20.25 | 6 | 6 | 29 | 29 |
| 925 | 11.25 | tr|F8W031|F8W031_HUMAN | Uncharacterized protein (Fragment) OS=Homo sapiens PE=1 SV=1 | 29223.9 | 263 | 38.02 | 8 | 6 | 57 | 42 |
| 926 | 11.24 | tr|A4D177|A4D177_HUMAN | Chromobox homolog 3 (HP1 gamma homolog, Drosophila) OS=Homo sapiens GN=CBX3 PE=4 SV=1 | 20811.2 | 183 | 36.07 | 6 | 5 | 50 | 41 |
| 927 | 11.24 | sp|Q16740|CLPP_HUMAN | ATP-dependent Clp protease proteolytic subunit, mitochondrial OS=Homo sapiens GN=CLPP PE=1 SV=1 | 30179.8 | 277 | 31.05 | 6 | 6 | 14 | 14 |
| 928 | 11.22 | sp|Q8TE67|ES8L3_HUMAN | Epidermal growth factor receptor kinase substrate 8-like protein 3 OS=Homo sapiens GN=EPS8L3 PE=1 SV=2 | 66860.5 | 593 | 15.51 | 7 | 7 | 16 | 16 |
| 929 | 11.2 | tr|Q53GD7|Q53GD7_HUMAN | FUS interacting protein (Serine-arginine rich) 1 isoform 2 variant (Fragment) OS=Homo sapiens PE=2 SV=1 | 31282.2 | 262 | 18.32 | 6 | 6 | 12 | 12 |
| 930 | 11.19 | tr|B3KMR5|B3KMR5_HUMAN | cDNA FLJ12434 fis, clone NT2RM1000037, highly similar to Homo sapiens KIAA0690 protein OS=Homo sapiens PE=2 SV=1 | 143670.7 | 1297 | 7.633 | 8 | 8 | 21 | 20 |
| 931 | 11.17 | sp|Q99426|TBCB_HUMAN | Tubulin-folding cofactor B OS=Homo sapiens GN=TBCB PE=1 SV=2 | 27325.3 | 244 | 23.77 | 6 | 6 | 26 | 26 |
| 932 | 11.15 | tr|B7Z5V3|B7Z5V3_HUMAN | cDNA FLJ53088, highly similar to Ras-related protein Rab-18 OS=Homo sapiens PE=2 SV=1 | 20719.3 | 184 | 40.22 | 7 | 5 | 45 | 23 |
| 933 | 11.14 | tr|A0A0S2Z5B3|A0A0S2Z5B3_HUMAN | Ethylmalonic encephalopathy 1 isoform 1 (Fragment) OS=Homo sapiens GN=ETHE1 PE=2 SV=1 | 27872.6 | 254 | 34.65 | 7 | 7 | 33 | 33 |
| 934 | 11.14 | tr|I3L504|I3L504_HUMAN | Eukaryotic translation initiation factor 5A-1 OS=Homo sapiens GN=EIF5A PE=1 SV=1 | 20503.5 | 186 | 38.71 | 7 | 7 | 110 | 110 |
| 935 | 11.11 | tr|B4DW90|B4DW90_HUMAN | cDNA FLJ58737, highly similar to Splicing factor 3A subunit 3 OS=Homo sapiens PE=2 SV=1 | 52427.3 | 448 | 16.07 | 6 | 6 | 30 | 29 |
| 936 | 11.11 | tr|A0A024RAE1|A0A024RAE1_HUMAN | Chromosome 1 open reading frame 33, isoform CRA_a OS=Homo sapiens GN=C1orf33 PE=4 SV=1 | 27560.3 | 239 | 35.15 | 6 | 6 | 13 | 13 |
| 937 | 11.08 | sp|Q92804|RBP56_HUMAN | TATA-binding protein-associated factor 2N OS=Homo sapiens GN=TAF15 PE=1 SV=1 | 61830 | 592 | 16.05 | 6 | 4 | 34 | 15 |
| 938 | 11.07 | tr|F8W727|F8W727_HUMAN | 60S ribosomal protein L32 OS=Homo sapiens GN=RPL32 PE=1 SV=1 | 17962 | 153 | 39.87 | 6 | 6 | 15 | 15 |
| 939 | 11.06 | sp|P42126|ECI1_HUMAN | Enoyl-CoA delta isomerase 1, mitochondrial OS=Homo sapiens GN=ECI1 PE=1 SV=1 | 32815.6 | 302 | 19.21 | 6 | 6 | 70 | 70 |
| 940 | 11.01 | tr|B4DWS6|B4DWS6_HUMAN | cDNA FLJ61181, highly similar to Homo sapiens hydroxysteroid (17-beta) dehydrogenase 12 (HSD17B12), mRNA OS=Homo sapiens PE=2 SV=1 | 33528 | 304 | 21.71 | 7 | 7 | 46 | 45 |
| 941 | 11.01 | sp|P16070|CD44_HUMAN | CD44 antigen OS=Homo sapiens GN=CD44 PE=1 SV=3 | 81537 | 742 | 9.299 | 6 | 6 | 45 | 45 |
| 942 | 11 | tr|Q59EF6|Q59EF6_HUMAN | Calpain 2, large [catalytic] subunit variant (Fragment) OS=Homo sapiens PE=2 SV=1 | 83107.9 | 729 | 11.25 | 6 | 6 | 20 | 20 |
| 943 | 10.98 | tr|A0A140VK46|A0A140VK46_HUMAN | Proteasome subunit beta type OS=Homo sapiens PE=2 SV=1 | 29204 | 264 | 33.71 | 6 | 6 | 53 | 53 |
| 944 | 10.97 | tr|Q53FG5|Q53FG5_HUMAN | DNA helicase (Fragment) OS=Homo sapiens PE=2 SV=1 | 82235.7 | 734 | 10.35 | 6 | 6 | 34 | 34 |
| 945 | 10.96 | tr|A0A024R1U0|A0A024R1U0_HUMAN | Ran GTPase activating protein 1, isoform CRA_d OS=Homo sapiens GN=RANGAP1 PE=4 SV=1 | 63541.3 | 587 | 15.5 | 8 | 8 | 17 | 17 |
| 946 | 10.96 | tr|A0A024R9U3|A0A024R9U3_HUMAN | OCIA domain containing 1, isoform CRA_a OS=Homo sapiens GN=OCIAD1 PE=4 SV=1 | 27625.9 | 245 | 30.2 | 6 | 6 | 17 | 17 |
| 947 | 10.92 | tr|A1L3A7|A1L3A7_HUMAN | Nuclear fragile X mental retardation protein interacting protein 2 OS=Homo sapiens GN=NUFIP2 PE=1 SV=1 | 76120.9 | 695 | 9.928 | 5 | 5 | 17 | 17 |
| 948 | 10.92 | sp|Q8WUY1|THEM6_HUMAN | Protein THEM6 OS=Homo sapiens GN=THEM6 PE=1 SV=2 | 23864.6 | 208 | 36.54 | 5 | 5 | 32 | 32 |
| 949 | 10.91 | tr|Q53G49|Q53G49_HUMAN | Ribosomal protein L19 (Fragment) OS=Homo sapiens PE=2 SV=1 | 23466.7 | 196 | 20.92 | 6 | 6 | 55 | 55 |
| 950 | 10.91 | tr|A0A024RB87|A0A024RB87_HUMAN | RAP1B, member of RAS oncogene family, isoform CRA_a OS=Homo sapiens GN=RAP1B PE=4 SV=1 | 20824.7 | 184 | 30.43 | 6 | 5 | 42 | 36 |
| 951 | 10.89 | sp|Q8NBJ5|GT251_HUMAN | Procollagen galactosyltransferase 1 OS=Homo sapiens GN=COLGALT1 PE=1 SV=1 | 71635.4 | 622 | 9.486 | 6 | 5 | 18 | 14 |
| 952 | 10.85 | sp|Q07960|RHG01_HUMAN | Rho GTPase-activating protein 1 OS=Homo sapiens GN=ARHGAP1 PE=1 SV=1 | 50435.3 | 439 | 12.76 | 6 | 6 | 25 | 24 |
| 953 | 10.85 | tr|B2RCT6|B2RCT6_HUMAN | cDNA, FLJ96276, highly similar to Homo sapiens G1 to S phase transition 1 (GSPT1), mRNA OS=Homo sapiens PE=2 SV=1 | 55727.5 | 499 | 14.63 | 7 | 7 | 16 | 15 |
| 954 | 10.77 | sp|Q14126|DSG2_HUMAN | Desmoglein-2 OS=Homo sapiens GN=DSG2 PE=1 SV=2 | 122293 | 1118 | 12.25 | 7 | 7 | 11 | 11 |
| 955 | 10.76 | sp|P08243|ASNS_HUMAN | Asparagine synthetase [glutamine-hydrolyzing] OS=Homo sapiens GN=ASNS PE=1 SV=4 | 64369.4 | 561 | 12.3 | 7 | 6 | 16 | 15 |
| 956 | 10.75 | tr|Q8TAS0|Q8TAS0_HUMAN | ATP synthase subunit gamma (Fragment) OS=Homo sapiens PE=2 SV=1 | 32246.8 | 291 | 24.74 | 7 | 6 | 29 | 27 |
| 957 | 10.75 | tr|Q53GF8|Q53GF8_HUMAN | Isocitrate dehydrogenase [NAD] subunit, mitochondrial (Fragment) OS=Homo sapiens PE=2 SV=1 | 39619.4 | 366 | 12.57 | 5 | 5 | 22 | 22 |
| 958 | 10.75 | tr|M0QY97|M0QY97_HUMAN | Zinc finger CCCH domain-containing protein 4 (Fragment) OS=Homo sapiens GN=ZC3H4 PE=1 SV=1 | 95482.9 | 910 | 12.64 | 6 | 6 | 14 | 14 |
| 959 | 10.74 | tr|Q05BV5|Q05BV5_HUMAN | OGFR protein OS=Homo sapiens GN=OGFR PE=2 SV=1 | 71800.5 | 660 | 11.67 | 5 | 5 | 13 | 13 |
| 960 | 10.71 | tr|A0A024R7M6|A0A024R7M6_HUMAN | GATA zinc finger domain containing 2A, isoform CRA_a OS=Homo sapiens GN=GATAD2A PE=4 SV=1 | 68133.2 | 634 | 12.62 | 6 | 5 | 15 | 9 |
| 961 | 10.7 | sp|P35268|RL22_HUMAN | 60S ribosomal protein L22 OS=Homo sapiens GN=RPL22 PE=1 SV=2 | 14786.9 | 128 | 40.63 | 6 | 6 | 60 | 58 |
| 962 | 10.69 | tr|A0A024RAM0|A0A024RAM0_HUMAN | Transportin 1, isoform CRA_a OS=Homo sapiens GN=TNPO1 PE=4 SV=1 | 102354 | 898 | 8.241 | 6 | 6 | 18 | 17 |
| 963 | 10.69 | sp|P47985|UCRI_HUMAN | Cytochrome b-c1 complex subunit Rieske, mitochondrial OS=Homo sapiens GN=UQCRFS1 PE=1 SV=2 | 29667.7 | 274 | 21.9 | 5 | 5 | 42 | 42 |
| 964 | 10.65 | tr|V9HWA2|V9HWA2_HUMAN | Aldo-keto reductase family 7, member A2 (Aflatoxin aldehyde reductase) OS=Homo sapiens GN=HEL-S-166mP PE=2 SV=1 | 39588.6 | 359 | 21.73 | 6 | 5 | 28 | 27 |
| 965 | 10.65 | tr|A0A024R5S5|A0A024R5S5_HUMAN | Eukaryotic translation initiation factor 3 subunit J OS=Homo sapiens GN=EIF3S1 PE=3 SV=1 | 29062.2 | 258 | 26.36 | 6 | 6 | 30 | 30 |
| 966 | 10.64 | sp|Q13884|SNTB1_HUMAN | Beta-1-syntrophin OS=Homo sapiens GN=SNTB1 PE=1 SV=3 | 58060.6 | 538 | 14.87 | 6 | 6 | 11 | 11 |
| 967 | 10.62 | tr|Q59EI9|Q59EI9_HUMAN | ADP,ATP carrier protein, liver isoform T2 variant (Fragment) OS=Homo sapiens PE=2 SV=1 | 35382.8 | 323 | 32.82 | 14 | 6 | 89 | 45 |
| 968 | 10.62 | sp|P62750|RL23A_HUMAN | 60S ribosomal protein L23a OS=Homo sapiens GN=RPL23A PE=1 SV=1 | 17694.9 | 156 | 30.13 | 6 | 6 | 53 | 53 |
| 969 | 10.62 | tr|B4DM33|B4DM33_HUMAN | cDNA FLJ52068, highly similar to Microtubule-associated protein RP/EB family member 1 OS=Homo sapiens PE=2 SV=1 | 26581.9 | 238 | 36.97 | 5 | 5 | 46 | 46 |
| 970 | 10.61 | sp|P52948|NUP98_HUMAN | Nuclear pore complex protein Nup98-Nup96 OS=Homo sapiens GN=NUP98 PE=1 SV=4 | 197577.7 | 1817 | 4.568 | 7 | 7 | 15 | 15 |
| 971 | 10.58 | sp|P48147|PPCE_HUMAN | Prolyl endopeptidase OS=Homo sapiens GN=PREP PE=1 SV=2 | 80698.9 | 710 | 9.859 | 7 | 7 | 25 | 24 |
| 972 | 10.55 | sp|Q13885|TBB2A_HUMAN | Tubulin beta-2A chain OS=Homo sapiens GN=TUBB2A PE=1 SV=1 | 49906.7 | 445 | 58.2 | 26 | 6 | 623 | 20 |
| 973 | 10.54 | tr|A8K168|A8K168_HUMAN | Malic enzyme OS=Homo sapiens PE=2 SV=1 | 64135.1 | 572 | 9.965 | 6 | 6 | 13 | 13 |
| 974 | 10.51 | sp|Q9UIG0|BAZ1B_HUMAN | Tyrosine-protein kinase BAZ1B OS=Homo sapiens GN=BAZ1B PE=1 SV=2 | 170901.4 | 1483 | 6.069 | 8 | 7 | 13 | 12 |
| 975 | 10.51 | sp|O60493|SNX3_HUMAN | Sorting nexin-3 OS=Homo sapiens GN=SNX3 PE=1 SV=3 | 18762.2 | 162 | 32.1 | 5 | 4 | 13 | 9 |
| 976 | 10.49 | tr|F8W930|F8W930_HUMAN | Insulin-like growth factor 2 mRNA-binding protein 2 OS=Homo sapiens GN=IGF2BP2 PE=1 SV=1 | 66785.8 | 605 | 15.87 | 7 | 6 | 54 | 29 |
| 977 | 10.47 | sp|P43155|CACP_HUMAN | Carnitine O-acetyltransferase OS=Homo sapiens GN=CRAT PE=1 SV=5 | 70857.1 | 626 | 12.94 | 7 | 7 | 26 | 26 |
| 978 | 10.43 | tr|Q6FII1|Q6FII1_HUMAN | Glutathione S-transferase kappa 1 OS=Homo sapiens GN=LOC51064 PE=2 SV=1 | 25496.6 | 226 | 26.11 | 5 | 5 | 83 | 83 |
| 979 | 10.41 | tr|Q59GW6|Q59GW6_HUMAN | Acetyl-CoA acetyltransferase, cytosolic variant (Fragment) OS=Homo sapiens PE=2 SV=1 | 42135.3 | 404 | 24.75 | 5 | 5 | 8 | 8 |
| 980 | 10.4 | sp|Q13501|SQSTM_HUMAN | Sequestosome-1 OS=Homo sapiens GN=SQSTM1 PE=1 SV=1 | 47686.7 | 440 | 21.82 | 6 | 6 | 21 | 20 |
| 981 | 10.39 | tr|B4E0Q4|B4E0Q4_HUMAN | Suppressor of Ty 5 homolog (S. cerevisiae), isoform CRA_a OS=Homo sapiens GN=SUPT5H PE=2 SV=1 | 104382.3 | 946 | 11.73 | 8 | 8 | 14 | 12 |
| 982 | 10.39 | tr|B4DZ08|B4DZ08_HUMAN | Aconitate hydratase, mitochondrial OS=Homo sapiens PE=2 SV=1 | 83412.4 | 761 | 10.78 | 6 | 6 | 22 | 22 |
| 983 | 10.36 | sp|P47813|IF1AX_HUMAN | Eukaryotic translation initiation factor 1A, X-chromosomal OS=Homo sapiens GN=EIF1AX PE=1 SV=2 | 16460.3 | 144 | 42.36 | 5 | 5 | 25 | 25 |
| 984 | 10.36 | sp|O00154|BACH_HUMAN | Cytosolic acyl coenzyme A thioester hydrolase OS=Homo sapiens GN=ACOT7 PE=1 SV=3 | 41795.8 | 380 | 23.95 | 7 | 7 | 26 | 25 |
| 985 | 10.36 | sp|O95881|TXD12_HUMAN | Thioredoxin domain-containing protein 12 OS=Homo sapiens GN=TXNDC12 PE=1 SV=1 | 19205.6 | 172 | 29.65 | 5 | 5 | 17 | 17 |
| 986 | 10.32 | sp|P52701|MSH6_HUMAN | DNA mismatch repair protein Msh6 OS=Homo sapiens GN=MSH6 PE=1 SV=2 | 152784.1 | 1360 | 7.5 | 9 | 9 | 25 | 24 |
| 987 | 10.3 | sp|Q96C86|DCPS_HUMAN | m7GpppX diphosphatase OS=Homo sapiens GN=DCPS PE=1 SV=2 | 38608.4 | 337 | 20.47 | 6 | 6 | 17 | 17 |
| 988 | 10.29 | sp|Q9H3U1|UN45A_HUMAN | Protein unc-45 homolog A OS=Homo sapiens GN=UNC45A PE=1 SV=1 | 103075.9 | 944 | 7.309 | 7 | 7 | 24 | 24 |
| 989 | 10.28 | tr|B4DL07|B4DL07_HUMAN | cDNA FLJ53353, highly similar to ATP-binding cassette sub-family D member 3 OS=Homo sapiens PE=2 SV=1 | 78459.6 | 683 | 11.27 | 6 | 6 | 19 | 19 |
| 990 | 10.26 | sp|P46937|YAP1_HUMAN | Transcriptional coactivator YAP1 OS=Homo sapiens GN=YAP1 PE=1 SV=2 | 54461.2 | 504 | 24.8 | 7 | 6 | 14 | 13 |
| 991 | 10.26 | tr|Q59ET7|Q59ET7_HUMAN | Thioredoxin reductase 2 isoform 1 variant (Fragment) OS=Homo sapiens PE=2 SV=1 | 55217.7 | 511 | 16.24 | 6 | 6 | 12 | 12 |
| 992 | 10.24 | tr|E9PR38|E9PR38_HUMAN | Pumilio homolog 1 OS=Homo sapiens GN=PUM1 PE=1 SV=1 | 100499.3 | 944 | 8.157 | 6 | 5 | 15 | 14 |
| 993 | 10.24 | tr|B4DDG3|B4DDG3_HUMAN | cDNA FLJ51688, highly similar to Cleavage stimulation factor 50 kDa subunit OS=Homo sapiens PE=2 SV=1 | 46836.5 | 418 | 26.08 | 7 | 7 | 16 | 16 |
| 994 | 10.24 | tr|F6TLX2|F6TLX2_HUMAN | Glyoxalase domain-containing protein 4 OS=Homo sapiens GN=GLOD4 PE=1 SV=1 | 54719.1 | 502 | 15.14 | 5 | 5 | 27 | 27 |
| 995 | 10.23 | sp|Q96T51|RUFY1_HUMAN | RUN and FYVE domain-containing protein 1 OS=Homo sapiens GN=RUFY1 PE=1 SV=2 | 79817.2 | 708 | 13.56 | 8 | 8 | 15 | 15 |
| 996 | 10.23 | sp|Q9P035|HACD3_HUMAN | Very-long-chain (3R)-3-hydroxyacyl-CoA dehydratase 3 OS=Homo sapiens GN=HACD3 PE=1 SV=2 | 43159.2 | 362 | 16.3 | 5 | 5 | 54 | 54 |
| 997 | 10.23 | tr|A0A140VJY7|A0A140VJY7_HUMAN | Testicular tissue protein Li 214 OS=Homo sapiens PE=2 SV=1 | 13940.7 | 123 | 42.28 | 6 | 6 | 56 | 56 |
| 998 | 10.22 | tr|A0A024RA75|A0A024RA75_HUMAN | 3-hydroxyisobutyrate dehydrogenase OS=Homo sapiens GN=HIBADH PE=3 SV=1 | 35328.5 | 336 | 24.11 | 6 | 6 | 27 | 27 |
| 999 | 10.22 | sp|P37108|SRP14_HUMAN | Signal recognition particle 14 kDa protein OS=Homo sapiens GN=SRP14 PE=1 SV=2 | 14569.8 | 136 | 48.53 | 6 | 6 | 63 | 63 |
| 1000 | 10.21 | sp|Q9BTY7|HGH1_HUMAN | Protein HGH1 homolog OS=Homo sapiens GN=HGH1 PE=1 SV=1 | 42128.7 | 390 | 23.85 | 6 | 6 | 14 | 14 |
| 1001 | 10.21 | sp|Q9H6T3|RPAP3_HUMAN | RNA polymerase II-associated protein 3 OS=Homo sapiens GN=RPAP3 PE=1 SV=2 | 75717.9 | 665 | 10.83 | 5 | 5 | 15 | 15 |
| 1002 | 10.2 | tr|A0A140VK83|A0A140VK83_HUMAN | Protein phosphatase 1, regulatory subunit 7, isoform CRA_b OS=Homo sapiens GN=PPP1R7 PE=2 SV=1 | 41563.8 | 360 | 23.33 | 7 | 7 | 19 | 19 |
| 1003 | 10.2 | sp|P21291|CSRP1_HUMAN | Cysteine and glycine-rich protein 1 OS=Homo sapiens GN=CSRP1 PE=1 SV=3 | 20567.3 | 193 | 45.6 | 6 | 6 | 32 | 32 |
| 1004 | 10.18 | tr|A0A024RBD0|A0A024RBD0_HUMAN | Nudix (Nucleoside diphosphate linked moiety X)-type motif 4, isoform CRA_c OS=Homo sapiens GN=NUDT4 PE=4 SV=1 | 20305.9 | 180 | 32.78 | 5 | 5 | 43 | 42 |
| 1005 | 10.14 | tr|A0MNN4|A0MNN4_HUMAN | CDW3/SMU1 OS=Homo sapiens GN=SMU1 PE=2 SV=1 | 57543.4 | 513 | 13.26 | 6 | 5 | 25 | 23 |
| 1006 | 10.14 | sp|P49458|SRP09_HUMAN | Signal recognition particle 9 kDa protein OS=Homo sapiens GN=SRP9 PE=1 SV=2 | 10111.7 | 86 | 46.51 | 5 | 5 | 50 | 50 |
| 1007 | 10.12 | sp|Q14165|MLEC_HUMAN | Malectin OS=Homo sapiens GN=MLEC PE=1 SV=1 | 32233.6 | 292 | 21.58 | 7 | 6 | 37 | 34 |
| 1008 | 10.11 | sp|Q14690|RRP5_HUMAN | Protein RRP5 homolog OS=Homo sapiens GN=PDCD11 PE=1 SV=3 | 208699.3 | 1871 | 5.345 | 7 | 7 | 17 | 17 |
| 1009 | 10.07 | sp|Q8NEV1|CSK23_HUMAN | Casein kinase II subunit alpha 3 OS=Homo sapiens GN=CSNK2A3 PE=1 SV=2 | 45219.4 | 391 | 27.11 | 7 | 6 | 18 | 17 |
| 1010 | 10.05 | sp|Q9NVH1|DJC11_HUMAN | DnaJ homolog subfamily C member 11 OS=Homo sapiens GN=DNAJC11 PE=1 SV=2 | 63277.6 | 559 | 10.55 | 5 | 5 | 13 | 13 |
| 1011 | 10.05 | tr|B2R6U8|B2R6U8_HUMAN | cDNA, FLJ93125, highly similar to Homo sapiens cleavage and polyadenylation specific factor 5, 25 kDa(CPSF5), mRNA OS=Homo sapiens PE=2 SV=1 | 26215.1 | 227 | 25.99 | 6 | 6 | 18 | 18 |
| 1012 | 10.05 | tr|A0A024RB72|A0A024RB72_HUMAN | Coatomer protein complex, subunit zeta 1, isoform CRA_a OS=Homo sapiens GN=COPZ1 PE=4 SV=1 | 20198 | 177 | 36.72 | 5 | 5 | 34 | 34 |
| 1013 | 10.05 | tr|Q549C5|Q549C5_HUMAN | HCG2010808, isoform CRA_a OS=Homo sapiens GN=MST065 PE=1 SV=1 | 15521.5 | 142 | 59.86 | 5 | 5 | 13 | 13 |
| 1014 | 10.04 | tr|A8K586|A8K586_HUMAN | AP-3 complex subunit beta OS=Homo sapiens PE=2 SV=1 | 121288.9 | 1094 | 6.764 | 6 | 6 | 10 | 10 |
| 1015 | 10.04 | sp|P05556|ITB1_HUMAN | Integrin beta-1 OS=Homo sapiens GN=ITGB1 PE=1 SV=2 | 88414.6 | 798 | 7.268 | 5 | 5 | 42 | 42 |
| 1016 | 10.04 | tr|B2R704|B2R704_HUMAN | cDNA, FLJ93207, highly similar to Homo sapiens microtubule-associated protein 7 (MAP7), mRNA OS=Homo sapiens PE=2 SV=1 | 83981.9 | 749 | 10.81 | 7 | 7 | 14 | 14 |
| 1017 | 10.04 | tr|A8K2S7|A8K2S7_HUMAN | cDNA FLJ77865 OS=Homo sapiens PE=2 SV=1 | 38947 | 344 | 16.28 | 5 | 5 | 18 | 17 |
| 1018 | 10.03 | tr|A0A024RAE4|A0A024RAE4_HUMAN | Cell division cycle 42 (GTP binding protein, 25kDa), isoform CRA_a OS=Homo sapiens GN=CDC42 PE=4 SV=1 | 21258.4 | 191 | 27.75 | 6 | 5 | 66 | 62 |
| 1019 | 10.02 | tr|A0A024R2Q4|A0A024R2Q4_HUMAN | Ribosomal protein L15 OS=Homo sapiens GN=RPL15 PE=3 SV=1 | 24145.9 | 204 | 23.53 | 5 | 5 | 27 | 27 |
| 1020 | 10.01 | tr|Q8TDJ5|Q8TDJ5_HUMAN | Tyrosine-protein kinase receptor OS=Homo sapiens GN=TFG/ALK fusion PE=2 SV=1 | 88670.1 | 803 | 10.34 | 6 | 5 | 57 | 55 |
| 1021 | 10.01 | tr|B7Z5S9|B7Z5S9_HUMAN | cDNA FLJ61359, highly similar to Adapter-relatedprotein complex 2 alpha- 2 subunit OS=Homo sapiens PE=2 SV=1 | 103056.4 | 930 | 5.806 | 5 | 3 | 13 | 10 |
| 1022 | 10.01 | tr|Q5U0A0|Q5U0A0_HUMAN | Proteasome subunit alpha type OS=Homo sapiens PE=2 SV=1 | 26424.8 | 241 | 24.48 | 5 | 5 | 85 | 85 |
| 1023 | 10 | tr|Q8TBK9|Q8TBK9_HUMAN | Prothymosin, alpha OS=Homo sapiens GN=PTMA PE=2 SV=1 | 12015.7 | 110 | 40.91 | 5 | 5 | 389 | 389 |
| 1024 | 10 | tr|Q6FHJ5|Q6FHJ5_HUMAN | SCAMP3 protein (Fragment) OS=Homo sapiens GN=SCAMP3 PE=2 SV=1 | 38242.5 | 347 | 23.63 | 5 | 5 | 38 | 38 |
| 1025 | 9.97 | tr|H7BXI1|H7BXI1_HUMAN | Extended synaptotagmin-2 (Fragment) OS=Homo sapiens GN=ESYT2 PE=1 SV=1 | 98008.8 | 884 | 6.674 | 5 | 5 | 12 | 12 |
| 1026 | 9.97 | tr|B2R4C5|B2R4C5_HUMAN | C-type lysozyme OS=Homo sapiens GN=LYZ PE=2 SV=1 | 16536.9 | 148 | 19.59 | 4 | 4 | 32 | 32 |
| 1027 | 9.96 | tr|B7ZLC9|B7ZLC9_HUMAN | GEMIN5 protein OS=Homo sapiens GN=GEMIN5 PE=2 SV=1 | 168430.8 | 1507 | 5.508 | 6 | 6 | 20 | 20 |
| 1028 | 9.95 | sp|O43765|SGTA_HUMAN | Small glutamine-rich tetratricopeptide repeat-containing protein alpha OS=Homo sapiens GN=SGTA PE=1 SV=1 | 34062.8 | 313 | 22.68 | 7 | 6 | 21 | 17 |
| 1029 | 9.94 | sp|Q8N257|H2B3B_HUMAN | Histone H2B type 3-B OS=Homo sapiens GN=HIST3H2BB PE=1 SV=3 | 13908 | 126 | 77.78 | 18 | 4 | 795 | 66 |
| 1030 | 9.94 | tr|V9HWK0|V9HWK0_HUMAN | Signal recognition particle subunit SRP72 OS=Homo sapiens GN=HEL103 PE=2 SV=1 | 74605.6 | 671 | 10.13 | 6 | 6 | 14 | 14 |
| 1031 | 9.94 | sp|P46776|RL27A_HUMAN | 60S ribosomal protein L27a OS=Homo sapiens GN=RPL27A PE=1 SV=2 | 16561.4 | 148 | 43.24 | 6 | 6 | 57 | 57 |
| 1032 | 9.93 | sp|P35908|K22E_HUMAN | Keratin, type II cytoskeletal 2 epidermal OS=Homo sapiens GN=KRT2 PE=1 SV=2 | 65432.7 | 639 | 18.47 | 16 | 6 | 189 | 14 |
| 1033 | 9.92 | tr|Q6IPN0|Q6IPN0_HUMAN | Reticulon OS=Homo sapiens GN=RTN4 PE=2 SV=1 | 36917.8 | 343 | 24.78 | 5 | 1 | 36 | 1 |
| 1034 | 9.92 | sp|Q9Y3U8|RL36_HUMAN | 60S ribosomal protein L36 OS=Homo sapiens GN=RPL36 PE=1 SV=3 | 12253.6 | 105 | 37.14 | 5 | 5 | 23 | 15 |
| 1035 | 9.92 | tr|Q86XN0|Q86XN0_HUMAN | MRPL43 protein (Fragment) OS=Homo sapiens GN=MRPL43 PE=2 SV=1 | 15960.9 | 145 | 32.41 | 5 | 5 | 14 | 14 |
| 1036 | 9.91 | tr|Q7Z5G3|Q7Z5G3_HUMAN | Acetyl-coenzyme A synthetase OS=Homo sapiens GN=ACSS1 PE=2 SV=1 | 74657.9 | 687 | 10.48 | 6 | 6 | 14 | 14 |
| 1037 | 9.9 | tr|E9PF16|E9PF16_HUMAN | Acyl-CoA synthetase family member 2, mitochondrial OS=Homo sapiens GN=ACSF2 PE=1 SV=1 | 63652.1 | 572 | 12.59 | 5 | 5 | 28 | 28 |
| 1038 | 9.89 | tr|A0A0S2Z4C6|A0A0S2Z4C6_HUMAN | Serine/threonine-protein phosphatase (Fragment) OS=Homo sapiens GN=PPP3CA PE=2 SV=1 | 58687.3 | 521 | 14.2 | 6 | 5 | 16 | 14 |
| 1039 | 9.89 | tr|B2R5R5|B2R5R5_HUMAN | cDNA, FLJ92583, highly similar to Homo sapiens glycogenin (GYG), mRNA OS=Homo sapiens PE=2 SV=1 | 39353.4 | 350 | 18.86 | 5 | 5 | 28 | 27 |
| 1040 | 9.88 | tr|Q59FL8|Q59FL8_HUMAN | Receptor protein-tyrosine kinase (Fragment) OS=Homo sapiens PE=2 SV=1 | 119950.7 | 1081 | 7.123 | 6 | 6 | 16 | 16 |
| 1041 | 9.88 | sp|Q9BX68|HINT2_HUMAN | Histidine triad nucleotide-binding protein 2, mitochondrial OS=Homo sapiens GN=HINT2 PE=1 SV=1 | 17161.6 | 163 | 44.17 | 5 | 5 | 31 | 30 |
| 1042 | 9.88 | tr|Q5U000|Q5U000_HUMAN | Cathepsin Z OS=Homo sapiens PE=2 SV=1 | 33867.7 | 303 | 16.5 | 5 | 5 | 30 | 30 |
| 1043 | 9.86 | tr|Q6ICQ8|Q6ICQ8_HUMAN | ARHG protein (Fragment) OS=Homo sapiens GN=ARHG PE=2 SV=1 | 21308.3 | 191 | 55.5 | 7 | 6 | 18 | 15 |
| 1044 | 9.86 | tr|A8K9U0|A8K9U0_HUMAN | cDNA FLJ78260, highly similar to Homo sapiens RNA binding motif protein 4, mRNA OS=Homo sapiens PE=2 SV=1 | 40283.4 | 364 | 18.96 | 5 | 2 | 26 | 19 |
| 1045 | 9.86 | tr|D3DUE7|D3DUE7_HUMAN | Cytokine-like nuclear factor n-pac, isoform CRA_a OS=Homo sapiens GN=N-PAC PE=4 SV=1 | 62184.9 | 569 | 14.06 | 5 | 5 | 10 | 10 |
| 1046 | 9.85 | tr|Q4VXZ2|Q4VXZ2_HUMAN | Vacuolar protein sorting 52 (Yeast) OS=Homo sapiens GN=VPS52 PE=4 SV=1 | 82220.4 | 723 | 10.37 | 5 | 5 | 13 | 13 |
| 1047 | 9.83 | tr|D3DU92|D3DU92_HUMAN | RNA binding protein S1 OS=Homo sapiens GN=RNPS1 PE=1 SV=1 | 34207.8 | 305 | 26.89 | 6 | 6 | 23 | 23 |
| 1048 | 9.83 | sp|P49006|MRP_HUMAN | MARCKS-related protein OS=Homo sapiens GN=MARCKSL1 PE=1 SV=2 | 19528.7 | 195 | 41.03 | 5 | 4 | 39 | 38 |
| 1049 | 9.82 | tr|A0A024R4T4|A0A024R4T4_HUMAN | Ubiquitin-conjugating enzyme E2M (UBC12 homolog, yeast), isoform CRA_a OS=Homo sapiens GN=UBE2M PE=3 SV=1 | 20899.8 | 183 | 32.79 | 6 | 6 | 30 | 30 |
| 1050 | 9.81 | tr|J3KQJ1|J3KQJ1_HUMAN | Sulfatase-modifying factor 2 OS=Homo sapiens GN=SUMF2 PE=1 SV=1 | 35922.3 | 320 | 16.88 | 5 | 5 | 17 | 17 |
| 1051 | 9.8 | tr|A0A140VK54|A0A140VK54_HUMAN | Testicular secretory protein Li 54 OS=Homo sapiens PE=2 SV=1 | 48076.5 | 424 | 18.87 | 6 | 6 | 9 | 9 |
| 1052 | 9.8 | sp|Q9Y320|TMX2_HUMAN | Thioredoxin-related transmembrane protein 2 OS=Homo sapiens GN=TMX2 PE=1 SV=1 | 34037.3 | 296 | 21.96 | 6 | 6 | 17 | 15 |
| 1053 | 9.76 | tr|A0A090N7Y2|A0A090N7Y2_HUMAN | ATP-binding cassette, sub-family F (GCN20), member 2 OS=Homo sapiens GN=ABCF2 PE=4 SV=1 | 72443.1 | 634 | 8.044 | 5 | 5 | 19 | 19 |
| 1054 | 9.74 | tr|A0A024RB32|A0A024RB32_HUMAN | Prostaglandin E synthase 3 (Cytosolic), isoform CRA_a OS=Homo sapiens GN=PTGES3 PE=4 SV=1 | 18697.2 | 160 | 40 | 7 | 7 | 12 | 12 |
| 1055 | 9.7 | sp|Q92979|NEP1_HUMAN | Ribosomal RNA small subunit methyltransferase NEP1 OS=Homo sapiens GN=EMG1 PE=1 SV=4 | 26719.9 | 244 | 23.36 | 5 | 5 | 11 | 11 |
| 1056 | 9.69 | tr|A8K5T7|A8K5T7_HUMAN | cDNA FLJ75365, highly similar to Homo sapiens SUGT1B (SUGT1) mRNA OS=Homo sapiens PE=2 SV=1 | 41051.8 | 365 | 21.64 | 6 | 6 | 19 | 19 |
| 1057 | 9.68 | tr|A0A140VK39|A0A140VK39_HUMAN | Protein phosphatase methylesterase 1 OS=Homo sapiens PE=2 SV=1 | 42315.1 | 386 | 17.62 | 5 | 5 | 20 | 20 |
| 1058 | 9.67 | sp|Q9H2G2|SLK_HUMAN | STE20-like serine/threonine-protein kinase OS=Homo sapiens GN=SLK PE=1 SV=1 | 142694 | 1235 | 5.02 | 6 | 6 | 17 | 17 |
| 1059 | 9.67 | sp|P09497|CLCB_HUMAN | Clathrin light chain B OS=Homo sapiens GN=CLTB PE=1 SV=1 | 25190.3 | 229 | 24.02 | 6 | 5 | 14 | 11 |
| 1060 | 9.66 | tr|A0A087WT45|A0A087WT45_HUMAN | GRIP1-associated protein 1 OS=Homo sapiens GN=GRIPAP1 PE=1 SV=1 | 90856.1 | 796 | 12.19 | 6 | 6 | 8 | 8 |
| 1061 | 9.66 | sp|Q56VL3|OCAD2_HUMAN | OCIA domain-containing protein 2 OS=Homo sapiens GN=OCIAD2 PE=1 SV=1 | 16953.4 | 154 | 44.16 | 6 | 6 | 17 | 17 |
| 1062 | 9.63 | sp|P04899|GNAI2_HUMAN | Guanine nucleotide-binding protein G(i) subunit alpha-2 OS=Homo sapiens GN=GNAI2 PE=1 SV=3 | 40450.5 | 355 | 22.82 | 7 | 4 | 82 | 24 |
| 1063 | 9.61 | tr|A0A0A0MSW4|A0A0A0MSW4_HUMAN | Phosphatidylinositol transfer protein beta isoform OS=Homo sapiens GN=PITPNB PE=1 SV=1 | 31581 | 271 | 20.3 | 5 | 4 | 13 | 9 |
| 1064 | 9.6 | tr|A0A0S2Z5U6|A0A0S2Z5U6_HUMAN | Pyrroline-5-carboxylate reductase (Fragment) OS=Homo sapiens GN=PYCR2 PE=2 SV=1 | 33636.8 | 320 | 24.06 | 6 | 5 | 24 | 22 |
| 1065 | 9.6 | sp|Q5BKZ1|ZN326_HUMAN | DBIRD complex subunit ZNF326 OS=Homo sapiens GN=ZNF326 PE=1 SV=2 | 65653.6 | 582 | 10.31 | 5 | 5 | 14 | 14 |
| 1066 | 9.6 | tr|A0A140VJJ2|A0A140VJJ2_HUMAN | S-formylglutathione hydrolase OS=Homo sapiens GN=ESD PE=2 SV=1 | 31462.5 | 282 | 23.05 | 5 | 5 | 18 | 17 |
| 1067 | 9.59 | tr|X5D8Y5|X5D8Y5_HUMAN | C-terminal binding protein 1 isoform C (Fragment) OS=Homo sapiens GN=CTBP1 PE=2 SV=1 | 47534.9 | 440 | 12.05 | 6 | 4 | 13 | 4 |
| 1068 | 9.55 | tr|X5D8S6|X5D8S6_HUMAN | Adenylosuccinate lyase (Fragment) OS=Homo sapiens GN=ADSL PE=2 SV=1 | 54888.7 | 484 | 14.67 | 5 | 5 | 8 | 8 |
| 1069 | 9.55 | sp|Q9BV40|VAMP8_HUMAN | Vesicle-associated membrane protein 8 OS=Homo sapiens GN=VAMP8 PE=1 SV=1 | 11438.2 | 100 | 41 | 5 | 5 | 17 | 17 |
| 1070 | 9.53 | tr|G1UI16|G1UI16_HUMAN | SCC-112 protein, isoform CRA_b OS=Homo sapiens GN=PDS5A PE=2 SV=1 | 150828.7 | 1337 | 8.153 | 7 | 7 | 8 | 8 |
| 1071 | 9.52 | tr|A0A087WYF6|A0A087WYF6_HUMAN | WASH complex subunit FAM21A OS=Homo sapiens GN=FAM21A PE=1 SV=1 | 136569.4 | 1245 | 8.755 | 6 | 6 | 8 | 8 |
| 1072 | 9.51 | tr|A0A087X0Q1|A0A087X0Q1_HUMAN | YTH domain-containing family protein 3 OS=Homo sapiens GN=YTHDF3 PE=1 SV=1 | 63674.8 | 583 | 10.29 | 5 | 3 | 10 | 5 |
| 1073 | 9.49 | sp|Q96A65|EXOC4_HUMAN | Exocyst complex component 4 OS=Homo sapiens GN=EXOC4 PE=1 SV=1 | 110497 | 974 | 6.366 | 5 | 5 | 20 | 19 |
| 1074 | 9.49 | tr|A0A087WZT3|A0A087WZT3_HUMAN | BolA-like protein 2 OS=Homo sapiens GN=BOLA2 PE=1 SV=2 | 16932.2 | 152 | 40.79 | 5 | 5 | 35 | 35 |
| 1075 | 9.47 | tr|Q6FI97|Q6FI97_HUMAN | BAF53A protein OS=Homo sapiens GN=BAF53A PE=2 SV=1 | 47380.5 | 429 | 23.31 | 6 | 6 | 36 | 36 |
| 1076 | 9.46 | sp|P41227|NAA10_HUMAN | N-alpha-acetyltransferase 10 OS=Homo sapiens GN=NAA10 PE=1 SV=1 | 26458.3 | 235 | 25.53 | 5 | 5 | 17 | 17 |
| 1077 | 9.44 | sp|Q06124|PTN11_HUMAN | Tyrosine-protein phosphatase non-receptor type 11 OS=Homo sapiens GN=PTPN11 PE=1 SV=2 | 68436 | 597 | 9.548 | 5 | 5 | 13 | 13 |
| 1078 | 9.44 | tr|Q5T6L4|Q5T6L4_HUMAN | Argininosuccinate synthase 1 isoform 1 OS=Homo sapiens GN=ASS1 PE=2 SV=1 | 46530.1 | 412 | 9.951 | 5 | 5 | 17 | 17 |
| 1079 | 9.42 | tr|Q6IB11|Q6IB11_HUMAN | PGRMC1 protein OS=Homo sapiens GN=PGRMC1 PE=2 SV=1 | 21670.9 | 195 | 18.97 | 5 | 4 | 40 | 39 |
| 1080 | 9.4 | sp|P14324|FPPS_HUMAN | Farnesyl pyrophosphate synthase OS=Homo sapiens GN=FDPS PE=1 SV=4 | 48275 | 419 | 20.29 | 8 | 8 | 24 | 24 |
| 1081 | 9.38 | sp|Q99447|PCY2_HUMAN | Ethanolamine-phosphate cytidylyltransferase OS=Homo sapiens GN=PCYT2 PE=1 SV=1 | 43835.2 | 389 | 16.71 | 5 | 5 | 22 | 22 |
| 1082 | 9.38 | tr|C9JEJ2|C9JEJ2_HUMAN | Choline-phosphate cytidylyltransferase A OS=Homo sapiens GN=PCYT1A PE=1 SV=1 | 43263.6 | 380 | 16.32 | 5 | 5 | 8 | 8 |
| 1083 | 9.38 | sp|Q6UN15|FIP1_HUMAN | Pre-mRNA 3'-end-processing factor FIP1 OS=Homo sapiens GN=FIP1L1 PE=1 SV=1 | 66525.8 | 594 | 12.79 | 5 | 5 | 18 | 18 |
| 1084 | 9.36 | sp|Q9NT62|ATG3_HUMAN | Ubiquitin-like-conjugating enzyme ATG3 OS=Homo sapiens GN=ATG3 PE=1 SV=1 | 35864.2 | 314 | 12.74 | 5 | 5 | 22 | 22 |
| 1085 | 9.34 | tr|Q2LE71|Q2LE71_HUMAN | Actin-related protein 2/3 complex subunit 3 OS=Homo sapiens GN=ARPC3 PE=2 SV=1 | 20564.5 | 178 | 31.46 | 5 | 5 | 12 | 12 |
| 1086 | 9.31 | tr|A8K5D5|A8K5D5_HUMAN | cDNA FLJ76832, highly similar to Homo sapiens mitochondrial ribosomal protein L19 (MRPL19), mRNA OS=Homo sapiens PE=2 SV=1 | 32380.3 | 280 | 41.43 | 8 | 8 | 27 | 27 |
| 1087 | 9.3 | tr|H0YBR2|H0YBR2_HUMAN | Epithelial-splicing regulatory protein 1 (Fragment) OS=Homo sapiens GN=ESRP1 PE=1 SV=1 | 56091.8 | 502 | 11.95 | 6 | 5 | 25 | 22 |
| 1088 | 9.3 | tr|A0A140VJG8|A0A140VJG8_HUMAN | Testicular tissue protein Li 42 OS=Homo sapiens PE=2 SV=1 | 30036.8 | 271 | 30.63 | 7 | 7 | 59 | 59 |
| 1089 | 9.3 | tr|B4DP28|B4DP28_HUMAN | cDNA FLJ51260, highly similar to DNA polymerase subunit delta 2 (EC 2.7.7.7) OS=Homo sapiens PE=2 SV=1 | 47171.4 | 435 | 15.63 | 5 | 5 | 8 | 7 |
| 1090 | 9.29 | tr|A0A140VJZ4|A0A140VJZ4_HUMAN | Ubiquitin carboxyl-terminal hydrolase OS=Homo sapiens PE=2 SV=1 | 26182.4 | 230 | 23.91 | 5 | 5 | 36 | 36 |
| 1091 | 9.28 | tr|A0A024R912|A0A024R912_HUMAN | Uridine kinase OS=Homo sapiens GN=UCK2 PE=3 SV=1 | 29298.9 | 261 | 30.27 | 5 | 5 | 12 | 12 |
| 1092 | 9.27 | tr|B4DLT2|B4DLT2_HUMAN | cDNA FLJ56637, highly similar to Nuclear pore complex protein Nup155 OS=Homo sapiens PE=2 SV=1 | 151232.2 | 1353 | 6.208 | 5 | 5 | 12 | 12 |
| 1093 | 9.26 | tr|B4E261|B4E261_HUMAN | cDNA FLJ55646, highly similar to Adapter-relatedprotein complex 2 beta-1 subunit OS=Homo sapiens PE=2 SV=1 | 76243.1 | 688 | 14.39 | 9 | 7 | 38 | 23 |
| 1094 | 9.25 | tr|V9HW51|V9HW51_HUMAN | Epididymis secretory protein Li 114 OS=Homo sapiens GN=HEL-S-114 PE=2 SV=1 | 32251.1 | 289 | 30.1 | 7 | 7 | 26 | 26 |
| 1095 | 9.25 | sp|P05204|HMGN2_HUMAN | Non-histone chromosomal protein HMG-17 OS=Homo sapiens GN=HMGN2 PE=1 SV=3 | 9392.6 | 90 | 51.11 | 5 | 5 | 9 | 9 |
| 1096 | 9.24 | tr|A0A0S2Z693|A0A0S2Z693_HUMAN | Methylcrotonoyl-CoA carboxylase 1 isoform 1 (Fragment) OS=Homo sapiens GN=MCCC1 PE=2 SV=1 | 80472.4 | 725 | 8.414 | 5 | 5 | 8 | 8 |
| 1097 | 9.22 | tr|Q5W9G0|Q5W9G0_HUMAN | KIAA0638 splice variant 2 (Fragment) OS=Homo sapiens GN=KIAA0638 PE=2 SV=1 | 124522 | 1125 | 5.778 | 5 | 5 | 14 | 14 |
| 1098 | 9.21 | tr|Q6FHC3|Q6FHC3_HUMAN | Calponin (Fragment) OS=Homo sapiens GN=CNN2 PE=2 SV=1 | 33728.9 | 309 | 17.15 | 5 | 5 | 28 | 28 |
| 1099 | 9.21 | tr|C9J0K6|C9J0K6_HUMAN | Sorcin OS=Homo sapiens GN=SRI PE=1 SV=1 | 17604.8 | 155 | 24.52 | 5 | 5 | 15 | 15 |
| 1100 | 9.19 | sp|Q9UHG3|PCYOX_HUMAN | Prenylcysteine oxidase 1 OS=Homo sapiens GN=PCYOX1 PE=1 SV=3 | 56639.7 | 505 | 12.87 | 5 | 5 | 14 | 14 |
| 1101 | 9.19 | tr|A0A024R883|A0A024R883_HUMAN | ATPase, H+ transporting, lysosomal 13kDa, V1 subunit G1, isoform CRA_a OS=Homo sapiens GN=ATP6V1G1 PE=4 SV=1 | 13757.4 | 118 | 33.9 | 6 | 6 | 17 | 16 |
| 1102 | 9.18 | tr|A8K964|A8K964_HUMAN | cDNA FLJ75071, highly similar to Homo sapiens pinin, desmosome associated protein (PNN), mRNA OS=Homo sapiens PE=2 SV=1 | 81541.1 | 717 | 10.46 | 6 | 6 | 15 | 15 |
| 1103 | 9.18 | sp|A5YKK6|CNOT1_HUMAN | CCR4-NOT transcription complex subunit 1 OS=Homo sapiens GN=CNOT1 PE=1 SV=2 | 266936.5 | 2376 | 3.157 | 7 | 7 | 15 | 15 |
| 1104 | 9.17 | tr|Q53RU4|Q53RU4_HUMAN | Putative uncharacterized protein MSH2 (Fragment) OS=Homo sapiens GN=MSH2 PE=4 SV=1 | 66275.1 | 586 | 15.36 | 8 | 8 | 14 | 14 |
| 1105 | 9.16 | tr|Q6IBK5|Q6IBK5_HUMAN | GTF2F1 protein OS=Homo sapiens GN=GTF2F1 PE=2 SV=1 | 58226 | 517 | 12.38 | 5 | 5 | 7 | 7 |
| 1106 | 9.16 | tr|B4E2T6|B4E2T6_HUMAN | cDNA FLJ58231, highly similar to NMDA receptor-regulated protein 1 OS=Homo sapiens PE=2 SV=1 | 86540.3 | 740 | 7.568 | 5 | 5 | 15 | 15 |
| 1107 | 9.15 | tr|B4E0H8|B4E0H8_HUMAN | cDNA FLJ60385, highly similar to Integrin alpha-3 OS=Homo sapiens PE=2 SV=1 | 115166.6 | 1037 | 6.557 | 5 | 5 | 17 | 17 |
| 1108 | 9.14 | tr|Q53XL8|Q53XL8_HUMAN | Proteasome (Prosome, macropain) 26S subunit, ATPase, 1 OS=Homo sapiens GN=PSMC1 PE=2 SV=1 | 49184.1 | 440 | 29.32 | 9 | 8 | 32 | 16 |
| 1109 | 9.14 | sp|Q9Y6M9|NDUB9_HUMAN | NADH dehydrogenase [ubiquinone] 1 beta subcomplex subunit 9 OS=Homo sapiens GN=NDUFB9 PE=1 SV=3 | 21830.7 | 179 | 31.84 | 5 | 5 | 16 | 16 |
| 1110 | 9.14 | tr|Q76LA1|Q76LA1_HUMAN | CSTB protein OS=Homo sapiens GN=CSTB PE=2 SV=1 | 11139.6 | 98 | 67.35 | 4 | 4 | 101 | 99 |
| 1111 | 9.14 | tr|A8K761|A8K761_HUMAN | NADH dehydrogenase (Ubiquinone) 1 beta subcomplex, 10, 22kDa, isoform CRA_b OS=Homo sapiens GN=NDUFB10 PE=2 SV=1 | 20776.5 | 172 | 38.37 | 5 | 5 | 10 | 10 |
| 1112 | 9.13 | tr|A0A140VKE9|A0A140VKE9_HUMAN | Testis tissue sperm-binding protein Li 66n OS=Homo sapiens PE=2 SV=1 | 71456.1 | 638 | 10.82 | 5 | 5 | 17 | 17 |
| 1113 | 9.13 | tr|V9HW90|V9HW90_HUMAN | Epididymis luminal protein 75 OS=Homo sapiens GN=HEL-75 PE=2 SV=1 | 56256.6 | 522 | 15.13 | 5 | 5 | 13 | 13 |
| 1114 | 9.13 | tr|Q6FI51|Q6FI51_HUMAN | DNAJB1 protein OS=Homo sapiens GN=DNAJB1 PE=2 SV=1 | 38009.8 | 340 | 21.18 | 6 | 6 | 20 | 20 |
| 1115 | 9.13 | sp|Q14376|GALE_HUMAN | UDP-glucose 4-epimerase OS=Homo sapiens GN=GALE PE=1 SV=2 | 38281.4 | 348 | 12.64 | 5 | 5 | 14 | 14 |
| 1116 | 9.11 | tr|B4DGM3|B4DGM3_HUMAN | SWI/SNF-related matrix-associated actin-dependent regulator of chromatin subfamily E member 1 OS=Homo sapiens GN=SMARCE1 PE=1 SV=1 | 44770 | 393 | 16.54 | 5 | 5 | 20 | 20 |
| 1117 | 9.1 | tr|A0A024R3V0|A0A024R3V0_HUMAN | DNA polymerase-transactivated protein 6, isoform CRA_a OS=Homo sapiens GN=DNAPTP6 PE=4 SV=1 | 61728.6 | 558 | 11.29 | 5 | 5 | 6 | 6 |
| 1118 | 9.09 | tr|A0A140VK12|A0A140VK12_HUMAN | Coatomer subunit gamma OS=Homo sapiens PE=2 SV=1 | 97621.3 | 871 | 9.185 | 7 | 5 | 21 | 15 |
| 1119 | 9.04 | tr|Q59GX2|Q59GX2_HUMAN | Solute carrier family 2 (Facilitated glucose transporter), member 1 variant (Fragment) OS=Homo sapiens PE=2 SV=1 | 57017.5 | 517 | 11.99 | 5 | 5 | 33 | 33 |
| 1120 | 9.04 | sp|Q01844|EWS_HUMAN | RNA-binding protein EWS OS=Homo sapiens GN=EWSR1 PE=1 SV=1 | 68478.2 | 656 | 14.79 | 6 | 6 | 36 | 36 |
| 1121 | 9.03 | tr|Q549N5|Q549N5_HUMAN | Signal recognition particle receptor beta subunit OS=Homo sapiens GN=SRPRB PE=2 SV=1 | 29701.9 | 271 | 20.3 | 6 | 5 | 22 | 21 |
| 1122 | 9.03 | sp|P62318|SMD3_HUMAN | Small nuclear ribonucleoprotein Sm D3 OS=Homo sapiens GN=SNRPD3 PE=1 SV=1 | 13916.2 | 126 | 42.06 | 5 | 5 | 28 | 28 |
| 1123 | 9.03 | sp|P60866|RS20_HUMAN | 40S ribosomal protein S20 OS=Homo sapiens GN=RPS20 PE=1 SV=1 | 13372.6 | 119 | 30.25 | 5 | 5 | 55 | 54 |
| 1124 | 9.01 | tr|A0A024R7M8|A0A024R7M8_HUMAN | Uncharacterized protein OS=Homo sapiens GN=LOC115098 PE=4 SV=1 | 25835 | 223 | 27.35 | 5 | 5 | 26 | 25 |
| 1125 | 9 | tr|J3KQ32|J3KQ32_HUMAN | Obg-like ATPase 1 OS=Homo sapiens GN=OLA1 PE=1 SV=1 | 46937.7 | 416 | 12.98 | 5 | 5 | 20 | 20 |
| 1126 | 8.98 | tr|F1T0A5|F1T0A5_HUMAN | PRP31 pre-mRNA processing factor 31 homolog (Yeast), isoform CRA_a OS=Homo sapiens GN=PRPF31 PE=2 SV=1 | 55455.6 | 499 | 17.23 | 6 | 6 | 28 | 28 |
| 1127 | 8.97 | sp|Q14696|MESD_HUMAN | LDLR chaperone MESD OS=Homo sapiens GN=MESDC2 PE=1 SV=2 | 26076.4 | 234 | 24.79 | 6 | 4 | 27 | 8 |
| 1128 | 8.97 | tr|D3DWB6|D3DWB6_HUMAN | Ubiquitin specific peptidase 9, X-linked, isoform CRA_b OS=Homo sapiens GN=USP9X PE=3 SV=1 | 271251.8 | 2379 | 2.942 | 6 | 5 | 8 | 6 |
| 1129 | 8.96 | sp|P16402|H13_HUMAN | Histone H1.3 OS=Homo sapiens GN=HIST1H1D PE=1 SV=2 | 22349.7 | 221 | 48.42 | 22 | 4 | 387 | 42 |
| 1130 | 8.95 | tr|E9PMD7|E9PMD7_HUMAN | Serine/threonine-protein phosphatase (Fragment) OS=Homo sapiens GN=PPP1CA PE=1 SV=1 | 28897.6 | 253 | 23.72 | 5 | 3 | 20 | 18 |
| 1131 | 8.95 | sp|Q13131|AAPK1_HUMAN | 5'-AMP-activated protein kinase catalytic subunit alpha-1 OS=Homo sapiens GN=PRKAA1 PE=1 SV=4 | 64008.6 | 559 | 9.839 | 5 | 5 | 15 | 15 |
| 1132 | 8.93 | tr|A4FTV9|A4FTV9_HUMAN | Histone H2A OS=Homo sapiens GN=HIST1H2AK PE=2 SV=1 | 14091.4 | 130 | 46.15 | 11 | 4 | 152 | 35 |
| 1133 | 8.92 | tr|A0A0S2Z4J6|A0A0S2Z4J6_HUMAN | Phosphomannomutase (Fragment) OS=Homo sapiens GN=PMM2 PE=2 SV=1 | 28081.9 | 246 | 21.95 | 5 | 5 | 22 | 22 |
| 1134 | 8.91 | tr|E9PK09|E9PK09_HUMAN | Bcl-2-associated transcription factor 1 (Fragment) OS=Homo sapiens GN=BCLAF1 PE=1 SV=7 | 83231.5 | 726 | 7.713 | 7 | 6 | 17 | 16 |
| 1135 | 8.89 | tr|Q5VSQ6|Q5VSQ6_HUMAN | Procollagen-proline, 2-oxoglutarate 4-dioxygenase (Proline 4-hydroxylase), alpha polypeptide I variant (Fragment) OS=Homo sapiens GN=P4HA1 PE=2 SV=1 | 60966.6 | 534 | 11.61 | 5 | 5 | 14 | 14 |
| 1136 | 8.87 | tr|B2R7B5|B2R7B5_HUMAN | cDNA, FLJ93365, highly similar to Homo sapiens KH domain containing, RNA binding, signal transduction associated 1 (KHDRBS1), mRNA OS=Homo sapiens PE=2 SV=1 | 48225 | 443 | 16.7 | 5 | 5 | 50 | 50 |
| 1137 | 8.85 | sp|P49792|RBP2_HUMAN | E3 SUMO-protein ligase RanBP2 OS=Homo sapiens GN=RANBP2 PE=1 SV=2 | 358196.4 | 3224 | 5.056 | 12 | 10 | 21 | 17 |
| 1138 | 8.84 | tr|Q96HI4|Q96HI4_HUMAN | Glycylpeptide N-tetradecanoyltransferase (Fragment) OS=Homo sapiens GN=NMT1 PE=2 SV=2 | 56603.6 | 494 | 8.502 | 4 | 4 | 40 | 40 |
| 1139 | 8.84 | tr|K7EQZ3|K7EQZ3_HUMAN | Uncharacterized protein (Fragment) OS=Homo sapiens PE=4 SV=1 | 16378.2 | 153 | 32.03 | 5 | 1 | 16 | 2 |
| 1140 | 8.83 | tr|A8KA74|A8KA74_HUMAN | cDNA FLJ76065 OS=Homo sapiens PE=2 SV=1 | 97669.5 | 857 | 7.585 | 5 | 5 | 17 | 17 |
| 1141 | 8.82 | tr|A0A024RBR1|A0A024RBR1_HUMAN | Restin (Reed-Steinberg cell-expressed intermediate filament-associated protein), isoform CRA_b OS=Homo sapiens GN=RSN PE=4 SV=1 | 160988.7 | 1427 | 6.377 | 9 | 8 | 18 | 13 |
| 1142 | 8.82 | tr|B2RNR6|B2RNR6_HUMAN | Zinc finger RNA binding protein OS=Homo sapiens GN=ZFR PE=2 SV=1 | 117013.1 | 1074 | 7.169 | 5 | 5 | 15 | 15 |
| 1143 | 8.82 | tr|A0A024RB22|A0A024RB22_HUMAN | SWI/SNF related, matrix associated, actin dependent regulator of chromatin, subfamily c, member 2, isoform CRA_a OS=Homo sapiens GN=SMARCC2 PE=4 SV=1 | 132878.5 | 1214 | 6.919 | 8 | 6 | 14 | 11 |
| 1144 | 8.81 | sp|O75436|VP26A_HUMAN | Vacuolar protein sorting-associated protein 26A OS=Homo sapiens GN=VPS26A PE=1 SV=2 | 38169.7 | 327 | 24.77 | 5 | 5 | 34 | 34 |
| 1145 | 8.79 | tr|Q6IBR8|Q6IBR8_HUMAN | EIF2S2 protein OS=Homo sapiens GN=EIF2S2 PE=2 SV=1 | 38388.1 | 333 | 15.02 | 6 | 6 | 13 | 13 |
| 1146 | 8.79 | sp|Q96EY8|MMAB_HUMAN | Cob(I)yrinic acid a,c-diamide adenosyltransferase, mitochondrial OS=Homo sapiens GN=MMAB PE=1 SV=1 | 27388 | 250 | 25.6 | 5 | 5 | 10 | 10 |
| 1147 | 8.78 | tr|V9HWH9|V9HWH9_HUMAN | Protein S100 OS=Homo sapiens GN=HEL-S-43 PE=2 SV=1 | 11740.3 | 105 | 49.52 | 5 | 5 | 16 | 16 |
| 1148 | 8.76 | tr|B2R7W3|B2R7W3_HUMAN | Breast carcinoma amplified sequence 2 OS=Homo sapiens GN=BCAS2 PE=2 SV=1 | 26131.3 | 225 | 20.89 | 4 | 4 | 12 | 12 |
| 1149 | 8.76 | sp|Q15738|NSDHL_HUMAN | Sterol-4-alpha-carboxylate 3-dehydrogenase, decarboxylating OS=Homo sapiens GN=NSDHL PE=1 SV=2 | 41900 | 373 | 16.89 | 5 | 5 | 16 | 16 |
| 1150 | 8.75 | tr|B4E0R6|B4E0R6_HUMAN | cDNA FLJ54573, highly similar to Importin beta-3 OS=Homo sapiens PE=2 SV=1 | 109357.7 | 972 | 9.362 | 6 | 6 | 20 | 20 |
| 1151 | 8.74 | tr|B7ZLP7|B7ZLP7_HUMAN | RNA binding motif protein 47 OS=Homo sapiens GN=RBM47 PE=1 SV=1 | 64098.6 | 593 | 13.32 | 5 | 5 | 19 | 19 |
| 1152 | 8.74 | tr|V9HW41|V9HW41_HUMAN | Epididymis secretory protein Li 71 OS=Homo sapiens GN=HEL-S-71 PE=2 SV=1 | 17137.6 | 152 | 47.37 | 6 | 6 | 82 | 82 |
| 1153 | 8.73 | sp|P42166|LAP2A_HUMAN | Lamina-associated polypeptide 2, isoform alpha OS=Homo sapiens GN=TMPO PE=1 SV=2 | 75491.3 | 694 | 25.36 | 13 | 5 | 92 | 20 |
| 1154 | 8.71 | sp|Q13596|SNX1_HUMAN | Sorting nexin-1 OS=Homo sapiens GN=SNX1 PE=1 SV=3 | 59069 | 522 | 11.88 | 5 | 4 | 22 | 19 |
| 1155 | 8.71 | sp|P51571|SSRD_HUMAN | Translocon-associated protein subunit delta OS=Homo sapiens GN=SSR4 PE=1 SV=1 | 18998.4 | 173 | 30.64 | 4 | 4 | 39 | 38 |
| 1156 | 8.67 | tr|Q53FG3|Q53FG3_HUMAN | Interleukin enhancer binding factor 2 variant (Fragment) OS=Homo sapiens PE=2 SV=1 | 43047.7 | 390 | 17.69 | 5 | 5 | 71 | 70 |
| 1157 | 8.66 | sp|Q14258|TRI25_HUMAN | E3 ubiquitin/ISG15 ligase TRIM25 OS=Homo sapiens GN=TRIM25 PE=1 SV=2 | 70972.8 | 630 | 10.32 | 5 | 5 | 24 | 24 |
| 1158 | 8.64 | sp|Q02338|BDH_HUMAN | D-beta-hydroxybutyrate dehydrogenase, mitochondrial OS=Homo sapiens GN=BDH1 PE=1 SV=3 | 38156.8 | 343 | 16.03 | 4 | 4 | 11 | 10 |
| 1159 | 8.62 | tr|A0A0F7KYT8|A0A0F7KYT8_HUMAN | Fragile X mental retardation autosomal homolog variant p2K OS=Homo sapiens GN=FXR1 PE=2 SV=1 | 76199.3 | 677 | 9.453 | 5 | 4 | 24 | 15 |
| 1160 | 8.6 | tr|A0A024R753|A0A024R753_HUMAN | RNA binding motif protein 28 isoform 1 OS=Homo sapiens GN=RBM28 PE=2 SV=1 | 85737.1 | 759 | 9.354 | 5 | 5 | 18 | 18 |
| 1161 | 8.6 | tr|Q499G7|Q499G7_HUMAN | Mitogen-activated protein kinase OS=Homo sapiens GN=MAPK1 PE=2 SV=1 | 41330.1 | 360 | 14.72 | 4 | 4 | 19 | 15 |
| 1162 | 8.6 | tr|Q6IRT1|Q6IRT1_HUMAN | S-(hydroxymethyl)glutathione dehydrogenase OS=Homo sapiens GN=ADH5 PE=2 SV=2 | 39723.9 | 374 | 17.38 | 6 | 6 | 12 | 12 |
| 1163 | 8.59 | sp|Q96QK1|VPS35_HUMAN | Vacuolar protein sorting-associated protein 35 OS=Homo sapiens GN=VPS35 PE=1 SV=2 | 91706.2 | 796 | 9.171 | 5 | 5 | 21 | 21 |
| 1164 | 8.59 | sp|P27144|KAD4_HUMAN | Adenylate kinase 4, mitochondrial OS=Homo sapiens GN=AK4 PE=1 SV=1 | 25267.8 | 223 | 34.53 | 5 | 5 | 18 | 18 |
| 1165 | 8.56 | tr|A0A087WTA5|A0A087WTA5_HUMAN | Translation initiation factor eIF-2B subunit delta OS=Homo sapiens GN=EIF2B4 PE=1 SV=1 | 57495.6 | 520 | 10 | 4 | 4 | 14 | 14 |
| 1166 | 8.56 | sp|Q9NWH9|SLTM_HUMAN | SAFB-like transcription modulator OS=Homo sapiens GN=SLTM PE=1 SV=2 | 117147.4 | 1034 | 5.126 | 4 | 4 | 13 | 13 |
| 1167 | 8.56 | sp|Q9NP79|VTA1_HUMAN | Vacuolar protein sorting-associated protein VTA1 homolog OS=Homo sapiens GN=VTA1 PE=1 SV=1 | 33878.9 | 307 | 19.87 | 5 | 5 | 15 | 15 |
| 1168 | 8.54 | sp|Q9HA77|SYCM_HUMAN | Probable cysteine--tRNA ligase, mitochondrial OS=Homo sapiens GN=CARS2 PE=1 SV=1 | 62223.3 | 564 | 7.979 | 4 | 4 | 18 | 18 |
| 1169 | 8.54 | sp|Q14320|FA50A_HUMAN | Protein FAM50A OS=Homo sapiens GN=FAM50A PE=1 SV=2 | 40241.3 | 339 | 14.75 | 5 | 5 | 25 | 24 |
| 1170 | 8.54 | tr|I4AY87|I4AY87_HUMAN | Macrophage migration inhibitory factor (Fragment) OS=Homo sapiens GN=MIF PE=1 SV=1 | 12476.2 | 115 | 74.78 | 5 | 5 | 149 | 149 |
| 1171 | 8.52 | tr|Q53HL1|Q53HL1_HUMAN | Myosin regulatory light chain MRCL3 variant (Fragment) OS=Homo sapiens PE=2 SV=1 | 19793 | 171 | 23.98 | 5 | 5 | 49 | 49 |
| 1172 | 8.51 | tr|A0A024R9D7|A0A024R9D7_HUMAN | 2,4-dienoyl CoA reductase 1, mitochondrial, isoform CRA_b OS=Homo sapiens GN=DECR1 PE=4 SV=1 | 36067.4 | 335 | 17.01 | 5 | 5 | 17 | 17 |
| 1173 | 8.51 | tr|V9HWH6|V9HWH6_HUMAN | Purine nucleoside phosphorylase OS=Homo sapiens GN=HEL-S-156an PE=2 SV=1 | 32117.7 | 289 | 24.57 | 7 | 7 | 51 | 50 |
| 1174 | 8.5 | tr|E9PKP7|E9PKP7_HUMAN | Nucleolar transcription factor 1 OS=Homo sapiens GN=UBTF PE=1 SV=1 | 87435.8 | 745 | 8.591 | 5 | 5 | 17 | 17 |
| 1175 | 8.49 | sp|Q9UBS4|DJB11_HUMAN | DnaJ homolog subfamily B member 11 OS=Homo sapiens GN=DNAJB11 PE=1 SV=1 | 40513.7 | 358 | 13.13 | 4 | 4 | 7 | 7 |
| 1176 | 8.48 | tr|B0LPF3|B0LPF3_HUMAN | Growth factor receptor-bound protein 2 OS=Homo sapiens GN=GRB2 PE=2 SV=1 | 25206.2 | 217 | 28.57 | 6 | 6 | 19 | 19 |
| 1177 | 8.47 | sp|Q9H0U4|RAB1B_HUMAN | Ras-related protein Rab-1B OS=Homo sapiens GN=RAB1B PE=1 SV=1 | 22171 | 201 | 72.14 | 14 | 4 | 188 | 25 |
| 1178 | 8.47 | tr|I6L9H2|I6L9H2_HUMAN | DNA (cytosine-5)-methyltransferase OS=Homo sapiens GN=DNMT1 PE=2 SV=1 | 171071.9 | 1511 | 3.309 | 5 | 5 | 9 | 9 |
| 1179 | 8.45 | sp|P11233|RALA_HUMAN | Ras-related protein Ral-A OS=Homo sapiens GN=RALA PE=1 SV=1 | 23566.6 | 206 | 22.33 | 4 | 2 | 30 | 11 |
| 1180 | 8.43 | tr|Q15182|Q15182_HUMAN | Small nuclear ribonucleoprotein-associated protein OS=Homo sapiens GN=SNRPB PE=2 SV=1 | 29670.7 | 285 | 18.95 | 6 | 6 | 19 | 19 |
| 1181 | 8.43 | tr|A0A024R8P8|A0A024R8P8_HUMAN | Ribosomal protein L38, isoform CRA_a OS=Homo sapiens GN=RPL38 PE=3 SV=1 | 8217.8 | 70 | 55.71 | 5 | 5 | 67 | 67 |
| 1182 | 8.42 | tr|D3DWY7|D3DWY7_HUMAN | von Hippel-Lindau binding protein 1, isoform CRA_b OS=Homo sapiens GN=VBP1 PE=4 SV=1 | 26535.1 | 233 | 18.03 | 6 | 5 | 25 | 23 |
| 1183 | 8.42 | tr|Q7L4Q3|Q7L4Q3_HUMAN | Glutathione peroxidase OS=Homo sapiens GN=GPX1 PE=2 SV=1 | 22087.9 | 203 | 30.54 | 5 | 5 | 17 | 17 |
| 1184 | 8.41 | sp|P61163|ACTZ_HUMAN | Alpha-centractin OS=Homo sapiens GN=ACTR1A PE=1 SV=1 | 42613.3 | 376 | 25 | 5 | 5 | 28 | 28 |
| 1185 | 8.4 | tr|Q8N2L6|Q8N2L6_HUMAN | cDNA FLJ90138 fis, clone HEMBB1000905, weakly similar to TRANSCRIPTIONAL REPRESSOR RCO-1 OS=Homo sapiens PE=2 SV=1 | 49823.5 | 447 | 9.843 | 4 | 4 | 12 | 11 |
| 1186 | 8.4 | tr|V9HW21|V9HW21_HUMAN | Epididymis luminal protein 76 OS=Homo sapiens GN=HEL-76 PE=2 SV=1 | 29245.9 | 260 | 19.23 | 4 | 4 | 16 | 16 |
| 1187 | 8.39 | tr|Q05DB4|Q05DB4_HUMAN | HEBP2 protein (Fragment) OS=Homo sapiens GN=HEBP2 PE=2 SV=1 | 24028.8 | 214 | 27.57 | 5 | 5 | 13 | 13 |
| 1188 | 8.38 | tr|B3KMV8|B3KMV8_HUMAN | cDNA FLJ12766 fis, clone NT2RP2001520, highly similar to Calcium-binding mitochondrial carrier protein Aralar1 OS=Homo sapiens PE=2 SV=1 | 74777.2 | 678 | 9.145 | 5 | 4 | 15 | 10 |
| 1189 | 8.38 | sp|Q5QJE6|TDIF2_HUMAN | Deoxynucleotidyltransferase terminal-interacting protein 2 OS=Homo sapiens GN=DNTTIP2 PE=1 SV=2 | 84468.3 | 756 | 10.85 | 5 | 5 | 14 | 14 |
| 1190 | 8.37 | sp|Q0VDF9|HSP7E_HUMAN | Heat shock 70 kDa protein 14 OS=Homo sapiens GN=HSPA14 PE=1 SV=1 | 54793.8 | 509 | 16.11 | 5 | 5 | 6 | 6 |
| 1191 | 8.36 | sp|P50402|EMD_HUMAN | Emerin OS=Homo sapiens GN=EMD PE=1 SV=1 | 28993.5 | 254 | 25.98 | 6 | 6 | 22 | 22 |
| 1192 | 8.35 | sp|O75976|CBPD_HUMAN | Carboxypeptidase D OS=Homo sapiens GN=CPD PE=1 SV=2 | 152929.9 | 1380 | 5.652 | 6 | 6 | 7 | 7 |
| 1193 | 8.34 | tr|B2RBL9|B2RBL9_HUMAN | cDNA, FLJ95582, highly similar to Homo sapiens breast cancer anti-estrogen resistance 1 (BCAR1),mRNA OS=Homo sapiens PE=2 SV=1 | 93404.1 | 870 | 10.46 | 5 | 5 | 12 | 12 |
| 1194 | 8.34 | tr|Q5DT02|Q5DT02_HUMAN | UDP-glucuronosyltransferase OS=Homo sapiens GN=UGT1A10 PE=2 SV=1 | 59809.1 | 530 | 15.47 | 6 | 3 | 32 | 6 |
| 1195 | 8.34 | sp|Q01780|EXOSX_HUMAN | Exosome component 10 OS=Homo sapiens GN=EXOSC10 PE=1 SV=2 | 100830.3 | 885 | 9.379 | 5 | 5 | 7 | 7 |
| 1196 | 8.34 | sp|O95721|SNP29_HUMAN | Synaptosomal-associated protein 29 OS=Homo sapiens GN=SNAP29 PE=1 SV=1 | 28970 | 258 | 22.09 | 4 | 4 | 13 | 13 |
| 1197 | 8.32 | tr|A0A024R4M8|A0A024R4M8_HUMAN | Retinol dehydrogenase 13 (All-trans and 9-cis), isoform CRA_a OS=Homo sapiens GN=RDH13 PE=3 SV=1 | 35931.8 | 331 | 19.03 | 5 | 5 | 6 | 6 |
| 1198 | 8.31 | sp|P24928|RPB1_HUMAN | DNA-directed RNA polymerase II subunit RPB1 OS=Homo sapiens GN=POLR2A PE=1 SV=2 | 217174.2 | 1970 | 6.091 | 7 | 7 | 11 | 11 |
| 1199 | 8.3 | tr|B3KPQ5|B3KPQ5_HUMAN | cDNA FLJ32057 fis, clone NTONG2001642, highly similar to Exportin-5 OS=Homo sapiens PE=2 SV=1 | 94038 | 832 | 6.25 | 5 | 5 | 7 | 7 |
| 1200 | 8.27 | sp|Q93009|UBP7_HUMAN | Ubiquitin carboxyl-terminal hydrolase 7 OS=Homo sapiens GN=USP7 PE=1 SV=2 | 128301.3 | 1102 | 6.261 | 6 | 6 | 11 | 11 |
| 1201 | 8.27 | tr|A0A024R6T8|A0A024R6T8_HUMAN | NEFA-interacting nuclear protein NIP30, isoform CRA_a OS=Homo sapiens GN=NIP30 PE=4 SV=1 | 28911.9 | 254 | 20.08 | 5 | 5 | 15 | 15 |
| 1202 | 8.26 | tr|A0A140VJR2|A0A140VJR2_HUMAN | Testicular tissue protein Li 138 OS=Homo sapiens PE=2 SV=1 | 54547.8 | 493 | 7.708 | 4 | 4 | 12 | 12 |
| 1203 | 8.25 | tr|A0A087WXS7|A0A087WXS7_HUMAN | ATPase ASNA1 OS=Homo sapiens GN=ASNA1 PE=1 SV=1 | 37118.7 | 331 | 24.17 | 6 | 6 | 14 | 13 |
| 1204 | 8.24 | tr|V9HWJ8|V9HWJ8_HUMAN | Epididymis secretory protein Li 283 OS=Homo sapiens GN=HEL-S-283 PE=2 SV=1 | 29505.8 | 254 | 24.8 | 6 | 6 | 51 | 51 |
| 1205 | 8.24 | sp|Q9UHB9|SRP68_HUMAN | Signal recognition particle subunit SRP68 OS=Homo sapiens GN=SRP68 PE=1 SV=2 | 70729 | 627 | 8.772 | 4 | 4 | 11 | 11 |
| 1206 | 8.23 | tr|A0A024R598|A0A024R598_HUMAN | Uncharacterized protein OS=Homo sapiens GN=LOC51035 PE=4 SV=1 | 33325 | 297 | 20.2 | 4 | 4 | 20 | 20 |
| 1207 | 8.23 | sp|Q14694|UBP10_HUMAN | Ubiquitin carboxyl-terminal hydrolase 10 OS=Homo sapiens GN=USP10 PE=1 SV=2 | 87133.1 | 798 | 8.145 | 5 | 5 | 14 | 13 |
| 1208 | 8.23 | sp|Q9NPJ3|ACO13_HUMAN | Acyl-coenzyme A thioesterase 13 OS=Homo sapiens GN=ACOT13 PE=1 SV=1 | 14960.4 | 140 | 37.86 | 4 | 4 | 14 | 14 |
| 1209 | 8.22 | tr|Q53GW1|Q53GW1_HUMAN | Vesicle transport-related protein isoform a variant (Fragment) OS=Homo sapiens PE=2 SV=1 | 72307.1 | 642 | 11.53 | 5 | 5 | 7 | 7 |
| 1210 | 8.22 | sp|Q9NVM6|DJC17_HUMAN | DnaJ homolog subfamily C member 17 OS=Homo sapiens GN=DNAJC17 PE=1 SV=1 | 34687.1 | 304 | 16.78 | 4 | 4 | 9 | 9 |
| 1211 | 8.2 | sp|Q9BYK8|HELZ2_HUMAN | Helicase with zinc finger domain 2 OS=Homo sapiens GN=HELZ2 PE=1 SV=6 | 294648.4 | 2649 | 2.907 | 5 | 5 | 7 | 7 |
| 1212 | 8.19 | sp|Q969X5|ERGI1_HUMAN | Endoplasmic reticulum-Golgi intermediate compartment protein 1 OS=Homo sapiens GN=ERGIC1 PE=1 SV=1 | 32592 | 290 | 14.48 | 4 | 4 | 10 | 10 |
| 1213 | 8.17 | tr|Q6DHZ8|Q6DHZ8_HUMAN | Activity-dependent neuroprotector homeobox OS=Homo sapiens GN=ADNP PE=2 SV=1 | 123445.9 | 1102 | 7.532 | 6 | 6 | 10 | 10 |
| 1214 | 8.16 | sp|P61326|MGN_HUMAN | Protein mago nashi homolog OS=Homo sapiens GN=MAGOH PE=1 SV=1 | 17163.5 | 146 | 30.82 | 4 | 4 | 17 | 17 |
| 1215 | 8.15 | sp|Q6IA69|NADE_HUMAN | Glutamine-dependent NAD(+) synthetase OS=Homo sapiens GN=NADSYN1 PE=1 SV=3 | 79283.9 | 706 | 6.374 | 4 | 4 | 14 | 14 |
| 1216 | 8.14 | sp|P78406|RAE1L_HUMAN | mRNA export factor OS=Homo sapiens GN=RAE1 PE=1 SV=1 | 40967.8 | 368 | 15.49 | 4 | 4 | 42 | 42 |
| 1217 | 8.13 | tr|D6W4Z6|D6W4Z6_HUMAN | HCG23833, isoform CRA_b OS=Homo sapiens GN=hCG_23833 PE=4 SV=1 | 51076.6 | 449 | 10.24 | 4 | 4 | 26 | 26 |
| 1218 | 8.13 | sp|P57740|NU107_HUMAN | Nuclear pore complex protein Nup107 OS=Homo sapiens GN=NUP107 PE=1 SV=1 | 106373.1 | 925 | 5.622 | 4 | 4 | 7 | 7 |
| 1219 | 8.11 | tr|B7Z920|B7Z920_HUMAN | cDNA FLJ61714, highly similar to Tripeptidyl-peptidase 2 (EC 3.4.14.10) (Fragment) OS=Homo sapiens PE=2 SV=1 | 117963.6 | 1069 | 6.268 | 4 | 4 | 9 | 9 |
| 1220 | 8.11 | sp|Q16630|CPSF6_HUMAN | Cleavage and polyadenylation specificity factor subunit 6 OS=Homo sapiens GN=CPSF6 PE=1 SV=2 | 59209.3 | 551 | 11.25 | 4 | 4 | 30 | 30 |
| 1221 | 8.1 | tr|L7RT18|L7RT18_HUMAN | V-crk sarcoma virus CT10 oncogene homolog (Avian) OS=Homo sapiens GN=CRK PE=4 SV=1 | 33830.4 | 304 | 26.97 | 6 | 4 | 21 | 17 |
| 1222 | 8.1 | sp|Q12996|CSTF3_HUMAN | Cleavage stimulation factor subunit 3 OS=Homo sapiens GN=CSTF3 PE=1 SV=1 | 82920.8 | 717 | 9.763 | 4 | 4 | 9 | 9 |
| 1223 | 8.1 | tr|A8KA84|A8KA84_HUMAN | cDNA FLJ78682, highly similar to Homo sapiens 2'-5'-oligoadenylate synthetase 3, 100kDa (OAS3), mRNA OS=Homo sapiens PE=2 SV=1 | 121210.2 | 1087 | 4.416 | 4 | 4 | 18 | 18 |
| 1224 | 8.1 | sp|O15031|PLXB2_HUMAN | Plexin-B2 OS=Homo sapiens GN=PLXNB2 PE=1 SV=3 | 205125.6 | 1838 | 2.938 | 4 | 4 | 12 | 12 |
| 1225 | 8.09 | tr|Q658U4|Q658U4_HUMAN | Putative uncharacterized protein DKFZp666D193 OS=Homo sapiens GN=DKFZp666D193 PE=2 SV=1 | 64131.4 | 582 | 8.247 | 4 | 4 | 13 | 13 |
| 1226 | 8.09 | tr|A0A087X1E4|A0A087X1E4_HUMAN | Arfaptin-2 OS=Homo sapiens GN=ARFIP2 PE=1 SV=1 | 41594.8 | 374 | 12.03 | 4 | 4 | 7 | 7 |
| 1227 | 8.08 | sp|Q9Y5B9|SP16H_HUMAN | FACT complex subunit SPT16 OS=Homo sapiens GN=SUPT16H PE=1 SV=1 | 119912.9 | 1047 | 5.349 | 5 | 4 | 18 | 12 |
| 1228 | 8.07 | tr|B4E284|B4E284_HUMAN | cDNA FLJ51188, highly similar to N-acetylglucosamine-6-sulfatase (EC3.1.6.14) OS=Homo sapiens PE=2 SV=1 | 65808.6 | 584 | 7.192 | 4 | 4 | 28 | 28 |
| 1229 | 8.07 | sp|Q52LJ0|FA98B_HUMAN | Protein FAM98B OS=Homo sapiens GN=FAM98B PE=1 SV=1 | 37190.5 | 330 | 11.82 | 4 | 4 | 22 | 22 |
| 1230 | 8.05 | sp|Q96EK5|KBP_HUMAN | KIF1-binding protein OS=Homo sapiens GN=KIF1BP PE=1 SV=1 | 71813.3 | 621 | 7.89 | 4 | 4 | 7 | 7 |
| 1231 | 8.05 | tr|Q5TZP7|Q5TZP7_HUMAN | DNA-(apurinic or apyrimidinic site) lyase OS=Homo sapiens GN=APEX1 PE=2 SV=1 | 35554.2 | 318 | 18.24 | 4 | 4 | 15 | 15 |
| 1232 | 8.05 | tr|V9HWC3|V9HWC3_HUMAN | Epididymis secretory sperm binding protein Li 1a OS=Homo sapiens GN=HEL-S-1a PE=2 SV=1 | 35102.1 | 312 | 22.12 | 4 | 4 | 11 | 11 |
| 1233 | 8.04 | sp|Q6KB66|K2C80_HUMAN | Keratin, type II cytoskeletal 80 OS=Homo sapiens GN=KRT80 PE=1 SV=2 | 50524.8 | 452 | 14.38 | 8 | 4 | 87 | 12 |
| 1234 | 8.04 | sp|Q9BYD6|RM01_HUMAN | 39S ribosomal protein L1, mitochondrial OS=Homo sapiens GN=MRPL1 PE=1 SV=2 | 36908.3 | 325 | 18.15 | 5 | 5 | 12 | 10 |
| 1235 | 8.03 | tr|A0A0U1RQZ9|A0A0U1RQZ9_HUMAN | Probable global transcription activator SNF2L2 OS=Homo sapiens GN=SMARCA2 PE=1 SV=1 | 162369.7 | 1428 | 3.291 | 4 | 4 | 12 | 12 |
| 1236 | 8.03 | tr|A0A024R578|A0A024R578_HUMAN | Mitochondrial ribosomal protein L49, isoform CRA_b OS=Homo sapiens GN=MRPL49 PE=4 SV=1 | 19197.9 | 166 | 23.49 | 4 | 4 | 21 | 21 |
| 1237 | 8.03 | tr|A0A0S2Z3C5|A0A0S2Z3C5_HUMAN | BCL2-like 1 isoform 1 (Fragment) OS=Homo sapiens GN=BCL2L1 PE=2 SV=1 | 26048.8 | 233 | 15.88 | 4 | 4 | 11 | 11 |
| 1238 | 8.03 | sp|P07108|ACBP_HUMAN | Acyl-CoA-binding protein OS=Homo sapiens GN=DBI PE=1 SV=2 | 10044.4 | 87 | 56.32 | 4 | 4 | 102 | 102 |
| 1239 | 8.02 | tr|Q53GX6|Q53GX6_HUMAN | Nucleobindin 1 variant (Fragment) OS=Homo sapiens PE=2 SV=1 | 53907 | 461 | 10.85 | 6 | 4 | 37 | 18 |
| 1240 | 8.02 | tr|Q71UA6|Q71UA6_HUMAN | Amino acid transporter OS=Homo sapiens GN=SLC1A5 PE=2 SV=1 | 56582.7 | 541 | 12.75 | 5 | 1 | 34 | 6 |
| 1241 | 8.01 | sp|P62316|SMD2_HUMAN | Small nuclear ribonucleoprotein Sm D2 OS=Homo sapiens GN=SNRPD2 PE=1 SV=1 | 13526.8 | 118 | 45.76 | 5 | 5 | 14 | 14 |
| 1242 | 8.01 | sp|P19623|SPEE_HUMAN | Spermidine synthase OS=Homo sapiens GN=SRM PE=1 SV=1 | 33824.5 | 302 | 14.9 | 4 | 4 | 44 | 44 |
| 1243 | 8.01 | tr|D3DPK5|D3DPK5_HUMAN | SH3 domain binding glutamic acid-rich protein like 3, isoform CRA_a (Fragment) OS=Homo sapiens GN=SH3BGRL3 PE=4 SV=1 | 26819.1 | 257 | 18.29 | 4 | 4 | 50 | 50 |
| 1244 | 8 | tr|Q5SU16|Q5SU16_HUMAN | Tubulin beta chain OS=Homo sapiens GN=TUBB PE=2 SV=1 | 49670.5 | 444 | 65.54 | 29 | 5 | 952 | 171 |
| 1245 | 8 | tr|B3KT21|B3KT21_HUMAN | cDNA FLJ37476 fis, clone BRAWH2012827, highly similar to Homo sapiens BH3 interacting domain death agonist (BID), transcript variant 1, mRNA OS=Homo sapiens PE=2 SV=1 | 26809 | 241 | 26.56 | 5 | 4 | 20 | 19 |
| 1246 | 8 | tr|A8K5U9|A8K5U9_HUMAN | cDNA FLJ75056, highly similar to Homo sapiens phosphatidylinositol binding clathrin assembly protein (PICALM), mRNA OS=Homo sapiens PE=2 SV=1 | 70754.9 | 652 | 9.356 | 4 | 4 | 9 | 9 |
| 1247 | 8 | tr|B3KVY9|B3KVY9_HUMAN | cDNA FLJ41755 fis, clone HSYRA2009102, highly similar to Adenosine 3'-phospho 5'-phosphosulfate transporter 1 OS=Homo sapiens PE=2 SV=1 | 45071.5 | 412 | 10.19 | 4 | 4 | 13 | 13 |
| 1248 | 8 | tr|Q8N995|Q8N995_HUMAN | cDNA FLJ38173 fis, clone FCBBF1000053, highly similar to HYDROXYMETHYLGLUTARYL-COA SYNTHASE, CYTOPLASMIC OS=Homo sapiens PE=2 SV=1 | 56237.9 | 509 | 13.75 | 4 | 4 | 29 | 29 |
| 1249 | 8 | tr|V9HW53|V9HW53_HUMAN | Dimethylarginine dimethylaminohydrolase 2, isoform CRA_a OS=Homo sapiens GN=HEL-S-277 PE=1 SV=1 | 29643.5 | 285 | 22.81 | 4 | 4 | 27 | 27 |
| 1250 | 8 | sp|Q9BYD1|RM13_HUMAN | 39S ribosomal protein L13, mitochondrial OS=Homo sapiens GN=MRPL13 PE=1 SV=1 | 20691.8 | 178 | 28.09 | 4 | 4 | 17 | 17 |
| 1251 | 8 | sp|Q8IYB8|SUV3_HUMAN | ATP-dependent RNA helicase SUPV3L1, mitochondrial OS=Homo sapiens GN=SUPV3L1 PE=1 SV=1 | 87990.3 | 786 | 7.888 | 4 | 4 | 12 | 12 |
| 1252 | 8 | tr|V9HW87|V9HW87_HUMAN | Abhydrolase domain containing 14B, isoform CRA_a OS=Homo sapiens GN=HEL-S-299 PE=2 SV=1 | 22345.5 | 210 | 28.1 | 4 | 4 | 21 | 20 |
| 1253 | 8 | sp|Q9H0C8|ILKAP_HUMAN | Integrin-linked kinase-associated serine/threonine phosphatase 2C OS=Homo sapiens GN=ILKAP PE=1 SV=1 | 42906.1 | 392 | 11.22 | 4 | 4 | 25 | 25 |
| 1254 | 8 | sp|Q9UK76|HN1_HUMAN | Hematological and neurological expressed 1 protein OS=Homo sapiens GN=HN1 PE=1 SV=3 | 16014.4 | 154 | 46.75 | 5 | 5 | 27 | 27 |
| 1255 | 8 | sp|Q6IAA8|LTOR1_HUMAN | Ragulator complex protein LAMTOR1 OS=Homo sapiens GN=LAMTOR1 PE=1 SV=2 | 17744.6 | 161 | 40.37 | 4 | 4 | 23 | 23 |
| 1256 | 8 | tr|A4D0W0|A4D0W0_HUMAN | LSM8 homolog, U6 small nuclear RNA associated (S. cerevisiae) OS=Homo sapiens GN=LSM8 PE=2 SV=1 | 10402.6 | 96 | 46.88 | 4 | 4 | 18 | 18 |
| 1257 | 8 | tr|A0A0S2Z4Q3|A0A0S2Z4Q3_HUMAN | CCHC-type zinc finger nucleic acid binding protein isoform 1 (Fragment) OS=Homo sapiens GN=CNBP PE=2 SV=1 | 18741.8 | 170 | 31.76 | 5 | 5 | 11 | 10 |
| 1258 | 8 | tr|A0A024R1X4|A0A024R1X4_HUMAN | HCG16955, isoform CRA_b OS=Homo sapiens GN=hCG_16955 PE=4 SV=1 | 11731.1 | 106 | 38.68 | 4 | 4 | 36 | 36 |
| 1259 | 7.98 | tr|I3L1L3|I3L1L3_HUMAN | Myb-binding protein 1A (Fragment) OS=Homo sapiens GN=MYBBP1A PE=1 SV=1 | 140187.4 | 1252 | 4.473 | 6 | 5 | 21 | 16 |
| 1260 | 7.98 | sp|O75844|FACE1_HUMAN | CAAX prenyl protease 1 homolog OS=Homo sapiens GN=ZMPSTE24 PE=1 SV=2 | 54812.4 | 475 | 10.95 | 4 | 4 | 25 | 24 |
| 1261 | 7.97 | tr|A0A024QZN2|A0A024QZN2_HUMAN | HCG2024613, isoform CRA_a OS=Homo sapiens GN=hCG_2024613 PE=4 SV=1 | 29909.5 | 260 | 21.92 | 4 | 4 | 24 | 24 |
| 1262 | 7.96 | tr|A0A0D9SFB1|A0A0D9SFB1_HUMAN | Dynamin-1 OS=Homo sapiens GN=DNM1 PE=1 SV=1 | 94016.2 | 835 | 10.06 | 10 | 4 | 28 | 8 |
| 1263 | 7.96 | tr|A8K3S1|A8K3S1_HUMAN | Glucosamine-6-phosphate isomerase OS=Homo sapiens PE=2 SV=1 | 32678.3 | 289 | 12.11 | 4 | 4 | 21 | 21 |
| 1264 | 7.96 | tr|A0A140VKF2|A0A140VKF2_HUMAN | Testis tissue sperm-binding protein Li 69n OS=Homo sapiens PE=2 SV=1 | 34576.9 | 310 | 16.13 | 4 | 4 | 34 | 33 |
| 1265 | 7.96 | tr|A0A024R7Z5|A0A024R7Z5_HUMAN | Syndecan binding protein (Syntenin), isoform CRA_c OS=Homo sapiens GN=SDCBP PE=4 SV=1 | 32444.1 | 298 | 24.16 | 5 | 5 | 20 | 20 |
| 1266 | 7.95 | tr|B2RCQ5|B2RCQ5_HUMAN | cDNA, FLJ96216, highly similar to Homo sapiens breast carcinoma amplified sequence 1 (BCAS1), mRNA OS=Homo sapiens PE=2 SV=1 | 61734.6 | 584 | 8.904 | 5 | 5 | 8 | 7 |
| 1267 | 7.95 | sp|O75964|ATP5L_HUMAN | ATP synthase subunit g, mitochondrial OS=Homo sapiens GN=ATP5L PE=1 SV=3 | 11428.4 | 103 | 58.25 | 5 | 5 | 24 | 24 |
| 1268 | 7.94 | tr|B3KT81|B3KT81_HUMAN | cDNA FLJ37836 fis, clone BRSSN2010587, weakly similar to Homo sapiens MDN1, midasin homolog (yeast) (MDN1), mRNA OS=Homo sapiens PE=2 SV=1 | 114776.5 | 1018 | 7.269 | 7 | 6 | 11 | 10 |
| 1269 | 7.94 | sp|Q6PJT7|ZC3HE_HUMAN | Zinc finger CCCH domain-containing protein 14 OS=Homo sapiens GN=ZC3H14 PE=1 SV=1 | 82875 | 736 | 7.065 | 4 | 4 | 10 | 10 |
| 1270 | 7.93 | sp|Q969H8|MYDGF_HUMAN | Myeloid-derived growth factor OS=Homo sapiens GN=MYDGF PE=1 SV=1 | 18795.1 | 173 | 27.17 | 5 | 5 | 59 | 56 |
| 1271 | 7.92 | sp|P20674|COX5A_HUMAN | Cytochrome c oxidase subunit 5A, mitochondrial OS=Homo sapiens GN=COX5A PE=1 SV=2 | 16762 | 150 | 41.33 | 5 | 5 | 39 | 39 |
| 1272 | 7.9 | sp|P21912|SDHB_HUMAN | Succinate dehydrogenase [ubiquinone] iron-sulfur subunit, mitochondrial OS=Homo sapiens GN=SDHB PE=1 SV=3 | 31629.4 | 280 | 11.79 | 4 | 4 | 21 | 21 |
| 1273 | 7.9 | sp|Q9Y3D0|MIP18_HUMAN | Mitotic spindle-associated MMXD complex subunit MIP18 OS=Homo sapiens GN=FAM96B PE=1 SV=1 | 17662.9 | 163 | 43.56 | 4 | 4 | 26 | 22 |
| 1274 | 7.89 | tr|A3R0T8|A3R0T8_HUMAN | Histone 1, H1e OS=Homo sapiens GN=HIST1H1E PE=2 SV=1 | 21865 | 219 | 57.08 | 24 | 5 | 391 | 31 |
| 1275 | 7.88 | sp|Q8IXI1|MIRO2_HUMAN | Mitochondrial Rho GTPase 2 OS=Homo sapiens GN=RHOT2 PE=1 SV=2 | 68117.2 | 618 | 9.709 | 4 | 4 | 16 | 16 |
| 1276 | 7.87 | tr|V9HWI1|V9HWI1_HUMAN | Epididymis secretory protein Li 10 OS=Homo sapiens GN=HEL-S-10 PE=2 SV=1 | 22119.2 | 206 | 41.26 | 5 | 5 | 14 | 14 |
| 1277 | 7.84 | tr|B2RAH5|B2RAH5_HUMAN | Protein phosphatase 1 regulatory subunit OS=Homo sapiens PE=2 SV=1 | 115327.7 | 1030 | 5.922 | 6 | 5 | 15 | 14 |
| 1278 | 7.84 | tr|A8MT37|A8MT37_HUMAN | Glycogen synthase kinase-3 alpha OS=Homo sapiens GN=GSK3A PE=1 SV=2 | 44919.2 | 401 | 13.22 | 5 | 4 | 8 | 7 |
| 1279 | 7.84 | tr|A0A087WWS1|A0A087WWS1_HUMAN | THO complex subunit 1 OS=Homo sapiens GN=THOC1 PE=1 SV=1 | 75651.6 | 657 | 8.067 | 4 | 4 | 12 | 12 |
| 1280 | 7.84 | sp|Q92522|H1X_HUMAN | Histone H1x OS=Homo sapiens GN=H1FX PE=1 SV=1 | 22487 | 213 | 29.11 | 5 | 5 | 17 | 17 |
| 1281 | 7.83 | tr|V9HW95|V9HW95_HUMAN | Epididymis secretory protein Li 84 OS=Homo sapiens GN=HEL-S-84 PE=2 SV=1 | 38889.7 | 331 | 14.5 | 4 | 4 | 27 | 27 |
| 1282 | 7.82 | tr|A0A0C4DFM1|A0A0C4DFM1_HUMAN | Transmembrane 9 superfamily member OS=Homo sapiens GN=TM9SF4 PE=1 SV=1 | 72540.7 | 625 | 11.2 | 4 | 4 | 19 | 19 |
| 1283 | 7.81 | tr|A0A024QZF2|A0A024QZF2_HUMAN | Related RAS viral (R-ras) oncogene homolog, isoform CRA_a OS=Homo sapiens GN=RRAS PE=4 SV=1 | 23480.3 | 218 | 26.15 | 4 | 2 | 14 | 2 |
| 1284 | 7.8 | tr|V9HWG9|V9HWG9_HUMAN | Epididymis secretory protein Li 21 OS=Homo sapiens GN=HEL-S-21 PE=2 SV=1 | 27565.6 | 241 | 14.11 | 4 | 4 | 21 | 21 |
| 1285 | 7.79 | sp|Q86W42|THOC6_HUMAN | THO complex subunit 6 homolog OS=Homo sapiens GN=THOC6 PE=1 SV=1 | 37534.6 | 341 | 13.49 | 4 | 4 | 16 | 15 |
| 1286 | 7.79 | tr|A0A140VJL8|A0A140VJL8_HUMAN | Testicular tissue protein Li 94 OS=Homo sapiens PE=2 SV=1 | 36694.4 | 336 | 15.18 | 4 | 4 | 18 | 18 |
| 1287 | 7.79 | tr|A0A087WZT2|A0A087WZT2_HUMAN | Methyltransferase-like protein 7B OS=Homo sapiens GN=METTL7B PE=1 SV=1 | 31287.6 | 276 | 14.13 | 4 | 4 | 15 | 15 |
| 1288 | 7.77 | tr|F8VXU5|F8VXU5_HUMAN | Vacuolar protein sorting-associated protein 29 OS=Homo sapiens GN=VPS29 PE=1 SV=1 | 23979.6 | 214 | 17.76 | 4 | 4 | 21 | 21 |
| 1289 | 7.75 | sp|P61221|ABCE1_HUMAN | ATP-binding cassette sub-family E member 1 OS=Homo sapiens GN=ABCE1 PE=1 SV=1 | 67313.7 | 599 | 8.848 | 5 | 5 | 18 | 18 |
| 1290 | 7.75 | sp|Q6P1J9|CDC73_HUMAN | Parafibromin OS=Homo sapiens GN=CDC73 PE=1 SV=1 | 60576.1 | 531 | 7.345 | 4 | 4 | 10 | 10 |
| 1291 | 7.74 | tr|B7ZC38|B7ZC38_HUMAN | Endophilin-B2 OS=Homo sapiens GN=SH3GLB2 PE=1 SV=1 | 44360.8 | 400 | 14.25 | 5 | 4 | 8 | 6 |
| 1292 | 7.74 | sp|Q14019|COTL1_HUMAN | Coactosin-like protein OS=Homo sapiens GN=COTL1 PE=1 SV=3 | 15944.9 | 142 | 30.28 | 4 | 4 | 17 | 17 |
| 1293 | 7.73 | tr|Q6FGV9|Q6FGV9_HUMAN | PMVK protein (Fragment) OS=Homo sapiens GN=PMVK PE=2 SV=1 | 21994.7 | 192 | 18.23 | 4 | 4 | 9 | 9 |
| 1294 | 7.69 | tr|A8K2L6|A8K2L6_HUMAN | Annexin OS=Homo sapiens PE=2 SV=1 | 39738.3 | 357 | 12.04 | 4 | 4 | 11 | 11 |
| 1295 | 7.69 | tr|D3DV26|D3DV26_HUMAN | S100 calcium binding protein A10 (Annexin II ligand, calpactin I, light polypeptide (P11)), isoform CRA_b (Fragment) OS=Homo sapiens GN=S100A10 PE=4 SV=1 | 22359.5 | 205 | 25.85 | 5 | 5 | 37 | 37 |
| 1296 | 7.69 | tr|Q6NTG0|Q6NTG0_HUMAN | SLC9A3R2 protein (Fragment) OS=Homo sapiens GN=SLC9A3R2 PE=2 SV=1 | 40575 | 372 | 12.37 | 5 | 5 | 10 | 10 |
| 1297 | 7.65 | sp|P23368|MAOM_HUMAN | NAD-dependent malic enzyme, mitochondrial OS=Homo sapiens GN=ME2 PE=1 SV=1 | 65442.9 | 584 | 7.021 | 4 | 4 | 13 | 13 |
| 1298 | 7.65 | tr|Q6LES8|Q6LES8_HUMAN | TFAM protein (Fragment) OS=Homo sapiens GN=TFAM PE=2 SV=1 | 29082.4 | 246 | 17.89 | 5 | 5 | 21 | 21 |
| 1299 | 7.64 | tr|Q86SZ7|Q86SZ7_HUMAN | Full-length cDNA clone CS0DJ015YJ12 of T cells (Jurkat cell line) of Homo sapiens (human) OS=Homo sapiens GN=PSME2 PE=2 SV=1 | 27401.4 | 239 | 10.88 | 4 | 4 | 14 | 14 |
| 1300 | 7.64 | tr|A0A140VJX3|A0A140VJX3_HUMAN | Sulfurtransferase OS=Homo sapiens PE=2 SV=1 | 33178.1 | 297 | 15.15 | 4 | 4 | 10 | 10 |
| 1301 | 7.63 | tr|Q53H22|Q53H22_HUMAN | Phosphoribosyl pyrophosphate amidotransferase proprotein variant (Fragment) OS=Homo sapiens PE=2 SV=1 | 57384.5 | 517 | 14.51 | 6 | 6 | 14 | 14 |
| 1302 | 7.62 | tr|V9HWB7|V9HWB7_HUMAN | Aconitate hydratase OS=Homo sapiens GN=HEL60 PE=2 SV=1 | 98398.1 | 889 | 8.549 | 5 | 5 | 16 | 16 |
| 1303 | 7.62 | tr|A0A024RBL2|A0A024RBL2_HUMAN | Serine dehydratase-like, isoform CRA_a OS=Homo sapiens GN=SDSL PE=4 SV=1 | 34674 | 329 | 17.02 | 4 | 4 | 7 | 7 |
| 1304 | 7.61 | sp|Q9P1Y5|CAMP3_HUMAN | Calmodulin-regulated spectrin-associated protein 3 OS=Homo sapiens GN=CAMSAP3 PE=1 SV=2 | 134748.1 | 1249 | 10.01 | 6 | 6 | 7 | 7 |
| 1305 | 7.59 | tr|A0A024R3R5|A0A024R3R5_HUMAN | Lamin B receptor, isoform CRA_a OS=Homo sapiens GN=LBR PE=4 SV=1 | 70729.5 | 615 | 8.455 | 5 | 5 | 10 | 10 |
| 1306 | 7.59 | sp|O94919|ENDD1_HUMAN | Endonuclease domain-containing 1 protein OS=Homo sapiens GN=ENDOD1 PE=1 SV=2 | 55016.1 | 500 | 8.4 | 4 | 4 | 19 | 19 |
| 1307 | 7.58 | tr|A0A024RBA9|A0A024RBA9_HUMAN | RAB21, member RAS oncogene family, isoform CRA_a OS=Homo sapiens GN=RAB21 PE=4 SV=1 | 24347.4 | 225 | 23.56 | 6 | 5 | 34 | 16 |
| 1308 | 7.57 | tr|K7EQ55|K7EQ55_HUMAN | DAZ-associated protein 1 OS=Homo sapiens GN=DAZAP1 PE=1 SV=2 | 43312.2 | 406 | 18.23 | 5 | 5 | 10 | 10 |
| 1309 | 7.55 | tr|A0A0S2Z4A1|A0A0S2Z4A1_HUMAN | Phosphofructokinase muscle isoform 3 (Fragment) OS=Homo sapiens GN=PFKM PE=2 SV=1 | 79236.2 | 725 | 20.28 | 11 | 7 | 36 | 19 |
| 1310 | 7.55 | tr|B2R858|B2R858_HUMAN | cDNA, FLJ93750, Homo sapiens DEAD (Asp-Glu-Ala-Asp) box polypeptide 6 (DDX6), mRNA OS=Homo sapiens PE=2 SV=1 | 53216.1 | 472 | 13.56 | 4 | 4 | 21 | 21 |
| 1311 | 7.54 | sp|Q9BY77|PDIP3_HUMAN | Polymerase delta-interacting protein 3 OS=Homo sapiens GN=POLDIP3 PE=1 SV=2 | 46088.9 | 421 | 16.39 | 4 | 4 | 19 | 19 |
| 1312 | 7.54 | tr|A8K3M3|A8K3M3_HUMAN | Tyrosine-protein phosphatase non-receptor type OS=Homo sapiens GN=PTPN1 PE=2 SV=1 | 49966.4 | 435 | 12.87 | 4 | 4 | 20 | 20 |
| 1313 | 7.53 | sp|O94826|TOM70_HUMAN | Mitochondrial import receptor subunit TOM70 OS=Homo sapiens GN=TOMM70 PE=1 SV=1 | 67454.2 | 608 | 7.566 | 6 | 6 | 10 | 8 |
| 1314 | 7.51 | sp|Q14651|PLSI_HUMAN | Plastin-1 OS=Homo sapiens GN=PLS1 PE=1 SV=2 | 70252.7 | 629 | 12.56 | 6 | 4 | 28 | 23 |
| 1315 | 7.5 | tr|A0M8W4|A0M8W4_HUMAN | Ubiquitin-conjugating enzyme E2 variant 2 OS=Homo sapiens GN=UBE2V2 PE=2 SV=1 | 16362.7 | 145 | 42.76 | 6 | 4 | 55 | 23 |
| 1316 | 7.5 | sp|Q15008|PSMD6_HUMAN | 26S proteasome non-ATPase regulatory subunit 6 OS=Homo sapiens GN=PSMD6 PE=1 SV=1 | 45530.9 | 389 | 12.6 | 5 | 5 | 11 | 11 |
| 1317 | 7.45 | tr|K7ERF1|K7ERF1_HUMAN | Eukaryotic translation initiation factor 3 subunit K OS=Homo sapiens GN=EIF3K PE=1 SV=1 | 22095 | 192 | 18.75 | 5 | 4 | 11 | 10 |
| 1318 | 7.44 | sp|O95373|IPO7_HUMAN | Importin-7 OS=Homo sapiens GN=IPO7 PE=1 SV=1 | 119515.5 | 1038 | 7.611 | 6 | 6 | 10 | 10 |
| 1319 | 7.43 | sp|Q8NFV4|ABHDB_HUMAN | Protein ABHD11 OS=Homo sapiens GN=ABHD11 PE=1 SV=1 | 34689.7 | 315 | 19.05 | 4 | 4 | 20 | 20 |
| 1320 | 7.42 | tr|A8K4A8|A8K4A8_HUMAN | cDNA FLJ76156, highly similar to Homo sapiens aspartyl-tRNA synthetase 2 (DARS2), mRNA OS=Homo sapiens PE=2 SV=1 | 73574 | 645 | 8.837 | 4 | 4 | 7 | 7 |
| 1321 | 7.41 | tr|B7Z6C9|B7Z6C9_HUMAN | Transmembrane 9 superfamily member OS=Homo sapiens PE=2 SV=1 | 92485.6 | 815 | 7.485 | 5 | 5 | 9 | 9 |
| 1322 | 7.4 | tr|B4DPD5|B4DPD5_HUMAN | cDNA FLJ56307, highly similar to Ubiquitin thioesterase protein OTUB1 (EC 3.4.-.-) OS=Homo sapiens PE=2 SV=1 | 35180.4 | 308 | 19.81 | 4 | 4 | 20 | 20 |
| 1323 | 7.4 | sp|O00204|ST2B1_HUMAN | Sulfotransferase family cytosolic 2B member 1 OS=Homo sapiens GN=SULT2B1 PE=1 SV=2 | 41307.3 | 365 | 14.52 | 4 | 4 | 17 | 17 |
| 1324 | 7.4 | tr|B7Z6L9|B7Z6L9_HUMAN | cDNA FLJ54755, highly similar to Vacuolar ATP synthase subunit d (EC 3.6.3.14) OS=Homo sapiens PE=2 SV=1 | 44661.5 | 392 | 8.673 | 4 | 4 | 5 | 5 |
| 1325 | 7.39 | tr|B4DZZ0|B4DZZ0_HUMAN | cDNA FLJ52128, highly similar to PRA1 family protein 3 OS=Homo sapiens PE=2 SV=1 | 19205.6 | 165 | 23.03 | 4 | 4 | 16 | 16 |
| 1326 | 7.38 | sp|Q5T4S7|UBR4_HUMAN | E3 ubiquitin-protein ligase UBR4 OS=Homo sapiens GN=UBR4 PE=1 SV=1 | 573835.3 | 5183 | 2.585 | 10 | 10 | 13 | 12 |
| 1327 | 7.37 | sp|P82933|RT09_HUMAN | 28S ribosomal protein S9, mitochondrial OS=Homo sapiens GN=MRPS9 PE=1 SV=2 | 45834.5 | 396 | 13.64 | 5 | 5 | 8 | 8 |
| 1328 | 7.36 | sp|Q92615|LAR4B_HUMAN | La-related protein 4B OS=Homo sapiens GN=LARP4B PE=1 SV=3 | 80551.2 | 738 | 4.878 | 4 | 4 | 10 | 10 |
| 1329 | 7.36 | tr|E5RHW4|E5RHW4_HUMAN | Erlin-2 (Fragment) OS=Homo sapiens GN=ERLIN2 PE=1 SV=1 | 37725.1 | 338 | 15.98 | 6 | 6 | 10 | 10 |
| 1330 | 7.35 | sp|Q13043|STK4_HUMAN | Serine/threonine-protein kinase 4 OS=Homo sapiens GN=STK4 PE=1 SV=2 | 55629.8 | 487 | 11.91 | 6 | 6 | 8 | 4 |
| 1331 | 7.34 | tr|A0A024R5U5|A0A024R5U5_HUMAN | ADAM metallopeptidase domain 10, isoform CRA_b OS=Homo sapiens GN=ADAM10 PE=4 SV=1 | 84141.5 | 748 | 6.684 | 4 | 4 | 8 | 8 |
| 1332 | 7.33 | sp|P50238|CRIP1_HUMAN | Cysteine-rich protein 1 OS=Homo sapiens GN=CRIP1 PE=1 SV=3 | 8532.8 | 77 | 48.05 | 7 | 6 | 14 | 13 |
| 1333 | 7.33 | tr|A0A024R571|A0A024R571_HUMAN | EH domain-containing protein 1 OS=Homo sapiens GN=EHD1 PE=1 SV=1 | 61926.8 | 548 | 13.32 | 6 | 5 | 18 | 11 |
| 1334 | 7.33 | tr|A0A096LPI6|A0A096LPI6_HUMAN | Uncharacterized protein (Fragment) OS=Homo sapiens PE=4 SV=2 | 30433.1 | 279 | 21.15 | 4 | 4 | 16 | 16 |
| 1335 | 7.31 | sp|Q8IXB1|DJC10_HUMAN | DnaJ homolog subfamily C member 10 OS=Homo sapiens GN=DNAJC10 PE=1 SV=2 | 91078.9 | 793 | 7.818 | 6 | 5 | 17 | 15 |
| 1336 | 7.28 | sp|P51812|KS6A3_HUMAN | Ribosomal protein S6 kinase alpha-3 OS=Homo sapiens GN=RPS6KA3 PE=1 SV=1 | 83735.3 | 740 | 5.811 | 5 | 4 | 9 | 7 |
| 1337 | 7.28 | tr|A0A0S2Z5V7|A0A0S2Z5V7_HUMAN | Histocompatibility 13 isoform 1 (Fragment) OS=Homo sapiens GN=HM13 PE=2 SV=1 | 41487.9 | 377 | 14.59 | 4 | 4 | 8 | 8 |
| 1338 | 7.27 | tr|B4DVA7|B4DVA7_HUMAN | Beta-hexosaminidase OS=Homo sapiens PE=2 SV=1 | 62012.9 | 540 | 11.11 | 5 | 5 | 13 | 12 |
| 1339 | 7.27 | sp|Q9H936|GHC1_HUMAN | Mitochondrial glutamate carrier 1 OS=Homo sapiens GN=SLC25A22 PE=1 SV=1 | 34469.8 | 323 | 17.03 | 5 | 5 | 12 | 12 |
| 1340 | 7.26 | tr|B3KQQ0|B3KQQ0_HUMAN | cDNA PSEC0007 fis, clone NT2RM1000634, highly similar to FK506-binding protein 9 (EC 5.2.1.8) OS=Homo sapiens PE=2 SV=1 | 63067.1 | 570 | 7.544 | 5 | 4 | 13 | 11 |
| 1341 | 7.26 | sp|Q8NFW8|NEUA_HUMAN | N-acylneuraminate cytidylyltransferase OS=Homo sapiens GN=CMAS PE=1 SV=2 | 48378.8 | 434 | 12.67 | 4 | 4 | 10 | 10 |
| 1342 | 7.25 | sp|P09496|CLCA_HUMAN | Clathrin light chain A OS=Homo sapiens GN=CLTA PE=1 SV=1 | 27076.5 | 248 | 21.37 | 5 | 4 | 40 | 37 |
| 1343 | 7.25 | tr|A0A024RBR3|A0A024RBR3_HUMAN | Density-regulated protein OS=Homo sapiens GN=DENR PE=3 SV=1 | 22091.8 | 198 | 18.69 | 4 | 4 | 13 | 13 |
| 1344 | 7.23 | tr|Q2TSD2|Q2TSD2_HUMAN | Aging-associated gene 7 protein OS=Homo sapiens PE=2 SV=1 | 41462.5 | 374 | 12.83 | 4 | 4 | 10 | 9 |
| 1345 | 7.22 | sp|Q16513|PKN2_HUMAN | Serine/threonine-protein kinase N2 OS=Homo sapiens GN=PKN2 PE=1 SV=1 | 112033.5 | 984 | 9.248 | 6 | 6 | 16 | 11 |
| 1346 | 7.22 | tr|A0A087WT12|A0A087WT12_HUMAN | Glutathione peroxidase OS=Homo sapiens GN=GPX4 PE=1 SV=1 | 26947.9 | 233 | 25.32 | 6 | 5 | 11 | 10 |
| 1347 | 7.22 | sp|Q12802|AKP13_HUMAN | A-kinase anchor protein 13 OS=Homo sapiens GN=AKAP13 PE=1 SV=2 | 307547.7 | 2813 | 3.555 | 7 | 7 | 10 | 9 |
| 1348 | 7.21 | tr|Q5LJA9|Q5LJA9_HUMAN | Ubiquitin carboxyl-terminal hydrolase (Fragment) OS=Homo sapiens GN=UCHL5 PE=1 SV=1 | 41694.3 | 368 | 13.04 | 4 | 4 | 10 | 10 |
| 1349 | 7.19 | tr|A8K5S3|A8K5S3_HUMAN | cDNA FLJ78449 OS=Homo sapiens PE=2 SV=1 | 39766.1 | 364 | 9.341 | 4 | 4 | 11 | 11 |
| 1350 | 7.19 | tr|A0A0R4J2G3|A0A0R4J2G3_HUMAN | Arylacetamide deacetylase-like 1 OS=Homo sapiens GN=NCEH1 PE=1 SV=1 | 49062.2 | 440 | 11.82 | 4 | 4 | 24 | 24 |
| 1351 | 7.18 | tr|A0A024R983|A0A024R983_HUMAN | TROVE domain family, member 2, isoform CRA_b OS=Homo sapiens GN=TROVE2 PE=4 SV=1 | 60670.1 | 538 | 7.249 | 4 | 4 | 13 | 13 |
| 1352 | 7.17 | sp|Q96AB3|ISOC2_HUMAN | Isochorismatase domain-containing protein 2 OS=Homo sapiens GN=ISOC2 PE=1 SV=1 | 22337 | 205 | 40 | 4 | 4 | 11 | 11 |
| 1353 | 7.16 | tr|E7DVW5|E7DVW5_HUMAN | Fatty acid binding protein 5 (Psoriasis-associated) OS=Homo sapiens GN=FABP5 PE=3 SV=1 | 15164.4 | 135 | 45.93 | 4 | 4 | 9 | 9 |
| 1354 | 7.15 | tr|A0A024R1T5|A0A024R1T5_HUMAN | 2',3'-cyclic-nucleotide 3'-phosphodiesterase OS=Homo sapiens GN=CNP PE=3 SV=1 | 45098.3 | 401 | 12.47 | 5 | 5 | 10 | 8 |
| 1355 | 7.15 | sp|P04920|B3A2_HUMAN | Anion exchange protein 2 OS=Homo sapiens GN=SLC4A2 PE=1 SV=4 | 137008 | 1241 | 8.944 | 6 | 6 | 15 | 15 |
| 1356 | 7.14 | sp|Q16643|DREB_HUMAN | Drebrin OS=Homo sapiens GN=DBN1 PE=1 SV=4 | 71428.6 | 649 | 7.858 | 4 | 4 | 12 | 12 |
| 1357 | 7.1 | tr|B4DW13|B4DW13_HUMAN | HCG23341, isoform CRA_d OS=Homo sapiens GN=hCG_23341 PE=2 SV=1 | 51399.9 | 454 | 10.35 | 4 | 4 | 14 | 14 |
| 1358 | 7.08 | sp|P85037|FOXK1_HUMAN | Forkhead box protein K1 OS=Homo sapiens GN=FOXK1 PE=1 SV=1 | 75456.5 | 733 | 8.731 | 5 | 5 | 10 | 9 |
| 1359 | 7.08 | tr|A0A024R3R0|A0A024R3R0_HUMAN | Mitochondrial ribosomal protein L55, isoform CRA_a OS=Homo sapiens GN=MRPL55 PE=4 SV=1 | 15128.2 | 128 | 38.28 | 4 | 4 | 12 | 12 |
| 1360 | 7.08 | sp|Q96IZ0|PAWR_HUMAN | PRKC apoptosis WT1 regulator protein OS=Homo sapiens GN=PAWR PE=1 SV=1 | 36567.2 | 340 | 12.65 | 4 | 4 | 4 | 4 |
| 1361 | 7.07 | tr|B4E0Y9|B4E0Y9_HUMAN | Serine/threonine-protein kinase 26 OS=Homo sapiens GN=STK26 PE=1 SV=1 | 49183.5 | 438 | 12.79 | 6 | 2 | 24 | 6 |
| 1362 | 7.07 | tr|D3DWL9|D3DWL9_HUMAN | Cleavage and polyadenylation specific factor 1, 160kDa, isoform CRA_a OS=Homo sapiens GN=CPSF1 PE=4 SV=1 | 151984.6 | 1365 | 4.542 | 5 | 4 | 11 | 10 |
| 1363 | 7.06 | sp|P35251|RFC1_HUMAN | Replication factor C subunit 1 OS=Homo sapiens GN=RFC1 PE=1 SV=4 | 128253.5 | 1148 | 4.443 | 4 | 4 | 5 | 5 |
| 1364 | 7.06 | tr|Q6FGG2|Q6FGG2_HUMAN | VAMP3 protein OS=Homo sapiens GN=VAMP3 PE=2 SV=1 | 11309 | 100 | 44 | 4 | 2 | 31 | 11 |
| 1365 | 7.04 | tr|A0A024QYS2|A0A024QYS2_HUMAN | Transmembrane 9 superfamily member OS=Homo sapiens PE=2 SV=1 | 67887.7 | 589 | 11.54 | 6 | 5 | 13 | 11 |
| 1366 | 7.04 | sp|Q13200|PSMD2_HUMAN | 26S proteasome non-ATPase regulatory subunit 2 OS=Homo sapiens GN=PSMD2 PE=1 SV=3 | 100198.8 | 908 | 6.057 | 4 | 4 | 14 | 14 |
| 1367 | 7.03 | tr|Q6IPI1|Q6IPI1_HUMAN | Ribosomal protein L29 OS=Homo sapiens GN=RPL29 PE=2 SV=1 | 17951.2 | 161 | 24.22 | 5 | 5 | 21 | 21 |
| 1368 | 7.02 | sp|Q13057|COASY_HUMAN | Bifunctional coenzyme A synthase OS=Homo sapiens GN=COASY PE=1 SV=4 | 62328.2 | 564 | 10.46 | 4 | 4 | 6 | 6 |
| 1369 | 7.02 | tr|A0A024R1Z6|A0A024R1Z6_HUMAN | Vesicle amine transport protein 1 homolog (T californica), isoform CRA_a OS=Homo sapiens GN=VAT1 PE=4 SV=1 | 41920 | 393 | 26.21 | 6 | 6 | 16 | 15 |
| 1370 | 6.99 | tr|C9J712|C9J712_HUMAN | Profilin OS=Homo sapiens GN=PFN2 PE=1 SV=1 | 9798.2 | 91 | 56.04 | 5 | 3 | 21 | 10 |
| 1371 | 6.98 | sp|P29353|SHC1_HUMAN | SHC-transforming protein 1 OS=Homo sapiens GN=SHC1 PE=1 SV=4 | 62821.6 | 583 | 13.72 | 5 | 5 | 10 | 10 |
| 1372 | 6.98 | tr|A0A024R5Y1|A0A024R5Y1_HUMAN | Spastic paraplegia 21 (Autosomal recessive, Mast syndrome), isoform CRA_a OS=Homo sapiens GN=SPG21 PE=4 SV=1 | 34959.8 | 308 | 18.18 | 4 | 4 | 8 | 8 |
| 1373 | 6.97 | sp|Q9UHD2|TBK1_HUMAN | Serine/threonine-protein kinase TBK1 OS=Homo sapiens GN=TBK1 PE=1 SV=1 | 83641.5 | 729 | 7.819 | 4 | 4 | 11 | 11 |
| 1374 | 6.97 | tr|Q6IAT9|Q6IAT9_HUMAN | Proteasome subunit beta type OS=Homo sapiens GN=PSMB6 PE=2 SV=1 | 25357.5 | 239 | 16.32 | 4 | 4 | 37 | 37 |
| 1375 | 6.96 | tr|A0A024R0J1|A0A024R0J1_HUMAN | p21(CDKN1A)-activated kinase 4, isoform CRA_a OS=Homo sapiens GN=PAK4 PE=4 SV=1 | 64071.5 | 591 | 8.46 | 4 | 4 | 9 | 9 |
| 1376 | 6.94 | sp|O15231|ZN185_HUMAN | Zinc finger protein 185 OS=Homo sapiens GN=ZNF185 PE=1 SV=3 | 73524.7 | 689 | 8.418 | 3 | 3 | 4 | 4 |
| 1377 | 6.94 | sp|Q8IVS2|FABD_HUMAN | Malonyl-CoA-acyl carrier protein transacylase, mitochondrial OS=Homo sapiens GN=MCAT PE=1 SV=2 | 42961.2 | 390 | 15.9 | 4 | 4 | 13 | 13 |
| 1378 | 6.92 | sp|Q14240|IF4A2_HUMAN | Eukaryotic initiation factor 4A-II OS=Homo sapiens GN=EIF4A2 PE=1 SV=2 | 46401.9 | 407 | 33.91 | 12 | 6 | 162 | 49 |
| 1379 | 6.91 | tr|B4DRU9|B4DRU9_HUMAN | cDNA FLJ57179, highly similar to Homo sapiens ATP-binding cassette, sub-family F (GCN20), member 3 (ABCF3), mRNA OS=Homo sapiens PE=2 SV=1 | 75573.5 | 668 | 10.63 | 5 | 5 | 6 | 6 |
| 1380 | 6.9 | tr|H3BNW0|H3BNW0_HUMAN | THUMP domain-containing protein 1 OS=Homo sapiens GN=THUMPD1 PE=1 SV=1 | 15761.6 | 145 | 25.52 | 4 | 4 | 8 | 8 |
| 1381 | 6.89 | sp|Q8NBX0|SCPDL_HUMAN | Saccharopine dehydrogenase-like oxidoreductase OS=Homo sapiens GN=SCCPDH PE=1 SV=1 | 47151 | 429 | 13.52 | 5 | 5 | 11 | 11 |
| 1382 | 6.88 | sp|O95168|NDUB4_HUMAN | NADH dehydrogenase [ubiquinone] 1 beta subcomplex subunit 4 OS=Homo sapiens GN=NDUFB4 PE=1 SV=3 | 15208.4 | 129 | 32.56 | 4 | 4 | 27 | 27 |
| 1383 | 6.87 | tr|B3KXF2|B3KXF2_HUMAN | cDNA FLJ45314 fis, clone BRHIP3005142, highly similar to Proteasome-associated protein ECM29 homolog OS=Homo sapiens PE=2 SV=1 | 145370.8 | 1314 | 5.327 | 5 | 5 | 6 | 6 |
| 1384 | 6.87 | sp|Q9BVG4|PBDC1_HUMAN | Protein PBDC1 OS=Homo sapiens GN=PBDC1 PE=1 SV=1 | 26056.6 | 233 | 20.6 | 4 | 4 | 22 | 22 |
| 1385 | 6.86 | tr|V9HWF9|V9HWF9_HUMAN | Epididymis luminal protein 20 OS=Homo sapiens GN=HEL20 PE=2 SV=1 | 33392 | 308 | 19.16 | 4 | 4 | 11 | 11 |
| 1386 | 6.84 | tr|A0A024R0H6|A0A024R0H6_HUMAN | Paf1, RNA polymerase II associated factor, homolog (S. cerevisiae), isoform CRA_a OS=Homo sapiens GN=PAF1 PE=4 SV=1 | 59975.2 | 531 | 9.04 | 4 | 4 | 11 | 10 |
| 1387 | 6.83 | tr|Q53H94|Q53H94_HUMAN | Aldehyde dehydrogenase 6A1 variant (Fragment) OS=Homo sapiens PE=2 SV=1 | 57825.3 | 535 | 7.103 | 4 | 4 | 8 | 8 |
| 1388 | 6.81 | sp|Q9NZZ3|CHMP5_HUMAN | Charged multivesicular body protein 5 OS=Homo sapiens GN=CHMP5 PE=1 SV=1 | 24570.5 | 219 | 24.2 | 4 | 4 | 11 | 11 |
| 1389 | 6.8 | sp|P61026|RAB10_HUMAN | Ras-related protein Rab-10 OS=Homo sapiens GN=RAB10 PE=1 SV=1 | 22540.7 | 200 | 33.5 | 8 | 4 | 108 | 10 |
| 1390 | 6.79 | tr|Q9UES0|Q9UES0_HUMAN | SNARE protein Ykt6 (Fragment) OS=Homo sapiens PE=2 SV=1 | 21606.4 | 191 | 19.9 | 3 | 3 | 15 | 15 |
| 1391 | 6.79 | tr|V9HW09|V9HW09_HUMAN | Epididymis secretory sperm binding protein Li 91n OS=Homo sapiens GN=HEL-S-91n PE=2 SV=1 | 39611.5 | 350 | 18.29 | 5 | 5 | 8 | 7 |
| 1392 | 6.79 | sp|Q05519|SRS11_HUMAN | Serine/arginine-rich splicing factor 11 OS=Homo sapiens GN=SRSF11 PE=1 SV=1 | 53541.6 | 484 | 12.4 | 4 | 4 | 11 | 11 |
| 1393 | 6.79 | tr|Q86U62|Q86U62_HUMAN | Proteasome subunit beta type OS=Homo sapiens PE=2 SV=1 | 30018.2 | 277 | 10.11 | 3 | 3 | 19 | 19 |
| 1394 | 6.79 | sp|O43768|ENSA_HUMAN | Alpha-endosulfine OS=Homo sapiens GN=ENSA PE=1 SV=1 | 13389 | 121 | 42.98 | 4 | 4 | 23 | 23 |
| 1395 | 6.76 | sp|P55795|HNRH2_HUMAN | Heterogeneous nuclear ribonucleoprotein H2 OS=Homo sapiens GN=HNRNPH2 PE=1 SV=1 | 49263.3 | 449 | 34.08 | 12 | 5 | 106 | 19 |
| 1396 | 6.76 | tr|B7Z2X0|B7Z2X0_HUMAN | cDNA FLJ60543, highly similar to Active breakpoint cluster region-related protein OS=Homo sapiens PE=2 SV=1 | 88073.8 | 769 | 6.242 | 4 | 3 | 9 | 7 |
| 1397 | 6.76 | tr|Q6IAM0|Q6IAM0_HUMAN | Eukaryotic translation initiation factor 3 subunit G OS=Homo sapiens GN=EIF3G PE=2 SV=1 | 35600.7 | 320 | 22.19 | 4 | 4 | 4 | 4 |
| 1398 | 6.75 | tr|A0A0A0MTB8|A0A0A0MTB8_HUMAN | WD repeat-containing protein 36 OS=Homo sapiens GN=WDR36 PE=1 SV=1 | 99365.3 | 895 | 5.698 | 5 | 4 | 17 | 16 |
| 1399 | 6.75 | tr|E7ETK0|E7ETK0_HUMAN | 40S ribosomal protein S24 OS=Homo sapiens GN=RPS24 PE=1 SV=1 | 15196.9 | 131 | 20.61 | 3 | 3 | 61 | 61 |
| 1400 | 6.74 | tr|A0A024R4X0|A0A024R4X0_HUMAN | NADH-cytochrome b5 reductase OS=Homo sapiens GN=CYB5R3 PE=3 SV=1 | 33238.4 | 291 | 22.34 | 5 | 4 | 27 | 24 |
| 1401 | 6.73 | sp|Q9BVJ6|UT14A_HUMAN | U3 small nucleolar RNA-associated protein 14 homolog A OS=Homo sapiens GN=UTP14A PE=1 SV=1 | 87977.2 | 771 | 6.744 | 4 | 4 | 8 | 8 |
| 1402 | 6.73 | tr|F5H423|F5H423_HUMAN | Uncharacterized protein OS=Homo sapiens PE=3 SV=1 | 23345.6 | 210 | 29.52 | 5 | 2 | 76 | 9 |
| 1403 | 6.73 | tr|A0A024R5Z3|A0A024R5Z3_HUMAN | Mothers against decapentaplegic homolog OS=Homo sapiens GN=SMAD3 PE=3 SV=1 | 48080.4 | 425 | 11.29 | 3 | 3 | 14 | 14 |
| 1404 | 6.72 | sp|P62310|LSM3_HUMAN | U6 snRNA-associated Sm-like protein LSm3 OS=Homo sapiens GN=LSM3 PE=1 SV=2 | 11845.3 | 102 | 27.45 | 4 | 4 | 25 | 25 |
| 1405 | 6.71 | sp|Q92621|NU205_HUMAN | Nuclear pore complex protein Nup205 OS=Homo sapiens GN=NUP205 PE=1 SV=3 | 227919.2 | 2012 | 2.783 | 4 | 4 | 7 | 7 |
| 1406 | 6.71 | sp|O00116|ADAS_HUMAN | Alkyldihydroxyacetonephosphate synthase, peroxisomal OS=Homo sapiens GN=AGPS PE=1 SV=1 | 72911.2 | 658 | 8.663 | 4 | 4 | 12 | 12 |
| 1407 | 6.71 | sp|Q9H3R2|MUC13_HUMAN | Mucin-13 OS=Homo sapiens GN=MUC13 PE=1 SV=3 | 54603.3 | 512 | 10.94 | 4 | 4 | 15 | 15 |
| 1408 | 6.7 | sp|O75312|ZPR1_HUMAN | Zinc finger protein ZPR1 OS=Homo sapiens GN=ZPR1 PE=1 SV=1 | 50924.8 | 459 | 10.68 | 4 | 4 | 15 | 15 |
| 1409 | 6.69 | sp|Q96QR8|PURB_HUMAN | Transcriptional activator protein Pur-beta OS=Homo sapiens GN=PURB PE=1 SV=3 | 33240.6 | 312 | 13.46 | 4 | 4 | 13 | 13 |
| 1410 | 6.68 | sp|Q01081|U2AF1_HUMAN | Splicing factor U2AF 35 kDa subunit OS=Homo sapiens GN=U2AF1 PE=1 SV=3 | 27871.9 | 240 | 19.58 | 4 | 4 | 9 | 9 |
| 1411 | 6.67 | tr|F8VZX2|F8VZX2_HUMAN | Poly(rC)-binding protein 2 OS=Homo sapiens GN=PCBP2 PE=1 SV=1 | 33799.6 | 321 | 31.78 | 9 | 4 | 140 | 33 |
| 1412 | 6.67 | tr|A8K2R3|A8K2R3_HUMAN | cDNA FLJ75083, highly similar to Homo sapiens amine oxidase (flavin containing) domain 2 (AOF2),mRNA OS=Homo sapiens PE=2 SV=1 | 81169 | 730 | 8.493 | 4 | 4 | 8 | 8 |
| 1413 | 6.67 | tr|E5RG17|E5RG17_HUMAN | Putative deoxyribonuclease TATDN1 (Fragment) OS=Homo sapiens GN=TATDN1 PE=1 SV=1 | 36418.6 | 322 | 12.42 | 4 | 4 | 9 | 8 |
| 1414 | 6.67 | sp|P49419|AL7A1_HUMAN | Alpha-aminoadipic semialdehyde dehydrogenase OS=Homo sapiens GN=ALDH7A1 PE=1 SV=5 | 58486.7 | 539 | 8.905 | 5 | 5 | 31 | 31 |
| 1415 | 6.65 | tr|B0YJ88|B0YJ88_HUMAN | Radixin OS=Homo sapiens GN=RDX PE=2 SV=1 | 68563.5 | 583 | 22.13 | 16 | 4 | 132 | 11 |
| 1416 | 6.65 | sp|P49756|RBM25_HUMAN | RNA-binding protein 25 OS=Homo sapiens GN=RBM25 PE=1 SV=3 | 100184.5 | 843 | 6.287 | 5 | 4 | 10 | 7 |
| 1417 | 6.64 | tr|B4DWS9|B4DWS9_HUMAN | cDNA FLJ57640, highly similar to Serpin B5 OS=Homo sapiens PE=2 SV=1 | 32460.2 | 287 | 19.51 | 4 | 4 | 9 | 9 |
| 1418 | 6.6 | tr|Q53HG0|Q53HG0_HUMAN | Eukaryotic translation initiation factor 3, subunit 3 gamma, 40kDa variant (Fragment) OS=Homo sapiens PE=2 SV=1 | 39870 | 352 | 11.65 | 4 | 4 | 34 | 34 |
| 1419 | 6.58 | sp|Q9H6T0|ESRP2_HUMAN | Epithelial splicing regulatory protein 2 OS=Homo sapiens GN=ESRP2 PE=1 SV=1 | 78400.3 | 727 | 6.465 | 5 | 3 | 8 | 4 |
| 1420 | 6.58 | sp|Q96CN7|ISOC1_HUMAN | Isochorismatase domain-containing protein 1 OS=Homo sapiens GN=ISOC1 PE=1 SV=3 | 32236.5 | 298 | 20.81 | 4 | 4 | 12 | 12 |
| 1421 | 6.58 | sp|O00193|SMAP_HUMAN | Small acidic protein OS=Homo sapiens GN=SMAP PE=1 SV=1 | 20332.4 | 183 | 25.14 | 3 | 3 | 15 | 15 |
| 1422 | 6.57 | tr|Q53GI5|Q53GI5_HUMAN | Adaptor-related protein complex 1, mu 2 subunit variant (Fragment) OS=Homo sapiens PE=2 SV=1 | 48109.1 | 423 | 10.87 | 5 | 3 | 23 | 18 |
| 1423 | 6.57 | sp|P62314|SMD1_HUMAN | Small nuclear ribonucleoprotein Sm D1 OS=Homo sapiens GN=SNRPD1 PE=1 SV=1 | 13281.5 | 119 | 54.62 | 4 | 4 | 16 | 16 |
| 1424 | 6.56 | tr|B2R6K4|B2R6K4_HUMAN | cDNA, FLJ92996, highly similar to Homo sapiens guanine nucleotide binding protein (G protein), beta polypeptide 1 (GNB1), mRNA OS=Homo sapiens PE=2 SV=1 | 37300.6 | 340 | 25.88 | 8 | 4 | 24 | 9 |
| 1425 | 6.56 | sp|Q7LBR1|CHM1B_HUMAN | Charged multivesicular body protein 1b OS=Homo sapiens GN=CHMP1B PE=1 SV=1 | 22109.2 | 199 | 18.59 | 5 | 5 | 13 | 12 |
| 1426 | 6.53 | tr|A0A024QZF6|A0A024QZF6_HUMAN | AKT1 substrate 1 (Proline-rich), isoform CRA_a OS=Homo sapiens GN=AKT1S1 PE=4 SV=1 | 27383 | 256 | 20.7 | 4 | 4 | 8 | 8 |
| 1427 | 6.53 | tr|B2R8N1|B2R8N1_HUMAN | cDNA, FLJ93976, highly similar to Homo sapiens COP9 homolog (COP9), mRNA OS=Homo sapiens PE=2 SV=1 | 23197.4 | 209 | 21.53 | 3 | 3 | 18 | 18 |
| 1428 | 6.49 | tr|E9PFR3|E9PFR3_HUMAN | Serine/threonine-protein phosphatase 2A 56 kDa regulatory subunit delta isoform OS=Homo sapiens GN=PPP2R5D PE=1 SV=1 | 69116.2 | 594 | 7.407 | 4 | 4 | 8 | 8 |
| 1429 | 6.49 | tr|A0A024R329|A0A024R329_HUMAN | GDP-mannose pyrophosphorylase B, isoform CRA_a OS=Homo sapiens GN=GMPPB PE=4 SV=1 | 42621.3 | 387 | 10.59 | 3 | 3 | 11 | 11 |
| 1430 | 6.49 | tr|A8K9V9|A8K9V9_HUMAN | cDNA FLJ76064 OS=Homo sapiens PE=2 SV=1 | 34532.1 | 310 | 21.94 | 4 | 4 | 13 | 13 |
| 1431 | 6.49 | sp|P82909|RT36_HUMAN | 28S ribosomal protein S36, mitochondrial OS=Homo sapiens GN=MRPS36 PE=1 SV=2 | 11466.1 | 103 | 41.75 | 3 | 3 | 6 | 6 |
| 1432 | 6.48 | tr|Q96I63|Q96I63_HUMAN | ATP-dependent zinc metalloprotease YME1L1 OS=Homo sapiens GN=YME1L1 PE=1 SV=1 | 82665.1 | 740 | 8.108 | 4 | 4 | 6 | 6 |
| 1433 | 6.48 | sp|P40222|TXLNA_HUMAN | Alpha-taxilin OS=Homo sapiens GN=TXLNA PE=1 SV=3 | 61890.8 | 546 | 11.72 | 3 | 3 | 13 | 6 |
| 1434 | 6.47 | tr|Q59G70|Q59G70_HUMAN | Mannosyl (Alpha-1,3-)-glycoprotein beta-1,2-N-acetylglucosaminyltransferase variant (Fragment) OS=Homo sapiens PE=2 SV=1 | 53503.8 | 473 | 7.611 | 3 | 3 | 6 | 6 |
| 1435 | 6.47 | tr|A0A024RBX9|A0A024RBX9_HUMAN | Pyruvate dehydrogenase E1 component subunit alpha OS=Homo sapiens GN=PDHA1 PE=4 SV=1 | 43295.3 | 390 | 10.26 | 4 | 4 | 8 | 8 |
| 1436 | 6.46 | tr|Q5U071|Q5U071_HUMAN | High-mobility group box 2 OS=Homo sapiens PE=2 SV=1 | 23904.5 | 208 | 30.29 | 8 | 5 | 22 | 11 |
| 1437 | 6.46 | tr|B7ZMD6|B7ZMD6_HUMAN | IRGQ protein OS=Homo sapiens GN=IRGQ PE=2 SV=1 | 62673.4 | 623 | 5.778 | 3 | 3 | 7 | 7 |
| 1438 | 6.46 | tr|H0Y7A7|H0Y7A7_HUMAN | Calmodulin (Fragment) OS=Homo sapiens GN=CALM2 PE=1 SV=1 | 20761.9 | 187 | 28.34 | 5 | 5 | 21 | 21 |
| 1439 | 6.45 | sp|Q9Y6W5|WASF2_HUMAN | Wiskott-Aldrich syndrome protein family member 2 OS=Homo sapiens GN=WASF2 PE=1 SV=3 | 54283.5 | 498 | 10.04 | 4 | 4 | 22 | 22 |
| 1440 | 6.44 | tr|S4R369|S4R369_HUMAN | 39S ribosomal protein L37, mitochondrial OS=Homo sapiens GN=MRPL37 PE=1 SV=1 | 54952.1 | 483 | 11.39 | 5 | 5 | 8 | 8 |
| 1441 | 6.43 | sp|P08574|CY1_HUMAN | Cytochrome c1, heme protein, mitochondrial OS=Homo sapiens GN=CYC1 PE=1 SV=3 | 35421.6 | 325 | 18.15 | 4 | 4 | 59 | 58 |
| 1442 | 6.42 | tr|B4DY34|B4DY34_HUMAN | cDNA FLJ60194, highly similar to WW domain-binding protein 11 OS=Homo sapiens PE=2 SV=1 | 66832.3 | 607 | 6.26 | 4 | 4 | 6 | 6 |
| 1443 | 6.42 | sp|Q96SB4|SRPK1_HUMAN | SRSF protein kinase 1 OS=Homo sapiens GN=SRPK1 PE=1 SV=2 | 74324.3 | 655 | 5.802 | 3 | 3 | 13 | 13 |
| 1444 | 6.42 | sp|Q04206|TF65_HUMAN | Transcription factor p65 OS=Homo sapiens GN=RELA PE=1 SV=2 | 60218.5 | 551 | 5.989 | 3 | 3 | 9 | 9 |
| 1445 | 6.41 | tr|A0A024RCZ1|A0A024RCZ1_HUMAN | LEM domain containing 2, isoform CRA_a OS=Homo sapiens GN=LEMD2 PE=4 SV=1 | 56974.5 | 503 | 9.543 | 4 | 4 | 7 | 7 |
| 1446 | 6.4 | sp|P46977|STT3A_HUMAN | Dolichyl-diphosphooligosaccharide--protein glycosyltransferase subunit STT3A OS=Homo sapiens GN=STT3A PE=1 SV=2 | 80528.8 | 705 | 8.794 | 7 | 6 | 16 | 15 |
| 1447 | 6.4 | tr|A8K5D8|A8K5D8_HUMAN | cDNA FLJ75934, highly similar to Homo sapiens vacuolar protein sorting 4B (yeast) (VPS4B), mRNA OS=Homo sapiens PE=2 SV=1 | 49230.5 | 444 | 6.532 | 3 | 2 | 10 | 2 |
| 1448 | 6.39 | tr|Q53XS4|Q53XS4_HUMAN | Tyrosine-protein phosphatase non-receptor type OS=Homo sapiens GN=PTPN6 PE=2 SV=1 | 67718.9 | 597 | 6.198 | 3 | 3 | 6 | 6 |
| 1449 | 6.38 | tr|H0YIV4|H0YIV4_HUMAN | Nucleosome assembly protein 1-like 1 (Fragment) OS=Homo sapiens GN=NAP1L1 PE=1 SV=1 | 44714.1 | 385 | 20 | 6 | 4 | 34 | 18 |
| 1450 | 6.37 | tr|Q5VU08|Q5VU08_HUMAN | Adducin 3 (Gamma), isoform CRA_a OS=Homo sapiens GN=ADD3 PE=2 SV=1 | 79154.1 | 706 | 8.357 | 6 | 4 | 19 | 14 |
| 1451 | 6.37 | tr|Q53FP3|Q53FP3_HUMAN | NFS1 nitrogen fixation 1 isoform a variant (Fragment) OS=Homo sapiens PE=2 SV=1 | 50185.2 | 457 | 9.19 | 3 | 3 | 8 | 8 |
| 1452 | 6.37 | sp|O43815|STRN_HUMAN | Striatin OS=Homo sapiens GN=STRN PE=1 SV=4 | 86131.3 | 780 | 8.333 | 4 | 4 | 6 | 6 |
| 1453 | 6.36 | sp|Q96PZ0|PUS7_HUMAN | Pseudouridylate synthase 7 homolog OS=Homo sapiens GN=PUS7 PE=1 SV=2 | 75034.9 | 661 | 9.531 | 4 | 3 | 7 | 6 |
| 1454 | 6.36 | sp|Q8WTS6|SETD7_HUMAN | Histone-lysine N-methyltransferase SETD7 OS=Homo sapiens GN=SETD7 PE=1 SV=1 | 40720.6 | 366 | 15.57 | 4 | 4 | 11 | 11 |
| 1455 | 6.35 | tr|Q549U1|Q549U1_HUMAN | Putative MAPK activating protein OS=Homo sapiens GN=HSU53209 PE=2 SV=1 | 32688.3 | 282 | 13.83 | 5 | 3 | 33 | 27 |
| 1456 | 6.34 | tr|A9UK01|A9UK01_HUMAN | Rho GTPase activating protein OS=Homo sapiens PE=2 SV=1 | 74977.1 | 663 | 8.748 | 4 | 4 | 8 | 8 |
| 1457 | 6.34 | sp|Q86X55|CARM1_HUMAN | Histone-arginine methyltransferase CARM1 OS=Homo sapiens GN=CARM1 PE=1 SV=3 | 65853.2 | 608 | 7.237 | 3 | 3 | 10 | 10 |
| 1458 | 6.33 | tr|H0YKC5|H0YKC5_HUMAN | Deoxyuridine 5'-triphosphate nucleotidohydrolase, mitochondrial (Fragment) OS=Homo sapiens GN=DUT PE=1 SV=1 | 23738.9 | 221 | 26.24 | 4 | 4 | 16 | 15 |
| 1459 | 6.32 | sp|P52788|SPSY_HUMAN | Spermine synthase OS=Homo sapiens GN=SMS PE=1 SV=2 | 41267.9 | 366 | 12.02 | 4 | 4 | 6 | 6 |
| 1460 | 6.29 | tr|F8VUA2|F8VUA2_HUMAN | Charged multivesicular body protein 1a OS=Homo sapiens GN=CHMP1A PE=1 SV=1 | 19531.6 | 181 | 18.23 | 4 | 4 | 10 | 10 |
| 1461 | 6.29 | sp|P40199|CEAM6_HUMAN | Carcinoembryonic antigen-related cell adhesion molecule 6 OS=Homo sapiens GN=CEACAM6 PE=1 SV=3 | 37194.6 | 344 | 21.8 | 4 | 4 | 11 | 11 |
| 1462 | 6.29 | tr|B2RD27|B2RD27_HUMAN | cDNA, FLJ96428, highly similar to Homo sapiens proteasome (prosome, macropain) 26S subunit, non-ATPase, 7 (Mov34 homolog) (PSMD7), mRNA OS=Homo sapiens PE=2 SV=1 | 37011.2 | 324 | 20.99 | 4 | 4 | 13 | 13 |
| 1463 | 6.28 | sp|O95453|PARN_HUMAN | Poly(A)-specific ribonuclease PARN OS=Homo sapiens GN=PARN PE=1 SV=1 | 73450.4 | 639 | 5.634 | 3 | 3 | 9 | 9 |
| 1464 | 6.28 | sp|P36405|ARL3_HUMAN | ADP-ribosylation factor-like protein 3 OS=Homo sapiens GN=ARL3 PE=1 SV=2 | 20455.3 | 182 | 25.82 | 4 | 4 | 6 | 6 |
| 1465 | 6.28 | tr|I3L4C2|I3L4C2_HUMAN | Brain-specific angiogenesis inhibitor 1-associated protein 2 OS=Homo sapiens GN=BAIAP2 PE=1 SV=1 | 61380.7 | 553 | 6.329 | 3 | 3 | 14 | 14 |
| 1466 | 6.27 | tr|H0Y2S1|H0Y2S1_HUMAN | Putative GTP-binding protein 6 OS=Homo sapiens GN=GTPBP6 PE=1 SV=2 | 56897 | 516 | 11.24 | 5 | 4 | 7 | 6 |
| 1467 | 6.26 | tr|A0A024R3S3|A0A024R3S3_HUMAN | Chaperone, ABC1 activity of bc1 complex like (S. pombe), isoform CRA_b OS=Homo sapiens GN=CABC1 PE=4 SV=1 | 71949.5 | 647 | 7.883 | 4 | 4 | 5 | 4 |
| 1468 | 6.26 | sp|P06730|IF4E_HUMAN | Eukaryotic translation initiation factor 4E OS=Homo sapiens GN=EIF4E PE=1 SV=2 | 25097.1 | 217 | 12.44 | 3 | 3 | 7 | 7 |
| 1469 | 6.25 | tr|L0R588|L0R588_HUMAN | Alternative protein C11orf48 OS=Homo sapiens GN=C11orf48 PE=4 SV=1 | 18395.7 | 159 | 30.19 | 4 | 4 | 15 | 15 |
| 1470 | 6.24 | tr|Q53GS0|Q53GS0_HUMAN | G protein-binding protein CRFG variant (Fragment) OS=Homo sapiens PE=2 SV=1 | 73887.8 | 634 | 6.309 | 4 | 4 | 22 | 22 |
| 1471 | 6.24 | tr|B4DS37|B4DS37_HUMAN | cDNA FLJ58258, highly similar to Cyclin G-associated kinase (EC 2.7.11.1) OS=Homo sapiens PE=2 SV=1 | 132143.7 | 1213 | 3.215 | 3 | 3 | 12 | 10 |
| 1472 | 6.24 | sp|Q6NZY4|ZCHC8_HUMAN | Zinc finger CCHC domain-containing protein 8 OS=Homo sapiens GN=ZCCHC8 PE=1 SV=2 | 78576.8 | 707 | 9.477 | 4 | 4 | 12 | 12 |
| 1473 | 6.24 | sp|Q969S3|ZN622_HUMAN | Zinc finger protein 622 OS=Homo sapiens GN=ZNF622 PE=1 SV=1 | 54271.7 | 477 | 11.74 | 4 | 4 | 8 | 8 |
| 1474 | 6.24 | tr|Q53F62|Q53F62_HUMAN | ADP-ribosylation factor GTPase activating protein 1 isoform a variant (Fragment) OS=Homo sapiens PE=2 SV=1 | 44668.6 | 406 | 17.73 | 4 | 4 | 4 | 4 |
| 1475 | 6.23 | sp|Q96RS6|NUDC1_HUMAN | NudC domain-containing protein 1 OS=Homo sapiens GN=NUDCD1 PE=1 SV=2 | 66755.2 | 583 | 9.949 | 4 | 4 | 18 | 18 |
| 1476 | 6.22 | tr|A0A087WZ13|A0A087WZ13_HUMAN | Ribonucleoprotein PTB-binding 1 OS=Homo sapiens GN=RAVER1 PE=1 SV=1 | 77842.7 | 739 | 9.472 | 4 | 4 | 6 | 6 |
| 1477 | 6.21 | sp|Q15050|RRS1_HUMAN | Ribosome biogenesis regulatory protein homolog OS=Homo sapiens GN=RRS1 PE=1 SV=2 | 41193.1 | 365 | 12.6 | 4 | 4 | 14 | 14 |
| 1478 | 6.2 | sp|Q13136|LIPA1_HUMAN | Liprin-alpha-1 OS=Homo sapiens GN=PPFIA1 PE=1 SV=1 | 135777.5 | 1202 | 4.326 | 4 | 4 | 12 | 10 |
| 1479 | 6.2 | tr|Q6NUN2|Q6NUN2_HUMAN | NFKB repressing factor OS=Homo sapiens GN=NKRF PE=2 SV=1 | 77703.4 | 690 | 5.507 | 4 | 4 | 4 | 4 |
| 1480 | 6.19 | tr|Q6FHG1|Q6FHG1_HUMAN | MAP2K3 protein (Fragment) OS=Homo sapiens GN=MAP2K3 PE=2 SV=1 | 39265 | 347 | 12.97 | 5 | 4 | 10 | 9 |
| 1481 | 6.18 | sp|P78318|IGBP1_HUMAN | Immunoglobulin-binding protein 1 OS=Homo sapiens GN=IGBP1 PE=1 SV=1 | 39221.6 | 339 | 17.4 | 4 | 4 | 8 | 8 |
| 1482 | 6.18 | sp|O43709|WBS22_HUMAN | Probable 18S rRNA (guanine-N(7))-methyltransferase OS=Homo sapiens GN=WBSCR22 PE=1 SV=2 | 31880.1 | 281 | 16.01 | 3 | 3 | 8 | 8 |
| 1483 | 6.18 | tr|A0A024R1U2|A0A024R1U2_HUMAN | PHD finger protein 5A, isoform CRA_a OS=Homo sapiens GN=PHF5A PE=4 SV=1 | 12405.3 | 110 | 25.45 | 4 | 4 | 9 | 9 |
| 1484 | 6.17 | tr|B1AQP2|B1AQP2_HUMAN | Prefoldin subunit 2 OS=Homo sapiens GN=PFDN2 PE=2 SV=1 | 16647.7 | 154 | 20.13 | 3 | 3 | 35 | 34 |
| 1485 | 6.16 | tr|A0A024RAQ1|A0A024RAQ1_HUMAN | Cold shock domain protein A, isoform CRA_a OS=Homo sapiens GN=CSDA PE=4 SV=1 | 40089.7 | 372 | 25 | 7 | 1 | 27 | 1 |
| 1486 | 6.16 | tr|Q53HG5|Q53HG5_HUMAN | KIAA0103 variant (Fragment) OS=Homo sapiens PE=2 SV=1 | 34761.1 | 297 | 13.13 | 3 | 3 | 11 | 11 |
| 1487 | 6.14 | tr|B4DWG1|B4DWG1_HUMAN | cDNA FLJ55643, highly similar to SEC23-interacting protein OS=Homo sapiens PE=2 SV=1 | 89706.4 | 789 | 8.238 | 4 | 4 | 5 | 5 |
| 1488 | 6.14 | tr|Q6LET3|Q6LET3_HUMAN | HPRT1 protein (Fragment) OS=Homo sapiens GN=HPRT1 PE=2 SV=1 | 24588.2 | 218 | 15.6 | 3 | 3 | 18 | 18 |
| 1489 | 6.14 | tr|A0A0S2Z3D0|A0A0S2Z3D0_HUMAN | Carbonic anhydrase IX isoform 1 (Fragment) OS=Homo sapiens GN=CA9 PE=2 SV=1 | 49697.4 | 459 | 10.02 | 3 | 3 | 15 | 15 |
| 1490 | 6.13 | sp|O43747|AP1G1_HUMAN | AP-1 complex subunit gamma-1 OS=Homo sapiens GN=AP1G1 PE=1 SV=5 | 91350.5 | 822 | 4.258 | 4 | 4 | 14 | 14 |
| 1491 | 6.13 | tr|J3QRV5|J3QRV5_HUMAN | Lethal(2) giant larvae protein homolog 2 OS=Homo sapiens GN=LLGL2 PE=1 SV=1 | 113375.9 | 1019 | 4.416 | 4 | 4 | 9 | 8 |
| 1492 | 6.12 | tr|A0A024R328|A0A024R328_HUMAN | Protein kinase C delta type OS=Homo sapiens GN=PRKCD PE=3 SV=1 | 77504.4 | 676 | 5.325 | 3 | 3 | 9 | 9 |
| 1494 | 6.11 | sp|Q5TFE4|NT5D1_HUMAN | 5'-nucleotidase domain-containing protein 1 OS=Homo sapiens GN=NT5DC1 PE=1 SV=1 | 51844.4 | 455 | 10.99 | 3 | 3 | 19 | 19 |
| 1495 | 6.11 | sp|O43818|U3IP2_HUMAN | U3 small nucleolar RNA-interacting protein 2 OS=Homo sapiens GN=RRP9 PE=1 SV=1 | 51840.2 | 475 | 6.316 | 3 | 3 | 14 | 14 |
| 1496 | 6.1 | tr|A0A140VJP2|A0A140VJP2_HUMAN | Testicular tissue protein Li 118 OS=Homo sapiens PE=2 SV=1 | 37551.5 | 334 | 14.97 | 3 | 3 | 13 | 13 |
| 1497 | 6.1 | tr|C9JP00|C9JP00_HUMAN | Muscleblind-like protein 1 OS=Homo sapiens GN=MBNL1 PE=1 SV=1 | 37898.3 | 348 | 13.22 | 3 | 3 | 17 | 15 |
| 1498 | 6.09 | tr|A8K9K1|A8K9K1_HUMAN | Calcium-transporting ATPase OS=Homo sapiens PE=2 SV=1 | 109179 | 998 | 9.319 | 8 | 3 | 25 | 4 |
| 1499 | 6.08 | sp|Q5VW32|BROX_HUMAN | BRO1 domain-containing protein BROX OS=Homo sapiens GN=BROX PE=1 SV=1 | 46475.6 | 411 | 11.19 | 3 | 3 | 5 | 5 |
| 1500 | 6.08 | tr|Q1AHP8|Q1AHP8_HUMAN | Hepatopoietin PCn127 OS=Homo sapiens PE=2 SV=1 | 28018.6 | 245 | 6.531 | 3 | 3 | 22 | 22 |
| 1501 | 6.08 | tr|Q9HC03|Q9HC03_HUMAN | Dolichyl-phosphate beta-glucosyltransferase OS=Homo sapiens PE=2 SV=1 | 36963.8 | 324 | 11.73 | 3 | 3 | 8 | 6 |
| 1502 | 6.08 | sp|Q9H3N1|TMX1_HUMAN | Thioredoxin-related transmembrane protein 1 OS=Homo sapiens GN=TMX1 PE=1 SV=1 | 31790.9 | 280 | 11.07 | 3 | 3 | 12 | 12 |
| 1503 | 6.08 | tr|Q53Y06|Q53Y06_HUMAN | ATPase, H+ transporting, lysosomal 31kDa, V1 subunit E isoform 1 OS=Homo sapiens GN=ATP6V1E1 PE=2 SV=1 | 26145.1 | 226 | 14.6 | 3 | 3 | 14 | 14 |
| 1504 | 6.07 | sp|Q9BX40|LS14B_HUMAN | Protein LSM14 homolog B OS=Homo sapiens GN=LSM14B PE=1 SV=1 | 42070.4 | 385 | 15.32 | 5 | 4 | 20 | 12 |
| 1505 | 6.07 | tr|A0A0C4DGX5|A0A0C4DGX5_HUMAN | Ras-related protein Rab-25 OS=Homo sapiens GN=RAB25 PE=1 SV=1 | 28288.9 | 256 | 20.7 | 5 | 4 | 33 | 27 |
| 1506 | 6.07 | sp|Q9UH65|SWP70_HUMAN | Switch-associated protein 70 OS=Homo sapiens GN=SWAP70 PE=1 SV=1 | 68997.2 | 585 | 5.128 | 3 | 3 | 5 | 5 |
| 1507 | 6.07 | tr|B4DR61|B4DR61_HUMAN | Protein transport protein Sec61 subunit alpha isoform 1 OS=Homo sapiens GN=SEC61A1 PE=1 SV=1 | 52949 | 482 | 4.357 | 3 | 3 | 8 | 8 |
| 1508 | 6.07 | sp|O43719|HTSF1_HUMAN | HIV Tat-specific factor 1 OS=Homo sapiens GN=HTATSF1 PE=1 SV=1 | 85852 | 755 | 6.358 | 3 | 3 | 7 | 7 |
| 1509 | 6.07 | tr|B4DLN1|B4DLN1_HUMAN | Uncharacterized protein OS=Homo sapiens PE=2 SV=1 | 48098.9 | 442 | 11.54 | 3 | 2 | 9 | 5 |
| 1510 | 6.06 | sp|Q13619|CUL4A_HUMAN | Cullin-4A OS=Homo sapiens GN=CUL4A PE=1 SV=3 | 87679.6 | 759 | 4.348 | 3 | 1 | 16 | 6 |
| 1511 | 6.06 | tr|A0A024R5J4|A0A024R5J4_HUMAN | Slingshot homolog 3 (Drosophila), isoform CRA_f OS=Homo sapiens GN=SSH3 PE=4 SV=1 | 72995 | 659 | 4.552 | 3 | 3 | 15 | 15 |
| 1512 | 6.06 | tr|M0QYH2|M0QYH2_HUMAN | Bifunctional polynucleotide phosphatase/kinase OS=Homo sapiens GN=PNKP PE=1 SV=1 | 53640 | 490 | 7.959 | 3 | 3 | 18 | 18 |
| 1513 | 6.05 | sp|P53634|CATC_HUMAN | Dipeptidyl peptidase 1 OS=Homo sapiens GN=CTSC PE=1 SV=2 | 51853.5 | 463 | 12.1 | 4 | 4 | 16 | 16 |
| 1514 | 6.05 | tr|Q8NBL9|Q8NBL9_HUMAN | cDNA PSEC0119 fis, clone PLACE1002376, highly similar to GPI transamidase component PIG-S OS=Homo sapiens PE=2 SV=1 | 61643.5 | 555 | 10.63 | 4 | 4 | 9 | 9 |
| 1515 | 6.05 | tr|B4DYK6|B4DYK6_HUMAN | cDNA FLJ56887, highly similar to Homo sapiens guanine nucleotide binding protein-like 1 (GNL1), mRNA OS=Homo sapiens PE=2 SV=1 | 68255.7 | 605 | 8.595 | 4 | 4 | 7 | 7 |
| 1516 | 6.05 | tr|Q6IBU0|Q6IBU0_HUMAN | EIF5 protein OS=Homo sapiens GN=EIF5 PE=2 SV=1 | 49151.2 | 431 | 7.193 | 3 | 3 | 16 | 16 |
| 1517 | 6.04 | tr|B4DH17|B4DH17_HUMAN | cDNA FLJ59298, highly similar to Eukaryotic translation initiation factor 4 gamma 3 OS=Homo sapiens PE=2 SV=1 | 134148.2 | 1189 | 3.616 | 4 | 3 | 14 | 8 |
| 1518 | 6.04 | tr|A8K651|A8K651_HUMAN | cDNA FLJ75700, highly similar to Homo sapiens complement component 1, q subcomponent binding protein (C1QBP), nuclear gene encoding mitochondrial protein, mRNA OS=Homo sapiens PE=2 SV=1 | 31379.9 | 282 | 12.77 | 3 | 3 | 60 | 60 |
| 1519 | 6.04 | tr|E9KL42|E9KL42_HUMAN | Epididymis secretory sperm binding protein Li 188n OS=Homo sapiens PE=2 SV=1 | 46277 | 421 | 14.01 | 4 | 4 | 13 | 13 |
| 1520 | 6.04 | sp|P36551|HEM6_HUMAN | Oxygen-dependent coproporphyrinogen-III oxidase, mitochondrial OS=Homo sapiens GN=CPOX PE=1 SV=3 | 50151.6 | 454 | 8.15 | 3 | 3 | 3 | 3 |
| 1521 | 6.04 | tr|A0A024QYX3|A0A024QYX3_HUMAN | RNA binding motif (RNP1, RRM) protein 3, isoform CRA_c OS=Homo sapiens GN=RBM3 PE=4 SV=1 | 17170.3 | 157 | 25.48 | 3 | 3 | 41 | 41 |
| 1522 | 6.03 | sp|P55081|MFAP1_HUMAN | Microfibrillar-associated protein 1 OS=Homo sapiens GN=MFAP1 PE=1 SV=2 | 51958.1 | 439 | 7.973 | 3 | 3 | 7 | 7 |
| 1523 | 6.03 | tr|B0UZZ8|B0UZZ8_HUMAN | Chromosome 6 open reading frame 11 OS=Homo sapiens GN=C6orf11 PE=2 SV=1 | 68040.5 | 610 | 9.344 | 3 | 3 | 8 | 8 |
| 1524 | 6.03 | tr|H7BY58|H7BY58_HUMAN | Protein-L-isoaspartate O-methyltransferase OS=Homo sapiens GN=PCMT1 PE=1 SV=1 | 30357.7 | 286 | 18.18 | 3 | 3 | 14 | 14 |
| 1525 | 6.03 | sp|Q16222|UAP1_HUMAN | UDP-N-acetylhexosamine pyrophosphorylase OS=Homo sapiens GN=UAP1 PE=1 SV=3 | 58768.7 | 522 | 10.54 | 4 | 4 | 20 | 20 |
| 1526 | 6.03 | tr|A8K556|A8K556_HUMAN | cDNA FLJ78217 OS=Homo sapiens PE=2 SV=1 | 40284.7 | 357 | 17.09 | 4 | 4 | 10 | 10 |
| 1527 | 6.03 | tr|Q59EL4|Q59EL4_HUMAN | PRPF4 protein variant (Fragment) OS=Homo sapiens PE=2 SV=1 | 60021.5 | 537 | 7.635 | 3 | 3 | 12 | 12 |
| 1528 | 6.03 | tr|Q658U3|Q658U3_HUMAN | Putative uncharacterized protein DKFZp666D023 (Fragment) OS=Homo sapiens GN=DKFZp666D023 PE=2 SV=1 | 18722.1 | 169 | 25.44 | 3 | 3 | 4 | 4 |
| 1529 | 6.03 | sp|P14854|CX6B1_HUMAN | Cytochrome c oxidase subunit 6B1 OS=Homo sapiens GN=COX6B1 PE=1 SV=2 | 10192.3 | 86 | 34.88 | 3 | 3 | 17 | 17 |
| 1530 | 6.02 | sp|O75489|NDUS3_HUMAN | NADH dehydrogenase [ubiquinone] iron-sulfur protein 3, mitochondrial OS=Homo sapiens GN=NDUFS3 PE=1 SV=1 | 30241.2 | 264 | 19.32 | 4 | 3 | 35 | 34 |
| 1531 | 6.02 | tr|Q9H4E3|Q9H4E3_HUMAN | DEAD box protein OS=Homo sapiens GN=E4-DBP PE=2 SV=1 | 50635 | 455 | 7.912 | 3 | 3 | 14 | 14 |
| 1532 | 6.02 | tr|A0A024RAC6|A0A024RAC6_HUMAN | Transcription elongation factor B (SIII), polypeptide 3 (110kDa, elongin A), isoform CRA_a OS=Homo sapiens GN=TCEB3 PE=1 SV=1 | 87229.3 | 772 | 4.922 | 3 | 3 | 7 | 7 |
| 1533 | 6.02 | tr|Q0VDC6|Q0VDC6_HUMAN | FKBP1A protein OS=Homo sapiens GN=FKBP1A PE=2 SV=1 | 15688.9 | 145 | 21.38 | 3 | 3 | 50 | 50 |
| 1534 | 6.02 | tr|A0A024R994|A0A024R994_HUMAN | Copine III, isoform CRA_a OS=Homo sapiens GN=CPNE3 PE=4 SV=1 | 60130.2 | 537 | 8.194 | 4 | 4 | 15 | 13 |
| 1535 | 6.02 | tr|Q6IBK3|Q6IBK3_HUMAN | SCAMP2 protein OS=Homo sapiens GN=SCAMP2 PE=2 SV=1 | 36535.5 | 329 | 13.68 | 3 | 3 | 27 | 27 |
| 1536 | 6.02 | sp|Q9BXW7|CECR5_HUMAN | Cat eye syndrome critical region protein 5 OS=Homo sapiens GN=CECR5 PE=1 SV=1 | 46321.1 | 423 | 10.17 | 3 | 3 | 4 | 4 |
| 1537 | 6.01 | tr|H7BXK9|H7BXK9_HUMAN | ATP-binding cassette sub-family B member 6, mitochondrial (Fragment) OS=Homo sapiens GN=ABCB6 PE=1 SV=1 | 77491.8 | 690 | 8.406 | 3 | 3 | 3 | 3 |
| 1538 | 6.01 | sp|O15173|PGRC2_HUMAN | Membrane-associated progesterone receptor component 2 OS=Homo sapiens GN=PGRMC2 PE=1 SV=1 | 23818.2 | 223 | 21.08 | 4 | 3 | 9 | 8 |
| 1539 | 6.01 | tr|L7RXH5|L7RXH5_HUMAN | Mitogen-activated protein kinase OS=Homo sapiens GN=MAPK3 PE=2 SV=1 | 43135.2 | 379 | 7.652 | 3 | 3 | 12 | 8 |
| 1540 | 6.01 | tr|Q54A15|Q54A15_HUMAN | DTGCU2 OS=Homo sapiens GN=DTGCU2 PE=2 SV=1 | 77408 | 677 | 6.204 | 3 | 3 | 7 | 7 |
| 1541 | 6.01 | tr|E9PR17|E9PR17_HUMAN | CD59 glycoprotein OS=Homo sapiens GN=CD59 PE=1 SV=1 | 14528.6 | 130 | 26.15 | 3 | 3 | 8 | 8 |
| 1542 | 6.01 | sp|Q86VR7|VS10L_HUMAN | V-set and immunoglobulin domain-containing protein 10-like OS=Homo sapiens GN=VSIG10L PE=2 SV=2 | 91624 | 867 | 4.96 | 3 | 3 | 12 | 12 |
| 1543 | 6.01 | tr|A0A024R4J8|A0A024R4J8_HUMAN | Kallikrein 6 (Neurosin, zyme), isoform CRA_b OS=Homo sapiens GN=KLK6 PE=3 SV=1 | 26855.5 | 244 | 22.95 | 4 | 4 | 18 | 18 |
| 1544 | 6.01 | tr|G3V2S9|G3V2S9_HUMAN | SRA stem-loop-interacting RNA-binding protein, mitochondrial OS=Homo sapiens GN=SLIRP PE=1 SV=1 | 13877.6 | 124 | 30.65 | 3 | 3 | 25 | 25 |
| 1545 | 6 | tr|M0QY43|M0QY43_HUMAN | Myosin-14 (Fragment) OS=Homo sapiens GN=MYH14 PE=1 SV=7 | 113853 | 1003 | 40.28 | 42 | 3 | 328 | 21 |
| 1546 | 6 | tr|A0A024R163|A0A024R163_HUMAN | ROD1 regulator of differentiation 1 (S. pombe), isoform CRA_b OS=Homo sapiens GN=ROD1 PE=4 SV=1 | 59689 | 552 | 19.57 | 9 | 4 | 40 | 5 |
| 1547 | 6 | sp|Q8NFU3|TSTD1_HUMAN | Thiosulfate sulfurtransferase/rhodanese-like domain-containing protein 1 OS=Homo sapiens GN=TSTD1 PE=1 SV=3 | 12530.1 | 115 | 33.04 | 4 | 3 | 8 | 7 |
| 1548 | 6 | tr|H0YC42|H0YC42_HUMAN | Uncharacterized protein OS=Homo sapiens PE=4 SV=2 | 31169.9 | 278 | 14.75 | 4 | 2 | 13 | 10 |
| 1549 | 6 | tr|J3KSY2|J3KSY2_HUMAN | Galectin OS=Homo sapiens GN=LGALS9C PE=4 SV=1 | 35878.9 | 323 | 10.84 | 4 | 3 | 15 | 13 |
| 1550 | 6 | tr|Q6IBH0|Q6IBH0_HUMAN | SLC25A11 protein OS=Homo sapiens GN=SLC25A11 PE=2 SV=1 | 34061.4 | 314 | 13.06 | 3 | 3 | 14 | 14 |
| 1551 | 6 | sp|P62304|RUXE_HUMAN | Small nuclear ribonucleoprotein E OS=Homo sapiens GN=SNRPE PE=1 SV=1 | 10803.6 | 92 | 29.35 | 3 | 3 | 45 | 45 |
| 1552 | 6 | tr|Q69YG1|Q69YG1_HUMAN | Myotrophin OS=Homo sapiens GN=DKFZp761E1322 PE=2 SV=1 | 12894.7 | 118 | 33.05 | 3 | 3 | 32 | 32 |
| 1553 | 6 | tr|A0A024R179|A0A024R179_HUMAN | Nuclear cap binding protein subunit 1, 80kDa, isoform CRA_a OS=Homo sapiens GN=NCBP1 PE=4 SV=1 | 91838.6 | 790 | 5.316 | 3 | 3 | 13 | 13 |
| 1554 | 6 | sp|O43678|NDUA2_HUMAN | NADH dehydrogenase [ubiquinone] 1 alpha subcomplex subunit 2 OS=Homo sapiens GN=NDUFA2 PE=1 SV=3 | 10921.4 | 99 | 41.41 | 3 | 3 | 23 | 23 |
| 1555 | 6 | tr|J3KNF8|J3KNF8_HUMAN | Cytochrome b5 type B OS=Homo sapiens GN=CYB5B PE=1 SV=1 | 16694.4 | 150 | 41.33 | 3 | 3 | 8 | 8 |
| 1556 | 6 | sp|Q86U42|PABP2_HUMAN | Polyadenylate-binding protein 2 OS=Homo sapiens GN=PABPN1 PE=1 SV=3 | 32748.8 | 306 | 22.88 | 3 | 3 | 24 | 24 |
| 1557 | 6 | tr|A0A024RB14|A0A024RB14_HUMAN | HCG20716 OS=Homo sapiens GN=RPS26 PE=4 SV=1 | 13015.3 | 115 | 20.87 | 3 | 3 | 35 | 35 |
| 1558 | 6 | sp|Q53FV1|ORML2_HUMAN | ORM1-like protein 2 OS=Homo sapiens GN=ORMDL2 PE=1 SV=2 | 17363.3 | 153 | 28.76 | 3 | 3 | 7 | 7 |
| 1559 | 6 | tr|A8K818|A8K818_HUMAN | cDNA FLJ75784, highly similar to Homo sapiens CD3E antigen, epsilon polypeptide associated protein (CD3EAP), mRNA OS=Homo sapiens PE=2 SV=1 | 54958.2 | 510 | 9.804 | 3 | 3 | 15 | 15 |
| 1560 | 6 | tr|Q53F59|Q53F59_HUMAN | GTP cyclohydrolase I feedback regulatory protein variant (Fragment) OS=Homo sapiens PE=2 SV=1 | 9679.1 | 84 | 50 | 3 | 3 | 10 | 10 |
| 1561 | 6 | sp|P60468|SC61B_HUMAN | Protein transport protein Sec61 subunit beta OS=Homo sapiens GN=SEC61B PE=1 SV=2 | 9974.4 | 96 | 37.5 | 3 | 3 | 10 | 10 |
| 1562 | 5.96 | sp|P53602|MVD1_HUMAN | Diphosphomevalonate decarboxylase OS=Homo sapiens GN=MVD PE=1 SV=1 | 43404.1 | 400 | 11 | 4 | 3 | 18 | 17 |
| 1563 | 5.96 | tr|A8K5Q1|A8K5Q1_HUMAN | cDNA FLJ77548, highly similar to Homo sapiens bin3, bicoid-interacting 3, homolog (Drosophila) (BCDIN3), mRNA OS=Homo sapiens PE=2 SV=1 | 74326.6 | 689 | 5.515 | 3 | 3 | 6 | 6 |
| 1564 | 5.93 | sp|Q96T88|UHRF1_HUMAN | E3 ubiquitin-protein ligase UHRF1 OS=Homo sapiens GN=UHRF1 PE=1 SV=1 | 89813.2 | 793 | 4.54 | 3 | 3 | 12 | 12 |
| 1565 | 5.93 | tr|Q5HY81|Q5HY81_HUMAN | Ubiquitin-like protein 4A OS=Homo sapiens GN=UBL4A PE=1 SV=2 | 20550.4 | 180 | 16.11 | 3 | 3 | 12 | 12 |
| 1566 | 5.93 | tr|A8K670|A8K670_HUMAN | Nitric oxide synthase-interacting protein OS=Homo sapiens PE=2 SV=1 | 33183.7 | 301 | 18.27 | 3 | 3 | 6 | 6 |
| 1567 | 5.93 | tr|A0A0A0MRT6|A0A0A0MRT6_HUMAN | Abl interactor 1 OS=Homo sapiens GN=ABI1 PE=1 SV=1 | 51769.4 | 475 | 9.053 | 3 | 3 | 14 | 14 |
| 1568 | 5.92 | sp|Q71UI9|H2AV_HUMAN | Histone H2A.V OS=Homo sapiens GN=H2AFV PE=1 SV=3 | 13508.6 | 128 | 31.25 | 6 | 3 | 115 | 26 |
| 1569 | 5.92 | tr|G1UI17|G1UI17_HUMAN | Glycogen debranching enzyme (Fragment) OS=Homo sapiens GN=AGL PE=2 SV=1 | 143721.1 | 1262 | 4.2 | 4 | 3 | 11 | 10 |
| 1570 | 5.92 | tr|A0A140VJC9|A0A140VJC9_HUMAN | Lysophospholipase II, isoform CRA_f OS=Homo sapiens GN=LYPLA2 PE=2 SV=1 | 24736.7 | 231 | 28.57 | 6 | 5 | 26 | 25 |
| 1571 | 5.92 | tr|A0AV58|A0AV58_HUMAN | Striatin, calmodulin binding protein 3 OS=Homo sapiens GN=STRN3 PE=2 SV=1 | 77717.1 | 713 | 6.452 | 3 | 3 | 4 | 4 |
| 1572 | 5.92 | sp|P98088|MUC5A_HUMAN | Mucin-5AC OS=Homo sapiens GN=MUC5AC PE=1 SV=4 | 585563.9 | 5654 | 3.944 | 6 | 6 | 18 | 18 |
| 1573 | 5.92 | sp|O60220|TIM8A_HUMAN | Mitochondrial import inner membrane translocase subunit Tim8 A OS=Homo sapiens GN=TIMM8A PE=1 SV=1 | 10998.3 | 97 | 54.64 | 3 | 3 | 11 | 11 |
| 1574 | 5.91 | sp|Q9H2U2|IPYR2_HUMAN | Inorganic pyrophosphatase 2, mitochondrial OS=Homo sapiens GN=PPA2 PE=1 SV=2 | 37919.9 | 334 | 18.56 | 4 | 4 | 19 | 15 |
| 1575 | 5.91 | sp|Q14008|CKAP5_HUMAN | Cytoskeleton-associated protein 5 OS=Homo sapiens GN=CKAP5 PE=1 SV=3 | 225492.5 | 2032 | 4.134 | 6 | 6 | 9 | 9 |
| 1576 | 5.91 | tr|J3KNF4|J3KNF4_HUMAN | Copper chaperone for superoxide dismutase OS=Homo sapiens GN=CCS PE=1 SV=1 | 27086.3 | 255 | 15.29 | 4 | 4 | 9 | 9 |
| 1577 | 5.89 | tr|Q6IAX6|Q6IAX6_HUMAN | 3'-phosphoadenosine 5'-phosphosulfate synthase 1 OS=Homo sapiens GN=PAPSS1 PE=2 SV=1 | 70832.7 | 624 | 9.776 | 5 | 4 | 7 | 5 |
| 1578 | 5.89 | tr|B4DY46|B4DY46_HUMAN | cDNA FLJ53447, highly similar to Syntaxin-binding protein 2 OS=Homo sapiens PE=2 SV=1 | 62698.6 | 559 | 12.16 | 4 | 4 | 9 | 9 |
| 1579 | 5.89 | tr|A0A024RDW4|A0A024RDW4_HUMAN | Uncharacterized protein OS=Homo sapiens GN=FLJ10154 PE=4 SV=1 | 33216 | 273 | 13.19 | 4 | 3 | 10 | 8 |
| 1580 | 5.89 | tr|Q59EL2|Q59EL2_HUMAN | COP9 constitutive photomorphogenic homolog subunit 2 variant (Fragment) OS=Homo sapiens PE=2 SV=1 | 52532.3 | 451 | 12.86 | 4 | 4 | 9 | 9 |
| 1581 | 5.88 | sp|O60502|OGA_HUMAN | Protein O-GlcNAcase OS=Homo sapiens GN=MGEA5 PE=1 SV=2 | 102914.2 | 916 | 5.24 | 4 | 4 | 9 | 9 |
| 1582 | 5.86 | tr|A0A024R4S1|A0A024R4S1_HUMAN | Epsin 1, isoform CRA_a OS=Homo sapiens GN=EPN1 PE=4 SV=1 | 60292.7 | 576 | 9.896 | 3 | 3 | 13 | 13 |
| 1583 | 5.85 | tr|Q3SYF1|Q3SYF1_HUMAN | Sorting nexin 12 OS=Homo sapiens GN=SNX12 PE=2 SV=1 | 18884.4 | 162 | 25.93 | 4 | 3 | 22 | 18 |
| 1584 | 5.85 | tr|A0A024R482|A0A024R482_HUMAN | GDP-mannose pyrophosphorylase A, isoform CRA_a OS=Homo sapiens GN=GMPPA PE=4 SV=1 | 46290.8 | 420 | 9.286 | 3 | 3 | 4 | 4 |
| 1585 | 5.84 | tr|Q96FT4|Q96FT4_HUMAN | Papillary renal cell carcinoma (Translocation-associated) OS=Homo sapiens GN=PRCC PE=2 SV=1 | 52421.3 | 491 | 19.14 | 4 | 4 | 4 | 4 |
| 1586 | 5.83 | tr|Q541A5|Q541A5_HUMAN | Ubiquitin fusion degradation 1 like (Yeast), isoform CRA_b OS=Homo sapiens GN=ufd1 PE=2 SV=1 | 34500.1 | 307 | 15.64 | 4 | 4 | 10 | 10 |
| 1587 | 5.8 | tr|Q6FI54|Q6FI54_HUMAN | RAB5B protein OS=Homo sapiens GN=RAB5B PE=2 SV=1 | 23707.6 | 215 | 31.63 | 6 | 3 | 17 | 6 |
| 1588 | 5.78 | tr|A8K9X5|A8K9X5_HUMAN | cDNA FLJ76472, highly similar to Homo sapiens Fas (TNFRSF6) associated factor 1 (FAF1), transcript variant 1, mRNA OS=Homo sapiens PE=2 SV=1 | 73919.4 | 650 | 5.538 | 3 | 3 | 5 | 5 |
| 1589 | 5.78 | sp|Q9NTJ5|SAC1_HUMAN | Phosphatidylinositide phosphatase SAC1 OS=Homo sapiens GN=SACM1L PE=1 SV=2 | 66966.4 | 587 | 6.644 | 3 | 3 | 10 | 10 |
| 1590 | 5.78 | sp|Q9H773|DCTP1_HUMAN | dCTP pyrophosphatase 1 OS=Homo sapiens GN=DCTPP1 PE=1 SV=1 | 18680.6 | 170 | 28.24 | 3 | 3 | 9 | 9 |
| 1591 | 5.77 | sp|Q9UNE7|CHIP_HUMAN | E3 ubiquitin-protein ligase CHIP OS=Homo sapiens GN=STUB1 PE=1 SV=2 | 34855.9 | 303 | 12.87 | 4 | 4 | 15 | 15 |
| 1592 | 5.77 | sp|P17706|PTN2_HUMAN | Tyrosine-protein phosphatase non-receptor type 2 OS=Homo sapiens GN=PTPN2 PE=1 SV=2 | 48472.9 | 415 | 11.08 | 3 | 3 | 6 | 6 |
| 1593 | 5.77 | tr|A0A0C4DFN3|A0A0C4DFN3_HUMAN | Monoglyceride lipase OS=Homo sapiens GN=MGLL PE=1 SV=1 | 34292.2 | 313 | 14.7 | 3 | 3 | 3 | 3 |
| 1594 | 5.75 | tr|X5D2R7|X5D2R7_HUMAN | Proteasome subunit beta type OS=Homo sapiens GN=PSM8 PE=1 SV=1 | 30354.1 | 276 | 17.39 | 4 | 3 | 13 | 7 |
| 1595 | 5.74 | tr|A0A024R6S1|A0A024R6S1_HUMAN | DnaJ (Hsp40) homolog, subfamily A, member 2, isoform CRA_a OS=Homo sapiens GN=DNAJA2 PE=3 SV=1 | 45745.4 | 412 | 8.738 | 4 | 4 | 4 | 4 |
| 1596 | 5.74 | sp|Q9BV79|MECR_HUMAN | Trans-2-enoyl-CoA reductase, mitochondrial OS=Homo sapiens GN=MECR PE=1 SV=2 | 40461.4 | 373 | 8.579 | 3 | 3 | 4 | 4 |
| 1597 | 5.73 | tr|Q53GY9|Q53GY9_HUMAN | Cation-dependent mannose-6-phosphate receptor variant (Fragment) OS=Homo sapiens PE=2 SV=1 | 31019.1 | 277 | 18.05 | 4 | 3 | 13 | 11 |
| 1598 | 5.72 | sp|Q09028|RBBP4_HUMAN | Histone-binding protein RBBP4 OS=Homo sapiens GN=RBBP4 PE=1 SV=3 | 47655.3 | 425 | 22.82 | 10 | 4 | 60 | 32 |
| 1599 | 5.72 | tr|B2R6C4|B2R6C4_HUMAN | Receptor expression-enhancing protein OS=Homo sapiens PE=2 SV=1 | 21116.4 | 185 | 10.27 | 3 | 3 | 8 | 8 |
| 1600 | 5.72 | tr|A0A024R0T1|A0A024R0T1_HUMAN | Short-chain dehydrogenase/reductase, isoform CRA_b OS=Homo sapiens GN=MGC4172 PE=3 SV=1 | 28308.1 | 260 | 15.77 | 3 | 3 | 8 | 8 |
| 1601 | 5.69 | tr|Q9UPE4|Q9UPE4_HUMAN | Mitochondrial import inner membrane translocase subunit TIM44 OS=Homo sapiens GN=hTIM44 PE=2 SV=1 | 47365.8 | 415 | 8.434 | 3 | 3 | 7 | 7 |
| 1602 | 5.68 | sp|Q5TDH0|DDI2_HUMAN | Protein DDI1 homolog 2 OS=Homo sapiens GN=DDI2 PE=1 SV=1 | 44522.1 | 399 | 10.03 | 3 | 3 | 15 | 15 |
| 1603 | 5.68 | sp|Q9H2U1|DHX36_HUMAN | ATP-dependent RNA helicase DHX36 OS=Homo sapiens GN=DHX36 PE=1 SV=2 | 114759.3 | 1008 | 4.563 | 3 | 3 | 7 | 7 |
| 1604 | 5.67 | tr|G3V198|G3V198_HUMAN | Nuclear pore complex protein Nup160 (Fragment) OS=Homo sapiens GN=NUP160 PE=1 SV=2 | 148916.7 | 1314 | 2.359 | 3 | 3 | 10 | 10 |
| 1605 | 5.66 | tr|B3KNI8|B3KNI8_HUMAN | cDNA FLJ14670 fis, clone NT2RP2003272, highly similar to Ubiquilin-1 OS=Homo sapiens PE=2 SV=1 | 62546.6 | 589 | 9.677 | 4 | 3 | 19 | 9 |
| 1606 | 5.66 | sp|O14776|TCRG1_HUMAN | Transcription elongation regulator 1 OS=Homo sapiens GN=TCERG1 PE=1 SV=2 | 123900.1 | 1098 | 4.007 | 5 | 5 | 6 | 6 |
| 1607 | 5.65 | sp|O75475|PSIP1_HUMAN | PC4 and SFRS1-interacting protein OS=Homo sapiens GN=PSIP1 PE=1 SV=1 | 60103 | 530 | 9.623 | 6 | 6 | 12 | 11 |
| 1608 | 5.64 | tr|B3KUN1|B3KUN1_HUMAN | Serine/threonine-protein phosphatase OS=Homo sapiens GN=PPP2CA PE=2 SV=1 | 35593.9 | 309 | 14.24 | 3 | 3 | 14 | 14 |
| 1609 | 5.64 | tr|Q53FE5|Q53FE5_HUMAN | Putative uncharacterized protein (Fragment) OS=Homo sapiens PE=2 SV=1 | 28342.7 | 263 | 18.25 | 3 | 3 | 4 | 4 |
| 1610 | 5.63 | sp|P51648|AL3A2_HUMAN | Fatty aldehyde dehydrogenase OS=Homo sapiens GN=ALDH3A2 PE=1 SV=1 | 54847.4 | 485 | 9.278 | 5 | 3 | 21 | 12 |
| 1611 | 5.63 | sp|Q9NQ55|SSF1_HUMAN | Suppressor of SWI4 1 homolog OS=Homo sapiens GN=PPAN PE=2 SV=1 | 53193.6 | 473 | 9.725 | 4 | 4 | 13 | 13 |
| 1612 | 5.62 | tr|B4DP80|B4DP80_HUMAN | NAD(P)H-hydrate epimerase OS=Homo sapiens GN=APOA1BP PE=2 SV=1 | 33627.5 | 307 | 9.446 | 3 | 3 | 9 | 9 |
| 1613 | 5.61 | tr|U3KQ56|U3KQ56_HUMAN | Glyoxylate reductase/hydroxypyruvate reductase OS=Homo sapiens GN=GRHPR PE=1 SV=1 | 38695.1 | 358 | 14.25 | 3 | 3 | 14 | 14 |
| 1614 | 5.61 | tr|A0A024R6X2|A0A024R6X2_HUMAN | Core-binding factor, beta subunit, isoform CRA_b OS=Homo sapiens GN=CBFB PE=4 SV=1 | 21508 | 182 | 18.13 | 3 | 3 | 10 | 9 |
| 1615 | 5.6 | tr|A0A024R7W9|A0A024R7W9_HUMAN | COP9 constitutive photomorphogenic homolog subunit 5 (Arabidopsis), isoform CRA_a OS=Homo sapiens GN=COPS5 PE=2 SV=1 | 37578.6 | 334 | 11.98 | 4 | 4 | 9 | 9 |
| 1616 | 5.59 | tr|B7Z6M0|B7Z6M0_HUMAN | cDNA FLJ56370, highly similar to Homo sapiens FK506 binding protein 8, 38kDa (FKBP8), mRNA OS=Homo sapiens PE=2 SV=1 | 47171.1 | 441 | 8.844 | 3 | 3 | 11 | 11 |
| 1617 | 5.58 | tr|B2R5M9|B2R5M9_HUMAN | cDNA, FLJ92537, highly similar to Homo sapiens procollagen-lysine, 2-oxoglutarate 5-dioxygenase (lysine hydroxylase, Ehlers-Danlos syndrome type VI) (PLOD), mRNA OS=Homo sapiens PE=2 SV=1 | 83503.5 | 727 | 5.915 | 3 | 3 | 8 | 8 |
| 1618 | 5.58 | tr|C9JQ41|C9JQ41_HUMAN | Coiled-coil domain-containing protein 58 OS=Homo sapiens GN=CCDC58 PE=1 SV=1 | 15296.5 | 130 | 38.46 | 4 | 4 | 9 | 9 |
| 1619 | 5.58 | sp|P49903|SPS1_HUMAN | Selenide, water dikinase 1 OS=Homo sapiens GN=SEPHS1 PE=1 SV=2 | 42910.3 | 392 | 16.84 | 4 | 4 | 12 | 12 |
| 1620 | 5.57 | tr|B3KWI1|B3KWI1_HUMAN | cDNA FLJ43111 fis, clone CTONG2025900, highly similar to Sterile alpha motif domain-containing protein 9 OS=Homo sapiens PE=2 SV=1 | 87589.8 | 754 | 5.438 | 3 | 3 | 3 | 3 |
| 1621 | 5.57 | sp|Q16186|ADRM1_HUMAN | Proteasomal ubiquitin receptor ADRM1 OS=Homo sapiens GN=ADRM1 PE=1 SV=2 | 42152.8 | 407 | 10.32 | 3 | 3 | 8 | 8 |
| 1622 | 5.56 | tr|D3YTB5|D3YTB5_HUMAN | Interleukin-1 receptor-associated kinase 1 OS=Homo sapiens GN=IRAK1 PE=1 SV=1 | 76059.3 | 708 | 5.791 | 3 | 3 | 8 | 8 |
| 1623 | 5.56 | tr|Q5T4U5|Q5T4U5_HUMAN | Acyl-Coenzyme A dehydrogenase, C-4 to C-12 straight chain, isoform CRA_a OS=Homo sapiens GN=ACADM PE=1 SV=1 | 50270.2 | 454 | 11.67 | 4 | 4 | 9 | 9 |
| 1624 | 5.55 | sp|Q96C23|GALM_HUMAN | Aldose 1-epimerase OS=Homo sapiens GN=GALM PE=1 SV=1 | 37765.4 | 342 | 9.942 | 3 | 3 | 5 | 5 |
| 1625 | 5.53 | tr|D0EKE5|D0EKE5_HUMAN | Aryl hydrocarbon receptor interacting protein OS=Homo sapiens GN=AIP PE=4 SV=1 | 38488.8 | 337 | 13.65 | 3 | 3 | 24 | 23 |
| 1626 | 5.53 | tr|A0A024R6K3|A0A024R6K3_HUMAN | SET domain containing 3, isoform CRA_a OS=Homo sapiens GN=SETD3 PE=4 SV=1 | 67256.5 | 594 | 7.576 | 4 | 3 | 6 | 5 |
| 1627 | 5.52 | sp|Q9ULC4|MCTS1_HUMAN | Malignant T-cell-amplified sequence 1 OS=Homo sapiens GN=MCTS1 PE=1 SV=1 | 20555.2 | 181 | 20.99 | 4 | 4 | 25 | 25 |
| 1628 | 5.52 | tr|B3KM21|B3KM21_HUMAN | Family with sequence similarity 36, member A, isoform CRA_a OS=Homo sapiens GN=FAM36A PE=2 SV=1 | 13291.1 | 118 | 33.05 | 3 | 3 | 3 | 3 |
| 1629 | 5.5 | tr|Q59EN5|Q59EN5_HUMAN | Prosaposin variant (Fragment) OS=Homo sapiens PE=2 SV=1 | 58726.7 | 530 | 5.849 | 4 | 4 | 12 | 12 |
| 1630 | 5.5 | tr|A0A0C4DFR6|A0A0C4DFR6_HUMAN | Protein SEC13 homolog OS=Homo sapiens GN=SEC13 PE=1 SV=1 | 35856.7 | 325 | 18.46 | 3 | 3 | 17 | 17 |
| 1631 | 5.49 | sp|Q8TDD1|DDX54_HUMAN | ATP-dependent RNA helicase DDX54 OS=Homo sapiens GN=DDX54 PE=1 SV=2 | 98594 | 881 | 3.405 | 3 | 3 | 5 | 5 |
| 1632 | 5.48 | sp|Q9HCD5|NCOA5_HUMAN | Nuclear receptor coactivator 5 OS=Homo sapiens GN=NCOA5 PE=1 SV=2 | 65535.8 | 579 | 6.045 | 3 | 3 | 10 | 10 |
| 1633 | 5.47 | sp|P43034|LIS1_HUMAN | Platelet-activating factor acetylhydrolase IB subunit alpha OS=Homo sapiens GN=PAFAH1B1 PE=1 SV=2 | 46637.7 | 410 | 9.756 | 4 | 4 | 32 | 17 |
| 1634 | 5.47 | tr|B2R9J4|B2R9J4_HUMAN | cDNA, FLJ94423, highly similar to Homo sapiens mitochondrial ribosomal protein L23 (MRPL23), nuclear gene encoding mitochondrial protein, mRNA OS=Homo sapiens PE=2 SV=1 | 17797.1 | 153 | 16.34 | 3 | 3 | 6 | 6 |
| 1635 | 5.47 | sp|Q92665|RT31_HUMAN | 28S ribosomal protein S31, mitochondrial OS=Homo sapiens GN=MRPS31 PE=1 SV=3 | 45318.1 | 395 | 11.14 | 3 | 3 | 12 | 12 |
| 1636 | 5.46 | tr|A0A024R837|A0A024R837_HUMAN | Haloacid dehalogenase-like hydrolase domain containing 3, isoform CRA_a OS=Homo sapiens GN=HDHD3 PE=4 SV=1 | 28071.8 | 251 | 19.12 | 3 | 3 | 16 | 16 |
| 1637 | 5.44 | tr|F5GXR3|F5GXR3_HUMAN | Parathymosin OS=Homo sapiens GN=PTMS PE=1 SV=1 | 12074.5 | 104 | 16.35 | 3 | 3 | 56 | 56 |
| 1638 | 5.42 | tr|B2RAR2|B2RAR2_HUMAN | cDNA, FLJ95064, highly similar to Homo sapiens nin one binding protein (NOB1P), mRNA OS=Homo sapiens PE=2 SV=1 | 46644.6 | 412 | 11.89 | 3 | 3 | 11 | 11 |
| 1639 | 5.41 | tr|A0A024R637|A0A024R637_HUMAN | TBC1 domain family, member 4, isoform CRA_b OS=Homo sapiens GN=TBC1D4 PE=4 SV=1 | 146547.9 | 1298 | 3.313 | 3 | 3 | 7 | 7 |
| 1640 | 5.41 | tr|H7BYN4|H7BYN4_HUMAN | Kinesin-like protein OS=Homo sapiens GN=KIF23 PE=1 SV=1 | 109129.1 | 952 | 3.676 | 3 | 2 | 8 | 2 |
| 1641 | 5.41 | sp|P46939|UTRO_HUMAN | Utrophin OS=Homo sapiens GN=UTRN PE=1 SV=2 | 394463.1 | 3433 | 2.185 | 6 | 6 | 7 | 7 |
| 1642 | 5.4 | tr|A0A024R9B7|A0A024R9B7_HUMAN | Cytochrome c oxidase subunit VIc, isoform CRA_a OS=Homo sapiens GN=COX6C PE=4 SV=1 | 8781.4 | 75 | 49.33 | 4 | 4 | 13 | 13 |
| 1643 | 5.39 | sp|Q08426|ECHP_HUMAN | Peroxisomal bifunctional enzyme OS=Homo sapiens GN=EHHADH PE=1 SV=3 | 79494.2 | 723 | 4.426 | 3 | 3 | 4 | 4 |
| 1644 | 5.39 | sp|Q99816|TS101_HUMAN | Tumor susceptibility gene 101 protein OS=Homo sapiens GN=TSG101 PE=1 SV=2 | 43943.9 | 390 | 7.692 | 3 | 3 | 12 | 12 |
| 1645 | 5.39 | tr|B7ZLW0|B7ZLW0_HUMAN | LPP protein OS=Homo sapiens GN=LPP PE=2 SV=1 | 65773.9 | 612 | 8.66 | 3 | 3 | 3 | 3 |
| 1646 | 5.39 | tr|I3L0U5|I3L0U5_HUMAN | Coiled-coil domain-containing protein 137 (Fragment) OS=Homo sapiens GN=CCDC137 PE=1 SV=1 | 33524.5 | 292 | 14.04 | 3 | 3 | 6 | 6 |
| 1647 | 5.38 | tr|U3KQC1|U3KQC1_HUMAN | WD repeat-containing protein 18 (Fragment) OS=Homo sapiens GN=WDR18 PE=1 SV=1 | 43294 | 394 | 9.898 | 4 | 4 | 10 | 10 |
| 1648 | 5.36 | sp|O60488|ACSL4_HUMAN | Long-chain-fatty-acid--CoA ligase 4 OS=Homo sapiens GN=ACSL4 PE=1 SV=2 | 79187.4 | 711 | 8.017 | 4 | 3 | 14 | 8 |
| 1649 | 5.36 | tr|A0A087WT44|A0A087WT44_HUMAN | Heme oxygenase 2 OS=Homo sapiens GN=HMOX2 PE=1 SV=1 | 41668.8 | 370 | 14.59 | 4 | 4 | 21 | 21 |
| 1650 | 5.35 | sp|O60508|PRP17_HUMAN | Pre-mRNA-processing factor 17 OS=Homo sapiens GN=CDC40 PE=1 SV=1 | 65520.8 | 579 | 5.527 | 3 | 3 | 4 | 4 |
| 1651 | 5.34 | tr|Q96CV8|Q96CV8_HUMAN | Thimet oligopeptidase 1 OS=Homo sapiens GN=THOP1 PE=2 SV=1 | 78823.1 | 689 | 9.434 | 4 | 4 | 6 | 6 |
| 1652 | 5.34 | sp|Q93034|CUL5_HUMAN | Cullin-5 OS=Homo sapiens GN=CUL5 PE=1 SV=4 | 90954.6 | 780 | 4.359 | 3 | 3 | 3 | 3 |
| 1653 | 5.34 | tr|V9HW00|V9HW00_HUMAN | Epididymis secretory sperm binding protein Li 77p (Fragment) OS=Homo sapiens GN=HEL-S-77p PE=2 SV=1 | 39013.7 | 338 | 13.31 | 3 | 3 | 12 | 12 |
| 1654 | 5.32 | tr|A8K287|A8K287_HUMAN | Synaptosomal-associated protein OS=Homo sapiens GN=SNAP23 PE=2 SV=1 | 23353.8 | 211 | 15.64 | 3 | 3 | 5 | 5 |
| 1655 | 5.31 | tr|B9ZVN9|B9ZVN9_HUMAN | DNA-directed RNA polymerase subunit OS=Homo sapiens GN=POLR1A PE=1 SV=1 | 187807 | 1659 | 2.471 | 3 | 3 | 8 | 8 |
| 1656 | 5.31 | sp|Q8IY37|DHX37_HUMAN | Probable ATP-dependent RNA helicase DHX37 OS=Homo sapiens GN=DHX37 PE=1 SV=1 | 129544.3 | 1157 | 3.025 | 3 | 3 | 8 | 8 |
| 1657 | 5.31 | sp|P14927|QCR7_HUMAN | Cytochrome b-c1 complex subunit 7 OS=Homo sapiens GN=UQCRB PE=1 SV=2 | 13530.3 | 111 | 36.94 | 4 | 4 | 6 | 6 |
| 1658 | 5.28 | tr|A0A024RA81|A0A024RA81_HUMAN | 5'-nucleotidase OS=Homo sapiens GN=NT5C3 PE=3 SV=1 | 33914.9 | 297 | 8.081 | 3 | 3 | 4 | 4 |
| 1659 | 5.28 | tr|Q6IBU4|Q6IBU4_HUMAN | SDF2 protein OS=Homo sapiens GN=SDF2 PE=2 SV=1 | 23025.8 | 211 | 16.59 | 3 | 3 | 4 | 4 |
| 1660 | 5.27 | tr|Q8IY44|Q8IY44_HUMAN | CTBP2 protein OS=Homo sapiens GN=CTBP2 PE=1 SV=1 | 56200.5 | 513 | 8.577 | 5 | 3 | 7 | 4 |
| 1661 | 5.27 | sp|Q9H845|ACAD9_HUMAN | Acyl-CoA dehydrogenase family member 9, mitochondrial OS=Homo sapiens GN=ACAD9 PE=1 SV=1 | 68759.7 | 621 | 8.696 | 4 | 4 | 10 | 10 |
| 1662 | 5.27 | tr|A0A024R9Y7|A0A024R9Y7_HUMAN | Melanoma antigen family D, 2, isoform CRA_a OS=Homo sapiens GN=MAGED2 PE=4 SV=1 | 64953.5 | 606 | 9.076 | 3 | 3 | 3 | 3 |
| 1663 | 5.27 | sp|Q9NPD3|EXOS4_HUMAN | Exosome complex component RRP41 OS=Homo sapiens GN=EXOSC4 PE=1 SV=3 | 26382.7 | 245 | 13.88 | 3 | 3 | 7 | 7 |
| 1664 | 5.26 | sp|Q9H6Z4|RANB3_HUMAN | Ran-binding protein 3 OS=Homo sapiens GN=RANBP3 PE=1 SV=1 | 60209.3 | 567 | 10.76 | 4 | 4 | 9 | 9 |
| 1665 | 5.25 | tr|A0A140VJL0|A0A140VJL0_HUMAN | 3-hydroxyisobutyryl-CoA hydrolase, mitochondrial OS=Homo sapiens PE=2 SV=1 | 43481.9 | 386 | 8.031 | 3 | 3 | 14 | 14 |
| 1666 | 5.22 | tr|A0A0A6YYA0|A0A0A6YYA0_HUMAN | Protein TMED7-TICAM2 OS=Homo sapiens GN=TMED7-TICAM2 PE=3 SV=1 | 21232.8 | 188 | 18.09 | 3 | 3 | 29 | 29 |
| 1667 | 5.21 | sp|Q9GZP4|PITH1_HUMAN | PITH domain-containing protein 1 OS=Homo sapiens GN=PITHD1 PE=1 SV=1 | 24177.6 | 211 | 19.91 | 3 | 3 | 13 | 13 |
| 1668 | 5.21 | sp|O00186|STXB3_HUMAN | Syntaxin-binding protein 3 OS=Homo sapiens GN=STXBP3 PE=1 SV=2 | 67763.6 | 592 | 4.561 | 3 | 3 | 4 | 4 |
| 1669 | 5.21 | tr|Q0VAB1|Q0VAB1_HUMAN | Translocase of inner mitochondrial membrane 50 homolog (S. cerevisiae) OS=Homo sapiens GN=TIMM50 PE=2 SV=1 | 50478.2 | 456 | 7.237 | 3 | 3 | 10 | 10 |
| 1670 | 5.2 | sp|Q9UBI6|GBG12_HUMAN | Guanine nucleotide-binding protein G(I)/G(S)/G(O) subunit gamma-12 OS=Homo sapiens GN=GNG12 PE=1 SV=3 | 8006.1 | 72 | 44.44 | 3 | 3 | 9 | 9 |
| 1671 | 5.2 | tr|E5RIM3|E5RIM3_HUMAN | Phospholipase A-2-activating protein OS=Homo sapiens GN=PLAA PE=1 SV=1 | 66733.8 | 609 | 8.046 | 3 | 3 | 6 | 6 |
| 1672 | 5.2 | sp|Q96EE3|SEH1_HUMAN | Nucleoporin SEH1 OS=Homo sapiens GN=SEH1L PE=1 SV=3 | 39648.3 | 360 | 20 | 4 | 4 | 5 | 4 |
| 1673 | 5.19 | tr|A0A140VKB1|A0A140VKB1_HUMAN | Testis secretory sperm-binding protein Li 238E OS=Homo sapiens PE=2 SV=1 | 86982.4 | 745 | 5.235 | 3 | 3 | 12 | 12 |
| 1674 | 5.19 | tr|A0A024RAC0|A0A024RAC0_HUMAN | Leucine zipper protein 1, isoform CRA_a OS=Homo sapiens GN=LUZP1 PE=4 SV=1 | 120302.8 | 1076 | 3.903 | 3 | 3 | 6 | 6 |
| 1675 | 5.19 | tr|B4DX34|B4DX34_HUMAN | cDNA FLJ51554, highly similar to Squalene monooxygenase (EC 1.14.99.7) OS=Homo sapiens PE=2 SV=1 | 52995 | 479 | 9.812 | 3 | 3 | 3 | 3 |
| 1676 | 5.17 | tr|A8K750|A8K750_HUMAN | cDNA FLJ78041, highly similar to Homo sapiens NADH dehydrogenase (ubiquinone) flavoprotein 2, 24kDa (NDUFV2), mRNA OS=Homo sapiens PE=2 SV=1 | 27379.3 | 249 | 24.1 | 5 | 4 | 9 | 8 |
| 1677 | 5.17 | tr|E5KT65|E5KT65_HUMAN | DNA-directed RNA polymerase subunit RPABC1 OS=Homo sapiens PE=3 SV=1 | 24551.1 | 210 | 19.05 | 4 | 4 | 14 | 14 |
| 1678 | 5.17 | tr|B3KNK9|B3KNK9_HUMAN | Epididymis luminal protein 46 OS=Homo sapiens GN=HEL46 PE=2 SV=1 | 45333.1 | 401 | 6.983 | 3 | 3 | 9 | 9 |
| 1679 | 5.17 | tr|A0A0S2Z4Y4|A0A0S2Z4Y4_HUMAN | Clathrin interactor 1 isoform 1 (Fragment) OS=Homo sapiens GN=CLINT1 PE=2 SV=1 | 68259 | 625 | 7.36 | 4 | 4 | 24 | 24 |
| 1680 | 5.16 | tr|A0A024R0Q0|A0A024R0Q0_HUMAN | Uncharacterized protein OS=Homo sapiens GN=FLJ12886 PE=4 SV=1 | 57650.2 | 520 | 9.038 | 3 | 3 | 6 | 6 |
| 1681 | 5.16 | sp|Q6P1N0|C2D1A_HUMAN | Coiled-coil and C2 domain-containing protein 1A OS=Homo sapiens GN=CC2D1A PE=1 SV=1 | 104061.5 | 951 | 3.26 | 3 | 3 | 4 | 4 |
| 1682 | 5.15 | tr|Q53EL3|Q53EL3_HUMAN | Tyrosine-protein kinase (Fragment) OS=Homo sapiens PE=2 SV=1 | 50616.9 | 449 | 10.24 | 4 | 4 | 12 | 12 |
| 1683 | 5.14 | tr|A8K4T6|A8K4T6_HUMAN | cDNA FLJ76282, highly similar to Homo sapiens proteasome (prosome, macropain) 26S subunit, non-ATPase, 5 (PSMD5), mRNA OS=Homo sapiens PE=2 SV=1 | 56223.2 | 504 | 10.32 | 4 | 4 | 7 | 7 |
| 1684 | 5.14 | sp|Q8IW45|NNRD_HUMAN | ATP-dependent (S)-NAD(P)H-hydrate dehydratase OS=Homo sapiens GN=NAXD PE=1 SV=1 | 36575.7 | 347 | 11.24 | 3 | 3 | 4 | 4 |
| 1685 | 5.14 | sp|Q9NXH8|TOR4A_HUMAN | Torsin-4A OS=Homo sapiens GN=TOR4A PE=1 SV=2 | 46913.5 | 423 | 8.274 | 3 | 3 | 5 | 5 |
| 1686 | 5.14 | tr|E9PI68|E9PI68_HUMAN | Signal peptidase complex subunit 2 OS=Homo sapiens GN=SPCS2 PE=1 SV=1 | 28502.6 | 257 | 13.62 | 3 | 3 | 23 | 23 |
| 1687 | 5.13 | sp|Q9BV57|MTND_HUMAN | 1,2-dihydroxy-3-keto-5-methylthiopentene dioxygenase OS=Homo sapiens GN=ADI1 PE=1 SV=1 | 21498.2 | 179 | 31.28 | 4 | 4 | 13 | 13 |
| 1688 | 5.12 | tr|A0A087WVC1|A0A087WVC1_HUMAN | ATP-dependent RNA helicase DDX50 OS=Homo sapiens GN=DDX50 PE=1 SV=1 | 82226.7 | 730 | 13.42 | 7 | 5 | 22 | 7 |
| 1689 | 5.12 | sp|Q5RKV6|EXOS6_HUMAN | Exosome complex component MTR3 OS=Homo sapiens GN=EXOSC6 PE=1 SV=1 | 28234.8 | 272 | 20.22 | 3 | 3 | 3 | 3 |
| 1690 | 5.11 | tr|V9HWF0|V9HWF0_HUMAN | Epididymis secretory protein Li 28 OS=Homo sapiens GN=HEL-S-28 PE=2 SV=1 | 51418.8 | 452 | 7.08 | 3 | 3 | 15 | 15 |
| 1691 | 5.11 | tr|B2R6X8|B2R6X8_HUMAN | cDNA, FLJ93169, highly similar to Homo sapiens GPAA1P anchor attachment protein 1 homolog (yeast) (GPAA1), mRNA OS=Homo sapiens PE=2 SV=1 | 67592.5 | 621 | 5.958 | 3 | 3 | 8 | 8 |
| 1692 | 5.1 | tr|A0A024RD07|A0A024RD07_HUMAN | Trinucleotide repeat containing 5, isoform CRA_c OS=Homo sapiens GN=TNRC5 PE=4 SV=1 | 30773.8 | 278 | 16.91 | 6 | 3 | 36 | 16 |
| 1693 | 5.1 | sp|P06132|DCUP_HUMAN | Uroporphyrinogen decarboxylase OS=Homo sapiens GN=UROD PE=1 SV=2 | 40786.6 | 367 | 15.53 | 4 | 4 | 5 | 5 |
| 1694 | 5.08 | tr|Q59EH7|Q59EH7_HUMAN | DnaJ (Hsp40) homolog, subfamily C, member 7 variant (Fragment) OS=Homo sapiens PE=2 SV=1 | 55417.1 | 483 | 7.867 | 4 | 4 | 8 | 8 |
| 1695 | 5.08 | sp|Q7Z7K6|CENPV_HUMAN | Centromere protein V OS=Homo sapiens GN=CENPV PE=1 SV=1 | 29945.6 | 275 | 17.45 | 3 | 3 | 6 | 6 |
| 1696 | 5.05 | tr|B3KP71|B3KP71_HUMAN | cDNA FLJ31282 fis, clone KIDNE2006775, highly similar to Homo sapiens mediator of RNA polymerase II transcription, subunit 25 homolog (yeast) (MED25), mRNA OS=Homo sapiens PE=2 SV=1 | 76043.9 | 724 | 6.354 | 3 | 3 | 7 | 5 |
| 1697 | 5.05 | tr|A8E631|A8E631_HUMAN | KIAA1881 protein (Fragment) OS=Homo sapiens GN=KIAA1881 PE=2 SV=1 | 64731.3 | 642 | 6.231 | 3 | 3 | 3 | 3 |
| 1698 | 5.04 | tr|E5KNH5|E5KNH5_HUMAN | Mitochondrial NADH dehydrogenase ubiquinone flavoprotein 1 OS=Homo sapiens GN=NDUFV1 PE=4 SV=1 | 50816.7 | 464 | 6.897 | 3 | 3 | 6 | 6 |
| 1699 | 5.04 | sp|Q7Z2W9|RM21_HUMAN | 39S ribosomal protein L21, mitochondrial OS=Homo sapiens GN=MRPL21 PE=1 SV=2 | 22814.4 | 205 | 17.07 | 3 | 3 | 5 | 5 |
| 1700 | 5.03 | tr|C9JLU1|C9JLU1_HUMAN | DNA-directed RNA polymerases I, II, and III subunit RPABC3 (Fragment) OS=Homo sapiens GN=POLR2H PE=1 SV=7 | 16995.9 | 149 | 21.48 | 3 | 3 | 5 | 5 |
| 1701 | 5.02 | tr|A0A0S2Z4Y5|A0A0S2Z4Y5_HUMAN | Dolichyl-phosphate mannosyltransferase polypeptide 1 catalytic subunit isoform 1 (Fragment) OS=Homo sapiens GN=DPM1 PE=2 SV=1 | 29634 | 260 | 7.692 | 3 | 3 | 6 | 6 |
| 1702 | 5.02 | tr|Q53T99|Q53T99_HUMAN | Ribosome biogenesis protein WDR12 OS=Homo sapiens GN=WDR12 PE=2 SV=1 | 47707.5 | 423 | 10.4 | 3 | 3 | 4 | 4 |
| 1703 | 5.01 | tr|A0A087WUK2|A0A087WUK2_HUMAN | Heterogeneous nuclear ribonucleoprotein D-like OS=Homo sapiens GN=HNRNPDL PE=1 SV=1 | 40039.9 | 363 | 17.36 | 6 | 4 | 49 | 20 |
| 1704 | 5.01 | sp|Q6NUM9|RETST_HUMAN | All-trans-retinol 13,14-reductase OS=Homo sapiens GN=RETSAT PE=1 SV=2 | 66818.9 | 610 | 5.902 | 3 | 3 | 10 | 5 |
| 1705 | 5 | sp|O15320|CTGE5_HUMAN | cTAGE family member 5 OS=Homo sapiens GN=CTAGE5 PE=1 SV=4 | 90995.5 | 804 | 3.109 | 3 | 3 | 5 | 5 |
| 1706 | 4.99 | tr|I3L2X7|I3L2X7_HUMAN | Centrosomal protein of 131 kDa (Fragment) OS=Homo sapiens GN=CEP131 PE=1 SV=1 | 62014.4 | 524 | 6.107 | 4 | 3 | 8 | 4 |
| 1707 | 4.99 | tr|A0A024R5X7|A0A024R5X7_HUMAN | ClpX caseinolytic peptidase X homolog (E. coli), isoform CRA_a OS=Homo sapiens GN=CLPX PE=4 SV=1 | 69223.2 | 633 | 5.529 | 3 | 3 | 9 | 9 |
| 1708 | 4.99 | sp|P50897|PPT1_HUMAN | Palmitoyl-protein thioesterase 1 OS=Homo sapiens GN=PPT1 PE=1 SV=1 | 34193.2 | 306 | 12.42 | 3 | 3 | 19 | 19 |
| 1709 | 4.98 | sp|Q6IA17|SIGIR_HUMAN | Single Ig IL-1-related receptor OS=Homo sapiens GN=SIGIRR PE=1 SV=3 | 45678.6 | 410 | 8.78 | 3 | 3 | 17 | 17 |
| 1710 | 4.98 | sp|P35914|HMGCL_HUMAN | Hydroxymethylglutaryl-CoA lyase, mitochondrial OS=Homo sapiens GN=HMGCL PE=1 SV=2 | 34359.8 | 325 | 10.15 | 3 | 3 | 6 | 6 |
| 1711 | 4.97 | sp|Q99797|MIPEP_HUMAN | Mitochondrial intermediate peptidase OS=Homo sapiens GN=MIPEP PE=1 SV=2 | 80639.9 | 713 | 7.153 | 3 | 3 | 5 | 5 |
| 1712 | 4.96 | tr|Q86VR6|Q86VR6_HUMAN | DDX10 protein (Fragment) OS=Homo sapiens GN=DDX10 PE=2 SV=1 | 85831.1 | 745 | 4.564 | 3 | 3 | 8 | 8 |
| 1713 | 4.95 | tr|B2R5T5|B2R5T5_HUMAN | Protein kinase, cAMP-dependent, regulatory, type I, alpha (Tissue specific extinguisher 1), isoform CRA_a OS=Homo sapiens GN=PRKAR1A PE=2 SV=1 | 42981.3 | 381 | 9.186 | 4 | 3 | 13 | 10 |
| 1714 | 4.95 | sp|Q9H269|VPS16_HUMAN | Vacuolar protein sorting-associated protein 16 homolog OS=Homo sapiens GN=VPS16 PE=1 SV=2 | 94692.7 | 839 | 5.125 | 4 | 4 | 9 | 9 |
| 1715 | 4.95 | sp|Q4G0J3|LARP7_HUMAN | La-related protein 7 OS=Homo sapiens GN=LARP7 PE=1 SV=1 | 66898.2 | 582 | 6.186 | 3 | 3 | 4 | 4 |
| 1716 | 4.95 | tr|B2R7E8|B2R7E8_HUMAN | cDNA, FLJ93412, highly similar to Homo sapiens replication protein A2, 32kDa (RPA2), mRNA OS=Homo sapiens PE=2 SV=1 | 29262.7 | 270 | 14.81 | 3 | 3 | 11 | 11 |
| 1717 | 4.95 | sp|Q9BY49|PECR_HUMAN | Peroxisomal trans-2-enoyl-CoA reductase OS=Homo sapiens GN=PECR PE=1 SV=2 | 32544.1 | 303 | 10.23 | 4 | 4 | 4 | 3 |
| 1718 | 4.94 | tr|A0A024R6D4|A0A024R6D4_HUMAN | Enhancer of rudimentary homolog OS=Homo sapiens GN=ERH PE=3 SV=1 | 12258.8 | 104 | 31.73 | 3 | 3 | 20 | 20 |
| 1719 | 4.94 | tr|H7C2Q8|H7C2Q8_HUMAN | EBNA1 binding protein 2, isoform CRA_d OS=Homo sapiens GN=EBNA1BP2 PE=1 SV=1 | 40684.2 | 361 | 16.62 | 4 | 4 | 13 | 13 |
| 1720 | 4.94 | tr|Q53XJ5|Q53XJ5_HUMAN | FK506 binding protein 2, 13kDa OS=Homo sapiens GN=FKBP2 PE=2 SV=1 | 15649.2 | 142 | 9.155 | 2 | 2 | 24 | 24 |
| 1721 | 4.93 | tr|A8K885|A8K885_HUMAN | cDNA FLJ77179, highly similar to Homo sapiens sorting nexin 6 (SNX6) mRNA OS=Homo sapiens PE=2 SV=1 | 46647.3 | 406 | 7.389 | 3 | 3 | 3 | 3 |
| 1722 | 4.93 | tr|A0A0U1RQC9|A0A0U1RQC9_HUMAN | Cellular tumor antigen p53 OS=Homo sapiens GN=TP53 PE=1 SV=1 | 45884 | 410 | 6.585 | 3 | 3 | 3 | 3 |
| 1723 | 4.92 | sp|Q8TAE8|G45IP_HUMAN | Growth arrest and DNA damage-inducible proteins-interacting protein 1 OS=Homo sapiens GN=GADD45GIP1 PE=1 SV=1 | 25383.6 | 222 | 21.17 | 3 | 3 | 8 | 8 |
| 1724 | 4.91 | sp|Q9Y3B8|ORN_HUMAN | Oligoribonuclease, mitochondrial OS=Homo sapiens GN=REXO2 PE=1 SV=3 | 26832.5 | 237 | 11.39 | 3 | 3 | 4 | 4 |
| 1725 | 4.91 | tr|A0A024R9L6|A0A024R9L6_HUMAN | ST3 beta-galactoside alpha-2,3-sialyltransferase 1, isoform CRA_a OS=Homo sapiens GN=ST3GAL1 PE=3 SV=1 | 39074.7 | 340 | 13.24 | 3 | 3 | 8 | 8 |
| 1726 | 4.9 | sp|P35580|MYH10_HUMAN | Myosin-10 OS=Homo sapiens GN=MYH10 PE=1 SV=3 | 228997.2 | 1976 | 9.211 | 21 | 5 | 67 | 7 |
| 1727 | 4.9 | sp|P82675|RT05_HUMAN | 28S ribosomal protein S5, mitochondrial OS=Homo sapiens GN=MRPS5 PE=1 SV=2 | 48006.1 | 430 | 6.744 | 3 | 3 | 4 | 4 |
| 1728 | 4.9 | sp|Q5T653|RM02_HUMAN | 39S ribosomal protein L2, mitochondrial OS=Homo sapiens GN=MRPL2 PE=1 SV=2 | 33300.6 | 305 | 16.39 | 4 | 4 | 31 | 31 |
| 1729 | 4.89 | tr|Q8IYQ9|Q8IYQ9_HUMAN | Importin subunit alpha OS=Homo sapiens GN=KPNA3 PE=2 SV=1 | 57900.5 | 521 | 7.869 | 4 | 3 | 12 | 3 |
| 1730 | 4.87 | tr|A0A024R3V8|A0A024R3V8_HUMAN | Translin-associated factor X, isoform CRA_c OS=Homo sapiens GN=TSNAX PE=4 SV=1 | 33112.3 | 290 | 17.24 | 4 | 4 | 5 | 5 |
| 1731 | 4.86 | tr|D6RGW0|D6RGW0_HUMAN | Endoplasmic reticulum aminopeptidase 2 (Fragment) OS=Homo sapiens GN=ERAP2 PE=1 SV=1 | 79731.4 | 694 | 5.187 | 3 | 3 | 17 | 17 |
| 1732 | 4.85 | sp|O43490|PROM1_HUMAN | Prominin-1 OS=Homo sapiens GN=PROM1 PE=1 SV=1 | 97201.1 | 865 | 5.318 | 3 | 3 | 7 | 7 |
| 1733 | 4.85 | sp|Q9NX20|RM16_HUMAN | 39S ribosomal protein L16, mitochondrial OS=Homo sapiens GN=MRPL16 PE=1 SV=1 | 28449 | 251 | 12.35 | 3 | 3 | 8 | 8 |
| 1734 | 4.84 | sp|Q9Y3Q8|T22D4_HUMAN | TSC22 domain family protein 4 OS=Homo sapiens GN=TSC22D4 PE=1 SV=2 | 41025.6 | 395 | 11.39 | 3 | 3 | 3 | 3 |
| 1735 | 4.83 | tr|B4DN31|B4DN31_HUMAN | cDNA FLJ55809 OS=Homo sapiens PE=2 SV=1 | 51050.2 | 454 | 10.79 | 4 | 4 | 8 | 8 |
| 1736 | 4.82 | sp|Q96GX9|MTNB_HUMAN | Methylthioribulose-1-phosphate dehydratase OS=Homo sapiens GN=APIP PE=1 SV=1 | 27125.1 | 242 | 16.94 | 3 | 3 | 6 | 6 |
| 1737 | 4.81 | sp|Q9H0B6|KLC2_HUMAN | Kinesin light chain 2 OS=Homo sapiens GN=KLC2 PE=1 SV=1 | 68934 | 622 | 11.58 | 5 | 4 | 5 | 4 |
| 1738 | 4.81 | tr|Q9UN78|Q9UN78_HUMAN | Uncharacterized protein OS=Homo sapiens PE=4 SV=1 | 40059.4 | 338 | 14.79 | 4 | 4 | 5 | 5 |
| 1739 | 4.8 | sp|Q92692|NECT2_HUMAN | Nectin-2 OS=Homo sapiens GN=NECTIN2 PE=1 SV=1 | 57741.5 | 538 | 6.506 | 7 | 4 | 31 | 10 |
| 1740 | 4.8 | tr|Q53X12|Q53X12_HUMAN | V-type proton ATPase subunit a OS=Homo sapiens PE=2 SV=1 | 95750.3 | 831 | 4.693 | 3 | 3 | 6 | 6 |
| 1741 | 4.8 | sp|Q8N3D4|EH1L1_HUMAN | EH domain-binding protein 1-like protein 1 OS=Homo sapiens GN=EHBP1L1 PE=1 SV=2 | 161853.2 | 1523 | 4.005 | 4 | 4 | 5 | 5 |
| 1742 | 4.79 | sp|P54105|ICLN_HUMAN | Methylosome subunit pICln OS=Homo sapiens GN=CLNS1A PE=1 SV=1 | 26215.1 | 237 | 16.03 | 3 | 3 | 19 | 19 |
| 1743 | 4.79 | sp|O15400|STX7_HUMAN | Syntaxin-7 OS=Homo sapiens GN=STX7 PE=1 SV=4 | 29815.3 | 261 | 13.03 | 3 | 3 | 12 | 12 |
| 1744 | 4.77 | sp|P42345|MTOR_HUMAN | Serine/threonine-protein kinase mTOR OS=Homo sapiens GN=MTOR PE=1 SV=1 | 288889 | 2549 | 2.903 | 6 | 5 | 14 | 12 |
| 1745 | 4.77 | sp|P48634|PRC2A_HUMAN | Protein PRRC2A OS=Homo sapiens GN=PRRC2A PE=1 SV=3 | 228861.2 | 2157 | 4.914 | 5 | 5 | 6 | 6 |
| 1746 | 4.76 | sp|Q9P015|RM15_HUMAN | 39S ribosomal protein L15, mitochondrial OS=Homo sapiens GN=MRPL15 PE=1 SV=1 | 33419.5 | 296 | 21.62 | 5 | 4 | 14 | 9 |
| 1747 | 4.76 | tr|A0A096LPH6|A0A096LPH6_HUMAN | Zinc finger protein 638 OS=Homo sapiens GN=ZNF638 PE=1 SV=1 | 104048.6 | 918 | 3.704 | 3 | 3 | 5 | 5 |
| 1748 | 4.75 | sp|P51398|RT29_HUMAN | 28S ribosomal protein S29, mitochondrial OS=Homo sapiens GN=DAP3 PE=1 SV=1 | 45566.1 | 398 | 11.31 | 3 | 3 | 7 | 7 |
| 1749 | 4.74 | tr|E9PK47|E9PK47_HUMAN | Alpha-1,4 glucan phosphorylase OS=Homo sapiens GN=PYGL PE=1 SV=1 | 94069.5 | 819 | 16 | 13 | 3 | 77 | 7 |
| 1750 | 4.74 | tr|Q6IT96|Q6IT96_HUMAN | Histone deacetylase OS=Homo sapiens GN=HDAC1 PE=2 SV=1 | 55102.6 | 482 | 9.959 | 4 | 3 | 15 | 14 |
| 1751 | 4.74 | tr|V9HW44|V9HW44_HUMAN | Epididymis secretory protein Li 303 OS=Homo sapiens GN=HEL-S-303 PE=2 SV=1 | 25569.1 | 229 | 16.59 | 3 | 3 | 12 | 12 |
| 1752 | 4.74 | sp|Q96P70|IPO9_HUMAN | Importin-9 OS=Homo sapiens GN=IPO9 PE=1 SV=3 | 115961.8 | 1041 | 4.419 | 3 | 3 | 10 | 10 |
| 1753 | 4.73 | tr|V9HWC4|V9HWC4_HUMAN | Epididymis secretory sperm binding protein Li 132P OS=Homo sapiens GN=HEL-S-132P PE=2 SV=1 | 13832.4 | 125 | 35.2 | 3 | 3 | 10 | 10 |
| 1754 | 4.72 | tr|B2R6X6|B2R6X6_HUMAN | Peptidyl-prolyl cis-trans isomerase OS=Homo sapiens PE=2 SV=1 | 22039.1 | 207 | 42.03 | 6 | 5 | 8 | 6 |
| 1755 | 4.72 | sp|Q9HD33|RM47_HUMAN | 39S ribosomal protein L47, mitochondrial OS=Homo sapiens GN=MRPL47 PE=1 SV=2 | 29450.1 | 250 | 11.2 | 3 | 3 | 8 | 8 |
| 1756 | 4.71 | tr|Q7Z5V0|Q7Z5V0_HUMAN | EPS15 protein OS=Homo sapiens PE=2 SV=1 | 83671.6 | 762 | 7.087 | 4 | 3 | 7 | 6 |
| 1757 | 4.71 | tr|B4DDH8|B4DDH8_HUMAN | cDNA FLJ55184, highly similar to Homo sapiens leukocyte receptor cluster (LRC) member 4 (LENG4), mRNA OS=Homo sapiens PE=2 SV=1 | 50562.7 | 454 | 6.167 | 3 | 3 | 15 | 15 |
| 1758 | 4.7 | sp|Q9Y5A9|YTHD2_HUMAN | YTH domain-containing family protein 2 OS=Homo sapiens GN=YTHDF2 PE=1 SV=2 | 62333.8 | 579 | 8.463 | 5 | 3 | 17 | 12 |
| 1759 | 4.7 | tr|Q8NAK2|Q8NAK2_HUMAN | cDNA FLJ35208 fis, clone PLACE6018938, highly similar to Mus musculus epithelial ankyrin 3 (Ank3) 5kb isoform mRNA (Fragment) OS=Homo sapiens PE=2 SV=1 | 61168.5 | 549 | 5.464 | 3 | 3 | 4 | 4 |
| 1760 | 4.7 | tr|D6RG19|D6RG19_HUMAN | Ribosomal protein L37 OS=Homo sapiens GN=RPL37 PE=1 SV=1 | 9722.3 | 82 | 18.29 | 3 | 3 | 8 | 8 |
| 1761 | 4.68 | tr|A0A0A0MR39|A0A0A0MR39_HUMAN | Myelin expression factor 2 OS=Homo sapiens GN=MYEF2 PE=1 SV=1 | 64149.4 | 600 | 8 | 3 | 3 | 8 | 8 |
| 1762 | 4.68 | sp|P49662|CASP4_HUMAN | Caspase-4 OS=Homo sapiens GN=CASP4 PE=1 SV=1 | 43261.9 | 377 | 6.631 | 3 | 3 | 3 | 3 |
| 1763 | 4.67 | tr|J3KQN4|J3KQN4_HUMAN | 60S ribosomal protein L36a OS=Homo sapiens GN=RPL36A PE=3 SV=1 | 16378.1 | 142 | 26.76 | 4 | 4 | 8 | 8 |
| 1764 | 4.67 | sp|Q8TC07|TBC15_HUMAN | TBC1 domain family member 15 OS=Homo sapiens GN=TBC1D15 PE=1 SV=2 | 79490 | 691 | 5.21 | 3 | 3 | 7 | 7 |
| 1765 | 4.67 | sp|O75306|NDUS2_HUMAN | NADH dehydrogenase [ubiquinone] iron-sulfur protein 2, mitochondrial OS=Homo sapiens GN=NDUFS2 PE=1 SV=2 | 52545.3 | 463 | 6.695 | 4 | 4 | 6 | 6 |
| 1766 | 4.67 | tr|Q53FR7|Q53FR7_HUMAN | Nucleolar protein NOP52 variant (Fragment) OS=Homo sapiens PE=2 SV=1 | 52852.8 | 461 | 7.809 | 3 | 3 | 8 | 8 |
| 1767 | 4.66 | tr|B3KQ33|B3KQ33_HUMAN | cDNA FLJ32715 fis, clone TESTI2000784, highly similar to Importin-4 OS=Homo sapiens PE=2 SV=1 | 118724.8 | 1081 | 5.828 | 4 | 4 | 11 | 11 |
| 1768 | 4.65 | tr|H7BXY3|H7BXY3_HUMAN | Putative ATP-dependent RNA helicase DHX30 OS=Homo sapiens GN=DHX30 PE=1 SV=1 | 130548.9 | 1166 | 4.46 | 5 | 5 | 11 | 6 |
| 1769 | 4.65 | tr|Q68CM6|Q68CM6_HUMAN | STXBP1 protein OS=Homo sapiens GN=stxbp1 PE=2 SV=1 | 67568 | 594 | 6.397 | 3 | 3 | 4 | 4 |
| 1770 | 4.65 | tr|Q96DP0|Q96DP0_HUMAN | cDNA FLJ31479 fis, clone NT2NE2001634, moderately similar to NADH-UBIQUINONE OXIDOREDUCTASE 9 KD SUBUNIT (EC 1.6.5.3) OS=Homo sapiens PE=2 SV=1 | 49244.6 | 456 | 7.237 | 3 | 3 | 3 | 3 |
| 1771 | 4.65 | tr|Q32Q14|Q32Q14_HUMAN | NDUFA7 protein (Fragment) OS=Homo sapiens GN=NDUFA7 PE=2 SV=1 | 13510.4 | 121 | 27.27 | 3 | 3 | 6 | 6 |
| 1772 | 4.65 | tr|J3KQ48|J3KQ48_HUMAN | Peptidyl-tRNA hydrolase 2, mitochondrial OS=Homo sapiens GN=PTRH2 PE=1 SV=1 | 19324.6 | 180 | 22.22 | 3 | 3 | 6 | 6 |
| 1773 | 4.65 | sp|P62308|RUXG_HUMAN | Small nuclear ribonucleoprotein G OS=Homo sapiens GN=SNRPG PE=1 SV=1 | 8496 | 76 | 17.11 | 2 | 2 | 10 | 10 |
| 1774 | 4.64 | tr|E9KL23|E9KL23_HUMAN | Epididymis secretory sperm binding protein Li 44a OS=Homo sapiens GN=SERPINA1 PE=2 SV=1 | 46736.2 | 418 | 5.981 | 3 | 3 | 6 | 6 |
| 1775 | 4.64 | sp|P48507|GSH0_HUMAN | Glutamate--cysteine ligase regulatory subunit OS=Homo sapiens GN=GCLM PE=1 SV=1 | 30726.7 | 274 | 15.33 | 3 | 3 | 6 | 6 |
| 1776 | 4.64 | sp|Q96A35|RM24_HUMAN | 39S ribosomal protein L24, mitochondrial OS=Homo sapiens GN=MRPL24 PE=1 SV=1 | 24914.7 | 216 | 13.43 | 2 | 2 | 6 | 6 |
| 1777 | 4.63 | tr|B2RD24|B2RD24_HUMAN | cDNA, FLJ96424 OS=Homo sapiens PE=2 SV=1 | 79638.3 | 733 | 6.685 | 4 | 3 | 13 | 7 |
| 1778 | 4.62 | tr|B8ZZN6|B8ZZN6_HUMAN | Small ubiquitin-related modifier 1 OS=Homo sapiens GN=SUMO1 PE=1 SV=1 | 16644.7 | 146 | 19.18 | 3 | 3 | 5 | 5 |
| 1779 | 4.61 | tr|C9J6U3|C9J6U3_HUMAN | Protein diaphanous homolog 2 OS=Homo sapiens GN=DIAPH2 PE=1 SV=3 | 125537.1 | 1103 | 8.16 | 5 | 5 | 8 | 7 |
| 1780 | 4.61 | sp|Q9BQ69|MACD1_HUMAN | O-acetyl-ADP-ribose deacetylase MACROD1 OS=Homo sapiens GN=MACROD1 PE=1 SV=2 | 35504.7 | 325 | 10.46 | 2 | 2 | 11 | 11 |
| 1781 | 4.61 | tr|A0A0A0MTC1|A0A0A0MTC1_HUMAN | E3 ubiquitin-protein ligase RNF213 OS=Homo sapiens GN=RNF213 PE=1 SV=1 | 596481.5 | 5256 | 1.065 | 5 | 5 | 13 | 11 |
| 1782 | 4.61 | tr|B7Z6C2|B7Z6C2_HUMAN | cDNA FLJ50663, highly similar to Phosphoglucomutase-1 (EC 5.4.2.2) OS=Homo sapiens PE=2 SV=1 | 63749.9 | 580 | 5.862 | 3 | 3 | 10 | 10 |
| 1783 | 4.61 | sp|O75569|PRKRA_HUMAN | Interferon-inducible double-stranded RNA-dependent protein kinase activator A OS=Homo sapiens GN=PRKRA PE=1 SV=1 | 34403.9 | 313 | 13.42 | 3 | 3 | 4 | 4 |
| 1784 | 4.59 | tr|A0A090N7W4|A0A090N7W4_HUMAN | Cyclin-dependent kinase 5 OS=Homo sapiens GN=CDK5 PE=2 SV=1 | 33304.1 | 292 | 14.38 | 4 | 3 | 27 | 9 |
| 1785 | 4.59 | tr|B9EGA3|B9EGA3_HUMAN | SMARCD2 protein OS=Homo sapiens GN=SMARCD2 PE=1 SV=1 | 55856.9 | 494 | 8.097 | 3 | 3 | 4 | 4 |
| 1786 | 4.59 | tr|B2RDJ6|B2RDJ6_HUMAN | Probable cytosolic iron-sulfur protein assembly protein CIAO1 OS=Homo sapiens GN=CIAO1 PE=2 SV=1 | 37779.6 | 339 | 12.98 | 3 | 3 | 6 | 6 |
| 1787 | 4.59 | sp|Q9Y3C1|NOP16_HUMAN | Nucleolar protein 16 OS=Homo sapiens GN=NOP16 PE=1 SV=2 | 21188.2 | 178 | 17.98 | 3 | 3 | 7 | 7 |
| 1788 | 4.58 | sp|P68133|ACTS_HUMAN | Actin, alpha skeletal muscle OS=Homo sapiens GN=ACTA1 PE=1 SV=1 | 42050.7 | 377 | 53.05 | 36 | 11 | 1236 | 20 |
| 1789 | 4.58 | sp|O95154|ARK73_HUMAN | Aflatoxin B1 aldehyde reductase member 3 OS=Homo sapiens GN=AKR7A3 PE=1 SV=2 | 37206.1 | 331 | 18.13 | 4 | 3 | 8 | 7 |
| 1790 | 4.58 | tr|X6RAY8|X6RAY8_HUMAN | 39S ribosomal protein L4, mitochondrial OS=Homo sapiens GN=MRPL4 PE=1 SV=1 | 39587.3 | 357 | 7.843 | 2 | 2 | 21 | 21 |
| 1791 | 4.58 | sp|Q6UXV4|MIC27_HUMAN | MICOS complex subunit MIC27 OS=Homo sapiens GN=APOOL PE=1 SV=1 | 29158.7 | 268 | 18.66 | 3 | 3 | 5 | 5 |
| 1792 | 4.58 | sp|O43159|RRP8_HUMAN | Ribosomal RNA-processing protein 8 OS=Homo sapiens GN=RRP8 PE=1 SV=2 | 50714.3 | 456 | 8.114 | 3 | 3 | 5 | 5 |
| 1793 | 4.58 | sp|Q9Y3B9|RRP15_HUMAN | RRP15-like protein OS=Homo sapiens GN=RRP15 PE=1 SV=2 | 31484 | 282 | 10.99 | 3 | 3 | 6 | 6 |
| 1794 | 4.57 | tr|A8K9Y5|A8K9Y5_HUMAN | cDNA FLJ76689, highly similar to Homo sapiens chromodomain helicase DNA binding protein 2 (CHD2), mRNA (Fragment) OS=Homo sapiens PE=2 SV=1 | 120347.9 | 1049 | 4.099 | 4 | 3 | 7 | 5 |
| 1795 | 4.54 | tr|A8K4W2|A8K4W2_HUMAN | cDNA FLJ78635, highly similar to Homo sapiens ATP synthase, H+ transporting, mitochondrial F0 complex, subunit b, isoform 1 (ATP5F1), transcript variant 1, mRNA OS=Homo sapiens PE=2 SV=1 | 28880.4 | 256 | 16.41 | 4 | 4 | 25 | 23 |
| 1796 | 4.54 | sp|Q96IR7|HPDL_HUMAN | 4-hydroxyphenylpyruvate dioxygenase-like protein OS=Homo sapiens GN=HPDL PE=1 SV=1 | 39385.5 | 371 | 12.13 | 3 | 3 | 6 | 6 |
| 1797 | 4.53 | sp|O14617|AP3D1_HUMAN | AP-3 complex subunit delta-1 OS=Homo sapiens GN=AP3D1 PE=1 SV=1 | 130156.8 | 1153 | 3.556 | 3 | 3 | 10 | 8 |
| 1798 | 4.53 | tr|A0A0S2Z5U1|A0A0S2Z5U1_HUMAN | NADH dehydrogenase 1 alpha subcomplex assembly factor 2 isoform 1 (Fragment) OS=Homo sapiens GN=NDUFAF2 PE=2 SV=1 | 19856.2 | 169 | 11.83 | 2 | 2 | 4 | 4 |
| 1799 | 4.52 | sp|P42766|RL35_HUMAN | 60S ribosomal protein L35 OS=Homo sapiens GN=RPL35 PE=1 SV=2 | 14551.4 | 123 | 28.46 | 3 | 3 | 23 | 23 |
| 1800 | 4.52 | tr|Q5W0H4|Q5W0H4_HUMAN | Translationally-controlled tumor protein OS=Homo sapiens GN=TPT1 PE=1 SV=1 | 21525.5 | 188 | 15.43 | 4 | 4 | 51 | 51 |
| 1801 | 4.51 | tr|A0A0S2Z5I7|A0A0S2Z5I7_HUMAN | Shwachman-Bodian-Diamond syndrome isoform 1 (Fragment) OS=Homo sapiens GN=SBDS PE=2 SV=1 | 28763.3 | 250 | 10.8 | 2 | 2 | 7 | 7 |
| 1802 | 4.51 | sp|Q9BU61|NDUF3_HUMAN | NADH dehydrogenase [ubiquinone] 1 alpha subcomplex assembly factor 3 OS=Homo sapiens GN=NDUFAF3 PE=1 SV=1 | 20350 | 184 | 26.09 | 3 | 3 | 8 | 8 |
| 1803 | 4.51 | tr|B4DDW4|B4DDW4_HUMAN | cDNA FLJ56650, highly similar to Matrilysin (EC 3.4.24.23) OS=Homo sapiens PE=2 SV=1 | 21918.8 | 192 | 19.27 | 2 | 2 | 2 | 2 |
| 1804 | 4.49 | tr|A0A024R6D8|A0A024R6D8_HUMAN | Splicing factor, arginine/serine-rich 5, isoform CRA_a OS=Homo sapiens GN=SFRS5 PE=4 SV=1 | 31263.4 | 272 | 14.71 | 3 | 2 | 15 | 2 |
| 1805 | 4.49 | tr|B5BUI8|B5BUI8_HUMAN | Dual specificity phosphatase 3 (Fragment) OS=Homo sapiens GN=DUSP3 PE=2 SV=1 | 20577.2 | 185 | 21.08 | 3 | 3 | 7 | 7 |
| 1806 | 4.48 | sp|Q9Y3B7|RM11_HUMAN | 39S ribosomal protein L11, mitochondrial OS=Homo sapiens GN=MRPL11 PE=1 SV=1 | 20683 | 192 | 30.73 | 6 | 5 | 12 | 10 |
| 1807 | 4.48 | tr|Q06TE6|Q06TE6_HUMAN | Cytochrome c oxidase subunit 2 OS=Homo sapiens GN=COX2 PE=3 SV=1 | 25530.7 | 227 | 7.489 | 2 | 2 | 12 | 12 |
| 1808 | 4.48 | tr|Q6FGB3|Q6FGB3_HUMAN | PCBD protein (Fragment) OS=Homo sapiens GN=PCBD PE=2 SV=1 | 12009.6 | 104 | 23.08 | 2 | 2 | 23 | 22 |
| 1809 | 4.47 | tr|B7ZLZ7|B7ZLZ7_HUMAN | Structural maintenance of chromosomes protein OS=Homo sapiens GN=SMC2 PE=2 SV=1 | 135699.1 | 1197 | 2.005 | 2 | 2 | 9 | 9 |
| 1810 | 4.47 | sp|P61086|UBE2K_HUMAN | Ubiquitin-conjugating enzyme E2 K OS=Homo sapiens GN=UBE2K PE=1 SV=3 | 22406.4 | 200 | 22.5 | 3 | 3 | 15 | 15 |
| 1811 | 4.47 | sp|Q9BZK7|TBL1R_HUMAN | F-box-like/WD repeat-containing protein TBL1XR1 OS=Homo sapiens GN=TBL1XR1 PE=1 SV=1 | 55594.4 | 514 | 7.393 | 2 | 2 | 6 | 6 |
| 1812 | 4.46 | tr|B2R673|B2R673_HUMAN | Dihydrolipoamide acetyltransferase component of pyruvate dehydrogenase complex OS=Homo sapiens PE=2 SV=1 | 54045.7 | 501 | 6.786 | 3 | 3 | 8 | 8 |
| 1813 | 4.46 | tr|A0A024QYR8|A0A024QYR8_HUMAN | Transmembrane 9 superfamily member OS=Homo sapiens GN=TM9SF2 PE=2 SV=1 | 75774.9 | 663 | 4.374 | 2 | 2 | 6 | 6 |
| 1814 | 4.46 | sp|P60983|GMFB_HUMAN | Glia maturation factor beta OS=Homo sapiens GN=GMFB PE=1 SV=2 | 16713 | 142 | 16.9 | 3 | 3 | 8 | 8 |
| 1815 | 4.46 | tr|E5KS95|E5KS95_HUMAN | Elongation factor Ts, mitochondrial OS=Homo sapiens GN=TSFM PE=3 SV=1 | 35390.4 | 325 | 24.62 | 3 | 3 | 6 | 6 |
| 1816 | 4.46 | tr|B3KPZ2|B3KPZ2_HUMAN | cDNA FLJ32487 fis, clone SKNSH1000002, highly similar to Prostaglandin E synthase 2 (EC 5.3.99.3) OS=Homo sapiens PE=2 SV=1 | 41968.8 | 377 | 8.753 | 3 | 3 | 4 | 4 |
| 1817 | 4.45 | sp|Q9HAV7|GRPE1_HUMAN | GrpE protein homolog 1, mitochondrial OS=Homo sapiens GN=GRPEL1 PE=1 SV=2 | 24278.9 | 217 | 21.66 | 4 | 3 | 8 | 7 |
| 1818 | 4.45 | tr|A0A0S2Z392|A0A0S2Z392_HUMAN | Protein-serine/threonine kinase (Fragment) OS=Homo sapiens GN=ADRBK1 PE=2 SV=1 | 79573 | 689 | 4.499 | 3 | 3 | 5 | 5 |
| 1819 | 4.45 | tr|C9J1V9|C9J1V9_HUMAN | HCG2043275 OS=Homo sapiens GN=EEF1E1-BLOC1S5 PE=4 SV=2 | 17017.5 | 151 | 13.91 | 2 | 2 | 22 | 22 |
| 1820 | 4.44 | sp|Q9UNS2|CSN3_HUMAN | COP9 signalosome complex subunit 3 OS=Homo sapiens GN=COPS3 PE=1 SV=3 | 47872.8 | 423 | 10.17 | 3 | 3 | 14 | 14 |
| 1821 | 4.43 | sp|Q14257|RCN2_HUMAN | Reticulocalbin-2 OS=Homo sapiens GN=RCN2 PE=1 SV=1 | 36876.1 | 317 | 9.464 | 2 | 2 | 7 | 7 |
| 1822 | 4.43 | sp|Q2NL82|TSR1_HUMAN | Pre-rRNA-processing protein TSR1 homolog OS=Homo sapiens GN=TSR1 PE=1 SV=1 | 91809.5 | 804 | 3.358 | 2 | 2 | 4 | 4 |
| 1823 | 4.43 | tr|A8K8K1|A8K8K1_HUMAN | cDNA FLJ76936, highly similar to Homo sapiens RNA terminal phosphate cyclase domain 1 (RTCD1), mRNA OS=Homo sapiens PE=2 SV=1 | 39320.5 | 366 | 12.57 | 3 | 3 | 7 | 7 |
| 1824 | 4.42 | tr|B2RE76|B2RE76_HUMAN | Chromatin modifying protein 2B OS=Homo sapiens GN=CHMP2B PE=2 SV=1 | 23906.4 | 213 | 12.21 | 3 | 3 | 6 | 6 |
| 1825 | 4.41 | tr|A0A024RDY3|A0A024RDY3_HUMAN | Lysosomal-associated membrane protein 1, isoform CRA_a OS=Homo sapiens GN=LAMP1 PE=4 SV=1 | 44881.9 | 417 | 8.633 | 6 | 3 | 57 | 37 |
| 1826 | 4.4 | tr|F6IQZ0|F6IQZ0_HUMAN | MHC class I antigen (Fragment) OS=Homo sapiens GN=HLA-A PE=3 SV=1 | 38149.6 | 337 | 24.04 | 5 | 2 | 59 | 4 |
| 1827 | 4.4 | tr|B4E2E1|B4E2E1_HUMAN | cDNA FLJ54322, highly similar to Protein transport protein Sec24B OS=Homo sapiens PE=2 SV=1 | 96175 | 867 | 4.383 | 3 | 3 | 4 | 4 |
| 1828 | 4.39 | tr|Q5U676|Q5U676_HUMAN | B3GAT3 protein (Fragment) OS=Homo sapiens GN=B3GAT3 PE=2 SV=1 | 37828 | 341 | 4.985 | 2 | 2 | 5 | 5 |
| 1829 | 4.39 | tr|A0A024RCB9|A0A024RCB9_HUMAN | Tumor suppressing subtransferable candidate 4, isoform CRA_a OS=Homo sapiens GN=TSSC4 PE=4 SV=1 | 34285.2 | 329 | 10.03 | 3 | 3 | 6 | 6 |
| 1830 | 4.39 | sp|Q9BTZ2|DHRS4_HUMAN | Dehydrogenase/reductase SDR family member 4 OS=Homo sapiens GN=DHRS4 PE=1 SV=3 | 29536.9 | 278 | 11.51 | 4 | 4 | 7 | 7 |
| 1831 | 4.39 | sp|Q13952|NFYC_HUMAN | Nuclear transcription factor Y subunit gamma OS=Homo sapiens GN=NFYC PE=1 SV=3 | 50302.3 | 458 | 5.022 | 2 | 2 | 2 | 2 |
| 1832 | 4.38 | sp|O43520|AT8B1_HUMAN | Phospholipid-transporting ATPase IC OS=Homo sapiens GN=ATP8B1 PE=1 SV=3 | 143694.1 | 1251 | 2.638 | 3 | 3 | 6 | 6 |
| 1833 | 4.38 | sp|Q92541|RTF1_HUMAN | RNA polymerase-associated protein RTF1 homolog OS=Homo sapiens GN=RTF1 PE=1 SV=4 | 80312.8 | 710 | 4.225 | 3 | 3 | 7 | 7 |
| 1834 | 4.35 | tr|A0A087X176|A0A087X176_HUMAN | Sulfhydryl oxidase OS=Homo sapiens GN=QSOX2 PE=1 SV=1 | 77249.6 | 696 | 4.454 | 2 | 2 | 4 | 4 |
| 1835 | 4.35 | sp|Q96CS3|FAF2_HUMAN | FAS-associated factor 2 OS=Homo sapiens GN=FAF2 PE=1 SV=2 | 52623 | 445 | 7.64 | 3 | 3 | 3 | 3 |
| 1836 | 4.35 | sp|P62273|RS29_HUMAN | 40S ribosomal protein S29 OS=Homo sapiens GN=RPS29 PE=1 SV=2 | 6676.7 | 56 | 33.93 | 2 | 2 | 28 | 28 |
| 1837 | 4.34 | tr|Q9BQD2|Q9BQD2_HUMAN | Signal transducer and activator of transcription OS=Homo sapiens PE=2 SV=1 | 76125.3 | 679 | 4.86 | 3 | 3 | 5 | 5 |
| 1838 | 4.34 | tr|B4E290|B4E290_HUMAN | cDNA FLJ50039, highly similar to Homo sapiens solute carrier family 25, member 24, transcript variant 1, mRNA OS=Homo sapiens PE=2 SV=1 | 53296 | 477 | 4.403 | 2 | 2 | 3 | 3 |
| 1839 | 4.33 | tr|A0A0A6YYH1|A0A0A6YYH1_HUMAN | Protein C15orf38-AP3S2 OS=Homo sapiens GN=C15orf38-AP3S2 PE=4 SV=1 | 43881.5 | 394 | 8.122 | 3 | 3 | 6 | 6 |
| 1840 | 4.32 | sp|P30838|AL3A1_HUMAN | Aldehyde dehydrogenase, dimeric NADP-preferring OS=Homo sapiens GN=ALDH3A1 PE=1 SV=3 | 50394.6 | 453 | 7.285 | 4 | 2 | 16 | 7 |
| 1841 | 4.32 | sp|P11274|BCR_HUMAN | Breakpoint cluster region protein OS=Homo sapiens GN=BCR PE=1 SV=2 | 142818.1 | 1271 | 3.777 | 4 | 3 | 6 | 4 |
| 1842 | 4.32 | sp|O00461|GOLI4_HUMAN | Golgi integral membrane protein 4 OS=Homo sapiens GN=GOLIM4 PE=1 SV=1 | 81880.1 | 696 | 5.46 | 3 | 3 | 3 | 3 |
| 1843 | 4.32 | tr|A0A024QZW7|A0A024QZW7_HUMAN | Nucleoporin 153kDa, isoform CRA_a OS=Homo sapiens GN=NUP153 PE=4 SV=1 | 153967 | 1475 | 2.576 | 3 | 3 | 4 | 3 |
| 1844 | 4.32 | sp|P30049|ATPD_HUMAN | ATP synthase subunit delta, mitochondrial OS=Homo sapiens GN=ATP5D PE=1 SV=2 | 17489.8 | 168 | 13.69 | 2 | 2 | 29 | 29 |
| 1845 | 4.3 | tr|A0A075BSP6|A0A075BSP6_HUMAN | Endoplasmic reticulum aminopeptidase 1 delta-Exon-15 isoform OS=Homo sapiens GN=ERAP1 PE=2 SV=1 | 81738.9 | 724 | 3.177 | 2 | 2 | 7 | 7 |
| 1846 | 4.3 | sp|Q14331|FRG1_HUMAN | Protein FRG1 OS=Homo sapiens GN=FRG1 PE=1 SV=1 | 29172.1 | 258 | 12.79 | 3 | 3 | 8 | 8 |
| 1847 | 4.29 | sp|Q9H501|ESF1_HUMAN | ESF1 homolog OS=Homo sapiens GN=ESF1 PE=1 SV=1 | 98795.3 | 851 | 4.113 | 3 | 3 | 6 | 6 |
| 1848 | 4.29 | sp|Q05209|PTN12_HUMAN | Tyrosine-protein phosphatase non-receptor type 12 OS=Homo sapiens GN=PTPN12 PE=1 SV=3 | 88105.7 | 780 | 4.231 | 2 | 2 | 6 | 6 |
| 1849 | 4.28 | tr|E9PCY5|E9PCY5_HUMAN | DNA topoisomerase 2 (Fragment) OS=Homo sapiens GN=TOP2B PE=1 SV=1 | 130479.8 | 1150 | 4.522 | 4 | 3 | 8 | 6 |
| 1850 | 4.28 | tr|B2RDG1|B2RDG1_HUMAN | cDNA, FLJ96593 OS=Homo sapiens PE=2 SV=1 | 59382.3 | 515 | 5.049 | 2 | 2 | 2 | 2 |
| 1851 | 4.28 | sp|P05161|ISG15_HUMAN | Ubiquitin-like protein ISG15 OS=Homo sapiens GN=ISG15 PE=1 SV=5 | 17887.4 | 165 | 13.94 | 3 | 3 | 11 | 11 |
| 1852 | 4.27 | tr|B2RC75|B2RC75_HUMAN | Transmembrane channel-like protein OS=Homo sapiens PE=2 SV=1 | 78750.3 | 706 | 7.082 | 3 | 3 | 5 | 5 |
| 1853 | 4.27 | tr|C9JVN9|C9JVN9_HUMAN | L-2-hydroxyglutarate dehydrogenase, mitochondrial OS=Homo sapiens GN=L2HGDH PE=1 SV=1 | 48474.9 | 441 | 7.71 | 3 | 3 | 7 | 7 |
| 1854 | 4.26 | tr|A0A0S2Z583|A0A0S2Z583_HUMAN | Retinol dehydrogenase 11 isoform 1 (Fragment) OS=Homo sapiens GN=RDH11 PE=2 SV=1 | 35386 | 318 | 16.35 | 4 | 3 | 12 | 10 |
| 1855 | 4.26 | sp|P52298|NCBP2_HUMAN | Nuclear cap-binding protein subunit 2 OS=Homo sapiens GN=NCBP2 PE=1 SV=1 | 18001 | 156 | 12.18 | 2 | 2 | 7 | 7 |
| 1856 | 4.26 | tr|B2RD51|B2RD51_HUMAN | Proteasome assembly chaperone 1 OS=Homo sapiens PE=2 SV=1 | 32881.6 | 288 | 12.85 | 3 | 3 | 6 | 6 |
| 1857 | 4.26 | sp|P41223|BUD31_HUMAN | Protein BUD31 homolog OS=Homo sapiens GN=BUD31 PE=1 SV=2 | 16999.6 | 144 | 20.83 | 2 | 2 | 4 | 4 |
| 1858 | 4.26 | sp|Q9BYB4|GNB1L_HUMAN | Guanine nucleotide-binding protein subunit beta-like protein 1 OS=Homo sapiens GN=GNB1L PE=1 SV=2 | 35617.6 | 327 | 7.034 | 2 | 2 | 8 | 8 |
| 1859 | 4.25 | sp|Q5VTR2|BRE1A_HUMAN | E3 ubiquitin-protein ligase BRE1A OS=Homo sapiens GN=RNF20 PE=1 SV=2 | 113661.5 | 975 | 6.154 | 5 | 4 | 6 | 4 |
| 1860 | 4.24 | tr|E7EUL7|E7EUL7_HUMAN | Sperm-specific antigen 2 OS=Homo sapiens GN=SSFA2 PE=1 SV=1 | 84592 | 768 | 2.734 | 3 | 2 | 7 | 3 |
| 1861 | 4.24 | tr|A0A024R7F9|A0A024R7F9_HUMAN | Glutaryl-Coenzyme A dehydrogenase, isoform CRA_a OS=Homo sapiens GN=GCDH PE=3 SV=1 | 48126.7 | 438 | 10.27 | 3 | 3 | 9 | 9 |
| 1862 | 4.24 | sp|Q96EY7|PTCD3_HUMAN | Pentatricopeptide repeat domain-containing protein 3, mitochondrial OS=Homo sapiens GN=PTCD3 PE=1 SV=3 | 78548.9 | 689 | 4.064 | 2 | 2 | 6 | 6 |
| 1863 | 4.23 | tr|Q6FHR4|Q6FHR4_HUMAN | Mitogen-activated protein kinase (Fragment) OS=Homo sapiens GN=MAPK13 PE=2 SV=1 | 42017.2 | 365 | 5.205 | 2 | 2 | 12 | 12 |
| 1864 | 4.23 | tr|V5J3L2|V5J3L2_HUMAN | STIM1 OS=Homo sapiens GN=STIM1 PE=2 SV=1 | 77406.7 | 685 | 4.526 | 2 | 2 | 3 | 3 |
| 1865 | 4.23 | tr|Q2NLD0|Q2NLD0_HUMAN | Phospholipid-transporting ATPase (Fragment) OS=Homo sapiens GN=ATP9A PE=2 SV=1 | 100274.3 | 891 | 2.694 | 2 | 2 | 5 | 5 |
| 1866 | 4.23 | tr|F5GXF5|F5GXF5_HUMAN | Nucleosome-remodeling factor subunit BPTF (Fragment) OS=Homo sapiens GN=BPTF PE=1 SV=2 | 271529 | 2457 | 2.076 | 4 | 4 | 4 | 3 |
| 1867 | 4.22 | tr|B3KVE5|B3KVE5_HUMAN | cDNA FLJ16456 fis, clone BRAWH3009013, highly similar to AT-hook-containing transcription factor 1 (Fragment) OS=Homo sapiens PE=2 SV=1 | 147032.9 | 1322 | 5.522 | 6 | 5 | 6 | 4 |
| 1868 | 4.22 | tr|A0A024R9J0|A0A024R9J0_HUMAN | RAD21 homolog (S. pombe), isoform CRA_a OS=Homo sapiens GN=RAD21 PE=4 SV=1 | 71688.9 | 631 | 3.645 | 2 | 2 | 6 | 6 |
| 1869 | 4.22 | tr|A0A090N7T9|A0A090N7T9_HUMAN | Secernin 1 OS=Homo sapiens GN=SCRN1 PE=4 SV=1 | 46381.6 | 414 | 8.454 | 3 | 3 | 5 | 5 |
| 1870 | 4.21 | tr|A0A024RAJ2|A0A024RAJ2_HUMAN | Bridging integrator 1, isoform CRA_h OS=Homo sapiens GN=BIN1 PE=4 SV=1 | 54947.4 | 497 | 8.853 | 3 | 3 | 8 | 8 |
| 1871 | 4.21 | sp|O43570|CAH12_HUMAN | Carbonic anhydrase 12 OS=Homo sapiens GN=CA12 PE=1 SV=1 | 39450.6 | 354 | 10.17 | 3 | 3 | 7 | 7 |
| 1872 | 4.21 | tr|C9JA28|C9JA28_HUMAN | Translocon-associated protein subunit gamma OS=Homo sapiens GN=SSR3 PE=1 SV=1 | 20161.2 | 174 | 12.64 | 3 | 3 | 29 | 29 |
| 1873 | 4.21 | tr|Q6FIE9|Q6FIE9_HUMAN | TOLLIP protein OS=Homo sapiens GN=TOLLIP PE=1 SV=1 | 30281.7 | 274 | 8.394 | 2 | 2 | 8 | 7 |
| 1874 | 4.2 | tr|A0A024R6Z0|A0A024R6Z0_HUMAN | Dynein, cytoplasmic 1, light intermediate chain 2, isoform CRA_a OS=Homo sapiens GN=DYNC1LI2 PE=4 SV=1 | 54098.9 | 492 | 4.878 | 2 | 2 | 12 | 12 |
| 1875 | 4.2 | sp|Q9NV31|IMP3_HUMAN | U3 small nucleolar ribonucleoprotein protein IMP3 OS=Homo sapiens GN=IMP3 PE=1 SV=1 | 21849.9 | 184 | 25 | 3 | 3 | 9 | 9 |
| 1876 | 4.19 | sp|Q9NW82|WDR70_HUMAN | WD repeat-containing protein 70 OS=Homo sapiens GN=WDR70 PE=1 SV=1 | 73200.6 | 654 | 5.963 | 3 | 3 | 4 | 4 |
| 1877 | 4.19 | sp|Q8IYS1|P20D2_HUMAN | Peptidase M20 domain-containing protein 2 OS=Homo sapiens GN=PM20D2 PE=1 SV=2 | 47775.7 | 436 | 4.587 | 2 | 2 | 4 | 4 |
| 1878 | 4.19 | tr|H0YMV8|H0YMV8_HUMAN | 40S ribosomal protein S27 OS=Homo sapiens GN=RPS27L PE=1 SV=1 | 11345.5 | 100 | 21 | 2 | 2 | 43 | 43 |
| 1879 | 4.18 | tr|K7EJC8|K7EJC8_HUMAN | Golgi SNAP receptor complex member 1 OS=Homo sapiens GN=GOSR1 PE=1 SV=1 | 26988.6 | 236 | 11.86 | 3 | 3 | 9 | 9 |
| 1880 | 4.18 | sp|Q6DD88|ATLA3_HUMAN | Atlastin-3 OS=Homo sapiens GN=ATL3 PE=1 SV=1 | 60541.5 | 541 | 7.394 | 3 | 3 | 17 | 17 |
| 1881 | 4.17 | tr|A0A0U1RR18|A0A0U1RR18_HUMAN | Solute carrier family 12 member 7 (Fragment) OS=Homo sapiens GN=SLC12A7 PE=1 SV=1 | 108984.9 | 989 | 4.348 | 4 | 3 | 6 | 5 |
| 1882 | 4.17 | tr|B4E1J8|B4E1J8_HUMAN | cDNA FLJ56285, highly similar to ADP-ribosylation factor-like protein 8B OS=Homo sapiens PE=2 SV=1 | 27252.4 | 238 | 11.34 | 3 | 2 | 7 | 4 |
| 1883 | 4.17 | sp|O15511|ARPC5_HUMAN | Actin-related protein 2/3 complex subunit 5 OS=Homo sapiens GN=ARPC5 PE=1 SV=3 | 16320.3 | 151 | 21.19 | 2 | 2 | 15 | 15 |
| 1884 | 4.17 | sp|Q9UNN8|EPCR_HUMAN | Endothelial protein C receptor OS=Homo sapiens GN=PROCR PE=1 SV=1 | 26671.2 | 238 | 15.13 | 3 | 3 | 8 | 8 |
| 1885 | 4.16 | tr|Q53YE7|Q53YE7_HUMAN | Amine oxidase [flavin-containing] OS=Homo sapiens GN=MAOA PE=2 SV=1 | 59681.3 | 527 | 8.159 | 4 | 3 | 10 | 8 |
| 1886 | 4.16 | sp|Q13948|CASP_HUMAN | Protein CASP OS=Homo sapiens GN=CUX1 PE=1 SV=2 | 77454.5 | 678 | 3.392 | 2 | 2 | 4 | 4 |
| 1887 | 4.16 | sp|Q9Y315|DEOC_HUMAN | Deoxyribose-phosphate aldolase OS=Homo sapiens GN=DERA PE=1 SV=2 | 35230.4 | 318 | 7.233 | 2 | 2 | 4 | 4 |
| 1888 | 4.16 | tr|A2A2Q9|A2A2Q9_HUMAN | Protein AAR2 homolog OS=Homo sapiens GN=AAR2 PE=1 SV=1 | 45033.6 | 398 | 7.035 | 2 | 2 | 5 | 5 |
| 1889 | 4.16 | sp|P62495|ERF1_HUMAN | Eukaryotic peptide chain release factor subunit 1 OS=Homo sapiens GN=ETF1 PE=1 SV=3 | 49030.5 | 437 | 11.44 | 4 | 4 | 17 | 17 |
| 1890 | 4.16 | tr|Q6NZ61|Q6NZ61_HUMAN | Ras homolog enriched in brain OS=Homo sapiens GN=RHEB PE=2 SV=1 | 20557.4 | 184 | 13.04 | 2 | 2 | 6 | 6 |
| 1891 | 4.15 | tr|E7ER68|E7ER68_HUMAN | Protein FAM91A1 OS=Homo sapiens GN=FAM91A1 PE=1 SV=1 | 88986.7 | 789 | 2.408 | 2 | 2 | 4 | 4 |
| 1892 | 4.15 | sp|P33897|ABCD1_HUMAN | ATP-binding cassette sub-family D member 1 OS=Homo sapiens GN=ABCD1 PE=1 SV=2 | 82936.1 | 745 | 4.564 | 2 | 2 | 2 | 2 |
| 1893 | 4.15 | sp|Q96JB5|CK5P3_HUMAN | CDK5 regulatory subunit-associated protein 3 OS=Homo sapiens GN=CDK5RAP3 PE=1 SV=2 | 56920.2 | 506 | 5.534 | 2 | 2 | 8 | 8 |
| 1894 | 4.15 | tr|Q8TEM4|Q8TEM4_HUMAN | FLJ00169 protein (Fragment) OS=Homo sapiens GN=FLJ00169 PE=2 SV=1 | 46513.1 | 432 | 11.57 | 3 | 3 | 8 | 8 |
| 1895 | 4.14 | tr|A0A0A0MTC5|A0A0A0MTC5_HUMAN | Double-stranded RNA-binding protein Staufen homolog 2 OS=Homo sapiens GN=STAU2 PE=1 SV=1 | 62608.1 | 570 | 12.11 | 6 | 3 | 9 | 3 |
| 1896 | 4.14 | tr|A8K2Q7|A8K2Q7_HUMAN | cDNA FLJ77928, highly similar to Homo sapiens acyl-CoA synthetase medium-chain family member 3 (ACSM3), transcript variant 1, mRNA OS=Homo sapiens PE=2 SV=1 | 66154.2 | 586 | 5.461 | 3 | 3 | 11 | 11 |
| 1897 | 4.14 | tr|Q6AI42|Q6AI42_HUMAN | Putative uncharacterized protein DKFZp686E1450 OS=Homo sapiens GN=DKFZp686E1450 PE=2 SV=1 | 35619.3 | 308 | 7.143 | 2 | 1 | 3 | 1 |
| 1898 | 4.14 | tr|D9HTE9|D9HTE9_HUMAN | Plasma membrane citrate carrier OS=Homo sapiens GN=SLC25A1 PE=2 SV=1 | 35052.5 | 318 | 6.289 | 2 | 2 | 27 | 27 |
| 1899 | 4.13 | sp|P46199|IF2M_HUMAN | Translation initiation factor IF-2, mitochondrial OS=Homo sapiens GN=MTIF2 PE=1 SV=2 | 81316.8 | 727 | 4.539 | 3 | 2 | 7 | 6 |
| 1900 | 4.13 | tr|A0A087WWP4|A0A087WWP4_HUMAN | Putative RNA-binding protein 15 OS=Homo sapiens GN=RBM15 PE=1 SV=1 | 102133.6 | 933 | 3.751 | 2 | 2 | 7 | 7 |
| 1901 | 4.13 | tr|B4DEM9|B4DEM9_HUMAN | Polymerase delta-interacting protein 2 OS=Homo sapiens GN=POLDIP2 PE=1 SV=1 | 39871.4 | 350 | 11.43 | 3 | 3 | 7 | 7 |
| 1902 | 4.12 | tr|A0A024RAV2|A0A024RAV2_HUMAN | RecQ protein-like (DNA helicase Q1-like), isoform CRA_a OS=Homo sapiens GN=RECQL PE=4 SV=1 | 73456.8 | 649 | 7.396 | 4 | 3 | 10 | 9 |
| 1903 | 4.11 | tr|B4DZI4|B4DZI4_HUMAN | Phospholipase A2 OS=Homo sapiens PE=2 SV=1 | 78260.2 | 689 | 4.935 | 3 | 3 | 11 | 11 |
| 1904 | 4.11 | tr|A0A0S2Z5P5|A0A0S2Z5P5_HUMAN | Chromosome 10 open reading frame 119, isoform CRA_c (Fragment) OS=Homo sapiens GN=MCMBP PE=2 SV=1 | 72748.2 | 640 | 3.125 | 2 | 2 | 3 | 3 |
| 1905 | 4.11 | tr|J3QRU8|J3QRU8_HUMAN | ARF GTPase-activating protein GIT1 OS=Homo sapiens GN=GIT1 PE=1 SV=1 | 76805.5 | 694 | 4.323 | 2 | 2 | 9 | 9 |
| 1906 | 4.11 | sp|O75190|DNJB6_HUMAN | DnaJ homolog subfamily B member 6 OS=Homo sapiens GN=DNAJB6 PE=1 SV=2 | 36086.8 | 326 | 7.975 | 2 | 2 | 9 | 9 |
| 1907 | 4.11 | sp|Q92520|FAM3C_HUMAN | Protein FAM3C OS=Homo sapiens GN=FAM3C PE=1 SV=1 | 24680.3 | 227 | 9.692 | 2 | 2 | 12 | 12 |
| 1908 | 4.11 | tr|Q05DF2|Q05DF2_HUMAN | SF3A2 protein (Fragment) OS=Homo sapiens GN=SF3A2 PE=2 SV=1 | 51475.5 | 481 | 4.158 | 2 | 2 | 3 | 3 |
| 1909 | 4.1 | tr|A8MXZ4|A8MXZ4_HUMAN | G-protein-coupled receptor family C group 5 member C (Fragment) OS=Homo sapiens GN=GPRC5C PE=1 SV=2 | 50167.9 | 462 | 5.628 | 2 | 2 | 5 | 5 |
| 1910 | 4.09 | tr|V9HW63|V9HW63_HUMAN | Epididymis secretory sperm binding protein Li 97n OS=Homo sapiens GN=HEL-S-97n PE=2 SV=1 | 30539.6 | 271 | 15.5 | 5 | 2 | 177 | 12 |
| 1911 | 4.09 | tr|A0A0C4DGX4|A0A0C4DGX4_HUMAN | Cullin-1 OS=Homo sapiens GN=CUL1 PE=1 SV=1 | 87387.3 | 752 | 4.122 | 2 | 2 | 10 | 10 |
| 1912 | 4.09 | tr|B4E2S2|B4E2S2_HUMAN | Poly [ADP-ribose] polymerase OS=Homo sapiens PE=2 SV=1 | 112791.1 | 1005 | 3.085 | 2 | 2 | 5 | 5 |
| 1913 | 4.09 | sp|Q9Y3D6|FIS1_HUMAN | Mitochondrial fission 1 protein OS=Homo sapiens GN=FIS1 PE=1 SV=2 | 16937.6 | 152 | 15.13 | 2 | 2 | 14 | 14 |
| 1914 | 4.09 | tr|A0A087WUB9|A0A087WUB9_HUMAN | Beta-catenin-like protein 1 OS=Homo sapiens GN=CTNNBL1 PE=1 SV=1 | 65702.4 | 568 | 5.634 | 2 | 2 | 9 | 9 |
| 1915 | 4.08 | tr|B2R9T9|B2R9T9_HUMAN | cDNA, FLJ94551 OS=Homo sapiens PE=2 SV=1 | 26193.7 | 243 | 11.11 | 3 | 2 | 21 | 20 |
| 1916 | 4.08 | sp|Q8IWA0|WDR75_HUMAN | WD repeat-containing protein 75 OS=Homo sapiens GN=WDR75 PE=1 SV=1 | 94497.9 | 830 | 4.458 | 3 | 3 | 4 | 4 |
| 1917 | 4.08 | tr|Q2TA84|Q2TA84_HUMAN | FK506 binding protein 5 OS=Homo sapiens GN=FKBP5 PE=2 SV=1 | 51177.8 | 457 | 4.158 | 2 | 2 | 2 | 2 |
| 1918 | 4.08 | tr|Q5T8U7|Q5T8U7_HUMAN | Surfeit 4 OS=Homo sapiens GN=SURF4 PE=4 SV=1 | 21129.4 | 188 | 14.89 | 2 | 2 | 18 | 18 |
| 1919 | 4.08 | tr|H0YGX7|H0YGX7_HUMAN | Rho GDP-dissociation inhibitor 2 (Fragment) OS=Homo sapiens GN=ARHGDIB PE=1 SV=1 | 22330.1 | 195 | 10.77 | 2 | 2 | 3 | 3 |
| 1920 | 4.07 | sp|Q5SRD1|TI23B_HUMAN | Putative mitochondrial import inner membrane translocase subunit Tim23B OS=Homo sapiens GN=TIMM23B PE=5 SV=2 | 28048.2 | 257 | 14.4 | 2 | 2 | 10 | 10 |
| 1921 | 4.07 | sp|Q96SQ9|CP2S1_HUMAN | Cytochrome P450 2S1 OS=Homo sapiens GN=CYP2S1 PE=1 SV=2 | 55816.2 | 504 | 4.762 | 2 | 2 | 12 | 12 |
| 1922 | 4.07 | tr|A3KFL2|A3KFL2_HUMAN | Exosome complex component RRP4 OS=Homo sapiens GN=EXOSC2 PE=1 SV=1 | 31993.8 | 285 | 7.368 | 2 | 2 | 8 | 8 |
| 1923 | 4.07 | sp|Q8NEN9|PDZD8_HUMAN | PDZ domain-containing protein 8 OS=Homo sapiens GN=PDZD8 PE=1 SV=1 | 128562.1 | 1154 | 2.686 | 2 | 2 | 3 | 3 |
| 1924 | 4.07 | tr|A0A0C4DGQ5|A0A0C4DGQ5_HUMAN | Calpain small subunit 1 OS=Homo sapiens GN=CAPNS1 PE=1 SV=1 | 33785.8 | 322 | 7.143 | 2 | 2 | 6 | 6 |
| 1925 | 4.07 | tr|H3BVD9|H3BVD9_HUMAN | Kunitz-type protease inhibitor 1 (Fragment) OS=Homo sapiens GN=SPINT1 PE=1 SV=1 | 52373.5 | 472 | 5.508 | 2 | 2 | 6 | 6 |
| 1926 | 4.06 | tr|A0A024R7I3|A0A024R7I3_HUMAN | RAB8A, member RAS oncogene family, isoform CRA_a OS=Homo sapiens GN=RAB8A PE=4 SV=1 | 23668 | 207 | 31.88 | 8 | 3 | 115 | 6 |
| 1927 | 4.06 | tr|Q6FHV6|Q6FHV6_HUMAN | ENO2 protein OS=Homo sapiens GN=ENO2 PE=1 SV=1 | 47268.1 | 434 | 19.35 | 9 | 4 | 98 | 16 |
| 1928 | 4.06 | tr|A0A090N8Q3|A0A090N8Q3_HUMAN | Chromosome 7 open reading frame 21 OS=Homo sapiens GN=C7orf21 PE=4 SV=1 | 26261 | 246 | 23.17 | 5 | 2 | 22 | 2 |
| 1929 | 4.06 | sp|Q14669|TRIPC_HUMAN | E3 ubiquitin-protein ligase TRIP12 OS=Homo sapiens GN=TRIP12 PE=1 SV=1 | 220431.5 | 1992 | 2.41 | 4 | 4 | 11 | 6 |
| 1930 | 4.06 | sp|Q86W92|LIPB1_HUMAN | Liprin-beta-1 OS=Homo sapiens GN=PPFIBP1 PE=1 SV=2 | 114023.1 | 1011 | 3.363 | 3 | 2 | 9 | 8 |
| 1931 | 4.06 | tr|B2RDD7|B2RDD7_HUMAN | Protein arginine N-methyltransferase 5 OS=Homo sapiens PE=2 SV=1 | 72709.3 | 637 | 3.611 | 3 | 3 | 4 | 4 |
| 1932 | 4.06 | tr|A0A024RDI6|A0A024RDI6_HUMAN | Nudix (Nucleoside diphosphate linked moiety X)-type motif 9, isoform CRA_b OS=Homo sapiens GN=NUDT9 PE=4 SV=1 | 39124.7 | 350 | 7.429 | 2 | 2 | 5 | 5 |
| 1933 | 4.06 | tr|Q59HH7|Q59HH7_HUMAN | X-ray repair cross complementing protein 1 variant (Fragment) OS=Homo sapiens PE=2 SV=1 | 71013.5 | 647 | 5.564 | 3 | 3 | 7 | 7 |
| 1934 | 4.06 | tr|W4VSQ9|W4VSQ9_HUMAN | Cdc42-interacting protein 4 OS=Homo sapiens GN=TRIP10 PE=1 SV=1 | 67730.3 | 593 | 3.035 | 2 | 2 | 3 | 3 |
| 1935 | 4.06 | tr|K7ERJ1|K7ERJ1_HUMAN | Thymidine kinase (Fragment) OS=Homo sapiens GN=TK1 PE=1 SV=1 | 20525.8 | 187 | 15.51 | 2 | 2 | 8 | 8 |
| 1936 | 4.05 | tr|A0A024R4F4|A0A024R4F4_HUMAN | DNA polymerase OS=Homo sapiens GN=POLD1 PE=3 SV=1 | 123629.9 | 1107 | 4.788 | 6 | 6 | 10 | 8 |
| 1937 | 4.05 | sp|Q14151|SAFB2_HUMAN | Scaffold attachment factor B2 OS=Homo sapiens GN=SAFB2 PE=1 SV=1 | 107472.6 | 953 | 3.673 | 3 | 2 | 5 | 4 |
| 1938 | 4.05 | sp|Q9UID3|VPS51_HUMAN | Vacuolar protein sorting-associated protein 51 homolog OS=Homo sapiens GN=VPS51 PE=1 SV=2 | 86041.4 | 782 | 3.836 | 2 | 2 | 3 | 3 |
| 1939 | 4.05 | tr|A0A024RD08|A0A024RD08_HUMAN | Mitochondrial carrier homolog 1 (C. elegans), isoform CRA_d OS=Homo sapiens GN=MTCH1 PE=3 SV=1 | 39919.3 | 372 | 5.645 | 2 | 2 | 3 | 3 |
| 1940 | 4.05 | sp|Q53F19|NCBP3_HUMAN | Nuclear cap-binding protein subunit 3 OS=Homo sapiens GN=NCBP3 PE=1 SV=2 | 70591.8 | 620 | 4.194 | 2 | 2 | 3 | 3 |
| 1941 | 4.05 | sp|P28799|GRN_HUMAN | Granulins OS=Homo sapiens GN=GRN PE=1 SV=2 | 63543.8 | 593 | 5.396 | 3 | 3 | 4 | 4 |
| 1942 | 4.05 | sp|Q96GA3|LTV1_HUMAN | Protein LTV1 homolog OS=Homo sapiens GN=LTV1 PE=1 SV=1 | 54854.4 | 475 | 4.632 | 2 | 2 | 3 | 3 |
| 1943 | 4.05 | tr|A0A024RAQ3|A0A024RAQ3_HUMAN | Dihydrofolate reductase, isoform CRA_a OS=Homo sapiens GN=DHFR PE=3 SV=1 | 28843.9 | 262 | 11.45 | 2 | 2 | 6 | 6 |
| 1944 | 4.05 | tr|A0A024RCA7|A0A024RCA7_HUMAN | Ribosomal protein, large, P2, isoform CRA_a OS=Homo sapiens GN=RPLP2 PE=3 SV=1 | 11664.8 | 115 | 39.13 | 2 | 2 | 15 | 14 |
| 1945 | 4.05 | tr|Q53HG1|Q53HG1_HUMAN | 13kDa differentiation-associated protein variant (Fragment) OS=Homo sapiens PE=2 SV=1 | 17214.5 | 146 | 19.86 | 2 | 2 | 3 | 3 |
| 1946 | 4.04 | sp|P08579|RU2B_HUMAN | U2 small nuclear ribonucleoprotein B'' OS=Homo sapiens GN=SNRPB2 PE=1 SV=1 | 25486.2 | 225 | 24.89 | 5 | 3 | 27 | 10 |
| 1947 | 4.04 | tr|V9HW04|V9HW04_HUMAN | Serine/threonine-protein phosphatase OS=Homo sapiens GN=HEL-S-80p PE=2 SV=1 | 37186.5 | 327 | 14.68 | 4 | 2 | 23 | 21 |
| 1948 | 4.04 | tr|A0A024R471|A0A024R471_HUMAN | COP9 constitutive photomorphogenic homolog subunit 7B (Arabidopsis), isoform CRA_b OS=Homo sapiens GN=COPS7B PE=4 SV=1 | 29621.7 | 264 | 13.64 | 3 | 2 | 8 | 7 |
| 1949 | 4.04 | sp|Q7Z7H5|TMED4_HUMAN | Transmembrane emp24 domain-containing protein 4 OS=Homo sapiens GN=TMED4 PE=1 SV=1 | 25942.7 | 227 | 17.62 | 3 | 2 | 5 | 3 |
| 1950 | 4.04 | tr|Q658K0|Q658K0_HUMAN | Putative uncharacterized protein DKFZp564P2364 OS=Homo sapiens GN=DKFZp564P2364 PE=2 SV=1 | 107639.7 | 940 | 2.34 | 2 | 2 | 2 | 2 |
| 1951 | 4.04 | sp|Q9Y3Y2|CHTOP_HUMAN | Chromatin target of PRMT1 protein OS=Homo sapiens GN=CHTOP PE=1 SV=2 | 26396.4 | 248 | 10.48 | 2 | 2 | 8 | 7 |
| 1952 | 4.04 | tr|B1AKZ5|B1AKZ5_HUMAN | Astrocytic phosphoprotein PEA-15 OS=Homo sapiens GN=PEA15 PE=1 SV=1 | 12530.2 | 108 | 12.04 | 2 | 2 | 6 | 6 |
| 1953 | 4.03 | sp|P08754|GNAI3_HUMAN | Guanine nucleotide-binding protein G(k) subunit alpha OS=Homo sapiens GN=GNAI3 PE=1 SV=3 | 40531.8 | 354 | 18.36 | 5 | 2 | 61 | 16 |
| 1954 | 4.03 | tr|V9HWE7|V9HWE7_HUMAN | Epididymis secretory protein Li 19 OS=Homo sapiens GN=HEL-S-19 PE=2 SV=1 | 42271.8 | 392 | 9.184 | 4 | 3 | 10 | 9 |
| 1955 | 4.03 | tr|Q8WUW2|Q8WUW2_HUMAN | 1-acyl-sn-glycerol-3-phosphate acyltransferase OS=Homo sapiens GN=AGPAT2 PE=2 SV=1 | 31027.2 | 279 | 13.98 | 4 | 2 | 21 | 2 |
| 1956 | 4.03 | tr|F6TB26|F6TB26_HUMAN | Tudor and KH domain-containing protein OS=Homo sapiens GN=TDRKH PE=1 SV=1 | 66967.1 | 605 | 5.62 | 2 | 2 | 3 | 3 |
| 1957 | 4.03 | tr|K7ELC2|K7ELC2_HUMAN | 40S ribosomal protein S15 OS=Homo sapiens GN=RPS15 PE=1 SV=1 | 17722.8 | 152 | 15.13 | 2 | 2 | 10 | 9 |
| 1958 | 4.03 | sp|Q8NBF2|NHLC2_HUMAN | NHL repeat-containing protein 2 OS=Homo sapiens GN=NHLRC2 PE=1 SV=1 | 79442.9 | 726 | 3.168 | 2 | 2 | 6 | 6 |
| 1959 | 4.03 | tr|Q1W6H1|Q1W6H1_HUMAN | DNA-3-methyladenine glycosylase OS=Homo sapiens GN=MPG PE=3 SV=1 | 32868.4 | 298 | 11.07 | 2 | 2 | 6 | 6 |
| 1960 | 4.03 | sp|Q9P013|CWC15_HUMAN | Spliceosome-associated protein CWC15 homolog OS=Homo sapiens GN=CWC15 PE=1 SV=2 | 26624.1 | 229 | 8.297 | 2 | 2 | 2 | 2 |
| 1961 | 4.03 | tr|A0A024R6Q2|A0A024R6Q2_HUMAN | Chromosome 14 open reading frame 172, isoform CRA_b OS=Homo sapiens GN=C14orf172 PE=4 SV=1 | 31381.5 | 289 | 14.88 | 2 | 2 | 2 | 2 |
| 1962 | 4.03 | sp|Q9BYG5|PAR6B_HUMAN | Partitioning defective 6 homolog beta OS=Homo sapiens GN=PARD6B PE=1 SV=1 | 41181.9 | 372 | 6.452 | 2 | 2 | 7 | 7 |
| 1963 | 4.03 | sp|Q96HR9|REEP6_HUMAN | Receptor expression-enhancing protein 6 OS=Homo sapiens GN=REEP6 PE=1 SV=1 | 20733 | 184 | 12.5 | 2 | 2 | 6 | 6 |
| 1964 | 4.03 | sp|Q99622|C10_HUMAN | Protein C10 OS=Homo sapiens GN=C12orf57 PE=1 SV=1 | 13178 | 126 | 24.6 | 2 | 2 | 6 | 6 |
| 1965 | 4.02 | sp|Q9BS40|LXN_HUMAN | Latexin OS=Homo sapiens GN=LXN PE=1 SV=2 | 25750.1 | 222 | 14.41 | 3 | 2 | 4 | 3 |
| 1966 | 4.02 | tr|Q0MQR4|Q0MQR4_HUMAN | Poly (ADP-ribose) glycohydrolase OS=Homo sapiens GN=PARG PE=2 SV=1 | 110969.3 | 976 | 1.844 | 2 | 2 | 3 | 3 |
| 1967 | 4.02 | tr|A0A024R8R4|A0A024R8R4_HUMAN | Nuclear protein localization 4 homolog (S. cerevisiae), isoform CRA_a OS=Homo sapiens GN=NPLOC4 PE=4 SV=1 | 68119.7 | 608 | 4.605 | 2 | 2 | 3 | 3 |
| 1968 | 4.02 | sp|Q5SRE5|NU188_HUMAN | Nucleoporin NUP188 homolog OS=Homo sapiens GN=NUP188 PE=1 SV=1 | 196041 | 1749 | 2.344 | 3 | 2 | 6 | 4 |
| 1969 | 4.02 | tr|A0A140VK05|A0A140VK05_HUMAN | Testicular secretory protein Li 5 OS=Homo sapiens PE=2 SV=1 | 97340.6 | 903 | 5.316 | 3 | 3 | 4 | 4 |
| 1970 | 4.02 | sp|Q9BT22|ALG1_HUMAN | Chitobiosyldiphosphodolichol beta-mannosyltransferase OS=Homo sapiens GN=ALG1 PE=1 SV=2 | 52517.7 | 464 | 4.741 | 2 | 2 | 2 | 2 |
| 1971 | 4.02 | sp|Q8TED0|UTP15_HUMAN | U3 small nucleolar RNA-associated protein 15 homolog OS=Homo sapiens GN=UTP15 PE=1 SV=3 | 58414.7 | 518 | 6.371 | 2 | 2 | 7 | 7 |
| 1972 | 4.02 | tr|D7RF68|D7RF68_HUMAN | AGTRAP-BRAF fusion protein OS=Homo sapiens PE=2 SV=1 | 66205.2 | 597 | 6.533 | 2 | 2 | 13 | 13 |
| 1973 | 4.02 | tr|H0Y714|H0Y714_HUMAN | U3 small nucleolar ribonucleoprotein protein IMP4 (Fragment) OS=Homo sapiens GN=IMP4 PE=1 SV=1 | 31892.4 | 280 | 8.571 | 2 | 2 | 5 | 5 |
| 1974 | 4.02 | tr|B4E0R5|B4E0R5_HUMAN | cDNA FLJ61517, highly similar to Homo sapiens ubiquitin associated protein 2 (UBAP2), transcript variant 1, mRNA OS=Homo sapiens PE=2 SV=1 | 87925.1 | 852 | 3.521 | 2 | 2 | 2 | 2 |
| 1975 | 4.02 | tr|B0QYN7|B0QYN7_HUMAN | SUMO-conjugating enzyme OS=Homo sapiens GN=UBE2I PE=1 SV=1 | 20457.4 | 184 | 12.5 | 2 | 2 | 14 | 14 |
| 1976 | 4.02 | tr|Q9NUF9|Q9NUF9_HUMAN | Nucleoside diphosphate kinase (Fragment) OS=Homo sapiens GN=c371H6.2 PE=3 SV=1 | 17295.7 | 153 | 15.69 | 2 | 2 | 16 | 15 |
| 1977 | 4.02 | sp|Q9Y5J1|UTP18_HUMAN | U3 small nucleolar RNA-associated protein 18 homolog OS=Homo sapiens GN=UTP18 PE=1 SV=3 | 62003.1 | 556 | 5.216 | 2 | 2 | 11 | 11 |
| 1978 | 4.02 | sp|Q8IVM0|CCD50_HUMAN | Coiled-coil domain-containing protein 50 OS=Homo sapiens GN=CCDC50 PE=1 SV=1 | 35821.7 | 306 | 6.536 | 2 | 2 | 4 | 4 |
| 1979 | 4.02 | sp|Q96HY6|DDRGK_HUMAN | DDRGK domain-containing protein 1 OS=Homo sapiens GN=DDRGK1 PE=1 SV=2 | 35610.6 | 314 | 10.19 | 2 | 2 | 13 | 13 |
| 1980 | 4.02 | tr|E7EQ69|E7EQ69_HUMAN | N-alpha-acetyltransferase 50 OS=Homo sapiens GN=NAA50 PE=1 SV=1 | 19311.1 | 168 | 17.26 | 2 | 2 | 9 | 9 |
| 1981 | 4.02 | sp|P84101|SERF2_HUMAN | Small EDRK-rich factor 2 OS=Homo sapiens GN=SERF2 PE=1 SV=1 | 6899.8 | 59 | 30.51 | 2 | 2 | 8 | 8 |
| 1982 | 4.01 | tr|B7Z213|B7Z213_HUMAN | cDNA FLJ50130, highly similar to Heterogeneous nuclear ribonucleoprotein Q OS=Homo sapiens PE=2 SV=1 | 28578.6 | 256 | 32.81 | 9 | 4 | 48 | 12 |
| 1983 | 4.01 | sp|Q15286|RAB35_HUMAN | Ras-related protein Rab-35 OS=Homo sapiens GN=RAB35 PE=1 SV=1 | 23025.1 | 201 | 30.35 | 5 | 3 | 98 | 8 |
| 1984 | 4.01 | sp|Q9NQ29|LUC7L_HUMAN | Putative RNA-binding protein Luc7-like 1 OS=Homo sapiens GN=LUC7L PE=1 SV=1 | 43727.5 | 371 | 15.09 | 5 | 2 | 13 | 3 |
| 1985 | 4.01 | tr|A0A087WZH7|A0A087WZH7_HUMAN | Myristoylated alanine-rich C-kinase substrate OS=Homo sapiens GN=MARCKS PE=1 SV=1 | 31595.4 | 330 | 17.27 | 3 | 2 | 3 | 2 |
| 1986 | 4.01 | sp|Q14BN4|SLMAP_HUMAN | Sarcolemmal membrane-associated protein OS=Homo sapiens GN=SLMAP PE=1 SV=1 | 95197.5 | 828 | 2.536 | 2 | 2 | 4 | 4 |
| 1987 | 4.01 | sp|Q01415|GALK2_HUMAN | N-acetylgalactosamine kinase OS=Homo sapiens GN=GALK2 PE=1 SV=1 | 50377.8 | 458 | 6.769 | 2 | 2 | 9 | 9 |
| 1988 | 4.01 | sp|Q9NRG0|CHRC1_HUMAN | Chromatin accessibility complex protein 1 OS=Homo sapiens GN=CHRAC1 PE=1 SV=1 | 14710.4 | 131 | 19.85 | 2 | 2 | 14 | 14 |
| 1989 | 4.01 | sp|Q13190|STX5_HUMAN | Syntaxin-5 OS=Homo sapiens GN=STX5 PE=1 SV=2 | 39672.2 | 355 | 7.887 | 2 | 2 | 7 | 7 |
| 1990 | 4.01 | tr|A0A024R9V3|A0A024R9V3_HUMAN | KIAA0746 protein, isoform CRA_a OS=Homo sapiens GN=KIAA0746 PE=4 SV=1 | 111761.1 | 979 | 3.064 | 2 | 2 | 4 | 4 |
| 1991 | 4.01 | tr|A8K905|A8K905_HUMAN | cDNA FLJ77615, highly similar to Homo sapiens nucleolar complex associated 3 homolog (S. cerevisiae) (NOC3L), mRNA OS=Homo sapiens PE=2 SV=1 | 92547.9 | 800 | 3.25 | 2 | 2 | 4 | 3 |
| 1992 | 4.01 | tr|Q5TDE9|Q5TDE9_HUMAN | Chromosome 1 open reading frame 57, isoform CRA_d OS=Homo sapiens GN=C1orf57 PE=2 SV=1 | 20712.9 | 190 | 14.21 | 2 | 2 | 11 | 11 |
| 1993 | 4.01 | tr|F2Z2X4|F2Z2X4_HUMAN | Exportin-4 OS=Homo sapiens GN=XPO4 PE=1 SV=1 | 130152.2 | 1151 | 2.78 | 2 | 2 | 5 | 5 |
| 1994 | 4.01 | tr|A8K946|A8K946_HUMAN | mRNA cap guanine-N7 methyltransferase OS=Homo sapiens PE=2 SV=1 | 57750.1 | 504 | 4.365 | 2 | 2 | 4 | 4 |
| 1995 | 4.01 | sp|Q96T76|MMS19_HUMAN | MMS19 nucleotide excision repair protein homolog OS=Homo sapiens GN=MMS19 PE=1 SV=2 | 113288.2 | 1030 | 3.301 | 2 | 2 | 3 | 3 |
| 1996 | 4.01 | tr|B2RTX8|B2RTX8_HUMAN | WAPAL protein OS=Homo sapiens GN=WAPAL PE=2 SV=1 | 132224.8 | 1184 | 2.534 | 2 | 2 | 4 | 4 |
| 1997 | 4.01 | tr|B7Z6D1|B7Z6D1_HUMAN | cDNA FLJ57430, highly similar to DNA-directed RNA polymerase II 33 kDa polypeptide (EC 2.7.7.6) OS=Homo sapiens PE=2 SV=1 | 47444.9 | 424 | 6.132 | 2 | 2 | 4 | 4 |
| 1998 | 4.01 | sp|Q6DKJ4|NXN_HUMAN | Nucleoredoxin OS=Homo sapiens GN=NXN PE=1 SV=2 | 48391.6 | 435 | 6.437 | 2 | 2 | 4 | 4 |
| 1999 | 4.01 | sp|P82673|RT35_HUMAN | 28S ribosomal protein S35, mitochondrial OS=Homo sapiens GN=MRPS35 PE=1 SV=1 | 36844 | 323 | 11.46 | 2 | 2 | 5 | 5 |
| 2000 | 4.01 | sp|O15294|OGT1_HUMAN | UDP-N-acetylglucosamine--peptide N-acetylglucosaminyltransferase 110 kDa subunit OS=Homo sapiens GN=OGT PE=1 SV=3 | 116923.5 | 1046 | 5.832 | 3 | 2 | 3 | 2 |
| 2001 | 4.01 | sp|Q96NC0|ZMAT2_HUMAN | Zinc finger matrin-type protein 2 OS=Homo sapiens GN=ZMAT2 PE=1 SV=1 | 23611.8 | 199 | 4.523 | 2 | 2 | 3 | 3 |
| 2002 | 4.01 | sp|P98172|EFNB1_HUMAN | Ephrin-B1 OS=Homo sapiens GN=EFNB1 PE=1 SV=1 | 38006.3 | 346 | 8.96 | 2 | 2 | 23 | 23 |
| 2003 | 4.01 | tr|A8MVM1|A8MVM1_HUMAN | Caspase-3 OS=Homo sapiens GN=CASP3 PE=1 SV=2 | 20508.2 | 182 | 12.09 | 2 | 2 | 4 | 4 |
| 2004 | 4.01 | tr|H0YH87|H0YH87_HUMAN | Ataxin-2 (Fragment) OS=Homo sapiens GN=ATXN2 PE=1 SV=1 | 98060.9 | 916 | 2.838 | 2 | 2 | 6 | 6 |
| 2005 | 4.01 | tr|B7Z2R7|B7Z2R7_HUMAN | Acyl-CoA-binding domain-containing protein 5 OS=Homo sapiens GN=ACBD5 PE=1 SV=1 | 58792 | 523 | 5.736 | 2 | 2 | 4 | 4 |
| 2006 | 4.01 | tr|B5ME97|B5ME97_HUMAN | Septin 10, isoform CRA_c OS=Homo sapiens GN=SEPT10 PE=1 SV=2 | 62944.6 | 544 | 4.596 | 2 | 2 | 6 | 6 |
| 2007 | 4.01 | tr|A0A023T787|A0A023T787_HUMAN | RNA-binding protein 8A OS=Homo sapiens GN=RBM8 PE=2 SV=1 | 19888.9 | 174 | 16.09 | 2 | 2 | 8 | 8 |
| 2008 | 4.01 | sp|Q9Y333|LSM2_HUMAN | U6 snRNA-associated Sm-like protein LSm2 OS=Homo sapiens GN=LSM2 PE=1 SV=1 | 10834.4 | 95 | 21.05 | 2 | 2 | 32 | 32 |
| 2009 | 4.01 | tr|G3XAN8|G3XAN8_HUMAN | Mitochondrial import inner membrane translocase subunit Tim8 B OS=Homo sapiens GN=TIMM8B PE=1 SV=1 | 11154.7 | 98 | 21.43 | 2 | 2 | 8 | 8 |
| 2010 | 4.01 | tr|Q7Z612|Q7Z612_HUMAN | Acidic ribosomal phosphoprotein P1 OS=Homo sapiens PE=2 SV=1 | 11398.7 | 113 | 56.64 | 3 | 3 | 19 | 19 |
| 2011 | 4 | tr|E9PMV1|E9PMV1_HUMAN | Plectin (Fragment) OS=Homo sapiens GN=PLEC PE=1 SV=1 | 80779.6 | 703 | 40.68 | 25 | 2 | 173 | 7 |
| 2012 | 4 | tr|C7DJS2|C7DJS2_HUMAN | Glutathione S-transferase pi (Fragment) OS=Homo sapiens GN=GSTP1 PE=2 SV=1 | 16666.8 | 151 | 43.71 | 6 | 2 | 173 | 59 |
| 2013 | 4 | tr|Q9UPN1|Q9UPN1_HUMAN | Serine/threonine-protein phosphatase (Fragment) OS=Homo sapiens GN=PPP1CC PE=3 SV=1 | 33773.5 | 294 | 16.67 | 4 | 2 | 8 | 6 |
| 2014 | 4 | tr|Q504R6|Q504R6_HUMAN | RAB13 protein (Fragment) OS=Homo sapiens GN=RAB13 PE=2 SV=1 | 27198.9 | 244 | 17.62 | 4 | 2 | 49 | 7 |
| 2015 | 4 | tr|Q8TBR3|Q8TBR3_HUMAN | Fusion (Involved in t(1216) in malignant liposarcoma) OS=Homo sapiens GN=FUS PE=2 SV=1 | 53400 | 526 | 7.414 | 4 | 2 | 27 | 8 |
| 2016 | 4 | tr|B3KRJ9|B3KRJ9_HUMAN | cDNA FLJ34439 fis, clone HLUNG2001146, highly similar to Splicing factor, arginine/serine-rich 12 OS=Homo sapiens PE=2 SV=1 | 58202.3 | 514 | 5.837 | 3 | 3 | 7 | 4 |
| 2017 | 4 | tr|Q59GV6|Q59GV6_HUMAN | Zinedin variant (Fragment) OS=Homo sapiens PE=2 SV=1 | 80588.2 | 755 | 3.841 | 2 | 2 | 4 | 4 |
| 2018 | 4 | sp|Q9BVI4|NOC4L_HUMAN | Nucleolar complex protein 4 homolog OS=Homo sapiens GN=NOC4L PE=1 SV=1 | 58467.1 | 516 | 4.264 | 2 | 2 | 11 | 11 |
| 2019 | 4 | tr|A0A0S2Z3Y7|A0A0S2Z3Y7_HUMAN | Galactose-1-phosphate uridylyltransferase (Fragment) OS=Homo sapiens GN=GALT PE=2 SV=1 | 43362.8 | 379 | 6.596 | 2 | 2 | 2 | 2 |
| 2020 | 4 | tr|E9PGT1|E9PGT1_HUMAN | Translin OS=Homo sapiens GN=TSN PE=1 SV=1 | 25572.1 | 223 | 12.11 | 2 | 2 | 18 | 18 |
| 2021 | 4 | tr|Q6FGR8|Q6FGR8_HUMAN | Major prion protein OS=Homo sapiens GN=PRNP PE=2 SV=1 | 27599.1 | 253 | 10.28 | 3 | 3 | 5 | 4 |
| 2022 | 4 | sp|Q9BYD2|RM09_HUMAN | 39S ribosomal protein L9, mitochondrial OS=Homo sapiens GN=MRPL9 PE=1 SV=2 | 30243.1 | 267 | 8.989 | 2 | 2 | 8 | 8 |
| 2023 | 4 | sp|P49720|PSB3_HUMAN | Proteasome subunit beta type-3 OS=Homo sapiens GN=PSMB3 PE=1 SV=2 | 22948.7 | 205 | 14.63 | 2 | 2 | 41 | 41 |
| 2024 | 4 | tr|Q53H29|Q53H29_HUMAN | Nucleoporin 54kDa variant (Fragment) OS=Homo sapiens PE=2 SV=1 | 55563.3 | 508 | 4.921 | 2 | 2 | 11 | 11 |
| 2025 | 4 | tr|A0A0K0K1J1|A0A0K0K1J1_HUMAN | Cystatin OS=Homo sapiens GN=HEL-S-2 PE=2 SV=1 | 15799.1 | 146 | 19.18 | 2 | 2 | 5 | 5 |
| 2026 | 4 | tr|A8K3Z5|A8K3Z5_HUMAN | Nucleoporin NUP53 OS=Homo sapiens PE=2 SV=1 | 34814.6 | 326 | 9.509 | 2 | 2 | 9 | 9 |
| 2027 | 4 | tr|Q53HA5|Q53HA5_HUMAN | CDP-diacylglycerol--inositol 3-phosphatidyltransferase isoform 1 variant (Fragment) OS=Homo sapiens PE=2 SV=1 | 23557.5 | 213 | 9.859 | 2 | 2 | 3 | 3 |
| 2028 | 4 | tr|E7EMP6|E7EMP6_HUMAN | Embryonic stem cell-specific 5-hydroxymethylcytosine-binding protein OS=Homo sapiens GN=HMCES PE=1 SV=1 | 35570.8 | 312 | 8.974 | 2 | 2 | 6 | 6 |
| 2029 | 4 | tr|D3DUP2|D3DUP2_HUMAN | WNK lysine deficient protein kinase 1, isoform CRA_d OS=Homo sapiens GN=WNK1 PE=4 SV=1 | 222700.9 | 2107 | 1.092 | 2 | 2 | 6 | 6 |
| 2030 | 4 | sp|Q96GC5|RM48_HUMAN | 39S ribosomal protein L48, mitochondrial OS=Homo sapiens GN=MRPL48 PE=1 SV=2 | 23934.6 | 212 | 11.79 | 2 | 2 | 8 | 8 |
| 2031 | 4 | sp|Q9HD43|PTPRH_HUMAN | Receptor-type tyrosine-protein phosphatase H OS=Homo sapiens GN=PTPRH PE=1 SV=3 | 122351.4 | 1115 | 3.139 | 2 | 2 | 5 | 5 |
| 2032 | 4 | sp|P13995|MTDC_HUMAN | Bifunctional methylenetetrahydrofolate dehydrogenase/cyclohydrolase, mitochondrial OS=Homo sapiens GN=MTHFD2 PE=1 SV=2 | 37894.8 | 350 | 6.571 | 2 | 2 | 16 | 15 |
| 2033 | 4 | sp|Q96I51|WBS16_HUMAN | Williams-Beuren syndrome chromosomal region 16 protein OS=Homo sapiens GN=WBSCR16 PE=1 SV=2 | 49996.5 | 464 | 8.621 | 2 | 2 | 2 | 2 |
| 2034 | 4 | sp|P25815|S100P_HUMAN | Protein S100-P OS=Homo sapiens GN=S100P PE=1 SV=2 | 10399.8 | 95 | 17.89 | 2 | 2 | 12 | 12 |
| 2035 | 4 | sp|P17568|NDUB7_HUMAN | NADH dehydrogenase [ubiquinone] 1 beta subcomplex subunit 7 OS=Homo sapiens GN=NDUFB7 PE=1 SV=4 | 16401.8 | 137 | 18.25 | 2 | 2 | 4 | 4 |
| 2036 | 4 | sp|Q96T58|MINT_HUMAN | Msx2-interacting protein OS=Homo sapiens GN=SPEN PE=1 SV=1 | 402244.7 | 3664 | 0.9552 | 3 | 3 | 6 | 3 |
| 2037 | 4 | tr|Q2M1J6|Q2M1J6_HUMAN | Oxidase (Cytochrome c) assembly 1-like OS=Homo sapiens GN=OXA1L PE=2 SV=1 | 55384.5 | 496 | 4.032 | 2 | 2 | 3 | 3 |
| 2038 | 4 | sp|Q13505|MTX1_HUMAN | Metaxin-1 OS=Homo sapiens GN=MTX1 PE=1 SV=2 | 51476.4 | 466 | 5.794 | 2 | 2 | 7 | 7 |
| 2039 | 4 | tr|A0A024R9H2|A0A024R9H2_HUMAN | Heat-responsive protein 12, isoform CRA_a OS=Homo sapiens GN=HRSP12 PE=4 SV=1 | 14493.5 | 137 | 18.98 | 2 | 2 | 8 | 8 |
| 2040 | 4 | sp|Q9BYN0|SRXN1_HUMAN | Sulfiredoxin-1 OS=Homo sapiens GN=SRXN1 PE=1 SV=2 | 14259 | 137 | 15.33 | 2 | 2 | 3 | 3 |
| 2041 | 4 | tr|B2RDZ9|B2RDZ9_HUMAN | cDNA, FLJ96850 OS=Homo sapiens PE=2 SV=1 | 31874.2 | 291 | 8.591 | 2 | 2 | 3 | 3 |
| 2042 | 4 | tr|A8K1F4|A8K1F4_HUMAN | cDNA FLJ78094, highly similar to Homo sapiens myeloid leukemia factor 2, mRNA OS=Homo sapiens PE=1 SV=1 | 28129.1 | 248 | 8.468 | 2 | 2 | 3 | 3 |
| 2043 | 4 | tr|J3KN29|J3KN29_HUMAN | 26S proteasome non-ATPase regulatory subunit 9 OS=Homo sapiens GN=PSMD9 PE=1 SV=1 | 24553.5 | 222 | 8.559 | 2 | 2 | 6 | 6 |
| 2044 | 4 | tr|A0A024R012|A0A024R012_HUMAN | NAD-dependent protein deacylase sirtuin-5, mitochondrial OS=Homo sapiens GN=SIRT5 PE=3 SV=1 | 33880.6 | 310 | 11.61 | 2 | 2 | 6 | 6 |
| 2045 | 4 | sp|Q9Y2V2|CHSP1_HUMAN | Calcium-regulated heat-stable protein 1 OS=Homo sapiens GN=CARHSP1 PE=1 SV=2 | 15892 | 147 | 16.33 | 2 | 2 | 3 | 3 |
| 2046 | 4 | tr|Q6FGA0|Q6FGA0_HUMAN | COX7A2L protein OS=Homo sapiens GN=COX7A2L PE=2 SV=1 | 12614.6 | 114 | 19.3 | 2 | 2 | 2 | 2 |
| 2047 | 4 | sp|Q14197|ICT1_HUMAN | Peptidyl-tRNA hydrolase ICT1, mitochondrial OS=Homo sapiens GN=ICT1 PE=1 SV=1 | 23629.9 | 206 | 11.65 | 2 | 2 | 8 | 8 |
| 2048 | 4 | tr|Q6IAW5|Q6IAW5_HUMAN | CALU protein OS=Homo sapiens GN=CALU PE=2 SV=1 | 37106.5 | 315 | 4.762 | 2 | 2 | 16 | 16 |
| 2049 | 4 | tr|C9JQV0|C9JQV0_HUMAN | Uncharacterized protein C7orf50 (Fragment) OS=Homo sapiens GN=C7orf50 PE=1 SV=1 | 21882.9 | 192 | 29.69 | 2 | 2 | 3 | 3 |
| 2050 | 4 | sp|O95989|NUDT3_HUMAN | Diphosphoinositol polyphosphate phosphohydrolase 1 OS=Homo sapiens GN=NUDT3 PE=1 SV=1 | 19470.9 | 172 | 13.95 | 2 | 2 | 9 | 9 |
| 2051 | 4 | sp|O14561|ACPM_HUMAN | Acyl carrier protein, mitochondrial OS=Homo sapiens GN=NDUFAB1 PE=1 SV=3 | 17417.1 | 156 | 12.82 | 2 | 2 | 42 | 42 |
| 2052 | 4 | sp|P56385|ATP5I_HUMAN | ATP synthase subunit e, mitochondrial OS=Homo sapiens GN=ATP5I PE=1 SV=2 | 7933.1 | 69 | 31.88 | 2 | 2 | 38 | 38 |
| 2053 | 3.98 | tr|X5D2U3|X5D2U3_HUMAN | Serine threonine kinase 39 isoform A (Fragment) OS=Homo sapiens GN=STK39 PE=2 SV=1 | 57936.8 | 526 | 6.084 | 2 | 2 | 2 | 2 |
| 2054 | 3.97 | tr|B2RCZ4|B2RCZ4_HUMAN | Protein kinase C OS=Homo sapiens PE=2 SV=1 | 67199.7 | 587 | 6.644 | 4 | 3 | 4 | 3 |
| 2055 | 3.97 | tr|B7ZKQ9|B7ZKQ9_HUMAN | SCARB1 protein OS=Homo sapiens GN=SCARB1 PE=1 SV=1 | 53847.2 | 481 | 4.366 | 2 | 2 | 4 | 4 |
| 2056 | 3.96 | tr|A0A024R0A8|A0A024R0A8_HUMAN | Adenosylhomocysteinase OS=Homo sapiens GN=AHCYL1 PE=3 SV=1 | 58950.9 | 530 | 4.528 | 2 | 2 | 5 | 5 |
| 2057 | 3.96 | sp|P61769|B2MG_HUMAN | Beta-2-microglobulin OS=Homo sapiens GN=B2M PE=1 SV=1 | 13714.4 | 119 | 13.45 | 2 | 2 | 3 | 3 |
| 2058 | 3.96 | sp|Q9NVM9|ASUN_HUMAN | Protein asunder homolog OS=Homo sapiens GN=ASUN PE=1 SV=2 | 80224.4 | 706 | 3.683 | 2 | 2 | 4 | 4 |
| 2059 | 3.96 | tr|Q53EY5|Q53EY5_HUMAN | MORF-related gene X variant (Fragment) OS=Homo sapiens PE=2 SV=1 | 32311.6 | 288 | 11.81 | 3 | 3 | 5 | 5 |
| 2060 | 3.96 | tr|A0A140TA86|A0A140TA86_HUMAN | MICOS complex subunit MIC13 OS=Homo sapiens GN=C19orf70 PE=1 SV=1 | 15435.6 | 140 | 7.857 | 2 | 2 | 4 | 4 |
| 2061 | 3.96 | sp|Q92506|DHB8_HUMAN | Estradiol 17-beta-dehydrogenase 8 OS=Homo sapiens GN=HSD17B8 PE=1 SV=2 | 26973.6 | 261 | 9.195 | 2 | 2 | 2 | 2 |
| 2062 | 3.95 | sp|P33121|ACSL1_HUMAN | Long-chain-fatty-acid--CoA ligase 1 OS=Homo sapiens GN=ACSL1 PE=1 SV=1 | 77942.7 | 698 | 6.877 | 5 | 3 | 10 | 8 |
| 2063 | 3.95 | tr|A0A024RCR6|A0A024RCR6_HUMAN | HLA-B associated transcript 3, isoform CRA_a OS=Homo sapiens GN=BAT3 PE=1 SV=1 | 118702.1 | 1126 | 4.263 | 3 | 3 | 13 | 13 |
| 2064 | 3.94 | sp|Q9NPE3|NOP10_HUMAN | H/ACA ribonucleoprotein complex subunit 3 OS=Homo sapiens GN=NOP10 PE=1 SV=1 | 7705.9 | 64 | 48.44 | 3 | 3 | 6 | 6 |
| 2065 | 3.93 | sp|Q7L4I2|RSRC2_HUMAN | Arginine/serine-rich coiled-coil protein 2 OS=Homo sapiens GN=RSRC2 PE=1 SV=1 | 50559.2 | 434 | 5.069 | 2 | 2 | 4 | 4 |
| 2066 | 3.92 | tr|A0A024RAG3|A0A024RAG3_HUMAN | V-ral simian leukemia viral oncogene homolog B (Ras related GTP binding protein), isoform CRA_a OS=Homo sapiens GN=RALB PE=4 SV=1 | 23408.4 | 206 | 22.82 | 5 | 3 | 34 | 15 |
| 2067 | 3.92 | sp|Q8N3C0|ASCC3_HUMAN | Activating signal cointegrator 1 complex subunit 3 OS=Homo sapiens GN=ASCC3 PE=1 SV=3 | 251458.2 | 2202 | 2.18 | 5 | 4 | 8 | 4 |
| 2068 | 3.92 | tr|B2R4I8|B2R4I8_HUMAN | cDNA, FLJ92106, highly similar to Homo sapiens adaptor-related protein complex 3, sigma 1 subunit(AP3S1), mRNA OS=Homo sapiens PE=2 SV=1 | 21704.9 | 193 | 10.36 | 2 | 2 | 3 | 3 |
| 2069 | 3.91 | tr|Q53F02|Q53F02_HUMAN | Cleavage and polyadenylation specific factor 3, 73kDa variant (Fragment) OS=Homo sapiens PE=2 SV=1 | 77469.4 | 684 | 2.632 | 2 | 2 | 9 | 9 |
| 2070 | 3.91 | tr|H0YCL9|H0YCL9_HUMAN | AMP deaminase 2 (Fragment) OS=Homo sapiens GN=AMPD2 PE=1 SV=1 | 21811.3 | 195 | 10.26 | 2 | 2 | 5 | 5 |
| 2071 | 3.9 | tr|A0A024R546|A0A024R546_HUMAN | Palmitoyltransferase OS=Homo sapiens GN=ZDHHC5 PE=3 SV=1 | 77544 | 715 | 5.874 | 2 | 2 | 2 | 2 |
| 2072 | 3.89 | tr|Q6FI27|Q6FI27_HUMAN | GSK3B protein OS=Homo sapiens GN=GSK3B PE=2 SV=1 | 46743.9 | 420 | 8.095 | 3 | 2 | 5 | 4 |
| 2073 | 3.89 | tr|B4DRX6|B4DRX6_HUMAN | cDNA FLJ60339, highly similar to Phosphatidylinositol-4-phosphate 3-kinase C2 domain-containing alpha polypeptide (EC 2.7.1.154) OS=Homo sapiens PE=2 SV=1 | 72755.4 | 652 | 5.061 | 3 | 3 | 3 | 3 |
| 2074 | 3.89 | sp|Q6FI81|CPIN1_HUMAN | Anamorsin OS=Homo sapiens GN=CIAPIN1 PE=1 SV=2 | 33581.9 | 312 | 8.654 | 2 | 2 | 12 | 12 |
| 2075 | 3.89 | tr|Q5U0J5|Q5U0J5_HUMAN | cAMP responsive element binding protein 1 OS=Homo sapiens GN=CREB1 PE=2 SV=1 | 36687.9 | 341 | 12.61 | 2 | 2 | 4 | 4 |
| 2076 | 3.89 | tr|A0A087WWM0|A0A087WWM0_HUMAN | Trafficking protein particle complex subunit 3 OS=Homo sapiens GN=TRAPPC3 PE=1 SV=1 | 21231 | 188 | 10.11 | 2 | 2 | 5 | 5 |
| 2077 | 3.89 | sp|Q9Y5L4|TIM13_HUMAN | Mitochondrial import inner membrane translocase subunit Tim13 OS=Homo sapiens GN=TIMM13 PE=1 SV=1 | 10500 | 95 | 38.95 | 3 | 3 | 4 | 4 |
| 2078 | 3.88 | tr|Q53R19|Q53R19_HUMAN | Actin related protein 2/3 complex, subunit 2, 34kDa, isoform CRA_a OS=Homo sapiens GN=ARPC2 PE=2 SV=1 | 34332.7 | 300 | 10.33 | 4 | 4 | 25 | 25 |
| 2079 | 3.87 | sp|O60888|CUTA_HUMAN | Protein CutA OS=Homo sapiens GN=CUTA PE=1 SV=2 | 19116.1 | 179 | 15.64 | 2 | 2 | 17 | 17 |
| 2080 | 3.87 | sp|Q68CZ2|TENS3_HUMAN | Tensin-3 OS=Homo sapiens GN=TNS3 PE=1 SV=2 | 155264.2 | 1445 | 2.63 | 3 | 3 | 5 | 5 |
| 2081 | 3.87 | sp|Q15061|WDR43_HUMAN | WD repeat-containing protein 43 OS=Homo sapiens GN=WDR43 PE=1 SV=3 | 74890.1 | 677 | 6.499 | 3 | 3 | 4 | 4 |
| 2082 | 3.86 | sp|Q3MHD2|LSM12_HUMAN | Protein LSM12 homolog OS=Homo sapiens GN=LSM12 PE=1 SV=2 | 21700.6 | 195 | 22.56 | 3 | 3 | 15 | 15 |
| 2083 | 3.86 | sp|Q96AT9|RPE_HUMAN | Ribulose-phosphate 3-epimerase OS=Homo sapiens GN=RPE PE=1 SV=1 | 24927.6 | 228 | 12.28 | 2 | 2 | 6 | 6 |
| 2084 | 3.85 | sp|Q5T280|CI114_HUMAN | Putative methyltransferase C9orf114 OS=Homo sapiens GN=C9orf114 PE=1 SV=3 | 42008.4 | 376 | 6.383 | 2 | 2 | 5 | 5 |
| 2085 | 3.85 | tr|F5GXX5|F5GXX5_HUMAN | Dolichyl-diphosphooligosaccharide--protein glycosyltransferase subunit DAD1 OS=Homo sapiens GN=DAD1 PE=1 SV=1 | 9554.1 | 85 | 25.88 | 2 | 2 | 11 | 11 |
| 2086 | 3.84 | tr|A0A0S2Z4V6|A0A0S2Z4V6_HUMAN | Wolfram syndrome 1 isoform 1 (Fragment) OS=Homo sapiens GN=WFS1 PE=2 SV=1 | 100290.4 | 890 | 2.697 | 2 | 2 | 2 | 2 |
| 2087 | 3.84 | tr|A0A024R499|A0A024R499_HUMAN | Insulin receptor substrate 1, isoform CRA_a OS=Homo sapiens GN=IRS1 PE=4 SV=1 | 131589.8 | 1242 | 2.093 | 2 | 2 | 2 | 2 |
| 2088 | 3.82 | tr|Q5HYD8|Q5HYD8_HUMAN | Putative uncharacterized protein DKFZp686M0919 OS=Homo sapiens GN=DKFZp686M0919 PE=2 SV=1 | 60616.2 | 533 | 4.878 | 2 | 2 | 5 | 5 |
| 2089 | 3.82 | tr|A8K7G2|A8K7G2_HUMAN | cDNA FLJ75762, highly similar to Homo sapiens protease, serine, 25 (PRSS25), nuclear gene encodingmitochondrial protein, transcript variant 1, mRNA OS=Homo sapiens PE=2 SV=1 | 48797.3 | 458 | 5.677 | 2 | 2 | 4 | 4 |
| 2090 | 3.82 | tr|B7ZAQ5|B7ZAQ5_HUMAN | cDNA, FLJ79269, highly similar to DNA polymerase subunit delta 3 OS=Homo sapiens PE=2 SV=1 | 51342 | 466 | 4.721 | 2 | 2 | 3 | 3 |
| 2091 | 3.81 | tr|Q8WUV3|Q8WUV3_HUMAN | PRMT3 protein (Fragment) OS=Homo sapiens GN=PRMT3 PE=2 SV=1 | 61966.6 | 548 | 4.38 | 2 | 2 | 7 | 7 |
| 2092 | 3.8 | tr|A0A024RDH2|A0A024RDH2_HUMAN | Ubiquitin-conjugating enzyme E2D 3 (UBC4/5 homolog, yeast), isoform CRA_a OS=Homo sapiens GN=UBE2D3 PE=3 SV=1 | 16687 | 147 | 19.73 | 2 | 2 | 15 | 15 |
| 2093 | 3.8 | tr|A0A024RD36|A0A024RD36_HUMAN | Ribosomal protein L7-like 1, isoform CRA_b OS=Homo sapiens GN=RPL7L1 PE=4 SV=1 | 29669 | 255 | 8.235 | 2 | 2 | 7 | 7 |
| 2094 | 3.8 | sp|Q96DG6|CMBL_HUMAN | Carboxymethylenebutenolidase homolog OS=Homo sapiens GN=CMBL PE=1 SV=1 | 28048 | 245 | 15.51 | 4 | 4 | 8 | 8 |
| 2095 | 3.8 | tr|Q53ER0|Q53ER0_HUMAN | Glycogen synthase 1 (Muscle) variant (Fragment) OS=Homo sapiens PE=2 SV=1 | 83856.8 | 737 | 6.784 | 3 | 3 | 7 | 7 |
| 2096 | 3.79 | tr|Q49AR9|Q49AR9_HUMAN | ANKS1A protein OS=Homo sapiens GN=ANKS1A PE=2 SV=1 | 63579.4 | 570 | 4.561 | 2 | 2 | 3 | 3 |
| 2097 | 3.79 | sp|Q9UK45|LSM7_HUMAN | U6 snRNA-associated Sm-like protein LSm7 OS=Homo sapiens GN=LSM7 PE=1 SV=1 | 11602.3 | 103 | 22.33 | 3 | 3 | 4 | 4 |
| 2098 | 3.78 | tr|B3KN59|B3KN59_HUMAN | cDNA FLJ13673 fis, clone PLACE1011858, highly similar to BAG family molecular chaperone regulator 2 OS=Homo sapiens PE=2 SV=1 | 23747.7 | 211 | 18.01 | 4 | 4 | 20 | 20 |
| 2099 | 3.78 | sp|Q9H061|T126A_HUMAN | Transmembrane protein 126A OS=Homo sapiens GN=TMEM126A PE=1 SV=1 | 21527 | 195 | 16.41 | 2 | 2 | 3 | 3 |
| 2100 | 3.77 | sp|Q9Y2H5|PKHA6_HUMAN | Pleckstrin homology domain-containing family A member 6 OS=Homo sapiens GN=PLEKHA6 PE=1 SV=4 | 117127 | 1048 | 2.004 | 2 | 2 | 3 | 3 |
| 2101 | 3.77 | sp|Q9H5X1|FA96A_HUMAN | MIP18 family protein FAM96A OS=Homo sapiens GN=FAM96A PE=1 SV=1 | 18354.9 | 160 | 15.63 | 3 | 2 | 5 | 4 |
| 2102 | 3.75 | tr|B4DU42|B4DU42_HUMAN | cDNA FLJ56153, highly similar to Homo sapiens transforming growth factor beta regulator 4 (TBRG4), transcript variant 1, mRNA OS=Homo sapiens PE=2 SV=1 | 71856.6 | 642 | 3.271 | 3 | 3 | 8 | 8 |
| 2103 | 3.74 | tr|A0A087WW40|A0A087WW40_HUMAN | Endophilin-B1 OS=Homo sapiens GN=SH3GLB1 PE=1 SV=1 | 44260 | 394 | 10.41 | 4 | 3 | 6 | 4 |
| 2104 | 3.74 | tr|A0A0S2Z4Z2|A0A0S2Z4Z2_HUMAN | Ring finger protein 40 isoform 2 OS=Homo sapiens GN=RNF40 PE=2 SV=1 | 107249.9 | 956 | 5.962 | 4 | 3 | 7 | 6 |
| 2105 | 3.74 | tr|A0A024R9E4|A0A024R9E4_HUMAN | Mal, T-cell differentiation protein 2, isoform CRA_a OS=Homo sapiens GN=MAL2 PE=4 SV=1 | 19125.1 | 176 | 18.18 | 2 | 2 | 23 | 23 |
| 2106 | 3.71 | sp|Q9NQ50|RM40_HUMAN | 39S ribosomal protein L40, mitochondrial OS=Homo sapiens GN=MRPL40 PE=1 SV=1 | 24490.1 | 206 | 16.5 | 2 | 2 | 9 | 9 |
| 2107 | 3.7 | tr|A0A024R705|A0A024R705_HUMAN | Vacuolar protein sorting 4A (Yeast), isoform CRA_c OS=Homo sapiens GN=VPS4A PE=3 SV=1 | 48897.4 | 437 | 10.53 | 4 | 3 | 15 | 7 |
| 2108 | 3.7 | sp|P42696|RBM34_HUMAN | RNA-binding protein 34 OS=Homo sapiens GN=RBM34 PE=1 SV=2 | 48564.5 | 430 | 5.349 | 2 | 2 | 5 | 5 |
| 2109 | 3.7 | sp|P23258|TBG1_HUMAN | Tubulin gamma-1 chain OS=Homo sapiens GN=TUBG1 PE=1 SV=2 | 51169.5 | 451 | 5.322 | 2 | 2 | 10 | 10 |
| 2110 | 3.69 | tr|Q5H9Q6|Q5H9Q6_HUMAN | Inosine-5'-monophosphate dehydrogenase OS=Homo sapiens GN=DKFZp781N0678 PE=2 SV=1 | 64536.8 | 599 | 9.015 | 5 | 4 | 18 | 9 |
| 2111 | 3.69 | tr|A0A024R2Z6|A0A024R2Z6_HUMAN | Guanine nucleotide binding protein-like 3 (Nucleolar), isoform CRA_b OS=Homo sapiens GN=GNL3 PE=4 SV=1 | 60571.9 | 537 | 11.36 | 4 | 4 | 6 | 6 |
| 2112 | 3.69 | tr|B4DVS2|B4DVS2_HUMAN | cDNA FLJ57617 OS=Homo sapiens PE=2 SV=1 | 88888.6 | 800 | 5.25 | 3 | 3 | 3 | 3 |
| 2113 | 3.68 | tr|Q6IB63|Q6IB63_HUMAN | RABGGTB protein OS=Homo sapiens GN=RABGGTB PE=2 SV=1 | 36924 | 331 | 6.647 | 2 | 2 | 5 | 5 |
| 2114 | 3.68 | tr|A8K3Y8|A8K3Y8_HUMAN | cDNA FLJ76141 OS=Homo sapiens PE=2 SV=1 | 33751.9 | 297 | 6.734 | 2 | 2 | 3 | 3 |
| 2115 | 3.67 | tr|A8K0F7|A8K0F7_HUMAN | cDNA FLJ76587, highly similar to Homo sapiens vitamin K epoxide reductase complex, subunit 1-like 1 (VKORC1L1), mRNA OS=Homo sapiens PE=2 SV=1 | 19775.3 | 176 | 10.23 | 3 | 3 | 7 | 7 |
| 2116 | 3.65 | sp|Q9NYH9|UTP6_HUMAN | U3 small nucleolar RNA-associated protein 6 homolog OS=Homo sapiens GN=UTP6 PE=2 SV=2 | 70193.2 | 597 | 5.863 | 3 | 3 | 6 | 5 |
| 2117 | 3.65 | tr|A0A024R1D6|A0A024R1D6_HUMAN | HCG2011153, isoform CRA_b OS=Homo sapiens GN=hCG_2011153 PE=4 SV=1 | 78507.2 | 683 | 4.246 | 3 | 3 | 4 | 4 |
| 2118 | 3.65 | tr|A0A0S2Z556|A0A0S2Z556_HUMAN | Polyglutamine binding protein 1 isoform 2 (Fragment) OS=Homo sapiens GN=PQBP1 PE=2 SV=1 | 32213.8 | 281 | 9.609 | 2 | 2 | 4 | 4 |
| 2119 | 3.64 | sp|Q14692|BMS1_HUMAN | Ribosome biogenesis protein BMS1 homolog OS=Homo sapiens GN=BMS1 PE=1 SV=1 | 145806.1 | 1282 | 2.028 | 2 | 2 | 4 | 4 |
| 2120 | 3.64 | tr|Q6IAQ1|Q6IAQ1_HUMAN | LYPLA1 protein OS=Homo sapiens GN=LYPLA1 PE=1 SV=1 | 24669.4 | 230 | 13.04 | 3 | 3 | 9 | 9 |
| 2121 | 3.63 | tr|A0A024QZU0|A0A024QZU0_HUMAN | Receptor (TNFRSF)-interacting serine-threonine kinase 1, isoform CRA_a OS=Homo sapiens GN=RIPK1 PE=4 SV=1 | 75930.3 | 671 | 4.471 | 2 | 2 | 2 | 2 |
| 2122 | 3.63 | tr|E7ESZ7|E7ESZ7_HUMAN | NADH dehydrogenase [ubiquinone] 1 alpha subcomplex subunit 10, mitochondrial OS=Homo sapiens GN=NDUFA10 PE=1 SV=1 | 44737.9 | 390 | 7.949 | 2 | 2 | 2 | 2 |
| 2123 | 3.62 | tr|Q96GR7|Q96GR7_HUMAN | UBE3A protein OS=Homo sapiens GN=UBE3A PE=2 SV=1 | 67928.3 | 585 | 4.274 | 2 | 2 | 8 | 8 |
| 2124 | 3.62 | sp|Q13308|PTK7_HUMAN | Inactive tyrosine-protein kinase 7 OS=Homo sapiens GN=PTK7 PE=1 SV=2 | 118390.7 | 1070 | 7.383 | 6 | 6 | 12 | 11 |
| 2125 | 3.62 | sp|Q8N5K1|CISD2_HUMAN | CDGSH iron-sulfur domain-containing protein 2 OS=Homo sapiens GN=CISD2 PE=1 SV=1 | 15278 | 135 | 15.56 | 2 | 2 | 2 | 2 |
| 2126 | 3.61 | sp|O94874|UFL1_HUMAN | E3 UFM1-protein ligase 1 OS=Homo sapiens GN=UFL1 PE=1 SV=2 | 89594.4 | 794 | 3.275 | 3 | 3 | 8 | 8 |
| 2127 | 3.61 | tr|B4DPG9|B4DPG9_HUMAN | cDNA FLJ59630, highly similar to Growth hormone-inducible transmembrane protein OS=Homo sapiens PE=2 SV=1 | 37535.3 | 348 | 6.034 | 2 | 2 | 3 | 3 |
| 2128 | 3.61 | tr|B3KY03|B3KY03_HUMAN | Condensin complex subunit 1 OS=Homo sapiens PE=2 SV=1 | 152858.7 | 1362 | 2.203 | 2 | 2 | 5 | 5 |
| 2129 | 3.61 | tr|B2R6D8|B2R6D8_HUMAN | CDC42 effector protein (Rho GTPase binding) 4, isoform CRA_a OS=Homo sapiens GN=CDC42EP4 PE=2 SV=1 | 37979.4 | 356 | 14.04 | 3 | 3 | 3 | 3 |
| 2130 | 3.6 | tr|B4DZF8|B4DZF8_HUMAN | Serine/threonine-protein phosphatase 2A activator OS=Homo sapiens PE=2 SV=1 | 42159.6 | 374 | 6.952 | 2 | 2 | 8 | 8 |
| 2131 | 3.6 | tr|X6R8F3|X6R8F3_HUMAN | Neutrophil gelatinase-associated lipocalin OS=Homo sapiens GN=LCN2 PE=1 SV=1 | 22788.1 | 200 | 15 | 2 | 2 | 10 | 10 |
| 2132 | 3.6 | tr|Q5QPA5|Q5QPA5_HUMAN | 28S ribosomal protein S18a, mitochondrial (Fragment) OS=Homo sapiens GN=MRPS18A PE=1 SV=8 | 29714.4 | 264 | 6.439 | 2 | 2 | 3 | 3 |
| 2133 | 3.6 | tr|Q6FHT8|Q6FHT8_HUMAN | RNP24 protein OS=Homo sapiens GN=RNP24 PE=2 SV=1 | 22761.1 | 201 | 7.96 | 2 | 2 | 7 | 7 |
| 2134 | 3.59 | tr|H7BXJ4|H7BXJ4_HUMAN | F-BAR domain only protein 2 OS=Homo sapiens GN=FCHO2 PE=1 SV=1 | 43420.3 | 378 | 5.82 | 3 | 3 | 5 | 4 |
| 2135 | 3.59 | tr|Q1RLN5|Q1RLN5_HUMAN | ARHGAP12 protein OS=Homo sapiens GN=ARHGAP12 PE=1 SV=1 | 90873.3 | 799 | 3.504 | 3 | 3 | 6 | 6 |
| 2136 | 3.58 | tr|Q56VW8|Q56VW8_HUMAN | Serine/threonine-protein phosphatase 2A 55 kDa regulatory subunit B OS=Homo sapiens PE=2 SV=1 | 51647.6 | 447 | 8.725 | 3 | 3 | 10 | 10 |
| 2137 | 3.58 | sp|Q9Y237|PIN4_HUMAN | Peptidyl-prolyl cis-trans isomerase NIMA-interacting 4 OS=Homo sapiens GN=PIN4 PE=1 SV=1 | 13809.9 | 131 | 38.93 | 3 | 3 | 3 | 3 |
| 2138 | 3.57 | sp|Q8IZW8|TENS4_HUMAN | Tensin-4 OS=Homo sapiens GN=TNS4 PE=1 SV=3 | 76763 | 715 | 5.175 | 3 | 3 | 4 | 4 |
| 2139 | 3.57 | tr|A0A024RC37|A0A024RC37_HUMAN | Uncharacterized protein OS=Homo sapiens GN=P15RS PE=4 SV=1 | 35719.3 | 312 | 8.974 | 2 | 2 | 4 | 4 |
| 2140 | 3.57 | sp|Q9NY61|AATF_HUMAN | Protein AATF OS=Homo sapiens GN=AATF PE=1 SV=1 | 63132.3 | 560 | 3.929 | 2 | 2 | 2 | 2 |
| 2141 | 3.56 | tr|Q6FGM0|Q6FGM0_HUMAN | SH3 domain GRB2-like 1 OS=Homo sapiens GN=SH3GL1 PE=2 SV=1 | 41489.6 | 368 | 5.435 | 2 | 2 | 7 | 7 |
| 2142 | 3.55 | sp|P19525|E2AK2_HUMAN | Interferon-induced, double-stranded RNA-activated protein kinase OS=Homo sapiens GN=EIF2AK2 PE=1 SV=2 | 62093.7 | 551 | 9.256 | 3 | 3 | 3 | 3 |
| 2143 | 3.54 | tr|A0A0C4DGI9|A0A0C4DGI9_HUMAN | Bromodomain adjacent to zinc finger domain protein 2A (Fragment) OS=Homo sapiens GN=BAZ2A PE=1 SV=1 | 94569.4 | 837 | 2.987 | 2 | 2 | 2 | 2 |
| 2144 | 3.52 | tr|Q56G89|Q56G89_HUMAN | Serum albumin OS=Homo sapiens PE=2 SV=1 | 69083.6 | 609 | 5.419 | 3 | 3 | 16 | 16 |
| 2145 | 3.51 | tr|A0A024R861|A0A024R861_HUMAN | Serine/threonine-protein phosphatase OS=Homo sapiens GN=PPP6C PE=2 SV=1 | 35143.9 | 305 | 7.213 | 2 | 2 | 2 | 2 |
| 2146 | 3.51 | tr|B4DL49|B4DL49_HUMAN | cDNA FLJ58073, moderately similar to Cathepsin B (EC 3.4.22.1) OS=Homo sapiens PE=2 SV=1 | 30767.5 | 273 | 12.82 | 3 | 3 | 4 | 4 |
| 2147 | 3.5 | tr|E7ETZ4|E7ETZ4_HUMAN | Basic leucine zipper and W2 domain-containing protein 2 (Fragment) OS=Homo sapiens GN=BZW2 PE=1 SV=1 | 46912.9 | 408 | 12.01 | 4 | 4 | 7 | 7 |
| 2148 | 3.49 | sp|Q5C9Z4|NOM1_HUMAN | Nucleolar MIF4G domain-containing protein 1 OS=Homo sapiens GN=NOM1 PE=1 SV=1 | 96256.4 | 860 | 3.256 | 3 | 3 | 6 | 2 |
| 2149 | 3.49 | tr|B3KSS4|B3KSS4_HUMAN | cDNA FLJ36858 fis, clone ASTRO2015185, highly similar to POLIOVIRUS RECEPTOR OS=Homo sapiens PE=2 SV=1 | 39579.5 | 362 | 7.735 | 3 | 3 | 12 | 12 |
| 2150 | 3.48 | sp|P51553|IDH3G_HUMAN | Isocitrate dehydrogenase [NAD] subunit gamma, mitochondrial OS=Homo sapiens GN=IDH3G PE=1 SV=1 | 42794 | 393 | 6.87 | 2 | 2 | 6 | 6 |
| 2151 | 3.47 | tr|A0A096LPJ3|A0A096LPJ3_HUMAN | COP9 signalosome complex subunit 1 OS=Homo sapiens GN=GPS1 PE=1 SV=1 | 55407.8 | 490 | 7.143 | 2 | 2 | 5 | 5 |
| 2152 | 3.47 | tr|A8K2I7|A8K2I7_HUMAN | cDNA FLJ76072, highly similar to Homo sapiens GIPC PDZ domain containing family, member 1 (GIPC1), transcript variant 1, mRNA OS=Homo sapiens PE=2 SV=1 | 36038.9 | 333 | 8.709 | 3 | 3 | 9 | 9 |
| 2153 | 3.47 | sp|Q96A33|CCD47_HUMAN | Coiled-coil domain-containing protein 47 OS=Homo sapiens GN=CCDC47 PE=1 SV=1 | 55873.5 | 483 | 5.797 | 3 | 3 | 4 | 4 |
| 2154 | 3.46 | sp|Q8NF37|PCAT1_HUMAN | Lysophosphatidylcholine acyltransferase 1 OS=Homo sapiens GN=LPCAT1 PE=1 SV=2 | 59150.7 | 534 | 7.116 | 3 | 3 | 4 | 4 |
| 2155 | 3.45 | tr|F8W8I8|F8W8I8_HUMAN | Septin-8 OS=Homo sapiens GN=SEPT8 PE=1 SV=1 | 53162.8 | 460 | 14.13 | 6 | 3 | 18 | 3 |
| 2156 | 3.44 | tr|B2R6S9|B2R6S9_HUMAN | cDNA, FLJ93097, highly similar to Homo sapiens low density lipoprotein receptor-related protein associated protein 1 (LRPAP1), mRNA OS=Homo sapiens PE=2 SV=1 | 41479.7 | 357 | 10.36 | 4 | 3 | 5 | 2 |
| 2157 | 3.44 | tr|Q8IXG0|Q8IXG0_HUMAN | Crn protein OS=Homo sapiens GN=crn PE=2 SV=1 | 83138.8 | 687 | 5.822 | 3 | 3 | 3 | 3 |
| 2158 | 3.44 | sp|O14732|IMPA2_HUMAN | Inositol monophosphatase 2 OS=Homo sapiens GN=IMPA2 PE=1 SV=1 | 31320.5 | 288 | 7.639 | 2 | 2 | 2 | 2 |
| 2159 | 3.41 | tr|E9PB90|E9PB90_HUMAN | Hexokinase-2 OS=Homo sapiens GN=HK2 PE=1 SV=1 | 98972.2 | 889 | 5.849 | 5 | 3 | 8 | 5 |
| 2160 | 3.41 | tr|B7Z600|B7Z600_HUMAN | cDNA FLJ53180, highly similar to Cullin-3 OS=Homo sapiens PE=2 SV=1 | 63309.4 | 550 | 4 | 2 | 2 | 6 | 6 |
| 2161 | 3.41 | tr|B3KMB9|B3KMB9_HUMAN | cDNA FLJ10659 fis, clone NT2RP2006071, highly similar to DCC-interacting protein 13 beta OS=Homo sapiens PE=2 SV=1 | 74432.7 | 664 | 6.476 | 3 | 3 | 3 | 3 |
| 2162 | 3.41 | sp|Q96HE7|ERO1A_HUMAN | ERO1-like protein alpha OS=Homo sapiens GN=ERO1A PE=1 SV=2 | 54392.1 | 468 | 4.487 | 2 | 2 | 22 | 22 |
| 2163 | 3.41 | tr|I3L3T0|I3L3T0_HUMAN | HCG15164, isoform CRA_b OS=Homo sapiens GN=PAM16 PE=4 SV=1 | 15942 | 145 | 23.45 | 2 | 2 | 3 | 3 |
| 2164 | 3.4 | sp|Q9BYG3|MK67I_HUMAN | MKI67 FHA domain-interacting nucleolar phosphoprotein OS=Homo sapiens GN=NIFK PE=1 SV=1 | 34222 | 293 | 12.97 | 4 | 4 | 5 | 5 |
| 2165 | 3.39 | sp|Q6RFH5|WDR74_HUMAN | WD repeat-containing protein 74 OS=Homo sapiens GN=WDR74 PE=1 SV=1 | 42440.9 | 385 | 7.013 | 2 | 2 | 3 | 3 |
| 2166 | 3.38 | sp|Q53FA7|QORX_HUMAN | Quinone oxidoreductase PIG3 OS=Homo sapiens GN=TP53I3 PE=1 SV=2 | 35535.9 | 332 | 6.627 | 2 | 2 | 3 | 3 |
| 2167 | 3.36 | tr|B3KM89|B3KM89_HUMAN | cDNA FLJ10528 fis, clone NT2RP2000943, highly similar to Protein transport protein Sec24D OS=Homo sapiens PE=2 SV=1 | 100247.7 | 906 | 2.87 | 3 | 2 | 3 | 2 |
| 2168 | 3.36 | tr|B2RDE0|B2RDE0_HUMAN | cDNA, FLJ96567, highly similar to Homo sapiens propionyl Coenzyme A carboxylase, alpha polypeptide(PCCA), mRNA OS=Homo sapiens PE=2 SV=1 | 77413.2 | 703 | 2.845 | 2 | 2 | 3 | 3 |
| 2169 | 3.36 | tr|B2R745|B2R745_HUMAN | cDNA, FLJ93277, highly similar to Homo sapiens solute carrier family 30 (zinc transporter), member 9(SLC30A9), mRNA OS=Homo sapiens PE=2 SV=1 | 63482.5 | 568 | 5.986 | 3 | 3 | 3 | 3 |
| 2170 | 3.36 | sp|Q9Y3B2|EXOS1_HUMAN | Exosome complex component CSL4 OS=Homo sapiens GN=EXOSC1 PE=1 SV=1 | 21451.6 | 195 | 15.38 | 3 | 3 | 3 | 2 |
| 2171 | 3.35 | tr|E7ET15|E7ET15_HUMAN | U2 snRNP-associated SURP motif-containing protein OS=Homo sapiens GN=U2SURP PE=1 SV=1 | 118246.8 | 1028 | 2.918 | 3 | 3 | 11 | 8 |
| 2172 | 3.35 | tr|B3KN67|B3KN67_HUMAN | cDNA FLJ13779 fis, clone PLACE4000445, highly similar to Homo sapiens GTPase activating protein and VPS9 domains 1 (GAPVD1), mRNA OS=Homo sapiens PE=2 SV=1 | 91530.2 | 823 | 3.402 | 2 | 2 | 6 | 6 |
| 2173 | 3.35 | tr|A0A024RD78|A0A024RD78_HUMAN | Mitochondrial ribosomal protein L14, isoform CRA_b OS=Homo sapiens GN=MRPL14 PE=3 SV=1 | 15947.6 | 145 | 19.31 | 2 | 2 | 7 | 7 |
| 2174 | 3.35 | sp|Q9Y639|NPTN_HUMAN | Neuroplastin OS=Homo sapiens GN=NPTN PE=1 SV=2 | 44386.8 | 398 | 5.528 | 2 | 2 | 5 | 5 |
| 2175 | 3.34 | sp|Q9P206|K1522_HUMAN | Uncharacterized protein KIAA1522 OS=Homo sapiens GN=KIAA1522 PE=1 SV=2 | 107093.8 | 1035 | 3.865 | 2 | 2 | 3 | 3 |
| 2176 | 3.33 | tr|A0A087X256|A0A087X256_HUMAN | WASH complex subunit 7 OS=Homo sapiens GN=KIAA1033 PE=1 SV=1 | 136501.3 | 1174 | 3.748 | 4 | 4 | 6 | 6 |
| 2177 | 3.33 | tr|A8K9T9|A8K9T9_HUMAN | cDNA FLJ75059, highly similar to Homo sapiens phosphoribosylformylglycinamidine synthase (FGAR amidotransferase) (PFAS), mRNA OS=Homo sapiens PE=2 SV=1 | 144642.1 | 1338 | 3.438 | 3 | 3 | 4 | 4 |
| 2178 | 3.33 | sp|O76027|ANXA9_HUMAN | Annexin A9 OS=Homo sapiens GN=ANXA9 PE=1 SV=3 | 38363.2 | 345 | 11.59 | 3 | 3 | 4 | 4 |
| 2179 | 3.33 | tr|E7EV01|E7EV01_HUMAN | Calpain-5 OS=Homo sapiens GN=CAPN5 PE=1 SV=2 | 77469.1 | 680 | 2.941 | 2 | 2 | 8 | 8 |
| 2180 | 3.32 | tr|V9HWC5|V9HWC5_HUMAN | Epididymis secretory protein Li 36 OS=Homo sapiens GN=HEL-S-36 PE=2 SV=1 | 31806.2 | 270 | 12.59 | 3 | 2 | 7 | 4 |
| 2181 | 3.32 | tr|B2R6N9|B2R6N9_HUMAN | cDNA, FLJ93042, highly similar to Homo sapiens signal sequence receptor, alpha (translocon-associated protein alpha) (SSR1), mRNA OS=Homo sapiens PE=2 SV=1 | 32192.9 | 286 | 19.58 | 5 | 3 | 24 | 5 |
| 2182 | 3.32 | sp|Q86VN1|VPS36_HUMAN | Vacuolar protein-sorting-associated protein 36 OS=Homo sapiens GN=VPS36 PE=1 SV=1 | 43816.4 | 386 | 4.404 | 2 | 2 | 4 | 4 |
| 2183 | 3.32 | tr|A0A087WUE9|A0A087WUE9_HUMAN | Symplekin OS=Homo sapiens GN=SYMPK PE=1 SV=1 | 117437.1 | 1058 | 2.363 | 2 | 2 | 5 | 5 |
| 2184 | 3.32 | tr|Q53GV6|Q53GV6_HUMAN | PRP3 pre-mRNA processing factor 3 homolog (Fragment) OS=Homo sapiens PE=2 SV=1 | 77440.3 | 683 | 5.124 | 3 | 3 | 4 | 4 |
| 2185 | 3.31 | tr|A4D0Z3|A4D0Z3_HUMAN | ADP-ribosylation factor 5 OS=Homo sapiens GN=ARF5 PE=2 SV=1 | 20529.5 | 180 | 43.33 | 6 | 3 | 82 | 15 |
| 2186 | 3.31 | tr|A0A024R9K7|A0A024R9K7_HUMAN | Chromosome 15 open reading frame 24, isoform CRA_b OS=Homo sapiens GN=C15orf24 PE=4 SV=1 | 35416.5 | 320 | 10.94 | 3 | 2 | 7 | 5 |
| 2187 | 3.31 | sp|Q86VM9|ZCH18_HUMAN | Zinc finger CCCH domain-containing protein 18 OS=Homo sapiens GN=ZC3H18 PE=1 SV=2 | 106377.4 | 953 | 3.043 | 2 | 2 | 2 | 2 |
| 2188 | 3.29 | sp|Q71RC2|LARP4_HUMAN | La-related protein 4 OS=Homo sapiens GN=LARP4 PE=1 SV=3 | 80595.4 | 724 | 4.834 | 3 | 3 | 7 | 7 |
| 2189 | 3.29 | tr|M0R2A8|M0R2A8_HUMAN | Dipeptidyl peptidase 9 OS=Homo sapiens GN=DPP9 PE=1 SV=1 | 59069.1 | 518 | 6.371 | 2 | 2 | 3 | 3 |
| 2190 | 3.29 | tr|B4E1J7|B4E1J7_HUMAN | cDNA FLJ56935, highly similar to Centromere/kinetochore protein zw10 homolog OS=Homo sapiens PE=2 SV=1 | 70188.3 | 616 | 5.682 | 3 | 3 | 5 | 3 |
| 2191 | 3.29 | sp|P62306|RUXF_HUMAN | Small nuclear ribonucleoprotein F OS=Homo sapiens GN=SNRPF PE=1 SV=1 | 9725.2 | 86 | 24.42 | 2 | 2 | 16 | 16 |
| 2192 | 3.28 | tr|A0A140VJD7|A0A140VJD7_HUMAN | Acylphosphatase OS=Homo sapiens PE=2 SV=1 | 11139.5 | 99 | 17.17 | 2 | 2 | 6 | 6 |
| 2193 | 3.28 | tr|A8K520|A8K520_HUMAN | cDNA FLJ78340, highly similar to Homo sapiens proline synthetase co-transcribed homolog (PROSC), mRNA OS=Homo sapiens PE=2 SV=1 | 30285.7 | 275 | 6.182 | 2 | 2 | 5 | 5 |
| 2194 | 3.28 | sp|P55789|ALR_HUMAN | FAD-linked sulfhydryl oxidase ALR OS=Homo sapiens GN=GFER PE=1 SV=2 | 23448.9 | 205 | 18.05 | 2 | 2 | 4 | 4 |
| 2195 | 3.27 | tr|A0A0B4J1S4|A0A0B4J1S4_HUMAN | 15 kDa selenoprotein OS=Homo sapiens GN=SEP15 PE=1 SV=1 | 18044.7 | 165 | 13.94 | 2 | 2 | 5 | 5 |
| 2196 | 3.26 | tr|A0A0D9SEI3|A0A0D9SEI3_HUMAN | Cyclin-dependent kinase 11B OS=Homo sapiens GN=CDK11B PE=1 SV=1 | 89437 | 772 | 3.627 | 3 | 3 | 7 | 7 |
| 2197 | 3.26 | sp|Q15018|F175B_HUMAN | BRISC complex subunit Abro1 OS=Homo sapiens GN=FAM175B PE=1 SV=2 | 46900.4 | 415 | 7.47 | 2 | 2 | 2 | 2 |
| 2198 | 3.26 | tr|E9PPW7|E9PPW7_HUMAN | NADH dehydrogenase [ubiquinone] iron-sulfur protein 8, mitochondrial (Fragment) OS=Homo sapiens GN=NDUFS8 PE=1 SV=1 | 20656.4 | 184 | 13.59 | 2 | 2 | 3 | 3 |
| 2199 | 3.25 | sp|Q16763|UBE2S_HUMAN | Ubiquitin-conjugating enzyme E2 S OS=Homo sapiens GN=UBE2S PE=1 SV=2 | 23845.1 | 222 | 11.71 | 2 | 2 | 8 | 8 |
| 2200 | 3.24 | tr|B2RCI6|B2RCI6_HUMAN | cDNA, FLJ96094, highly similar to Homo sapiens numb homolog (Drosophila) (NUMB), mRNA OS=Homo sapiens PE=2 SV=1 | 69358.6 | 640 | 4.062 | 2 | 2 | 2 | 2 |
| 2201 | 3.23 | sp|Q9BUP3|HTAI2_HUMAN | Oxidoreductase HTATIP2 OS=Homo sapiens GN=HTATIP2 PE=1 SV=2 | 27048.8 | 242 | 12.81 | 4 | 3 | 8 | 4 |
| 2202 | 3.21 | tr|B3KTL6|B3KTL6_HUMAN | cDNA FLJ38470 fis, clone FEBRA2022013, highly similar to CCR4-NOT transcription complex subunit 2 (Fragment) OS=Homo sapiens PE=2 SV=1 | 59754.3 | 540 | 4.63 | 2 | 2 | 4 | 4 |
| 2203 | 3.2 | sp|O14524|NEMP1_HUMAN | Nuclear envelope integral membrane protein 1 OS=Homo sapiens GN=NEMP1 PE=1 SV=2 | 50639.6 | 444 | 4.73 | 3 | 2 | 4 | 2 |
| 2204 | 3.2 | tr|A8K607|A8K607_HUMAN | cDNA FLJ76855, highly similar to Homo sapiens exportin 7 (XPO7), mRNA OS=Homo sapiens PE=2 SV=1 | 123925.1 | 1087 | 2.852 | 2 | 2 | 2 | 2 |
| 2205 | 3.2 | tr|Q495G5|Q495G5_HUMAN | MMAA protein OS=Homo sapiens GN=MMAA PE=1 SV=1 | 47135.6 | 424 | 6.132 | 2 | 2 | 2 | 2 |
| 2206 | 3.2 | tr|A0A087X1G1|A0A087X1G1_HUMAN | NADH dehydrogenase [ubiquinone] 1 alpha subcomplex subunit 5 OS=Homo sapiens GN=NDUFA5 PE=1 SV=1 | 11964.9 | 101 | 34.65 | 2 | 2 | 4 | 4 |
| 2207 | 3.19 | tr|G5E9V5|G5E9V5_HUMAN | 28S ribosomal protein S22, mitochondrial OS=Homo sapiens GN=MRPS22 PE=1 SV=1 | 41209 | 359 | 8.357 | 3 | 3 | 3 | 2 |
| 2208 | 3.18 | tr|B2R4A5|B2R4A5_HUMAN | cDNA, FLJ92019, highly similar to Homo sapiens mitochondrial ribosomal protein S14 (MRPS14), nuclear gene encoding mitochondrial protein, mRNA OS=Homo sapiens PE=2 SV=1 | 15157.6 | 128 | 18.75 | 2 | 2 | 7 | 7 |
| 2209 | 3.18 | tr|A0A024R0Q4|A0A024R0Q4_HUMAN | Phospholipase D family, member 3, isoform CRA_b OS=Homo sapiens GN=PLD3 PE=4 SV=1 | 54704.9 | 490 | 7.755 | 2 | 2 | 7 | 7 |
| 2210 | 3.17 | sp|O75323|NIPS2_HUMAN | Protein NipSnap homolog 2 OS=Homo sapiens GN=GBAS PE=1 SV=1 | 33742.4 | 286 | 6.993 | 3 | 2 | 8 | 4 |
| 2211 | 3.17 | sp|O95169|NDUB8_HUMAN | NADH dehydrogenase [ubiquinone] 1 beta subcomplex subunit 8, mitochondrial OS=Homo sapiens GN=NDUFB8 PE=1 SV=1 | 21765.7 | 186 | 10.75 | 2 | 2 | 6 | 6 |
| 2212 | 3.16 | sp|O00178|GTPB1_HUMAN | GTP-binding protein 1 OS=Homo sapiens GN=GTPBP1 PE=1 SV=3 | 72453.3 | 669 | 3.886 | 2 | 2 | 5 | 5 |
| 2213 | 3.16 | sp|Q5SY16|NOL9_HUMAN | Polynucleotide 5'-hydroxyl-kinase NOL9 OS=Homo sapiens GN=NOL9 PE=1 SV=1 | 79321.9 | 702 | 4.416 | 2 | 2 | 6 | 6 |
| 2214 | 3.15 | sp|Q9H2W6|RM46_HUMAN | 39S ribosomal protein L46, mitochondrial OS=Homo sapiens GN=MRPL46 PE=1 SV=1 | 31704.8 | 279 | 11.47 | 3 | 2 | 8 | 7 |
| 2215 | 3.15 | tr|Q6PCC8|Q6PCC8_HUMAN | URB2 protein (Fragment) OS=Homo sapiens GN=URB2 PE=2 SV=1 | 107315.1 | 957 | 4.075 | 4 | 4 | 4 | 3 |
| 2216 | 3.15 | sp|Q96DV4|RM38_HUMAN | 39S ribosomal protein L38, mitochondrial OS=Homo sapiens GN=MRPL38 PE=1 SV=2 | 44596.1 | 380 | 5.263 | 2 | 2 | 4 | 4 |
| 2217 | 3.13 | tr|B7Z6Q5|B7Z6Q5_HUMAN | Beta-galactosidase OS=Homo sapiens PE=2 SV=1 | 81802.7 | 725 | 6.069 | 4 | 3 | 7 | 5 |
| 2218 | 3.13 | tr|Q6FHI2|Q6FHI2_HUMAN | PTE1 protein OS=Homo sapiens GN=PTE1 PE=2 SV=1 | 35957 | 319 | 10.03 | 2 | 2 | 6 | 6 |
| 2219 | 3.12 | tr|A8K813|A8K813_HUMAN | cDNA FLJ77763, highly similar to Homo sapiens prolactin regulatory element binding (PREB), mRNA OS=Homo sapiens PE=2 SV=1 | 45481.9 | 417 | 7.194 | 2 | 2 | 3 | 3 |
| 2220 | 3.12 | sp|Q9NX24|NHP2_HUMAN | H/ACA ribonucleoprotein complex subunit 2 OS=Homo sapiens GN=NHP2 PE=1 SV=1 | 17200.9 | 153 | 18.95 | 2 | 2 | 23 | 23 |
| 2221 | 3.11 | sp|Q9BWJ5|SF3B5_HUMAN | Splicing factor 3B subunit 5 OS=Homo sapiens GN=SF3B5 PE=1 SV=1 | 10135.3 | 86 | 36.05 | 2 | 2 | 9 | 9 |
| 2222 | 3.1 | sp|P19784|CSK22_HUMAN | Casein kinase II subunit alpha' OS=Homo sapiens GN=CSNK2A2 PE=1 SV=1 | 41212.9 | 350 | 10.86 | 4 | 2 | 4 | 2 |
| 2223 | 3.1 | sp|Q06587|RING1_HUMAN | E3 ubiquitin-protein ligase RING1 OS=Homo sapiens GN=RING1 PE=1 SV=2 | 42429 | 406 | 5.419 | 2 | 2 | 2 | 2 |
| 2224 | 3.1 | sp|Q8NI27|THOC2_HUMAN | THO complex subunit 2 OS=Homo sapiens GN=THOC2 PE=1 SV=2 | 182773.1 | 1593 | 2.699 | 4 | 4 | 5 | 5 |
| 2225 | 3.1 | tr|B2R7R5|B2R7R5_HUMAN | cDNA, FLJ93570, highly similar to Homo sapiens phosphoribosyl pyrophosphate synthetase-associated protein 2 (PRPSAP2), mRNA OS=Homo sapiens PE=2 SV=1 | 40883.2 | 369 | 11.92 | 3 | 3 | 3 | 3 |
| 2226 | 3.09 | tr|B4DYF9|B4DYF9_HUMAN | cDNA FLJ57669, highly similar to SWI/SNF-related matrix-associatedactin-dependent regulator of chromatin subfamily C member 1 OS=Homo sapiens PE=2 SV=1 | 67011.1 | 612 | 9.477 | 5 | 3 | 5 | 3 |
| 2227 | 3.09 | tr|Q59FM5|Q59FM5_HUMAN | Guanine nucleotide-binding protein G, alpha subunit variant (Fragment) OS=Homo sapiens PE=2 SV=1 | 41890.6 | 357 | 5.602 | 2 | 2 | 2 | 2 |
| 2228 | 3.09 | tr|A0A024R2F9|A0A024R2F9_HUMAN | Transmembrane protein 43 isoform 1 OS=Homo sapiens GN=TMEM43 PE=2 SV=1 | 44875.1 | 400 | 11 | 3 | 3 | 9 | 9 |
| 2229 | 3.08 | sp|Q7KZ85|SPT6H_HUMAN | Transcription elongation factor SPT6 OS=Homo sapiens GN=SUPT6H PE=1 SV=2 | 199071.1 | 1726 | 1.217 | 2 | 2 | 6 | 6 |
| 2230 | 3.08 | tr|I3L2L5|I3L2L5_HUMAN | Mapk-regulated corepressor-interacting protein 1 OS=Homo sapiens GN=MCRIP1 PE=1 SV=1 | 10462.7 | 92 | 48.91 | 3 | 3 | 4 | 4 |
| 2231 | 3.07 | sp|Q9H488|OFUT1_HUMAN | GDP-fucose protein O-fucosyltransferase 1 OS=Homo sapiens GN=POFUT1 PE=1 SV=1 | 43955.4 | 388 | 5.412 | 2 | 2 | 2 | 2 |
| 2232 | 3.07 | sp|Q8WV92|MITD1_HUMAN | MIT domain-containing protein 1 OS=Homo sapiens GN=MITD1 PE=1 SV=1 | 29314.2 | 249 | 11.65 | 2 | 2 | 7 | 7 |
| 2233 | 3.06 | tr|Q59EC9|Q59EC9_HUMAN | Glyceronephosphate O-acyltransferase variant (Fragment) OS=Homo sapiens PE=2 SV=1 | 78593.7 | 693 | 4.04 | 3 | 3 | 4 | 3 |
| 2234 | 3.06 | tr|B4DLT9|B4DLT9_HUMAN | cDNA FLJ56462, highly similar to SAPS domain family member 1 OS=Homo sapiens PE=2 SV=1 | 43148 | 396 | 13.38 | 2 | 2 | 2 | 2 |
| 2235 | 3.06 | tr|B7Z5S1|B7Z5S1_HUMAN | cDNA FLJ61699, highly similar to Homo sapiens androgen-induced proliferation inhibitor (APRIN), transcript variant 1, mRNA OS=Homo sapiens PE=2 SV=1 | 148738.5 | 1302 | 2.611 | 2 | 2 | 4 | 4 |
| 2236 | 3.05 | tr|E5KMT5|E5KMT5_HUMAN | tRNA pseudouridine synthase OS=Homo sapiens PE=3 SV=1 | 47469.7 | 427 | 8.197 | 3 | 3 | 5 | 5 |
| 2237 | 3.05 | tr|Q59FC4|Q59FC4_HUMAN | Presynaptic protein SAP97 variant (Fragment) OS=Homo sapiens PE=4 SV=1 | 75960 | 687 | 3.348 | 2 | 2 | 5 | 5 |
| 2238 | 3.05 | tr|B2RAY1|B2RAY1_HUMAN | cDNA, FLJ95184, highly similar to Homo sapiens signal transducing adaptor molecule (SH3 domain and ITAM motif) 1 (STAM), mRNA OS=Homo sapiens PE=2 SV=1 | 59149.1 | 540 | 4.815 | 2 | 2 | 2 | 2 |
| 2239 | 3.04 | sp|Q9NSI2|F207A_HUMAN | Protein FAM207A OS=Homo sapiens GN=FAM207A PE=1 SV=2 | 25456 | 230 | 9.13 | 2 | 2 | 4 | 4 |
| 2240 | 3.04 | tr|B3KNS8|B3KNS8_HUMAN | cDNA FLJ30322 fis, clone BRACE2006703, highly similar to Surfeit locus protein 6 OS=Homo sapiens PE=2 SV=1 | 41508.1 | 361 | 7.479 | 3 | 3 | 3 | 2 |
| 2241 | 3.04 | tr|B3KM78|B3KM78_HUMAN | cDNA FLJ10442 fis, clone NT2RP1000738, highly similar to Negative elongation factor A OS=Homo sapiens PE=2 SV=1 | 58527.7 | 539 | 5.566 | 2 | 2 | 4 | 4 |
| 2242 | 3.04 | tr|H7C5N8|H7C5N8_HUMAN | Protein PRRC2C (Fragment) OS=Homo sapiens GN=PRRC2C PE=1 SV=1 | 135918.3 | 1300 | 2.692 | 2 | 2 | 2 | 2 |
| 2243 | 3.03 | sp|Q13257|MD2L1_HUMAN | Mitotic spindle assembly checkpoint protein MAD2A OS=Homo sapiens GN=MAD2L1 PE=1 SV=1 | 23509.7 | 205 | 14.63 | 3 | 3 | 8 | 7 |
| 2244 | 3.02 | sp|Q9Y277|VDAC3_HUMAN | Voltage-dependent anion-selective channel protein 3 OS=Homo sapiens GN=VDAC3 PE=1 SV=1 | 30658.5 | 283 | 8.834 | 3 | 2 | 15 | 3 |
| 2245 | 3.02 | sp|Q9H974|QTRD1_HUMAN | Queuine tRNA-ribosyltransferase subunit QTRTD1 OS=Homo sapiens GN=QTRTD1 PE=1 SV=1 | 46712.4 | 415 | 8.434 | 2 | 2 | 6 | 6 |
| 2246 | 3.02 | sp|Q9BUH6|PAXX_HUMAN | Protein PAXX OS=Homo sapiens GN=C9orf142 PE=1 SV=2 | 21639.2 | 204 | 11.76 | 2 | 2 | 3 | 3 |
| 2247 | 3.02 | sp|Q08722|CD47_HUMAN | Leukocyte surface antigen CD47 OS=Homo sapiens GN=CD47 PE=1 SV=1 | 35213.3 | 323 | 6.192 | 2 | 2 | 5 | 5 |
| 2248 | 3.01 | tr|Q53RX3|Q53RX3_HUMAN | Putative uncharacterized protein RDH14 OS=Homo sapiens GN=RDH14 PE=2 SV=1 | 36864.4 | 336 | 4.762 | 2 | 2 | 4 | 3 |
| 2249 | 3.01 | tr|B8ZZY2|B8ZZY2_HUMAN | Arf-GAP domain and FG repeat-containing protein 1 OS=Homo sapiens GN=AGFG1 PE=1 SV=1 | 56412.7 | 541 | 6.839 | 2 | 2 | 5 | 5 |
| 2250 | 3.01 | tr|Q59GX7|Q59GX7_HUMAN | Stearoyl-CoA desaturase variant (Fragment) OS=Homo sapiens PE=2 SV=1 | 42337.2 | 366 | 10.38 | 2 | 2 | 2 | 2 |
| 2251 | 3.01 | sp|O14949|QCR8_HUMAN | Cytochrome b-c1 complex subunit 8 OS=Homo sapiens GN=UQCRQ PE=1 SV=4 | 9906.3 | 82 | 25.61 | 2 | 2 | 17 | 17 |
| 2252 | 3 | tr|V9HWA6|V9HWA6_HUMAN | Epididymis luminal protein 32 OS=Homo sapiens GN=HEL32 PE=2 SV=1 | 18505.5 | 165 | 27.88 | 5 | 3 | 56 | 42 |
| 2253 | 3 | tr|A0A024R3D4|A0A024R3D4_HUMAN | Uncharacterized protein OS=Homo sapiens GN=DKFZp547C195 PE=4 SV=1 | 77666.7 | 703 | 2.845 | 2 | 2 | 2 | 2 |
| 2254 | 3 | sp|Q9BW92|SYTM_HUMAN | Threonine--tRNA ligase, mitochondrial OS=Homo sapiens GN=TARS2 PE=1 SV=1 | 81035.3 | 718 | 4.318 | 2 | 2 | 5 | 5 |
| 2255 | 2.99 | tr|E7ETU7|E7ETU7_HUMAN | 39S ribosomal protein L3, mitochondrial OS=Homo sapiens GN=MRPL3 PE=1 SV=1 | 41627.9 | 375 | 7.2 | 2 | 2 | 2 | 2 |
| 2256 | 2.99 | sp|O15382|BCAT2_HUMAN | Branched-chain-amino-acid aminotransferase, mitochondrial OS=Homo sapiens GN=BCAT2 PE=1 SV=2 | 44287.4 | 392 | 7.908 | 3 | 3 | 8 | 8 |
| 2257 | 2.99 | tr|A0A024R120|A0A024R120_HUMAN | Transcription factor CP2, isoform CRA_a OS=Homo sapiens GN=TFCP2 PE=4 SV=1 | 57255.3 | 502 | 7.171 | 2 | 2 | 2 | 2 |
| 2258 | 2.98 | tr|A0A024R324|A0A024R324_HUMAN | Ras homolog gene family, member A, isoform CRA_a OS=Homo sapiens GN=RHOA PE=4 SV=1 | 21767.9 | 193 | 35.75 | 6 | 2 | 33 | 12 |
| 2259 | 2.98 | tr|V9HWG0|V9HWG0_HUMAN | Chromobox homolog 5 (HP1 alpha homolog, Drosophila), isoform CRA_b OS=Homo sapiens GN=HEL25 PE=2 SV=1 | 22224.8 | 191 | 12.57 | 2 | 2 | 4 | 3 |
| 2260 | 2.98 | sp|Q9Y2S0|RPAC2_HUMAN | DNA-directed RNA polymerases I and III subunit RPAC2 OS=Homo sapiens GN=POLR1D PE=1 SV=1 | 15237.1 | 133 | 15.79 | 2 | 2 | 7 | 7 |
| 2261 | 2.98 | tr|A0A024R184|A0A024R184_HUMAN | Asparagine-linked glycosylation 2 homolog (Yeast, alpha-1,3-mannosyltransferase), isoform CRA_a OS=Homo sapiens GN=ALG2 PE=4 SV=1 | 47091.1 | 416 | 8.173 | 2 | 2 | 7 | 7 |
| 2262 | 2.98 | tr|B7Z592|B7Z592_HUMAN | cDNA FLJ61635, highly similar to Homo sapiens likely ortholog of mouse immediate early response, erythropoietin 4 (LEREPO4), mRNA OS=Homo sapiens PE=2 SV=1 | 44890.5 | 392 | 5.357 | 2 | 2 | 16 | 16 |
| 2263 | 2.97 | sp|Q5F1R6|DJC21_HUMAN | DnaJ homolog subfamily C member 21 OS=Homo sapiens GN=DNAJC21 PE=1 SV=2 | 62027.5 | 531 | 5.838 | 2 | 2 | 2 | 2 |
| 2264 | 2.96 | tr|A0A024RDE5|A0A024RDE5_HUMAN | Ras-GTPase activating protein SH3 domain-binding protein 2, isoform CRA_a OS=Homo sapiens GN=G3BP2 PE=4 SV=1 | 54120.9 | 482 | 10.37 | 5 | 4 | 9 | 5 |
| 2265 | 2.96 | sp|Q9GZN8|CT027_HUMAN | UPF0687 protein C20orf27 OS=Homo sapiens GN=C20orf27 PE=1 SV=3 | 19290.9 | 174 | 13.79 | 2 | 2 | 4 | 4 |
| 2266 | 2.96 | tr|B4DG73|B4DG73_HUMAN | cDNA FLJ56431, highly similar to Conserved oligomeric Golgi complex component 6 OS=Homo sapiens PE=2 SV=1 | 76798.4 | 690 | 2.899 | 2 | 2 | 2 | 2 |
| 2267 | 2.96 | sp|Q9Y6X8|ZHX2_HUMAN | Zinc fingers and homeoboxes protein 2 OS=Homo sapiens GN=ZHX2 PE=1 SV=1 | 92306.3 | 837 | 3.106 | 2 | 2 | 2 | 2 |
| 2268 | 2.96 | sp|Q00688|FKBP3_HUMAN | Peptidyl-prolyl cis-trans isomerase FKBP3 OS=Homo sapiens GN=FKBP3 PE=1 SV=1 | 25176.7 | 224 | 16.96 | 4 | 4 | 12 | 12 |
| 2269 | 2.95 | sp|Q9Y3B4|SF3B6_HUMAN | Splicing factor 3B subunit 6 OS=Homo sapiens GN=SF3B6 PE=1 SV=1 | 14584.8 | 125 | 17.6 | 2 | 2 | 3 | 3 |
| 2270 | 2.95 | tr|B5MCF9|B5MCF9_HUMAN | Pescadillo homolog OS=Homo sapiens GN=PES1 PE=1 SV=1 | 66076.8 | 571 | 4.378 | 3 | 3 | 4 | 4 |
| 2271 | 2.95 | sp|Q15334|L2GL1_HUMAN | Lethal(2) giant larvae protein homolog 1 OS=Homo sapiens GN=LLGL1 PE=1 SV=3 | 115416.9 | 1064 | 3.665 | 4 | 4 | 12 | 4 |
| 2272 | 2.94 | sp|O95782|AP2A1_HUMAN | AP-2 complex subunit alpha-1 OS=Homo sapiens GN=AP2A1 PE=1 SV=3 | 107544.7 | 977 | 4.299 | 4 | 2 | 9 | 6 |
| 2273 | 2.94 | sp|Q9UHA4|LTOR3_HUMAN | Ragulator complex protein LAMTOR3 OS=Homo sapiens GN=LAMTOR3 PE=1 SV=1 | 13622.6 | 124 | 41.13 | 2 | 2 | 7 | 7 |
| 2274 | 2.93 | tr|A0A0S2Z3W7|A0A0S2Z3W7_HUMAN | Nucleoside-triphosphate diphosphatase (Fragment) OS=Homo sapiens GN=ITPA PE=2 SV=1 | 21445.5 | 194 | 17.01 | 3 | 3 | 6 | 6 |
| 2275 | 2.92 | sp|Q9BSJ2|GCP2_HUMAN | Gamma-tubulin complex component 2 OS=Homo sapiens GN=TUBGCP2 PE=1 SV=2 | 102533 | 902 | 3.437 | 3 | 2 | 5 | 2 |
| 2276 | 2.91 | tr|G8JLD3|G8JLD3_HUMAN | ELKS/Rab6-interacting/CAST family member 1 OS=Homo sapiens GN=ERC1 PE=1 SV=1 | 124819.1 | 1086 | 3.315 | 4 | 3 | 7 | 4 |
| 2277 | 2.91 | tr|B4DXW2|B4DXW2_HUMAN | cDNA FLJ60947, highly similar to Coiled-coil domain-containing protein 9 OS=Homo sapiens PE=2 SV=1 | 58012.7 | 513 | 5.068 | 3 | 3 | 4 | 4 |
| 2278 | 2.9 | sp|Q96K76|UBP47_HUMAN | Ubiquitin carboxyl-terminal hydrolase 47 OS=Homo sapiens GN=USP47 PE=1 SV=3 | 157309.9 | 1375 | 1.964 | 2 | 2 | 4 | 4 |
| 2279 | 2.89 | tr|Q53HS1|Q53HS1_HUMAN | Achalasia, adrenocortical insufficiency, alacrimia (Allgrove, triple-A) variant (Fragment) OS=Homo sapiens PE=2 SV=1 | 59583.6 | 546 | 4.945 | 2 | 2 | 3 | 3 |
| 2280 | 2.89 | sp|Q7L5A8|FA2H_HUMAN | Fatty acid 2-hydroxylase OS=Homo sapiens GN=FA2H PE=1 SV=1 | 42791 | 372 | 8.602 | 2 | 2 | 3 | 3 |
| 2281 | 2.89 | tr|H0YNG3|H0YNG3_HUMAN | Signal peptidase complex catalytic subunit SEC11 OS=Homo sapiens GN=SEC11A PE=1 SV=1 | 18650.7 | 163 | 7.362 | 2 | 2 | 5 | 5 |
| 2282 | 2.88 | tr|A0A024R3U8|A0A024R3U8_HUMAN | Uncharacterized protein OS=Homo sapiens GN=FLJ22555 PE=4 SV=1 | 32544.3 | 291 | 11.34 | 3 | 3 | 6 | 6 |
| 2283 | 2.88 | sp|Q92917|GPKOW_HUMAN | G patch domain and KOW motifs-containing protein OS=Homo sapiens GN=GPKOW PE=1 SV=2 | 52228.4 | 476 | 5.882 | 2 | 2 | 5 | 5 |
| 2284 | 2.88 | sp|Q9Y5V0|ZN706_HUMAN | Zinc finger protein 706 OS=Homo sapiens GN=ZNF706 PE=1 SV=1 | 8497.8 | 76 | 30.26 | 2 | 2 | 8 | 8 |
| 2285 | 2.88 | sp|Q9NUL5|RYDEN_HUMAN | Repressor of yield of DENV protein OS=Homo sapiens GN=RYDEN PE=1 SV=2 | 33109.9 | 291 | 6.873 | 2 | 2 | 6 | 6 |
| 2286 | 2.87 | tr|B4DGM5|B4DGM5_HUMAN | cDNA FLJ53983, highly similar to IWS1 homolog OS=Homo sapiens PE=2 SV=1 | 82121 | 729 | 3.018 | 3 | 2 | 5 | 4 |
| 2287 | 2.86 | tr|A0A024QZR5|A0A024QZR5_HUMAN | Adaptor-related protein complex 3, mu 1 subunit, isoform CRA_a OS=Homo sapiens GN=AP3M1 PE=3 SV=1 | 46938.8 | 418 | 10.53 | 3 | 3 | 3 | 3 |
| 2288 | 2.86 | tr|E7EQY1|E7EQY1_HUMAN | Protein FAM136A OS=Homo sapiens GN=FAM136A PE=1 SV=1 | 26775.6 | 245 | 7.347 | 2 | 2 | 11 | 11 |
| 2289 | 2.86 | sp|Q9NW64|RBM22_HUMAN | Pre-mRNA-splicing factor RBM22 OS=Homo sapiens GN=RBM22 PE=1 SV=1 | 46895.2 | 420 | 6.905 | 3 | 3 | 6 | 5 |
| 2290 | 2.86 | tr|A0A140VKA9|A0A140VKA9_HUMAN | Testis secretory sperm-binding protein Li 236P OS=Homo sapiens PE=2 SV=1 | 25789.3 | 244 | 17.21 | 3 | 3 | 6 | 6 |
| 2291 | 2.86 | tr|A0A024R8S3|A0A024R8S3_HUMAN | Small ubiquitin-related modifier OS=Homo sapiens GN=SUMO2 PE=3 SV=1 | 10871.2 | 95 | 40 | 4 | 4 | 42 | 42 |
| 2292 | 2.85 | tr|A0A140VJQ4|A0A140VJQ4_HUMAN | Testicular tissue protein Li 130 OS=Homo sapiens PE=2 SV=1 | 48534.4 | 439 | 8.2 | 3 | 2 | 7 | 6 |
| 2293 | 2.85 | tr|A0A024RCX8|A0A024RCX8_HUMAN | Peptidyl-prolyl cis-trans isomerase OS=Homo sapiens GN=PPIL1 PE=3 SV=1 | 18236.7 | 166 | 15.66 | 3 | 3 | 14 | 2 |
| 2294 | 2.85 | tr|Q53XL7|Q53XL7_HUMAN | Glycine cleavage system protein H (Aminomethyl carrier) OS=Homo sapiens GN=GCSH PE=2 SV=1 | 18910.4 | 173 | 17.34 | 2 | 2 | 5 | 5 |
| 2295 | 2.85 | tr|A0A024R6Y2|A0A024R6Y2_HUMAN | Nuclear transport factor 2, isoform CRA_a OS=Homo sapiens GN=NUTF2 PE=4 SV=1 | 14478.4 | 127 | 16.54 | 2 | 2 | 10 | 10 |
| 2296 | 2.85 | tr|A0A024QYT6|A0A024QYT6_HUMAN | Adaptor-related protein complex 1, sigma 1 subunit, isoform CRA_b OS=Homo sapiens GN=AP1S1 PE=4 SV=1 | 18732.8 | 158 | 15.82 | 2 | 2 | 15 | 15 |
| 2297 | 2.84 | tr|E5RJR5|E5RJR5_HUMAN | S-phase kinase-associated protein 1 OS=Homo sapiens GN=SKP1 PE=1 SV=1 | 18719.9 | 163 | 35.58 | 4 | 3 | 7 | 5 |
| 2298 | 2.84 | tr|B8ZZW5|B8ZZW5_HUMAN | Late secretory pathway protein AVL9 homolog OS=Homo sapiens GN=AVL9 PE=1 SV=1 | 69800.5 | 630 | 5.397 | 3 | 3 | 4 | 4 |
| 2299 | 2.83 | sp|Q9NQH7|XPP3_HUMAN | Probable Xaa-Pro aminopeptidase 3 OS=Homo sapiens GN=XPNPEP3 PE=1 SV=1 | 57033.1 | 507 | 6.706 | 3 | 3 | 30 | 4 |
| 2300 | 2.83 | tr|A0A024RDJ4|A0A024RDJ4_HUMAN | Nuclear factor of kappa light polypeptide gene enhancer in B-cells 1 (P105), isoform CRA_b OS=Homo sapiens GN=NFKB1 PE=4 SV=1 | 105355.2 | 968 | 4.855 | 3 | 3 | 4 | 4 |
| 2301 | 2.82 | tr|B3KM97|B3KM97_HUMAN | cDNA FLJ10554 fis, clone NT2RP2002385, highly similar to Synaptic glycoprotein SC2 OS=Homo sapiens PE=2 SV=1 | 36053.1 | 308 | 6.494 | 2 | 2 | 5 | 5 |
| 2302 | 2.81 | tr|A0A140VK65|A0A140VK65_HUMAN | Testicular secretory protein Li 65 OS=Homo sapiens PE=2 SV=1 | 56500.2 | 511 | 15.07 | 5 | 2 | 31 | 5 |
| 2303 | 2.81 | tr|A0A024R577|A0A024R577_HUMAN | Transmembrane 7 superfamily member 2, isoform CRA_a OS=Homo sapiens GN=TM7SF2 PE=4 SV=1 | 46405.3 | 418 | 5.981 | 3 | 2 | 9 | 4 |
| 2304 | 2.8 | tr|A8K9T8|A8K9T8_HUMAN | cDNA FLJ76106, highly similar to Homo sapiens neurolysin (metallopeptidase M3 family) (NLN), mRNA OS=Homo sapiens PE=2 SV=1 | 80677.1 | 704 | 4.972 | 3 | 3 | 7 | 3 |
| 2305 | 2.8 | tr|B3KN28|B3KN28_HUMAN | cDNA FLJ13370 fis, clone PLACE1000653, highly similar to Phosphoacetylglucosamine mutase (EC 5.4.2.3) OS=Homo sapiens PE=2 SV=1 | 59865.6 | 542 | 3.137 | 2 | 2 | 3 | 3 |
| 2306 | 2.8 | tr|E9PL57|E9PL57_HUMAN | Protein NEDD8-MDP1 (Fragment) OS=Homo sapiens GN=NEDD8-MDP1 PE=4 SV=1 | 19536.3 | 170 | 14.71 | 3 | 3 | 5 | 3 |
| 2307 | 2.79 | sp|O15020|SPTN2_HUMAN | Spectrin beta chain, non-erythrocytic 2 OS=Homo sapiens GN=SPTBN2 PE=1 SV=3 | 271322.5 | 2390 | 2.72 | 6 | 5 | 17 | 2 |
| 2308 | 2.79 | sp|Q5T6V5|CI064_HUMAN | UPF0553 protein C9orf64 OS=Homo sapiens GN=C9orf64 PE=1 SV=1 | 39028.1 | 341 | 6.158 | 3 | 2 | 3 | 2 |
| 2309 | 2.79 | sp|O95801|TTC4_HUMAN | Tetratricopeptide repeat protein 4 OS=Homo sapiens GN=TTC4 PE=1 SV=3 | 44678.1 | 387 | 5.685 | 2 | 2 | 2 | 2 |
| 2310 | 2.78 | sp|Q99496|RING2_HUMAN | E3 ubiquitin-protein ligase RING2 OS=Homo sapiens GN=RNF2 PE=1 SV=1 | 37655 | 336 | 7.44 | 2 | 2 | 2 | 2 |
| 2311 | 2.78 | tr|A0A140VK08|A0A140VK08_HUMAN | Testicular secretory protein Li 8 OS=Homo sapiens PE=2 SV=1 | 31539.6 | 271 | 7.749 | 2 | 2 | 11 | 11 |
| 2312 | 2.78 | tr|B4DQK3|B4DQK3_HUMAN | cDNA FLJ61156, highly similar to Bcl-2-binding component 3 OS=Homo sapiens PE=2 SV=1 | 21046.7 | 199 | 9.045 | 2 | 2 | 2 | 2 |
| 2313 | 2.78 | tr|Q6IRX3|Q6IRX3_HUMAN | RNA binding motif protein 7 OS=Homo sapiens GN=RBM7 PE=2 SV=1 | 30531.3 | 266 | 9.023 | 2 | 2 | 5 | 5 |
| 2314 | 2.77 | sp|Q9Y6U3|ADSV_HUMAN | Adseverin OS=Homo sapiens GN=SCIN PE=1 SV=4 | 80488.3 | 715 | 4.196 | 2 | 2 | 2 | 1 |
| 2315 | 2.77 | tr|A0A024R4R3|A0A024R4R3_HUMAN | CCR4-NOT transcription complex, subunit 3, isoform CRA_a OS=Homo sapiens GN=CNOT3 PE=4 SV=1 | 81871.3 | 753 | 3.586 | 2 | 2 | 2 | 2 |
| 2316 | 2.76 | sp|O95470|SGPL1_HUMAN | Sphingosine-1-phosphate lyase 1 OS=Homo sapiens GN=SGPL1 PE=1 SV=3 | 63523.3 | 568 | 7.042 | 3 | 3 | 10 | 10 |
| 2317 | 2.76 | tr|A0A024R084|A0A024R084_HUMAN | Stromal cell derived factor 4, isoform CRA_c OS=Homo sapiens GN=SDF4 PE=4 SV=1 | 41806.5 | 362 | 3.315 | 1 | 1 | 10 | 10 |
| 2318 | 2.75 | tr|A0A024R5S4|A0A024R5S4_HUMAN | Ubiquitin specific peptidase 8, isoform CRA_a OS=Homo sapiens GN=USP8 PE=3 SV=1 | 127522.2 | 1118 | 2.504 | 2 | 2 | 2 | 2 |
| 2319 | 2.75 | sp|Q9UJC3|HOOK1_HUMAN | Protein Hook homolog 1 OS=Homo sapiens GN=HOOK1 PE=1 SV=2 | 84647.2 | 728 | 3.022 | 2 | 2 | 5 | 5 |
| 2320 | 2.75 | tr|A0A087WT20|A0A087WT20_HUMAN | DDB1- and CUL4-associated factor 13 OS=Homo sapiens GN=DCAF13 PE=1 SV=1 | 67550.6 | 597 | 3.015 | 2 | 2 | 6 | 6 |
| 2321 | 2.75 | sp|Q96A57|TM230_HUMAN | Transmembrane protein 230 OS=Homo sapiens GN=TMEM230 PE=1 SV=1 | 13188.2 | 120 | 19.17 | 2 | 2 | 3 | 3 |
| 2322 | 2.74 | sp|O15357|SHIP2_HUMAN | Phosphatidylinositol 3,4,5-trisphosphate 5-phosphatase 2 OS=Homo sapiens GN=INPPL1 PE=1 SV=2 | 138597.5 | 1258 | 2.464 | 3 | 2 | 3 | 2 |
| 2323 | 2.74 | sp|Q9NXN4|GDAP2_HUMAN | Ganglioside-induced differentiation-associated protein 2 OS=Homo sapiens GN=GDAP2 PE=1 SV=1 | 56224.2 | 497 | 7.847 | 3 | 2 | 4 | 3 |
| 2324 | 2.74 | tr|A0A140VK00|A0A140VK00_HUMAN | Testicular tissue protein Li 227 OS=Homo sapiens PE=2 SV=1 | 34258.5 | 298 | 7.383 | 2 | 2 | 2 | 2 |
| 2325 | 2.74 | tr|Q5BKZ2|Q5BKZ2_HUMAN | Importin subunit alpha OS=Homo sapiens GN=KPNA1 PE=2 SV=1 | 60308.3 | 538 | 3.532 | 2 | 2 | 6 | 6 |
| 2326 | 2.74 | tr|B4E1C9|B4E1C9_HUMAN | cDNA FLJ52963, highly similar to Synapse-associated protein 1 OS=Homo sapiens PE=2 SV=1 | 36758 | 318 | 15.72 | 2 | 2 | 2 | 2 |
| 2327 | 2.74 | tr|Q6NW12|Q6NW12_HUMAN | TANK protein OS=Homo sapiens GN=TANK PE=2 SV=1 | 47843.3 | 425 | 11.29 | 3 | 3 | 5 | 5 |
| 2328 | 2.72 | tr|Q53Y51|Q53Y51_HUMAN | D-dopachrome tautomerase OS=Homo sapiens GN=DDT PE=2 SV=1 | 12711.6 | 118 | 17.8 | 2 | 2 | 2 | 2 |
| 2329 | 2.72 | tr|A0MNP2|A0MNP2_HUMAN | CDW11/WDR57 OS=Homo sapiens GN=WDR57 PE=2 SV=1 | 39310.2 | 357 | 5.322 | 2 | 2 | 3 | 3 |
| 2330 | 2.71 | sp|Q5T440|CAF17_HUMAN | Putative transferase CAF17, mitochondrial OS=Homo sapiens GN=IBA57 PE=1 SV=1 | 38154.6 | 356 | 8.427 | 2 | 2 | 2 | 2 |
| 2331 | 2.71 | tr|D3DQF6|D3DQF6_HUMAN | POU domain, class 4, transcription factor 3, isoform CRA_a OS=Homo sapiens GN=POU4F3 PE=4 SV=1 | 84105.7 | 771 | 3.113 | 2 | 2 | 2 | 2 |
| 2332 | 2.71 | tr|E9PMR4|E9PMR4_HUMAN | Tetraspanin OS=Homo sapiens GN=CD151 PE=1 SV=1 | 28066.6 | 251 | 5.976 | 2 | 2 | 4 | 4 |
| 2333 | 2.7 | sp|O94851|MICA2_HUMAN | Protein-methionine sulfoxide oxidase MICAL2 OS=Homo sapiens GN=MICAL2 PE=1 SV=1 | 126688 | 1124 | 2.224 | 2 | 2 | 2 | 2 |
| 2334 | 2.7 | sp|Q712K3|UB2R2_HUMAN | Ubiquitin-conjugating enzyme E2 R2 OS=Homo sapiens GN=UBE2R2 PE=1 SV=1 | 27165.7 | 238 | 8.824 | 2 | 2 | 7 | 7 |
| 2335 | 2.69 | tr|B7Z5W1|B7Z5W1_HUMAN | cDNA FLJ54854, highly similar to Junctional adhesion molecule A OS=Homo sapiens PE=2 SV=1 | 32858 | 303 | 16.17 | 3 | 3 | 6 | 6 |
| 2336 | 2.69 | tr|A0A0S2Z5X1|A0A0S2Z5X1_HUMAN | DnaJ-like protein subfamily C member 19 isoform 1 (Fragment) OS=Homo sapiens GN=DNAJC19 PE=2 SV=1 | 12498.5 | 116 | 12.07 | 1 | 1 | 5 | 5 |
| 2337 | 2.67 | tr|B7Z6G2|B7Z6G2_HUMAN | cDNA FLJ56152, highly similar to Rho guanine nucleotide exchange factor 7 OS=Homo sapiens PE=2 SV=1 | 81403.9 | 721 | 4.993 | 3 | 3 | 3 | 3 |
| 2338 | 2.67 | tr|A0A087X0H9|A0A087X0H9_HUMAN | RNA-binding protein 26 OS=Homo sapiens GN=RBM26 PE=1 SV=1 | 113793.6 | 1009 | 2.775 | 3 | 3 | 4 | 4 |
| 2339 | 2.67 | tr|A0MNN5|A0MNN5_HUMAN | CDW4/GRWD1 OS=Homo sapiens GN=GRWD1 PE=2 SV=1 | 49418.8 | 446 | 6.726 | 2 | 2 | 3 | 3 |
| 2340 | 2.67 | tr|Q7LD69|Q7LD69_HUMAN | NADH-ubiquinone oxidoreductase Fe-S protein 7 variant OS=Homo sapiens PE=2 SV=1 | 23563.3 | 213 | 20.19 | 5 | 5 | 9 | 9 |
| 2341 | 2.66 | sp|Q7L2H7|EIF3M_HUMAN | Eukaryotic translation initiation factor 3 subunit M OS=Homo sapiens GN=EIF3M PE=1 SV=1 | 42502.5 | 374 | 12.83 | 3 | 3 | 3 | 3 |
| 2342 | 2.65 | tr|A0A0S2Z3H6|A0A0S2Z3H6_HUMAN | Cleft lip and palate associated transmembrane protein 1 isoform 1 (Fragment) OS=Homo sapiens GN=CLPTM1 PE=2 SV=1 | 77958.2 | 686 | 3.207 | 2 | 2 | 4 | 4 |
| 2343 | 2.65 | sp|Q9NPF4|OSGEP_HUMAN | Probable tRNA N6-adenosine threonylcarbamoyltransferase OS=Homo sapiens GN=OSGEP PE=1 SV=1 | 36426.6 | 335 | 9.851 | 2 | 2 | 5 | 5 |
| 2344 | 2.65 | tr|C9J7T7|C9J7T7_HUMAN | Nuclear receptor corepressor 2 OS=Homo sapiens GN=NCOR2 PE=1 SV=3 | 220999 | 2062 | 1.649 | 3 | 3 | 4 | 3 |
| 2345 | 2.64 | tr|Q6FHY4|Q6FHY4_HUMAN | N-ethylmaleimide-sensitive factor attachment protein, gamma, isoform CRA_b OS=Homo sapiens GN=NAPG PE=2 SV=1 | 34746 | 312 | 6.731 | 2 | 2 | 2 | 2 |
| 2346 | 2.62 | tr|A8KAM9|A8KAM9_HUMAN | Peptidyl-prolyl cis-trans isomerase E OS=Homo sapiens PE=2 SV=1 | 33416.5 | 301 | 10.3 | 4 | 3 | 18 | 12 |
| 2347 | 2.62 | tr|B3KUY5|B3KUY5_HUMAN | cDNA FLJ40905 fis, clone UTERU2004664, highly similar to Conserved oligomeric Golgi complex component 1 OS=Homo sapiens PE=2 SV=1 | 99283 | 894 | 3.02 | 2 | 2 | 8 | 8 |
| 2348 | 2.62 | tr|Q8NG23|Q8NG23_HUMAN | GTP binding protein OS=Homo sapiens PE=2 SV=1 | 22336.7 | 198 | 7.071 | 2 | 2 | 8 | 8 |
| 2349 | 2.62 | tr|A0A024QZ78|A0A024QZ78_HUMAN | Agmatine ureohydrolase (Agmatinase), isoform CRA_a OS=Homo sapiens GN=AGMAT PE=3 SV=1 | 37660.1 | 352 | 13.35 | 3 | 3 | 3 | 3 |
| 2350 | 2.62 | tr|C9J8T6|C9J8T6_HUMAN | Cytochrome c oxidase copper chaperone OS=Homo sapiens GN=COX17 PE=1 SV=1 | 10853.3 | 98 | 22.45 | 2 | 2 | 3 | 2 |
| 2351 | 2.61 | tr|A8K894|A8K894_HUMAN | cDNA FLJ77927 OS=Homo sapiens PE=2 SV=1 | 59666.4 | 537 | 7.076 | 3 | 2 | 9 | 5 |
| 2352 | 2.61 | sp|Q96S19|CP013_HUMAN | UPF0585 protein C16orf13 OS=Homo sapiens GN=C16orf13 PE=1 SV=2 | 22577.9 | 204 | 14.22 | 2 | 2 | 7 | 7 |
| 2353 | 2.61 | tr|A0A024R6H1|A0A024R6H1_HUMAN | Serine palmitoyltransferase, long chain base subunit 2, isoform CRA_a OS=Homo sapiens GN=SPTLC2 PE=3 SV=1 | 62923.8 | 562 | 6.584 | 2 | 2 | 6 | 6 |
| 2354 | 2.6 | tr|D6RCD0|D6RCD0_HUMAN | Estradiol 17-beta-dehydrogenase 11 OS=Homo sapiens GN=HSD17B11 PE=1 SV=2 | 28102.1 | 256 | 10.94 | 3 | 2 | 3 | 2 |
| 2355 | 2.6 | tr|M0QXT0|M0QXT0_HUMAN | Upstream stimulatory factor 2 (Fragment) OS=Homo sapiens GN=USF2 PE=1 SV=2 | 28148.3 | 253 | 10.28 | 2 | 2 | 2 | 2 |
| 2356 | 2.6 | sp|Q6ULP2|AFTIN_HUMAN | Aftiphilin OS=Homo sapiens GN=AFTPH PE=1 SV=2 | 102199 | 937 | 2.775 | 2 | 2 | 2 | 2 |
| 2357 | 2.6 | sp|P78310|CXAR_HUMAN | Coxsackievirus and adenovirus receptor OS=Homo sapiens GN=CXADR PE=1 SV=1 | 40029.5 | 365 | 6.575 | 2 | 2 | 2 | 1 |
| 2358 | 2.59 | sp|Q9UHY7|ENOPH_HUMAN | Enolase-phosphatase E1 OS=Homo sapiens GN=ENOPH1 PE=1 SV=1 | 28932.4 | 261 | 11.11 | 2 | 2 | 3 | 3 |
| 2359 | 2.59 | tr|A4D1A1|A4D1A1_HUMAN | Proline rich 15 OS=Homo sapiens GN=LOC222171 PE=2 SV=1 | 13715 | 129 | 17.83 | 2 | 2 | 6 | 6 |
| 2360 | 2.58 | sp|Q9HC52|CBX8_HUMAN | Chromobox protein homolog 8 OS=Homo sapiens GN=CBX8 PE=1 SV=3 | 43395.3 | 389 | 4.884 | 2 | 2 | 2 | 2 |
| 2361 | 2.57 | tr|B4E0J9|B4E0J9_HUMAN | cDNA FLJ57348, highly similar to Homo sapiens hexokinase domain containing 1 (HKDC1), mRNA OS=Homo sapiens PE=2 SV=1 | 86697.7 | 780 | 1.282 | 1 | 1 | 2 | 2 |
| 2362 | 2.57 | sp|Q9H3H3|CK068_HUMAN | UPF0696 protein C11orf68 OS=Homo sapiens GN=C11orf68 PE=1 SV=2 | 27354.5 | 251 | 9.562 | 2 | 2 | 3 | 3 |
| 2363 | 2.56 | tr|B4DSQ5|B4DSQ5_HUMAN | cDNA FLJ53608, highly similar to Protein transport protein Sec23A OS=Homo sapiens PE=2 SV=1 | 82927.4 | 736 | 5.299 | 3 | 1 | 20 | 4 |
| 2364 | 2.56 | sp|Q969U7|PSMG2_HUMAN | Proteasome assembly chaperone 2 OS=Homo sapiens GN=PSMG2 PE=1 SV=1 | 29395.8 | 264 | 6.818 | 2 | 2 | 10 | 10 |
| 2365 | 2.56 | sp|O94880|PHF14_HUMAN | PHD finger protein 14 OS=Homo sapiens GN=PHF14 PE=1 SV=2 | 100052.4 | 888 | 2.477 | 2 | 2 | 6 | 6 |
| 2366 | 2.55 | tr|A0A024RDL9|A0A024RDL9_HUMAN | Phosphoserine phosphatase, isoform CRA_b OS=Homo sapiens GN=PSPH PE=4 SV=1 | 28082.1 | 252 | 17.86 | 5 | 5 | 12 | 12 |
| 2367 | 2.55 | tr|Q53GE7|Q53GE7_HUMAN | Tetratricopeptide repeat domain 1 variant (Fragment) OS=Homo sapiens PE=2 SV=1 | 33594.9 | 292 | 3.425 | 1 | 1 | 5 | 5 |
| 2368 | 2.55 | tr|E9PR30|E9PR30_HUMAN | 40S ribosomal protein S30 OS=Homo sapiens GN=FAU PE=1 SV=1 | 10904.8 | 98 | 11.22 | 2 | 2 | 33 | 33 |
| 2369 | 2.54 | sp|Q9HA65|TBC17_HUMAN | TBC1 domain family member 17 OS=Homo sapiens GN=TBC1D17 PE=1 SV=2 | 72727.5 | 648 | 4.784 | 3 | 2 | 6 | 5 |
| 2370 | 2.54 | sp|Q86VR2|F134C_HUMAN | Protein FAM134C OS=Homo sapiens GN=FAM134C PE=1 SV=1 | 51395.6 | 466 | 10.94 | 3 | 3 | 4 | 4 |
| 2371 | 2.54 | sp|Q9Y388|RBMX2_HUMAN | RNA-binding motif protein, X-linked 2 OS=Homo sapiens GN=RBMX2 PE=1 SV=2 | 37335.3 | 322 | 7.764 | 2 | 2 | 3 | 3 |
| 2372 | 2.53 | sp|P08708|RS17_HUMAN | 40S ribosomal protein S17 OS=Homo sapiens GN=RPS17 PE=1 SV=2 | 15550 | 135 | 24.44 | 2 | 2 | 9 | 9 |
| 2373 | 2.51 | tr|B4E302|B4E302_HUMAN | cDNA FLJ56209, highly similar to Transforming acidic coiled-coil-containing protein 1 OS=Homo sapiens PE=2 SV=1 | 86163 | 792 | 5.051 | 3 | 3 | 3 | 3 |
| 2374 | 2.51 | tr|Q9H836|Q9H836_HUMAN | cDNA FLJ13963 fis, clone Y79AA1001299, highly similar to Homo sapiens integrase interactor 1b protein (INI1B) OS=Homo sapiens PE=2 SV=1 | 45050.8 | 394 | 3.299 | 1 | 1 | 5 | 5 |
| 2375 | 2.51 | tr|A4FUT8|A4FUT8_HUMAN | JMJD1B protein (Fragment) OS=Homo sapiens GN=JMJD1B PE=2 SV=1 | 169053.6 | 1551 | 1.805 | 2 | 2 | 2 | 2 |
| 2376 | 2.5 | tr|J3KR35|J3KR35_HUMAN | Coiled-coil domain containing 12, isoform CRA_a OS=Homo sapiens GN=CCDC12 PE=1 SV=1 | 20501 | 179 | 9.497 | 1 | 1 | 5 | 5 |
| 2377 | 2.5 | sp|Q9UIJ7|KAD3_HUMAN | GTP:AMP phosphotransferase AK3, mitochondrial OS=Homo sapiens GN=AK3 PE=1 SV=4 | 25565.2 | 227 | 9.251 | 2 | 2 | 3 | 3 |
| 2378 | 2.49 | tr|Q9HAM0|Q9HAM0_HUMAN | Protein YIPF OS=Homo sapiens PE=2 SV=1 | 30590.1 | 280 | 13.93 | 2 | 2 | 2 | 2 |
| 2379 | 2.49 | sp|P62875|RPAB5_HUMAN | DNA-directed RNA polymerases I, II, and III subunit RPABC5 OS=Homo sapiens GN=POLR2L PE=1 SV=1 | 7645 | 67 | 13.43 | 1 | 1 | 6 | 6 |
| 2380 | 2.48 | sp|Q8WY22|BRI3B_HUMAN | BRI3-binding protein OS=Homo sapiens GN=BRI3BP PE=1 SV=1 | 27835.2 | 251 | 14.74 | 5 | 2 | 23 | 3 |
| 2381 | 2.48 | sp|Q6PGP7|TTC37_HUMAN | Tetratricopeptide repeat protein 37 OS=Homo sapiens GN=TTC37 PE=1 SV=1 | 175484.6 | 1564 | 1.215 | 2 | 2 | 2 | 2 |
| 2382 | 2.47 | tr|F8W9X7|F8W9X7_HUMAN | Coiled-coil domain-containing protein 93 OS=Homo sapiens GN=CCDC93 PE=1 SV=1 | 73040.8 | 630 | 3.651 | 2 | 2 | 2 | 2 |
| 2383 | 2.46 | tr|B2R7G6|B2R7G6_HUMAN | cDNA, FLJ93437, highly similar to Homo sapiens histidyl-tRNA synthetase-like (HARSL), mRNA OS=Homo sapiens PE=2 SV=1 | 56888.8 | 506 | 12.06 | 6 | 2 | 19 | 6 |
| 2384 | 2.46 | sp|O43660|PLRG1_HUMAN | Pleiotropic regulator 1 OS=Homo sapiens GN=PLRG1 PE=1 SV=1 | 57193.5 | 514 | 5.253 | 2 | 2 | 17 | 17 |
| 2385 | 2.46 | tr|B7Z1T7|B7Z1T7_HUMAN | cDNA FLJ61691, highly similar to Homo sapiens myosin head domain containing 1 (MYOHD1), transcript variant 2, mRNA OS=Homo sapiens PE=2 SV=1 | 47884.3 | 428 | 4.907 | 2 | 2 | 3 | 1 |
| 2386 | 2.46 | tr|V9HW45|V9HW45_HUMAN | Epididymis secretory protein Li 302 OS=Homo sapiens GN=HEL-S-302 PE=2 SV=1 | 29987.8 | 261 | 17.62 | 2 | 2 | 6 | 6 |
| 2387 | 2.46 | tr|B4DW81|B4DW81_HUMAN | cDNA FLJ58863, highly similar to Protein NipSnap3A OS=Homo sapiens PE=2 SV=1 | 26180.7 | 227 | 8.811 | 2 | 2 | 4 | 4 |
| 2388 | 2.45 | sp|Q01650|LAT1_HUMAN | Large neutral amino acids transporter small subunit 1 OS=Homo sapiens GN=SLC7A5 PE=1 SV=2 | 55009.6 | 507 | 6.312 | 2 | 2 | 11 | 11 |
| 2389 | 2.45 | sp|Q9BUR5|MIC26_HUMAN | MICOS complex subunit MIC26 OS=Homo sapiens GN=APOO PE=1 SV=1 | 22284.5 | 198 | 8.586 | 2 | 2 | 5 | 5 |
| 2390 | 2.44 | tr|B4DIE3|B4DIE3_HUMAN | Protein disulfide-isomerase TMX3 OS=Homo sapiens GN=TMX3 PE=1 SV=1 | 22670.1 | 197 | 10.15 | 2 | 1 | 6 | 1 |
| 2391 | 2.44 | tr|B8QGS9|B8QGS9_HUMAN | Plakophilin-2 OS=Homo sapiens GN=PKP2 PE=1 SV=1 | 91239 | 824 | 2.549 | 2 | 2 | 6 | 6 |
| 2392 | 2.44 | tr|B4DST5|B4DST5_HUMAN | cDNA FLJ58078, highly similar to Tyrosine-protein phosphatase non-receptortype 23 (EC 3.1.3.48) OS=Homo sapiens PE=2 SV=1 | 164565.4 | 1510 | 2.781 | 2 | 2 | 3 | 3 |
| 2393 | 2.44 | tr|A8K9K8|A8K9K8_HUMAN | rRNA adenine N(6)-methyltransferase OS=Homo sapiens PE=2 SV=1 | 35237 | 313 | 7.987 | 2 | 2 | 4 | 4 |
| 2394 | 2.44 | tr|A8K1R1|A8K1R1_HUMAN | Receptor expression-enhancing protein OS=Homo sapiens PE=2 SV=1 | 29291.8 | 255 | 7.843 | 2 | 2 | 3 | 3 |
| 2395 | 2.44 | sp|Q96L92|SNX27_HUMAN | Sorting nexin-27 OS=Homo sapiens GN=SNX27 PE=1 SV=2 | 61264.9 | 541 | 4.067 | 2 | 2 | 3 | 2 |
| 2396 | 2.44 | tr|A0A024R5H0|A0A024R5H0_HUMAN | Barrier to autointegration factor 1, isoform CRA_a OS=Homo sapiens GN=BANF1 PE=4 SV=1 | 10058.5 | 89 | 7.865 | 1 | 1 | 5 | 5 |
| 2397 | 2.44 | sp|Q6QNY0|BL1S3_HUMAN | Biogenesis of lysosome-related organelles complex 1 subunit 3 OS=Homo sapiens GN=BLOC1S3 PE=1 SV=1 | 21255.3 | 202 | 9.406 | 1 | 1 | 2 | 2 |
| 2398 | 2.43 | sp|Q00403|TF2B_HUMAN | Transcription initiation factor IIB OS=Homo sapiens GN=GTF2B PE=1 SV=1 | 34832.5 | 316 | 4.43 | 1 | 1 | 1 | 1 |
| 2399 | 2.42 | tr|Q53Y03|Q53Y03_HUMAN | COX4 neighbor, isoform CRA_a OS=Homo sapiens GN=COX4NB PE=1 SV=1 | 23772.8 | 210 | 12.38 | 2 | 2 | 7 | 7 |
| 2400 | 2.42 | sp|P55210|CASP7_HUMAN | Caspase-7 OS=Homo sapiens GN=CASP7 PE=1 SV=1 | 34276.5 | 303 | 10.89 | 2 | 2 | 5 | 5 |
| 2401 | 2.42 | sp|P16930|FAAA_HUMAN | Fumarylacetoacetase OS=Homo sapiens GN=FAH PE=1 SV=2 | 46374 | 419 | 4.057 | 2 | 2 | 6 | 6 |
| 2402 | 2.42 | sp|P13688|CEAM1_HUMAN | Carcinoembryonic antigen-related cell adhesion molecule 1 OS=Homo sapiens GN=CEACAM1 PE=1 SV=2 | 57560 | 526 | 7.224 | 2 | 2 | 5 | 5 |
| 2403 | 2.41 | sp|Q6IBS0|TWF2_HUMAN | Twinfilin-2 OS=Homo sapiens GN=TWF2 PE=1 SV=2 | 39547.7 | 349 | 12.32 | 3 | 2 | 4 | 3 |
| 2404 | 2.41 | tr|B4DS95|B4DS95_HUMAN | Ribonucleoside-diphosphate reductase (Fragment) OS=Homo sapiens PE=2 SV=1 | 77625.4 | 683 | 5.271 | 2 | 2 | 8 | 8 |
| 2405 | 2.41 | tr|A0A140VJS6|A0A140VJS6_HUMAN | Proteasome subunit beta type OS=Homo sapiens PE=2 SV=1 | 22836 | 201 | 7.463 | 1 | 1 | 17 | 17 |
| 2406 | 2.41 | sp|Q15392|DHC24_HUMAN | Delta(24)-sterol reductase OS=Homo sapiens GN=DHCR24 PE=1 SV=2 | 60100.8 | 516 | 1.744 | 1 | 1 | 2 | 2 |
| 2407 | 2.4 | tr|Q659C9|Q659C9_HUMAN | Putative uncharacterized protein DKFZp434L1715 OS=Homo sapiens GN=DKFZp434L1715 PE=2 SV=1 | 84637 | 753 | 1.594 | 1 | 1 | 1 | 1 |
| 2408 | 2.4 | tr|A8K2G0|A8K2G0_HUMAN | cDNA FLJ76605, highly similar to Homo sapiens secretory carrier membrane protein 1 (SCAMP1), transcript variant 1, mRNA OS=Homo sapiens PE=2 SV=1 | 37833 | 338 | 8.58 | 2 | 2 | 7 | 7 |
| 2409 | 2.39 | sp|Q6P587|FAHD1_HUMAN | Acylpyruvase FAHD1, mitochondrial OS=Homo sapiens GN=FAHD1 PE=1 SV=2 | 24842.7 | 224 | 3.571 | 1 | 1 | 14 | 14 |
| 2410 | 2.38 | sp|Q9Y2I8|WDR37_HUMAN | WD repeat-containing protein 37 OS=Homo sapiens GN=WDR37 PE=1 SV=2 | 54664.9 | 494 | 3.036 | 1 | 1 | 1 | 1 |
| 2411 | 2.38 | sp|Q15645|PCH2_HUMAN | Pachytene checkpoint protein 2 homolog OS=Homo sapiens GN=TRIP13 PE=1 SV=2 | 48550.3 | 432 | 7.407 | 3 | 3 | 6 | 6 |
| 2412 | 2.37 | tr|J3QRU1|J3QRU1_HUMAN | Tyrosine-protein kinase OS=Homo sapiens GN=YES1 PE=1 SV=1 | 61386.5 | 548 | 10.04 | 6 | 3 | 40 | 8 |
| 2413 | 2.37 | sp|Q9NUQ3|TXLNG_HUMAN | Gamma-taxilin OS=Homo sapiens GN=TXLNG PE=1 SV=2 | 60585.3 | 528 | 3.409 | 2 | 2 | 8 | 1 |
| 2414 | 2.37 | sp|Q9H8Y8|GORS2_HUMAN | Golgi reassembly-stacking protein 2 OS=Homo sapiens GN=GORASP2 PE=1 SV=3 | 47144.8 | 452 | 5.31 | 2 | 2 | 10 | 8 |
| 2415 | 2.36 | tr|H0YF29|H0YF29_HUMAN | UPF0598 protein C8orf82 (Fragment) OS=Homo sapiens GN=C8orf82 PE=1 SV=1 | 28543.7 | 261 | 5.747 | 2 | 1 | 7 | 2 |
| 2416 | 2.35 | sp|Q8TDZ2|MICA1_HUMAN | Protein-methionine sulfoxide oxidase MICAL1 OS=Homo sapiens GN=MICAL1 PE=1 SV=2 | 117873.8 | 1067 | 1.406 | 1 | 1 | 2 | 2 |
| 2417 | 2.35 | sp|Q96H20|SNF8_HUMAN | Vacuolar-sorting protein SNF8 OS=Homo sapiens GN=SNF8 PE=1 SV=1 | 28864.1 | 258 | 5.426 | 1 | 1 | 3 | 3 |
| 2418 | 2.35 | sp|O75391|SPAG7_HUMAN | Sperm-associated antigen 7 OS=Homo sapiens GN=SPAG7 PE=1 SV=2 | 26034.2 | 227 | 5.727 | 1 | 1 | 1 | 1 |
| 2419 | 2.35 | sp|Q9H6Y2|WDR55_HUMAN | WD repeat-containing protein 55 OS=Homo sapiens GN=WDR55 PE=1 SV=2 | 42069.6 | 383 | 9.661 | 2 | 2 | 4 | 4 |
| 2420 | 2.34 | tr|A0A140VJK1|A0A140VJK1_HUMAN | Testicular tissue protein Li 75 OS=Homo sapiens PE=2 SV=1 | 37431.7 | 335 | 3.284 | 1 | 1 | 2 | 2 |
| 2421 | 2.34 | tr|A0A0S2Z5D4|A0A0S2Z5D4_HUMAN | Dynactin 4 isoform 1 (Fragment) OS=Homo sapiens GN=DCTN4 PE=2 SV=1 | 52336.6 | 460 | 3.043 | 1 | 1 | 1 | 1 |
| 2422 | 2.34 | tr|B2RBA0|B2RBA0_HUMAN | cDNA, FLJ95388, highly similar to Homo sapiens step II splicing factor SLU7 (SLU7), mRNA OS=Homo sapiens PE=2 SV=1 | 68356.2 | 586 | 2.901 | 1 | 1 | 2 | 2 |
| 2423 | 2.33 | tr|J3KQY1|J3KQY1_HUMAN | 39S ribosomal protein L22, mitochondrial OS=Homo sapiens GN=MRPL22 PE=1 SV=1 | 26474.7 | 232 | 10.34 | 2 | 2 | 9 | 9 |
| 2424 | 2.33 | tr|W6A4U0|W6A4U0_HUMAN | Tetraspanin (Fragment) OS=Homo sapiens GN=CD63 PE=2 SV=1 | 25637.4 | 238 | 10.08 | 3 | 3 | 10 | 10 |
| 2425 | 2.32 | tr|A0A024R4U0|A0A024R4U0_HUMAN | ADP-ribosylation factor GTPase activating protein 3, isoform CRA_a OS=Homo sapiens GN=ARFGAP3 PE=4 SV=1 | 56927.9 | 516 | 5.233 | 2 | 2 | 3 | 3 |
| 2426 | 2.32 | tr|Q58A70|Q58A70_HUMAN | Polypeptide N-acetylgalactosaminyltransferase OS=Homo sapiens GN=GalNAc-T10 PE=2 SV=1 | 67035.9 | 581 | 1.549 | 1 | 1 | 1 | 1 |
| 2427 | 2.32 | sp|Q96IQ7|VSIG2_HUMAN | V-set and immunoglobulin domain-containing protein 2 OS=Homo sapiens GN=VSIG2 PE=1 SV=1 | 34348 | 327 | 4.893 | 1 | 1 | 7 | 7 |
| 2428 | 2.31 | tr|G3V1D0|G3V1D0_HUMAN | Echinoderm microtubule associated protein like 3, isoform CRA_f OS=Homo sapiens GN=EML3 PE=1 SV=1 | 95024.4 | 889 | 1.8 | 1 | 1 | 1 | 1 |
| 2429 | 2.31 | tr|A0A024R957|A0A024R957_HUMAN | Torsin A interacting protein 2, isoform CRA_b OS=Homo sapiens GN=TOR1AIP2 PE=4 SV=1 | 51262.9 | 470 | 2.34 | 1 | 1 | 3 | 3 |
| 2430 | 2.31 | sp|Q9NQ88|TIGAR_HUMAN | Fructose-2,6-bisphosphatase TIGAR OS=Homo sapiens GN=TIGAR PE=1 SV=1 | 30062.3 | 270 | 6.296 | 1 | 1 | 5 | 5 |
| 2431 | 2.31 | sp|Q9BRT3|MIEN1_HUMAN | Migration and invasion enhancer 1 OS=Homo sapiens GN=MIEN1 PE=1 SV=1 | 12402.8 | 115 | 29.57 | 2 | 2 | 6 | 6 |
| 2432 | 2.31 | tr|B7ZW01|B7ZW01_HUMAN | Metallothionein (Fragment) OS=Homo sapiens PE=2 SV=1 | 6605.7 | 66 | 18.18 | 1 | 1 | 5 | 5 |
| 2433 | 2.3 | sp|Q6PJG6|BRAT1_HUMAN | BRCA1-associated ATM activator 1 OS=Homo sapiens GN=BRAT1 PE=1 SV=2 | 88118 | 821 | 3.532 | 3 | 2 | 3 | 2 |
| 2434 | 2.3 | tr|Q7Z4X2|Q7Z4X2_HUMAN | Neuronal protein OS=Homo sapiens PE=2 SV=1 | 17942.2 | 158 | 6.962 | 1 | 1 | 6 | 6 |
| 2435 | 2.3 | tr|B3KW23|B3KW23_HUMAN | cDNA FLJ41961 fis, clone PUAEN2004083, highly similar to Nucleoporin p58/p45 OS=Homo sapiens PE=2 SV=1 | 58866.2 | 576 | 2.257 | 1 | 1 | 1 | 1 |
| 2436 | 2.3 | sp|Q9P0M9|RM27_HUMAN | 39S ribosomal protein L27, mitochondrial OS=Homo sapiens GN=MRPL27 PE=1 SV=1 | 16072.7 | 148 | 6.757 | 1 | 1 | 3 | 3 |
| 2437 | 2.3 | sp|P14735|IDE_HUMAN | Insulin-degrading enzyme OS=Homo sapiens GN=IDE PE=1 SV=4 | 117967.5 | 1019 | 1.374 | 1 | 1 | 2 | 2 |
| 2438 | 2.3 | sp|Q5JRA6|MIA3_HUMAN | Melanoma inhibitory activity protein 3 OS=Homo sapiens GN=MIA3 PE=1 SV=1 | 213700.8 | 1907 | 1.73 | 3 | 3 | 4 | 4 |
| 2439 | 2.3 | sp|P32929|CGL_HUMAN | Cystathionine gamma-lyase OS=Homo sapiens GN=CTH PE=1 SV=3 | 44507.6 | 405 | 10.86 | 2 | 2 | 2 | 2 |
| 2440 | 2.3 | sp|P61962|DCAF7_HUMAN | DDB1- and CUL4-associated factor 7 OS=Homo sapiens GN=DCAF7 PE=1 SV=1 | 38925.8 | 342 | 4.386 | 1 | 1 | 4 | 4 |
| 2441 | 2.29 | sp|Q9GZP8|IMUP_HUMAN | Immortalization up-regulated protein OS=Homo sapiens GN=IMUP PE=1 SV=1 | 10897 | 106 | 43.4 | 3 | 1 | 27 | 20 |
| 2442 | 2.29 | tr|Q6FHF7|Q6FHF7_HUMAN | RABGGTA protein (Fragment) OS=Homo sapiens GN=RABGGTA PE=2 SV=1 | 65041.1 | 567 | 1.764 | 1 | 1 | 1 | 1 |
| 2443 | 2.29 | tr|G5E9D5|G5E9D5_HUMAN | ElaC homolog 2 (E. coli), isoform CRA_a OS=Homo sapiens GN=ELAC2 PE=1 SV=1 | 90035.8 | 807 | 1.735 | 1 | 1 | 1 | 1 |
| 2444 | 2.29 | sp|Q9BSH4|TACO1_HUMAN | Translational activator of cytochrome c oxidase 1 OS=Homo sapiens GN=TACO1 PE=1 SV=1 | 32476.8 | 297 | 6.061 | 1 | 1 | 2 | 2 |
| 2445 | 2.29 | sp|Q9UHL4|DPP2_HUMAN | Dipeptidyl peptidase 2 OS=Homo sapiens GN=DPP7 PE=1 SV=3 | 54340.9 | 492 | 5.081 | 2 | 2 | 4 | 4 |
| 2446 | 2.29 | tr|Q6IBT5|Q6IBT5_HUMAN | NEU1 protein OS=Homo sapiens GN=NEU1 PE=2 SV=1 | 45493 | 415 | 8.675 | 3 | 3 | 6 | 6 |
| 2447 | 2.27 | sp|Q96Q11|TRNT1_HUMAN | CCA tRNA nucleotidyltransferase 1, mitochondrial OS=Homo sapiens GN=TRNT1 PE=1 SV=2 | 50127.1 | 434 | 6.452 | 3 | 3 | 3 | 3 |
| 2448 | 2.27 | tr|A0A140VJI5|A0A140VJI5_HUMAN | Dual specificity phosphatase 23 OS=Homo sapiens GN=DUSP23 PE=2 SV=1 | 16588 | 150 | 8 | 2 | 2 | 3 | 2 |
| 2449 | 2.27 | sp|Q96S44|PRPK_HUMAN | TP53-regulating kinase OS=Homo sapiens GN=TP53RK PE=1 SV=2 | 28160.1 | 253 | 11.86 | 2 | 2 | 10 | 10 |
| 2450 | 2.27 | sp|Q96GM8|TOE1_HUMAN | Target of EGR1 protein 1 OS=Homo sapiens GN=TOE1 PE=1 SV=1 | 56547.3 | 510 | 4.118 | 1 | 1 | 2 | 2 |
| 2451 | 2.27 | sp|Q99471|PFD5_HUMAN | Prefoldin subunit 5 OS=Homo sapiens GN=PFDN5 PE=1 SV=2 | 17328.1 | 154 | 26.62 | 3 | 3 | 4 | 4 |
| 2452 | 2.27 | sp|Q9Y6Y0|NS1BP_HUMAN | Influenza virus NS1A-binding protein OS=Homo sapiens GN=IVNS1ABP PE=1 SV=3 | 71728.8 | 642 | 4.517 | 2 | 2 | 4 | 4 |
| 2453 | 2.27 | sp|P49914|MTHFS_HUMAN | 5-formyltetrahydrofolate cyclo-ligase OS=Homo sapiens GN=MTHFS PE=1 SV=2 | 23255.4 | 203 | 10.34 | 2 | 2 | 6 | 6 |
| 2454 | 2.27 | tr|A8K0B5|A8K0B5_HUMAN | Protein archease OS=Homo sapiens GN=ZBTB8OS PE=1 SV=1 | 20675 | 179 | 9.497 | 1 | 1 | 3 | 3 |
| 2455 | 2.27 | tr|Q5SRQ6|Q5SRQ6_HUMAN | Casein kinase II subunit beta OS=Homo sapiens GN=CSNK2B PE=1 SV=2 | 26925.5 | 234 | 4.701 | 1 | 1 | 1 | 1 |
| 2456 | 2.26 | tr|Q6IBN6|Q6IBN6_HUMAN | CBX1 protein OS=Homo sapiens GN=CBX1 PE=2 SV=1 | 21417.6 | 185 | 18.38 | 3 | 2 | 10 | 2 |
| 2457 | 2.26 | tr|A0A024R897|A0A024R897_HUMAN | Actin-related protein 2/3 complex subunit 5 OS=Homo sapiens GN=ARPC5L PE=3 SV=1 | 16941 | 153 | 12.42 | 2 | 1 | 4 | 2 |
| 2458 | 2.26 | tr|A0A024R5C4|A0A024R5C4_HUMAN | Reticulon OS=Homo sapiens GN=RTN3 PE=4 SV=1 | 25608.8 | 236 | 22.88 | 2 | 2 | 4 | 4 |
| 2459 | 2.26 | tr|Q8TCM3|Q8TCM3_HUMAN | Putative uncharacterized protein DKFZp547M048 (Fragment) OS=Homo sapiens GN=DKFZp547M048 PE=2 SV=1 | 40277.2 | 353 | 5.099 | 1 | 1 | 1 | 1 |
| 2460 | 2.26 | tr|Q53XX5|Q53XX5_HUMAN | Cold inducible RNA binding protein OS=Homo sapiens GN=CIRBP PE=2 SV=1 | 18647.9 | 172 | 6.395 | 1 | 1 | 2 | 2 |
| 2461 | 2.26 | tr|B4DXL5|B4DXL5_HUMAN | cDNA FLJ52798 OS=Homo sapiens PE=2 SV=1 | 37499.4 | 336 | 9.226 | 2 | 2 | 4 | 4 |
| 2462 | 2.25 | tr|Q08AJ9|Q08AJ9_HUMAN | Histone H2A OS=Homo sapiens GN=HIST1H2AB PE=2 SV=1 | 14135.4 | 130 | 40.77 | 9 | 2 | 130 | 7 |
| 2463 | 2.25 | sp|Q9BSC4|NOL10_HUMAN | Nucleolar protein 10 OS=Homo sapiens GN=NOL10 PE=1 SV=1 | 80300.9 | 688 | 2.326 | 2 | 2 | 3 | 1 |
| 2464 | 2.25 | tr|Q5H9P4|Q5H9P4_HUMAN | Putative uncharacterized protein DKFZp686M19106 (Fragment) OS=Homo sapiens GN=DKFZp686M19106 PE=4 SV=1 | 99823.5 | 924 | 1.407 | 1 | 1 | 2 | 2 |
| 2465 | 2.25 | sp|Q9Y305|ACOT9_HUMAN | Acyl-coenzyme A thioesterase 9, mitochondrial OS=Homo sapiens GN=ACOT9 PE=1 SV=2 | 49901.3 | 439 | 5.923 | 2 | 2 | 5 | 5 |
| 2466 | 2.25 | tr|A0A0U1RRE5|A0A0U1RRE5_HUMAN | HCG1997177 OS=Homo sapiens GN=LINC01420 PE=1 SV=1 | 7024.9 | 68 | 39.71 | 1 | 1 | 8 | 8 |
| 2467 | 2.25 | sp|P56381|ATP5E_HUMAN | ATP synthase subunit epsilon, mitochondrial OS=Homo sapiens GN=ATP5E PE=1 SV=2 | 5779.7 | 51 | 29.41 | 2 | 2 | 2 | 2 |
| 2468 | 2.24 | tr|Q9BUN6|Q9BUN6_HUMAN | MRPS30 protein (Fragment) OS=Homo sapiens GN=MRPS30 PE=2 SV=2 | 50003.7 | 435 | 2.989 | 1 | 1 | 7 | 7 |
| 2469 | 2.24 | tr|A0A024R3W2|A0A024R3W2_HUMAN | Translocase of outer mitochondrial membrane 20 homolog (Yeast), isoform CRA_a OS=Homo sapiens GN=TOMM20 PE=3 SV=1 | 16297.7 | 145 | 8.966 | 1 | 1 | 10 | 10 |
| 2470 | 2.24 | tr|H3BNT2|H3BNT2_HUMAN | Ubiquinone biosynthesis protein COQ9, mitochondrial (Fragment) OS=Homo sapiens GN=COQ9 PE=1 SV=7 | 33942.8 | 304 | 6.908 | 1 | 1 | 1 | 1 |
| 2471 | 2.24 | tr|A0A087WV05|A0A087WV05_HUMAN | Uncharacterized protein OS=Homo sapiens PE=4 SV=1 | 12754.6 | 110 | 10 | 1 | 1 | 5 | 5 |
| 2472 | 2.23 | tr|B4DFY5|B4DFY5_HUMAN | Mitogen-activated protein kinase kinase 1, isoform CRA_d OS=Homo sapiens GN=MAP2K1 PE=2 SV=1 | 41378.4 | 371 | 4.582 | 2 | 1 | 4 | 3 |
| 2473 | 2.23 | sp|Q86Y56|DAAF5_HUMAN | Dynein assembly factor 5, axonemal OS=Homo sapiens GN=DNAAF5 PE=1 SV=4 | 93519.9 | 855 | 4.444 | 3 | 3 | 4 | 3 |
| 2474 | 2.23 | tr|A0A0A0MTJ5|A0A0A0MTJ5_HUMAN | Inactive tyrosine-protein kinase transmembrane receptor ROR1 OS=Homo sapiens GN=ROR1 PE=1 SV=1 | 98362.9 | 882 | 1.02 | 1 | 1 | 1 | 1 |
| 2475 | 2.23 | tr|B0AZM4|B0AZM4_HUMAN | cDNA, FLJ79464, highly similar to Homo sapiens aquarius (Fragment) OS=Homo sapiens PE=2 SV=1 | 156836.1 | 1353 | 1.996 | 2 | 2 | 4 | 4 |
[truncated: 113,220 more chars]
