# Supplementary material for: EspF of Enterohemorrhagic Escherichia coli Enhances Apoptosis via Endoplasmic Reticulum Stress in Intestinal Epithelial Cells: An Isobaric Tags for Relative and Absolute Quantitation-Based Comparative Proteomic Analysis
Source: Front Microbiol. 2022 Jun 30;13:900919. doi: 10.3389/fmicb.2022.900919 (PMC9279134; doi:10.3389/fmicb.2022.900919)
Supplement: Supplementary file 2 [file Table_2.DOCX]

**Table S2. The COG, KEGG function analysis of altered proteins within host cells after infection.**

**A. The differently expressed proteins comparing Δ*espF* to control group.**

| **N** | **Accession** | **Name** | **Homologous proteins** | **Up or down regulated** | **Expression ratio** | **P value** | **COG function classification** | **Kegg function**  **enrichment** |  |
| --- | --- | --- | --- | --- | --- | --- | --- | --- | --- |
| 5 | sp\|O75369\|FLNB_HUMAN | Filamin-B OS=Homo sapiens GN=FLNB PE=1 SV=2 | blank | down | 0.6256 | 0.00006 | Cytoskeleton ; | ko04010,MAPK signaling pathway;ko04510,Focal adhesion |  |
| 15 | tr\|A0A0S2Z3G9\|A0A0S2Z3G9_HUMAN | Actinin alpha 4 isoform 1 (Fragment) OS=Homo sapiens GN=ACTN4 PE=2 SV=1 | sp\|O43707\|ACTN4_HUMAN | up | 1.7658 | 0.01204 | Signal transduction mechanisms ; Cytoskeleton ; Cell cycle control, cell division, chromosome partitioning ; General function prediction only ;Cytoskeleton ; | ko04530,Tight junction;ko04520,Adherens junction;ko04810,Regulation of actin cytoskeleton;ko04670,Leukocyte transendothelial migration;ko05322,Systemic lupus erythematosus;ko04510,Focal adhesion;ko05146,Amoebiasis;ko05412,Arrhythmogenic right ventricular cardiomyopathy (ARVC) |  |
| 25 | tr\|V9HW80\|V9HW80_HUMAN | Epididymis luminal protein 220 OS=Homo sapiens GN=HEL-S-70 PE=2 SV=1 | sp\|P55072\|TERA_HUMAN | up | 1.7077 | 0.00006 | Posttranslational modification, protein turnover, chaperones ; | ko04141,Protein processing in endoplasmic reticulum |  |
| 32 | tr\|V9HWF4\|V9HWF4_HUMAN | Phosphoglycerate kinase OS=Homo sapiens GN=HEL-S-68p PE=2 SV=1 | sp\|P00558\|PGK1_HUMAN | up | 1.5664 | 0.00035 | Carbohydrate transport and metabolism ; | ko00010,Glycolysis / Gluconeogenesis;ko01120,Microbial metabolism in diverse environments;ko00710,Carbon fixation in photosynthetic organisms;ko01110,Biosynthesis of secondary metabolites;ko01100,Metabolic pathways |  |
| 34 | tr\|A0A090N8Y2\|A0A090N8Y2_HUMAN | Protein disulfide-isomerase A4 OS=Homo sapiens GN=ERP70 PE=2 SV=1 | sp\|P13667\|PDIA4_HUMAN | up | 1.9195 | 0.00937 | Posttranslational modification, protein turnover, chaperones ; Energy production and conversion ; | ko05110,Vibrio cholerae infection;ko04141,Protein processing in endoplasmic reticulum |  |
| 38 | tr\|Q5CAQ5\|Q5CAQ5_HUMAN | Tumor rejection antigen (Gp96) 1 OS=Homo sapiens GN=TRA1 PE=2 SV=1 | / | up | 1.5571 | 0.03793 | Posttranslational modification, protein turnover, chaperones ; | ko04621,NOD-like receptor signaling pathway;ko05200,Pathways in cancer;ko04626,Plant-pathogen interaction;ko05215,Prostate cancer;ko04141,Protein processing in endoplasmic reticulum |  |
| 42 | sp\|P11216\|PYGB_HUMAN | Glycogen phosphorylase, brain form OS=Homo sapiens GN=PYGB PE=1 SV=5 | / | up | 2.1491 | 0.00028 | Carbohydrate transport and metabolism ; | ko04910,Insulin signaling pathway;ko00500,Starch and sucrose metabolism |  |
| 43 | tr\|A0A0S2Z4G4\|A0A0S2Z4G4_HUMAN | Tropomyosin 3 isoform 1 (Fragment) OS=Homo sapiens GN=TPM3 PE=2 SV=1 | / | down | 0.4301 | 0.01263 | / | ko04260,Cardiac muscle contraction;ko05410,Hypertrophic cardiomyopathy (HCM);ko05414,Dilated cardiomyopathy;ko05200,Pathways in cancer;ko05216,Thyroid cancer |  |
| 46 | tr\|A0A024R9C1\|A0A024R9C1_HUMAN | Polyadenylate-binding protein OS=Homo sapiens GN=PABPC1 PE=3 SV=1 | sp\|P11940\|PABP1_HUMAN | down | 0.4547 | 0.00004 | General function prediction only ; | ko03015,mRNA surveillance pathway;ko03013,RNA transport |  |
| 52 | tr\|Q5TZZ9\|Q5TZZ9_HUMAN | Annexin OS=Homo sapiens GN=ANXA1 PE=2 SV=1 | sp\|P04083\|ANXA1_HUMAN | up | 1.7171 | 0.00086 | / | / |  |
| 57 | tr\|A0A024R1A3\|A0A024R1A3_HUMAN | Testicular secretory protein Li 63 OS=Homo sapiens GN=UBE1 PE=2 SV=1 | sp\|P22314\|UBA1_HUMAN | up | 1.9177 | 0.00544 | Coenzyme transport and metabolism ; | ko04120,Ubiquitin mediated proteolysis;ko05012,Parkinson's disease | |
| 58 | tr\|V9HVZ4\|V9HVZ4_HUMAN | Glyceraldehyde-3-phosphate dehydrogenase OS=Homo sapiens GN=HEL-S-162eP PE=2 SV=1 | sp\|P04406\|G3P_HUMAN | up | 1.6019 | 0.00878 | Carbohydrate transport and metabolism ; | ko05010,Alzheimer's disease;ko00010,Glycolysis / Gluconeogenesis;ko01120,Microbial metabolism in diverse environments;ko01110,Biosynthesis of secondary metabolites;ko01100,Metabolic pathways | |
| 60 | sp\|P35221\|CTNA1_HUMAN | Catenin alpha-1 OS=Homo sapiens GN=CTNNA1 PE=1 SV=1 | tr\|B4E2G8\|B4E2G8_HUMAN | down | 0.5702 | 0.00206 | / | ko04530,Tight junction;ko04520,Adherens junction;ko05213,Endometrial cancer;ko05100,Bacterial invasion of epithelial cells;ko05200,Pathways in cancer;ko04670,Leukocyte transendothelial migration;ko05412,Arrhythmogenic right ventricular cardiomyopathy (ARVC) | |
| 63 | sp\|P60174\|TPIS_HUMAN | Triosephosphate isomerase OS=Homo sapiens GN=TPI1 PE=1 SV=3 | / | up | 3.2393 | 0.00406 | Carbohydrate transport and metabolism ; | ko00051,Fructose and mannose metabolism;ko00010,Glycolysis / Gluconeogenesis;ko00562,Inositol phosphate metabolism;ko01120,Microbial metabolism in diverse environments;ko00710,Carbon fixation in photosynthetic organisms;ko01110,Biosynthesis of secondary metabolites;ko01100,Metabolic pathways | |
| 65 | tr\|V9HW72\|V9HW72_HUMAN | Epididymis secretory sperm binding protein Li 94n OS=Homo sapiens GN=HEL-S-94n PE=2 SV=1 | tr\|A8K690\|A8K690_HUMAN;sp\|P31948\|STIP1_HUMAN | up | 1.5520 | 0.01134 | General function prediction only ; | ko05020,Prion diseases | |
| 67 | sp\|P04843\|RPN1_HUMAN | Dolichyl-diphosphooligosaccharide--protein glycosyltransferase subunit 1 OS=Homo sapiens GN=RPN1 PE=1 SV=1 | / | down | 0.6373 | 0.03314 | / | ko01100,Metabolic pathways;ko00510,N-Glycan biosynthesis;ko04141,Protein processing in endoplasmic reticulum | |
| 95 | tr\|A0A0S2Z491\|A0A0S2Z491_HUMAN | Nucleophosmin isoform 2 (Fragment) OS=Homo sapiens GN=NPM1 PE=2 SV=1 | sp\|P06748\|NPM_HUMAN | up | 2.2443 | 0.01582 | / | / | |
| 108 | tr\|F5H5D3\|F5H5D3_HUMAN | Tubulin alpha chain OS=Homo sapiens GN=TUBA1C PE=1 SV=1 | / | up | 1.7122 | 0.03987 | Cytoskeleton ; | ko04540,Gap junction;ko05130,Pathogenic Escherichia coli infection;ko04145,Phagosome | |
| 112 | tr\|A0A0D9SFK2\|A0A0D9SFK2_HUMAN | Unconventional myosin-XVIIIa OS=Homo sapiens GN=MYO18A PE=1 SV=1 | / | down | 0.6205 | 0.00981 | Cytoskeleton ; | / | |
| 126 | tr\|K9JA46\|K9JA46_HUMAN | Epididymis luminal secretory protein 52 OS=Homo sapiens GN=EL52 PE=2 SV=1 | sp\|P07900\|HS90A_HUMAN | up | 1.9434 | 0.00243 | Posttranslational modification, protein turnover, chaperones ; | ko04621,NOD-like receptor signaling pathway;ko05200,Pathways in cancer;ko04626,Plant-pathogen interaction;ko04612,Antigen processing and presentation;ko04914,Progesterone-mediated oocyte maturation;ko05215,Prostate cancer;ko04141,Protein processing in endoplasmic reticulum | |
| 130 | tr\|V9HWJ2\|V9HWJ2_HUMAN | Isocitrate dehydrogenase [NADP] OS=Homo sapiens GN=HEL-S-26 PE=2 SV=1 | tr\|B2R5M8\|B2R5M8_HUMAN;sp\|O75874\|IDHC_HUMAN | up | 1.8009 | 0.04576 | Energy production and conversion ; | ko00480,Glutathione metabolism;ko04146,Peroxisome;ko01120,Microbial metabolism in diverse environments;ko01110,Biosynthesis of secondary metabolites;ko00020,Citrate cycle (TCA cycle);ko00720,Reductive carboxylate cycle (CO2 fixation);ko01100,Metabolic pathways | |
| 135 | sp\|P14866\|HNRPL_HUMAN | Heterogeneous nuclear ribonucleoprotein L OS=Homo sapiens GN=HNRNPL PE=1 SV=2 | / | down | 0.5411 | 0.00103 | / | / | |
| 143 | sp\|Q03252\|LMNB2_HUMAN | Lamin-B2 OS=Homo sapiens GN=LMNB2 PE=1 SV=4 | / | up | 1.8820 | 0.01534 | / | / | |
| 153 | sp\|Q13263\|TIF1B_HUMAN | Transcription intermediary factor 1-beta OS=Homo sapiens GN=TRIM28 PE=1 SV=5 | / | down | 0.6377 | 0.00008 | / | / | |
| 154 | sp\|P49588\|SYAC_HUMAN | Alanine--tRNA ligase, cytoplasmic OS=Homo sapiens GN=AARS PE=1 SV=2 | / | up | 1.8634 | 0.00569 | Translation, ribosomal structure and biogenesis ; | ko00970,Aminoacyl-tRNA biosynthesis | |
| 155 | tr\|B2R491\|B2R491_HUMAN | 40S ribosomal protein S4 OS=Homo sapiens GN=RPS4X PE=2 SV=1 | sp\|P62701\|RS4X_HUMAN;tr\|Q96IR1\|Q96IR1_HUMAN | up | 1.5181 | 0.00169 | Translation, ribosomal structure and biogenesis ; | ko03010,Ribosome | |
| 171 | sp\|P12429\|ANXA3_HUMAN | Annexin A3 OS=Homo sapiens GN=ANXA3 PE=1 SV=3 | / | up | 1.6455 | 0.01752 | / | / | |
| 174 | tr\|A0A024RCL8\|A0A024RCL8_HUMAN | Histone H2B OS=Homo sapiens GN=HIST1H2BK PE=3 SV=1 | sp\|O60814\|H2B1K_HUMAN | down | 0.1930 | 0.03848 | / | ko05322,Systemic lupus erythematosus | |
| 176 | tr\|A0A140VK53\|A0A140VK53_HUMAN | Testicular secretory protein Li 53 OS=Homo sapiens PE=2 SV=1 | sp\|Q9UQ35\|SRRM2_HUMAN | down | 0.6349 | 0.01144 | / | / | |
| 177 | sp\|P61604\|CH10_HUMAN | 10 kDa heat shock protein, mitochondrial OS=Homo sapiens GN=HSPE1 PE=1 SV=2 | / | up | 2.8127 | 0.01913 | Posttranslational modification, protein turnover, chaperones ; |  |  |
| 196 | tr\|V9HW69\|V9HW69_HUMAN | Epididymis secretory protein Li 66 OS=Homo sapiens GN=HEL-S-66 PE=2 SV=1 | tr\|B4DU58\|B4DU58_HUMAN;tr\|B2R9S4\|B2R9S4_HUMAN;sp\|P40121\|CAPG_HUMAN | up | 1.6868 | 0.00159 | / | / | |
| 200 | sp\|Q15019\|SEPT2_HUMAN | Septin-2 OS=Homo sapiens GN=SEPT2 PE=1 SV=1 | / | down | 0.6599 | 0.01712 | Cell cycle control, cell division, chromosome partitioning ; Cytoskeleton ; | ko05012,Parkinson's disease | |
| 202 | tr\|Q8N5Z7\|Q8N5Z7_HUMAN | 60S ribosomal protein L6 OS=Homo sapiens GN=RPL6 PE=2 SV=1 | tr\|A0A024RBK3\|A0A024RBK3_HUMAN;sp\|Q02878\|RL6_HUMAN;tr\|Q9HBB3\|Q9HBB3_HUMAN;tr\|Q8TBK5\|Q8TBK5_HUMAN | up | 1.5927 | 0.01863 | Translation, ribosomal structure and biogenesis ; | ko03010,Ribosome | |
| 215 | sp\|P23284\|PPIB_HUMAN | Peptidyl-prolyl cis-trans isomerase B OS=Homo sapiens GN=PPIB PE=1 SV=2 | tr\|V9HWC6\|V9HWC6_HUMAN | up | 3.7295 | 0.00038 | Posttranslational modification, protein turnover, chaperones ; | / | |
| 237 | tr\|B5BUB5\|B5BUB5_HUMAN | Autoantigen La (Fragment) OS=Homo sapiens GN=SSB PE=2 SV=1 | sp\|P05455\|LA_HUMAN | up | 1.7020 | 0.00896 | Posttranslational modification, protein turnover, chaperones ; Translation, ribosomal structure and biogenesis ; | ko05322,Systemic lupus erythematosus | |
| 244 | sp\|Q8NBS9\|TXND5_HUMAN | Thioredoxin domain-containing protein 5 OS=Homo sapiens GN=TXNDC5 PE=1 SV=2 | / | down | 0.6449 | 0.03442 | Posttranslational modification, protein turnover, chaperones ; Energy production and conversion ; | ko04141,Protein processing in endoplasmic reticulum | |
| 258 | tr\|V9HWE9\|V9HWE9_HUMAN | Epididymis secretory protein Li 22 OS=Homo sapiens GN=HEL-S-22 PE=2 SV=1 | sp\|P09211\|GSTP1_HUMAN | up | 1.6868 | 0.04152 | Posttranslational modification, protein turnover, chaperones ; | ko00480,Glutathione metabolism;ko00982,Drug metabolism - cytochrome P450;ko00980,Metabolism of xenobiotics by cytochrome P450 | |
| 262 | sp\|Q14789\|GOGB1_HUMAN | Golgin subfamily B member 1 OS=Homo sapiens GN=GOLGB1 PE=1 SV=2 | / | down | 0.5752 | 0.00126 | / | / | |
| 273 | tr\|Q8TC62\|Q8TC62_HUMAN | Septin 7 OS=Homo sapiens GN=SEPT7 PE=2 SV=3 | tr\|E7ES33\|E7ES33_HUMAN;tr\|E7EPK1\|E7EPK1_HUMAN;tr\|B4DUD6\|B4DUD6_HUMAN;tr\|A4GYY8\|A4GYY8_HUMAN;sp\|Q16181\|SEPT7_HUMAN;tr\|Q3LIE9\|Q3LIE9_HUMAN | down | 0.4324 | 0.01523 | Cell cycle control, cell division, chromosome partitioning ; Cytoskeleton ; | ko05012,Parkinson's disease | |
| 286 | tr\|A0A0S2Z4A5\|A0A0S2Z4A5_HUMAN | DNA helicase (Fragment) OS=Homo sapiens GN=MCM7 PE=2 SV=1 | sp\|P33993\|MCM7_HUMAN;tr\|B2RBA6\|B2RBA6_HUMAN | down | 0.5337 | 0.02155 | Replication, recombination and repair ; | ko04113,Meiosis - yeast;ko04111,Cell cycle - yeast;ko03030,DNA replication;ko04110,Cell cycle | |
| 290 | sp\|Q7Z2K6\|ERMP1_HUMAN | Endoplasmic reticulum metallopeptidase 1 OS=Homo sapiens GN=ERMP1 PE=1 SV=2 | / | up | 1.5051 | 0.04847 | General function prediction only ; | / | |
| 300 | sp\|P07737\|PROF1_HUMAN | Profilin-1 OS=Homo sapiens GN=PFN1 PE=1 SV=2 | / | up | 1.6315 | 0.00012 | / | ko04810,Regulation of actin cytoskeleton;ko05131,Shigellosis | |
| 301 | tr\|A0A024R1K8\|A0A024R1K8_HUMAN | Splicing factor 3a, subunit 1, 120kDa, isoform CRA_a OS=Homo sapiens GN=SF3A1 PE=4 SV=1 | sp\|Q15459\|SF3A1_HUMAN | down | 0.6581 | 0.00557 | / | ko03040,Spliceosome | |
| 303 | tr\|A0A0S2Z4N8\|A0A0S2Z4N8_HUMAN | Vasodilator-stimulated phosphoprotein isoform 2 (Fragment) OS=Homo sapiens GN=VASP PE=2 SV=1 | tr\|A0A024R0V4\|A0A024R0V4_HUMAN;sp\|P50552\|VASP_HUMAN;tr\|A0A0S2Z4I9\|A0A0S2Z4I9_HUMAN | down | 0.4248 | 0.00055 | / | ko04666,Fc gamma R-mediated phagocytosis;ko04670,Leukocyte transendothelial migration;ko04510,Focal adhesion | |
| 304 | sp\|P16401\|H15_HUMAN | Histone H1.5 OS=Homo sapiens GN=HIST1H1B PE=1 SV=3 | / | up | 2.5202 | 0.04653 | / | / | |
| 313 | tr\|A0A140VJT8\|A0A140VJT8_HUMAN | Testicular tissue protein Li 164 OS=Homo sapiens PE=2 SV=1 | tr\|A0A024RC87\|A0A024RC87_HUMAN;sp\|P13489\|RINI_HUMAN | down | 0.5312 | 0.04665 | / | ko04621,NOD-like receptor signaling pathway | |
| 328 | sp\|P27824\|CALX_HUMAN | Calnexin OS=Homo sapiens GN=CANX PE=1 SV=2 | / | up | 1.7963 | 0.03609 | / | ko04612,Antigen processing and presentation;ko04145,Phagosome;ko04141,Protein processing in endoplasmic reticulum | |
| 336 | sp\|P11387\|TOP1_HUMAN | DNA topoisomerase 1 OS=Homo sapiens GN=TOP1 PE=1 SV=2 | / | up | 2.2340 | 0.00421 | Replication, recombination and repair ; | / | |
| 339 | sp\|P12004\|PCNA_HUMAN | Proliferating cell nuclear antigen OS=Homo sapiens GN=PCNA PE=1 SV=1 | / | down | 0.6038 | 0.00035 | Replication, recombination and repair ; | ko03430,Mismatch repair;ko03410,Base excision repair;ko03030,DNA replication;ko04110,Cell cycle;ko03420,Nucleotide excision repair | |
| 350 | sp\|P62277\|RS13_HUMAN | 40S ribosomal protein S13 OS=Homo sapiens GN=RPS13 PE=1 SV=2 | / | up | 1.7428 | 0.01157 | Translation, ribosomal structure and biogenesis ; | ko03010,Ribosome | |
| 378 | sp\|Q8TCS8\|PNPT1_HUMAN | Polyribonucleotide nucleotidyltransferase 1, mitochondrial OS=Homo sapiens GN=PNPT1 PE=1 SV=2 | / | down | 0.4988 | 0.00158 | Translation, ribosomal structure and biogenesis ; | ko00240,Pyrimidine metabolism;ko00230,Purine metabolism;ko03018,RNA degradation | |
| 381 | tr\|A0A024RAI1\|A0A024RAI1_HUMAN | ARP3 actin-related protein 3 homolog (Yeast), isoform CRA_a OS=Homo sapiens GN=ACTR3 PE=3 SV=1 | sp\|P61158\|ARP3_HUMAN | down | 0.6205 | 0.03520 | Cytoskeleton ; | ko04530,Tight junction;ko04520,Adherens junction;ko05410,Hypertrophic cardiomyopathy (HCM);ko04810,Regulation of actin cytoskeleton;ko05414,Dilated cardiomyopathy;ko04670,Leukocyte transendothelial migration;ko05416,Viral myocarditis;ko04510,Focal adhesion;ko05131,Shigellosis;ko04745,Phototransduction - fly;ko05110,Vibrio cholerae infection;ko05100,Bacterial invasion of epithelial cells;ko05130,Pathogenic Escherichia coli infection;ko05412,Arrhythmogenic right ventricular cardiomyopathy (ARVC);ko04145,Phagosome | |
| 400 | sp\|P15880\|RS2_HUMAN | 40S ribosomal protein S2 OS=Homo sapiens GN=RPS2 PE=1 SV=2 | / | up | 1.7381 | 0.00881 | Translation, ribosomal structure and biogenesis ; | ko03010,Ribosome | |
| 416 | tr\|D6RD18\|D6RD18_HUMAN | Heterogeneous nuclear ribonucleoprotein A/B OS=Homo sapiens GN=HNRNPAB PE=1 SV=1 | / | up | 1.9545 | 0.01893 | General function prediction only ; | / | |
| 419 | sp\|P08727\|K1C19_HUMAN | Keratin, type I cytoskeletal 19 OS=Homo sapiens GN=KRT19 PE=1 SV=4 | / | up | 1.8385 | 0.01588 | / | / | |
| 428 | sp\|Q15075\|EEA1_HUMAN | Early endosome antigen 1 OS=Homo sapiens GN=EEA1 PE=1 SV=2 | / | up | 1.8268 | 0.03712 | / | ko04144,Endocytosis;ko04145,Phagosome | |
| 438 | tr\|B5BU83\|B5BU83_HUMAN | Stathmin OS=Homo sapiens GN=STMN1 PE=2 SV=1 | sp\|P16949\|STMN1_HUMAN | down | 0.6541 | 0.04035 | / | ko04010,MAPK signaling pathway | |
| 443 | tr\|V9HW01\|V9HW01_HUMAN | Epididymis secretory protein Li 310 OS=Homo sapiens GN=HEL-S-310 PE=2 SV=1 | sp\|P83731\|RL24_HUMAN | down | 0.6121 | 0.00671 | Translation, ribosomal structure and biogenesis ; | ko03010,Ribosome | |
| 459 | tr\|Q53XM7\|Q53XM7_HUMAN | VAMP (Vesicle-associated membrane protein)-associated protein B and C OS=Homo sapiens GN=VAPB PE=1 SV=1 | sp\|O95292\|VAPB_HUMAN | down | 0.6004 | 0.04663 | Intracellular trafficking, secretion, and vesicular transport ; | / | |
| 470 | tr\|A0A024R5C5\|A0A024R5C5_HUMAN | Pyruvate carboxylase OS=Homo sapiens GN=PC PE=4 SV=1 | sp\|P11498\|PYC_HUMAN | up | 1.7868 | 0.01974 | Energy production and conversion ;Lipid transport and metabolism ; | ko01120,Microbial metabolism in diverse environments;ko00020,Citrate cycle (TCA cycle);ko01100,Metabolic pathways;ko00620,Pyruvate metabolism | |
| 487 | sp\|Q71DI3\|H32_HUMAN | Histone H3.2 OS=Homo sapiens GN=HIST2H3A PE=1 SV=3 | / | up | 2.3730 | 0.01006 | Chromatin structure and dynamics ; | ko05322,Systemic lupus erythematosus | |
| 489 | sp\|P22087\|FBRL_HUMAN | rRNA 2'-O-methyltransferase fibrillarin OS=Homo sapiens GN=FBL PE=1 SV=2 | / | down | 0.3800 | 0.01269 | Translation, ribosomal structure and biogenesis ; | / | |
| 502 | sp\|P35527\|K1C9_HUMAN | Keratin, type I cytoskeletal 9 OS=Homo sapiens GN=KRT9 PE=1 SV=3 | / | down | 0.6383 | 0.01326 | / | / | |
| 511 | sp\|Q12797\|ASPH_HUMAN | Aspartyl/asparaginyl beta-hydroxylase OS=Homo sapiens GN=ASPH PE=1 SV=3 | / | up | 1.6455 | 0.03607 | Posttranslational modification, protein turnover, chaperones ; |  |  |
| 516 | tr\|A8K4Z4\|A8K4Z4_HUMAN | 60S acidic ribosomal protein P0 OS=Homo sapiens PE=2 SV=1 | tr\|A0A024RBS2\|A0A024RBS2_HUMAN;sp\|P05388\|RLA0_HUMAN | up | 1.6167 | 0.04399 | Translation, ribosomal structure and biogenesis ; | ko03010,Ribosome | |
| 536 | sp\|Q9UMS4\|PRP19_HUMAN | Pre-mRNA-processing factor 19 OS=Homo sapiens GN=PRPF19 PE=1 SV=1 | / | down | 0.6189 | 0.02709 | General function prediction only ; | ko04120,Ubiquitin mediated proteolysis;ko03040,Spliceosome | |
| 539 | sp\|P06703\|S10A6_HUMAN | Protein S100-A6 OS=Homo sapiens GN=S100A6 PE=1 SV=1 | / | up | 5.2158 | 0.01334 | / | / | |
| 557 | tr\|Q59FR8\|Q59FR8_HUMAN | Galectin (Fragment) OS=Homo sapiens PE=2 SV=1 | tr\|Q86TY5\|Q86TY5_HUMAN;tr\|Q6FGL0\|Q6FGL0_HUMAN;tr\|A0A024R693\|A0A024R693_HUMAN;sp\|P17931\|LEG3_HUMAN | up | 1.6506 | 0.03646 | / | / | |
| 563 | sp\|P62263\|RS14_HUMAN | 40S ribosomal protein S14 OS=Homo sapiens GN=RPS14 PE=1 SV=3 | / | down | 0.6574 | 0.03737 | Translation, ribosomal structure and biogenesis ; | ko03010,Ribosome | |
| 584 | sp\|P20290\|BTF3_HUMAN | Transcription factor BTF3 OS=Homo sapiens GN=BTF3 PE=1 SV=1 | / | down | 0.3756 | 0.00297 | / | / | |
| 594 | tr\|D6W5K2\|D6W5K2_HUMAN | Thymosin, beta 10, isoform CRA_a (Fragment) OS=Homo sapiens GN=TMSB10 PE=4 SV=1 | / | down | 0.5631 | 0.00658 | / | / | |
| 611 | tr\|A0A024RDG6\|A0A024RDG6_HUMAN | Scavenger receptor class B, member 2, isoform CRA_a OS=Homo sapiens GN=SCARB2 PE=3 SV=1 | sp\|Q14108\|SCRB2_HUMAN | up | 1.9058 | 0.02257 | / | ko04142,Lysosome | |
| 616 | tr\|B2R761\|B2R761_HUMAN | cDNA, FLJ93299, highly similar to Homo sapiens sterol carrier protein 2 (SCP2), mRNA OS=Homo sapiens PE=2 SV=1 | / | up | 1.6396 | 0.03100 | Lipid transport and metabolism ; | ko04146,Peroxisome;ko03320,PPAR signaling pathway;ko01100,Metabolic pathways;ko00120,Primary bile acid biosynthesis | |
| 641 | tr\|J3QQ67\|J3QQ67_HUMAN | 60S ribosomal protein L18 (Fragment) OS=Homo sapiens GN=RPL18 PE=1 SV=1 | tr\|A0A024QZD1\|A0A024QZD1_HUMAN;sp\|Q07020\|RL18_HUMAN;tr\|G3V203\|G3V203_HUMAN | down | 0.6105 | 0.00212 | Translation, ribosomal structure and biogenesis ; | ko03010,Ribosome | |
| 647 | tr\|B4DDI9\|B4DDI9_HUMAN | NADPH:adrenodoxin oxidoreductase, mitochondrial OS=Homo sapiens PE=2 SV=1 | / | down | 0.5874 | 0.01138 | Amino acid transport and metabolism ; General function prediction only ; |  |  |
| 648 | tr\|D9IAI1\|D9IAI1_HUMAN | Epididymis secretory protein Li 34 OS=Homo sapiens GN=PEBP1 PE=2 SV=1 | sp\|P30086\|PEBP1_HUMAN | up | 1.7862 | 0.02639 | General function prediction only ; | / | |
| 668 | sp\|P31689\|DNJA1_HUMAN | DnaJ homolog subfamily A member 1 OS=Homo sapiens GN=DNAJA1 PE=1 SV=2 | / | down | 0.4820 | 0.01041 | Posttranslational modification, protein turnover, chaperones ; | ko04141,Protein processing in endoplasmic reticulum | |
| 674 | sp\|P11388\|TOP2A_HUMAN | DNA topoisomerase 2-alpha OS=Homo sapiens GN=TOP2A PE=1 SV=3 | / | down | 0.4896 | 0.00743 | Replication, recombination and repair ; | / | |
| 683 | tr\|A0A140VKA6\|A0A140VKA6_HUMAN | Testis secretory sperm-binding protein Li 233m OS=Homo sapiens PE=2 SV=1 | sp\|P50502\|F10A1_HUMAN | up | 1.8286 | 0.04480 | General function prediction only ; | / | |
| 691 | tr\|E9PB61\|E9PB61_HUMAN | THO complex subunit 4 OS=Homo sapiens GN=ALYREF PE=1 SV=1 | sp\|Q86V81\|THOC4_HUMAN | down | 0.5242 | 0.02611 | / | ko03015,mRNA surveillance pathway;ko03040,Spliceosome;ko03013,RNA transport | |
| 696 | sp\|Q15942\|ZYX_HUMAN | Zyxin OS=Homo sapiens GN=ZYX PE=1 SV=1 | tr\|H0Y2Y8\|H0Y2Y8_HUMAN | down | 0.4640 | 0.01979 | / | ko04510,Focal adhesion | |
| 719 | tr\|Q6NVW7\|Q6NVW7_HUMAN | Importin subunit alpha OS=Homo sapiens GN=KPNA2 PE=2 SV=1 | / | down | 0.5263 | 0.01146 | Intracellular trafficking, secretion, and vesicular transport ; | / | |
| 737 | tr\|B2RBM7\|B2RBM7_HUMAN | cDNA, FLJ95595, highly similar to Homo sapiens proteasome (prosome, macropain) 26S subunit, non-ATPase, 13 (PSMD13), mRNA OS=Homo sapiens PE=2 SV=1 | sp\|Q9UNM6\|PSD13_HUMAN;tr\|Q9Y6E3\|Q9Y6E3_HUMAN;tr\|Q53GN6\|Q53GN6_HUMAN;tr\|A0A024R201\|A0A024R201_HUMAN | up | 1.5352 | 0.02923 | / | ko03050,Proteasome | |
| 756 | sp\|Q9BS26\|ERP44_HUMAN | Endoplasmic reticulum resident protein 44 OS=Homo sapiens GN=ERP44 PE=1 SV=1 | / | up | 1.6073 | 0.02639 | Posttranslational modification, protein turnover, chaperones ; Energy production and conversion ; | ko05110,Vibrio cholerae infection;ko04141,Protein processing in endoplasmic reticulum | |
| 759 | sp\|P35611\|ADDA_HUMAN | Alpha-adducin OS=Homo sapiens GN=ADD1 PE=1 SV=2 | / | down | 0.5141 | 0.04724 | Carbohydrate transport and metabolism ; | / | |
| 770 | tr\|Q0P5N8\|Q0P5N8_HUMAN | TMSB4X protein (Fragment) OS=Homo sapiens GN=TMSB4X PE=2 SV=1 | tr\|Q0P5U7\|Q0P5U7_HUMAN;tr\|Q0P5P4\|Q0P5P4_HUMAN;tr\|Q0P5T0\|Q0P5T0_HUMAN;tr\|Q0P5Q0\|Q0P5Q0_HUMAN;tr\|A2VCK8\|A2VCK8_HUMAN;sp\|P62328\|TYB4_HUMAN | up | 1.6026 | 0.00927 | / | ko04810,Regulation of actin cytoskeleton | |
| 783 | sp\|Q13404\|UB2V1_HUMAN | Ubiquitin-conjugating enzyme E2 variant 1 OS=Homo sapiens GN=UBE2V1 PE=1 SV=2 | / | up | 2.8635 | 0.04000 | / | / | |
| 794 | tr\|A0A024R277\|A0A024R277_HUMAN | Serine palmitoyltransferase, long chain base subunit 1, isoform CRA_a OS=Homo sapiens GN=SPTLC1 PE=4 SV=1 | sp\|O15269\|SPTC1_HUMAN | down | 0.5042 | 0.04415 | Coenzyme transport and metabolism ; | ko00600,Sphingolipid metabolism;ko01100,Metabolic pathways | |
| 796 | tr\|B3KM90\|B3KM90_HUMAN | cDNA FLJ10529 fis, clone NT2RP2000965, highly similar to Targeting protein for Xklp2 OS=Homo sapiens PE=2 SV=1 | / | down | 0.3312 | 0.00652 | / | / | |
| 812 | tr\|Q6FI03\|Q6FI03_HUMAN | G3BP protein OS=Homo sapiens GN=G3BP PE=2 SV=1 | tr\|Q5U0Q1\|Q5U0Q1_HUMAN;sp\|Q13283\|G3BP1_HUMAN | down | 0.5390 | 0.04273 | / | ko03015,mRNA surveillance pathway;ko03040,Spliceosome;ko03013,RNA transport | |
| 816 | tr\|A0A024R3W7\|A0A024R3W7_HUMAN | Eukaryotic translation elongation factor 1 beta 2, isoform CRA_a OS=Homo sapiens GN=EEF1B2 PE=3 SV=1 | sp\|P24534\|EF1B_HUMAN | down | 0.5665 | 0.02462 | Translation, ribosomal structure and biogenesis ; | / | |
| 823 | tr\|B3KNJ4\|B3KNJ4_HUMAN | SUMO-1 activating enzyme subunit 1, isoform CRA_a OS=Homo sapiens GN=SAE1 PE=1 SV=1 | / | down | 0.3599 | 0.04546 | Coenzyme transport and metabolism ; | ko04120,Ubiquitin mediated proteolysis | |
| 833 | tr\|Q5STK2\|Q5STK2_HUMAN | Prefoldin subunit 6, isoform CRA_b OS=Homo sapiens GN=PFDN6 PE=2 SV=1 | sp\|O15212\|PFD6_HUMAN | down | 0.6258 | 0.03505 | Posttranslational modification, protein turnover, chaperones ; | / | |
| 864 | tr\|Q6FIA3\|Q6FIA3_HUMAN | PACSIN2 protein OS=Homo sapiens GN=PACSIN2 PE=2 SV=1 | sp\|Q9UNF0\|PACN2_HUMAN | up | 5.0947 | 0.01098 | / | ko04530,Tight junction;ko05100,Bacterial invasion of epithelial cells;ko05016,Huntington's disease;ko04660,T cell receptor signaling pathway;ko05130,Pathogenic Escherichia coli infection;ko05131,Shigellosis | |
| 880 | tr\|B2R4C1\|B2R4C1_HUMAN | cDNA, FLJ92036, highly similar to Homo sapiens ribosomal protein L31 (RPL31), mRNA OS=Homo sapiens PE=2 SV=1 | sp\|P62899\|RL31_HUMAN | down | 0.6534 | 0.00307 | Translation, ribosomal structure and biogenesis ; | ko03010,Ribosome | |
| 890 | tr\|A0A024R0Q5\|A0A024R0Q5_HUMAN | Protein phosphatase 1, regulatory (Inhibitor) subunit 13 like, isoform CRA_a OS=Homo sapiens GN=PPP1R13L PE=4 SV=1 | sp\|Q8WUF5\|IASPP_HUMAN | down | 0.6546 | 0.02261 | / | ko04270,Vascular smooth muscle contraction;ko04810,Regulation of actin cytoskeleton;ko04510,Focal adhesion;ko04720,Long-term potentiation | |
| 892 | sp\|P25398\|RS12_HUMAN | 40S ribosomal protein S12 OS=Homo sapiens GN=RPS12 PE=1 SV=3 | / | up | 1.6121 | 0.04125 | Translation, ribosomal structure and biogenesis ; | ko03010,Ribosome | |
| 915 | sp\|A6NHR9\|SMHD1_HUMAN | Structural maintenance of chromosomes flexible hinge domain-containing protein 1 OS=Homo sapiens GN=SMCHD1 PE=1 SV=2 | / | down | 0.6292 | 0.03120 | / | / | |
| 926 | tr\|A4D177\|A4D177_HUMAN | Chromobox homolog 3 (HP1 gamma homolog, Drosophila) OS=Homo sapiens GN=CBX3 PE=4 SV=1 | sp\|Q13185\|CBX3_HUMAN | down | 0.3478 | 0.01537 | / | / | |
| 934 | tr\|I3L504\|I3L504_HUMAN | Eukaryotic translation initiation factor 5A-1 OS=Homo sapiens GN=EIF5A PE=1 SV=1 | / | up | 1.5626 | 0.04173 | Translation, ribosomal structure and biogenesis ; | / | |
| 949 | tr\|Q53G49\|Q53G49_HUMAN | Ribosomal protein L19 (Fragment) OS=Homo sapiens PE=2 SV=1 | tr\|J3QR09\|J3QR09_HUMAN;tr\|J3KTE4\|J3KTE4_HUMAN;sp\|P84098\|RL19_HUMAN | up | 1.9488 | 0.00835 | Translation, ribosomal structure and biogenesis ; | ko03010,Ribosome | |
| 954 | sp\|Q14126\|DSG2_HUMAN | Desmoglein-2 OS=Homo sapiens GN=DSG2 PE=1 SV=2 | / | down | 0.5619 | 0.01973 | / | ko05412,Arrhythmogenic right ventricular cardiomyopathy (ARVC) | |
| 955 | sp\|P08243\|ASNS_HUMAN | Asparagine synthetase [glutamine-hydrolyzing] OS=Homo sapiens GN=ASNS PE=1 SV=4 | / | up | 2.4234 | 0.02292 | Amino acid transport and metabolism ; | ko01120,Microbial metabolism in diverse environments;ko01110,Biosynthesis of secondary metabolites;ko00250,Alanine, aspartate and glutamate metabolism;ko00910,Nitrogen metabolism;ko01100,Metabolic pathways | |
| 986 | sp\|P52701\|MSH6_HUMAN | DNA mismatch repair protein Msh6 OS=Homo sapiens GN=MSH6 PE=1 SV=2 | / | down | 0.5029 | 0.04635 | Replication, recombination and repair ; | ko05200,Pathways in cancer;ko03430,Mismatch repair;ko05210,Colorectal cancer | |
| 1001 | sp\|Q9H6T3\|RPAP3_HUMAN | RNA polymerase II-associated protein 3 OS=Homo sapiens GN=RPAP3 PE=1 SV=2 | / | up | 2.2148 | 0.02199 | General function prediction only ; | ko05020,Prion diseases | |
| 1019 | tr\|A0A024R2Q4\|A0A024R2Q4_HUMAN | Ribosomal protein L15 OS=Homo sapiens GN=RPL15 PE=3 SV=1 | sp\|P61313\|RL15_HUMAN | down | 0.5569 | 0.01575 | Translation, ribosomal structure and biogenesis ; | ko03010,Ribosome | |
| 1026 | tr\|B2R4C5\|B2R4C5_HUMAN | C-type lysozyme OS=Homo sapiens GN=LYZ PE=2 SV=1 | sp\|P61626\|LYSC_HUMAN | down | 0.4806 | 0.02045 | / | ko04970,Salivary secretion | |
| 1031 | sp\|P46776\|RL27A_HUMAN | 60S ribosomal protein L27a OS=Homo sapiens GN=RPL27A PE=1 SV=2 | / | up | 1.8666 | 0.01151 | Translation, ribosomal structure and biogenesis ; | ko03010,Ribosome | |
| 1080 | sp\|P14324\|FPPS_HUMAN | Farnesyl pyrophosphate synthase OS=Homo sapiens GN=FDPS PE=1 SV=4 | / | up | 1.9237 | 0.04553 | Coenzyme transport and metabolism ; | ko00900,Terpenoid backbone biosynthesis;ko01110,Biosynthesis of secondary metabolites;ko01100,Metabolic pathways | |
| 1082 | tr\|C9JEJ2\|C9JEJ2_HUMAN | Choline-phosphate cytidylyltransferase A OS=Homo sapiens GN=PCYT1A PE=1 SV=1 | sp\|P49585\|PCY1A_HUMAN | down | 0.6150 | 0.04375 | Cell wall/membrane/envelope biogenesis ; Lipid transport and metabolism ; | ko00440,Phosphonate and phosphinate metabolism;ko00564,Glycerophospholipid metabolism;ko01100,Metabolic pathways | |
| 1145 | tr\|Q6IBR8\|Q6IBR8_HUMAN | EIF2S2 protein OS=Homo sapiens GN=EIF2S2 PE=2 SV=1 | tr\|B5BU01\|B5BU01_HUMAN;sp\|P20042\|IF2B_HUMAN | up | 3.0551 | 0.01866 | Translation, ribosomal structure and biogenesis ; | ko03013,RNA transport | |
| 1164 | sp\|P27144\|KAD4_HUMAN | Adenylate kinase 4, mitochondrial OS=Homo sapiens GN=AK4 PE=1 SV=1 | / | up | 1.8527 | 0.03162 | Nucleotide transport and metabolism ; | ko01110,Biosynthesis of secondary metabolites;ko00230,Purine metabolism;ko01100,Metabolic pathways | |
| 1243 | tr\|D3DPK5\|D3DPK5_HUMAN | SH3 domain binding glutamic acid-rich protein like 3, isoform CRA_a (Fragment) OS=Homo sapiens GN=SH3BGRL3 PE=4 SV=1 | / | down | 0.3758 | 0.01299 | / | ko04972,Pancreatic secretion;ko04370,VEGF signaling pathway;ko00591,Linoleic acid metabolism;ko04270,Vascular smooth muscle contraction;ko04730,Long-term depression;ko04664,Fc epsilon RI signaling pathway;ko00564,Glycerophospholipid metabolism;ko01100,Metabolic pathways;ko05145,Toxoplasmosis;ko04912,GnRH signaling pathway;ko00590,Arachidonic acid metabolism;ko04010,MAPK signaling pathway;ko00565,Ether lipid metabolism;ko00592,alpha-Linolenic acid metabolism | |
| 1244 | tr\|Q5SU16\|Q5SU16_HUMAN | Tubulin beta chain OS=Homo sapiens GN=TUBB PE=2 SV=1 | sp\|P07437\|TBB5_HUMAN | down | 0.6625 | 0.02779 | Cytoskeleton ; | ko04540,Gap junction;ko05130,Pathogenic Escherichia coli infection;ko04145,Phagosome | |
| 1261 | tr\|A0A024QZN2\|A0A024QZN2_HUMAN | HCG2024613, isoform CRA_a OS=Homo sapiens GN=hCG_2024613 PE=4 SV=1 | sp\|Q8WXX5\|DNJC9_HUMAN | up | 2.3023 | 0.04046 | Posttranslational modification, protein turnover, chaperones ; | / | |
| 1353 | tr\|E7DVW5\|E7DVW5_HUMAN | Fatty acid binding protein 5 (Psoriasis-associated) OS=Homo sapiens GN=FABP5 PE=3 SV=1 | sp\|Q01469\|FABP5_HUMAN | down | 0.3863 | 0.01860 | / | ko03320,PPAR signaling pathway | |
| 1387 | tr\|Q53H94\|Q53H94_HUMAN | Aldehyde dehydrogenase 6A1 variant (Fragment) OS=Homo sapiens PE=2 SV=1 | tr\|Q53FN8\|Q53FN8_HUMAN;tr\|A0A024R6G4\|A0A024R6G4_HUMAN;sp\|Q02252\|MMSA_HUMAN | up | 1.8700 | 0.01643 | Energy production and conversion ; | ko00562,Inositol phosphate metabolism;ko00280,Valine, leucine and isoleucine degradation;ko01100,Metabolic pathways;ko00640,Propanoate metabolism | |
| 1393 | tr\|Q86U62\|Q86U62_HUMAN | Proteasome subunit beta type OS=Homo sapiens PE=2 SV=1 | tr\|Q6IBT1\|Q6IBT1_HUMAN;tr\|E9KL30\|E9KL30_HUMAN;tr\|B2RAQ9\|B2RAQ9_HUMAN;sp\|Q99436\|PSB7_HUMAN | up | 2.9155 | 0.04862 | Posttranslational modification, protein turnover, chaperones ; | ko03050,Proteasome | |
| 1406 | sp\|O00116\|ADAS_HUMAN | Alkyldihydroxyacetonephosphate synthase, peroxisomal OS=Homo sapiens GN=AGPS PE=1 SV=1 | / | down | 0.6292 | 0.02890 | Energy production and conversion ; | ko04146,Peroxisome;ko00565,Ether lipid metabolism;ko01100,Metabolic pathways | |
| 1497 | tr\|C9JP00\|C9JP00_HUMAN | Muscleblind-like protein 1 OS=Homo sapiens GN=MBNL1 PE=1 SV=1 | tr\|A0A0A0MQX8\|A0A0A0MQX8_HUMAN;sp\|Q9NR56\|MBNL1_HUMAN | down | 0.5735 | 0.03219 | / | / | |
| 1527 | tr\|Q59EL4\|Q59EL4_HUMAN | PRPF4 protein variant (Fragment) OS=Homo sapiens PE=2 SV=1 | / | down | 0.4334 | 0.03941 | General function prediction only ; | ko03040,Spliceosome | |
| 1535 | tr\|Q6IBK3\|Q6IBK3_HUMAN | SCAMP2 protein OS=Homo sapiens GN=SCAMP2 PE=2 SV=1 | tr\|A8K769\|A8K769_HUMAN;tr\|A0A140VK92\|A0A140VK92_HUMAN;sp\|O15127\|SCAM2_HUMAN | up | 1.7487 | 0.01592 | / | / | |
| 1570 | tr\|A0A140VJC9\|A0A140VJC9_HUMAN | Lysophospholipase II, isoform CRA_f OS=Homo sapiens GN=LYPLA2 PE=2 SV=1 | sp\|O95372\|LYPA2_HUMAN | up | 2.5771 | 0.04305 | General function prediction only ; | ko00564,Glycerophospholipid metabolism | |
| 1631 | sp\|Q8TDD1\|DDX54_HUMAN | ATP-dependent RNA helicase DDX54 OS=Homo sapiens GN=DDX54 PE=1 SV=2 | / | down | 0.6302 | 0.03878 | Replication, recombination and repair ; Transcription ; Translation, ribosomal structure and biogenesis ; |  |  |
| 1682 | tr\|Q53EL3\|Q53EL3_HUMAN | Tyrosine-protein kinase (Fragment) OS=Homo sapiens PE=2 SV=1 | tr\|B5BU52\|B5BU52_HUMAN;tr\|B2R6Q4\|B2R6Q4_HUMAN;tr\|A8K3B6\|A8K3B6_HUMAN;sp\|P41240\|CSK_HUMAN | down | 0.3851 | 0.03027 | General function prediction only ; Signal transduction mechanisms ; Transcription ; Replication, recombination and repair ; | ko04810,Regulation of actin cytoskeleton;ko04062,Chemokine signaling pathway;ko04722,Neurotrophin signaling pathway;ko05120,Epithelial cell signaling in Helicobacter pylori infection;ko05131,Shigellosis | |
| 1721 | tr\|A8K885\|A8K885_HUMAN | cDNA FLJ77179, highly similar to Homo sapiens sorting nexin 6 (SNX6) mRNA OS=Homo sapiens PE=2 SV=1 | tr\|A0A0A0MRI2\|A0A0A0MRI2_HUMAN;sp\|Q9UNH7\|SNX6_HUMAN | up | 6.9119 | 0.00583 | Intracellular trafficking, secretion, and vesicular transport ; General function prediction only ; |  |  |
| 1781 | tr\|A0A0A0MTC1\|A0A0A0MTC1_HUMAN | E3 ubiquitin-protein ligase RNF213 OS=Homo sapiens GN=RNF213 PE=1 SV=1 | tr\|A0A0A0MTR7\|A0A0A0MTR7_HUMAN;sp\|Q63HN8\|RN213_HUMAN | up | 3.1426 | 0.00311 | / | / | |
| 1801 | tr\|A0A0S2Z5I7\|A0A0S2Z5I7_HUMAN | Shwachman-Bodian-Diamond syndrome isoform 1 (Fragment) OS=Homo sapiens GN=SBDS PE=2 SV=1 | sp\|Q9Y3A5\|SBDS_HUMAN | down | 0.3208 | 0.02951 | Translation, ribosomal structure and biogenesis ; | / | |
| 1815 | tr\|E5KS95\|E5KS95_HUMAN | Elongation factor Ts, mitochondrial OS=Homo sapiens GN=TSFM PE=3 SV=1 | sp\|P43897\|EFTS_HUMAN | down | 0.6351 | 0.01645 | Translation, ribosomal structure and biogenesis ; | / | |
| 1863 | tr\|Q6FHR4\|Q6FHR4_HUMAN | Mitogen-activated protein kinase (Fragment) OS=Homo sapiens GN=MAPK13 PE=2 SV=1 | tr\|B5BTY5\|B5BTY5_HUMAN;tr\|A0A0S2Z542\|A0A0S2Z542_HUMAN;tr\|A0A024RD04\|A0A024RD04_HUMAN;sp\|O15264\|MK13_HUMAN | up | 3.8651 | 0.03465 | General function prediction only ; Signal transduction mechanisms ; Transcription ; Replication, recombination and repair ; | ko04370,VEGF signaling pathway;ko04621,NOD-like receptor signaling pathway;ko05142,Chagas disease;ko05160,Hepatitis C;ko04670,Leukocyte transendothelial migration;ko04380,Osteoclast differentiation;ko04660,T cell receptor signaling pathway;ko05145,Toxoplasmosis;ko05120,Epithelial cell signaling in Helicobacter pylori infection;ko04620,Toll-like receptor signaling pathway;ko04664,Fc epsilon RI signaling pathway;ko04914,Progesterone-mediated oocyte maturation;ko05131,Shigellosis;ko04011,MAPK signaling pathway - yeast;ko04622,RIG-I-like receptor signaling pathway;ko04912,GnRH signaling pathway;ko05140,Leishmaniasis;ko04010,MAPK signaling pathway;ko04722,Neurotrophin signaling pathway;ko05014,Amyotrophic lateral sclerosis (ALS) | |
| 1866 | tr\|F5GXF5\|F5GXF5_HUMAN | Nucleosome-remodeling factor subunit BPTF (Fragment) OS=Homo sapiens GN=BPTF PE=1 SV=2 | / | up | 2.8509 | 0.04076 | / | / | |
| 1893 | sp\|Q96JB5\|CK5P3_HUMAN | CDK5 regulatory subunit-associated protein 3 OS=Homo sapiens GN=CDK5RAP3 PE=1 SV=2 | / | up | 2.3330 | 0.04075 | / | / | |
| 1906 | sp\|O75190\|DNJB6_HUMAN | DnaJ homolog subfamily B member 6 OS=Homo sapiens GN=DNAJB6 PE=1 SV=2 | tr\|B4DVN1\|B4DVN1_HUMAN | down | 0.6148 | 0.04302 | Posttranslational modification, protein turnover, chaperones ; | / | |
| 2027 | tr\|Q53HA5\|Q53HA5_HUMAN | CDP-diacylglycerol--inositol 3-phosphatidyltransferase isoform 1 variant (Fragment) OS=Homo sapiens PE=2 SV=1 | tr\|A8K3L7\|A8K3L7_HUMAN;sp\|O14735\|CDIPT_HUMAN;tr\|B3KY94\|B3KY94_HUMAN;tr\|B3KSW0\|B3KSW0_HUMAN | up | 5.2520 | 0.03293 | Lipid transport and metabolism ; | ko00562,Inositol phosphate metabolism;ko00564,Glycerophospholipid metabolism;ko01100,Metabolic pathways;ko04070,Phosphatidylinositol signaling system | |
| 2149 | tr\|B3KSS4\|B3KSS4_HUMAN | cDNA FLJ36858 fis, clone ASTRO2015185, highly similar to POLIOVIRUS RECEPTOR OS=Homo sapiens PE=2 SV=1 | tr\|A8K4I1\|A8K4I1_HUMAN;tr\|A0A0C4DG49\|A0A0C4DG49_HUMAN;sp\|P15151\|PVR_HUMAN;tr\|A0A0A0MSA9\|A0A0A0MSA9_HUMAN | down | 0.5698 | 0.03322 | / | ko04514,Cell adhesion molecules (CAMs) | |
| 2154 | sp\|Q8NF37\|PCAT1_HUMAN | Lysophosphatidylcholine acyltransferase 1 OS=Homo sapiens GN=LPCAT1 PE=1 SV=2 | / | up | 4.5239 | 0.04074 | Lipid transport and metabolism ; | ko00565,Ether lipid metabolism;ko00564,Glycerophospholipid metabolism;ko01100,Metabolic pathways | |
| 2342 | tr\|A0A0S2Z3H6\|A0A0S2Z3H6_HUMAN | Cleft lip and palate associated transmembrane protein 1 isoform 1 (Fragment) OS=Homo sapiens GN=CLPTM1 PE=2 SV=1 | tr\|A0A0S2Z3H2\|A0A0S2Z3H2_HUMAN;sp\|O96005\|CLPT1_HUMAN | up | 2.6155 | 0.03930 | / | / | |
| 2382 | tr\|F8W9X7\|F8W9X7_HUMAN | Coiled-coil domain-containing protein 93 OS=Homo sapiens GN=CCDC93 PE=1 SV=1 | sp\|Q567U6\|CCD93_HUMAN | up | 5.0332 | 0.00556 | / | / | |
| 2420 | tr\|A0A140VJK1\|A0A140VJK1_HUMAN | Testicular tissue protein Li 75 OS=Homo sapiens PE=2 SV=1 | sp\|O76003\|GLRX3_HUMAN | up | 2.1556 | 0.04947 | Posttranslational modification, protein turnover, chaperones ; | / | |
| 2481 | sp\|Q9C0C9\|UBE2O_HUMAN | (E3-independent) E2 ubiquitin-conjugating enzyme OS=Homo sapiens GN=UBE2O PE=1 SV=3 | / | up | 2.2165 | 0.00413 | Posttranslational modification, protein turnover, chaperones ; | ko04120,Ubiquitin mediated proteolysis | |
| 2542 | tr\|A0A024R1I3\|A0A024R1I3_HUMAN | Pyridoxal (Pyridoxine, vitamin B6) phosphatase, isoform CRA_a OS=Homo sapiens GN=PDXP PE=2 SV=1 | sp\|Q96GD0\|PLPP_HUMAN | up | 1.9317 | 0.03401 | Carbohydrate transport and metabolism ; | ko00750,Vitamin B6 metabolism;ko01100,Metabolic pathways | |
| 2553 | sp\|O75648\|MTU1_HUMAN | Mitochondrial tRNA-specific 2-thiouridylase 1 OS=Homo sapiens GN=TRMU PE=1 SV=2 | / | up | 2.7472 | 0.03651 | Translation, ribosomal structure and biogenesis ; | ko04122,Sulfur relay system | |
| 2586 | sp\|Q9BTU6\|P4K2A_HUMAN | Phosphatidylinositol 4-kinase type 2-alpha OS=Homo sapiens GN=PI4K2A PE=1 SV=1 | / | up | 3.9303 | 0.00299 | / | ko00562,Inositol phosphate metabolism;ko01100,Metabolic pathways;ko04070,Phosphatidylinositol signaling system | |
| 2596 | sp\|Q99418\|CYH2_HUMAN | Cytohesin-2 OS=Homo sapiens GN=CYTH2 PE=1 SV=2 | / | up | 2.7462 | 0.02648 | General function prediction only ; | ko04144,Endocytosis | |
| 2744 | tr\|A8KAH1\|A8KAH1_HUMAN | cDNA FLJ75839, highly similar to Homo sapiens phosphatidylserine synthase 1 (PTDSS1), mRNA OS=Homo sapiens PE=2 SV=1 | sp\|P48651\|PTSS1_HUMAN | up | 5.4253 | 0.03856 | / | ko00564,Glycerophospholipid metabolism;ko01100,Metabolic pathways | |

**B. The differently expressed proteins comparing Δ*espF* to WT group.**

| **N** | **Accession** | **Name** | **Homologous proteins** | **Up or down regulated** | **Expression ratio** | **P value** | **COG function classification** | **Kegg function**  **enrichment** |  |
| --- | --- | --- | --- | --- | --- | --- | --- | --- | --- |
| 4 | sp\|Q7Z406\|MYH14_HUMAN | Myosin-14 OS=Homo sapiens GN=MYH14 PE=1 SV=2 | / | down | 0.5235 | 0.00006 | Cytoskeleton ; | ko04530,Tight junction;ko05416,Viral myocarditis | |
| 5 | sp\|O75369\|FLNB_HUMAN | Filamin-B OS=Homo sapiens GN=FLNB PE=1 SV=2 | / | down | 0.5276 | 0.00006 | Cytoskeleton ; | ko04010,MAPK signaling pathway;ko04510,Focal adhesion | |
| 6 | tr\|A0A024R1N1\|A0A024R1N1_HUMAN | Myosin, heavy polypeptide 9, non-muscle, isoform CRA_a OS=Homo sapiens GN=MYH9 PE=3 SV=1 | sp\|P35579\|MYH9_HUMAN | down | 0.5270 | 0.00035 | Cytoskeleton ; | ko04530,Tight junction;ko05416,Viral myocarditis | |
| 8 | sp\|P02545\|LMNA_HUMAN | Prelamin-A/C OS=Homo sapiens GN=LMNA PE=1 SV=1 | / | down | 0.3856 | 0.00000 | / | ko05410,Hypertrophic cardiomyopathy (HCM);ko05414,Dilated cardiomyopathy;ko05412,Arrhythmogenic right ventricular cardiomyopathy (ARVC) | |
| 9 | sp\|Q13813\|SPTN1_HUMAN | Spectrin alpha chain, non-erythrocytic 1 OS=Homo sapiens GN=SPTAN1 PE=1 SV=3 | / | down | 0.6138 | 0.00025 | Signal transduction mechanisms ; Cytoskeleton ; Cell cycle control, cell division, chromosome partitioning ; General function prediction only ; | / | |
| 12 | tr\|A0A024R4A0\|A0A024R4A0_HUMAN | Nucleolin, isoform CRA_b OS=Homo sapiens GN=NCL PE=4 SV=1 | sp\|P19338\|NUCL_HUMAN | down | 0.5123 | 0.00029 | General function prediction only ; | ko05130,Pathogenic Escherichia coli infection | |
| 15 | tr\|A0A0S2Z3G9\|A0A0S2Z3G9_HUMAN | Actinin alpha 4 isoform 1 (Fragment) OS=Homo sapiens GN=ACTN4 PE=2 SV=1 | sp\|O43707\|ACTN4_HUMAN | down | 0.5033 | 0.00024 | Signal transduction mechanisms ; Cytoskeleton ; Cell cycle control, cell division, chromosome partitioning ; General function prediction only ;Cytoskeleton ; | ko04520,Adherens junction;ko04530,Tight junction;ko04810,Regulation of actin cytoskeleton;ko04670,Leukocyte transendothelial migration;ko04510,Focal adhesion;ko05322,Systemic lupus erythematosus;ko05146,Amoebiasis;ko05412,Arrhythmogenic right ventricular cardiomyopathy (ARVC) | |
| 16 | tr\|A0A024RC65\|A0A024RC65_HUMAN | HCG1991735, isoform CRA_a OS=Homo sapiens GN=hCG_1991735 PE=4 SV=1 | sp\|P46940\|IQGA1_HUMAN | down | 0.6502 | 0.00943 | Cell cycle control, cell division, chromosome partitioning ; Signal transduction mechanisms ; | ko04810,Regulation of actin cytoskeleton | |
| 17 | sp\|Q14980\|NUMA1_HUMAN | Nuclear mitotic apparatus protein 1 OS=Homo sapiens GN=NUMA1 PE=1 SV=2 | / | down | 0.5490 | 0.00019 | / | / | |
| 18 | tr\|D6W5C0\|D6W5C0_HUMAN | Spectrin, beta, non-erythrocytic 1, isoform CRA_b OS=Homo sapiens GN=SPTBN1 PE=4 SV=1 | / | down | 0.5639 | 0.00046 | Cytoskeleton ; | / | |
| 19 | tr\|V9HW22\|V9HW22_HUMAN | Epididymis luminal protein 33 OS=Homo sapiens GN=HEL-S-72p PE=2 SV=1 | sp\|P11142\|HSP7C_HUMAN | down | 0.4734 | 0.00038 | Posttranslational modification, protein turnover, chaperones ; | ko05145,Toxoplasmosis;ko04144,Endocytosis;ko04010,MAPK signaling pathway;ko03040,Spliceosome;ko04612,Antigen processing and presentation;ko04141,Protein processing in endoplasmic reticulum | |
| 21 | tr\|A0A024RD80\|A0A024RD80_HUMAN | Heat shock protein 90kDa alpha (Cytosolic), class B member 1, isoform CRA_a OS=Homo sapiens GN=HSP90AB1 PE=3 SV=1 | sp\|P08238\|HS90B_HUMAN | down | 0.4667 | 0.00003 | Posttranslational modification, protein turnover, chaperones ; | ko04621,NOD-like receptor signaling pathway;ko05200,Pathways in cancer;ko04626,Plant-pathogen interaction;ko04612,Antigen processing and presentation;ko04914,Progesterone-mediated oocyte maturation;ko05215,Prostate cancer;ko04141,Protein processing in endoplasmic reticulum | |
| 26 | tr\|V9HWB4\|V9HWB4_HUMAN | Epididymis secretory sperm binding protein Li 89n OS=Homo sapiens GN=HEL-S-89n PE=2 SV=1 | sp\|P11021\|GRP78_HUMAN | down | 0.3515 | 0.00000 | Posttranslational modification, protein turnover, chaperones ; | ko03060,Protein export;ko05020,Prion diseases;ko04141,Protein processing in endoplasmic reticulum | |
| 27 | tr\|V9HVY3\|V9HVY3_HUMAN | Protein disulfide-isomerase OS=Homo sapiens GN=HEL-S-269 PE=2 SV=1 | sp\|P30101\|PDIA3_HUMAN | down | 0.5227 | 0.00000 | Posttranslational modification, protein turnover, chaperones ; Energy production and conversion ; | ko04612,Antigen processing and presentation;ko04141,Protein processing in endoplasmic reticulum | |
| 28 | tr\|A0A024R4F1\|A0A024R4F1_HUMAN | Enolase 1, (Alpha), isoform CRA_a OS=Homo sapiens GN=ENO1 PE=2 SV=1 | sp\|P06733\|ENOA_HUMAN | down | 0.4189 | 0.00055 | Carbohydrate transport and metabolism ; | ko00010,Glycolysis / Gluconeogenesis;ko01120,Microbial metabolism in diverse environments;ko01110,Biosynthesis of secondary metabolites;ko00680,Methane metabolism;ko01100,Metabolic pathways;ko03018,RNA degradation | |
| 31 | tr\|V9HWC0\|V9HWC0_HUMAN | Epididymis luminal protein 70 OS=Homo sapiens GN=HEL70 PE=2 SV=1 | sp\|P26038\|MOES_HUMAN | down | 0.6184 | 0.03577 | / | ko04810,Regulation of actin cytoskeleton;ko04670,Leukocyte transendothelial migration | |
| 32 | tr\|V9HWF4\|V9HWF4_HUMAN | Phosphoglycerate kinase OS=Homo sapiens GN=HEL-S-68p PE=2 SV=1 | sp\|P00558\|PGK1_HUMAN | down | 0.6599 | 0.00660 | Carbohydrate transport and metabolism ; | ko00010,Glycolysis / Gluconeogenesis;ko00710,Carbon fixation in photosynthetic organisms;ko01120,Microbial metabolism in diverse environments;ko01110,Biosynthesis of secondary metabolites;ko01100,Metabolic pathways | |
| 33 | sp\|P35900\|K1C20_HUMAN | Keratin, type I cytoskeletal 20 OS=Homo sapiens GN=KRT20 PE=1 SV=1 | / | down | 0.3708 | 0.00002 | / | / | |
| 34 | tr\|A0A090N8Y2\|A0A090N8Y2_HUMAN | Protein disulfide-isomerase A4 OS=Homo sapiens GN=ERP70 PE=2 SV=1 | sp\|P13667\|PDIA4_HUMAN | down | 0.4716 | 0.00003 | Posttranslational modification, protein turnover, chaperones ; Energy production and conversion ; | ko05110,Vibrio cholerae infection;ko04141,Protein processing in endoplasmic reticulum | |
| 36 | tr\|A8K5I0\|A8K5I0_HUMAN | Epididymis secretory protein Li 103 OS=Homo sapiens GN=HEL-S-103 PE=2 SV=1 | tr\|A0A0G2JIW1\|A0A0G2JIW1_HUMAN;sp\|P0DMV9\|HS71B_HUMAN;sp\|P0DMV8\|HS71A_HUMAN | down | 0.3831 | 0.00000 | Posttranslational modification, protein turnover, chaperones ; | ko05145,Toxoplasmosis;ko04144,Endocytosis;ko04010,MAPK signaling pathway;ko03040,Spliceosome;ko04612,Antigen processing and presentation;ko04141,Protein processing in endoplasmic reticulum | |
| 39 | tr\|A0A024R8S5\|A0A024R8S5_HUMAN | Protein disulfide-isomerase OS=Homo sapiens GN=P4HB PE=2 SV=1 | sp\|P07237\|PDIA1_HUMAN | down | 0.4953 | 0.00006 | Posttranslational modification, protein turnover, chaperones ; Energy production and conversion ; | ko04141,Protein processing in endoplasmic reticulum | |
| 41 | tr\|A7BI36\|A7BI36_HUMAN | p180/ribosome receptor OS=Homo sapiens GN=RRBP1 PE=2 SV=2 | / | down | 0.6047 | 0.00774 | Function unknown ;Cell motility ; | ko04141,Protein processing in endoplasmic reticulum | |
| 43 | tr\|A0A0S2Z4G4\|A0A0S2Z4G4_HUMAN | Tropomyosin 3 isoform 1 (Fragment) OS=Homo sapiens GN=TPM3 PE=2 SV=1 | / | down | 0.3140 | 0.02723 | / | ko04260,Cardiac muscle contraction;ko05410,Hypertrophic cardiomyopathy (HCM);ko05414,Dilated cardiomyopathy;ko05200,Pathways in cancer;ko05216,Thyroid cancer | |
| 45 | tr\|Q8N1C8\|Q8N1C8_HUMAN | HSPA9 protein (Fragment) OS=Homo sapiens GN=HSPA9 PE=2 SV=1 | / | down | 0.4777 | 0.00255 | Posttranslational modification, protein turnover, chaperones ; | ko03018,RNA degradation | |
| 46 | tr\|A0A024R9C1\|A0A024R9C1_HUMAN | Polyadenylate-binding protein OS=Homo sapiens GN=PABPC1 PE=3 SV=1 | sp\|P11940\|PABP1_HUMAN | down | 0.4166 | 0.01072 | General function prediction only ; | ko03015,mRNA surveillance pathway;ko03013,RNA transport | |
| 51 | tr\|Q53EM5\|Q53EM5_HUMAN | Transketolase (Fragment) OS=Homo sapiens PE=2 SV=1 | tr\|V9HWD9\|V9HWD9_HUMAN;sp\|P29401\|TKT_HUMAN | down | 0.3688 | 0.00148 | Carbohydrate transport and metabolism ; | ko00710,Carbon fixation in photosynthetic organisms;ko01120,Microbial metabolism in diverse environments;ko01110,Biosynthesis of secondary metabolites;ko01051,Biosynthesis of ansamycins;ko00030,Pentose phosphate pathway;ko01100,Metabolic pathways | |
| 55 | tr\|E7EQR4\|E7EQR4_HUMAN | Ezrin OS=Homo sapiens GN=EZR PE=1 SV=3 | sp\|P15311\|EZRI_HUMAN | down | 0.4535 | 0.00551 | / | ko04810,Regulation of actin cytoskeleton;ko04670,Leukocyte transendothelial migration;ko05130,Pathogenic Escherichia coli infection;ko04971,Gastric acid secretion | |
| 58 | tr\|V9HVZ4\|V9HVZ4_HUMAN | Glyceraldehyde-3-phosphate dehydrogenase OS=Homo sapiens GN=HEL-S-162eP PE=2 SV=1 | sp\|P04406\|G3P_HUMAN | down | 0.4997 | 0.00037 | Carbohydrate transport and metabolism ; | ko05010,Alzheimer's disease;ko00010,Glycolysis / Gluconeogenesis;ko01120,Microbial metabolism in diverse environments;ko01110,Biosynthesis of secondary metabolites;ko01100,Metabolic pathways | |
| 60 | sp\|P35221\|CTNA1_HUMAN | Catenin alpha-1 OS=Homo sapiens GN=CTNNA1 PE=1 SV=1 | tr\|B4E2G8\|B4E2G8_HUMAN | down | 0.3720 | 0.00578 | / | ko04520,Adherens junction;ko04530,Tight junction;ko05213,Endometrial cancer;ko05100,Bacterial invasion of epithelial cells;ko05200,Pathways in cancer;ko04670,Leukocyte transendothelial migration;ko05412,Arrhythmogenic right ventricular cardiomyopathy (ARVC) | |
| 61 | tr\|B2ZZ90\|B2ZZ90_HUMAN | Acetyl-Coenzyme A carboxylase alpha OS=Homo sapiens GN=ACACA PE=2 SV=1 | sp\|Q13085\|ACACA_HUMAN | down | 0.4810 | 0.00653 | Lipid transport and metabolism ; | ko04910,Insulin signaling pathway;ko00061,Fatty acid biosynthesis;ko01100,Metabolic pathways;ko00640,Propanoate metabolism;ko00620,Pyruvate metabolism | |
| 63 | sp\|P60174\|TPIS_HUMAN | Triosephosphate isomerase OS=Homo sapiens GN=TPI1 PE=1 SV=3 | / | down | 0.5545 | 0.01111 | Carbohydrate transport and metabolism ; | ko00051,Fructose and mannose metabolism;ko00562,Inositol phosphate metabolism;ko00010,Glycolysis / Gluconeogenesis;ko00710,Carbon fixation in photosynthetic organisms;ko01120,Microbial metabolism in diverse environments;ko01110,Biosynthesis of secondary metabolites;ko01100,Metabolic pathways | |
| 64 | tr\|Q6FHZ0\|Q6FHZ0_HUMAN | Malate dehydrogenase OS=Homo sapiens GN=MDH2 PE=2 SV=1 | tr\|A0A024R4K3\|A0A024R4K3_HUMAN;sp\|P40926\|MDHM_HUMAN;tr\|Q75MT9\|Q75MT9_HUMAN | down | 0.4930 | 0.00000 | Energy production and conversion ; | ko00630,Glyoxylate and dicarboxylate metabolism;ko00710,Carbon fixation in photosynthetic organisms;ko01120,Microbial metabolism in diverse environments;ko00020,Citrate cycle (TCA cycle);ko01110,Biosynthesis of secondary metabolites;ko01100,Metabolic pathways;ko00620,Pyruvate metabolism | |
| 67 | sp\|P04843\|RPN1_HUMAN | Dolichyl-diphosphooligosaccharide--protein glycosyltransferase subunit 1 OS=Homo sapiens GN=RPN1 PE=1 SV=1 | / | down | 0.4487 | 0.00045 | / | ko01100,Metabolic pathways;ko00510,N-Glycan biosynthesis;ko04141,Protein processing in endoplasmic reticulum | |
| 70 | tr\|E9KL48\|E9KL48_HUMAN | Epididymis tissue sperm binding protein Li 18mP OS=Homo sapiens GN=GLUD1 PE=2 SV=1 | sp\|P00367\|DHE3_HUMAN | down | 0.3821 | 0.00001 | Amino acid transport and metabolism ; | ko00471,D-Glutamine and D-glutamate metabolism;ko04964,Proximal tubule bicarbonate reclamation;ko00250,Alanine, aspartate and glutamate metabolism;ko00910,Nitrogen metabolism;ko01100,Metabolic pathways;ko00330,Arginine and proline metabolism | |
| 74 | tr\|B4E0X8\|B4E0X8_HUMAN | cDNA FLJ61021, highly similar to Far upstream element-binding protein 1 OS=Homo sapiens PE=2 SV=1 | / | down | 0.4100 | 0.01523 | / | / | |
| 77 | tr\|V9HW26\|V9HW26_HUMAN | ATP synthase subunit alpha OS=Homo sapiens GN=HEL-S-123m PE=2 SV=1 | sp\|P25705\|ATPA_HUMAN | down | 0.3834 | 0.00288 | Energy production and conversion ; | ko05010,Alzheimer's disease;ko00190,Oxidative phosphorylation;ko05016,Huntington's disease;ko05012,Parkinson's disease;ko01100,Metabolic pathways | |
| 79 | tr\|Q53HM9\|Q53HM9_HUMAN | Eukaryotic translation elongation factor 1 alpha 1 variant (Fragment) OS=Homo sapiens PE=2 SV=1 | tr\|Q6IPT9\|Q6IPT9_HUMAN;tr\|Q6IPS9\|Q6IPS9_HUMAN;sp\|P68104\|EF1A1_HUMAN | down | 0.3870 | 0.01150 | Translation, ribosomal structure and biogenesis ; | ko03013,RNA transport | |
| 85 | tr\|V9HW29\|V9HW29_HUMAN | Kinesin-like protein OS=Homo sapiens GN=HEL-S-61 PE=2 SV=1 | sp\|P33176\|KINH_HUMAN | down | 0.5951 | 0.00469 | Cytoskeleton ; | / | |
| 86 | sp\|Q14204\|DYHC1_HUMAN | Cytoplasmic dynein 1 heavy chain 1 OS=Homo sapiens GN=DYNC1H1 PE=1 SV=5 | / | down | 0.5852 | 0.01761 | Cytoskeleton ; | ko04962,Vasopressin-regulated water reabsorption;ko04145,Phagosome | |
| 92 | sp\|Q02790\|FKBP4_HUMAN | Peptidyl-prolyl cis-trans isomerase FKBP4 OS=Homo sapiens GN=FKBP4 PE=1 SV=3 | / | down | 0.4356 | 0.00019 | Posttranslational modification, protein turnover, chaperones ; | / | |
| 93 | tr\|V9HWB9\|V9HWB9_HUMAN | L-lactate dehydrogenase OS=Homo sapiens GN=HEL-S-133P PE=2 SV=1 | sp\|P00338\|LDHA_HUMAN | down | 0.3291 | 0.00031 | Energy production and conversion ; | ko00010,Glycolysis / Gluconeogenesis;ko01120,Microbial metabolism in diverse environments;ko00270,Cysteine and methionine metabolism;ko01110,Biosynthesis of secondary metabolites;ko01100,Metabolic pathways;ko00640,Propanoate metabolism;ko00620,Pyruvate metabolism | |
| 101 | tr\|Q59ER5\|Q59ER5_HUMAN | WD repeat-containing protein 1 isoform 1 variant (Fragment) OS=Homo sapiens PE=2 SV=1 | / | down | 0.5834 | 0.01501 | General function prediction only ; | ko03040,Spliceosome;ko03018,RNA degradation | |
| 102 | tr\|V9HW31\|V9HW31_HUMAN | ATP synthase subunit beta OS=Homo sapiens GN=HEL-S-271 PE=2 SV=1 | sp\|P06576\|ATPB_HUMAN | down | 0.3754 | 0.00007 | Energy production and conversion ; | ko05010,Alzheimer's disease;ko00190,Oxidative phosphorylation;ko05016,Huntington's disease;ko05012,Parkinson's disease;ko01100,Metabolic pathways | |
| 107 | sp\|P11413\|G6PD_HUMAN | Glucose-6-phosphate 1-dehydrogenase OS=Homo sapiens GN=G6PD PE=1 SV=4 | / | down | 0.5523 | 0.02698 | Carbohydrate transport and metabolism ; | ko00480,Glutathione metabolism;ko01120,Microbial metabolism in diverse environments;ko01110,Biosynthesis of secondary metabolites;ko00030,Pentose phosphate pathway;ko01100,Metabolic pathways | |
| 111 | tr\|A0A0A0MTS2\|A0A0A0MTS2_HUMAN | Glucose-6-phosphate isomerase (Fragment) OS=Homo sapiens GN=GPI PE=1 SV=1 | sp\|P06744\|G6PI_HUMAN | down | 0.6426 | 0.00182 | Carbohydrate transport and metabolism ; | ko00010,Glycolysis / Gluconeogenesis;ko01120,Microbial metabolism in diverse environments;ko01110,Biosynthesis of secondary metabolites;ko00520,Amino sugar and nucleotide sugar metabolism;ko00500,Starch and sucrose metabolism;ko00030,Pentose phosphate pathway;ko01100,Metabolic pathways | |
| 117 | sp\|Q99623\|PHB2_HUMAN | Prohibitin-2 OS=Homo sapiens GN=PHB2 PE=1 SV=2 | / | down | 0.6027 | 0.02895 | Posttranslational modification, protein turnover, chaperones ; | / | |
| 119 | sp\|P22626\|ROA2_HUMAN | Heterogeneous nuclear ribonucleoproteins A2/B1 OS=Homo sapiens GN=HNRNPA2B1 PE=1 SV=2 | / | down | 0.4653 | 0.00195 | General function prediction only ; | / | |
| 120 | tr\|V9HW37\|V9HW37_HUMAN | Epididymis secretory protein Li 69 OS=Homo sapiens GN=HEL-S-69 PE=2 SV=1 | sp\|P48643\|TCPE_HUMAN | down | 0.5113 | 0.04750 | Posttranslational modification, protein turnover, chaperones ; | / | |
| 121 | tr\|A0A024R8V0\|A0A024R8V0_HUMAN | Septin 9, isoform CRA_a OS=Homo sapiens GN=SEPT9 PE=3 SV=1 | / | down | 0.5028 | 0.03293 | Cell cycle control, cell division, chromosome partitioning ; Cytoskeleton ; | ko05012,Parkinson's disease | |
| 122 | sp\|Q15393\|SF3B3_HUMAN | Splicing factor 3B subunit 3 OS=Homo sapiens GN=SF3B3 PE=1 SV=4 | / | down | 0.5234 | 0.03019 | / | ko03040,Spliceosome | |
| 125 | tr\|V9HWG3\|V9HWG3_HUMAN | Epididymis secretory protein Li 45 OS=Homo sapiens GN=HEL-S-45 PE=2 SV=1 | sp\|P21980\|TGM2_HUMAN | down | 0.4639 | 0.00152 | / | ko05016,Huntington's disease | |
| 126 | tr\|K9JA46\|K9JA46_HUMAN | Epididymis luminal secretory protein 52 OS=Homo sapiens GN=EL52 PE=2 SV=1 | sp\|P07900\|HS90A_HUMAN | down | 0.4652 | 0.00003 | Posttranslational modification, protein turnover, chaperones ; | ko04621,NOD-like receptor signaling pathway;ko05200,Pathways in cancer;ko04626,Plant-pathogen interaction;ko04612,Antigen processing and presentation;ko04914,Progesterone-mediated oocyte maturation;ko05215,Prostate cancer;ko04141,Protein processing in endoplasmic reticulum | |
| 127 | sp\|P50991\|TCPD_HUMAN | T-complex protein 1 subunit delta OS=Homo sapiens GN=CCT4 PE=1 SV=4 | / | down | 0.6137 | 0.00603 | Posttranslational modification, protein turnover, chaperones ; | / | |
| 131 | tr\|V9HWF5\|V9HWF5_HUMAN | Peptidyl-prolyl cis-trans isomerase OS=Homo sapiens GN=HEL-S-69p PE=2 SV=1 | sp\|P62937\|PPIA_HUMAN | down | 0.3778 | 0.00129 | Posttranslational modification, protein turnover, chaperones ; | / | |
| 135 | sp\|P14866\|HNRPL_HUMAN | Heterogeneous nuclear ribonucleoprotein L OS=Homo sapiens GN=HNRNPL PE=1 SV=2 | / | down | 0.3137 | 0.00036 | / | / | |
| 136 | tr\|Q53YD7\|Q53YD7_HUMAN | EEF1G protein OS=Homo sapiens GN=EEF1G PE=2 SV=1 | sp\|P26641\|EF1G_HUMAN | down | 0.4647 | 0.00417 | Posttranslational modification, protein turnover, chaperones ; | / | |
| 145 | sp\|P13010\|XRCC5_HUMAN | X-ray repair cross-complementing protein 5 OS=Homo sapiens GN=XRCC5 PE=1 SV=3 | / | down | 0.3993 | 0.03142 | / | ko03450,Non-homologous end-joining | |
| 146 | tr\|F4ZW66\|F4ZW66_HUMAN | NF110b OS=Homo sapiens PE=2 SV=1 | / | down | 0.5869 | 0.00413 | / | / | |
| 149 | tr\|B2R4R0\|B2R4R0_HUMAN | Histone H4 OS=Homo sapiens GN=HIST1H4L PE=2 SV=1 | sp\|P62805\|H4_HUMAN | down | 0.3112 | 0.00001 | Chromatin structure and dynamics ; | ko05322,Systemic lupus erythematosus | |
| 151 | sp\|P49411\|EFTU_HUMAN | Elongation factor Tu, mitochondrial OS=Homo sapiens GN=TUFM PE=1 SV=2 | / | down | 0.6124 | 0.00597 | Translation, ribosomal structure and biogenesis ; | ko04626,Plant-pathogen interaction | |
| 153 | sp\|Q13263\|TIF1B_HUMAN | Transcription intermediary factor 1-beta OS=Homo sapiens GN=TRIM28 PE=1 SV=5 | / | down | 0.4394 | 0.00012 | / | / | |
| 155 | tr\|B2R491\|B2R491_HUMAN | 40S ribosomal protein S4 OS=Homo sapiens GN=RPS4X PE=2 SV=1 | sp\|P62701\|RS4X_HUMAN;tr\|Q96IR1\|Q96IR1_HUMAN | down | 0.5629 | 0.00032 | Translation, ribosomal structure and biogenesis ; | ko03010,Ribosome | |
| 156 | sp\|O75643\|U520_HUMAN | U5 small nuclear ribonucleoprotein 200 kDa helicase OS=Homo sapiens GN=SNRNP200 PE=1 SV=2 | / | down | 0.5144 | 0.00153 | General function prediction only ; | ko03040,Spliceosome | |
| 157 | tr\|Q53GX7\|Q53GX7_HUMAN | Threonyl-tRNA synthetase variant (Fragment) OS=Homo sapiens PE=2 SV=1 | sp\|P26639\|SYTC_HUMAN | down | 0.6038 | 0.04570 | Translation, ribosomal structure and biogenesis ; | ko00970,Aminoacyl-tRNA biosynthesis | |
| 158 | sp\|Q15084\|PDIA6_HUMAN | Protein disulfide-isomerase A6 OS=Homo sapiens GN=PDIA6 PE=1 SV=1 | / | down | 0.6432 | 0.02389 | Posttranslational modification, protein turnover, chaperones ; Energy production and conversion ; | ko04141,Protein processing in endoplasmic reticulum | |
| 159 | sp\|Q16531\|DDB1_HUMAN | DNA damage-binding protein 1 OS=Homo sapiens GN=DDB1 PE=1 SV=1 | / | down | 0.6528 | 0.00180 | / | ko04120,Ubiquitin mediated proteolysis;ko03420,Nucleotide excision repair | |
| 165 | sp\|Q06830\|PRDX1_HUMAN | Peroxiredoxin-1 OS=Homo sapiens GN=PRDX1 PE=1 SV=1 | / | down | 0.3406 | 0.01739 | Posttranslational modification, protein turnover, chaperones ; | ko04146,Peroxisome | |
| 169 | tr\|V9HWI5\|V9HWI5_HUMAN | Cofilin 1 (Non-muscle), isoform CRA_b OS=Homo sapiens GN=HEL-S-15 PE=2 SV=1 | sp\|P23528\|COF1_HUMAN;tr\|E9PK25\|E9PK25_HUMAN | down | 0.3455 | 0.00108 | / | ko04360,Axon guidance;ko04810,Regulation of actin cytoskeleton;ko04666,Fc gamma R-mediated phagocytosis | |
| 170 | tr\|Q59EK6\|Q59EK6_HUMAN | TNF receptor-associated protein 1 variant (Fragment) OS=Homo sapiens PE=3 SV=1 | tr\|A0A140VJY2\|A0A140VJY2_HUMAN;sp\|Q12931\|TRAP1_HUMAN | down | 0.5330 | 0.00213 | Posttranslational modification, protein turnover, chaperones ; | / | |
| 175 | tr\|Q5U077\|Q5U077_HUMAN | L-lactate dehydrogenase OS=Homo sapiens GN=LDHB PE=2 SV=1 | sp\|P07195\|LDHB_HUMAN | down | 0.4868 | 0.00010 | Energy production and conversion ; | ko00010,Glycolysis / Gluconeogenesis;ko01120,Microbial metabolism in diverse environments;ko00270,Cysteine and methionine metabolism;ko01110,Biosynthesis of secondary metabolites;ko01100,Metabolic pathways;ko00640,Propanoate metabolism;ko00620,Pyruvate metabolism | |
| 182 | tr\|A0A140VK56\|A0A140VK56_HUMAN | Transaldolase OS=Homo sapiens PE=2 SV=1 | sp\|P37837\|TALDO_HUMAN | down | 0.4226 | 0.00004 | Carbohydrate transport and metabolism ; | ko01120,Microbial metabolism in diverse environments;ko01110,Biosynthesis of secondary metabolites;ko00030,Pentose phosphate pathway;ko01100,Metabolic pathways | |
| 184 | tr\|A2RUM7\|A2RUM7_HUMAN | Ribosomal protein L5 OS=Homo sapiens GN=RPL5 PE=2 SV=1 | sp\|P46777\|RL5_HUMAN | down | 0.5143 | 0.01558 | Translation, ribosomal structure and biogenesis ; | ko03010,Ribosome | |
| 189 | tr\|B0YIW6\|B0YIW6_HUMAN | Archain 1, isoform CRA_a OS=Homo sapiens GN=ARCN1 PE=1 SV=1 | tr\|B0YIW5\|B0YIW5_HUMAN;sp\|P48444\|COPD_HUMAN | down | 0.5835 | 0.02609 | / | / | |
| 191 | sp\|Q9NY33\|DPP3_HUMAN | Dipeptidyl peptidase 3 OS=Homo sapiens GN=DPP3 PE=1 SV=2 | / | down | 0.4971 | 0.00466 | / | / | |
| 195 | tr\|B4DLV7\|B4DLV7_HUMAN | cDNA FLJ60299, highly similar to Rab GDP dissociation inhibitor beta OS=Homo sapiens PE=2 SV=1 | / | down | 0.2834 | 0.01000 | Posttranslational modification, protein turnover, chaperones ; | / | |
| 196 | tr\|V9HW69\|V9HW69_HUMAN | Epididymis secretory protein Li 66 OS=Homo sapiens GN=HEL-S-66 PE=2 SV=1 | tr\|B4DU58\|B4DU58_HUMAN;tr\|B2R9S4\|B2R9S4_HUMAN;sp\|P40121\|CAPG_HUMAN | down | 0.6394 | 0.01593 | / | / | |
| 200 | sp\|Q15019\|SEPT2_HUMAN | Septin-2 OS=Homo sapiens GN=SEPT2 PE=1 SV=1 |  | down | 0.5765 | 0.02002 | Cell cycle control, cell division, chromosome partitioning ; Cytoskeleton ; | ko05012,Parkinson's disease | |
| 203 | tr\|Q6FHG5\|Q6FHG5_HUMAN | Gamma-synuclein OS=Homo sapiens GN=SNCG PE=2 SV=1 | sp\|O76070\|SYUG_HUMAN | down | 0.5043 | 0.00176 | / | ko05010,Alzheimer's disease;ko05012,Parkinson's disease | |
| 204 | tr\|B2RDW1\|B2RDW1_HUMAN | Epididymis luminal protein 112 OS=Homo sapiens GN=RPS27A PE=2 SV=1 | sp\|P62979\|RS27A_HUMAN | down | 0.3016 | 0.01458 | Posttranslational modification, protein turnover, chaperones ;Translation, ribosomal structure and biogenesis ; | ko03010,Ribosome | |
| 214 | tr\|A0A024RAZ7\|A0A024RAZ7_HUMAN | Heterogeneous nuclear ribonucleoprotein A1, isoform CRA_b OS=Homo sapiens GN=HNRPA1 PE=4 SV=1 | sp\|P09651\|ROA1_HUMAN | down | 0.6225 | 0.00262 | General function prediction only ; | ko03040,Spliceosome | |
| 215 | sp\|P23284\|PPIB_HUMAN | Peptidyl-prolyl cis-trans isomerase B OS=Homo sapiens GN=PPIB PE=1 SV=2 | tr\|V9HWC6\|V9HWC6_HUMAN | down | 0.4174 | 0.00023 | Posttranslational modification, protein turnover, chaperones ; | / | |
| 216 | tr\|V9HW83\|V9HW83_HUMAN | Aldehyde dehydrogenase 1 family, member A1, isoform CRA_a OS=Homo sapiens GN=HEL-S-53e PE=2 SV=1 | tr\|V9HVX6\|V9HVX6_HUMAN;sp\|P00352\|AL1A1_HUMAN | down | 0.5169 | 0.04834 | Energy production and conversion ; | ko00830,Retinol metabolism;ko01100,Metabolic pathways | |
| 221 | sp\|P17987\|TCPA_HUMAN | T-complex protein 1 subunit alpha OS=Homo sapiens GN=TCP1 PE=1 SV=1 | / | down | 0.5679 | 0.00604 | Posttranslational modification, protein turnover, chaperones ; | / | |
| 222 | tr\|B4E266\|B4E266_HUMAN | cDNA FLJ58466, highly similar to Leucyl-tRNA synthetase, cytoplasmic (EC 6.1.1.4) OS=Homo sapiens PE=2 SV=1 | / | down | 0.6073 | 0.03070 | Translation, ribosomal structure and biogenesis ; | ko00290,Valine, leucine and isoleucine biosynthesis;ko00970,Aminoacyl-tRNA biosynthesis | |
| 224 | tr\|V9HWH1\|V9HWH1_HUMAN | Epididymis luminal protein 57 OS=Homo sapiens GN=HEL57 PE=2 SV=1 | tr\|B4E3A8\|B4E3A8_HUMAN;sp\|P30740\|ILEU_HUMAN | down | 0.5980 | 0.04241 | Posttranslational modification, protein turnover, chaperones ; | ko05146,Amoebiasis | |
| 225 | tr\|V9HW98\|V9HW98_HUMAN | Epididymis luminal protein 2 OS=Homo sapiens GN=HEL2 PE=2 SV=1 | sp\|P62258\|1433E_HUMAN | down | 0.4835 | 0.00890 | Signal transduction mechanisms ; | ko04114,Oocyte meiosis;ko04722,Neurotrophin signaling pathway;ko04110,Cell cycle | |
| 226 | tr\|Q53G72\|Q53G72_HUMAN | B-cell receptor-associated protein 31 variant (Fragment) OS=Homo sapiens PE=2 SV=1 | sp\|P51572\|BAP31_HUMAN | down | 0.4134 | 0.00101 | Function unknown ; | ko04141,Protein processing in endoplasmic reticulum | |
| 232 | sp\|P62081\|RS7_HUMAN | 40S ribosomal protein S7 OS=Homo sapiens GN=RPS7 PE=1 SV=1 | / | down | 0.6498 | 0.03225 | / | ko03010,Ribosome | |
| 234 | tr\|Q5U0I6\|Q5U0I6_HUMAN | H.sapiens ras-related Hrab1A protein OS=Homo sapiens GN=RAB1A PE=2 SV=1 | sp\|P62820\|RAB1A_HUMAN | down | 0.5486 | 0.02160 | General function prediction only ; | / | |
| 237 | tr\|B5BUB5\|B5BUB5_HUMAN | Autoantigen La (Fragment) OS=Homo sapiens GN=SSB PE=2 SV=1 | sp\|P05455\|LA_HUMAN | down | 0.4614 | 0.00773 | Posttranslational modification, protein turnover, chaperones ; Translation, ribosomal structure and biogenesis ; | ko05322,Systemic lupus erythematosus | |
| 241 | tr\|A0A024R814\|A0A024R814_HUMAN | Ribosomal protein L7, isoform CRA_a OS=Homo sapiens GN=RPL7 PE=4 SV=1 | sp\|P18124\|RL7_HUMAN | down | 0.6611 | 0.04625 | Translation, ribosomal structure and biogenesis ; | ko03010,Ribosome | |
| 244 | sp\|Q8NBS9\|TXND5_HUMAN | Thioredoxin domain-containing protein 5 OS=Homo sapiens GN=TXNDC5 PE=1 SV=2 | / | down | 0.5243 | 0.03685 | Posttranslational modification, protein turnover, chaperones ; Energy production and conversion ; | ko04141,Protein processing in endoplasmic reticulum | |
| 250 | sp\|Q14683\|SMC1A_HUMAN | Structural maintenance of chromosomes protein 1A OS=Homo sapiens GN=SMC1A PE=1 SV=2 | / | down | 0.5509 | 0.00864 | Cell cycle control, cell division, chromosome partitioning ; | ko04114,Oocyte meiosis;ko04113,Meiosis - yeast;ko04111,Cell cycle - yeast;ko04110,Cell cycle | |
| 254 | tr\|Q8N9M2\|Q8N9M2_HUMAN | cDNA FLJ36887 fis, clone BNGH42005504, highly similar to 26S PROTEASOME REGULATORY SUBUNIT S3 OS=Homo sapiens PE=2 SV=1 | sp\|O43242\|PSMD3_HUMAN;tr\|B3KNN7\|B3KNN7_HUMAN | down | 0.6228 | 0.00053 | / | ko03050,Proteasome | |
| 256 | tr\|B5MDF5\|B5MDF5_HUMAN | GTP-binding nuclear protein Ran OS=Homo sapiens GN=RAN PE=1 SV=1 | sp\|P62826\|RAN_HUMAN | down | 0.3511 | 0.01392 | General function prediction only ; | ko03013,RNA transport | |
| 261 | tr\|A0A024QZ30\|A0A024QZ30_HUMAN | Succinate dehydrogenase [ubiquinone] flavoprotein subunit, mitochondrial OS=Homo sapiens GN=SDHA PE=3 SV=1 | sp\|P31040\|SDHA_HUMAN | down | 0.5800 | 0.04517 | Energy production and conversion ; | ko05010,Alzheimer's disease;ko00190,Oxidative phosphorylation;ko00020,Citrate cycle (TCA cycle);ko01110,Biosynthesis of secondary metabolites;ko05016,Huntington's disease;ko05012,Parkinson's disease;ko01100,Metabolic pathways | |
| 265 | sp\|P37802\|TAGL2_HUMAN | Transgelin-2 OS=Homo sapiens GN=TAGLN2 PE=1 SV=3 | / | down | 0.3372 | 0.01303 | Cytoskeleton ; | / | |
| 275 | tr\|Q6PUJ7\|Q6PUJ7_HUMAN | Epididymis luminal protein 215 OS=Homo sapiens GN=HEL-215 PE=2 SV=1 | tr\|A8K401\|A8K401_HUMAN;sp\|P35232\|PHB_HUMAN | down | 0.5464 | 0.01270 | Posttranslational modification, protein turnover, chaperones ; | / | |
| 281 | tr\|A0A024R713\|A0A024R713_HUMAN | Dihydrolipoyl dehydrogenase OS=Homo sapiens GN=DLD PE=4 SV=1 | sp\|P09622\|DLDH_HUMAN;tr\|E9PEX6\|E9PEX6_HUMAN;tr\|B4DMK9\|B4DMK9_HUMAN | down | 0.5870 | 0.04212 | Energy production and conversion ; | ko01120,Microbial metabolism in diverse environments;ko00280,Valine, leucine and isoleucine degradation;ko01110,Biosynthesis of secondary metabolites;ko01100,Metabolic pathways;ko00620,Pyruvate metabolism;ko00010,Glycolysis / Gluconeogenesis;ko00020,Citrate cycle (TCA cycle);ko00260,Glycine, serine and threonine metabolism | |
| 283 | tr\|A8K7B7\|A8K7B7_HUMAN | Protein phosphatase 2 (Formerly 2A), regulatory subunit A (PR 65), alpha isoform OS=Homo sapiens GN=PPP2R1A PE=1 SV=1 | tr\|A8K3H8\|A8K3H8_HUMAN;sp\|P30153\|2AAA_HUMAN | down | 0.4283 | 0.04750 | / | ko04530,Tight junction;ko05142,Chagas disease;ko04350,TGF-beta signaling pathway;ko04113,Meiosis - yeast;ko05160,Hepatitis C;ko03015,mRNA surveillance pathway;ko04730,Long-term depression;ko04310,Wnt signaling pathway;ko04114,Oocyte meiosis;ko04111,Cell cycle - yeast | |
| 284 | tr\|Q5JR94\|Q5JR94_HUMAN | 40S ribosomal protein S8 OS=Homo sapiens GN=RPS8 PE=2 SV=1 | sp\|P62241\|RS8_HUMAN | down | 0.4456 | 0.00103 | Translation, ribosomal structure and biogenesis ; | ko03010,Ribosome | |
| 287 | tr\|E9KL35\|E9KL35_HUMAN | Epididymis tissue sperm binding protein Li 3a OS=Homo sapiens PE=1 SV=1 | sp\|P63244\|RACK1_HUMAN | down | 0.5158 | 0.01119 | General function prediction only ; | ko00565,Ether lipid metabolism;ko01100,Metabolic pathways | |
| 300 | sp\|P07737\|PROF1_HUMAN | Profilin-1 OS=Homo sapiens GN=PFN1 PE=1 SV=2 | / | down | 0.2997 | 0.00016 | / | ko04810,Regulation of actin cytoskeleton;ko05131,Shigellosis | |
| 301 | tr\|A0A024R1K8\|A0A024R1K8_HUMAN | Splicing factor 3a, subunit 1, 120kDa, isoform CRA_a OS=Homo sapiens GN=SF3A1 PE=4 SV=1 | sp\|Q15459\|SF3A1_HUMAN | down | 0.5744 | 0.00628 | / | ko03040,Spliceosome | |
| 303 | tr\|A0A0S2Z4N8\|A0A0S2Z4N8_HUMAN | Vasodilator-stimulated phosphoprotein isoform 2 (Fragment) OS=Homo sapiens GN=VASP PE=2 SV=1 | tr\|A0A024R0V4\|A0A024R0V4_HUMAN;sp\|P50552\|VASP_HUMAN;tr\|A0A0S2Z4I9\|A0A0S2Z4I9_HUMAN | down | 0.5131 | 0.00120 | / | ko04670,Leukocyte transendothelial migration;ko04666,Fc gamma R-mediated phagocytosis;ko04510,Focal adhesion | |
| 309 | tr\|A0A024R1S8\|A0A024R1S8_HUMAN | LIM and SH3 protein 1, isoform CRA_b OS=Homo sapiens GN=LASP1 PE=4 SV=1 | sp\|Q14847\|LASP1_HUMAN | down | 0.6199 | 0.03401 | / | ko04530,Tight junction;ko04144,Endocytosis;ko05100,Bacterial invasion of epithelial cells;ko05130,Pathogenic Escherichia coli infection;ko05131,Shigellosis | |
| 312 | sp\|P25786\|PSA1_HUMAN | Proteasome subunit alpha type-1 OS=Homo sapiens GN=PSMA1 PE=1 SV=1 | / | down | 0.6543 | 0.04864 | Posttranslational modification, protein turnover, chaperones ; | ko03050,Proteasome | |
| 315 | sp\|P36578\|RL4_HUMAN | 60S ribosomal protein L4 OS=Homo sapiens GN=RPL4 PE=1 SV=5 | / | down | 0.4299 | 0.01073 | Translation, ribosomal structure and biogenesis ; | ko03010,Ribosome | |
| 325 | tr\|Q8WVX7\|Q8WVX7_HUMAN | Ribosomal protein S19 (Fragment) OS=Homo sapiens PE=2 SV=1 | tr\|B0ZBD0\|B0ZBD0_HUMAN;sp\|P39019\|RS19_HUMAN | down | 0.6045 | 0.03602 | Translation, ribosomal structure and biogenesis ; | ko03010,Ribosome | |
| 326 | sp\|Q15717\|ELAV1_HUMAN | ELAV-like protein 1 OS=Homo sapiens GN=ELAVL1 PE=1 SV=2 | / | down | 0.4393 | 0.00708 | General function prediction only ; | / | |
| 327 | tr\|G8JLA2\|G8JLA2_HUMAN | Myosin light polypeptide 6 OS=Homo sapiens GN=MYL6 PE=1 SV=1 | / | down | 0.3737 | 0.00007 | Signal transduction mechanisms ; Cytoskeleton ; Cell cycle control, cell division, chromosome partitioning ; General function prediction only ; | ko04270,Vascular smooth muscle contraction | |
| 338 | tr\|B7ZAX9\|B7ZAX9_HUMAN | cDNA, FLJ79343, highly similar to SWI/SNF-related matrix-associated actin-dependent regulator of chromatin subfamily A member 5 (EC 3.6.1.-) OS=Homo sapiens PE=2 SV=1 | tr\|B4DZC0\|B4DZC0_HUMAN | down | 0.4682 | 0.00231 | Transcription ; Replication, recombination and repair ; | / | |
| 339 | sp\|P12004\|PCNA_HUMAN | Proliferating cell nuclear antigen OS=Homo sapiens GN=PCNA PE=1 SV=1 | / | down | 0.2968 | 0.00046 | Replication, recombination and repair ; | ko03430,Mismatch repair;ko03410,Base excision repair;ko03030,DNA replication;ko04110,Cell cycle;ko03420,Nucleotide excision repair | |
| 349 | sp\|P04844\|RPN2_HUMAN | Dolichyl-diphosphooligosaccharide--protein glycosyltransferase subunit 2 OS=Homo sapiens GN=RPN2 PE=1 SV=3 | tr\|B2RE46\|B2RE46_HUMAN | down | 0.3851 | 0.02350 | / | ko01100,Metabolic pathways;ko00510,N-Glycan biosynthesis;ko04141,Protein processing in endoplasmic reticulum | |
| 354 | tr\|A0A024R845\|A0A024R845_HUMAN | RAB14, member RAS oncogene family, isoform CRA_a OS=Homo sapiens GN=RAB14 PE=4 SV=1 | sp\|P61106\|RAB14_HUMAN | down | 0.5775 | 0.02571 | General function prediction only ; | / | |
| 359 | sp\|O00425\|IF2B3_HUMAN | Insulin-like growth factor 2 mRNA-binding protein 3 OS=Homo sapiens GN=IGF2BP3 PE=1 SV=2 | / | down | 0.6089 | 0.02239 | / | / | |
| 361 | sp\|P62249\|RS16_HUMAN | 40S ribosomal protein S16 OS=Homo sapiens GN=RPS16 PE=1 SV=2 | / | down | 0.5287 | 0.03211 | Translation, ribosomal structure and biogenesis ; | ko03010,Ribosome | |
| 367 | tr\|B9EKV4\|B9EKV4_HUMAN | Aldehyde dehydrogenase 9 family, member A1 OS=Homo sapiens GN=ALDH9A1 PE=2 SV=1 | sp\|P49189\|AL9A1_HUMAN | down | 0.4372 | 0.01456 | Energy production and conversion ; | ko00310,Lysine degradation;ko00340,Histidine metabolism;ko01120,Microbial metabolism in diverse environments;ko00280,Valine, leucine and isoleucine degradation;ko00071,Fatty acid metabolism;ko01110,Biosynthesis of secondary metabolites;ko00410,beta-Alanine metabolism;ko01100,Metabolic pathways;ko00640,Propanoate metabolism;ko00620,Pyruvate metabolism;ko00010,Glycolysis / Gluconeogenesis;ko00380,Tryptophan metabolism;ko00561,Glycerolipid metabolism;ko00053,Ascorbate and aldarate metabolism;ko00330,Arginine and proline metabolism | |
| 372 | sp\|P40429\|RL13A_HUMAN | 60S ribosomal protein L13a OS=Homo sapiens GN=RPL13A PE=1 SV=2 | / | down | 0.3598 | 0.01059 | Translation, ribosomal structure and biogenesis ; | ko03010,Ribosome | |
| 374 | tr\|V9HWC7\|V9HWC7_HUMAN | Epididymis secretory sperm binding protein Li 128m OS=Homo sapiens GN=HEL-S-128m PE=2 SV=1 | sp\|P30041\|PRDX6_HUMAN | down | 0.5152 | 0.00557 | Posttranslational modification, protein turnover, chaperones ; | ko01120,Microbial metabolism in diverse environments;ko01110,Biosynthesis of secondary metabolites;ko00360,Phenylalanine metabolism;ko00680,Methane metabolism;ko01100,Metabolic pathways;ko00940,Phenylpropanoid biosynthesis | |
| 384 | sp\|Q14444\|CAPR1_HUMAN | Caprin-1 OS=Homo sapiens GN=CAPRIN1 PE=1 SV=2 | / | down | 0.3939 | 0.00227 | / | / | |
| 389 | tr\|Q6NZ55\|Q6NZ55_HUMAN | 60S ribosomal protein L13 OS=Homo sapiens GN=RPL13 PE=2 SV=1 | tr\|A8K4C8\|A8K4C8_HUMAN;sp\|P26373\|RL13_HUMAN | down | 0.3520 | 0.00164 | Translation, ribosomal structure and biogenesis ; | ko03010,Ribosome | |
| 394 | sp\|P61254\|RL26_HUMAN | 60S ribosomal protein L26 OS=Homo sapiens GN=RPL26 PE=1 SV=1 | / | down | 0.3568 | 0.00510 | Translation, ribosomal structure and biogenesis ; | ko03010,Ribosome | |
| 401 | tr\|Q53Z07\|Q53Z07_HUMAN | NPC-A-16 OS=Homo sapiens GN=RPL9 PE=2 SV=1 | sp\|P32969\|RL9_HUMAN | down | 0.4745 | 0.00439 | Translation, ribosomal structure and biogenesis ; | ko03010,Ribosome | |
| 406 | tr\|A0A140VJX1\|A0A140VJX1_HUMAN | Testicular tissue protein Li 198 OS=Homo sapiens PE=2 SV=1 | sp\|P24752\|THIL_HUMAN | down | 0.6269 | 0.00048 | Lipid transport and metabolism ; | ko00310,Lysine degradation;ko00900,Terpenoid backbone biosynthesis;ko01120,Microbial metabolism in diverse environments;ko00280,Valine, leucine and isoleucine degradation;ko02020,Two-component system;ko00071,Fatty acid metabolism;ko00362,Benzoate degradation;ko01110,Biosynthesis of secondary metabolites;ko01100,Metabolic pathways;ko00640,Propanoate metabolism;ko00620,Pyruvate metabolism;ko00072,Synthesis and degradation of ketone bodies;ko00650,Butanoate metabolism;ko00380,Tryptophan metabolism | |
| 409 | tr\|A0A024QZN9\|A0A024QZN9_HUMAN | Voltage-dependent anion channel 2, isoform CRA_a OS=Homo sapiens GN=VDAC2 PE=4 SV=1 | tr\|A0A024QZT0\|A0A024QZT0_HUMAN;sp\|P45880\|VDAC2_HUMAN;tr\|A0A0A0MR02\|A0A0A0MR02_HUMAN | down | 0.4240 | 0.02002 | / | ko04020,Calcium signaling pathway;ko05016,Huntington's disease;ko05012,Parkinson's disease | |
| 419 | sp\|P08727\|K1C19_HUMAN | Keratin, type I cytoskeletal 19 OS=Homo sapiens GN=KRT19 PE=1 SV=4 | / | down | 0.4089 | 0.01226 | / | / | |
| 425 | tr\|A0A024R4M0\|A0A024R4M0_HUMAN | 40S ribosomal protein S9 OS=Homo sapiens GN=RPS9 PE=1 SV=1 | sp\|P46781\|RS9_HUMAN | down | 0.4954 | 0.02041 | Translation, ribosomal structure and biogenesis ; | ko03010,Ribosome | |
| 443 | tr\|V9HW01\|V9HW01_HUMAN | Epididymis secretory protein Li 310 OS=Homo sapiens GN=HEL-S-310 PE=2 SV=1 | sp\|P83731\|RL24_HUMAN | down | 0.4069 | 0.00826 | Translation, ribosomal structure and biogenesis ; | ko03010,Ribosome | |
| 453 | tr\|B2RDE8\|B2RDE8_HUMAN | cDNA, FLJ96580, highly similar to Homo sapiens hepatoma-derived growth factor (high-mobility group protein 1-like) (HDGF), mRNA OS=Homo sapiens PE=2 SV=1 | sp\|P51858\|HDGF_HUMAN | down | 0.4391 | 0.00987 | / | ko00280,Valine, leucine and isoleucine degradation;ko01100,Metabolic pathways | |
| 459 | tr\|Q53XM7\|Q53XM7_HUMAN | VAMP (Vesicle-associated membrane protein)-associated protein B and C OS=Homo sapiens GN=VAPB PE=1 SV=1 | sp\|O95292\|VAPB_HUMAN | down | 0.5164 | 0.02352 | Intracellular trafficking, secretion, and vesicular transport ; | / | |
| 462 | tr\|A8K8U1\|A8K8U1_HUMAN | cDNA FLJ77762, highly similar to Homo sapiens cullin-associated and neddylation-dissociated 1 (CAND1), mRNA OS=Homo sapiens PE=2 SV=1 | sp\|Q86VP6\|CAND1_HUMAN | down | 0.5594 | 0.00893 | / | / | |
| 465 | sp\|O43776\|SYNC_HUMAN | Asparagine--tRNA ligase, cytoplasmic OS=Homo sapiens GN=NARS PE=1 SV=1 | / | down | 0.6183 | 0.01542 | Translation, ribosomal structure and biogenesis ; | ko00970,Aminoacyl-tRNA biosynthesis | |
| 466 | tr\|B3KSL5\|B3KSL5_HUMAN | cDNA FLJ36545 fis, clone TRACH2006670, highly similar to RNA-binding protein Luc7-like 2 OS=Homo sapiens PE=2 SV=1 | sp\|Q9Y383\|LC7L2_HUMAN | down | 0.4948 | 0.02136 | RNA processing and modification ; |  |  |
| 467 | sp\|P29373\|RABP2_HUMAN | Cellular retinoic acid-binding protein 2 OS=Homo sapiens GN=CRABP2 PE=1 SV=2 | / | down | 0.2506 | 0.00324 | / | ko03320,PPAR signaling pathway | |
| 470 | tr\|A0A024R5C5\|A0A024R5C5_HUMAN | Pyruvate carboxylase OS=Homo sapiens GN=PC PE=4 SV=1 | sp\|P11498\|PYC_HUMAN | up | 2.2066 | 0.04684 | Energy production and conversion ;Lipid transport and metabolism ; | ko01120,Microbial metabolism in diverse environments;ko00020,Citrate cycle (TCA cycle);ko01100,Metabolic pathways;ko00620,Pyruvate metabolism | |
| 472 | tr\|A2A3R6\|A2A3R6_HUMAN | 40S ribosomal protein S6 OS=Homo sapiens GN=RPS6 PE=2 SV=1 | sp\|P62753\|RS6_HUMAN | down | 0.4905 | 0.00020 | Translation, ribosomal structure and biogenesis ; | ko03010,Ribosome;ko04910,Insulin signaling pathway;ko04150,mTOR signaling pathway | |
| 481 | sp\|Q9HDC9\|APMAP_HUMAN | Adipocyte plasma membrane-associated protein OS=Homo sapiens GN=APMAP PE=1 SV=2 | / | down | 0.5897 | 0.04280 | Carbohydrate transport and metabolism ; | ko00901,Indole alkaloid biosynthesis;ko01110,Biosynthesis of secondary metabolites;ko01100,Metabolic pathways | |
| 484 | tr\|B4DV28\|B4DV28_HUMAN | cDNA FLJ54170, highly similar to Cytosolic nonspecific dipeptidase OS=Homo sapiens PE=2 SV=1 | / | down | 0.6607 | 0.01635 | Amino acid transport and metabolism ; | / | |
| 487 | sp\|Q71DI3\|H32_HUMAN | Histone H3.2 OS=Homo sapiens GN=HIST2H3A PE=1 SV=3 | / | down | 0.4220 | 0.02689 | Chromatin structure and dynamics ; | ko05322,Systemic lupus erythematosus | |
| 491 | tr\|Q6FHZ4\|Q6FHZ4_HUMAN | Galectin OS=Homo sapiens GN=LGALS4 PE=2 SV=1 | sp\|P56470\|LEG4_HUMAN | down | 0.6156 | 0.00073 | / | / | |
| 496 | tr\|B4DQI7\|B4DQI7_HUMAN | cDNA FLJ58042, highly similar to Protein NipSnap1 OS=Homo sapiens PE=2 SV=1 | sp\|Q9BPW8\|NIPS1_HUMAN | down | 0.5400 | 0.02135 | / | / | |
| 497 | tr\|A0A024R231\|A0A024R231_HUMAN | Guanine deaminase, isoform CRA_b OS=Homo sapiens GN=GDA PE=4 SV=1 | sp\|Q9Y2T3\|GUAD_HUMAN | down | 0.4882 | 0.03711 | Nucleotide transport and metabolism ; General function prediction only ; | ko00230,Purine metabolism;ko01100,Metabolic pathways | |
| 502 | sp\|P35527\|K1C9_HUMAN | Keratin, type I cytoskeletal 9 OS=Homo sapiens GN=KRT9 PE=1 SV=3 | / | down | 0.4656 | 0.00114 | / | / | |
| 506 | tr\|A8K0T9\|A8K0T9_HUMAN | cDNA FLJ75422, highly similar to Homo sapiens capping protein (actin filament) muscle Z-line, alpha 1, mRNA OS=Homo sapiens PE=2 SV=1 | tr\|A0A024R0E5\|A0A024R0E5_HUMAN;sp\|P52907\|CAZA1_HUMAN | down | 0.4214 | 0.03661 | / | / | |
| 511 | sp\|Q12797\|ASPH_HUMAN | Aspartyl/asparaginyl beta-hydroxylase OS=Homo sapiens GN=ASPH PE=1 SV=3 | / | up | 1.7434 | 0.00734 | Posttranslational modification, protein turnover, chaperones ; | / | |
| 514 | tr\|A0A090N7V5\|A0A090N7V5_HUMAN | Chromosome 7 open reading frame 24 OS=Homo sapiens GN=C7orf24 PE=4 SV=1 | sp\|O75223\|GGCT_HUMAN | down | 0.6130 | 0.04473 | / | ko00480,Glutathione metabolism | |
| 515 | tr\|A0A024RDG1\|A0A024RDG1_HUMAN | Vesicle docking protein p115, isoform CRA_a OS=Homo sapiens GN=VDP PE=4 SV=1 | sp\|O60763\|USO1_HUMAN | down | 0.6114 | 0.02468 | / | / | |
| 525 | sp\|P61160\|ARP2_HUMAN | Actin-related protein 2 OS=Homo sapiens GN=ACTR2 PE=1 SV=1 | / | down | 0.4309 | 0.01354 | Cytoskeleton ; | ko04520,Adherens junction;ko04530,Tight junction;ko05410,Hypertrophic cardiomyopathy (HCM);ko05414,Dilated cardiomyopathy;ko04810,Regulation of actin cytoskeleton;ko04670,Leukocyte transendothelial migration;ko04510,Focal adhesion;ko05416,Viral myocarditis;ko05131,Shigellosis;ko05110,Vibrio cholerae infection;ko04745,Phototransduction - fly;ko05100,Bacterial invasion of epithelial cells;ko05130,Pathogenic Escherichia coli infection;ko04145,Phagosome;ko05412,Arrhythmogenic right ventricular cardiomyopathy (ARVC) | |
| 536 | sp\|Q9UMS4\|PRP19_HUMAN | Pre-mRNA-processing factor 19 OS=Homo sapiens GN=PRPF19 PE=1 SV=1 | / | down | 0.3348 | 0.03853 | General function prediction only ; | ko04120,Ubiquitin mediated proteolysis;ko03040,Spliceosome | |
| 537 | tr\|A0A024R588\|A0A024R588_HUMAN | Splicing factor 1, isoform CRA_d OS=Homo sapiens GN=SF1 PE=4 SV=1 | / | down | 0.6005 | 0.04544 | RNA processing and modification ; | / | |
| 541 | sp\|Q9H444\|CHM4B_HUMAN | Charged multivesicular body protein 4b OS=Homo sapiens GN=CHMP4B PE=1 SV=1 | / | down | 0.5895 | 0.01214 | / | ko04144,Endocytosis | |
| 577 | tr\|H0Y4R1\|H0Y4R1_HUMAN | Inosine-5'-monophosphate dehydrogenase 2 (Fragment) OS=Homo sapiens GN=IMPDH2 PE=1 SV=1 | sp\|P12268\|IMDH2_HUMAN | up | 2.2712 | 0.01902 | Nucleotide transport and metabolism ;General function prediction only ; | ko00983,Drug metabolism - other enzymes;ko01110,Biosynthesis of secondary metabolites;ko00230,Purine metabolism;ko01100,Metabolic pathways | |
| 584 | sp\|P20290\|BTF3_HUMAN | Transcription factor BTF3 OS=Homo sapiens GN=BTF3 PE=1 SV=1 | / | down | 0.3002 | 0.02770 | / | / | |
| 587 | tr\|B3KSH1\|B3KSH1_HUMAN | Eukaryotic translation initiation factor 3 subunit F OS=Homo sapiens GN=EIF3F PE=2 SV=1 | / | down | 0.5952 | 0.03787 | General function prediction only ; | ko03013,RNA transport | |
| 589 | tr\|B2R4W8\|B2R4W8_HUMAN | HCG1994130, isoform CRA_a OS=Homo sapiens GN=hCG_1994130 PE=2 SV=1 | sp\|P62244\|RS15A_HUMAN | down | 0.1731 | 0.00795 | Translation, ribosomal structure and biogenesis ; | ko03010,Ribosome | |
| 594 | tr\|D6W5K2\|D6W5K2_HUMAN | Thymosin, beta 10, isoform CRA_a (Fragment) OS=Homo sapiens GN=TMSB10 PE=4 SV=1 | / | down | 0.2875 | 0.00194 | / | / | |
| 616 | tr\|B2R761\|B2R761_HUMAN | cDNA, FLJ93299, highly similar to Homo sapiens sterol carrier protein 2 (SCP2), mRNA OS=Homo sapiens PE=2 SV=1 | / | down | 0.6080 | 0.00608 | Lipid transport and metabolism ; | ko04146,Peroxisome;ko03320,PPAR signaling pathway;ko01100,Metabolic pathways;ko00120,Primary bile acid biosynthesis | |
| 633 | tr\|A0A087WV66\|A0A087WV66_HUMAN | Antigen KI-67 OS=Homo sapiens GN=MKI67 PE=1 SV=1 | sp\|P46013\|KI67_HUMAN | up | 1.8991 | 0.00222 | / | / | |
| 638 | tr\|V9HW71\|V9HW71_HUMAN | Endoplasmic reticulum resident protein 29 OS=Homo sapiens GN=HEL-S-107 PE=2 SV=1 | sp\|P30040\|ERP29_HUMAN | down | 0.6278 | 0.04595 | Posttranslational modification, protein turnover, chaperones ; Energy production and conversion ; | ko04141,Protein processing in endoplasmic reticulum | |
| 641 | tr\|J3QQ67\|J3QQ67_HUMAN | 60S ribosomal protein L18 (Fragment) OS=Homo sapiens GN=RPL18 PE=1 SV=1 | tr\|A0A024QZD1\|A0A024QZD1_HUMAN;sp\|Q07020\|RL18_HUMAN;tr\|G3V203\|G3V203_HUMAN | down | 0.2761 | 0.00503 | Translation, ribosomal structure and biogenesis ; | ko03010,Ribosome | |
| 643 | sp\|P30044\|PRDX5_HUMAN | Peroxiredoxin-5, mitochondrial OS=Homo sapiens GN=PRDX5 PE=1 SV=4 | tr\|V9HW35\|V9HW35_HUMAN | down | 0.5221 | 0.01936 | Posttranslational modification, protein turnover, chaperones ; | ko04146,Peroxisome | |
| 669 | tr\|H9ZYJ2\|H9ZYJ2_HUMAN | Thioredoxin OS=Homo sapiens GN=TXN PE=2 SV=1 | sp\|P10599\|THIO_HUMAN | down | 0.3958 | 0.00597 | Posttranslational modification, protein turnover, chaperones ; Energy production and conversion ; | / | |
| 671 | tr\|B3KML1\|B3KML1_HUMAN | cDNA FLJ11308 fis, clone PLACE1010074, highly similar to Sorting nexin-2 OS=Homo sapiens PE=2 SV=1 | sp\|O60749\|SNX2_HUMAN;tr\|B3KN57\|B3KN57_HUMAN | down | 0.6335 | 0.04821 | Intracellular trafficking, secretion, and vesicular transport ; General function prediction only ; | / | |
| 675 | tr\|A0A024RDF4\|A0A024RDF4_HUMAN | Heterogeneous nuclear ribonucleoprotein D (AU-rich element RNA binding protein 1, 37kDa), isoform CRA_e OS=Homo sapiens GN=HNRPD PE=4 SV=1 | tr\|A0A024RDF3\|A0A024RDF3_HUMAN;sp\|Q14103\|HNRPD_HUMAN | down | 0.3988 | 0.01151 | General function prediction only ; | / | |
| 680 | sp\|Q9Y2W1\|TR150_HUMAN | Thyroid hormone receptor-associated protein 3 OS=Homo sapiens GN=THRAP3 PE=1 SV=2 | / | down | 0.6666 | 0.01422 | / | / | |
| 696 | sp\|Q15942\|ZYX_HUMAN | Zyxin OS=Homo sapiens GN=ZYX PE=1 SV=1 | tr\|H0Y2Y8\|H0Y2Y8_HUMAN | down | 0.5064 | 0.01272 | / | ko04510,Focal adhesion | |
| 700 | tr\|D3DND1\|D3DND1_HUMAN | CDV3 homolog (Mouse), isoform CRA_a OS=Homo sapiens GN=CDV3 PE=4 SV=1 | / | down | 0.6309 | 0.03779 | / | / | |
| 745 | tr\|X5DNI9\|X5DNI9_HUMAN | 7-dehydrocholesterol reductase isoform A (Fragment) OS=Homo sapiens GN=DHCR7 PE=2 SV=1 | tr\|A0A024R5F7\|A0A024R5F7_HUMAN;sp\|Q9UBM7\|DHCR7_HUMAN | down | 0.3138 | 0.02915 | / | ko00100,Steroid biosynthesis;ko01110,Biosynthesis of secondary metabolites;ko01100,Metabolic pathways | |
| 758 | sp\|P54727\|RD23B_HUMAN | UV excision repair protein RAD23 homolog B OS=Homo sapiens GN=RAD23B PE=1 SV=1 | / | down | 0.4650 | 0.03117 | Posttranslational modification, protein turnover, chaperones ; | ko03420,Nucleotide excision repair;ko04141,Protein processing in endoplasmic reticulum | |
| 765 | tr\|A0A0S2Z3X8\|A0A0S2Z3X8_HUMAN | GDP dissociation inhibitor 1 isoform 1 (Fragment) OS=Homo sapiens GN=GDI1 PE=2 SV=1 | sp\|P31150\|GDIA_HUMAN;tr\|B4DHX4\|B4DHX4_HUMAN | down | 0.4729 | 0.03669 | Posttranslational modification, protein turnover, chaperones ; | / | |
| 767 | tr\|B2RDF5\|B2RDF5_HUMAN | cDNA, FLJ96587, highly similar to Homo sapiens SUMO-1 activating enzyme subunit 2 (UBA2), mRNA OS=Homo sapiens PE=2 SV=1 | / | down | 0.6438 | 0.02515 | Coenzyme transport and metabolism ; | ko04120,Ubiquitin mediated proteolysis | |
| 770 | tr\|Q0P5N8\|Q0P5N8_HUMAN | TMSB4X protein (Fragment) OS=Homo sapiens GN=TMSB4X PE=2 SV=1 | tr\|Q0P5U7\|Q0P5U7_HUMAN;tr\|Q0P5P4\|Q0P5P4_HUMAN;tr\|Q0P5T0\|Q0P5T0_HUMAN;tr\|Q0P5Q0\|Q0P5Q0_HUMAN;tr\|A2VCK8\|A2VCK8_HUMAN;sp\|P62328\|TYB4_HUMAN | down | 0.1503 | 0.00876 | / | ko04810,Regulation of actin cytoskeleton | |
| 808 | tr\|F6WQW2\|F6WQW2_HUMAN | Ran-specific GTPase-activating protein OS=Homo sapiens GN=RANBP1 PE=1 SV=1 | / | down | 0.3531 | 0.03363 | Intracellular trafficking, secretion, and vesicular transport ; | ko03013,RNA transport | |
| 809 | tr\|A0A024R1V4\|A0A024R1V4_HUMAN | 60S ribosomal protein L27 OS=Homo sapiens GN=RPL27 PE=3 SV=1 | sp\|P61353\|RL27_HUMAN | down | 0.5705 | 0.01281 | Translation, ribosomal structure and biogenesis ; | ko03010,Ribosome | |
| 812 | tr\|Q6FI03\|Q6FI03_HUMAN | G3BP protein OS=Homo sapiens GN=G3BP PE=2 SV=1 | tr\|Q5U0Q1\|Q5U0Q1_HUMAN;sp\|Q13283\|G3BP1_HUMAN | down | 0.4119 | 0.01935 | / | ko03015,mRNA surveillance pathway;ko03040,Spliceosome;ko03013,RNA transport | |
| 817 | sp\|P59998\|ARPC4_HUMAN | Actin-related protein 2/3 complex subunit 4 OS=Homo sapiens GN=ARPC4 PE=1 SV=3 | / | down | 0.3679 | 0.02157 | / | ko05100,Bacterial invasion of epithelial cells;ko04810,Regulation of actin cytoskeleton;ko04666,Fc gamma R-mediated phagocytosis;ko05130,Pathogenic Escherichia coli infection;ko05131,Shigellosis | |
| 825 | tr\|A0A140VKC8\|A0A140VKC8_HUMAN | Testis tissue sperm-binding protein Li 45a OS=Homo sapiens PE=2 SV=1 | sp\|Q13630\|FCL_HUMAN | down | 0.5661 | 0.01401 | Cell wall/membrane/envelope biogenesis ; Carbohydrate transport and metabolism ; | ko00051,Fructose and mannose metabolism;ko00520,Amino sugar and nucleotide sugar metabolism;ko01100,Metabolic pathways | |
| 833 | tr\|Q5STK2\|Q5STK2_HUMAN | Prefoldin subunit 6, isoform CRA_b OS=Homo sapiens GN=PFDN6 PE=2 SV=1 | sp\|O15212\|PFD6_HUMAN | down | 0.3759 | 0.00656 | Posttranslational modification, protein turnover, chaperones ; | / | |
| 856 | sp\|P48047\|ATPO_HUMAN | ATP synthase subunit O, mitochondrial OS=Homo sapiens GN=ATP5O PE=1 SV=1 | / | down | 0.6465 | 0.02573 | Energy production and conversion ; | ko05010,Alzheimer's disease;ko00190,Oxidative phosphorylation;ko05016,Huntington's disease;ko05012,Parkinson's disease;ko01100,Metabolic pathways | |
| 864 | tr\|Q6FIA3\|Q6FIA3_HUMAN | PACSIN2 protein OS=Homo sapiens GN=PACSIN2 PE=2 SV=1 | sp\|Q9UNF0\|PACN2_HUMAN | up | 2.4138 | 0.00833 | / | ko04530,Tight junction;ko05100,Bacterial invasion of epithelial cells;ko04660,T cell receptor signaling pathway;ko05016,Huntington's disease;ko05130,Pathogenic Escherichia coli infection;ko05131,Shigellosis | |
| 875 | tr\|B2RCX0\|B2RCX0_HUMAN | cDNA, FLJ96345, Homo sapiens SET translocation (myeloid leukemia-associated) (SET),mRNA OS=Homo sapiens PE=2 SV=1 | tr\|A0A024R895\|A0A024R895_HUMAN | down | 0.5770 | 0.03551 | / | / | |
| 879 | sp\|Q07812\|BAX_HUMAN | Apoptosis regulator BAX OS=Homo sapiens GN=BAX PE=1 SV=1 | / | down | 0.5475 | 0.02608 | / | ko05200,Pathways in cancer;ko04115,p53 signaling pathway;ko05020,Prion diseases;ko05016,Huntington's disease;ko04210,Apoptosis;ko04722,Neurotrophin signaling pathway;ko05210,Colorectal cancer;ko05014,Amyotrophic lateral sclerosis (ALS);ko04141,Protein processing in endoplasmic reticulum | |
| 880 | tr\|B2R4C1\|B2R4C1_HUMAN | cDNA, FLJ92036, highly similar to Homo sapiens ribosomal protein L31 (RPL31), mRNA OS=Homo sapiens PE=2 SV=1 | sp\|P62899\|RL31_HUMAN | down | 0.4498 | 0.01045 | Translation, ribosomal structure and biogenesis ; | ko03010,Ribosome | |
| 908 | tr\|A8KAN3\|A8KAN3_HUMAN | cDNA FLJ77226, highly similar to Homo sapiens p21 activated kinase 1B (PAK1B) mRNA OS=Homo sapiens PE=2 SV=1 | / | up | 1.7130 | 0.02721 | General function prediction only ; Signal transduction mechanisms ; Transcription ; Replication, recombination and repair ; | ko04360,Axon guidance;ko04810,Regulation of actin cytoskeleton;ko04062,Chemokine signaling pathway;ko04660,T cell receptor signaling pathway;ko04510,Focal adhesion;ko04650,Natural killer cell mediated cytotoxicity;ko05211,Renal cell carcinoma;ko04012,ErbB signaling pathway;ko04011,MAPK signaling pathway - yeast;ko04666,Fc gamma R-mediated phagocytosis;ko04010,MAPK signaling pathway;ko05120,Epithelial cell signaling in Helicobacter pylori infection | |
| 909 | tr\|A0A0S2Z5U7\|A0A0S2Z5U7_HUMAN | Diablo-like protein isoform 1 (Fragment) OS=Homo sapiens GN=DIABLO PE=2 SV=1 | sp\|Q9NR28\|DBLOH_HUMAN | down | 0.4840 | 0.03590 | / | / | |
| 916 | tr\|Q5T5C7\|Q5T5C7_HUMAN | Serine--tRNA ligase, cytoplasmic OS=Homo sapiens GN=SARS PE=1 SV=1 | tr\|Q53HA4\|Q53HA4_HUMAN;tr\|Q0VGA5\|Q0VGA5_HUMAN;sp\|P49591\|SYSC_HUMAN | down | 0.4921 | 0.03950 | Translation, ribosomal structure and biogenesis ; | ko00970,Aminoacyl-tRNA biosynthesis | |
| 925 | tr\|F8W031\|F8W031_HUMAN | Uncharacterized protein (Fragment) OS=Homo sapiens PE=1 SV=1 | / | down | 0.4386 | 0.03984 | / | ko00630,Glyoxylate and dicarboxylate metabolism;ko01120,Microbial metabolism in diverse environments;ko00020,Citrate cycle (TCA cycle);ko01110,Biosynthesis of secondary metabolites;ko01100,Metabolic pathways | |
| 931 | sp\|Q99426\|TBCB_HUMAN | Tubulin-folding cofactor B OS=Homo sapiens GN=TBCB PE=1 SV=2 | / | down | 0.3968 | 0.04036 | Cell cycle control, cell division, chromosome partitioning ; | ko04962,Vasopressin-regulated water reabsorption;ko05016,Huntington's disease | |
| 949 | tr\|Q53G49\|Q53G49_HUMAN | Ribosomal protein L19 (Fragment) OS=Homo sapiens PE=2 SV=1 | tr\|J3QR09\|J3QR09_HUMAN;tr\|J3KTE4\|J3KTE4_HUMAN;sp\|P84098\|RL19_HUMAN | down | 0.4188 | 0.00378 | Translation, ribosomal structure and biogenesis ; | ko03010,Ribosome | |
| 961 | sp\|P35268\|RL22_HUMAN | 60S ribosomal protein L22 OS=Homo sapiens GN=RPL22 PE=1 SV=2 | / | down | 0.5281 | 0.04984 | / | ko03010,Ribosome | |
| 968 | sp\|P62750\|RL23A_HUMAN | 60S ribosomal protein L23a OS=Homo sapiens GN=RPL23A PE=1 SV=1 | / | down | 0.5237 | 0.04261 | Translation, ribosomal structure and biogenesis ; | ko03010,Ribosome | |
| 1019 | tr\|A0A024R2Q4\|A0A024R2Q4_HUMAN | Ribosomal protein L15 OS=Homo sapiens GN=RPL15 PE=3 SV=1 | sp\|P61313\|RL15_HUMAN | down | 0.5299 | 0.00974 | Translation, ribosomal structure and biogenesis ; | ko03010,Ribosome | |
| 1025 | tr\|H7BXI1\|H7BXI1_HUMAN | Extended synaptotagmin-2 (Fragment) OS=Homo sapiens GN=ESYT2 PE=1 SV=1 | / | down | 0.5652 | 0.02311 | General function prediction only ; | ko05214,Glioma;ko04370,VEGF signaling pathway;ko04972,Pancreatic secretion;ko04530,Tight junction;ko05200,Pathways in cancer;ko04670,Leukocyte transendothelial migration;ko04510,Focal adhesion;ko04730,Long-term depression;ko04540,Gap junction;ko04012,ErbB signaling pathway;ko04070,Phosphatidylinositol signaling system;ko04666,Fc gamma R-mediated phagocytosis;ko04720,Long-term potentiation;ko05146,Amoebiasis;ko04270,Vascular smooth muscle contraction;ko05223,Non-small cell lung cancer;ko04650,Natural killer cell mediated cytotoxicity;ko04310,Wnt signaling pathway;ko01100,Metabolic pathways;ko04011,MAPK signaling pathway - yeast;ko00562,Inositol phosphate metabolism;ko04970,Salivary secretion;ko04745,Phototransduction - fly;ko05110,Vibrio cholerae infection;ko04020,Calcium signaling pathway;ko04010,MAPK signaling pathway;ko04960,Aldosterone-regulated sodium reabsorption;ko04971,Gastric acid secretion;ko04916,Melanogenesis | |
| 1042 | tr\|Q5U000\|Q5U000_HUMAN | Cathepsin Z OS=Homo sapiens PE=2 SV=1 | sp\|Q9UBR2\|CATZ_HUMAN | down | 0.4737 | 0.00462 | Posttranslational modification, protein turnover, chaperones ; | ko04142,Lysosome | |
| 1048 | sp\|P49006\|MRP_HUMAN | MARCKS-related protein OS=Homo sapiens GN=MARCKSL1 PE=1 SV=2 | / | up | 2.1746 | 0.02488 | / | ko05140,Leishmaniasis;ko04666,Fc gamma R-mediated phagocytosis | |
| 1079 | tr\|Q6IB11\|Q6IB11_HUMAN | PGRMC1 protein OS=Homo sapiens GN=PGRMC1 PE=2 SV=1 | sp\|O00264\|PGRC1_HUMAN | down | 0.4473 | 0.00765 | / | / | |
| 1095 | sp\|P05204\|HMGN2_HUMAN | Non-histone chromosomal protein HMG-17 OS=Homo sapiens GN=HMGN2 PE=1 SV=3 | / | down | 0.3296 | 0.00043 | / | / | |
| 1106 | tr\|B4E2T6\|B4E2T6_HUMAN | cDNA FLJ58231, highly similar to NMDA receptor-regulated protein 1 OS=Homo sapiens PE=2 SV=1 | / | down | 0.6015 | 0.01897 | General function prediction only ; | / | |
| 1177 | sp\|Q9H0U4\|RAB1B_HUMAN | Ras-related protein Rab-1B OS=Homo sapiens GN=RAB1B PE=1 SV=1 | / | down | 0.4349 | 0.01862 | General function prediction only ; | / | |
| 1204 | tr\|V9HWJ8\|V9HWJ8_HUMAN | Epididymis secretory protein Li 283 OS=Homo sapiens GN=HEL-S-283 PE=2 SV=1 | sp\|P61289\|PSME3_HUMAN | down | 0.4165 | 0.00879 | / | ko05160,Hepatitis C;ko03050,Proteasome;ko04612,Antigen processing and presentation | |
| 1243 | tr\|D3DPK5\|D3DPK5_HUMAN | SH3 domain binding glutamic acid-rich protein like 3, isoform CRA_a (Fragment) OS=Homo sapiens GN=SH3BGRL3 PE=4 SV=1 | / | down | 0.2843 | 0.01248 | / | ko04370,VEGF signaling pathway;ko04972,Pancreatic secretion;ko00591,Linoleic acid metabolism;ko04270,Vascular smooth muscle contraction;ko04730,Long-term depression;ko00564,Glycerophospholipid metabolism;ko04664,Fc epsilon RI signaling pathway;ko01100,Metabolic pathways;ko05145,Toxoplasmosis;ko04912,GnRH signaling pathway;ko00590,Arachidonic acid metabolism;ko04010,MAPK signaling pathway;ko00565,Ether lipid metabolism;ko00592,alpha-Linolenic acid metabolism | |
| 1245 | tr\|B3KT21\|B3KT21_HUMAN | cDNA FLJ37476 fis, clone BRAWH2012827, highly similar to Homo sapiens BH3 interacting domain death agonist (BID), transcript variant 1, mRNA OS=Homo sapiens PE=2 SV=1 | tr\|A8ASI8\|A8ASI8_HUMAN;sp\|P55957\|BID_HUMAN | down | 0.5596 | 0.04793 | / | ko05010,Alzheimer's disease;ko05200,Pathways in cancer;ko04115,p53 signaling pathway;ko05416,Viral myocarditis;ko04650,Natural killer cell mediated cytotoxicity;ko05014,Amyotrophic lateral sclerosis (ALS);ko04210,Apoptosis | |
| 1261 | tr\|A0A024QZN2\|A0A024QZN2_HUMAN | HCG2024613, isoform CRA_a OS=Homo sapiens GN=hCG_2024613 PE=4 SV=1 | sp\|Q8WXX5\|DNJC9_HUMAN | up | 1.5027 | 0.01918 | Posttranslational modification, protein turnover, chaperones ; | / | |
| 1295 | tr\|D3DV26\|D3DV26_HUMAN | S100 calcium binding protein A10 (Annexin II ligand, calpactin I, light polypeptide (P11)), isoform CRA_b (Fragment) OS=Homo sapiens GN=S100A10 PE=4 SV=1 | / | down | 0.6178 | 0.03050 | / | / | |
| 1297 | sp\|P23368\|MAOM_HUMAN | NAD-dependent malic enzyme, mitochondrial OS=Homo sapiens GN=ME2 PE=1 SV=1 | / | down | 0.5941 | 0.04706 | Energy production and conversion ; | ko02020,Two-component system;ko00620,Pyruvate metabolism | |
| 1316 | sp\|Q15008\|PSMD6_HUMAN | 26S proteasome non-ATPase regulatory subunit 6 OS=Homo sapiens GN=PSMD6 PE=1 SV=1 | / | up | 1.7404 | 0.03641 | Posttranslational modification, protein turnover, chaperones ; | ko03050,Proteasome | |
| 1343 | tr\|A0A024RBR3\|A0A024RBR3_HUMAN | Density-regulated protein OS=Homo sapiens GN=DENR PE=3 SV=1 | sp\|O43583\|DENR_HUMAN | down | 0.5402 | 0.02108 | Translation, ribosomal structure and biogenesis ; | / | |
| 1378 | sp\|Q14240\|IF4A2_HUMAN | Eukaryotic initiation factor 4A-II OS=Homo sapiens GN=EIF4A2 PE=1 SV=2 | / | down | 0.3546 | 0.04365 | Replication, recombination and repair ; Transcription ; Translation, ribosomal structure and biogenesis ; | ko03013,RNA transport | |
| 1389 | sp\|P61026\|RAB10_HUMAN | Ras-related protein Rab-10 OS=Homo sapiens GN=RAB10 PE=1 SV=1 | / | down | 0.3559 | 0.04531 | General function prediction only ; | / | |
| 1457 | sp\|Q86X55\|CARM1_HUMAN | Histone-arginine methyltransferase CARM1 OS=Homo sapiens GN=CARM1 PE=1 SV=3 | / | up | 1.6590 | 0.01463 | Secondary metabolites biosynthesis, transport and catabolism ; General function prediction only ; | / | |
| 1474 | tr\|Q53F62\|Q53F62_HUMAN | ADP-ribosylation factor GTPase activating protein 1 isoform a variant (Fragment) OS=Homo sapiens PE=2 SV=1 | sp\|Q8N6T3\|ARFG1_HUMAN | down | 0.5978 | 0.02817 | Intracellular trafficking, secretion, and vesicular transport ; | ko04144,Endocytosis | |
| 1479 | tr\|Q6NUN2\|Q6NUN2_HUMAN | NFKB repressing factor OS=Homo sapiens GN=NKRF PE=2 SV=1 | tr\|A3F769\|A3F769_HUMAN;tr\|A3F768\|A3F768_HUMAN;sp\|O15226\|NKRF_HUMAN | up | 3.2007 | 0.04282 | / | / | |
| 1486 | tr\|Q53HG5\|Q53HG5_HUMAN | KIAA0103 variant (Fragment) OS=Homo sapiens PE=2 SV=1 | tr\|A8K4K9\|A8K4K9_HUMAN;sp\|Q15006\|EMC2_HUMAN | down | 0.4006 | 0.02940 | / | / | |
| 1527 | tr\|Q59EL4\|Q59EL4_HUMAN | PRPF4 protein variant (Fragment) OS=Homo sapiens PE=2 SV=1 | / | down | 0.3825 | 0.01882 | General function prediction only ; | ko03040,Spliceosome | |
| 1625 | tr\|D0EKE5\|D0EKE5_HUMAN | Aryl hydrocarbon receptor interacting protein OS=Homo sapiens GN=AIP PE=4 SV=1 | tr\|G9I2H4\|G9I2H4_HUMAN;tr\|D5LTB3\|D5LTB3_HUMAN;tr\|D0EKE6\|D0EKE6_HUMAN;tr\|C8CK05\|C8CK05_HUMAN;tr\|C8CHN4\|C8CHN4_HUMAN;tr\|B7SBB1\|B7SBB1_HUMAN | down | 0.4537 | 0.04769 | / | ko04020,Calcium signaling pathway;ko05016,Huntington's disease;ko05012,Parkinson's disease | |
| 1633 | sp\|P43034\|LIS1_HUMAN | Platelet-activating factor acetylhydrolase IB subunit alpha OS=Homo sapiens GN=PAFAH1B1 PE=1 SV=2 | / | down | 0.4698 | 0.01573 | General function prediction only ; | ko00565,Ether lipid metabolism;ko01100,Metabolic pathways | |
| 1679 | tr\|A0A0S2Z4Y4\|A0A0S2Z4Y4_HUMAN | Clathrin interactor 1 isoform 1 (Fragment) OS=Homo sapiens GN=CLINT1 PE=2 SV=1 | sp\|Q14677\|EPN4_HUMAN;tr\|A0A0S2Z5H3\|A0A0S2Z5H3_HUMAN | up | 1.9424 | 0.01759 | / | ko04144,Endocytosis | |
| 1717 | sp\|Q9BY49\|PECR_HUMAN | Peroxisomal trans-2-enoyl-CoA reductase OS=Homo sapiens GN=PECR PE=1 SV=2 | / | up | 1.5711 | 0.04656 | Lipid transport and metabolism ; Secondary metabolites biosynthesis, transport and catabolism ; General function prediction only ; | ko04146,Peroxisome;ko01040,Biosynthesis of unsaturated fatty acids | |
| 1849 | tr\|E9PCY5\|E9PCY5_HUMAN | DNA topoisomerase 2 (Fragment) OS=Homo sapiens GN=TOP2B PE=1 SV=1 | / | down | 0.4586 | 0.03250 | Replication, recombination and repair ; | / | |
| 1873 | tr\|Q6FIE9\|Q6FIE9_HUMAN | TOLLIP protein OS=Homo sapiens GN=TOLLIP PE=1 SV=1 | sp\|Q9H0E2\|TOLIP_HUMAN | up | 2.2745 | 0.01019 | / | ko04620,Toll-like receptor signaling pathway | |
| 1923 | sp\|Q8NEN9\|PDZD8_HUMAN | PDZ domain-containing protein 8 OS=Homo sapiens GN=PDZD8 PE=1 SV=1 | / | up | 15.5285 | 0.02967 | / | / | |
| 1944 | tr\|A0A024RCA7\|A0A024RCA7_HUMAN | Ribosomal protein, large, P2, isoform CRA_a OS=Homo sapiens GN=RPLP2 PE=3 SV=1 | sp\|P05387\|RLA2_HUMAN | down | 0.6438 | 0.01568 | Translation, ribosomal structure and biogenesis ; | ko03010,Ribosome | |
| 2251 | sp\|O14949\|QCR8_HUMAN | Cytochrome b-c1 complex subunit 8 OS=Homo sapiens GN=UQCRQ PE=1 SV=4 | / | up | 4.0471 | 0.04388 | / | ko04260,Cardiac muscle contraction;ko05010,Alzheimer's disease;ko00190,Oxidative phosphorylation;ko05016,Huntington's disease;ko05012,Parkinson's disease;ko01100,Metabolic pathways | |
| 2360 | sp\|Q9HC52\|CBX8_HUMAN | Chromobox protein homolog 8 OS=Homo sapiens GN=CBX8 PE=1 SV=3 | / | up | 1.5193 | 0.00917 | / | / | |
| 2382 | tr\|F8W9X7\|F8W9X7_HUMAN | Coiled-coil domain-containing protein 93 OS=Homo sapiens GN=CCDC93 PE=1 SV=1 | sp\|Q567U6\|CCD93_HUMAN | up | 2.2468 | 0.03857 | / | / | |
| 2577 | tr\|Q1WWM3\|Q1WWM3_HUMAN | EPB41 protein (Fragment) OS=Homo sapiens GN=EPB41 PE=2 SV=1 | / | up | 2.8312 | 0.03939 | / | ko04530,Tight junction | |
| 2586 | sp\|Q9BTU6\|P4K2A_HUMAN | Phosphatidylinositol 4-kinase type 2-alpha OS=Homo sapiens GN=PI4K2A PE=1 SV=1 | / | up | 2.9031 | 0.04456 | / | ko00562,Inositol phosphate metabolism;ko04070,Phosphatidylinositol signaling system;ko01100,Metabolic pathways | |
| 2596 | sp\|Q99418\|CYH2_HUMAN | Cytohesin-2 OS=Homo sapiens GN=CYTH2 PE=1 SV=2 | / | up | 3.8030 | 0.01296 | General function prediction only ; | ko04144,Endocytosis | |
| 2691 | sp\|Q6P9B9\|INT5_HUMAN | Integrator complex subunit 5 OS=Homo sapiens GN=INTS5 PE=1 SV=1 | / | up | 1.6209 | 0.03467 | / | / | |

**C. The differently expressed proteins comparing WT to control group.**

| **N** | **Accession** | **Name** | **Homologous proteins** | **Up or down regulated** | **Expression ratio** | **P value** | **COG function classification** | **Kegg function**  **enrichment** |  |
| --- | --- | --- | --- | --- | --- | --- | --- | --- | --- |
| 4 | sp\|Q7Z406\|MYH14_HUMAN | Myosin-14 OS=Homo sapiens GN=MYH14 PE=1 SV=2 | / | up | 2.0329 | 0.00036 | Cytoskeleton ; | ko04530,Tight junction;ko05416,Viral myocarditis | |
| 6 | tr\|A0A024R1N1\|A0A024R1N1_HUMAN | Myosin, heavy polypeptide 9, non-muscle, isoform CRA_a OS=Homo sapiens GN=MYH9 PE=3 SV=1 | sp\|P35579\|MYH9_HUMAN | up | 1.6177 | 0.01542 | Cytoskeleton ; | ko04530,Tight junction;ko05416,Viral myocarditis | |
| 8 | sp\|P02545\|LMNA_HUMAN | Prelamin-A/C OS=Homo sapiens GN=LMNA PE=1 SV=1 | / | up | 3.3629 | 0.00000 | / | ko05410,Hypertrophic cardiomyopathy (HCM);ko05414,Dilated cardiomyopathy;ko05412,Arrhythmogenic right ventricular cardiomyopathy (ARVC) | |
| 9 | sp\|Q13813\|SPTN1_HUMAN | Spectrin alpha chain, non-erythrocytic 1 OS=Homo sapiens GN=SPTAN1 PE=1 SV=3 | / | up | 1.7891 | 0.00000 | Signal transduction mechanisms ; Cytoskeleton ; Cell cycle control, cell division, chromosome partitioning ; General function prediction only ; | / | |
| 12 | tr\|A0A024R4A0\|A0A024R4A0_HUMAN | Nucleolin, isoform CRA_b OS=Homo sapiens GN=NCL PE=4 SV=1 | sp\|P19338\|NUCL_HUMAN | up | 1.9102 | 0.01479 | General function prediction only ; | ko05130,Pathogenic Escherichia coli infection | |
| 15 | tr\|A0A0S2Z3G9\|A0A0S2Z3G9_HUMAN | Actinin alpha 4 isoform 1 (Fragment) OS=Homo sapiens GN=ACTN4 PE=2 SV=1 | sp\|O43707\|ACTN4_HUMAN | up | 3.3141 | 0.00001 | Signal transduction mechanisms ; Cytoskeleton ; Cell cycle control, cell division, chromosome partitioning ; General function prediction only ;Cytoskeleton ; | ko04520,Adherens junction;ko04530,Tight junction;ko04810,Regulation of actin cytoskeleton;ko04670,Leukocyte transendothelial migration;ko05322,Systemic lupus erythematosus;ko04510,Focal adhesion;ko05146,Amoebiasis;ko05412,Arrhythmogenic right ventricular cardiomyopathy (ARVC) | |
| 16 | tr\|A0A024RC65\|A0A024RC65_HUMAN | HCG1991735, isoform CRA_a OS=Homo sapiens GN=hCG_1991735 PE=4 SV=1 | sp\|P46940\|IQGA1_HUMAN | up | 2.2574 | 0.00025 | Cell cycle control, cell division, chromosome partitioning ; Signal transduction mechanisms ; | ko04810,Regulation of actin cytoskeleton | |
| 18 | tr\|D6W5C0\|D6W5C0_HUMAN | Spectrin, beta, non-erythrocytic 1, isoform CRA_b OS=Homo sapiens GN=SPTBN1 PE=4 SV=1 | / | up | 1.7370 | 0.00381 | Cytoskeleton ; | / | |
| 19 | tr\|V9HW22\|V9HW22_HUMAN | Epididymis luminal protein 33 OS=Homo sapiens GN=HEL-S-72p PE=2 SV=1 | sp\|P11142\|HSP7C_HUMAN | up | 1.6096 | 0.00103 | Posttranslational modification, protein turnover, chaperones ; | ko05145,Toxoplasmosis;ko04144,Endocytosis;ko04010,MAPK signaling pathway;ko03040,Spliceosome;ko04612,Antigen processing and presentation;ko04141,Protein processing in endoplasmic reticulum | |
| 21 | tr\|A0A024RD80\|A0A024RD80_HUMAN | Heat shock protein 90kDa alpha (Cytosolic), class B member 1, isoform CRA_a OS=Homo sapiens GN=HSP90AB1 PE=3 SV=1 | sp\|P08238\|HS90B_HUMAN | up | 2.4682 | 0.00234 | Posttranslational modification, protein turnover, chaperones ; | ko04621,NOD-like receptor signaling pathway;ko05200,Pathways in cancer;ko04626,Plant-pathogen interaction;ko04612,Antigen processing and presentation;ko04914,Progesterone-mediated oocyte maturation;ko05215,Prostate cancer;ko04141,Protein processing in endoplasmic reticulum | |
| 23 | sp\|P20700\|LMNB1_HUMAN | Lamin-B1 OS=Homo sapiens GN=LMNB1 PE=1 SV=2 | / | up | 1.7177 | 0.03684 | / | / | |
| 25 | tr\|V9HW80\|V9HW80_HUMAN | Epididymis luminal protein 220 OS=Homo sapiens GN=HEL-S-70 PE=2 SV=1 | sp\|P55072\|TERA_HUMAN | up | 3.2073 | 0.00011 | Posttranslational modification, protein turnover, chaperones ; | ko04141,Protein processing in endoplasmic reticulum | |
| 26 | tr\|V9HWB4\|V9HWB4_HUMAN | Epididymis secretory sperm binding protein Li 89n OS=Homo sapiens GN=HEL-S-89n PE=2 SV=1 | sp\|P11021\|GRP78_HUMAN | up | 2.2525 | 0.00000 | Posttranslational modification, protein turnover, chaperones ; | ko03060,Protein export;ko05020,Prion diseases;ko04141,Protein processing in endoplasmic reticulum | |
| 27 | tr\|V9HVY3\|V9HVY3_HUMAN | Protein disulfide-isomerase OS=Homo sapiens GN=HEL-S-269 PE=2 SV=1 | sp\|P30101\|PDIA3_HUMAN | up | 2.3408 | 0.00000 | Posttranslational modification, protein turnover, chaperones ; Energy production and conversion ; | ko04612,Antigen processing and presentation;ko04141,Protein processing in endoplasmic reticulum | |
| 28 | tr\|A0A024R4F1\|A0A024R4F1_HUMAN | Enolase 1, (Alpha), isoform CRA_a OS=Homo sapiens GN=ENO1 PE=2 SV=1 | sp\|P06733\|ENOA_HUMAN | up | 2.3564 | 0.00055 | Carbohydrate transport and metabolism ; | ko00010,Glycolysis / Gluconeogenesis;ko01120,Microbial metabolism in diverse environments;ko01110,Biosynthesis of secondary metabolites;ko01100,Metabolic pathways;ko00680,Methane metabolism;ko03018,RNA degradation | |
| 31 | tr\|V9HWC0\|V9HWC0_HUMAN | Epididymis luminal protein 70 OS=Homo sapiens GN=HEL70 PE=2 SV=1 | sp\|P26038\|MOES_HUMAN | up | 2.4752 | 0.00276 | / | ko04810,Regulation of actin cytoskeleton;ko04670,Leukocyte transendothelial migration | |
| 32 | tr\|V9HWF4\|V9HWF4_HUMAN | Phosphoglycerate kinase OS=Homo sapiens GN=HEL-S-68p PE=2 SV=1 | sp\|P00558\|PGK1_HUMAN | up | 2.2483 | 0.02371 | Carbohydrate transport and metabolism ; | ko00010,Glycolysis / Gluconeogenesis;ko01120,Microbial metabolism in diverse environments;ko00710,Carbon fixation in photosynthetic organisms;ko01110,Biosynthesis of secondary metabolites;ko01100,Metabolic pathways | |
| 33 | sp\|P35900\|K1C20_HUMAN | Keratin, type I cytoskeletal 20 OS=Homo sapiens GN=KRT20 PE=1 SV=1 | / | up | 2.9017 | 0.00764 | / | / | |
| 34 | tr\|A0A090N8Y2\|A0A090N8Y2_HUMAN | Protein disulfide-isomerase A4 OS=Homo sapiens GN=ERP70 PE=2 SV=1 | sp\|P13667\|PDIA4_HUMAN | up | 3.5065 | 0.00001 | Posttranslational modification, protein turnover, chaperones ; Energy production and conversion ; | ko05110,Vibrio cholerae infection;ko04141,Protein processing in endoplasmic reticulum | |
| 36 | tr\|A8K5I0\|A8K5I0_HUMAN | Epididymis secretory protein Li 103 OS=Homo sapiens GN=HEL-S-103 PE=2 SV=1 | tr\|A0A0G2JIW1\|A0A0G2JIW1_HUMAN;sp\|P0DMV9\|HS71B_HUMAN;sp\|P0DMV8\|HS71A_HUMAN | up | 2.2517 | 0.00015 | Posttranslational modification, protein turnover, chaperones ; | ko05145,Toxoplasmosis;ko04144,Endocytosis;ko04010,MAPK signaling pathway;ko03040,Spliceosome;ko04612,Antigen processing and presentation;ko04141,Protein processing in endoplasmic reticulum | |
| 38 | tr\|Q5CAQ5\|Q5CAQ5_HUMAN | Tumor rejection antigen (Gp96) 1 OS=Homo sapiens GN=TRA1 PE=2 SV=1 | / | up | 2.2396 | 0.00089 | Posttranslational modification, protein turnover, chaperones ; | ko04621,NOD-like receptor signaling pathway;ko05200,Pathways in cancer;ko04626,Plant-pathogen interaction;ko05215,Prostate cancer;ko04141,Protein processing in endoplasmic reticulum | |
| 39 | tr\|A0A024R8S5\|A0A024R8S5_HUMAN | Protein disulfide-isomerase OS=Homo sapiens GN=P4HB PE=2 SV=1 | sp\|P07237\|PDIA1_HUMAN | up | 2.6203 | 0.00000 | Posttranslational modification, protein turnover, chaperones ; Energy production and conversion ; | ko04141,Protein processing in endoplasmic reticulum | |
| 42 | sp\|P11216\|PYGB_HUMAN | Glycogen phosphorylase, brain form OS=Homo sapiens GN=PYGB PE=1 SV=5 | / | up | 2.0655 | 0.00839 | Carbohydrate transport and metabolism ; | ko04910,Insulin signaling pathway;ko00500,Starch and sucrose metabolism | |
| 45 | tr\|Q8N1C8\|Q8N1C8_HUMAN | HSPA9 protein (Fragment) OS=Homo sapiens GN=HSPA9 PE=2 SV=1 | / | up | 2.1836 | 0.00009 | Posttranslational modification, protein turnover, chaperones ; | ko03018,RNA degradation | |
| 48 | sp\|Q00610\|CLH1_HUMAN | Clathrin heavy chain 1 OS=Homo sapiens GN=CLTC PE=1 SV=5 | / | up | 1.9912 | 0.00329 | / | ko04144,Endocytosis;ko05100,Bacterial invasion of epithelial cells;ko05016,Huntington's disease;ko04142,Lysosome | |
| 50 | tr\|B4DLR3\|B4DLR3_HUMAN | cDNA FLJ54020, highly similar to Heterogeneous nuclear ribonucleoprotein U OS=Homo sapiens PE=2 SV=1 | / | up | 1.8720 | 0.00987 | / | ko03040,Spliceosome | |
| 51 | tr\|Q53EM5\|Q53EM5_HUMAN | Transketolase (Fragment) OS=Homo sapiens PE=2 SV=1 | tr\|V9HWD9\|V9HWD9_HUMAN;sp\|P29401\|TKT_HUMAN | up | 3.7466 | 0.00130 | Carbohydrate transport and metabolism ; | ko01120,Microbial metabolism in diverse environments;ko00710,Carbon fixation in photosynthetic organisms;ko01110,Biosynthesis of secondary metabolites;ko00030,Pentose phosphate pathway;ko01051,Biosynthesis of ansamycins;ko01100,Metabolic pathways | |
| 52 | tr\|Q5TZZ9\|Q5TZZ9_HUMAN | Annexin OS=Homo sapiens GN=ANXA1 PE=2 SV=1 | sp\|P04083\|ANXA1_HUMAN | up | 2.1435 | 0.00038 | / | / | |
| 55 | tr\|E7EQR4\|E7EQR4_HUMAN | Ezrin OS=Homo sapiens GN=EZR PE=1 SV=3 | sp\|P15311\|EZRI_HUMAN | up | 1.9152 | 0.01206 | / | ko04810,Regulation of actin cytoskeleton;ko04670,Leukocyte transendothelial migration;ko05130,Pathogenic Escherichia coli infection;ko04971,Gastric acid secretion | |
| 56 | tr\|B7ZLD5\|B7ZLD5_HUMAN | Integrin beta OS=Homo sapiens GN=ITGB4 PE=2 SV=1 | tr\|B7ZLD8\|B7ZLD8_HUMAN;tr\|A0A024R8K7\|A0A024R8K7_HUMAN | up | 1.5897 | 0.03139 | / | ko05410,Hypertrophic cardiomyopathy (HCM);ko04810,Regulation of actin cytoskeleton;ko05414,Dilated cardiomyopathy;ko04510,Focal adhesion;ko04512,ECM-receptor interaction;ko05412,Arrhythmogenic right ventricular cardiomyopathy (ARVC) | |
| 57 | tr\|A0A024R1A3\|A0A024R1A3_HUMAN | Testicular secretory protein Li 63 OS=Homo sapiens GN=UBE1 PE=2 SV=1 | sp\|P22314\|UBA1_HUMAN | up | 1.9693 | 0.00807 | Coenzyme transport and metabolism ; | ko04120,Ubiquitin mediated proteolysis;ko05012,Parkinson's disease | |
| 58 | tr\|V9HVZ4\|V9HVZ4_HUMAN | Glyceraldehyde-3-phosphate dehydrogenase OS=Homo sapiens GN=HEL-S-162eP PE=2 SV=1 | sp\|P04406\|G3P_HUMAN | up | 2.8730 | 0.00003 | Carbohydrate transport and metabolism ; | ko05010,Alzheimer's disease;ko00010,Glycolysis / Gluconeogenesis;ko01120,Microbial metabolism in diverse environments;ko01110,Biosynthesis of secondary metabolites;ko01100,Metabolic pathways | |
| 63 | sp\|P60174\|TPIS_HUMAN | Triosephosphate isomerase OS=Homo sapiens GN=TPI1 PE=1 SV=3 | / | up | 4.7432 | 0.00020 | Carbohydrate transport and metabolism ; | ko00051,Fructose and mannose metabolism;ko00562,Inositol phosphate metabolism;ko00010,Glycolysis / Gluconeogenesis;ko01120,Microbial metabolism in diverse environments;ko00710,Carbon fixation in photosynthetic organisms;ko01110,Biosynthesis of secondary metabolites;ko01100,Metabolic pathways | |
| 64 | tr\|Q6FHZ0\|Q6FHZ0_HUMAN | Malate dehydrogenase OS=Homo sapiens GN=MDH2 PE=2 SV=1 | tr\|A0A024R4K3\|A0A024R4K3_HUMAN;sp\|P40926\|MDHM_HUMAN;tr\|Q75MT9\|Q75MT9_HUMAN | up | 1.9624 | 0.00068 | Energy production and conversion ; | ko00630,Glyoxylate and dicarboxylate metabolism;ko01120,Microbial metabolism in diverse environments;ko00710,Carbon fixation in photosynthetic organisms;ko00020,Citrate cycle (TCA cycle);ko01110,Biosynthesis of secondary metabolites;ko01100,Metabolic pathways;ko00620,Pyruvate metabolism | |
| 65 | tr\|V9HW72\|V9HW72_HUMAN | Epididymis secretory sperm binding protein Li 94n OS=Homo sapiens GN=HEL-S-94n PE=2 SV=1 | tr\|A8K690\|A8K690_HUMAN;sp\|P31948\|STIP1_HUMAN | up | 1.9256 | 0.00937 | General function prediction only ; | ko05020,Prion diseases | |
| 66 | tr\|V9HW59\|V9HW59_HUMAN | Annexin OS=Homo sapiens GN=HEL-S-274 PE=2 SV=1 | tr\|Q6LES2\|Q6LES2_HUMAN;sp\|P09525\|ANXA4_HUMAN | up | 1.7131 | 0.00311 | / | / | |
| 70 | tr\|E9KL48\|E9KL48_HUMAN | Epididymis tissue sperm binding protein Li 18mP OS=Homo sapiens GN=GLUD1 PE=2 SV=1 | sp\|P00367\|DHE3_HUMAN | up | 2.4090 | 0.00657 | Amino acid transport and metabolism ; | ko00471,D-Glutamine and D-glutamate metabolism;ko04964,Proximal tubule bicarbonate reclamation;ko00250,Alanine, aspartate and glutamate metabolism;ko00910,Nitrogen metabolism;ko01100,Metabolic pathways;ko00330,Arginine and proline metabolism | |
| 74 | tr\|B4E0X8\|B4E0X8_HUMAN | cDNA FLJ61021, highly similar to Far upstream element-binding protein 1 OS=Homo sapiens PE=2 SV=1 | / | up | 2.2299 | 0.02206 | / | / | |
| 77 | tr\|V9HW26\|V9HW26_HUMAN | ATP synthase subunit alpha OS=Homo sapiens GN=HEL-S-123m PE=2 SV=1 | sp\|P25705\|ATPA_HUMAN | up | 1.9895 | 0.00013 | Energy production and conversion ; | ko05010,Alzheimer's disease;ko00190,Oxidative phosphorylation;ko05016,Huntington's disease;ko05012,Parkinson's disease;ko01100,Metabolic pathways | |
| 78 | sp\|Q92841\|DDX17_HUMAN | Probable ATP-dependent RNA helicase DDX17 OS=Homo sapiens GN=DDX17 PE=1 SV=2 | / | up | 1.7101 | 0.03800 | Replication, recombination and repair ; Transcription ; Translation, ribosomal structure and biogenesis ; | / | |
| 79 | tr\|Q53HM9\|Q53HM9_HUMAN | Eukaryotic translation elongation factor 1 alpha 1 variant (Fragment) OS=Homo sapiens PE=2 SV=1 | tr\|Q6IPT9\|Q6IPT9_HUMAN;tr\|Q6IPS9\|Q6IPS9_HUMAN;sp\|P68104\|EF1A1_HUMAN | up | 2.9515 | 0.00508 | Translation, ribosomal structure and biogenesis ; | ko03013,RNA transport | |
| 86 | sp\|Q14204\|DYHC1_HUMAN | Cytoplasmic dynein 1 heavy chain 1 OS=Homo sapiens GN=DYNC1H1 PE=1 SV=5 | / | up | 2.1813 | 0.01058 | Cytoskeleton ; | ko04962,Vasopressin-regulated water reabsorption;ko04145,Phagosome | |
| 89 | tr\|V9HW96\|V9HW96_HUMAN | Chaperonin containing TCP1, subunit 2 (Beta), isoform CRA_b OS=Homo sapiens GN=HEL-S-100n PE=2 SV=1 | sp\|P78371\|TCPB_HUMAN | up | 1.6016 | 0.03077 | Posttranslational modification, protein turnover, chaperones ; | / | |
| 91 | tr\|Q6NXR8\|Q6NXR8_HUMAN | 40S ribosomal protein S3a OS=Homo sapiens GN=RPS3A PE=2 SV=1 | sp\|P61247\|RS3A_HUMAN | up | 1.9281 | 0.01696 | Translation, ribosomal structure and biogenesis ; | ko03010,Ribosome | |
| 92 | sp\|Q02790\|FKBP4_HUMAN | Peptidyl-prolyl cis-trans isomerase FKBP4 OS=Homo sapiens GN=FKBP4 PE=1 SV=3 | / | up | 1.7583 | 0.00520 | Posttranslational modification, protein turnover, chaperones ; | / | |
| 93 | tr\|V9HWB9\|V9HWB9_HUMAN | L-lactate dehydrogenase OS=Homo sapiens GN=HEL-S-133P PE=2 SV=1 | sp\|P00338\|LDHA_HUMAN | up | 3.4634 | 0.02500 | Energy production and conversion ; | ko00010,Glycolysis / Gluconeogenesis;ko01120,Microbial metabolism in diverse environments;ko00270,Cysteine and methionine metabolism;ko01110,Biosynthesis of secondary metabolites;ko01100,Metabolic pathways;ko00640,Propanoate metabolism;ko00620,Pyruvate metabolism | |
| 95 | tr\|A0A0S2Z491\|A0A0S2Z491_HUMAN | Nucleophosmin isoform 2 (Fragment) OS=Homo sapiens GN=NPM1 PE=2 SV=1 | sp\|P06748\|NPM_HUMAN | up | 2.9812 | 0.00251 | / | / | |
| 96 | tr\|B2R659\|B2R659_HUMAN | cDNA, FLJ92803, highly similar to Homo sapiens hydroxysteroid (17-beta) dehydrogenase 4 (HSD17B4), mRNA OS=Homo sapiens PE=2 SV=1 | tr\|A0A0S2Z4J1\|A0A0S2Z4J1_HUMAN;sp\|P51659\|DHB4_HUMAN | up | 1.8195 | 0.00073 | Lipid transport and metabolism ; Secondary metabolites biosynthesis, transport and catabolism ; General function prediction only ;Lipid transport and metabolism ; | ko04146,Peroxisome;ko01100,Metabolic pathways;ko00120,Primary bile acid biosynthesis | |
| 98 | tr\|E9KL44\|E9KL44_HUMAN | Epididymis tissue sperm binding protein Li 14m OS=Homo sapiens PE=2 SV=1 | sp\|P40939\|ECHA_HUMAN | up | 1.5532 | 0.01659 | Lipid transport and metabolism ; | ko00310,Lysine degradation;ko01120,Microbial metabolism in diverse environments;ko00280,Valine, leucine and isoleucine degradation;ko00071,Fatty acid metabolism;ko00362,Benzoate degradation;ko01040,Biosynthesis of unsaturated fatty acids;ko00930,Caprolactam degradation;ko01110,Biosynthesis of secondary metabolites;ko00410,beta-Alanine metabolism;ko01100,Metabolic pathways;ko00640,Propanoate metabolism;ko00062,Fatty acid elongation in mitochondria;ko00627,Aminobenzoate degradation;ko00650,Butanoate metabolism;ko00380,Tryptophan metabolism | |
| 99 | tr\|E1NZA1\|E1NZA1_HUMAN | Peroxisome proliferator activated receptor interacting complex protein OS=Homo sapiens GN=PRIC295 PE=2 SV=1 | / | up | 1.5190 | 0.00162 | / | / | |
| 102 | tr\|V9HW31\|V9HW31_HUMAN | ATP synthase subunit beta OS=Homo sapiens GN=HEL-S-271 PE=2 SV=1 | sp\|P06576\|ATPB_HUMAN | up | 1.9046 | 0.00186 | Energy production and conversion ; | ko05010,Alzheimer's disease;ko00190,Oxidative phosphorylation;ko05016,Huntington's disease;ko05012,Parkinson's disease;ko01100,Metabolic pathways | |
| 103 | tr\|A0A024RDQ0\|A0A024RDQ0_HUMAN | Heat shock 105kDa/110kDa protein 1, isoform CRA_a OS=Homo sapiens GN=HSPH1 PE=3 SV=1 | / | up | 1.8839 | 0.03064 | Posttranslational modification, protein turnover, chaperones ; | ko04141,Protein processing in endoplasmic reticulum | |
| 106 | tr\|A0A0S2Z3Y1\|A0A0S2Z3Y1_HUMAN | Lectin galactoside-binding soluble 3 binding protein isoform 1 (Fragment) OS=Homo sapiens GN=LGALS3BP PE=2 SV=1 | sp\|Q08380\|LG3BP_HUMAN | up | 1.9609 | 0.02231 | / | ko04970,Salivary secretion | |
| 116 | tr\|Q53GG0\|Q53GG0_HUMAN | Epithelial protein lost in neoplasm beta variant (Fragment) OS=Homo sapiens PE=2 SV=1 | sp\|Q9UHB6\|LIMA1_HUMAN | up | 1.8261 | 0.02415 | / | / | |
| 117 | sp\|Q99623\|PHB2_HUMAN | Prohibitin-2 OS=Homo sapiens GN=PHB2 PE=1 SV=2 | / | up | 1.6254 | 0.02707 | Posttranslational modification, protein turnover, chaperones ; | / | |
| 118 | sp\|P39023\|RL3_HUMAN | 60S ribosomal protein L3 OS=Homo sapiens GN=RPL3 PE=1 SV=2 | / | up | 2.1603 | 0.00403 | Translation, ribosomal structure and biogenesis ; | ko03010,Ribosome | |
| 119 | sp\|P22626\|ROA2_HUMAN | Heterogeneous nuclear ribonucleoproteins A2/B1 OS=Homo sapiens GN=HNRNPA2B1 PE=1 SV=2 | / | up | 3.0157 | 0.00637 | General function prediction only ; | / | |
| 121 | tr\|A0A024R8V0\|A0A024R8V0_HUMAN | Septin 9, isoform CRA_a OS=Homo sapiens GN=SEPT9 PE=3 SV=1 | / | up | 1.6241 | 0.02850 | Cell cycle control, cell division, chromosome partitioning ; Cytoskeleton ; | ko05012,Parkinson's disease | |
| 125 | tr\|V9HWG3\|V9HWG3_HUMAN | Epididymis secretory protein Li 45 OS=Homo sapiens GN=HEL-S-45 PE=2 SV=1 | sp\|P21980\|TGM2_HUMAN | up | 2.6121 | 0.00016 | / | ko05016,Huntington's disease | |
| 126 | tr\|K9JA46\|K9JA46_HUMAN | Epididymis luminal secretory protein 52 OS=Homo sapiens GN=EL52 PE=2 SV=1 | sp\|P07900\|HS90A_HUMAN | up | 3.8366 | 0.00000 | Posttranslational modification, protein turnover, chaperones ; | ko04621,NOD-like receptor signaling pathway;ko05200,Pathways in cancer;ko04626,Plant-pathogen interaction;ko04612,Antigen processing and presentation;ko04914,Progesterone-mediated oocyte maturation;ko05215,Prostate cancer;ko04141,Protein processing in endoplasmic reticulum | |
| 127 | sp\|P50991\|TCPD_HUMAN | T-complex protein 1 subunit delta OS=Homo sapiens GN=CCT4 PE=1 SV=4 | / | up | 1.5828 | 0.00406 | Posttranslational modification, protein turnover, chaperones ; | / | |
| 128 | tr\|A0A087X054\|A0A087X054_HUMAN | Hypoxia up-regulated protein 1 OS=Homo sapiens GN=HYOU1 PE=1 SV=1 | / | up | 1.8431 | 0.00413 | Posttranslational modification, protein turnover, chaperones ; | ko04141,Protein processing in endoplasmic reticulum | |
| 130 | tr\|V9HWJ2\|V9HWJ2_HUMAN | Isocitrate dehydrogenase [NADP] OS=Homo sapiens GN=HEL-S-26 PE=2 SV=1 | tr\|B2R5M8\|B2R5M8_HUMAN;sp\|O75874\|IDHC_HUMAN | up | 1.5346 | 0.03138 | Energy production and conversion ; | ko00480,Glutathione metabolism;ko04146,Peroxisome;ko01120,Microbial metabolism in diverse environments;ko00020,Citrate cycle (TCA cycle);ko01110,Biosynthesis of secondary metabolites;ko00720,Reductive carboxylate cycle (CO2 fixation);ko01100,Metabolic pathways | |
| 135 | sp\|P14866\|HNRPL_HUMAN | Heterogeneous nuclear ribonucleoprotein L OS=Homo sapiens GN=HNRNPL PE=1 SV=2 | / | up | 1.7175 | 0.00775 | / | / | |
| 136 | tr\|Q53YD7\|Q53YD7_HUMAN | EEF1G protein OS=Homo sapiens GN=EEF1G PE=2 SV=1 | sp\|P26641\|EF1G_HUMAN | up | 2.1305 | 0.01964 | Posttranslational modification, protein turnover, chaperones ; | / | |
| 137 | sp\|O43143\|DHX15_HUMAN | Pre-mRNA-splicing factor ATP-dependent RNA helicase DHX15 OS=Homo sapiens GN=DHX15 PE=1 SV=2 | / | up | 1.7586 | 0.03593 | Replication, recombination and repair ; | ko03040,Spliceosome | |
| 140 | sp\|P23396\|RS3_HUMAN | 40S ribosomal protein S3 OS=Homo sapiens GN=RPS3 PE=1 SV=2 | / | up | 1.7041 | 0.02364 | Translation, ribosomal structure and biogenesis ; | ko03010,Ribosome | |
| 149 | tr\|B2R4R0\|B2R4R0_HUMAN | Histone H4 OS=Homo sapiens GN=HIST1H4L PE=2 SV=1 | sp\|P62805\|H4_HUMAN | up | 2.8905 | 0.00321 | Chromatin structure and dynamics ; | ko05322,Systemic lupus erythematosus | |
| 151 | sp\|P49411\|EFTU_HUMAN | Elongation factor Tu, mitochondrial OS=Homo sapiens GN=TUFM PE=1 SV=2 | / | up | 1.8788 | 0.00284 | Translation, ribosomal structure and biogenesis ; | ko04626,Plant-pathogen interaction | |
| 154 | sp\|P49588\|SYAC_HUMAN | Alanine--tRNA ligase, cytoplasmic OS=Homo sapiens GN=AARS PE=1 SV=2 | / | up | 2.6136 | 0.00928 | Translation, ribosomal structure and biogenesis ; | ko00970,Aminoacyl-tRNA biosynthesis | |
| 155 | tr\|B2R491\|B2R491_HUMAN | 40S ribosomal protein S4 OS=Homo sapiens GN=RPS4X PE=2 SV=1 | sp\|P62701\|RS4X_HUMAN;tr\|Q96IR1\|Q96IR1_HUMAN | up | 2.5601 | 0.00107 | Translation, ribosomal structure and biogenesis ; | ko03010,Ribosome | |
| 156 | sp\|O75643\|U520_HUMAN | U5 small nuclear ribonucleoprotein 200 kDa helicase OS=Homo sapiens GN=SNRNP200 PE=1 SV=2 | / | up | 1.6375 | 0.02128 | General function prediction only ; | ko03040,Spliceosome | |
| 157 | tr\|Q53GX7\|Q53GX7_HUMAN | Threonyl-tRNA synthetase variant (Fragment) OS=Homo sapiens PE=2 SV=1 | sp\|P26639\|SYTC_HUMAN | up | 1.7016 | 0.02375 | Translation, ribosomal structure and biogenesis ; | ko00970,Aminoacyl-tRNA biosynthesis | |
| 158 | sp\|Q15084\|PDIA6_HUMAN | Protein disulfide-isomerase A6 OS=Homo sapiens GN=PDIA6 PE=1 SV=1 | / | up | 2.3359 | 0.00307 | Posttranslational modification, protein turnover, chaperones ; Energy production and conversion ; | ko04141,Protein processing in endoplasmic reticulum | |
| 162 | sp\|P26599\|PTBP1_HUMAN | Polypyrimidine tract-binding protein 1 OS=Homo sapiens GN=PTBP1 PE=1 SV=1 | / | down | 0.5374 | 0.02582 | / | / | |
| 165 | sp\|Q06830\|PRDX1_HUMAN | Peroxiredoxin-1 OS=Homo sapiens GN=PRDX1 PE=1 SV=1 | / | up | 2.7106 | 0.00077 | Posttranslational modification, protein turnover, chaperones ; | ko04146,Peroxisome | |
| 169 | tr\|V9HWI5\|V9HWI5_HUMAN | Cofilin 1 (Non-muscle), isoform CRA_b OS=Homo sapiens GN=HEL-S-15 PE=2 SV=1 | sp\|P23528\|COF1_HUMAN;tr\|E9PK25\|E9PK25_HUMAN | up | 2.8302 | 0.00058 | / | ko04360,Axon guidance;ko04810,Regulation of actin cytoskeleton;ko04666,Fc gamma R-mediated phagocytosis | |
| 170 | tr\|Q59EK6\|Q59EK6_HUMAN | TNF receptor-associated protein 1 variant (Fragment) OS=Homo sapiens PE=3 SV=1 | tr\|A0A140VJY2\|A0A140VJY2_HUMAN;sp\|Q12931\|TRAP1_HUMAN | up | 2.6099 | 0.00069 | Posttranslational modification, protein turnover, chaperones ; | / | |
| 171 | sp\|P12429\|ANXA3_HUMAN | Annexin A3 OS=Homo sapiens GN=ANXA3 PE=1 SV=3 | / | up | 1.7073 | 0.03150 | / | / | |
| 172 | tr\|A0A024R1N4\|A0A024R1N4_HUMAN | X-ray repair complementing defective repair in Chinese hamster cells 6 (Ku autoantigen, 70kDa), isoform CRA_a OS=Homo sapiens GN=XRCC6 PE=4 SV=1 | sp\|P12956\|XRCC6_HUMAN | up | 1.8391 | 0.00632 | / | ko03450,Non-homologous end-joining | |
| 175 | tr\|Q5U077\|Q5U077_HUMAN | L-lactate dehydrogenase OS=Homo sapiens GN=LDHB PE=2 SV=1 | sp\|P07195\|LDHB_HUMAN | up | 2.4027 | 0.00002 | Energy production and conversion ; | ko00010,Glycolysis / Gluconeogenesis;ko01120,Microbial metabolism in diverse environments;ko00270,Cysteine and methionine metabolism;ko01110,Biosynthesis of secondary metabolites;ko01100,Metabolic pathways;ko00640,Propanoate metabolism;ko00620,Pyruvate metabolism | |
| 178 | tr\|V9HW88\|V9HW88_HUMAN | Calreticulin, isoform CRA_b OS=Homo sapiens GN=HEL-S-99n PE=2 SV=1 | sp\|P27797\|CALR_HUMAN | up | 2.9330 | 0.00741 | / | ko05142,Chagas disease;ko04612,Antigen processing and presentation;ko04145,Phagosome;ko04141,Protein processing in endoplasmic reticulum | |
| 182 | tr\|A0A140VK56\|A0A140VK56_HUMAN | Transaldolase OS=Homo sapiens PE=2 SV=1 | sp\|P37837\|TALDO_HUMAN | up | 2.6347 | 0.00233 | Carbohydrate transport and metabolism ; | ko01120,Microbial metabolism in diverse environments;ko01110,Biosynthesis of secondary metabolites;ko00030,Pentose phosphate pathway;ko01100,Metabolic pathways | |
| 184 | tr\|A2RUM7\|A2RUM7_HUMAN | Ribosomal protein L5 OS=Homo sapiens GN=RPL5 PE=2 SV=1 | sp\|P46777\|RL5_HUMAN | up | 2.1144 | 0.00061 | Translation, ribosomal structure and biogenesis ; | ko03010,Ribosome | |
| 189 | tr\|B0YIW6\|B0YIW6_HUMAN | Archain 1, isoform CRA_a OS=Homo sapiens GN=ARCN1 PE=1 SV=1 | tr\|B0YIW5\|B0YIW5_HUMAN;sp\|P48444\|COPD_HUMAN | up | 1.9315 | 0.04355 | / | / | |
| 191 | sp\|Q9NY33\|DPP3_HUMAN | Dipeptidyl peptidase 3 OS=Homo sapiens GN=DPP3 PE=1 SV=2 | / | up | 2.1681 | 0.00079 | / | / | |
| 195 | tr\|B4DLV7\|B4DLV7_HUMAN | cDNA FLJ60299, highly similar to Rab GDP dissociation inhibitor beta OS=Homo sapiens PE=2 SV=1 | / | up | 2.4291 | 0.01837 | Posttranslational modification, protein turnover, chaperones ; | / | |
| 196 | tr\|V9HW69\|V9HW69_HUMAN | Epididymis secretory protein Li 66 OS=Homo sapiens GN=HEL-S-66 PE=2 SV=1 | tr\|B4DU58\|B4DU58_HUMAN;tr\|B2R9S4\|B2R9S4_HUMAN;sp\|P40121\|CAPG_HUMAN | up | 2.6835 | 0.00005 | / | / | |
| 198 | sp\|P35606\|COPB2_HUMAN | Coatomer subunit beta' OS=Homo sapiens GN=COPB2 PE=1 SV=2 | / | up | 1.6925 | 0.00369 | General function prediction only ; | / | |
| 202 | tr\|Q8N5Z7\|Q8N5Z7_HUMAN | 60S ribosomal protein L6 OS=Homo sapiens GN=RPL6 PE=2 SV=1 | tr\|A0A024RBK3\|A0A024RBK3_HUMAN;sp\|Q02878\|RL6_HUMAN;tr\|Q9HBB3\|Q9HBB3_HUMAN;tr\|Q8TBK5\|Q8TBK5_HUMAN | up | 2.2836 | 0.03256 | Translation, ribosomal structure and biogenesis ; | ko03010,Ribosome | |
| 203 | tr\|Q6FHG5\|Q6FHG5_HUMAN | Gamma-synuclein OS=Homo sapiens GN=SNCG PE=2 SV=1 | sp\|O76070\|SYUG_HUMAN | up | 1.9262 | 0.00826 | / | ko05010,Alzheimer's disease;ko05012,Parkinson's disease | |
| 204 | tr\|B2RDW1\|B2RDW1_HUMAN | Epididymis luminal protein 112 OS=Homo sapiens GN=RPS27A PE=2 SV=1 | sp\|P62979\|RS27A_HUMAN | up | 3.0322 | 0.02689 | Posttranslational modification, protein turnover, chaperones ;Translation, ribosomal structure and biogenesis ; | ko03010,Ribosome | |
| 214 | tr\|A0A024RAZ7\|A0A024RAZ7_HUMAN | Heterogeneous nuclear ribonucleoprotein A1, isoform CRA_b OS=Homo sapiens GN=HNRPA1 PE=4 SV=1 | sp\|P09651\|ROA1_HUMAN | up | 1.9412 | 0.00848 | General function prediction only ; | ko03040,Spliceosome | |
| 215 | sp\|P23284\|PPIB_HUMAN | Peptidyl-prolyl cis-trans isomerase B OS=Homo sapiens GN=PPIB PE=1 SV=2 | tr\|V9HWC6\|V9HWC6_HUMAN | up | 7.2293 | 0.00000 | Posttranslational modification, protein turnover, chaperones ; | / | |
| 216 | tr\|V9HW83\|V9HW83_HUMAN | Aldehyde dehydrogenase 1 family, member A1, isoform CRA_a OS=Homo sapiens GN=HEL-S-53e PE=2 SV=1 | tr\|V9HVX6\|V9HVX6_HUMAN;sp\|P00352\|AL1A1_HUMAN | up | 2.2291 | 0.00044 | Energy production and conversion ; | ko00830,Retinol metabolism;ko01100,Metabolic pathways | |
| 224 | tr\|V9HWH1\|V9HWH1_HUMAN | Epididymis luminal protein 57 OS=Homo sapiens GN=HEL57 PE=2 SV=1 | tr\|B4E3A8\|B4E3A8_HUMAN;sp\|P30740\|ILEU_HUMAN | up | 2.9142 | 0.04683 | Posttranslational modification, protein turnover, chaperones ; | ko05146,Amoebiasis | |
| 225 | tr\|V9HW98\|V9HW98_HUMAN | Epididymis luminal protein 2 OS=Homo sapiens GN=HEL2 PE=2 SV=1 | sp\|P62258\|1433E_HUMAN | up | 3.8075 | 0.00556 | Signal transduction mechanisms ; | ko04114,Oocyte meiosis;ko04722,Neurotrophin signaling pathway;ko04110,Cell cycle | |
| 226 | tr\|Q53G72\|Q53G72_HUMAN | B-cell receptor-associated protein 31 variant (Fragment) OS=Homo sapiens PE=2 SV=1 | sp\|P51572\|BAP31_HUMAN | up | 3.0709 | 0.00298 | Function unknown ; | ko04141,Protein processing in endoplasmic reticulum | |
| 230 | sp\|P21796\|VDAC1_HUMAN | Voltage-dependent anion-selective channel protein 1 OS=Homo sapiens GN=VDAC1 PE=1 SV=2 | / | up | 2.8712 | 0.01297 | / | ko04020,Calcium signaling pathway;ko05016,Huntington's disease;ko05012,Parkinson's disease | |
| 232 | sp\|P62081\|RS7_HUMAN | 40S ribosomal protein S7 OS=Homo sapiens GN=RPS7 PE=1 SV=1 | / | up | 1.6027 | 0.00615 | / | ko03010,Ribosome | |
| 234 | tr\|Q5U0I6\|Q5U0I6_HUMAN | H.sapiens ras-related Hrab1A protein OS=Homo sapiens GN=RAB1A PE=2 SV=1 | sp\|P62820\|RAB1A_HUMAN | up | 2.0710 | 0.01998 | General function prediction only ; | / | |
| 237 | tr\|B5BUB5\|B5BUB5_HUMAN | Autoantigen La (Fragment) OS=Homo sapiens GN=SSB PE=2 SV=1 | sp\|P05455\|LA_HUMAN | up | 3.6595 | 0.00014 | Posttranslational modification, protein turnover, chaperones ; Translation, ribosomal structure and biogenesis ; | ko05322,Systemic lupus erythematosus | |
| 241 | tr\|A0A024R814\|A0A024R814_HUMAN | Ribosomal protein L7, isoform CRA_a OS=Homo sapiens GN=RPL7 PE=4 SV=1 | sp\|P18124\|RL7_HUMAN | up | 1.8734 | 0.00328 | Translation, ribosomal structure and biogenesis ; | ko03010,Ribosome | |
| 258 | tr\|V9HWE9\|V9HWE9_HUMAN | Epididymis secretory protein Li 22 OS=Homo sapiens GN=HEL-S-22 PE=2 SV=1 | sp\|P09211\|GSTP1_HUMAN | up | 4.0537 | 0.04168 | Posttranslational modification, protein turnover, chaperones ; | ko00480,Glutathione metabolism;ko00982,Drug metabolism - cytochrome P450;ko00980,Metabolism of xenobiotics by cytochrome P450 | |
| 265 | sp\|P37802\|TAGL2_HUMAN | Transgelin-2 OS=Homo sapiens GN=TAGLN2 PE=1 SV=3 | / | up | 3.6206 | 0.00129 | Cytoskeleton ; | / | |
| 268 | tr\|Q4W4Y1\|Q4W4Y1_HUMAN | Dopamine receptor interacting protein 4 OS=Homo sapiens GN=DRIP4 PE=2 SV=1 | / | up | 1.7844 | 0.02554 | / | ko04144,Endocytosis | |
| 269 | sp\|P13645\|K1C10_HUMAN | Keratin, type I cytoskeletal 10 OS=Homo sapiens GN=KRT10 PE=1 SV=6 | / | up | 1.8872 | 0.00459 | / | / | |
| 275 | tr\|Q6PUJ7\|Q6PUJ7_HUMAN | Epididymis luminal protein 215 OS=Homo sapiens GN=HEL-215 PE=2 SV=1 | tr\|A8K401\|A8K401_HUMAN;sp\|P35232\|PHB_HUMAN | up | 1.7581 | 0.00367 | Posttranslational modification, protein turnover, chaperones ; | / | |
| 276 | sp\|Q07955\|SRSF1_HUMAN | Serine/arginine-rich splicing factor 1 OS=Homo sapiens GN=SRSF1 PE=1 SV=2 | tr\|J3KTL2\|J3KTL2_HUMAN | up | 2.0351 | 0.03700 | General function prediction only ; | ko03040,Spliceosome | |
| 281 | tr\|A0A024R713\|A0A024R713_HUMAN | Dihydrolipoyl dehydrogenase OS=Homo sapiens GN=DLD PE=4 SV=1 | sp\|P09622\|DLDH_HUMAN;tr\|E9PEX6\|E9PEX6_HUMAN;tr\|B4DMK9\|B4DMK9_HUMAN | up | 2.9697 | 0.02925 | Energy production and conversion ; | ko01120,Microbial metabolism in diverse environments;ko00280,Valine, leucine and isoleucine degradation;ko01110,Biosynthesis of secondary metabolites;ko01100,Metabolic pathways;ko00620,Pyruvate metabolism;ko00010,Glycolysis / Gluconeogenesis;ko00020,Citrate cycle (TCA cycle);ko00260,Glycine, serine and threonine metabolism | |
| 284 | tr\|Q5JR94\|Q5JR94_HUMAN | 40S ribosomal protein S8 OS=Homo sapiens GN=RPS8 PE=2 SV=1 | sp\|P62241\|RS8_HUMAN | up | 2.8338 | 0.00079 | Translation, ribosomal structure and biogenesis ; | ko03010,Ribosome | |
| 287 | tr\|E9KL35\|E9KL35_HUMAN | Epididymis tissue sperm binding protein Li 3a OS=Homo sapiens PE=1 SV=1 | sp\|P63244\|RACK1_HUMAN | up | 2.5970 | 0.04485 | General function prediction only ; | ko00565,Ether lipid metabolism;ko01100,Metabolic pathways | |
| 288 | tr\|Q7RU04\|Q7RU04_HUMAN | Aminopeptidase B OS=Homo sapiens GN=RNPEP PE=4 SV=1 | sp\|Q9H4A4\|AMPB_HUMAN | down | 0.6584 | 0.04076 | Amino acid transport and metabolism ; | / | |
| 289 | tr\|A0A0S2Z4R1\|A0A0S2Z4R1_HUMAN | Tyrosine--tRNA ligase (Fragment) OS=Homo sapiens GN=YARS PE=2 SV=1 | sp\|P54577\|SYYC_HUMAN | up | 1.9407 | 0.00426 | General function prediction only ;Translation, ribosomal structure and biogenesis ; | ko00970,Aminoacyl-tRNA biosynthesis | |
| 293 | tr\|V9HWI3\|V9HWI3_HUMAN | Cathepsin D (Lysosomal aspartyl peptidase), isoform CRA_a OS=Homo sapiens GN=HEL-S-130P PE=2 SV=1 | sp\|P07339\|CATD_HUMAN | up | 1.5162 | 0.02915 | / | ko04142,Lysosome | |
| 298 | tr\|V9HW12\|V9HW12_HUMAN | Epididymis secretory sperm binding protein Li 2a OS=Homo sapiens GN=HEL-S-2a PE=2 SV=1 | sp\|P32119\|PRDX2_HUMAN | up | 1.7179 | 0.01565 | Posttranslational modification, protein turnover, chaperones ; | / | |
| 300 | sp\|P07737\|PROF1_HUMAN | Profilin-1 OS=Homo sapiens GN=PFN1 PE=1 SV=2 | / | up | 4.7282 | 0.00056 | / | ko04810,Regulation of actin cytoskeleton;ko05131,Shigellosis | |
| 309 | tr\|A0A024R1S8\|A0A024R1S8_HUMAN | LIM and SH3 protein 1, isoform CRA_b OS=Homo sapiens GN=LASP1 PE=4 SV=1 | sp\|Q14847\|LASP1_HUMAN | up | 2.0565 | 0.00936 | / | ko04530,Tight junction;ko04144,Endocytosis;ko05100,Bacterial invasion of epithelial cells;ko05130,Pathogenic Escherichia coli infection;ko05131,Shigellosis | |
| 319 | sp\|P53618\|COPB_HUMAN | Coatomer subunit beta OS=Homo sapiens GN=COPB1 PE=1 SV=3 | / | up | 1.7930 | 0.00200 | Intracellular trafficking, secretion, and vesicular transport ; | ko04142,Lysosome | |
| 325 | tr\|Q8WVX7\|Q8WVX7_HUMAN | Ribosomal protein S19 (Fragment) OS=Homo sapiens PE=2 SV=1 | tr\|B0ZBD0\|B0ZBD0_HUMAN;sp\|P39019\|RS19_HUMAN | up | 1.7975 | 0.00505 | Translation, ribosomal structure and biogenesis ; | ko03010,Ribosome | |
| 326 | sp\|Q15717\|ELAV1_HUMAN | ELAV-like protein 1 OS=Homo sapiens GN=ELAVL1 PE=1 SV=2 | / | up | 1.5759 | 0.02314 | General function prediction only ; | / | |
| 327 | tr\|G8JLA2\|G8JLA2_HUMAN | Myosin light polypeptide 6 OS=Homo sapiens GN=MYL6 PE=1 SV=1 | / | up | 2.2407 | 0.00545 | Signal transduction mechanisms ; Cytoskeleton ; Cell cycle control, cell division, chromosome partitioning ; General function prediction only ; | ko04270,Vascular smooth muscle contraction | |
| 328 | sp\|P27824\|CALX_HUMAN | Calnexin OS=Homo sapiens GN=CANX PE=1 SV=2 | / | up | 2.3254 | 0.00575 | / | ko04612,Antigen processing and presentation;ko04145,Phagosome;ko04141,Protein processing in endoplasmic reticulum | |
| 338 | tr\|B7ZAX9\|B7ZAX9_HUMAN | cDNA, FLJ79343, highly similar to SWI/SNF-related matrix-associated actin-dependent regulator of chromatin subfamily A member 5 (EC 3.6.1.-) OS=Homo sapiens PE=2 SV=1 | tr\|B4DZC0\|B4DZC0_HUMAN | up | 1.6029 | 0.00600 | Transcription ; Replication, recombination and repair ; | / | |
| 350 | sp\|P62277\|RS13_HUMAN | 40S ribosomal protein S13 OS=Homo sapiens GN=RPS13 PE=1 SV=2 | / | up | 2.8891 | 0.02950 | Translation, ribosomal structure and biogenesis ; | ko03010,Ribosome | |
| 362 | tr\|A0A140VK42\|A0A140VK42_HUMAN | Testicular secretory protein Li 42 OS=Homo sapiens PE=2 SV=1 | sp\|P17980\|PRS6A_HUMAN;tr\|R4GNH3\|R4GNH3_HUMAN | up | 1.6680 | 0.04050 | Posttranslational modification, protein turnover, chaperones ; | ko03050,Proteasome | |
| 363 | sp\|Q12929\|EPS8_HUMAN | Epidermal growth factor receptor kinase substrate 8 OS=Homo sapiens GN=EPS8 PE=1 SV=1 | / | down | 0.6048 | 0.00448 | / | ko04660,T cell receptor signaling pathway;ko05130,Pathogenic Escherichia coli infection;ko04012,ErbB signaling pathway | |
| 368 | sp\|O60701\|UGDH_HUMAN | UDP-glucose 6-dehydrogenase OS=Homo sapiens GN=UGDH PE=1 SV=1 | / | up | 1.7644 | 0.02170 | Cell wall/membrane/envelope biogenesis ; | ko01110,Biosynthesis of secondary metabolites;ko00520,Amino sugar and nucleotide sugar metabolism;ko00500,Starch and sucrose metabolism;ko01100,Metabolic pathways;ko00040,Pentose and glucuronate interconversions;ko00053,Ascorbate and aldarate metabolism | |
| 374 | tr\|V9HWC7\|V9HWC7_HUMAN | Epididymis secretory sperm binding protein Li 128m OS=Homo sapiens GN=HEL-S-128m PE=2 SV=1 | sp\|P30041\|PRDX6_HUMAN | up | 2.2676 | 0.00000 | Posttranslational modification, protein turnover, chaperones ; | ko01120,Microbial metabolism in diverse environments;ko01110,Biosynthesis of secondary metabolites;ko00360,Phenylalanine metabolism;ko01100,Metabolic pathways;ko00680,Methane metabolism;ko00940,Phenylpropanoid biosynthesis | |
| 384 | sp\|Q14444\|CAPR1_HUMAN | Caprin-1 OS=Homo sapiens GN=CAPRIN1 PE=1 SV=2 | / | up | 3.1980 | 0.01720 | / | / | |
| 385 | sp\|P41252\|SYIC_HUMAN | Isoleucine--tRNA ligase, cytoplasmic OS=Homo sapiens GN=IARS PE=1 SV=2 | / | up | 1.8280 | 0.03349 | Translation, ribosomal structure and biogenesis ; | ko00290,Valine, leucine and isoleucine biosynthesis;ko00970,Aminoacyl-tRNA biosynthesis | |
| 389 | tr\|Q6NZ55\|Q6NZ55_HUMAN | 60S ribosomal protein L13 OS=Homo sapiens GN=RPL13 PE=2 SV=1 | tr\|A8K4C8\|A8K4C8_HUMAN;sp\|P26373\|RL13_HUMAN | up | 2.0298 | 0.00341 | Translation, ribosomal structure and biogenesis ; | ko03010,Ribosome | |
| 394 | sp\|P61254\|RL26_HUMAN | 60S ribosomal protein L26 OS=Homo sapiens GN=RPL26 PE=1 SV=1 | / | up | 2.3340 | 0.02142 | Translation, ribosomal structure and biogenesis ; | ko03010,Ribosome | |
| 409 | tr\|A0A024QZN9\|A0A024QZN9_HUMAN | Voltage-dependent anion channel 2, isoform CRA_a OS=Homo sapiens GN=VDAC2 PE=4 SV=1 | tr\|A0A024QZT0\|A0A024QZT0_HUMAN;sp\|P45880\|VDAC2_HUMAN;tr\|A0A0A0MR02\|A0A0A0MR02_HUMAN | up | 2.2756 | 0.04577 | / | ko04020,Calcium signaling pathway;ko05016,Huntington's disease;ko05012,Parkinson's disease | |
| 416 | tr\|D6RD18\|D6RD18_HUMAN | Heterogeneous nuclear ribonucleoprotein A/B OS=Homo sapiens GN=HNRNPAB PE=1 SV=1 | / | up | 2.6237 | 0.00266 | General function prediction only ; | / | |
| 419 | sp\|P08727\|K1C19_HUMAN | Keratin, type I cytoskeletal 19 OS=Homo sapiens GN=KRT19 PE=1 SV=4 | / | up | 4.3267 | 0.00082 | / | / | |
| 425 | tr\|A0A024R4M0\|A0A024R4M0_HUMAN | 40S ribosomal protein S9 OS=Homo sapiens GN=RPS9 PE=1 SV=1 | sp\|P46781\|RS9_HUMAN | up | 2.2545 | 0.01641 | Translation, ribosomal structure and biogenesis ; | ko03010,Ribosome | |
| 427 | tr\|Q6FHM2\|Q6FHM2_HUMAN | GNB2 protein OS=Homo sapiens GN=GNB2 PE=2 SV=1 | sp\|P62879\|GBB2_HUMAN | down | 0.4251 | 0.03412 | General function prediction only ; | ko04062,Chemokine signaling pathway | |
| 436 | tr\|A8K4T9\|A8K4T9_HUMAN | cDNA FLJ77421, highly similar to Homo sapiens autoantigen p542 mRNA OS=Homo sapiens PE=2 SV=1 | tr\|Q5QPL9\|Q5QPL9_HUMAN;tr\|Q53GL6\|Q53GL6_HUMAN;sp\|Q9UKM9\|RALY_HUMAN | up | 2.0003 | 0.00449 | / | / | |
| 441 | sp\|P15559\|NQO1_HUMAN | NAD(P)H dehydrogenase [quinone] 1 OS=Homo sapiens GN=NQO1 PE=1 SV=1 | / | up | 3.1670 | 0.00355 | General function prediction only ; | / | |
| 445 | tr\|V9HWE8\|V9HWE8_HUMAN | Epididymis secretory sperm binding protein Li 47e OS=Homo sapiens GN=HEL-S-47e PE=2 SV=1 | sp\|P52565\|GDIR1_HUMAN | up | 2.7348 | 0.04958 | / | ko04962,Vasopressin-regulated water reabsorption;ko04722,Neurotrophin signaling pathway | |
| 449 | tr\|B3KMX0\|B3KMX0_HUMAN | DNA helicase OS=Homo sapiens PE=2 SV=1 | tr\|A0A024R7U6\|A0A024R7U6_HUMAN;sp\|P33991\|MCM4_HUMAN | down | 0.5949 | 0.02074 | Replication, recombination and repair ; | ko04113,Meiosis - yeast;ko03030,DNA replication;ko04111,Cell cycle - yeast;ko04110,Cell cycle | |
| 462 | tr\|A8K8U1\|A8K8U1_HUMAN | cDNA FLJ77762, highly similar to Homo sapiens cullin-associated and neddylation-dissociated 1 (CAND1), mRNA OS=Homo sapiens PE=2 SV=1 | sp\|Q86VP6\|CAND1_HUMAN | up | 1.6684 | 0.00141 | / | / | |
| 467 | sp\|P29373\|RABP2_HUMAN | Cellular retinoic acid-binding protein 2 OS=Homo sapiens GN=CRABP2 PE=1 SV=2 | / | up | 4.5721 | 0.00228 | / | ko03320,PPAR signaling pathway | |
| 472 | tr\|A2A3R6\|A2A3R6_HUMAN | 40S ribosomal protein S6 OS=Homo sapiens GN=RPS6 PE=2 SV=1 | sp\|P62753\|RS6_HUMAN | up | 1.6868 | 0.04387 | Translation, ribosomal structure and biogenesis ; | ko03010,Ribosome;ko04910,Insulin signaling pathway;ko04150,mTOR signaling pathway | |
| 474 | tr\|A0A024R261\|A0A024R261_HUMAN | HCG24487, isoform CRA_c OS=Homo sapiens GN=hCG_24487 PE=3 SV=1 | sp\|P18621\|RL17_HUMAN | up | 1.9341 | 0.02825 | Translation, ribosomal structure and biogenesis ; | ko03010,Ribosome | |
| 478 | sp\|Q9Y3F4\|STRAP_HUMAN | Serine-threonine kinase receptor-associated protein OS=Homo sapiens GN=STRAP PE=1 SV=1 | / | up | 1.5530 | 0.04524 | General function prediction only ; | ko03013,RNA transport | |
| 484 | tr\|B4DV28\|B4DV28_HUMAN | cDNA FLJ54170, highly similar to Cytosolic nonspecific dipeptidase OS=Homo sapiens PE=2 SV=1 | / | up | 1.7777 | 0.01874 | Amino acid transport and metabolism ; | / | |
| 514 | tr\|A0A090N7V5\|A0A090N7V5_HUMAN | Chromosome 7 open reading frame 24 OS=Homo sapiens GN=C7orf24 PE=4 SV=1 | sp\|O75223\|GGCT_HUMAN | up | 1.9358 | 0.00786 | / | ko00480,Glutathione metabolism | |
| 519 | tr\|A0A024R4Q8\|A0A024R4Q8_HUMAN | Ribosomal protein S5, isoform CRA_a OS=Homo sapiens GN=RPS5 PE=3 SV=1 | sp\|P46782\|RS5_HUMAN;tr\|M0R0F0\|M0R0F0_HUMAN | up | 1.6594 | 0.04954 | Translation, ribosomal structure and biogenesis ; | ko03010,Ribosome | |
| 523 | sp\|P62269\|RS18_HUMAN | 40S ribosomal protein S18 OS=Homo sapiens GN=RPS18 PE=1 SV=3 | / | up | 2.1712 | 0.00470 | Translation, ribosomal structure and biogenesis ; | ko03010,Ribosome | |
| 539 | sp\|P06703\|S10A6_HUMAN | Protein S100-A6 OS=Homo sapiens GN=S100A6 PE=1 SV=1 | / | up | 6.4953 | 0.00266 | / | / | |
| 543 | tr\|Q9BR63\|Q9BR63_HUMAN | FARSB protein (Fragment) OS=Homo sapiens GN=FARSB PE=2 SV=2 | sp\|Q9NSD9\|SYFB_HUMAN | up | 1.6687 | 0.00961 | Translation, ribosomal structure and biogenesis ; | ko00970,Aminoacyl-tRNA biosynthesis | |
| 545 | tr\|B4DS05\|B4DS05_HUMAN | cDNA FLJ59403, highly similar to Nucleosome assembly protein 1-like 4 OS=Homo sapiens PE=2 SV=1 | tr\|A0A024RCC9\|A0A024RCC9_HUMAN;sp\|Q99733\|NP1L4_HUMAN;tr\|B7ZB83\|B7ZB83_HUMAN;tr\|B7ZAK9\|B7ZAK9_HUMAN | up | 1.8163 | 0.04990 | / | / | |
| 557 | tr\|Q59FR8\|Q59FR8_HUMAN | Galectin (Fragment) OS=Homo sapiens PE=2 SV=1 | tr\|Q86TY5\|Q86TY5_HUMAN;tr\|Q6FGL0\|Q6FGL0_HUMAN;tr\|A0A024R693\|A0A024R693_HUMAN;sp\|P17931\|LEG3_HUMAN | up | 1.8665 | 0.03448 | / | / | |
| 569 | tr\|B3KWX7\|B3KWX7_HUMAN | cDNA FLJ44170 fis, clone THYMU2035319, highly similar to RNA-binding region-containing protein 2 OS=Homo sapiens PE=2 SV=1 | / | up | 1.9843 | 0.03821 | General function prediction only ; | / | |
| 575 | sp\|P62906\|RL10A_HUMAN | 60S ribosomal protein L10a OS=Homo sapiens GN=RPL10A PE=1 SV=2 | / | up | 2.4952 | 0.01508 | Translation, ribosomal structure and biogenesis ; | ko03010,Ribosome | |
| 577 | tr\|H0Y4R1\|H0Y4R1_HUMAN | Inosine-5'-monophosphate dehydrogenase 2 (Fragment) OS=Homo sapiens GN=IMPDH2 PE=1 SV=1 | sp\|P12268\|IMDH2_HUMAN | down | 0.5910 | 0.01919 | Nucleotide transport and metabolism ;General function prediction only ; | ko00983,Drug metabolism - other enzymes;ko01110,Biosynthesis of secondary metabolites;ko00230,Purine metabolism;ko01100,Metabolic pathways | |
| 594 | tr\|D6W5K2\|D6W5K2_HUMAN | Thymosin, beta 10, isoform CRA_a (Fragment) OS=Homo sapiens GN=TMSB10 PE=4 SV=1 | / | up | 1.9810 | 0.01851 | / | / | |
| 611 | tr\|A0A024RDG6\|A0A024RDG6_HUMAN | Scavenger receptor class B, member 2, isoform CRA_a OS=Homo sapiens GN=SCARB2 PE=3 SV=1 | sp\|Q14108\|SCRB2_HUMAN | up | 1.8116 | 0.03986 | / | ko04142,Lysosome | |
| 616 | tr\|B2R761\|B2R761_HUMAN | cDNA, FLJ93299, highly similar to Homo sapiens sterol carrier protein 2 (SCP2), mRNA OS=Homo sapiens PE=2 SV=1 | / | up | 2.7108 | 0.00123 | Lipid transport and metabolism ; | ko04146,Peroxisome;ko03320,PPAR signaling pathway;ko01100,Metabolic pathways;ko00120,Primary bile acid biosynthesis | |
| 617 | sp\|O75396\|SC22B_HUMAN | Vesicle-trafficking protein SEC22b OS=Homo sapiens GN=SEC22B PE=1 SV=4 | / | up | 2.6707 | 0.01169 | Intracellular trafficking, secretion, and vesicular transport ; | ko04130,SNARE interactions in vesicular transport;ko04145,Phagosome | |
| 633 | tr\|A0A087WV66\|A0A087WV66_HUMAN | Antigen KI-67 OS=Homo sapiens GN=MKI67 PE=1 SV=1 | sp\|P46013\|KI67_HUMAN | down | 0.4377 | 0.00431 | / | / | |
| 640 | tr\|Q8NCF7\|Q8NCF7_HUMAN | cDNA FLJ90278 fis, clone NT2RP1000325, highly similar to Phosphate carrier protein, mitochondrialprecursor OS=Homo sapiens PE=2 SV=1 | tr\|B2RE88\|B2RE88_HUMAN;tr\|A0A024RBE8\|A0A024RBE8_HUMAN | up | 1.8837 | 0.01376 | / | / | |
| 641 | tr\|J3QQ67\|J3QQ67_HUMAN | 60S ribosomal protein L18 (Fragment) OS=Homo sapiens GN=RPL18 PE=1 SV=1 | tr\|A0A024QZD1\|A0A024QZD1_HUMAN;sp\|Q07020\|RL18_HUMAN;tr\|G3V203\|G3V203_HUMAN | up | 2.1696 | 0.00964 | Translation, ribosomal structure and biogenesis ; | ko03010,Ribosome | |
| 643 | sp\|P30044\|PRDX5_HUMAN | Peroxiredoxin-5, mitochondrial OS=Homo sapiens GN=PRDX5 PE=1 SV=4 | tr\|V9HW35\|V9HW35_HUMAN | up | 2.6531 | 0.01706 | Posttranslational modification, protein turnover, chaperones ; | ko04146,Peroxisome | |
| 648 | tr\|D9IAI1\|D9IAI1_HUMAN | Epididymis secretory protein Li 34 OS=Homo sapiens GN=PEBP1 PE=2 SV=1 | sp\|P30086\|PEBP1_HUMAN | up | 4.3688 | 0.00127 | General function prediction only ; | / | |
| 661 | tr\|B2RB06\|B2RB06_HUMAN | cDNA, FLJ95242, highly similar to Homo sapiens L-3-hydroxyacyl-Coenzyme A dehydrogenase, short chain (HADHSC), mRNA OS=Homo sapiens PE=2 SV=1 | / | up | 3.0558 | 0.00674 | Lipid transport and metabolism ; | ko00310,Lysine degradation;ko00281,Geraniol degradation;ko01120,Microbial metabolism in diverse environments;ko00280,Valine, leucine and isoleucine degradation;ko00071,Fatty acid metabolism;ko00930,Caprolactam degradation;ko01110,Biosynthesis of secondary metabolites;ko01100,Metabolic pathways;ko00062,Fatty acid elongation in mitochondria;ko00650,Butanoate metabolism;ko00380,Tryptophan metabolism | |
| 674 | sp\|P11388\|TOP2A_HUMAN | DNA topoisomerase 2-alpha OS=Homo sapiens GN=TOP2A PE=1 SV=3 | / | down | 0.6662 | 0.02785 | Replication, recombination and repair ; | / | |
| 688 | sp\|Q9UJZ1\|STML2_HUMAN | Stomatin-like protein 2, mitochondrial OS=Homo sapiens GN=STOML2 PE=1 SV=1 | / | up | 1.6859 | 0.00779 | Posttranslational modification, protein turnover, chaperones ; | ko04120,Ubiquitin mediated proteolysis;ko04111,Cell cycle - yeast;ko04914,Progesterone-mediated oocyte maturation;ko04110,Cell cycle | |
| 714 | sp\|Q9NYL9\|TMOD3_HUMAN | Tropomodulin-3 OS=Homo sapiens GN=TMOD3 PE=1 SV=1 | / | up | 2.0678 | 0.00886 | / | / | |
| 719 | tr\|Q6NVW7\|Q6NVW7_HUMAN | Importin subunit alpha OS=Homo sapiens GN=KPNA2 PE=2 SV=1 | / | down | 0.5654 | 0.02219 | Intracellular trafficking, secretion, and vesicular transport ; | / | |
| 721 | sp\|Q14974\|IMB1_HUMAN | Importin subunit beta-1 OS=Homo sapiens GN=KPNB1 PE=1 SV=2 | tr\|B2RBR9\|B2RBR9_HUMAN | up | 1.6942 | 0.01993 | Intracellular trafficking, secretion, and vesicular transport ; | ko03013,RNA transport | |
| 725 | tr\|Q59G24\|Q59G24_HUMAN | Activated RNA polymerase II transcription cofactor 4 variant (Fragment) OS=Homo sapiens PE=2 SV=1 | tr\|Q6IBA2\|Q6IBA2_HUMAN;sp\|P53999\|TCP4_HUMAN | up | 1.5487 | 0.01187 | / | / | |
| 728 | sp\|O75947\|ATP5H_HUMAN | ATP synthase subunit d, mitochondrial OS=Homo sapiens GN=ATP5H PE=1 SV=3 | / | up | 2.4400 | 0.03182 | / | ko05010,Alzheimer's disease;ko00190,Oxidative phosphorylation;ko05016,Huntington's disease;ko05012,Parkinson's disease;ko01100,Metabolic pathways | |
| 730 | tr\|A8K7T4\|A8K7T4_HUMAN | cDNA FLJ75774, highly similar to Homo sapiens lectin, mannose-binding 2 (LMAN2), mRNA OS=Homo sapiens PE=2 SV=1 | sp\|Q12907\|LMAN2_HUMAN | up | 1.8024 | 0.03827 | / | ko04141,Protein processing in endoplasmic reticulum | |
| 745 | tr\|X5DNI9\|X5DNI9_HUMAN | 7-dehydrocholesterol reductase isoform A (Fragment) OS=Homo sapiens GN=DHCR7 PE=2 SV=1 | tr\|A0A024R5F7\|A0A024R5F7_HUMAN;sp\|Q9UBM7\|DHCR7_HUMAN | up | 2.7907 | 0.01007 | / | ko00100,Steroid biosynthesis;ko01110,Biosynthesis of secondary metabolites;ko01100,Metabolic pathways | |
| 755 | sp\|P27487\|DPP4_HUMAN | Dipeptidyl peptidase 4 OS=Homo sapiens GN=DPP4 PE=1 SV=2 | / | up | 1.9096 | 0.04511 | Amino acid transport and metabolism ; | ko04974,Protein digestion and absorption | |
| 758 | sp\|P54727\|RD23B_HUMAN | UV excision repair protein RAD23 homolog B OS=Homo sapiens GN=RAD23B PE=1 SV=1 | / | up | 3.8885 | 0.00240 | Posttranslational modification, protein turnover, chaperones ; | ko03420,Nucleotide excision repair;ko04141,Protein processing in endoplasmic reticulum | |
| 767 | tr\|B2RDF5\|B2RDF5_HUMAN | cDNA, FLJ96587, highly similar to Homo sapiens SUMO-1 activating enzyme subunit 2 (UBA2), mRNA OS=Homo sapiens PE=2 SV=1 | / | up | 2.1871 | 0.00508 | Coenzyme transport and metabolism ; | ko04120,Ubiquitin mediated proteolysis | |
| 770 | tr\|Q0P5N8\|Q0P5N8_HUMAN | TMSB4X protein (Fragment) OS=Homo sapiens GN=TMSB4X PE=2 SV=1 | tr\|Q0P5U7\|Q0P5U7_HUMAN;tr\|Q0P5P4\|Q0P5P4_HUMAN;tr\|Q0P5T0\|Q0P5T0_HUMAN;tr\|Q0P5Q0\|Q0P5Q0_HUMAN;tr\|A2VCK8\|A2VCK8_HUMAN;sp\|P62328\|TYB4_HUMAN | up | 8.5736 | 0.03819 | / | ko04810,Regulation of actin cytoskeleton | |
| 783 | sp\|Q13404\|UB2V1_HUMAN | Ubiquitin-conjugating enzyme E2 variant 1 OS=Homo sapiens GN=UBE2V1 PE=1 SV=2 | / | up | 4.0514 | 0.00739 | / | / | |
| 808 | tr\|F6WQW2\|F6WQW2_HUMAN | Ran-specific GTPase-activating protein OS=Homo sapiens GN=RANBP1 PE=1 SV=1 | / | up | 3.5925 | 0.04260 | Intracellular trafficking, secretion, and vesicular transport ; | ko03013,RNA transport | |
| 830 | sp\|P55036\|PSMD4_HUMAN | 26S proteasome non-ATPase regulatory subunit 4 OS=Homo sapiens GN=PSMD4 PE=1 SV=1 | / | up | 3.2171 | 0.02965 | Posttranslational modification, protein turnover, chaperones ; | ko03050,Proteasome | |
| 833 | tr\|Q5STK2\|Q5STK2_HUMAN | Prefoldin subunit 6, isoform CRA_b OS=Homo sapiens GN=PFDN6 PE=2 SV=1 | sp\|O15212\|PFD6_HUMAN | up | 1.7339 | 0.01504 | Posttranslational modification, protein turnover, chaperones ; | / | |
| 850 | tr\|C9JRZ6\|C9JRZ6_HUMAN | MICOS complex subunit OS=Homo sapiens GN=CHCHD3 PE=1 SV=1 | / | up | 1.6694 | 0.00667 | / | / | |
| 856 | sp\|P48047\|ATPO_HUMAN | ATP synthase subunit O, mitochondrial OS=Homo sapiens GN=ATP5O PE=1 SV=1 | / | up | 1.9857 | 0.04087 | Energy production and conversion ; | ko05010,Alzheimer's disease;ko00190,Oxidative phosphorylation;ko05016,Huntington's disease;ko05012,Parkinson's disease;ko01100,Metabolic pathways | |
| 868 | tr\|Q53GL5\|Q53GL5_HUMAN | Isocitrate dehydrogenase 2 (NADP+), mitochondrial variant (Fragment) OS=Homo sapiens PE=2 SV=1 | sp\|P48735\|IDHP_HUMAN | up | 3.3151 | 0.00484 | Energy production and conversion ;Energy production and conversion ; Amino acid transport and metabolism ; | ko00480,Glutathione metabolism;ko04146,Peroxisome;ko01120,Microbial metabolism in diverse environments;ko00020,Citrate cycle (TCA cycle);ko01110,Biosynthesis of secondary metabolites;ko00720,Reductive carboxylate cycle (CO2 fixation);ko01100,Metabolic pathways | |
| 886 | tr\|D3XNU5\|D3XNU5_HUMAN | E-cadherin 1 OS=Homo sapiens GN=CDH1 PE=4 SV=1 | tr\|A0A0U2ZQU7\|A0A0U2ZQU7_HUMAN;tr\|A0A0E3XJU3\|A0A0E3XJU3_HUMAN;sp\|P12830\|CADH1_HUMAN | up | 1.7826 | 0.03773 | / | ko04520,Adherens junction;ko05200,Pathways in cancer;ko04514,Cell adhesion molecules (CAMs);ko05213,Endometrial cancer;ko05100,Bacterial invasion of epithelial cells;ko05218,Melanoma;ko05130,Pathogenic Escherichia coli infection;ko05219,Bladder cancer;ko05216,Thyroid cancer | |
| 909 | tr\|A0A0S2Z5U7\|A0A0S2Z5U7_HUMAN | Diablo-like protein isoform 1 (Fragment) OS=Homo sapiens GN=DIABLO PE=2 SV=1 | sp\|Q9NR28\|DBLOH_HUMAN | up | 1.7192 | 0.04478 | / | / | |
| 918 | sp\|P25789\|PSA4_HUMAN | Proteasome subunit alpha type-4 OS=Homo sapiens GN=PSMA4 PE=1 SV=1 | / | up | 1.8465 | 0.00683 | Posttranslational modification, protein turnover, chaperones ; | ko03050,Proteasome | |
| 940 | tr\|B4DWS6\|B4DWS6_HUMAN | cDNA FLJ61181, highly similar to Homo sapiens hydroxysteroid (17-beta) dehydrogenase 12 (HSD17B12), mRNA OS=Homo sapiens PE=2 SV=1 | / | up | 3.7647 | 0.01269 | General function prediction only ; | ko00140,Steroid hormone biosynthesis;ko01040,Biosynthesis of unsaturated fatty acids;ko01100,Metabolic pathways | |
| 941 | sp\|P16070\|CD44_HUMAN | CD44 antigen OS=Homo sapiens GN=CD44 PE=1 SV=3 | tr\|A8K309\|A8K309_HUMAN;tr\|H0YD13\|H0YD13_HUMAN;tr\|H0Y2P0\|H0Y2P0_HUMAN;tr\|B4DN59\|B4DN59_HUMAN | up | 1.8154 | 0.03718 | / | ko04512,ECM-receptor interaction;ko05131,Shigellosis;ko04640,Hematopoietic cell lineage | |
| 949 | tr\|Q53G49\|Q53G49_HUMAN | Ribosomal protein L19 (Fragment) OS=Homo sapiens PE=2 SV=1 | tr\|J3QR09\|J3QR09_HUMAN;tr\|J3KTE4\|J3KTE4_HUMAN;sp\|P84098\|RL19_HUMAN | up | 4.1856 | 0.03536 | Translation, ribosomal structure and biogenesis ; | ko03010,Ribosome | |
| 953 | tr\|B2RCT6\|B2RCT6_HUMAN | cDNA, FLJ96276, highly similar to Homo sapiens G1 to S phase transition 1 (GSPT1), mRNA OS=Homo sapiens PE=2 SV=1 | tr\|Q7KZX8\|Q7KZX8_HUMAN;sp\|P15170\|ERF3A_HUMAN | up | 1.6059 | 0.00497 | Translation, ribosomal structure and biogenesis ; | ko03015,mRNA surveillance pathway | |
| 961 | sp\|P35268\|RL22_HUMAN | 60S ribosomal protein L22 OS=Homo sapiens GN=RPL22 PE=1 SV=2 | / | up | 2.2996 | 0.00896 | / | ko03010,Ribosome | |
| 968 | sp\|P62750\|RL23A_HUMAN | 60S ribosomal protein L23a OS=Homo sapiens GN=RPL23A PE=1 SV=1 | / | up | 2.1909 | 0.03744 | Translation, ribosomal structure and biogenesis ; | ko03010,Ribosome | |
| 985 | sp\|O95881\|TXD12_HUMAN | Thioredoxin domain-containing protein 12 OS=Homo sapiens GN=TXNDC12 PE=1 SV=1 | / | up | 1.6549 | 0.04552 | / | ko00480,Glutathione metabolism | |
| 999 | sp\|P37108\|SRP14_HUMAN | Signal recognition particle 14 kDa protein OS=Homo sapiens GN=SRP14 PE=1 SV=2 | / | up | 2.4511 | 0.01920 | / | ko03060,Protein export | |
| 1030 | tr\|V9HWK0\|V9HWK0_HUMAN | Signal recognition particle subunit SRP72 OS=Homo sapiens GN=HEL103 PE=2 SV=1 | sp\|O76094\|SRP72_HUMAN | up | 2.3102 | 0.01555 | / | ko03060,Protein export | |
| 1034 | sp\|Q9Y3U8\|RL36_HUMAN | 60S ribosomal protein L36 OS=Homo sapiens GN=RPL36 PE=1 SV=3 | / | up | 1.7998 | 0.03618 | Translation, ribosomal structure and biogenesis ; | ko03010,Ribosome | |
| 1095 | sp\|P05204\|HMGN2_HUMAN | Non-histone chromosomal protein HMG-17 OS=Homo sapiens GN=HMGN2 PE=1 SV=3 | / | up | 2.1704 | 0.00563 | / | / | |
| 1099 | tr\|C9J0K6\|C9J0K6_HUMAN | Sorcin OS=Homo sapiens GN=SRI PE=1 SV=1 | sp\|P30626\|SORCN_HUMAN | up | 2.0654 | 0.03553 | / | / | |
| 1107 | tr\|B4E0H8\|B4E0H8_HUMAN | cDNA FLJ60385, highly similar to Integrin alpha-3 OS=Homo sapiens PE=2 SV=1 | / | down | 0.4739 | 0.01213 | / | ko05410,Hypertrophic cardiomyopathy (HCM);ko04810,Regulation of actin cytoskeleton;ko05414,Dilated cardiomyopathy;ko05200,Pathways in cancer;ko04510,Focal adhesion;ko05222,Small cell lung cancer;ko04512,ECM-receptor interaction;ko05412,Arrhythmogenic right ventricular cardiomyopathy (ARVC);ko04640,Hematopoietic cell lineage | |
| 1110 | tr\|Q76LA1\|Q76LA1_HUMAN | CSTB protein OS=Homo sapiens GN=CSTB PE=2 SV=1 | sp\|P04080\|CYTB_HUMAN | up | 3.9595 | 0.03562 | / | / | |
| 1175 | sp\|Q9UBS4\|DJB11_HUMAN | DnaJ homolog subfamily B member 11 OS=Homo sapiens GN=DNAJB11 PE=1 SV=1 | / | up | 2.6161 | 0.02781 | Posttranslational modification, protein turnover, chaperones ; | ko04141,Protein processing in endoplasmic reticulum | |
| 1210 | sp\|Q9NVM6\|DJC17_HUMAN | DnaJ homolog subfamily C member 17 OS=Homo sapiens GN=DNAJC17 PE=1 SV=1 | / | up | 1.5187 | 0.04670 | Posttranslational modification, protein turnover, chaperones ; | / | |
| 1241 | sp\|P62316\|SMD2_HUMAN | Small nuclear ribonucleoprotein Sm D2 OS=Homo sapiens GN=SNRPD2 PE=1 SV=1 | / | up | 2.0499 | 0.03472 | Transcription ; | ko03040,Spliceosome | |
| 1292 | sp\|Q14019\|COTL1_HUMAN | Coactosin-like protein OS=Homo sapiens GN=COTL1 PE=1 SV=3 | / | up | 4.0391 | 0.02381 | / | / | |
| 1293 | tr\|Q6FGV9\|Q6FGV9_HUMAN | PMVK protein (Fragment) OS=Homo sapiens GN=PMVK PE=2 SV=1 | sp\|Q15126\|PMVK_HUMAN | up | 1.6628 | 0.03245 | / | ko00900,Terpenoid backbone biosynthesis;ko04146,Peroxisome;ko01100,Metabolic pathways | |
| 1295 | tr\|D3DV26\|D3DV26_HUMAN | S100 calcium binding protein A10 (Annexin II ligand, calpactin I, light polypeptide (P11)), isoform CRA_b (Fragment) OS=Homo sapiens GN=S100A10 PE=4 SV=1 | / | up | 2.1080 | 0.02360 | / | / | |
| 1391 | tr\|V9HW09\|V9HW09_HUMAN | Epididymis secretory sperm binding protein Li 91n OS=Homo sapiens GN=HEL-S-91n PE=2 SV=1 | sp\|P48556\|PSMD8_HUMAN;tr\|R4GMR5\|R4GMR5_HUMAN | up | 1.7864 | 0.03476 | / | ko03050,Proteasome | |
| 1427 | tr\|B2R8N1\|B2R8N1_HUMAN | cDNA, FLJ93976, highly similar to Homo sapiens COP9 homolog (COP9), mRNA OS=Homo sapiens PE=2 SV=1 | tr\|A0A024R4D1\|A0A024R4D1_HUMAN;sp\|Q99627\|CSN8_HUMAN;tr\|E9PGT6\|E9PGT6_HUMAN | down | 0.5698 | 0.03640 | / | / | |
| 1446 | sp\|P46977\|STT3A_HUMAN | Dolichyl-diphosphooligosaccharide--protein glycosyltransferase subunit STT3A OS=Homo sapiens GN=STT3A PE=1 SV=2 | / | up | 1.6464 | 0.04313 | General function prediction only ; | ko00510,N-Glycan biosynthesis;ko01100,Metabolic pathways;ko04141,Protein processing in endoplasmic reticulum | |
| 1591 | sp\|Q9UNE7\|CHIP_HUMAN | E3 ubiquitin-protein ligase CHIP OS=Homo sapiens GN=STUB1 PE=1 SV=2 | / | down | 0.5105 | 0.04200 | Posttranslational modification, protein turnover, chaperones ;General function prediction only ; | ko04120,Ubiquitin mediated proteolysis;ko04141,Protein processing in endoplasmic reticulum | |
| 1633 | sp\|P43034\|LIS1_HUMAN | Platelet-activating factor acetylhydrolase IB subunit alpha OS=Homo sapiens GN=PAFAH1B1 PE=1 SV=2 | / | up | 2.4507 | 0.03789 | General function prediction only ; | ko00565,Ether lipid metabolism;ko01100,Metabolic pathways | |
| 1693 | sp\|P06132\|DCUP_HUMAN | Uroporphyrinogen decarboxylase OS=Homo sapiens GN=UROD PE=1 SV=2 | / | down | 0.4447 | 0.04529 | Coenzyme transport and metabolism ; | ko01110,Biosynthesis of secondary metabolites;ko00860,Porphyrin and chlorophyll metabolism;ko01100,Metabolic pathways | |
| 1721 | tr\|A8K885\|A8K885_HUMAN | cDNA FLJ77179, highly similar to Homo sapiens sorting nexin 6 (SNX6) mRNA OS=Homo sapiens PE=2 SV=1 | tr\|A0A0A0MRI2\|A0A0A0MRI2_HUMAN;sp\|Q9UNH7\|SNX6_HUMAN | up | 6.4176 | 0.02290 | Intracellular trafficking, secretion, and vesicular transport ; General function prediction only ; | / | |
| 1781 | tr\|A0A0A0MTC1\|A0A0A0MTC1_HUMAN | E3 ubiquitin-protein ligase RNF213 OS=Homo sapiens GN=RNF213 PE=1 SV=1 | tr\|A0A0A0MTR7\|A0A0A0MTR7_HUMAN;sp\|Q63HN8\|RN213_HUMAN | up | 1.6559 | 0.00438 | / | / | |
| 1873 | tr\|Q6FIE9\|Q6FIE9_HUMAN | TOLLIP protein OS=Homo sapiens GN=TOLLIP PE=1 SV=1 | sp\|Q9H0E2\|TOLIP_HUMAN | down | 0.5680 | 0.01729 | / | ko04620,Toll-like receptor signaling pathway | |
| 1944 | tr\|A0A024RCA7\|A0A024RCA7_HUMAN | Ribosomal protein, large, P2, isoform CRA_a OS=Homo sapiens GN=RPLP2 PE=3 SV=1 | sp\|P05387\|RLA2_HUMAN | up | 1.9693 | 0.00284 | Translation, ribosomal structure and biogenesis ; | ko03010,Ribosome | |
| 2027 | tr\|Q53HA5\|Q53HA5_HUMAN | CDP-diacylglycerol--inositol 3-phosphatidyltransferase isoform 1 variant (Fragment) OS=Homo sapiens PE=2 SV=1 | tr\|A8K3L7\|A8K3L7_HUMAN;sp\|O14735\|CDIPT_HUMAN;tr\|B3KY94\|B3KY94_HUMAN;tr\|B3KSW0\|B3KSW0_HUMAN | up | 5.6781 | 0.03239 | Lipid transport and metabolism ; | ko00562,Inositol phosphate metabolism;ko00564,Glycerophospholipid metabolism;ko01100,Metabolic pathways;ko04070,Phosphatidylinositol signaling system | |
| 2154 | sp\|Q8NF37\|PCAT1_HUMAN | Lysophosphatidylcholine acyltransferase 1 OS=Homo sapiens GN=LPCAT1 PE=1 SV=2 | / | up | 2.7941 | 0.04809 | Lipid transport and metabolism ; | ko00565,Ether lipid metabolism;ko00564,Glycerophospholipid metabolism;ko01100,Metabolic pathways | |
| 2173 | tr\|A0A024RD78\|A0A024RD78_HUMAN | Mitochondrial ribosomal protein L14, isoform CRA_b OS=Homo sapiens GN=MRPL14 PE=3 SV=1 | sp\|Q6P1L8\|RM14_HUMAN | up | 2.0745 | 0.04000 | / | / | |
| 2459 | tr\|Q8TCM3\|Q8TCM3_HUMAN | Putative uncharacterized protein DKFZp547M048 (Fragment) OS=Homo sapiens GN=DKFZp547M048 PE=2 SV=1 | / | down | 0.4903 | 0.04143 | / | / | |
| 2542 | tr\|A0A024R1I3\|A0A024R1I3_HUMAN | Pyridoxal (Pyridoxine, vitamin B6) phosphatase, isoform CRA_a OS=Homo sapiens GN=PDXP PE=2 SV=1 | sp\|Q96GD0\|PLPP_HUMAN | up | 1.5749 | 0.03596 | Carbohydrate transport and metabolism ; | ko00750,Vitamin B6 metabolism;ko01100,Metabolic pathways | |
| 2553 | sp\|O75648\|MTU1_HUMAN | Mitochondrial tRNA-specific 2-thiouridylase 1 OS=Homo sapiens GN=TRMU PE=1 SV=2 | / | up | 2.7016 | 0.03712 | Translation, ribosomal structure and biogenesis ; | ko04122,Sulfur relay system | |
| 2586 | sp\|Q9BTU6\|P4K2A_HUMAN | Phosphatidylinositol 4-kinase type 2-alpha OS=Homo sapiens GN=PI4K2A PE=1 SV=1 | / | up | 2.0406 | 0.00467 | / | ko00562,Inositol phosphate metabolism;ko01100,Metabolic pathways;ko04070,Phosphatidylinositol signaling system | |
| 2744 | tr\|A8KAH1\|A8KAH1_HUMAN | cDNA FLJ75839, highly similar to Homo sapiens phosphatidylserine synthase 1 (PTDSS1), mRNA OS=Homo sapiens PE=2 SV=1 | sp\|P48651\|PTSS1_HUMAN | up | 2.3929 | 0.04440 | / | ko00564,Glycerophospholipid metabolism;ko01100,Metabolic pathways | |
