# Supplementary material for: EspF of Enterohemorrhagic Escherichia coli Enhances Apoptosis via Endoplasmic Reticulum Stress in Intestinal Epithelial Cells: An Isobaric Tags for Relative and Absolute Quantitation-Based Comparative Proteomic Analysis
Source: Front Microbiol. 2022 Jun 30;13:900919. doi: 10.3389/fmicb.2022.900919 (PMC9279134; doi:10.3389/fmicb.2022.900919)
Supplement: Supplementary file 3 [file Table_3.DOCX]

**Table S3. The GO function analysis of altered proteins within host cells treated by** **Δ*espF* or WT infection.**

| **Differentially expressed proteins** | **Gene Ontology function** |
| --- | --- |
| sp|A0MZ66|SHOT1_ HUMAN | GO:0007411 GO:0050839 GO:0044295 GO:0030027 GO:0038007 GO:0061163 GO:0060327 GO:0032488 GO:0030175 GO:0019894 GO:0005829 GO:0005874 GO:0051899 GO:0045773 GO:0006930 GO:0051015 GO:0005875 GO:0061573 GO:0048471 GO:2001224 GO:0043204 GO:2000114 |
| sp|A2RTX5|SYTC2_ HUMAN | GO:0006435 GO:0004829 GO:0005524 GO:0005515 GO:0005737 |
| sp|A5YKK6|CNOT1_ HUMAN | GO:0033147 GO:0032947 GO:0042974 GO:0001829 GO:2000036 GO:0005829 GO:0004535 GO:0005654 GO:0005615 GO:0005778 GO:0044822 GO:0070016 GO:0090503 GO:0035195 GO:0000122 GO:0000932 GO:0060213 GO:0048387 GO:0030015 GO:0010606 GO:0030331 |
| sp|A6NHR9|SMHD1_ HUMAN | GO:0051276 GO:0000784 GO:0060821 GO:0001740 GO:0005524 |
| sp|A8MXV4|NUD19_ HUMAN | GO:0047429 GO:0005102 GO:0005739 |
| sp|C9JI98|TM238_ HUMAN | GO:0016021 |
| sp|O00116|ADAS_ HUMAN | GO:0008611 GO:0055114 GO:0005739 GO:0005730 GO:0005778 GO:0016614 GO:0005515 GO:0008609 GO:0071949 GO:0005782 |
| sp|O00154|BACH_ HUMAN | GO:0043005 GO:0005654 GO:0015937 GO:0009062 GO:0044297 GO:0052689 GO:0005829 GO:0019432 GO:0035338 GO:1900535 GO:0070062 GO:0042803 GO:0036114 GO:0051792 GO:0036042 GO:0016290 GO:0036116 GO:0005739 |
| sp|O00159|MYO1C_ HUMAN | GO:0045121 GO:0016023 GO:0005654 GO:0051028 GO:0005102 GO:0045815 GO:0008022 GO:0060171 GO:0031941 GO:0030898 GO:0045160 GO:0009925 GO:0005902 GO:0005543 GO:0005730 GO:0005829 GO:0003779 GO:0005643 GO:0001726 GO:0016328 GO:0017160 GO:0003774 GO:0005903 GO:0038096 GO:0001725 GO:2000810 GO:0090314 GO:0070062 GO:1900748 GO:0005516 GO:0045087 GO:0005524 GO:0038089 GO:0005739 |
| sp|O00178|GTPB1_ HUMAN | GO:0005829 GO:0000177 GO:0003746 GO:0006414 GO:0061014 GO:0006955 GO:0044822 GO:0003924 GO:0007165 GO:0016020 GO:0005525 |
| sp|O00186|STXB3_ HUMAN | GO:0051291 GO:0042581 GO:0032868 GO:0070527 GO:0016323 GO:0006904 GO:0070820 GO:0005829 GO:0061024 GO:0022615 GO:0031091 GO:0007420 GO:0015758 GO:0030073 GO:0043312 GO:0045955 GO:0017075 GO:0070062 GO:0016324 |
| sp|O00204|ST2B1_ HUMAN | GO:0018722 GO:0018721 GO:0005829 GO:0080131 GO:0018727 GO:0018726 GO:0000103 GO:0050427 GO:0006805 GO:0018723 GO:0050294 GO:0018724 GO:0070062 GO:0051922 GO:0050698 GO:0004394 GO:0005515 GO:0001537 GO:0018725 GO:0001517 GO:0017095 GO:0008202 GO:0050694 GO:0016232 GO:0005783 GO:0005634 GO:0034930 |
| sp|O00231|PSD11_ HUMAN | GO:0048011 GO:0051436 GO:0002479 GO:0090263 GO:0007411 GO:0048010 GO:0000186 GO:0006977 GO:0005838 GO:0005829 GO:0002223 GO:0051437 GO:0005654 GO:0006595 GO:0043248 GO:0031145 GO:0048863 GO:0038061 GO:0007265 GO:0090090 GO:0033209 GO:0070062 GO:0016032 GO:0006521 GO:0043488 GO:0005515 GO:0000084 GO:0007173 GO:0038095 GO:0050852 GO:0000090 GO:0043066 GO:0008543 GO:0000209 GO:0016020 GO:0008286 |
| sp|O00425|IF2B3_ HUMAN | GO:0042035 GO:0003730 GO:0005829 GO:0017148 GO:0045182 GO:0048027 GO:0005515 GO:0000166 GO:0009653 GO:0005634 GO:0051028 |
| sp|O00429|DNM1L_ HUMAN | GO:0005525 GO:0030672 GO:0042803 GO:0090149 GO:0005777 GO:0008289 GO:1900063 GO:0051289 GO:0016559 GO:0031625 GO:0005903 GO:2001244 GO:0043234 GO:0005829 GO:0032459 GO:0070584 GO:1903146 GO:0090200 GO:0030054 GO:0070585 GO:1903578 GO:0006921 GO:0050714 GO:0048471 GO:0090141 GO:0005794 GO:0006897 GO:0061025 GO:0005874 GO:0005741 GO:0070266 GO:0003924 GO:0003374 GO:0060047 GO:0005905 GO:0043653 |
| sp|O00461|GOLI4_ HUMAN | GO:0032580 GO:0005654 GO:0030139 GO:0006810 GO:0005796 GO:0016021 GO:0005801 GO:0010008 |
| sp|O00566|MPP10_ HUMAN | GO:0000375 GO:0005732 GO:0044822 GO:0042254 GO:0005515 GO:0032040 GO:0034457 GO:0010923 |
| sp|O14524|NEMP1_ HUMAN | GO:0016021 GO:0005515 GO:0005637 |
| sp|O14561|ACPM_ HUMAN | GO:0006810 GO:0009249 GO:0000036 GO:0005759 GO:0008137 GO:0006120 GO:0032981 GO:0005509 GO:0006633 GO:0005747 GO:0046487 GO:0005504 |
| sp|O14617|AP3D1_ HUMAN | GO:0048490 GO:0030117 GO:0005654 GO:0005802 GO:0006829 GO:0000139 GO:0005515 GO:0006726 GO:0008565 GO:0061088 GO:0032438 GO:0072657 GO:0005765 GO:0035646 GO:0006886 GO:0048007 GO:0043195 GO:0051138 GO:0033365 GO:0048499 GO:0010008 |
| sp|O14639|ABLM1_ HUMAN | GO:0007411 GO:0007010 GO:0030032 GO:0030027 GO:0008270 GO:0005737 GO:0003779 GO:0007601 GO:0042384 GO:0009887 GO:0001725 GO:0045944 GO:0097481 |
| sp|O14640|DVL1_ HUMAN | GO:0060134 GO:0048675 GO:0005109 GO:0090263 GO:0030426 GO:0007411 GO:0014069 GO:0007507 GO:0007416 GO:0032091 GO:0071340 GO:1990909 GO:0005829 GO:1902474 GO:0008013 GO:0090179 GO:0090103 GO:0048668 GO:0030424 GO:0001934 GO:0022007 GO:0031122 GO:0006469 GO:0090090 GO:0035176 GO:2000463 GO:0042802 GO:0030136 GO:0019901 GO:0045893 GO:0006366 GO:0050821 GO:0005874 GO:0010976 GO:0035556 GO:0032436 GO:1903827 GO:0034504 GO:0043025 GO:0060997 GO:0035372 GO:0007269 GO:0043197 GO:0016328 GO:0048365 |
| sp|O14646|CHD1_ HUMAN | GO:0043923 GO:0032508 GO:0006357 GO:0006333 GO:0005737 GO:0003677 GO:0006338 GO:0004003 GO:0003682 GO:0005634 GO:0005524 GO:0035064 |
| sp|O14657|TOR1B_ HUMAN | GO:0019894 GO:0051082 GO:0045104 GO:0031175 GO:0007029 GO:0005788 GO:0051085 GO:0044319 GO:0051260 GO:0051584 GO:0006986 GO:0071712 GO:1900244 GO:0070062 GO:0005524 GO:0051787 GO:0016887 GO:0072321 GO:0007155 GO:0031965 GO:0071763 GO:0000338 GO:0042406 GO:2000008 GO:0008021 |
| sp|O14732|IMPA2_ HUMAN | GO:0046855 GO:0005829 GO:0052833 GO:0006021 GO:0052832 GO:0008934 GO:0000287 GO:0046854 GO:0007165 GO:0042803 |
| sp|O14737|PDCD5_ HUMAN | GO:0010628 GO:0090200 GO:0010698 GO:1903638 GO:0005737 GO:0008285 GO:0071560 GO:0008201 GO:0003677 GO:1903645 GO:0070062 GO:0048487 GO:0043280 GO:0005634 |
| sp|O14776|TCRG1_ HUMAN | GO:0016363 GO:0001106 GO:0070064 GO:0005730 GO:0000122 GO:0044822 GO:0015629 GO:0001103 GO:0003713 GO:0003700 |
| sp|O14907|TX1B3_ HUMAN | GO:0005829 GO:2000009 GO:0008013 GO:0008022 GO:0030178 GO:0090630 GO:0070062 GO:0008285 GO:0015629 GO:0060028 GO:0007266 GO:0005634 GO:0005886 |
| sp|O14933|UB2L6_ HUMAN | GO:0005829 GO:0032020 GO:0019941 GO:0045087 GO:0019221 GO:0004842 GO:0005524 GO:0016567 GO:0032480 GO:0005515 GO:0019985 GO:0042296 GO:0005654 |
| sp|O14949|QCR8_ HUMAN | GO:0021860 GO:0016021 GO:0008121 GO:0021539 GO:1902600 GO:0005743 GO:0021854 GO:0021548 GO:0030901 GO:0021794 GO:0022904 GO:0070469 GO:0021766 GO:0021680 GO:0044281 |
| sp|O14980|XPO1_ HUMAN | GO:0000278 GO:0042176 GO:0000122 GO:0051028 GO:0015030 GO:0030512 GO:0000236 GO:0046825 GO:0075733 GO:0008565 GO:0031965 GO:0034504 GO:0000056 GO:0008536 GO:0043488 GO:0000055 GO:0005730 GO:0005829 GO:0005487 GO:0000776 GO:0019904 GO:0007264 GO:0042493 GO:0003723 GO:0000090 GO:0005642 GO:0030529 GO:0010824 |
| sp|O15020|SPTN2_ HUMAN | GO:0048011 GO:0016081 GO:0016324 GO:0016363 GO:0043687 GO:0007411 GO:0008091 GO:0048010 GO:0030534 GO:0007416 GO:0000186 GO:0005543 GO:0001917 GO:0005829 GO:0005200 GO:0051693 GO:0005615 GO:0035264 GO:0030054 GO:0017158 GO:0000139 GO:0006888 GO:0019886 GO:0045087 GO:0007265 GO:0071361 GO:0048471 GO:0007173 GO:0038095 GO:0021692 GO:0018279 GO:0061024 GO:0008543 GO:0043025 GO:0005768 GO:0008286 GO:0003779 GO:0008021 |
| sp|O15031|PLXB2_ HUMAN | GO:0043087 GO:1902287 GO:0017154 GO:0005887 GO:0008360 GO:0005515 GO:0007405 GO:0007162 GO:0002040 GO:0050772 GO:0009986 GO:0007420 GO:0001932 GO:0001843 GO:0070062 GO:0002116 GO:2001222 |
| sp|O15160|RPAC1_ HUMAN | GO:0005829 GO:0006362 GO:0045815 GO:0005736 GO:0006386 GO:0045087 GO:0003677 GO:0006363 GO:0046983 GO:0006361 GO:0032481 GO:0001054 GO:0005666 GO:0001056 GO:0045814 GO:0006385 |
| sp|O15173|PGRC2_ HUMAN | GO:0012505 GO:0005515 GO:0043401 GO:0020037 GO:0003707 GO:0016021 GO:0005496 |
| sp|O15231|ZN185_ HUMAN | GO:0005737 GO:0015629 GO:0008270 GO:0005886 GO:0005925 |
| sp|O15294|OGT1_ HUMAN | GO:0006915 GO:0000791 GO:0043025 GO:0001934 GO:0061087 GO:0043085 GO:0070208 GO:0070207 GO:0045793 GO:0043982 GO:1900182 GO:0008134 GO:1903428 GO:0080182 GO:0043981 GO:0043984 GO:0097363 GO:0006493 GO:0031397 GO:0071300 GO:0007584 GO:0048029 GO:0060548 GO:0000123 GO:0005815 GO:0045944 GO:0030900 GO:0051571 GO:0010801 GO:0006041 GO:0005886 GO:0008047 GO:0033137 GO:0042277 GO:0043996 GO:0005739 GO:0030854 GO:0043005 GO:0090526 GO:0046626 GO:0071333 GO:0032922 GO:0005829 GO:0006110 GO:0097237 GO:0019904 GO:0071222 GO:0048015 GO:0090315 GO:0016262 GO:0045862 GO:1900038 GO:0046972 GO:0035020 GO:0005547 GO:0043995 GO:0042588 GO:0048312 GO:0070688 |
| sp|O15320|CTGE5_ HUMAN | GO:0016021 GO:0008047 GO:0018279 GO:0061024 GO:0043687 GO:0005515 GO:0006888 GO:0005789 GO:0043085 |
| sp|O15357|SHIP2_ HUMAN | GO:0005856 GO:0006006 GO:0007015 GO:0017124 GO:0010642 GO:0007420 GO:0046856 GO:0010977 GO:0006661 GO:0097178 GO:0008156 GO:0030027 GO:0005829 GO:0042169 GO:0001958 GO:0009791 GO:0030175 GO:0005547 GO:0005886 GO:0010629 GO:0005794 GO:0043407 GO:0006897 GO:0002376 GO:0004445 GO:0008285 GO:0046627 GO:0007155 GO:0042493 GO:0032957 GO:0043569 GO:0003779 |
| sp|O15371|EIF3D_ HUMAN | GO:0003743 GO:0001731 GO:0005829 GO:0016282 GO:0071541 GO:0001732 GO:0033290 GO:0044822 GO:0005515 GO:0006446 GO:0016020 |
| sp|O15382|BCAT2_ HUMAN | GO:0052655 GO:0006551 GO:0052656 GO:0006550 GO:0034641 GO:0006573 GO:0010817 GO:0052654 GO:0009082 GO:0005759 |
| sp|O15400|STX7_ HUMAN | GO:0001916 GO:0055037 GO:0005484 GO:1903076 GO:0006906 GO:0005765 GO:0042582 GO:0001772 GO:0070820 GO:0006886 GO:0031902 GO:0019869 GO:0030139 GO:1902685 GO:0016021 GO:0008333 GO:0048278 GO:0043195 GO:0048471 GO:0032403 GO:0070062 GO:0016079 GO:0031901 GO:0031201 GO:0070925 GO:0019905 |
| sp|O15511|ARPC5_ HUMAN | GO:0005925 GO:0007411 GO:0030027 GO:0048013 GO:0034314 GO:0005829 GO:0007264 GO:0005885 GO:0038096 GO:0051015 GO:0070062 GO:0045087 GO:0016477 GO:0005200 |
| sp|O15541|R113A_ HUMAN | GO:0008270 GO:0005515 |
| sp|O43143|DHX15_ HUMAN | GO:0009636 GO:0071008 GO:0004004 GO:0005730 GO:0043279 GO:0008380 GO:0003725 GO:0005524 GO:0044822 GO:0005515 GO:0006397 GO:0005654 GO:0005689 |
| sp|O43159|RRP8_ HUMAN | GO:0016436 GO:0005654 GO:0000183 GO:0044822 GO:0072332 GO:0005677 GO:0016278 GO:0051995 GO:0016300 GO:0016433 GO:0016423 GO:0005737 GO:0005730 GO:0016427 GO:0016434 GO:0042149 GO:0033553 GO:0046015 GO:0032259 GO:0008169 GO:0003880 GO:0071158 GO:0016429 GO:0005886 GO:0006364 GO:0035064 GO:0016568 GO:0016274 |
| sp|O43175|SERA_ HUMAN | GO:0005829 GO:0051287 GO:0070314 GO:0055114 GO:0021510 GO:0006544 GO:0019530 GO:0021915 GO:0010468 GO:0007420 GO:0006541 GO:0031175 GO:0070062 GO:0043209 GO:0004617 GO:0009055 GO:0034641 GO:0006566 GO:0021782 GO:0009448 GO:0006564 |
| sp|O43290|SNUT1_ HUMAN | GO:0015030 GO:0005515 GO:0046540 GO:0044822 GO:0000481 GO:0005794 GO:0005829 GO:0097193 GO:0007050 GO:0071013 GO:0045292 GO:0045585 GO:0000387 |
| sp|O43390|HNRPR_ HUMAN | GO:0005654 GO:0043086 GO:0005515 GO:0000166 GO:0061157 GO:0030426 GO:0003730 GO:0061014 GO:0043679 GO:0005730 GO:0071013 GO:0030425 GO:0019013 GO:0005783 GO:0000398 GO:0007623 |
| sp|O43490|PROM1_ HUMAN | GO:0060219 GO:0005887 GO:0010842 GO:0005793 GO:0045296 GO:0045494 GO:0005615 GO:0031528 GO:0042805 GO:0009986 GO:0072139 GO:0005903 GO:0042622 GO:0072112 GO:0032420 GO:0070062 GO:0005783 GO:0071914 GO:2000768 GO:0016324 |
| sp|O43520|AT8B1_ HUMAN | GO:0045332 GO:0005887 GO:0007605 GO:0005515 GO:0021650 GO:0031526 GO:0060119 GO:0006855 GO:1901612 GO:0015721 GO:0005794 GO:0008206 GO:0007030 GO:0045892 GO:0032534 GO:0000287 GO:0034220 GO:0004012 GO:0032420 GO:0005783 GO:0005524 GO:0016324 |
| sp|O43570|CAH12_ HUMAN | GO:0015670 GO:0055064 GO:0005886 GO:0006730 GO:0004089 GO:0006885 GO:0016021 GO:0008270 GO:0015701 |
| sp|O43592|XPOT_ HUMAN | GO:0005829 GO:0016363 GO:0008536 GO:0015932 GO:0008033 GO:0005643 GO:0000049 GO:0006886 GO:0005654 GO:0071528 |
| sp|O43660|PLRG1_ HUMAN | GO:0016021 GO:0031965 GO:0071011 GO:0005730 GO:0000398 GO:0080008 GO:0016607 GO:0034504 GO:1900087 GO:0005515 GO:0000974 GO:0071013 GO:0005662 |
| sp|O43678|NDUA2_ HUMAN | GO:0008137 GO:0005747 GO:0006120 GO:0032981 |
| sp|O43688|PLPP2_ HUMAN | GO:0006644 GO:0006470 GO:0008195 GO:0046839 GO:0042577 GO:0005887 GO:0030148 GO:0005515 GO:0007165 GO:0044281 GO:0004721 |
| sp|O43709|WBS22_ HUMAN | GO:0009019 GO:0008174 GO:0051994 GO:0034541 GO:0018423 GO:0030792 GO:0008650 GO:0016205 GO:0008988 GO:0044822 GO:0034931 GO:0016428 GO:0043770 GO:0052735 GO:0008172 GO:0005737 GO:0005730 GO:0043791 GO:0006355 GO:0009008 GO:0071424 GO:0018707 GO:0043851 GO:0043782 GO:0043777 GO:0052666 GO:0043776 GO:0080012 GO:0052667 GO:0043780 GO:0016435 GO:0043834 GO:0009383 GO:0032259 GO:0008425 GO:0034807 GO:0019702 GO:0034933 GO:0043852 GO:0043833 GO:0052665 GO:0000179 GO:0052624 GO:0043827 GO:0043803 GO:0016279 GO:0006364 GO:0016568 GO:0070677 GO:0004809 |
| sp|O43719|HTSF1_ HUMAN | GO:0019079 GO:0016021 GO:0006357 GO:0005686 GO:0000398 GO:0005684 GO:0044822 GO:0032784 GO:0000166 GO:0005654 |
| sp|O43747|AP1G1_ HUMAN | GO:0030669 GO:0000226 GO:0055037 GO:0030131 GO:0043323 GO:0008022 GO:0030742 GO:0008565 GO:0017137 GO:0015630 GO:0032588 GO:0032438 GO:0019894 GO:0005765 GO:0035646 GO:0006886 GO:0005829 GO:0061024 GO:0019886 GO:0050690 GO:0048471 GO:0016032 GO:0005518 GO:0090160 |
| sp|O43752|STX6_ HUMAN | GO:0042147 GO:0016021 GO:0006906 GO:1903827 GO:0030136 GO:0090161 GO:0048278 GO:0005484 GO:0043195 GO:0005769 GO:0055037 GO:0048193 GO:0048471 GO:0019905 GO:0045335 GO:0006886 GO:0007032 GO:0032588 GO:0031201 GO:0005886 |
| sp|O43765|SGTA_ HUMAN | GO:0005829 GO:1903071 GO:1904288 GO:0046982 GO:0016032 GO:0042803 GO:0016020 |
| sp|O43768|ENSA_ HUMAN | GO:0019870 GO:0051721 GO:0005654 GO:0043086 GO:0009749 GO:0007067 GO:0005102 GO:0051301 GO:0050796 GO:0005737 GO:0004864 GO:0008601 GO:0000088 GO:0000086 GO:0007584 GO:0034047 |
| sp|O43776|SYNC_ HUMAN | GO:0005829 GO:0004816 GO:0006421 GO:0003676 GO:0005739 GO:0070062 GO:0005524 |
| sp|O43815|STRN_ HUMAN | GO:0016358 GO:0051721 GO:0016055 GO:0043197 GO:0007626 GO:0000159 GO:0048306 GO:0070830 GO:0005737 GO:0070016 GO:0008285 GO:0045211 GO:0007169 GO:0043025 GO:0018108 GO:0016021 GO:0014069 GO:0030331 GO:0004714 GO:0005923 GO:0032403 GO:0005516 GO:0005524 GO:0000187 |
| sp|O43818|U3IP2_ HUMAN | GO:0032040 GO:0031428 GO:0006364 GO:0034511 GO:0044822 |
| sp|O60218|AK1BA_ HUMAN | GO:0044597 GO:0007603 GO:0005829 GO:0005764 GO:0055114 GO:0001523 GO:0044598 GO:0006703 GO:0070062 GO:0006775 GO:0016488 GO:0047718 GO:0001758 GO:0007586 GO:0005515 GO:0004303 GO:0006081 GO:0005759 GO:0004033 GO:0045550 |
| sp|O60220|TIM8A_ HUMAN | GO:0072321 GO:0044267 GO:0008270 GO:0005743 GO:0006626 GO:0005758 GO:0007399 GO:0005515 |
| sp|O60488|ACSL4_ HUMAN | GO:0030307 GO:0060996 GO:0044233 GO:0070672 GO:0060136 GO:0001676 GO:0005789 GO:0047676 GO:0005741 GO:0019432 GO:0043025 GO:0004467 GO:0016021 GO:0035338 GO:0032307 GO:0070062 GO:0005811 GO:0019217 GO:0005524 GO:0007584 GO:0005778 GO:0031957 |
| sp|O60493|SNX3_ HUMAN | GO:0030111 GO:0032009 GO:0051224 GO:0010324 GO:0032266 GO:0010955 GO:0042177 GO:0005829 GO:0070676 GO:0030904 GO:0019898 GO:0070273 GO:0042541 GO:0050765 GO:0010314 GO:0033572 GO:0022615 GO:0070062 GO:0030136 GO:0046597 GO:0080025 GO:0019903 GO:0033157 GO:0006783 GO:0010976 GO:0031901 GO:0030218 GO:2000642 GO:0009617 |
| sp|O60502|OGA_ HUMAN | GO:0032024 GO:0010524 GO:0045862 GO:0051054 GO:0046326 GO:0005829 GO:0010616 GO:0006516 GO:0060051 GO:0006612 GO:0031343 GO:0051901 GO:0016231 GO:0007568 GO:0048545 GO:0070265 GO:0006044 GO:0005739 GO:0046060 GO:0043243 GO:0004415 GO:0016573 GO:0004402 GO:0005634 GO:0006517 GO:0016020 GO:0060124 |
| sp|O60506|HNRPQ_ HUMAN | GO:0048027 GO:0005654 GO:0017148 GO:0005515 GO:0001649 GO:0016020 GO:0000166 GO:0090367 GO:0003730 GO:0070934 GO:0045727 GO:1990635 GO:0071204 GO:0071013 GO:0043025 GO:0008143 GO:0071346 GO:0016032 GO:0019013 GO:0005783 GO:0000398 GO:0070937 GO:0097452 GO:0007623 |
| sp|O60508|PRP17_ HUMAN | GO:0000386 GO:0006369 GO:0000350 GO:0031124 GO:0051301 GO:0044822 GO:0005515 GO:0006406 GO:0071013 GO:0005654 |
| sp|O60547|GMDS_ HUMAN | GO:0021564 GO:0007219 GO:0005515 GO:0021744 GO:0010842 GO:0019673 GO:0008446 GO:0070401 GO:0036065 GO:0008417 GO:0006488 GO:0005829 GO:0042351 GO:0070062 GO:0043687 GO:0018279 |
| sp|O60568|PLOD3_ HUMAN | GO:0005515 GO:0033823 GO:0001886 GO:0048730 GO:0005581 GO:0042311 GO:0008475 GO:0005506 GO:0008104 GO:0030199 GO:0060425 GO:0030867 GO:0006464 GO:0050211 GO:0070831 GO:0055114 GO:0070062 GO:0031418 GO:0032870 GO:0001701 GO:0021915 |
| sp|O60664|PLIN3_ HUMAN | GO:0005794 GO:0016192 GO:0010008 GO:0005515 GO:0005811 |
| sp|O60701|UGDH_ HUMAN | GO:0001702 GO:0003979 GO:0005829 GO:0051287 GO:0006024 GO:0055114 GO:0052695 GO:0006805 GO:0070062 GO:0009055 GO:0006011 GO:0005654 GO:0006065 |
| sp|O60832|DKC1_ HUMAN | GO:0070034 GO:0031429 GO:1990481 GO:0000495 GO:0005515 GO:0072589 GO:1904851 GO:0044822 GO:0032212 GO:0090661 GO:0008283 GO:0031118 GO:0005737 GO:0009982 GO:1904874 GO:0051973 GO:0003720 GO:0034513 GO:0016021 GO:0090669 GO:0031120 |
| sp|O60869|EDF1_ HUMAN | GO:0045893 GO:0004402 GO:0007275 GO:0008168 GO:0044822 GO:0045446 GO:0005737 GO:0005730 GO:0019216 GO:0003713 GO:0043565 GO:0005669 GO:0070062 GO:0043388 GO:0005516 GO:0003700 |
| sp|O60888|CUTA_ HUMAN | GO:0016021 GO:0008104 GO:0070062 GO:0010038 GO:0019899 |
| sp|O60925|PFD1_ HUMAN | GO:0005829 GO:0044267 GO:0051086 GO:0007049 GO:0006355 GO:0044183 GO:0021549 GO:0042113 GO:0030036 GO:0021537 GO:0051082 GO:0016272 GO:0003700 |
| sp|O75116|ROCK2_ HUMAN | GO:0016525 GO:0007249 GO:0048813 GO:0001843 GO:0007411 GO:0010595 GO:0048010 GO:0072518 GO:0005198 GO:1903140 GO:0045616 GO:0046872 GO:0005829 GO:1903347 GO:0090002 GO:0030866 GO:0036464 GO:0051492 GO:0051893 GO:0010825 GO:0048013 GO:0001934 GO:2000114 GO:0030261 GO:0017048 GO:0005886 GO:0031616 GO:0044822 GO:0071394 GO:0035509 GO:0000910 GO:0005524 GO:0008625 GO:0006939 GO:0010628 GO:0007266 GO:0005634 GO:0042752 |
| sp|O75190|DNJB6_ HUMAN | GO:0005654 GO:0051087 GO:0060710 GO:0006457 GO:0016020 GO:1900034 GO:0030198 GO:0034504 GO:0060715 GO:0005829 GO:0031072 GO:0030036 GO:0090084 GO:0001671 GO:0045892 GO:0043154 GO:0032880 GO:0003677 GO:0051082 GO:0045109 GO:0060717 GO:0032781 GO:0030018 GO:0048471 |
| sp|O75306|NDUS2_ HUMAN | GO:0006810 GO:0005654 GO:0051287 GO:0005759 GO:0051539 GO:0008137 GO:0006120 GO:0009055 GO:0032981 GO:0046872 GO:0005747 GO:0031625 GO:0048038 GO:0006979 |
| sp|O75312|ZPR1_ HUMAN | GO:0031369 GO:0033120 GO:0030426 GO:0061564 GO:0021510 GO:0097504 GO:0031641 GO:0071364 GO:0030971 GO:2000672 GO:1990261 GO:0030424 GO:1902742 GO:0007165 GO:0045927 GO:0032797 GO:0042307 GO:0043204 GO:0000226 GO:0048471 GO:0042023 GO:0008270 GO:0001833 GO:0015030 GO:0030576 GO:0071931 GO:0006397 GO:0005730 GO:0001834 |
| sp|O75323|NIPS2_ HUMAN | GO:0005739 GO:0006754 GO:0005887 GO:0005515 GO:0006119 GO:2000984 |
| sp|O75330|HMMR_ HUMAN | GO:0005737 GO:0009986 GO:0005975 GO:0005540 GO:0030214 GO:0005886 GO:0005515 GO:0044281 |
| sp|O75367|H2AY_ HUMAN | GO:0000979 GO:0045618 GO:0061187 GO:0035098 GO:0061086 GO:0005721 GO:0001558 GO:1902750 GO:1904815 GO:0031492 GO:0000182 GO:0007549 GO:0000784 GO:0010385 GO:0000793 GO:0034184 GO:0070062 GO:0001739 GO:0000122 GO:0001740 GO:0019901 GO:0046982 GO:0033128 GO:0051572 GO:1901837 GO:0019216 GO:0045815 GO:0006334 GO:0005813 GO:0005730 GO:0071901 GO:0030291 GO:0071169 GO:0000786 |
| sp|O75369|FLNB_ HUMAN | GO:0005829 GO:0016021 GO:0030018 GO:0007016 GO:0005903 GO:0003382 GO:0070062 GO:0003334 GO:0042802 GO:0019221 GO:0030036 GO:0001725 GO:0007519 GO:0005938 GO:0044822 GO:0005925 GO:0003779 GO:0005886 |
| sp|O75391|SPAG7_ HUMAN | GO:0005634 GO:0003676 |
| sp|O75396|SC22B_ HUMAN | GO:0012507 GO:0000139 GO:0005484 GO:0033043 GO:0042470 GO:0015031 GO:0005789 GO:0048280 GO:0045732 GO:0033116 GO:0048208 GO:0016021 GO:0043687 GO:0018279 GO:0019905 |
| sp|O75400|PR40A_ HUMAN | GO:0016477 GO:0016363 GO:0070064 GO:0008360 GO:0032465 GO:0000398 GO:0007010 GO:0005685 GO:0044822 GO:0071004 GO:0005654 GO:0005737 GO:0016020 |
| sp|O75427|LRCH4_ HUMAN | GO:0016021 GO:0007399 GO:0016605 GO:0005515 GO:0005737 GO:0045892 |
| sp|O75436|VP26A_ HUMAN | GO:0005769 GO:1990126 GO:0030906 GO:0005515 GO:0097422 GO:0042147 GO:0008565 GO:0005829 GO:0010008 GO:0070062 GO:0015031 |
| sp|O75439|MPPB_ HUMAN | GO:0008270 GO:0005759 GO:0044267 GO:0016485 GO:0004222 GO:0009060 GO:0005750 GO:0006626 GO:0006122 |
| sp|O75475|PSIP1_ HUMAN | GO:0005654 GO:0033613 GO:0034399 GO:0097100 GO:0000395 GO:0044822 GO:0001105 GO:0075713 GO:0005730 GO:0005829 GO:0005720 GO:0009408 GO:0045944 GO:0003682 GO:0035327 GO:0006979 |
| sp|O75489|NDUS3_ HUMAN | GO:0006810 GO:0005515 GO:0005759 GO:0008137 GO:0006120 GO:0009055 GO:0032981 GO:2001243 GO:0043209 GO:0005747 GO:0030308 GO:0072593 GO:0021762 GO:0005634 |
| sp|O75533|SF3B1_ HUMAN | GO:0016363 GO:0003729 GO:0045815 GO:0071011 GO:0005686 GO:0001825 GO:0009952 GO:0000245 GO:0034693 GO:0016607 GO:0000785 GO:0005515 GO:0071004 GO:0071013 GO:0005689 GO:0003682 |
| sp|O75569|PRKRA_ HUMAN | GO:0009615 GO:0005654 GO:0016301 GO:0016020 GO:0043085 GO:0042473 GO:0019901 GO:0044822 GO:0006955 GO:0008047 GO:0031054 GO:0030422 GO:0005829 GO:0008285 GO:0008284 GO:0006468 GO:0042474 GO:0048705 GO:0048471 GO:0003725 GO:0042803 GO:0034599 GO:2001244 |
| sp|O75643|U520_ HUMAN | GO:0019013 GO:0004004 GO:0042802 GO:0005524 GO:0044822 GO:0000354 GO:0001649 GO:0005682 GO:0071013 GO:0005654 GO:0016020 |
| sp|O75648|MTU1_ HUMAN | GO:0002143 GO:0016783 GO:0005739 GO:0046872 GO:0032259 GO:0005524 GO:0000049 GO:0005654 GO:0008168 GO:0070903 |
| sp|O75688|PPM1B_ HUMAN | GO:0006499 GO:0030145 GO:0005515 GO:0016020 GO:0032688 GO:0035970 GO:0005829 GO:0019221 GO:0050687 GO:0000287 GO:0042347 GO:0004722 |
| sp|O75817|POP7_ HUMAN | GO:0004526 GO:0005655 GO:0090502 GO:0008033 GO:0044822 GO:0005515 GO:0005654 GO:0005737 |
| sp|O75843|AP1G2_ HUMAN | GO:0008565 GO:0006892 GO:0061024 GO:0005515 GO:0005802 GO:0016032 GO:0006886 GO:0010008 GO:0030121 |
| sp|O75844|FACE1_ HUMAN | GO:0016021 GO:0006998 GO:0030327 GO:0071586 GO:0046872 GO:0070062 GO:0004222 GO:0008235 GO:0005789 GO:0005637 |
| sp|O75880|SCO1_ HUMAN | GO:0038162 GO:0048170 GO:0030218 GO:0030016 GO:0038093 GO:0030217 GO:0005020 GO:0002551 GO:0002020 GO:0001541 GO:0046777 GO:0097324 GO:0031274 GO:0009898 GO:0008360 GO:0008535 GO:0030335 GO:0060374 GO:0035234 GO:0008354 GO:0035019 GO:0000187 GO:0042803 GO:0005743 GO:0072492 GO:0030032 GO:0032762 GO:0005507 GO:0008284 GO:0002371 GO:0042511 GO:0006954 GO:0002320 GO:0006687 GO:0009897 GO:0048103 GO:0038109 GO:0035162 GO:0042629 GO:0019955 GO:0035701 GO:0035855 GO:0002318 GO:0050910 GO:0002327 GO:0042517 GO:0007286 GO:0048565 GO:0006878 GO:0001669 GO:0043069 GO:0005615 GO:0048863 GO:0030318 GO:0000166 GO:0006091 GO:0097326 GO:0097067 GO:0043303 GO:0051091 GO:0005911 GO:0042523 GO:0050673 GO:0006825 GO:0031532 GO:0048070 GO:0045747 GO:0008542 |
| sp|O75940|SPF30_ HUMAN | GO:0005737 GO:0015030 GO:0005681 GO:0005515 GO:0000375 GO:0045111 GO:0016607 GO:0006397 GO:0006915 GO:0044822 |
| sp|O75947|ATP5H_ HUMAN | GO:0005654 GO:0042776 GO:0015078 GO:0016887 GO:0070062 GO:0000276 GO:0022904 |
| sp|O75964|ATP5L_ HUMAN | GO:0047624 GO:0042776 GO:0015078 GO:0016887 GO:0070062 GO:0000276 GO:0022904 |
| sp|O75976|CBPD_ HUMAN | GO:0006518 GO:0051721 GO:0004181 GO:0016021 GO:0004185 GO:0008270 GO:0071352 GO:0070062 GO:0032403 GO:0005615 GO:0005802 GO:0048471 GO:0016485 GO:0005634 GO:0005886 |
| sp|O76021|RL1D1_ HUMAN | GO:0005730 GO:0001649 GO:0042981 GO:2000772 GO:0016020 GO:0032880 GO:0044822 |
| sp|O76027|ANXA9_ HUMAN | GO:0005829 GO:0015464 GO:0005509 GO:0005544 GO:0098794 GO:0070062 GO:0009986 GO:0016337 GO:0099565 GO:0001786 GO:0042803 |
| sp|O94763|RMP_ HUMAN | GO:0009615 GO:0071363 GO:0000122 GO:0001558 GO:0051219 GO:2001243 GO:0030425 GO:0005665 GO:0004864 GO:0001106 GO:0010923 GO:0071383 GO:0003682 GO:0005739 |
| sp|O94826|TOM70_ HUMAN | GO:0016021 GO:0000423 GO:0044267 GO:0005742 GO:0071806 GO:0008320 GO:0070062 GO:0006626 GO:0005515 |
| sp|O94832|MYO1D_ HUMAN | GO:0061502 GO:0005790 GO:0030898 GO:0097440 GO:0044853 GO:0048306 GO:0016323 GO:0043209 GO:0000146 GO:0019904 GO:0043025 GO:0031410 GO:0005903 GO:0005768 GO:0051015 GO:0010923 GO:0070062 GO:0005516 GO:0005524 GO:0016459 GO:0030673 |
| sp|O94851|MICA2_ HUMAN | GO:0034526 GO:0018630 GO:0010735 GO:0018653 GO:0018650 GO:0018638 GO:0008270 GO:0018641 GO:0080014 GO:0034785 GO:0043731 GO:0018649 GO:0018637 GO:0019417 GO:0018639 GO:0034560 GO:0018646 GO:0018660 GO:0018631 GO:0018656 GO:0018644 GO:0018652 GO:0018643 GO:0071949 GO:0001947 GO:0034894 GO:0003779 GO:0018640 GO:0018634 GO:0043719 GO:0043914 GO:0018642 GO:0018636 GO:0018632 GO:0034791 GO:0018657 GO:0034819 GO:0018655 GO:0018654 GO:0052872 GO:0034534 GO:0018633 GO:0018471 GO:0019113 GO:0008688 GO:0018651 GO:0005634 GO:0034802 GO:0030042 GO:0018647 |
| sp|O94874|UFL1_ HUMAN | GO:0043005 GO:0005654 GO:0070740 GO:0070738 GO:0043773 GO:0005515 GO:0001649 GO:0033146 GO:0043774 GO:0005789 GO:0008766 GO:0070736 GO:0005829 GO:0032088 GO:0032434 GO:0034976 GO:0032880 GO:0060252 GO:0043234 GO:1902065 GO:0071568 GO:0070737 GO:0031397 GO:0018169 GO:1990592 |
| sp|O94880|PHF14_ HUMAN | GO:0048286 GO:0008270 GO:2000584 GO:0000122 GO:2000791 GO:0005634 |
| sp|O94906|PRP6_ HUMAN | GO:0050681 GO:0045944 GO:0000245 GO:0016607 GO:0000244 GO:0043021 GO:0044822 GO:0005682 GO:0046540 GO:0071013 GO:0003713 GO:0006403 GO:0016020 |
| sp|O94919|ENDD1_ HUMAN | GO:0046872 GO:0004519 GO:0090305 GO:0003676 GO:0016020 GO:0070062 |
| sp|O94925|GLSK_ HUMAN | GO:0014047 GO:0006367 GO:0005515 GO:0002087 GO:0051289 GO:0004359 GO:0005759 GO:0007269 GO:0006537 GO:0005829 GO:0001967 GO:0006543 |
| sp|O95154|ARK73_ HUMAN | GO:0044597 GO:0005829 GO:0004032 GO:0055114 GO:0005739 GO:0044598 GO:0006805 GO:0005975 GO:0070062 GO:0009055 GO:0019119 GO:0005515 GO:0006081 GO:0005794 |
| sp|O95168|NDUB4_ HUMAN | GO:0006979 GO:0016021 GO:0008137 GO:0031965 GO:0005747 GO:0070062 GO:0006120 GO:0032981 GO:0005654 |
| sp|O95169|NDUB8_ HUMAN | GO:0016021 GO:0008137 GO:0005747 GO:0006120 GO:0032981 GO:0005783 |
| sp|O95202|LETM1_ HUMAN | GO:0042407 GO:0005743 GO:0005515 GO:0005509 GO:0016021 |
| sp|O95251|KAT7_ HUMAN | GO:0005515 GO:0008270 GO:0004402 GO:0000123 GO:0005737 GO:0005730 GO:0006355 GO:0043983 GO:0043966 GO:1900182 GO:0043984 GO:0043982 GO:0006260 GO:0003700 GO:0043981 |
| sp|O95373|IPO7_ HUMAN | GO:0005829 GO:0008565 GO:0030695 GO:0008536 GO:0042393 GO:0005643 GO:0045087 GO:0007165 GO:0016032 GO:0050790 GO:0005654 GO:0006610 GO:0016020 |
| sp|O95433|AHSA1_ HUMAN | GO:0001671 GO:0051087 GO:0006950 GO:0032781 GO:0005829 GO:0005783 GO:0070062 |
| sp|O95453|PARN_ HUMAN | GO:0003730 GO:0005829 GO:0009451 GO:0000289 GO:0005730 GO:0046872 GO:0007292 GO:0004535 GO:0043488 GO:0000166 GO:0090503 GO:0019901 GO:0000184 |
| sp|O95470|SGPL1_ HUMAN | GO:0030097 GO:0060021 GO:0005515 GO:0001822 GO:0006672 GO:0008210 GO:0010761 GO:0008209 GO:0007283 GO:0060325 GO:0009791 GO:0008117 GO:0097190 GO:0006631 GO:0030170 GO:0001553 GO:0048008 GO:0048705 GO:0030176 GO:0033327 GO:0016831 GO:0040014 GO:0030149 GO:0030148 GO:0001570 |
| sp|O95671|ASML_ HUMAN | GO:0005737 GO:0032259 GO:0005515 GO:0008171 |
| sp|O95721|SNP29_ HUMAN | GO:0016082 GO:0000139 GO:0005484 GO:0015031 GO:0031629 GO:0097352 GO:0005813 GO:0031410 GO:0020018 GO:0042384 GO:0043195 GO:0000421 GO:0006903 GO:0031201 GO:0019905 |
| sp|O95782|AP2A1_ HUMAN | GO:0007411 GO:0030122 GO:0042059 GO:0008022 GO:0019901 GO:0008565 GO:0030141 GO:0048011 GO:0048013 GO:0016323 GO:0030130 GO:0036020 GO:1900126 GO:0006886 GO:0005829 GO:0019886 GO:0050690 GO:0007268 GO:0032433 GO:0032403 GO:0016032 GO:0006895 GO:0072583 GO:0035615 GO:0016324 |
| sp|O95831|AIFM1_ HUMAN | GO:0019152 GO:0032867 GO:1902065 GO:0009636 GO:0018451 GO:0052677 GO:0016021 GO:0071392 GO:0032442 GO:0033709 GO:0043525 GO:0034840 GO:0044105 GO:0051990 GO:0004495 GO:0032866 GO:0005829 GO:0005758 GO:0000252 GO:0018452 GO:1904045 GO:0035380 GO:0034831 GO:0004174 GO:0070301 GO:0044103 GO:0004448 GO:0030261 GO:0071949 GO:0006919 GO:0016174 GO:0030182 GO:0048471 GO:0071732 GO:0055114 GO:0003677 GO:0008875 GO:0009055 GO:0008637 GO:1902510 GO:0048258 GO:0005741 GO:0070059 GO:0002931 GO:0018453 GO:0045454 GO:0033765 GO:0035410 GO:0046983 GO:0043713 GO:0032981 GO:0005743 GO:0005634 GO:0090650 |
| sp|O95834|EMAL2_ HUMAN | GO:0031115 GO:0008017 GO:0016021 GO:0005875 GO:0072686 GO:0010968 GO:0005874 GO:0008022 GO:0005102 GO:0007601 GO:0007605 GO:0003824 GO:0005737 |
| sp|O95881|TXD12_ HUMAN | GO:0055114 GO:0015037 GO:1902236 GO:0045454 GO:0019153 GO:0005788 GO:0005515 |
| sp|O95989|NUDT3_ HUMAN | GO:0005829 GO:0052845 GO:0007267 GO:0052843 GO:0052848 GO:0070062 GO:0015961 GO:0052847 GO:0000287 GO:0052840 GO:0052844 GO:0052846 GO:0071544 GO:0008486 |
| sp|P00167|CYB5_ HUMAN | GO:0019852 GO:0004768 GO:0004129 GO:0016021 GO:0006631 GO:0055114 GO:1902600 GO:0046872 GO:0070062 GO:0019899 GO:0005741 GO:0020037 GO:0046686 GO:0005789 GO:0004033 |
| sp|P00505|AATM_ HUMAN | GO:0004069 GO:0097052 GO:0006094 GO:0006107 GO:0034641 GO:0006532 GO:0006533 GO:0005759 GO:0045471 GO:0044822 GO:0016597 GO:0005743 GO:0019470 GO:0005543 GO:0043209 GO:0080130 GO:0009986 GO:0016212 GO:0043234 GO:0030170 GO:0070062 GO:0042803 GO:0019899 GO:0005886 GO:0015908 GO:0043204 GO:0019550 GO:0019551 |
| sp|P00995|ISK1_ HUMAN | GO:0090281 GO:0060046 GO:0001669 GO:0005515 GO:0010751 GO:0005615 GO:0004867 GO:0050732 GO:1900004 GO:0070062 GO:2001256 |
| sp|P01133|EGF_ HUMAN | GO:0038095 GO:0008083 GO:0000186 GO:0042059 GO:0090370 GO:0021940 GO:0043235 GO:0035413 GO:0043406 GO:1900127 GO:0045087 GO:0045840 GO:0051048 GO:0042813 GO:0007262 GO:0001525 GO:0060070 GO:0002576 GO:0060749 GO:0005765 GO:0005509 GO:0048010 GO:2000060 GO:0005886 GO:0048011 GO:0070062 GO:0016021 GO:0030168 GO:0010800 GO:0048754 GO:0044332 GO:0006260 GO:0017147 GO:0048015 GO:0005615 GO:0007411 GO:0031093 GO:2000008 GO:0043388 GO:0008543 GO:0090279 GO:0070371 GO:0007171 GO:0007265 GO:0045741 GO:0005154 GO:0030297 GO:0008286 GO:0045893 |
| sp|P02545|LMNA_ HUMAN | GO:0030334 GO:0009888 GO:0007077 GO:0005198 GO:2001234 GO:0005829 GO:0071456 GO:0005654 GO:0000088 GO:0007517 GO:0090343 GO:0006921 GO:0048471 GO:0005515 GO:0036498 GO:0030154 GO:0031965 GO:0044267 GO:0005638 GO:0000090 GO:0051239 GO:0034504 GO:0007084 GO:0030951 |
| sp|P02786|TFR1_ HUMAN | GO:0010042 GO:0055038 GO:0051087 GO:0046718 GO:0033572 GO:0072562 GO:0030316 GO:0005887 GO:1990712 GO:0042127 GO:0030544 GO:0006879 GO:0004998 GO:0045780 GO:0006898 GO:0001558 GO:0044822 GO:0032526 GO:0007568 GO:0016323 GO:0042470 GO:0010039 GO:0001618 GO:0005905 GO:0001948 GO:0006953 GO:0046688 GO:0001666 GO:0009897 GO:0035690 GO:0097286 GO:0055085 GO:0048471 GO:0070062 GO:0003725 GO:0033570 GO:0042803 GO:0007584 GO:0005739 |
| sp|P04040|CATA_ HUMAN | GO:0042744 GO:0006641 GO:0047962 GO:0010193 GO:0005925 GO:0042803 GO:0006805 GO:0004046 GO:0010288 GO:0035898 GO:0016021 GO:0005764 GO:0051289 GO:0042542 GO:0005783 GO:0047961 GO:0032787 GO:0050661 GO:0014068 GO:0005102 GO:0006195 GO:0032088 GO:0046872 GO:0005829 GO:0055074 GO:0020037 GO:0005759 GO:0005758 GO:0005615 GO:0008203 GO:0005778 GO:0045471 GO:0032868 GO:0051781 GO:0005262 GO:0009060 GO:0001666 GO:0009650 GO:0014854 GO:0009642 GO:0080184 GO:0001649 GO:0007568 GO:0006544 GO:0005886 GO:0070509 GO:0070062 GO:0033591 GO:0005794 GO:0014823 GO:0051092 GO:0001657 GO:0032355 GO:0055093 GO:0006637 GO:0033554 GO:0005516 GO:0033197 GO:0033189 GO:0020027 GO:0098869 GO:0071363 GO:0046686 GO:0006144 GO:0004096 GO:0042493 GO:0070588 GO:0043066 GO:0070542 GO:0019899 GO:0005782 |
| sp|P04066|FUCO_ HUMAN | GO:0004560 GO:0018279 GO:0070062 GO:0043202 GO:0043687 GO:0016139 GO:0006004 GO:0006027 GO:0030246 |
| sp|P04843|RPN1_ HUMAN | GO:0006412 GO:0016021 GO:0008250 GO:0018279 GO:0043687 GO:0015833 GO:0042470 GO:0044822 GO:0005515 GO:0005791 GO:0006614 GO:0004579 |
| sp|P04844|RPN2_ HUMAN | GO:0006412 GO:0016021 GO:0008250 GO:0018279 GO:0043022 GO:0043687 GO:0005515 GO:0005791 GO:0007568 GO:0000421 GO:0042493 GO:0006614 GO:0004579 GO:0005634 |
| sp|P04899|GNAI2_ HUMAN | GO:0004871 GO:0005525 GO:0007214 GO:0007213 GO:0007216 GO:0008284 GO:0007584 GO:0051301 GO:0030496 GO:0000186 GO:0046872 GO:0005829 GO:0008016 GO:0005834 GO:0005654 GO:0045121 GO:0031683 GO:0030168 GO:0051924 GO:0050805 GO:0007194 GO:0001973 GO:0070062 GO:0005765 GO:0031821 GO:0007049 GO:0030425 GO:0003924 GO:0043434 GO:0007193 GO:0005813 GO:0044297 |
| sp|P04920|B3A2_ HUMAN | GO:0048565 GO:0016021 GO:0015701 GO:1902476 GO:0007283 GO:0019899 GO:0005925 GO:0016324 GO:0016323 GO:0005452 GO:0051453 GO:0015108 |
| sp|P05023|AT1A1_ HUMAN | GO:0051087 GO:0015991 GO:0030506 GO:0002028 GO:0043548 GO:0016324 GO:0005901 GO:0005783 GO:0030955 GO:0051117 GO:0060081 GO:0016323 GO:0097481 GO:0030315 GO:0071383 GO:0008217 GO:0002026 GO:0030007 GO:0031402 GO:0045989 GO:0031947 GO:0043209 GO:0070062 GO:1903779 GO:0036376 GO:0019904 GO:0045822 GO:0005794 GO:0006883 GO:0086013 GO:0005524 GO:0086064 GO:0042470 GO:0019901 GO:0045823 GO:1990573 GO:0055119 GO:0005890 GO:0010248 GO:0005391 GO:1903416 GO:0042493 GO:0014704 GO:1990239 GO:0043531 GO:0071260 GO:0016311 GO:0005768 GO:0016791 GO:0086004 |
| sp|P05161|ISG15_ HUMAN | GO:0005654 GO:0005515 GO:0019985 GO:0051607 GO:0042742 GO:0005576 GO:0005829 GO:0032649 GO:0031386 GO:0032480 GO:0045071 GO:0060337 GO:0045648 GO:0031397 GO:0019941 GO:0032020 |
| sp|P05204|HMGN2_ HUMAN | GO:0050789 GO:0003676 GO:0044424 |
| sp|P05556|ITB1_ HUMAN | GO:2000811 GO:0043236 GO:0014823 GO:0034679 GO:0001894 GO:0019901 GO:0034698 GO:0045214 GO:0034667 GO:0046718 GO:0002020 GO:0071438 GO:0010710 GO:0001968 GO:0050731 GO:0006874 GO:0050901 GO:0003779 GO:0046982 GO:0001701 GO:0030335 GO:0030183 GO:0034681 GO:0005604 GO:0055007 GO:0008354 GO:0035024 GO:0045807 GO:0014704 GO:0043065 GO:0046872 GO:0005925 GO:0010976 GO:0034678 GO:0071260 GO:0043149 GO:0007229 GO:0007161 GO:0051726 GO:0008284 GO:0008277 GO:0042470 GO:0034666 GO:0097060 GO:0007596 GO:0055037 GO:0032587 GO:0002042 GO:0001708 GO:0008285 GO:0021943 GO:0001948 GO:0070062 GO:0009897 GO:0071305 GO:0042277 GO:0031345 GO:0048333 GO:0090004 GO:0006968 GO:0045121 GO:0071479 GO:0071404 GO:0001669 GO:0019904 GO:0043547 GO:0030027 GO:0007156 GO:0034680 GO:0007411 GO:0001618 GO:0070830 GO:0034665 GO:0045665 GO:0035748 GO:0032594 GO:0098639 GO:0034677 GO:0007179 GO:0033631 GO:0010811 GO:0030198 GO:0050776 GO:0031594 GO:0043410 GO:0048471 GO:0048813 GO:0051393 GO:0030175 GO:0048675 GO:0031623 GO:0000082 GO:0005178 GO:0032154 GO:0042383 GO:0042493 GO:0043197 GO:0030056 GO:0060135 GO:0034113 GO:0008542 |
| sp|P05787|K2C8_ HUMAN | GO:0051599 GO:0005911 GO:0005654 GO:0097284 GO:0005198 GO:0016010 GO:0051707 GO:0043034 GO:0045214 GO:0033209 GO:0045095 GO:0042383 GO:0000904 GO:0060706 GO:0016363 GO:0030018 GO:0032403 GO:0070062 GO:0016032 GO:0097191 GO:0097110 |
| sp|P06132|DCUP_ HUMAN | GO:0046689 GO:0005829 GO:0008198 GO:0045471 GO:0001889 GO:0010039 GO:0032355 GO:0060992 GO:0006782 GO:0004853 GO:0014075 GO:0005515 GO:0071243 GO:0046502 GO:0005654 GO:0051597 |
| sp|P06703|S10A6_ HUMAN | GO:0051781 GO:0008270 GO:0050786 GO:0007409 GO:0044822 GO:0048306 GO:0098586 GO:0044548 GO:0005615 GO:0047485 GO:0005509 GO:0043123 GO:0005829 GO:0001726 GO:0005635 GO:0043025 GO:0005523 GO:0015075 GO:0034220 GO:0048471 GO:0070062 GO:0042803 GO:0048146 GO:0019899 GO:0031234 GO:0007049 |
| sp|P06730|IF4E_ HUMAN | GO:0006413 GO:0030324 GO:0017148 GO:0045665 GO:0008286 GO:0006406 GO:0019827 GO:0044822 GO:0016442 GO:0045931 GO:0031370 GO:0003743 GO:0016281 GO:0005829 GO:0019221 GO:0010494 GO:0005845 GO:0070491 GO:0033391 GO:0000082 GO:0000932 GO:0001662 GO:0048471 GO:0070062 GO:0016032 GO:0000339 GO:0000289 GO:0019899 |
| sp|P06753|TPM3_ HUMAN | GO:0030863 GO:0030426 GO:0031941 GO:0030049 GO:0005829 GO:0008092 GO:0007420 GO:0032154 GO:0005903 GO:0005862 GO:0001725 GO:0070062 GO:0002102 |
| sp|P07108|ACBP_ HUMAN | GO:0006810 GO:0018345 GO:0007611 GO:0000159 GO:0042742 GO:0001942 GO:0046983 GO:0005794 GO:0060291 GO:0036151 GO:0021670 GO:0016021 GO:0097038 GO:0006914 GO:0008289 GO:0008601 GO:0001662 GO:0030156 GO:0006641 GO:0070062 GO:0036042 GO:0005778 GO:0034047 GO:0005739 |
| sp|P07305|H10_ HUMAN | GO:0000786 GO:0006309 GO:0030261 GO:0005730 GO:0033554 GO:0005719 GO:0044822 GO:0005515 GO:0015629 GO:0031490 GO:0006334 GO:0005654 GO:0005794 |
| sp|P07384|CAN1_ HUMAN | GO:0008092 GO:0005829 GO:0005764 GO:0005509 GO:0005739 GO:0022617 GO:0070062 GO:0016540 GO:0032801 GO:0005925 GO:0004198 GO:0060056 GO:0008284 GO:0005886 |
| sp|P07737|PROF1_ HUMAN | GO:0005856 GO:0003785 GO:1900029 GO:0005925 GO:0030838 GO:0001843 GO:0043005 GO:0051054 GO:0032781 GO:0072562 GO:0005102 GO:0005829 GO:0030168 GO:0070064 GO:0000774 GO:0045944 GO:0017048 GO:0051496 GO:0044822 GO:0070062 GO:0002576 GO:0050434 GO:0042989 GO:0045202 GO:0050821 GO:0051497 GO:0071363 GO:0005546 GO:0010634 GO:0005634 GO:0005938 GO:0016020 |
| sp|P07741|APT_ HUMAN | GO:0003999 GO:0005654 GO:0007595 GO:0006168 GO:0032869 GO:0016208 GO:0007625 GO:0002055 GO:0005829 GO:0070062 GO:0044209 |
| sp|P07814|SYEP_ HUMAN | GO:0017148 GO:0016020 GO:0004827 GO:0051020 GO:0006400 GO:0006461 GO:0035613 GO:0017101 GO:0005829 GO:0006433 GO:0001887 GO:0071346 GO:0030529 GO:0004818 GO:0006424 GO:0097452 GO:0005524 |
| sp|P07919|QCR6_ HUMAN | GO:0051291 GO:0009060 GO:1902600 GO:0008121 GO:0032403 GO:0005750 GO:0006122 |
| sp|P08243|ASNS_ HUMAN | GO:0043200 GO:0048037 GO:0006541 GO:0043066 GO:0009612 GO:0032354 GO:0045931 GO:0009636 GO:0044267 GO:0005829 GO:0036499 GO:0042149 GO:0009416 GO:0031427 GO:0042803 GO:0001889 GO:0070981 GO:0032870 GO:0005524 GO:0004066 |
| sp|P08397|HEM3_ HUMAN | GO:0043200 GO:0051597 GO:0043176 GO:0071549 GO:0031100 GO:0006782 GO:0071284 GO:0032355 GO:0009743 GO:0031406 GO:0018160 GO:0005829 GO:0071236 GO:0010043 GO:0050662 GO:0001666 GO:0033273 GO:0030424 GO:0000793 GO:0071418 GO:0042493 GO:0071243 GO:0071345 GO:0004418 GO:0044281 GO:0004852 GO:0005634 GO:0032025 GO:0048708 |
| sp|P08574|CY1_ HUMAN | GO:0046872 GO:0045155 GO:0005743 GO:0070469 GO:0005515 GO:0044281 GO:0020037 GO:0006810 GO:0033762 GO:0016021 GO:0005634 GO:0022904 |
| sp|P08579|RU2B_ HUMAN | GO:0070990 GO:0019013 GO:0035614 GO:0005730 GO:0005686 GO:0098792 GO:0000398 GO:0005515 GO:0000166 GO:0071013 GO:0005654 GO:0002230 |
| sp|P08708|RS17_ HUMAN | GO:0005925 GO:0000184 GO:0006415 GO:0006413 GO:0016020 GO:0034101 GO:0000028 GO:0044822 GO:0016259 GO:0006414 GO:0016787 GO:0003735 GO:0019083 GO:0006614 GO:0070062 GO:0006364 GO:0022627 |
| sp|P08727|K1C19_ HUMAN | GO:0060706 GO:0043627 GO:0030018 GO:0008307 GO:0045214 GO:0043034 GO:1990357 GO:0070062 GO:0032403 GO:0007219 GO:0005200 GO:0042383 GO:0016032 GO:0016010 GO:0005882 |
| sp|P08754|GNAI3_ HUMAN | GO:0004871 GO:0005525 GO:0006906 GO:0046039 GO:0051301 GO:0030496 GO:0019003 GO:0008016 GO:0005834 GO:0000287 GO:0045121 GO:0031683 GO:0030168 GO:1904322 GO:0043949 GO:0000139 GO:0007212 GO:0050805 GO:0007194 GO:0070062 GO:0042588 GO:0019904 GO:0005765 GO:0031821 GO:0007049 GO:0003924 GO:0032794 GO:0007193 GO:0005634 GO:0005813 GO:0051048 |
| sp|P09012|SNRPA_ HUMAN | GO:0035614 GO:0030619 GO:0005681 GO:1990446 GO:0005730 GO:0000398 GO:0005685 GO:0044822 GO:1900363 GO:0005515 GO:0005654 GO:0005737 |
| sp|P09327|VILI_ HUMAN | GO:0061041 GO:0042803 GO:0030836 GO:0005903 GO:0032433 GO:0071364 GO:0030027 GO:0005654 GO:0035729 GO:0035727 GO:0043027 GO:0032432 GO:0060327 GO:0051016 GO:0005886 GO:0005737 GO:0040018 GO:0070062 GO:0007173 GO:1902896 GO:0043154 GO:0032532 GO:0090004 GO:0032233 GO:0030855 GO:0005902 GO:2000394 GO:0051015 GO:0005546 GO:0005509 GO:0008360 GO:0010634 GO:0051125 GO:0051014 GO:0001726 GO:0001951 GO:0030033 GO:0009617 |
| sp|P09382|LEG1_ HUMAN | GO:0004871 GO:0071333 GO:0005534 GO:0043236 GO:0042981 GO:0033555 GO:0031295 GO:0044822 GO:0071407 GO:0002317 GO:0005615 GO:0045445 GO:0043123 GO:0034120 GO:0005737 GO:0045185 GO:0009986 GO:0001948 GO:2001200 GO:0005578 GO:0007157 GO:0010812 GO:0048678 GO:0030395 GO:0042493 GO:0046598 GO:0010977 GO:0070062 GO:0042803 GO:0005634 |
| sp|P09496|CLCA_ HUMAN | GO:0007411 GO:0030669 GO:0030132 GO:0042059 GO:0005198 GO:0071439 GO:0032050 GO:0048011 GO:0032588 GO:0030130 GO:0036020 GO:0006886 GO:0005829 GO:0061024 GO:0019886 GO:0042277 GO:0006892 GO:0072583 |
| sp|P09497|CLCB_ HUMAN | GO:0042277 GO:0030132 GO:0016183 GO:0030130 GO:0005326 GO:0032050 GO:0060170 GO:0007269 GO:0005802 GO:0006886 GO:0005198 |
| sp|P09758|TACD2_ HUMAN | GO:0009986 GO:0007601 GO:0005887 GO:0010633 GO:0050678 GO:0009925 GO:0004872 GO:0005829 GO:0005615 GO:2000738 GO:0070062 GO:0005515 GO:0007166 GO:0051497 GO:1900028 GO:0090191 GO:0005634 GO:0016328 GO:1900025 |
| sp|P09917|LOX5_ HUMAN | GO:0005829 GO:0016363 GO:0019369 GO:0055114 GO:0005641 GO:0019370 GO:0031965 GO:0002526 GO:0030425 GO:0002540 GO:0007584 GO:0045907 GO:0005615 GO:0019233 GO:0019372 GO:0005515 GO:0042383 GO:2001300 GO:0005506 GO:0055093 GO:0004051 |
| sp|P10114|RAP2A_ HUMAN | GO:0048814 GO:0005525 GO:1903506 GO:0005923 GO:0030496 GO:0045198 GO:0030336 GO:0031532 GO:0019003 GO:0005829 GO:0046328 GO:0072659 GO:0003713 GO:0005886 GO:0055038 GO:0070062 GO:0006886 GO:0032486 GO:0090557 GO:0005515 GO:0006913 GO:0035690 GO:0003924 GO:0061097 GO:0030033 GO:0031954 GO:0044291 |
| sp|P10606|COX5B_ HUMAN | GO:0004129 GO:0006367 GO:1902600 GO:0005743 GO:0046872 GO:0043434 GO:0070062 GO:0022904 GO:0005515 GO:0045277 GO:0007585 GO:0044281 |
| sp|P11177|ODPB_ HUMAN | GO:0004739 GO:0046487 GO:0061732 GO:0070062 GO:0034604 GO:0006099 GO:0045254 GO:0005515 GO:0005759 GO:0006006 GO:0005654 GO:0010510 |
| sp|P11182|ODB2_ HUMAN | GO:0046487 GO:0031625 GO:0042645 GO:0034641 GO:0009083 GO:0043754 GO:0005947 |
| sp|P11216|PYGB_ HUMAN | GO:0008184 GO:0008144 GO:0005980 GO:0030424 GO:0070062 GO:0006006 GO:0042803 GO:0005737 GO:0030170 GO:0030246 GO:0016020 |
| sp|P11233|RALA_ HUMAN | GO:0005925 GO:0007265 GO:0017157 GO:0030659 GO:0048011 GO:0005525 GO:0017022 GO:0043209 GO:0005829 GO:0061024 GO:0051665 GO:0051117 GO:0030139 GO:0031625 GO:0032154 GO:0001843 GO:0006935 GO:0030496 GO:0031755 GO:0070062 GO:0016032 GO:0019003 GO:0003924 GO:0031532 GO:0005886 GO:0008152 GO:0051491 GO:0000910 |
| sp|P11274|BCR_ HUMAN | GO:0043314 GO:0004713 GO:0035023 GO:0050728 GO:0004674 GO:0050885 GO:0005089 GO:0014069 GO:0007420 GO:0018108 GO:0002692 GO:0043234 GO:0043547 GO:0005829 GO:0051726 GO:0048008 GO:0030054 GO:0070062 GO:0005524 GO:0045211 GO:0043114 GO:0042472 GO:0046777 GO:0050766 GO:0030036 GO:0005096 GO:0019899 GO:0032496 GO:0060313 |
| sp|P11387|TOP1_ HUMAN | GO:0001650 GO:0005654 GO:0003917 GO:0010332 GO:0032922 GO:0012501 GO:0031100 GO:0044822 GO:0001046 GO:0071373 GO:0040016 GO:0016310 GO:0006265 GO:0001651 GO:0009303 GO:0007059 GO:0006338 GO:0042493 GO:0051591 GO:0000932 GO:0016925 GO:0009266 GO:0032403 GO:0016032 GO:0043687 GO:0031298 GO:0003918 GO:0006260 GO:0043204 GO:0031490 |
| sp|P11388|TOP2A_ HUMAN | GO:0043065 GO:0016925 GO:0043130 GO:0005080 GO:0042803 GO:0021707 GO:0019035 GO:0008022 GO:0043687 GO:0071107 GO:0000712 GO:0006266 GO:0006974 GO:0000819 GO:0005654 GO:0000287 GO:0044774 GO:0045870 GO:0002244 GO:0009330 GO:0008144 GO:0005814 GO:0043566 GO:0040016 GO:0045944 GO:0000793 GO:0007568 GO:0044822 GO:0030263 GO:0000228 GO:0005524 GO:0003682 GO:0046982 GO:0043565 GO:0008301 GO:0042493 GO:0006265 GO:0003918 GO:0005730 GO:0042752 GO:0009295 GO:0042826 GO:0021702 |
| sp|P11413|G6PD_ HUMAN | GO:0006006 GO:0042803 GO:0046390 GO:0043249 GO:0019322 GO:0032613 GO:0004345 GO:0032094 GO:0006740 GO:0050661 GO:0010041 GO:0005829 GO:0009051 GO:0014070 GO:0040014 GO:0006749 GO:0021762 GO:0045471 GO:0010734 GO:0002033 GO:0006741 GO:0070062 GO:0043523 GO:0006695 GO:0006367 GO:0005536 GO:0061052 GO:2000378 GO:0009898 GO:1904879 GO:0005634 GO:0005813 GO:0001998 GO:0034599 GO:0032615 |
| sp|P12004|PCNA_ HUMAN | GO:0044849 GO:1902065 GO:0005663 GO:0016925 GO:0097421 GO:0042769 GO:0006298 GO:0000307 GO:0043687 GO:0000722 GO:0006297 GO:0007507 GO:0070182 GO:0045739 GO:0030894 GO:0006283 GO:0070911 GO:0030971 GO:0005652 GO:0000083 GO:0005654 GO:0008134 GO:0030337 GO:0000784 GO:0070301 GO:0032139 GO:0071548 GO:0043626 GO:0045740 GO:0034644 GO:1902990 GO:0005737 GO:0070062 GO:0006272 GO:0006287 GO:0070557 GO:0032077 GO:0042802 GO:0000701 GO:0014823 GO:0000084 GO:0071466 GO:0000122 GO:0032355 GO:0003682 GO:0042276 GO:0008283 GO:0030855 GO:0046686 GO:0031297 GO:0003684 GO:0070987 GO:0005813 GO:0035035 GO:0043596 GO:0032405 GO:0030331 GO:0033683 GO:0009617 |
| sp|P12081|SYHC_ HUMAN | GO:0005829 GO:0005739 GO:0006427 GO:0032543 GO:0005524 GO:0004821 |
| sp|P12270|TPR_ HUMAN | GO:0043523 GO:0030165 GO:0019221 GO:0035249 GO:0043687 GO:0072686 GO:0019054 GO:0007077 GO:0070849 GO:0044615 GO:0007157 GO:0045947 GO:0010827 GO:0005737 GO:0042803 GO:0031047 GO:0000776 GO:0004970 GO:0005487 GO:0031453 GO:0060079 GO:0031072 GO:0043578 GO:0010793 GO:0035457 GO:0031990 GO:0097110 GO:0030054 GO:0014069 GO:0046832 GO:0015631 GO:0008033 GO:0006404 GO:0006811 GO:0005654 GO:0005234 GO:0034399 GO:0090267 GO:1900034 GO:1901673 GO:0005868 GO:0046827 GO:0000189 GO:0031965 GO:0042405 GO:0008855 GO:0000088 GO:0070840 GO:0006308 GO:0003729 GO:0055085 GO:0008328 GO:0044281 GO:0051292 GO:0016925 GO:0000122 GO:0031647 GO:0019083 GO:0009318 GO:0019898 GO:0045211 GO:0051019 GO:0010975 GO:0003682 GO:0021707 GO:0005975 GO:0043197 GO:0043495 GO:0060134 GO:0042307 |
| sp|P12429|ANXA3_ HUMAN | GO:0006909 GO:0030670 GO:0043086 GO:0042581 GO:0031100 GO:0070848 GO:0045766 GO:0021766 GO:0048306 GO:0051091 GO:0005509 GO:0042742 GO:0051384 GO:0030425 GO:0019834 GO:0051054 GO:0043025 GO:0030424 GO:0005544 GO:0043312 GO:0010595 GO:0070062 GO:0005886 |
| sp|P12532|KCRU_ HUMAN | GO:0046314 GO:0004111 GO:0005743 GO:0070062 GO:0043209 GO:0032091 GO:0005524 GO:0005515 GO:0016310 GO:0006595 GO:0006600 GO:0043066 |
| sp|P12931|SRC_ HUMAN | GO:0007173 GO:0020037 GO:0034614 GO:2000811 GO:0030331 GO:0010907 GO:0009615 GO:0050900 GO:0033146 GO:0071393 GO:0045056 GO:0005770 GO:0036035 GO:0048477 GO:0031667 GO:0033625 GO:0050731 GO:0038083 GO:0010954 GO:0014911 GO:2000394 GO:0046875 GO:0070555 GO:1900182 GO:2001243 GO:0045892 GO:0005158 GO:0043406 GO:0009612 GO:0042542 GO:0005743 GO:0002223 GO:0005080 GO:0043065 GO:0031648 GO:0060065 GO:0051219 GO:0071456 GO:0070851 GO:0043149 GO:0007229 GO:0005884 GO:0038096 GO:0051602 GO:0097110 GO:0045124 GO:0031954 GO:0010634 GO:0060491 GO:0043552 GO:0046628 GO:0030900 GO:0032587 GO:0045737 GO:0014069 GO:0048010 GO:0051385 GO:0048011 GO:0034446 GO:0071498 GO:0070062 GO:0061024 GO:0086098 GO:0043114 GO:0030168 GO:0043005 GO:0005634 GO:2001286 GO:0007172 GO:0010641 GO:0008022 GO:0005829 GO:0036120 GO:0071803 GO:0051897 GO:0071222 GO:0007411 GO:0042127 GO:0048013 GO:0071398 GO:0018105 GO:0060444 GO:0043154 GO:2000641 GO:0043393 GO:0005524 GO:0010447 GO:0007179 GO:0050715 GO:0090263 GO:0032148 GO:0016032 GO:0048471 GO:0044325 GO:0070374 GO:0051974 GO:2000573 GO:2001237 GO:0008543 GO:0031295 GO:0005178 GO:0005901 GO:0005070 GO:0007265 GO:0042493 GO:0032463 GO:0051895 GO:0051902 GO:0004715 GO:0032211 GO:0031234 GO:0042169 GO:0050847 GO:0045893 GO:0005764 |
| sp|P13010|XRCC5_ HUMAN | GO:0050769 GO:0005654 GO:0032481 GO:0032508 GO:0003684 GO:0008022 GO:0006310 GO:0044822 GO:0048660 GO:0060218 GO:0006303 GO:0071481 GO:0075713 GO:0005730 GO:0005829 GO:0043564 GO:0044212 GO:0070419 GO:0045892 GO:0031625 GO:1904430 GO:0000783 GO:0071480 GO:0003691 GO:0051575 GO:0004003 GO:0005886 GO:0045087 GO:0005524 GO:0044877 |
| sp|P13073|COX41_ HUMAN | GO:0006123 GO:0004129 GO:0006367 GO:0005751 GO:0005515 GO:0016021 GO:0009060 GO:1902600 GO:0070062 GO:0005634 GO:0007584 |
| sp|P13639|EF2_ HUMAN | GO:0045121 GO:0002039 GO:0051593 GO:0042542 GO:0019901 GO:0014009 GO:0045471 GO:0044822 GO:0008097 GO:0003009 GO:0003746 GO:0007568 GO:0014076 GO:1990416 GO:0006414 GO:0005525 GO:0032355 GO:0002931 GO:0016235 GO:0043022 GO:0005829 GO:0035914 GO:0034976 GO:2000767 GO:0042788 GO:0051015 GO:0002244 GO:0017183 GO:0070062 GO:0043687 GO:0003924 GO:0005886 GO:0005634 |
| sp|P13645|K1C10_ HUMAN | GO:0005737 GO:0005615 GO:0030280 GO:0071277 GO:0030216 GO:0016020 GO:0005634 GO:0070062 GO:0045095 |
| sp|P13688|CEAM1_ HUMAN | GO:0001525 GO:0071575 GO:0007156 GO:0010832 GO:0007229 GO:0070062 GO:0034109 GO:0050900 GO:2000811 GO:0034235 GO:0042803 GO:0016323 GO:0007596 |
| sp|P13995|MTDC_ HUMAN | GO:0046653 GO:0055114 GO:0005739 GO:0004487 GO:0042301 GO:0004488 GO:0009396 GO:0005615 GO:0004477 GO:0006730 GO:0005515 GO:0000287 GO:0004329 |
| sp|P14324|FPPS_ HUMAN | GO:0004337 GO:0005829 GO:0016021 GO:0033384 GO:0005739 GO:0046872 GO:0045337 GO:0004161 GO:0044822 GO:0016032 GO:0005654 GO:0006695 |
| sp|P14635|CCNB1_ HUMAN | GO:0006461 GO:0005113 GO:0071407 GO:0048146 GO:0055015 GO:0031442 GO:0000236 GO:0001701 GO:0000082 GO:0007080 GO:0009612 GO:0033129 GO:0007077 GO:0060045 GO:0005829 GO:0071456 GO:0051437 GO:0005654 GO:0007283 GO:0031145 GO:0000088 GO:0001933 GO:0051987 GO:0048565 GO:0000922 GO:0042246 GO:0071283 GO:0010629 GO:0032403 GO:0043148 GO:0045931 GO:0019901 GO:0035173 GO:1904145 GO:0000086 GO:0042493 GO:0046680 GO:0090266 GO:0005813 GO:0000942 GO:2000775 GO:0060623 GO:0016020 GO:0071398 |
| sp|P14735|IDE_ HUMAN | GO:0051603 GO:1901143 GO:0008340 GO:0051291 GO:0005654 GO:0046718 GO:0010815 GO:0031626 GO:0005102 GO:0008270 GO:0051289 GO:0008286 GO:0043559 GO:0031597 GO:0005615 GO:0001618 GO:0043130 GO:0001540 GO:0009986 GO:0001948 GO:0045861 GO:0016485 GO:0004222 GO:0050435 GO:0016021 GO:0016887 GO:0042803 GO:0010992 GO:0005886 GO:0032461 GO:0005524 GO:0005782 GO:0042447 GO:0005739 |
| sp|P14854|CX6B1_ HUMAN | GO:0004129 GO:0021762 GO:0006367 GO:1902600 GO:0005743 GO:0005758 GO:0022904 GO:0045277 GO:0044281 |
| sp|P14866|HNRPL_ HUMAN | GO:0005654 GO:0034198 GO:0005515 GO:0016020 GO:0000166 GO:0003730 GO:0048025 GO:1902416 GO:0045727 GO:0005829 GO:0044212 GO:0045120 GO:1990715 GO:0030529 GO:0048471 GO:0070062 GO:0019013 |
| sp|P14927|QCR7_ HUMAN | GO:0006810 GO:0051291 GO:0005750 GO:0032403 GO:0006122 GO:0009060 |
| sp|P15559|NQO1_ HUMAN | GO:0004128 GO:0043086 GO:0006521 GO:0043066 GO:0045471 GO:0044822 GO:0003955 GO:0007568 GO:0032355 GO:0019430 GO:0005829 GO:0006809 GO:0043025 GO:0042802 GO:0006805 GO:0004784 GO:0016021 GO:0055114 GO:0070062 GO:0043525 GO:0007271 GO:0006595 GO:0007584 |
| sp|P15880|RS2_ HUMAN | GO:0005925 GO:0000184 GO:0006415 GO:0006413 GO:0005654 GO:0016020 GO:0016259 GO:0006414 GO:0005730 GO:0003735 GO:0051347 GO:0019083 GO:0006614 GO:0070062 GO:0017134 GO:0019899 GO:0003729 GO:0022627 |
| sp|P15924|DESP_ HUMAN | GO:0050839 GO:0005080 GO:0086091 GO:0006921 GO:0001533 GO:0031214 GO:0098911 GO:0034332 GO:0044822 GO:0086083 GO:0016323 GO:0071460 GO:0005882 GO:0005737 GO:0030674 GO:0030057 GO:0042060 GO:0086073 GO:0005578 GO:0002934 GO:0018149 GO:0045109 GO:0070062 GO:0030216 GO:0005916 GO:0005634 GO:0005200 GO:0003223 GO:0097110 GO:0071896 |
| sp|P16070|CD44_ HUMAN | GO:0002246 GO:0033138 GO:0005925 GO:0005887 GO:0007160 GO:0070374 GO:0005540 GO:1902166 GO:0044344 GO:0016323 GO:0007596 GO:1900625 GO:0033674 GO:0060333 GO:0016055 GO:0035692 GO:0050900 GO:0043518 GO:0050731 GO:0044281 GO:0004896 GO:0060442 GO:0070062 GO:0051216 GO:0005794 GO:0009897 GO:0022617 GO:0005975 GO:0004415 GO:0043154 GO:0001658 GO:0005518 GO:0010628 GO:0034116 GO:0030214 |
| sp|P16104|H2AX_ HUMAN | GO:0000781 GO:0005654 GO:0042393 GO:0003684 GO:0090398 GO:0001741 GO:0008963 GO:0051321 GO:0007283 GO:0000724 GO:0006303 GO:0005789 GO:0006342 GO:0000786 GO:0046982 GO:0006487 GO:0021987 GO:0000077 GO:0035861 GO:0001673 GO:0016021 GO:0071480 GO:0000794 GO:0000790 GO:0070062 GO:0016032 GO:0006334 GO:0019899 GO:0019408 GO:0045739 GO:0005657 |
| sp|P16152|CBR1_ HUMAN | GO:0004090 GO:0005829 GO:0055114 GO:0050221 GO:0070062 GO:0047021 GO:0042373 GO:0030855 GO:0019371 GO:0005902 GO:0017144 GO:0016655 GO:0005634 GO:0005886 |
| sp|P16401|H15_ HUMAN | GO:0030307 GO:0000122 GO:0042826 GO:0071169 GO:0044822 GO:0007517 GO:0050821 GO:0000786 GO:0005720 GO:0070062 GO:0006334 GO:0051574 GO:0031490 |
| sp|P16402|H13_ HUMAN | GO:0098532 GO:0000122 GO:0005515 GO:0044822 GO:0005719 GO:0016584 GO:0030261 GO:0000786 GO:0005730 GO:0005720 GO:0080182 GO:0070062 GO:0006334 GO:0031490 |
| sp|P16403|H12_ HUMAN | GO:0098532 GO:0000122 GO:0005515 GO:0044822 GO:0005719 GO:0016584 GO:0030261 GO:0000786 GO:0005730 GO:0005720 GO:0080182 GO:0070062 GO:0006334 GO:0035327 GO:0031490 |
| sp|P16422|EPCAM_ HUMAN | GO:2000147 GO:0043066 GO:2000048 GO:0001657 GO:0016323 GO:0098641 GO:0009986 GO:0016328 GO:0023019 GO:0016021 GO:0005923 GO:0048863 GO:0045944 GO:0032403 GO:0070062 GO:0016324 GO:2000648 |
| sp|P16930|FAAA_ HUMAN | GO:0005829 GO:0006527 GO:0046872 GO:0004334 GO:0006559 GO:0070062 GO:0034641 GO:0006572 GO:0005515 |
| sp|P17301|ITA2_ HUMAN | GO:0005925 GO:0033343 GO:0002687 GO:0033627 GO:0001618 GO:0014850 GO:0071392 GO:0007411 GO:0045727 GO:0071107 GO:0043679 GO:0046872 GO:0005178 GO:0006971 GO:0045785 GO:0014911 GO:0007596 GO:0048333 GO:0031346 GO:0038065 GO:0014075 GO:0001666 GO:0050966 GO:0034666 GO:0043589 GO:0051971 GO:0098639 GO:0045178 GO:0048471 GO:0033591 GO:0045184 GO:0009897 GO:0030198 GO:0060100 GO:0038064 GO:0046982 GO:0070365 GO:0050729 GO:0007229 GO:0007565 GO:0045987 GO:0046718 GO:0006929 GO:0010634 GO:0030879 GO:0042493 GO:0010694 GO:0048661 GO:0005634 GO:0071260 GO:0043236 GO:0048041 GO:0032967 GO:0043388 GO:0050927 |
| sp|P17568|NDUB7_ HUMAN | GO:0008137 GO:0005747 GO:0005758 GO:0006120 GO:0032981 |
| sp|P17706|PTN2_ HUMAN | GO:1903899 GO:0050728 GO:0060339 GO:0016021 GO:1902215 GO:0045650 GO:0005783 GO:1902212 GO:1902237 GO:0030971 GO:1902202 GO:2000587 GO:0005178 GO:1902227 GO:0005654 GO:0042518 GO:0050860 GO:0070373 GO:0042593 GO:0070104 GO:0042524 GO:0042512 GO:0005886 GO:0042059 GO:0045722 GO:1902233 GO:0004726 GO:0030183 GO:0010804 GO:0008285 GO:0046627 GO:0005793 GO:1902206 GO:0019905 GO:0061099 GO:0060336 GO:0030218 GO:0035335 GO:0042527 GO:0050922 GO:0010888 |
| sp|P17812|PYRG1_ HUMAN | GO:0005829 GO:0006541 GO:0003883 GO:0005524 GO:0044210 GO:0042098 GO:0042493 GO:0015949 GO:0016020 GO:0042100 |
| sp|P17844|DDX5_ HUMAN | GO:0009299 GO:0005654 GO:0000122 GO:0043517 GO:0045667 GO:0016020 GO:0000381 GO:0044822 GO:0072332 GO:0060765 GO:0048306 GO:0036002 GO:0033148 GO:0005730 GO:0071013 GO:0003713 GO:0030331 GO:0045944 GO:0016049 GO:0070062 GO:0019899 GO:0005516 GO:0004004 GO:0045069 GO:2001014 GO:0005524 GO:0050681 GO:0010501 GO:0001701 GO:0007623 |
| sp|P17858|PFKAL_ HUMAN | GO:0005945 GO:0009749 GO:0004331 GO:0016020 GO:0051289 GO:0030388 GO:0046676 GO:0046872 GO:0070095 GO:0042802 GO:0016311 GO:0070062 GO:0070061 GO:0061621 GO:0006002 GO:0003872 GO:0005524 GO:0019900 |
| sp|P17987|TCPA_ HUMAN | GO:0051082 GO:0031625 GO:0007021 GO:0000242 GO:0090666 GO:0002199 GO:2000109 GO:0007339 GO:0005720 GO:1904871 GO:0043209 GO:1904874 GO:0044822 GO:0070062 GO:0001669 GO:0007286 GO:0005832 GO:0005794 GO:0005524 GO:0051084 GO:0044053 GO:0032212 GO:0050821 GO:0005874 GO:0044267 GO:1901998 GO:0051973 GO:0044297 GO:1904851 |
| sp|P18077|RL35A_ HUMAN | GO:0000184 GO:0006415 GO:0006413 GO:0005515 GO:0016020 GO:0002181 GO:0044822 GO:0016259 GO:0006414 GO:0042273 GO:0022625 GO:0043009 GO:0030218 GO:0003735 GO:0019083 GO:0000049 GO:0006614 GO:0070062 GO:0006364 GO:0005739 |
| sp|P18283|GPX2_ HUMAN | GO:0042744 GO:1902531 GO:0048468 GO:0017124 GO:0009609 GO:0008631 GO:0042542 GO:0090201 GO:0010269 GO:0001659 GO:0006195 GO:0097413 GO:0005829 GO:1902905 GO:0006749 GO:0005759 GO:0019372 GO:0014902 GO:0004602 GO:0009650 GO:0033599 GO:0042060 GO:0002862 GO:0070062 GO:0051702 GO:0006367 GO:0055114 GO:0043154 GO:0009055 GO:0019369 GO:0098869 GO:0045454 GO:0061136 GO:0060047 GO:0006144 GO:1902042 GO:0040029 |
| sp|P18583|SON_ HUMAN | GO:0043484 GO:0000910 GO:0051726 GO:0050733 GO:0016607 GO:0000226 GO:0003677 GO:0044822 GO:0006397 GO:0043066 |
| sp|P18615|NELFE_ HUMAN | GO:0005515 GO:0070374 GO:0000166 GO:0044822 GO:1900364 GO:0051571 GO:0032021 GO:0034244 GO:0050434 GO:0045944 GO:0003682 GO:0005739 |
| sp|P19525|E2AK2_ HUMAN | GO:0043065 GO:0009636 GO:0034047 GO:0009612 GO:0000186 GO:0018108 GO:1900225 GO:0005829 GO:1901224 GO:0004694 GO:0030968 GO:0019054 GO:0043330 GO:0017148 GO:0003725 GO:0045087 GO:1902033 GO:0030683 GO:0044822 GO:0032722 GO:0048471 GO:1902036 GO:0051092 GO:0005515 GO:0034198 GO:0005524 GO:0010998 GO:0046777 GO:0045071 GO:0005840 GO:0033197 GO:0008601 GO:0032874 GO:0033689 GO:0043066 GO:0004715 GO:0005634 GO:0035455 GO:0016020 GO:0032496 |
| sp|P19623|SPEE_ HUMAN | GO:0016477 GO:0005829 GO:0038083 GO:0008295 GO:0045087 GO:0007169 GO:0005102 GO:0005524 GO:0030154 GO:0004766 GO:0042127 GO:0042803 GO:0004715 GO:0044281 GO:0031234 |
| sp|P19784|CSK22_ HUMAN | GO:0004674 GO:0000236 GO:0006915 GO:0047485 GO:0071174 GO:0007411 GO:0000087 GO:0005956 GO:0005829 GO:1903146 GO:0031519 GO:0016055 GO:0005524 GO:1903955 GO:0006355 GO:0006468 |
| sp|P20290|BTF3_ HUMAN | GO:0016021 GO:0005730 GO:0072643 GO:0006355 GO:0002456 GO:0044822 GO:0005515 GO:0001701 GO:0005794 GO:0005886 GO:0006366 |
| sp|P20585|MSH3_ HUMAN | GO:0032357 GO:0045910 GO:0005654 GO:0000403 GO:0000404 GO:0016020 GO:0016447 GO:0032302 GO:0032181 GO:0003697 GO:0032142 GO:0019237 GO:0016446 GO:0000406 GO:0051096 GO:0042803 GO:0032137 GO:0019899 GO:0006298 GO:0005524 GO:0043570 |
| sp|P20674|COX5A_ HUMAN | GO:0006123 GO:0004129 GO:0006367 GO:0005751 GO:0005515 GO:0043209 GO:0046872 GO:1902600 GO:0070062 |
| sp|P20700|LMNB1_ HUMAN | GO:0005654 GO:0005198 GO:0006921 GO:0005637 GO:0046677 GO:0043274 GO:0005737 GO:0008432 GO:0005638 GO:1904609 GO:0042493 GO:0016363 GO:0046330 GO:0022008 GO:0071386 GO:0031662 |
| sp|P21291|CSRP1_ HUMAN | GO:0070527 GO:0008270 GO:0051393 GO:0070062 GO:0031252 GO:0030036 GO:0001725 GO:0007399 GO:0044822 GO:0005925 GO:0051219 GO:0005737 GO:0005634 |
| sp|P21333|FLNA_ HUMAN | GO:0004871 GO:0043198 GO:0005080 GO:0005925 GO:0042803 GO:0051220 GO:0017160 GO:0005802 GO:0032231 GO:2001046 GO:0042993 GO:0007195 GO:0005903 GO:0031532 GO:0042384 GO:0031523 GO:0070527 GO:0042177 GO:0005884 GO:0005829 GO:0016479 GO:0008134 GO:0043433 GO:0030863 GO:0001837 GO:0032432 GO:0043123 GO:0045022 GO:0045216 GO:0005886 GO:0001974 GO:0044822 GO:0070062 GO:0002576 GO:0048471 GO:0007264 GO:0034329 GO:0003007 GO:0031852 GO:0034394 GO:0001948 GO:0050821 GO:0090307 GO:0034988 GO:0043113 GO:0001525 GO:1900026 GO:0051015 GO:0051764 GO:0043066 GO:0005730 GO:0043025 GO:0046332 GO:0042789 GO:0048365 GO:0097440 |
| sp|P21796|VDAC1_ HUMAN | GO:0045121 GO:0007270 GO:0008308 GO:0000166 GO:0019901 GO:1903146 GO:0007612 GO:0015288 GO:0046930 GO:0005743 GO:0006915 GO:0043209 GO:0042645 GO:0005741 GO:0000423 GO:1903959 GO:0006851 GO:0008021 GO:2000378 GO:0001662 GO:0030855 GO:0032403 GO:0070062 GO:0016032 GO:0044325 GO:0005886 GO:0005634 |
| sp|P21912|SDHB_ HUMAN | GO:0006810 GO:0006105 GO:0048039 GO:0051537 GO:0046872 GO:0070062 GO:0005749 GO:0009055 GO:0006099 GO:0051539 GO:0051538 GO:0022904 GO:0005515 GO:0008177 GO:0005654 GO:0005886 |
| sp|P22059|OSBP1_ HUMAN | GO:0005829 GO:0030054 GO:0070273 GO:0005730 GO:0000139 GO:0015248 GO:0015918 GO:0008142 GO:0015485 GO:0019904 GO:0048471 GO:0005654 GO:0005789 GO:0044128 |
| sp|P22087|FBRL_ HUMAN | GO:0009019 GO:0008174 GO:0051994 GO:0034541 GO:0018423 GO:0030792 GO:0015030 GO:0031167 GO:0008650 GO:0016205 GO:0016020 GO:0001649 GO:0008988 GO:0044822 GO:0034931 GO:0001652 GO:0016428 GO:0043770 GO:1990258 GO:0052735 GO:0008172 GO:0001651 GO:0031428 GO:0043791 GO:0009008 GO:0071424 GO:0018707 GO:0043851 GO:0000494 GO:0051117 GO:0043782 GO:0043777 GO:0052666 GO:0005694 GO:0043776 GO:0080012 GO:0052667 GO:0008033 GO:0043780 GO:0048254 GO:0016435 GO:0043834 GO:0009383 GO:0008425 GO:0034807 GO:0001094 GO:0019702 GO:0034933 GO:0032040 GO:0043852 GO:0043833 GO:0070062 GO:0052665 GO:0000179 GO:0052624 GO:0043827 GO:1990259 GO:0043803 GO:0016279 GO:0070677 GO:0004809 |
| sp|P22626|ROA2_ HUMAN | GO:0051385 GO:0000785 GO:0030324 GO:0005654 GO:0000122 GO:0048709 GO:0005515 GO:0016020 GO:0097157 GO:0000166 GO:1990247 GO:0006406 GO:0043047 GO:0031053 GO:0003730 GO:0048025 GO:0008584 GO:0071013 GO:0016233 GO:0071598 GO:0035198 GO:1990715 GO:0070062 GO:0001069 GO:0043204 GO:1990428 |
| sp|P22695|QCR2_ HUMAN | GO:0006810 GO:0008270 GO:0005750 GO:0070062 GO:0043209 GO:0032403 GO:0004222 GO:0006122 GO:0009060 GO:0016485 GO:0005654 |
| sp|P23193|TCEA1_ HUMAN | GO:0005654 GO:0005515 GO:0008270 GO:0032784 GO:0003746 GO:0006283 GO:0006414 GO:0005730 GO:0003677 GO:0030218 GO:0050434 GO:0045944 GO:0006368 GO:1901919 |
| sp|P23229|ITA6_ HUMAN | GO:0005925 GO:0046847 GO:0005178 GO:0030056 GO:0031581 GO:0005604 GO:0007160 GO:0033627 GO:0010668 GO:0030198 GO:0072001 GO:0050873 GO:0009925 GO:0042327 GO:0050900 GO:0007229 GO:0046872 GO:0008305 GO:0035878 GO:0007596 GO:0043065 GO:0022409 GO:0009897 GO:0043588 GO:0048565 GO:0031668 GO:0097186 GO:2001237 GO:0045944 GO:0043547 |
| sp|P23246|SFPQ_ HUMAN | GO:0042754 GO:0000785 GO:0000980 GO:0000122 GO:0070932 GO:0042826 GO:0044822 GO:0090575 GO:0000724 GO:0001047 GO:0045876 GO:0016363 GO:0042382 GO:0000380 GO:0003682 GO:1902177 |
| sp|P23258|TBG1_ HUMAN | GO:0031513 GO:0055037 GO:0005515 GO:0005814 GO:0007052 GO:0005525 GO:0031122 GO:0005881 GO:0005829 GO:0031252 GO:0007020 GO:0000242 GO:0005827 GO:0007088 GO:0036064 GO:0045177 GO:0000794 GO:0000930 GO:0003924 GO:0000086 GO:0008152 GO:0005200 GO:0000212 |
| sp|P23284|PPIB_ HUMAN | GO:0005925 GO:0061077 GO:0070063 GO:0005790 GO:0016020 GO:0060348 GO:0005788 GO:0030198 GO:0040018 GO:0044822 GO:0042470 GO:0050821 GO:0044829 GO:0051082 GO:0042277 GO:1901873 GO:0003755 GO:0034663 GO:0048471 GO:0070062 GO:0005518 GO:0051169 GO:0000413 GO:0005634 |
| sp|P23368|MAOM_ HUMAN | GO:0000790 GO:0001011 GO:0042803 GO:0016525 GO:0043425 GO:0008022 GO:0051287 GO:0008948 GO:0016339 GO:0005759 GO:0000978 GO:0001087 GO:0001093 GO:0003714 GO:0045944 GO:0005886 GO:0001077 GO:0043621 GO:0006367 GO:0005667 GO:1900746 GO:0055114 GO:0045666 GO:0000122 GO:0006108 GO:0003682 GO:0009055 GO:1902031 GO:0046982 GO:0042118 GO:0004471 GO:0065004 GO:0005509 GO:0007156 GO:0070888 |
| sp|P23396|RS3_ HUMAN | GO:1902231 GO:0032357 GO:0005925 GO:0042769 GO:0003735 GO:0007067 GO:0071159 GO:0043507 GO:0032358 GO:0051301 GO:0045739 GO:0031116 GO:0051018 GO:0045738 GO:2001272 GO:0007059 GO:0006414 GO:0003729 GO:1901224 GO:0016259 GO:0044390 GO:0012505 GO:0005759 GO:0032183 GO:1902546 GO:0006413 GO:0005844 GO:0070301 GO:0031397 GO:0017148 GO:0051879 GO:0072686 GO:0006415 GO:0097100 GO:0061481 GO:0008534 GO:0006614 GO:0070062 GO:0051092 GO:0032079 GO:0070181 GO:0019901 GO:0019083 GO:0030425 GO:0051536 GO:0051225 GO:1990090 GO:0051059 GO:0000184 GO:0005743 GO:0030544 GO:0005730 GO:0032587 GO:0022627 GO:0008017 |
| sp|P23526|SAHH_ HUMAN | GO:0043005 GO:0051287 GO:0071268 GO:0004013 GO:0043621 GO:0002439 GO:0042470 GO:0030554 GO:0019510 GO:0005829 GO:0006730 GO:0042745 GO:0042802 GO:0001666 GO:0006805 GO:0001887 GO:0032259 GO:0033353 GO:0070062 GO:0005507 GO:0005634 GO:0007584 |
| sp|P24390|ERD21_ HUMAN | GO:0016021 GO:0005046 GO:0018279 GO:0030133 GO:0061024 GO:0070062 GO:0043687 GO:0033116 GO:0006888 GO:0005801 GO:0006886 GO:0006621 GO:0030663 GO:0005789 |
| sp|P24928|RPB1_ HUMAN | GO:0031047 GO:0006367 GO:0006370 GO:0034587 GO:0001055 GO:0006353 GO:0003968 GO:0044822 GO:0005719 GO:0006283 GO:0001047 GO:0005730 GO:0001172 GO:0006355 GO:0046872 GO:0031625 GO:0014070 GO:0005665 GO:0009790 GO:0000974 GO:0035019 GO:0050434 GO:0033120 GO:0000398 GO:0006368 GO:0071453 |
| sp|P25398|RS12_ HUMAN | GO:0000184 GO:0006415 GO:0006413 GO:0016020 GO:0044822 GO:0016259 GO:0006414 GO:0032543 GO:0005763 GO:0003735 GO:0019083 GO:0006614 GO:0022627 |
| sp|P25786|PSA1_ HUMAN | GO:0048011 GO:0051436 GO:0002479 GO:0090263 GO:0007411 GO:0048010 GO:0000186 GO:0019773 GO:0006977 GO:0005829 GO:0002223 GO:0051437 GO:0001530 GO:0005654 GO:0006595 GO:0031145 GO:0005844 GO:0038061 GO:0007265 GO:0090090 GO:0033209 GO:0002862 GO:0070062 GO:0016032 GO:0006521 GO:0043488 GO:0005515 GO:0000084 GO:0007173 GO:0038095 GO:0003723 GO:0050852 GO:0004298 GO:0000090 GO:0043066 GO:0008543 GO:0005813 GO:0000209 GO:0008286 |
| sp|P25789|PSA4_ HUMAN | GO:0048011 GO:0051436 GO:0002479 GO:0090263 GO:0007411 GO:0048010 GO:0000186 GO:0019773 GO:0006977 GO:0005829 GO:0002223 GO:0051437 GO:0005654 GO:0006595 GO:0031145 GO:0038061 GO:0007265 GO:0005886 GO:0090090 GO:0033209 GO:0070062 GO:0016032 GO:0006521 GO:0043488 GO:0005515 GO:0000084 GO:0007173 GO:0038095 GO:0000932 GO:0050852 GO:0004298 GO:0000090 GO:0043066 GO:0008543 GO:0000209 GO:0008286 |
| sp|P25815|S100P_ HUMAN | GO:0043542 GO:0050786 GO:0007613 GO:0007417 GO:0010033 GO:0048306 GO:0044548 GO:0000902 GO:0008283 GO:0048168 GO:0031528 GO:0005509 GO:0043123 GO:0001726 GO:0043025 GO:0000287 GO:0006950 GO:0048471 GO:0070062 GO:0042803 GO:0022008 GO:0005634 |
| sp|P26368|U2AF2_ HUMAN | GO:0071004 GO:0000166 GO:0006406 GO:0016607 GO:0048025 GO:1903146 GO:0044822 GO:0046660 GO:0031124 GO:0030628 GO:0089701 GO:1903241 GO:1903955 GO:0000243 GO:0006369 GO:0000974 GO:0008187 GO:0033120 GO:0070742 GO:0019013 GO:0019899 |
| sp|P26599|PTBP1_ HUMAN | GO:0032024 GO:1904411 GO:0045727 GO:0003697 GO:0000381 GO:0051148 GO:0048025 GO:0003729 GO:0005654 GO:0070935 GO:0006259 GO:0045944 GO:0003231 GO:0005737 GO:0008187 GO:0000166 GO:0070062 GO:0036002 GO:0005515 GO:0000956 GO:0000014 GO:0001069 GO:0010976 GO:0043565 GO:0044306 GO:0005730 GO:0016020 |
| sp|P27144|KAD4_ HUMAN | GO:0046034 GO:0004550 GO:0005759 GO:0006172 GO:0046899 GO:0005525 GO:0006165 GO:0007420 GO:0015949 GO:0042493 GO:0046039 GO:0009142 GO:0070062 GO:0046033 GO:0001889 GO:0005524 GO:0004017 |
| sp|P27348|1433T_ HUMAN | GO:0005925 GO:0006367 GO:0000075 GO:0030659 GO:0008022 GO:0034766 GO:1900740 GO:0006605 GO:0047485 GO:0005829 GO:0097193 GO:0004497 GO:0045892 GO:0019904 GO:0007264 GO:0043234 GO:0071889 GO:0055114 GO:0021762 GO:0070062 GO:0044325 GO:0005739 |
| sp|P27487|DPP4_ HUMAN | GO:0005925 GO:0045121 GO:0043542 GO:0046718 GO:0005102 GO:0008239 GO:0004252 GO:0031295 GO:0035641 GO:0071438 GO:0005765 GO:0033632 GO:0001618 GO:0009986 GO:0010716 GO:0008284 GO:0030139 GO:0001666 GO:0016021 GO:0002020 GO:0001662 GO:0031258 GO:0006508 GO:0070062 GO:0042803 GO:0046581 GO:0036343 GO:0016324 |
| sp|P27694|RFA1_ HUMAN | GO:0030097 GO:0005662 GO:0000075 GO:0042769 GO:0005515 GO:0048873 GO:0003684 GO:1900034 GO:0000784 GO:0016605 GO:0036297 GO:0000724 GO:0006283 GO:0006297 GO:0070911 GO:0003697 GO:0046872 GO:0008284 GO:0000722 GO:0033683 GO:0006284 GO:0001673 GO:0000082 GO:0000800 GO:0016925 GO:0032201 GO:0000084 GO:0003682 GO:0043687 GO:0070987 GO:0042276 GO:0006298 GO:0007126 GO:0001701 GO:0006271 |
| sp|P27816|MAP4_ HUMAN | GO:0072686 GO:0043005 GO:0005198 GO:1902856 GO:0051294 GO:0044822 GO:0007052 GO:0051012 GO:0051301 GO:0031175 GO:0005874 GO:0008017 GO:0005875 GO:0005930 GO:0070062 GO:0005886 GO:0097481 |
| sp|P27824|CALX_ HUMAN | GO:0061077 GO:0030246 GO:0034975 GO:0043197 GO:0005790 GO:0044233 GO:0005840 GO:0005788 GO:0044822 GO:0009306 GO:0071556 GO:0002474 GO:0007568 GO:0042470 GO:0005509 GO:0043209 GO:0035255 GO:0001948 GO:0019886 GO:0043025 GO:0043234 GO:0030424 GO:0051082 GO:0032839 GO:0070062 GO:0043687 GO:0005791 GO:0034185 GO:0018279 GO:0048488 |
| sp|P28074|PSB5_ HUMAN | GO:0048011 GO:0051436 GO:0002479 GO:0090263 GO:0007411 GO:0048010 GO:0000186 GO:0006977 GO:0005829 GO:0002223 GO:0051437 GO:0005654 GO:0006595 GO:0031145 GO:0038061 GO:0006979 GO:0007265 GO:0090090 GO:0033209 GO:0070062 GO:0016032 GO:0006521 GO:0043488 GO:0005515 GO:0000084 GO:0007173 GO:0038095 GO:0005839 GO:0050852 GO:0004298 GO:0000090 GO:0043066 GO:0008543 GO:0005813 GO:0000209 GO:0008286 |
| sp|P28799|GRN_ HUMAN | GO:0061351 GO:0008083 GO:0004252 GO:0060179 GO:0060999 GO:0035988 GO:0005125 GO:0044822 GO:0006955 GO:0005615 GO:0032355 GO:0016485 GO:0007165 GO:0007566 GO:0050679 GO:0070062 GO:0001835 GO:0005739 GO:0048488 |
| sp|P29353|SHC1_ HUMAN | GO:0048011 GO:0004713 GO:0009636 GO:0005154 GO:0007411 GO:0042542 GO:0007507 GO:0048010 GO:0000186 GO:0031532 GO:0005543 GO:0005829 GO:0005759 GO:0031100 GO:0030168 GO:0070435 GO:0005159 GO:0001666 GO:0045740 GO:0001784 GO:0051721 GO:0050900 GO:0007176 GO:0045087 GO:0007265 GO:0007568 GO:0040008 GO:0016032 GO:0005158 GO:0005068 GO:0051384 GO:0010008 GO:0005168 GO:0036498 GO:0038095 GO:0016337 GO:0001525 GO:0000187 GO:0035094 GO:0048661 GO:0008543 GO:0005634 GO:0045907 GO:0046875 GO:0008286 |
| sp|P29373|RABP2_ HUMAN | GO:0006810 GO:0001972 GO:0005654 GO:0008544 GO:0048672 GO:0035115 GO:0005829 GO:0048385 GO:0006355 GO:0016918 GO:0005215 GO:0030332 GO:0070062 GO:0005783 GO:0042573 GO:0019841 |
| sp|P30044|PRDX5_ HUMAN | GO:0006367 GO:0042744 GO:0070995 GO:0005102 GO:0098869 GO:0034614 GO:0005759 GO:0051354 GO:0043027 GO:0005615 GO:0072541 GO:0046983 GO:0005829 GO:0004601 GO:0016480 GO:0043154 GO:0001016 GO:0031410 GO:0006954 GO:2001057 GO:0048471 GO:0070062 GO:0060785 GO:0005634 GO:0005782 GO:0032967 |
| sp|P30048|PRDX3_ HUMAN | GO:0008385 GO:0032496 GO:0042744 GO:0098869 GO:0042542 GO:0008022 GO:0019901 GO:0034614 GO:0033673 GO:0005759 GO:0043027 GO:0008379 GO:0043209 GO:0018171 GO:0008284 GO:0001893 GO:0043154 GO:0042802 GO:0051881 GO:0030099 GO:0005769 GO:0007005 GO:0070062 GO:0051092 GO:0008785 |
| sp|P30049|ATPD_ HUMAN | GO:0046961 GO:0005759 GO:0046933 GO:0042776 GO:0043531 GO:0006119 GO:0046688 GO:0032403 GO:0000275 GO:0005524 GO:0022904 |
| sp|P30050|RL12_ HUMAN | GO:0005925 GO:0000184 GO:0006415 GO:0006413 GO:0005515 GO:0016020 GO:0044822 GO:0016259 GO:0000027 GO:0022625 GO:0005730 GO:0070180 GO:0003735 GO:0019083 GO:0006614 GO:0070062 GO:0045901 |
| sp|P30084|ECHM_ HUMAN | GO:0005515 GO:0005759 GO:0006635 GO:0070062 GO:0004300 |
| sp|P30711|GSTT1_ HUMAN | GO:0005829 GO:0009751 GO:0098869 GO:0004602 GO:0006304 GO:0070062 GO:0004364 GO:0006749 GO:0042493 GO:0033197 GO:0018900 GO:0010269 GO:0044281 GO:1901687 GO:0005634 GO:0047651 |
| sp|P30838|AL3A1_ HUMAN | GO:0001666 GO:0004030 GO:0018479 GO:0005829 GO:0051384 GO:0016021 GO:0055114 GO:0006805 GO:0007584 GO:0005615 GO:0004028 GO:0005515 GO:0007568 GO:0006081 GO:0042493 GO:0008284 GO:0005654 GO:0044281 GO:0051591 GO:0005783 GO:0005886 GO:0008106 |
| sp|P31689|DNJA1_ HUMAN | GO:0051087 GO:0030544 GO:0042769 GO:0006457 GO:0070585 GO:0043066 GO:0051223 GO:0007283 GO:0030521 GO:0050750 GO:0005829 GO:0030317 GO:0000151 GO:0055131 GO:0046872 GO:0098554 GO:0031625 GO:0043065 GO:0051082 GO:1901998 GO:0009408 GO:0001664 GO:0043508 GO:0031397 GO:0048471 GO:0070062 GO:0005634 GO:0005524 GO:0006986 GO:0005739 |
| sp|P31930|QCR1_ HUMAN | GO:0008270 GO:0014823 GO:0043209 GO:0016485 GO:0004222 GO:0031625 GO:0043279 GO:0009060 GO:1902600 GO:0008121 GO:0032403 GO:0005750 GO:0006122 |
| sp|P31947|1433S_ HUMAN | GO:0000075 GO:0005925 GO:1900740 GO:0044824 GO:0000079 GO:0005829 GO:0005759 GO:0005758 GO:0005615 GO:0030659 GO:0008426 GO:0061436 GO:0009117 GO:0046827 GO:0010482 GO:0010839 GO:0042578 GO:0070062 GO:0000175 GO:0090503 GO:0030307 GO:0019904 GO:0007264 GO:0006367 GO:0031424 GO:0003676 GO:0043154 GO:0019901 GO:0051219 GO:0001836 GO:0008630 GO:0045606 GO:0003334 GO:0005730 GO:0071901 GO:0043765 |
| sp|P32929|CGL_ HUMAN | GO:0018272 GO:0005654 GO:0005887 GO:2001234 GO:0051289 GO:0019343 GO:1904831 GO:0080146 GO:0043123 GO:0005829 GO:0070814 GO:0044540 GO:0030170 GO:0044524 GO:0004123 GO:0001887 GO:0047982 GO:0000098 GO:0070062 GO:0051092 GO:0005516 GO:0030968 GO:0019346 |
| sp|P33121|ACSL1_ HUMAN | GO:0043651 GO:0042178 GO:0033211 GO:0005789 GO:0005741 GO:0019432 GO:0044539 GO:0004467 GO:0014070 GO:0042493 GO:0016021 GO:0035338 GO:0071902 GO:0036109 GO:0005886 GO:0034201 GO:0005524 GO:0007584 GO:0005778 |
| sp|P33552|CKS2_ HUMAN | GO:0044772 GO:0000079 GO:0006355 GO:0051301 GO:0007127 GO:0016301 GO:0008283 GO:0003682 GO:0016538 GO:0009790 |
| sp|P33897|ABCD1_ HUMAN | GO:0043651 GO:0005325 GO:0015910 GO:0005829 GO:0015919 GO:0007031 GO:0042626 GO:0042760 GO:0033540 GO:0055085 GO:0005779 GO:0036109 GO:0048471 GO:0032000 GO:0042803 GO:0019899 GO:0042758 GO:0005524 GO:0005739 |
| sp|P34932|HSP74_ HUMAN | GO:0010628 GO:0032092 GO:0033613 GO:0043066 GO:0001822 GO:0010629 GO:0045766 GO:0051131 GO:0043392 GO:0005829 GO:0001933 GO:0045040 GO:0001085 GO:0009408 GO:0032403 GO:0070062 GO:0005811 GO:0005634 GO:0005524 GO:0006986 |
| sp|P34949|MPI_ HUMAN | GO:0061619 GO:0008270 GO:0009298 GO:0006488 GO:0005829 GO:0070062 GO:0004476 GO:0043687 GO:0061611 GO:0018279 |
| sp|P35221|CTNA1_ HUMAN | GO:0071681 GO:0005925 GO:0007015 GO:0016264 GO:0051149 GO:0017166 GO:2001045 GO:0001541 GO:2001240 GO:0043297 GO:0048010 GO:0031103 GO:0005198 GO:0042475 GO:0030027 GO:0005829 GO:0015629 GO:0008013 GO:0090136 GO:0007163 GO:0016342 GO:0007568 GO:0044822 GO:0001669 GO:0034332 GO:0007264 GO:0005794 GO:2001241 GO:2000146 GO:0007406 GO:0016600 GO:0045880 GO:0045296 GO:0043627 GO:0008584 GO:0051015 GO:0034613 GO:0051291 GO:0014704 GO:0045295 GO:0005915 |
| sp|P35251|RFC1_ HUMAN | GO:0031526 GO:0005663 GO:0042769 GO:0006271 GO:0006284 GO:0016324 GO:0005887 GO:0000722 GO:0006297 GO:0003690 GO:0043085 GO:0015350 GO:0006283 GO:0070911 GO:0003689 GO:0032201 GO:0016323 GO:0005654 GO:0007004 GO:0008518 GO:0015884 GO:0008047 GO:0005737 GO:0070062 GO:0051958 GO:0019904 GO:0000278 GO:0000084 GO:0000122 GO:0005524 GO:0008517 GO:0042276 GO:0005542 GO:0045893 GO:0007565 GO:0043565 GO:0046655 GO:0070987 GO:0005730 GO:0098838 GO:0033683 |
| sp|P35268|RL22_ HUMAN | GO:0005925 GO:0000184 GO:0006415 GO:0006413 GO:0005515 GO:0002181 GO:0044822 GO:0016259 GO:0006414 GO:0022625 GO:0008201 GO:0033077 GO:0003735 GO:0019083 GO:0006614 GO:0070062 GO:0046632 GO:0005634 |
| sp|P35270|SPRE_ HUMAN | GO:0009986 GO:0042713 GO:0032230 GO:0010193 GO:0016496 GO:0042803 GO:0002687 GO:0006558 GO:0070474 GO:0045760 GO:0048660 GO:0005887 GO:0007204 GO:0032224 GO:0006809 GO:0042415 GO:0042417 GO:0005198 GO:0050661 GO:0046887 GO:0046878 GO:0010996 GO:0005829 GO:0040014 GO:0035106 GO:0004033 GO:0005654 GO:0019233 GO:0008306 GO:0018149 GO:0003051 GO:0045471 GO:0050679 GO:0050882 GO:0004757 GO:0019889 GO:0009725 GO:0014910 GO:0060083 GO:0051496 GO:0009408 GO:0070062 GO:0007217 GO:0030216 GO:0005739 GO:0055114 GO:0042755 GO:0045777 GO:0007166 GO:0048667 GO:0002118 GO:0030425 GO:0006729 GO:0016265 GO:0048266 GO:0042428 GO:0050999 GO:0010634 GO:0035094 GO:0045778 GO:0001533 GO:0045907 GO:0043117 GO:0035815 GO:0050671 GO:0007616 GO:0044297 |
| sp|P35527|K1C9_ HUMAN | GO:0045109 GO:0008544 GO:0070062 GO:0005615 GO:0007283 GO:0043588 GO:0045095 GO:0048471 GO:0005200 GO:0016020 GO:0005634 |
| sp|P35580|MYH10_ HUMAN | GO:0007411 GO:0001764 GO:0007097 GO:0043197 GO:0008360 GO:0030027 GO:0021680 GO:0031594 GO:0007155 GO:0030426 GO:0030048 GO:0055015 GO:0030898 GO:0021592 GO:0016460 GO:0097513 GO:0055003 GO:0001778 GO:0008283 GO:0048013 GO:0003279 GO:0043531 GO:0060976 GO:0035904 GO:0006887 GO:0005829 GO:0000146 GO:0005819 GO:0060041 GO:0007264 GO:0043025 GO:0032154 GO:0021670 GO:0030424 GO:0005903 GO:0006930 GO:0030496 GO:0050885 GO:0051015 GO:0001725 GO:0021678 GO:0070062 GO:0000281 GO:0005886 GO:0005516 GO:0005634 GO:0005524 GO:0008152 GO:0001701 GO:0007512 GO:0005938 GO:0005739 |
| sp|P35606|COPB2_ HUMAN | GO:0005080 GO:0030126 GO:0005829 GO:0018279 GO:0030133 GO:0006890 GO:0006891 GO:0043687 GO:0006888 GO:0048205 GO:1901998 GO:0006886 GO:0005198 |
| sp|P35611|ADDA_ HUMAN | GO:0005925 GO:0030507 GO:0005654 GO:0032092 GO:0005198 GO:0043197 GO:0006921 GO:0036498 GO:0048873 GO:0006884 GO:0051017 GO:0044822 GO:0045766 GO:0000902 GO:0044267 GO:0005829 GO:0045111 GO:0046982 GO:0071300 GO:0030218 GO:0008290 GO:0014069 GO:0051015 GO:0006811 GO:0020027 GO:0042608 GO:0045807 GO:0055085 GO:0048471 GO:0042803 GO:0035264 GO:0005886 GO:0005516 GO:0008134 GO:0051016 GO:0001701 |
| sp|P35658|NU214_ HUMAN | GO:0005925 GO:0031047 GO:0005654 GO:0007077 GO:0051028 GO:0005515 GO:1900034 GO:0010827 GO:0006611 GO:0043488 GO:0005829 GO:0019221 GO:0005643 GO:0005975 GO:0005049 GO:0008033 GO:0044281 GO:0019083 GO:0016925 GO:0000088 GO:0055085 GO:0043687 GO:0019054 |
| sp|P35659|DEK_ HUMAN | GO:0019079 GO:0045815 GO:0006357 GO:0042393 GO:0044822 GO:2000779 GO:0007165 GO:0005654 |
| sp|P35900|K1C20_ HUMAN | GO:0045109 GO:0006915 GO:0033554 GO:0050708 GO:0005515 GO:0005200 GO:0005737 GO:0005882 |
| sp|P35908|K22E_ HUMAN | GO:0031069 GO:0045109 GO:0031424 GO:0043616 GO:0070062 GO:0032980 GO:0003334 GO:0005615 GO:0005515 GO:0045095 GO:0005200 GO:0051546 GO:0016020 GO:0005794 GO:0005634 |
| sp|P35914|HMGCL_ HUMAN | GO:0000062 GO:0005777 GO:0042594 GO:0005743 GO:0030145 GO:0001889 GO:0006637 GO:0070542 GO:0007584 GO:0006552 GO:0004419 GO:0005102 GO:0051262 GO:0031406 GO:0007005 GO:0000287 GO:0005759 GO:0042803 GO:0044255 GO:0046951 GO:0009790 |
| sp|P36405|ARL3_ HUMAN | GO:0042461 GO:0000139 GO:0060271 GO:0001822 GO:0005525 GO:0005881 GO:0042073 GO:0046872 GO:0008017 GO:0007264 GO:0005813 GO:0032391 GO:0007224 GO:0030496 GO:0006892 GO:0070062 GO:0005876 GO:0019003 GO:0003924 GO:0005634 GO:0008152 GO:0000910 |
| sp|P36507|MP2K2_ HUMAN | GO:0048011 GO:0035419 GO:0004713 GO:0030878 GO:0005925 GO:0004674 GO:0007346 GO:0032947 GO:0034138 GO:0090170 GO:0007411 GO:0034142 GO:0007507 GO:0048010 GO:0060324 GO:0005783 GO:0004708 GO:0000186 GO:0018108 GO:0050772 GO:0046872 GO:0005829 GO:0060440 GO:0043539 GO:0005778 GO:0070371 GO:2000147 GO:0036289 GO:0097110 GO:0034146 GO:0007265 GO:0002755 GO:0035681 GO:0042981 GO:0032872 GO:0005911 GO:0010629 GO:0048471 GO:0005769 GO:0005794 GO:0005739 GO:0048679 GO:0007173 GO:0005524 GO:0038095 GO:2000641 GO:0005874 GO:0034134 GO:0030165 GO:0009898 GO:0008543 GO:0005634 GO:0005770 GO:0060502 GO:0034154 GO:0005938 GO:0048538 GO:0008286 GO:0035682 GO:0005576 |
| sp|P36551|HEM6_ HUMAN | GO:0016021 GO:0055114 GO:0004109 GO:0005743 GO:0010039 GO:0006782 GO:0005758 GO:0017085 GO:0005212 GO:0010288 GO:0042803 GO:0046685 GO:0044281 GO:0051597 |
| sp|P36578|RL4_ HUMAN | GO:0005925 GO:0000184 GO:0006415 GO:0006413 GO:0005515 GO:0016020 GO:0044822 GO:0016259 GO:0006414 GO:0022625 GO:0005730 GO:0003735 GO:0019083 GO:0006614 GO:0070062 |
| sp|P37108|SRP14_ HUMAN | GO:0006412 GO:0005829 GO:0005786 GO:0030942 GO:0045171 GO:0005730 GO:0008312 GO:0070062 GO:0044822 GO:0005515 GO:0042493 GO:0006614 |
| sp|P37802|TAGL2_ HUMAN | GO:0070062 GO:0030855 GO:0005515 |
| sp|P38117|ETFB_ HUMAN | GO:0006810 GO:0017133 GO:0033539 GO:0070062 GO:0009055 GO:0022904 |
| sp|P38159|RBMX_ HUMAN | GO:0005515 GO:0071347 GO:0001649 GO:0016020 GO:0000381 GO:0006376 GO:0005719 GO:0044530 GO:0005615 GO:0001047 GO:0003727 GO:0006509 GO:0071013 GO:0045944 GO:0070062 GO:0003682 GO:0003729 |
| sp|P38432|COIL_ HUMAN | GO:0005730 GO:0042802 GO:0001674 GO:0015030 GO:0015036 GO:0016020 GO:0008022 GO:0055114 |
| sp|P38606|VATA_ HUMAN | GO:0046961 GO:0033572 GO:0005887 GO:0046034 GO:0008286 GO:0006879 GO:0033180 GO:0015991 GO:0005902 GO:0005765 GO:0043209 GO:0005829 GO:0045454 GO:0070062 GO:0005524 GO:0016324 GO:0005739 |
| sp|P39023|RL3_ HUMAN | GO:0005925 GO:0000184 GO:0006415 GO:0006413 GO:0005654 GO:0005515 GO:0071353 GO:0044822 GO:0008097 GO:0016259 GO:0000027 GO:0006414 GO:0022625 GO:0005730 GO:0003735 GO:0019083 GO:0006614 GO:0070062 |
| sp|P39656|OST48_ HUMAN | GO:0005515 GO:0006412 GO:0042110 GO:0016021 GO:0004579 GO:0034097 GO:0006614 GO:0043687 GO:0005886 GO:0045087 GO:0008250 GO:0018279 |
| sp|P40199|CEAM6_ HUMAN | GO:0001525 GO:0071575 GO:0007156 GO:0007267 GO:0010832 GO:0007229 GO:0070062 GO:0034109 GO:0005615 GO:0050900 GO:2000811 GO:0034235 GO:0042803 GO:0016323 GO:0007596 GO:0031225 |
| sp|P40222|TXLNA_ HUMAN | GO:0005737 GO:0030372 GO:0019905 GO:0008283 GO:0006887 GO:0005125 GO:0098779 GO:0005576 GO:0016020 GO:0042113 |
| sp|P40429|RL13A_ HUMAN | GO:0005925 GO:0003735 GO:0097452 GO:0006414 GO:0032844 GO:1901194 GO:0003729 GO:0016259 GO:0071493 GO:0048246 GO:0071480 GO:0006415 GO:0022625 GO:0006614 GO:1904628 GO:0071320 GO:0060425 GO:0019083 GO:0071346 GO:0000184 GO:0005730 GO:0016020 GO:0032496 |
| sp|P41091|IF2G_ HUMAN | GO:0055085 GO:0003743 GO:0001731 GO:0005829 GO:0070062 GO:0005515 GO:0003924 GO:0005525 GO:0005634 |
| sp|P41223|BUD31_ HUMAN | GO:0007275 GO:0005681 GO:0000790 GO:0035257 GO:0000398 GO:0030374 GO:2000825 GO:0003700 |
| sp|P41227|NAA10_ HUMAN | GO:0034851 GO:0043741 GO:0034915 GO:0004147 GO:1990190 GO:0018031 GO:0005730 GO:0016412 GO:0008951 GO:0018030 GO:0052858 GO:0016749 GO:0031415 GO:0043806 GO:0034738 GO:0016419 GO:0034945 GO:0016753 GO:0034919 GO:0016414 GO:0032216 GO:0043022 GO:0019705 GO:0016750 GO:0034737 GO:0016411 GO:0017198 GO:0016418 GO:0016751 GO:0043849 GO:0005515 GO:0016454 GO:0090595 GO:0006475 GO:0016406 GO:0016413 GO:0046941 GO:0018713 GO:0043764 GO:0018712 GO:0034848 GO:0016416 GO:1990189 GO:0018711 GO:0006323 GO:0022626 GO:0018002 GO:0016020 GO:0019186 |
| sp|P41250|SYG_ HUMAN | GO:0005829 GO:0030141 GO:0030424 GO:0006426 GO:0070062 GO:0015966 GO:0005524 GO:0046983 GO:0005759 GO:0005654 GO:0004820 |
| sp|P41252|SYIC_ HUMAN | GO:0005829 GO:0004822 GO:0002161 GO:0006428 GO:0006450 GO:0070062 GO:0001887 GO:0005524 GO:0001649 GO:0051020 GO:0005654 GO:0016020 |
| sp|P42126|ECI1_ HUMAN | GO:0005743 GO:0070062 GO:0042802 GO:0004165 GO:0006635 GO:0005759 |
| sp|P42166|LAP2A_ HUMAN | GO:0005521 GO:0016021 GO:0006355 GO:0003677 GO:0000785 GO:0005789 GO:0005637 |
| sp|P42224|STAT1_ HUMAN | GO:0004871 GO:0000979 GO:0000790 GO:0042803 GO:0071407 GO:0002053 GO:0016525 GO:0060397 GO:0007584 GO:0042542 GO:0031663 GO:0009612 GO:0005829 GO:0005654 GO:0000978 GO:0060334 GO:0043124 GO:0060338 GO:0051607 GO:0043330 GO:0030424 GO:0043542 GO:0051721 GO:0045944 GO:0001937 GO:0006919 GO:0032869 GO:0046725 GO:0033209 GO:0048471 GO:0000983 GO:0034240 GO:0072308 GO:0031730 GO:0000122 GO:0035458 GO:0051591 GO:0030425 GO:0072136 GO:0008015 GO:0042493 GO:0048661 GO:0072162 GO:0005164 GO:0005730 GO:0003340 |
| sp|P42345|MTOR_ HUMAN | GO:0007173 GO:0038095 GO:0043025 GO:0006281 GO:0019901 GO:0060048 GO:0048714 GO:0045945 GO:0030163 GO:0035264 GO:1904213 GO:0046777 GO:1901216 GO:0031931 GO:0050731 GO:0048255 GO:0010831 GO:0031529 GO:0005741 GO:0005789 GO:0007050 GO:0042220 GO:0016605 GO:0031641 GO:0045087 GO:0018107 GO:1904206 GO:0031998 GO:0007569 GO:0007281 GO:0031397 GO:0051496 GO:1901838 GO:0051219 GO:0071456 GO:0007584 GO:0014042 GO:1903147 GO:1904058 GO:0001156 GO:0043200 GO:1904000 GO:0007616 GO:0003179 GO:0090335 GO:0043022 GO:0030838 GO:0005765 GO:1904193 GO:0048010 GO:0021510 GO:0061051 GO:0001032 GO:0048011 GO:0045429 GO:0001938 GO:0043087 GO:0043610 GO:0031669 GO:0010592 GO:1900034 GO:0035176 GO:0045727 GO:0004674 GO:0007420 GO:0005829 GO:0019904 GO:0001031 GO:0051897 GO:0048015 GO:0060252 GO:0009791 GO:0018105 GO:0008144 GO:0050882 GO:0003007 GO:0006367 GO:0005524 GO:0031932 GO:0006207 GO:1904197 GO:1904056 GO:0060999 GO:0005942 GO:0045670 GO:0001030 GO:0042060 GO:0014736 GO:0001933 GO:0008543 GO:0031295 GO:0030425 GO:0045792 GO:0048661 GO:0000139 GO:0005979 GO:0046889 GO:0032095 GO:0051534 GO:0031929 GO:0060135 GO:0008286 GO:0090559 GO:0008542 GO:0045859 GO:0043278 |
| sp|P42696|RBM34_ HUMAN | GO:0005730 GO:0044822 GO:0000166 |
| sp|P42766|RL35_ HUMAN | GO:0000184 GO:0006415 GO:0006413 GO:0016020 GO:0000463 GO:0016259 GO:0006414 GO:0022625 GO:0005730 GO:0071493 GO:0003735 GO:0019083 GO:0006614 GO:0003729 |
| sp|P43034|LIS1_ HUMAN | GO:0000235 GO:0031512 GO:0021766 GO:0042803 GO:0046329 GO:0000236 GO:0050885 GO:0007067 GO:0000132 GO:0016042 GO:0030426 GO:0090176 GO:0031252 GO:0008201 GO:0061003 GO:2000574 GO:0007268 GO:0007405 GO:0040019 GO:0010977 GO:0005829 GO:0000776 GO:0051081 GO:0019226 GO:0047179 GO:0007611 GO:0021540 GO:0031513 GO:0030424 GO:0021819 GO:0034452 GO:0060117 GO:0045505 GO:0008090 GO:0070062 GO:0048471 GO:0008344 GO:0001675 GO:0016787 GO:0043622 GO:0007264 GO:0046469 GO:0005871 GO:0090102 GO:0045931 GO:0043274 GO:0051219 GO:0047496 GO:0031965 GO:0021895 GO:0036035 GO:0001961 GO:0043087 GO:0007097 GO:0001667 GO:0030036 GO:0000090 GO:0000086 GO:0042249 GO:0048854 GO:0051660 GO:0005813 GO:0043025 GO:0017145 GO:0031023 GO:0005938 GO:0070507 GO:0009306 GO:0008017 GO:0001764 GO:0045773 |
| sp|P43155|CACP_ HUMAN | GO:0006810 GO:0005743 GO:0033540 GO:0004092 GO:0005102 GO:0019254 GO:0005782 GO:0005783 |
| sp|P46087|NOP2_ HUMAN | GO:0005730 GO:0008757 GO:0008284 GO:0005515 GO:0006364 GO:0032259 GO:0044822 |
| sp|P46109|CRKL_ HUMAN | GO:0004871 GO:0007265 GO:0000186 GO:0007507 GO:0044822 GO:0048011 GO:0001568 GO:0005829 GO:0007254 GO:0008284 GO:0048538 GO:0009887 GO:0005768 GO:0009952 GO:0005070 GO:0070062 GO:0060017 |
| sp|P46199|IF2M_ HUMAN | GO:0003743 GO:0032790 GO:0043024 GO:0044822 GO:0070124 GO:0003924 GO:0006446 GO:0005759 GO:0005654 GO:0005525 |
| sp|P46776|RL27A_ HUMAN | GO:0000184 GO:0006415 GO:0006413 GO:0005515 GO:0016020 GO:0044822 GO:0016259 GO:0006414 GO:0022625 GO:0003735 GO:0019083 GO:0006614 |
| sp|P46779|RL28_ HUMAN | GO:0000184 GO:0006415 GO:0006413 GO:0005515 GO:0016020 GO:1903146 GO:0044822 GO:0016259 GO:0044297 GO:0006414 GO:0022625 GO:0030425 GO:1903955 GO:0036464 GO:0003735 GO:0019083 GO:0006614 GO:0070062 |
| sp|P46937|YAP1_ HUMAN | GO:0000979 GO:1902459 GO:0000902 GO:0060487 GO:0008284 GO:0048339 GO:0035019 GO:0090263 GO:0008022 GO:0006975 GO:0010837 GO:0050767 GO:0035329 GO:0007420 GO:0005829 GO:1903507 GO:0060242 GO:0048916 GO:0005654 GO:0048368 GO:0030903 GO:0030054 GO:0003714 GO:0072307 GO:0003713 GO:0070064 GO:0071480 GO:0045944 GO:0030857 GO:0007492 GO:0046622 GO:0071300 GO:0051216 GO:0060449 GO:0001076 GO:0030216 GO:0006367 GO:0005667 GO:0003682 GO:0001570 GO:2000737 GO:0003143 GO:0072091 GO:2001237 GO:0016020 |
| sp|P46939|UTRO_ HUMAN | GO:0005178 GO:0005654 GO:0008270 GO:0031594 GO:0016010 GO:0019901 GO:0030426 GO:2000649 GO:0007517 GO:0006936 GO:0045211 GO:0007528 GO:0042383 GO:0001954 GO:0031527 GO:0051015 GO:0030054 GO:0017166 GO:0070062 GO:0030864 GO:0070938 |
| sp|P46977|STT3A_ HUMAN | GO:0016021 GO:0008250 GO:0018279 GO:0043687 GO:0005515 GO:0004579 GO:0043686 |
| sp|P47813|IF1AX_ HUMAN | GO:0031101 GO:0006413 GO:0005515 GO:0003743 GO:0005829 GO:0043023 GO:0044822 |
| sp|P47985|UCRI_ HUMAN | GO:0008121 GO:0051537 GO:0046677 GO:1902600 GO:0046872 GO:0005750 GO:0043209 GO:0032403 GO:0009725 GO:0022904 GO:0042493 GO:0044281 |
| sp|P48047|ATPO_ HUMAN | GO:0008144 GO:0046933 GO:0042776 GO:0043209 GO:0005496 GO:0032403 GO:0070062 GO:0000276 GO:0005886 GO:0005634 GO:0022904 |
| sp|P48147|PPCE_ HUMAN | GO:0004252 GO:0004222 GO:0005515 GO:0016485 GO:0005759 GO:0042277 GO:0016020 GO:0005634 GO:0008270 GO:0005829 GO:0070008 |
| sp|P48507|GSH0_ HUMAN | GO:0008637 GO:0071333 GO:0097069 GO:0071372 GO:0035729 GO:0051900 GO:0051409 GO:0014823 GO:0007568 GO:0005829 GO:0046982 GO:1901687 GO:0006534 GO:0035229 GO:0006805 GO:0042493 GO:0017109 GO:0006536 GO:2001237 GO:0043524 GO:0044344 GO:0006750 GO:0050880 GO:0006979 GO:0004357 GO:0044752 GO:0035226 GO:0030234 |
| sp|P48634|PRC2A_ HUMAN | GO:0016020 GO:0044822 GO:0070062 GO:0005737 GO:0005515 GO:0043231 |
| sp|P48668|K2C6C_ HUMAN | GO:0042060 GO:0098792 GO:0070062 GO:0098779 GO:0030154 GO:0005515 GO:0045095 GO:0045104 GO:0005200 GO:0007398 GO:0008284 GO:0002009 GO:0002230 GO:0016020 GO:0005634 |
| sp|P49006|MRP_ HUMAN | GO:0008284 GO:0005886 GO:0005515 GO:0070062 |
| sp|P49137|MAPK2_ HUMAN | GO:0048011 GO:0004871 GO:0006954 GO:0034138 GO:0034142 GO:0048010 GO:0034097 GO:0044351 GO:0005829 GO:0034162 GO:0005654 GO:0042535 GO:0035924 GO:0034166 GO:0070935 GO:0032675 GO:0038124 GO:0034146 GO:0009931 GO:0007265 GO:0002755 GO:0006691 GO:0004683 GO:0031572 GO:0070062 GO:0038066 GO:0005524 GO:0048839 GO:0018105 GO:0035666 GO:0019369 GO:0046777 GO:0034134 GO:0005516 GO:1900034 GO:0000187 GO:0038123 GO:0005813 GO:0032496 |
| sp|P49247|RPIA_ HUMAN | GO:0009052 GO:0005975 GO:0048029 GO:0005515 GO:0043231 GO:0004751 GO:0016021 GO:0005829 |
| sp|P49321|NASP_ HUMAN | GO:0033574 GO:0043234 GO:0042393 GO:0007049 GO:0000790 GO:0051879 GO:0006260 GO:0032403 GO:0006336 GO:0008584 GO:0006335 GO:0001824 GO:0043486 GO:0005654 GO:0008283 GO:0005737 GO:0015031 |
| sp|P49411|EFTU_ HUMAN | GO:0003746 GO:0070125 GO:0005743 GO:0045471 GO:0070062 GO:0043209 GO:0042645 GO:0044822 GO:0005515 GO:0003924 GO:0005525 |
| sp|P49419|AL7A1_ HUMAN | GO:0005829 GO:0008802 GO:0055114 GO:0004043 GO:0070062 GO:0005515 GO:0006554 GO:0006081 GO:0005759 GO:0007605 GO:0019285 GO:0005634 |
| sp|P49458|SRP09_ HUMAN | GO:0005047 GO:0005515 GO:0005786 GO:0008312 GO:0016787 GO:0005829 GO:0005785 GO:0006614 GO:0070062 GO:0045900 |
| sp|P49588|SYAC_ HUMAN | GO:0043200 GO:0006457 GO:0005515 GO:0021680 GO:0016020 GO:0004813 GO:0006400 GO:0006450 GO:0016597 GO:0001942 GO:0005829 GO:0046872 GO:0050885 GO:0002161 GO:0006419 GO:0000049 GO:0070062 GO:0043524 GO:0030968 GO:0005524 GO:0005739 |
| sp|P49662|CASP4_ HUMAN | GO:0050718 GO:0042981 GO:0070269 GO:1904646 GO:0005789 GO:0097153 GO:0035234 GO:0072559 GO:0072557 GO:0006508 GO:0070059 GO:0050727 GO:0097169 GO:0005739 |
| sp|P49720|PSB3_ HUMAN | GO:0048011 GO:0051436 GO:0002479 GO:0090263 GO:0007411 GO:0048010 GO:0000186 GO:0006977 GO:0005829 GO:0002223 GO:0015629 GO:0051437 GO:0005654 GO:0006595 GO:0031145 GO:0038061 GO:0007265 GO:0090090 GO:0033209 GO:0070062 GO:0016032 GO:0006521 GO:0005794 GO:0043488 GO:0005739 GO:0005515 GO:0000084 GO:0007173 GO:0038095 GO:0005839 GO:0050852 GO:0004298 GO:0000090 GO:0043066 GO:0008543 GO:0000209 GO:0008286 |
| sp|P49756|RBM25_ HUMAN | GO:0003729 GO:0005681 GO:0016607 GO:0000381 GO:0042981 |
| sp|P49773|HINT1_ HUMAN | GO:0005080 GO:0052770 GO:0000166 GO:0052771 GO:0072332 GO:0050850 GO:0005737 GO:0006355 GO:0005856 GO:0000118 GO:0009154 GO:0070062 GO:0005886 GO:0003923 |
| sp|P49792|RBP2_ HUMAN | GO:0016925 GO:0016874 GO:0000236 GO:0043687 GO:0044614 GO:0033132 GO:0007077 GO:0000413 GO:0005829 GO:1990723 GO:0000088 GO:0044615 GO:0019221 GO:0001975 GO:0019054 GO:0042405 GO:0051028 GO:0090526 GO:0008536 GO:0032403 GO:0007264 GO:0008270 GO:0005739 GO:0003723 GO:0019083 GO:0010827 GO:0031965 GO:1900034 GO:0031047 GO:0003755 GO:0000090 GO:0006457 GO:0008033 GO:0006607 GO:0055085 |
| sp|P49903|SPS1_ HUMAN | GO:0031965 GO:0004756 GO:0006464 GO:0005524 GO:0016310 GO:0046982 GO:0042803 GO:0005737 GO:0005525 GO:0005886 |
| sp|P49914|MTHFS_ HUMAN | GO:0030272 GO:0005542 GO:0046872 GO:0009396 GO:0046657 GO:0035999 GO:0005524 GO:0006536 GO:0005759 GO:0015942 |
| sp|P50135|HNMT_ HUMAN | GO:0007585 GO:0043005 GO:0006548 GO:0005654 GO:0046539 GO:0051384 GO:0006972 GO:0005829 GO:0035902 GO:0007420 GO:1903955 GO:0002347 GO:0001887 GO:0032259 GO:0001505 GO:0070555 GO:0070062 GO:0042220 GO:0014075 |
| sp|P50238|CRIP1_ HUMAN | GO:0042277 GO:0008270 GO:0010468 GO:0071236 GO:0060741 GO:0008301 GO:0003680 GO:0007507 GO:0071493 GO:0006955 GO:0008630 GO:0010043 GO:0008283 GO:0005737 GO:0010033 |
| sp|P50402|EMD_ HUMAN | GO:0071363 GO:0007077 GO:0060828 GO:0005637 GO:0005652 GO:0035414 GO:0007084 GO:0005640 GO:0006936 GO:0046827 GO:0005874 GO:0003779 GO:0005819 GO:0035914 GO:0016021 GO:0000090 GO:0000088 GO:0005783 GO:0048147 GO:0048487 |
| sp|P50570|DYN2_ HUMAN | GO:0043065 GO:0045334 GO:0005525 GO:0005925 GO:0060271 GO:0017124 GO:0050998 GO:0005802 GO:0030512 GO:0030426 GO:0045429 GO:1903526 GO:0014069 GO:0030496 GO:0048489 GO:0043234 GO:0030027 GO:0044351 GO:0001917 GO:0005829 GO:1903358 GO:0050699 GO:0060976 GO:0007283 GO:0031749 GO:0071245 GO:0009416 GO:0001891 GO:1903408 GO:0006893 GO:0000139 GO:0019886 GO:0044281 GO:0035904 GO:0033572 GO:0042220 GO:0003281 GO:0070062 GO:0048471 GO:0071732 GO:1903351 GO:0002031 GO:0030516 GO:0071481 GO:0035020 GO:0045211 GO:0019901 GO:0045893 GO:0050766 GO:0005874 GO:0030666 GO:0003924 GO:0036312 GO:0010592 GO:1900026 GO:0050999 GO:0000086 GO:0005634 GO:0005813 GO:0005905 GO:0032587 GO:0008017 GO:0005768 |
| sp|P50851|LRBA_ HUMAN | GO:0008104 GO:0051018 GO:0016197 GO:0005886 GO:0005764 GO:0007165 GO:0016021 GO:0016023 GO:0005783 GO:0005802 |
| sp|P50897|PPT1_ HUMAN | GO:0045121 GO:0044257 GO:0048666 GO:0008474 GO:0051181 GO:0007269 GO:0007625 GO:0005615 GO:0048549 GO:0015031 GO:0008306 GO:0005794 GO:0050896 GO:0050803 GO:0005829 GO:0019432 GO:0030425 GO:0007042 GO:0007420 GO:0043025 GO:0007601 GO:0048260 GO:0030308 GO:0002084 GO:0030424 GO:0044281 GO:0008021 GO:0035338 GO:0032429 GO:0070062 GO:0043524 GO:0043202 GO:0031579 GO:0005634 GO:0016290 GO:0030149 GO:0008344 |
| sp|P50914|RL14_ HUMAN | GO:0000184 GO:0006415 GO:0006413 GO:0005515 GO:0016020 GO:0044822 GO:0016259 GO:0006414 GO:0042273 GO:0022625 GO:0003735 GO:0019083 GO:0006614 GO:0070062 GO:0006364 |
| sp|P50991|TCPD_ HUMAN | GO:0005654 GO:0005929 GO:1904851 GO:0044822 GO:0032212 GO:0044297 GO:0042470 GO:0050821 GO:0044267 GO:0005832 GO:0005874 GO:0051973 GO:1904874 GO:0007339 GO:0005813 GO:1904871 GO:0051082 GO:0051085 GO:1901998 GO:0090666 GO:0070062 GO:0005524 GO:0002199 |
| sp|P51116|FXR2_ HUMAN | GO:0003729 GO:0017148 GO:0030425 GO:0098792 GO:0070062 GO:0042802 GO:0005844 GO:0002230 GO:0022625 GO:0016020 GO:0005634 |
| sp|P51398|RT29_ HUMAN | GO:0008637 GO:0005654 GO:0043197 GO:0030165 GO:0006461 GO:0044822 GO:0005743 GO:0005730 GO:0001540 GO:0045211 GO:0097190 GO:0043025 GO:0070126 GO:0070124 GO:0005763 GO:0014069 GO:0003735 GO:0030054 GO:0097110 GO:0070125 GO:0017146 GO:0032947 |
| sp|P51553|IDH3G_ HUMAN | GO:0051287 GO:0005962 GO:0045926 GO:0006103 GO:0005730 GO:0006734 GO:0005975 GO:0004449 GO:0006099 GO:0005524 GO:0000287 GO:0006102 GO:0005654 |
| sp|P51571|SSRD_ HUMAN | GO:0006412 GO:0005784 GO:0016021 GO:0070062 GO:0006614 |
| sp|P51610|HCFC1_ HUMAN | GO:0019046 GO:0000122 GO:0010628 GO:0043995 GO:0016020 GO:0048188 GO:0046972 GO:0050821 GO:0043996 GO:0070461 GO:0005671 GO:0030425 GO:0043025 GO:0003713 GO:0042802 GO:0030424 GO:0007005 GO:0070688 GO:0043254 GO:0003682 GO:0043984 GO:0001205 GO:0045787 GO:0043982 GO:0071339 GO:0005739 GO:0043981 |
| sp|P51648|AL3A2_ HUMAN | GO:0052814 GO:0004030 GO:0005829 GO:0007417 GO:0046577 GO:0050061 GO:0016021 GO:0055114 GO:0005777 GO:0000302 GO:0005743 GO:0042406 GO:0046292 GO:0008544 GO:0070062 GO:0004028 GO:0006714 GO:0030148 GO:0007422 GO:0033306 GO:0005634 |
| sp|P51812|KS6A3_ HUMAN | GO:0048011 GO:0004674 GO:0043555 GO:0034138 GO:0007411 GO:0001501 GO:0034142 GO:0007268 GO:0005829 GO:0034162 GO:0005654 GO:0000287 GO:0034166 GO:0043027 GO:0045597 GO:0045944 GO:0038124 GO:0034146 GO:0002755 GO:0051403 GO:0030307 GO:0005524 GO:0043154 GO:0035666 GO:0007049 GO:0019901 GO:0007417 GO:0034134 GO:0038123 GO:0043620 GO:0032496 |
| sp|P52209|6PGD_ HUMAN | GO:0006564 GO:0030246 GO:0006541 GO:0051287 GO:0008114 GO:0050661 GO:0010468 GO:0009448 GO:0031406 GO:0031175 GO:0043209 GO:0019322 GO:0005829 GO:0019530 GO:0009051 GO:0006544 GO:0021510 GO:0070314 GO:0055114 GO:0070062 GO:0004617 GO:0006566 GO:0005634 GO:0004616 GO:0021915 GO:0021782 GO:0019521 |
| sp|P52272|HNRPM_ HUMAN | GO:1904591 GO:0005887 GO:0000166 GO:0044822 GO:0048306 GO:1990405 GO:0005730 GO:0009986 GO:0071013 GO:0019904 GO:1990831 GO:0016363 GO:0042382 GO:0000380 GO:0070062 GO:0071222 GO:1900182 GO:0031012 |
| sp|P52298|NCBP2_ HUMAN | GO:0031047 GO:0000184 GO:0005654 GO:0006370 GO:0005515 GO:0000166 GO:0006406 GO:0031442 GO:0008334 GO:0046833 GO:0006408 GO:0098789 GO:0000340 GO:0017069 GO:0005829 GO:0005845 GO:0045292 GO:0034660 GO:0006369 GO:0006446 GO:0005846 GO:0050434 GO:1900363 GO:0006368 GO:0003729 GO:0000387 |
| sp|P52306|GDS1_ HUMAN | GO:0005829 GO:0005739 GO:0007264 GO:0070062 GO:0051561 GO:0032471 GO:0005515 GO:0043547 GO:0005096 GO:0005783 |
| sp|P52701|MSH6_ HUMAN | GO:0032357 GO:0045910 GO:0008340 GO:0005654 GO:0032405 GO:0045830 GO:0000400 GO:0000710 GO:0008630 GO:0043531 GO:0009411 GO:0032142 GO:0005794 GO:0032143 GO:0016446 GO:0000287 GO:0051096 GO:0000790 GO:0016887 GO:0016032 GO:0042803 GO:0003682 GO:0032137 GO:0005886 GO:0005524 GO:0035064 GO:0032301 GO:0043570 |
| sp|P52735|VAV2_ HUMAN | GO:0007411 GO:0001525 GO:0048010 GO:0005154 GO:0030032 GO:0030168 GO:0035023 GO:0038095 GO:0048011 GO:0030193 GO:0010468 GO:0048013 GO:0005829 GO:0046872 GO:0097190 GO:0008361 GO:0043065 GO:0043552 GO:0038096 GO:0043547 GO:0005886 GO:0005089 GO:0045087 GO:0016477 |
| sp|P52788|SPSY_ HUMAN | GO:0006597 GO:0016768 GO:0005829 GO:0070062 GO:0006555 |
| sp|P52815|RM12_ HUMAN | GO:0045893 GO:0005515 GO:0005762 GO:0005743 GO:0070126 GO:0006390 GO:0070124 GO:0003735 GO:0003723 GO:0070125 |
| sp|P52948|NUP98_ HUMAN | GO:0017056 GO:0031080 GO:0042405 GO:0031965 GO:0051292 GO:0006260 GO:0044615 GO:0008139 GO:0000776 GO:0034399 GO:0005515 GO:0005654 GO:0000059 GO:0051028 |
| sp|P53602|MVD1_ HUMAN | GO:0005829 GO:0018279 GO:0019287 GO:0004163 GO:0006489 GO:0043687 GO:0005524 GO:0042493 GO:0008284 GO:0042803 GO:0005782 GO:0006695 GO:0030544 |
| sp|P53618|COPB_ HUMAN | GO:0030126 GO:0005829 GO:0018279 GO:0030133 GO:0006890 GO:0005793 GO:0006891 GO:0043687 GO:0005515 GO:0006888 GO:0048205 GO:0016032 GO:0006886 GO:0005198 GO:0005783 GO:0005886 |
| sp|P53621|COPA_ HUMAN | GO:0005198 GO:0030133 GO:0006891 GO:0005615 GO:0006886 GO:0005829 GO:0005179 GO:0006888 GO:0030126 GO:0006890 GO:0070062 GO:0043687 GO:0030157 GO:0018279 GO:0048205 |
| sp|P53634|CATC_ HUMAN | GO:0004197 GO:0051087 GO:0000139 GO:0031638 GO:0004252 GO:0031404 GO:0001913 GO:0005788 GO:0010033 GO:0043621 GO:0007568 GO:0005615 GO:0005764 GO:1903052 GO:0030134 GO:2001235 GO:0016505 GO:0042802 GO:0019902 GO:0033116 GO:0048208 GO:0070062 GO:0043687 GO:0018279 |
| sp|P54105|ICLN_ HUMAN | GO:0034709 GO:0005829 GO:0005856 GO:0034715 GO:0000387 GO:0044822 GO:0006821 GO:0034660 GO:0046982 GO:0005654 GO:0006884 GO:0005886 |
| sp|P54136|SYRC_ HUMAN | GO:0006420 GO:0005829 GO:0017101 GO:0005739 GO:0070062 GO:0001887 GO:0005524 GO:0034618 GO:0005515 GO:0004814 GO:0000049 GO:0005654 GO:0016020 |
| sp|P54727|RD23B_ HUMAN | GO:0070911 GO:0031593 GO:0007283 GO:0048568 GO:0003697 GO:0005654 GO:0005737 GO:0032434 GO:0071942 GO:0000715 |
| sp|P54886|P5CS_ HUMAN | GO:0019240 GO:0055114 GO:0005743 GO:0034641 GO:0005524 GO:0004350 GO:0044822 GO:0055129 GO:0005515 GO:0006536 GO:0016310 GO:0006592 GO:0004349 |
| sp|P55036|PSMD4_ HUMAN | GO:0048011 GO:0051436 GO:0031593 GO:0008540 GO:0002479 GO:0042044 GO:0090263 GO:0007411 GO:0048010 GO:0000186 GO:0006977 GO:0005829 GO:0002223 GO:0051437 GO:0005654 GO:0008134 GO:0006595 GO:0043248 GO:0031145 GO:0038061 GO:0007265 GO:0090090 GO:0033209 GO:0044822 GO:0016032 GO:0006521 GO:0042802 GO:0043488 GO:0000084 GO:0007173 GO:0038095 GO:0050852 GO:0000090 GO:0043066 GO:0008543 GO:0000209 GO:0008286 |
| sp|P55039|DRG2_ HUMAN | GO:0005654 GO:0005525 GO:0005515 GO:0007165 GO:0016020 GO:0005739 |
| sp|P55060|XPO2_ HUMAN | GO:0005829 GO:0008536 GO:0006915 GO:0006606 GO:0006611 GO:0070062 GO:0005049 GO:0005635 GO:0005654 GO:0008283 GO:0016020 |
| sp|P55081|MFAP1_ HUMAN | GO:0044822 GO:0001527 GO:0005515 GO:0030198 |
| sp|P55084|ECHB_ HUMAN | GO:0016507 GO:0051287 GO:0016508 GO:0000062 GO:0003988 GO:0005743 GO:0046474 GO:0035965 GO:0070062 GO:0032403 GO:0042645 GO:0005741 GO:0044822 GO:0006635 GO:0016509 GO:0004300 GO:0005783 |
| sp|P55210|CASP7_ HUMAN | GO:0009411 GO:0051402 GO:0097200 GO:0005829 GO:0097193 GO:0006921 GO:0008635 GO:0007507 GO:0005515 GO:0007568 GO:0004190 GO:0005654 |
| sp|P55789|ALR_ HUMAN | GO:0015035 GO:0097421 GO:0050660 GO:0008083 GO:0005758 GO:0043066 GO:0045953 GO:0071356 GO:0005615 GO:0044267 GO:0016971 GO:0072717 GO:0005829 GO:1903204 GO:0055114 GO:0071222 GO:0006626 GO:2000573 |
| sp|P55795|HNRH2_ HUMAN | GO:0019013 GO:0043484 GO:0008266 GO:0000398 GO:0019899 GO:0044822 GO:0000166 GO:0071013 GO:0005654 GO:0005737 GO:1901653 GO:0016020 |
| sp|P56192|SYMC_ HUMAN | GO:0009303 GO:0005829 GO:0005739 GO:0005730 GO:0070062 GO:0004825 GO:0001887 GO:0005524 GO:0000049 GO:0016020 GO:0006431 |
| sp|P56381|ATP5E_ HUMAN | GO:0046961 GO:0008553 GO:0005759 GO:0046933 GO:0042776 GO:0000275 GO:0022904 |
| sp|P56385|ATP5I_ HUMAN | GO:0042776 GO:0022857 GO:0016887 GO:0000276 GO:0022904 |
| sp|P56537|IF6_ HUMAN | GO:0003743 GO:0006413 GO:0000054 GO:0005730 GO:0042273 GO:0005638 GO:0070062 GO:0043023 GO:0043022 GO:0005852 GO:0005515 GO:0005654 GO:0042256 |
| sp|P56589|PEX3_ HUMAN | GO:0002230 GO:0005654 GO:0005887 GO:0032994 GO:0045046 GO:0016557 GO:0046983 GO:0005829 GO:0098792 GO:0003333 GO:0043234 GO:0015171 GO:0008289 GO:0005779 GO:0005783 GO:0098779 |
| sp|P57740|NU107_ HUMAN | GO:0051292 GO:0031047 GO:0005654 GO:0007077 GO:0005515 GO:1900034 GO:0034399 GO:0006406 GO:0000236 GO:0000973 GO:0031965 GO:0015288 GO:0010827 GO:0046930 GO:0000777 GO:0005829 GO:0006355 GO:0005487 GO:0019221 GO:0005975 GO:0007264 GO:0005813 GO:0008033 GO:0044281 GO:0000090 GO:0019083 GO:0016925 GO:0000088 GO:0055085 GO:0017056 GO:0031080 GO:0043687 GO:0019054 GO:0006606 |
| sp|P57772|SELB_ HUMAN | GO:0003746 GO:0005739 GO:0035368 GO:0001514 GO:0043021 GO:0030529 GO:0005515 GO:0003924 GO:0000049 GO:0016259 GO:0005634 GO:0005525 |
| sp|P58107|EPIPL_ HUMAN | GO:0044822 GO:0005737 GO:0030496 |
| sp|P59998|ARPC4_ HUMAN | GO:0007411 GO:0048013 GO:0034314 GO:0005829 GO:0030674 GO:0007264 GO:0005885 GO:0038096 GO:0051015 GO:0042995 GO:0070062 GO:0019899 GO:0045087 GO:0005200 |
| sp|P60174|TPIS_ HUMAN | GO:0006098 GO:0006094 GO:0004807 GO:0005615 GO:0005829 GO:0031625 GO:0009790 GO:0019563 GO:0046166 GO:0070062 GO:0061621 GO:0005634 |
| sp|P60468|SC61B_ HUMAN | GO:0048408 GO:0036498 GO:0006412 GO:0044822 GO:0031205 GO:0043022 GO:0005829 GO:0002479 GO:0071806 GO:0000060 GO:0015450 GO:0016021 GO:0030970 GO:0006614 GO:0030433 |
| sp|P60866|RS20_ HUMAN | GO:0000184 GO:0006415 GO:0006413 GO:0005515 GO:0016020 GO:0044822 GO:0016259 GO:0006414 GO:0003735 GO:0019083 GO:0006614 GO:0070062 GO:0022627 |
| sp|P60983|GMFB_ HUMAN | GO:0004871 GO:0007626 GO:0043085 GO:0008083 GO:0007612 GO:0008047 GO:1904030 GO:0003779 GO:0005622 GO:0004860 GO:0034316 GO:0007165 GO:0071933 GO:0007399 |
| sp|P61011|SRP54_ HUMAN | GO:0005515 GO:0008144 GO:0043021 GO:0016607 GO:0005786 GO:0006412 GO:0044822 GO:0008312 GO:0005525 GO:0005730 GO:0005829 GO:0042493 GO:0030942 GO:0019003 GO:0003924 GO:0006617 GO:0006616 |
| sp|P61026|RAB10_ HUMAN | GO:0005925 GO:0030670 GO:0055038 GO:0005802 GO:0019882 GO:0000139 GO:0032869 GO:0045200 GO:0007409 GO:0005525 GO:0071786 GO:0005789 GO:0032593 GO:0016197 GO:0072372 GO:0071236 GO:0000145 GO:0007264 GO:0030859 GO:0097051 GO:0048471 GO:0070062 GO:0043001 GO:0019003 GO:0003924 GO:0005886 GO:0031489 GO:0071782 GO:0061467 |
| sp|P61086|UBE2K_ HUMAN | GO:0070936 GO:0034450 GO:0010994 GO:0060340 GO:0015629 GO:0016874 GO:0005737 GO:0045111 GO:0035458 GO:0032434 GO:0061631 GO:0031625 GO:0032433 GO:0070062 GO:0070059 GO:0010800 GO:0005634 GO:0005524 |
| sp|P61160|ARP2_ HUMAN | GO:0005925 GO:0030478 GO:0004675 GO:0016021 GO:0007178 GO:0051653 GO:0007411 GO:0042384 GO:0046872 GO:0005829 GO:0005200 GO:0016482 GO:0038096 GO:0007163 GO:0048013 GO:0042995 GO:0045087 GO:0070062 GO:0016344 GO:0004702 GO:0051216 GO:0007264 GO:0034314 GO:0005524 GO:0051015 GO:0030902 GO:0008356 GO:0005885 GO:0001755 GO:0006468 GO:0033206 |
| sp|P61163|ACTZ_ HUMAN | GO:0016192 GO:0005829 GO:0006996 GO:1990752 GO:0000086 GO:0070062 GO:0043209 GO:0005813 GO:0005524 GO:0019886 GO:0005869 GO:0016020 |
| sp|P61221|ABCE1_ HUMAN | GO:0055085 GO:0009615 GO:0006401 GO:0005524 GO:0008428 GO:0043086 GO:0016887 GO:0005515 GO:0005759 GO:0016032 GO:0016020 |
| sp|P61244|MAX_ HUMAN | GO:0030097 GO:0070206 GO:0006357 GO:0016605 GO:0048264 GO:0010629 GO:0032868 GO:0000978 GO:0005737 GO:0051402 GO:0046982 GO:0030425 GO:0060041 GO:0003713 GO:0048678 GO:0032403 GO:0042803 GO:0000983 GO:0071375 GO:0009267 GO:0071141 GO:0071339 |
| sp|P61254|RL26_ HUMAN | GO:0000184 GO:0006415 GO:0006413 GO:0005515 GO:0016020 GO:0002181 GO:0044822 GO:0016259 GO:0006414 GO:1990928 GO:0042273 GO:0022625 GO:0003735 GO:0019083 GO:0043195 GO:0006614 GO:0070062 GO:0006364 |
| sp|P61326|MGN_ HUMAN | GO:0000184 GO:0005515 GO:0006406 GO:0016607 GO:0000381 GO:0044822 GO:0035145 GO:0031124 GO:0005829 GO:0071013 GO:0043025 GO:0006369 GO:0006417 GO:0007292 |
| sp|P61513|RL37A_ HUMAN | GO:0005925 GO:0000184 GO:0006415 GO:0006413 GO:0005515 GO:0044822 GO:0016259 GO:0006414 GO:0022625 GO:0046872 GO:0003735 GO:0019083 GO:0006614 GO:0070062 GO:0005634 |
| sp|P61604|CH10_ HUMAN | GO:0006457 GO:0051087 GO:0070062 GO:0005524 GO:0044822 GO:0006919 GO:0001649 GO:0005759 GO:0051082 GO:0006986 GO:0016020 |
| sp|P61769|B2MG_ HUMAN | GO:0019731 GO:0005925 GO:0042026 GO:0050830 GO:0002726 GO:0002479 GO:0071281 GO:1900121 GO:0001895 GO:0005788 GO:0005615 GO:0033077 GO:0060333 GO:0030670 GO:0050690 GO:0012507 GO:0001916 GO:0000139 GO:0002480 GO:0071222 GO:0031905 GO:0070062 GO:0016032 GO:0002481 GO:0042802 GO:1904437 GO:0009897 GO:0048260 GO:0003254 GO:0055072 GO:0001948 GO:1990712 GO:0042612 GO:0044267 GO:1903991 GO:0031901 GO:0046686 GO:0042493 GO:0050829 GO:1904434 GO:0019885 |
| sp|P61960|UFM1_ HUMAN | GO:0005737 GO:0033146 GO:0034976 GO:1990592 GO:0005634 GO:0070062 |
| sp|P61962|DCAF7_ HUMAN | GO:0005737 GO:0005654 GO:0005515 GO:0080008 GO:0016567 GO:0016363 GO:0051216 |
| sp|P61964|WDR5_ HUMAN | GO:0001501 GO:0043995 GO:0048188 GO:0046972 GO:0043996 GO:0031175 GO:0005671 GO:0035948 GO:0009790 GO:0051568 GO:0043966 GO:0042800 GO:0043984 GO:0035064 GO:0043982 GO:0071339 GO:0043981 |
| sp|P61981|1433G_ HUMAN | GO:0000075 GO:0005080 GO:0005925 GO:1900740 GO:0048167 GO:0007507 GO:0007420 GO:0030971 GO:0005829 GO:0097193 GO:0030659 GO:0005159 GO:0008426 GO:0045664 GO:0032869 GO:0006605 GO:0043209 GO:0044822 GO:0070062 GO:0019904 GO:0007264 GO:0005739 GO:0006367 GO:0055114 GO:0000086 GO:0004497 GO:0071901 GO:0003779 |
| sp|P62081|RS7_ HUMAN | GO:0005925 GO:0030097 GO:0000184 GO:0006415 GO:0006413 GO:0016020 GO:0005815 GO:0044822 GO:0016259 GO:0006414 GO:0005730 GO:0046982 GO:0051726 GO:0001843 GO:0030154 GO:0003735 GO:0019083 GO:0042274 GO:0006614 GO:0032040 GO:0070062 GO:0005783 GO:0030686 GO:0006364 GO:0022627 |
| sp|P62249|RS16_ HUMAN | GO:0005925 GO:0000184 GO:0006415 GO:0006413 GO:0005515 GO:0016020 GO:0044822 GO:0016259 GO:0006414 GO:0016787 GO:0000462 GO:0003735 GO:0019083 GO:0006614 GO:0070062 GO:0022627 |
| sp|P62263|RS14_ HUMAN | GO:0005925 GO:0000184 GO:0048027 GO:0006413 GO:0006415 GO:0000122 GO:0005515 GO:0000028 GO:0016259 GO:0048821 GO:0006414 GO:0016787 GO:0005730 GO:0045182 GO:0043009 GO:0070181 GO:0000462 GO:0006417 GO:0016021 GO:0003735 GO:0019083 GO:0006614 GO:0070062 GO:0005739 GO:0022627 |
| sp|P62269|RS18_ HUMAN | GO:0005925 GO:0000184 GO:0006415 GO:0006413 GO:0005515 GO:0016020 GO:0007275 GO:0007218 GO:0044822 GO:0005581 GO:0016259 GO:0006414 GO:0004966 GO:0051726 GO:0006417 GO:0003735 GO:0019083 GO:0006614 GO:0070062 GO:0005634 GO:0019843 GO:0022627 |
| sp|P62273|RS29_ HUMAN | GO:0005925 GO:0001525 GO:0000184 GO:0006415 GO:0006413 GO:0008270 GO:0072332 GO:0060218 GO:0016259 GO:0048821 GO:0006414 GO:0016787 GO:0043009 GO:0051726 GO:0003735 GO:0019083 GO:0006614 GO:0070062 GO:0001570 GO:0022627 |
| sp|P62277|RS13_ HUMAN | GO:0005925 GO:0000184 GO:0006413 GO:0006415 GO:0005515 GO:0016020 GO:0016259 GO:0006414 GO:0016787 GO:0005730 GO:0033119 GO:0070181 GO:0003735 GO:0019083 GO:0006614 GO:0070062 GO:0003729 GO:0022627 |
| sp|P62280|RS11_ HUMAN | GO:0005925 GO:0000184 GO:0006415 GO:0006413 GO:0005515 GO:0001649 GO:0016020 GO:0044822 GO:0016259 GO:0006414 GO:0005730 GO:0003735 GO:0019083 GO:0006614 GO:0070062 GO:0019843 GO:0022627 |
| sp|P62304|RUXE_ HUMAN | GO:0005687 GO:0034709 GO:0019013 GO:0005829 GO:0003723 GO:0006369 GO:1990446 GO:0071011 GO:0005686 GO:0005683 GO:0000245 GO:0070062 GO:0005697 GO:0042633 GO:0031124 GO:0005685 GO:0034715 GO:0000387 GO:0005515 GO:0008334 GO:0034660 GO:0005682 GO:0046540 GO:0071013 GO:0034719 GO:0005654 GO:0005689 |
| sp|P62306|RUXF_ HUMAN | GO:0005687 GO:0034709 GO:0019013 GO:0005829 GO:0003723 GO:0006369 GO:0005732 GO:0005683 GO:0031124 GO:0005685 GO:0034715 GO:0098779 GO:0000387 GO:0008334 GO:0005515 GO:0034660 GO:0071013 GO:0034719 GO:0005654 GO:0005689 |
| sp|P62308|RUXG_ HUMAN | GO:0005687 GO:0034709 GO:0019013 GO:0005829 GO:0006369 GO:1990446 GO:0071011 GO:0005686 GO:0005732 GO:0005683 GO:0000245 GO:0031124 GO:0097526 GO:0005685 GO:0000387 GO:0044822 GO:0005515 GO:0008334 GO:0034660 GO:0043186 GO:0071004 GO:0005682 GO:0071013 GO:0034719 GO:0005654 GO:0005689 |
| sp|P62310|LSM3_ HUMAN | GO:0005829 GO:0071011 GO:0030629 GO:0000932 GO:0000398 GO:0033962 GO:0005688 GO:0044822 GO:0005515 GO:0046540 GO:0071013 GO:0005654 GO:1990726 GO:0043928 |
| sp|P62314|SMD1_ HUMAN | GO:0002230 GO:0005654 GO:0034715 GO:0005515 GO:0005689 GO:0046540 GO:0007507 GO:0044822 GO:0071011 GO:0034719 GO:0005829 GO:0071013 GO:0071010 GO:0098792 GO:0000243 GO:0000245 GO:0034660 GO:0034709 GO:0005682 GO:0005686 GO:0070062 GO:0019013 GO:0005687 GO:0098779 GO:1990446 GO:0005685 GO:0000387 |
| sp|P62316|SMD2_ HUMAN | GO:0005687 GO:0034709 GO:0019013 GO:0005829 GO:1990446 GO:0071011 GO:0005686 GO:0000245 GO:0070062 GO:0005685 GO:0034715 GO:0000387 GO:0044822 GO:0005515 GO:0034660 GO:0005682 GO:0046540 GO:0071013 GO:0034719 GO:0005654 GO:0005689 |
| sp|P62318|SMD3_ HUMAN | GO:0005687 GO:0034709 GO:0019013 GO:0005829 GO:0006369 GO:0071208 GO:0071010 GO:1990446 GO:0071209 GO:0071011 GO:0005686 GO:0005683 GO:0000245 GO:0070062 GO:0006479 GO:0005697 GO:0031124 GO:0097526 GO:0005685 GO:0019899 GO:0034715 GO:0000387 GO:0044822 GO:0008334 GO:0034660 GO:0030620 GO:0070034 GO:0005682 GO:0000243 GO:0071013 GO:0034719 GO:0005654 GO:0005689 |
| sp|P62424|RL7A_ HUMAN | GO:0005925 GO:0000184 GO:0006415 GO:0006413 GO:0016020 GO:0002181 GO:0000463 GO:0016259 GO:0006414 GO:0022625 GO:0005730 GO:0003677 GO:0042788 GO:0003735 GO:0019083 GO:0006614 GO:0070062 GO:0042803 GO:0003729 |
| sp|P62495|ERF1_ HUMAN | GO:0005829 GO:0016149 GO:0007420 GO:0006479 GO:0043022 GO:0044822 GO:0006449 GO:0005515 GO:0016032 GO:0000184 GO:0005634 |
| sp|P62750|RL23A_ HUMAN | GO:0000184 GO:0006415 GO:0006413 GO:0005515 GO:0000166 GO:0044822 GO:0031932 GO:0016259 GO:0000027 GO:0008283 GO:0006414 GO:0022625 GO:0005730 GO:0070180 GO:0003735 GO:0019083 GO:0006614 GO:0070062 |
| sp|P62851|RS25_ HUMAN | GO:0000184 GO:0006415 GO:0006413 GO:0005515 GO:0000028 GO:0044822 GO:0016259 GO:0006414 GO:0005730 GO:0003735 GO:0019083 GO:0006614 GO:0070062 GO:0022627 |
| sp|P62875|RPAB5_ HUMAN | GO:0031047 GO:0006385 GO:0006367 GO:0006370 GO:0006362 GO:0032481 GO:0005515 GO:0034587 GO:0008270 GO:0045815 GO:0001055 GO:0005666 GO:0001056 GO:0006283 GO:0006363 GO:0006386 GO:0005829 GO:0005665 GO:0003677 GO:0009790 GO:0045814 GO:0035019 GO:0050434 GO:0000398 GO:0006368 GO:0006361 GO:0045087 GO:0006356 GO:0005736 GO:0001054 |
| sp|P62906|RL10A_ HUMAN | GO:0005925 GO:0000184 GO:0006415 GO:0006413 GO:0005515 GO:0016020 GO:0045471 GO:0044822 GO:0016259 GO:0006414 GO:0022625 GO:0005730 GO:0003735 GO:0019083 GO:0006614 GO:0070062 GO:0005739 |
| sp|P62917|RL8_ HUMAN | GO:0005925 GO:0000184 GO:0006415 GO:0006413 GO:0014028 GO:0005515 GO:0002181 GO:0044822 GO:0008097 GO:0016259 GO:0006414 GO:0022625 GO:0005730 GO:0060034 GO:0003735 GO:0019083 GO:0006614 GO:0005886 |
| sp|P63096|GNAI1_ HUMAN | GO:0031821 GO:0043949 GO:0045121 GO:0004871 GO:0030168 GO:0007193 GO:1904322 GO:0032794 GO:0005834 GO:0043434 GO:0005525 GO:0051301 GO:0005765 GO:0031683 GO:0005813 GO:0000287 GO:0030496 GO:0070062 GO:0019003 GO:0003924 GO:0050805 GO:0005634 GO:0008152 GO:0007049 |
| sp|P67809|YBOX1_ HUMAN | GO:0002039 GO:0005654 GO:0000122 GO:0005689 GO:0043066 GO:0051020 GO:0001077 GO:0070934 GO:1990124 GO:0031965 GO:0000978 GO:0003697 GO:0071204 GO:0010494 GO:0051154 GO:0030425 GO:0008284 GO:0046627 GO:0045944 GO:0048471 GO:0070062 GO:0003682 GO:0000398 GO:0070937 GO:0008134 GO:0001701 GO:0003729 GO:1903608 |
| sp|P68133|ACTS_ HUMAN | GO:0005925 GO:0003272 GO:0072562 GO:0048741 GO:0010628 GO:0030027 GO:0016020 GO:0043066 GO:0045471 GO:0048545 GO:0009612 GO:0060048 GO:0043503 GO:0055003 GO:0030049 GO:0030175 GO:0048306 GO:0044297 GO:0090131 GO:0043531 GO:0005865 GO:0017022 GO:0005829 GO:0030240 GO:0055008 GO:0031674 GO:0042643 GO:0042802 GO:0043234 GO:0042493 GO:0036302 GO:0001725 GO:0009991 GO:0016049 GO:0070062 GO:0016887 GO:0005524 GO:0005200 GO:0010226 |
| sp|P68366|TBA4A_ HUMAN | GO:0048741 GO:0030168 GO:0007017 GO:0014823 GO:0030705 GO:0005525 GO:0044267 GO:0051301 GO:0005881 GO:0005829 GO:0006996 GO:0002576 GO:0070062 GO:0003924 GO:0019899 GO:0051084 GO:0005634 GO:0000086 GO:0005200 |
| sp|P78310|CXAR_ HUMAN | GO:0030593 GO:0009986 GO:0001618 GO:0005887 GO:0005923 GO:0030426 GO:0050776 GO:0016327 GO:0031532 GO:0043234 GO:0007157 GO:0031594 GO:0086072 GO:0005178 GO:0016323 GO:0007596 GO:0048739 GO:0005654 GO:0005615 GO:0045121 GO:0008013 GO:0030175 GO:0007005 GO:0051607 GO:0005912 GO:0045216 GO:0098904 GO:0001669 GO:0042802 GO:0008354 GO:0071253 GO:0010669 GO:0046629 GO:0030165 GO:0046718 GO:0070633 GO:0014704 GO:0086082 GO:0060054 GO:0060044 GO:0044297 |
| sp|P78318|IGBP1_ HUMAN | GO:0035308 GO:0051721 GO:0035306 GO:0000122 GO:2001234 GO:0031434 GO:0032873 GO:0005829 GO:0005874 GO:0019904 GO:0043154 GO:0034612 GO:0008601 GO:0042113 GO:0045944 GO:0070555 GO:0032403 GO:0060632 GO:0034047 |
| sp|P78356|PI42B_ HUMAN | GO:0005654 GO:0005776 GO:0007166 GO:0005057 GO:0005515 GO:0016309 GO:0005789 GO:0005829 GO:0016308 GO:0044281 GO:0006661 GO:2000786 GO:0005886 GO:0005524 GO:0035556 GO:0046854 |
| sp|P78406|RAE1L_ HUMAN | GO:0031047 GO:0007077 GO:1900034 GO:0006406 GO:0071407 GO:0010827 GO:0005737 GO:0005730 GO:0019221 GO:0005643 GO:0008017 GO:0005975 GO:0008033 GO:0044281 GO:0004519 GO:0003723 GO:0019083 GO:0016925 GO:0000088 GO:0055085 GO:0043687 GO:0019054 GO:0090305 |
| sp|P78524|ST5_ HUMAN | GO:0070374 GO:0017112 GO:0043547 |
| sp|P78527|PRKDC_ HUMAN | GO:0043065 GO:0048146 GO:0048660 GO:0032481 GO:0003690 GO:0007507 GO:0007420 GO:0005829 GO:2001229 GO:0033152 GO:0005654 GO:0008134 GO:0033077 GO:0016233 GO:0072431 GO:0000784 GO:0001933 GO:0002684 GO:0045944 GO:0045087 GO:0035234 GO:0032869 GO:0048536 GO:0044822 GO:0048639 GO:0031648 GO:0005958 GO:0014823 GO:0005667 GO:0002360 GO:0005524 GO:0018105 GO:0002328 GO:0001756 GO:0002326 GO:2000773 GO:0008630 GO:0097681 GO:0033153 GO:0043066 GO:0004677 GO:0019899 GO:0005730 GO:0002638 GO:0048538 GO:0016020 GO:0042752 |
| sp|P82673|RT35_ HUMAN | GO:0070126 GO:0070125 GO:0042769 GO:0005743 GO:0003735 GO:0005763 GO:0044822 GO:0070124 |
| sp|P82675|RT05_ HUMAN | GO:0070126 GO:0070125 GO:0005743 GO:0003735 GO:0005763 GO:0044822 GO:0070124 |
| sp|P82909|RT36_ HUMAN | GO:0009353 GO:0070126 GO:0055114 GO:0070125 GO:0006103 GO:0005743 GO:0003735 GO:0005763 GO:0070124 GO:0004591 |
| sp|P82912|RT11_ HUMAN | GO:0070126 GO:0000462 GO:0070125 GO:0042769 GO:0005743 GO:0003735 GO:0070181 GO:0005763 GO:0048027 GO:0070124 GO:0000028 |
| sp|P82933|RT09_ HUMAN | GO:0070126 GO:0000462 GO:0070125 GO:0042769 GO:0005730 GO:0005743 GO:0003735 GO:0005763 GO:0044822 GO:0005515 GO:0070124 |
| sp|P82979|SARNP_ HUMAN | GO:0050733 GO:0000122 GO:0008022 GO:0006406 GO:0016607 GO:0044822 GO:0000978 GO:0001078 GO:0000346 |
| sp|P84101|SERF2_ HUMAN | GO:0005634 GO:0005829 |
| sp|P85037|FOXK1_ HUMAN | GO:0045893 GO:0006357 GO:0000981 GO:0030308 GO:0007517 GO:0000977 GO:0030154 GO:0005515 GO:0009653 GO:0045892 GO:0005634 |
| sp|P98088|MUC5A_ HUMAN | GO:0005796 GO:0036438 GO:0070062 GO:0005615 GO:0048015 GO:0043205 GO:0043687 GO:0030197 GO:0000166 GO:0016266 GO:0043206 |
| sp|P98172|EFNB1_ HUMAN | GO:0007411 GO:0045121 GO:0009880 GO:0005887 GO:0007267 GO:0031295 GO:0003404 GO:0046875 GO:0048013 GO:0001755 GO:0042102 GO:0005829 GO:0048514 GO:0070062 GO:0005634 GO:0045202 |
| sp|Q00403|TF2B_ HUMAN | GO:0001047 GO:0016021 GO:0017025 GO:0008270 GO:0006367 GO:0005730 GO:0006355 GO:0006368 GO:0046966 GO:0016032 GO:0005654 |
| sp|Q00534|CDK6_ HUMAN | GO:0009615 GO:0005654 GO:0007219 GO:0010628 GO:0048699 GO:0045638 GO:0043697 GO:0004693 GO:0060218 GO:0051301 GO:0045646 GO:0005829 GO:0007050 GO:0001726 GO:0000080 GO:0006468 GO:0005813 GO:0045668 GO:0033077 GO:0001954 GO:0021670 GO:0050680 GO:0000082 GO:0003323 GO:2000145 GO:0030332 GO:0000307 GO:0048146 GO:0014002 GO:0005524 GO:0021542 GO:2000773 |
| sp|Q00610|CLH1_ HUMAN | GO:0030506 GO:0006892 GO:0005925 GO:0008022 GO:0030132 GO:0005198 GO:0031523 GO:0030130 GO:0005829 GO:0036020 GO:0030315 GO:0042147 GO:0032588 GO:0071439 GO:0019886 GO:0001649 GO:0003725 GO:0033572 GO:0043195 GO:0043209 GO:0044822 GO:0070062 GO:0006886 GO:0042277 GO:0005739 GO:0031623 GO:0032051 GO:0042470 GO:0005819 GO:0019901 GO:0030669 GO:0090307 GO:1900126 GO:0007030 GO:1903077 GO:0031072 |
| sp|Q00653|NFKB2_ HUMAN | GO:0005654 GO:0032496 GO:0000122 GO:0048511 GO:0032481 GO:0002223 GO:0005515 GO:0038123 GO:0033257 GO:0034142 GO:0001077 GO:0002467 GO:0030198 GO:0002268 GO:0007568 GO:0034162 GO:0034134 GO:0000978 GO:0038061 GO:0034166 GO:0035666 GO:0005829 GO:0033209 GO:0034146 GO:0003713 GO:0048535 GO:0006954 GO:0034138 GO:0045944 GO:0051092 GO:0003682 GO:0002755 GO:0038124 GO:0048536 GO:0007249 |
| sp|Q00688|FKBP3_ HUMAN | GO:0004872 GO:0005789 GO:0005528 GO:0005515 GO:0000413 GO:0005634 GO:0061077 GO:0003755 GO:0044822 |
| sp|Q01081|U2AF1_ HUMAN | GO:0050733 GO:0015030 GO:0000166 GO:0006406 GO:0016607 GO:1903146 GO:0044822 GO:0031124 GO:0089701 GO:0046872 GO:0071013 GO:1903955 GO:0006369 GO:0000398 |
| sp|Q01415|GALK2_ HUMAN | GO:0005737 GO:0046835 GO:0005524 GO:0006012 GO:0004335 GO:0033858 |
| sp|Q01650|LAT1_ HUMAN | GO:0098655 GO:0005829 GO:0042605 GO:0016021 GO:0015820 GO:0005730 GO:0070062 GO:0015190 GO:0006520 GO:0050900 GO:0007399 GO:0015827 GO:0030154 GO:0015196 GO:1902475 GO:0016324 GO:0015823 GO:0015192 GO:0015297 GO:0007596 |
| sp|Q01780|EXOSX_ HUMAN | GO:0000176 GO:0004534 GO:0005730 GO:0009048 GO:0044822 GO:0071048 GO:0005515 GO:0071034 GO:0000166 GO:0090503 GO:0000460 GO:0035327 GO:0071044 GO:0005737 GO:0016020 GO:0000184 GO:0000175 GO:0071035 |
| sp|Q01813|PFKAP_ HUMAN | GO:0005945 GO:0016020 GO:0051289 GO:0030388 GO:0046872 GO:0070095 GO:0032403 GO:0070062 GO:0061621 GO:0098779 GO:0006002 GO:0003872 GO:0005634 GO:0005524 |
| sp|Q01844|EWS_ HUMAN | GO:0005730 GO:0042802 GO:0044822 |
| sp|Q01970|PLCB3_ HUMAN | GO:0005829 GO:0035556 GO:0005516 GO:0043234 GO:0004435 GO:0005509 GO:0004871 GO:0009395 GO:0006892 GO:0043647 GO:0007268 GO:0042383 GO:0007186 GO:0005654 GO:0003073 |
| sp|Q02338|BDH_ HUMAN | GO:0009636 GO:0032868 GO:0055114 GO:0042594 GO:0005743 GO:0045471 GO:0001889 GO:0007420 GO:0032355 GO:0005543 GO:0007584 GO:0046952 GO:0060612 GO:0005759 GO:0042493 GO:0060416 GO:0003858 GO:0044255 GO:0005654 GO:0046686 GO:0051412 GO:0046951 |
| sp|Q02543|RL18A_ HUMAN | GO:0005925 GO:0000184 GO:0006415 GO:0006413 GO:0005515 GO:0016020 GO:0097327 GO:0044822 GO:0016259 GO:0006414 GO:0022625 GO:0005730 GO:0003735 GO:0019083 GO:0006614 |
| sp|Q02790|FKBP4_ HUMAN | GO:0031503 GO:0061077 GO:0005528 GO:0005654 GO:0044295 GO:1900034 GO:0051219 GO:0044822 GO:0005525 GO:0030521 GO:0005789 GO:0030850 GO:0048156 GO:0030674 GO:0005829 GO:0005874 GO:0031072 GO:0006463 GO:0043025 GO:0043234 GO:0007566 GO:0031115 GO:0032767 GO:0003755 GO:0046661 GO:0010977 GO:0048471 GO:0070062 GO:0006825 GO:0035259 GO:0000413 GO:0005524 GO:0005739 |
| sp|Q03252|LMNB2_ HUMAN | GO:0005638 GO:0005637 GO:0005198 |
| sp|Q04206|TF65_ HUMAN | GO:0048011 GO:0033613 GO:0070491 GO:0042803 GO:0034138 GO:0008284 GO:0071159 GO:0047485 GO:0032481 GO:0034142 GO:0031625 GO:0000980 GO:2000630 GO:0042177 GO:0005829 GO:0002223 GO:0006117 GO:0034162 GO:0001942 GO:0005654 GO:0035729 GO:0001889 GO:0000978 GO:0045084 GO:0034166 GO:0071356 GO:0035994 GO:0019221 GO:0051607 GO:0070301 GO:0071316 GO:0043123 GO:0032495 GO:0045944 GO:0032332 GO:0038124 GO:0010224 GO:0034146 GO:0002755 GO:0071222 GO:0007568 GO:0014040 GO:0043200 GO:0001077 GO:0050727 GO:0031293 GO:0016032 GO:0032403 GO:0033256 GO:0051092 GO:0005667 GO:0032570 GO:0070431 GO:0033590 GO:0000122 GO:0042301 GO:0001078 GO:0038095 GO:0035666 GO:0071354 GO:0019901 GO:0046627 GO:0071347 GO:0050852 GO:0051591 GO:0046982 GO:0034134 GO:0071532 GO:0051059 GO:0006968 GO:0042493 GO:0043278 GO:0038123 GO:0001205 GO:1901223 GO:2001237 GO:0031490 GO:0009887 GO:0042826 GO:0042805 |
| sp|Q04695|K1C17_ HUMAN | GO:0030307 GO:0071944 GO:0051798 GO:0031424 GO:0045727 GO:0005737 GO:0031069 GO:0045095 GO:0042289 GO:0007165 GO:0045109 GO:0070062 GO:0032395 GO:0005200 |
| sp|Q04727|TLE4_ HUMAN | GO:0003002 GO:0007164 GO:0001709 GO:0003705 GO:0030901 GO:0007219 GO:0000122 GO:0003714 GO:0016055 GO:0070491 GO:0010628 GO:0048859 GO:0005654 GO:0003682 |
| sp|Q05209|PTN12_ HUMAN | GO:0005829 GO:0035335 GO:0004726 GO:0002102 GO:0042995 GO:0005925 GO:0042246 GO:0005886 GO:0017124 |
| sp|Q05519|SRS11_ HUMAN | GO:0006369 GO:0000398 GO:0031124 GO:0044822 GO:0005515 GO:0006406 GO:0005654 |
| sp|Q06055|AT5G2_ HUMAN | GO:0045471 GO:0015991 GO:0042776 GO:0015078 GO:0005753 GO:0005741 GO:0016021 GO:0045263 GO:0008289 GO:0022904 |
| sp|Q06124|PTN11_ HUMAN | GO:0048011 GO:0006641 GO:0030220 GO:0035855 GO:0035265 GO:0048609 GO:0031748 GO:0043254 GO:0007411 GO:0070374 GO:0046676 GO:0048873 GO:0043234 GO:0060020 GO:0046887 GO:0030971 GO:0031295 GO:0005829 GO:0040014 GO:0060334 GO:0030168 GO:0060338 GO:0048008 GO:0030054 GO:0042593 GO:0048013 GO:0048806 GO:0051428 GO:0048015 GO:0050900 GO:0042445 GO:0036302 GO:0060325 GO:0043560 GO:0000077 GO:0005158 GO:0019904 GO:0005739 GO:0021697 GO:0007173 GO:2001275 GO:0004726 GO:0001725 GO:0038095 GO:0048839 GO:0045931 GO:0061582 GO:0005070 GO:0043274 GO:0009755 GO:0032528 GO:0007229 GO:0060125 GO:0033629 GO:0000187 GO:0008543 GO:0005634 GO:0035335 GO:0051463 GO:0046825 GO:0016020 GO:0008286 GO:0033277 |
| sp|Q06210|GFPT1_ HUMAN | GO:0006042 GO:0045719 GO:0030246 GO:0006541 GO:0036498 GO:0051289 GO:0032869 GO:0032922 GO:0016597 GO:0004360 GO:0006488 GO:0009744 GO:0005829 GO:0006048 GO:0070062 GO:0043687 GO:0006002 GO:0018279 |
| sp|Q06587|RING1_ HUMAN | GO:0048593 GO:0097027 GO:0000122 GO:0005515 GO:0008270 GO:0016607 GO:0016874 GO:0005737 GO:0001739 GO:0050790 GO:0035102 GO:0009952 GO:0016925 GO:0033554 GO:0003682 GO:0043687 GO:0036353 GO:0071339 |
| sp|Q06830|PRDX1_ HUMAN | GO:0001501 GO:0032872 GO:0006367 GO:0042744 GO:0034101 GO:0005759 GO:0042267 GO:0044822 GO:0005719 GO:0020037 GO:0008283 GO:0005615 GO:0042470 GO:0019430 GO:0008379 GO:0043209 GO:0005730 GO:0005829 GO:0001895 GO:0042345 GO:0055114 GO:0070062 GO:0042803 GO:0005782 |
| sp|Q07812|BAX_ HUMAN | GO:0097144 GO:0008289 GO:0043621 GO:0004882 GO:0043525 GO:0008053 GO:0030331 GO:0048087 GO:0006808 GO:0030521 GO:2001244 GO:2001022 GO:0051281 GO:1902263 GO:0001541 GO:0021854 GO:0034644 GO:0002358 GO:0071850 GO:0032403 GO:0060442 GO:0014068 GO:2001241 GO:0043491 GO:0046982 GO:0060058 GO:0005741 GO:0051781 GO:0045892 GO:0005789 GO:0035234 GO:0097162 GO:0008134 GO:0010332 GO:0042220 GO:0042803 GO:0043401 GO:0007281 GO:0045136 GO:1990009 GO:0071456 GO:1902445 GO:0035260 GO:0031072 GO:0006987 GO:0001822 GO:0032471 GO:0051412 GO:0060011 GO:0008284 GO:0045333 GO:0046688 GO:0009636 GO:1901030 GO:0051087 GO:0008625 GO:0030295 GO:0060664 GO:0045944 GO:0001764 GO:0006687 GO:0001844 GO:1902512 GO:0033137 GO:0001974 GO:0008656 GO:0070062 GO:0002262 GO:2000836 GO:0007008 GO:0045931 GO:0001047 GO:0008635 GO:0060041 GO:0008630 GO:0048147 GO:0070584 GO:0043524 GO:0042826 GO:0046666 GO:0032976 GO:2001234 GO:0034349 GO:0071899 GO:0070059 GO:0045930 GO:0051434 GO:1903896 GO:0048597 GO:0005829 GO:0009566 GO:0002904 GO:0043497 GO:0005635 GO:0043653 GO:0072332 GO:0005757 GO:0007568 GO:1990117 GO:0033599 GO:0015267 GO:1902262 GO:0070242 GO:0043496 GO:0048873 GO:0000976 GO:0000983 GO:0051260 GO:0097296 GO:0030284 GO:0016032 GO:0060068 GO:0001777 GO:0033574 GO:0048471 GO:0006927 GO:0009651 GO:0042475 GO:0032147 GO:0021987 GO:0042493 GO:0060770 GO:0043569 GO:0048515 GO:0051881 GO:0032461 GO:0002352 GO:0090200 GO:0010248 GO:0048678 GO:0001782 GO:0007007 |
| sp|Q07955|SRSF1_ HUMAN | GO:0097421 GO:0050733 GO:0048709 GO:0006406 GO:0016607 GO:0000395 GO:0060048 GO:0035145 GO:0005737 GO:0031124 GO:0071013 GO:0006369 GO:0033120 GO:0000380 GO:0070062 GO:0001701 GO:0003729 |
| sp|Q07960|RHG01_ HUMAN | GO:0009967 GO:0005829 GO:0016021 GO:0001726 GO:0005070 GO:0070062 GO:0043547 GO:0051056 GO:0048471 GO:0005096 GO:0007266 GO:0005886 GO:0017124 |
| sp|Q08170|SRSF4_ HUMAN | GO:0000166 GO:0006406 GO:0016607 GO:0048025 GO:0031124 GO:0006369 GO:0002244 GO:1990825 |
| sp|Q08211|DHX9_ HUMAN | GO:0005654 GO:0032481 GO:0032508 GO:0001649 GO:0016020 GO:0070934 GO:0044822 GO:0005730 GO:0005829 GO:0097165 GO:0005813 GO:0036464 GO:0003677 GO:0001085 GO:0034605 GO:0004003 GO:0000398 GO:0070937 GO:0004004 GO:0045087 GO:0005524 GO:0007623 GO:1903608 |
| sp|Q08257|QOR_ HUMAN | GO:0070404 GO:0003730 GO:0005829 GO:0055114 GO:0008270 GO:0070062 GO:0042178 GO:0003677 GO:0003960 GO:0007601 GO:0070402 GO:0005212 GO:0051289 GO:0005794 |
| sp|Q08378|GOGA3_ HUMAN | GO:0005730 GO:0032580 GO:0006891 GO:0005654 GO:0090498 GO:0005515 GO:0005829 GO:0017119 GO:0005215 |
| sp|Q08426|ECHP_ HUMAN | GO:0005829 GO:0005777 GO:0005739 GO:0006637 GO:0004165 GO:0019899 GO:0005102 GO:0003857 GO:0006635 GO:0006475 GO:0004300 |
| sp|Q08722|CD47_ HUMAN | GO:0008228 GO:0007229 GO:0050766 GO:0070062 GO:0050729 GO:0050900 GO:0009617 GO:0070053 GO:0005887 GO:0005515 GO:0008284 GO:0030198 GO:0050870 GO:0007596 |
| sp|Q08J23|NSUN2_ HUMAN | GO:0007067 GO:0016428 GO:0005730 GO:0033391 GO:0007286 GO:0051301 GO:0044822 GO:0033313 GO:0030488 GO:0005819 GO:0000049 GO:0005654 |
| sp|Q09028|RBBP4_ HUMAN | GO:0033186 GO:0000980 GO:0008094 GO:0042393 GO:0042826 GO:0031492 GO:0035098 GO:0016581 GO:0000978 GO:0006335 GO:0031101 GO:0008285 GO:0006355 GO:0016589 GO:0051726 GO:0009790 GO:0016580 GO:0045814 GO:0033554 GO:0060416 GO:0034080 GO:0000086 GO:0006260 |
| sp|Q09666|AHNK_ HUMAN | GO:0005925 GO:0045121 GO:0044291 GO:0044822 GO:0043484 GO:0015629 GO:0043034 GO:0044548 GO:0005765 GO:0005829 GO:0051259 GO:0097493 GO:1901385 GO:0030315 GO:0070062 GO:0019013 GO:0005634 |
| sp|Q0VDF9|HSP7E_ HUMAN | GO:0051083 GO:0005840 GO:0005515 GO:0005524 GO:0016020 GO:0005829 |
| sp|Q10567|AP1B1_ HUMAN | GO:0030669 GO:0030131 GO:0019901 GO:0008565 GO:0007507 GO:0032588 GO:0005765 GO:0006886 GO:0005829 GO:0061024 GO:0019886 GO:0050690 GO:0007368 GO:0006892 GO:0016032 |
| sp|Q10713|MPPA_ HUMAN | GO:0008270 GO:0005743 GO:0006627 GO:0005615 GO:0004222 GO:0005759 |
| sp|Q12792|TWF1_ HUMAN | GO:0005925 GO:0005911 GO:0005546 GO:0042989 GO:0032587 GO:0043538 GO:0015629 GO:0030175 GO:0004713 GO:0030016 GO:0018108 GO:0003785 GO:0048471 GO:0032403 GO:0005524 GO:0051016 |
| sp|Q12797|ASPH_ HUMAN | GO:0060316 GO:0005513 GO:0030176 GO:0031585 GO:1901879 GO:0005829 GO:0034704 GO:0097202 GO:0032541 GO:0044325 GO:0010881 GO:0010649 GO:0005886 GO:0014701 GO:0060325 GO:0071277 GO:0042264 GO:1903779 GO:0055114 GO:0009055 GO:0008285 GO:0045893 GO:0004597 GO:0032237 GO:0035108 GO:0007389 GO:0008307 GO:0005509 GO:0060021 GO:0033018 GO:0031647 GO:0090316 GO:0033198 |
| sp|Q12802|AKP13_ HUMAN | GO:0004871 GO:1900169 GO:0016020 GO:0035023 GO:0051168 GO:0045859 GO:0010611 GO:0048011 GO:0051018 GO:0005829 GO:0046872 GO:0097190 GO:0008104 GO:0043065 GO:0017048 GO:0048471 GO:0043547 GO:0005089 GO:0005634 GO:0004691 GO:0032947 |
| sp|Q12849|GRSF1_ HUMAN | GO:0003676 GO:0043232 GO:0007275 GO:0005739 |
| sp|Q12904|AIMP1_ HUMAN | GO:0001525 GO:0009611 GO:0030133 GO:0007267 GO:0016020 GO:0007155 GO:0051020 GO:0005125 GO:0006418 GO:0051607 GO:0005615 GO:0006915 GO:0017101 GO:0050900 GO:0005794 GO:0005829 GO:0009986 GO:0001937 GO:0006006 GO:0007165 GO:0001887 GO:0006935 GO:0006954 GO:0000049 GO:0042803 GO:0005783 GO:0005634 |
| sp|Q12929|EPS8_ HUMAN | GO:0008360 GO:0032587 GO:0048149 GO:0030426 GO:0051017 GO:0008283 GO:0007173 GO:0003779 GO:0051764 GO:0036336 GO:0009967 GO:0016601 GO:0005903 GO:0070358 GO:0014069 GO:0048365 GO:0010458 GO:0030054 GO:0032420 GO:0005070 GO:0070062 GO:0031532 GO:0051016 GO:0005938 GO:0017146 GO:0008344 |
| sp|Q12996|CSTF3_ HUMAN | GO:0006379 GO:0016021 GO:0003729 GO:0006369 GO:0048936 GO:0006378 GO:0000398 GO:0005515 GO:0005654 |
| sp|Q13011|ECH1_ HUMAN | GO:0008416 GO:0005777 GO:0000247 GO:0006635 GO:0005102 GO:0016020 GO:0070062 GO:0005739 GO:0018842 |
| sp|Q13017|RHG05_ HUMAN | GO:0005829 GO:0042169 GO:0030879 GO:0002053 GO:0008361 GO:0007155 GO:0030335 GO:0043547 GO:0003924 GO:0051056 GO:0005096 GO:0007266 GO:0016020 GO:0005783 GO:0005525 |
| sp|Q13043|STK4_ HUMAN | GO:0033138 GO:0004674 GO:0042803 GO:0000902 GO:0003157 GO:0007346 GO:0032092 GO:0001841 GO:0035329 GO:0032147 GO:0043234 GO:1902043 GO:0005829 GO:0043539 GO:0005654 GO:0008134 GO:0000287 GO:0023014 GO:0090090 GO:0071902 GO:0030216 GO:0046621 GO:0097284 GO:0005524 GO:0001569 GO:0008285 GO:0007417 GO:0046777 GO:0050821 GO:0031098 GO:0060215 GO:0045600 GO:0060800 |
| sp|Q13045|FLII_ HUMAN | GO:0005815 GO:0045214 GO:0005903 GO:0051014 GO:0006355 GO:0005925 GO:0003779 GO:0007275 GO:0005654 GO:0005737 |
| sp|Q13057|COASY_ HUMAN | GO:0005654 GO:0005515 GO:0004595 GO:0015939 GO:0015937 GO:0005759 GO:0004140 GO:0016310 GO:0005741 GO:0070062 GO:0005524 |
| sp|Q13123|RED_ HUMAN | GO:0005730 GO:0042802 GO:0005615 GO:0005654 GO:0007267 GO:0006955 |
| sp|Q13126|MTAP_ HUMAN | GO:0017061 GO:0005829 GO:0006166 GO:0019509 GO:0006738 GO:0070062 GO:0032259 GO:0005515 GO:0006595 GO:0004645 GO:0005634 |
| sp|Q13131|AAPK1_ HUMAN | GO:0014823 GO:0042752 GO:0050995 GO:0042149 GO:0048643 GO:0032007 GO:0035174 GO:0047322 GO:0010332 GO:0070301 GO:0007050 GO:0000187 GO:0019900 GO:0019395 GO:0046872 GO:0042593 GO:0016236 GO:0071456 GO:0045821 GO:0043066 GO:0016324 GO:0008284 GO:0071380 GO:0006633 GO:0009631 GO:0004691 GO:2000505 GO:0031588 GO:0010508 GO:0005654 GO:0033135 GO:0006006 GO:0071361 GO:0035404 GO:0010628 GO:0050321 GO:0008022 GO:0005829 GO:0051291 GO:0006355 GO:0004679 GO:0006367 GO:0005524 GO:0045542 GO:0009411 GO:0016055 GO:0050405 GO:0055089 GO:0003682 GO:1901563 GO:0031000 GO:2001274 GO:0060627 GO:0008286 GO:0046318 |
| sp|Q13136|LIPA1_ HUMAN | GO:0005829 GO:0016021 GO:0004871 GO:0051497 GO:0007160 GO:0014047 GO:0005925 GO:0007269 GO:0005515 GO:0007165 GO:0090005 GO:0048786 |
| sp|Q13151|ROA0_ HUMAN | GO:0019013 GO:0006954 GO:0000398 GO:0030529 GO:0017091 GO:0044822 GO:0070935 GO:0000166 GO:0019901 GO:0005654 GO:0032496 |
| sp|Q13177|PAK2_ HUMAN | GO:0005856 GO:0043296 GO:0071407 GO:0007411 GO:0043297 GO:0048010 GO:0030496 GO:0032147 GO:2001271 GO:0030027 GO:0031295 GO:0005829 GO:0002223 GO:0043408 GO:0005654 GO:0004697 GO:0060996 GO:0050690 GO:0006921 GO:0071560 GO:0003300 GO:0006469 GO:0005886 GO:0040008 GO:0016032 GO:0048471 GO:0032154 GO:0042802 GO:0030141 GO:0005524 GO:0038095 GO:0018105 GO:0045931 GO:0061098 GO:0019901 GO:0050852 GO:2000145 GO:0046777 GO:2001238 GO:0031098 GO:0010631 GO:0030036 GO:0007266 GO:0030296 GO:0032467 GO:0006355 GO:0042826 GO:0048365 |
| sp|Q13190|STX5_ HUMAN | GO:0005654 GO:0012507 GO:0000139 GO:0034498 GO:0005484 GO:0000149 GO:1903358 GO:0090166 GO:0047485 GO:0005789 GO:0006886 GO:0048280 GO:0045732 GO:0033116 GO:0048208 GO:0016021 GO:0048278 GO:0043687 GO:0031201 GO:0018279 |
| sp|Q13200|PSMD2_ HUMAN | GO:0048011 GO:0051436 GO:0030234 GO:0008540 GO:0002479 GO:0090263 GO:0007411 GO:0048010 GO:0000186 GO:0006977 GO:0002223 GO:0051437 GO:0005654 GO:0006595 GO:0031145 GO:0038061 GO:0034515 GO:0007265 GO:0004175 GO:0090090 GO:0033209 GO:0070062 GO:0016032 GO:0006521 GO:0043488 GO:0005515 GO:0000084 GO:0007173 GO:0038095 GO:0050852 GO:0000090 GO:0043066 GO:0008543 GO:0000209 GO:0016020 GO:0008286 |
| sp|Q13257|MD2L1_ HUMAN | GO:0072686 GO:0043066 GO:0000236 GO:0000777 GO:0090267 GO:0005829 GO:0005643 GO:0007264 GO:0060564 GO:0000090 GO:0048471 GO:0042803 GO:0000922 |
| sp|Q13263|TIF1B_ HUMAN | GO:1902187 GO:0016925 GO:0004842 GO:0070736 GO:0004672 GO:0060669 GO:0031625 GO:0045739 GO:0070087 GO:0042993 GO:1901536 GO:0070738 GO:0016567 GO:0070740 GO:0005654 GO:0001105 GO:0001837 GO:0003714 GO:0043774 GO:0005719 GO:0005720 GO:0045944 GO:0045087 GO:0043773 GO:0044822 GO:0003700 GO:0008270 GO:0006367 GO:0018169 GO:0000122 GO:0007566 GO:0090575 GO:0060028 GO:0046777 GO:2000653 GO:0043565 GO:0045869 GO:0043045 GO:0051259 GO:0005730 GO:0008766 GO:0035851 GO:0070737 GO:0043388 |
| sp|Q13308|PTK7_ HUMAN | GO:0005925 GO:0003401 GO:0005911 GO:0005887 GO:0005515 GO:0060026 GO:0007155 GO:0060484 GO:0004713 GO:0060976 GO:0045198 GO:1904929 GO:0042060 GO:0090103 GO:0071300 GO:0090179 GO:0018108 GO:0010976 GO:0003281 GO:0031532 GO:0005524 GO:0016477 GO:0060070 |
| sp|Q13404|UB2V1_ HUMAN | GO:0002223 GO:0070534 GO:0038123 GO:0038095 GO:0034142 GO:0031624 GO:0034134 GO:0034162 GO:0034166 GO:0043123 GO:0035370 GO:0005829 GO:0000151 GO:0061631 GO:0034146 GO:0031625 GO:0042275 GO:0050852 GO:0006282 GO:0070423 GO:0030154 GO:0034138 GO:0070062 GO:0051092 GO:0002755 GO:0005634 GO:0038124 GO:0061630 |
| sp|Q13428|TCOF_ HUMAN | GO:0005730 GO:0001501 GO:0044822 GO:0005515 GO:0005215 GO:0005737 |
| sp|Q13442|HAP28_ HUMAN | GO:0044822 GO:0007165 |
| sp|Q13501|SQSTM_ HUMAN | GO:0043065 GO:0031410 GO:0048011 GO:0016235 GO:0043122 GO:0005080 GO:0004674 GO:0042803 GO:0097190 GO:0097225 GO:0031625 GO:0005783 GO:0016605 GO:0070530 GO:0008104 GO:0030971 GO:0044753 GO:0005829 GO:0098779 GO:0003712 GO:0042169 GO:0016239 GO:0046578 GO:0001934 GO:0045944 GO:0010821 GO:0044130 GO:0070062 GO:0008270 GO:0000122 GO:0002376 GO:0030154 GO:0000932 GO:0044754 GO:0000407 GO:0043066 GO:0051291 GO:0005770 GO:0006511 GO:0016197 GO:0061635 |
| sp|Q13505|MTX1_ HUMAN | GO:0016021 GO:0044267 GO:0005743 GO:0006626 GO:0005741 |
| sp|Q13574|DGKZ_ HUMAN | GO:0005654 GO:0043086 GO:0030027 GO:0030168 GO:0008022 GO:0046834 GO:0046580 GO:0007205 GO:0005737 GO:0046872 GO:0004143 GO:0031571 GO:0001727 GO:0005886 GO:0004857 GO:0005524 GO:0016477 |
| sp|Q13596|SNX1_ HUMAN | GO:0072673 GO:0005154 GO:0030027 GO:0030512 GO:0034498 GO:0035091 GO:0005794 GO:0006886 GO:0005829 GO:0045732 GO:0016050 GO:0046982 GO:1990460 GO:0031623 GO:0042803 GO:0031901 GO:0019898 GO:1990459 GO:0030905 GO:0005158 |
| sp|Q13601|KRR1_ HUMAN | GO:0005737 GO:0005730 GO:0032040 GO:0005515 GO:0006364 GO:0016020 GO:0045171 GO:0044822 |
| sp|Q13619|CUL4A_ HUMAN | GO:0005654 GO:0042769 GO:1900087 GO:0019985 GO:0042787 GO:0006283 GO:0030853 GO:0070911 GO:0097193 GO:0045732 GO:0008285 GO:0007050 GO:0043161 GO:2000819 GO:0008284 GO:0031464 GO:0031625 GO:0033683 GO:0035019 GO:0000715 GO:2000001 GO:0016032 GO:0061630 GO:0001701 |
| sp|Q13724|MOGS_ HUMAN | GO:0016021 GO:0018279 GO:0006457 GO:0070062 GO:0043687 GO:0004573 GO:0009311 GO:0005789 |
| sp|Q13813|SPTN1_ HUMAN | GO:0048011 GO:0005916 GO:0047485 GO:0043687 GO:0007411 GO:0008091 GO:0048010 GO:0000186 GO:0031532 GO:0043234 GO:0005829 GO:0005200 GO:0051693 GO:0032437 GO:0043231 GO:0006888 GO:0006921 GO:0045087 GO:0007265 GO:0070062 GO:0032403 GO:0007173 GO:0038095 GO:0046982 GO:0005516 GO:0019905 GO:0005509 GO:0018279 GO:0061024 GO:0030018 GO:0008543 GO:0015630 GO:0008286 GO:0016328 GO:0003779 |
| sp|Q13884|SNTB1_ HUMAN | GO:0005737 GO:0005856 GO:0042383 GO:0006936 GO:0003779 GO:0005925 GO:0016010 GO:0045202 GO:0005516 GO:0030165 GO:0005198 |
| sp|Q13885|TBB2A_ HUMAN | GO:0051084 GO:0001764 GO:0044267 GO:0005874 GO:0070062 GO:0005515 GO:0007017 GO:0003924 GO:0005200 GO:0005737 GO:0005525 GO:0005634 |
| sp|Q13895|BYST_ HUMAN | GO:0071363 GO:0000462 GO:0005730 GO:0071347 GO:0071407 GO:0007420 GO:0007565 GO:0005881 GO:0007155 GO:0042995 GO:0044822 GO:0005515 GO:0001829 GO:0048471 GO:0008283 GO:0071222 GO:0045177 GO:0016020 GO:0030182 |
| sp|Q13948|CASP_ HUMAN | GO:0050775 GO:0043005 GO:0005654 GO:0030324 GO:0000122 GO:0001822 GO:0000977 GO:0030173 GO:0005829 GO:0030674 GO:0000301 GO:0045944 GO:0003682 GO:0001205 GO:0042491 |
| sp|Q13952|NFYC_ HUMAN | GO:0006457 GO:0045944 GO:0001077 GO:0032993 GO:0016602 GO:0008134 GO:0003713 GO:0005654 GO:0044281 GO:0000978 |
| sp|Q14008|CKAP5_ HUMAN | GO:0005829 GO:0007067 GO:0050658 GO:0000086 GO:0000922 GO:0007264 GO:0005813 GO:0051301 GO:0051297 GO:0000090 GO:0007051 GO:0005515 GO:0000236 GO:0000930 GO:0016020 GO:0030951 GO:0035371 |
| sp|Q14019|COTL1_ HUMAN | GO:0005856 GO:0070062 GO:0030041 GO:0019899 GO:0003779 GO:0001755 GO:0005737 GO:0050832 GO:0005634 |
| sp|Q14126|DSG2_ HUMAN | GO:0050839 GO:0060135 GO:0086091 GO:0003165 GO:0006921 GO:0098911 GO:0086083 GO:0007156 GO:0005509 GO:0030057 GO:0009986 GO:0086073 GO:0016328 GO:0002934 GO:0016021 GO:0032570 GO:0014704 GO:0070062 GO:0016324 |
| sp|Q14151|SAFB2_ HUMAN | GO:0070062 GO:0042802 GO:0044822 GO:0005654 |
| sp|Q14157|UBP2L_ HUMAN | GO:0005671 GO:0007339 GO:0031519 GO:0005515 GO:0061484 GO:0044822 |
| sp|Q14165|MLEC_ HUMAN | GO:0005789 GO:0006457 GO:0018279 GO:0030246 GO:0019899 GO:0016021 GO:0043687 |
| sp|Q14197|ICT1_ HUMAN | GO:0070126 GO:0005762 GO:0070125 GO:0072344 GO:0005743 GO:0004045 GO:0016149 GO:0043022 GO:0016150 GO:0070124 |
| sp|Q14204|DYHC1_ HUMAN | GO:0016020 GO:0044822 GO:0007052 GO:0030175 GO:0005829 GO:0034063 GO:0005874 GO:0061024 GO:0003777 GO:0019886 GO:0005813 GO:0051959 GO:0006888 GO:0016887 GO:0070062 GO:0051293 GO:0043687 GO:0005868 GO:0007018 GO:0005524 GO:0000086 GO:0033962 GO:0018279 |
| sp|Q14240|IF4A2_ HUMAN | GO:0005515 GO:0044822 GO:1900260 GO:0003743 GO:0016281 GO:0005829 GO:0019221 GO:0006446 GO:0048471 GO:0016032 GO:0000289 GO:0004004 GO:0005524 GO:0010501 |
| sp|Q14257|RCN2_ HUMAN | GO:0005730 GO:0005509 GO:0005515 GO:0005788 |
| sp|Q14258|TRI25_ HUMAN | GO:0005654 GO:1902187 GO:0005515 GO:0008270 GO:0019985 GO:0033280 GO:0006513 GO:0060333 GO:0042787 GO:0044822 GO:0043627 GO:0051607 GO:0043123 GO:0005829 GO:0036503 GO:0046597 GO:0016881 GO:0032480 GO:0051092 GO:1904264 GO:0003700 |
| sp|Q14320|FA50A_ HUMAN | GO:0007283 GO:0005654 GO:0044822 |
| sp|Q14331|FRG1_ HUMAN | GO:0030018 GO:0015030 GO:0005730 GO:0006364 GO:0000398 GO:0007517 GO:0044822 GO:0003779 GO:0071013 |
| sp|Q14376|GALE_ HUMAN | GO:0005829 GO:0033499 GO:0070062 GO:0061623 GO:0003978 GO:0003974 GO:0050662 GO:0042803 |
| sp|Q14444|CAPR1_ HUMAN | GO:0017148 GO:0005887 GO:0044822 GO:0010494 GO:0042995 GO:0000932 GO:0061003 |
| sp|Q14573|ITPR3_ HUMAN | GO:0048011 GO:0035023 GO:0050917 GO:0005791 GO:0005887 GO:0005089 GO:0035091 GO:0048010 GO:0005903 GO:0005220 GO:0043547 GO:0005829 GO:0002223 GO:0007613 GO:0005654 GO:0000822 GO:0038096 GO:0031095 GO:0030168 GO:0048016 GO:0051260 GO:0007202 GO:0051592 GO:0030658 GO:0044281 GO:0006112 GO:0050916 GO:0043235 GO:0050913 GO:0043209 GO:0070679 GO:0048471 GO:1903779 GO:0030141 GO:0071320 GO:0005515 GO:0007173 GO:0043533 GO:0038095 GO:0007186 GO:0030425 GO:0060291 GO:0045177 GO:0005509 GO:0050796 GO:0005640 GO:0051291 GO:0051209 GO:0008543 GO:0005730 GO:0043025 GO:0005789 |
| sp|Q14651|PLSI_ HUMAN | GO:0090004 GO:1990357 GO:0040018 GO:0005509 GO:0032532 GO:0001951 GO:0005903 GO:0030033 GO:0051015 GO:0070062 GO:1902896 GO:0005200 |
| sp|Q14669|TRIPC_ HUMAN | GO:0005654 GO:0070740 GO:0046966 GO:0070738 GO:0043773 GO:0042787 GO:0043774 GO:0004842 GO:0008766 GO:0070736 GO:1901315 GO:0009790 GO:0070737 GO:0018169 GO:2000780 |
| sp|Q14683|SMC1A_ HUMAN | GO:0005654 GO:0036033 GO:0000236 GO:0019827 GO:0042770 GO:0044822 GO:0007052 GO:0030893 GO:0000724 GO:0000777 GO:0005730 GO:0005829 GO:0046982 GO:0003777 GO:0008280 GO:0016021 GO:0000090 GO:0000794 GO:0007064 GO:0016925 GO:0009314 GO:0000084 GO:0003682 GO:0043687 GO:0000398 GO:0007093 GO:0032876 GO:0007126 GO:0005524 GO:0000910 |
| sp|Q14690|RRP5_ HUMAN | GO:0005730 GO:0032040 GO:0006397 GO:0006364 GO:0008134 GO:0005829 GO:0044822 |
| sp|Q14692|BMS1_ HUMAN | GO:0005730 GO:0005524 GO:0042255 GO:0044822 |
| sp|Q14694|UBP10_ HUMAN | GO:0010506 GO:0006511 GO:0002039 GO:0030330 GO:0044325 GO:0004197 GO:0005769 GO:0044822 GO:0004843 GO:0016579 GO:0019985 GO:0005654 |
| sp|Q14696|MESD_ HUMAN | GO:0006457 GO:0034394 GO:0007498 GO:0005886 GO:0050750 |
| sp|Q14728|MFS10_ HUMAN | GO:0030659 GO:0016021 GO:0043252 GO:0031526 GO:0008493 GO:0008514 GO:0015904 |
| sp|Q14789|GOGB1_ HUMAN | GO:0016021 GO:0018279 GO:0000139 GO:0005793 GO:0061024 GO:0005795 GO:0043687 GO:0044822 GO:0005515 GO:0007030 GO:0006888 GO:0005801 |
| sp|Q147X3|NAA30_ HUMAN | GO:0005844 GO:0031417 GO:0005515 GO:0017196 GO:0004596 |
| sp|Q14974|IMB1_ HUMAN | GO:0008139 GO:0005654 GO:0008270 GO:0034399 GO:0000059 GO:0007080 GO:0075733 GO:0008565 GO:0031291 GO:0044822 GO:0031965 GO:0051879 GO:0019894 GO:0008536 GO:0006610 GO:0005829 GO:0019221 GO:0005643 GO:0006607 GO:0019904 GO:0040001 GO:0006309 GO:0090307 GO:0043234 GO:0000060 GO:0044281 GO:0030953 GO:0032403 GO:0070062 GO:0007079 GO:0019054 GO:0071782 |
| sp|Q14980|NUMA1_ HUMAN | GO:0005654 GO:0005198 GO:0007067 GO:0000139 GO:0051321 GO:0060487 GO:0000132 GO:0061673 GO:0051301 GO:0005829 GO:0030425 GO:0008017 GO:0043025 GO:0005694 GO:0005813 GO:0097431 GO:0006997 GO:0016363 GO:0045177 GO:0070062 GO:0000086 GO:0005938 |
| sp|Q14BN4|SLMAP_ HUMAN | GO:0005615 GO:0005887 GO:0005815 GO:0042383 GO:0006936 GO:0005515 GO:0005790 |
| sp|Q15008|PSMD6_ HUMAN | GO:0048011 GO:0051436 GO:0060271 GO:0070306 GO:0002479 GO:0090263 GO:0007411 GO:0048010 GO:0000186 GO:0006977 GO:0005838 GO:0005829 GO:0002223 GO:0051437 GO:0005654 GO:0006595 GO:0031145 GO:0070121 GO:0038061 GO:0007265 GO:0090090 GO:0033209 GO:0070062 GO:0016032 GO:0006521 GO:0043488 GO:0005515 GO:0000084 GO:0007173 GO:0038095 GO:0016887 GO:0050852 GO:0000090 GO:0043066 GO:0008543 GO:0007368 GO:0000209 GO:0008286 |
| sp|Q15018|F175B_ HUMAN | GO:0031593 GO:0008017 GO:0008608 GO:0002931 GO:0031616 GO:0030496 GO:0036449 GO:0090307 GO:0071497 GO:0051301 GO:0070536 GO:0070552 GO:0005737 GO:0043066 |
| sp|Q15019|SEPT2_ HUMAN | GO:0005654 GO:0007067 GO:0035869 GO:0000777 GO:0005525 GO:0031175 GO:0043209 GO:0005730 GO:0000145 GO:0051258 GO:0032880 GO:0032154 GO:0042802 GO:0032391 GO:0007224 GO:0002036 GO:0050790 GO:0042384 GO:0030496 GO:0005930 GO:0048471 GO:0070062 GO:0005876 GO:0003924 GO:0031105 GO:0060170 GO:0045202 GO:0000910 GO:0030234 GO:0005826 GO:0032947 |
| sp|Q15020|SART3_ HUMAN | GO:1903586 GO:0042393 GO:0015030 GO:0000244 GO:0071002 GO:0046540 GO:0000166 GO:0016607 GO:1990381 GO:0044822 GO:0030624 GO:0000902 GO:0010468 GO:0005737 GO:0000245 GO:0048872 GO:0017070 GO:0005691 GO:0071001 GO:0006334 GO:0071425 GO:0030621 |
| sp|Q15046|SYK_ HUMAN | GO:0005829 GO:0017101 GO:0015630 GO:0004824 GO:0046872 GO:0008033 GO:0015966 GO:0001887 GO:0005524 GO:0005515 GO:0005759 GO:0016597 GO:0006430 GO:0016032 GO:0000049 GO:0005576 GO:0005634 GO:0005886 |
| sp|Q15050|RRS1_ HUMAN | GO:0005730 GO:0000794 GO:0007080 GO:0042273 GO:0002244 GO:0044822 GO:0005515 GO:0030687 GO:0000447 GO:0005783 GO:0000055 |
| sp|Q15056|IF4H_ HUMAN | GO:0003743 GO:0016281 GO:0005829 GO:0019953 GO:0048589 GO:0044822 GO:0005515 GO:0006446 GO:0016020 |
| sp|Q15061|WDR43_ HUMAN | GO:0031023 GO:0007097 GO:0008017 GO:0005875 GO:0005874 GO:2000574 GO:0047496 GO:0000003 GO:0005813 GO:0005938 GO:0044822 GO:0000776 GO:0045502 GO:0000132 GO:0001650 |
| sp|Q15075|EEA1_ HUMAN | GO:0005829 GO:0031901 GO:0005516 GO:0019897 GO:0006906 GO:0008270 GO:0005545 GO:0016189 GO:0044308 GO:0070062 GO:0031410 GO:0030742 GO:0055037 GO:0045022 GO:0006897 GO:0042803 GO:0005969 |
| sp|Q15084|PDIA6_ HUMAN | GO:0070527 GO:0003756 GO:0044267 GO:0006457 GO:0034663 GO:0005793 GO:0045454 GO:0070062 GO:0042470 GO:0005788 GO:0005515 GO:0036498 GO:0005789 GO:0005886 |
| sp|Q15149|PLEC_ HUMAN | GO:0005829 GO:0047485 GO:0008307 GO:0043034 GO:0009925 GO:0006921 GO:0005903 GO:0030506 GO:0031581 GO:0007584 GO:0007565 GO:0070062 GO:0045111 GO:0016528 GO:0044822 GO:0030056 GO:0005925 GO:0003779 GO:0030855 GO:0048471 GO:0005200 GO:0016324 GO:0042383 GO:0030198 |
| sp|Q15155|NOMO1_ HUMAN | GO:0016021 GO:0031648 GO:0005789 GO:0030246 GO:0005515 |
| sp|Q15286|RAB35_ HUMAN | GO:0019882 GO:0005546 GO:0000139 GO:0005515 GO:0032456 GO:0031253 GO:0042470 GO:0005525 GO:0015031 GO:0005789 GO:0031175 GO:0005905 GO:0007264 GO:0045171 GO:0006888 GO:0070062 GO:0019003 GO:0003924 GO:0008152 GO:0048227 GO:0010008 GO:1990090 GO:0036010 GO:0000910 GO:0005739 GO:0045334 |
| sp|Q15334|L2GL1_ HUMAN | GO:0050708 GO:0017157 GO:0005198 GO:0035748 GO:0032878 GO:0006893 GO:0019901 GO:0006461 GO:0017137 GO:0051294 GO:0007409 GO:0032588 GO:0007420 GO:0005096 GO:0030424 GO:0035090 GO:0043547 GO:0030864 GO:0005886 GO:0030866 GO:0000137 GO:0031901 GO:0008593 GO:0019905 |
| sp|Q15370|ELOB_ HUMAN | GO:0070449 GO:0006461 GO:0003746 GO:0004842 GO:0006414 GO:0061418 GO:0005829 GO:0031466 GO:0005667 GO:0031625 GO:0003713 GO:0016567 GO:0050434 GO:0031462 GO:0045944 GO:0032403 GO:0070062 GO:0030891 GO:0006368 |
| sp|Q15392|DHC24_ HUMAN | GO:0050660 GO:0007265 GO:0009888 GO:0000139 GO:0031639 GO:0009725 GO:0050614 GO:0005789 GO:0005829 GO:0061024 GO:0008285 GO:0007050 GO:0043154 GO:0016614 GO:0008104 GO:0005856 GO:0016021 GO:0043588 GO:1901214 GO:0030539 GO:0055114 GO:0042605 GO:0033489 GO:0042987 GO:0000246 GO:0033490 GO:0019899 GO:0005634 GO:0006979 |
| sp|Q15393|SF3B3_ HUMAN | GO:0030532 GO:0003723 GO:0005730 GO:0042177 GO:0000398 GO:0006461 GO:0005515 GO:0071013 GO:0005654 GO:0005689 |
| sp|Q15404|RSU1_ HUMAN | GO:0005829 GO:0007265 GO:0070062 GO:0010811 GO:0005925 GO:0005515 GO:0043547 GO:0034329 GO:2000179 |
| sp|Q15418|KS6A1_ HUMAN | GO:0048011 GO:0043555 GO:0034138 GO:2000491 GO:0007411 GO:0034142 GO:0007268 GO:0005829 GO:0034162 GO:0005654 GO:0008134 GO:0000287 GO:0072574 GO:0034166 GO:0043027 GO:0004712 GO:0045597 GO:0045944 GO:0038124 GO:0034146 GO:0002755 GO:0051403 GO:0030307 GO:0005524 GO:0004711 GO:0043154 GO:0005819 GO:0035666 GO:0007049 GO:0034134 GO:0005840 GO:0038123 GO:0043620 |
| sp|Q15645|PCH2_ HUMAN | GO:0007144 GO:0007130 GO:0007131 GO:0006366 GO:1903506 GO:0003712 GO:0007141 GO:0006302 GO:0042802 GO:0007286 GO:0001673 GO:0001556 GO:0005524 |
| sp|Q15714|T22D1_ HUMAN | GO:0050794 |
| sp|Q15717|ELAV1_ HUMAN | GO:0005829 GO:0035925 GO:0003725 GO:0045727 GO:0070935 GO:0000166 GO:0019901 GO:0007275 GO:0005654 GO:2000036 GO:0016020 |
| sp|Q15738|NSDHL_ HUMAN | GO:0007224 GO:0016021 GO:0055114 GO:0060716 GO:0003854 GO:0001942 GO:0047012 GO:0005811 GO:0005789 GO:0006695 |
| sp|Q15785|TOM34_ HUMAN | GO:0016021 GO:0031072 GO:0006626 GO:0005741 GO:0005654 |
| sp|Q15814|TBCC_ HUMAN | GO:0032391 GO:0051087 GO:0000902 GO:0051084 GO:0015631 GO:0007023 GO:0044267 GO:0005829 GO:0003924 GO:0005874 |
| sp|Q15904|VAS1_ HUMAN | GO:0015991 GO:0016021 GO:0070374 GO:0045669 GO:0005774 GO:0045780 GO:0045921 GO:0033180 GO:0046961 GO:0045851 GO:0051656 GO:0007568 GO:0017137 GO:0033572 GO:0070062 GO:0070070 GO:0010008 GO:0008219 GO:0005524 GO:0046933 GO:2001206 GO:0008286 GO:0006879 |
| sp|Q15942|ZYX_ HUMAN | GO:0007267 GO:0046872 GO:0007229 GO:0005913 GO:0007160 GO:0043149 GO:0001725 GO:0005887 GO:0044822 GO:0005925 GO:0005515 GO:0007179 GO:0005737 GO:0005634 |
| sp|Q16186|ADRM1_ HUMAN | GO:0005654 GO:0005887 GO:0033081 GO:0070628 GO:0072520 GO:0061133 GO:0005737 GO:0007286 GO:0000502 GO:0048538 GO:0060009 GO:0042699 GO:0060612 GO:0002020 GO:0010950 GO:0048477 GO:0006368 GO:0043248 GO:0060399 |
| sp|Q16222|UAP1_ HUMAN | GO:0005829 GO:0018279 GO:0042802 GO:0043687 GO:0006048 GO:0003977 GO:0005654 GO:0006488 GO:0030246 GO:0005886 |
| sp|Q16513|PKN2_ HUMAN | GO:0070063 GO:0005654 GO:0042826 GO:0030027 GO:0010631 GO:0007155 GO:0032467 GO:0043296 GO:0044822 GO:0045931 GO:0006915 GO:0005829 GO:0045111 GO:0006355 GO:0018105 GO:0007264 GO:0043297 GO:0032154 GO:0005813 GO:0016021 GO:0045070 GO:0030496 GO:2000145 GO:0048471 GO:0005886 GO:0005524 GO:0004697 |
| sp|Q16531|DDB1_ HUMAN | GO:0046726 GO:0005654 GO:0016055 GO:0042769 GO:0005515 GO:1901990 GO:0003684 GO:0043066 GO:0000784 GO:0019985 GO:0042787 GO:0006283 GO:0005615 GO:0070911 GO:0005737 GO:1902188 GO:0043161 GO:0031464 GO:0033683 GO:0070914 GO:0045070 GO:0000715 GO:0031465 GO:0051702 GO:0070062 GO:0035518 |
| sp|Q16626|MEA1_ HUMAN | GO:0005737 GO:0007283 GO:0030154 GO:0005515 GO:0008584 |
| sp|Q16630|CPSF6_ HUMAN | GO:0042382 GO:0003729 GO:0006378 GO:0030529 GO:0005849 GO:0051262 GO:0005515 GO:0000166 GO:0016020 |
| sp|Q16637|SMN_ HUMAN | GO:0015030 GO:0006353 GO:0007409 GO:0097504 GO:0010975 GO:0034719 GO:0005829 GO:0032797 GO:0042802 GO:0000245 GO:0034660 GO:0007019 GO:0033120 GO:0043232 GO:0000387 |
| sp|Q16643|DREB_ HUMAN | GO:0060134 GO:0043198 GO:1904622 GO:0005921 GO:0090327 GO:0044308 GO:0005522 GO:0061003 GO:0014069 GO:0007420 GO:0014076 GO:0044295 GO:0030027 GO:0010644 GO:0005884 GO:0032232 GO:1902737 GO:0010643 GO:0098828 GO:0007568 GO:0005737 GO:2000463 GO:0014823 GO:0071481 GO:0045211 GO:0042641 GO:0051489 GO:0030833 GO:1900026 GO:0051015 GO:1904113 GO:0048286 GO:0032507 GO:0043025 GO:0048168 GO:1902897 GO:0043197 GO:0045773 GO:0032279 |
| sp|Q16740|CLPP_ HUMAN | GO:0042802 GO:0004252 GO:0009368 GO:0051260 GO:0005759 GO:0006515 |
| sp|Q16763|UBE2S_ HUMAN | GO:0070979 GO:0070534 GO:0010994 GO:0044314 GO:0005680 GO:0051301 GO:0005737 GO:0035519 GO:0061631 GO:0051488 GO:0031625 GO:0010458 GO:0031145 GO:0085020 GO:0005524 GO:0061630 |
| sp|Q16851|UGPA_ HUMAN | GO:0003983 GO:0032557 GO:0005829 GO:0052695 GO:0006805 GO:0046872 GO:0070062 GO:0042802 GO:0019255 GO:0005536 GO:0006011 GO:0006006 GO:0006065 GO:0005634 GO:0005978 |
| sp|Q16881|TRXR1_ HUMAN | GO:0005829 GO:0055114 GO:0098625 GO:0098869 GO:0098626 GO:0000302 GO:0006367 GO:0005739 GO:0005730 GO:0033554 GO:0045454 GO:0070062 GO:0009055 GO:0001887 GO:0005515 GO:0050660 GO:0007165 GO:0044255 GO:0005654 GO:0008283 GO:0001707 GO:0015949 GO:0004791 GO:0015035 |
| sp|Q1KMD3|HNRL2_ HUMAN | GO:0016020 GO:0044822 GO:0005654 GO:0005515 |
| sp|Q27J81|INF2_ HUMAN | GO:0003779 GO:0090140 GO:0030036 GO:0017048 GO:0032535 GO:0048471 |
| sp|Q2M2I8|AAK1_ HUMAN | GO:2000369 GO:0046777 GO:0045747 GO:0019897 GO:0004674 GO:0032880 GO:0035612 GO:0030136 GO:0031252 GO:0050821 GO:0005905 GO:0043195 GO:0005524 GO:0005112 |
| sp|Q2NL82|TSR1_ HUMAN | GO:0005730 GO:0042255 GO:0044822 |
| sp|Q2PZI1|D19L1_ HUMAN | GO:0019187 GO:0004583 GO:0042283 GO:0016021 GO:0000033 GO:0018717 GO:0004576 GO:0018718 GO:0016759 GO:0003980 GO:0052757 GO:0018715 GO:0008755 GO:0018716 GO:0018406 GO:0045140 GO:0008376 GO:0052639 GO:0052638 GO:0052640 GO:0052641 GO:0052824 GO:0080062 GO:0033556 GO:0046921 GO:0004376 GO:0008921 GO:0035496 GO:0000026 GO:0005637 GO:0046920 GO:0001962 GO:0031278 GO:0008375 GO:0042281 |
| sp|Q32P28|P3H1_ HUMAN | GO:0061077 GO:0050708 GO:0032991 GO:0019511 GO:0030278 GO:0005604 GO:0016020 GO:0060348 GO:0005788 GO:1901874 GO:0050821 GO:0008285 GO:0005506 GO:0030199 GO:0055114 GO:0016049 GO:0070062 GO:0031418 GO:0005518 GO:0032963 GO:0005634 GO:0019797 |
| sp|Q3ZAQ7|VMA21_ HUMAN | GO:0005764 GO:0043462 GO:0070072 GO:0030127 GO:0005789 |
| sp|Q4G0J3|LARP7_ HUMAN | GO:0005654 GO:0005794 GO:0005515 GO:0019013 GO:0030529 GO:0000166 GO:0006396 GO:0044822 |
| sp|Q52LJ0|FA98B_ HUMAN | GO:0005737 GO:0005654 GO:0072669 GO:0008033 GO:0044822 |
| sp|Q53EP0|FND3B_ HUMAN | GO:0016477 GO:0016021 GO:0048146 GO:0045600 GO:0045668 GO:0044822 GO:0034446 GO:0005515 GO:0060510 GO:0005783 |
| sp|Q53F19|NCBP3_ HUMAN | GO:0003729 GO:0000340 GO:0006370 GO:0000166 GO:0005737 GO:0051607 GO:0005634 GO:0051028 |
| sp|Q53FA7|QORX_ HUMAN | GO:0003960 GO:0006739 GO:0048038 GO:0070402 GO:0008270 GO:0070062 GO:0042803 GO:0055114 |
| sp|Q53FV1|ORML2_ HUMAN | GO:0016021 GO:0003407 GO:0006672 GO:0090155 GO:0005789 |
| sp|Q53GA4|PHLA2_ HUMAN | GO:0070873 GO:0006915 GO:0010468 GO:0045995 GO:0001890 GO:0009887 GO:0030334 GO:0005737 GO:0016020 |
| sp|Q56VL3|OCAD2_ HUMAN | GO:0005743 GO:0005768 |
| sp|Q5BKZ1|ZN326_ HUMAN | GO:0045893 GO:0016363 GO:0005681 GO:0008270 GO:0000993 GO:0008380 GO:0044609 GO:0003677 GO:0044822 GO:0032784 GO:0006397 GO:0005654 |
| sp|Q5C9Z4|NOM1_ HUMAN | GO:0005730 GO:0005515 GO:0042274 GO:0022008 GO:0044822 |
| sp|Q5F1R6|DJC21_ HUMAN | GO:0006457 GO:0005840 GO:0044822 GO:0008270 GO:0005515 |
| sp|Q5HYI7|MTX3_ HUMAN | GO:0006626 GO:0005741 |
| sp|Q5JRA6|MIA3_ HUMAN | GO:0030336 GO:0005515 GO:0002063 GO:0007162 GO:0015031 GO:0005789 GO:0006887 GO:0002687 GO:0061024 GO:0030501 GO:0042060 GO:0030199 GO:0006888 GO:0016021 GO:0043687 GO:0018279 |
| sp|Q5JRX3|PREP_ HUMAN | GO:0008047 GO:0004222 GO:0016485 GO:0005759 GO:0043085 GO:0008270 |
| sp|Q5JTV8|TOIP1_ HUMAN | GO:0008092 GO:0071763 GO:0005521 GO:0016021 GO:0034504 GO:0032781 GO:0001671 GO:0051117 GO:0005637 |
| sp|Q5JTZ9|SYAM_ HUMAN | GO:0005829 GO:0005739 GO:0004813 GO:0046872 GO:0070143 GO:0005524 GO:0033108 GO:0016597 GO:0000049 GO:0006400 |
| sp|Q5K4L6|S27A3_ HUMAN | GO:0034943 GO:0052686 GO:0004774 GO:0034842 GO:0034841 GO:0010435 GO:0052685 GO:0016021 GO:0015908 GO:0018855 GO:0070251 GO:0034793 GO:0031966 GO:0018854 GO:0043762 GO:0006637 GO:0004321 GO:0034823 GO:0034865 GO:0018857 GO:0043955 GO:0034796 GO:0018856 GO:0034942 GO:0000166 GO:0004467 GO:0001676 GO:0090409 GO:0003996 GO:0052688 GO:0034783 GO:0031957 GO:0052687 GO:0043759 |
| sp|Q5QJE6|TDIF2_ HUMAN | GO:0005730 GO:0006355 GO:0044822 |
| sp|Q5RKV6|EXOS6_ HUMAN | GO:0045006 GO:0000176 GO:0045830 GO:0044822 GO:0071051 GO:0071028 GO:0043928 GO:0043488 GO:0005730 GO:0000177 GO:0005829 GO:0034475 GO:0004532 GO:0034427 GO:0031125 GO:0016075 |
| sp|Q5SRD1|TI23B_ HUMAN | GO:0031305 GO:0044267 GO:0015450 GO:0030150 GO:0005744 GO:0015266 GO:0019899 GO:0005758 |
| sp|Q5SRE5|NU188_ HUMAN | GO:0031047 GO:0007077 GO:0051028 GO:0016020 GO:1900034 GO:0044611 GO:0010827 GO:0019221 GO:0005975 GO:0008033 GO:0044281 GO:0019083 GO:0016925 GO:0000088 GO:0055085 GO:0017056 GO:0043687 GO:0019054 GO:0006606 GO:0006405 |
| sp|Q5SSJ5|HP1B3_ HUMAN | GO:0071456 GO:0000786 GO:0070828 GO:0006355 GO:0003677 GO:0097298 GO:0031491 GO:0042127 GO:0006334 GO:0005634 |
| sp|Q5SY16|NOL9_ HUMAN | GO:0005515 GO:0016020 GO:0052671 GO:0052668 GO:0016310 GO:0042556 GO:0005730 GO:0052813 GO:0000448 GO:0051731 GO:0003723 GO:0016307 GO:0019200 GO:0005524 |
| sp|Q5T280|CI114_ HUMAN | GO:0034933 GO:0052666 GO:0043803 GO:0009008 GO:0043851 GO:0018707 GO:0018423 GO:0008172 GO:0016435 GO:0016428 GO:0007049 GO:0071424 GO:0034931 GO:0016205 GO:0009019 GO:0016279 GO:0043834 GO:0043776 GO:0052624 GO:0043777 GO:0034807 GO:0052667 GO:0032259 GO:0043833 GO:0051301 GO:0043780 GO:0009383 GO:0008988 GO:0008174 GO:0030792 GO:0043827 GO:0051994 GO:0043791 GO:0004809 GO:0044822 GO:0000179 GO:0052735 GO:0034541 GO:0043852 GO:0052665 GO:0043770 GO:0043782 GO:0019702 GO:0000777 GO:0008425 GO:0080012 GO:0008650 GO:0070677 |
| sp|Q5T2E6|CJ076_ HUMAN | GO:0016021 |
| sp|Q5T3I0|GPTC4_ HUMAN | GO:0044822 GO:0002244 GO:0005515 |
| sp|Q5T4S7|UBR4_ HUMAN | GO:0016021 GO:0005516 GO:0008270 GO:0070740 GO:0043773 GO:0043774 GO:0008766 GO:0070736 GO:0005813 GO:0004842 GO:0042787 GO:0070738 GO:0018169 GO:0016032 GO:0005654 GO:0005737 GO:0070737 |
| sp|Q5T5X7|BEND3_ HUMAN | GO:0043967 GO:0098532 GO:0000122 GO:0005515 GO:0000183 GO:0034773 GO:0005730 GO:0006306 GO:0005720 GO:0080182 GO:0000182 GO:0036124 |
| sp|Q5T653|RM02_ HUMAN | GO:0070126 GO:0005762 GO:0070125 GO:0005743 GO:0003735 GO:0002181 GO:0016740 GO:0044822 GO:0070124 |
| sp|Q5TDH0|DDI2_ HUMAN | GO:0005654 GO:0005737 GO:0006508 GO:0004190 |
| sp|Q5TFE4|NT5D1_ HUMAN | GO:0052867 GO:0008253 GO:0046872 GO:0052830 GO:0052825 GO:1990003 GO:0043812 GO:0030352 GO:0016311 GO:0030351 GO:0019178 GO:0008579 GO:0019198 GO:0052828 GO:0043726 GO:0052829 GO:0008330 GO:0017017 GO:0008969 GO:0030487 GO:0017161 GO:0016021 GO:0052629 GO:0052831 GO:0043813 |
| sp|Q5VT66|MARC1_ HUMAN | GO:0030170 GO:0005743 GO:0042126 GO:0030151 GO:0051410 GO:0016021 GO:0005741 GO:0008940 GO:0043546 GO:0055114 |
| sp|Q5VTR2|BRE1A_ HUMAN | GO:0000209 GO:0045893 GO:0002039 GO:0005654 GO:0030336 GO:0010390 GO:0042393 GO:0008270 GO:0003730 GO:0016874 GO:0004842 GO:2001168 GO:0031062 GO:1900364 GO:0005730 GO:0006511 GO:0033503 GO:0031625 GO:0003713 GO:0003682 GO:0007346 |
| sp|Q5VW32|BROX_ HUMAN | GO:0070062 GO:0016020 |
| sp|Q5W0Z9|ZDH20_ HUMAN | GO:0019706 GO:0005886 GO:0016021 GO:0018345 GO:0008270 |
| sp|Q643R3|LPCT4_ HUMAN | GO:0016021 GO:0036148 GO:0036150 GO:0047184 GO:0071617 GO:0047192 GO:0019432 GO:0006654 GO:0047166 GO:0036151 GO:0005789 GO:0036152 |
| sp|Q68CZ2|TENS3_ HUMAN | GO:0048286 GO:0005737 GO:0046872 GO:0035556 GO:0008284 GO:0016477 GO:0005515 GO:0005925 |
| sp|Q6DD88|ATLA3_ HUMAN | GO:0016021 GO:0007029 GO:0051260 GO:0042802 GO:0007030 GO:0006888 GO:0003924 GO:0008152 GO:0005789 GO:0005525 |
| sp|Q6DKJ4|NXN_ HUMAN | GO:0005829 GO:0055114 GO:0047134 GO:0098869 GO:0072358 GO:0030178 GO:0030154 GO:0005515 GO:0031397 GO:0004791 GO:0005634 |
| sp|Q6EEV4|GL1AD_ HUMAN | GO:0016591 GO:0005635 |
| sp|Q6FI81|CPIN1_ HUMAN | GO:0051537 GO:0005730 GO:0016226 GO:0046872 GO:0030097 GO:0009055 GO:0032259 GO:0005758 GO:0005515 GO:0005654 GO:0044281 GO:0008168 GO:0043066 |
| sp|Q6IA17|SIGIR_ HUMAN | GO:0001960 GO:0005515 GO:0031665 GO:0043433 GO:0006953 GO:0016021 GO:0045079 |
| sp|Q6IA69|NADE_ HUMAN | GO:0005829 GO:0009435 GO:0004359 GO:0003952 GO:0005524 GO:0005515 GO:0006767 GO:0008795 |
| sp|Q6IAA8|LTOR1_ HUMAN | GO:0006367 GO:0034613 GO:0008286 GO:0016236 GO:0007032 GO:0010872 GO:0007040 GO:0005765 GO:0005794 GO:0071986 GO:0007050 GO:0043009 GO:0032008 GO:0060620 GO:0033554 GO:0016049 GO:0070062 GO:0032439 GO:0010874 GO:0043547 GO:0043410 GO:0005886 GO:0032418 GO:0071230 GO:0001919 GO:0042632 GO:0005085 GO:0032947 |
| sp|Q6IBS0|TWF2_ HUMAN | GO:0005080 GO:0071363 GO:0005546 GO:0030027 GO:0042989 GO:0030426 GO:0044822 GO:0030175 GO:0032532 GO:0030016 GO:0071300 GO:0045773 GO:0005856 GO:0003785 GO:0032420 GO:0010592 GO:0048471 GO:0070062 GO:0005524 GO:0051016 GO:0035556 |
| sp|Q6IN84|MRM1_ HUMAN | GO:0070039 GO:0005515 GO:0000453 GO:0005739 GO:0044822 |
| sp|Q6IPR3|TYW3_ HUMAN | GO:0006400 GO:0032259 GO:0008168 |
| sp|Q6KB66|K2C80_ HUMAN | GO:0005737 GO:0030057 GO:0005198 GO:0005515 GO:0045095 |
| sp|Q6NUM9|RETST_ HUMAN | GO:0005789 GO:0051786 GO:0005640 GO:0042572 GO:0055114 |
| sp|Q6NZY4|ZCHC8_ HUMAN | GO:0005654 GO:0000398 GO:0005515 GO:0008270 GO:0071013 GO:0044822 |
| sp|Q6P161|RM54_ HUMAN | GO:0070126 GO:0005840 GO:0070125 GO:0005743 GO:0044822 GO:0070124 |
| sp|Q6P1J9|CDC73_ HUMAN | GO:0031442 GO:0050680 GO:0051301 GO:0007507 GO:0045638 GO:0030177 GO:0019827 GO:0001711 GO:0000784 GO:0016593 GO:0032968 GO:2000134 GO:0071222 GO:0005737 GO:0010390 GO:0031648 GO:0021529 GO:0000122 GO:0000993 GO:0006378 GO:0030218 GO:0048147 GO:0033523 |
| sp|Q6P1N0|C2D1A_ HUMAN | GO:0004871 GO:0000122 GO:0016020 GO:0005815 GO:0000978 GO:0043123 GO:0005737 GO:0001078 GO:0070062 GO:0005634 |
| sp|Q6P1R4|DUS1L_ HUMAN | GO:0034912 GO:0016631 GO:0050660 GO:0052850 GO:0002943 GO:0034809 GO:0052849 GO:0018503 GO:0034838 GO:0018499 GO:0034790 GO:0034805 GO:0018500 GO:0043786 GO:0005829 GO:0018501 GO:0017150 GO:0034824 GO:0018502 GO:0055114 GO:0018498 |
| sp|Q6P2E9|EDC4_ HUMAN | GO:0005654 GO:0043928 GO:0005515 GO:0010467 GO:0000932 GO:0016020 GO:0005829 |
| sp|Q6P2Q9|PRP8_ HUMAN | GO:0000386 GO:0030619 GO:0070530 GO:0030623 GO:0097157 GO:0016607 GO:0071356 GO:0000244 GO:0044822 GO:0030620 GO:0017070 GO:0005682 GO:0071013 GO:0071222 GO:0016020 |
| sp|Q6P587|FAHD1_ HUMAN | GO:0047621 GO:0046872 GO:0008948 GO:0018773 GO:0005743 GO:0005654 GO:0034545 GO:0008152 GO:0005829 |
| sp|Q6P996|PDXD1_ HUMAN | GO:0030170 GO:0005794 GO:0016831 GO:0019752 |
| sp|Q6P9B9|INT5_ HUMAN | GO:0005515 GO:0016180 GO:0016021 GO:0032039 |
| sp|Q6PGP7|TTC37_ HUMAN | GO:0035327 GO:0005654 GO:0055087 GO:0005515 |
| sp|Q6PJG6|BRAT1_ HUMAN | GO:0016477 GO:0016049 GO:0010212 GO:0006915 GO:0005515 GO:0051646 GO:0006006 GO:0006974 GO:0001934 GO:0008283 GO:0005737 GO:0016020 GO:0005634 |
| sp|Q6PJT7|ZC3HE_ HUMAN | GO:0005730 GO:0046872 GO:0016607 GO:0008143 GO:0044822 GO:0043488 GO:0005515 GO:1900364 GO:0005737 |
| sp|Q6PKG0|LARP1_ HUMAN | GO:0048027 GO:0006413 GO:0005654 GO:0016020 GO:0003730 GO:0016239 GO:0008283 GO:0045727 GO:0005737 GO:0008190 GO:0045070 GO:0000339 GO:0008494 GO:0031929 GO:0031931 |
| sp|Q6QNY0|BL1S3_ HUMAN | GO:0048490 GO:0032402 GO:0030133 GO:0030168 GO:0005515 GO:0060155 GO:0032438 GO:0031175 GO:0035646 GO:0008320 GO:0033299 GO:0071806 GO:0031083 GO:0001654 GO:0030424 GO:0042493 GO:0032816 |
| sp|Q6RFH5|WDR74_ HUMAN | GO:0005730 GO:0001825 GO:0005515 GO:0016070 |
| sp|Q6ULP2|AFTIN_ HUMAN | GO:0005829 GO:0030276 GO:0046907 GO:0005654 GO:0030121 GO:0015031 |
| sp|Q6UN15|FIP1_ HUMAN | GO:0006369 GO:0000398 GO:0031124 GO:0044822 GO:0005515 GO:0006406 GO:0005847 |
| sp|Q6UWP7|LCLT1_ HUMAN | GO:0016024 GO:0034851 GO:0043741 GO:0034915 GO:0016021 GO:0004147 GO:0018031 GO:0016412 GO:0008951 GO:0018030 GO:0052858 GO:0016749 GO:0043806 GO:0034738 GO:0016419 GO:0034945 GO:0016753 GO:0035965 GO:0034919 GO:0016414 GO:0032216 GO:0019705 GO:0016750 GO:0034737 GO:0016418 GO:0016751 GO:0043849 GO:0005515 GO:0003841 GO:0019432 GO:0016454 GO:0090595 GO:0006654 GO:0016406 GO:0016413 GO:0046941 GO:0018713 GO:0043764 GO:0018712 GO:0034848 GO:0007275 GO:0016416 GO:0044281 GO:0005789 GO:0018711 GO:0019186 |
| sp|Q6UX04|CWC27_ HUMAN | GO:0006457 GO:0000398 GO:0000413 GO:0003755 GO:0071013 |
| sp|Q6UXV4|MIC27_ HUMAN | GO:0005515 GO:0061617 |
| sp|Q6XQN6|PNCB_ HUMAN | GO:0006979 GO:0005829 GO:0004514 GO:0009435 GO:0006769 GO:0019358 GO:0070062 GO:0016874 GO:0004516 GO:0005515 GO:0005654 GO:0005794 |
| sp|Q6YN16|HSDL2_ HUMAN | GO:0016020 GO:0005777 GO:0016491 GO:0005739 GO:0055114 |
| sp|Q6ZRV2|FA83H_ HUMAN | GO:0044380 GO:1990254 GO:0030335 GO:0045095 GO:0045104 GO:0019901 |
| sp|Q712K3|UB2R2_ HUMAN | GO:0090261 GO:0043951 GO:0005654 GO:0070936 GO:0070848 GO:0006513 GO:0016874 GO:0051301 GO:0005737 GO:0035458 GO:0043161 GO:0061631 GO:0031625 GO:0043525 GO:0005524 GO:0061630 GO:0007049 |
| sp|Q71DI3|H32_ HUMAN | GO:0031047 GO:0002230 GO:0007186 GO:0030246 GO:0005654 GO:0042393 GO:0045815 GO:0000183 GO:0016020 GO:0000784 GO:0004930 GO:0051290 GO:0044267 GO:0006335 GO:0032776 GO:0046982 GO:0060968 GO:0098792 GO:0007264 GO:0000788 GO:0007596 GO:0003677 GO:0009790 GO:0033554 GO:0070062 |
| sp|Q71RC2|LARP4_ HUMAN | GO:0022604 GO:0005515 GO:0007010 GO:0016020 GO:0000166 GO:0044822 |
| sp|Q71UI9|H2AV_ HUMAN | GO:0000980 GO:0007275 GO:0032869 GO:0031492 GO:0000979 GO:0005719 GO:0000978 GO:0006342 GO:0000786 GO:0046982 GO:0005720 GO:0045944 GO:0001740 GO:0070062 GO:0071392 |
| sp|Q75N03|HAKAI_ HUMAN | GO:0045807 GO:0042802 GO:0007162 GO:0061630 GO:0016337 GO:0016567 GO:0030335 GO:0070735 GO:0070739 GO:0000151 GO:0005634 |
| sp|Q7KZ85|SPT6H_ HUMAN | GO:0042393 GO:0061086 GO:0032968 GO:0044822 GO:0050684 GO:0051147 GO:0008380 GO:0003677 GO:0006338 GO:0045191 GO:0010793 GO:0016032 GO:0005634 GO:0003700 |
| sp|Q7L2H7|EIF3M_ HUMAN | GO:0003743 GO:0001731 GO:0005829 GO:0016282 GO:0071541 GO:0002183 GO:0033290 GO:0006446 GO:0016787 GO:0031369 |
| sp|Q7L3T8|SYPM_ HUMAN | GO:0004827 GO:0005524 GO:0005759 GO:0006433 |
| sp|Q7L4I2|RSRC2_ HUMAN | GO:0005515 GO:0044822 |
| sp|Q7L5A8|FA2H_ HUMAN | GO:0006810 GO:0016021 GO:0006633 GO:0055114 GO:0043738 GO:0043883 GO:0080132 GO:0043914 GO:0042634 GO:0030148 GO:0043826 GO:0008748 GO:0042127 GO:0020037 GO:0032286 GO:0032287 GO:0030258 GO:0005789 GO:0001949 GO:0005506 GO:0052693 |
| sp|Q7LBR1|CHM1B_ HUMAN | GO:0030117 GO:0039702 GO:0007034 GO:0007080 GO:1901673 GO:0000920 GO:0000815 GO:0036258 GO:0015031 GO:1904903 GO:0005829 GO:0031902 GO:0019904 GO:0006997 GO:0070062 GO:0010824 |
| sp|Q7Z2K6|ERMP1_ HUMAN | GO:0005789 GO:0001541 GO:0046872 GO:0016021 GO:0006508 GO:0008237 GO:0008234 |
| sp|Q7Z2W4|ZCCHV_ HUMAN | GO:0005515 GO:0061014 GO:0044822 GO:0039507 GO:1900246 GO:0005764 GO:0050691 GO:0043123 GO:0005794 GO:0005770 GO:0003950 GO:0032728 GO:0046872 GO:0071360 GO:0032727 GO:0045071 GO:0005634 |
| sp|Q7Z2W9|RM21_ HUMAN | GO:0070126 GO:0005762 GO:0070125 GO:0005743 GO:0003735 GO:0044822 GO:0070124 |
| sp|Q7Z3K3|POGZ_ HUMAN | GO:0051382 GO:0000790 GO:0007064 GO:0046872 GO:0051301 GO:0003677 GO:0005515 GO:0005654 GO:0005737 |
| sp|Q7Z406|MYH14_ HUMAN | GO:0070584 GO:0007411 GO:0007605 GO:0016020 GO:0030426 GO:0030048 GO:0030898 GO:0016460 GO:0097513 GO:0003009 GO:0071625 GO:0048013 GO:0043209 GO:0005829 GO:0000146 GO:0007264 GO:0007519 GO:0030424 GO:0005903 GO:0051015 GO:0001725 GO:0070062 GO:0019228 GO:0005524 GO:0031032 |
| sp|Q7Z434|MAVS_ HUMAN | GO:0004871 GO:0016021 GO:1900063 GO:0032728 GO:0042993 GO:0032727 GO:0005778 GO:0042742 GO:0039529 GO:0043123 GO:0001934 GO:0045944 GO:0071660 GO:0051092 GO:0032480 GO:0019901 GO:0045071 GO:0060340 GO:0071360 GO:0002230 GO:0005741 GO:0071651 GO:0032760 GO:0032757 GO:0050700 GO:0033160 |
| sp|Q7Z6I8|CE024_ HUMAN | GO:0005515 |
| sp|Q7Z7H5|TMED4_ HUMAN | GO:0016021 GO:0004871 GO:0043123 GO:0070062 GO:0005789 GO:0015031 |
| sp|Q7Z7K6|CENPV_ HUMAN | GO:0001667 GO:0007067 GO:0033044 GO:0032467 GO:0008152 GO:0031508 GO:0000777 GO:0005654 GO:0005737 GO:0051233 GO:0016846 |
| sp|Q7Z7N9|T179B_ HUMAN | GO:0016020 |
| sp|Q86TB9|PATL1_ HUMAN | GO:0017148 GO:0005515 GO:0016607 GO:0016605 GO:0044822 GO:0034046 GO:0030371 GO:0043928 GO:0030014 GO:0005829 GO:0000932 GO:0000290 GO:0002151 GO:0008266 GO:0033962 |
| sp|Q86TJ2|TAD2B_ HUMAN | GO:0005515 GO:0008270 GO:0004402 GO:0006357 GO:0003713 GO:0003677 GO:0006338 GO:0003682 GO:0030914 GO:0035066 GO:0003700 |
| sp|Q86U28|ISCA2_ HUMAN | GO:0006790 GO:0097428 GO:0008198 GO:0016226 GO:0005515 GO:0005759 GO:0051537 GO:0051539 GO:0005198 |
| sp|Q86U42|PABP2_ HUMAN | GO:0016973 GO:0005654 GO:0019058 GO:0000166 GO:0046778 GO:0044822 GO:0043621 GO:0042405 GO:0006936 GO:0005737 GO:0006369 GO:0008143 GO:0030529 GO:0000398 GO:0006378 |
| sp|Q86VM9|ZCH18_ HUMAN | GO:0046872 GO:0044822 GO:0005654 GO:0005515 |
| sp|Q86VN1|VPS36_ HUMAN | GO:0032266 GO:0000814 GO:0008022 GO:0003730 GO:0036258 GO:0005764 GO:0043130 GO:0005829 GO:0061024 GO:0006355 GO:0031902 GO:0006914 GO:0070062 GO:0005634 GO:0043328 |
| sp|Q86VQ0|LCA5_ HUMAN | GO:0032403 GO:0045494 GO:0042073 GO:0036064 GO:0005930 |
| sp|Q86VR2|F134C_ HUMAN | GO:0005515 GO:0010976 GO:0016021 |
| sp|Q86VR7|VS10L_ HUMAN | GO:0016021 |
| sp|Q86W42|THOC6_ HUMAN | GO:0007417 GO:0003723 GO:0016607 GO:0008380 GO:0046784 GO:0000445 GO:0005515 GO:0000784 GO:0006397 GO:0043066 |
| sp|Q86W92|LIPB1_ HUMAN | GO:0007155 GO:0005886 GO:0005925 |
| sp|Q86X55|CARM1_ HUMAN | GO:2000171 GO:0042803 GO:0016021 GO:0008284 GO:0032091 GO:0005829 GO:0005654 GO:0070577 GO:0008380 GO:0008013 GO:0044255 GO:0044212 GO:0044281 GO:0007568 GO:0019919 GO:0016032 GO:0034970 GO:0034971 GO:0030374 GO:0003420 GO:0035242 GO:1902415 GO:0090575 GO:0051591 GO:0035642 GO:0048742 GO:0045600 GO:0006397 GO:0006355 GO:0033146 |
| sp|Q86XP3|DDX42_ HUMAN | GO:0010501 GO:0015030 GO:0004004 GO:0016607 GO:0005524 GO:0044822 GO:0005515 GO:0005737 GO:0008104 GO:0016020 |
| sp|Q86Y56|DAAF5_ HUMAN | GO:0005737 GO:0036159 GO:0045505 GO:0036158 GO:0003341 GO:0031514 |
| sp|Q86YV9|HPS6_ HUMAN | GO:0072657 GO:0031901 GO:0006996 GO:0017137 GO:0030318 GO:0030742 GO:0031084 GO:0005783 GO:0007596 |
| sp|Q8IUF8|MINA_ HUMAN | GO:0005829 GO:0055114 GO:0005730 GO:0046872 GO:0005667 GO:0051213 GO:0000122 GO:0042254 GO:0001191 GO:0008283 |
| sp|Q8IVD9|NUDC3_ HUMAN | GO:0005515 |
| sp|Q8IVM0|CCD50_ HUMAN | GO:0005737 GO:0005874 GO:0031625 GO:0007605 |
| sp|Q8IVS2|FABD_ HUMAN | GO:0006633 GO:0004314 GO:0005739 GO:0044822 |
| sp|Q8IVT2|MISP_ HUMAN | GO:0005856 GO:0007067 GO:0003779 GO:0005886 GO:0005925 GO:0043231 GO:0005938 GO:0051301 |
| sp|Q8IW45|NNRD_ HUMAN | GO:0005515 GO:0052855 GO:0005524 GO:0008152 GO:0005829 GO:0005783 GO:0005739 GO:0047453 |
| sp|Q8IWA0|WDR75_ HUMAN | GO:0006357 GO:0003712 GO:1903146 GO:0044822 GO:0000124 GO:0005730 GO:1903955 GO:0016573 |
| sp|Q8IWV7|UBR1_ HUMAN | GO:0005829 GO:0000502 GO:0008270 GO:0032007 GO:0070728 GO:0016874 GO:0061630 GO:0071233 GO:0016567 GO:0071596 GO:0000151 |
| sp|Q8IX12|CCAR1_ HUMAN | GO:0005654 GO:0003714 GO:0005515 GO:0006351 GO:0044822 GO:0005641 GO:0001047 GO:0005737 GO:0008284 GO:0043065 GO:0000398 GO:0030374 GO:0030335 GO:1903507 |
| sp|Q8IXB1|DJC10_ HUMAN | GO:0015035 GO:0034975 GO:0051087 GO:0030544 GO:0005788 GO:0001933 GO:0045454 GO:0001671 GO:0051117 GO:0016021 GO:0016671 GO:0034663 GO:0051787 GO:0032781 GO:0055114 GO:0070059 GO:0030433 |
| sp|Q8IXI1|MIRO2_ HUMAN | GO:0005515 GO:0031307 GO:0097345 GO:0051056 GO:0005525 GO:0005509 GO:0005743 GO:0005829 GO:0019725 GO:0047497 GO:0070062 GO:0003924 GO:0005886 GO:0008152 |
| sp|Q8IXM3|RM41_ HUMAN | GO:0070126 GO:0005762 GO:0070125 GO:0007049 GO:0005743 GO:0006915 GO:0003735 GO:0044822 GO:0005515 GO:0070124 |
| sp|Q8IY37|DHX37_ HUMAN | GO:0006396 GO:0004004 GO:0005730 GO:0005524 GO:0044822 |
| sp|Q8IY95|TM192_ HUMAN | GO:0005654 GO:0005794 GO:0005770 GO:0005886 GO:0005765 GO:0016021 GO:0005783 GO:0070062 GO:0042803 GO:0048471 |
| sp|Q8IYB8|SUV3_ HUMAN | GO:0070584 GO:0030307 GO:0000962 GO:0032508 GO:0043066 GO:0006310 GO:0044822 GO:0045025 GO:0000965 GO:0042645 GO:0003677 GO:0035946 GO:0035945 GO:0070827 GO:0003725 GO:0042803 GO:0000958 GO:0004004 GO:0005634 GO:0005524 GO:0034458 GO:0003678 |
| sp|Q8IYL3|CA174_ HUMAN | GO:0005634 GO:0005515 |
| sp|Q8IYS1|P20D2_ HUMAN | GO:0070062 GO:0005654 GO:0008152 GO:0016787 |
| sp|Q8IYS2|K2013_ HUMAN | GO:0016021 |
| sp|Q8IZW8|TENS4_ HUMAN | GO:0008104 GO:0044424 GO:0005925 |
| sp|Q8N1F7|NUP93_ HUMAN | GO:0051292 GO:0031047 GO:0002230 GO:0016973 GO:0007077 GO:1900034 GO:0034399 GO:0031965 GO:0010827 GO:0019221 GO:0005643 GO:0098792 GO:0005975 GO:0008033 GO:0044281 GO:0019083 GO:0016925 GO:0000088 GO:0055085 GO:0017056 GO:0043687 GO:0019054 GO:0098779 GO:0006606 |
| sp|Q8N257|H2B3B_ HUMAN | GO:0019731 GO:0070062 GO:0005615 GO:0000788 GO:0002227 GO:0003677 GO:0006334 GO:0005654 GO:0005737 GO:0050830 |
| sp|Q8N2Z9|CENPS_ HUMAN | GO:0007067 GO:0000236 GO:0036297 GO:0031398 GO:0000712 GO:0000777 GO:0051301 GO:0071821 GO:0031297 GO:0005576 GO:0005829 GO:0046982 GO:0007264 GO:0005179 GO:0043240 GO:0000090 GO:0003690 GO:0003682 GO:0034080 |
| sp|Q8N3C0|ASCC3_ HUMAN | GO:0032508 GO:0006307 GO:0006355 GO:0005524 GO:0044822 GO:0005515 GO:0043140 GO:0005654 GO:0008283 GO:0016020 GO:0005794 |
| sp|Q8N3D4|EH1L1_ HUMAN | GO:0016020 |
| sp|Q8N4A0|GALT4_ HUMAN | GO:0016021 GO:0000139 GO:0046872 GO:0004653 GO:0070062 GO:0043687 GO:0048471 GO:0030246 GO:0016266 |
| sp|Q8N4V1|MMGT1_ HUMAN | GO:0031901 GO:1903874 GO:0015693 GO:0006825 GO:0006824 GO:0015095 GO:0000139 GO:0015087 GO:0015093 GO:0072546 GO:1903830 GO:0005886 |
| sp|Q8N573|OXR1_ HUMAN | GO:0055114 GO:0005739 GO:0005730 GO:0016491 GO:0071447 GO:0005515 GO:0043524 GO:0007628 |
| sp|Q8N5I2|ARRD1_ HUMAN | GO:0070062 GO:0031410 GO:0005886 |
| sp|Q8N5K1|CISD2_ HUMAN | GO:0010506 GO:0016021 GO:0043234 GO:0051537 GO:0046872 GO:0010259 GO:0005741 GO:0044822 GO:0048471 GO:0042803 GO:0005789 GO:0000422 |
| sp|Q8N684|CPSF7_ HUMAN | GO:0006369 GO:0000398 GO:0031124 GO:0005849 GO:0044822 GO:0051262 GO:0005515 GO:0016020 |
| sp|Q8N6N7|ACBD7_ HUMAN | GO:0005783 GO:0000062 GO:0005794 GO:0006810 GO:0008289 |
| sp|Q8N766|EMC1_ HUMAN | GO:0072546 GO:0034975 |
| sp|Q8NAV1|PR38A_ HUMAN | GO:0031965 GO:0008380 GO:0005515 GO:0071011 GO:0006397 GO:0044822 |
| sp|Q8NBF2|NHLC2_ HUMAN | GO:0005515 |
| sp|Q8NBJ5|GT251_ HUMAN | GO:0005788 GO:0050211 GO:0008152 GO:0016020 GO:0030198 |
| sp|Q8NBS9|TXND5_ HUMAN | GO:0003756 GO:0034976 GO:0006457 GO:0006892 GO:0045454 GO:0061024 GO:0070062 GO:0043202 GO:0005788 GO:0005515 GO:0008152 GO:0043066 GO:0043277 |
| sp|Q8NBX0|SCPDL_ HUMAN | GO:0043826 GO:0008748 GO:0043883 GO:0043738 GO:0043914 GO:0005811 GO:0030496 GO:0016021 GO:0005634 GO:0052693 GO:0005739 GO:0055114 |
| sp|Q8ND56|LS14A_ HUMAN | GO:0043231 GO:0060340 GO:0044822 GO:0003727 GO:0010494 GO:0000932 GO:0003690 GO:0003725 GO:0039529 GO:0033962 |
| sp|Q8NDH3|PEPL1_ HUMAN | GO:0005737 GO:0006508 GO:0005634 GO:0030145 GO:0008235 GO:0005515 GO:0004177 |
| sp|Q8NE86|MCU_ HUMAN | GO:0031305 GO:0060027 GO:0005262 GO:0008016 GO:0036444 GO:0051561 GO:0035786 GO:0043009 GO:0007015 GO:1990246 GO:0042802 GO:0019722 GO:0015292 GO:0032024 GO:0042593 |
| sp|Q8NEN9|PDZD8_ HUMAN | GO:0046872 GO:0035556 GO:0022604 GO:0005622 GO:0007010 GO:0016032 GO:0016020 |
| sp|Q8NEV1|CSK23_ HUMAN | GO:0004674 GO:0000236 GO:0008284 GO:0047485 GO:0071174 GO:0007411 GO:0000087 GO:0030177 GO:0005956 GO:0005829 GO:0016581 GO:0061077 GO:0008013 GO:0031519 GO:0051879 GO:0019888 GO:0005886 GO:0030307 GO:0005524 GO:0048511 GO:0043154 GO:0046777 GO:0016580 GO:0006355 GO:0045732 |
| sp|Q8NEW0|ZNT7_ HUMAN | GO:0005385 GO:0071577 GO:0016021 GO:0044267 GO:0008270 GO:0061088 GO:0016023 GO:0070062 GO:0048471 GO:0010043 GO:0005794 |
| sp|Q8NF37|PCAT1_ HUMAN | GO:0043129 GO:0000139 GO:2001246 GO:0047191 GO:0005509 GO:0005789 GO:0036148 GO:0045732 GO:0019432 GO:0060041 GO:0036151 GO:0047159 GO:0047184 GO:0016021 GO:0006654 GO:0005811 GO:0047192 |
| sp|Q8NFH4|NUP37_ HUMAN | GO:0031047 GO:0005654 GO:0007077 GO:0051028 GO:0007067 GO:0005515 GO:1900034 GO:0000236 GO:0010827 GO:0000777 GO:0015031 GO:0051301 GO:0005829 GO:0019221 GO:0005975 GO:0007264 GO:0008033 GO:0007059 GO:0044281 GO:0000090 GO:0019083 GO:0016925 GO:0000088 GO:0055085 GO:0031080 GO:0043687 GO:0019054 |
| sp|Q8NFU3|TSTD1_ HUMAN | GO:0048471 GO:0005739 |
| sp|Q8NFV4|ABHDB_ HUMAN | GO:0008152 GO:0016787 GO:0005739 |
| sp|Q8NFW8|NEUA_ HUMAN | GO:0005829 GO:0090633 GO:0018279 GO:0008781 GO:0006055 GO:0006054 GO:0043687 GO:0090632 GO:0005654 GO:0006488 GO:0016020 |
| sp|Q8NHG7|SVIP_ HUMAN | GO:0036513 GO:0010508 GO:0043621 GO:1903061 GO:0051117 GO:0031333 GO:1904153 GO:1903070 GO:0070062 GO:0031225 |
| sp|Q8NI27|THOC2_ HUMAN | GO:0016973 GO:0017145 GO:0005515 GO:0016607 GO:0000784 GO:0006397 GO:0000902 GO:0001824 GO:0046784 GO:0008380 GO:0000445 GO:0010793 GO:0010977 GO:0003729 |
| sp|Q8NI37|PPTC7_ HUMAN | GO:0046872 GO:0004721 GO:0006470 GO:0005739 |
| sp|Q8TAE8|G45IP_ HUMAN | GO:0005515 GO:1903862 GO:0071850 GO:0005761 GO:0008284 GO:0070126 GO:0070124 GO:0016032 GO:0005634 GO:0070125 |
| sp|Q8TB96|TIP_ HUMAN | GO:0005886 GO:0007229 GO:0005515 GO:0016021 GO:0070062 |
| sp|Q8TC07|TBC15_ HUMAN | GO:0005739 GO:0070062 GO:1902017 GO:0005515 GO:0043547 GO:0005096 |
| sp|Q8TCC3|RM30_ HUMAN | GO:0070126 GO:0070125 GO:0005743 GO:0005761 GO:0070124 |
| sp|Q8TCJ2|STT3B_ HUMAN | GO:0016021 GO:0008250 GO:0018279 GO:0006516 GO:0043687 GO:0030433 GO:0006986 GO:0004579 GO:0043686 |
| sp|Q8TCS8|PNPT1_ HUMAN | GO:0070584 GO:0000962 GO:0005515 GO:0016020 GO:0005758 GO:0097222 GO:2000772 GO:0061014 GO:0044822 GO:0071042 GO:0045926 GO:0034046 GO:0090503 GO:0045025 GO:0071850 GO:0035458 GO:0035928 GO:0004654 GO:0035198 GO:0043457 GO:2000627 GO:0000958 GO:0008266 GO:0000175 GO:0034599 GO:0000964 GO:0070207 |
| sp|Q8TDB6|DTX3L_ HUMAN | GO:0042393 GO:0008270 GO:0010390 GO:0006302 GO:0004842 GO:0070735 GO:0070739 GO:0005654 GO:0005737 |
| sp|Q8TDD1|DDX54_ HUMAN | GO:0003714 GO:0016020 GO:0044822 GO:0005730 GO:0006355 GO:0006396 GO:0030331 GO:0030520 GO:0004004 GO:0005524 GO:1903507 GO:0010501 |
| sp|Q8TDZ2|MICA1_ HUMAN | GO:0008270 GO:0019417 GO:0017137 GO:0005882 GO:0005737 GO:0071949 GO:0003779 GO:0001933 GO:0043154 GO:0007165 GO:0017124 GO:0016709 GO:0030042 |
| sp|Q8TE67|ES8L3_ HUMAN | GO:0005737 |
| sp|Q8TE68|ES8L1_ HUMAN | GO:0032587 GO:0043234 GO:0030676 GO:1900029 GO:0051015 GO:0070062 GO:0043547 GO:0016310 GO:0016301 GO:0035023 GO:0042608 GO:0005737 GO:0016601 |
| sp|Q8TED0|UTP15_ HUMAN | GO:0005737 GO:0001650 GO:0030054 GO:0005515 GO:0006364 GO:0044822 |
| sp|Q8TEM1|PO210_ HUMAN | GO:0031047 GO:0007077 GO:0051028 GO:1900034 GO:0031965 GO:0010827 GO:0015031 GO:0005789 GO:0046983 GO:0019221 GO:0005643 GO:0005975 GO:0008033 GO:0016021 GO:0044281 GO:0019083 GO:0016925 GO:0000088 GO:0055085 GO:0017056 GO:0043687 GO:0019054 |
| sp|Q8TEQ8|PIGO_ HUMAN | GO:0016021 GO:0006501 GO:0051377 GO:0016254 GO:0005515 GO:0005789 |
| sp|Q8WTS6|SETD7_ HUMAN | GO:0045893 GO:0002039 GO:0005654 GO:0018024 GO:0045471 GO:0006974 GO:0018026 GO:0005730 GO:0051570 GO:0005694 GO:0018027 GO:0051348 GO:0070828 GO:0003682 |
| sp|Q8WUA2|PPIL4_ HUMAN | GO:0005737 GO:0006457 GO:0005654 GO:0000413 GO:0003755 GO:0000166 GO:0044822 |
| sp|Q8WUK0|PTPM1_ HUMAN | GO:0031305 GO:0035335 GO:0046488 GO:0004439 GO:0004725 GO:0008138 GO:0005515 GO:0044281 GO:0008962 GO:0005634 GO:0032049 |
| sp|Q8WUP2|FBLI1_ HUMAN | GO:0005829 GO:0008270 GO:0008360 GO:0031005 GO:0016337 GO:0001725 GO:0005938 GO:0005925 GO:0034329 GO:0033623 |
| sp|Q8WUY1|THEM6_ HUMAN | GO:0005576 |
| sp|Q8WV74|NUDT8_ HUMAN | GO:0036222 GO:0044606 GO:0008758 GO:0046872 GO:0019176 GO:0008413 GO:0008796 GO:0036218 GO:0019177 GO:0035870 GO:0008152 GO:0004787 GO:0008828 GO:0005739 GO:0043141 |
| sp|Q8WV92|MITD1_ HUMAN | GO:0000920 GO:0030496 GO:0019898 GO:0070062 GO:0035091 GO:0031902 GO:0032091 GO:0071985 GO:0000281 GO:0019904 GO:0039702 GO:0042803 |
| sp|Q8WVC0|LEO1_ HUMAN | GO:0016593 GO:0001711 GO:0010390 GO:0016055 GO:0045638 GO:0031442 GO:0019827 GO:0032968 GO:1990269 GO:0005730 GO:0005813 GO:0033523 GO:0006378 |
| sp|Q8WVJ2|NUDC2_ HUMAN | GO:0005737 GO:0005815 GO:0005515 GO:0000777 GO:0000922 GO:0070062 |
| sp|Q8WVV4|POF1B_ HUMAN | GO:0003779 GO:0030057 GO:0005923 |
| sp|Q8WW12|PCNP_ HUMAN | GO:0005654 GO:0005515 GO:0016567 GO:0043161 |
| sp|Q8WW59|SPRY4_ HUMAN | GO:0005634 GO:0005739 |
| sp|Q8WWM7|ATX2L_ HUMAN | GO:0005515 GO:0010603 GO:0016607 GO:0010494 GO:0034063 GO:0016020 GO:0044822 |
| sp|Q8WY22|BRI3B_ HUMAN | GO:0016020 GO:0005739 |
| sp|Q92506|DHB8_ HUMAN | GO:0006633 GO:0051087 GO:0019152 GO:0032867 GO:0042803 GO:0051131 GO:0071577 GO:0018451 GO:0006486 GO:0052677 GO:0016021 GO:0002479 GO:0051082 GO:0047485 GO:0030521 GO:0047035 GO:0006955 GO:0006703 GO:0032442 GO:0031625 GO:0033709 GO:0034840 GO:0005783 GO:0031396 GO:0044105 GO:0051990 GO:0032147 GO:0004495 GO:0045892 GO:0032866 GO:0006974 GO:0006952 GO:0043547 GO:0003857 GO:0005740 GO:0000792 GO:0005654 GO:0008134 GO:0005759 GO:2001235 GO:0045580 GO:0000252 GO:0018452 GO:0002039 GO:0035380 GO:0005085 GO:0034831 GO:0016272 GO:0032040 GO:0008209 GO:0003714 GO:0002587 GO:0044103 GO:0019886 GO:0004448 GO:0005385 GO:0005201 GO:0044822 GO:0050681 GO:0008378 GO:0007264 GO:0008270 GO:0005794 GO:1901216 GO:0055114 GO:0003677 GO:0000281 GO:0000462 GO:0035064 GO:0008875 GO:0019901 GO:0031060 GO:0042612 GO:0042605 GO:0048258 GO:0042613 GO:0006338 GO:0006882 GO:0018453 GO:0000775 GO:0004303 GO:0033765 GO:0030686 GO:0035410 GO:0043713 GO:1903955 GO:0006334 GO:0005730 GO:0006457 GO:0070603 GO:0019885 GO:0030295 GO:0023026 |
| sp|Q92520|FAM3C_ HUMAN | GO:0005794 GO:0007275 GO:0005125 GO:0016021 GO:0016023 GO:0070062 |
| sp|Q92522|H1X_ HUMAN | GO:0005730 GO:0000786 GO:0003677 GO:0006334 GO:0005515 GO:0044822 |
| sp|Q92541|RTF1_ HUMAN | GO:0016593 GO:0001711 GO:0000122 GO:0005515 GO:0019827 GO:0032968 GO:0044822 GO:0003697 GO:0005730 GO:0051571 GO:0001832 GO:0080182 |
| sp|Q92542|NICA_ HUMAN | GO:0005925 GO:0007411 GO:0004175 GO:0002262 GO:0007219 GO:0005887 GO:0005515 GO:0022617 GO:0043085 GO:0048011 GO:0050673 GO:0048013 GO:0042470 GO:0005765 GO:0031293 GO:0005794 GO:0006509 GO:0097190 GO:0016485 GO:0007220 GO:0050435 GO:0043065 GO:0070062 GO:0042987 GO:0005783 GO:0042098 |
| sp|Q92615|LAR4B_ HUMAN | GO:0005829 GO:0010494 GO:0005730 GO:0045727 GO:0044822 GO:0005515 GO:0000166 GO:0042788 GO:0016020 |
| sp|Q92621|NU205_ HUMAN | GO:0051292 GO:0031047 GO:0005654 GO:0007077 GO:0051028 GO:0005515 GO:1900034 GO:0034399 GO:0000059 GO:0044611 GO:0031965 GO:0010827 GO:0005737 GO:0019221 GO:0005975 GO:0008033 GO:0044281 GO:0019083 GO:0016925 GO:0000088 GO:0055085 GO:0017056 GO:0043687 GO:0019054 |
| sp|Q92665|RT31_ HUMAN | GO:0070126 GO:0070125 GO:0005730 GO:0005743 GO:0003735 GO:0005763 GO:0044822 GO:0019904 GO:0070124 |
| sp|Q92692|NECT2_ HUMAN | GO:0005925 GO:0050839 GO:0007010 GO:0060370 GO:0042271 GO:0005887 GO:0005102 GO:0033005 GO:0002891 GO:0002860 GO:0001675 GO:0034332 GO:0032990 GO:0009566 GO:0015026 GO:0019064 GO:0007289 GO:0007156 GO:0005915 GO:0001618 GO:0009986 GO:0034329 GO:0007157 GO:0008037 GO:0007165 GO:0044782 GO:0051654 GO:0030382 GO:0046814 GO:0070062 GO:0042803 GO:0046596 |
| sp|Q92796|DLG3_ HUMAN | GO:0007411 GO:0043198 GO:0008022 GO:0030165 GO:0030426 GO:0016323 GO:0005615 GO:0046037 GO:0045197 GO:0005737 GO:0008285 GO:0045211 GO:0035255 GO:0001736 GO:0004385 GO:0043025 GO:0031625 GO:0007165 GO:0019903 GO:0007268 GO:0014069 GO:0046710 GO:0032281 GO:0005923 GO:0010923 GO:0097120 GO:0019900 GO:0043113 |
| sp|Q92804|RBP56_ HUMAN | GO:0006367 GO:0004402 GO:0000125 GO:0044822 GO:0051091 GO:0005737 GO:0005730 GO:0003713 GO:0003677 GO:0005669 GO:0043966 GO:0030914 GO:0008134 GO:0033276 GO:0003700 |
| sp|Q92841|DDX17_ HUMAN | GO:0005654 GO:0016020 GO:0044822 GO:0009791 GO:0033148 GO:0005730 GO:0006396 GO:0003713 GO:0072358 GO:0030331 GO:0045944 GO:0004004 GO:2001014 GO:0005524 GO:0010501 |
| sp|Q92878|RAD50_ HUMAN | GO:0000075 GO:0000790 GO:0031860 GO:0007346 GO:0004003 GO:0003690 GO:0007507 GO:0000019 GO:0090305 GO:0046872 GO:0035861 GO:1904354 GO:0005654 GO:0032508 GO:0007004 GO:0033674 GO:0000784 GO:0045120 GO:0044752 GO:0048471 GO:0032206 GO:0005524 GO:0000014 GO:0046597 GO:0006303 GO:0045003 GO:0007131 GO:0008408 GO:0000794 GO:0016234 GO:0030674 GO:0030870 GO:0051291 GO:0016020 GO:0031954 GO:0070192 |
| sp|Q92882|OSTF1_ HUMAN | GO:0005737 GO:0001503 GO:0007165 GO:0070062 GO:0017124 |
| sp|Q92896|GSLG1_ HUMAN | GO:0005102 GO:0000139 GO:0030512 GO:0060349 GO:0050900 GO:0006886 GO:0005578 GO:0007596 GO:0016021 GO:0010955 GO:0005797 GO:0070062 GO:0017134 GO:0005886 GO:0032330 |
| sp|Q92917|GPKOW_ HUMAN | GO:0003676 GO:0005654 GO:0005515 |
| sp|Q92945|FUBP2_ HUMAN | GO:0003730 GO:0005829 GO:0006810 GO:0010494 GO:0000375 GO:0030425 GO:2000628 GO:0006402 GO:0043488 GO:0005515 GO:0043025 GO:0006397 GO:0005654 GO:0006403 GO:0016020 GO:0006351 |
| sp|Q92974|ARHG2_ HUMAN | GO:0043065 GO:0060546 GO:0048011 GO:0032755 GO:0043198 GO:0050768 GO:0035023 GO:0005925 GO:0007015 GO:0000902 GO:0007067 GO:0000132 GO:0005923 GO:0051301 GO:0043234 GO:0043547 GO:0005829 GO:0097481 GO:0008134 GO:0071356 GO:0045944 GO:0050731 GO:0042127 GO:0045087 GO:0006886 GO:0008270 GO:0005794 GO:0051092 GO:0071474 GO:0005819 GO:0071225 GO:0005874 GO:1902219 GO:0032760 GO:0016023 GO:0030676 GO:1902042 GO:0043025 GO:0032587 GO:0008017 GO:0007026 GO:0048365 |
| sp|Q92979|NEP1_ HUMAN | GO:0009019 GO:0008174 GO:0051994 GO:0034541 GO:0070037 GO:0018423 GO:0030792 GO:0005515 GO:0008650 GO:0016205 GO:0008988 GO:0044822 GO:0034931 GO:0016428 GO:0043770 GO:0001824 GO:0052735 GO:0008172 GO:0005737 GO:0005730 GO:0043791 GO:0009008 GO:0071424 GO:0018707 GO:0043851 GO:0043782 GO:0043777 GO:0052666 GO:0043776 GO:0080012 GO:0052667 GO:0043780 GO:0016435 GO:0043834 GO:0009383 GO:0008425 GO:0034807 GO:0019702 GO:0034933 GO:0070475 GO:0042274 GO:0032040 GO:0043852 GO:0043833 GO:0052665 GO:0000179 GO:0052624 GO:0043827 GO:0043803 GO:0016279 GO:0017126 GO:0070677 GO:0019843 GO:0004809 |
| sp|Q93009|UBP7_ HUMAN | GO:0004197 GO:0002039 GO:0007275 GO:0008022 GO:0016579 GO:0016605 GO:0006283 GO:0050821 GO:0010216 GO:0005829 GO:0006511 GO:1904353 GO:0032088 GO:0031625 GO:0004843 GO:0043065 GO:0016032 GO:0042803 GO:0008134 |
| sp|Q93034|CUL5_ HUMAN | GO:0000209 GO:0007186 GO:0021942 GO:0005262 GO:0005000 GO:0042787 GO:0005829 GO:0097193 GO:0008285 GO:0046982 GO:0007050 GO:0031466 GO:0051480 GO:0006970 GO:0031625 GO:0021799 GO:0000082 GO:0070588 GO:0016032 GO:0005886 GO:0005634 GO:0061630 |
| sp|Q969E2|SCAM4_ HUMAN | GO:0019098 GO:0016021 GO:0030133 GO:0015031 |
| sp|Q969H8|MYDGF_ HUMAN | GO:0001938 GO:0005515 GO:0036498 GO:0005793 GO:0043066 GO:0005788 GO:0045766 GO:0005615 GO:0014068 GO:0051897 GO:0045944 GO:0070062 GO:0043410 |
| sp|Q969K7|TMM54_ HUMAN | GO:0016021 |
| sp|Q969S3|ZN622_ HUMAN | GO:0033674 GO:0005515 GO:0008270 GO:0008631 GO:0044822 GO:0042273 GO:0005794 GO:0022625 GO:0005730 GO:0005801 GO:0043065 GO:0061709 GO:0046330 GO:0019233 GO:0043524 GO:0030176 GO:0030687 |
| sp|Q969U7|PSMG2_ HUMAN | GO:0005515 GO:0043066 GO:0007094 GO:0005634 GO:0043248 |
| sp|Q969X5|ERGI1_ HUMAN | GO:0000139 GO:0005789 GO:0033116 GO:0005515 GO:0016021 GO:0006888 |
| sp|Q96A33|CCD47_ HUMAN | GO:0006983 GO:0016021 GO:0005509 GO:0007029 GO:0044822 GO:0030433 GO:0005791 GO:0005515 GO:0055074 GO:0009791 GO:0001649 GO:0009790 |
| sp|Q96A35|RM24_ HUMAN | GO:0070126 GO:0005840 GO:0070125 GO:0005743 GO:0003735 GO:0070124 |
| sp|Q96A57|TM230_ HUMAN | GO:0016021 |
| sp|Q96A65|EXOC4_ HUMAN | GO:0043198 GO:0048709 GO:0035748 GO:0030165 GO:0051223 GO:0032584 GO:0006904 GO:0047485 GO:0005902 GO:0044267 GO:0006612 GO:0005794 GO:0048341 GO:0050850 GO:0005829 GO:0006996 GO:0000145 GO:0043025 GO:0017160 GO:0044091 GO:0007268 GO:0014069 GO:0005768 GO:0030010 GO:0055108 GO:0032403 GO:0006903 |
| sp|Q96AB3|ISOC2_ HUMAN | GO:0031648 GO:0008152 GO:0003824 GO:0005739 GO:0005634 GO:0005515 |
| sp|Q96AP7|ESAM_ HUMAN | GO:0005912 GO:0050900 GO:0005923 GO:0016338 GO:0016337 GO:0005886 GO:0007596 GO:0016021 GO:0007156 GO:0070062 |
| sp|Q96AT9|RPE_ HUMAN | GO:0005829 GO:0030054 GO:0048029 GO:0008157 GO:0048870 GO:0046872 GO:0031532 GO:0004864 GO:0045202 GO:0070062 GO:0009052 GO:0043149 GO:0044262 GO:0019323 GO:0043086 GO:0003779 GO:0042803 GO:0004750 GO:0008599 GO:0005634 |
| sp|Q96B49|TOM6_ HUMAN | GO:0000423 GO:0044267 GO:0005742 GO:0006626 |
| sp|Q96BI1|S22AI_ HUMAN | GO:0015238 GO:0006855 GO:0015293 GO:0006820 GO:0031625 GO:0008514 GO:0005887 GO:0007588 GO:0016324 GO:0005635 GO:0015695 GO:0005737 |
| sp|Q96BP3|PPWD1_ HUMAN | GO:0006457 GO:0005654 GO:0000398 GO:0000413 GO:0003755 GO:0071013 |
| sp|Q96C23|GALM_ HUMAN | GO:0005737 GO:0030246 GO:0006006 GO:0006012 GO:0004034 GO:0070062 GO:0042803 |
| sp|Q96C86|DCPS_ HUMAN | GO:0005829 GO:0045735 GO:0000290 GO:0050072 GO:0005739 GO:0000340 GO:0045292 GO:0043069 GO:0005515 GO:0036245 GO:0005654 GO:0043928 |
| sp|Q96CN7|ISOC1_ HUMAN | GO:0005777 GO:0070062 GO:0008152 GO:0003824 GO:0005515 |
| sp|Q96CS3|FAF2_ HUMAN | GO:0016021 GO:0031625 GO:0043130 GO:0034098 GO:0035473 GO:0030433 GO:0043086 GO:0030970 GO:0034389 GO:0006986 GO:0055102 GO:0005811 |
| sp|Q96DB5|RMD1_ HUMAN | GO:0005813 GO:0005739 GO:0005874 GO:0000922 |
| sp|Q96DG6|CMBL_ HUMAN | GO:0042578 GO:0006805 GO:0044281 GO:0043765 GO:0005829 GO:0044824 GO:0070062 |
| sp|Q96DV4|RM38_ HUMAN | GO:0070126 GO:0005840 GO:0070125 GO:0005743 GO:0005515 GO:0070124 |
| sp|Q96EE3|SEH1_ HUMAN | GO:0034629 GO:0031047 GO:0051315 GO:0007077 GO:0034198 GO:0051028 GO:0005515 GO:1900034 GO:0000236 GO:0061700 GO:0007080 GO:0002534 GO:0010827 GO:0000777 GO:0015031 GO:0051301 GO:0006999 GO:0005829 GO:0032008 GO:0019221 GO:0005975 GO:0007264 GO:0008033 GO:0044281 GO:0000090 GO:0019083 GO:0016925 GO:0000088 GO:0055085 GO:0031080 GO:0043687 GO:0019054 GO:0050830 |
| sp|Q96EK5|KBP_ HUMAN | GO:0005856 GO:0030154 GO:0019894 GO:0007399 GO:0006839 GO:0005739 |
| sp|Q96EY7|PTCD3_ HUMAN | GO:0070126 GO:0070125 GO:0006417 GO:0005743 GO:0019843 GO:0043024 GO:0044822 GO:0005515 GO:0070124 |
| sp|Q96EY8|MMAB_ HUMAN | GO:0008817 GO:0005524 GO:0009236 GO:0005759 |
| sp|Q96FQ6|S10AG_ HUMAN | GO:0044763 GO:0008270 GO:0044822 GO:0031323 GO:0051592 GO:0005509 GO:0048156 GO:0005730 GO:0005829 GO:0071638 GO:0070062 GO:0042803 GO:0005886 |
| sp|Q96FZ7|CHMP6_ HUMAN | GO:0005829 GO:0000920 GO:0047485 GO:1904902 GO:0007080 GO:1903541 GO:0070062 GO:0016197 GO:0000815 GO:0006997 GO:0039702 GO:0036258 GO:0006914 |
| sp|Q96G03|PGM2_ HUMAN | GO:0005829 GO:0005980 GO:0006098 GO:0019388 GO:0070062 GO:0005515 GO:0000287 GO:0046386 GO:0006006 GO:0008973 GO:0004614 GO:0005978 |
| sp|Q96GA3|LTV1_ HUMAN | GO:0005654 GO:0005737 GO:0005515 |
| sp|Q96GC5|RM48_ HUMAN | GO:0070126 GO:0070125 GO:0005743 GO:0005761 GO:0005515 GO:0070124 |
| sp|Q96GD4|AURKB_ HUMAN | GO:0043988 GO:0016925 GO:0000236 GO:0070938 GO:0051256 GO:0045171 GO:0043687 GO:0051983 GO:0030496 GO:0032466 GO:0032091 GO:0010369 GO:0009838 GO:0031577 GO:0032133 GO:0046872 GO:0005829 GO:0000776 GO:0005654 GO:1904355 GO:0031145 GO:0004712 GO:0042585 GO:0034644 GO:0034501 GO:0002903 GO:0005876 GO:0007568 GO:0008608 GO:0031616 GO:0000780 GO:1990385 GO:0007264 GO:0005515 GO:0000122 GO:0005524 GO:0032212 GO:0008283 GO:0046777 GO:0043146 GO:0036089 GO:0035174 GO:0000090 GO:0051973 GO:0032467 |
| sp|Q96GG9|DCNL1_ HUMAN | GO:0051443 GO:0031624 GO:0032182 GO:0097602 GO:0045116 GO:0000151 GO:0005634 |
| sp|Q96GM8|TOE1_ HUMAN | GO:0005737 GO:0005730 GO:0004535 GO:0046872 GO:0015030 GO:0003676 GO:0005515 GO:0016607 GO:0090503 |
| sp|Q96GS4|CQ059_ HUMAN | GO:0005515 |
| sp|Q96GW9|SYMM_ HUMAN | GO:0004825 GO:0005524 GO:0005759 GO:0006431 |
| sp|Q96GX9|MTNB_ HUMAN | GO:0046570 GO:0019509 GO:0008270 GO:0042802 GO:0006595 GO:0019284 GO:0070372 GO:0005737 GO:0043066 |
| sp|Q96H20|SNF8_ HUMAN | GO:0005654 GO:0055037 GO:0010628 GO:0061635 GO:0000814 GO:0071985 GO:0008022 GO:0006357 GO:0032456 GO:0036258 GO:0047485 GO:1903772 GO:0005829 GO:0045732 GO:0031902 GO:0005667 GO:0016247 GO:0043405 GO:0010797 GO:0006914 GO:0045022 GO:0048471 GO:0070062 GO:0042803 GO:1903543 GO:0005886 GO:0008134 GO:0043328 |
| sp|Q96HE7|ERO1A_ HUMAN | GO:0015035 GO:0005515 GO:0010260 GO:0005788 GO:0030198 GO:0070191 GO:0050873 GO:0003756 GO:0008113 GO:0033743 GO:0000302 GO:0030425 GO:0045454 GO:0071456 GO:0051085 GO:0006464 GO:0019471 GO:0055114 GO:0009266 GO:0070059 GO:0030176 GO:0030968 GO:0051209 GO:0022417 |
| sp|Q96HR9|REEP6_ HUMAN | GO:0016021 GO:0044317 GO:0060042 GO:0032386 GO:0005515 GO:0005789 GO:0045177 GO:0005634 GO:0005886 |
| sp|Q96HS1|PGAM5_ HUMAN | GO:0006470 GO:0016021 GO:0035556 GO:0032403 GO:0005741 GO:0009400 GO:0070266 GO:0043547 GO:0005096 |
| sp|Q96HY6|DDRGK_ HUMAN | GO:1903721 GO:0033146 GO:0005789 GO:1901800 GO:0051092 GO:1990592 GO:0044389 |
| sp|Q96I36|COX14_ HUMAN | GO:0033617 GO:0016021 GO:0031966 |
| sp|Q96I51|WBS16_ HUMAN | GO:0005087 GO:0005739 GO:0044822 GO:0072378 |
| sp|Q96I59|SYNM_ HUMAN | GO:0004816 GO:0006421 GO:0003676 GO:0005524 GO:0005759 |
| sp|Q96IQ7|VSIG2_ HUMAN | GO:0005887 |
| sp|Q96IR7|HPDL_ HUMAN | GO:0046872 GO:0034533 GO:0009072 GO:0034535 GO:0018553 GO:0018554 GO:0003868 GO:0034788 GO:0034527 GO:0055114 GO:0034563 |
| sp|Q96IZ0|PAWR_ HUMAN | GO:0005080 GO:0071392 GO:2000391 GO:0097190 GO:0030889 GO:0032516 GO:0043522 GO:0043525 GO:0042094 GO:1903238 GO:0005884 GO:0060450 GO:0097202 GO:0050860 GO:0003714 GO:0030424 GO:1904457 GO:0050966 GO:0010040 GO:0090281 GO:0005886 GO:0005737 GO:0008157 GO:0042130 GO:0071306 GO:0000122 GO:0071347 GO:0071372 GO:0050965 GO:0042986 GO:0051017 GO:1990035 GO:0005634 GO:0043025 GO:1901082 GO:0009611 GO:0032496 GO:0003779 |
| sp|Q96JB5|CK5P3_ HUMAN | GO:0007095 GO:0000079 GO:0007420 GO:0043234 GO:0032088 GO:1903363 GO:0012505 GO:0030968 GO:0045664 GO:0045944 GO:1900182 GO:0005737 GO:0032403 GO:0097371 GO:0043407 GO:0044389 GO:0030332 GO:0008283 GO:0005874 GO:0071569 GO:0051059 GO:2000060 GO:1901798 GO:0030262 GO:0044387 GO:0010921 GO:0005813 GO:0005730 GO:0016020 GO:0051019 |
| sp|Q96K76|UBP47_ HUMAN | GO:0030307 GO:0071987 GO:1902230 GO:0005737 GO:0019005 GO:0006511 GO:0045892 GO:0043154 GO:0004843 GO:0035520 GO:0006284 GO:0042493 GO:0034644 GO:0010972 GO:0005634 |
| sp|Q96KA5|CLP1L_ HUMAN | GO:0016021 GO:0006915 |
| sp|Q96L92|SNX27_ HUMAN | GO:0005829 GO:0031901 GO:0030904 GO:1990126 GO:0008333 GO:0032266 GO:0071203 GO:0007399 GO:0005515 GO:0007165 GO:0001772 GO:0042493 GO:0006886 GO:0005654 GO:0001770 |
| sp|Q96LA8|ANM6_ HUMAN | GO:0005654 GO:0000122 GO:0035241 GO:0042393 GO:0034970 GO:0090398 GO:0070612 GO:0035242 GO:0070611 GO:0005829 GO:0006284 GO:0043985 GO:0016049 GO:0016032 GO:0003682 GO:0019919 GO:0044020 |
| sp|Q96LJ7|DHRS1_ HUMAN | GO:0034847 GO:0018449 GO:0005743 GO:0034863 GO:0018450 GO:0018447 GO:0034868 GO:0005515 GO:0034901 GO:0034817 GO:0045703 GO:0034918 GO:0042469 GO:0034944 GO:0034871 GO:0034891 GO:0018448 GO:0005783 GO:0034582 GO:0034821 GO:0034778 GO:0055114 GO:0034522 GO:0018446 |
| sp|Q96M27|PRRC1_ HUMAN | GO:0005794 GO:0042802 |
| sp|Q96NC0|ZMAT2_ HUMAN | GO:0003677 GO:0046540 GO:0003723 GO:0000398 GO:0005515 GO:0008270 GO:0019233 |
| sp|Q96P70|IPO9_ HUMAN | GO:0005829 GO:0008565 GO:0008536 GO:0042393 GO:0042991 GO:0050821 GO:0042254 GO:0005635 GO:0006610 GO:0016020 |
| sp|Q96PZ0|PUS7_ HUMAN | GO:0009982 GO:0006400 GO:0001522 GO:0019899 GO:0005634 GO:0044822 |
| sp|Q96Q11|TRNT1_ HUMAN | GO:0006626 GO:0001680 GO:0052928 GO:0090646 GO:0009022 GO:0005524 GO:0005759 GO:0052929 GO:0000049 GO:0052927 GO:0005654 |
| sp|Q96QK1|VPS35_ HUMAN | GO:0005515 GO:0042147 GO:0031647 GO:0008565 GO:0005765 GO:0061357 GO:0006886 GO:0005829 GO:0005770 GO:0097422 GO:0090263 GO:0030906 GO:1902823 GO:0005769 GO:0016021 GO:0006624 GO:1990126 GO:0045056 GO:0070062 GO:0097481 |
| sp|Q96QR8|PURB_ HUMAN | GO:0017148 GO:0000122 GO:0005662 GO:0042127 GO:0036477 GO:0000900 GO:0001227 GO:0000977 GO:0006915 GO:0003697 GO:0032422 GO:0008134 GO:0045637 GO:0003729 GO:0046332 |
| sp|Q96RF0|SNX18_ HUMAN | GO:0030659 GO:0007067 GO:0036089 GO:0030136 GO:0070062 GO:0005546 GO:0016197 GO:0005515 GO:0043547 GO:0000281 GO:0043025 GO:0006897 GO:0030426 GO:0006886 GO:0010008 GO:0005654 GO:0031234 GO:0016050 |
| sp|Q96RS6|NUDC1_ HUMAN | GO:0005654 GO:0005737 GO:0002376 GO:0005515 |
| sp|Q96S44|PRPK_ HUMAN | GO:0070525 GO:0005829 GO:0006468 GO:0000408 GO:0002039 GO:0004674 GO:0005524 GO:0016787 GO:0006400 GO:0016020 GO:0005634 |
| sp|Q96S55|WRIP1_ HUMAN | GO:0046872 GO:0042802 GO:0003677 GO:0000731 GO:0005524 GO:0016887 GO:0030174 GO:0000784 GO:0048471 GO:0016020 |
| sp|Q96SB4|SRPK1_ HUMAN | GO:0005515 GO:0048024 GO:0044822 GO:0006468 GO:0004674 GO:0007059 GO:0000287 GO:0045071 GO:0045070 GO:0016363 GO:0005783 GO:0035092 GO:0005886 GO:0045087 GO:0005524 GO:0035556 |
| sp|Q96SQ9|CP2S1_ HUMAN | GO:0005789 GO:0006805 GO:0003676 GO:0005506 GO:0005515 GO:0044281 GO:0020037 GO:0016021 GO:0008395 GO:0070330 GO:0055114 GO:0000166 |
| sp|Q96T51|RUFY1_ HUMAN | GO:0008289 GO:0005770 GO:0005515 GO:0031901 GO:0008565 GO:0005634 GO:0005829 GO:0008270 GO:0030100 GO:0015031 |
| sp|Q96T58|MINT_ HUMAN | GO:0050769 GO:0045893 GO:0005654 GO:0000122 GO:0007219 GO:0003714 GO:0000166 GO:0001191 GO:0044822 GO:0017053 GO:0003697 GO:0001085 GO:0070062 GO:0016032 GO:0000398 GO:0003700 |
| sp|Q96T76|MMS19_ HUMAN | GO:0045893 GO:0016020 GO:0009725 GO:0030159 GO:0071817 GO:0016226 GO:0030674 GO:0005675 GO:0006289 GO:0003713 GO:0007059 GO:0097361 GO:0030331 GO:0044281 GO:0000160 GO:0019899 |
| sp|Q96T88|UHRF1_ HUMAN | GO:0032270 GO:0051865 GO:0000122 GO:0010390 GO:0008270 GO:0042787 GO:0016874 GO:0008283 GO:0043434 GO:0031493 GO:0000791 GO:0010216 GO:0032776 GO:0005720 GO:0042802 GO:0031410 GO:2000373 GO:0090308 GO:0016363 GO:0008327 GO:0045944 GO:0000987 GO:0005886 GO:0035064 GO:0044729 GO:0061630 GO:0003700 GO:0007049 GO:0006281 GO:0005657 |
| sp|Q99418|CYH2_ HUMAN | GO:0030155 GO:0001726 GO:0005086 GO:0032012 GO:0008289 GO:2000171 GO:0030036 GO:0005515 GO:0043547 GO:0006897 GO:0030426 GO:0070679 GO:0005737 GO:0005886 |
| sp|Q99426|TBCB_ HUMAN | GO:0030154 GO:0005654 GO:0051084 GO:0005515 GO:0007399 GO:0007023 GO:0044267 GO:0005829 GO:0005874 |
| sp|Q99442|SEC62_ HUMAN | GO:0005829 GO:0016021 GO:0016235 GO:0044267 GO:0004872 GO:0045111 GO:0005791 GO:0006620 GO:0006613 GO:0036498 GO:0005789 |
| sp|Q99447|PCY2_ HUMAN | GO:0005789 GO:0006646 GO:0044281 GO:0004306 |
| sp|Q99471|PFD5_ HUMAN | GO:0005829 GO:0044267 GO:0051086 GO:0090090 GO:0003714 GO:0051082 GO:0060041 GO:0016272 GO:0045892 GO:0005634 |
| sp|Q99496|RING2_ HUMAN | GO:0060042 GO:0016925 GO:0070736 GO:0000791 GO:0071339 GO:1902254 GO:0043687 GO:0021510 GO:0036353 GO:0070738 GO:0070740 GO:0048701 GO:0043433 GO:0043774 GO:0071535 GO:0016604 GO:0033339 GO:0001702 GO:0043773 GO:0001739 GO:0032330 GO:0008270 GO:0018169 GO:0000278 GO:0000122 GO:0003682 GO:0007281 GO:0033554 GO:0061630 GO:0035102 GO:0009948 GO:0008766 GO:0032526 GO:0070737 |
| sp|Q99622|C10_ HUMAN | GO:0005737 |
| sp|Q99623|PHB2_ HUMAN | GO:0033600 GO:0009986 GO:0060744 GO:0033147 GO:0047485 GO:0008022 GO:0016363 GO:0070374 GO:0051091 GO:0043234 GO:0007005 GO:0043433 GO:0033218 GO:0030449 GO:0000060 GO:0070062 GO:0007062 GO:1902808 GO:0060762 GO:0050821 GO:0031536 GO:0005741 GO:0071944 GO:0043066 GO:0005743 GO:0060749 GO:0030331 |
| sp|Q99735|MGST2_ HUMAN | GO:0016021 GO:0008047 GO:0055114 GO:0006750 GO:0098869 GO:0019370 GO:0006805 GO:0004602 GO:0004364 GO:0010243 GO:0004464 GO:0032496 GO:0005789 GO:1901687 GO:0043085 GO:0005886 |
| sp|Q99797|MIPEP_ HUMAN | GO:0006518 GO:0046872 GO:0006627 GO:0004222 GO:0005759 |
| sp|Q99816|TS101_ HUMAN | GO:0043130 GO:0042803 GO:0046790 GO:0005771 GO:0016021 GO:0043405 GO:0051301 GO:0031625 GO:0001558 GO:0031902 GO:0019082 GO:0045892 GO:0000813 GO:0097352 GO:0003714 GO:1903543 GO:0036258 GO:0075733 GO:0005886 GO:0070062 GO:1903774 GO:0030216 GO:0005769 GO:0007050 GO:0003677 GO:0030374 GO:2000397 GO:0048306 GO:0043162 GO:0008285 GO:1903551 GO:1902188 GO:0008333 GO:0005730 GO:0006513 |
| sp|Q99832|TCPH_ HUMAN | GO:1904851 GO:0032212 GO:0044297 GO:0050821 GO:0044267 GO:0005832 GO:0005874 GO:1904874 GO:0007339 GO:0042802 GO:1904871 GO:0051082 GO:1901998 GO:0070062 GO:0051084 GO:0005524 GO:0002199 GO:0005739 |
| sp|Q9BPU6|DPYL5_ HUMAN | GO:0007411 GO:0005829 GO:0008017 GO:0043234 GO:0016810 GO:0030425 GO:0043025 GO:0007165 GO:0008152 |
| sp|Q9BQ04|RBM4B_ HUMAN | GO:0046822 GO:0010628 GO:0005515 GO:0008270 GO:0000166 GO:0032922 GO:0016607 GO:0003730 GO:0000381 GO:0035278 GO:0046685 GO:0045947 GO:0097167 GO:0005730 GO:0032055 GO:0010494 GO:0035198 GO:0002192 GO:0043153 GO:0051149 GO:0097158 GO:0051403 |
| sp|Q9BQ69|MACD1_ HUMAN | GO:0042278 GO:0016811 GO:0005739 GO:0019213 GO:0051725 GO:0004844 GO:0005515 GO:0006974 GO:0005634 |
| sp|Q9BQC3|DPH2_ HUMAN | GO:0017183 GO:0090560 GO:0005515 GO:0043687 GO:0005829 |
| sp|Q9BRP1|PDD2L_ HUMAN | GO:0005737 GO:0007049 GO:0016020 |
| sp|Q9BRP4|PAAF1_ HUMAN | GO:0016032 GO:0016021 GO:0000502 GO:0005515 |
| sp|Q9BRP8|PYM1_ HUMAN | GO:0035145 GO:0005730 GO:1903259 GO:0043022 GO:0045727 GO:0044822 GO:0005515 GO:0005654 GO:0005737 GO:0000184 |
| sp|Q9BRR6|ADPGK_ HUMAN | GO:0043843 GO:0016021 GO:0046872 GO:0006006 GO:0005576 GO:0006096 GO:0005783 |
| sp|Q9BRT3|MIEN1_ HUMAN | GO:0005829 GO:0070062 GO:0030335 GO:0005515 GO:0051491 GO:0005654 GO:0043066 GO:0031235 |
| sp|Q9BS26|ERP44_ HUMAN | GO:0003756 GO:0034976 GO:0006457 GO:0005793 GO:0045454 GO:0070062 GO:0009986 GO:0005788 GO:0005515 GO:0006986 GO:0005789 GO:0009100 |
| sp|Q9BS40|LXN_ HUMAN | GO:0006954 GO:0010951 GO:0070062 GO:0005515 GO:0008191 GO:0008201 GO:0005737 GO:0050965 |
| sp|Q9BSC4|NOL10_ HUMAN | GO:0005730 GO:0044822 |
| sp|Q9BSH4|TACO1_ HUMAN | GO:0005654 GO:0006417 GO:0005739 |
| sp|Q9BSJ2|GCP2_ HUMAN | GO:0000923 GO:0005654 GO:0016020 GO:0051298 GO:0090063 GO:0043015 GO:0031122 GO:0005881 GO:0007229 GO:0005829 GO:0005813 GO:0090307 GO:0005816 GO:0008237 GO:0051415 GO:0006508 GO:0008275 GO:0007126 GO:0000086 GO:0005200 GO:0051011 |
| sp|Q9BST9|RTKN_ HUMAN | GO:0005829 GO:0005095 GO:0006915 GO:0034260 GO:0017049 GO:0007266 GO:0005525 |
| sp|Q9BT22|ALG1_ HUMAN | GO:0009103 GO:0016021 GO:0018279 GO:0043687 GO:0004578 GO:0097502 GO:0006488 GO:0005789 |
| sp|Q9BTC0|DIDO1_ HUMAN | GO:0008270 GO:0044822 GO:0005819 GO:0097190 GO:0005737 GO:0005634 GO:0006351 |
| sp|Q9BTD8|RBM42_ HUMAN | GO:0044822 GO:0048025 GO:0000166 GO:0005737 GO:0005634 |
| sp|Q9BTU6|P4K2A_ HUMAN | GO:0045121 GO:0044231 GO:0005887 GO:0035838 GO:0042734 GO:0030672 GO:0005765 GO:0005794 GO:0030425 GO:0031083 GO:0000287 GO:0002561 GO:0044281 GO:0030054 GO:0035651 GO:0006661 GO:0098779 GO:0031901 GO:0005524 GO:0043204 GO:0004430 GO:0046854 GO:0005739 |
| sp|Q9BTZ2|DHRS4_ HUMAN | GO:0004090 GO:0042574 GO:0055114 GO:0006066 GO:0031965 GO:0005739 GO:0070062 GO:0000253 GO:0018455 GO:0005778 GO:0005102 GO:0001758 GO:0051262 GO:0042180 GO:0008202 GO:0005789 GO:0016655 |
| sp|Q9BU61|NDUF3_ HUMAN | GO:0005743 GO:0005515 GO:0044281 GO:0005634 GO:0032981 GO:0022904 |
| sp|Q9BUF5|TBB6_ HUMAN | GO:0051084 GO:0044267 GO:0005874 GO:0070062 GO:0007017 GO:0003924 GO:0005200 GO:0005737 GO:0005634 GO:0005525 |
| sp|Q9BUH6|PAXX_ HUMAN | GO:0006303 GO:0035861 GO:0070419 GO:0032947 GO:0051103 GO:0043564 GO:0070062 GO:0042803 |
| sp|Q9BUL9|RPP25_ HUMAN | GO:0005815 GO:0005654 GO:0090501 GO:0005515 GO:0016787 GO:0008033 GO:0044822 |
| sp|Q9BUP3|HTAI2_ HUMAN | GO:0005654 GO:0005515 GO:0051287 GO:0016020 GO:0016620 GO:0043066 GO:0006357 GO:0051170 GO:0005737 GO:0005635 GO:0003713 GO:0030154 GO:0045765 GO:0055114 GO:0016032 |
| sp|Q9BUR5|MIC26_ HUMAN | GO:0034362 GO:0000139 GO:0006869 GO:0005515 GO:0034364 GO:0034361 GO:0005789 GO:0042407 GO:0061617 |
| sp|Q9BV40|VAMP8_ HUMAN | GO:0009986 GO:1903595 GO:0019869 GO:0005887 GO:0030496 GO:0031902 GO:0055037 GO:0005829 GO:1903531 GO:0097352 GO:0035493 GO:0043001 GO:0031301 GO:0070254 GO:0070062 GO:0048471 GO:0005739 GO:0043308 GO:0005484 GO:0035577 GO:1903076 GO:0043312 GO:0030100 GO:0031901 GO:0019905 GO:0031201 GO:0046718 GO:0098594 GO:0014704 GO:0007519 |
| sp|Q9BV57|MTND_ HUMAN | GO:0005829 GO:0010309 GO:0019509 GO:0055114 GO:0046872 GO:0005515 GO:0006595 GO:0005654 GO:0005794 GO:0005886 |
| sp|Q9BV79|MECR_ HUMAN | GO:0006633 GO:0019166 GO:0016922 GO:0005634 GO:0005829 GO:0008270 GO:0005739 GO:0055114 |
| sp|Q9BV81|EMC6_ HUMAN | GO:0072546 GO:0000045 GO:0097631 GO:0005515 |
| sp|Q9BVC5|ASHWN_ HUMAN | GO:0048598 GO:0008033 GO:0072669 GO:0005654 |
| sp|Q9BVI4|NOC4L_ HUMAN | GO:0016021 GO:0031965 GO:0005730 GO:0006364 GO:0044822 GO:0005515 GO:0032040 GO:0030692 |
| sp|Q9BVJ6|UT14A_ HUMAN | GO:0044763 GO:0005730 GO:0044767 GO:0032501 GO:0044822 GO:0042254 GO:0005515 GO:0032040 GO:0044702 |
| sp|Q9BVQ7|SPA5L_ HUMAN | GO:0005737 GO:0005524 |
| sp|Q9BW04|SARG_ HUMAN | GO:0070062 GO:0005737 GO:0005515 |
| sp|Q9BW60|ELOV1_ HUMAN | GO:0035338 GO:0043651 GO:0030176 GO:0036109 GO:0034625 GO:0019367 GO:0030148 GO:0009922 GO:0005515 GO:0019432 GO:0042761 |
| sp|Q9BW92|SYTM_ HUMAN | GO:0004829 GO:0005524 GO:0005759 GO:0070159 |
| sp|Q9BWH2|FUND2_ HUMAN | GO:0000422 GO:0031307 GO:0005634 |
| sp|Q9BWJ5|SF3B5_ HUMAN | GO:0005686 GO:0005654 GO:0007052 GO:0000398 GO:0071011 GO:0005689 |
| sp|Q9BX40|LS14B_ HUMAN | GO:0044822 |
| sp|Q9BX68|HINT2_ HUMAN | GO:0005730 GO:0052770 GO:0006694 GO:0052771 GO:0003923 GO:0006915 GO:0005739 GO:0000166 |
| sp|Q9BXP5|SRRT_ HUMAN | GO:0050769 GO:0005654 GO:0005515 GO:0031053 GO:0044822 GO:0008283 GO:0046685 GO:0005737 GO:0006355 GO:0003677 GO:0097150 |
| sp|Q9BXW7|CECR5_ HUMAN | GO:0046474 GO:0005739 |
| sp|Q9BY44|EIF2A_ HUMAN | GO:0010494 GO:0000049 GO:0032057 GO:0003743 GO:0072562 GO:0005829 GO:0098779 GO:0009967 GO:0005851 GO:0044207 GO:0005844 GO:0036499 GO:0034605 GO:0034644 GO:0005850 GO:0007568 GO:0044822 GO:0070062 GO:0098792 GO:0043022 GO:0005515 GO:0034198 GO:0097451 GO:0043614 GO:2000676 GO:0046777 GO:0032933 GO:0002230 GO:0005840 GO:0033290 GO:0005634 GO:0042255 GO:0055085 GO:0016020 GO:1990737 |
| sp|Q9BY49|PECR_ HUMAN | GO:0055114 GO:0005739 GO:0005778 GO:0005102 GO:0008670 GO:0019166 GO:0033306 GO:0030497 |
| sp|Q9BY77|PDIP3_ HUMAN | GO:0035145 GO:0016607 GO:0045727 GO:0044822 GO:0005515 GO:0000346 GO:0000166 GO:0005737 GO:0016973 |
| sp|Q9BYB4|GNB1L_ HUMAN | GO:0005737 GO:0035556 GO:0035176 GO:0007186 GO:0009898 |
| sp|Q9BYD1|RM13_ HUMAN | GO:0070126 GO:0005762 GO:0003729 GO:0070125 GO:0005743 GO:0003735 GO:0005515 GO:0070124 GO:0016787 |
| sp|Q9BYD2|RM09_ HUMAN | GO:0070126 GO:0070125 GO:0005743 GO:0005761 GO:0003735 GO:0044822 GO:0005515 GO:0070124 |
| sp|Q9BYD6|RM01_ HUMAN | GO:0070126 GO:0005762 GO:0070125 GO:0005743 GO:0003735 GO:0044822 GO:0005515 GO:0070124 |
| sp|Q9BYG3|MK67I_ HUMAN | GO:0009303 GO:0005730 GO:0000794 GO:0006461 GO:0044822 GO:0005515 GO:0000166 GO:0005654 GO:0005737 GO:0016072 GO:0010923 |
| sp|Q9BYG5|PAR6B_ HUMAN | GO:0005829 GO:0043234 GO:0007049 GO:0007163 GO:0070062 GO:0051301 GO:0006461 GO:0030334 GO:0005938 GO:0005923 GO:0005515 GO:0007409 GO:0070830 GO:0045177 GO:0005634 GO:0005886 |
| sp|Q9BYK8|HELZ2_ HUMAN | GO:0045944 GO:0030374 GO:0046872 GO:0003677 GO:0005524 GO:0044822 GO:0005515 GO:0004386 GO:0044255 GO:0005654 GO:0044281 GO:0016020 |
| sp|Q9BYN0|SRXN1_ HUMAN | GO:0098869 GO:0032542 GO:0006979 GO:0005524 GO:0005829 GO:0055114 |
| sp|Q9BYN8|RT26_ HUMAN | GO:0070126 GO:0070125 GO:0042769 GO:0005743 GO:0003735 GO:0005763 GO:0044822 GO:0070124 GO:0005654 |
| sp|Q9BYV2|TRI54_ HUMAN | GO:0007026 GO:0008017 GO:0005875 GO:0030018 GO:0008270 GO:0004871 GO:0005874 GO:0030154 GO:0007165 GO:0007275 |
| sp|Q9BZD3|GCOM2_ HUMAN | GO:0003899 GO:0035556 GO:0016591 GO:0051685 GO:0043025 GO:0005635 GO:0006351 |
| sp|Q9BZE9|ASPC1_ HUMAN | GO:0005829 GO:0046324 GO:0019898 GO:0061024 GO:0042593 GO:0033116 GO:0005515 GO:0012506 GO:0012505 GO:0048471 GO:0006886 GO:0005654 GO:0009898 |
| sp|Q9BZK7|TBL1R_ HUMAN | GO:0050872 GO:0000122 GO:0090207 GO:0007219 GO:0042393 GO:0030814 GO:0003714 GO:0016042 GO:0016575 GO:0017053 GO:0047485 GO:0043161 GO:0044212 GO:0002021 GO:0016021 GO:0000118 GO:0045944 GO:0005876 GO:0060613 GO:0035264 GO:0008013 GO:0060070 |
| sp|Q9BZL6|KPCD2_ HUMAN | GO:0050862 GO:0033138 GO:0032743 GO:0045743 GO:0030949 GO:0070374 GO:2000573 GO:0045766 GO:0046872 GO:0098779 GO:0002250 GO:0045785 GO:0005654 GO:0004697 GO:0089700 GO:1901727 GO:0045944 GO:0005886 GO:0032793 GO:0005794 GO:0051092 GO:0005515 GO:0008219 GO:0043536 GO:0005524 GO:0046777 GO:0038033 GO:0032757 GO:0061154 GO:0001938 |
| sp|Q9C0C9|UBE2O_ HUMAN | GO:0030513 GO:0070534 GO:0042147 GO:0006513 GO:0044822 GO:0005737 GO:0061631 GO:0031625 GO:0005634 GO:0005524 GO:0061630 |
| sp|Q9GZP4|PITH1_ HUMAN | GO:0005634 |
| sp|Q9GZP8|IMUP_ HUMAN | GO:0003697 GO:0005634 GO:0003690 |
| sp|Q9GZP9|DERL2_ HUMAN | GO:0030307 GO:0006457 GO:0005515 GO:0005770 GO:0008284 GO:0005769 GO:0001967 GO:1904153 GO:0030176 GO:0043687 GO:0030968 GO:0018279 GO:0030433 |
| sp|Q9H008|LHPP_ HUMAN | GO:0052825 GO:0005829 GO:0008969 GO:0008579 GO:0043812 GO:0006470 GO:0006144 GO:0043813 GO:0052830 GO:0030487 GO:0043726 GO:0017161 GO:0017017 GO:0052629 GO:0019198 GO:0009168 GO:0030352 GO:0052828 GO:0019178 GO:0052829 GO:0000287 GO:0008330 GO:0004427 GO:0052831 GO:0052867 GO:0030351 GO:0042803 GO:0005654 GO:1990003 |
| sp|Q9H061|T126A_ HUMAN | GO:0005743 GO:0021554 GO:0016021 |
| sp|Q9H0A0|NAT10_ HUMAN | GO:0005515 GO:0016020 GO:0000784 GO:0044822 GO:0032211 GO:0005697 GO:0005730 GO:0008033 GO:0005524 GO:0006364 GO:0008080 |
| sp|Q9H0B6|KLC2_ HUMAN | GO:0003777 GO:0005829 GO:0008017 GO:0043005 GO:0005874 GO:0019894 GO:0007264 GO:0008088 GO:0031514 GO:0035253 GO:0008152 GO:0019886 GO:0016938 GO:0016020 GO:0007596 |
| sp|Q9H0C8|ILKAP_ HUMAN | GO:0033262 GO:0006470 GO:0046872 GO:0007229 GO:0004722 GO:0005515 GO:0016310 GO:0016301 GO:0005737 |
| sp|Q9H0L4|CSTFT_ HUMAN | GO:0005847 GO:0003729 GO:0098789 GO:0005515 GO:0000166 |
| sp|Q9H0U4|RAB1B_ HUMAN | GO:0005525 GO:0048208 GO:0032402 GO:0043687 GO:0034045 GO:0030252 GO:0042742 GO:0016477 GO:0033116 GO:0000139 GO:0034446 GO:0005886 GO:0070062 GO:0007264 GO:0005739 GO:0006897 GO:0090557 GO:0005515 GO:0072606 GO:0042470 GO:0047496 GO:2000785 GO:0090110 GO:0030133 GO:0007030 GO:0018279 GO:1903020 GO:0019068 GO:0005789 |
| sp|Q9H269|VPS16_ HUMAN | GO:0005776 GO:0055037 GO:0046718 GO:0005884 GO:0032889 GO:0030123 GO:0035542 GO:0033263 GO:0005765 GO:0097352 GO:0006886 GO:0031902 GO:0043025 GO:0030424 GO:0008333 GO:0030897 GO:0051015 GO:0030136 GO:0031901 |
| sp|Q9H2G2|SLK_ HUMAN | GO:0031098 GO:0033129 GO:0030334 GO:0051893 GO:0031122 GO:0032147 GO:0005737 GO:0046777 GO:0031252 GO:0023014 GO:0004674 GO:0043065 GO:0070062 GO:0042803 GO:0007346 GO:0005524 |
| sp|Q9H2U1|DHX36_ HUMAN | GO:0000781 GO:0009615 GO:0008094 GO:0032481 GO:0042826 GO:0032206 GO:0044822 GO:0001047 GO:0001503 GO:0005829 GO:0006396 GO:0016021 GO:0051880 GO:0045944 GO:0002151 GO:0070062 GO:0003725 GO:0004004 GO:0045087 GO:0005634 GO:0005524 GO:0010501 GO:0043330 |
| sp|Q9H2U2|IPYR2_ HUMAN | GO:0006470 GO:0071344 GO:0070062 GO:0004722 GO:0000287 GO:0004427 GO:0006418 GO:0005759 |
| sp|Q9H2W6|RM46_ HUMAN | GO:0030054 GO:0070126 GO:0005762 GO:0070125 GO:0005743 GO:0003735 GO:0070124 GO:0005654 GO:0016787 |
| sp|Q9H330|TM245_ HUMAN | GO:0016021 |
| sp|Q9H3H3|CK068_ HUMAN | GO:0005515 GO:0044822 |
| sp|Q9H3N1|TMX1_ HUMAN | GO:0016021 GO:0003756 GO:0055114 GO:0034976 GO:0006457 GO:0005730 GO:0045454 GO:0030239 GO:0071822 GO:0005789 GO:0015036 |
| sp|Q9H3R2|MUC13_ HUMAN | GO:0005829 GO:0005796 GO:0016021 GO:0070062 GO:0005615 GO:0030277 GO:0043687 GO:0016324 GO:0042803 GO:0016266 |
| sp|Q9H3U1|UN45A_ HUMAN | GO:0030154 GO:0007517 GO:0005654 GO:0005794 GO:0051879 GO:0061077 GO:0048471 |
| sp|Q9H444|CHM4B_ HUMAN | GO:0030117 GO:0039702 GO:0007080 GO:1901673 GO:0090611 GO:1902902 GO:0000920 GO:0000815 GO:0006620 GO:0036258 GO:0016197 GO:1902188 GO:0005829 GO:0036438 GO:0006997 GO:0030496 GO:0070062 GO:0010824 GO:0042803 GO:0051260 GO:0009898 GO:0005634 GO:1901215 |
| sp|Q9H479|FN3K_ HUMAN | GO:0043841 GO:0052812 GO:0043771 GO:0005829 GO:0052670 GO:0008819 GO:0051734 GO:0008443 GO:0043915 GO:0052810 GO:0043798 GO:0008607 GO:0030387 GO:0051735 GO:0052811 GO:0052836 GO:0043687 GO:0052672 GO:0035004 GO:0043899 GO:0052669 GO:0043743 GO:0018720 GO:0044605 GO:0052839 GO:0030855 GO:0019914 GO:0033785 GO:0016310 GO:0032942 GO:0042557 GO:0052835 GO:0010276 GO:0034071 GO:0030393 GO:0016538 |
| sp|Q9H488|OFUT1_ HUMAN | GO:0001525 GO:0036066 GO:0007507 GO:0007219 GO:0006355 GO:0007399 GO:0046922 GO:0006004 GO:0001756 GO:0016020 GO:0016266 GO:0005783 |
| sp|Q9H490|PIGU_ HUMAN | GO:0042765 GO:0006501 GO:0046425 GO:0016255 GO:0034235 GO:0005886 GO:0003923 |
| sp|Q9H4A6|GOLP3_ HUMAN | GO:0050901 GO:0005654 GO:0005802 GO:0030032 GO:0005758 GO:0043066 GO:0010467 GO:0048194 GO:0008283 GO:0070273 GO:0005829 GO:0032008 GO:0050714 GO:0016021 GO:0005768 GO:0010821 GO:0032580 GO:0009101 GO:0045053 GO:0060352 GO:0043001 GO:0019899 GO:0005886 GO:0090164 |
| sp|Q9H501|ESF1_ HUMAN | GO:0005730 GO:0005615 GO:0006355 GO:0044822 GO:0005654 |
| sp|Q9H5Q4|TFB2M_ HUMAN | GO:0045893 GO:0016436 GO:0031167 GO:0003712 GO:0044822 GO:0016278 GO:0051995 GO:0016300 GO:0016423 GO:0042645 GO:0006391 GO:0016427 GO:0016434 GO:0008169 GO:0003880 GO:0030054 GO:0000179 GO:0016429 GO:0016274 |
| sp|Q9H5X1|FA96A_ HUMAN | GO:0005737 GO:0046872 GO:0007059 GO:0005654 GO:0016226 GO:0005515 |
| sp|Q9H6S3|ES8L2_ HUMAN | GO:0032587 GO:0043234 GO:0030676 GO:1900029 GO:0051015 GO:0070062 GO:0043547 GO:0035023 GO:0005737 GO:0016601 |
| sp|Q9H6T0|ESRP2_ HUMAN | GO:0003729 GO:0005654 GO:0043484 GO:0006397 GO:0000166 |
| sp|Q9H6T3|RPAP3_ HUMAN | GO:0005515 |
| sp|Q9H6Y2|WDR55_ HUMAN | GO:0005737 GO:0005730 GO:0042273 GO:0006364 |
| sp|Q9H6Z4|RANB3_ HUMAN | GO:0005737 GO:0046907 GO:0005654 GO:0008536 GO:0070412 GO:0015031 |
| sp|Q9H773|DCTP1_ HUMAN | GO:0005829 GO:0032556 GO:0009143 GO:0042802 GO:0000287 GO:0051289 GO:0047840 GO:0015949 |
| sp|Q9H7D7|WDR26_ HUMAN | GO:0005654 GO:0005737 |
| sp|Q9H845|ACAD9_ HUMAN | GO:0052759 GO:0016937 GO:0052758 GO:0000062 GO:0005743 GO:0030425 GO:0052890 GO:0034916 GO:0052760 GO:0033539 GO:0070991 GO:0009055 GO:0043830 GO:0034845 GO:0022904 GO:0005515 GO:0050660 GO:0017099 GO:0055088 GO:0032981 GO:0043820 GO:0034580 GO:0005634 |
| sp|Q9H8Y8|GORS2_ HUMAN | GO:0000278 GO:0005797 GO:0007030 GO:0032580 GO:0000088 GO:0005515 |
| sp|Q9H936|GHC1_ HUMAN | GO:0006412 GO:0016021 GO:0005743 GO:0015293 GO:0003735 GO:0089711 GO:0005654 GO:0005313 |
| sp|Q9H944|MED20_ HUMAN | GO:0003899 GO:0006357 GO:0006367 GO:0035914 GO:0061630 GO:0016567 GO:0005515 GO:0001104 GO:0000151 GO:0016592 GO:0003713 |
| sp|Q9H974|QTRD1_ HUMAN | GO:0008270 GO:0005741 GO:0005515 GO:0008479 GO:0006400 GO:0008616 |
| sp|Q9H9B4|SFXN1_ HUMAN | GO:0016021 GO:0005743 GO:0030218 GO:0055072 GO:0006826 GO:0034220 GO:0015075 |
| sp|Q9HA65|TBC17_ HUMAN | GO:0042147 GO:0005829 GO:1902017 GO:0031410 GO:0005515 GO:0043547 GO:0005776 GO:0005096 GO:0006914 GO:0015031 |
| sp|Q9HA77|SYCM_ HUMAN | GO:0005829 GO:0046872 GO:0004817 GO:0006423 GO:0005524 GO:0005759 |
| sp|Q9HAB8|PPCS_ HUMAN | GO:0005829 GO:0015937 GO:0070062 GO:0004632 GO:0015939 |
| sp|Q9HAU0|PKHA5_ HUMAN | GO:0010314 GO:0061458 GO:0005545 GO:0005654 GO:0005515 GO:0070273 GO:0080025 GO:0016020 GO:0005829 GO:0032266 |
| sp|Q9HAU5|RENT2_ HUMAN | GO:0042162 GO:0035145 GO:0005829 GO:0003723 GO:0031100 GO:0001889 GO:0005515 GO:0006406 GO:0048471 GO:0006986 GO:0000184 |
| sp|Q9HAV7|GRPE1_ HUMAN | GO:0044267 GO:0000774 GO:0006457 GO:0051087 GO:0006626 GO:0005759 GO:0051082 GO:0042803 GO:0050790 GO:0005634 |
| sp|Q9HB07|MYG1_ HUMAN | GO:0005654 GO:0005515 GO:0043473 GO:0035641 GO:0070062 GO:0005739 |
| sp|Q9HB90|RRAGC_ HUMAN | GO:0006367 GO:0005654 GO:0034198 GO:0034613 GO:0008286 GO:0032006 GO:0016236 GO:0005525 GO:0005764 GO:0006915 GO:0005829 GO:0046982 GO:0007050 GO:0007264 GO:0008380 GO:1990131 GO:0000287 GO:0016049 GO:0019003 GO:0003924 GO:0071230 |
| sp|Q9HBL7|PLRKT_ HUMAN | GO:0006954 GO:0005739 GO:0006935 GO:0005887 GO:0005515 GO:0010756 |
| sp|Q9HC07|TM165_ HUMAN | GO:0031901 GO:0016021 GO:0005765 GO:0032472 GO:0006874 GO:0031902 GO:0035751 GO:0032588 GO:0006487 |
| sp|Q9HC35|EMAL4_ HUMAN | GO:0005737 GO:0007067 GO:0007017 GO:0016020 GO:0005874 |
| sp|Q9HC36|MRM3_ HUMAN | GO:0034933 GO:0052666 GO:0009008 GO:0043803 GO:0001510 GO:0043851 GO:0018707 GO:0018423 GO:0008172 GO:0016435 GO:0016428 GO:0005739 GO:0071424 GO:0006364 GO:0034931 GO:0016205 GO:0009019 GO:0016279 GO:0043834 GO:0043776 GO:0052624 GO:0043777 GO:0034807 GO:0052667 GO:0043833 GO:0043780 GO:0009383 GO:0008988 GO:0008174 GO:0030792 GO:0043827 GO:0051994 GO:0043791 GO:0004809 GO:0044822 GO:0000179 GO:0052735 GO:0034541 GO:0005515 GO:0043852 GO:0052665 GO:0043770 GO:0043782 GO:0019702 GO:0008425 GO:0080012 GO:0008650 GO:0070677 |
| sp|Q9HC52|CBX8_ HUMAN | GO:0005654 GO:0097027 GO:0000122 GO:0070301 GO:0006342 GO:0003727 GO:0016574 GO:0008284 GO:0000792 GO:0050790 GO:0035102 GO:0000790 GO:0016925 GO:0043687 GO:0035064 GO:0032967 GO:0045739 |
| sp|Q9HCD5|NCOA5_ HUMAN | GO:0042593 GO:0046627 GO:0005615 GO:0006355 GO:0044822 GO:0005515 GO:0015629 GO:0003682 GO:0005634 |
| sp|Q9HCY8|S10AE_ HUMAN | GO:0005654 GO:0032496 GO:0042127 GO:0034142 GO:0042379 GO:0015630 GO:0007283 GO:0048306 GO:0005509 GO:0042742 GO:0006915 GO:0055074 GO:0090026 GO:0030054 GO:0048471 GO:0070062 GO:0005886 GO:0071624 |
| sp|Q9HD33|RM47_ HUMAN | GO:0070126 GO:0005762 GO:0070125 GO:0005743 GO:0003735 GO:0070124 |
| sp|Q9HD43|PTPRH_ HUMAN | GO:0005737 GO:0005887 GO:0005001 GO:0005515 GO:0035335 GO:0006915 |
| sp|Q9HD47|MOG1_ HUMAN | GO:0002027 GO:0005654 GO:0032527 GO:2000010 GO:0090004 GO:0006913 GO:0008565 GO:0017080 GO:2000649 GO:0008536 GO:1900825 GO:0006888 GO:0005087 GO:0014704 GO:0043547 GO:0005901 GO:0044325 GO:0005791 |
| sp|Q9HDC9|APMAP_ HUMAN | GO:0009986 GO:0009058 GO:0004064 GO:0016021 GO:0005783 GO:0016844 GO:0070062 |
| sp|Q9NP79|VTA1_ HUMAN | GO:0005829 GO:0008022 GO:0070062 GO:1904903 GO:0071985 GO:0046755 GO:0016197 GO:0036258 GO:0010008 GO:0006914 GO:0015031 |
| sp|Q9NPD3|EXOS4_ HUMAN | GO:0030307 GO:0045006 GO:0000176 GO:0005515 GO:0017091 GO:0071051 GO:0090503 GO:0071028 GO:0051607 GO:0043928 GO:0000460 GO:0043488 GO:0005730 GO:0000177 GO:0005829 GO:0034475 GO:0034427 GO:0031125 GO:0071044 GO:0000175 GO:0016075 GO:0035327 |
| sp|Q9NPE3|NOP10_ HUMAN | GO:0019013 GO:0031429 GO:0090661 GO:0060215 GO:0072589 GO:1904874 GO:0005515 GO:0000454 GO:0007004 GO:0034513 GO:0070034 |
| sp|Q9NPF4|OSGEP_ HUMAN | GO:0005737 GO:0046872 GO:0061711 GO:0002949 GO:0005634 |
| sp|Q9NPJ3|ACO13_ HUMAN | GO:0035338 GO:0005829 GO:0034869 GO:0005739 GO:0070062 GO:0034843 GO:0019432 GO:0044466 GO:0005819 GO:0034946 GO:0051289 GO:0044281 GO:0005634 |
| sp|Q9NPQ8|RIC8A_ HUMAN | GO:0005829 GO:0008277 GO:0005085 GO:0071711 GO:0001944 GO:0007193 GO:0043547 GO:0008542 GO:0042074 GO:0005096 GO:0001701 GO:0070586 GO:0001965 GO:0005886 |
| sp|Q9NQ29|LUC7L_ HUMAN | GO:0003729 GO:0045843 GO:0050733 GO:0042802 GO:0006376 GO:0005685 GO:0071004 |
| sp|Q9NQ50|RM40_ HUMAN | GO:0070126 GO:0070125 GO:0005730 GO:0005743 GO:0005761 GO:0044822 GO:0005515 GO:0070124 GO:0009653 |
| sp|Q9NQ55|SSF1_ HUMAN | GO:0016021 GO:0005730 GO:0000027 GO:0019843 GO:0004930 GO:0001560 GO:0044822 GO:0030687 GO:0007186 |
| sp|Q9NQ88|TIGAR_ HUMAN | GO:0004331 GO:1904024 GO:1901525 GO:0033673 GO:0010332 GO:0045739 GO:0043069 GO:0005829 GO:0071456 GO:0043456 GO:1901215 GO:1903301 GO:0030388 GO:1902153 GO:0006367 GO:0005515 GO:0071279 GO:0010666 GO:0004083 GO:0005741 GO:2000378 GO:0002931 GO:0045820 GO:0006003 GO:0005634 GO:0016311 GO:0060576 GO:0009410 |
| sp|Q9NQC3|RTN4_ HUMAN | GO:0048011 GO:0030334 GO:0030176 GO:0043005 GO:0071786 GO:0045687 GO:0043234 GO:0097481 GO:0060317 GO:0033603 GO:0048709 GO:0051930 GO:0030517 GO:0007568 GO:0042981 GO:0005886 GO:0043209 GO:0044822 GO:0021553 GO:0070062 GO:0032403 GO:0014823 GO:0005635 GO:0070571 GO:0001525 GO:0021801 GO:0007413 GO:0043025 GO:2000172 |
| sp|Q9NQG5|RPR1B_ HUMAN | GO:0010564 GO:0045944 GO:0016591 GO:0000993 GO:0070940 GO:0005813 GO:0008284 GO:0005737 |
| sp|Q9NQH7|XPP3_ HUMAN | GO:0030145 GO:0003094 GO:0016485 GO:0004177 GO:0008237 GO:0070062 GO:0005739 |
| sp|Q9NQP4|PFD4_ HUMAN | GO:0051086 GO:0051087 GO:0044267 GO:0051082 GO:0016021 GO:0016272 GO:0005634 GO:0005829 GO:0005739 |
| sp|Q9NQW7|XPP1_ HUMAN | GO:0070006 GO:0030145 GO:0006508 GO:0005829 GO:0070062 GO:0042803 GO:0010815 |
| sp|Q9NQZ2|SAS10_ HUMAN | GO:0000462 GO:0005730 GO:0007420 GO:0044822 GO:0005515 GO:0032040 |
| sp|Q9NR12|PDLI7_ HUMAN | GO:0005925 GO:0008270 GO:0007275 GO:0033613 GO:0031941 GO:0006913 GO:0006898 GO:0043433 GO:0030036 GO:0001726 GO:0032880 GO:0001725 GO:0048471 GO:0030864 GO:0045669 GO:0005634 |
| sp|Q9NR30|DDX21_ HUMAN | GO:0009615 GO:0010501 GO:0045815 GO:0004004 GO:0097322 GO:0005730 GO:0006364 GO:0019843 GO:0003725 GO:0005524 GO:0044822 GO:0005515 GO:0030515 GO:0001649 GO:0043330 GO:0005654 GO:0016020 GO:0006366 |
| sp|Q9NR45|SIAS_ HUMAN | GO:0009103 GO:0005829 GO:0018279 GO:0008781 GO:0050462 GO:0047444 GO:0070062 GO:0043687 GO:0006488 |
| sp|Q9NR48|ASH1L_ HUMAN | GO:0046975 GO:0005654 GO:0008270 GO:0097676 GO:0005794 GO:0043124 GO:0032635 GO:0043409 GO:0003677 GO:0002674 GO:0051568 GO:0042800 GO:0045944 GO:0070062 GO:0003682 |
| sp|Q9NRG0|CHRC1_ HUMAN | GO:0003887 GO:0003677 GO:0008622 GO:0008623 GO:0046982 GO:0071897 GO:0006338 |
| sp|Q9NSE4|SYIM_ HUMAN | GO:0004822 GO:0016021 GO:0002161 GO:0006428 GO:0006450 GO:0005524 GO:0005759 |
| sp|Q9NSI2|F207A_ HUMAN | GO:0005515 |
| sp|Q9NT62|ATG3_ HUMAN | GO:0000422 GO:0070740 GO:0070738 GO:0043773 GO:0044804 GO:0000045 GO:0043774 GO:0000153 GO:0006612 GO:0008766 GO:0070736 GO:0005829 GO:0016567 GO:0019777 GO:0043653 GO:0070737 GO:0033554 GO:0019776 GO:0018169 GO:0019899 GO:1902017 GO:0050765 |
| sp|Q9NTJ5|SAC1_ HUMAN | GO:0052825 GO:0008969 GO:0008579 GO:0043812 GO:0004438 GO:0043813 GO:0030176 GO:0052830 GO:0030487 GO:0000139 GO:0043726 GO:0017161 GO:0017017 GO:0052629 GO:0019198 GO:0006661 GO:0030352 GO:0052828 GO:0046856 GO:0019178 GO:0052829 GO:0005515 GO:0008330 GO:0052831 GO:0052867 GO:0030351 GO:1990003 GO:0044281 GO:0032281 |
| sp|Q9NTZ6|RBM12_ HUMAN | GO:0044822 GO:0005654 GO:0000166 |
| sp|Q9NUJ1|ABHDA_ HUMAN | GO:0005829 GO:0052695 GO:0004553 GO:0006805 GO:0019391 GO:0005759 |
| sp|Q9NUL5|RYDEN_ HUMAN | GO:0005737 GO:0051607 GO:0034341 GO:0045071 GO:0003723 GO:0005515 GO:0034340 GO:0005634 GO:0034342 |
| sp|Q9NUQ3|TXLNG_ HUMAN | GO:0005829 GO:0010564 GO:0031965 GO:0006355 GO:0030500 GO:0046982 GO:0019905 |
| sp|Q9NV31|IMP3_ HUMAN | GO:0009653 GO:0030660 GO:0048027 GO:0034457 GO:0017148 GO:0051028 GO:0000166 GO:0003730 GO:0071556 GO:0005765 GO:0045903 GO:0031293 GO:0045182 GO:0005829 GO:0050776 GO:0031902 GO:0042500 GO:0006509 GO:0042035 GO:0030519 GO:0030515 GO:0003735 GO:0032040 GO:0070062 GO:0019013 GO:0042803 GO:0005886 GO:0006364 GO:0015935 GO:0071458 GO:0019843 |
| sp|Q9NVH1|DJC11_ HUMAN | GO:0005743 GO:0005515 GO:0070062 |
| sp|Q9NVI7|ATD3A_ HUMAN | GO:0016021 GO:0016049 GO:0005743 GO:0042645 GO:0005524 GO:0043066 |
| sp|Q9NVM6|DJC17_ HUMAN | GO:0003676 GO:0000122 GO:1901998 GO:0005737 GO:0005634 |
| sp|Q9NVM9|ASUN_ HUMAN | GO:0080154 GO:0030317 GO:0007067 GO:0090435 GO:0007052 GO:0051301 GO:0051642 GO:0005515 GO:0007275 GO:0005737 GO:0005634 GO:0007346 |
| sp|Q9NW64|RBM22_ HUMAN | GO:0005654 GO:0090316 GO:0000166 GO:0044822 GO:0048306 GO:0036002 GO:0005737 GO:0043009 GO:0005487 GO:0046872 GO:0071006 GO:0045292 GO:0000060 GO:0017070 GO:0035690 GO:0000974 GO:0033120 GO:0071007 GO:0000387 |
| sp|Q9NW82|WDR70_ HUMAN | GO:0019899 |
| sp|Q9NWH9|SLTM_ HUMAN | GO:0044822 GO:0005654 GO:0006351 |
| sp|Q9NWV4|CA123_ HUMAN | GO:0070062 |
| sp|Q9NX20|RM16_ HUMAN | GO:0070126 GO:0005762 GO:0070125 GO:0005743 GO:0019843 GO:0003735 GO:0070124 |
| sp|Q9NX24|NHP2_ HUMAN | GO:0031429 GO:0090661 GO:0072589 GO:1904874 GO:0044822 GO:0005515 GO:0007004 GO:0034513 GO:0070034 GO:0000784 GO:0005737 GO:0031118 |
| sp|Q9NX46|ARHL2_ HUMAN | GO:0046872 GO:0004649 GO:0005654 GO:0005759 GO:0008152 GO:0071451 |
| sp|Q9NX58|LYAR_ HUMAN | GO:0005730 GO:0046872 GO:0044822 GO:0005515 |
| sp|Q9NXG6|P4HTM_ HUMAN | GO:0016021 GO:0055114 GO:0005509 GO:0052634 GO:0043734 GO:0019798 GO:0000907 GO:0031418 GO:0045646 GO:0052635 GO:0018602 GO:0005789 GO:0005506 GO:0034792 |
| sp|Q9NXH8|TOR4A_ HUMAN | GO:0016021 GO:0005524 |
| sp|Q9NXN4|GDAP2_ HUMAN | GO:0005515 GO:0005765 GO:0032526 |
| sp|Q9NY33|DPP3_ HUMAN | GO:0005737 GO:0005654 GO:0008239 GO:0005886 GO:0005515 GO:0006508 GO:0008270 GO:0008237 GO:0070062 |
| sp|Q9NY61|AATF_ HUMAN | GO:0005925 GO:0007155 GO:0043066 GO:0044822 GO:0048011 GO:0042985 GO:0040016 GO:0006974 GO:0048156 GO:0005794 GO:0005730 GO:0043522 GO:0097190 GO:0005813 GO:0043065 GO:0045944 GO:0007346 GO:0032929 GO:0042254 GO:0003700 |
| sp|Q9NYH9|UTP6_ HUMAN | GO:0032040 GO:0030515 GO:0034388 GO:0000462 |
| sp|Q9NYK5|RM39_ HUMAN | GO:0070126 GO:0005762 GO:0070125 GO:0005743 GO:0003735 GO:0000002 GO:0044822 GO:0000166 GO:0070124 |
| sp|Q9NYL9|TMOD3_ HUMAN | GO:0030027 GO:0031941 GO:0048821 GO:0006936 GO:0051694 GO:0005865 GO:0030239 GO:0003779 GO:0001726 GO:0005523 GO:1901992 GO:0051271 GO:0051011 |
| sp|Q9NZ09|UBAP1_ HUMAN | GO:0005794 GO:0005886 GO:0043162 GO:0043130 GO:0000813 GO:0005829 GO:0015031 |
| sp|Q9NZB2|F120A_ HUMAN | GO:0044822 GO:0005737 GO:0005634 GO:0005886 |
| sp|Q9NZM1|MYOF_ HUMAN | GO:0030659 GO:0016021 GO:0031965 GO:0005901 GO:0030947 GO:0005543 GO:0001778 GO:0070062 GO:0005515 GO:0034605 GO:0008015 GO:0006936 |
| sp|Q9NZZ3|CHMP5_ HUMAN | GO:0071985 GO:0005515 GO:0007080 GO:1901673 GO:0007040 GO:0000920 GO:0036258 GO:0015031 GO:1904903 GO:0005829 GO:0046755 GO:0006997 GO:0008333 GO:0070062 GO:0010824 GO:0005634 GO:0010008 GO:0001919 |
| sp|Q9P013|CWC15_ HUMAN | GO:0005654 GO:0000398 GO:0005515 GO:0071013 |
| sp|Q9P015|RM15_ HUMAN | GO:0070126 GO:0005762 GO:0070125 GO:0031965 GO:0005743 GO:0003735 GO:0000002 GO:0044822 GO:0005515 GO:0070124 |
| sp|Q9P031|TAP26_ HUMAN | GO:0044267 GO:0006355 GO:0044822 GO:0005515 GO:0005654 |
| sp|Q9P035|HACD3_ HUMAN | GO:0005925 GO:0046726 GO:0007266 GO:0030497 GO:0007257 GO:0031965 GO:0005789 GO:0018812 GO:0042761 GO:0005096 GO:0016601 GO:0016021 GO:0045070 GO:0043547 GO:0019899 GO:0007249 GO:0005739 |
| sp|Q9P0I2|EMC3_ HUMAN | GO:0072546 GO:0045494 GO:0060041 GO:0030140 GO:0034975 |
| sp|Q9P0J0|NDUAD_ HUMAN | GO:0016021 GO:0045892 GO:0005654 GO:0043280 GO:0072593 GO:2001243 GO:0005747 GO:0044281 GO:0045039 GO:0097191 GO:0071300 GO:0070062 GO:0022904 GO:0005515 GO:0005524 GO:0035458 GO:0032981 GO:0030308 GO:0045732 GO:0008137 |
| sp|Q9P0J7|KCMF1_ HUMAN | GO:0016567 GO:0061630 GO:0070739 GO:0008270 GO:0070735 |
| sp|Q9P0L0|VAPA_ HUMAN | GO:0004871 GO:0034975 GO:0000139 GO:0044828 GO:0033149 GO:0031982 GO:0015630 GO:0044791 GO:0070972 GO:0005789 GO:0031175 GO:0043123 GO:0007029 GO:0046982 GO:0044829 GO:0070971 GO:0008017 GO:0061025 GO:0090114 GO:0016021 GO:0008219 GO:0044281 GO:0005923 GO:0048471 GO:0005886 GO:0030148 |
| sp|Q9P0M9|RM27_ HUMAN | GO:0070126 GO:0005762 GO:0070125 GO:0007049 GO:0005743 GO:0006915 GO:0003735 GO:0044822 GO:0005515 GO:0070124 |
| sp|Q9P1Y5|CAMP3_ HUMAN | GO:0005915 GO:0090136 GO:0051011 GO:0036449 GO:0034453 GO:0045218 GO:0005813 GO:0070507 GO:0005737 GO:0010923 |
| sp|Q9P2M7|CING_ HUMAN | GO:0008017 GO:0016459 GO:0003382 GO:0000226 GO:0005923 GO:0003774 GO:0003779 GO:0070830 GO:0007179 GO:0008152 |
| sp|Q9UBI6|GBG12_ HUMAN | GO:0008277 GO:0004871 GO:0021987 GO:0042301 GO:0070062 GO:0007626 GO:0005834 GO:0030165 GO:0007268 GO:0005884 GO:0007168 GO:0071377 GO:0006112 GO:0001662 GO:0044281 GO:0032496 |
| sp|Q9UBS4|DJB11_ HUMAN | GO:0033617 GO:0016556 GO:0044267 GO:0005739 GO:0006457 GO:0034663 GO:0032781 GO:0005788 GO:0051082 GO:0036498 GO:0016020 GO:0005634 |
| sp|Q9UBV8|PEF1_ HUMAN | GO:0005737 GO:0004198 GO:0005509 GO:0006508 GO:0016020 GO:0046982 GO:0070062 GO:0044822 GO:0051592 |
| sp|Q9UBW8|CSN7A_ HUMAN | GO:0005737 GO:0008180 GO:0005654 GO:0005515 GO:0006283 GO:0070911 GO:0000715 GO:0010388 |
| sp|Q9UFG5|CS025_ HUMAN | GO:0005515 |
| sp|Q9UH65|SWP70_ HUMAN | GO:0032233 GO:0033633 GO:0007204 GO:0030027 GO:0005515 GO:0030835 GO:0005509 GO:0005829 GO:0032880 GO:0003677 GO:0005856 GO:1902309 GO:0005886 GO:0060754 GO:0005634 GO:0005524 GO:0045190 |
| sp|Q9UHA4|LTOR3_ HUMAN | GO:0005925 GO:0007411 GO:0048010 GO:0006367 GO:0007265 GO:0016301 GO:0034613 GO:0008286 GO:0000186 GO:0038095 GO:0016236 GO:0048011 GO:0005765 GO:0007173 GO:0071986 GO:0007050 GO:0032008 GO:0019209 GO:0033554 GO:0070062 GO:0043547 GO:0008543 GO:0045087 GO:0071230 GO:0005085 GO:0032947 |
| sp|Q9UHB9|SRP68_ HUMAN | GO:0006412 GO:0005829 GO:0005786 GO:0030942 GO:0005840 GO:0005730 GO:0008312 GO:0044822 GO:0005925 GO:0005515 GO:0042493 GO:0006614 GO:0005783 GO:0005047 |
| sp|Q9UHD2|TBK1_ HUMAN | GO:0044565 GO:0033138 GO:0034142 GO:0051219 GO:0010629 GO:0051607 GO:0043123 GO:0035666 GO:0005829 GO:0003676 GO:0004674 GO:0032480 GO:0032727 GO:0006954 GO:1901214 GO:0034138 GO:0045944 GO:0016032 GO:1904417 GO:0050830 GO:0045359 GO:0005524 GO:0010008 |
| sp|Q9UHG3|PCYOX_ HUMAN | GO:0005764 GO:0055114 GO:0030327 GO:1902476 GO:0008555 GO:0030328 GO:0070062 GO:0005774 GO:0001735 GO:0034361 GO:0005886 |
| sp|Q9UHL4|DPP2_ HUMAN | GO:0004185 GO:0005794 GO:0008239 GO:0005764 GO:0006508 GO:0005829 GO:0016023 GO:0070062 |
| sp|Q9UHR4|BI2L1_ HUMAN | GO:0005654 GO:0051017 GO:0015629 GO:0007009 GO:0009617 GO:0005829 GO:0003779 GO:0070064 GO:0051764 GO:0046626 GO:0030838 GO:0070062 GO:0005886 GO:2000251 GO:0008093 |
| sp|Q9UHY7|ENOPH_ HUMAN | GO:0005829 GO:0019509 GO:0016311 GO:0070062 GO:0043874 GO:0000287 GO:0006595 GO:0005634 |
| sp|Q9UID3|VPS51_ HUMAN | GO:1990745 GO:0000938 GO:0005515 GO:0042147 GO:0006869 GO:0016021 GO:0032456 GO:0017119 GO:0006914 GO:0015031 |
| sp|Q9UIG0|BAZ1B_ HUMAN | GO:0003007 GO:0005654 GO:0008270 GO:2000273 GO:0004715 GO:0071884 GO:0048096 GO:0006333 GO:0035173 GO:0006302 GO:0000793 GO:0018108 GO:0070577 GO:0005721 GO:0016572 GO:0003682 GO:0005524 GO:0043596 |
| sp|Q9UII2|ATIF1_ HUMAN | GO:0009986 GO:0042803 GO:1903052 GO:0051289 GO:0006091 GO:0051117 GO:0032780 GO:0098779 GO:0072593 GO:0030272 GO:1903578 GO:0001937 GO:0035999 GO:0042030 GO:0005753 GO:0051882 GO:0005516 GO:0006783 GO:0001525 GO:0009396 GO:1901030 GO:0030218 GO:0043532 GO:1903214 |
| sp|Q9UIJ7|KAD3_ HUMAN | GO:0005758 GO:0004550 GO:0005759 GO:0006756 GO:0006172 GO:0046899 GO:0007517 GO:0005525 GO:0006165 GO:0007596 GO:0042802 GO:0046041 GO:0021549 GO:0042594 GO:0015949 GO:0042493 GO:0046060 GO:0046051 GO:0046039 GO:0070062 GO:0051260 GO:0001889 GO:0021772 GO:0005524 GO:0004017 |
| sp|Q9UJ70|NAGK_ HUMAN | GO:0006051 GO:0009384 GO:0045127 GO:0070062 GO:0019262 GO:0046835 GO:0005524 GO:0005515 GO:0006044 |
| sp|Q9UJ83|HACL1_ HUMAN | GO:0030976 GO:0051259 GO:0016830 GO:0042802 GO:0001561 GO:0005102 GO:0000287 GO:0005782 |
| sp|Q9UJC3|HOOK1_ HUMAN | GO:0070695 GO:0008017 GO:0030897 GO:0005874 GO:0008333 GO:0042802 GO:0007286 GO:0003779 GO:0045022 GO:0007275 GO:0007032 GO:0015031 GO:0007040 |
| sp|Q9UJC5|SH3L2_ HUMAN | GO:0070062 GO:0005654 GO:0017124 |
| sp|Q9UJS0|CMC2_ HUMAN | GO:0006094 GO:0005887 GO:0089712 GO:0006412 GO:0051592 GO:0005509 GO:0005743 GO:0043490 GO:0006754 GO:0005313 GO:0003735 GO:0089711 GO:0015183 |
| sp|Q9UJU6|DBNL_ HUMAN | GO:0030027 GO:0000139 GO:0006921 GO:0008022 GO:0048812 GO:0007257 GO:0006898 GO:0008047 GO:0030665 GO:0097178 GO:0005829 GO:0071800 GO:0030425 GO:0001726 GO:0019904 GO:0002250 GO:0016601 GO:0005769 GO:0014069 GO:0051015 GO:0030054 GO:0070062 GO:0005886 GO:0007416 GO:0002102 GO:0005938 |
| sp|Q9UJX2|CDC23_ HUMAN | GO:0005654 GO:0070979 GO:0005515 GO:0007080 GO:0005680 GO:0004842 GO:0005829 GO:0007096 GO:0000090 GO:0033554 GO:0031145 GO:0007094 GO:0051437 |
| sp|Q9UJY5|GGA1_ HUMAN | GO:0016192 GO:0044267 GO:0030131 GO:0045732 GO:0005515 GO:1901998 GO:0006886 GO:0010008 GO:0005654 GO:0005794 |
| sp|Q9UJZ1|STML2_ HUMAN | GO:0042101 GO:0034982 GO:0010918 GO:0010876 GO:0051646 GO:0006851 GO:0001772 GO:0090297 GO:0005102 GO:0015629 GO:0042776 GO:0005758 GO:0045121 GO:0006874 GO:0035710 GO:1900210 GO:0008180 GO:1990046 GO:0032623 GO:0019897 GO:0050852 GO:0051259 GO:0051020 GO:0005743 GO:1901612 |
| sp|Q9UK45|LSM7_ HUMAN | GO:0005829 GO:0005732 GO:0000398 GO:0097526 GO:0005688 GO:0017070 GO:0071004 GO:0046982 GO:0071013 GO:0005654 GO:1990726 GO:0005689 GO:0043928 |
| sp|Q9UK59|DBR1_ HUMAN | GO:0046872 GO:0000398 GO:0090502 GO:0008419 GO:0005634 GO:0044822 |
| sp|Q9UK76|HN1_ HUMAN | GO:0005730 GO:0031965 |
| sp|Q9UKG1|DP13A_ HUMAN | GO:0012506 GO:0008286 GO:0097192 GO:0090003 GO:0046324 GO:0008283 GO:0016581 GO:0005829 GO:0042802 GO:0043422 GO:0070062 GO:0031901 GO:0042593 GO:0007049 |
| sp|Q9ULC4|MCTS1_ HUMAN | GO:0040008 GO:0003743 GO:0001731 GO:0007049 GO:0032790 GO:0022627 GO:0006355 GO:0002192 GO:0008284 GO:0006974 GO:0005886 |
| sp|Q9ULC5|ACSL5_ HUMAN | GO:0009749 GO:0032869 GO:0001676 GO:2001236 GO:0005743 GO:0005789 GO:0009744 GO:0005730 GO:0010867 GO:0005741 GO:0004467 GO:0070723 GO:0016021 GO:0035338 GO:0010747 GO:0032000 GO:0008654 GO:0005524 GO:0007584 |
| sp|Q9ULX9|MAFF_ HUMAN | GO:0005654 GO:0035914 GO:0007596 GO:0043565 GO:0045604 GO:0001228 GO:0045944 GO:0007567 GO:0001701 GO:0005739 |
| sp|Q9UMS4|PRP19_ HUMAN | GO:0070740 GO:0005662 GO:0070738 GO:0043773 GO:0045665 GO:0034613 GO:0016020 GO:0000244 GO:0070534 GO:0016607 GO:0008610 GO:0034450 GO:0043774 GO:0072422 GO:0010498 GO:0006283 GO:0006303 GO:0008766 GO:0005737 GO:0070736 GO:0001833 GO:0071013 GO:0005819 GO:0042802 GO:0000245 GO:0048026 GO:0035861 GO:0000974 GO:0045666 GO:0048711 GO:0070737 GO:0005811 GO:0018169 |
| sp|Q9UMX5|NENF_ HUMAN | GO:0043410 GO:0046872 GO:0005615 GO:0032099 GO:0012505 GO:0005654 GO:0008083 GO:0016020 |
| sp|Q9UNE7|CHIP_ HUMAN | GO:0051865 GO:0005654 GO:0070740 GO:0070738 GO:0043773 GO:0030544 GO:0006457 GO:0030512 GO:0070534 GO:0032091 GO:0031647 GO:0034450 GO:0042787 GO:0043774 GO:0071218 GO:0051604 GO:0042405 GO:0051879 GO:0030579 GO:0030911 GO:0008766 GO:0031943 GO:0070736 GO:0030674 GO:0005829 GO:0045111 GO:0000151 GO:0032436 GO:0006515 GO:0036503 GO:0031625 GO:0001664 GO:0051787 GO:0070737 GO:0030018 GO:0051443 GO:0070062 GO:0042803 GO:0005783 GO:0031371 GO:0018169 GO:0005886 GO:1904264 GO:0030968 GO:0019900 GO:0046332 GO:0090035 GO:0006281 |
| sp|Q9UNN8|EPCR_ HUMAN | GO:0050819 GO:0008022 GO:0070062 GO:0004872 GO:0009986 GO:0005813 GO:0050900 GO:0006955 GO:0005887 GO:0005925 GO:0007596 |
| sp|Q9UNS2|CSN3_ HUMAN | GO:0070911 GO:0006511 GO:0006283 GO:0009416 GO:0010388 GO:0005515 GO:0008180 GO:0007165 GO:0005654 GO:0005737 GO:0001701 GO:0003351 GO:0000715 |
| sp|Q9UNW1|MINP1_ HUMAN | GO:0008969 GO:0006797 GO:0016311 GO:0052826 GO:0070062 GO:0048015 GO:0043647 GO:0003993 GO:0030352 GO:0030282 GO:0005788 GO:0030351 GO:0034417 GO:0005886 |
| sp|Q9UPP1|PHF8_ HUMAN | GO:0005654 GO:0035575 GO:0033169 GO:0045943 GO:0008270 GO:0071558 GO:0031965 GO:0005730 GO:0051864 GO:0007420 GO:0005506 GO:0061188 GO:0032454 GO:0071557 GO:0000082 GO:0035574 GO:0000088 GO:0055114 GO:0070544 GO:0003682 GO:0035064 |
| sp|Q9Y230|RUVB2_ HUMAN | GO:0000979 GO:0042803 GO:0071392 GO:0051082 GO:0006281 GO:0071339 GO:0016363 GO:0000980 GO:0051117 GO:0001094 GO:0071899 GO:0000812 GO:0071733 GO:0032508 GO:0005719 GO:0034644 GO:0043968 GO:0043967 GO:0005737 GO:0040008 GO:1904874 GO:0035066 GO:0070062 GO:0005524 GO:0006310 GO:0043141 GO:0031011 GO:0006338 GO:0030529 GO:0003684 GO:0043531 GO:0006457 GO:0031490 GO:0035267 GO:0071169 GO:0016020 |
| sp|Q9Y237|PIN4_ HUMAN | GO:0003681 GO:0003690 GO:0000413 GO:0005730 GO:0006364 GO:0070062 GO:0003755 GO:0044822 GO:0005515 GO:0005819 GO:0005759 GO:0030684 |
| sp|Q9Y265|RUVB1_ HUMAN | GO:0034080 GO:0006357 GO:0010756 GO:0007067 GO:0006281 GO:0071339 GO:0016363 GO:0051301 GO:0051117 GO:0001094 GO:0000812 GO:2000269 GO:0007283 GO:1903146 GO:0032508 GO:0043968 GO:0043967 GO:0040008 GO:1904874 GO:0070062 GO:0005794 GO:0005524 GO:0006310 GO:0005815 GO:0043141 GO:0031011 GO:0043140 GO:0030529 GO:1903955 GO:0043531 GO:0035267 GO:0016020 |
| sp|Q9Y266|NUDC_ HUMAN | GO:0005829 GO:0007097 GO:0016021 GO:0000910 GO:0007067 GO:0005874 GO:0007264 GO:0043434 GO:0000090 GO:0005515 GO:0000236 GO:0007275 GO:0005654 GO:0008283 GO:0005794 |
| sp|Q9Y277|VDAC3_ HUMAN | GO:0007270 GO:0008308 GO:0015853 GO:0000166 GO:0007612 GO:0015288 GO:0046930 GO:0005743 GO:0005741 GO:1903959 GO:0008021 GO:0001662 GO:0070062 GO:0005791 GO:0005634 GO:1902017 |
| sp|Q9Y295|DRG1_ HUMAN | GO:0055038 GO:0005844 GO:0006351 GO:0017137 GO:0043015 GO:0045296 GO:0005913 GO:0005525 GO:0032287 GO:0043209 GO:0005829 GO:0005874 GO:0008285 GO:0008017 GO:0071456 GO:0042802 GO:0005813 GO:0010038 GO:0045576 GO:0030330 GO:0048471 GO:0070062 GO:0090232 GO:0005886 GO:0008134 GO:0005634 |
| sp|Q9Y2A7|NCKP1_ HUMAN | GO:0005925 GO:0048010 GO:0007492 GO:0045176 GO:0007417 GO:0048617 GO:0001756 GO:0048340 GO:0031209 GO:0042074 GO:0050821 GO:0030950 GO:0006915 GO:0048570 GO:0035050 GO:0005829 GO:2000601 GO:0032880 GO:0045175 GO:0001843 GO:0016601 GO:0016021 GO:0038096 GO:0048365 GO:0031258 GO:0008078 GO:0010592 GO:0032403 GO:0070062 GO:0016032 GO:0010172 GO:0007354 GO:0045087 GO:0001701 |
| sp|Q9Y2P8|RCL1_ HUMAN | GO:0005730 GO:0004521 GO:0003723 GO:0000479 GO:0003963 |
| sp|Q9Y2Q5|LTOR2_ HUMAN | GO:0007411 GO:0048010 GO:0006367 GO:0007265 GO:0034613 GO:0008286 GO:0000186 GO:0038095 GO:0016236 GO:0048011 GO:0005765 GO:0007173 GO:0071986 GO:0007050 GO:0032008 GO:0033554 GO:0016049 GO:0070062 GO:0043547 GO:0008543 GO:0045087 GO:0071230 GO:0005085 GO:0032947 |
| sp|Q9Y2S0|RPAC2_ HUMAN | GO:0005829 GO:0006362 GO:0045815 GO:0005736 GO:0006386 GO:0045087 GO:0003677 GO:0006363 GO:0046983 GO:0006361 GO:0032481 GO:0001054 GO:0005666 GO:0001056 GO:0045814 GO:0006385 |
| sp|Q9Y2V2|CHSP1_ HUMAN | GO:0003730 GO:0005829 GO:0000177 GO:0000932 GO:0070062 GO:0006355 GO:0003677 GO:0019902 GO:0043488 GO:0043186 GO:0019722 |
| sp|Q9Y2W1|TR150_ HUMAN | GO:0006367 GO:0042809 GO:0046966 GO:0000166 GO:0016607 GO:0016592 GO:0000381 GO:0001104 GO:0051219 GO:0044822 GO:0001046 GO:0030521 GO:0000956 GO:0035145 GO:0048255 GO:0048026 GO:0045944 GO:0070062 GO:0004872 GO:0042753 GO:0030374 |
| sp|Q9Y2X3|NOP58_ HUMAN | GO:0006608 GO:0015030 GO:0016049 GO:0000154 GO:0016925 GO:0001094 GO:0043687 GO:0044822 GO:0030515 GO:0032040 GO:0048254 GO:0030519 GO:0051117 GO:0070761 GO:0031428 GO:0005737 GO:0016020 |
| sp|Q9Y2Z4|SYYM_ HUMAN | GO:0005829 GO:0005524 GO:0044822 GO:0072545 GO:0070184 GO:0005759 GO:0000049 GO:0042803 GO:0004831 |
| sp|Q9Y305|ACOT9_ HUMAN | GO:0035338 GO:0034869 GO:0003986 GO:0034843 GO:0052689 GO:0005515 GO:0019432 GO:0016290 GO:0044466 GO:0005759 GO:0034946 GO:0044281 |
| sp|Q9Y315|DEOC_ HUMAN | GO:0046121 GO:0070062 GO:0004139 GO:0016052 GO:0046386 GO:0005737 GO:0009264 GO:0005634 |
| sp|Q9Y320|TMX2_ HUMAN | GO:0005623 GO:0045454 GO:0016021 |
| sp|Q9Y333|LSM2_ HUMAN | GO:0005829 GO:0071011 GO:0022008 GO:0000932 GO:0000244 GO:0017160 GO:0000381 GO:0005688 GO:0044822 GO:0017070 GO:0019901 GO:0046540 GO:0071013 GO:0005654 GO:1990726 GO:0043928 |
| sp|Q9Y388|RBMX2_ HUMAN | GO:0071011 GO:0005686 GO:0070274 GO:0000398 GO:0000384 GO:0044822 GO:0005515 GO:0000166 GO:0006406 GO:0071013 |
| sp|Q9Y3B2|EXOS1_ HUMAN | GO:0000176 GO:0005829 GO:0003723 GO:0005730 GO:0006364 GO:0043488 GO:0005515 GO:0005654 GO:0004532 GO:0043928 |
| sp|Q9Y3B4|SF3B6_ HUMAN | GO:0055114 GO:0008270 GO:0071011 GO:0005686 GO:0001825 GO:0000398 GO:0016491 GO:0005684 GO:0044822 GO:0000166 GO:0071013 GO:0005654 GO:0005689 |
| sp|Q9Y3B7|RM11_ HUMAN | GO:0070126 GO:0005762 GO:0070125 GO:0005743 GO:0000027 GO:0019843 GO:0003735 GO:0044822 GO:0005515 GO:0070124 |
| sp|Q9Y3B8|ORN_ HUMAN | GO:0003676 GO:0005730 GO:0043765 GO:0009117 GO:0005758 GO:0005925 GO:0044824 GO:0090503 GO:0005759 GO:0042578 GO:0000175 |
| sp|Q9Y3B9|RRP15_ HUMAN | GO:0005730 GO:0005739 |
| sp|Q9Y3C1|NOP16_ HUMAN | GO:0005730 GO:0044822 GO:0042273 |
| sp|Q9Y3C8|UFC1_ HUMAN | GO:0034976 GO:0071568 GO:0005515 GO:1990592 GO:0070062 |
| sp|Q9Y3D0|MIP18_ HUMAN | GO:0007059 GO:0005654 GO:0016226 GO:0005515 GO:0044281 GO:0097361 GO:0071817 |
| sp|Q9Y3D6|FIS1_ HUMAN | GO:0070584 GO:0000422 GO:0007204 GO:0005102 GO:0031307 GO:0090141 GO:0051561 GO:0035584 GO:0005829 GO:0016559 GO:0043234 GO:0008053 GO:0032471 GO:0043653 GO:0090314 GO:0005779 GO:0051260 GO:0005783 GO:0001836 GO:0043280 GO:0006626 GO:2001244 |
| sp|Q9Y3F4|STRAP_ HUMAN | GO:0005654 GO:0000122 GO:0005102 GO:0030512 GO:0044822 GO:0010633 GO:0030277 GO:0010719 GO:0060394 GO:0034719 GO:0005829 GO:0032797 GO:0050680 GO:0005886 GO:0000387 |
| sp|Q9Y3I0|RTCB_ HUMAN | GO:0003972 GO:0017166 GO:0046872 GO:0001890 GO:0072669 GO:0005524 GO:0044822 GO:0005635 GO:0005654 GO:0005789 GO:0001701 GO:0006388 |
| sp|Q9Y3P9|RBGP1_ HUMAN | GO:0005829 GO:0016021 GO:0005875 GO:0017137 GO:0007049 GO:0004930 GO:1902017 GO:0005813 GO:0003677 GO:0043547 GO:0015631 GO:0007186 GO:0005096 |
| sp|Q9Y3Q8|T22D4_ HUMAN | GO:0006970 GO:0005515 GO:0005737 GO:0045892 GO:0003700 GO:0005634 |
| sp|Q9Y3U8|RL36_ HUMAN | GO:0000184 GO:0006415 GO:0006413 GO:0016020 GO:0002181 GO:0044822 GO:0016259 GO:0006414 GO:0022625 GO:0005730 GO:0003735 GO:0019083 GO:0006614 |
| sp|Q9Y3Y2|CHTOP_ HUMAN | GO:0031981 GO:0006810 GO:0044260 GO:0044699 GO:0010467 GO:0009893 GO:0003676 GO:0044238 GO:0050794 |
| sp|Q9Y3Z3|SAMH1_ HUMAN | GO:0005654 GO:0032567 GO:0005515 GO:0008270 GO:0051289 GO:0051607 GO:0046061 GO:0008832 GO:0045088 GO:0060337 GO:0003723 GO:0005886 GO:0006203 |
| sp|Q9Y446|PKP3_ HUMAN | GO:0090002 GO:0016337 GO:0030057 GO:0005886 GO:0050839 GO:0002159 GO:0045294 GO:0005914 GO:0005634 |
| sp|Q9Y490|TLN1_ HUMAN | GO:0005925 GO:0007411 GO:0005178 GO:0005911 GO:0036498 GO:0032587 GO:0005815 GO:0007043 GO:0007016 GO:0070527 GO:0044267 GO:0006936 GO:0005829 GO:0030274 GO:0009986 GO:0007044 GO:0002576 GO:0051015 GO:0017166 GO:0070062 GO:0016032 GO:0030866 GO:0005200 |
| sp|Q9Y4W2|LAS1L_ HUMAN | GO:0005737 GO:0005730 GO:0005815 GO:0006364 GO:0071339 GO:0016020 GO:0044822 |
| sp|Q9Y512|SAM50_ HUMAN | GO:0016021 GO:0044267 GO:0005743 GO:0070062 GO:0045040 GO:0005515 GO:0033108 GO:0001401 GO:0042407 |
| sp|Q9Y5A9|YTHD2_ HUMAN | GO:0005829 GO:1990247 GO:1903679 GO:0000932 GO:0006959 GO:0044822 GO:0043488 GO:0005515 GO:0005634 |
| sp|Q9Y5B9|SP16H_ HUMAN | GO:0005654 GO:0005515 GO:0044822 GO:0006337 GO:0032786 GO:0016787 GO:0005694 GO:0050434 GO:0006368 GO:0006260 GO:0006281 |
| sp|Q9Y5J1|UTP18_ HUMAN | GO:0032040 GO:0006364 GO:0034388 GO:0044822 |
| sp|Q9Y5K6|CD2AP_ HUMAN | GO:0005911 GO:0007067 GO:0008022 GO:0031941 GO:0006461 GO:0045296 GO:0048259 GO:0005172 GO:2000249 GO:0051301 GO:0032911 GO:0005730 GO:0016050 GO:0043161 GO:0001726 GO:0030139 GO:0007165 GO:0006930 GO:0016337 GO:0048471 GO:0032403 GO:0070062 GO:1900182 GO:0008013 GO:0005886 GO:0017124 GO:0005200 GO:0005938 |
| sp|Q9Y5L4|TIM13_ HUMAN | GO:0072321 GO:0044267 GO:0008270 GO:0005730 GO:0005743 GO:0006626 GO:0042719 GO:0007605 |
| sp|Q9Y5V0|ZN706_ HUMAN | GO:0006417 GO:0046872 GO:1902455 GO:0005737 GO:0045892 GO:0005634 |
| sp|Q9Y5X3|SNX5_ HUMAN | GO:0042147 GO:0031901 GO:0030659 GO:0006907 GO:0001726 GO:0070685 GO:0097422 GO:0034452 GO:0035091 GO:0031313 GO:0030905 GO:0001891 GO:0046982 GO:0006886 GO:0031234 GO:0016050 |
| sp|Q9Y608|LRRF2_ HUMAN | GO:0030275 GO:0016055 |
| sp|Q9Y639|NPTN_ HUMAN | GO:0050839 GO:1900273 GO:0007204 GO:0070374 GO:0008542 GO:0060077 GO:0005105 GO:0042734 GO:0007156 GO:0030155 GO:0045743 GO:0005829 GO:0045211 GO:0009986 GO:1903829 GO:0030425 GO:0048170 GO:0018108 GO:0016021 GO:0014069 GO:0004714 GO:0010976 GO:0044325 |
| sp|Q9Y678|COPG1_ HUMAN | GO:0030126 GO:0005829 GO:0018279 GO:0030133 GO:0006890 GO:0006891 GO:0043687 GO:0072384 GO:0005515 GO:0006888 GO:0048205 GO:0006886 GO:0005198 GO:0051683 |
| sp|Q9Y6C9|MTCH2_ HUMAN | GO:0070585 GO:0016021 GO:0005743 GO:0097284 GO:0070062 GO:1902108 GO:0005741 GO:0005634 |
| sp|Q9Y6D9|MD1L1_ HUMAN | GO:0072686 GO:0005515 GO:0000236 GO:0015629 GO:0090235 GO:0000777 GO:0042130 GO:0005829 GO:0005643 GO:0007264 GO:0005813 GO:0048538 GO:0000090 GO:0007094 GO:0000922 |
| sp|Q9Y6M9|NDUB9_ HUMAN | GO:0006810 GO:0007605 GO:0005515 GO:0008137 GO:0006120 GO:0032981 GO:0005747 GO:0070062 GO:0098779 |
| sp|Q9Y6U3|ADSV_ HUMAN | GO:0005546 GO:0042989 GO:0051127 GO:0051047 GO:0001786 GO:0005509 GO:0051014 GO:0008285 GO:0005545 GO:0017156 GO:0043065 GO:0043234 GO:0045654 GO:0005856 GO:0005903 GO:0051693 GO:0051015 GO:0070062 GO:0032330 GO:0005938 |
| sp|Q9Y6W5|WASF2_ HUMAN | GO:0001525 GO:0051497 GO:0048010 GO:0001667 GO:0005911 GO:0072673 GO:0030027 GO:0030048 GO:0035855 GO:0015629 GO:0031209 GO:0005829 GO:0003779 GO:0001726 GO:0016601 GO:0005769 GO:0038096 GO:0010592 GO:0032403 GO:0070062 GO:0016032 GO:0007188 GO:0045087 |
| sp|Q9Y6X8|ZHX2_ HUMAN | GO:0005654 GO:0000122 GO:0003714 GO:0045665 GO:0005737 GO:0005730 GO:0046982 GO:0046872 GO:0006402 GO:0003677 GO:0035019 GO:0042803 GO:0005886 GO:0003700 |
| sp|Q9Y6Y0|NS1BP_ HUMAN | GO:0009615 GO:0005681 GO:0006383 GO:0005667 GO:0008380 GO:0005515 GO:0015629 GO:2001243 GO:0016032 GO:0005654 GO:0005737 |
| tr|A0A023T787|A0A023T787_ HUMAN | GO:0000184 GO:0005515 GO:0000166 GO:0006406 GO:0016607 GO:0000381 GO:0035145 GO:0031124 GO:0005829 GO:0071013 GO:0030425 GO:0043025 GO:0006369 GO:0006417 GO:0003729 |
| tr|A0A024QYR8|A0A024QYR8_ HUMAN | GO:0005887 GO:0006810 GO:0010008 GO:0070062 |
| tr|A0A024QYS2|A0A024QYS2_ HUMAN | GO:0016021 |
| tr|A0A024QYT6|A0A024QYT6_ HUMAN | GO:0006892 GO:0050885 GO:0006281 GO:0031625 GO:0043161 GO:0008565 GO:0005829 GO:0036465 GO:0042147 GO:0050690 GO:0008542 GO:0006898 GO:0045214 GO:0032588 GO:0019886 GO:0045444 GO:0043195 GO:0070062 GO:0016032 GO:0006886 GO:0060612 GO:0006515 GO:0071218 GO:0005524 GO:0005765 GO:0030121 GO:0030669 GO:0061024 GO:0061630 GO:0005634 GO:0005905 GO:0000209 GO:0006513 |
| tr|A0A024QYW3|A0A024QYW3_ HUMAN | GO:0016021 GO:0006935 GO:0070062 GO:0019221 GO:0034220 GO:0005789 GO:0015075 GO:0019956 GO:0005886 |
| tr|A0A024QYX3|A0A024QYX3_ HUMAN | GO:0005654 GO:0005515 GO:0000166 GO:0044822 GO:0043023 GO:0015934 GO:0045727 GO:0005730 GO:0030425 GO:0009409 GO:0035196 |
| tr|A0A024QZ30|A0A024QZ30_ HUMAN | GO:0006810 GO:0006105 GO:0043209 GO:0005749 GO:0006099 GO:0007399 GO:0022904 GO:0005515 GO:0050660 GO:0008177 |
| tr|A0A024QZ70|A0A024QZ70_ HUMAN | GO:0042981 GO:0000186 GO:0030295 GO:0000236 GO:0019901 GO:0097194 GO:0031572 GO:0051493 GO:0051301 GO:0005829 GO:0007264 GO:0031577 GO:0004709 GO:0046330 GO:0000090 GO:0070062 GO:0007346 GO:0005634 GO:0005524 GO:0006281 |
| tr|A0A024QZ77|A0A024QZ77_ HUMAN | GO:0005515 GO:0005509 GO:0016020 |
| tr|A0A024QZ78|A0A024QZ78_ HUMAN | GO:0033388 GO:0008295 GO:0005739 GO:0046872 GO:0070062 GO:0097055 GO:0008783 |
| tr|A0A024QZD5|A0A024QZD5_ HUMAN | GO:0048731 GO:0005654 GO:0043462 GO:0005515 GO:0071004 GO:0071011 GO:0005737 GO:0061084 GO:0000243 GO:0048026 GO:1904715 GO:1990446 GO:0005685 GO:0030619 GO:0003729 |
| tr|A0A024QZF2|A0A024QZF2_ HUMAN | GO:0005925 GO:0007411 GO:0051896 GO:0045199 GO:0007265 GO:0030336 GO:0007219 GO:0070372 GO:0045766 GO:0060325 GO:0005525 GO:0005622 GO:0007268 GO:0032403 GO:0070062 GO:0019003 GO:0002521 GO:0003924 GO:0005886 |
| tr|A0A024QZF6|A0A024QZF6_ HUMAN | GO:0005654 GO:0043523 GO:0005515 GO:0032007 GO:0008286 GO:1900034 GO:0038095 GO:0006469 GO:0048011 GO:0007173 GO:0005829 GO:0048015 GO:0045792 GO:0008543 GO:0045087 GO:0031931 |
| tr|A0A024QZH6|A0A024QZH6_ HUMAN | GO:0019904 GO:0006397 GO:0006366 |
| tr|A0A024QZM5|A0A024QZM5_ HUMAN | GO:0004725 GO:0005515 GO:0035335 GO:0005634 GO:0005829 |
| tr|A0A024QZN2|A0A024QZN2_ HUMAN | GO:0005737 GO:0005615 GO:0005886 GO:0031072 GO:0032781 GO:0035176 GO:0005634 |
| tr|A0A024QZN4|A0A024QZN4_ HUMAN | GO:0005925 GO:0045121 GO:0030336 GO:0030032 GO:0005198 GO:0005884 GO:0031594 GO:0007160 GO:0030486 GO:1990357 GO:0005927 GO:0045296 GO:0043034 GO:0070527 GO:0005743 GO:0090136 GO:0006936 GO:0005915 GO:0034394 GO:0045294 GO:0005829 GO:0034333 GO:0048675 GO:0042383 GO:0043297 GO:0002009 GO:0031625 GO:0090637 GO:0002162 GO:0043234 GO:0005903 GO:0017048 GO:0002576 GO:0051371 GO:0051015 GO:0001725 GO:0090636 GO:0017166 GO:0030018 GO:0070062 GO:0042803 GO:0005916 GO:0008013 GO:0098723 GO:0097110 |
| tr|A0A024QZN9|A0A024QZN9_ HUMAN | GO:0045121 GO:0008308 GO:0005515 GO:0000166 GO:0015288 GO:0046930 GO:2001243 GO:0005743 GO:0043209 GO:0042645 GO:0005741 GO:1903959 GO:0008021 GO:0032272 GO:0070062 GO:0005634 |
| tr|A0A024QZR5|A0A024QZR5_ HUMAN | GO:0048490 GO:0030659 GO:0017137 GO:0005765 GO:0030424 GO:0030131 GO:0006622 GO:0005794 |
| tr|A0A024QZT4|A0A024QZT4_ HUMAN | GO:0010628 GO:0016020 GO:0001913 GO:0042270 GO:0043027 GO:0005615 GO:0004867 GO:0042742 GO:0070233 GO:0005829 GO:0002448 GO:0071391 GO:0043154 GO:0033668 GO:0002438 GO:0002020 GO:0070062 GO:0008233 GO:0005634 |
| tr|A0A024QZU0|A0A024QZU0_ HUMAN | GO:0004674 GO:0097527 GO:0005123 GO:0034138 GO:0032481 GO:2001240 GO:0034142 GO:0031625 GO:2000379 GO:0070231 GO:0060545 GO:0045121 GO:0043124 GO:1902041 GO:0051260 GO:0043123 GO:0010803 GO:0045944 GO:0036289 GO:0044257 GO:0043235 GO:0016032 GO:0032403 GO:1990000 GO:0042802 GO:0005739 GO:0051092 GO:0005524 GO:0007257 GO:0043154 GO:0070513 GO:0071550 GO:0035666 GO:1901026 GO:2001238 GO:0031264 GO:0071363 GO:0032757 GO:0032760 GO:0097296 GO:0051291 GO:0097342 GO:0045651 GO:0070926 |
| tr|A0A024QZW3|A0A024QZW3_ HUMAN | GO:0007411 GO:0042393 GO:0008565 GO:0070373 GO:0008536 GO:0005829 GO:0007020 GO:0005635 GO:0005813 GO:0005875 GO:0042991 GO:0005886 |
| tr|A0A024QZW7|A0A024QZW7_ HUMAN | GO:0051292 GO:0031047 GO:0008139 GO:0005654 GO:0007077 GO:0046718 GO:0051028 GO:0075732 GO:0007067 GO:0008270 GO:1900034 GO:0034399 GO:0044615 GO:0031965 GO:0010827 GO:0042405 GO:0008536 GO:0015031 GO:0005737 GO:0005730 GO:0005487 GO:0019221 GO:0005975 GO:0042802 GO:0008033 GO:0044281 GO:0043495 GO:0019083 GO:0005642 GO:0016925 GO:0000088 GO:0055085 GO:0003690 GO:0017056 GO:0003682 GO:0043687 GO:0019054 GO:0046832 |
| tr|A0A024R012|A0A024R012_ HUMAN | GO:0036047 GO:0008270 GO:0006476 GO:0010667 GO:0005758 GO:0005759 GO:0036055 GO:0070403 GO:0036049 GO:0005743 GO:0006342 GO:0005829 GO:0003950 GO:0061697 GO:0061699 GO:2000378 GO:0010566 GO:0005634 GO:0036054 GO:0031667 GO:0006471 |
| tr|A0A024R029|A0A024R029_ HUMAN | GO:0005515 GO:0000480 GO:0000166 GO:0006357 GO:0044822 GO:0000447 GO:0005730 GO:0005667 GO:0003713 GO:0003677 GO:0000472 GO:0021522 GO:0034462 |
| tr|A0A024R084|A0A024R084_ HUMAN | GO:0005796 GO:0005509 GO:0032059 GO:0045471 GO:0009650 GO:0070062 GO:0042802 GO:0021549 GO:0070625 GO:0005770 GO:0005886 GO:0045444 |
| tr|A0A024R0A8|A0A024R0A8_ HUMAN | GO:0031440 GO:0005515 GO:0004013 GO:0038166 GO:0051592 GO:0010765 GO:0032412 GO:0006611 GO:0005789 GO:0042045 GO:1903779 GO:0005829 GO:0006730 GO:0003723 GO:0033353 GO:0009953 GO:0070062 GO:0044070 GO:0048598 GO:0006378 GO:0016324 |
| tr|A0A024R0G0|A0A024R0G0_ HUMAN | GO:0005794 GO:0005254 GO:1902476 GO:0005634 GO:0005783 GO:0005739 GO:0034707 |
| tr|A0A024R0G2|A0A024R0G2_ HUMAN | GO:0005737 GO:0017112 GO:0005515 GO:0043547 |
| tr|A0A024R0H6|A0A024R0H6_ HUMAN | GO:0031442 GO:0050680 GO:0045638 GO:0005779 GO:0019827 GO:0001711 GO:0016584 GO:0030054 GO:0016593 GO:0016055 GO:0032968 GO:0042632 GO:0016558 GO:0071222 GO:0045540 GO:0031062 GO:0010390 GO:0031648 GO:0008270 GO:0000122 GO:0003682 GO:0000993 GO:0006378 GO:0048147 GO:0034504 GO:0006635 GO:0006699 GO:0033523 GO:0001764 GO:0000038 |
| tr|A0A024R0H7|A0A024R0H7_ HUMAN | GO:0005654 GO:0005515 GO:0006357 GO:0060528 GO:0005794 GO:0005829 GO:0008284 GO:0034660 GO:0034709 GO:0060770 GO:0008327 GO:0030374 GO:0006325 GO:0000387 |
| tr|A0A024R0J1|A0A024R0J1_ HUMAN | GO:0005925 GO:0031098 GO:0007266 GO:0060996 GO:0042981 GO:0071407 GO:0008283 GO:0043408 GO:0005794 GO:0032147 GO:0006355 GO:0030036 GO:0004674 GO:0048365 GO:0016049 GO:0007346 GO:0005524 GO:0016477 |
| tr|A0A024R0J9|A0A024R0J9_ HUMAN | GO:0009615 GO:0019013 GO:0000398 GO:0006355 GO:0030529 GO:0019899 GO:0044822 GO:0005654 |
| tr|A0A024R0L6|A0A024R0L6_ HUMAN | GO:0042802 GO:0007283 GO:0003847 GO:0016042 GO:0047179 GO:0007420 GO:0016020 GO:0005829 GO:0046982 GO:0070062 |
| tr|A0A024R0P9|A0A024R0P9_ HUMAN | GO:0000423 GO:0044267 GO:0015288 GO:0030150 GO:0005742 GO:0031307 GO:0015266 GO:0070062 GO:0006811 GO:0046930 GO:0005654 |
| tr|A0A024R0Q0|A0A024R0Q0_ HUMAN | GO:0042802 GO:0000184 GO:0010467 GO:0005829 |
| tr|A0A024R0Q4|A0A024R0Q4_ HUMAN | GO:0016021 GO:0006655 GO:0070290 GO:0016042 GO:0070062 GO:0004630 GO:0005515 GO:0044281 GO:0005789 |
| tr|A0A024R0Q5|A0A024R0Q5_ HUMAN | GO:0000122 GO:0003714 GO:0042633 GO:0003215 GO:0045597 GO:0060048 GO:0009791 GO:0006915 GO:0005737 GO:0042802 GO:0003229 GO:0045171 GO:0031076 GO:0030054 GO:0035264 GO:0008134 GO:0005634 GO:0048871 |
| tr|A0A024R0Q7|A0A024R0Q7_ HUMAN | GO:0048011 GO:0004871 GO:0010288 GO:0007067 GO:0008289 GO:0007411 GO:0048010 GO:0000186 GO:0043234 GO:0006351 GO:0046872 GO:0005829 GO:0005654 GO:0016576 GO:0001933 GO:1901215 GO:0070301 GO:0043123 GO:2000324 GO:0045087 GO:0007265 GO:0005886 GO:0043204 GO:0042802 GO:0007173 GO:0005524 GO:0038095 GO:0006302 GO:0003723 GO:0004722 GO:0001965 GO:1990635 GO:0043278 GO:0051291 GO:0008543 GO:0043531 GO:0071276 GO:0008017 GO:0031072 GO:0008286 |
| tr|A0A024R0R1|A0A024R0R1_ HUMAN | GO:0005089 GO:0005829 GO:0048011 GO:0044822 GO:0005515 GO:0043547 GO:0050771 GO:0035023 GO:0008283 GO:0005096 GO:0060548 GO:0005886 |
| tr|A0A024R0R9|A0A024R0R9_ HUMAN | GO:0005774 GO:0005483 GO:0045176 GO:0043129 GO:0097486 GO:0000139 GO:0006891 GO:0097208 GO:0005615 GO:0005764 GO:0043209 GO:0006886 GO:0035249 GO:0005829 GO:0007420 GO:0035494 GO:0061025 GO:0010807 GO:0048208 GO:0004190 GO:0030163 GO:0006892 GO:0043195 GO:0070044 GO:0070062 GO:0043687 GO:0018279 GO:0033619 GO:0019905 GO:0030182 |
| tr|A0A024R0T1|A0A024R0T1_ HUMAN | GO:0018450 GO:0045703 GO:0034871 GO:0005576 GO:0018449 GO:0034868 GO:0034778 GO:0018448 GO:0034944 GO:0034522 GO:0018447 GO:0042469 GO:0034821 GO:0055114 GO:0034847 GO:0034817 GO:0034918 GO:0034901 GO:0034582 GO:0034891 GO:0018446 GO:0034863 |
| tr|A0A024R120|A0A024R120_ HUMAN | GO:0045893 GO:0006357 GO:0007420 GO:0005667 GO:0008022 GO:0043565 GO:0071542 GO:0003700 GO:0005634 |
| tr|A0A024R151|A0A024R151_ HUMAN | GO:0055085 GO:0016021 GO:0006656 GO:0015220 GO:0015871 GO:0070062 GO:0005741 GO:0005654 GO:0005886 |
| tr|A0A024R163|A0A024R163_ HUMAN | GO:0045595 GO:0043249 GO:0044822 GO:0048025 GO:0000166 GO:0009653 GO:0005634 |
| tr|A0A024R172|A0A024R172_ HUMAN | GO:0009636 GO:0055114 GO:0008270 GO:0036132 GO:0070062 GO:0047522 GO:0006691 GO:0019371 GO:0032440 GO:0005737 GO:2001300 |
| tr|A0A024R179|A0A024R179_ HUMAN | GO:0031047 GO:0000184 GO:0030307 GO:0005654 GO:0006370 GO:0005515 GO:0006406 GO:0031442 GO:0044822 GO:0008334 GO:0098789 GO:0000340 GO:0005829 GO:0005845 GO:0045292 GO:0034660 GO:0006369 GO:0006446 GO:0005846 GO:0050434 GO:0030529 GO:1900363 GO:0006368 GO:0005739 GO:0000387 |
| tr|A0A024R184|A0A024R184_ HUMAN | GO:0001938 GO:0032007 GO:0000033 GO:0031965 GO:0045766 GO:0036324 GO:0048306 GO:0051592 GO:0006919 GO:0047485 GO:0033577 GO:0005509 GO:0005789 GO:0060090 GO:0006488 GO:0006886 GO:0046982 GO:0070971 GO:0097190 GO:0051898 GO:0031410 GO:0030948 GO:0016021 GO:0005768 GO:0043495 GO:0034605 GO:0010595 GO:0048471 GO:0070062 GO:0042803 GO:0004198 GO:0043687 GO:0004378 GO:0097502 GO:0018279 |
| tr|A0A024R1A3|A0A024R1A3_ HUMAN | GO:0005515 GO:0007409 GO:0044822 GO:0004842 GO:0006974 GO:0004839 GO:0005765 GO:0005829 GO:0006511 GO:0030057 GO:0016567 GO:0000792 GO:0030867 GO:0070062 GO:0005634 GO:0005524 GO:0010008 GO:0005739 |
| tr|A0A024R1A4|A0A024R1A4_ HUMAN | GO:0097027 GO:0070979 GO:0071385 GO:0042787 GO:0044822 GO:0008283 GO:0005737 GO:0000151 GO:0006355 GO:0044770 GO:0061631 GO:1903955 GO:0031625 GO:0003713 GO:0051443 GO:0070062 GO:0005634 GO:0005524 |
| tr|A0A024R1D6|A0A024R1D6_ HUMAN | GO:0005654 GO:0005515 GO:0000784 GO:0006397 GO:0000902 GO:0032786 GO:0001824 GO:0046784 GO:0005737 GO:0060215 GO:0045650 GO:0008380 GO:0000445 GO:0010793 GO:2000035 GO:0030224 GO:2000002 GO:0003729 |
| tr|A0A024R1I3|A0A024R1I3_ HUMAN | GO:0004721 GO:0005911 GO:0019838 GO:0032361 GO:0032587 GO:0031247 GO:0015629 GO:0032465 GO:0005829 GO:0031072 GO:0004647 GO:0030836 GO:0032154 GO:0000287 GO:0007088 GO:0030496 GO:0031258 GO:0070062 GO:0006470 GO:0071318 GO:0070938 GO:0033883 |
| tr|A0A024R1J2|A0A024R1J2_ HUMAN | GO:0001889 GO:0002673 GO:0050708 GO:0045732 GO:0009410 GO:0005515 GO:0032815 GO:0000165 |
| tr|A0A024R1K7|A0A024R1K7_ HUMAN | GO:0006713 GO:0000075 GO:0005080 GO:0005925 GO:2000649 GO:1900740 GO:0086010 GO:0007010 GO:0048167 GO:0007088 GO:0050774 GO:0030971 GO:0005829 GO:0097193 GO:0021762 GO:0030659 GO:0035259 GO:0005159 GO:0008426 GO:0044325 GO:0032869 GO:0005886 GO:0006605 GO:0043209 GO:0044822 GO:0070062 GO:0042921 GO:0019904 GO:0007264 GO:0005739 GO:0006367 GO:0055114 GO:0045893 GO:0046982 GO:0000086 GO:0043066 GO:0004497 GO:0014704 GO:0071901 GO:0017080 GO:0003779 |
| tr|A0A024R1K8|A0A024R1K8_ HUMAN | GO:0005686 GO:0044822 GO:0005515 GO:0071004 GO:0071013 GO:0005654 GO:0000389 |
| tr|A0A024R1N1|A0A024R1N1_ HUMAN | GO:0030220 GO:0005925 GO:0042803 GO:0032796 GO:0000212 GO:0005826 GO:0000146 GO:0030048 GO:0001701 GO:0007411 GO:0001772 GO:0005903 GO:0051295 GO:0031532 GO:0070527 GO:0031594 GO:0030898 GO:0005829 GO:0015031 GO:0006911 GO:0051693 GO:0043495 GO:1903919 GO:0016460 GO:0006509 GO:0001768 GO:0043534 GO:0048013 GO:0007520 GO:0001931 GO:0050900 GO:0008180 GO:0044822 GO:0070062 GO:0032154 GO:0090662 GO:0007264 GO:0019904 GO:0000910 GO:0005913 GO:0005524 GO:0001725 GO:0005819 GO:1903923 GO:0031032 GO:0030224 GO:0005516 GO:0001525 GO:0007229 GO:0051015 GO:0010248 GO:0005391 GO:0008360 GO:0043531 GO:0097513 GO:0008305 GO:0001726 |
| tr|A0A024R1N4|A0A024R1N4_ HUMAN | GO:0044877 GO:0051575 GO:0048660 GO:0043564 GO:0008022 GO:0004003 GO:0032481 GO:0007420 GO:0033151 GO:0050769 GO:0006266 GO:0045892 GO:0097680 GO:0005829 GO:0005654 GO:0003691 GO:0032508 GO:0000783 GO:0044212 GO:0071480 GO:0045944 GO:0045087 GO:0070419 GO:0000723 GO:0044822 GO:0051290 GO:0005667 GO:0071481 GO:0005524 GO:0075713 GO:0003684 GO:0005730 GO:0016020 GO:0071475 |
| tr|A0A024R1S8|A0A024R1S8_ HUMAN | GO:0009967 GO:0008270 GO:0005070 GO:0030864 GO:0051015 GO:0070062 GO:0005925 GO:0034220 GO:0015075 |
| tr|A0A024R1T1|A0A024R1T1_ HUMAN | GO:0005730 GO:0005840 GO:0005654 GO:0019899 GO:0044822 |
| tr|A0A024R1T5|A0A024R1T5_ HUMAN | GO:0042803 GO:0009636 GO:0003419 GO:0007168 GO:0045669 GO:0007268 GO:0035749 GO:0040014 GO:0030900 GO:0005654 GO:0009791 GO:0005615 GO:0003418 GO:0021762 GO:0048709 GO:0009214 GO:0051053 GO:0004113 GO:0030814 GO:0051428 GO:0007409 GO:0030551 GO:0007568 GO:0005886 GO:0005184 GO:0000226 GO:0070062 GO:0048471 GO:0008344 GO:0030141 GO:0042470 GO:0003723 GO:0008285 GO:1900194 GO:0030828 GO:0046902 GO:0005874 GO:0005741 GO:0005902 GO:0045909 GO:0051447 GO:0035748 GO:0005743 GO:0031143 GO:0032496 |
| tr|A0A024R1T9|A0A024R1T9_ HUMAN | GO:0048037 GO:0031325 GO:0005654 GO:0006101 GO:0006107 GO:0005515 GO:0003878 GO:0006112 GO:0005829 GO:0006633 GO:0019432 GO:0046872 GO:0006695 GO:0035338 GO:0016829 GO:0070062 GO:0005886 GO:0006085 GO:0005524 GO:0005739 |
| tr|A0A024R1U0|A0A024R1U0_ HUMAN | GO:0000278 GO:0072686 GO:1904117 GO:1990723 GO:0000236 GO:0031965 GO:0044614 GO:0000777 GO:0008536 GO:0005829 GO:1904115 GO:0030425 GO:0007264 GO:0031625 GO:0005096 GO:0048678 GO:0000090 GO:0016925 GO:0043547 GO:0043687 GO:0046826 GO:0000922 |
| tr|A0A024R1U2|A0A024R1U2_ HUMAN | GO:0045893 GO:0016363 GO:0071011 GO:0005730 GO:0005686 GO:0016607 GO:0019233 GO:0003677 GO:0044822 GO:0045292 GO:0010906 GO:0005515 GO:0071013 GO:0005689 GO:0003700 |
| tr|A0A024R1U4|A0A024R1U4_ HUMAN | GO:0030100 GO:0001525 GO:0005515 GO:0007032 GO:0042470 GO:0005525 GO:0097443 GO:0001702 GO:0015031 GO:0005765 GO:0030139 GO:0007264 GO:0016337 GO:0070062 GO:0019003 GO:0005811 GO:0003924 GO:0005886 GO:0031901 GO:0048227 |
| tr|A0A024R1V4|A0A024R1V4_ HUMAN | GO:0005925 GO:0000184 GO:0006415 GO:0006413 GO:0016020 GO:0044822 GO:0016259 GO:0006414 GO:0016787 GO:0022625 GO:0030218 GO:0003735 GO:0019083 GO:0006614 GO:0070062 GO:1904044 GO:0005634 |
| tr|A0A024R1X3|A0A024R1X3_ HUMAN | GO:0005654 GO:0005198 GO:0000814 GO:1903900 GO:0036258 GO:0047485 GO:0005730 GO:0005829 GO:0061024 GO:0006355 GO:0006468 GO:0004674 GO:0006914 GO:0070062 GO:0042803 GO:0005524 GO:0043328 GO:0005739 |
| tr|A0A024R1X4|A0A024R1X4_ HUMAN | GO:0031305 GO:0033617 GO:0070131 GO:0005515 |
| tr|A0A024R1Z6|A0A024R1Z6_ HUMAN | GO:0043826 GO:0010637 GO:0008270 GO:0070083 GO:0043883 GO:0052693 GO:0033603 GO:0005789 GO:0043738 GO:0006855 GO:0008748 GO:0015222 GO:0005741 GO:0043914 GO:0051612 GO:0071285 GO:0098793 GO:0016021 GO:0015238 GO:0055114 GO:0070062 GO:0019899 |
| tr|A0A024R231|A0A024R231_ HUMAN | GO:0015297 GO:0005515 GO:0008270 GO:0015939 GO:0006412 GO:0008892 GO:0005743 GO:0009108 GO:0006195 GO:0005829 GO:0016021 GO:0003735 GO:0055085 GO:0006147 GO:0007399 GO:0070062 GO:0031116 |
| tr|A0A024R233|A0A024R233_ HUMAN | GO:0005829 GO:0030674 GO:0005912 GO:0006921 GO:0008022 GO:0046037 GO:0090559 GO:0046710 GO:0090557 GO:0004385 GO:0005923 GO:0019904 GO:0005654 GO:0035329 GO:0050892 GO:0010033 GO:0005886 |
| tr|A0A024R261|A0A024R261_ HUMAN | GO:0000184 GO:0006413 GO:0006415 GO:0034198 GO:0005515 GO:1900087 GO:0044822 GO:0016259 GO:0006414 GO:1990928 GO:0022625 GO:0031672 GO:0003735 GO:0019083 GO:0006614 GO:0005634 |
| tr|A0A024R277|A0A024R277_ HUMAN | GO:0005515 GO:1904504 GO:0046513 GO:0046512 GO:0006686 GO:0035339 GO:0004758 GO:0030170 GO:0016021 GO:0046511 |
| tr|A0A024R281|A0A024R281_ HUMAN | GO:1903861 GO:0016874 GO:0004842 GO:0030512 GO:0086005 GO:0043161 GO:0019058 GO:2001288 GO:0005829 GO:0005654 GO:0019871 GO:0019870 GO:0044325 GO:0005886 GO:0007588 GO:0070062 GO:0006367 GO:0006883 GO:2000650 GO:0000122 GO:0003254 GO:0010038 GO:0060306 GO:1901017 GO:2000009 GO:0042787 GO:0030104 GO:0006513 GO:0070936 GO:0045732 |
| tr|A0A024R2F4|A0A024R2F4_ HUMAN | GO:0005730 GO:0003723 GO:0005737 GO:0032259 GO:0008168 |
| tr|A0A024R2F9|A0A024R2F9_ HUMAN | GO:0071763 GO:0005788 GO:0005794 GO:0043621 GO:0005639 |
| tr|A0A024R2M6|A0A024R2M6_ HUMAN | GO:0000038 GO:0036109 GO:0016401 GO:0005739 GO:0003988 GO:0033540 GO:0007584 GO:0008206 GO:0005515 GO:0048545 GO:0042493 GO:0005782 GO:0016020 |
| tr|A0A024R2M7|A0A024R2M7_ HUMAN | GO:0031098 GO:0004702 GO:0005515 GO:1901017 GO:0018107 GO:2000687 GO:0042981 GO:0032147 GO:0005737 GO:0023014 GO:0000287 GO:0071476 GO:0070062 GO:0007346 GO:0005524 GO:0006979 |
| tr|A0A024R2Q4|A0A024R2Q4_ HUMAN | GO:0000184 GO:0006415 GO:0006413 GO:0005515 GO:0016020 GO:0045471 GO:0002181 GO:0044822 GO:0016259 GO:0006414 GO:0022625 GO:0031672 GO:0003735 GO:0019083 GO:0006614 GO:0070062 GO:0005634 |
| tr|A0A024R2Q9|A0A024R2Q9_ HUMAN | GO:0006637 GO:0051790 GO:0004315 GO:0051792 GO:0005739 |
| tr|A0A024R2Z6|A0A024R2Z6_ HUMAN | GO:0005654 GO:0042127 GO:0032206 GO:0005515 GO:0016020 GO:0044822 GO:0005615 GO:0005525 GO:1902895 GO:0005730 GO:1904816 GO:0033235 GO:0003924 GO:0042254 |
| tr|A0A024R324|A0A024R324_ HUMAN | GO:0030424 GO:0019003 GO:0043525 GO:0038027 GO:0030521 GO:0071393 GO:0051022 GO:0043280 GO:0005525 GO:2000177 GO:0042346 GO:0021861 GO:0006357 GO:0051924 GO:0033144 GO:0090307 GO:0030496 GO:0017022 GO:0005789 GO:0030335 GO:0036089 GO:0009612 GO:0046039 GO:0050773 GO:0045907 GO:0005925 GO:0051496 GO:0007266 GO:0021795 GO:0007229 GO:0005938 GO:0043200 GO:0060071 GO:0032587 GO:0030838 GO:0048010 GO:0048011 GO:0043931 GO:0070062 GO:0043366 GO:1902766 GO:0090324 GO:0090051 GO:0043524 GO:0005739 GO:0030168 GO:0005768 GO:0005634 GO:0032467 GO:0045987 GO:1903427 GO:0045727 GO:0001666 GO:0005829 GO:0019904 GO:0030027 GO:0071803 GO:0007519 GO:0048015 GO:2000290 GO:0007411 GO:0048013 GO:0030307 GO:0060317 GO:0051384 GO:0043296 GO:0003924 GO:0021762 GO:0042476 GO:0009749 GO:0043124 GO:0045471 GO:0070507 GO:0007179 GO:0060193 GO:0050771 GO:0043297 GO:0031098 GO:0033688 GO:0016032 GO:0050919 GO:0050772 GO:0044319 GO:0097498 GO:0061383 GO:0007160 GO:0002363 GO:0046638 GO:0032154 GO:0042493 GO:0051056 GO:0005856 |
| tr|A0A024R325|A0A024R325_ HUMAN | GO:0034943 GO:0005815 GO:0045244 GO:0052686 GO:0034842 GO:0034841 GO:0010435 GO:0052685 GO:0018855 GO:0070251 GO:0006105 GO:0034793 GO:0018854 GO:0043762 GO:0046872 GO:0006104 GO:0034823 GO:0015645 GO:0034865 GO:0018857 GO:0043955 GO:0006099 GO:0019003 GO:0004777 GO:0034796 GO:0005524 GO:0018856 GO:0034942 GO:0046982 GO:0090409 GO:0003996 GO:0004776 GO:0052688 GO:0034783 GO:0005634 GO:0005525 GO:0005886 GO:0052687 GO:0043759 |
| tr|A0A024R328|A0A024R328_ HUMAN | GO:0007173 GO:0023021 GO:0034351 GO:0014070 GO:0019901 GO:0060326 GO:0016363 GO:0042149 GO:0042742 GO:0035307 GO:0004699 GO:0030837 GO:2001022 GO:0046777 GO:0032963 GO:2000753 GO:0032091 GO:0042119 GO:0070301 GO:0043406 GO:0043407 GO:0009612 GO:0070976 GO:0018107 GO:0002223 GO:0051490 GO:0046872 GO:0032079 GO:0032613 GO:0046627 GO:0015810 GO:0032615 GO:0038096 GO:0090398 GO:0043200 GO:0050728 GO:0090331 GO:0043488 GO:0043560 GO:0007049 GO:0048010 GO:0005783 GO:0005886 GO:0008047 GO:0048011 GO:0070062 GO:0005654 GO:0050821 GO:0005739 GO:0050732 GO:0071447 GO:0006921 GO:0001666 GO:0060333 GO:0007202 GO:0005829 GO:0046326 GO:0010469 GO:0018105 GO:0032930 GO:0009749 GO:0045471 GO:0005524 GO:2001235 GO:0016064 GO:2000755 GO:0009408 GO:0005911 GO:0048471 GO:0042100 GO:0008543 GO:0032147 GO:0042493 GO:1900163 GO:0016572 GO:0004715 GO:0008631 GO:2000304 GO:0042307 |
| tr|A0A024R329|A0A024R329_ HUMAN | GO:0004475 GO:0018279 GO:0005739 GO:0070062 GO:0043687 GO:0048747 GO:0005515 GO:0009298 GO:0006488 GO:0005525 |
| tr|A0A024R332|A0A024R332_ HUMAN | GO:0003723 GO:0016607 GO:0008380 GO:0046784 GO:0000445 GO:0005515 GO:0000784 GO:0006397 GO:0005737 |
| tr|A0A024R394|A0A024R394_ HUMAN | GO:2000299 GO:0043531 GO:0008270 GO:0005509 GO:0051879 GO:0005524 GO:1900034 GO:0010824 GO:0061077 |
| tr|A0A024R395|A0A024R395_ HUMAN | GO:0000790 GO:0031860 GO:0007095 GO:0008022 GO:0004003 GO:0032481 GO:0003690 GO:0007507 GO:0000019 GO:0090305 GO:0016605 GO:0035861 GO:0005829 GO:0031573 GO:0032508 GO:0007004 GO:0033674 GO:0007129 GO:0000784 GO:0030145 GO:0045087 GO:0048471 GO:0007062 GO:0032876 GO:0032206 GO:0006303 GO:0000014 GO:0046597 GO:0045003 GO:0008283 GO:0007131 GO:0008408 GO:0000794 GO:0030870 GO:0043066 GO:0031954 |
| tr|A0A024R3A8|A0A024R3A8_ HUMAN | GO:0008380 GO:0005515 GO:0006397 GO:0005634 GO:0000166 GO:0044822 |
| tr|A0A024R3D4|A0A024R3D4_ HUMAN | GO:0008270 |
| tr|A0A024R3P9|A0A024R3P9_ HUMAN | GO:0000139 GO:0034237 |
| tr|A0A024R3R0|A0A024R3R0_ HUMAN | GO:0070126 GO:0005762 GO:0070125 GO:0005743 GO:0003735 GO:0070124 |
| tr|A0A024R3R5|A0A024R3R5_ HUMAN | GO:0005521 GO:0055114 GO:0006998 GO:0007067 GO:0030176 GO:0005652 GO:0016628 GO:0051087 GO:0005639 GO:0005643 GO:0003677 GO:0008139 GO:0044822 GO:0070087 GO:0006695 |
| tr|A0A024R3S3|A0A024R3S3_ HUMAN | GO:0016021 GO:0043531 GO:0005739 GO:0006744 GO:0005524 GO:0005515 GO:0016310 GO:0016301 |
| tr|A0A024R3U8|A0A024R3U8_ HUMAN | GO:0005739 |
| tr|A0A024R3V0|A0A024R3V0_ HUMAN | GO:0005730 GO:0044822 GO:0005737 GO:0043234 |
| tr|A0A024R3V7|A0A024R3V7_ HUMAN | GO:0045893 GO:0005739 GO:1903507 GO:0042802 GO:0008134 GO:0030182 GO:0005634 |
| tr|A0A024R3V8|A0A024R3V8_ HUMAN | GO:0031687 GO:0005829 GO:0008565 GO:0046872 GO:0032403 GO:0007283 GO:0031047 GO:0043565 GO:0044822 GO:0030154 GO:0048471 GO:0003697 GO:0007275 GO:0015031 GO:0005794 GO:0005634 |
| tr|A0A024R3W2|A0A024R3W2_ HUMAN | GO:0005742 GO:0030943 GO:0031307 GO:0044233 GO:0006357 GO:0044267 GO:0016031 GO:0070096 GO:0000423 GO:0030150 GO:0044212 GO:0051082 GO:0015450 GO:0005634 GO:0015266 |
| tr|A0A024R3W4|A0A024R3W4_ HUMAN | GO:0005829 GO:0008412 GO:0052623 GO:0043888 GO:0004661 GO:0033384 GO:0046428 GO:0046872 GO:0045337 GO:0008495 GO:0004161 GO:0043919 GO:0005515 GO:0052622 GO:0033386 GO:0048045 GO:0043918 GO:0006695 |
| tr|A0A024R3W7|A0A024R3W7_ HUMAN | GO:0003746 GO:0005853 GO:0005515 GO:0005634 GO:0005829 GO:0005783 GO:0006414 |
| tr|A0A024R3X4|A0A024R3X4_ HUMAN | GO:0009986 GO:0051087 GO:0046696 GO:0043524 GO:0051131 GO:0042026 GO:0032735 GO:0032729 GO:0005791 GO:0051082 GO:0043032 GO:0042542 GO:0031625 GO:0003697 GO:0071866 GO:0032733 GO:0005829 GO:0001530 GO:0032727 GO:0051085 GO:0005759 GO:0005615 GO:0045121 GO:0002039 GO:0042100 GO:0006986 GO:0002020 GO:0001666 GO:0043559 GO:0003725 GO:0006919 GO:0002755 GO:0005886 GO:0019907 GO:0042220 GO:0002842 GO:0043209 GO:0009408 GO:0044822 GO:0070062 GO:0042588 GO:0016032 GO:0032403 GO:0005769 GO:0005794 GO:0014823 GO:0051384 GO:0005524 GO:0051787 GO:1904469 GO:0016887 GO:0008637 GO:0002368 GO:0046982 GO:0050821 GO:0050729 GO:0050870 GO:0043627 GO:0002931 GO:0030135 GO:0009409 GO:0042493 GO:0030061 GO:1903427 GO:2000778 GO:0005782 GO:0003688 GO:0005905 GO:0048291 GO:0002236 GO:0032496 GO:0033198 |
| tr|A0A024R3Z5|A0A024R3Z5_ HUMAN | GO:0005737 GO:0004930 GO:0005887 GO:0050750 GO:0007186 GO:0008152 GO:0008270 GO:0003824 GO:0070062 GO:0017124 GO:0043295 |
| tr|A0A024R415|A0A024R415_ HUMAN | GO:0016021 GO:0042053 GO:0046929 GO:0005739 GO:0046872 GO:0019243 GO:0032225 GO:0005515 GO:0050884 GO:0004416 GO:0005634 |
| tr|A0A024R471|A0A024R471_ HUMAN | GO:0005737 GO:0008180 GO:0005654 GO:0005515 GO:0006283 GO:0070911 GO:0000715 GO:0010388 |
| tr|A0A024R473|A0A024R473_ HUMAN | GO:0005515 GO:0005840 GO:0031053 GO:0044822 GO:0090502 GO:0005743 GO:0031054 GO:0030422 GO:0070126 GO:0070124 GO:0016443 GO:0003725 GO:0005634 GO:0070125 |
| tr|A0A024R482|A0A024R482_ HUMAN | GO:0016779 GO:0005829 GO:0018279 GO:0070062 GO:0043687 GO:0005515 GO:0009298 GO:0006488 |
| tr|A0A024R491|A0A024R491_ HUMAN | GO:0070584 GO:0090200 GO:0090141 GO:0032592 GO:0005777 GO:1900063 GO:0005741 GO:0016559 GO:0008053 GO:0008021 GO:0030054 GO:0043653 GO:0090314 GO:0042803 GO:0051260 GO:0006626 |
| tr|A0A024R499|A0A024R499_ HUMAN | GO:0048011 GO:0004871 GO:0010468 GO:0005080 GO:0002053 GO:0090275 GO:0043548 GO:0060397 GO:0005901 GO:0007411 GO:0048010 GO:0046676 GO:0071478 GO:0000186 GO:0070094 GO:0005829 GO:0042169 GO:0045725 GO:0043552 GO:0042593 GO:0005159 GO:0036064 GO:0045087 GO:0007265 GO:0007568 GO:0005158 GO:0005068 GO:0031000 GO:0014823 GO:0007173 GO:2001275 GO:0048009 GO:0046628 GO:0030335 GO:0038095 GO:0014065 GO:0046627 GO:1990416 GO:0043491 GO:1904385 GO:0030879 GO:0051291 GO:0008543 GO:0005634 GO:0034504 GO:0005899 GO:0032000 |
| tr|A0A024R4A0|A0A024R4A0_ HUMAN | GO:0001525 GO:0097421 GO:0001650 GO:0005654 GO:0042393 GO:0043236 GO:0016020 GO:0032760 GO:0008022 GO:2000232 GO:0000166 GO:0043066 GO:0007283 GO:0042162 GO:1990631 GO:0005509 GO:0003697 GO:1901838 GO:0001651 GO:0009986 GO:2000778 GO:0042802 GO:0035368 GO:0036464 GO:0006897 GO:0045944 GO:0042134 GO:0070062 GO:0071222 GO:0005938 |
| tr|A0A024R4A5|A0A024R4A5_ HUMAN | GO:0044763 GO:0051716 GO:0017148 GO:0016020 GO:0007275 GO:0044822 GO:0007610 |
| tr|A0A024R4E2|A0A024R4E2_ HUMAN | GO:0070935 GO:0006366 GO:0071765 GO:0003730 GO:0043922 GO:0006355 GO:0001933 GO:0016604 GO:0008380 GO:0051726 GO:0042802 GO:0048522 GO:0003690 GO:0030264 GO:0001205 |
| tr|A0A024R4F1|A0A024R4F1_ HUMAN | GO:0009615 GO:0000015 GO:0043005 GO:0006094 GO:0000122 GO:0051099 GO:0003714 GO:0097060 GO:0051020 GO:0044822 GO:0005615 GO:0019897 GO:0031430 GO:0071229 GO:0046982 GO:0031072 GO:0004634 GO:0003677 GO:0030308 GO:0000287 GO:0070062 GO:0042803 GO:0061621 GO:0005634 GO:0001701 GO:0003700 |
| tr|A0A024R4F4|A0A024R4F4_ HUMAN | GO:0000278 GO:0005654 GO:0055089 GO:0042769 GO:0003887 GO:0016020 GO:0003684 GO:0000166 GO:0000784 GO:0051539 GO:0019985 GO:0000731 GO:0006283 GO:0045004 GO:0006297 GO:0070911 GO:0006287 GO:0016235 GO:0005737 GO:0046872 GO:0000722 GO:0033683 GO:0000109 GO:0034644 GO:0044281 GO:0000084 GO:0032201 GO:0003682 GO:0019899 GO:0006298 GO:0043625 GO:0008296 GO:0006271 |
| tr|A0A024R4G1|A0A024R4G1_ HUMAN | GO:0009328 GO:0006432 GO:0044822 GO:0005515 GO:0004826 |
| tr|A0A024R4J8|A0A024R4J8_ HUMAN | GO:0070997 GO:0005654 GO:0005515 GO:0004252 GO:0007417 GO:0016811 GO:0042982 GO:0042246 GO:0042445 GO:0031965 GO:0015630 GO:0005615 GO:0010975 GO:0045745 GO:0005730 GO:0016540 GO:0043234 GO:0030574 GO:0045171 GO:0070062 GO:0005783 GO:0042552 GO:0005739 |
| tr|A0A024R4M0|A0A024R4M0_ HUMAN | GO:0005925 GO:0000184 GO:0006415 GO:0006413 GO:0005515 GO:0016020 GO:0044822 GO:0016259 GO:0016787 GO:0045903 GO:0005730 GO:0045182 GO:0008284 GO:0003735 GO:0019083 GO:0006614 GO:0070062 GO:0019843 GO:0022627 |
| tr|A0A024R4M8|A0A024R4M8_ HUMAN | GO:0008875 GO:0018453 GO:0033709 GO:0004495 GO:0055114 GO:0018452 GO:0032867 GO:0048258 GO:0005743 GO:0034831 GO:0042462 GO:0035410 GO:0052677 GO:0004448 GO:0010842 GO:0033765 GO:0051990 GO:0044105 GO:0043713 GO:0032866 GO:0019152 GO:0044103 GO:0000252 GO:0032442 GO:0034840 GO:0009644 GO:0035380 GO:0018451 |
| tr|A0A024R4Q8|A0A024R4Q8_ HUMAN | GO:0005925 GO:0000184 GO:0006415 GO:0006413 GO:0005515 GO:0016020 GO:0000028 GO:0006450 GO:0016259 GO:0003735 GO:0019083 GO:0006614 GO:0070062 GO:0003729 GO:0019843 GO:0022627 |
| tr|A0A024R4R3|A0A024R4R3_ HUMAN | GO:0001829 GO:0017148 GO:0005515 GO:0005829 GO:0030015 GO:2000036 GO:0000932 GO:0000289 |
| tr|A0A024R4S0|A0A024R4S0_ HUMAN | GO:0030117 GO:0051291 GO:0010324 GO:0039702 GO:0060548 GO:0007034 GO:0007080 GO:1901673 GO:0000920 GO:0000815 GO:0036258 GO:0015031 GO:1904903 GO:0016197 GO:1902188 GO:0005829 GO:0031902 GO:0019904 GO:0051258 GO:0031210 GO:0006997 GO:0006914 GO:0006892 GO:1903723 GO:0070062 GO:0051260 GO:1903543 |
| tr|A0A024R4S1|A0A024R4S1_ HUMAN | GO:0042059 GO:0007565 GO:0044325 GO:0007219 GO:0043195 GO:0006897 GO:0008134 GO:0048568 GO:0001701 GO:0005634 GO:0005886 |
| tr|A0A024R4T4|A0A024R4T4_ HUMAN | GO:0070740 GO:0070738 GO:0002223 GO:0043773 GO:0007179 GO:0043774 GO:0019788 GO:0038061 GO:0008766 GO:0070736 GO:0005829 GO:0033209 GO:0045116 GO:0031625 GO:0016567 GO:0070737 GO:0070062 GO:0043525 GO:0018169 GO:0005524 GO:0061630 |
| tr|A0A024R4U0|A0A024R4U0_ HUMAN | GO:0005829 GO:0008565 GO:0009306 GO:0018279 GO:0000139 GO:0046872 GO:0061024 GO:0043687 GO:0005515 GO:0043547 GO:0006888 GO:0006886 GO:0005096 |
| tr|A0A024R4U3|A0A024R4U3_ HUMAN | GO:0016874 GO:0006464 GO:0005524 |
| tr|A0A024R4X0|A0A024R4X0_ HUMAN | GO:0051287 GO:0019852 GO:0055114 GO:0043531 GO:0005743 GO:0070062 GO:0005741 GO:0016208 GO:0004128 GO:0071949 GO:0005811 GO:0008015 GO:0005789 GO:0006695 GO:0005833 |
| tr|A0A024R4Z6|A0A024R4Z6_ HUMAN | GO:0006281 GO:0005730 GO:0050434 GO:0006260 GO:0006355 GO:0003677 GO:0006368 GO:0044822 GO:0005515 GO:0005694 GO:0005654 GO:0005737 GO:0003682 |
| tr|A0A024R534|A0A024R534_ HUMAN | GO:0043044 GO:0000980 GO:0000122 GO:0004407 GO:0042826 GO:0008270 GO:0016020 GO:0001103 GO:0010762 GO:0006323 GO:0031492 GO:0016575 GO:0006333 GO:0016581 GO:0000978 GO:0005667 GO:0006306 GO:0000989 GO:0000790 GO:0045944 GO:0003700 |
| tr|A0A024R542|A0A024R542_ HUMAN | GO:0005829 GO:0005856 GO:0000289 GO:0030014 GO:0030506 GO:0005724 GO:0019899 GO:0007004 GO:0010467 GO:0005654 GO:0005886 |
| tr|A0A024R546|A0A024R546_ HUMAN | GO:0005737 GO:0030425 GO:0019706 GO:0005886 GO:0005515 GO:0016021 GO:0018345 GO:0008270 |
| tr|A0A024R563|A0A024R563_ HUMAN | GO:0006468 GO:0008270 GO:0004674 GO:0042325 GO:0004864 GO:0007283 GO:0005524 GO:0043086 GO:0005801 GO:0046982 GO:0005737 GO:0008599 GO:0005634 |
| tr|A0A024R565|A0A024R565_ HUMAN | GO:0030488 GO:0043234 GO:0070476 GO:0008276 GO:0018364 GO:0005515 GO:0070062 |
| tr|A0A024R571|A0A024R571_ HUMAN | GO:0010886 GO:0055038 GO:0017137 GO:2001137 GO:0034383 GO:0005525 GO:0031095 GO:0005509 GO:0031175 GO:0043209 GO:1901741 GO:0006886 GO:0061512 GO:0030139 GO:0007596 GO:0006897 GO:0020018 GO:0042384 GO:0048471 GO:0070062 GO:0051260 GO:0005811 GO:0031901 GO:0005524 GO:1990090 GO:0042632 |
| tr|A0A024R576|A0A024R576_ HUMAN | GO:0016192 GO:0016021 GO:0008270 GO:0006355 GO:0003677 GO:0005515 GO:0005794 GO:0005634 |
| tr|A0A024R577|A0A024R577_ HUMAN | GO:0055114 GO:0043235 GO:0030176 GO:0050613 GO:0005887 GO:0006695 GO:0005637 |
| tr|A0A024R578|A0A024R578_ HUMAN | GO:0070126 GO:0005762 GO:0070125 GO:0005743 GO:0003735 GO:0005515 GO:0070124 |
| tr|A0A024R588|A0A024R588_ HUMAN | GO:0048662 GO:0005681 GO:0009888 GO:0007538 GO:0000980 GO:0001553 GO:0005543 GO:1903507 GO:2000195 GO:0010259 GO:0003707 GO:0005654 GO:0030522 GO:0003714 GO:0045131 GO:0003713 GO:0045944 GO:0042445 GO:0004879 GO:0044822 GO:0042802 GO:0008270 GO:0006367 GO:0033327 GO:0003682 GO:0007267 GO:0090575 GO:0030238 GO:0003705 GO:0005840 GO:0051457 GO:0050810 GO:0043401 GO:0019899 GO:2000020 GO:0030325 GO:0000389 |
| tr|A0A024R598|A0A024R598_ HUMAN | GO:0071796 GO:0036477 GO:0034098 GO:0005829 GO:1904293 GO:0051117 GO:0032435 GO:0031625 GO:0000502 GO:2000157 GO:0036435 GO:0031397 GO:1903094 |
| tr|A0A024R5C4|A0A024R5C4_ HUMAN | GO:0000139 GO:0005789 GO:0005615 GO:0005515 GO:0071786 GO:0016192 GO:0016032 GO:0016021 GO:0006915 GO:0070062 |
| tr|A0A024R5C5|A0A024R5C5_ HUMAN | GO:0006094 GO:0006107 GO:0005515 GO:0004075 GO:0005759 GO:0010629 GO:0006090 GO:0044791 GO:0004736 GO:0005743 GO:0005829 GO:0046872 GO:0003677 GO:0006768 GO:0019074 GO:0071073 GO:0009374 GO:0005524 |
| tr|A0A024R5H0|A0A024R5H0_ HUMAN | GO:0009615 GO:0005829 GO:0000088 GO:0070062 GO:0003677 GO:0075713 GO:0000090 GO:0000793 GO:0042803 GO:0005635 GO:0005654 GO:0015074 GO:0007077 GO:0007084 |
| tr|A0A024R5H8|A0A024R5H8_ HUMAN | GO:0005802 GO:0019882 GO:0000139 GO:0018125 GO:0034498 GO:0006913 GO:0005525 GO:0005829 GO:0001671 GO:0051117 GO:0019904 GO:0070381 GO:0032482 GO:0000042 GO:0072385 GO:0032781 GO:0006890 GO:0070062 GO:0016032 GO:0003924 GO:0031489 |
| tr|A0A024R5J4|A0A024R5J4_ HUMAN | GO:0035335 GO:0005856 GO:0050770 GO:0010591 GO:0008064 GO:0003677 GO:0004725 GO:0008138 GO:0003779 GO:0005737 GO:0005634 |
| tr|A0A024R5K1|A0A024R5K1_ HUMAN | GO:0005925 GO:2000394 GO:0030027 GO:0005884 GO:0031529 GO:0090135 GO:0051017 GO:0071672 GO:0036120 GO:0005829 GO:0042060 GO:2000393 GO:0042802 GO:0034316 GO:0071933 GO:1902463 GO:0051015 GO:0001725 GO:0048471 GO:0070062 GO:0035767 GO:0005886 |
| tr|A0A024R5K8|A0A024R5K8_ HUMAN | GO:0045121 GO:0005793 GO:0010951 GO:0005788 GO:0044822 GO:0051604 GO:0005581 GO:0005615 GO:0004867 GO:0032964 GO:0030199 GO:0051082 GO:0003433 GO:0070062 GO:0005518 GO:0006986 |
| tr|A0A024R5Q1|A0A024R5Q1_ HUMAN | GO:0015630 GO:0005654 GO:0005737 GO:0005515 |
| tr|A0A024R5Q7|A0A024R5Q7_ HUMAN | GO:0046040 GO:0071257 GO:0042301 GO:0044208 GO:0005525 GO:0006144 GO:0005829 GO:0006531 GO:0004019 GO:0000287 GO:0002376 GO:0051015 GO:0070062 GO:0042803 GO:0003924 GO:0005886 GO:0014074 GO:0060359 |
| tr|A0A024R5S4|A0A024R5S4_ HUMAN | GO:0005829 GO:0007265 GO:0006511 GO:0019897 GO:0030496 GO:0031313 GO:0070536 GO:0001669 GO:0004197 GO:0005769 GO:0004843 GO:0000281 GO:0071108 GO:0005654 GO:0008283 GO:0007032 GO:0005794 GO:0017124 |
| tr|A0A024R5S5|A0A024R5S5_ HUMAN | GO:0003743 GO:0001731 GO:0005829 GO:0016282 GO:0002181 GO:0005852 GO:0033290 GO:0005515 GO:0006446 |
| tr|A0A024R5U5|A0A024R5U5_ HUMAN | GO:0005925 GO:0007411 GO:0005178 GO:0051089 GO:0042117 GO:0030307 GO:0007219 GO:0005802 GO:0007267 GO:0000139 GO:0008270 GO:0022617 GO:0019901 GO:0006913 GO:0097197 GO:0048013 GO:0007162 GO:0007173 GO:0007229 GO:0042169 GO:0009986 GO:0010820 GO:0008284 GO:0016485 GO:0004222 GO:0007220 GO:0006468 GO:0034612 GO:0030574 GO:0016021 GO:0014069 GO:0097038 GO:0070062 GO:0005798 GO:0042803 GO:0017124 GO:0005634 GO:0051088 GO:0001701 |
| tr|A0A024R5X2|A0A024R5X2_ HUMAN | GO:0000098 GO:0005743 GO:0070221 GO:0070813 GO:0048038 GO:0034641 GO:0070224 |
| tr|A0A024R5X7|A0A024R5X7_ HUMAN | GO:0006457 GO:0005743 GO:0046872 GO:0042645 GO:0004176 GO:0051603 GO:0005524 GO:0051082 GO:0005654 GO:0016504 GO:0009841 GO:0010952 |
| tr|A0A024R5Y1|A0A024R5Y1_ HUMAN | GO:0005829 GO:0042609 GO:0050851 GO:0030140 GO:0010008 GO:0005654 |
| tr|A0A024R5Z3|A0A024R5Z3_ HUMAN | GO:0019901 GO:0005637 GO:1901203 GO:0019902 GO:0045599 GO:0005160 GO:0048340 GO:0030878 GO:0023019 GO:0016202 GO:0043235 GO:0030512 GO:0035413 GO:0001657 GO:0046982 GO:0030501 GO:0001701 GO:0030335 GO:0007050 GO:0042803 GO:0048701 GO:0008270 GO:0035556 GO:0043130 GO:0031625 GO:0060395 GO:0051894 GO:0071144 GO:0051496 GO:0008013 GO:0031490 GO:0043066 GO:0030618 GO:0032731 GO:0033689 GO:0042110 GO:0050728 GO:0048589 GO:0045944 GO:0031053 GO:0010694 GO:0050678 GO:0042993 GO:0005886 GO:0035326 GO:0009880 GO:0005654 GO:0050821 GO:0043425 GO:0070306 GO:0001756 GO:0005518 GO:0038092 GO:0045668 GO:0001889 GO:0030308 GO:0010718 GO:0051098 GO:0007492 GO:0045930 GO:0007183 GO:0001666 GO:0005829 GO:0042177 GO:0032909 GO:0006367 GO:0001947 GO:0060039 GO:0019049 GO:0000122 GO:0000790 GO:0000978 GO:0000983 GO:0090263 GO:0032332 GO:0060290 GO:0050776 GO:0070412 GO:0097296 GO:0070410 GO:0032916 GO:0050927 GO:0001933 GO:0048617 GO:0001102 GO:0000988 GO:0002520 GO:0002076 GO:0097191 GO:0001707 GO:0061045 |
| tr|A0A024R5Z7|A0A024R5Z7_ HUMAN | GO:0009986 GO:0031340 GO:0006900 GO:0048146 GO:1900121 GO:0030496 GO:0002091 GO:0031902 GO:0005829 GO:0035749 GO:0030199 GO:0016323 GO:0071229 GO:0072661 GO:0005615 GO:0005544 GO:0045121 GO:0005604 GO:0030054 GO:0031214 GO:0005262 GO:0007589 GO:0044354 GO:0002020 GO:0001934 GO:1990667 GO:0030546 GO:0051917 GO:0042383 GO:0017137 GO:0070509 GO:0006936 GO:0044822 GO:0070062 GO:0005811 GO:0048471 GO:0051290 GO:0005769 GO:0001786 GO:0001725 GO:0001948 GO:0042470 GO:0048306 GO:0005765 GO:0032804 GO:0019897 GO:0097066 GO:0036035 GO:0001525 GO:0043086 GO:0051015 GO:0001765 GO:0051099 GO:0005546 GO:0005509 GO:2000273 GO:0070588 GO:0005634 GO:0005938 GO:0043220 GO:0044548 GO:0001726 GO:0019834 |
| tr|A0A024R637|A0A024R637_ HUMAN | GO:0030659 GO:0032869 GO:0070062 GO:0031339 GO:0043547 GO:0005096 |
| tr|A0A024R652|A0A024R652_ HUMAN | GO:0004329 GO:0016020 GO:0061053 GO:0007507 GO:0004486 GO:0001780 GO:0035999 GO:0009086 GO:0005829 GO:0009257 GO:0048702 GO:0004477 GO:0048703 GO:0009070 GO:0000105 GO:0004488 GO:0001843 GO:0046655 GO:0006164 GO:0055114 GO:0070062 GO:0004487 GO:0005524 GO:0019346 GO:0005739 |
| tr|A0A024R663|A0A024R663_ HUMAN | GO:0005887 GO:0007018 GO:0019894 GO:0030176 GO:0007264 GO:0044822 GO:0015031 |
| tr|A0A024R683|A0A024R683_ HUMAN | GO:0033572 GO:0005929 GO:0015992 GO:0005515 GO:0008286 GO:0006879 GO:0005765 GO:0005829 GO:0061512 GO:0045454 GO:0005813 GO:0042384 GO:0033176 GO:0034220 GO:0042626 GO:0070062 |
| tr|A0A024R6C9|A0A024R6C9_ HUMAN | GO:0004149 GO:0051087 GO:0005759 GO:0045252 GO:0043209 GO:0006099 GO:0031072 GO:0046487 GO:0006103 GO:0033512 GO:0070062 GO:0005886 GO:0005634 GO:0006734 |
| tr|A0A024R6D4|A0A024R6D4_ HUMAN | GO:0034709 GO:0045747 GO:0007049 GO:0030496 GO:0042742 GO:0006213 GO:0006221 GO:0044822 GO:0005515 GO:0008327 GO:0001649 GO:0016020 |
| tr|A0A024R6D8|A0A024R6D8_ HUMAN | GO:0097421 GO:0050733 GO:0009611 GO:0032869 GO:0000166 GO:0006406 GO:0016607 GO:0044822 GO:0006376 GO:0005737 GO:0005730 GO:0031124 GO:0006355 GO:0051726 GO:0006369 |
| tr|A0A024R6H1|A0A024R6H1_ HUMAN | GO:0017059 GO:1904504 GO:0046513 GO:0046512 GO:0006686 GO:0004758 GO:0030170 GO:0016021 GO:0046511 GO:0005739 |
| tr|A0A024R6H3|A0A024R6H3_ HUMAN | GO:0005829 GO:0052825 GO:0052726 GO:0052830 GO:0016311 GO:0021915 GO:0005524 GO:0000287 GO:0016310 GO:0007165 GO:0016853 GO:0052831 GO:0016324 GO:0052835 GO:0047325 GO:0052725 GO:0007596 GO:0032957 |
| tr|A0A024R6K1|A0A024R6K1_ HUMAN | GO:0007179 GO:0007067 GO:0001701 GO:0045737 GO:0008353 GO:0044828 GO:0006974 GO:0005654 GO:0005922 GO:0071157 GO:0055077 GO:0002944 GO:0045944 GO:2001165 GO:0034765 GO:0006367 GO:0004693 GO:0007267 GO:0019901 GO:0006368 GO:0016538 GO:0002945 GO:0005244 |
| tr|A0A024R6K3|A0A024R6K3_ HUMAN | GO:0046975 GO:0005654 GO:0018023 GO:0018026 GO:0003713 GO:0018027 GO:0051568 GO:0001102 GO:0042800 GO:0000790 GO:0045944 GO:0010452 GO:0051149 |
| tr|A0A024R6K8|A0A024R6K8_ HUMAN | GO:0005829 GO:0004830 GO:0070062 GO:0008285 GO:0006436 GO:0005524 GO:0005515 GO:0045765 GO:0005634 |
| tr|A0A024R6Q2|A0A024R6Q2_ HUMAN | GO:0030488 GO:0016429 GO:0005654 GO:0031515 |
| tr|A0A024R6S1|A0A024R6S1_ HUMAN | GO:0005829 GO:0018885 GO:0031072 GO:0042026 GO:0051087 GO:0046872 GO:0001948 GO:0070062 GO:0009408 GO:0005524 GO:0051082 GO:0008284 GO:0016020 GO:0005634 |
| tr|A0A024R6T8|A0A024R6T8_ HUMAN | GO:0005634 GO:0005515 |
| tr|A0A024R6X2|A0A024R6X2_ HUMAN | GO:0030098 GO:0060216 GO:0045944 GO:0000209 GO:0003677 GO:0030099 GO:0005515 GO:0001649 GO:0048469 GO:0003713 GO:0003700 GO:0016020 GO:0005634 |
| tr|A0A024R6Y2|A0A024R6Y2_ HUMAN | GO:0005829 GO:0008565 GO:0008536 GO:0044613 GO:0005640 GO:0042307 GO:0000060 GO:0006611 GO:0070062 GO:1904046 |
| tr|A0A024R6Y3|A0A024R6Y3_ HUMAN | GO:0007267 GO:0010862 GO:0008083 GO:0018206 GO:0042981 GO:0007179 GO:0006412 GO:0005125 GO:0031365 GO:0005160 GO:0043408 GO:0005615 GO:0048468 GO:1901741 GO:0008284 GO:0060395 GO:0005506 GO:0042586 GO:0070062 GO:0005739 |
| tr|A0A024R6Z0|A0A024R6Z0_ HUMAN | GO:0003777 GO:0005829 GO:0030424 GO:0018279 GO:0005874 GO:0061024 GO:0005813 GO:0000226 GO:0043687 GO:0005868 GO:0005524 GO:0051642 GO:0006888 GO:0019886 GO:0016020 GO:0008090 |
| tr|A0A024R6Z7|A0A024R6Z7_ HUMAN | GO:0018503 GO:1904030 GO:0004860 GO:0005829 GO:0034912 GO:0055114 GO:0052849 GO:0005739 GO:0018499 GO:0034838 GO:0043786 GO:0017150 GO:0002943 GO:0018502 GO:0034824 GO:0018500 GO:0003725 GO:0018498 GO:0034809 GO:0005515 GO:0050660 GO:0052850 GO:0034790 GO:0034805 GO:0060548 GO:0018501 GO:0005783 GO:0016631 |
| tr|A0A024R702|A0A024R702_ HUMAN | GO:0005737 GO:0046785 GO:0015631 GO:0001578 GO:0070062 GO:0005874 |
| tr|A0A024R704|A0A024R704_ HUMAN | GO:0001525 GO:0005654 GO:0010628 GO:0048024 GO:0044822 GO:0035063 GO:0005737 GO:0005730 GO:0008284 GO:0006468 GO:0004674 GO:0000245 GO:0000287 GO:0045071 GO:0045070 GO:0030154 GO:0071889 GO:0043525 GO:0045087 GO:0005524 GO:0045787 GO:0035556 |
| tr|A0A024R705|A0A024R705_ HUMAN | GO:0005774 GO:0008022 GO:0008568 GO:0007080 GO:0090611 GO:0090543 GO:0000920 GO:0000815 GO:0036258 GO:1903076 GO:0007033 GO:0005764 GO:0015031 GO:1904903 GO:0016197 GO:0061738 GO:1902188 GO:0005829 GO:0031902 GO:0019904 GO:0072319 GO:0005813 GO:1903774 GO:0005769 GO:0017048 GO:0006997 GO:0009838 GO:0032367 GO:0048471 GO:0032466 GO:0070062 GO:1903543 GO:0034058 GO:0005886 GO:0005634 GO:0005524 GO:0043162 GO:0000922 |
| tr|A0A024R713|A0A024R713_ HUMAN | GO:0043159 GO:0048240 GO:0050660 GO:0005654 GO:0004148 GO:0005929 GO:0007369 GO:0051287 GO:0010510 GO:0005759 GO:0006120 GO:0007568 GO:0034604 GO:0009083 GO:0045252 GO:0051068 GO:0009106 GO:0043209 GO:0006099 GO:0045454 GO:0006554 GO:0045254 GO:0046487 GO:0006103 GO:0006508 GO:0043544 GO:0042391 GO:0061732 |
| tr|A0A024R718|A0A024R718_ HUMAN | GO:0009435 GO:0005654 GO:0007267 GO:0008144 GO:0008286 GO:0032922 GO:0051770 GO:0005125 GO:0005615 GO:0005829 GO:0006769 GO:0007565 GO:0048661 GO:0014070 GO:0060612 GO:0004514 GO:0030054 GO:0045944 GO:0070062 GO:0042803 GO:0047280 |
| tr|A0A024R753|A0A024R753_ HUMAN | GO:0005730 GO:0008380 GO:0005681 GO:0006397 GO:0000166 GO:0044822 |
| tr|A0A024R7B7|A0A024R7B7_ HUMAN | GO:0051087 GO:0006457 GO:0060338 GO:0032587 GO:0008022 GO:1990565 GO:0051879 GO:0006605 GO:0050821 GO:0051301 GO:0060334 GO:0005829 GO:0000079 GO:0051082 GO:0043422 GO:0019887 GO:0010608 GO:0070062 GO:0098779 GO:0005524 GO:0031435 |
| tr|A0A024R7D5|A0A024R7D5_ HUMAN | GO:0030669 GO:0005041 GO:0070508 GO:1990666 GO:0001523 GO:0046718 GO:0005887 GO:0030299 GO:0042159 GO:0030229 GO:0010899 GO:0006898 GO:0032050 GO:0034383 GO:0016323 GO:0008203 GO:0005764 GO:0015914 GO:0005509 GO:0006775 GO:0030169 GO:0001618 GO:0005794 GO:0010867 GO:0005770 GO:0005905 GO:0001948 GO:0042802 GO:0009897 GO:0005771 GO:0005769 GO:0043235 GO:0002020 GO:0045177 GO:0007603 GO:0005901 GO:2000188 GO:0034362 GO:0010008 |
| tr|A0A024R7F9|A0A024R7F9_ HUMAN | GO:0000062 GO:0004361 GO:0005743 GO:0052890 GO:0033539 GO:0009055 GO:0006568 GO:0050660 GO:0006554 GO:0005759 GO:0055088 GO:0046949 |
| tr|A0A024R7G2|A0A024R7G2_ HUMAN | GO:0005525 GO:0016188 GO:0061670 GO:0008022 GO:0032781 GO:0048790 GO:0051602 GO:0051117 GO:0043234 GO:0005829 GO:0015031 GO:0032482 GO:0005881 GO:0009791 GO:0036465 GO:0007005 GO:0031630 GO:0007409 GO:0007274 GO:0005886 GO:0043195 GO:0003016 GO:0070062 GO:0042588 GO:0001669 GO:0005739 GO:0001671 GO:0050975 GO:0018125 GO:0030742 GO:0031489 GO:0045453 GO:0003924 GO:1903307 GO:0051020 GO:0048172 GO:1900271 GO:0005768 GO:0030324 GO:0008021 |
| tr|A0A024R7H5|A0A024R7H5_ HUMAN | GO:0045121 GO:0004871 GO:0030336 GO:0005887 GO:0007267 GO:0007275 GO:0010951 GO:0044822 GO:0006959 GO:0008283 GO:0051607 GO:0043123 GO:0005794 GO:0005770 GO:0009986 GO:0032956 GO:0030308 GO:0005771 GO:0045071 GO:0035455 GO:0008191 GO:0034341 GO:0060337 GO:1901253 GO:0042113 GO:0070062 GO:0002737 GO:0042803 GO:0031225 GO:0016324 GO:0035456 |
| tr|A0A024R7I3|A0A024R7I3_ HUMAN | GO:0005525 GO:0048169 GO:0032402 GO:0048210 GO:0030140 GO:0014069 GO:0042384 GO:0045046 GO:0019003 GO:0005102 GO:0046326 GO:0005829 GO:0032456 GO:0005654 GO:0005778 GO:0072659 GO:0030670 GO:0051223 GO:0031513 GO:0042593 GO:0005814 GO:0000139 GO:0007409 GO:0036064 GO:0032869 GO:0005886 GO:0017137 GO:0055038 GO:0070062 GO:0097546 GO:0019882 GO:0007264 GO:0005739 GO:0006904 GO:0035845 GO:0019901 GO:0031489 GO:0030425 GO:0003924 GO:0000086 GO:0005730 GO:0043025 GO:0009306 GO:0008021 |
| tr|A0A024R7L5|A0A024R7L5_ HUMAN | GO:0009048 GO:0006406 GO:0071044 GO:0042162 GO:0006281 GO:0006449 GO:0005829 GO:0004004 GO:0032201 GO:0061158 GO:0005654 GO:0000784 GO:0071222 GO:0044822 GO:0000294 GO:0008270 GO:0005515 GO:0061014 GO:0005524 GO:0003682 GO:0000932 GO:0044530 GO:0071347 GO:0000785 GO:0000184 GO:0035145 |
| tr|A0A024R7M6|A0A024R7M6_ HUMAN | GO:0000122 GO:0001842 GO:0008270 GO:0016607 GO:0012501 GO:0000977 GO:0001568 GO:0016581 GO:0030674 GO:0006306 GO:0021506 GO:0010172 GO:0001701 GO:0003700 |
| tr|A0A024R7M8|A0A024R7M8_ HUMAN | GO:0005856 GO:0044822 GO:0005737 GO:0005886 GO:0044763 |
| tr|A0A024R7P3|A0A024R7P3_ HUMAN | GO:0098655 GO:0046872 GO:0006874 GO:0019829 GO:0005887 GO:0005524 GO:0008152 GO:0005789 |
| tr|A0A024R7T3|A0A024R7T3_ HUMAN | GO:0019013 GO:0043484 GO:0017025 GO:0003727 GO:0000398 GO:0044822 GO:0000166 GO:0071013 GO:0005654 GO:0005737 GO:0016020 |
| tr|A0A024R7V6|A0A024R7V6_ HUMAN | GO:0000278 GO:0000088 GO:0005765 GO:0000139 GO:0070062 GO:0006913 GO:0019003 GO:0033116 GO:0042470 GO:0032482 GO:0005515 GO:0007030 GO:0006888 GO:0043025 GO:0003924 GO:0006886 GO:0005789 GO:0005525 GO:0005634 |
| tr|A0A024R7W9|A0A024R7W9_ HUMAN | GO:0005852 GO:0006413 GO:0005654 GO:0005515 GO:0016579 GO:0008180 GO:0006283 GO:0070911 GO:0003743 GO:0010388 GO:0005667 GO:0046872 GO:1990182 GO:0003713 GO:0051726 GO:0004843 GO:0008635 GO:0008237 GO:0008021 GO:0000715 GO:0030054 GO:0045944 GO:0048471 GO:0046328 GO:1903894 GO:0005739 |
| tr|A0A024R7Z5|A0A024R7Z5_ HUMAN | GO:0005856 GO:0006930 GO:0005109 GO:0005925 GO:0042803 GO:0007346 GO:0008284 GO:0047485 GO:0008022 GO:0007411 GO:0007268 GO:0002091 GO:0072562 GO:0010862 GO:0005829 GO:0030511 GO:0045121 GO:1903553 GO:0045545 GO:0005137 GO:0006612 GO:0010718 GO:0048013 GO:0050839 GO:0032435 GO:1903543 GO:0007265 GO:0070062 GO:0030307 GO:0030335 GO:0008093 GO:0001948 GO:0042470 GO:0005895 GO:0019838 GO:0046982 GO:0046330 GO:0030036 GO:0005634 GO:0046875 GO:0005789 |
| tr|A0A024R814|A0A024R814_ HUMAN | GO:0005925 GO:0000184 GO:0006415 GO:0006413 GO:0016020 GO:0005844 GO:0002181 GO:0000463 GO:0016259 GO:0006414 GO:0022625 GO:0005730 GO:0003677 GO:0003735 GO:0019083 GO:0006614 GO:0070062 GO:0042803 GO:0003729 |
| tr|A0A024R837|A0A024R837_ HUMAN | GO:0008152 GO:0016787 GO:0005739 GO:0005515 |
| tr|A0A024R843|A0A024R843_ HUMAN | GO:1902187 GO:0045862 GO:0043130 GO:0051155 GO:0031369 GO:0070736 GO:0001894 GO:0032481 GO:0061564 GO:0017022 GO:0070738 GO:1903265 GO:1902230 GO:0005829 GO:0070740 GO:0007014 GO:0043123 GO:0043774 GO:0009411 GO:0003713 GO:0045444 GO:0045087 GO:0005863 GO:0043773 GO:0046716 GO:1902173 GO:1903883 GO:0030307 GO:0043621 GO:0008270 GO:0051092 GO:0018169 GO:1903886 GO:0045666 GO:0030335 GO:0003723 GO:0030957 GO:0045787 GO:0042787 GO:0061630 GO:0005634 GO:0048147 GO:0000209 GO:0008766 GO:0070737 GO:0032897 GO:0045732 |
| tr|A0A024R845|A0A024R845_ HUMAN | GO:0045176 GO:0030140 GO:0055037 GO:0005802 GO:0030659 GO:0000139 GO:0097208 GO:0045335 GO:0032456 GO:0007589 GO:0042175 GO:0005525 GO:0015031 GO:0042742 GO:0005765 GO:0072372 GO:0005829 GO:0005770 GO:0061024 GO:0001948 GO:0007264 GO:0032880 GO:0009790 GO:0005795 GO:0048471 GO:0070062 GO:0019003 GO:0008543 GO:0006895 GO:0003924 GO:0031489 GO:0090387 GO:0005791 GO:0031901 GO:0008152 GO:0016324 |
| tr|A0A024R861|A0A024R861_ HUMAN | GO:0005829 GO:0006470 GO:0001878 GO:0018279 GO:0000139 GO:0046872 GO:0043687 GO:0048208 GO:0004722 GO:0005515 GO:0000082 |
| tr|A0A024R872|A0A024R872_ HUMAN | GO:0005730 GO:0005912 GO:0005654 GO:0005886 GO:0043066 GO:0005829 GO:0015629 GO:0070062 |
| tr|A0A024R883|A0A024R883_ HUMAN | GO:0033572 GO:0008286 GO:0006879 GO:0008553 GO:0005765 GO:0005829 GO:0045454 GO:0051117 GO:0016471 GO:0008021 GO:1902600 GO:0070062 GO:0005886 GO:0008152 |
| tr|A0A024R897|A0A024R897_ HUMAN | GO:0005885 GO:0070062 GO:0034314 GO:0042995 GO:0005925 GO:0003779 GO:0005737 |
| tr|A0A024R8A7|A0A024R8A7_ HUMAN | GO:0001077 GO:0045944 GO:0044822 GO:0005515 GO:0003697 GO:0005737 GO:0016020 GO:0000978 GO:0005634 |
| tr|A0A024R8D2|A0A024R8D2_ HUMAN | GO:0034943 GO:0052685 GO:0043955 GO:0004774 GO:0000166 GO:0052688 GO:0043762 GO:0031526 GO:0034823 GO:0001676 GO:0034783 GO:0034942 GO:0005902 GO:0001579 GO:0005789 GO:0034841 GO:0052687 GO:0070251 GO:0044539 GO:0004467 GO:0018855 GO:0043759 GO:0034842 GO:0090409 GO:0016021 GO:0043588 GO:0034865 GO:0034796 GO:0018854 GO:0042760 GO:0010435 GO:0055085 GO:0052686 GO:0003996 GO:0018857 GO:0015245 GO:0034793 GO:0018856 GO:0007584 GO:0031957 |
| tr|A0A024R8H5|A0A024R8H5_ HUMAN | GO:0004871 GO:0090073 GO:0034351 GO:0043623 GO:0016874 GO:0004842 GO:0032743 GO:0007250 GO:0046625 GO:0002726 GO:0034138 GO:0043507 GO:0034142 GO:0031625 GO:0010939 GO:1903265 GO:0005829 GO:0045121 GO:1902041 GO:1901215 GO:0051023 GO:0070534 GO:1990597 GO:1903721 GO:0005174 GO:0032403 GO:0071732 GO:0042802 GO:0097057 GO:0008270 GO:0051092 GO:0043154 GO:0031996 GO:0071550 GO:0035666 GO:0000151 GO:2001238 GO:0019903 GO:0050870 GO:0070059 GO:0070266 GO:0051865 GO:0097296 GO:0009898 GO:0051291 GO:0035631 GO:0030163 GO:0005164 GO:1990604 GO:0005938 GO:0012506 GO:0031435 GO:0070207 |
| tr|A0A024R8L0|A0A024R8L0_ HUMAN | GO:0070126 GO:0003729 GO:0070125 GO:0005743 GO:0019843 GO:0003735 GO:0005763 GO:0070124 GO:0000028 |
| tr|A0A024R8L7|A0A024R8L7_ HUMAN | GO:0000038 GO:0047485 GO:0003995 GO:0000062 GO:0016559 GO:0006693 GO:0036109 GO:0016401 GO:0005739 GO:0005730 GO:0052890 GO:0033540 GO:0006091 GO:0033539 GO:0009055 GO:2000189 GO:0007283 GO:0005778 GO:0005102 GO:0030165 GO:0071949 GO:0005654 GO:0005782 GO:0005886 |
| tr|A0A024R8L8|A0A024R8L8_ HUMAN | GO:0018149 GO:0030674 GO:0031424 GO:0070062 GO:0045111 GO:0019215 GO:0005198 GO:0005737 GO:0030057 GO:0001533 |
| tr|A0A024R8P8|A0A024R8P8_ HUMAN | GO:0005925 GO:0001501 GO:0000184 GO:0006413 GO:0006415 GO:0007605 GO:0016259 GO:0006414 GO:0001503 GO:0048318 GO:0022625 GO:0043009 GO:0034463 GO:0042474 GO:0006417 GO:0003735 GO:0003723 GO:0033291 GO:0019083 GO:0006614 |
| tr|A0A024R8R4|A0A024R8R4_ HUMAN | GO:0005654 GO:0008270 GO:0034098 GO:0070530 GO:0005829 GO:0036501 GO:0007030 GO:0051117 GO:0061025 GO:0031625 GO:0036435 GO:0030970 GO:0032403 GO:0070987 GO:0030433 |
| tr|A0A024R8S3|A0A024R8S3_ HUMAN | GO:0046965 GO:0019789 GO:0034613 GO:0016605 GO:0044822 GO:0000724 GO:0070911 GO:0032436 GO:0045892 GO:0031386 GO:0031625 GO:0033235 GO:0045944 GO:0043687 |
| tr|A0A024R8S5|A0A024R8S5_ HUMAN | GO:0005925 GO:0005178 GO:0004175 GO:0006457 GO:0005793 GO:0005788 GO:0030198 GO:0044822 GO:0003756 GO:0042470 GO:1902175 GO:0000302 GO:0042157 GO:0046982 GO:0045454 GO:0071456 GO:0034976 GO:0009897 GO:0034663 GO:0046598 GO:0055114 GO:0006508 GO:0070062 GO:0016222 GO:0004656 GO:0019899 GO:0018401 |
| tr|A0A024R8T9|A0A024R8T9_ HUMAN | GO:0031594 GO:0030672 GO:0005515 GO:0006605 GO:0016021 GO:0070062 |
| tr|A0A024R8U5|A0A024R8U5_ HUMAN | GO:0005080 GO:0003714 GO:0007067 GO:0035061 GO:0000166 GO:0006406 GO:0016607 GO:0000381 GO:0016605 GO:0044822 GO:0036002 GO:0005737 GO:0031124 GO:0005681 GO:0006369 GO:0070062 GO:1903507 |
| tr|A0A024R8U8|A0A024R8U8_ HUMAN | GO:0016021 GO:0032580 GO:0045134 GO:0005509 GO:0004871 GO:0043123 GO:0070062 GO:0030166 GO:0005789 GO:0004382 GO:0009191 |
| tr|A0A024R8V0|A0A024R8V0_ HUMAN | GO:1902857 GO:0051291 GO:0007049 GO:0005874 GO:0051301 GO:0031105 GO:0001725 GO:0005515 GO:0003924 GO:0048471 GO:0031513 GO:0005525 GO:0005930 |
| tr|A0A024R8V6|A0A024R8V6_ HUMAN | GO:0016579 GO:1903146 GO:0044822 GO:0005730 GO:0006511 GO:1903955 GO:0004843 |
| tr|A0A024R8W0|A0A024R8W0_ HUMAN | GO:1904574 GO:0000289 GO:0045727 GO:0000381 GO:0090394 GO:0005829 GO:0004004 GO:0014070 GO:0016259 GO:0048701 GO:0008306 GO:0006364 GO:0035640 GO:0008143 GO:0019221 GO:0035613 GO:0045944 GO:0051028 GO:0035368 GO:0005515 GO:0071013 GO:0005524 GO:1904570 GO:1990416 GO:0043021 GO:0010501 GO:0048026 GO:0030425 GO:0000184 GO:0043025 GO:0072715 GO:0035145 GO:0016607 GO:0016020 |
| tr|A0A024R904|A0A024R904_ HUMAN | GO:0071277 GO:0030877 GO:0043005 GO:0005654 GO:0060548 GO:0055007 GO:0007568 GO:0044297 GO:0005641 GO:0070062 GO:0042803 GO:0045740 GO:0060416 |
| tr|A0A024R912|A0A024R912_ HUMAN | GO:0006238 GO:0005515 GO:0043231 GO:0016579 GO:0004849 GO:0016310 GO:0044206 GO:0005829 GO:0048678 GO:0044211 GO:0007631 GO:0006206 GO:0005524 GO:0071453 |
| tr|A0A024R957|A0A024R957_ HUMAN | GO:0016021 GO:0031965 GO:0007029 GO:0090435 GO:0032781 GO:0001671 GO:0051117 GO:0005789 |
| tr|A0A024R978|A0A024R978_ HUMAN | GO:0034976 GO:0001933 GO:0070062 GO:0045727 GO:0005515 GO:0001934 GO:0005737 GO:0005886 |
| tr|A0A024R983|A0A024R983_ HUMAN | GO:0009411 GO:0060271 GO:0007224 GO:0002520 GO:0006383 GO:0046872 GO:0030529 GO:0030620 GO:0005654 GO:0005737 |
| tr|A0A024R994|A0A024R994_ HUMAN | GO:0071363 GO:0016192 GO:0005829 GO:0006468 GO:0038128 GO:0004674 GO:0046474 GO:0005544 GO:0070062 GO:0044822 GO:0005925 GO:0030335 GO:0048306 GO:0071277 GO:0005215 GO:0030971 GO:0044281 GO:0005634 GO:0005886 |
| tr|A0A024R9B5|A0A024R9B5_ HUMAN | GO:0048188 GO:0043627 GO:0006974 GO:0005730 GO:0006355 GO:0044212 GO:0009790 GO:0051568 GO:0042800 GO:0035064 GO:0071339 |
| tr|A0A024R9B7|A0A024R9B7_ HUMAN | GO:0004129 GO:0016021 GO:0005751 GO:0006367 GO:1902600 GO:0022904 GO:0044281 |
| tr|A0A024R9C1|A0A024R9C1_ HUMAN | GO:0005925 GO:0031047 GO:0006413 GO:0016020 GO:0008022 GO:0000166 GO:1990124 GO:0045727 GO:0005829 GO:0071013 GO:0010494 GO:0048255 GO:0030425 GO:0008143 GO:2000623 GO:0045070 GO:0060213 GO:0070062 GO:0008494 GO:0000398 GO:0008266 GO:0006378 GO:0003729 |
| tr|A0A024R9D2|A0A024R9D2_ HUMAN | GO:0010508 GO:0000122 GO:0043066 GO:0044822 GO:0031965 GO:0045766 GO:0070830 GO:0031663 GO:0005789 GO:0043123 GO:0005730 GO:0016604 GO:0003713 GO:0001085 GO:0016021 GO:0051059 GO:0051897 GO:0005923 GO:0048471 GO:0003725 GO:0051092 GO:0046581 GO:0016324 |
| tr|A0A024R9D3|A0A024R9D3_ HUMAN | GO:0005925 GO:0000184 GO:0097421 GO:1904571 GO:0006413 GO:0006415 GO:0005515 GO:0016020 GO:0016259 GO:0022625 GO:0035368 GO:0003735 GO:0019083 GO:0006614 GO:0070062 GO:0005634 |
| tr|A0A024R9D7|A0A024R9D7_ HUMAN | GO:0070062 GO:0008670 GO:0006635 GO:0070402 GO:0005759 GO:0051289 GO:0005654 GO:0016651 |
| tr|A0A024R9D9|A0A024R9D9_ HUMAN | GO:0045893 GO:0016973 GO:0006357 GO:0000124 GO:0005643 GO:0070390 GO:0016578 GO:0061179 GO:0071819 GO:0003682 GO:0006368 GO:0030374 |
| tr|A0A024R9E4|A0A024R9E4_ HUMAN | GO:0012505 GO:0016324 GO:0005515 GO:0045121 GO:0016021 GO:0070062 GO:0048471 |
| tr|A0A024R9G0|A0A024R9G0_ HUMAN | GO:0031047 GO:0006385 GO:0006367 GO:0006370 GO:0006362 GO:0032481 GO:0034587 GO:0008270 GO:0045815 GO:0001055 GO:0005666 GO:0001056 GO:0006283 GO:0006363 GO:0006386 GO:0005829 GO:0005665 GO:0003677 GO:0009790 GO:0045814 GO:0035019 GO:0050434 GO:0000398 GO:0006368 GO:0006361 GO:0045087 GO:0006356 GO:0005736 GO:0001054 |
| tr|A0A024R9G3|A0A024R9G3_ HUMAN | GO:0036513 GO:0036502 GO:0032092 GO:0019060 GO:1990381 GO:0034098 GO:0031398 GO:0031648 GO:0042288 GO:0071712 GO:0005770 GO:0051117 GO:0043657 GO:0031625 GO:0005769 GO:0030970 GO:0004872 GO:0051260 GO:0030176 GO:0030968 |
| tr|A0A024R9G4|A0A024R9G4_ HUMAN | GO:0016021 GO:0070062 GO:0005929 GO:0005515 |
| tr|A0A024R9H2|A0A024R9H2_ HUMAN | GO:0046914 GO:1904012 GO:0030324 GO:0017148 GO:0036041 GO:0001822 GO:0044822 GO:1902074 GO:0090502 GO:0006449 GO:0005777 GO:0005829 GO:0009986 GO:0007420 GO:1904013 GO:0070314 GO:0050680 GO:0016892 GO:0070062 GO:0042803 GO:0033993 GO:0005634 GO:0005739 |
| tr|A0A024R9J0|A0A024R9J0_ HUMAN | GO:0000785 GO:0005654 GO:0034991 GO:0007067 GO:0005515 GO:0007131 GO:0016020 GO:0000236 GO:0006915 GO:0006302 GO:0005829 GO:0000775 GO:0007059 GO:0001228 GO:0000090 GO:0016925 GO:0045944 GO:0000084 GO:0043687 GO:0071168 GO:0000910 |
| tr|A0A024R9K7|A0A024R9K7_ HUMAN | GO:0072546 GO:0030246 |
| tr|A0A024R9L6|A0A024R9L6_ HUMAN | GO:0006468 GO:0032580 GO:0018279 GO:0030173 GO:0006054 GO:0003836 GO:0070062 GO:0043687 GO:0001574 GO:0009311 GO:0006488 GO:0097503 GO:0016266 GO:0018146 |
| tr|A0A024R9M3|A0A024R9M3_ HUMAN | GO:0070062 GO:0016020 |
| tr|A0A024R9N6|A0A024R9N6_ HUMAN | GO:0030100 GO:0071363 GO:0055038 GO:0005515 GO:0032456 GO:0006907 GO:0005525 GO:0005509 GO:0003676 GO:0050731 GO:0048471 GO:0070062 GO:0051260 GO:0005783 GO:0005886 GO:0031901 GO:0005634 GO:0005524 |
| tr|A0A024R9P6|A0A024R9P6_ HUMAN | GO:0016021 GO:0005874 GO:0000922 GO:0006915 GO:0006874 GO:0005741 GO:0030154 GO:0005515 GO:0005634 |
| tr|A0A024R9R4|A0A024R9R4_ HUMAN | GO:0018722 GO:0018721 GO:0006024 GO:0005796 GO:0080131 GO:0018727 GO:0016021 GO:0018726 GO:0018723 GO:0018724 GO:0005975 GO:0051922 GO:0050698 GO:0004394 GO:0001537 GO:0018725 GO:0001517 GO:0017095 GO:0008467 GO:0016232 GO:0050694 GO:0044281 GO:0034930 |
| tr|A0A024R9U3|A0A024R9U3_ HUMAN | GO:0016020 GO:0005768 GO:0005739 GO:0005515 |
| tr|A0A024R9V3|A0A024R9V3_ HUMAN | GO:0016021 GO:0005654 |
| tr|A0A024R9Y7|A0A024R9Y7_ HUMAN | GO:0005515 GO:0016020 |
| tr|A0A024R9Z0|A0A024R9Z0_ HUMAN | GO:0004129 GO:0006367 GO:0005751 GO:0008137 GO:0006120 GO:0005747 GO:0016021 GO:1902600 GO:0032403 GO:0070062 |
| tr|A0A024RA52|A0A024RA52_ HUMAN | GO:0048011 GO:0051436 GO:0002479 GO:0090263 GO:0007411 GO:0048010 GO:0000186 GO:0019773 GO:0006977 GO:0005829 GO:0002223 GO:0051437 GO:0005654 GO:0006595 GO:0031145 GO:0038061 GO:0007265 GO:0090090 GO:0033209 GO:0070062 GO:0016032 GO:0006521 GO:0043488 GO:0005515 GO:0000084 GO:0007173 GO:0038095 GO:0000932 GO:0050852 GO:0009615 GO:0004298 GO:0000090 GO:0043066 GO:0008543 GO:0000209 GO:0008286 |
| tr|A0A024RA75|A0A024RA75_ HUMAN | GO:0006574 GO:0051287 GO:0008442 GO:0005759 GO:0034641 GO:0004616 GO:0055114 |
| tr|A0A024RA81|A0A024RA81_ HUMAN | GO:0005829 GO:0005739 GO:0016311 GO:0009117 GO:0008253 GO:0046135 GO:0046085 GO:0006206 GO:0000287 GO:0008665 GO:0000166 GO:0005783 |
| tr|A0A024RAC0|A0A024RAC0_ HUMAN | GO:0021503 GO:0060840 GO:0003281 GO:0016020 GO:0005634 GO:0070062 |
| tr|A0A024RAC5|A0A024RAC5_ HUMAN | GO:0007067 GO:1900025 GO:0010762 GO:0000236 GO:0019901 GO:0044822 GO:0010971 GO:0051895 GO:0051301 GO:0007229 GO:0005730 GO:0005829 GO:0005874 GO:0008017 GO:0019904 GO:0007264 GO:1900027 GO:0090630 GO:0045184 GO:0034506 GO:0030496 GO:1990023 GO:0048365 GO:0000090 GO:0072356 GO:0005886 GO:0031901 GO:0034260 GO:0051987 |
| tr|A0A024RAC6|A0A024RAC6_ HUMAN | GO:0070449 GO:0003746 GO:0016021 GO:0003690 GO:0006357 GO:0050434 GO:0005615 GO:0006414 GO:0006368 GO:0005515 GO:0005737 |
| tr|A0A024RAD8|A0A024RAD8_ HUMAN | GO:0016021 GO:0055114 GO:0006537 GO:0042802 GO:0009055 GO:0034641 GO:0019470 GO:0003842 GO:0005759 GO:0010133 GO:0006561 GO:0004029 |
| tr|A0A024RAE1|A0A024RAE1_ HUMAN | GO:0005730 GO:0031965 GO:0005840 GO:0005654 GO:0005515 GO:0042254 GO:0044822 |
| tr|A0A024RAE4|A0A024RAE4_ HUMAN | GO:0045185 GO:0043525 GO:0035088 GO:0030742 GO:0043025 GO:0060684 GO:0042059 GO:0031333 GO:0051233 GO:0046330 GO:0072686 GO:0051022 GO:0035264 GO:0005525 GO:0031274 GO:0016567 GO:0051489 GO:0030496 GO:0033138 GO:0005789 GO:0036464 GO:0007088 GO:0045087 GO:0045177 GO:0042051 GO:0051988 GO:0043209 GO:1900026 GO:0060070 GO:0005925 GO:0042802 GO:0090316 GO:0007266 GO:0045860 GO:0001822 GO:0038096 GO:0031996 GO:0005815 GO:0007097 GO:0007596 GO:0072384 GO:0043552 GO:0031435 GO:0090135 GO:0061630 GO:0051149 GO:0060071 GO:0034332 GO:0048010 GO:0070062 GO:0031424 GO:0051835 GO:0060041 GO:0060789 GO:0060661 GO:0045494 GO:0043005 GO:0060501 GO:0032467 GO:0003334 GO:0010628 GO:0005829 GO:0002040 GO:0043497 GO:0003161 GO:0032402 GO:0060047 GO:0007411 GO:0048013 GO:0042176 GO:0030307 GO:0003924 GO:0021762 GO:0045740 GO:0009749 GO:0030141 GO:0017157 GO:0090136 GO:0031647 GO:0048554 GO:0071338 GO:0010629 GO:0005911 GO:0030010 GO:0042074 GO:0051017 GO:0030225 GO:0000322 GO:0030175 GO:0034191 GO:0036336 GO:0031295 GO:0031069 GO:0051683 GO:0016197 GO:0000139 GO:0048664 GO:0051056 GO:0007030 GO:0031256 |
| tr|A0A024RAG3|A0A024RAG3_ HUMAN | GO:2000786 GO:0048011 GO:0005525 GO:0060092 GO:0001928 GO:0030097 GO:0006915 GO:0032092 GO:0007507 GO:0031625 GO:0030496 GO:0032091 GO:0051117 GO:0019003 GO:0005776 GO:0005829 GO:0048701 GO:0060178 GO:0048070 GO:0007265 GO:0015816 GO:0005886 GO:0007626 GO:0070062 GO:0071902 GO:0006886 GO:0000910 GO:0000145 GO:0009267 GO:0006913 GO:1902475 GO:0071360 GO:0003924 GO:0098794 GO:0030036 GO:0008543 GO:0060080 GO:0015187 |
| tr|A0A024RAH8|A0A024RAH8_ HUMAN | GO:0010501 GO:0004004 GO:0005730 GO:0005524 GO:0044822 GO:0005515 GO:0071392 GO:0016020 |
| tr|A0A024RAI1|A0A024RAI1_ HUMAN | GO:0030056 GO:0005925 GO:0060271 GO:0051653 GO:0007411 GO:0050775 GO:0005903 GO:0002102 GO:0030027 GO:0005884 GO:0005829 GO:0005200 GO:0038096 GO:0007163 GO:0048013 GO:0000139 GO:0046677 GO:0045087 GO:0070062 GO:0060076 GO:0005911 GO:0016344 GO:0007264 GO:0051491 GO:0034314 GO:0005524 GO:0010592 GO:0051015 GO:0008356 GO:0005885 GO:0009743 GO:0043519 GO:0033206 |
| tr|A0A024RAJ2|A0A024RAJ2_ HUMAN | GO:0070063 GO:0044300 GO:0051020 GO:0043196 GO:0071156 GO:0015629 GO:0043679 GO:0008283 GO:0060987 GO:0060988 GO:0033268 GO:0048156 GO:0046982 GO:0045664 GO:0042802 GO:0043065 GO:0008021 GO:0048711 GO:0042692 GO:0045807 GO:0030018 GO:0030315 GO:0032403 GO:0016032 GO:0043547 GO:0043194 GO:0005634 |
| tr|A0A024RAJ4|A0A024RAJ4_ HUMAN | GO:0031369 GO:0045948 GO:0035019 GO:0000288 GO:0003697 GO:0006283 GO:0009790 GO:0034587 GO:0048739 GO:0003899 GO:0034402 GO:0003727 GO:0000166 GO:0050434 GO:0005665 GO:0006367 GO:0000932 GO:0006368 GO:0000398 GO:0031047 GO:0006370 GO:0030018 GO:0031990 |
| tr|A0A024RAJ6|A0A024RAJ6_ HUMAN | GO:0042803 GO:0008654 GO:0042340 GO:0050885 GO:0001501 GO:0030207 GO:0008375 GO:0030246 GO:0007605 GO:0042552 GO:0008049 GO:0006874 GO:0045944 GO:0016231 GO:0043202 GO:0019915 GO:0006044 GO:0007626 GO:0070062 GO:0001669 GO:0042582 GO:0006689 GO:0046982 GO:0044267 GO:0008360 GO:0043615 GO:0007341 GO:0048477 GO:0030214 GO:0016020 GO:0009313 GO:0007040 |
| tr|A0A024RAM0|A0A024RAM0_ HUMAN | GO:0005829 GO:0008565 GO:0006996 GO:0008536 GO:0006607 GO:0031965 GO:0000060 GO:0070062 GO:0072372 GO:0008139 GO:0044822 GO:0034399 GO:0043488 GO:0016032 GO:0006610 GO:0000059 |
| tr|A0A024RAQ1|A0A024RAQ1_ HUMAN | GO:0060546 GO:0005921 GO:0001701 GO:0005923 GO:0003697 GO:0003730 GO:0009566 GO:0007283 GO:0031100 GO:0048642 GO:0070935 GO:0071356 GO:0005844 GO:0003714 GO:0017048 GO:0046622 GO:0048471 GO:0001227 GO:0071474 GO:0000122 GO:1902219 GO:0008584 GO:0009409 GO:0000977 GO:0005634 GO:2000767 |
| tr|A0A024RAQ3|A0A024RAQ3_ HUMAN | GO:0051000 GO:0005654 GO:0005515 GO:0051870 GO:0004146 GO:0005829 GO:0006730 GO:0031103 GO:0006729 GO:0070402 GO:2000121 GO:0000083 GO:0046209 GO:0046655 GO:0005542 GO:0031427 GO:0055114 GO:0009165 GO:0046452 GO:0006545 GO:0003729 GO:0046654 |
| tr|A0A024RAS8|A0A024RAS8_ HUMAN | GO:0007623 GO:0042168 GO:0020037 GO:0005829 GO:0070062 GO:0005739 |
| tr|A0A024RAV2|A0A024RAV2_ HUMAN | GO:0032508 GO:0006310 GO:0006281 GO:0004003 GO:0036310 GO:0003677 GO:0005524 GO:0005515 GO:0000733 GO:0005654 GO:0016020 |
| tr|A0A024RAV4|A0A024RAV4_ HUMAN | GO:0060546 GO:0005921 GO:0001701 GO:0005923 GO:0003697 GO:0003730 GO:0009566 GO:0007283 GO:0031100 GO:0048642 GO:0070935 GO:0071356 GO:0005844 GO:0003714 GO:0017048 GO:0046622 GO:0048471 GO:0001227 GO:0071474 GO:0000122 GO:1902219 GO:0008584 GO:0009409 GO:0000977 GO:0005634 GO:2000767 GO:0016020 |
| tr|A0A024RAY2|A0A024RAY2_ HUMAN | GO:0009653 GO:0071944 GO:0097284 GO:0005198 GO:0043066 GO:0044822 GO:0045104 GO:0005730 GO:0033209 GO:0034451 GO:0045095 GO:0043000 GO:0048471 GO:0070062 GO:0016032 GO:0097191 GO:0097110 GO:0007049 |
| tr|A0A024RAZ7|A0A024RAZ7_ HUMAN | GO:1990826 GO:0019087 GO:0030324 GO:0051028 GO:0010628 GO:0016020 GO:0045760 GO:0000166 GO:1904579 GO:0003730 GO:0033592 GO:0035865 GO:0051170 GO:0036310 GO:0032212 GO:0032211 GO:0008584 GO:0005829 GO:0045111 GO:0061752 GO:0071013 GO:0071236 GO:0000733 GO:1904577 GO:0035690 GO:0000380 GO:0070062 GO:0019013 GO:0071364 GO:0098505 GO:1990825 GO:0008134 GO:0006405 |
| tr|A0A024RB03|A0A024RB03_ HUMAN | GO:0030659 GO:0032587 GO:0005815 GO:0038028 GO:0005509 GO:0031340 GO:0065002 GO:0005544 GO:0010828 GO:0090314 GO:0005938 |
| tr|A0A024RB14|A0A024RB14_ HUMAN | GO:0000184 GO:0006415 GO:0006413 GO:0005515 GO:0016020 GO:0016259 GO:0006414 GO:0005730 GO:0033119 GO:0003735 GO:0019083 GO:0006614 GO:0070062 GO:0003729 GO:0022627 |
| tr|A0A024RB17|A0A024RB17_ HUMAN | GO:0046872 GO:0030176 GO:0044232 GO:0008289 GO:0005886 GO:0005515 GO:0006869 |
| tr|A0A024RB22|A0A024RB22_ HUMAN | GO:0043044 GO:0045893 GO:0000980 GO:0005654 GO:0000122 GO:0071565 GO:0016514 GO:0031492 GO:0006337 GO:0071564 GO:0021882 GO:0017053 GO:0000978 GO:0003713 GO:0000790 |
| tr|A0A024RB32|A0A024RB32_ HUMAN | GO:0000781 GO:0002039 GO:0043005 GO:0060430 GO:0005654 GO:0010628 GO:0050220 GO:0005884 GO:1900034 GO:0060548 GO:0042921 GO:0051879 GO:0070389 GO:0019371 GO:0008283 GO:0005697 GO:0005829 GO:0003720 GO:0043025 GO:0043234 GO:0005978 GO:0051082 GO:0000723 GO:0006278 GO:0043588 GO:0019233 GO:0048471 GO:0070062 GO:0019899 |
| tr|A0A024RB62|A0A024RB62_ HUMAN | GO:0005730 GO:0036265 GO:0043527 GO:0005515 GO:0030488 GO:0000049 GO:0005654 GO:0008176 |
| tr|A0A024RB72|A0A024RB72_ HUMAN | GO:0030126 GO:0005829 GO:0018279 GO:0030133 GO:0006890 GO:0006891 GO:0043687 GO:0006888 GO:0048205 GO:1901998 GO:0006886 GO:0002088 |
| tr|A0A024RB75|A0A024RB75_ HUMAN | GO:0001843 GO:0016021 GO:0043005 GO:0008284 GO:0097225 GO:0031625 GO:0071158 GO:0031594 GO:0044853 GO:0001707 GO:2000810 GO:0005759 GO:0043457 GO:0004712 GO:0004691 GO:0031588 GO:0005952 GO:0046827 GO:0018107 GO:1901621 GO:0006107 GO:0017137 GO:0048240 GO:0044822 GO:0070062 GO:0097546 GO:0048471 GO:0006084 GO:0032403 GO:0051966 GO:0004108 GO:0005794 GO:0071333 GO:0045667 GO:0005975 GO:0005524 GO:0071374 GO:0018105 GO:0019901 GO:0046777 GO:0061136 GO:0051447 GO:0006099 GO:0034237 GO:0005634 GO:0005813 GO:0070613 |
| tr|A0A024RB85|A0A024RB85_ HUMAN | GO:0005654 GO:0016020 GO:0043066 GO:0045597 GO:0044822 GO:0008283 GO:0016787 GO:0005737 GO:0005730 GO:0007050 GO:0045892 GO:0031625 GO:0003677 GO:0006417 GO:0030529 GO:0070062 GO:0006364 GO:0003700 |
| tr|A0A024RB87|A0A024RB87_ HUMAN | GO:0061028 GO:0005525 GO:0070374 GO:0007507 GO:0004708 GO:0019003 GO:0005829 GO:0015031 GO:0032525 GO:0045121 GO:0030168 GO:2000114 GO:0003428 GO:0005886 GO:1901888 GO:0070062 GO:0005911 GO:0005811 GO:0032403 GO:0032486 GO:0071320 GO:0008283 GO:0035690 GO:2000301 GO:0003924 GO:0097211 GO:0009743 GO:0030033 |
| tr|A0A024RBA9|A0A024RBA9_ HUMAN | GO:0005925 GO:0050775 GO:2000643 GO:0005802 GO:0017157 GO:0030659 GO:0005515 GO:0098559 GO:0008089 GO:0005525 GO:0015031 GO:0005789 GO:0007264 GO:0032154 GO:0048260 GO:0030424 GO:0032580 GO:0070062 GO:0019003 GO:0003924 GO:0009898 GO:0030516 |
| tr|A0A024RBD0|A0A024RBD0_ HUMAN | GO:0005829 GO:0052845 GO:0008828 GO:0046488 GO:0050072 GO:0052843 GO:0052848 GO:0046872 GO:0019177 GO:0008413 GO:0046831 GO:0008758 GO:0036222 GO:0043647 GO:0019176 GO:0043141 GO:0052847 GO:0036218 GO:0004787 GO:0035870 GO:0005515 GO:0009187 GO:0030515 GO:0052840 GO:0019935 GO:0008796 GO:0052844 GO:0052846 GO:0044606 GO:0019722 GO:0008486 |
| tr|A0A024RBE7|A0A024RBE7_ HUMAN | GO:0005521 GO:0016021 GO:0031468 GO:0006355 GO:0003677 GO:0000785 GO:0005789 GO:0005637 |
| tr|A0A024RBH2|A0A024RBH2_ HUMAN | GO:0005856 GO:0005789 GO:0009986 GO:0005811 GO:0005886 GO:0044267 GO:0016021 GO:0042599 GO:0070062 GO:0048471 GO:0044822 |
| tr|A0A024RBL2|A0A024RBL2_ HUMAN | GO:0030170 GO:0006520 GO:0004794 GO:0070062 GO:0005739 GO:0003941 |
| tr|A0A024RBR1|A0A024RBR1_ HUMAN | GO:0007067 GO:0030659 GO:0008270 GO:0044861 GO:0000236 GO:0035371 GO:0005881 GO:0005882 GO:0044354 GO:0005829 GO:0003676 GO:0000776 GO:0001726 GO:0007264 GO:0005813 GO:0001578 GO:0000090 GO:0042803 GO:0031116 GO:0051010 |
| tr|A0A024RBR3|A0A024RBR3_ HUMAN | GO:0032790 GO:0002192 GO:0005515 GO:0001731 GO:0003743 |
| tr|A0A024RBR4|A0A024RBR4_ HUMAN | GO:0005856 GO:0030665 GO:0035091 GO:0005905 GO:0003779 GO:0030276 GO:0072583 GO:0006915 GO:0048471 |
| tr|A0A024RBV2|A0A024RBV2_ HUMAN | GO:0050872 GO:0071333 GO:0005515 GO:0071394 GO:0030729 GO:0046951 GO:0045471 GO:0071397 GO:0005829 GO:0006631 GO:0042594 GO:0042493 GO:0060612 GO:0001889 GO:0034201 GO:0014074 GO:0005524 GO:0047760 GO:0007584 GO:0032024 |
| tr|A0A024RBX9|A0A024RBX9_ HUMAN | GO:0004739 GO:0046487 GO:0061732 GO:0043209 GO:0034604 GO:0006099 GO:0006006 GO:0005967 GO:0010510 GO:0005634 |
| tr|A0A024RC37|A0A024RC37_ HUMAN | GO:0016591 GO:0070940 GO:0005515 |
| tr|A0A024RC65|A0A024RC65_ HUMAN | GO:0072015 GO:0005925 GO:1990138 GO:0036057 GO:0030426 GO:0030496 GO:0071364 GO:0043234 GO:0005884 GO:0043547 GO:0036120 GO:0005829 GO:0043539 GO:0036464 GO:0034260 GO:0048008 GO:0030424 GO:0005547 GO:0044281 GO:0006112 GO:0031234 GO:0070062 GO:0071277 GO:0071902 GO:0032403 GO:0019904 GO:0007264 GO:0007173 GO:0001817 GO:0035305 GO:0019901 GO:0019903 GO:0005874 GO:0005095 GO:0005516 GO:0005509 GO:0050796 GO:0005096 GO:0008543 GO:0001726 GO:0044548 GO:0016328 GO:0048365 |
| tr|A0A024RCA7|A0A024RCA7_ HUMAN | GO:0005925 GO:0000184 GO:0006415 GO:0006413 GO:0005515 GO:0016020 GO:0043021 GO:0016259 GO:0006414 GO:0022625 GO:0003735 GO:0019083 GO:0006614 GO:0070062 |
| tr|A0A024RCB9|A0A024RCB9_ HUMAN | GO:0005515 |
| tr|A0A024RCL8|A0A024RCL8_ HUMAN | GO:0019731 GO:0070062 GO:0005615 GO:0000788 GO:0002227 GO:0003677 GO:0006334 GO:0046982 GO:0005654 GO:0005737 GO:0050830 |
| tr|A0A024RCM3|A0A024RCM3_ HUMAN | GO:1904707 GO:0016021 GO:0005681 GO:0017070 GO:0016363 GO:0045727 GO:0005687 GO:0061051 GO:2000573 GO:0004004 GO:0001889 GO:0000245 GO:0000346 GO:0046784 GO:0030621 GO:0005737 GO:0044822 GO:0032403 GO:2000002 GO:0005524 GO:0005688 GO:0010501 GO:0043008 GO:0032786 GO:0016607 |
| tr|A0A024RCN6|A0A024RCN6_ HUMAN | GO:0005829 GO:0004832 GO:0002161 GO:0006438 GO:0005739 GO:0006450 GO:0005524 GO:0005515 |
| tr|A0A024RCR6|A0A024RCR6_ HUMAN | GO:0005654 GO:0071816 GO:0016020 GO:0042981 GO:0071818 GO:1990381 GO:0051276 GO:0048513 GO:0043022 GO:0018393 GO:0006511 GO:0044702 GO:0045861 GO:0031625 GO:1904294 GO:0051787 GO:0042771 GO:1904378 |
| tr|A0A024RCS7|A0A024RCS7_ HUMAN | GO:0016020 GO:0005815 GO:0007080 GO:0007283 GO:0051301 GO:0005874 GO:0003777 GO:0008017 GO:0005819 GO:0007596 GO:0090307 GO:0005769 GO:0016887 GO:0007018 GO:0005871 GO:0005634 GO:0005524 GO:0008152 |
| tr|A0A024RCX8|A0A024RCX8_ HUMAN | GO:0006457 GO:0000398 GO:0005515 GO:0000413 GO:0003755 GO:0070062 GO:0071013 |
| tr|A0A024RCZ1|A0A024RCZ1_ HUMAN | GO:0006998 GO:0043409 GO:0051898 GO:0022008 GO:0035914 GO:0005639 GO:0060914 |
| tr|A0A024RCZ8|A0A024RCZ8_ HUMAN | GO:0005737 GO:0005515 GO:0008152 GO:0005871 GO:0003777 GO:0005874 |
| tr|A0A024RD03|A0A024RD03_ HUMAN | GO:0070126 GO:0070125 GO:0005743 GO:0098792 GO:0003735 GO:0098779 GO:0005763 GO:0070124 GO:0002230 |
| tr|A0A024RD07|A0A024RD07_ HUMAN | GO:0002224 GO:0005788 GO:0005102 |
| tr|A0A024RD08|A0A024RD08_ HUMAN | GO:0006810 GO:0016021 GO:0045161 GO:0005743 GO:0005515 GO:0009966 GO:0006919 |
| tr|A0A024RD30|A0A024RD30_ HUMAN | GO:0003746 GO:0043231 GO:0072344 GO:0070966 GO:0005515 GO:0003924 GO:0005525 |
| tr|A0A024RD36|A0A024RD36_ HUMAN | GO:0005730 GO:0001825 GO:0003735 GO:0002181 GO:0000463 GO:0044822 GO:0022625 |
| tr|A0A024RD78|A0A024RD78_ HUMAN | GO:0070126 GO:0005840 GO:0070125 GO:0005743 GO:0003735 GO:0044822 GO:0070124 |
| tr|A0A024RD80|A0A024RD80_ HUMAN | GO:0009986 GO:0031526 GO:0005525 GO:0002135 GO:0043524 GO:0071407 GO:0019062 GO:0002134 GO:0051082 GO:0016324 GO:0032092 GO:0017098 GO:0007411 GO:0045429 GO:1990913 GO:0031396 GO:0005829 GO:0032564 GO:0016323 GO:1903660 GO:0005654 GO:0009651 GO:0038096 GO:0060334 GO:0060338 GO:0035872 GO:0006986 GO:0008144 GO:0032435 GO:0044325 GO:0045793 GO:0003725 GO:1990917 GO:0008180 GO:0042220 GO:0044822 GO:0070062 GO:0071902 GO:0005739 GO:0001890 GO:0061741 GO:0005524 GO:0001948 GO:0042470 GO:0005765 GO:0071353 GO:0030911 GO:0019901 GO:0019887 GO:0035690 GO:0097435 GO:1900034 GO:0016234 GO:0030235 GO:0006457 GO:0033160 GO:0061635 GO:0042826 GO:0023026 |
| tr|A0A024RD93|A0A024RD93_ HUMAN | GO:0005829 GO:0004639 GO:0070062 GO:0004638 GO:0042802 GO:0005524 GO:0046084 GO:0000082 GO:0016020 GO:0006189 |
| tr|A0A024RDA1|A0A024RDA1_ HUMAN | GO:0005829 GO:0006996 GO:0098592 GO:0044267 GO:0000145 GO:0061024 GO:0006887 GO:0050714 GO:0048015 GO:0005546 GO:0017049 GO:0051601 GO:0006893 |
| tr|A0A024RDB0|A0A024RDB0_ HUMAN | GO:0005829 GO:0016021 GO:0007612 GO:0021764 GO:0060996 GO:0007626 GO:0005524 GO:0042787 GO:0019780 GO:0005515 GO:0021766 GO:0004839 |
| tr|A0A024RDE5|A0A024RDE5_ HUMAN | GO:0007265 GO:0007253 GO:0044822 GO:0030159 GO:0005737 |
| tr|A0A024RDE8|A0A024RDE8_ HUMAN | GO:0005080 GO:0043005 GO:0008270 GO:0015629 GO:0047485 GO:0030159 GO:0005829 GO:0042805 GO:0045211 GO:0003779 GO:0061001 GO:0014069 GO:0030054 GO:0030018 GO:0061049 GO:0051963 |
| tr|A0A024RDF4|A0A024RDF4_ HUMAN | GO:0045893 GO:0005654 GO:0051602 GO:0042826 GO:0071732 GO:0000166 GO:0006913 GO:1904383 GO:0051592 GO:0014076 GO:0042162 GO:0045727 GO:0097167 GO:0005829 GO:0048255 GO:0061158 GO:1904586 GO:0021549 GO:1901355 GO:0030529 GO:0000291 GO:0071392 GO:0070062 GO:0019013 GO:0003682 GO:0001889 GO:0003680 GO:0000398 GO:0035925 GO:0042752 GO:0008134 GO:0071230 GO:1990828 |
| tr|A0A024RDG1|A0A024RDG1_ HUMAN | GO:0000278 GO:0090498 GO:0012507 GO:0008565 GO:0048219 GO:0044822 GO:0006886 GO:0005730 GO:0048280 GO:0005829 GO:0007030 GO:0048211 GO:0042802 GO:0005795 GO:0048208 GO:0000088 GO:0045056 GO:0048471 GO:0051260 GO:0005783 GO:0043687 GO:0018279 |
| tr|A0A024RDG6|A0A024RDG6_ HUMAN | GO:0046718 GO:0016021 GO:0005765 GO:0001618 GO:0070062 GO:0043202 GO:0019899 GO:0007155 GO:0005925 GO:0006622 |
| tr|A0A024RDH2|A0A024RDH2_ HUMAN | GO:0007179 GO:0070979 GO:0006915 GO:0006281 GO:0034138 GO:0034142 GO:0043161 GO:0061631 GO:0005829 GO:0002223 GO:0005654 GO:0061418 GO:0030509 GO:0005886 GO:0070062 GO:0006367 GO:0005515 GO:0010008 GO:0000122 GO:0005524 GO:0032480 GO:0038095 GO:0035666 GO:0050852 GO:0000151 GO:0051865 GO:1903955 GO:0006513 GO:0070936 |
| tr|A0A024RDH6|A0A024RDH6_ HUMAN | GO:0048666 GO:0000139 GO:0036498 GO:0002474 GO:0048306 GO:0051592 GO:0030127 GO:0015031 GO:0005789 GO:0005829 GO:0070971 GO:0019886 GO:0018108 GO:0048208 GO:0004714 GO:0048471 GO:0043687 GO:0000187 GO:0018279 |
| tr|A0A024RDH8|A0A024RDH8_ HUMAN | GO:0000184 GO:0006415 GO:0006413 GO:0016259 GO:0006414 GO:0022625 GO:0005730 GO:0003735 GO:0003723 GO:0019083 GO:0006614 GO:0070062 GO:0042254 GO:0005739 |
| tr|A0A024RDI6|A0A024RDI6_ HUMAN | GO:0034656 GO:0046709 GO:0019144 GO:0070062 GO:0043262 GO:0046032 GO:0047631 GO:0005759 |
| tr|A0A024RDJ4|A0A024RDJ4_ HUMAN | GO:0048011 GO:0050728 GO:0042803 GO:0007249 GO:0071322 GO:0043005 GO:0034138 GO:0090263 GO:0032481 GO:0034142 GO:0000980 GO:2000630 GO:0005829 GO:0002223 GO:0045083 GO:0034162 GO:0005654 GO:0008134 GO:1900127 GO:0034166 GO:0071356 GO:0035994 GO:0032375 GO:1904630 GO:0071316 GO:0038061 GO:0006979 GO:0045944 GO:0038124 GO:0034146 GO:0002755 GO:0071222 GO:0032269 GO:0031293 GO:0051403 GO:0033256 GO:0001227 GO:0046688 GO:0005739 GO:0051092 GO:0010744 GO:0000122 GO:0003682 GO:0038095 GO:0035666 GO:0071354 GO:0071347 GO:1990416 GO:0010956 GO:0050852 GO:0071375 GO:1904632 GO:0046982 GO:0034134 GO:0010884 GO:0038123 GO:0043066 GO:0071359 GO:0071260 GO:0001205 GO:0031072 GO:0042805 |
| tr|A0A024RDL1|A0A024RDL1_ HUMAN | GO:0001669 GO:0044183 GO:0071987 GO:1904851 GO:0044822 GO:0032212 GO:0044297 GO:0050821 GO:0044267 GO:0005832 GO:0005874 GO:1904874 GO:0007339 GO:1904871 GO:0051082 GO:1901998 GO:0051086 GO:0070062 GO:0005524 GO:0002199 |
| tr|A0A024RDL8|A0A024RDL8_ HUMAN | GO:0007626 GO:0005515 GO:0070852 GO:0000050 GO:0009791 GO:0006527 GO:0006475 GO:0005829 GO:0005741 GO:0004056 GO:0048471 GO:0070062 GO:0042450 GO:0005783 GO:0001889 GO:0019676 GO:0000053 GO:0051262 GO:0005634 GO:0006595 GO:0043204 |
| tr|A0A024RDL9|A0A024RDL9_ HUMAN | GO:0033574 GO:0005829 GO:0043005 GO:0005509 GO:0016311 GO:0031667 GO:0034641 GO:0000287 GO:0004647 GO:0042803 GO:0009612 GO:0006564 |
| tr|A0A024RDQ0|A0A024RDQ0_ HUMAN | GO:0043524 GO:0071682 GO:1903753 GO:0005829 GO:0005654 GO:0051085 GO:0006986 GO:0006898 GO:0045345 GO:0045944 GO:0070062 GO:0005524 GO:1903751 GO:0061098 GO:0005874 GO:1900034 GO:0051135 GO:0070507 GO:1903748 GO:0043014 |
| tr|A0A024RDR0|A0A024RDR0_ HUMAN | GO:0033034 GO:2001200 GO:0000401 GO:0006334 GO:0070182 GO:0002270 GO:0005769 GO:0051450 GO:0097350 GO:0042056 GO:0034165 GO:0046330 GO:0043280 GO:1901224 GO:0003697 GO:0032072 GO:0003681 GO:0034341 GO:0014911 GO:0017053 GO:0042393 GO:0006309 GO:0007623 GO:0051103 GO:0002053 GO:0042104 GO:0045663 GO:0070491 GO:0050786 GO:0002840 GO:0010976 GO:0006284 GO:0050831 GO:0002643 GO:0006265 GO:0002407 GO:0003727 GO:0043371 GO:0090303 GO:0030295 GO:0030324 GO:0044822 GO:0045944 GO:0045322 GO:0002437 GO:0019958 GO:0002281 GO:0045063 GO:0009986 GO:0005886 GO:0032868 GO:0001530 GO:0050930 GO:0010508 GO:0005654 GO:0007204 GO:0032760 GO:0032496 GO:0010858 GO:0045931 GO:0005793 GO:0008201 GO:0042277 GO:0000402 GO:0017055 GO:2000778 GO:0071642 GO:0050727 GO:0000902 GO:0032728 GO:0090026 GO:0043005 GO:0034137 GO:0003690 GO:0005829 GO:0003700 GO:0005615 GO:0033151 GO:0032392 GO:0034145 GO:2000426 GO:0046983 GO:0044378 GO:0051384 GO:0001786 GO:0016829 GO:0045819 GO:0009749 GO:0003684 GO:2000819 GO:0000793 GO:0032733 GO:0097100 GO:1990774 GO:0051861 GO:0032735 GO:0000405 GO:0001773 GO:0000790 GO:0035767 GO:0008097 GO:0032689 GO:0009408 GO:0001935 GO:0035711 GO:0045639 GO:0051106 GO:0043388 GO:0070374 GO:1903672 GO:0050718 GO:0002322 GO:0005125 GO:0003725 GO:0006338 GO:0042493 GO:0008156 GO:1902741 GO:0071347 GO:0032425 GO:0031532 GO:0002755 GO:0001654 GO:0032757 GO:0043537 |
| tr|A0A024RDY3|A0A024RDY3_ HUMAN | GO:0046718 GO:0061474 GO:0005887 GO:0043323 GO:0072594 GO:0097208 GO:1902513 GO:0042470 GO:0050821 GO:0001618 GO:0005770 GO:0048102 GO:0030425 GO:0044194 GO:0019904 GO:0042383 GO:0043025 GO:0009897 GO:0005771 GO:0008021 GO:0006914 GO:0048471 GO:0070062 GO:0008626 GO:0019899 GO:0090160 GO:0010008 |
| tr|A0A024RE04|A0A024RE04_ HUMAN | GO:0045893 GO:0045579 GO:0005515 GO:2000117 GO:0043066 GO:0043488 GO:0090267 GO:0048536 |
| tr|A0A024RE27|A0A024RE27_ HUMAN | GO:0046872 GO:0008152 GO:0005739 GO:0052770 GO:0003923 GO:0052771 |
| tr|A0A075B746|A0A075B746_ HUMAN | GO:0070126 GO:0070125 GO:0005743 GO:0003735 GO:0005763 GO:0044822 GO:0070124 |
| tr|A0A075BSP6|A0A075BSP6_ HUMAN | GO:0043171 GO:0008270 GO:0005164 GO:0005788 GO:0045766 GO:0009617 GO:0005615 GO:0005138 GO:0005789 GO:0005151 GO:0005829 GO:0045444 GO:0006509 GO:0045088 GO:0002250 GO:0042277 GO:0016021 GO:0019885 GO:0070062 GO:0005886 GO:0070006 GO:0008217 |
| tr|A0A087WT12|A0A087WT12_ HUMAN | GO:0042744 GO:0098869 GO:0007275 GO:0007283 GO:0007568 GO:0005743 GO:0032355 GO:0005829 GO:0004602 GO:0019372 GO:0005635 GO:0006749 GO:0043295 GO:0008430 GO:0019369 GO:0055114 GO:0070062 GO:0050727 GO:0006644 GO:0047066 GO:0006979 GO:0006325 |
| tr|A0A087WT20|A0A087WT20_ HUMAN | GO:0030054 GO:0000462 GO:0005730 GO:0080008 GO:0005813 GO:0016567 GO:0044822 GO:0032040 GO:0005737 |
| tr|A0A087WT44|A0A087WT44_ HUMAN | GO:0055085 GO:0001666 GO:0006979 GO:0016021 GO:0042167 GO:0046872 GO:0004392 GO:0006879 GO:0005515 GO:0044281 GO:0005789 GO:0005886 GO:0006788 |
| tr|A0A087WT45|A0A087WT45_ HUMAN | GO:0072562 GO:0005768 GO:0005515 |
| tr|A0A087WTA5|A0A087WTA5_ HUMAN | GO:0046523 GO:0009749 GO:0001541 GO:0019509 GO:0014003 GO:0043434 GO:0032057 GO:0003743 GO:0005829 GO:0009408 GO:0031369 GO:0043547 GO:0042552 GO:0005851 GO:0005085 |
| tr|A0A087WUB9|A0A087WUB9_ HUMAN | GO:0005681 GO:0000398 GO:0043065 GO:0019899 GO:0000974 GO:0005654 GO:0005737 GO:0016445 GO:0016020 |
| tr|A0A087WUE9|A0A087WUE9_ HUMAN | GO:0005856 GO:0006378 GO:0007155 GO:0005923 GO:0005515 GO:0035307 GO:0005654 GO:0005737 GO:0005886 |
| tr|A0A087WUK2|A0A087WUK2_ HUMAN | GO:0019013 GO:0006396 GO:0003690 GO:0005681 GO:0070062 GO:0016071 GO:0006355 GO:0008143 GO:0044822 GO:0005515 GO:0034046 GO:0000166 GO:0003697 GO:0005654 GO:0005737 |
| tr|A0A087WUT6|A0A087WUT6_ HUMAN | GO:0003743 GO:0005829 GO:0044822 GO:0005515 GO:0003924 GO:0006446 GO:0005525 GO:0005634 |
| tr|A0A087WV05|A0A087WV05_ HUMAN | GO:0005739 GO:0006355 GO:0007283 GO:0005515 GO:0003713 GO:0005634 |
| tr|A0A087WV66|A0A087WV66_ HUMAN | GO:0031100 GO:0006259 GO:0005730 GO:0008022 GO:0030212 GO:0000775 GO:0005524 GO:0014070 GO:0044822 GO:0072574 GO:0000793 GO:1990705 GO:0007126 GO:0034605 GO:0005737 GO:0016020 |
| tr|A0A087WVC1|A0A087WVC1_ HUMAN | GO:0010501 GO:0004004 GO:0005730 GO:0005524 GO:0044822 GO:0005886 |
| tr|A0A087WW40|A0A087WW40_ HUMAN | GO:2000786 GO:0031410 GO:0090148 GO:0042803 GO:1900740 GO:1902254 GO:0030496 GO:0005783 GO:0043234 GO:0005829 GO:1903778 GO:0043552 GO:0042171 GO:0000139 GO:0032465 GO:0000421 GO:0070062 GO:0005504 GO:0042149 GO:0034198 GO:0048102 GO:0032461 GO:0051084 GO:0005741 GO:1903527 GO:0032801 GO:1903955 GO:0031647 GO:1902255 GO:0006654 |
| tr|A0A087WW66|A0A087WW66_ HUMAN | GO:0048011 GO:0051436 GO:0030234 GO:0008540 GO:0016021 GO:0002479 GO:0090263 GO:0007411 GO:0048010 GO:0000186 GO:0006977 GO:0002223 GO:0051437 GO:0005654 GO:0006595 GO:0031145 GO:0038061 GO:0034515 GO:0007265 GO:0004175 GO:0090090 GO:0033209 GO:0070062 GO:0016032 GO:0006521 GO:0043488 GO:0005515 GO:0000084 GO:0007173 GO:0038095 GO:0050852 GO:0000090 GO:0043066 GO:0008543 GO:0000209 GO:0008286 |
| tr|A0A087WWM0|A0A087WWM0_ HUMAN | GO:0005829 GO:0018279 GO:0000139 GO:0030008 GO:0043687 GO:0048208 GO:0005515 GO:0005783 |
| tr|A0A087WWP4|A0A087WWP4_ HUMAN | GO:0060674 GO:0001569 GO:0060412 GO:0045638 GO:0005515 GO:0000166 GO:0016607 GO:0044822 GO:0031965 GO:0045892 GO:0007221 GO:0016032 GO:0000398 GO:0048536 |
| tr|A0A087WWS1|A0A087WWS1_ HUMAN | GO:0000445 GO:0045171 GO:0016363 GO:0048297 GO:0008380 GO:0000784 GO:0046784 GO:0007165 GO:0042981 GO:0005737 GO:2000002 GO:0005515 GO:0003677 GO:0003723 GO:0031297 GO:0006397 GO:0032786 GO:0016607 |
| tr|A0A087WXR2|A0A087WXR2_ HUMAN | GO:0035360 GO:0008289 GO:0005902 GO:0005515 GO:0032782 GO:0016020 GO:0005829 GO:0031514 GO:0046581 |
| tr|A0A087WXS7|A0A087WXS7_ HUMAN | GO:0045048 GO:0044267 GO:0005730 GO:0046872 GO:0015105 GO:0070062 GO:0005524 GO:0015698 GO:0016887 GO:0005515 GO:0036498 GO:0005789 |
| tr|A0A087WY71|A0A087WY71_ HUMAN | GO:0007411 GO:1903077 GO:0030122 GO:0042059 GO:0030133 GO:0030141 GO:0048011 GO:0048013 GO:0050750 GO:0036020 GO:0006886 GO:0005829 GO:0019886 GO:0050690 GO:0007268 GO:0005215 GO:0008289 GO:0043195 GO:0070062 GO:0016032 GO:0072583 GO:0044325 GO:0005048 GO:0035615 GO:0005739 |
| tr|A0A087WY88|A0A087WY88_ HUMAN | GO:0006887 GO:0038158 GO:1990266 GO:0005515 GO:0044425 GO:0030223 GO:0050832 GO:0005783 GO:0002446 |
| tr|A0A087WYF6|A0A087WYF6_ HUMAN | GO:0005730 GO:0005769 GO:0042147 GO:0071203 GO:0016020 |
| tr|A0A087WYM3|A0A087WYM3_ HUMAN | GO:0048487 GO:0044224 GO:0008759 GO:0034876 GO:0018748 GO:0034882 GO:0072686 GO:0045599 GO:0034571 GO:0035035 GO:0034885 GO:0030426 GO:0034983 GO:0061433 GO:0070933 GO:1900195 GO:0043491 GO:0030496 GO:0071872 GO:0018749 GO:0008134 GO:0034573 GO:0010507 GO:0033270 GO:0031641 GO:0045087 GO:0070446 GO:0043909 GO:0008270 GO:1900119 GO:0052773 GO:0043130 GO:0006348 GO:0043220 GO:0048715 GO:0043066 GO:0051775 GO:0043747 GO:0034576 GO:0047419 GO:0033010 GO:0045944 GO:0005677 GO:0007096 GO:0010801 GO:0000781 GO:0043748 GO:0005814 GO:0070932 GO:0005886 GO:0097456 GO:0051987 GO:2000777 GO:0006471 GO:0042826 GO:0070403 GO:0048012 GO:0043204 GO:2000378 GO:0044242 GO:0035729 GO:0042903 GO:0022011 GO:0005829 GO:0042177 GO:0000183 GO:0043219 GO:0072687 GO:0061428 GO:0003950 GO:0045843 GO:0046970 GO:0021762 GO:0014065 GO:0035748 GO:0034599 GO:0045836 GO:1901026 GO:0008418 GO:0048471 GO:0043388 GO:0003682 GO:0005874 GO:1900425 GO:0043864 GO:0052790 GO:0005720 GO:1900226 GO:0090042 GO:0071219 GO:0034781 |
| tr|A0A087WYR0|A0A087WYR0_ HUMAN | GO:0006412 GO:0005829 GO:0005786 GO:0005739 GO:0005730 GO:0008312 GO:0044822 GO:0042493 GO:0005654 GO:0006614 |
| tr|A0A087WZ13|A0A087WZ13_ HUMAN | GO:0005737 GO:0000398 GO:0005634 GO:0000166 GO:0044822 |
| tr|A0A087WZH7|A0A087WZH7_ HUMAN | GO:0005080 GO:0005925 GO:0043085 GO:0005543 GO:0044463 GO:0015629 GO:0042585 GO:0044281 GO:0006112 GO:0048513 GO:0005886 GO:0070062 GO:0045202 GO:0030425 GO:0005516 GO:0010976 GO:0051015 GO:0050796 GO:0005813 GO:0005938 |
| tr|A0A087WZN1|A0A087WZN1_ HUMAN | GO:0051287 GO:0006103 GO:0006734 GO:0004449 GO:0009055 GO:0006099 GO:0000287 GO:0005759 GO:0006102 GO:0005634 |
| tr|A0A087WZT2|A0A087WZT2_ HUMAN | GO:0034933 GO:0052666 GO:0043803 GO:0009008 GO:0043851 GO:0018707 GO:0018423 GO:0008172 GO:0016435 GO:0016428 GO:0071424 GO:0034931 GO:0016205 GO:0009019 GO:0016279 GO:0043834 GO:0043776 GO:0052624 GO:0070062 GO:0043777 GO:0034807 GO:0052667 GO:0032259 GO:0043833 GO:0043780 GO:0009383 GO:0008988 GO:0008174 GO:0030792 GO:0043827 GO:0051994 GO:0043791 GO:0004809 GO:0000179 GO:0052735 GO:0034541 GO:0043852 GO:0052665 GO:0043770 GO:0043782 GO:0019702 GO:0008425 GO:0005811 GO:0005789 GO:0080012 GO:0008650 GO:0070677 |
| tr|A0A087WZT3|A0A087WZT3_ HUMAN | GO:0070062 |
| tr|A0A087X054|A0A087X054_ HUMAN | GO:0005925 GO:0051087 GO:0005790 GO:0016020 GO:0036498 GO:0005788 GO:0006898 GO:0044267 GO:0002931 GO:1903298 GO:0006888 GO:0071682 GO:0034663 GO:0070062 GO:0005524 GO:1903382 |
| tr|A0A087X0H9|A0A087X0H9_ HUMAN | GO:0044822 GO:0005515 GO:0010923 |
| tr|A0A087X0Q1|A0A087X0Q1_ HUMAN | GO:0005515 GO:0044822 GO:1990247 |
| tr|A0A087X0W9|A0A087X0W9_ HUMAN | GO:0006508 GO:0036459 |
| tr|A0A087X0X1|A0A087X0X1_ HUMAN | GO:0005739 GO:0005544 GO:0005509 GO:0005515 |
| tr|A0A087X176|A0A087X176_ HUMAN | GO:0031965 GO:0005615 GO:0006457 GO:0005886 GO:0045454 GO:0016971 GO:0003756 GO:0030173 GO:0055114 |
| tr|A0A087X1B1|A0A087X1B1_ HUMAN | GO:0042348 GO:0004871 GO:0006954 GO:0042803 GO:0051650 GO:0007250 GO:0034138 GO:0032481 GO:0008385 GO:0034142 GO:0031625 GO:0006974 GO:0070530 GO:0046872 GO:0002223 GO:0034162 GO:0034166 GO:1901215 GO:0043123 GO:0010803 GO:0001782 GO:0007254 GO:0045944 GO:0038124 GO:0072686 GO:0034146 GO:0002755 GO:0000922 GO:0016032 GO:0019904 GO:0051092 GO:0070423 GO:0038095 GO:1902236 GO:0035666 GO:0050852 GO:0000151 GO:0042975 GO:0046982 GO:0034134 GO:0009615 GO:0000187 GO:0038123 GO:0016301 GO:0005634 GO:1990450 |
| tr|A0A087X1E4|A0A087X1E4_ HUMAN | GO:0006928 GO:0048365 GO:0001726 GO:0030032 GO:0070273 GO:0007264 GO:0034315 GO:0005938 GO:0030742 GO:0019904 GO:0032588 GO:0005525 GO:0031529 GO:0005886 |
| tr|A0A087X1G1|A0A087X1G1_ HUMAN | GO:0008137 GO:0005747 GO:0006120 GO:0032981 |
| tr|A0A087X1N8|A0A087X1N8_ HUMAN | GO:0005829 GO:0004867 GO:0043234 GO:0071470 GO:0010951 GO:0070062 GO:0005615 GO:0002020 GO:0007605 GO:0005634 |
| tr|A0A087X1Q3|A0A087X1Q3_ HUMAN | GO:0004843 GO:0050691 GO:0030334 GO:0071108 GO:0090315 GO:0071586 GO:0010955 GO:1900027 GO:0043547 GO:0034260 GO:0070536 GO:0042127 GO:0042981 GO:0031064 GO:0005515 GO:0005730 GO:0007093 GO:0006511 GO:1900246 GO:1900245 GO:0005789 |
| tr|A0A087X1U6|A0A087X1U6_ HUMAN | GO:0044822 GO:0005737 GO:0030496 |
| tr|A0A087X208|A0A087X208_ HUMAN | GO:0009986 GO:0007213 GO:0002162 GO:0016021 GO:0035374 GO:0032092 GO:0030548 GO:0043525 GO:0045162 GO:0006027 GO:0007009 GO:0071340 GO:0044295 GO:0043547 GO:0005200 GO:1903407 GO:0005615 GO:0055117 GO:0006024 GO:0030054 GO:0043395 GO:2000541 GO:0086036 GO:0044325 GO:0007603 GO:0045944 GO:0045213 GO:0042383 GO:0001523 GO:0005796 GO:0043202 GO:0070062 GO:0042030 GO:0051290 GO:0051491 GO:0005975 GO:1902667 GO:0030198 GO:0036122 GO:0045202 GO:0061098 GO:1903277 GO:0050431 GO:0030204 GO:0045887 GO:0005605 GO:0005509 GO:0033691 GO:0006775 GO:0043236 GO:0070507 GO:0099601 |
| tr|A0A087X256|A0A087X256_ HUMAN | GO:0005769 GO:0005654 GO:0016197 GO:0007032 GO:0071203 GO:0031083 GO:0015031 |
| tr|A0A090N7T9|A0A090N7T9_ HUMAN | GO:0005737 GO:0031965 GO:0005515 GO:0006887 GO:0016805 GO:0006508 |
| tr|A0A090N7V5|A0A090N7V5_ HUMAN | GO:0001836 GO:0005829 GO:0006750 GO:0006805 GO:0070062 GO:0003839 GO:0042803 GO:0044281 GO:1901687 |
| tr|A0A090N7W4|A0A090N7W4_ HUMAN | GO:0030424 GO:0043525 GO:0021766 GO:0001963 GO:0019233 GO:0019901 GO:0035249 GO:0008045 GO:0090314 GO:0071156 GO:2000273 GO:0046777 GO:0030426 GO:0060059 GO:0048488 GO:0046875 GO:0035173 GO:0007416 GO:0045892 GO:0021634 GO:0031914 GO:0030866 GO:0008283 GO:0004693 GO:0033136 GO:2000251 GO:0018107 GO:0005176 GO:0031397 GO:0035418 GO:0048935 GO:0060079 GO:0030517 GO:0045860 GO:0014044 GO:0010842 GO:0030054 GO:0007596 GO:0022038 GO:0001764 GO:0014069 GO:0030334 GO:0070509 GO:0008092 GO:0021697 GO:0021819 GO:0098793 GO:0043524 GO:0048709 GO:0045956 GO:0061001 GO:0043204 GO:0046826 GO:0045861 GO:0016079 GO:0050321 GO:0005829 GO:0005730 GO:0051301 GO:0030027 GO:0002039 GO:0007519 GO:0048148 GO:0043113 GO:0005524 GO:0016533 GO:0021954 GO:0032801 GO:0031594 GO:0032092 GO:0045211 GO:0048511 GO:0030175 GO:1903421 GO:0043125 GO:0007160 GO:0030425 GO:0016572 GO:0005856 GO:0008542 GO:0030549 |
| tr|A0A090N7Y2|A0A090N7Y2_ HUMAN | GO:0006810 GO:0000786 GO:0043190 GO:0003677 GO:0005524 GO:0016887 GO:0005215 GO:0008152 GO:0046982 GO:0005740 GO:0005634 |
| tr|A0A090N8Q3|A0A090N8Q3_ HUMAN | GO:0043232 GO:0030433 GO:0005654 GO:0005737 GO:0016020 |
| tr|A0A090N8Y2|A0A090N8Y2_ HUMAN | GO:0003756 GO:0009306 GO:0034976 GO:1903334 GO:0034663 GO:0045454 GO:0009986 GO:0042470 GO:0044822 GO:0005788 GO:0005515 GO:0061077 GO:0005790 |
| tr|A0A096LPH6|A0A096LPH6_ HUMAN | GO:0003690 GO:0008270 GO:0016607 GO:0008380 GO:0006355 GO:0044822 GO:0000166 GO:0005737 |
| tr|A0A096LPI6|A0A096LPI6_ HUMAN | GO:0005739 |
| tr|A0A096LPJ3|A0A096LPJ3_ HUMAN | GO:0005095 GO:0005654 GO:0005515 GO:0008180 GO:0006283 GO:0070911 GO:0010388 GO:0005737 GO:0007254 GO:0000188 GO:0000715 GO:0034260 GO:0007049 |
| tr|A0A097PIC4|A0A097PIC4_ HUMAN | GO:0005925 GO:0050699 GO:0007411 GO:0030027 GO:0008154 GO:0030175 GO:0005829 GO:0050852 GO:0001843 GO:0001725 GO:0005522 GO:0005886 GO:0017124 |
| tr|A0A0A0MR36|A0A0A0MR36_ HUMAN | GO:1902109 GO:0055037 GO:0043197 GO:0030659 GO:0006833 GO:0017137 GO:0044822 GO:0003988 GO:0005743 GO:0015031 GO:1901029 GO:0035255 GO:0003779 GO:0000146 GO:0003091 GO:0016192 GO:0071456 GO:0006631 GO:0006695 GO:0043025 GO:0032880 GO:0045179 GO:0005903 GO:0055085 GO:0048471 GO:0070062 GO:0032439 GO:0005516 GO:0005524 GO:0016459 |
| tr|A0A0A0MR39|A0A0A0MR39_ HUMAN | GO:0003677 GO:0044822 GO:0005515 GO:0000166 GO:0005654 GO:0005737 GO:0014902 GO:0030182 GO:0006351 |
| tr|A0A0A0MRM8|A0A0A0MRM8_ HUMAN | GO:0016461 GO:0045334 GO:0030048 GO:0048167 GO:0007416 GO:0016358 GO:0005829 GO:0007605 GO:0042491 GO:0030665 GO:0060001 GO:0030424 GO:0045944 GO:0014047 GO:0006605 GO:0007626 GO:0070062 GO:0048471 GO:0030330 GO:0005794 GO:0016591 GO:0006897 GO:0031941 GO:0005524 GO:0005765 GO:0042472 GO:0051046 GO:0031965 GO:0005516 GO:0005902 GO:0051015 GO:0045177 GO:0061024 GO:0042493 GO:0043531 GO:0043025 GO:0005905 GO:0032587 GO:0005938 GO:0071257 |
| tr|A0A0A0MRT6|A0A0A0MRT6_ HUMAN | GO:0048010 GO:0072673 GO:0030296 GO:0098794 GO:0043005 GO:0030027 GO:0008154 GO:0035855 GO:0001756 GO:0030175 GO:0031209 GO:0005829 GO:0008285 GO:0008092 GO:0007264 GO:0018108 GO:0038096 GO:0006928 GO:0043232 GO:0032403 GO:0070062 GO:0005783 GO:0005886 GO:0008134 GO:0045087 |
| tr|A0A0A0MRW6|A0A0A0MRW6_ HUMAN | GO:0005739 GO:0000794 GO:0006364 GO:0034456 GO:0044822 GO:0006409 GO:0005515 GO:0032545 GO:0032040 |
| tr|A0A0A0MS51|A0A0A0MS51_ HUMAN | GO:0097017 GO:0005925 GO:0060271 GO:0045159 GO:0030478 GO:1903906 GO:0016528 GO:0051593 GO:0014003 GO:0002102 GO:0072562 GO:0030027 GO:0005829 GO:0006911 GO:1903903 GO:1902174 GO:0045471 GO:1903689 GO:0048015 GO:0006921 GO:0051016 GO:0007568 GO:0005886 GO:0043209 GO:0042246 GO:0070062 GO:0048471 GO:1990000 GO:0031648 GO:0019904 GO:0042989 GO:0071801 GO:0051127 GO:0022617 GO:0090527 GO:0097284 GO:0046597 GO:1903923 GO:0014891 GO:1903909 GO:0005509 GO:0005634 GO:0051014 GO:2001269 GO:0071276 GO:0001726 GO:0003779 |
| tr|A0A0A0MSG2|A0A0A0MSG2_ HUMAN | GO:0005925 GO:0045893 GO:0005654 GO:0000122 GO:0060347 GO:0008270 GO:0001649 GO:0043066 GO:0009725 GO:0055015 GO:0015629 GO:0044255 GO:0055014 GO:0030521 GO:0031430 GO:0003713 GO:0042802 GO:0044281 GO:0030018 GO:0008134 GO:0050681 |
| tr|A0A0A0MSJ2|A0A0A0MSJ2_ HUMAN | GO:0051497 GO:0005802 GO:0051270 GO:0008360 GO:0005547 GO:0051056 GO:0030037 GO:0001921 GO:0005829 GO:0046872 GO:0005096 GO:0031410 GO:0031702 GO:0032580 GO:0043547 GO:0005886 GO:0051491 |
| tr|A0A0A0MSS8|A0A0A0MSS8_ HUMAN | GO:0047787 GO:0006805 GO:0034614 GO:0045703 GO:0008284 GO:0007584 GO:0047035 GO:0045550 GO:0047115 GO:0004032 GO:0047006 GO:0004745 GO:0047042 GO:2000379 GO:0019371 GO:0005829 GO:0047086 GO:0032052 GO:0047045 GO:0051260 GO:0001758 GO:0042632 GO:1900053 GO:0044597 GO:0007603 GO:0036131 GO:0000060 GO:0044598 GO:0070293 GO:0070062 GO:0071277 GO:2000353 GO:2000224 GO:0030216 GO:0008206 GO:0015721 GO:0005515 GO:0055114 GO:0046683 GO:0030299 GO:0009267 GO:0016488 GO:0051897 GO:0018636 GO:0007186 GO:0008584 GO:0071395 GO:0071384 GO:0035410 GO:0044259 GO:0042574 GO:0005634 GO:0016655 GO:0006775 GO:0047020 GO:0071276 GO:0042448 GO:0048385 GO:0047017 GO:0047718 GO:0071799 |
| tr|A0A0A0MSW4|A0A0A0MSW4_ HUMAN | GO:0008526 GO:0000139 GO:0046474 GO:0005543 GO:0070062 GO:0015914 GO:0044281 GO:0005789 GO:0001701 |
| tr|A0A0A0MT30|A0A0A0MT30_ HUMAN | GO:0007186 GO:0042448 GO:0047718 GO:0047115 GO:0030299 GO:0047006 GO:0005515 GO:0016655 GO:0006693 GO:0071395 GO:0046683 GO:0006775 GO:0015721 GO:0005829 GO:0008206 GO:0008284 GO:0071799 GO:0044597 GO:0018636 GO:0006805 GO:0047086 GO:0042574 GO:0032052 GO:0051897 GO:0047026 GO:0007603 GO:0055114 GO:0030855 GO:0070062 GO:0051260 GO:0004032 GO:0047042 GO:0044598 GO:0042632 |
| tr|A0A0A0MTB8|A0A0A0MTB8_ HUMAN | GO:0050896 GO:0005730 GO:0006364 GO:0007601 GO:0044822 GO:0030516 GO:0032040 GO:0001895 |
| tr|A0A0A0MTC5|A0A0A0MTC5_ HUMAN | GO:0006810 GO:0043198 GO:0051019 GO:0030544 GO:1990124 GO:0044822 GO:0031965 GO:0010468 GO:0019894 GO:0043022 GO:0005730 GO:0005874 GO:0010494 GO:0043025 GO:0032956 GO:0048592 GO:0030424 GO:0003725 GO:0005783 GO:1900454 GO:0051965 GO:0051489 GO:0034599 GO:0061003 |
| tr|A0A0A0MTJ5|A0A0A0MTJ5_ HUMAN | GO:1904929 GO:0060071 GO:0043235 GO:0018108 GO:0017147 GO:0007169 GO:0005887 GO:0005524 GO:0004714 GO:0005737 |
| tr|A0A0A0MTS2|A0A0A0MTS2_ HUMAN | GO:0001525 GO:0006367 GO:0043005 GO:0006094 GO:0005654 GO:0007599 GO:0004347 GO:0008083 GO:0007611 GO:0005125 GO:0006959 GO:0051156 GO:0005615 GO:0046185 GO:0005829 GO:0043154 GO:0031625 GO:0019242 GO:0048029 GO:0016866 GO:0070062 GO:0043524 GO:0061621 GO:0005886 |
| tr|A0A0A1HAN9|A0A0A1HAN9_ HUMAN | GO:0030100 GO:0045121 GO:0055037 GO:2000286 GO:0051036 GO:0045335 GO:0098559 GO:0007032 GO:0015629 GO:0042470 GO:0005525 GO:0015031 GO:0045921 GO:0005829 GO:0030425 GO:0001726 GO:0048661 GO:0007264 GO:0043025 GO:0051021 GO:0007596 GO:0043234 GO:2000300 GO:0008021 GO:0036465 GO:0043195 GO:0045022 GO:0048169 GO:0048471 GO:0070062 GO:0019003 GO:0005811 GO:0030136 GO:0003924 GO:0005886 GO:0014911 GO:0051489 GO:0042589 |
| tr|A0A0A1HAV9|A0A0A1HAV9_ HUMAN | GO:0030100 GO:0055037 GO:0019882 GO:0006913 GO:0008565 GO:0001944 GO:0005525 GO:0032593 GO:0006886 GO:0005829 GO:0061024 GO:0035255 GO:0001671 GO:0051117 GO:0032482 GO:0032781 GO:0048471 GO:0070062 GO:0019003 GO:0003924 GO:0005886 GO:0019905 |
| tr|A0A0A6YYA0|A0A0A6YYA0_ HUMAN | GO:0032755 GO:0004871 GO:0006954 GO:0032729 GO:0035669 GO:0016021 GO:0034138 GO:0043687 GO:0034145 GO:0030127 GO:0031902 GO:0005543 GO:2000494 GO:0015031 GO:0070671 GO:0043123 GO:0033116 GO:0006888 GO:0071222 GO:0005886 GO:0097191 GO:0070062 GO:0071650 GO:0005515 GO:0035666 GO:0034144 GO:0071651 GO:0031901 GO:0097296 GO:0018279 GO:0061024 GO:0030126 GO:0005789 |
| tr|A0A0A6YYH1|A0A0A6YYH1_ HUMAN | GO:0048490 GO:0030336 GO:0005802 GO:0030659 GO:0030027 GO:0005515 GO:0033058 GO:0030123 GO:0008565 GO:0051126 GO:0006886 GO:2000393 GO:0030424 |
| tr|A0A0B4J1S4|A0A0B4J1S4_ HUMAN | GO:0051084 GO:0098869 GO:0035092 GO:0070062 GO:0005788 GO:0005515 GO:0008379 GO:0008430 |
| tr|A0A0B4J211|A0A0B4J211_ HUMAN | GO:0005654 GO:0042393 GO:0031936 GO:0045944 GO:0070062 GO:0016887 GO:0003682 GO:0005524 |
| tr|A0A0C4DFM1|A0A0C4DFM1_ HUMAN | GO:0016021 |
| tr|A0A0C4DFN3|A0A0C4DFN3_ HUMAN | GO:0005654 GO:0030168 GO:0043196 GO:0004622 GO:0046474 GO:0005789 GO:0060292 GO:0019433 GO:0047372 GO:0005829 GO:0006633 GO:0008289 GO:0019369 GO:0051930 GO:0042803 GO:0050727 GO:2000124 GO:0005886 GO:0030516 GO:0019898 GO:0045202 GO:0036155 |
| tr|A0A0C4DFR6|A0A0C4DFR6_ HUMAN | GO:0000278 GO:0005654 GO:0051028 GO:0012507 GO:0000139 GO:0005515 GO:0000236 GO:0061700 GO:0002474 GO:0005789 GO:0006886 GO:0005829 GO:0032008 GO:0019886 GO:0000776 GO:0007264 GO:0048208 GO:0000090 GO:0070062 GO:0031080 GO:0043687 GO:0018279 |
| tr|A0A0C4DGB5|A0A0C4DGB5_ HUMAN | GO:0007343 GO:0005654 GO:0006362 GO:0003899 GO:0045815 GO:0016020 GO:0010951 GO:0031100 GO:0071157 GO:0044822 GO:0007568 GO:0006363 GO:0000120 GO:0045445 GO:0005829 GO:0007520 GO:0007169 GO:0009303 GO:0007420 GO:0005694 GO:0045814 GO:0002020 GO:0005783 GO:0001889 GO:2000675 GO:0006361 GO:0005736 GO:0010859 GO:0005739 |
| tr|A0A0C4DGH2|A0A0C4DGH2_ HUMAN | GO:0007265 GO:0070062 GO:0006913 GO:0005925 GO:1901214 GO:0030335 GO:0005515 GO:0001649 GO:0003924 GO:0008152 GO:0006886 GO:0005783 GO:0005525 GO:0005886 |
| tr|A0A0C4DGI9|A0A0C4DGI9_ HUMAN | GO:0070869 GO:0005654 GO:0008270 GO:0000183 GO:0051567 GO:0001164 GO:0005677 GO:0070933 GO:0005737 GO:0005730 GO:0006306 GO:0016922 GO:0033553 GO:0003723 GO:0070577 GO:0034770 |
| tr|A0A0C4DGQ5|A0A0C4DGQ5_ HUMAN | GO:0022617 GO:0008284 GO:0005886 GO:0004198 GO:0005509 GO:0006508 GO:0005829 GO:0046982 GO:0070062 |
| tr|A0A0C4DGS5|A0A0C4DGS5_ HUMAN | GO:0048208 GO:0061676 GO:0030134 GO:0051289 GO:0043687 GO:0090161 GO:0032091 GO:0043234 GO:0050772 GO:0090166 GO:0000088 GO:0033116 GO:0072686 GO:0000922 GO:0051645 GO:0032580 GO:0010507 GO:0005801 GO:0090306 GO:0019901 GO:0051297 GO:0005874 GO:0090307 GO:0000137 GO:0019905 GO:0008356 GO:0060050 GO:0007020 GO:0018279 GO:0005634 GO:0043025 GO:0008017 |
| tr|A0A0C4DGV4|A0A0C4DGV4_ HUMAN | GO:0009615 GO:0006367 GO:0008286 GO:0016236 GO:0019079 GO:0005765 GO:0005829 GO:0071986 GO:0007050 GO:0032008 GO:0043154 GO:0008361 GO:0061462 GO:0033554 GO:0043547 GO:0071230 GO:0005085 GO:0032947 |
| tr|A0A0C4DGV5|A0A0C4DGV5_ HUMAN | GO:0046872 GO:0008380 GO:0006355 GO:0044822 GO:0005515 GO:0005654 GO:0003700 |
| tr|A0A0C4DGX4|A0A0C4DGX4_ HUMAN | GO:0005654 GO:0007219 GO:0002223 GO:0038123 GO:0038095 GO:0034142 GO:0006513 GO:0034162 GO:0034134 GO:0038061 GO:1990452 GO:0034166 GO:0035666 GO:0005829 GO:0097193 GO:0019005 GO:0008285 GO:0007050 GO:0033209 GO:0000080 GO:0034146 GO:0031625 GO:0051439 GO:0050852 GO:0009887 GO:0000082 GO:0031146 GO:0034138 GO:0016032 GO:0031145 GO:0000084 GO:0002755 GO:0000086 GO:0038124 GO:0061630 GO:0051403 GO:0051437 GO:0007623 |
| tr|A0A0C4DGX5|A0A0C4DGX5_ HUMAN | GO:0016023 GO:0000186 GO:0006913 GO:0003382 GO:0005525 GO:0005765 GO:0006886 GO:0071986 GO:0032008 GO:0060627 GO:0008284 GO:0007264 GO:0031268 GO:0016049 GO:0070062 GO:0043547 GO:0031260 GO:0003924 GO:0010634 GO:0031489 GO:0071230 GO:0005085 GO:0032947 |
| tr|A0A0D9SEI3|A0A0D9SEI3_ HUMAN | GO:0006468 GO:0007067 GO:0051726 GO:0006915 GO:0050684 GO:0001558 GO:0006355 GO:0051301 GO:0005524 GO:0004693 GO:0044822 GO:0005515 GO:0008283 GO:0005737 GO:0005634 |
| tr|A0A0D9SF53|A0A0D9SF53_ HUMAN | GO:0010494 GO:0043024 GO:0045948 GO:0008190 GO:0004003 GO:0032728 GO:0007059 GO:0004004 GO:0043273 GO:0071243 GO:1900087 GO:0008134 GO:0043280 GO:0032508 GO:0008143 GO:2001243 GO:0042256 GO:0016055 GO:0017148 GO:0071470 GO:0035613 GO:1903608 GO:0045944 GO:0045087 GO:0031333 GO:0070062 GO:0030307 GO:0048027 GO:0003677 GO:0005524 GO:0043154 GO:0008625 GO:0010501 GO:0005741 GO:0009615 GO:0003924 GO:0071651 GO:0030308 GO:0045070 GO:0022627 GO:0005852 GO:0016607 GO:0034063 |
| tr|A0A0D9SFB1|A0A0D9SFB1_ HUMAN | GO:0007411 GO:0030117 GO:0007605 GO:0002031 GO:0008022 GO:0019901 GO:0043196 GO:0007032 GO:0044822 GO:0042584 GO:0048013 GO:0050998 GO:0005525 GO:0043209 GO:0046983 GO:0005794 GO:0005874 GO:1903423 GO:0042802 GO:0031749 GO:1901998 GO:0008021 GO:0032403 GO:0070062 GO:0051932 GO:0003924 GO:0072583 GO:0051262 GO:0005886 GO:0045920 GO:0001917 GO:0008344 |
| tr|A0A0D9SFK2|A0A0D9SFK2_ HUMAN | GO:0005654 GO:0008094 GO:0005802 GO:0000139 GO:0005793 GO:0043066 GO:0044822 GO:0048194 GO:0043531 GO:0006259 GO:0050714 GO:0003774 GO:0003677 GO:0005903 GO:0051015 GO:0005524 GO:0016477 GO:0042641 GO:0090164 GO:0031032 GO:0016459 |
| tr|A0A0D9SGC1|A0A0D9SGC1_ HUMAN | GO:0016461 GO:0045334 GO:0030048 GO:0048167 GO:0007416 GO:0016358 GO:0005829 GO:0007605 GO:0042491 GO:0030665 GO:0060001 GO:0030424 GO:0045944 GO:0014047 GO:0006605 GO:0007626 GO:0070062 GO:0048471 GO:0030330 GO:0005794 GO:0016591 GO:0006897 GO:0031941 GO:0005524 GO:0005765 GO:0042472 GO:0051046 GO:0031965 GO:0005516 GO:0005902 GO:0051015 GO:0045177 GO:0061024 GO:0042493 GO:0043531 GO:0043025 GO:0005905 GO:0032587 GO:0005938 GO:0071257 |
| tr|A0A0D9SGE8|A0A0D9SGE8_ HUMAN | GO:0042393 GO:0008270 GO:0005730 GO:0097110 GO:0043021 GO:0006355 GO:0003677 GO:0044822 GO:0051219 GO:0015631 GO:0000777 GO:0005654 GO:0042826 |
| tr|A0A0F7KYT8|A0A0F7KYT8_ HUMAN | GO:0003730 GO:0002151 GO:0017148 GO:0030424 GO:0043034 GO:0005730 GO:0006915 GO:0007517 GO:0035770 GO:0043197 GO:0030154 GO:0005515 GO:0005844 GO:0048471 GO:0016020 |
| tr|A0A0F7NGI8|A0A0F7NGI8_ HUMAN | GO:0005829 GO:0006357 GO:0005856 GO:0045087 GO:0003677 GO:0003725 GO:0032481 GO:0042803 GO:0045892 GO:0005634 GO:0005886 |
| tr|A0A0G2JMZ8|A0A0G2JMZ8_ HUMAN | GO:0005829 GO:0008119 GO:0046498 GO:0016021 GO:0006921 GO:0030139 GO:0032259 GO:0009986 GO:0006461 GO:0016327 GO:0005923 GO:0070830 GO:0019904 GO:0016324 GO:0046500 |
| tr|A0A0G2JNZ2|A0A0G2JNZ2_ HUMAN | GO:0005654 GO:0030027 GO:0005515 GO:0035748 GO:0060561 GO:0045930 GO:0050918 GO:0042734 GO:0005913 GO:0034750 GO:0008283 GO:0016323 GO:0016080 GO:0001921 GO:0045211 GO:0060603 GO:0042060 GO:0008105 GO:0035089 GO:0090630 GO:0043065 GO:0001843 GO:0016337 GO:0070062 GO:0016032 GO:0043615 GO:0060088 GO:0071896 GO:0021747 GO:0048488 |
| tr|A0A0J9YXX5|A0A0J9YXX5_ HUMAN | GO:0030054 GO:0005739 GO:0006915 GO:0042802 GO:0008380 GO:0006355 GO:0030529 GO:0003677 GO:0044822 GO:0000166 GO:0019907 GO:0006397 GO:0005654 GO:0005794 |
| tr|A0A0K0K1J1|A0A0K0K1J1_ HUMAN | GO:0001775 GO:0004869 GO:0009636 GO:0005771 GO:0006915 GO:0005764 GO:0008284 GO:0005783 GO:0007420 GO:0006952 GO:0031667 GO:0005615 GO:0005604 GO:0001540 GO:0043292 GO:0001654 GO:0070301 GO:0030424 GO:0002020 GO:0001666 GO:0045740 GO:0010716 GO:0043067 GO:0070062 GO:0042747 GO:0048471 GO:0042802 GO:0032355 GO:0010951 GO:0007566 GO:0060548 GO:0031965 GO:0060009 GO:0060311 GO:0042493 GO:0007431 GO:0043206 GO:0009743 GO:0043025 GO:0010711 GO:0048678 GO:0060313 |
| tr|A0A0K0K1K4|A0A0K0K1K4_ HUMAN | GO:0048011 GO:0051436 GO:0002479 GO:0090263 GO:0007411 GO:0048010 GO:0000186 GO:0019773 GO:0006977 GO:0005829 GO:0002223 GO:0051437 GO:0005654 GO:0006595 GO:0031145 GO:0038061 GO:0007265 GO:0090090 GO:0033209 GO:0070062 GO:0016032 GO:0006521 GO:0042802 GO:0043488 GO:0000084 GO:0007173 GO:0038095 GO:0050852 GO:0004298 GO:0000090 GO:0043066 GO:0008543 GO:0000209 GO:0008286 |
| tr|A0A0K0K1K7|A0A0K0K1K7_ HUMAN | GO:0005975 GO:0048029 GO:0005515 GO:0005829 GO:0070062 GO:0009051 GO:0017057 |
| tr|A0A0R4J2E8|A0A0R4J2E8_ HUMAN | GO:0016363 GO:0003281 GO:0010608 GO:0008270 GO:0044822 GO:0005515 GO:0000166 GO:0003170 GO:0005198 GO:0005654 GO:0005737 GO:0005637 |
| tr|A0A0R4J2G3|A0A0R4J2G3_ HUMAN | GO:0017171 GO:0006805 GO:0042301 GO:0016042 GO:0006470 GO:0016021 GO:0060395 GO:0005783 GO:0052689 |
| tr|A0A0S2Q0B1|A0A0S2Q0B1_ HUMAN | GO:0043044 GO:0060674 GO:0005654 GO:0000122 GO:0003408 GO:0071565 GO:0031491 GO:0055007 GO:0048096 GO:0016514 GO:0006337 GO:0071564 GO:0042921 GO:0006344 GO:0030521 GO:0030900 GO:0003713 GO:0016922 GO:0003677 GO:0003205 GO:0001843 GO:1901998 GO:0030520 GO:0000790 GO:0042766 |
| tr|A0A0S2Z392|A0A0S2Z392_ HUMAN | GO:0048011 GO:0043198 GO:0031694 GO:0007213 GO:0016324 GO:0005901 GO:0004703 GO:0042542 GO:0007507 GO:0042699 GO:0047696 GO:0060048 GO:0019079 GO:0005829 GO:0014070 GO:0016323 GO:0072372 GO:0030424 GO:0007202 GO:0018107 GO:0045087 GO:0007568 GO:0007217 GO:0006886 GO:0002031 GO:0007173 GO:0005524 GO:0031755 GO:0018105 GO:0045988 GO:0033605 GO:0046718 GO:0008543 GO:0003108 GO:0043197 |
| tr|A0A0S2Z3C5|A0A0S2Z3C5_ HUMAN | GO:0043524 GO:0030672 GO:0042803 GO:0010288 GO:0016021 GO:0001701 GO:0008284 GO:0001541 GO:2001240 GO:0042542 GO:0071230 GO:0090201 GO:0051602 GO:2001244 GO:0034097 GO:0051434 GO:1902230 GO:0021987 GO:0005829 GO:0051881 GO:0009566 GO:0070584 GO:0005759 GO:0007283 GO:0035872 GO:0030054 GO:0090005 GO:0043027 GO:0060154 GO:0071839 GO:2000811 GO:1900118 GO:0001666 GO:0071480 GO:0040007 GO:0045087 GO:0007568 GO:0097136 GO:0097371 GO:0010507 GO:0000910 GO:0006897 GO:0071312 GO:0097284 GO:0019050 GO:0043154 GO:0007281 GO:0019901 GO:0046982 GO:0046902 GO:0031965 GO:0005741 GO:0043434 GO:0002931 GO:0008584 GO:0046898 GO:0005743 GO:0005813 GO:0005730 GO:0007093 |
| tr|A0A0S2Z3D0|A0A0S2Z3D0_ HUMAN | GO:0008270 GO:0015701 GO:0016323 GO:0031528 GO:0061418 GO:0005730 GO:0006730 GO:0004089 GO:0002009 GO:0042493 GO:0016021 GO:0046903 GO:0033574 |
| tr|A0A0S2Z3G9|A0A0S2Z3G9_ HUMAN | GO:0005925 GO:0030050 GO:0042803 GO:1903506 GO:1902396 GO:0043005 GO:0047485 GO:0001882 GO:0035357 GO:0032417 GO:0042974 GO:0005903 GO:0048549 GO:0043234 GO:0005829 GO:0015031 GO:0005178 GO:1901224 GO:0005615 GO:0030168 GO:0030863 GO:0001666 GO:0044325 GO:0070830 GO:0042981 GO:0044822 GO:0070062 GO:0002576 GO:0005911 GO:0048471 GO:0048384 GO:0001725 GO:0030335 GO:0030374 GO:0031093 GO:0051015 GO:0030529 GO:0051017 GO:0005509 GO:0030018 GO:0000977 GO:0005634 GO:0051271 GO:0031490 GO:0031143 GO:1900025 |
| tr|A0A0S2Z3H6|A0A0S2Z3H6_ HUMAN | GO:0033081 GO:0009897 GO:0005887 GO:0005515 |
| tr|A0A0S2Z3L0|A0A0S2Z3L0_ HUMAN | GO:0003995 GO:0006810 GO:0000062 GO:0017133 GO:0052890 GO:0033539 GO:0070062 GO:0009055 GO:0022904 GO:0005515 GO:0050660 GO:0055088 |
| tr|A0A0S2Z3L2|A0A0S2Z3L2_ HUMAN | GO:0098909 GO:0005887 GO:0008022 GO:0034976 GO:0097470 GO:0006996 GO:0008152 GO:1903515 GO:0007596 GO:0031095 GO:0010882 GO:0002026 GO:0032470 GO:0086036 GO:0008544 GO:0010460 GO:0031234 GO:0014801 GO:0033292 GO:0006984 GO:1990036 GO:0048471 GO:1903779 GO:0045822 GO:0086039 GO:0005524 GO:0014883 GO:1903233 GO:0007155 GO:0055119 GO:0014898 GO:0090534 GO:0043434 GO:0005509 GO:0070588 GO:0019899 GO:0014704 GO:0031775 GO:0033017 GO:0044548 GO:0034599 GO:0012506 |
| tr|A0A0S2Z3S5|A0A0S2Z3S5_ HUMAN | GO:0071514 GO:0007190 GO:0043025 GO:0001894 GO:0045672 GO:0006306 GO:0001934 GO:0005525 GO:0071107 GO:0031698 GO:0006357 GO:0051430 GO:0050796 GO:0045776 GO:0004016 GO:0001726 GO:0006112 GO:0031681 GO:0048701 GO:0047391 GO:0043014 GO:0046872 GO:0007565 GO:0031683 GO:0055074 GO:0001965 GO:0040032 GO:0007608 GO:0008284 GO:0071380 GO:0071880 GO:0030054 GO:0035814 GO:0007191 GO:0071377 GO:0006833 GO:0050890 GO:0035255 GO:0070062 GO:0005159 GO:0004871 GO:0060789 GO:0005768 GO:0005634 GO:0045121 GO:0031852 GO:0001958 GO:0032588 GO:0005829 GO:0019904 GO:0043547 GO:0003091 GO:0003924 GO:0043950 GO:0051216 GO:0055085 GO:2000828 GO:0046907 GO:0035116 GO:0005834 GO:0010765 GO:0048471 GO:0031748 GO:0031224 GO:0070527 GO:0030425 GO:0042383 GO:0042493 GO:0040015 GO:0045669 GO:0008021 GO:0071870 |
| tr|A0A0S2Z3W7|A0A0S2Z3W7_ HUMAN | GO:0006193 GO:0005829 GO:0051276 GO:0046872 GO:0036222 GO:0035870 GO:0000166 GO:0009204 GO:0036220 |
| tr|A0A0S2Z3X8|A0A0S2Z3X8_ HUMAN | GO:0043005 GO:0050771 GO:0005093 GO:0051056 GO:0017137 GO:0051592 GO:0016491 GO:0043209 GO:0005794 GO:0005829 GO:0005096 GO:0043234 GO:0032482 GO:0030496 GO:0055114 GO:0043547 GO:0090315 |
| tr|A0A0S2Z3Y1|A0A0S2Z3Y1_ HUMAN | GO:0005044 GO:0072562 GO:0005515 GO:0007155 GO:0007165 GO:0006898 GO:0016020 GO:0005578 GO:0070062 GO:0006968 |
| tr|A0A0S2Z3Y7|A0A0S2Z3Y7_ HUMAN | GO:0031526 GO:0042803 GO:0002064 GO:0018715 GO:0008755 GO:0008376 GO:0033580 GO:0060046 GO:0016021 GO:0035496 GO:0003831 GO:0046921 GO:0051270 GO:0048754 GO:0047276 GO:0008375 GO:0030057 GO:0030112 GO:0005534 GO:0061623 GO:0005829 GO:0016323 GO:0018717 GO:0005615 GO:0030175 GO:0042283 GO:0052824 GO:0060055 GO:0046920 GO:0030145 GO:0052638 GO:0052639 GO:0007339 GO:0004576 GO:0050900 GO:0048487 GO:0002526 GO:0052757 GO:0052640 GO:0003945 GO:0004583 GO:0070062 GO:0003980 GO:0016759 GO:0032580 GO:0008270 GO:0005536 GO:0006258 GO:0046316 GO:0009897 GO:0004376 GO:0030198 GO:0033499 GO:0045136 GO:0000026 GO:0008285 GO:0019901 GO:0018716 GO:0005989 GO:0007155 GO:0018718 GO:0001962 GO:0000138 GO:0080062 GO:0019187 GO:0042281 GO:0004461 GO:0008108 GO:0060058 GO:0033556 GO:0007341 GO:0060054 GO:0006487 GO:0008921 GO:0045140 GO:0031278 GO:0043014 GO:0000033 GO:0052641 |
| tr|A0A0S2Z404|A0A0S2Z404_ HUMAN | GO:0042393 GO:0031965 GO:0031492 GO:0051225 GO:0000790 GO:0000794 GO:0007059 GO:0007052 GO:0043547 GO:0005087 GO:0000082 GO:0016032 GO:0005654 GO:0005737 GO:0007088 |
| tr|A0A0S2Z410|A0A0S2Z410_ HUMAN | GO:0003857 GO:0051287 GO:0051289 GO:0044822 GO:0047015 GO:0009083 GO:0090646 GO:0005743 GO:0004303 GO:0007569 GO:0042645 GO:0001540 GO:0030283 GO:0018454 GO:0008709 GO:0042802 GO:0006629 GO:0005496 GO:0030331 GO:0070900 GO:0055114 GO:0005783 GO:0033327 GO:0005886 |
| tr|A0A0S2Z422|A0A0S2Z422_ HUMAN | GO:0000062 GO:0031966 GO:0052890 GO:0051260 GO:0033539 GO:0009055 GO:0006552 GO:0034641 GO:0008470 GO:0050660 GO:0005759 GO:0055088 GO:0005654 |
| tr|A0A0S2Z433|A0A0S2Z433_ HUMAN | GO:0019933 GO:0008137 GO:0006120 GO:0032981 GO:0007420 GO:0005747 GO:0001932 GO:0051591 GO:0072593 GO:0048146 |
| tr|A0A0S2Z471|A0A0S2Z471_ HUMAN | GO:0004111 GO:0005829 GO:0021762 GO:0005739 GO:0030644 GO:0031625 GO:0070062 GO:0043209 GO:0005615 GO:0006603 GO:0005524 GO:0016310 GO:0006595 GO:0006600 GO:0005886 |
| tr|A0A0S2Z487|A0A0S2Z487_ HUMAN | GO:0004871 GO:0005916 GO:0030056 GO:0071681 GO:0005925 GO:0042803 GO:0086073 GO:0045294 GO:0090263 GO:0043588 GO:0048010 GO:0016327 GO:0051091 GO:0030057 GO:0005829 GO:0090002 GO:0015629 GO:0071665 GO:0005199 GO:0016477 GO:0005882 GO:0016342 GO:0003713 GO:0042127 GO:0042307 GO:0032993 GO:0071603 GO:0002159 GO:0050982 GO:0070062 GO:0034333 GO:0098911 GO:0019901 GO:0086083 GO:0019903 GO:0007016 GO:0045296 GO:0086091 GO:0009898 GO:0030018 GO:0051291 GO:0005915 GO:0016328 |
| tr|A0A0S2Z489|A0A0S2Z489_ HUMAN | GO:0048011 GO:0051436 GO:0031595 GO:0002479 GO:0090263 GO:0007411 GO:0048010 GO:0000186 GO:0008541 GO:0006977 GO:0005829 GO:0002223 GO:0051437 GO:0005654 GO:0006595 GO:0031145 GO:0038061 GO:0007265 GO:0090090 GO:0033209 GO:0070062 GO:0016032 GO:0006521 GO:0043488 GO:0000084 GO:0007173 GO:0038095 GO:0050852 GO:0000090 GO:0043066 GO:0008543 GO:0000209 GO:0016020 GO:0008286 |
| tr|A0A0S2Z491|A0A0S2Z491_ HUMAN | GO:0034080 GO:0005925 GO:0042803 GO:0043024 GO:0051082 GO:0006281 GO:0008284 GO:1902751 GO:0045727 GO:0043023 GO:0005829 GO:0005654 GO:0046599 GO:0007569 GO:0060699 GO:1904030 GO:0007165 GO:0010826 GO:0003713 GO:0031616 GO:0044822 GO:0016032 GO:0006886 GO:0051092 GO:0060735 GO:0042393 GO:0006913 GO:0008285 GO:0019901 GO:0046982 GO:0030957 GO:0032071 GO:0030529 GO:0051059 GO:0051259 GO:0004860 GO:0043066 GO:0044387 GO:0042255 GO:0005730 GO:0016020 |
| tr|A0A0S2Z4A1|A0A0S2Z4A1_ HUMAN | GO:0005945 GO:0051289 GO:0005980 GO:0008022 GO:0046835 GO:0016208 GO:0046716 GO:0093001 GO:0030388 GO:0046872 GO:0070095 GO:0097228 GO:0070062 GO:0042803 GO:0070061 GO:0061621 GO:0006002 GO:0001678 GO:0003872 GO:0005524 GO:0019900 GO:0032024 GO:0016324 |
| tr|A0A0S2Z4A5|A0A0S2Z4A5_ HUMAN | GO:0000785 GO:0005654 GO:0000075 GO:0005515 GO:0016020 GO:0000784 GO:0006268 GO:0008283 GO:0006974 GO:0003697 GO:0005829 GO:0006355 GO:0071466 GO:0042325 GO:0042493 GO:0042555 GO:0000082 GO:0000084 GO:0004003 GO:0071364 GO:0006270 GO:0005524 GO:0006271 |
| tr|A0A0S2Z4C3|A0A0S2Z4C3_ HUMAN | GO:0005829 GO:0048873 GO:0070062 GO:0006108 GO:0006099 GO:0004333 GO:0051262 GO:0005515 GO:0045239 GO:0005759 GO:0006106 |
| tr|A0A0S2Z4C6|A0A0S2Z4C6_ HUMAN | GO:0051533 GO:0033555 GO:0000082 GO:0006470 GO:0046676 GO:0051091 GO:0050774 GO:0005829 GO:0002223 GO:0050804 GO:0005654 GO:0045955 GO:0001975 GO:0008144 GO:0035562 GO:1903244 GO:0051592 GO:0005955 GO:0045944 GO:0042383 GO:0048741 GO:0060079 GO:0005739 GO:0071333 GO:0038095 GO:0014883 GO:0035690 GO:0046982 GO:0033173 GO:0005516 GO:0033192 GO:0098794 GO:0005509 GO:0030018 GO:0019899 GO:0042110 GO:0006816 |
| tr|A0A0S2Z4F6|A0A0S2Z4F6_ HUMAN | GO:0005925 GO:0017148 GO:0019901 GO:0035091 GO:0050900 GO:0007173 GO:0005829 GO:0034063 GO:0010494 GO:0007596 GO:0009967 GO:0042802 GO:0042995 GO:0003723 GO:0005070 GO:0005886 GO:0030335 |
| tr|A0A0S2Z4G4|A0A0S2Z4G4_ HUMAN | GO:0005829 GO:0030863 GO:0005903 GO:0007420 GO:0002102 GO:0070062 GO:0030049 GO:0001725 GO:0005862 GO:0032154 GO:0003779 GO:0031941 GO:0030426 |
| tr|A0A0S2Z4J6|A0A0S2Z4J6_ HUMAN | GO:0005829 GO:0018279 GO:0070062 GO:0043687 GO:0005515 GO:0043025 GO:0009298 GO:0004615 GO:0006488 |
| tr|A0A0S2Z4N8|A0A0S2Z4N8_ HUMAN | GO:0005925 GO:0007411 GO:0051289 GO:0015629 GO:0005829 GO:0003779 GO:0034329 GO:0050852 GO:0001843 GO:0031527 GO:0005923 GO:0005522 GO:0031258 GO:0070062 GO:0030838 GO:0017124 |
| tr|A0A0S2Z4Q3|A0A0S2Z4Q3_ HUMAN | GO:0005829 GO:0008270 GO:0003727 GO:0045944 GO:0044822 GO:0005515 GO:0003697 GO:0008284 GO:0006695 GO:0003700 GO:0005783 GO:0005634 |
| tr|A0A0S2Z4Q4|A0A0S2Z4Q4_ HUMAN | GO:0042176 GO:0042059 GO:0010324 GO:0010628 GO:0030141 GO:0036258 GO:0072657 GO:0043130 GO:0005829 GO:0033565 GO:0008285 GO:0046426 GO:0019904 GO:0005769 GO:0043405 GO:0008333 GO:0006914 GO:0006622 GO:0006892 GO:0070062 GO:1903543 GO:0010008 |
| tr|A0A0S2Z4R1|A0A0S2Z4R1_ HUMAN | GO:0005829 GO:0004832 GO:0004871 GO:0006915 GO:0005615 GO:0005524 GO:0006437 GO:0044822 GO:0007165 GO:0005153 GO:0000049 GO:0004831 GO:0005634 |
| tr|A0A0S2Z4V6|A0A0S2Z4V6_ HUMAN | GO:0033613 GO:0043524 GO:0031016 GO:0007601 GO:0030176 GO:0030433 GO:0031625 GO:0000502 GO:0051117 GO:0007605 GO:0051726 GO:0045762 GO:0051928 GO:0031398 GO:0043433 GO:0001822 GO:0032469 GO:0042593 GO:0045927 GO:0006983 GO:0042048 GO:0000122 GO:0036498 GO:0022417 GO:0048306 GO:1902236 GO:2000675 GO:0030425 GO:0050821 GO:0005516 GO:1903892 GO:0003091 |
| tr|A0A0S2Z4W4|A0A0S2Z4W4_ HUMAN | GO:0005654 GO:0000122 GO:0070935 GO:0008270 GO:0000166 GO:0048025 GO:0044822 GO:0034393 GO:0008285 GO:0042802 GO:0032403 |
| tr|A0A0S2Z4Y4|A0A0S2Z4Y4_ HUMAN | GO:0005829 GO:0006892 GO:0048268 GO:0030136 GO:0030276 GO:0005802 GO:0048471 GO:0005654 GO:0005798 GO:0016020 |
| tr|A0A0S2Z4Y5|A0A0S2Z4Y5_ HUMAN | GO:0006501 GO:0035269 GO:0006506 GO:0018279 GO:0019673 GO:0043178 GO:0005537 GO:0033185 GO:0004582 GO:0004169 GO:0005515 GO:0006488 GO:0005789 GO:0005634 GO:0019348 |
| tr|A0A0S2Z4Z0|A0A0S2Z4Z0_ HUMAN | GO:0000166 GO:0009725 GO:0016592 GO:0001104 GO:0006310 GO:0044822 GO:0042921 GO:0016575 GO:0046600 GO:0005730 GO:0030674 GO:0005667 GO:0060395 GO:0030520 GO:0030529 GO:0045944 GO:0030374 GO:0006260 GO:0006281 |
| tr|A0A0S2Z4Z2|A0A0S2Z4Z2_ HUMAN | GO:0005654 GO:0070740 GO:0010390 GO:0070738 GO:0043773 GO:0008270 GO:0003730 GO:0031624 GO:0043774 GO:0043679 GO:0004842 GO:2001168 GO:1900364 GO:0043434 GO:0008766 GO:0070736 GO:0005829 GO:0006511 GO:0033503 GO:0031625 GO:1902916 GO:1901800 GO:0070737 GO:0017075 GO:0032403 GO:0042803 GO:0007346 GO:0018169 GO:0019898 |
| tr|A0A0S2Z4Z9|A0A0S2Z4Z9_ HUMAN | GO:0000980 GO:0016020 GO:1903377 GO:0044822 GO:0090575 GO:0001047 GO:0006974 GO:0006259 GO:0045892 GO:0042802 GO:0016363 GO:0042382 GO:0003682 GO:0000398 GO:0042752 |
| tr|A0A0S2Z556|A0A0S2Z556_ HUMAN | GO:0043021 GO:0008022 GO:0060271 GO:0016607 GO:0043484 GO:0097546 GO:0072372 GO:0034063 GO:0006355 GO:0010494 GO:0071598 GO:0003713 GO:0005813 GO:0003677 GO:0000380 GO:0048814 |
| tr|A0A0S2Z583|A0A0S2Z583_ HUMAN | GO:0042572 GO:0016021 GO:0042574 GO:0055114 GO:0004745 GO:0006775 GO:0016062 GO:0042622 GO:0052650 GO:0001917 GO:0005789 |
| tr|A0A0S2Z5B3|A0A0S2Z5B3_ HUMAN | GO:0050313 GO:0070221 GO:0006749 GO:0005759 GO:0070813 GO:0000098 GO:0005654 GO:0005506 |
| tr|A0A0S2Z5D2|A0A0S2Z5D2_ HUMAN | GO:0070126 GO:0070125 GO:0042769 GO:0005743 GO:0003735 GO:0005763 GO:0044822 GO:0070124 |
| tr|A0A0S2Z5D4|A0A0S2Z5D4_ HUMAN | GO:0005829 GO:0047485 GO:0007097 GO:0018279 GO:0061024 GO:0005813 GO:0043687 GO:0005868 GO:0006888 GO:0019886 GO:0005869 GO:0005634 |
| tr|A0A0S2Z5I7|A0A0S2Z5I7_ HUMAN | GO:0005654 GO:0044822 GO:0002573 GO:0043148 GO:0043022 GO:0005737 GO:0005730 GO:0001833 GO:0008017 GO:0030282 GO:0042256 GO:0048539 GO:0031017 GO:0006364 GO:0030595 GO:0000922 GO:0019843 |
| tr|A0A0S2Z5P5|A0A0S2Z5P5_ HUMAN | GO:0007067 GO:0042555 GO:0006261 GO:0051301 GO:0007062 GO:0005515 GO:0005654 GO:0005737 GO:0003682 GO:0005886 |
| tr|A0A0S2Z5U1|A0A0S2Z5U1_ HUMAN | GO:0008137 GO:0009055 GO:0032981 GO:0005743 GO:0061179 GO:0044281 GO:0072593 GO:0022904 |
| tr|A0A0S2Z5U6|A0A0S2Z5U6_ HUMAN | GO:1903206 GO:0055114 GO:0042802 GO:0034641 GO:0051881 GO:0055129 GO:0005759 GO:0004735 |
| tr|A0A0S2Z5U7|A0A0S2Z5U7_ HUMAN | GO:0051402 GO:0005829 GO:0008635 GO:0008631 GO:0008625 GO:0005758 GO:0005515 GO:0035631 GO:0009898 |
| tr|A0A0S2Z5V7|A0A0S2Z5V7_ HUMAN | GO:0042500 GO:0071556 GO:0006509 GO:0036513 GO:0031625 GO:0071458 GO:0009986 GO:0031293 GO:0005791 GO:0051289 GO:0042803 GO:1904211 GO:0005886 |
| tr|A0A0S2Z5X1|A0A0S2Z5X1_ HUMAN | GO:0016021 GO:0043234 GO:0044267 GO:0006457 GO:0005743 GO:0006626 GO:0007601 GO:0005515 GO:0048806 |
| tr|A0A0S2Z693|A0A0S2Z693_ HUMAN | GO:0005829 GO:0005743 GO:0046872 GO:0006552 GO:0009374 GO:0005524 GO:0005515 GO:0005759 GO:0004075 GO:0004485 GO:0006768 |
| tr|A0A0U1RQC9|A0A0U1RQC9_ HUMAN | GO:2000772 GO:0043621 GO:0043525 GO:0000060 GO:1901525 GO:0051721 GO:0031497 GO:0061419 GO:0016363 GO:0042149 GO:0010666 GO:0048512 GO:0031052 GO:2001244 GO:0035264 GO:0002020 GO:0005657 GO:0035035 GO:0071850 GO:0030512 GO:0050731 GO:0002326 GO:0030971 GO:0034103 GO:0009303 GO:0021549 GO:0010165 GO:0046982 GO:0035690 GO:0051097 GO:0000979 GO:0001701 GO:0010332 GO:0016605 GO:1902253 GO:0008270 GO:1900119 GO:0031625 GO:0007219 GO:0035861 GO:0042802 GO:0071494 GO:0005507 GO:0002309 GO:0043066 GO:0006284 GO:0006302 GO:1900740 GO:0001077 GO:0060411 GO:0007596 GO:0051087 GO:0006983 GO:0007369 GO:2000379 GO:0031065 GO:0005783 GO:1904024 GO:0002931 GO:0048147 GO:0001756 GO:2000269 GO:0072363 GO:0005759 GO:0033077 GO:0046677 GO:0030308 GO:0000733 GO:2000378 GO:0071479 GO:0006289 GO:0045861 GO:0006977 GO:0005829 GO:0048568 GO:0005730 GO:0001085 GO:0002039 GO:0070266 GO:1990440 GO:0005669 GO:0006367 GO:0003684 GO:0007406 GO:0005524 GO:0042771 GO:0035033 GO:0006978 GO:0000122 GO:0047485 GO:0002360 GO:0000790 GO:0090403 GO:0016032 GO:0051974 GO:0003682 GO:0070245 GO:0009651 GO:0090399 GO:0007265 GO:0032461 GO:0043504 GO:0043153 GO:0090200 GO:0097252 GO:1990144 GO:0051262 GO:0008340 |
| tr|A0A0U1RQF0|A0A0U1RQF0_ HUMAN | GO:0030879 GO:0004320 GO:0031325 GO:0004317 GO:0071353 GO:0015939 GO:0001649 GO:0008144 GO:0047117 GO:0044822 GO:0006112 GO:0042470 GO:0004313 GO:0004316 GO:0005794 GO:0005829 GO:0006633 GO:0016295 GO:0019432 GO:0004319 GO:0006084 GO:0004314 GO:0047451 GO:0070402 GO:0016296 GO:0035338 GO:0004315 GO:0070062 GO:0042803 GO:0005886 GO:0005739 GO:0042587 |
| tr|A0A0U1RQQ1|A0A0U1RQQ1_ HUMAN | GO:0030032 GO:0030027 GO:0031529 GO:0031941 GO:1902745 GO:0046415 GO:0044354 GO:0005829 GO:0007596 GO:0051639 GO:2000813 GO:0032403 GO:0051638 GO:0070062 GO:0044351 GO:0051496 GO:0005886 GO:0030335 GO:0005634 GO:1900026 |
| tr|A0A0U1RQV4|A0A0U1RQV4_ HUMAN | GO:0043524 GO:0004674 GO:0032059 GO:0016525 GO:0007249 GO:0003383 GO:0007411 GO:0048010 GO:0032091 GO:1903140 GO:0045616 GO:0030027 GO:0050901 GO:0046872 GO:0005829 GO:1903347 GO:0090002 GO:0030866 GO:0051492 GO:0032060 GO:0051451 GO:0022614 GO:0048013 GO:0005814 GO:0000139 GO:2000114 GO:0006921 GO:0005886 GO:0017049 GO:0035509 GO:0005524 GO:0006939 GO:2000145 GO:0051894 GO:0007266 GO:0001726 GO:0006468 |
| tr|A0A0U1RQZ9|A0A0U1RQZ9_ HUMAN | GO:0048731 GO:0005654 GO:0000122 GO:0008094 GO:0071565 GO:0005515 GO:0000166 GO:0016514 GO:0001105 GO:0071564 GO:0045111 GO:0008285 GO:0004386 GO:0044212 GO:0030308 GO:0006338 GO:0030154 GO:0000790 GO:0045944 |
| tr|A0A0U1RR18|A0A0U1RR18_ HUMAN | GO:0015379 GO:1902476 GO:0005887 GO:0071805 GO:0007268 GO:0019901 GO:0022820 GO:0006884 |
| tr|A0A0U1RRM1|A0A0U1RRM1_ HUMAN | GO:0043044 GO:0000980 GO:0000122 GO:0008270 GO:0016607 GO:0031492 GO:0016581 GO:0000978 GO:0006306 GO:0000790 GO:0003700 |
| tr|A0A0U1WL47|A0A0U1WL47_ HUMAN | GO:0005829 GO:0036155 GO:0016021 GO:0046474 GO:0005515 GO:0034389 GO:0004806 GO:0010898 GO:0044281 GO:0005811 GO:0005789 GO:0010891 GO:0005886 |
| tr|A0A0X1KG71|A0A0X1KG71_ HUMAN | GO:0034244 GO:0050434 GO:0032021 GO:0005515 GO:0005737 |
| tr|A0A140TA86|A0A140TA86_ HUMAN | GO:0042407 GO:0044284 GO:0061617 |
| tr|A0A140VJC9|A0A140VJC9_ HUMAN | GO:0005737 GO:0034869 GO:0044466 GO:0034843 GO:0008474 GO:0034946 GO:0002084 GO:0006631 GO:0070062 GO:0052689 |
| tr|A0A140VJD0|A0A140VJD0_ HUMAN | GO:0016568 GO:0005815 GO:0035064 GO:0005664 GO:0031933 GO:0005721 GO:0006270 GO:0008327 GO:0005737 GO:0003682 GO:0071169 |
| tr|A0A140VJD7|A0A140VJD7_ HUMAN | GO:0006796 GO:0005739 GO:0005515 GO:0003998 |
| tr|A0A140VJF3|A0A140VJF3_ HUMAN | GO:0005654 GO:0005515 GO:0000236 GO:0051321 GO:0008608 GO:0000777 GO:0005829 GO:0007264 GO:0000090 GO:0031145 GO:0007094 |
| tr|A0A140VJG8|A0A140VJG8_ HUMAN | GO:0016206 GO:0016036 GO:0032496 GO:0043197 GO:0005515 GO:0042420 GO:0048662 GO:0008210 GO:0035814 GO:0007612 GO:0044297 GO:0042135 GO:0050668 GO:0005829 GO:0007614 GO:0045211 GO:0007565 GO:0042136 GO:0014070 GO:0030424 GO:0006805 GO:0000287 GO:0042493 GO:0007268 GO:0016021 GO:0046498 GO:0048609 GO:0032259 GO:0051930 GO:0070062 GO:0048265 GO:0046500 GO:0045963 GO:0032502 GO:0005739 |
| tr|A0A140VJH7|A0A140VJH7_ HUMAN | GO:0006071 GO:0005515 GO:0046835 GO:0061624 GO:0005829 GO:0034012 GO:0046872 GO:0061625 GO:0039534 GO:0070062 GO:0004371 GO:0005634 GO:0005524 GO:0050354 |
| tr|A0A140VJI5|A0A140VJI5_ HUMAN | GO:0004725 GO:0008138 GO:0004722 GO:0005515 GO:0035335 GO:0005634 GO:0005829 GO:0070062 |
| tr|A0A140VJJ2|A0A140VJJ2_ HUMAN | GO:0047374 GO:0018738 GO:0006805 GO:0016023 GO:0070062 GO:0046294 GO:0005788 GO:0005515 GO:0005654 GO:1901687 GO:0005794 GO:0005886 |
| tr|A0A140VJK1|A0A140VJK1_ HUMAN | GO:0002026 GO:0005080 GO:0030018 GO:0055114 GO:0051536 GO:0030425 GO:0046872 GO:0045454 GO:0070062 GO:0009055 GO:0005938 GO:0044822 GO:0010614 GO:0005634 GO:0015035 |
| tr|A0A140VJK2|A0A140VJK2_ HUMAN | GO:0016021 GO:0043010 GO:0055114 GO:0009331 GO:0005509 GO:0005743 GO:0006734 GO:0004367 GO:0035264 GO:0019563 GO:0006072 GO:0006094 GO:0004345 GO:0050661 GO:0052591 GO:0044255 |
| tr|A0A140VJK7|A0A140VJK7_ HUMAN | GO:0070062 GO:0005739 GO:0005515 |
| tr|A0A140VJL0|A0A140VJL0_ HUMAN | GO:0006574 GO:0005759 GO:0034641 GO:0003860 GO:0016836 GO:0070062 |
| tr|A0A140VJL8|A0A140VJL8_ HUMAN | GO:0046855 GO:0005829 GO:0031403 GO:0030145 GO:0052833 GO:0006021 GO:0070062 GO:0052832 GO:0006661 GO:0008934 GO:0000287 GO:0046854 GO:0007165 GO:0042803 |
| tr|A0A140VJP1|A0A140VJP1_ HUMAN | GO:0001764 GO:0000422 GO:0016055 GO:0005515 GO:0005884 GO:0018107 GO:0030295 GO:0044822 GO:0051493 GO:0051646 GO:0045197 GO:0032147 GO:0046777 GO:0045180 GO:0016328 GO:0004674 GO:0000287 GO:0008289 GO:0030010 GO:0010976 GO:0097427 GO:0005634 GO:0005524 GO:0050321 GO:0050770 GO:0035556 GO:0005739 |
| tr|A0A140VJP2|A0A140VJP2_ HUMAN | GO:0005829 GO:0005739 GO:0006805 GO:0048269 GO:0070062 GO:0006556 GO:0006730 GO:0032259 GO:0019899 GO:0048270 GO:0050790 GO:0005634 |
| tr|A0A140VJP5|A0A140VJP5_ HUMAN | GO:0005829 GO:0004478 GO:0006805 GO:0009416 GO:0048269 GO:0046872 GO:0009725 GO:0006556 GO:0006730 GO:0032259 GO:0007623 GO:0005524 GO:0005515 GO:0042493 GO:0016597 GO:0051591 |
| tr|A0A140VJQ4|A0A140VJQ4_ HUMAN | GO:0034214 GO:0003992 GO:0042802 GO:0004587 GO:0034641 GO:0010121 GO:0006591 GO:0007601 GO:0055129 GO:0005759 GO:0019544 GO:0030170 |
| tr|A0A140VJR2|A0A140VJR2_ HUMAN | GO:0030145 GO:0004181 GO:0005515 GO:0004177 GO:0016805 GO:0006520 GO:0030574 GO:0006508 GO:0070062 |
| tr|A0A140VJS3|A0A140VJS3_ HUMAN | GO:0031410 GO:0048011 GO:0031531 GO:0051436 GO:0045899 GO:0031595 GO:0008540 GO:0002479 GO:0030433 GO:0090263 GO:0007411 GO:0048010 GO:0000186 GO:0072562 GO:0045892 GO:0006977 GO:0003712 GO:0002223 GO:0051437 GO:0006595 GO:0031145 GO:0031597 GO:0005675 GO:0017025 GO:0038061 GO:0007265 GO:0090090 GO:0033209 GO:0070062 GO:0016032 GO:0006521 GO:0036402 GO:0043488 GO:0000084 GO:0007173 GO:0005524 GO:0038095 GO:1901800 GO:0050852 GO:0016234 GO:0090261 GO:0000090 GO:0043066 GO:0008543 GO:0000209 GO:0016020 GO:0008286 |
| tr|A0A140VJS6|A0A140VJS6_ HUMAN | GO:0048011 GO:0051436 GO:0002479 GO:0090263 GO:0007411 GO:0048010 GO:0000186 GO:0006977 GO:0005829 GO:0002223 GO:0014070 GO:0051437 GO:0005654 GO:0006595 GO:0031145 GO:0038061 GO:0007265 GO:0090090 GO:0033209 GO:0070062 GO:0016032 GO:0006521 GO:0043488 GO:0005515 GO:0000084 GO:0007173 GO:0038095 GO:0005839 GO:0050852 GO:0004298 GO:0000090 GO:0043066 GO:0008543 GO:0000209 GO:0016020 GO:0008286 |
| tr|A0A140VJT8|A0A140VJT8_ HUMAN | GO:0032311 GO:0090502 GO:0003723 GO:0005739 GO:0070062 GO:0004523 GO:0043137 GO:0008428 GO:0006402 GO:0043086 GO:0005515 GO:0000287 GO:0045765 GO:0005654 |
| tr|A0A140VJW5|A0A140VJW5_ HUMAN | GO:0005829 GO:0017101 GO:0070062 GO:0006461 GO:0006422 GO:0001887 GO:0005524 GO:0004815 GO:0044822 GO:0004046 GO:0005515 GO:0016020 |
| tr|A0A140VJX1|A0A140VJX1_ HUMAN | GO:0010878 GO:0033344 GO:0010742 GO:0034641 GO:0034736 GO:0046951 GO:0009725 GO:0015485 GO:0005759 GO:0034435 GO:0009083 GO:0008203 GO:0005743 GO:0005789 GO:0034379 GO:0005777 GO:0072229 GO:0046872 GO:0007420 GO:0014070 GO:0042594 GO:0016021 GO:0060612 GO:0000062 GO:0070062 GO:0042803 GO:0051260 GO:0001889 GO:0019899 GO:0003985 GO:0042986 GO:0042632 GO:0046952 |
| tr|A0A140VJX3|A0A140VJX3_ HUMAN | GO:0019346 GO:0005829 GO:0009636 GO:0009440 GO:0030054 GO:0021510 GO:0043005 GO:0004792 GO:0005743 GO:0001889 GO:0045202 GO:0070062 GO:0042802 GO:0070814 GO:0001822 GO:0016784 |
| tr|A0A140VJY7|A0A140VJY7_ HUMAN | GO:0098869 GO:0005515 GO:0047134 GO:0004601 GO:0033209 GO:0005829 GO:0070062 GO:0055114 |
| tr|A0A140VJZ1|A0A140VJZ1_ HUMAN | GO:0005764 GO:0008242 GO:0008270 GO:0043130 GO:0004197 GO:0004843 GO:0071108 GO:0032436 |
| tr|A0A140VJZ4|A0A140VJZ4_ HUMAN | GO:0006511 GO:0032869 GO:0045600 GO:0042755 GO:0070062 GO:0043130 GO:0004843 GO:0016579 GO:0060041 GO:0007628 GO:0005654 GO:0005737 |
| tr|A0A140VK00|A0A140VK00_ HUMAN | GO:0042605 GO:0002474 GO:0090501 GO:0071806 GO:0001948 GO:0008320 GO:0001580 GO:0070062 GO:0005615 GO:0006955 GO:0008285 GO:0007155 GO:0001895 GO:0004540 GO:0042612 GO:0005634 |
| tr|A0A140VK05|A0A140VK05_ HUMAN | GO:0016021 GO:0032947 GO:1904146 GO:0051534 GO:0031594 GO:0005829 GO:0007596 GO:0005759 GO:0010614 GO:0016310 GO:0010738 GO:0035308 GO:0048487 GO:0044822 GO:0005811 GO:0019904 GO:0071320 GO:0045211 GO:0019901 GO:0071375 GO:0005741 GO:0030061 GO:0034237 GO:0016301 GO:0030346 GO:0008017 |
| tr|A0A140VK08|A0A140VK08_ HUMAN | GO:0005921 GO:0043195 GO:0051480 GO:0005509 GO:0005634 GO:0005829 |
| tr|A0A140VK09|A0A140VK09_ HUMAN | GO:0030220 GO:0045653 GO:2000256 GO:0016324 GO:0008022 GO:0030426 GO:0070374 GO:0005783 GO:0032433 GO:0010977 GO:0030027 GO:0043495 GO:0005654 GO:0071356 GO:1904030 GO:0030424 GO:0044325 GO:0070886 GO:0031122 GO:0042383 GO:0097191 GO:0070062 GO:0071902 GO:0048471 GO:0051898 GO:0007286 GO:0030307 GO:0005794 GO:0051092 GO:0038163 GO:0090314 GO:0051302 GO:0006302 GO:0048554 GO:0008427 GO:0090004 GO:0008285 GO:0019901 GO:0090050 GO:0030425 GO:0033630 GO:0007229 GO:0002931 GO:1900026 GO:1990090 GO:0007113 GO:0005509 GO:0043066 GO:0005813 GO:0043025 GO:0071901 GO:0032587 GO:0030291 GO:0001954 GO:0017016 GO:0007026 |
| tr|A0A140VK12|A0A140VK12_ HUMAN | GO:0030126 GO:0005829 GO:0018279 GO:0030133 GO:0006890 GO:0061024 GO:0006891 GO:0043687 GO:0006888 GO:0006886 GO:0005198 |
| tr|A0A140VK27|A0A140VK27_ HUMAN | GO:0005654 GO:0043171 GO:0004463 GO:0005515 GO:0008270 GO:0044822 GO:0043434 GO:0060509 GO:0004301 GO:0005829 GO:0010043 GO:0042277 GO:0006954 GO:0019369 GO:0019370 GO:0006508 GO:0070062 GO:0005886 GO:0070006 |
| tr|A0A140VK29|A0A140VK29_ HUMAN | GO:0015936 GO:0005829 GO:0006552 GO:0005524 GO:0005515 GO:0005759 GO:0004485 GO:0006768 |
| tr|A0A140VK39|A0A140VK39_ HUMAN | GO:0034047 GO:0008601 GO:0051721 GO:0006482 GO:0004864 GO:0002028 GO:0043086 GO:0019901 GO:0051722 |
| tr|A0A140VK42|A0A140VK42_ HUMAN | GO:0048011 GO:0051436 GO:0045899 GO:0031595 GO:0008540 GO:0001824 GO:0002479 GO:0030433 GO:0090263 GO:0007411 GO:0048010 GO:0000186 GO:0006977 GO:1903507 GO:0002223 GO:0051437 GO:0005654 GO:0006595 GO:0031145 GO:0031597 GO:0003714 GO:0017025 GO:0038061 GO:0003713 GO:0007265 GO:0090090 GO:0033209 GO:0016032 GO:0048471 GO:0006521 GO:0036402 GO:0043488 GO:0000084 GO:0007173 GO:0005524 GO:0038095 GO:1901800 GO:0000932 GO:0050852 GO:0000090 GO:0043066 GO:0008543 GO:0000209 GO:0016020 GO:0008286 |
| tr|A0A140VK46|A0A140VK46_ HUMAN | GO:0048011 GO:0051436 GO:0002479 GO:0090263 GO:0007411 GO:0048010 GO:0000186 GO:0006977 GO:0005829 GO:0002223 GO:0051437 GO:0001530 GO:0005654 GO:0006595 GO:0031145 GO:0038061 GO:0007265 GO:0090090 GO:0033209 GO:0002862 GO:0070062 GO:0016032 GO:0006521 GO:0043488 GO:0005515 GO:0000084 GO:0007173 GO:0038095 GO:0005839 GO:0050852 GO:0004298 GO:0000090 GO:0043066 GO:0008543 GO:0000209 GO:0008286 |
| tr|A0A140VK53|A0A140VK53_ HUMAN | GO:0015030 GO:0000398 GO:0016607 GO:0047485 GO:0070742 GO:0071013 GO:0044822 |
| tr|A0A140VK54|A0A140VK54_ HUMAN | GO:0014067 GO:0005654 GO:0016579 GO:0007259 GO:0046580 GO:0046872 GO:0008284 GO:0019904 GO:0032154 GO:0004843 GO:0005769 GO:0008237 GO:0070062 GO:0016920 GO:0000281 GO:0043524 GO:0005886 |
| tr|A0A140VK56|A0A140VK56_ HUMAN | GO:0005829 GO:0005999 GO:0048029 GO:0004801 GO:0070062 GO:0009052 GO:0006002 GO:0005515 GO:0006112 GO:0005634 |
| tr|A0A140VK65|A0A140VK65_ HUMAN | GO:0046961 GO:0033572 GO:0005515 GO:0046034 GO:0008286 GO:0006879 GO:0033180 GO:0015991 GO:0005902 GO:0042470 GO:0005765 GO:0043209 GO:0007035 GO:0005829 GO:0045454 GO:0001726 GO:0016021 GO:0012505 GO:0070062 GO:0005886 GO:0005524 |
| tr|A0A140VK67|A0A140VK67_ HUMAN | GO:0005515 GO:0006915 |
| tr|A0A140VK69|A0A140VK69_ HUMAN | GO:0004069 GO:0006094 GO:0007219 GO:0055089 GO:0006107 GO:0032869 GO:0006532 GO:0006533 GO:0019509 GO:0043679 GO:0005764 GO:0031406 GO:0051384 GO:0005829 GO:0080130 GO:0006114 GO:0042802 GO:0030170 GO:0047801 GO:0070062 GO:0004609 GO:0005634 GO:0006595 GO:0019550 GO:0019551 GO:0005739 |
| tr|A0A140VK70|A0A140VK70_ HUMAN | GO:0048011 GO:0051436 GO:0045899 GO:0008233 GO:0031595 GO:0008540 GO:0002479 GO:0030433 GO:0090263 GO:0007411 GO:0048010 GO:0000186 GO:0006977 GO:0002223 GO:0051437 GO:0005654 GO:0006595 GO:0031145 GO:0031597 GO:0017025 GO:0038061 GO:0001649 GO:0007265 GO:0090090 GO:0033209 GO:0016032 GO:0006521 GO:0036402 GO:0043488 GO:0000084 GO:0007173 GO:0005524 GO:0038095 GO:1901800 GO:0000932 GO:0050852 GO:0000090 GO:0043066 GO:0008543 GO:0000209 GO:0016020 GO:0008286 |
| tr|A0A140VK83|A0A140VK83_ HUMAN | GO:0070062 GO:0007059 GO:0005515 GO:0035307 GO:0005694 GO:0050790 GO:0005737 GO:0008599 GO:0005634 |
| tr|A0A140VK93|A0A140VK93_ HUMAN | GO:0005758 GO:0046939 GO:0006172 GO:0005743 GO:0005829 GO:0006119 GO:0046083 GO:0007420 GO:0097066 GO:0015949 GO:0046060 GO:0070062 GO:0046033 GO:0001889 GO:0097226 GO:0005524 GO:0004017 |
| tr|A0A140VKA6|A0A140VKA6_ HUMAN | GO:0005829 GO:0030674 GO:0043234 GO:0070389 GO:0051087 GO:0051260 GO:0070062 GO:0032403 GO:0042802 GO:0032564 GO:0019904 GO:0061084 GO:0051082 GO:0030544 |
| tr|A0A140VKA9|A0A140VKA9_ HUMAN | GO:0070404 GO:0005829 GO:0055114 GO:0035690 GO:0043005 GO:0005739 GO:0033762 GO:0001889 GO:0051066 GO:0006729 GO:0006559 GO:0070062 GO:0009055 GO:0010044 GO:0004155 GO:0070402 GO:0010288 GO:0042803 |
| tr|A0A140VKB1|A0A140VKB1_ HUMAN | GO:0005654 GO:0042787 GO:0061418 GO:0005730 GO:0005829 GO:0097193 GO:0008285 GO:0007050 GO:0031625 GO:0000082 GO:0031462 GO:0032403 GO:0016032 GO:0030891 GO:0061630 |
| tr|A0A140VKC8|A0A140VKC8_ HUMAN | GO:0019835 GO:0005829 GO:0016021 GO:0055114 GO:0018279 GO:0019673 GO:0042356 GO:0070062 GO:0050577 GO:0009055 GO:0043687 GO:0016853 GO:0007159 GO:0050662 GO:0006488 GO:0042351 |
| tr|A0A140VKE7|A0A140VKE7_ HUMAN | GO:0006457 GO:0005515 GO:0006986 GO:0005524 GO:0005634 GO:0005829 |
| tr|A0A140VKE9|A0A140VKE9_ HUMAN | GO:0003777 GO:0005829 GO:0006996 GO:0018279 GO:0005874 GO:0000086 GO:0061024 GO:0032403 GO:0005813 GO:0043687 GO:0031982 GO:0005868 GO:0007018 GO:0006888 GO:0016032 GO:0019886 |
| tr|A0A140VKF2|A0A140VKF2_ HUMAN | GO:0048011 GO:0051436 GO:0002479 GO:0090263 GO:0007411 GO:0048010 GO:0000186 GO:0008541 GO:0016920 GO:0046872 GO:0006977 GO:0002223 GO:0001878 GO:0051437 GO:0005654 GO:0006595 GO:0031145 GO:0045471 GO:0031597 GO:0038061 GO:0070536 GO:0007265 GO:0004175 GO:0090090 GO:0033209 GO:0070062 GO:0070628 GO:0016032 GO:0006521 GO:0043488 GO:0010950 GO:0000084 GO:0007173 GO:0038095 GO:0006303 GO:0050852 GO:0008237 GO:0061136 GO:0003984 GO:0000090 GO:0043066 GO:0008543 GO:0000724 GO:0061133 GO:0000209 GO:0008286 |
| tr|A0A140VKH3|A0A140VKH3_ HUMAN | GO:0005737 GO:0005654 GO:0016363 GO:0017151 GO:0044822 |
| tr|A0A158RFU6|A0A158RFU6_ HUMAN | GO:0032419 GO:0005525 GO:0019076 GO:0048524 GO:0034045 GO:0031902 GO:0019003 GO:0006622 GO:0030904 GO:0042147 GO:0007174 GO:0097208 GO:0030670 GO:0033162 GO:1903543 GO:0045022 GO:0019886 GO:0090383 GO:0043195 GO:0022615 GO:0000421 GO:0070062 GO:0005811 GO:0007264 GO:0005794 GO:0006913 GO:0045453 GO:0003924 GO:2000785 GO:0008333 GO:0090385 GO:0061724 GO:0045732 GO:0048365 |
| tr|A0AV58|A0AV58_ HUMAN | GO:0051721 GO:0005654 GO:0000122 GO:0000159 GO:0032355 GO:0005794 GO:0070016 GO:0030425 GO:0033147 GO:0043025 GO:0045944 GO:0032403 GO:0005886 GO:0005516 GO:0003700 |
| tr|A0M8W4|A0M8W4_ HUMAN | GO:0005654 GO:0000075 GO:0070534 GO:0000724 GO:0006303 GO:0008283 GO:0070911 GO:0005737 GO:0032436 GO:0031372 GO:0061631 GO:0031625 GO:0042275 GO:0010976 GO:0000729 GO:0070062 GO:0043524 GO:0051965 GO:0061630 GO:0045739 |
| tr|A0MNN4|A0MNN4_ HUMAN | GO:0005634 GO:0005515 GO:0005737 |
| tr|A0MNN5|A0MNN5_ HUMAN | GO:0005730 GO:0044822 GO:0005737 |
| tr|A0MNP2|A0MNP2_ HUMAN | GO:0005737 GO:0005682 GO:0005654 GO:0005732 GO:0000398 GO:0005515 GO:0019013 GO:0071011 GO:0016021 GO:0071013 GO:0044822 |
| tr|A0PJ48|A0PJ48_ HUMAN | GO:0030659 GO:0008360 GO:0031434 GO:0042981 GO:0000186 GO:0001558 GO:0031572 GO:0006612 GO:0048041 GO:0005730 GO:0030036 GO:0030425 GO:0005856 GO:0016021 GO:0043235 GO:0004709 GO:0046330 GO:0007346 GO:0005524 GO:0016477 |
| tr|A1A4E9|A1A4E9_ HUMAN | GO:0043587 GO:0009314 GO:0071300 GO:0005515 GO:0007010 GO:0005634 GO:0070062 GO:0045095 GO:0005198 |
| tr|A1L3A7|A1L3A7_ HUMAN | GO:0016020 GO:0044822 GO:0042788 GO:0005634 GO:0005515 |
| tr|A2A2Q9|A2A2Q9_ HUMAN | GO:0000244 |
| tr|A2A3R6|A2A3R6_ HUMAN | GO:0043065 GO:0000028 GO:0003735 GO:0007067 GO:0007369 GO:0000082 GO:0002309 GO:0006414 GO:0006924 GO:0036464 GO:0016259 GO:0033077 GO:0006364 GO:0006413 GO:0005844 GO:0042593 GO:0031929 GO:0006415 GO:0022605 GO:0048821 GO:0006614 GO:0044822 GO:0048471 GO:0001890 GO:0019901 GO:0019083 GO:0030425 GO:0000184 GO:0043066 GO:0005730 GO:0022627 GO:0007093 GO:0044297 GO:0016020 GO:0008286 |
| tr|A2A3U5|A2A3U5_ HUMAN | GO:0008144 GO:0030054 GO:0038003 GO:0005640 GO:0004985 GO:0006869 GO:0005887 GO:0097481 GO:0007399 GO:0005515 GO:0045211 GO:0043523 GO:0030426 GO:0005811 GO:0005789 GO:0005637 |
| tr|A2AB90|A2AB90_ HUMAN | GO:0046979 GO:0061635 GO:0042825 GO:0000139 GO:0071556 GO:0006955 GO:0010468 GO:0042288 GO:0002479 GO:0002397 GO:0015433 GO:0051082 GO:0046978 GO:0015833 GO:0019885 GO:0050823 GO:0006890 GO:0042605 GO:0006952 |
| tr|A2ACR1|A2ACR1_ HUMAN | GO:0007173 GO:0038095 GO:0048536 GO:0000186 GO:0046967 GO:0046979 GO:0090090 GO:0098586 GO:0000084 GO:0034341 GO:0000209 GO:0006595 GO:0042613 GO:0031902 GO:0004298 GO:0005839 GO:0050852 GO:0019886 GO:0002223 GO:0002506 GO:0043066 GO:0045582 GO:0014889 GO:0038061 GO:0043488 GO:0071257 GO:1990111 GO:0000090 GO:0048010 GO:0050890 GO:0070628 GO:0048011 GO:0048538 GO:0070062 GO:0005654 GO:0009897 GO:0006521 GO:0005739 GO:0001889 GO:0023029 GO:0002479 GO:0006977 GO:0005829 GO:2000116 GO:0043531 GO:0007411 GO:0051437 GO:0042288 GO:0055085 GO:0005524 GO:0023026 GO:1901423 GO:0033209 GO:0090263 GO:0043657 GO:0002587 GO:0015440 GO:0042605 GO:0042825 GO:0008543 GO:0031145 GO:0043279 GO:0051436 GO:0007265 GO:0042493 GO:0019060 GO:0002503 GO:0071347 GO:0008286 GO:0015833 GO:0019885 GO:0005764 |
| tr|A2BEK1|A2BEK1_ HUMAN | GO:0032515 GO:0005515 GO:0000164 GO:0004865 GO:0005634 |
| tr|A2RUM7|A2RUM7_ HUMAN | GO:0005925 GO:0000184 GO:0006415 GO:0006413 GO:0005515 GO:0016020 GO:0044822 GO:0008097 GO:0016259 GO:0000027 GO:0006414 GO:0022625 GO:0005730 GO:0003735 GO:0019083 GO:0006614 GO:0070062 GO:0006364 |
| tr|A3KFL2|A3KFL2_ HUMAN | GO:0030307 GO:0000176 GO:0005515 GO:0071051 GO:0008312 GO:0071038 GO:0043928 GO:0043488 GO:0005730 GO:0000177 GO:0005829 GO:0034475 GO:0071034 GO:0071035 GO:0071049 GO:0034427 GO:0000467 GO:0000175 |
| tr|A3R0T8|A3R0T8_ HUMAN | GO:0098532 GO:0000122 GO:0005515 GO:0044822 GO:0016584 GO:0030261 GO:0005730 GO:0005720 GO:0080182 GO:0070062 GO:0031490 |
| tr|A3RJH1|A3RJH1_ HUMAN | GO:0009615 GO:0072669 GO:0071920 GO:0032508 GO:0033677 GO:0005515 GO:0016020 GO:0007275 GO:0006388 GO:0003712 GO:0044822 GO:0004527 GO:0006302 GO:0006355 GO:0010494 GO:0000245 GO:0003677 GO:0006446 GO:0008143 GO:0003725 GO:0003682 GO:0090305 GO:0004004 GO:0005524 GO:0010501 GO:0043330 GO:1903608 |
| tr|A4D0U5|A4D0U5_ HUMAN | GO:0005737 GO:0043234 GO:0008285 GO:0005886 GO:0005515 GO:0005925 GO:0005634 GO:0008270 GO:0044822 |
| tr|A4D0V4|A4D0V4_ HUMAN | GO:0048146 GO:0010952 GO:2000811 GO:0061180 GO:0009267 GO:0019901 GO:0060716 GO:0008290 GO:0051899 GO:0060056 GO:0007140 GO:0045019 GO:0005903 GO:0000188 GO:2001244 GO:0090090 GO:0001960 GO:1900085 GO:0060355 GO:0042524 GO:0030674 GO:0071455 GO:1901380 GO:0070836 GO:0034141 GO:0005929 GO:0032570 GO:0043627 GO:1901844 GO:0003779 GO:0046982 GO:0032091 GO:0033138 GO:0002053 GO:2000535 GO:0006641 GO:0030193 GO:0051117 GO:0001525 GO:0098903 GO:0031397 GO:0045907 GO:0005925 GO:0042802 GO:0033278 GO:0051001 GO:0098911 GO:0043046 GO:0016324 GO:0005109 GO:0009617 GO:0007283 GO:0060045 GO:0061099 GO:0031398 GO:0030182 GO:0002080 GO:0045944 GO:0050769 GO:0050998 GO:0001937 GO:0033137 GO:0005578 GO:0007267 GO:0002931 GO:0070062 GO:1900027 GO:0030857 GO:0051592 GO:0086098 GO:0010608 GO:0016504 GO:0034587 GO:0060546 GO:0005768 GO:0060501 GO:0086091 GO:0001666 GO:0071360 GO:0030863 GO:0033484 GO:0005829 GO:0071375 GO:0016323 GO:0007519 GO:0005615 GO:0010524 GO:0008104 GO:1903598 GO:0032947 GO:0048365 GO:0060317 GO:0045165 GO:0070320 GO:0055009 GO:2001238 GO:0007595 GO:0006940 GO:0000122 GO:0019217 GO:0071546 GO:0005887 GO:0051260 GO:0090263 GO:0048554 GO:0051091 GO:0032092 GO:0060492 GO:0044325 GO:1901979 GO:0034098 GO:0030514 GO:0072584 GO:2000286 GO:0031295 GO:0005125 GO:0005901 GO:0071560 GO:0042632 GO:0000139 GO:0051016 GO:0001570 GO:0019065 GO:0019915 GO:0048550 GO:0003057 |
| tr|A4D0W0|A4D0W0_ HUMAN | GO:0005654 GO:0017070 GO:0046540 GO:0005681 GO:0000398 GO:0005515 GO:0044822 |
| tr|A4D0Z3|A4D0Z3_ HUMAN | GO:0045176 GO:0005154 GO:0043197 GO:0060996 GO:0032587 GO:0043066 GO:0007612 GO:0031584 GO:0005525 GO:2000377 GO:0015031 GO:0045197 GO:0005794 GO:0007173 GO:0005829 GO:0061024 GO:0061512 GO:0006996 GO:0007420 GO:0007264 GO:0048678 GO:0006888 GO:0016021 GO:0045944 GO:0048471 GO:0070062 GO:0043687 GO:0003924 GO:0016477 GO:0018279 GO:0006471 |
| tr|A4D105|A4D105_ HUMAN | GO:0005654 GO:0005662 GO:0042127 GO:0000075 GO:0042769 GO:0005515 GO:0003684 GO:1900034 GO:0036297 GO:0000724 GO:0006283 GO:0006297 GO:0070911 GO:0003697 GO:0000722 GO:0033683 GO:0006284 GO:0000082 GO:0000084 GO:0032201 GO:0007346 GO:0070987 GO:0042276 GO:0006298 GO:0006271 |
| tr|A4D177|A4D177_ HUMAN | GO:1990226 GO:0048511 GO:0005637 GO:0000784 GO:0071549 GO:0005719 GO:0010369 GO:0005819 GO:0045892 GO:0019904 GO:0042802 GO:0035985 GO:0006338 GO:0000976 GO:0031618 GO:0000779 |
| tr|A4D1A1|A4D1A1_ HUMAN | GO:0007275 |
| tr|A4D1K0|A4D1K0_ HUMAN | GO:0046961 GO:0033572 GO:0060036 GO:0005515 GO:0008286 GO:0006879 GO:0033180 GO:0042624 GO:0015991 GO:0005829 GO:0045454 GO:0060041 GO:0016471 GO:0070062 |
| tr|A4D275|A4D275_ HUMAN | GO:0005925 GO:0007411 GO:0048013 GO:0034314 GO:0005829 GO:0007264 GO:0005885 GO:0038096 GO:0051015 GO:0070062 GO:0045087 GO:0005200 |
| tr|A4D2P1|A4D2P1_ HUMAN | GO:0006972 GO:0030742 GO:0038095 GO:0003676 GO:0019901 GO:0090502 GO:0051022 GO:0001934 GO:0010310 GO:0005525 GO:0002551 GO:0051668 GO:0051932 GO:0005789 GO:0003382 GO:0036464 GO:0008283 GO:0001891 GO:0045087 GO:1900026 GO:0017137 GO:0043065 GO:0051894 GO:1904948 GO:0005925 GO:0051496 GO:0071260 GO:0015031 GO:0010762 GO:0071526 GO:0005884 GO:0038096 GO:0002093 GO:0042470 GO:0006954 GO:0031996 GO:0043552 GO:0004522 GO:0060071 GO:0032587 GO:0030838 GO:0048010 GO:0048011 GO:0050690 GO:0070062 GO:0097190 GO:0048532 GO:0048261 GO:0001755 GO:0010592 GO:0042826 GO:0030168 GO:0005634 GO:0072659 GO:0005829 GO:0030027 GO:0031901 GO:0007411 GO:0048013 GO:0097178 GO:0007520 GO:0021831 GO:0090103 GO:0003924 GO:0045740 GO:0045453 GO:0060263 GO:0048873 GO:0090023 GO:0007186 GO:0016601 GO:0060297 GO:0016032 GO:0042074 GO:0048813 GO:0031295 GO:0021799 GO:0021894 GO:0008361 GO:0014041 GO:0000139 GO:0043652 GO:0031532 GO:0032707 GO:0035025 GO:0019897 GO:0005802 |
| tr|A4FTV9|A4FTV9_ HUMAN | GO:0000786 GO:0000790 GO:0070062 GO:0019899 GO:0008285 GO:0003677 GO:0006342 GO:0046982 |
| tr|A4FUT8|A4FUT8_ HUMAN | GO:0016568 GO:0072718 GO:0055114 GO:0098869 GO:0046872 GO:0052634 GO:0043734 GO:0006355 GO:0019798 GO:0000907 GO:0016209 GO:0052635 GO:0018602 GO:0005654 GO:0034792 |
| tr|A4FVC0|A4FVC0_ HUMAN | GO:0035068 GO:0048011 GO:0035278 GO:0008022 GO:0003743 GO:0035197 GO:0046872 GO:0005829 GO:0016442 GO:0003729 GO:0005654 GO:0009791 GO:0090502 GO:0090625 GO:0000340 GO:0010586 GO:0035280 GO:0005844 GO:0070578 GO:0035087 GO:0048015 GO:0045944 GO:0003725 GO:0031054 GO:0045087 GO:0003727 GO:0007219 GO:0090624 GO:0005739 GO:0007173 GO:0038095 GO:0030154 GO:0035198 GO:0000932 GO:0060213 GO:0010501 GO:0001047 GO:0030425 GO:0000993 GO:0035279 GO:0005845 GO:0008543 GO:0045947 GO:0016020 GO:0070551 |
| tr|A4ZPI7|A4ZPI7_ HUMAN | GO:0008584 GO:0014823 GO:0010560 GO:0010042 GO:0019903 GO:0010310 GO:0005525 GO:0032403 GO:0038083 GO:0071363 GO:0051425 GO:0030335 GO:0031995 GO:0000187 GO:0019087 GO:0045725 GO:0045840 GO:0031405 GO:0042593 GO:0045444 GO:0045821 GO:0045202 GO:0051290 GO:0051446 GO:0031017 GO:0008284 GO:0045995 GO:0031994 GO:0030325 GO:0043560 GO:0045429 GO:0043559 GO:0070062 GO:0010008 GO:0005899 GO:0005159 GO:2000194 GO:0043548 GO:0030238 GO:0005634 GO:0005829 GO:0043423 GO:0051897 GO:0046326 GO:0051384 GO:0045740 GO:0008544 GO:0003007 GO:0045471 GO:0005524 GO:0034612 GO:0007186 GO:0032148 GO:0010629 GO:0032355 GO:0033574 GO:0001933 GO:0005009 GO:0005901 GO:0033280 GO:0004716 GO:0008286 GO:0032410 GO:0048639 GO:0045893 GO:0060267 |
| tr|A5PKY0|A5PKY0_ HUMAN | GO:0030132 GO:0005509 GO:0042059 GO:0005515 GO:0006897 GO:0005634 |
| tr|A6N6J7|A6N6J7_ HUMAN | GO:0055114 GO:0034720 GO:0032453 GO:0008270 GO:0016706 GO:0032259 GO:0048511 GO:0003677 GO:0005654 GO:0008168 GO:0045892 |
| tr|A6NFX8|A6NFX8_ HUMAN | GO:0019303 GO:0005829 GO:0034656 GO:0050072 GO:0019144 GO:0009117 GO:0070062 GO:0017110 GO:0047631 GO:0000287 GO:0030515 GO:0005634 GO:0009191 |
| tr|A6NGP5|A6NGP5_ HUMAN | GO:0005737 GO:0005634 GO:0003677 GO:0005886 |
| tr|A6NMH8|A6NMH8_ HUMAN | GO:0005925 GO:0016324 GO:0005887 GO:0050776 GO:0001772 GO:0061462 GO:1990459 GO:0046813 GO:1904352 GO:0045944 GO:0050731 GO:0030890 GO:0070062 GO:0043128 GO:0030307 GO:0031623 GO:0007166 GO:2000145 GO:0046718 GO:0000187 GO:0071404 GO:0031647 GO:0009611 GO:0023026 |
| tr|A6QKW0|A6QKW0_ HUMAN | GO:0017056 GO:0005643 GO:0051292 GO:0005515 GO:0071786 GO:0042470 GO:0016021 GO:0005783 GO:0070062 GO:0034613 |
| tr|A7BI36|A7BI36_ HUMAN | GO:0006412 GO:0005840 GO:0030176 GO:0004872 GO:0044822 GO:0001649 GO:0007165 GO:0015031 |
| tr|A8E631|A8E631_ HUMAN | GO:0043231 GO:0005811 GO:0005737 GO:0005886 |
| tr|A8K088|A8K088_ HUMAN | GO:0005515 GO:0016020 GO:0031100 GO:0003743 GO:0016281 GO:0005829 GO:0019221 GO:0006446 GO:0070062 GO:0003725 GO:0016032 GO:0000339 GO:0000289 GO:0004004 GO:0005634 GO:0005524 GO:0010501 GO:0003729 |
| tr|A8K0B5|A8K0B5_ HUMAN | GO:0046872 GO:0005654 GO:0072669 GO:0006388 GO:0070062 |
| tr|A8K0F7|A8K0F7_ HUMAN | GO:0005789 GO:0042373 GO:0048038 GO:0034599 GO:0017187 GO:0047057 GO:0016021 GO:0055114 |
| tr|A8K0I0|A8K0I0_ HUMAN | GO:0005796 GO:0070740 GO:0043773 GO:0000413 GO:0043774 GO:0006457 GO:0000209 GO:0008766 GO:0070736 GO:0050900 GO:0072659 GO:0003755 GO:0070738 GO:0018169 GO:0005515 GO:0034450 GO:0005654 GO:0070737 GO:0007596 GO:0005886 |
| tr|A8K0T9|A8K0T9_ HUMAN | GO:0005829 GO:0006928 GO:0030863 GO:0051016 GO:0005903 GO:0051015 GO:0070062 GO:0045087 GO:0071203 GO:0005911 GO:0008290 GO:0016020 GO:0007596 |
| tr|A8K168|A8K168_ HUMAN | GO:1902031 GO:0030145 GO:0035497 GO:0051287 GO:0009725 GO:0001077 GO:0071837 GO:0090575 GO:0044255 GO:0050661 GO:0007156 GO:0009055 GO:0043531 GO:0000978 GO:0005509 GO:0009743 GO:0008948 GO:0004473 GO:0005829 GO:0046982 GO:0006108 GO:0004471 GO:0005975 GO:0045666 GO:0006741 GO:0000790 GO:0055114 GO:0045944 GO:0042803 GO:0051262 GO:0005886 GO:0043425 GO:0046332 GO:0005739 GO:0070888 |
| tr|A8K1F4|A8K1F4_ HUMAN | GO:0016020 GO:0006952 GO:0005737 GO:0005634 GO:0005515 |
| tr|A8K1R1|A8K1R1_ HUMAN | GO:0005789 GO:0007067 GO:0007084 GO:0016021 GO:0051301 GO:0005874 |
| tr|A8K274|A8K274_ HUMAN | GO:0002039 GO:1902254 GO:0016020 GO:0031398 GO:0043518 GO:0005737 GO:1903364 GO:0070062 GO:0006606 GO:0005634 |
| tr|A8K287|A8K287_ HUMAN | GO:0005925 GO:0016082 GO:0005654 GO:0017157 GO:0030659 GO:0005484 GO:0042581 GO:0015031 GO:0042582 GO:0031629 GO:0016021 GO:0006892 GO:0002553 GO:0043195 GO:0070062 GO:0006903 GO:0005886 GO:0042629 GO:0031201 GO:0019905 |
| tr|A8K2G0|A8K2G0_ HUMAN | GO:0030054 GO:0016021 GO:0006892 GO:0030672 GO:0030136 GO:0006887 GO:0043195 GO:0005802 GO:0019904 GO:0006897 GO:0055038 GO:0042589 GO:0005654 GO:0015031 |
| tr|A8K2I7|A8K2I7_ HUMAN | GO:0014047 GO:0001525 GO:0007186 GO:0043198 GO:0043542 GO:0012506 GO:0043197 GO:0005102 GO:0048845 GO:0030165 GO:0048844 GO:0032467 GO:0031647 GO:0006605 GO:0048167 GO:0017022 GO:0005829 GO:0003779 GO:0001946 GO:0030139 GO:0032435 GO:0005096 GO:0004707 GO:0005903 GO:0008021 GO:0030511 GO:0070062 GO:0042803 GO:0005938 |
| tr|A8K2L6|A8K2L6_ HUMAN | GO:0045121 GO:0005654 GO:0070382 GO:0042998 GO:0001786 GO:0016323 GO:0005615 GO:1901611 GO:0005509 GO:0031210 GO:0005544 GO:0030154 GO:0042997 GO:0070062 GO:0016324 |
| tr|A8K2M0|A8K2M0_ HUMAN | GO:0048011 GO:0051436 GO:0045899 GO:0008233 GO:0031595 GO:0008540 GO:0001824 GO:0002479 GO:0030433 GO:0090263 GO:0007411 GO:0048010 GO:0000186 GO:0006977 GO:0002223 GO:0051437 GO:0005654 GO:0006595 GO:0031145 GO:0031597 GO:0017025 GO:0038061 GO:0007265 GO:0090090 GO:0033209 GO:0016032 GO:0006521 GO:0036402 GO:0043488 GO:0000084 GO:0007173 GO:0005524 GO:0038095 GO:1901800 GO:0050852 GO:0016234 GO:0000090 GO:0043066 GO:0008543 GO:0000209 GO:0016020 GO:0008286 |
| tr|A8K2Q6|A8K2Q6_ HUMAN | GO:0005737 GO:0006457 GO:0005515 GO:0016018 GO:0000413 GO:0003755 GO:0070062 |
| tr|A8K2Q7|A8K2Q7_ HUMAN | GO:0006633 GO:0046872 GO:0008217 GO:0015645 GO:0005524 GO:0005759 GO:0047760 GO:0042632 |
| tr|A8K2R3|A8K2R3_ HUMAN | GO:0000790 GO:0051573 GO:0042162 GO:0001701 GO:0030851 GO:0051091 GO:1902166 GO:2000648 GO:0032091 GO:0043392 GO:0033169 GO:0045654 GO:0007596 GO:0005654 GO:0050660 GO:1990391 GO:0034648 GO:0002039 GO:0043433 GO:0000784 GO:0010725 GO:0016491 GO:0001085 GO:2000179 GO:0034644 GO:0055001 GO:0044212 GO:0071480 GO:0045944 GO:0043518 GO:0010569 GO:0050681 GO:0003700 GO:0007264 GO:0005667 GO:0055114 GO:0000122 GO:0003682 GO:0021983 GO:0030374 GO:0032454 GO:0043426 GO:0061752 GO:0045648 GO:0051572 GO:0034720 GO:1903827 GO:0046886 GO:0019899 GO:0033184 |
| tr|A8K2S7|A8K2S7_ HUMAN | GO:0016021 GO:1904153 GO:0070972 GO:0005515 GO:0005789 |
| tr|A8K3C5|A8K3C5_ HUMAN | GO:0003729 GO:0016607 GO:0006376 GO:0005685 GO:0003677 GO:0005515 GO:0071004 |
| tr|A8K3J5|A8K3J5_ HUMAN | GO:0048311 GO:0005741 GO:0008152 GO:0003924 |
| tr|A8K3M3|A8K3M3_ HUMAN | GO:0031410 GO:0035791 GO:0060397 GO:0016021 GO:1903896 GO:0031532 GO:0030971 GO:1902202 GO:0005829 GO:0031225 GO:0097443 GO:0060334 GO:0030168 GO:0060338 GO:0070373 GO:0030948 GO:0051721 GO:1990264 GO:2000646 GO:0005886 GO:0044822 GO:0005158 GO:0005769 GO:0008270 GO:1903898 GO:0043407 GO:0007257 GO:1902236 GO:0061098 GO:0046627 GO:0033157 GO:0006987 GO:0030100 GO:0004725 GO:0046875 GO:0098554 |
| tr|A8K3M9|A8K3M9_ HUMAN | GO:0006369 GO:0005730 GO:0031124 GO:0006376 GO:0044822 GO:0005515 GO:0048025 GO:0000166 GO:0006406 GO:0005654 |
| tr|A8K3S1|A8K3S1_ HUMAN | GO:0004342 GO:0007340 GO:0006091 GO:0046370 GO:0070062 GO:0006043 GO:0006002 GO:0005515 GO:0006044 GO:0005737 GO:0016787 |
| tr|A8K3S3|A8K3S3_ HUMAN | GO:0016020 GO:0000236 GO:0007080 GO:0051983 GO:0035371 GO:0008283 GO:0000777 GO:0051301 GO:0005881 GO:0005829 GO:0003777 GO:0019237 GO:0030951 GO:0019886 GO:0007264 GO:0007596 GO:0007019 GO:0000090 GO:0016887 GO:0007018 GO:0005871 GO:0005634 GO:0005524 GO:0051010 GO:0008152 |
| tr|A8K3Y8|A8K3Y8_ HUMAN | GO:0032886 GO:0016021 GO:0005829 GO:0042277 GO:0005634 GO:0005515 |
| tr|A8K3Z5|A8K3Z5_ HUMAN | GO:0031047 GO:0005654 GO:0007077 GO:0051028 GO:1900034 GO:0000166 GO:0005652 GO:0044615 GO:0031965 GO:0010827 GO:0005543 GO:0003697 GO:0006999 GO:0045111 GO:0006355 GO:0005487 GO:0019221 GO:0006607 GO:0005975 GO:0008033 GO:0044281 GO:0019083 GO:0016925 GO:0000088 GO:0055085 GO:0042803 GO:0043687 GO:0019054 GO:0005886 GO:0044613 |
| tr|A8K435|A8K435_ HUMAN | GO:0006541 GO:0009450 GO:0051287 GO:0051289 GO:0006083 GO:0007417 GO:0046459 GO:0005759 GO:0007269 GO:0042135 GO:0004777 GO:0009791 GO:0006105 GO:0031406 GO:0006650 GO:0004029 GO:0006681 GO:0006006 GO:0006749 GO:0006536 GO:0009013 GO:0042803 GO:0022904 GO:0006678 |
| tr|A8K4A1|A8K4A1_ HUMAN | GO:0005515 GO:0016180 GO:0032039 |
| tr|A8K4A8|A8K4A8_ HUMAN | GO:0050560 GO:0070145 GO:0005524 GO:0004815 GO:0005759 GO:0000049 GO:0042803 GO:0005654 |
| tr|A8K4M4|A8K4M4_ HUMAN | GO:0016021 GO:0031965 GO:0050291 GO:0003677 GO:0005515 GO:0046513 GO:0044281 GO:0005789 |
| tr|A8K4T6|A8K4T6_ HUMAN | GO:0048011 GO:0051436 GO:0008540 GO:0002479 GO:0090263 GO:0007411 GO:0048010 GO:0000186 GO:0006977 GO:0005829 GO:0002223 GO:0051437 GO:0005654 GO:0006595 GO:0031145 GO:0038061 GO:0007265 GO:0090090 GO:0033209 GO:0016032 GO:0006521 GO:0043488 GO:0005515 GO:0000084 GO:0007173 GO:0038095 GO:0050852 GO:0070682 GO:0000090 GO:0043066 GO:0008543 GO:0000209 GO:0008286 |
| tr|A8K4T9|A8K4T9_ HUMAN | GO:0000398 GO:0005515 GO:0071013 GO:0044822 |
| tr|A8K4U4|A8K4U4_ HUMAN | GO:0005737 GO:0022604 GO:0005515 GO:0030866 GO:0005634 GO:0070062 |
| tr|A8K4V2|A8K4V2_ HUMAN | GO:0005654 GO:0005759 GO:0008137 GO:0006120 GO:0032981 GO:0005747 GO:0050662 GO:1901006 GO:0032403 GO:0006814 |
| tr|A8K4W2|A8K4W2_ HUMAN | GO:0005654 GO:0005515 GO:0005759 GO:0046933 GO:0042776 GO:0043209 GO:0016021 GO:0021762 GO:0070062 GO:0000276 GO:0022904 |
| tr|A8K4Z4|A8K4Z4_ HUMAN | GO:0005925 GO:0000184 GO:0006415 GO:0006413 GO:0005515 GO:0071353 GO:0016020 GO:0044822 GO:0016259 GO:0006414 GO:0022625 GO:0030425 GO:0036464 GO:0003735 GO:0019083 GO:0006614 GO:0070062 GO:0005634 GO:0042254 |
| tr|A8K4Z6|A8K4Z6_ HUMAN | GO:0070274 GO:0000398 GO:0044822 GO:0005515 GO:0006406 |
| tr|A8K517|A8K517_ HUMAN | GO:0000184 GO:0006415 GO:0006413 GO:0005515 GO:0016020 GO:0044822 GO:0016259 GO:0006414 GO:0005730 GO:0045182 GO:0006417 GO:0003735 GO:0019083 GO:0006614 GO:0022627 |
| tr|A8K520|A8K520_ HUMAN | GO:0030170 GO:0070062 GO:0005739 |
| tr|A8K556|A8K556_ HUMAN | GO:0030659 GO:0004930 GO:0070062 GO:0005887 GO:0007175 GO:0005515 GO:0007186 |
| tr|A8K586|A8K586_ HUMAN | GO:0048490 GO:0005802 GO:0006829 GO:0030131 GO:0030742 GO:0030123 GO:0032438 GO:0005765 GO:0030665 GO:0007596 GO:0030424 GO:0019903 GO:0016021 GO:0048007 GO:0006622 GO:0051138 |
| tr|A8K5B0|A8K5B0_ HUMAN | GO:0045944 GO:0031307 GO:0005515 GO:0034613 |
| tr|A8K5D5|A8K5D5_ HUMAN | GO:0070126 GO:0005762 GO:0070125 GO:0031965 GO:0005743 GO:0003735 GO:0070124 |
| tr|A8K5D8|A8K5D8_ HUMAN | GO:0007033 GO:0042803 GO:0010971 GO:1903724 GO:1903542 GO:0032510 GO:0008022 GO:0007080 GO:0051261 GO:0031902 GO:0039702 GO:0005829 GO:0015031 GO:0000920 GO:0030301 GO:0006997 GO:1903543 GO:0036258 GO:0033993 GO:0000922 GO:0070062 GO:0005524 GO:0043162 GO:0008568 GO:0060548 GO:0006813 GO:1902188 GO:0061738 GO:1901673 GO:1904903 GO:0090611 GO:0090543 GO:0005634 GO:0005813 |
| tr|A8K5I0|A8K5I0_ HUMAN | GO:0005925 GO:0042026 GO:1901029 GO:0001618 GO:0051082 GO:1902380 GO:2001240 GO:0031625 GO:0044183 GO:1904722 GO:0072562 GO:1903265 GO:0005829 GO:0005654 GO:0031397 GO:0005814 GO:0070434 GO:0042623 GO:0048471 GO:0043488 GO:0005739 GO:0051092 GO:0001664 GO:0005524 GO:1902236 GO:0070370 GO:0090084 GO:0000151 GO:0050821 GO:1900034 GO:0016234 GO:0032757 GO:0046718 GO:0046034 GO:0055131 GO:0034599 GO:0031072 GO:0042826 |
| tr|A8K5J1|A8K5J1_ HUMAN | GO:0005829 GO:0007595 GO:0035690 GO:0044205 GO:0007565 GO:0004590 GO:0006207 GO:0005634 GO:0004588 |
| tr|A8K5Q1|A8K5Q1_ HUMAN | GO:0000122 GO:1900087 GO:0008276 GO:0016426 GO:0001510 GO:0044822 GO:0040031 GO:0016273 GO:0016433 GO:0016423 GO:0008171 GO:0035562 GO:0016427 GO:0016434 GO:0008169 |
| tr|A8K5S3|A8K5S3_ HUMAN | GO:0016023 GO:0002230 GO:0046745 GO:0048672 GO:0005793 GO:0090543 GO:0005829 GO:0098792 GO:0090541 GO:0005813 GO:0045184 GO:0009838 GO:0019076 GO:0032403 GO:0070062 GO:0098779 GO:0045862 GO:0000910 |
| tr|A8K5T7|A8K5T7_ HUMAN | GO:0005829 GO:0007067 GO:0045087 GO:0035872 GO:0031647 GO:0000776 GO:0005515 GO:0000151 GO:0043947 GO:0005634 |
| tr|A8K5U9|A8K5U9_ HUMAN | GO:0016188 GO:0030097 GO:0048813 GO:0048261 GO:1902963 GO:0032880 GO:0035615 GO:0035459 GO:0042734 GO:0072583 GO:0007409 GO:0048268 GO:0097459 GO:1902961 GO:0010629 GO:0048471 GO:0032050 GO:0030122 GO:0005794 GO:1901216 GO:0031623 GO:0045211 GO:0055072 GO:0045893 GO:0008283 GO:0005634 GO:0043025 GO:0005545 GO:0097418 GO:1902004 GO:0016197 |
| tr|A8K607|A8K607_ HUMAN | GO:0005737 GO:0005643 GO:0005049 GO:0008536 GO:0051028 GO:0006611 |
| tr|A8K651|A8K651_ HUMAN | GO:0043065 GO:0009986 GO:0097177 GO:0007597 GO:0005080 GO:0005540 GO:0006958 GO:0032689 GO:0031690 GO:0005829 GO:0001849 GO:0048025 GO:0003729 GO:0032695 GO:0008134 GO:0005759 GO:0005615 GO:0042256 GO:0030449 GO:0003714 GO:0005886 GO:0039534 GO:0016032 GO:0070131 GO:0000122 GO:0051897 GO:0014065 GO:0030984 GO:1901165 GO:0090023 GO:1900026 GO:2000510 GO:0039536 GO:0005730 |
| tr|A8K670|A8K670_ HUMAN | GO:0005829 GO:0000139 GO:0046209 GO:0004842 GO:0016567 GO:0044822 GO:0051001 GO:0005515 GO:0005654 GO:0044281 |
| tr|A8K6I4|A8K6I4_ HUMAN | GO:0000178 GO:0005730 GO:0006401 GO:0000398 GO:0005524 GO:0044822 GO:0005515 GO:0071013 GO:0000460 GO:0005654 GO:0003724 |
| tr|A8K6X3|A8K6X3_ HUMAN | GO:0010501 GO:0015030 GO:0004004 GO:0010468 GO:0000398 GO:0016607 GO:0005524 GO:0044822 GO:0016020 |
| tr|A8K718|A8K718_ HUMAN | GO:0042578 GO:0005515 GO:0043765 GO:0008152 GO:0005634 GO:0008270 GO:0044824 GO:0070062 |
| tr|A8K750|A8K750_ HUMAN | GO:0006810 GO:0008137 GO:0006120 GO:0009055 GO:0032981 GO:0043209 GO:0046872 GO:0051537 GO:0048738 GO:0005747 GO:0007399 |
| tr|A8K761|A8K761_ HUMAN | GO:0006810 GO:0008137 GO:0005747 GO:0070062 GO:0006120 GO:0005515 GO:0032981 |
| tr|A8K7B7|A8K7B7_ HUMAN | GO:0030111 GO:1903538 GO:0006461 GO:0051306 GO:0034047 GO:0007143 GO:0070262 GO:0019932 GO:0000159 GO:0005829 GO:0042518 GO:0008380 GO:0006672 GO:0000188 GO:0051232 GO:0051754 GO:0030155 GO:0070062 GO:0005739 GO:2001241 GO:0006275 GO:0004722 GO:0046982 GO:0008601 GO:0000775 GO:0000090 GO:0000086 GO:0000184 GO:0008543 GO:0005634 GO:0030308 GO:0003823 GO:0006355 GO:0015630 GO:0007084 GO:0016020 |
| tr|A8K7G2|A8K7G2_ HUMAN | GO:0005856 GO:0051082 GO:0005829 GO:0009635 GO:0040014 GO:0030900 GO:0005758 GO:1903146 GO:0006672 GO:1901215 GO:0031966 GO:0097194 GO:0016540 GO:0019742 GO:0034605 GO:0044257 GO:0007568 GO:0071300 GO:0048666 GO:2001241 GO:0007628 GO:0035458 GO:1902176 GO:0004252 GO:0008630 GO:0045786 GO:0071363 GO:0009898 GO:0000785 GO:0035631 GO:1903955 GO:0005634 GO:2001269 GO:0005789 |
| tr|A8K7J6|A8K7J6_ HUMAN | GO:0070126 GO:0005762 GO:0070125 GO:0005743 GO:0003735 GO:0070124 |
| tr|A8K7J7|A8K7J7_ HUMAN | GO:0045121 GO:0004340 GO:0005515 GO:0046835 GO:0072656 GO:1903599 GO:0051156 GO:0005536 GO:0008865 GO:0072655 GO:0005829 GO:0005741 GO:0015758 GO:0019158 GO:0097228 GO:0055085 GO:0061621 GO:0001678 GO:0005524 |
| tr|A8K7T4|A8K7T4_ HUMAN | GO:0031072 GO:0000139 GO:0046872 GO:0030137 GO:0005537 GO:0006890 GO:0001948 GO:0050766 GO:0070062 GO:0005615 GO:0009986 GO:0033116 GO:0005887 GO:0048471 GO:0005789 GO:0015031 |
| tr|A8K813|A8K813_ HUMAN | GO:0000139 GO:0036498 GO:0001077 GO:0015031 GO:0005789 GO:0003677 GO:0048208 GO:0016021 GO:0045944 GO:0043687 GO:0005634 GO:0018279 |
| tr|A8K818|A8K818_ HUMAN | GO:0003899 GO:0009303 GO:0000120 GO:0006362 GO:0045815 GO:0005739 GO:0005736 GO:0007169 GO:0044822 GO:0006363 GO:0005515 GO:0006361 GO:0005694 GO:0045814 GO:0005654 |
| tr|A8K878|A8K878_ HUMAN | GO:0002014 GO:0005615 GO:0008083 GO:0006986 GO:0005634 GO:0005783 GO:0048471 GO:0044822 |
| tr|A8K885|A8K885_ HUMAN | GO:0034713 GO:0042803 GO:0097422 GO:0035091 GO:0030512 GO:0045892 GO:1903593 GO:0042147 GO:0019898 GO:0034452 GO:0006886 GO:0007175 GO:0006897 GO:0046982 GO:0031901 GO:0016023 GO:0005634 GO:0016050 GO:0030905 |
| tr|A8K894|A8K894_ HUMAN | GO:0019013 GO:0003729 GO:0030529 GO:0033120 GO:0005515 GO:0000166 GO:0006397 GO:0016020 GO:0005634 |
| tr|A8K8B0|A8K8B0_ HUMAN | GO:0034047 GO:0008601 GO:0060561 GO:2001241 GO:0070062 GO:0006461 GO:0005515 GO:0045121 |
| tr|A8K8K1|A8K8K1_ HUMAN | GO:0005654 GO:0005524 GO:0006396 GO:0044822 GO:0003963 |
| tr|A8K8Q4|A8K8Q4_ HUMAN | GO:0005925 GO:0050769 GO:0005654 GO:0010165 GO:0010332 GO:0008022 GO:0007417 GO:0000012 GO:0033152 GO:0051102 GO:0000784 GO:0006297 GO:0051103 GO:0075713 GO:0005958 GO:0051301 GO:0005737 GO:0046872 GO:0006273 GO:0071897 GO:0071285 GO:0097680 GO:0033077 GO:0003677 GO:0000793 GO:2001252 GO:0035019 GO:0043524 GO:0033153 GO:0048146 GO:0002328 GO:0005886 GO:0003910 GO:0032807 GO:0005524 GO:0001701 GO:0007049 GO:0045190 |
| tr|A8K8U1|A8K8U1_ HUMAN | GO:0045899 GO:0005654 GO:0043086 GO:0016020 GO:0005794 GO:0031461 GO:0010265 GO:0016567 GO:0017025 GO:0030154 GO:0070062 |
| tr|A8K905|A8K905_ HUMAN | GO:0005730 GO:0007517 GO:0003682 GO:0045444 GO:0016607 GO:0019216 GO:0044822 |
| tr|A8K946|A8K946_ HUMAN | GO:0003723 GO:0043235 GO:0005845 GO:0005730 GO:0004482 GO:0036265 GO:0006370 GO:0005515 GO:0016032 GO:0005654 GO:0006366 |
| tr|A8K964|A8K964_ HUMAN | GO:0035145 GO:0000398 GO:0016607 GO:0016337 GO:0044822 GO:0071013 GO:0005198 GO:0005737 GO:0030057 GO:0005882 GO:0006351 GO:0005886 |
| tr|A8K972|A8K972_ HUMAN | GO:0030488 GO:0008757 GO:0008175 GO:0031591 GO:0005515 GO:0005829 |
| tr|A8K9D2|A8K9D2_ HUMAN | GO:0070126 GO:0070125 GO:0005743 GO:0035928 GO:0005761 GO:0003735 GO:0005615 GO:0070124 GO:0008097 |
| tr|A8K9K1|A8K9K1_ HUMAN | GO:0005887 GO:0005515 GO:0006874 GO:0031965 GO:0031095 GO:1903779 GO:0033017 GO:0046872 GO:0007596 GO:0005388 GO:0070588 GO:0005524 GO:0008152 |
| tr|A8K9K6|A8K9K6_ HUMAN | GO:0000154 GO:1990226 GO:0044822 GO:0030515 GO:0032040 GO:0070761 GO:0031428 GO:0005654 GO:0005737 GO:0016020 |
| tr|A8K9K8|A8K9K8_ HUMAN | GO:0034933 GO:0052666 GO:0009008 GO:0043803 GO:0043851 GO:0018707 GO:0018423 GO:0008172 GO:0031167 GO:0016435 GO:0016428 GO:0052909 GO:0005730 GO:0071424 GO:0034931 GO:0016205 GO:0009019 GO:0016279 GO:0043834 GO:0043776 GO:0052624 GO:0043777 GO:0034807 GO:0052667 GO:0043833 GO:0043780 GO:0009383 GO:0008174 GO:0030792 GO:0043827 GO:0051994 GO:0043791 GO:0004809 GO:0044822 GO:0000179 GO:0052735 GO:0034541 GO:0043852 GO:0052665 GO:0043770 GO:0043782 GO:0019702 GO:0008425 GO:0005737 GO:0080012 GO:0008650 GO:0070677 |
| tr|A8K9T2|A8K9T2_ HUMAN | GO:0005654 GO:0005515 GO:0080008 GO:0016567 |
| tr|A8K9T8|A8K9T8_ HUMAN | GO:0005829 GO:0006518 GO:0042277 GO:1902809 GO:0006111 GO:0046872 GO:0070012 GO:0004222 GO:0006508 GO:0005758 GO:0005886 |
| tr|A8K9T9|A8K9T9_ HUMAN | GO:0005829 GO:0006144 GO:0046872 GO:0006541 GO:0070062 GO:0005524 GO:0016740 GO:0042493 GO:0006189 GO:0004642 |
| tr|A8K9U0|A8K9U0_ HUMAN | GO:0046685 GO:0046822 GO:0035278 GO:0031016 GO:0010494 GO:0051149 GO:0045727 GO:0000381 GO:0035773 GO:0032055 GO:0003730 GO:0032922 GO:0097158 GO:0097167 GO:0000166 GO:0051403 GO:0048471 GO:0008270 GO:0043153 GO:0005515 GO:0035883 GO:0035198 GO:0046626 GO:0002192 GO:0005730 GO:0045947 GO:0016607 |
[truncated: 304,266 more chars]
