# Supplementary material for: EspF of Enterohemorrhagic Escherichia coli Enhances Apoptosis via Endoplasmic Reticulum Stress in Intestinal Epithelial Cells: An Isobaric Tags for Relative and Absolute Quantitation-Based Comparative Proteomic Analysis
Source: Front Microbiol. 2022 Jun 30;13:900919. doi: 10.3389/fmicb.2022.900919 (PMC9279134; doi:10.3389/fmicb.2022.900919)
Supplement: Supplementary file 4 [file Table_4.DOCX]

**Table S4a. The details of PPI interaction nodes in the Δ*espF* vs control group**

| **Node1-gene** | **Node2-gene** | **Node1-uniprotein** | **Node2-unprotein** | **Score** |
| --- | --- | --- | --- | --- |
| SSB | PRPF4 | tr\|B5BUB5\|B5BUB5_HUMAN | tr\|Q59EL4\|Q59EL4_HUMAN | 476 |
| SSB | EIF2S2 | tr\|B5BUB5\|B5BUB5_HUMAN | tr\|Q6IBR8\|Q6IBR8_HUMAN | 420 |
| PDXP | RNH1 | tr\|A0A024R1I3\|A0A024R1I3_HUMAN | tr\|A0A140VJT8\|A0A140VJT8_HUMAN | 416 |
| HSPE1 | CBX3 | sp\|P61604\|CH10_HUMAN | tr\|A4D177\|A4D177_HUMAN | 550 |
| HSPE1 | TSFM | sp\|P61604\|CH10_HUMAN | tr\|E5KS95\|E5KS95_HUMAN | 557 |
| HSPE1 | EL52 | sp\|P61604\|CH10_HUMAN | tr\|K9JA46\|K9JA46_HUMAN | 960 |
| HSPE1 | HEL-S-26 | sp\|P61604\|CH10_HUMAN | tr\|V9HWJ2\|V9HWJ2_HUMAN | 567 |
| HSPE1 | RPS2 | sp\|P61604\|CH10_HUMAN | sp\|P15880\|RS2_HUMAN | 421 |
| HSPE1 | HEL-S-162eP | sp\|P61604\|CH10_HUMAN | tr\|V9HVZ4\|V9HVZ4_HUMAN | 517 |
| HSPE1 | TRA1 | sp\|P61604\|CH10_HUMAN | tr\|Q5CAQ5\|Q5CAQ5_HUMAN | 894 |
| HSPE1 | DNAJB6 | sp\|P61604\|CH10_HUMAN | sp\|O75190\|DNJB6_HUMAN | 657 |
| HSPE1 | TOP2A | sp\|P61604\|CH10_HUMAN | sp\|P11388\|TOP2A_HUMAN | 596 |
| HSPE1 | PCNA | sp\|P61604\|CH10_HUMAN | sp\|P12004\|PCNA_HUMAN | 477 |
| HSPE1 | LYZ | sp\|P61604\|CH10_HUMAN | tr\|B2R4C5\|B2R4C5_HUMAN | 468 |
| HSPE1 | HEL-S-94n | sp\|P61604\|CH10_HUMAN | tr\|V9HW72\|V9HW72_HUMAN | 562 |
| HSPE1 | DNAJA1 | sp\|P61604\|CH10_HUMAN | sp\|P31689\|DNJA1_HUMAN | 661 |
| HSPE1 | TXNDC5 | sp\|P61604\|CH10_HUMAN | sp\|Q8NBS9\|TXND5_HUMAN | 401 |
| HSPE1 | ERP70 | sp\|P61604\|CH10_HUMAN | tr\|A0A090N8Y2\|A0A090N8Y2_HUMAN | 461 |
| TUBB | PCNA | tr\|Q5SU16\|Q5SU16_HUMAN | sp\|P12004\|PCNA_HUMAN | 653 |
| TUBB | EL52 | tr\|Q5SU16\|Q5SU16_HUMAN | tr\|K9JA46\|K9JA46_HUMAN | 546 |
| TUBB | HEL-S-162eP | tr\|Q5SU16\|Q5SU16_HUMAN | tr\|V9HVZ4\|V9HVZ4_HUMAN | 617 |
| TUBB | TUBA1C | tr\|Q5SU16\|Q5SU16_HUMAN | tr\|F5H5D3\|F5H5D3_HUMAN | 644 |
| TUBB | HIST2H3A | tr\|Q5SU16\|Q5SU16_HUMAN | sp\|Q71DI3\|H32_HUMAN | 501 |
| TUBB | TOP2A | tr\|Q5SU16\|Q5SU16_HUMAN | sp\|P11388\|TOP2A_HUMAN | 451 |
| RPLP0P6 | RPL18 | tr\|A8K4Z4\|A8K4Z4_HUMAN | tr\|J3QQ67\|J3QQ67_HUMAN | 999 |
| RPLP0P6 | RPS2 | tr\|A8K4Z4\|A8K4Z4_HUMAN | sp\|P15880\|RS2_HUMAN | 999 |
| RPLP0P6 | HEL-S-162eP | tr\|A8K4Z4\|A8K4Z4_HUMAN | tr\|V9HVZ4\|V9HVZ4_HUMAN | 700 |
| RPLP0P6 | HEL-S-68p | tr\|A8K4Z4\|A8K4Z4_HUMAN | tr\|V9HWF4\|V9HWF4_HUMAN | 454 |
| RPLP0P6 | RPL31 | tr\|A8K4Z4\|A8K4Z4_HUMAN | tr\|B2R4C1\|B2R4C1_HUMAN | 999 |
| RPLP0P6 | DNAJA1 | tr\|A8K4Z4\|A8K4Z4_HUMAN | sp\|P31689\|DNJA1_HUMAN | 591 |
| RPLP0P6 | RPS14 | tr\|A8K4Z4\|A8K4Z4_HUMAN | sp\|P62263\|RS14_HUMAN | 999 |
| RPLP0P6 | RPS12 | tr\|A8K4Z4\|A8K4Z4_HUMAN | sp\|P25398\|RS12_HUMAN | 999 |
| RPLP0P6 | DDX54 | tr\|A8K4Z4\|A8K4Z4_HUMAN | sp\|Q8TDD1\|DDX54_HUMAN | 522 |
| RPLP0P6 | RPL27A | tr\|A8K4Z4\|A8K4Z4_HUMAN | sp\|P46776\|RL27A_HUMAN | 999 |
| RPLP0P6 | EIF2S2 | tr\|A8K4Z4\|A8K4Z4_HUMAN | tr\|Q6IBR8\|Q6IBR8_HUMAN | 909 |
| RPLP0P6 | RPS13 | tr\|A8K4Z4\|A8K4Z4_HUMAN | sp\|P62277\|RS13_HUMAN | 999 |
| RPLP0P6 | HEL-S-310 | tr\|A8K4Z4\|A8K4Z4_HUMAN | tr\|V9HW01\|V9HW01_HUMAN | 999 |
| TOP2A | MSH6 | sp\|P11388\|TOP2A_HUMAN | sp\|P52701\|MSH6_HUMAN | 738 |
| TOP2A | TSFM | sp\|P11388\|TOP2A_HUMAN | tr\|E5KS95\|E5KS95_HUMAN | 619 |
| TOP2A | RPS2 | sp\|P11388\|TOP2A_HUMAN | sp\|P15880\|RS2_HUMAN | 617 |
| TOP2A | HEL-S-162eP | sp\|P11388\|TOP2A_HUMAN | tr\|V9HVZ4\|V9HVZ4_HUMAN | 723 |
| TOP2A | RPLP0P6 | sp\|P11388\|TOP2A_HUMAN | tr\|A8K4Z4\|A8K4Z4_HUMAN | 444 |
| TOP2A | PCNA | sp\|P11388\|TOP2A_HUMAN | sp\|P12004\|PCNA_HUMAN | 992 |
| TOP2A | DNAJA1 | sp\|P11388\|TOP2A_HUMAN | sp\|P31689\|DNJA1_HUMAN | 597 |
| TOP2A | TOP1 | sp\|P11388\|TOP2A_HUMAN | sp\|P11387\|TOP1_HUMAN | 998 |
| TOP2A | RPS14 | sp\|P11388\|TOP2A_HUMAN | sp\|P62263\|RS14_HUMAN | 626 |
| TOP2A | RPL27A | sp\|P11388\|TOP2A_HUMAN | sp\|P46776\|RL27A_HUMAN | 584 |
| TOP2A | TUBA1C | sp\|P11388\|TOP2A_HUMAN | tr\|F5H5D3\|F5H5D3_HUMAN | 450 |
| TOP2A | TXNDC5 | sp\|P11388\|TOP2A_HUMAN | sp\|Q8NBS9\|TXND5_HUMAN | 518 |
| TOP2A | RPS13 | sp\|P11388\|TOP2A_HUMAN | sp\|P62277\|RS13_HUMAN | 418 |
| TOP2A | ALYREF | sp\|P11388\|TOP2A_HUMAN | tr\|E9PB61\|E9PB61_HUMAN | 573 |
| TOP2A | EL52 | sp\|P11388\|TOP2A_HUMAN | tr\|K9JA46\|K9JA46_HUMAN | 648 |
| TOP2A | TPI1 | sp\|P11388\|TOP2A_HUMAN | sp\|P60174\|TPIS_HUMAN | 571 |
| TOP2A | TRA1 | sp\|P11388\|TOP2A_HUMAN | tr\|Q5CAQ5\|Q5CAQ5_HUMAN | 577 |
| TOP2A | MCM7 | sp\|P11388\|TOP2A_HUMAN | tr\|A0A0S2Z4A5\|A0A0S2Z4A5_HUMAN | 631 |
| TOP2A | HEL-S-68p | sp\|P11388\|TOP2A_HUMAN | tr\|V9HWF4\|V9HWF4_HUMAN | 419 |
| TOP2A | ERP44 | sp\|P11388\|TOP2A_HUMAN | sp\|Q9BS26\|ERP44_HUMAN | 518 |
| TOP2A | DNAJB6 | sp\|P11388\|TOP2A_HUMAN | sp\|O75190\|DNJB6_HUMAN | 563 |
| TOP2A | TPX2 | sp\|P11388\|TOP2A_HUMAN | tr\|B3KM90\|B3KM90_HUMAN | 606 |
| TOP2A | ERP70 | sp\|P11388\|TOP2A_HUMAN | tr\|A0A090N8Y2\|A0A090N8Y2_HUMAN | 519 |
| TOP2A | hCG_2024613 | sp\|P11388\|TOP2A_HUMAN | tr\|A0A024QZN2\|A0A024QZN2_HUMAN | 478 |
| DNAJA1 | HEL-S-94n | sp\|P31689\|DNJA1_HUMAN | tr\|V9HW72\|V9HW72_HUMAN | 925 |
| DNAJA1 | TRA1 | sp\|P31689\|DNJA1_HUMAN | tr\|Q5CAQ5\|Q5CAQ5_HUMAN | 945 |
| DNAJA1 | ERP70 | sp\|P31689\|DNJA1_HUMAN | tr\|A0A090N8Y2\|A0A090N8Y2_HUMAN | 500 |
| DNAJA1 | CANX | sp\|P31689\|DNJA1_HUMAN | sp\|P27824\|CALX_HUMAN | 686 |
| DNAJA1 | DNAJB6 | sp\|P31689\|DNJA1_HUMAN | sp\|O75190\|DNJB6_HUMAN | 535 |
| ACTR3 | PFN1 | tr\|A0A024RAI1\|A0A024RAI1_HUMAN | sp\|P07737\|PROF1_HUMAN | 863 |
| ACTR3 | ADD1 | tr\|A0A024RAI1\|A0A024RAI1_HUMAN | sp\|P35611\|ADDA_HUMAN | 710 |
| ACTR3 | TPM3 | tr\|A0A024RAI1\|A0A024RAI1_HUMAN | tr\|A0A0S2Z4G4\|A0A0S2Z4G4_HUMAN | 700 |
| ACTR3 | CTNNA1 | tr\|A0A024RAI1\|A0A024RAI1_HUMAN | sp\|P35221\|CTNA1_HUMAN | 632 |
| ACTR3 | TUBA1C | tr\|A0A024RAI1\|A0A024RAI1_HUMAN | tr\|F5H5D3\|F5H5D3_HUMAN | 505 |
| ACTR3 | VASP | tr\|A0A024RAI1\|A0A024RAI1_HUMAN | tr\|A0A0S2Z4N8\|A0A0S2Z4N8_HUMAN | 592 |
| ACTR3 | RPS4X | tr\|A0A024RAI1\|A0A024RAI1_HUMAN | tr\|B2R491\|B2R491_HUMAN | 769 |
| PRPF19 | SRRM2 | sp\|Q9UMS4\|PRP19_HUMAN | tr\|A0A140VK53\|A0A140VK53_HUMAN | 823 |
| PRPF19 | PRPF4 | sp\|Q9UMS4\|PRP19_HUMAN | tr\|Q59EL4\|Q59EL4_HUMAN | 988 |
| PRPF19 | SAE1 | sp\|Q9UMS4\|PRP19_HUMAN | tr\|B3KNJ4\|B3KNJ4_HUMAN | 491 |
| PRPF19 | HNRNPAB | sp\|Q9UMS4\|PRP19_HUMAN | tr\|D6RD18\|D6RD18_HUMAN | 534 |
| CDIPT | PI4K2A | tr\|Q53HA5\|Q53HA5_HUMAN | sp\|Q9BTU6\|P4K2A_HUMAN | 903 |
| CBX3 | BPTF | tr\|A4D177\|A4D177_HUMAN | tr\|F5GXF5\|F5GXF5_HUMAN | 406 |
| CBX3 | HEL-S-26 | tr\|A4D177\|A4D177_HUMAN | tr\|V9HWJ2\|V9HWJ2_HUMAN | 538 |
| CBX3 | TRIM28 | tr\|A4D177\|A4D177_HUMAN | sp\|Q13263\|TIF1B_HUMAN | 987 |
| CBX3 | HIST1H1B | tr\|A4D177\|A4D177_HUMAN | sp\|P16401\|H15_HUMAN | 431 |
| TMSB4X | ACTN4 | tr\|Q0P5N8\|Q0P5N8_HUMAN | tr\|A0A0S2Z3G9\|A0A0S2Z3G9_HUMAN | 900 |
| PFN1 | TMSB4X | sp\|P07737\|PROF1_HUMAN | tr\|Q0P5N8\|Q0P5N8_HUMAN | 489 |
| PFN1 | TPM3 | sp\|P07737\|PROF1_HUMAN | tr\|A0A0S2Z4G4\|A0A0S2Z4G4_HUMAN | 478 |
| PFN1 | TMSB10 | sp\|P07737\|PROF1_HUMAN | tr\|D6W5K2\|D6W5K2_HUMAN | 471 |
| PFN1 | TUBA1C | sp\|P07737\|PROF1_HUMAN | tr\|F5H5D3\|F5H5D3_HUMAN | 570 |
| PFN1 | VASP | sp\|P07737\|PROF1_HUMAN | tr\|A0A0S2Z4N8\|A0A0S2Z4N8_HUMAN | 935 |
| HNRNPAB | ALYREF | tr\|D6RD18\|D6RD18_HUMAN | tr\|E9PB61\|E9PB61_HUMAN | 528 |
| MCM7 | PCNA | tr\|A0A0S2Z4A5\|A0A0S2Z4A5_HUMAN | sp\|P12004\|PCNA_HUMAN | 998 |
| MCM7 | MSH6 | tr\|A0A0S2Z4A5\|A0A0S2Z4A5_HUMAN | sp\|P52701\|MSH6_HUMAN | 493 |
| MCM7 | SF3A1 | tr\|A0A0S2Z4A5\|A0A0S2Z4A5_HUMAN | tr\|A0A024R1K8\|A0A024R1K8_HUMAN | 545 |
| AARS | TRMU | sp\|P49588\|SYAC_HUMAN | sp\|O75648\|MTU1_HUMAN | 577 |
| AARS | RPS2 | sp\|P49588\|SYAC_HUMAN | sp\|P15880\|RS2_HUMAN | 438 |
| AARS | UBE1 | sp\|P49588\|SYAC_HUMAN | tr\|A0A024R1A3\|A0A024R1A3_HUMAN | 974 |
| PFDN6 | RPAP3 | tr\|Q5STK2\|Q5STK2_HUMAN | sp\|Q9H6T3\|RPAP3_HUMAN | 803 |
| DDX54 | HEL-S-70 | sp\|Q8TDD1\|DDX54_HUMAN | tr\|V9HW80\|V9HW80_HUMAN | 461 |
| RPS12 | RPL18 | sp\|P25398\|RS12_HUMAN | tr\|J3QQ67\|J3QQ67_HUMAN | 999 |
| RPS12 | RPS2 | sp\|P25398\|RS12_HUMAN | sp\|P15880\|RS2_HUMAN | 999 |
| RPS12 | BTF3 | sp\|P25398\|RS12_HUMAN | sp\|P20290\|BTF3_HUMAN | 579 |
| RPS12 | RPL31 | sp\|P25398\|RS12_HUMAN | tr\|B2R4C1\|B2R4C1_HUMAN | 999 |
| RPS12 | RPS14 | sp\|P25398\|RS12_HUMAN | sp\|P62263\|RS14_HUMAN | 999 |
| RPS12 | RPL27A | sp\|P25398\|RS12_HUMAN | sp\|P46776\|RL27A_HUMAN | 998 |
| RPS12 | EIF2S2 | sp\|P25398\|RS12_HUMAN | tr\|Q6IBR8\|Q6IBR8_HUMAN | 907 |
| RPS12 | RPS13 | sp\|P25398\|RS12_HUMAN | sp\|P62277\|RS13_HUMAN | 999 |
| RPS12 | HEL-S-310 | sp\|P25398\|RS12_HUMAN | tr\|V9HW01\|V9HW01_HUMAN | 999 |
| KPNA2 | PCNA | tr\|Q6NVW7\|Q6NVW7_HUMAN | sp\|P12004\|PCNA_HUMAN | 440 |
| KPNA2 | TPX2 | tr\|Q6NVW7\|Q6NVW7_HUMAN | tr\|B3KM90\|B3KM90_HUMAN | 915 |
| KPNA2 | PCYT1A | tr\|Q6NVW7\|Q6NVW7_HUMAN | tr\|C9JEJ2\|C9JEJ2_HUMAN | 801 |
| PYGB | TPI1 | sp\|P11216\|PYGB_HUMAN | sp\|P60174\|TPIS_HUMAN | 650 |
| PYGB | HEL-S-162eP | sp\|P11216\|PYGB_HUMAN | tr\|V9HVZ4\|V9HVZ4_HUMAN | 564 |
| PYGB | HEL-S-68p | sp\|P11216\|PYGB_HUMAN | tr\|V9HWF4\|V9HWF4_HUMAN | 589 |
| STMN1 | HEL-S-94n | tr\|B5BU83\|B5BU83_HUMAN | tr\|V9HW72\|V9HW72_HUMAN | 974 |
| STMN1 | MAPK13 | tr\|B5BU83\|B5BU83_HUMAN | tr\|Q6FHR4\|Q6FHR4_HUMAN | 900 |
| RNH1 | SCARB2 | tr\|A0A140VJT8\|A0A140VJT8_HUMAN | tr\|A0A024RDG6\|A0A024RDG6_HUMAN | 663 |
| RNH1 | VASP | tr\|A0A140VJT8\|A0A140VJT8_HUMAN | tr\|A0A0S2Z4N8\|A0A0S2Z4N8_HUMAN | 472 |
| PCYT1A | VAPB | tr\|C9JEJ2\|C9JEJ2_HUMAN | tr\|Q53XM7\|Q53XM7_HUMAN | 523 |
| ERP70 | ACTN4 | tr\|A0A090N8Y2\|A0A090N8Y2_HUMAN | tr\|A0A0S2Z3G9\|A0A0S2Z3G9_HUMAN | 528 |
| ERP70 | HEL-S-162eP | tr\|A0A090N8Y2\|A0A090N8Y2_HUMAN | tr\|V9HVZ4\|V9HVZ4_HUMAN | 532 |
| ERP70 | TRA1 | tr\|A0A090N8Y2\|A0A090N8Y2_HUMAN | tr\|Q5CAQ5\|Q5CAQ5_HUMAN | 993 |
| ERP70 | CANX | tr\|A0A090N8Y2\|A0A090N8Y2_HUMAN | sp\|P27824\|CALX_HUMAN | 872 |
| RPN1 | RPL18 | sp\|P04843\|RPN1_HUMAN | tr\|J3QQ67\|J3QQ67_HUMAN | 905 |
| RPN1 | RPS2 | sp\|P04843\|RPN1_HUMAN | sp\|P15880\|RS2_HUMAN | 903 |
| RPN1 | RPL15 | sp\|P04843\|RPN1_HUMAN | tr\|A0A024R2Q4\|A0A024R2Q4_HUMAN | 900 |
| RPN1 | RPLP0P6 | sp\|P04843\|RPN1_HUMAN | tr\|A8K4Z4\|A8K4Z4_HUMAN | 903 |
| RPN1 | RPL31 | sp\|P04843\|RPN1_HUMAN | tr\|B2R4C1\|B2R4C1_HUMAN | 900 |
| RPN1 | RPS14 | sp\|P04843\|RPN1_HUMAN | sp\|P62263\|RS14_HUMAN | 903 |
| RPN1 | RPS12 | sp\|P04843\|RPN1_HUMAN | sp\|P25398\|RS12_HUMAN | 900 |
| RPN1 | RPL27A | sp\|P04843\|RPN1_HUMAN | sp\|P46776\|RL27A_HUMAN | 900 |
| RPN1 | RPL6 | sp\|P04843\|RPN1_HUMAN | tr\|Q8N5Z7\|Q8N5Z7_HUMAN | 914 |
| RPN1 | RPS13 | sp\|P04843\|RPN1_HUMAN | sp\|P62277\|RS13_HUMAN | 900 |
| RPN1 | CANX | sp\|P04843\|RPN1_HUMAN | sp\|P27824\|CALX_HUMAN | 526 |
| RPN1 | RPS4X | sp\|P04843\|RPN1_HUMAN | tr\|B2R491\|B2R491_HUMAN | 902 |
| RPN1 | HEL-S-310 | sp\|P04843\|RPN1_HUMAN | tr\|V9HW01\|V9HW01_HUMAN | 900 |
| RPN1 | RPL19 | sp\|P04843\|RPN1_HUMAN | tr\|Q53G49\|Q53G49_HUMAN | 902 |
| HEL-S-162eP | HEL-S-26 | tr\|V9HVZ4\|V9HVZ4_HUMAN | tr\|V9HWJ2\|V9HWJ2_HUMAN | 624 |
| HEL-S-162eP | TPI1 | tr\|V9HVZ4\|V9HVZ4_HUMAN | sp\|P60174\|TPIS_HUMAN | 913 |
| HEL-S-162eP | RPS2 | tr\|V9HVZ4\|V9HVZ4_HUMAN | sp\|P15880\|RS2_HUMAN | 677 |
| HEL-S-162eP | TRA1 | tr\|V9HVZ4\|V9HVZ4_HUMAN | tr\|Q5CAQ5\|Q5CAQ5_HUMAN | 650 |
| HEL-S-162eP | JAK2 | tr\|V9HVZ4\|V9HVZ4_HUMAN | tr\|Q53EL3\|Q53EL3_HUMAN | 602 |
| HEL-S-162eP | HEL-S-68p | tr\|V9HVZ4\|V9HVZ4_HUMAN | tr\|V9HWF4\|V9HWF4_HUMAN | 988 |
| HEL-S-162eP | PCNA | tr\|V9HVZ4\|V9HVZ4_HUMAN | sp\|P12004\|PCNA_HUMAN | 952 |
| HEL-S-162eP | HEL-S-94n | tr\|V9HVZ4\|V9HVZ4_HUMAN | tr\|V9HW72\|V9HW72_HUMAN | 769 |
| HEL-S-162eP | RPL27A | tr\|V9HVZ4\|V9HVZ4_HUMAN | sp\|P46776\|RL27A_HUMAN | 597 |
| HEL-S-162eP | RPS13 | tr\|V9HVZ4\|V9HVZ4_HUMAN | sp\|P62277\|RS13_HUMAN | 431 |
| HEL-S-162eP | CANX | tr\|V9HVZ4\|V9HVZ4_HUMAN | sp\|P27824\|CALX_HUMAN | 599 |
| SRRM2 | ALYREF | tr\|A0A140VK53\|A0A140VK53_HUMAN | tr\|E9PB61\|E9PB61_HUMAN | 658 |
| PPP1R13L | JAK2 | tr\|A0A024R0Q5\|A0A024R0Q5_HUMAN | tr\|Q53EL3\|Q53EL3_HUMAN | 404 |
| SF3A1 | ACTN4 | tr\|A0A024R1K8\|A0A024R1K8_HUMAN | tr\|A0A0S2Z3G9\|A0A0S2Z3G9_HUMAN | 804 |
| SF3A1 | SRRM2 | tr\|A0A024R1K8\|A0A024R1K8_HUMAN | tr\|A0A140VK53\|A0A140VK53_HUMAN | 657 |
| SF3A1 | ALYREF | tr\|A0A024R1K8\|A0A024R1K8_HUMAN | tr\|E9PB61\|E9PB61_HUMAN | 900 |
| SF3A1 | PRPF4 | tr\|A0A024R1K8\|A0A024R1K8_HUMAN | tr\|Q59EL4\|Q59EL4_HUMAN | 994 |
| SF3A1 | PRPF19 | tr\|A0A024R1K8\|A0A024R1K8_HUMAN | sp\|Q9UMS4\|PRP19_HUMAN | 996 |
| SF3A1 | HNRNPL | tr\|A0A024R1K8\|A0A024R1K8_HUMAN | sp\|P14866\|HNRPL_HUMAN | 904 |
| CTNNA1 | ACTN4 | sp\|P35221\|CTNA1_HUMAN | tr\|A0A0S2Z3G9\|A0A0S2Z3G9_HUMAN | 956 |
| CTNNA1 | VASP | sp\|P35221\|CTNA1_HUMAN | tr\|A0A0S2Z4N8\|A0A0S2Z4N8_HUMAN | 915 |
| CTNNA1 | ZYX | sp\|P35221\|CTNA1_HUMAN | sp\|Q15942\|ZYX_HUMAN | 937 |
| EIF2S2 | RPL18 | tr\|Q6IBR8\|Q6IBR8_HUMAN | tr\|J3QQ67\|J3QQ67_HUMAN | 918 |
| EIF2S2 | RPS14 | tr\|Q6IBR8\|Q6IBR8_HUMAN | sp\|P62263\|RS14_HUMAN | 919 |
| EIF2S2 | RPL27A | tr\|Q6IBR8\|Q6IBR8_HUMAN | sp\|P46776\|RL27A_HUMAN | 907 |
| EIF2S2 | RPS2 | tr\|Q6IBR8\|Q6IBR8_HUMAN | sp\|P15880\|RS2_HUMAN | 907 |
| EIF2S2 | RPS13 | tr\|Q6IBR8\|Q6IBR8_HUMAN | sp\|P62277\|RS13_HUMAN | 919 |
| EIF2S2 | HEL-S-310 | tr\|Q6IBR8\|Q6IBR8_HUMAN | tr\|V9HW01\|V9HW01_HUMAN | 916 |
| EIF2S2 | RPL31 | tr\|Q6IBR8\|Q6IBR8_HUMAN | tr\|B2R4C1\|B2R4C1_HUMAN | 919 |
| TUBA1C | PCNA | tr\|F5H5D3\|F5H5D3_HUMAN | sp\|P12004\|PCNA_HUMAN | 477 |
| TUBA1C | PFDN6 | tr\|F5H5D3\|F5H5D3_HUMAN | tr\|Q5STK2\|Q5STK2_HUMAN | 403 |
| TUBA1C | EL52 | tr\|F5H5D3\|F5H5D3_HUMAN | tr\|K9JA46\|K9JA46_HUMAN | 579 |
| TUBA1C | LMNB2 | tr\|F5H5D3\|F5H5D3_HUMAN | sp\|Q03252\|LMNB2_HUMAN | 418 |
| TUBA1C | HEL-S-162eP | tr\|F5H5D3\|F5H5D3_HUMAN | tr\|V9HVZ4\|V9HVZ4_HUMAN | 775 |
| TUBA1C | STMN1 | tr\|F5H5D3\|F5H5D3_HUMAN | tr\|B5BU83\|B5BU83_HUMAN | 905 |
| TUBA1C | FLNB | tr\|F5H5D3\|F5H5D3_HUMAN | sp\|O75369\|FLNB_HUMAN | 669 |
| RPS13 | RPL18 | sp\|P62277\|RS13_HUMAN | tr\|J3QQ67\|J3QQ67_HUMAN | 999 |
| RPS13 | TPI1 | sp\|P62277\|RS13_HUMAN | sp\|P60174\|TPIS_HUMAN | 625 |
| RPS13 | RPS14 | sp\|P62277\|RS13_HUMAN | sp\|P62263\|RS14_HUMAN | 999 |
| RPS13 | RPL27A | sp\|P62277\|RS13_HUMAN | sp\|P46776\|RL27A_HUMAN | 999 |
| RPS13 | RPS2 | sp\|P62277\|RS13_HUMAN | sp\|P15880\|RS2_HUMAN | 999 |
| RPS13 | HEL-S-310 | sp\|P62277\|RS13_HUMAN | tr\|V9HW01\|V9HW01_HUMAN | 999 |
| RPS13 | BTF3 | sp\|P62277\|RS13_HUMAN | sp\|P20290\|BTF3_HUMAN | 585 |
| RPS13 | RPL31 | sp\|P62277\|RS13_HUMAN | tr\|B2R4C1\|B2R4C1_HUMAN | 999 |
| HEL-S-310 | RPL18 | tr\|V9HW01\|V9HW01_HUMAN | tr\|J3QQ67\|J3QQ67_HUMAN | 999 |
| HEL-S-310 | RPS14 | tr\|V9HW01\|V9HW01_HUMAN | sp\|P62263\|RS14_HUMAN | 999 |
| HEL-S-310 | RPL27A | tr\|V9HW01\|V9HW01_HUMAN | sp\|P46776\|RL27A_HUMAN | 999 |
| HEL-S-310 | RPS2 | tr\|V9HW01\|V9HW01_HUMAN | sp\|P15880\|RS2_HUMAN | 999 |
| HEL-S-310 | BTF3 | tr\|V9HW01\|V9HW01_HUMAN | sp\|P20290\|BTF3_HUMAN | 542 |
| HEL-S-310 | RPL31 | tr\|V9HW01\|V9HW01_HUMAN | tr\|B2R4C1\|B2R4C1_HUMAN | 999 |
| RPS4X | RPL15 | tr\|B2R491\|B2R491_HUMAN | tr\|A0A024R2Q4\|A0A024R2Q4_HUMAN | 999 |
| RPS4X | RPS2 | tr\|B2R491\|B2R491_HUMAN | sp\|P15880\|RS2_HUMAN | 999 |
| RPS4X | RPLP0P6 | tr\|B2R491\|B2R491_HUMAN | tr\|A8K4Z4\|A8K4Z4_HUMAN | 999 |
| RPS4X | EIF5A | tr\|B2R491\|B2R491_HUMAN | tr\|I3L504\|I3L504_HUMAN | 576 |
| RPS4X | HEL-S-94n | tr\|B2R491\|B2R491_HUMAN | tr\|V9HW72\|V9HW72_HUMAN | 424 |
| RPS4X | RPS14 | tr\|B2R491\|B2R491_HUMAN | sp\|P62263\|RS14_HUMAN | 999 |
| RPS4X | RPL6 | tr\|B2R491\|B2R491_HUMAN | tr\|Q8N5Z7\|Q8N5Z7_HUMAN | 999 |
| RPS4X | RPL27A | tr\|B2R491\|B2R491_HUMAN | sp\|P46776\|RL27A_HUMAN | 999 |
| RPS4X | EIF2S2 | tr\|B2R491\|B2R491_HUMAN | tr\|Q6IBR8\|Q6IBR8_HUMAN | 913 |
| RPS4X | RPS13 | tr\|B2R491\|B2R491_HUMAN | sp\|P62277\|RS13_HUMAN | 999 |
| RPS4X | HEL-S-310 | tr\|B2R491\|B2R491_HUMAN | tr\|V9HW01\|V9HW01_HUMAN | 999 |
| RPS4X | RPL19 | tr\|B2R491\|B2R491_HUMAN | tr\|Q53G49\|Q53G49_HUMAN | 999 |
| RPS4X | RPL18 | tr\|B2R491\|B2R491_HUMAN | tr\|J3QQ67\|J3QQ67_HUMAN | 999 |
| RPS4X | NPM1 | tr\|B2R491\|B2R491_HUMAN | tr\|A0A0S2Z491\|A0A0S2Z491_HUMAN | 589 |
| RPS4X | FBL | tr\|B2R491\|B2R491_HUMAN | sp\|P22087\|FBRL_HUMAN | 882 |
| RPS4X | PABPC1 | tr\|B2R491\|B2R491_HUMAN | tr\|A0A024R9C1\|A0A024R9C1_HUMAN | 966 |
| RPS4X | EEF1B2 | tr\|B2R491\|B2R491_HUMAN | tr\|A0A024R3W7\|A0A024R3W7_HUMAN | 782 |
| RPS4X | UBE1 | tr\|B2R491\|B2R491_HUMAN | tr\|A0A024R1A3\|A0A024R1A3_HUMAN | 440 |
| RPS4X | RPL31 | tr\|B2R491\|B2R491_HUMAN | tr\|B2R4C1\|B2R4C1_HUMAN | 999 |
| RPS4X | RPS12 | tr\|B2R491\|B2R491_HUMAN | sp\|P25398\|RS12_HUMAN | 999 |
| MBNL1 | HNRNPL | tr\|C9JP00\|C9JP00_HUMAN | sp\|P14866\|HNRPL_HUMAN | 672 |
| ACTN4 | HEL-S-70 | tr\|A0A0S2Z3G9\|A0A0S2Z3G9_HUMAN | tr\|V9HW80\|V9HW80_HUMAN | 503 |
| NPM1 | EL52 | tr\|A0A0S2Z491\|A0A0S2Z491_HUMAN | tr\|K9JA46\|K9JA46_HUMAN | 602 |
| NPM1 | HEL-S-26 | tr\|A0A0S2Z491\|A0A0S2Z491_HUMAN | tr\|V9HWJ2\|V9HWJ2_HUMAN | 644 |
| NPM1 | TPM3 | tr\|A0A0S2Z491\|A0A0S2Z491_HUMAN | tr\|A0A0S2Z4G4\|A0A0S2Z4G4_HUMAN | 536 |
| NPM1 | FBL | tr\|A0A0S2Z491\|A0A0S2Z491_HUMAN | sp\|P22087\|FBRL_HUMAN | 586 |
| NPM1 | HEL-S-162eP | tr\|A0A0S2Z491\|A0A0S2Z491_HUMAN | tr\|V9HVZ4\|V9HVZ4_HUMAN | 819 |
| NPM1 | JAK2 | tr\|A0A0S2Z491\|A0A0S2Z491_HUMAN | tr\|Q53EL3\|Q53EL3_HUMAN | 525 |
| NPM1 | TRIM28 | tr\|A0A0S2Z491\|A0A0S2Z491_HUMAN | sp\|Q13263\|TIF1B_HUMAN | 436 |
| NPM1 | ZYX | tr\|A0A0S2Z491\|A0A0S2Z491_HUMAN | sp\|Q15942\|ZYX_HUMAN | 675 |
| NPM1 | CDK5RAP3 | tr\|A0A0S2Z491\|A0A0S2Z491_HUMAN | sp\|Q96JB5\|CK5P3_HUMAN | 621 |
| NPM1 | DNAJA1 | tr\|A0A0S2Z491\|A0A0S2Z491_HUMAN | sp\|P31689\|DNJA1_HUMAN | 416 |
| NPM1 | TOP1 | tr\|A0A0S2Z491\|A0A0S2Z491_HUMAN | sp\|P11387\|TOP1_HUMAN | 654 |
| NPM1 | RPL6 | tr\|A0A0S2Z491\|A0A0S2Z491_HUMAN | tr\|Q8N5Z7\|Q8N5Z7_HUMAN | 593 |
| NPM1 | HEL-S-70 | tr\|A0A0S2Z491\|A0A0S2Z491_HUMAN | tr\|V9HW80\|V9HW80_HUMAN | 448 |
| NPM1 | HEL-S-310 | tr\|A0A0S2Z491\|A0A0S2Z491_HUMAN | tr\|V9HW01\|V9HW01_HUMAN | 633 |
| SAE1 | PCNA | tr\|B3KNJ4\|B3KNJ4_HUMAN | sp\|P12004\|PCNA_HUMAN | 530 |
| TRA1 | PCNA | tr\|Q5CAQ5\|Q5CAQ5_HUMAN | sp\|P12004\|PCNA_HUMAN | 727 |
| TRA1 | HEL-S-94n | tr\|Q5CAQ5\|Q5CAQ5_HUMAN | tr\|V9HW72\|V9HW72_HUMAN | 985 |
| TRA1 | HEL-S-70 | tr\|Q5CAQ5\|Q5CAQ5_HUMAN | tr\|V9HW80\|V9HW80_HUMAN | 559 |
| TRA1 | MAPK13 | tr\|Q5CAQ5\|Q5CAQ5_HUMAN | tr\|Q6FHR4\|Q6FHR4_HUMAN | 463 |
| TRA1 | CANX | tr\|Q5CAQ5\|Q5CAQ5_HUMAN | sp\|P27824\|CALX_HUMAN | 968 |
| TRA1 | DNAJB6 | tr\|Q5CAQ5\|Q5CAQ5_HUMAN | sp\|O75190\|DNJB6_HUMAN | 802 |
| HIST2H3A | BPTF | sp\|Q71DI3\|H32_HUMAN | tr\|F5GXF5\|F5GXF5_HUMAN | 550 |
| HIST2H3A | HIST1H2BK | sp\|Q71DI3\|H32_HUMAN | tr\|A0A024RCL8\|A0A024RCL8_HUMAN | 715 |
| HIST2H3A | HIST1H1B | sp\|Q71DI3\|H32_HUMAN | sp\|P16401\|H15_HUMAN | 446 |
| HEL-S-68p | TPI1 | tr\|V9HWF4\|V9HWF4_HUMAN | sp\|P60174\|TPIS_HUMAN | 997 |
| PABPC1 | RPL15 | tr\|A0A024R9C1\|A0A024R9C1_HUMAN | tr\|A0A024R2Q4\|A0A024R2Q4_HUMAN | 913 |
| PABPC1 | RPS2 | tr\|A0A024R9C1\|A0A024R9C1_HUMAN | sp\|P15880\|RS2_HUMAN | 926 |
| PABPC1 | SSB | tr\|A0A024R9C1\|A0A024R9C1_HUMAN | tr\|B5BUB5\|B5BUB5_HUMAN | 605 |
| PABPC1 | RPLP0P6 | tr\|A0A024R9C1\|A0A024R9C1_HUMAN | tr\|A8K4Z4\|A8K4Z4_HUMAN | 923 |
| PABPC1 | EIF5A | tr\|A0A024R9C1\|A0A024R9C1_HUMAN | tr\|I3L504\|I3L504_HUMAN | 458 |
| PABPC1 | RPS14 | tr\|A0A024R9C1\|A0A024R9C1_HUMAN | sp\|P62263\|RS14_HUMAN | 907 |
| PABPC1 | RPL6 | tr\|A0A024R9C1\|A0A024R9C1_HUMAN | tr\|Q8N5Z7\|Q8N5Z7_HUMAN | 914 |
| PABPC1 | RPL27A | tr\|A0A024R9C1\|A0A024R9C1_HUMAN | sp\|P46776\|RL27A_HUMAN | 909 |
| PABPC1 | EIF2S2 | tr\|A0A024R9C1\|A0A024R9C1_HUMAN | tr\|Q6IBR8\|Q6IBR8_HUMAN | 910 |
| PABPC1 | RPS13 | tr\|A0A024R9C1\|A0A024R9C1_HUMAN | sp\|P62277\|RS13_HUMAN | 911 |
| PABPC1 | HEL-S-310 | tr\|A0A024R9C1\|A0A024R9C1_HUMAN | tr\|V9HW01\|V9HW01_HUMAN | 906 |
| PABPC1 | RPL19 | tr\|A0A024R9C1\|A0A024R9C1_HUMAN | tr\|Q53G49\|Q53G49_HUMAN | 914 |
| PABPC1 | PSMB11 | tr\|A0A024R9C1\|A0A024R9C1_HUMAN | tr\|Q86U62\|Q86U62_HUMAN | 900 |
| PABPC1 | RPL18 | tr\|A0A024R9C1\|A0A024R9C1_HUMAN | tr\|J3QQ67\|J3QQ67_HUMAN | 907 |
| PABPC1 | ALYREF | tr\|A0A024R9C1\|A0A024R9C1_HUMAN | tr\|E9PB61\|E9PB61_HUMAN | 512 |
| PABPC1 | G3BP | tr\|A0A024R9C1\|A0A024R9C1_HUMAN | tr\|Q6FI03\|Q6FI03_HUMAN | 831 |
| PABPC1 | FBL | tr\|A0A024R9C1\|A0A024R9C1_HUMAN | sp\|P22087\|FBRL_HUMAN | 425 |
| PABPC1 | HEL-S-68p | tr\|A0A024R9C1\|A0A024R9C1_HUMAN | tr\|V9HWF4\|V9HWF4_HUMAN | 684 |
| PABPC1 | UBE1 | tr\|A0A024R9C1\|A0A024R9C1_HUMAN | tr\|A0A024R1A3\|A0A024R1A3_HUMAN | 454 |
| PABPC1 | RPL31 | tr\|A0A024R9C1\|A0A024R9C1_HUMAN | tr\|B2R4C1\|B2R4C1_HUMAN | 912 |
| PABPC1 | RPS12 | tr\|A0A024R9C1\|A0A024R9C1_HUMAN | sp\|P25398\|RS12_HUMAN | 906 |
| PABPC1 | MAPK13 | tr\|A0A024R9C1\|A0A024R9C1_HUMAN | tr\|Q6FHR4\|Q6FHR4_HUMAN | 449 |
| LPCAT1 | PTDSS1 | sp\|Q8NF37\|PCAT1_HUMAN | tr\|A8KAH1\|A8KAH1_HUMAN | 904 |
| LPCAT1 | SCAMP2 | sp\|Q8NF37\|PCAT1_HUMAN | tr\|Q6IBK3\|Q6IBK3_HUMAN | 404 |
| ZYX | ACTN4 | sp\|Q15942\|ZYX_HUMAN | tr\|A0A0S2Z3G9\|A0A0S2Z3G9_HUMAN | 948 |
| ZYX | PPIB | sp\|Q15942\|ZYX_HUMAN | sp\|P23284\|PPIB_HUMAN | 573 |
| ZYX | UBE2V1 | sp\|Q15942\|ZYX_HUMAN | sp\|Q13404\|UB2V1_HUMAN | 907 |
| ZYX | VASP | sp\|Q15942\|ZYX_HUMAN | tr\|A0A0S2Z4N8\|A0A0S2Z4N8_HUMAN | 999 |
| HNRNPL | ALYREF | sp\|P14866\|HNRPL_HUMAN | tr\|E9PB61\|E9PB61_HUMAN | 982 |
| HNRNPL | TOP1 | sp\|P14866\|HNRPL_HUMAN | sp\|P11387\|TOP1_HUMAN | 514 |
| HNRNPL | PRPF4 | sp\|P14866\|HNRPL_HUMAN | tr\|Q59EL4\|Q59EL4_HUMAN | 900 |
| LYZ | HEL-S-162eP | tr\|B2R4C5\|B2R4C5_HUMAN | tr\|V9HVZ4\|V9HVZ4_HUMAN | 502 |
| BPTF | JAK2 | tr\|F5GXF5\|F5GXF5_HUMAN | tr\|Q53EL3\|Q53EL3_HUMAN | 412 |
| LMNB2 | PCNA | sp\|Q03252\|LMNB2_HUMAN | sp\|P12004\|PCNA_HUMAN | 409 |
| SBDS | SRRM2 | tr\|A0A0S2Z5I7\|A0A0S2Z5I7_HUMAN | tr\|A0A140VK53\|A0A140VK53_HUMAN | 553 |
| SBDS | HEL-S-26 | tr\|A0A0S2Z5I7\|A0A0S2Z5I7_HUMAN | tr\|V9HWJ2\|V9HWJ2_HUMAN | 416 |
| SBDS | RPS14 | tr\|A0A0S2Z5I7\|A0A0S2Z5I7_HUMAN | sp\|P62263\|RS14_HUMAN | 733 |
| SBDS | RPL15 | tr\|A0A0S2Z5I7\|A0A0S2Z5I7_HUMAN | tr\|A0A024R2Q4\|A0A024R2Q4_HUMAN | 539 |
| SBDS | RPL6 | tr\|A0A0S2Z5I7\|A0A0S2Z5I7_HUMAN | tr\|Q8N5Z7\|Q8N5Z7_HUMAN | 408 |
| ASNS | TOP1 | sp\|P08243\|ASNS_HUMAN | sp\|P11387\|TOP1_HUMAN | 813 |
| ASNS | ERP70 | sp\|P08243\|ASNS_HUMAN | tr\|A0A090N8Y2\|A0A090N8Y2_HUMAN | 514 |
| ASNS | AARS | sp\|P08243\|ASNS_HUMAN | sp\|P49588\|SYAC_HUMAN | 983 |
| HIST1H1B | HIST1H2BK | sp\|P16401\|H15_HUMAN | tr\|A0A024RCL8\|A0A024RCL8_HUMAN | 403 |
| TSFM | PNPT1 | tr\|E5KS95\|E5KS95_HUMAN | sp\|Q8TCS8\|PNPT1_HUMAN | 504 |
| TSFM | TRMU | tr\|E5KS95\|E5KS95_HUMAN | sp\|O75648\|MTU1_HUMAN | 470 |
| TSFM | RPS2 | tr\|E5KS95\|E5KS95_HUMAN | sp\|P15880\|RS2_HUMAN | 908 |
| TSFM | HEL-S-162eP | tr\|E5KS95\|E5KS95_HUMAN | tr\|V9HVZ4\|V9HVZ4_HUMAN | 449 |
| TSFM | RPLP0P6 | tr\|E5KS95\|E5KS95_HUMAN | tr\|A8K4Z4\|A8K4Z4_HUMAN | 697 |
| TSFM | EEF1B2 | tr\|E5KS95\|E5KS95_HUMAN | tr\|A0A024R3W7\|A0A024R3W7_HUMAN | 535 |
| TSFM | AARS | tr\|E5KS95\|E5KS95_HUMAN | sp\|P49588\|SYAC_HUMAN | 546 |
| TSFM | RPS14 | tr\|E5KS95\|E5KS95_HUMAN | sp\|P62263\|RS14_HUMAN | 655 |
| TSFM | RPL27A | tr\|E5KS95\|E5KS95_HUMAN | sp\|P46776\|RL27A_HUMAN | 705 |
| TSFM | RPS13 | tr\|E5KS95\|E5KS95_HUMAN | sp\|P62277\|RS13_HUMAN | 535 |
| MSH6 | PCNA | sp\|P52701\|MSH6_HUMAN | sp\|P12004\|PCNA_HUMAN | 999 |
| MSH6 | EL52 | sp\|P52701\|MSH6_HUMAN | tr\|K9JA46\|K9JA46_HUMAN | 430 |
| MSH6 | TOP1 | sp\|P52701\|MSH6_HUMAN | sp\|P11387\|TOP1_HUMAN | 501 |
| PNPT1 | SRRM2 | sp\|Q8TCS8\|PNPT1_HUMAN | tr\|A0A140VK53\|A0A140VK53_HUMAN | 473 |
| PNPT1 | ALYREF | sp\|Q8TCS8\|PNPT1_HUMAN | tr\|E9PB61\|E9PB61_HUMAN | 565 |
| PNPT1 | G3BP | sp\|Q8TCS8\|PNPT1_HUMAN | tr\|Q6FI03\|Q6FI03_HUMAN | 585 |
| PNPT1 | AK4 | sp\|Q8TCS8\|PNPT1_HUMAN | sp\|P27144\|KAD4_HUMAN | 911 |
| PNPT1 | RPS2 | sp\|Q8TCS8\|PNPT1_HUMAN | sp\|P15880\|RS2_HUMAN | 625 |
| PNPT1 | RPS13 | sp\|Q8TCS8\|PNPT1_HUMAN | sp\|P62277\|RS13_HUMAN | 720 |
| PNPT1 | PRPF19 | sp\|Q8TCS8\|PNPT1_HUMAN | sp\|Q9UMS4\|PRP19_HUMAN | 738 |
| TPM3 | ACTN4 | tr\|A0A0S2Z4G4\|A0A0S2Z4G4_HUMAN | tr\|A0A0S2Z3G9\|A0A0S2Z3G9_HUMAN | 693 |
| TPM3 | EL52 | tr\|A0A0S2Z4G4\|A0A0S2Z4G4_HUMAN | tr\|K9JA46\|K9JA46_HUMAN | 759 |
| TPM3 | HEL-S-162eP | tr\|A0A0S2Z4G4\|A0A0S2Z4G4_HUMAN | tr\|V9HVZ4\|V9HVZ4_HUMAN | 403 |
| TPM3 | MYO18A | tr\|A0A0S2Z4G4\|A0A0S2Z4G4_HUMAN | tr\|A0A0D9SFK2\|A0A0D9SFK2_HUMAN | 603 |
| RPS2 | RPL18 | sp\|P15880\|RS2_HUMAN | tr\|J3QQ67\|J3QQ67_HUMAN | 999 |
| RPS2 | TPI1 | sp\|P15880\|RS2_HUMAN | sp\|P60174\|TPIS_HUMAN | 461 |
| RPS2 | RPS14 | sp\|P15880\|RS2_HUMAN | sp\|P62263\|RS14_HUMAN | 999 |
| RPS2 | RPL27A | sp\|P15880\|RS2_HUMAN | sp\|P46776\|RL27A_HUMAN | 999 |
| RPS2 | BTF3 | sp\|P15880\|RS2_HUMAN | sp\|P20290\|BTF3_HUMAN | 597 |
| RPS2 | RPL31 | sp\|P15880\|RS2_HUMAN | tr\|B2R4C1\|B2R4C1_HUMAN | 998 |
| FDPS | HEL-S-26 | sp\|P14324\|FPPS_HUMAN | tr\|V9HWJ2\|V9HWJ2_HUMAN | 578 |
| PCNA | UBE1 | sp\|P12004\|PCNA_HUMAN | tr\|A0A024R1A3\|A0A024R1A3_HUMAN | 438 |
| TOP1 | PCNA | sp\|P11387\|TOP1_HUMAN | sp\|P12004\|PCNA_HUMAN | 844 |
| TOP1 | EL52 | sp\|P11387\|TOP1_HUMAN | tr\|K9JA46\|K9JA46_HUMAN | 449 |
| TOP1 | RPS14 | sp\|P11387\|TOP1_HUMAN | sp\|P62263\|RS14_HUMAN | 545 |
| TOP1 | FBL | sp\|P11387\|TOP1_HUMAN | sp\|P22087\|FBRL_HUMAN | 713 |
| TOP1 | HEL-S-162eP | sp\|P11387\|TOP1_HUMAN | tr\|V9HVZ4\|V9HVZ4_HUMAN | 418 |
| RPS14 | RPL18 | sp\|P62263\|RS14_HUMAN | tr\|J3QQ67\|J3QQ67_HUMAN | 999 |
| RPS14 | RPL27A | sp\|P62263\|RS14_HUMAN | sp\|P46776\|RL27A_HUMAN | 999 |
| RPS14 | BTF3 | sp\|P62263\|RS14_HUMAN | sp\|P20290\|BTF3_HUMAN | 535 |
| RPS14 | RPL31 | sp\|P62263\|RS14_HUMAN | tr\|B2R4C1\|B2R4C1_HUMAN | 999 |
| AK4 | RPS14 | sp\|P27144\|KAD4_HUMAN | sp\|P62263\|RS14_HUMAN | 600 |
| AK4 | RPL27A | sp\|P27144\|KAD4_HUMAN | sp\|P46776\|RL27A_HUMAN | 680 |
| AK4 | RPS2 | sp\|P27144\|KAD4_HUMAN | sp\|P15880\|RS2_HUMAN | 600 |
| ADD1 | HEL-S-66 | sp\|P35611\|ADDA_HUMAN | tr\|V9HW69\|V9HW69_HUMAN | 729 |
| RPL27A | RPL18 | sp\|P46776\|RL27A_HUMAN | tr\|J3QQ67\|J3QQ67_HUMAN | 999 |
| RPL27A | BTF3 | sp\|P46776\|RL27A_HUMAN | sp\|P20290\|BTF3_HUMAN | 546 |
| RPL27A | RPL31 | sp\|P46776\|RL27A_HUMAN | tr\|B2R4C1\|B2R4C1_HUMAN | 999 |
| RPL6 | RPL15 | tr\|Q8N5Z7\|Q8N5Z7_HUMAN | tr\|A0A024R2Q4\|A0A024R2Q4_HUMAN | 999 |
| RPL6 | RPS2 | tr\|Q8N5Z7\|Q8N5Z7_HUMAN | sp\|P15880\|RS2_HUMAN | 999 |
| RPL6 | RPLP0P6 | tr\|Q8N5Z7\|Q8N5Z7_HUMAN | tr\|A8K4Z4\|A8K4Z4_HUMAN | 999 |
| RPL6 | EIF5A | tr\|Q8N5Z7\|Q8N5Z7_HUMAN | tr\|I3L504\|I3L504_HUMAN | 521 |
| RPL6 | RPS14 | tr\|Q8N5Z7\|Q8N5Z7_HUMAN | sp\|P62263\|RS14_HUMAN | 999 |
| RPL6 | RPL27A | tr\|Q8N5Z7\|Q8N5Z7_HUMAN | sp\|P46776\|RL27A_HUMAN | 999 |
| RPL6 | EIF2S2 | tr\|Q8N5Z7\|Q8N5Z7_HUMAN | tr\|Q6IBR8\|Q6IBR8_HUMAN | 923 |
| RPL6 | RPS13 | tr\|Q8N5Z7\|Q8N5Z7_HUMAN | sp\|P62277\|RS13_HUMAN | 999 |
| RPL6 | HEL-S-310 | tr\|Q8N5Z7\|Q8N5Z7_HUMAN | tr\|V9HW01\|V9HW01_HUMAN | 999 |
| RPL6 | RPL19 | tr\|Q8N5Z7\|Q8N5Z7_HUMAN | tr\|Q53G49\|Q53G49_HUMAN | 999 |
| RPL6 | RPL18 | tr\|Q8N5Z7\|Q8N5Z7_HUMAN | tr\|J3QQ67\|J3QQ67_HUMAN | 999 |
| RPL6 | FBL | tr\|Q8N5Z7\|Q8N5Z7_HUMAN | sp\|P22087\|FBRL_HUMAN | 872 |
| RPL6 | BTF3 | tr\|Q8N5Z7\|Q8N5Z7_HUMAN | sp\|P20290\|BTF3_HUMAN | 587 |
| RPL6 | EEF1B2 | tr\|Q8N5Z7\|Q8N5Z7_HUMAN | tr\|A0A024R3W7\|A0A024R3W7_HUMAN | 783 |
| RPL6 | RPL31 | tr\|Q8N5Z7\|Q8N5Z7_HUMAN | tr\|B2R4C1\|B2R4C1_HUMAN | 999 |
| RPL6 | RPS12 | tr\|Q8N5Z7\|Q8N5Z7_HUMAN | sp\|P25398\|RS12_HUMAN | 998 |
| TXNDC5 | EL52 | sp\|Q8NBS9\|TXND5_HUMAN | tr\|K9JA46\|K9JA46_HUMAN | 815 |
| TXNDC5 | TRA1 | sp\|Q8NBS9\|TXND5_HUMAN | tr\|Q5CAQ5\|Q5CAQ5_HUMAN | 628 |
| TXNDC5 | PPIB | sp\|Q8NBS9\|TXND5_HUMAN | sp\|P23284\|PPIB_HUMAN | 430 |
| TXNDC5 | PRPF4 | sp\|Q8NBS9\|TXND5_HUMAN | tr\|Q59EL4\|Q59EL4_HUMAN | 644 |
| TXNDC5 | UBE2V1 | sp\|Q8NBS9\|TXND5_HUMAN | sp\|Q13404\|UB2V1_HUMAN | 552 |
| TXNDC5 | HEL-S-70 | sp\|Q8NBS9\|TXND5_HUMAN | tr\|V9HW80\|V9HW80_HUMAN | 781 |
| TXNDC5 | ERP70 | sp\|Q8NBS9\|TXND5_HUMAN | tr\|A0A090N8Y2\|A0A090N8Y2_HUMAN | 486 |
| TXNDC5 | CANX | sp\|Q8NBS9\|TXND5_HUMAN | sp\|P27824\|CALX_HUMAN | 638 |
| CANX | HEL-S-70 | sp\|P27824\|CALX_HUMAN | tr\|V9HW80\|V9HW80_HUMAN | 977 |
| FDXR | SCP2 | tr\|B4DDI9\|B4DDI9_HUMAN | tr\|B2R761\|B2R761_HUMAN | 758 |
| ANXA1 | RPS14 | tr\|Q5TZZ9\|Q5TZZ9_HUMAN | sp\|P62263\|RS14_HUMAN | 576 |
| ANXA1 | HEL-S-162eP | tr\|Q5TZZ9\|Q5TZZ9_HUMAN | tr\|V9HVZ4\|V9HVZ4_HUMAN | 442 |
| ANXA1 | RPLP0P6 | tr\|Q5TZZ9\|Q5TZZ9_HUMAN | tr\|A8K4Z4\|A8K4Z4_HUMAN | 620 |
| HEL-S-22 | PCNA | tr\|V9HWE9\|V9HWE9_HUMAN | sp\|P12004\|PCNA_HUMAN | 472 |
| HEL-S-22 | HEL-S-162eP | tr\|V9HWE9\|V9HWE9_HUMAN | tr\|V9HVZ4\|V9HVZ4_HUMAN | 424 |
| KRT19 | PCNA | sp\|P08727\|K1C19_HUMAN | sp\|P12004\|PCNA_HUMAN | 537 |
| KRT19 | HEL-S-162eP | sp\|P08727\|K1C19_HUMAN | tr\|V9HVZ4\|V9HVZ4_HUMAN | 504 |
| G3BP | JAK2 | tr\|Q6FI03\|Q6FI03_HUMAN | tr\|Q53EL3\|Q53EL3_HUMAN | 575 |
| G3BP | FLNB | tr\|Q6FI03\|Q6FI03_HUMAN | sp\|O75369\|FLNB_HUMAN | 740 |
| TPI1 | HEL-S-26 | sp\|P60174\|TPIS_HUMAN | tr\|V9HWJ2\|V9HWJ2_HUMAN | 420 |
| GLRX3 | HEL-S-26 | tr\|A0A140VJK1\|A0A140VJK1_HUMAN | tr\|V9HWJ2\|V9HWJ2_HUMAN | 546 |
| EEA1 | HEL-S-70 | sp\|Q15075\|EEA1_HUMAN | tr\|V9HW80\|V9HW80_HUMAN | 598 |
| EEA1 | CANX | sp\|Q15075\|EEA1_HUMAN | sp\|P27824\|CALX_HUMAN | 567 |
| BTF3 | RPL18 | sp\|P20290\|BTF3_HUMAN | tr\|J3QQ67\|J3QQ67_HUMAN | 586 |
| BTF3 | RPL31 | sp\|P20290\|BTF3_HUMAN | tr\|B2R4C1\|B2R4C1_HUMAN | 773 |
| ERP44 | HEL-S-162eP | sp\|Q9BS26\|ERP44_HUMAN | tr\|V9HVZ4\|V9HVZ4_HUMAN | 425 |
| ERP44 | GLRX3 | sp\|Q9BS26\|ERP44_HUMAN | tr\|A0A140VJK1\|A0A140VJK1_HUMAN | 406 |
| ERP44 | TRA1 | sp\|Q9BS26\|ERP44_HUMAN | tr\|Q5CAQ5\|Q5CAQ5_HUMAN | 616 |
| ERP44 | CANX | sp\|Q9BS26\|ERP44_HUMAN | sp\|P27824\|CALX_HUMAN | 600 |
| RPL31 | RPL18 | tr\|B2R4C1\|B2R4C1_HUMAN | tr\|J3QQ67\|J3QQ67_HUMAN | 999 |
| AGPS | SCP2 | sp\|O00116\|ADAS_HUMAN | tr\|B2R761\|B2R761_HUMAN | 852 |
| PRPF4 | ALYREF | tr\|Q59EL4\|Q59EL4_HUMAN | tr\|E9PB61\|E9PB61_HUMAN | 961 |
| SCP2 | SRRM2 | tr\|B2R761\|B2R761_HUMAN | tr\|A0A140VK53\|A0A140VK53_HUMAN | 551 |
| S100A6 | HEL-S-162eP | sp\|P06703\|S10A6_HUMAN | tr\|V9HVZ4\|V9HVZ4_HUMAN | 625 |
| FLNB | HEL-S-70 | sp\|O75369\|FLNB_HUMAN | tr\|V9HW80\|V9HW80_HUMAN | 708 |
| MYO18A | ACTN4 | tr\|A0A0D9SFK2\|A0A0D9SFK2_HUMAN | tr\|A0A0S2Z3G9\|A0A0S2Z3G9_HUMAN | 507 |
| MYO18A | EL52 | tr\|A0A0D9SFK2\|A0A0D9SFK2_HUMAN | tr\|K9JA46\|K9JA46_HUMAN | 740 |
| HEL-S-26 | JAK2 | tr\|V9HWJ2\|V9HWJ2_HUMAN | tr\|Q53EL3\|Q53EL3_HUMAN | 518 |
| RPL15 | RPS2 | tr\|A0A024R2Q4\|A0A024R2Q4_HUMAN | sp\|P15880\|RS2_HUMAN | 999 |
| RPL15 | RPLP0P6 | tr\|A0A024R2Q4\|A0A024R2Q4_HUMAN | tr\|A8K4Z4\|A8K4Z4_HUMAN | 999 |
| RPL15 | EIF5A | tr\|A0A024R2Q4\|A0A024R2Q4_HUMAN | tr\|I3L504\|I3L504_HUMAN | 977 |
| RPL15 | RPS14 | tr\|A0A024R2Q4\|A0A024R2Q4_HUMAN | sp\|P62263\|RS14_HUMAN | 999 |
| RPL15 | RPL27A | tr\|A0A024R2Q4\|A0A024R2Q4_HUMAN | sp\|P46776\|RL27A_HUMAN | 999 |
| RPL15 | EIF2S2 | tr\|A0A024R2Q4\|A0A024R2Q4_HUMAN | tr\|Q6IBR8\|Q6IBR8_HUMAN | 925 |
| RPL15 | RPS13 | tr\|A0A024R2Q4\|A0A024R2Q4_HUMAN | sp\|P62277\|RS13_HUMAN | 999 |
| RPL15 | HEL-S-310 | tr\|A0A024R2Q4\|A0A024R2Q4_HUMAN | tr\|V9HW01\|V9HW01_HUMAN | 999 |
| RPL15 | RPL19 | tr\|A0A024R2Q4\|A0A024R2Q4_HUMAN | tr\|Q53G49\|Q53G49_HUMAN | 999 |
| RPL15 | RPL18 | tr\|A0A024R2Q4\|A0A024R2Q4_HUMAN | tr\|J3QQ67\|J3QQ67_HUMAN | 999 |
| RPL15 | FBL | tr\|A0A024R2Q4\|A0A024R2Q4_HUMAN | sp\|P22087\|FBRL_HUMAN | 966 |
| RPL15 | BTF3 | tr\|A0A024R2Q4\|A0A024R2Q4_HUMAN | sp\|P20290\|BTF3_HUMAN | 712 |
| RPL15 | EEF1B2 | tr\|A0A024R2Q4\|A0A024R2Q4_HUMAN | tr\|A0A024R3W7\|A0A024R3W7_HUMAN | 894 |
| RPL15 | RPL31 | tr\|A0A024R2Q4\|A0A024R2Q4_HUMAN | tr\|B2R4C1\|B2R4C1_HUMAN | 999 |
| RPL15 | RPS12 | tr\|A0A024R2Q4\|A0A024R2Q4_HUMAN | sp\|P25398\|RS12_HUMAN | 999 |
| RPL15 | DDX54 | tr\|A0A024R2Q4\|A0A024R2Q4_HUMAN | sp\|Q8TDD1\|DDX54_HUMAN | 503 |
| VAPB | HEL-S-70 | tr\|Q53XM7\|Q53XM7_HUMAN | tr\|V9HW80\|V9HW80_HUMAN | 865 |
| 2-Sep | SRRM2 | sp\|Q15019\|SEPT2_HUMAN | tr\|A0A140VK53\|A0A140VK53_HUMAN | 622 |
| 2-Sep | 7-Sep | sp\|Q15019\|SEPT2_HUMAN | tr\|Q8TC62\|Q8TC62_HUMAN | 996 |
| hCG_1990625 | EL52 | tr\|A0A140VKA6\|A0A140VKA6_HUMAN | tr\|K9JA46\|K9JA46_HUMAN | 891 |
| EIF5A | RPL18 | tr\|I3L504\|I3L504_HUMAN | tr\|J3QQ67\|J3QQ67_HUMAN | 525 |
| EIF5A | RPS2 | tr\|I3L504\|I3L504_HUMAN | sp\|P15880\|RS2_HUMAN | 834 |
| EIF5A | HEL-S-162eP | tr\|I3L504\|I3L504_HUMAN | tr\|V9HVZ4\|V9HVZ4_HUMAN | 494 |
| EIF5A | RPLP0P6 | tr\|I3L504\|I3L504_HUMAN | tr\|A8K4Z4\|A8K4Z4_HUMAN | 756 |
| EIF5A | EEF1B2 | tr\|I3L504\|I3L504_HUMAN | tr\|A0A024R3W7\|A0A024R3W7_HUMAN | 687 |
| EIF5A | RPL31 | tr\|I3L504\|I3L504_HUMAN | tr\|B2R4C1\|B2R4C1_HUMAN | 745 |
| EIF5A | HEL-S-94n | tr\|I3L504\|I3L504_HUMAN | tr\|V9HW72\|V9HW72_HUMAN | 451 |
| EIF5A | RPS12 | tr\|I3L504\|I3L504_HUMAN | sp\|P25398\|RS12_HUMAN | 492 |
| EIF5A | RPS14 | tr\|I3L504\|I3L504_HUMAN | sp\|P62263\|RS14_HUMAN | 658 |
| EIF5A | RPL27A | tr\|I3L504\|I3L504_HUMAN | sp\|P46776\|RL27A_HUMAN | 695 |
| EIF5A | EIF2S2 | tr\|I3L504\|I3L504_HUMAN | tr\|Q6IBR8\|Q6IBR8_HUMAN | 484 |
| EIF5A | RPS13 | tr\|I3L504\|I3L504_HUMAN | sp\|P62277\|RS13_HUMAN | 823 |
| EIF5A | HEL-S-310 | tr\|I3L504\|I3L504_HUMAN | tr\|V9HW01\|V9HW01_HUMAN | 492 |
| EIF5A | RPL19 | tr\|I3L504\|I3L504_HUMAN | tr\|Q53G49\|Q53G49_HUMAN | 638 |
| HEL-S-94n | HEL-S-70 | tr\|V9HW72\|V9HW72_HUMAN | tr\|V9HW80\|V9HW80_HUMAN | 587 |
| HEL-S-94n | DNAJB6 | tr\|V9HW72\|V9HW72_HUMAN | sp\|O75190\|DNJB6_HUMAN | 679 |
| HEL-S-94n | UBE1 | tr\|V9HW72\|V9HW72_HUMAN | tr\|A0A024R1A3\|A0A024R1A3_HUMAN | 437 |
| PPIB | EL52 | sp\|P23284\|PPIB_HUMAN | tr\|K9JA46\|K9JA46_HUMAN | 585 |
| PPIB | HEL-S-162eP | sp\|P23284\|PPIB_HUMAN | tr\|V9HVZ4\|V9HVZ4_HUMAN | 574 |
| PPIB | TRA1 | sp\|P23284\|PPIB_HUMAN | tr\|Q5CAQ5\|Q5CAQ5_HUMAN | 819 |
| PPIB | CANX | sp\|P23284\|PPIB_HUMAN | sp\|P27824\|CALX_HUMAN | 431 |
| PPIB | ERP70 | sp\|P23284\|PPIB_HUMAN | tr\|A0A090N8Y2\|A0A090N8Y2_HUMAN | 951 |
| UBE2V1 | PCNA | sp\|Q13404\|UB2V1_HUMAN | sp\|P12004\|PCNA_HUMAN | 885 |
| UBE2V1 | UBE2O | sp\|Q13404\|UB2V1_HUMAN | sp\|Q9C0C9\|UBE2O_HUMAN | 453 |
| UBE2V1 | UBE1 | sp\|Q13404\|UB2V1_HUMAN | tr\|A0A024R1A3\|A0A024R1A3_HUMAN | 498 |
| HEL-S-70 | JAK2 | tr\|V9HW80\|V9HW80_HUMAN | tr\|Q53EL3\|Q53EL3_HUMAN | 659 |
| HEL-S-70 | UBE1 | tr\|V9HW80\|V9HW80_HUMAN | tr\|A0A024R1A3\|A0A024R1A3_HUMAN | 699 |
| RPL19 | RPL18 | tr\|Q53G49\|Q53G49_HUMAN | tr\|J3QQ67\|J3QQ67_HUMAN | 999 |
| RPL19 | FBL | tr\|Q53G49\|Q53G49_HUMAN | sp\|P22087\|FBRL_HUMAN | 724 |
| RPL19 | RPS2 | tr\|Q53G49\|Q53G49_HUMAN | sp\|P15880\|RS2_HUMAN | 999 |
| RPL19 | HEL-S-162eP | tr\|Q53G49\|Q53G49_HUMAN | tr\|V9HVZ4\|V9HVZ4_HUMAN | 519 |
| RPL19 | RPLP0P6 | tr\|Q53G49\|Q53G49_HUMAN | tr\|A8K4Z4\|A8K4Z4_HUMAN | 999 |
| RPL19 | EEF1B2 | tr\|Q53G49\|Q53G49_HUMAN | tr\|A0A024R3W7\|A0A024R3W7_HUMAN | 741 |
| RPL19 | BTF3 | tr\|Q53G49\|Q53G49_HUMAN | sp\|P20290\|BTF3_HUMAN | 532 |
| RPL19 | RPL31 | tr\|Q53G49\|Q53G49_HUMAN | tr\|B2R4C1\|B2R4C1_HUMAN | 999 |
| RPL19 | RPS12 | tr\|Q53G49\|Q53G49_HUMAN | sp\|P25398\|RS12_HUMAN | 999 |
| RPL19 | RPS14 | tr\|Q53G49\|Q53G49_HUMAN | sp\|P62263\|RS14_HUMAN | 999 |
| RPL19 | RPL27A | tr\|Q53G49\|Q53G49_HUMAN | sp\|P46776\|RL27A_HUMAN | 999 |
| RPL19 | EIF2S2 | tr\|Q53G49\|Q53G49_HUMAN | tr\|Q6IBR8\|Q6IBR8_HUMAN | 909 |
| RPL19 | RPS13 | tr\|Q53G49\|Q53G49_HUMAN | sp\|P62277\|RS13_HUMAN | 999 |
| RPL19 | HEL-S-310 | tr\|Q53G49\|Q53G49_HUMAN | tr\|V9HW01\|V9HW01_HUMAN | 999 |
| EL52 | HEL-S-162eP | tr\|K9JA46\|K9JA46_HUMAN | tr\|V9HVZ4\|V9HVZ4_HUMAN | 871 |
| EL52 | JAK2 | tr\|K9JA46\|K9JA46_HUMAN | tr\|Q53EL3\|Q53EL3_HUMAN | 952 |
| EL52 | TRA1 | tr\|K9JA46\|K9JA46_HUMAN | tr\|Q5CAQ5\|Q5CAQ5_HUMAN | 655 |
| EL52 | HEL-S-68p | tr\|K9JA46\|K9JA46_HUMAN | tr\|V9HWF4\|V9HWF4_HUMAN | 659 |
| EL52 | DNAJB6 | tr\|K9JA46\|K9JA46_HUMAN | sp\|O75190\|DNJB6_HUMAN | 990 |
| EL52 | UBE1 | tr\|K9JA46\|K9JA46_HUMAN | tr\|A0A024R1A3\|A0A024R1A3_HUMAN | 466 |
| EL52 | RPAP3 | tr\|K9JA46\|K9JA46_HUMAN | sp\|Q9H6T3\|RPAP3_HUMAN | 955 |
| EL52 | PCNA | tr\|K9JA46\|K9JA46_HUMAN | sp\|P12004\|PCNA_HUMAN | 571 |
| EL52 | DNAJA1 | tr\|K9JA46\|K9JA46_HUMAN | sp\|P31689\|DNJA1_HUMAN | 995 |
| EL52 | HEL-S-94n | tr\|K9JA46\|K9JA46_HUMAN | tr\|V9HW72\|V9HW72_HUMAN | 999 |
| EL52 | HEL-S-70 | tr\|K9JA46\|K9JA46_HUMAN | tr\|V9HW80\|V9HW80_HUMAN | 888 |
| EL52 | MAPK13 | tr\|K9JA46\|K9JA46_HUMAN | tr\|Q6FHR4\|Q6FHR4_HUMAN | 728 |
| EL52 | ERP70 | tr\|K9JA46\|K9JA46_HUMAN | tr\|A0A090N8Y2\|A0A090N8Y2_HUMAN | 757 |
| EL52 | CANX | tr\|K9JA46\|K9JA46_HUMAN | sp\|P27824\|CALX_HUMAN | 941 |
| FBL | RPL18 | sp\|P22087\|FBRL_HUMAN | tr\|J3QQ67\|J3QQ67_HUMAN | 792 |
| FBL | RPS2 | sp\|P22087\|FBRL_HUMAN | sp\|P15880\|RS2_HUMAN | 921 |
| FBL | SSB | sp\|P22087\|FBRL_HUMAN | tr\|B5BUB5\|B5BUB5_HUMAN | 935 |
| FBL | RPLP0P6 | sp\|P22087\|FBRL_HUMAN | tr\|A8K4Z4\|A8K4Z4_HUMAN | 612 |
| FBL | PCNA | sp\|P22087\|FBRL_HUMAN | sp\|P12004\|PCNA_HUMAN | 449 |
| FBL | SRRM2 | sp\|P22087\|FBRL_HUMAN | tr\|A0A140VK53\|A0A140VK53_HUMAN | 593 |
| FBL | RPS14 | sp\|P22087\|FBRL_HUMAN | sp\|P62263\|RS14_HUMAN | 966 |
| FBL | RPS12 | sp\|P22087\|FBRL_HUMAN | sp\|P25398\|RS12_HUMAN | 772 |
| FBL | EIF2S2 | sp\|P22087\|FBRL_HUMAN | tr\|Q6IBR8\|Q6IBR8_HUMAN | 429 |
| FBL | PRPF19 | sp\|P22087\|FBRL_HUMAN | sp\|Q9UMS4\|PRP19_HUMAN | 597 |
| FBL | RPS13 | sp\|P22087\|FBRL_HUMAN | sp\|P62277\|RS13_HUMAN | 970 |
| FBL | HEL-S-310 | sp\|P22087\|FBRL_HUMAN | tr\|V9HW01\|V9HW01_HUMAN | 444 |
| EEF1B2 | RPL18 | tr\|A0A024R3W7\|A0A024R3W7_HUMAN | tr\|J3QQ67\|J3QQ67_HUMAN | 744 |
| EEF1B2 | RPS2 | tr\|A0A024R3W7\|A0A024R3W7_HUMAN | sp\|P15880\|RS2_HUMAN | 883 |
| EEF1B2 | RPLP0P6 | tr\|A0A024R3W7\|A0A024R3W7_HUMAN | tr\|A8K4Z4\|A8K4Z4_HUMAN | 959 |
| EEF1B2 | RPL31 | tr\|A0A024R3W7\|A0A024R3W7_HUMAN | tr\|B2R4C1\|B2R4C1_HUMAN | 781 |
| EEF1B2 | AARS | tr\|A0A024R3W7\|A0A024R3W7_HUMAN | sp\|P49588\|SYAC_HUMAN | 607 |
| EEF1B2 | DNAJA1 | tr\|A0A024R3W7\|A0A024R3W7_HUMAN | sp\|P31689\|DNJA1_HUMAN | 535 |
| EEF1B2 | RPS12 | tr\|A0A024R3W7\|A0A024R3W7_HUMAN | sp\|P25398\|RS12_HUMAN | 654 |
| EEF1B2 | RPS14 | tr\|A0A024R3W7\|A0A024R3W7_HUMAN | sp\|P62263\|RS14_HUMAN | 531 |
| EEF1B2 | RPL27A | tr\|A0A024R3W7\|A0A024R3W7_HUMAN | sp\|P46776\|RL27A_HUMAN | 774 |
| EEF1B2 | RPS13 | tr\|A0A024R3W7\|A0A024R3W7_HUMAN | sp\|P62277\|RS13_HUMAN | 608 |
| EEF1B2 | HEL-S-310 | tr\|A0A024R3W7\|A0A024R3W7_HUMAN | tr\|V9HW01\|V9HW01_HUMAN | 706 |
| PC | HEL-S-26 | tr\|A0A024R5C5\|A0A024R5C5_HUMAN | tr\|V9HWJ2\|V9HWJ2_HUMAN | 405 |
| PC | TPI1 | tr\|A0A024R5C5\|A0A024R5C5_HUMAN | sp\|P60174\|TPIS_HUMAN | 435 |
| PC | HEL-S-162eP | tr\|A0A024R5C5\|A0A024R5C5_HUMAN | tr\|V9HVZ4\|V9HVZ4_HUMAN | 606 |
|  |  |  |  |  |

**Table S4b. The details of PPI interaction nodes in the WT vs control group**

| **Node1-gene** | **Node2-gene** | **Node1-uniprotein** | **Node2-unprotein** | **Score** |
| --- | --- | --- | --- | --- |
| MRPL14 | TUFM | tr\|A0A024RD78\|A0A024RD78_HUMAN | sp\|P49411\|EFTU_HUMAN | 578 |
| FKBP4 | STUB1 | sp\|Q02790\|FKBP4_HUMAN | sp\|Q9UNE7\|CHIP_HUMAN | 408 |
| FKBP4 | EL52 | sp\|Q02790\|FKBP4_HUMAN | tr\|K9JA46\|K9JA46_HUMAN | 999 |
| FKBP4 | HEL-S-269 | sp\|Q02790\|FKBP4_HUMAN | tr\|V9HVY3\|V9HVY3_HUMAN | 418 |
| FKBP4 | HEL-S-72p | sp\|Q02790\|FKBP4_HUMAN | tr\|V9HW22\|V9HW22_HUMAN | 962 |
| FKBP4 | TRA1 | sp\|Q02790\|FKBP4_HUMAN | tr\|Q5CAQ5\|Q5CAQ5_HUMAN | 497 |
| FKBP4 | PDIA6 | sp\|Q02790\|FKBP4_HUMAN | sp\|Q15084\|PDIA6_HUMAN | 446 |
| FKBP4 | HEL-S-103 | sp\|Q02790\|FKBP4_HUMAN | tr\|A8K5I0\|A8K5I0_HUMAN | 442 |
| FKBP4 | HEL-S-94n | sp\|Q02790\|FKBP4_HUMAN | tr\|V9HW72\|V9HW72_HUMAN | 883 |
| FKBP4 | PPIB | sp\|Q02790\|FKBP4_HUMAN | sp\|P23284\|PPIB_HUMAN | 481 |
| FKBP4 | HSPH1 | sp\|Q02790\|FKBP4_HUMAN | tr\|A0A024RDQ0\|A0A024RDQ0_HUMAN | 953 |
| FKBP4 | HSP90AB1 | sp\|Q02790\|FKBP4_HUMAN | tr\|A0A024RD80\|A0A024RD80_HUMAN | 991 |
| FKBP4 | TRAP1 | sp\|Q02790\|FKBP4_HUMAN | tr\|Q59EK6\|Q59EK6_HUMAN | 583 |
| FKBP4 | S100A6 | sp\|Q02790\|FKBP4_HUMAN | sp\|P06703\|S10A6_HUMAN | 692 |
| FKBP4 | HEL2 | sp\|Q02790\|FKBP4_HUMAN | tr\|V9HW98\|V9HW98_HUMAN | 902 |
| FKBP4 | HSPA9 | sp\|Q02790\|FKBP4_HUMAN | tr\|Q8N1C8\|Q8N1C8_HUMAN | 409 |
| PMVK | DHCR7 | tr\|Q6FGV9\|Q6FGV9_HUMAN | tr\|X5DNI9\|X5DNI9_HUMAN | 570 |
| TOP2A | hCG_24487 | sp\|P11388\|TOP2A_HUMAN | tr\|A0A024R261\|A0A024R261_HUMAN | 564 |
| TOP2A | IARS | sp\|P11388\|TOP2A_HUMAN | sp\|P41252\|SYIC_HUMAN | 643 |
| TOP2A | HEL-S-103 | sp\|P11388\|TOP2A_HUMAN | tr\|A8K5I0\|A8K5I0_HUMAN | 637 |
| TOP2A | HEL-S-89n | sp\|P11388\|TOP2A_HUMAN | tr\|V9HWB4\|V9HWB4_HUMAN | 654 |
| TOP2A | RPL7 | sp\|P11388\|TOP2A_HUMAN | tr\|A0A024R814\|A0A024R814_HUMAN | 531 |
| TOP2A | RPL5 | sp\|P11388\|TOP2A_HUMAN | tr\|A2RUM7\|A2RUM7_HUMAN | 632 |
| TOP2A | RPS18 | sp\|P11388\|TOP2A_HUMAN | sp\|P62269\|RS18_HUMAN | 598 |
| TOP2A | PRDX1 | sp\|P11388\|TOP2A_HUMAN | sp\|Q06830\|PRDX1_HUMAN | 589 |
| TOP2A | TRAP1 | sp\|P11388\|TOP2A_HUMAN | tr\|Q59EK6\|Q59EK6_HUMAN | 553 |
| TOP2A | RANBP1 | sp\|P11388\|TOP2A_HUMAN | tr\|F6WQW2\|F6WQW2_HUMAN | 433 |
| TOP2A | YARS | sp\|P11388\|TOP2A_HUMAN | tr\|A0A0S2Z4R1\|A0A0S2Z4R1_HUMAN | 728 |
| TOP2A | IMPDH2 | sp\|P11388\|TOP2A_HUMAN | tr\|H0Y4R1\|H0Y4R1_HUMAN | 651 |
| TOP2A | TPI1 | sp\|P11388\|TOP2A_HUMAN | sp\|P60174\|TPIS_HUMAN | 571 |
| TOP2A | DLD | sp\|P11388\|TOP2A_HUMAN | tr\|A0A024R713\|A0A024R713_HUMAN | 480 |
| TOP2A | TARS | sp\|P11388\|TOP2A_HUMAN | tr\|Q53GX7\|Q53GX7_HUMAN | 617 |
| TOP2A | HYOU1 | sp\|P11388\|TOP2A_HUMAN | tr\|A0A087X054\|A0A087X054_HUMAN | 601 |
| TOP2A | ELAVL1 | sp\|P11388\|TOP2A_HUMAN | sp\|Q15717\|ELAV1_HUMAN | 824 |
| TOP2A | RPS5 | sp\|P11388\|TOP2A_HUMAN | tr\|A0A024R4Q8\|A0A024R4Q8_HUMAN | 621 |
| TOP2A | TKT | sp\|P11388\|TOP2A_HUMAN | tr\|Q53EM5\|Q53EM5_HUMAN | 479 |
| TOP2A | P4HB | sp\|P11388\|TOP2A_HUMAN | tr\|A0A024R8S5\|A0A024R8S5_HUMAN | 548 |
| TOP2A | UROD | sp\|P11388\|TOP2A_HUMAN | sp\|P06132\|DCUP_HUMAN | 664 |
| TOP2A | DNAJC17 | sp\|P11388\|TOP2A_HUMAN | sp\|Q9NVM6\|DJC17_HUMAN | 451 |
| TOP2A | HEL-S-100n | sp\|P11388\|TOP2A_HUMAN | tr\|V9HW96\|V9HW96_HUMAN | 629 |
| TOP2A | RPL23A | sp\|P11388\|TOP2A_HUMAN | sp\|P62750\|RL23A_HUMAN | 509 |
| TOP2A | HEL-S-2a | sp\|P11388\|TOP2A_HUMAN | tr\|V9HW12\|V9HW12_HUMAN | 585 |
| TOP2A | ERP70 | sp\|P11388\|TOP2A_HUMAN | tr\|A0A090N8Y2\|A0A090N8Y2_HUMAN | 519 |
| TOP2A | HSPA9 | sp\|P11388\|TOP2A_HUMAN | tr\|Q8N1C8\|Q8N1C8_HUMAN | 601 |
| TOP2A | HEL-S-133P | sp\|P11388\|TOP2A_HUMAN | tr\|V9HWB9\|V9HWB9_HUMAN | 618 |
| TOP2A | RPS9 | sp\|P11388\|TOP2A_HUMAN | tr\|A0A024R4M0\|A0A024R4M0_HUMAN | 599 |
| TOP2A | TUFM | sp\|P11388\|TOP2A_HUMAN | sp\|P49411\|EFTU_HUMAN | 655 |
| TOP2A | MKI67 | sp\|P11388\|TOP2A_HUMAN | tr\|A0A087WV66\|A0A087WV66_HUMAN | 472 |
| TOP2A | HEL-S-162eP | sp\|P11388\|TOP2A_HUMAN | tr\|V9HVZ4\|V9HVZ4_HUMAN | 723 |
| TOP2A | HADHA | sp\|P11388\|TOP2A_HUMAN | tr\|E9KL44\|E9KL44_HUMAN | 400 |
| TOP2A | HEL-S-72p | sp\|P11388\|TOP2A_HUMAN | tr\|V9HW22\|V9HW22_HUMAN | 629 |
| TOP2A | HEL-S-123m | sp\|P11388\|TOP2A_HUMAN | tr\|V9HW26\|V9HW26_HUMAN | 566 |
| TOP2A | GLUD1 | sp\|P11388\|TOP2A_HUMAN | tr\|E9KL48\|E9KL48_HUMAN | 504 |
| TOP2A | HEL-S-53e | sp\|P11388\|TOP2A_HUMAN | tr\|V9HW83\|V9HW83_HUMAN | 471 |
| TOP2A | UBA2 | sp\|P11388\|TOP2A_HUMAN | tr\|B2RDF5\|B2RDF5_HUMAN | 790 |
| TOP2A | RNPEP | sp\|P11388\|TOP2A_HUMAN | tr\|Q7RU04\|Q7RU04_HUMAN | 428 |
| TOP2A | ENO1 | sp\|P11388\|TOP2A_HUMAN | tr\|A0A024R4F1\|A0A024R4F1_HUMAN | 577 |
| TOP2A | DDX17 | sp\|P11388\|TOP2A_HUMAN | sp\|Q92841\|DDX17_HUMAN | 559 |
| TOP2A | RPL26 | sp\|P11388\|TOP2A_HUMAN | sp\|P61254\|RL26_HUMAN | 591 |
| TOP2A | HSPH1 | sp\|P11388\|TOP2A_HUMAN | tr\|A0A024RDQ0\|A0A024RDQ0_HUMAN | 631 |
| TOP2A | RPL10A | sp\|P11388\|TOP2A_HUMAN | sp\|P62906\|RL10A_HUMAN | 598 |
| TOP2A | HEL2 | sp\|P11388\|TOP2A_HUMAN | tr\|V9HW98\|V9HW98_HUMAN | 638 |
| TOP2A | RPS13 | sp\|P11388\|TOP2A_HUMAN | sp\|P62277\|RS13_HUMAN | 418 |
| TOP2A | LDHB | sp\|P11388\|TOP2A_HUMAN | tr\|Q5U077\|Q5U077_HUMAN | 585 |
| TOP2A | RPS3 | sp\|P11388\|TOP2A_HUMAN | sp\|P23396\|RS3_HUMAN | 604 |
| TOP2A | EL52 | sp\|P11388\|TOP2A_HUMAN | tr\|K9JA46\|K9JA46_HUMAN | 648 |
| TOP2A | HEL-S-269 | sp\|P11388\|TOP2A_HUMAN | tr\|V9HVY3\|V9HVY3_HUMAN | 518 |
| TOP2A | TRA1 | sp\|P11388\|TOP2A_HUMAN | tr\|Q5CAQ5\|Q5CAQ5_HUMAN | 577 |
| TOP2A | PDIA6 | sp\|P11388\|TOP2A_HUMAN | sp\|Q15084\|PDIA6_HUMAN | 518 |
| TOP2A | MDH2 | sp\|P11388\|TOP2A_HUMAN | tr\|Q6FHZ0\|Q6FHZ0_HUMAN | 576 |
| TOP2A | HEL-S-68p | sp\|P11388\|TOP2A_HUMAN | tr\|V9HWF4\|V9HWF4_HUMAN | 419 |
| TOP2A | HEL-S-271 | sp\|P11388\|TOP2A_HUMAN | tr\|V9HW31\|V9HW31_HUMAN | 579 |
| TOP2A | CCT4 | sp\|P11388\|TOP2A_HUMAN | sp\|P50991\|TCPD_HUMAN | 655 |
| TOP2A | RPL3 | sp\|P11388\|TOP2A_HUMAN | sp\|P39023\|RL3_HUMAN | 623 |
| TOP2A | HEL-S-128m | sp\|P11388\|TOP2A_HUMAN | tr\|V9HWC7\|V9HWC7_HUMAN | 580 |
| TOP2A | HSP90AB1 | sp\|P11388\|TOP2A_HUMAN | tr\|A0A024RD80\|A0A024RD80_HUMAN | 554 |
| NCL | SRSF1 | tr\|A0A024R4A0\|A0A024R4A0_HUMAN | sp\|Q07955\|SRSF1_HUMAN | 426 |
| NCL | RPS3A | tr\|A0A024R4A0\|A0A024R4A0_HUMAN | tr\|Q6NXR8\|Q6NXR8_HUMAN | 675 |
| NCL | MKI67 | tr\|A0A024R4A0\|A0A024R4A0_HUMAN | tr\|A0A087WV66\|A0A087WV66_HUMAN | 408 |
| NCL | HEL-S-162eP | tr\|A0A024R4A0\|A0A024R4A0_HUMAN | tr\|V9HVZ4\|V9HVZ4_HUMAN | 505 |
| NCL | SSB | tr\|A0A024R4A0\|A0A024R4A0_HUMAN | tr\|B5BUB5\|B5BUB5_HUMAN | 853 |
| NCL | RPL7 | tr\|A0A024R4A0\|A0A024R4A0_HUMAN | tr\|A0A024R814\|A0A024R814_HUMAN | 677 |
| NCL | RPL5 | tr\|A0A024R4A0\|A0A024R4A0_HUMAN | tr\|A2RUM7\|A2RUM7_HUMAN | 766 |
| NCL | RPL26 | tr\|A0A024R4A0\|A0A024R4A0_HUMAN | sp\|P61254\|RL26_HUMAN | 913 |
| NCL | RPL6 | tr\|A0A024R4A0\|A0A024R4A0_HUMAN | tr\|Q8N5Z7\|Q8N5Z7_HUMAN | 695 |
| NCL | HNRPU | tr\|A0A024R4A0\|A0A024R4A0_HUMAN | tr\|B4DLR3\|B4DLR3_HUMAN | 586 |
| NCL | XRCC6 | tr\|A0A024R4A0\|A0A024R4A0_HUMAN | tr\|A0A024R1N4\|A0A024R1N4_HUMAN | 939 |
| NCL | RPS4X | tr\|A0A024R4A0\|A0A024R4A0_HUMAN | tr\|B2R491\|B2R491_HUMAN | 621 |
| NCL | PTBP1 | tr\|A0A024R4A0\|A0A024R4A0_HUMAN | sp\|P26599\|PTBP1_HUMAN | 814 |
| NCL | RPL19 | tr\|A0A024R4A0\|A0A024R4A0_HUMAN | tr\|Q53G49\|Q53G49_HUMAN | 628 |
| NCL | RPL18 | tr\|A0A024R4A0\|A0A024R4A0_HUMAN | tr\|J3QQ67\|J3QQ67_HUMAN | 670 |
| NCL | NPM1 | tr\|A0A024R4A0\|A0A024R4A0_HUMAN | tr\|A0A0S2Z491\|A0A0S2Z491_HUMAN | 999 |
| NCL | EL52 | tr\|A0A024R4A0\|A0A024R4A0_HUMAN | tr\|K9JA46\|K9JA46_HUMAN | 993 |
| NCL | ELAVL1 | tr\|A0A024R4A0\|A0A024R4A0_HUMAN | sp\|Q15717\|ELAV1_HUMAN | 540 |
| NCL | LMNA | tr\|A0A024R4A0\|A0A024R4A0_HUMAN | sp\|P02545\|LMNA_HUMAN | 531 |
| NCL | RPS5 | tr\|A0A024R4A0\|A0A024R4A0_HUMAN | tr\|A0A024R4Q8\|A0A024R4Q8_HUMAN | 705 |
| NCL | NAP1L4b | tr\|A0A024R4A0\|A0A024R4A0_HUMAN | tr\|B4DS05\|B4DS05_HUMAN | 699 |
| NCL | RPL3 | tr\|A0A024R4A0\|A0A024R4A0_HUMAN | sp\|P39023\|RL3_HUMAN | 973 |
| NCL | RPS6 | tr\|A0A024R4A0\|A0A024R4A0_HUMAN | tr\|A2A3R6\|A2A3R6_HUMAN | 732 |
| NCL | MYH9 | tr\|A0A024R4A0\|A0A024R4A0_HUMAN | tr\|A0A024R1N1\|A0A024R1N1_HUMAN | 624 |
| NCL | RPS19 | tr\|A0A024R4A0\|A0A024R4A0_HUMAN | tr\|Q8WVX7\|Q8WVX7_HUMAN | 731 |
| LGALS3BP | TRAP1 | tr\|A0A0S2Z3Y1\|A0A0S2Z3Y1_HUMAN | tr\|Q59EK6\|Q59EK6_HUMAN | 499 |
| ITGA3 | ACTN4 | tr\|B4E0H8\|B4E0H8_HUMAN | tr\|A0A0S2Z3G9\|A0A0S2Z3G9_HUMAN | 830 |
| ITGA3 | ITGB4 | tr\|B4E0H8\|B4E0H8_HUMAN | tr\|B7ZLD5\|B7ZLD5_HUMAN | 972 |
| ITGA3 | HEL-S-99n | tr\|B4E0H8\|B4E0H8_HUMAN | tr\|V9HW88\|V9HW88_HUMAN | 822 |
| SLC25A3 | HEL-S-123m | tr\|Q8NCF7\|Q8NCF7_HUMAN | tr\|V9HW26\|V9HW26_HUMAN | 563 |
| SLC25A3 | HEL-S-271 | tr\|Q8NCF7\|Q8NCF7_HUMAN | tr\|V9HW31\|V9HW31_HUMAN | 833 |
| SLC25A3 | RPL3 | tr\|Q8NCF7\|Q8NCF7_HUMAN | sp\|P39023\|RL3_HUMAN | 417 |
| YARS | hCG_24487 | tr\|A0A0S2Z4R1\|A0A0S2Z4R1_HUMAN | tr\|A0A024R261\|A0A024R261_HUMAN | 644 |
| YARS | IARS | tr\|A0A0S2Z4R1\|A0A0S2Z4R1_HUMAN | sp\|P41252\|SYIC_HUMAN | 923 |
| YARS | SSB | tr\|A0A0S2Z4R1\|A0A0S2Z4R1_HUMAN | tr\|B5BUB5\|B5BUB5_HUMAN | 672 |
| YARS | HEL-S-103 | tr\|A0A0S2Z4R1\|A0A0S2Z4R1_HUMAN | tr\|A8K5I0\|A8K5I0_HUMAN | 578 |
| YARS | HEL-S-89n | tr\|A0A0S2Z4R1\|A0A0S2Z4R1_HUMAN | tr\|V9HWB4\|V9HWB4_HUMAN | 608 |
| YARS | RPL7 | tr\|A0A0S2Z4R1\|A0A0S2Z4R1_HUMAN | tr\|A0A024R814\|A0A024R814_HUMAN | 495 |
| YARS | RPS18 | tr\|A0A0S2Z4R1\|A0A0S2Z4R1_HUMAN | sp\|P62269\|RS18_HUMAN | 652 |
| YARS | IMPDH2 | tr\|A0A0S2Z4R1\|A0A0S2Z4R1_HUMAN | tr\|H0Y4R1\|H0Y4R1_HUMAN | 462 |
| YARS | TPI1 | tr\|A0A0S2Z4R1\|A0A0S2Z4R1_HUMAN | sp\|P60174\|TPIS_HUMAN | 427 |
| YARS | TARS | tr\|A0A0S2Z4R1\|A0A0S2Z4R1_HUMAN | tr\|Q53GX7\|Q53GX7_HUMAN | 956 |
| YARS | HYOU1 | tr\|A0A0S2Z4R1\|A0A0S2Z4R1_HUMAN | tr\|A0A087X054\|A0A087X054_HUMAN | 582 |
| YARS | LMNA | tr\|A0A0S2Z4R1\|A0A0S2Z4R1_HUMAN | sp\|P02545\|LMNA_HUMAN | 405 |
| YARS | RPS5 | tr\|A0A0S2Z4R1\|A0A0S2Z4R1_HUMAN | tr\|A0A024R4Q8\|A0A024R4Q8_HUMAN | 629 |
| YARS | AARS | tr\|A0A0S2Z4R1\|A0A0S2Z4R1_HUMAN | sp\|P49588\|SYAC_HUMAN | 922 |
| YARS | RPL23A | tr\|A0A0S2Z4R1\|A0A0S2Z4R1_HUMAN | sp\|P62750\|RL23A_HUMAN | 594 |
| YARS | HEL-S-100n | tr\|A0A0S2Z4R1\|A0A0S2Z4R1_HUMAN | tr\|V9HW96\|V9HW96_HUMAN | 791 |
| YARS | HSPA9 | tr\|A0A0S2Z4R1\|A0A0S2Z4R1_HUMAN | tr\|Q8N1C8\|Q8N1C8_HUMAN | 645 |
| YARS | TUFM | tr\|A0A0S2Z4R1\|A0A0S2Z4R1_HUMAN | sp\|P49411\|EFTU_HUMAN | 556 |
| YARS | RPS9 | tr\|A0A0S2Z4R1\|A0A0S2Z4R1_HUMAN | tr\|A0A024R4M0\|A0A024R4M0_HUMAN | 659 |
| YARS | HEL-S-72p | tr\|A0A0S2Z4R1\|A0A0S2Z4R1_HUMAN | tr\|V9HW22\|V9HW22_HUMAN | 739 |
| YARS | RNPEP | tr\|A0A0S2Z4R1\|A0A0S2Z4R1_HUMAN | tr\|Q7RU04\|Q7RU04_HUMAN | 505 |
| YARS | ENO1 | tr\|A0A0S2Z4R1\|A0A0S2Z4R1_HUMAN | tr\|A0A024R4F1\|A0A024R4F1_HUMAN | 673 |
| YARS | EEF1G | tr\|A0A0S2Z4R1\|A0A0S2Z4R1_HUMAN | tr\|Q53YD7\|Q53YD7_HUMAN | 545 |
| YARS | HSPH1 | tr\|A0A0S2Z4R1\|A0A0S2Z4R1_HUMAN | tr\|A0A024RDQ0\|A0A024RDQ0_HUMAN | 592 |
| YARS | RPL10A | tr\|A0A0S2Z4R1\|A0A0S2Z4R1_HUMAN | sp\|P62906\|RL10A_HUMAN | 631 |
| YARS | RPS13 | tr\|A0A0S2Z4R1\|A0A0S2Z4R1_HUMAN | sp\|P62277\|RS13_HUMAN | 595 |
| YARS | RPS27A | tr\|A0A0S2Z4R1\|A0A0S2Z4R1_HUMAN | tr\|B2RDW1\|B2RDW1_HUMAN | 410 |
| YARS | RPS3 | tr\|A0A0S2Z4R1\|A0A0S2Z4R1_HUMAN | sp\|P23396\|RS3_HUMAN | 645 |
| YARS | DYNC1H1 | tr\|A0A0S2Z4R1\|A0A0S2Z4R1_HUMAN | sp\|Q14204\|DYHC1_HUMAN | 910 |
| YARS | RAD23B | tr\|A0A0S2Z4R1\|A0A0S2Z4R1_HUMAN | sp\|P54727\|RD23B_HUMAN | 410 |
| YARS | CCT4 | tr\|A0A0S2Z4R1\|A0A0S2Z4R1_HUMAN | sp\|P50991\|TCPD_HUMAN | 766 |
| YARS | RPL3 | tr\|A0A0S2Z4R1\|A0A0S2Z4R1_HUMAN | sp\|P39023\|RL3_HUMAN | 673 |
| YARS | FARSB | tr\|A0A0S2Z4R1\|A0A0S2Z4R1_HUMAN | tr\|Q9BR63\|Q9BR63_HUMAN | 930 |
| YARS | FLJ00064 | tr\|A0A0S2Z4R1\|A0A0S2Z4R1_HUMAN | tr\|B4DV28\|B4DV28_HUMAN | 405 |
| HEL-S-91n | RPS27A | tr\|V9HW09\|V9HW09_HUMAN | tr\|B2RDW1\|B2RDW1_HUMAN | 933 |
| HEL-S-91n | PSMC3 | tr\|V9HW09\|V9HW09_HUMAN | tr\|A0A140VK42\|A0A140VK42_HUMAN | 999 |
| HEL-S-91n | EL52 | tr\|V9HW09\|V9HW09_HUMAN | tr\|K9JA46\|K9JA46_HUMAN | 688 |
| HEL-S-91n | HEL-S-72p | tr\|V9HW09\|V9HW09_HUMAN | tr\|V9HW22\|V9HW22_HUMAN | 901 |
| HEL-S-91n | RAD23B | tr\|V9HW09\|V9HW09_HUMAN | sp\|P54727\|RD23B_HUMAN | 891 |
| HEL-S-91n | CCT4 | tr\|V9HW09\|V9HW09_HUMAN | sp\|P50991\|TCPD_HUMAN | 430 |
| HEL-S-91n | RPLP2 | tr\|V9HW09\|V9HW09_HUMAN | tr\|A0A024RCA7\|A0A024RCA7_HUMAN | 808 |
| HEL-S-91n | HSP90AB1 | tr\|V9HW09\|V9HW09_HUMAN | tr\|A0A024RD80\|A0A024RD80_HUMAN | 659 |
| HEL-S-91n | PSMA4 | tr\|V9HW09\|V9HW09_HUMAN | sp\|P25789\|PSA4_HUMAN | 999 |
| HEL-S-91n | TMSB10 | tr\|V9HW09\|V9HW09_HUMAN | tr\|D6W5K2\|D6W5K2_HUMAN | 486 |
| HEL-S-91n | PSMD4 | tr\|V9HW09\|V9HW09_HUMAN | sp\|P55036\|PSMD4_HUMAN | 999 |
| CD44 | KRT19 | sp\|P16070\|CD44_HUMAN | sp\|P08727\|K1C19_HUMAN | 472 |
| CD44 | EZR | sp\|P16070\|CD44_HUMAN | tr\|E7EQR4\|E7EQR4_HUMAN | 991 |
| CD44 | hCG_1991735 | sp\|P16070\|CD44_HUMAN | tr\|A0A024RC65\|A0A024RC65_HUMAN | 919 |
| CD44 | HEL70 | sp\|P16070\|CD44_HUMAN | tr\|V9HWC0\|V9HWC0_HUMAN | 978 |
| CD44 | DPP4 | sp\|P16070\|CD44_HUMAN | sp\|P27487\|DPP4_HUMAN | 407 |
| CD44 | ELAVL1 | sp\|P16070\|CD44_HUMAN | sp\|Q15717\|ELAV1_HUMAN | 431 |
| CD44 | HEL-S-53e | sp\|P16070\|CD44_HUMAN | tr\|V9HW83\|V9HW83_HUMAN | 581 |
| CD44 | CDH1 | sp\|P16070\|CD44_HUMAN | tr\|D3XNU5\|D3XNU5_HUMAN | 923 |
| PFN1 | TMSB4X | sp\|P07737\|PROF1_HUMAN | tr\|Q0P5N8\|Q0P5N8_HUMAN | 489 |
| PFN1 | STUB1 | sp\|P07737\|PROF1_HUMAN | sp\|Q9UNE7\|CHIP_HUMAN | 585 |
| PFN1 | TMSB10 | sp\|P07737\|PROF1_HUMAN | tr\|D6W5K2\|D6W5K2_HUMAN | 471 |
| PFN1 | HEL-S-15 | sp\|P07737\|PROF1_HUMAN | tr\|V9HWI5\|V9HWI5_HUMAN | 982 |
| CLTC | RNF213 | sp\|Q00610\|CLH1_HUMAN | tr\|A0A0A0MTC1\|A0A0A0MTC1_HUMAN | 414 |
| CLTC | COPB1 | sp\|Q00610\|CLH1_HUMAN | sp\|P53618\|COPB_HUMAN | 722 |
| CLTC | COPB2 | sp\|Q00610\|CLH1_HUMAN | sp\|P35606\|COPB2_HUMAN | 641 |
| CLTC | NPM1 | sp\|Q00610\|CLH1_HUMAN | tr\|A0A0S2Z491\|A0A0S2Z491_HUMAN | 616 |
| CLTC | KPNB1 | sp\|Q00610\|CLH1_HUMAN | sp\|Q14974\|IMB1_HUMAN | 573 |
| CLTC | HEL2 | sp\|Q00610\|CLH1_HUMAN | tr\|V9HW98\|V9HW98_HUMAN | 533 |
| hCG_1991735 | ACTN4 | tr\|A0A024RC65\|A0A024RC65_HUMAN | tr\|A0A0S2Z3G9\|A0A0S2Z3G9_HUMAN | 799 |
| hCG_1991735 | EZR | tr\|A0A024RC65\|A0A024RC65_HUMAN | tr\|E7EQR4\|E7EQR4_HUMAN | 706 |
| hCG_1991735 | PAFAH1B1 | tr\|A0A024RC65\|A0A024RC65_HUMAN | sp\|P43034\|LIS1_HUMAN | 941 |
| hCG_1991735 | HEL-S-269 | tr\|A0A024RC65\|A0A024RC65_HUMAN | tr\|V9HVY3\|V9HVY3_HUMAN | 786 |
| hCG_1991735 | MYH14 | tr\|A0A024RC65\|A0A024RC65_HUMAN | sp\|Q7Z406\|MYH14_HUMAN | 749 |
| hCG_1991735 | HEL-S-89n | tr\|A0A024RC65\|A0A024RC65_HUMAN | tr\|V9HWB4\|V9HWB4_HUMAN | 645 |
| hCG_1991735 | HEL-S-47e | tr\|A0A024RC65\|A0A024RC65_HUMAN | tr\|V9HWE8\|V9HWE8_HUMAN | 416 |
| hCG_1991735 | HEL-S-100n | tr\|A0A024RC65\|A0A024RC65_HUMAN | tr\|V9HW96\|V9HW96_HUMAN | 422 |
| hCG_1991735 | MYH9 | tr\|A0A024RC65\|A0A024RC65_HUMAN | tr\|A0A024R1N1\|A0A024R1N1_HUMAN | 719 |
| hCG_1991735 | HNRPA1 | tr\|A0A024RC65\|A0A024RC65_HUMAN | tr\|A0A024RAZ7\|A0A024RAZ7_HUMAN | 541 |
| hCG_1991735 | CDH1 | tr\|A0A024RC65\|A0A024RC65_HUMAN | tr\|D3XNU5\|D3XNU5_HUMAN | 996 |
| hCG_1991735 | ERP70 | tr\|A0A024RC65\|A0A024RC65_HUMAN | tr\|A0A090N8Y2\|A0A090N8Y2_HUMAN | 430 |
| hCG_1991735 | PTBP1 | tr\|A0A024RC65\|A0A024RC65_HUMAN | sp\|P26599\|PTBP1_HUMAN | 403 |
| HYOU1 | HEL-S-162eP | tr\|A0A087X054\|A0A087X054_HUMAN | tr\|V9HVZ4\|V9HVZ4_HUMAN | 513 |
| HYOU1 | HEL-S-72p | tr\|A0A087X054\|A0A087X054_HUMAN | tr\|V9HW22\|V9HW22_HUMAN | 557 |
| HYOU1 | HEL-S-103 | tr\|A0A087X054\|A0A087X054_HUMAN | tr\|A8K5I0\|A8K5I0_HUMAN | 538 |
| HYOU1 | HEL-S-89n | tr\|A0A087X054\|A0A087X054_HUMAN | tr\|V9HWB4\|V9HWB4_HUMAN | 877 |
| HYOU1 | ENO1 | tr\|A0A087X054\|A0A087X054_HUMAN | tr\|A0A024R4F1\|A0A024R4F1_HUMAN | 437 |
| HYOU1 | HEL-S-94n | tr\|A0A087X054\|A0A087X054_HUMAN | tr\|V9HW72\|V9HW72_HUMAN | 537 |
| HYOU1 | CAND1 | tr\|A0A087X054\|A0A087X054_HUMAN | tr\|A8K8U1\|A8K8U1_HUMAN | 516 |
| HYOU1 | PPIB | tr\|A0A087X054\|A0A087X054_HUMAN | sp\|P23284\|PPIB_HUMAN | 506 |
| HYOU1 | TRAP1 | tr\|A0A087X054\|A0A087X054_HUMAN | tr\|Q59EK6\|Q59EK6_HUMAN | 692 |
| HYOU1 | PRDX1 | tr\|A0A087X054\|A0A087X054_HUMAN | sp\|Q06830\|PRDX1_HUMAN | 492 |
| HYOU1 | HEL-S-70 | tr\|A0A087X054\|A0A087X054_HUMAN | tr\|V9HW80\|V9HW80_HUMAN | 521 |
| HYOU1 | CANX | tr\|A0A087X054\|A0A087X054_HUMAN | sp\|P27824\|CALX_HUMAN | 826 |
| HYOU1 | IMPDH2 | tr\|A0A087X054\|A0A087X054_HUMAN | tr\|H0Y4R1\|H0Y4R1_HUMAN | 610 |
| HYOU1 | RPS3 | tr\|A0A087X054\|A0A087X054_HUMAN | sp\|P23396\|RS3_HUMAN | 439 |
| HYOU1 | EL52 | tr\|A0A087X054\|A0A087X054_HUMAN | tr\|K9JA46\|K9JA46_HUMAN | 960 |
| HYOU1 | TARS | tr\|A0A087X054\|A0A087X054_HUMAN | tr\|Q53GX7\|Q53GX7_HUMAN | 400 |
| HYOU1 | HEL-S-269 | tr\|A0A087X054\|A0A087X054_HUMAN | tr\|V9HVY3\|V9HVY3_HUMAN | 804 |
| HYOU1 | TRA1 | tr\|A0A087X054\|A0A087X054_HUMAN | tr\|Q5CAQ5\|Q5CAQ5_HUMAN | 983 |
| HYOU1 | PDIA6 | tr\|A0A087X054\|A0A087X054_HUMAN | sp\|Q15084\|PDIA6_HUMAN | 843 |
| HYOU1 | CCT4 | tr\|A0A087X054\|A0A087X054_HUMAN | sp\|P50991\|TCPD_HUMAN | 770 |
| HYOU1 | HEL-S-99n | tr\|A0A087X054\|A0A087X054_HUMAN | tr\|V9HW88\|V9HW88_HUMAN | 813 |
| HYOU1 | AARS | tr\|A0A087X054\|A0A087X054_HUMAN | sp\|P49588\|SYAC_HUMAN | 584 |
| HYOU1 | DNAJC17 | tr\|A0A087X054\|A0A087X054_HUMAN | sp\|Q9NVM6\|DJC17_HUMAN | 718 |
| HYOU1 | P4HB | tr\|A0A087X054\|A0A087X054_HUMAN | tr\|A0A024R8S5\|A0A024R8S5_HUMAN | 726 |
| HYOU1 | HSP90AB1 | tr\|A0A087X054\|A0A087X054_HUMAN | tr\|A0A024RD80\|A0A024RD80_HUMAN | 936 |
| HYOU1 | HEL-S-100n | tr\|A0A087X054\|A0A087X054_HUMAN | tr\|V9HW96\|V9HW96_HUMAN | 767 |
| HYOU1 | HEL-S-2a | tr\|A0A087X054\|A0A087X054_HUMAN | tr\|V9HW12\|V9HW12_HUMAN | 502 |
| HYOU1 | ERP70 | tr\|A0A087X054\|A0A087X054_HUMAN | tr\|A0A090N8Y2\|A0A090N8Y2_HUMAN | 927 |
| ELAVL1 | SRSF1 | sp\|Q15717\|ELAV1_HUMAN | sp\|Q07955\|SRSF1_HUMAN | 568 |
| ELAVL1 | HEL-S-162eP | sp\|Q15717\|ELAV1_HUMAN | tr\|V9HVZ4\|V9HVZ4_HUMAN | 563 |
| ELAVL1 | SSB | sp\|Q15717\|ELAV1_HUMAN | tr\|B5BUB5\|B5BUB5_HUMAN | 445 |
| ELAVL1 | HEL-S-72p | sp\|Q15717\|ELAV1_HUMAN | tr\|V9HW22\|V9HW22_HUMAN | 485 |
| ELAVL1 | VDAC2 | sp\|Q15717\|ELAV1_HUMAN | tr\|A0A024QZN9\|A0A024QZN9_HUMAN | 644 |
| ELAVL1 | HEL-S-103 | sp\|Q15717\|ELAV1_HUMAN | tr\|A8K5I0\|A8K5I0_HUMAN | 427 |
| ELAVL1 | LMNB1 | sp\|Q15717\|ELAV1_HUMAN | sp\|P20700\|LMNB1_HUMAN | 441 |
| ELAVL1 | DDX17 | sp\|Q15717\|ELAV1_HUMAN | sp\|Q92841\|DDX17_HUMAN | 458 |
| ELAVL1 | KRT10 | sp\|Q15717\|ELAV1_HUMAN | sp\|P13645\|K1C10_HUMAN | 644 |
| ELAVL1 | RANBP1 | sp\|Q15717\|ELAV1_HUMAN | tr\|F6WQW2\|F6WQW2_HUMAN | 409 |
| ELAVL1 | HEL-S-70 | sp\|Q15717\|ELAV1_HUMAN | tr\|V9HW80\|V9HW80_HUMAN | 602 |
| ELAVL1 | HNRPU | sp\|Q15717\|ELAV1_HUMAN | tr\|B4DLR3\|B4DLR3_HUMAN | 416 |
| ELAVL1 | HNRPA1 | sp\|Q15717\|ELAV1_HUMAN | tr\|A0A024RAZ7\|A0A024RAZ7_HUMAN | 626 |
| ELAVL1 | CANX | sp\|Q15717\|ELAV1_HUMAN | sp\|P27824\|CALX_HUMAN | 419 |
| ELAVL1 | PTBP1 | sp\|Q15717\|ELAV1_HUMAN | sp\|P26599\|PTBP1_HUMAN | 898 |
| ELAVL1 | S100A10 | sp\|Q15717\|ELAV1_HUMAN | tr\|D3DV26\|D3DV26_HUMAN | 644 |
| ELAVL1 | HNRNPL | sp\|Q15717\|ELAV1_HUMAN | sp\|P14866\|HNRPL_HUMAN | 626 |
| ELAVL1 | HEL-S-99n | sp\|Q15717\|ELAV1_HUMAN | tr\|V9HW88\|V9HW88_HUMAN | 427 |
| ELAVL1 | HEL70 | sp\|Q15717\|ELAV1_HUMAN | tr\|V9HWC0\|V9HWC0_HUMAN | 405 |
| ELAVL1 | KPNB1 | sp\|Q15717\|ELAV1_HUMAN | sp\|Q14974\|IMB1_HUMAN | 748 |
| MYH14 | MYL6 | sp\|Q7Z406\|MYH14_HUMAN | tr\|G8JLA2\|G8JLA2_HUMAN | 984 |
| MYH14 | ACTN4 | sp\|Q7Z406\|MYH14_HUMAN | tr\|A0A0S2Z3G9\|A0A0S2Z3G9_HUMAN | 669 |
| MYH14 | EZR | sp\|Q7Z406\|MYH14_HUMAN | tr\|E7EQR4\|E7EQR4_HUMAN | 619 |
| MYH14 | SPTBN1 | sp\|Q7Z406\|MYH14_HUMAN | tr\|D6W5C0\|D6W5C0_HUMAN | 460 |
| MYH14 | EL52 | sp\|Q7Z406\|MYH14_HUMAN | tr\|K9JA46\|K9JA46_HUMAN | 837 |
| MYH14 | HEL-S-162eP | sp\|Q7Z406\|MYH14_HUMAN | tr\|V9HVZ4\|V9HVZ4_HUMAN | 739 |
| MYH14 | TRA1 | sp\|Q7Z406\|MYH14_HUMAN | tr\|Q5CAQ5\|Q5CAQ5_HUMAN | 472 |
| MYH14 | HEL-S-15 | sp\|Q7Z406\|MYH14_HUMAN | tr\|V9HWI5\|V9HWI5_HUMAN | 424 |
| MYH14 | SPTAN1 | sp\|Q7Z406\|MYH14_HUMAN | sp\|Q13813\|SPTN1_HUMAN | 482 |
| MYH14 | HEL-S-47e | sp\|Q7Z406\|MYH14_HUMAN | tr\|V9HWE8\|V9HWE8_HUMAN | 526 |
| MYH14 | HEL70 | sp\|Q7Z406\|MYH14_HUMAN | tr\|V9HWC0\|V9HWC0_HUMAN | 920 |
| MYH14 | HSP90AB1 | sp\|Q7Z406\|MYH14_HUMAN | tr\|A0A024RD80\|A0A024RD80_HUMAN | 770 |
| MYH14 | MYH9 | sp\|Q7Z406\|MYH14_HUMAN | tr\|A0A024R1N1\|A0A024R1N1_HUMAN | 952 |
| MYH14 | CDH1 | sp\|Q7Z406\|MYH14_HUMAN | tr\|D3XNU5\|D3XNU5_HUMAN | 561 |
| RBM39 | SRSF1 | tr\|B3KWX7\|B3KWX7_HUMAN | sp\|Q07955\|SRSF1_HUMAN | 754 |
| RBM39 | RALY | tr\|B3KWX7\|B3KWX7_HUMAN | tr\|A8K4T9\|A8K4T9_HUMAN | 732 |
| RBM39 | KPNB1 | tr\|B3KWX7\|B3KWX7_HUMAN | sp\|Q14974\|IMB1_HUMAN | 474 |
| RBM39 | HNRPA1 | tr\|B3KWX7\|B3KWX7_HUMAN | tr\|A0A024RAZ7\|A0A024RAZ7_HUMAN | 506 |
| RBM39 | HNRPU | tr\|B3KWX7\|B3KWX7_HUMAN | tr\|B4DLR3\|B4DLR3_HUMAN | 682 |
| HEL-S-47e | GDI2 | tr\|V9HWE8\|V9HWE8_HUMAN | tr\|B4DLV7\|B4DLV7_HUMAN | 960 |
| HEL-S-47e | EZR | tr\|V9HWE8\|V9HWE8_HUMAN | tr\|E7EQR4\|E7EQR4_HUMAN | 987 |
| HEL-S-47e | P4HB | tr\|V9HWE8\|V9HWE8_HUMAN | tr\|A0A024R8S5\|A0A024R8S5_HUMAN | 803 |
| HEL-S-47e | HEL70 | tr\|V9HWE8\|V9HWE8_HUMAN | tr\|V9HWC0\|V9HWC0_HUMAN | 972 |
| SPTAN1 | ACTN4 | sp\|Q13813\|SPTN1_HUMAN | tr\|A0A0S2Z3G9\|A0A0S2Z3G9_HUMAN | 908 |
| SPTAN1 | EZR | sp\|Q13813\|SPTN1_HUMAN | tr\|E7EQR4\|E7EQR4_HUMAN | 906 |
| SPTAN1 | SPTBN1 | sp\|Q13813\|SPTN1_HUMAN | tr\|D6W5C0\|D6W5C0_HUMAN | 999 |
| SPTAN1 | DHCR7 | sp\|Q13813\|SPTN1_HUMAN | tr\|X5DNI9\|X5DNI9_HUMAN | 506 |
| SPTAN1 | RPL22 | sp\|Q13813\|SPTN1_HUMAN | sp\|P35268\|RL22_HUMAN | 472 |
| SPTAN1 | HEL-S-89n | sp\|Q13813\|SPTN1_HUMAN | tr\|V9HWB4\|V9HWB4_HUMAN | 576 |
| SPTAN1 | LMNB1 | sp\|Q13813\|SPTN1_HUMAN | sp\|P20700\|LMNB1_HUMAN | 593 |
| SPTAN1 | PHB2 | sp\|Q13813\|SPTN1_HUMAN | sp\|Q99623\|PHB2_HUMAN | 814 |
| SPTAN1 | HEL70 | sp\|Q13813\|SPTN1_HUMAN | tr\|V9HWC0\|V9HWC0_HUMAN | 570 |
| SPTAN1 | TAGLN2 | sp\|Q13813\|SPTN1_HUMAN | sp\|P37802\|TAGL2_HUMAN | 527 |
| SPTAN1 | MYH9 | sp\|Q13813\|SPTN1_HUMAN | tr\|A0A024R1N1\|A0A024R1N1_HUMAN | 821 |
| SPTAN1 | GSPT1 | sp\|Q13813\|SPTN1_HUMAN | tr\|B2RCT6\|B2RCT6_HUMAN | 675 |
| SPTAN1 | PTBP1 | sp\|Q13813\|SPTN1_HUMAN | sp\|P26599\|PTBP1_HUMAN | 457 |
| RPL36 | DHX15 | sp\|Q9Y3U8\|RL36_HUMAN | sp\|O43143\|DHX15_HUMAN | 627 |
| RPL36 | hCG_24487 | sp\|Q9Y3U8\|RL36_HUMAN | tr\|A0A024R261\|A0A024R261_HUMAN | 999 |
| RPL36 | RPS9 | sp\|Q9Y3U8\|RL36_HUMAN | tr\|A0A024R4M0\|A0A024R4M0_HUMAN | 980 |
| RPL36 | RPS3A | sp\|Q9Y3U8\|RL36_HUMAN | tr\|Q6NXR8\|Q6NXR8_HUMAN | 997 |
| RPL36 | RPS8 | sp\|Q9Y3U8\|RL36_HUMAN | tr\|Q5JR94\|Q5JR94_HUMAN | 999 |
| RPL36 | RPL22 | sp\|Q9Y3U8\|RL36_HUMAN | sp\|P35268\|RL22_HUMAN | 999 |
| RPL36 | RPL7 | sp\|Q9Y3U8\|RL36_HUMAN | tr\|A0A024R814\|A0A024R814_HUMAN | 999 |
| RPL36 | RPL13 | sp\|Q9Y3U8\|RL36_HUMAN | tr\|Q6NZ55\|Q6NZ55_HUMAN | 999 |
| RPL36 | HEL103 | sp\|Q9Y3U8\|RL36_HUMAN | tr\|V9HWK0\|V9HWK0_HUMAN | 900 |
| RPL36 | RPL5 | sp\|Q9Y3U8\|RL36_HUMAN | tr\|A2RUM7\|A2RUM7_HUMAN | 999 |
| RPL36 | RPL26 | sp\|Q9Y3U8\|RL36_HUMAN | sp\|P61254\|RL26_HUMAN | 999 |
| RPL36 | RPS18 | sp\|Q9Y3U8\|RL36_HUMAN | sp\|P62269\|RS18_HUMAN | 981 |
| RPL36 | GNB2L1 | sp\|Q9Y3U8\|RL36_HUMAN | tr\|E9KL35\|E9KL35_HUMAN | 962 |
| RPL36 | RPL10A | sp\|Q9Y3U8\|RL36_HUMAN | sp\|P62906\|RL10A_HUMAN | 999 |
| RPL36 | RPL6 | sp\|Q9Y3U8\|RL36_HUMAN | tr\|Q8N5Z7\|Q8N5Z7_HUMAN | 999 |
| RPL36 | RPS7 | sp\|Q9Y3U8\|RL36_HUMAN | sp\|P62081\|RS7_HUMAN | 998 |
| RPL36 | RPS13 | sp\|Q9Y3U8\|RL36_HUMAN | sp\|P62277\|RS13_HUMAN | 999 |
| RPL36 | RPS4X | sp\|Q9Y3U8\|RL36_HUMAN | tr\|B2R491\|B2R491_HUMAN | 998 |
| RPL36 | PTBP1 | sp\|Q9Y3U8\|RL36_HUMAN | sp\|P26599\|PTBP1_HUMAN | 644 |
| RPL36 | RPL19 | sp\|Q9Y3U8\|RL36_HUMAN | tr\|Q53G49\|Q53G49_HUMAN | 999 |
| RPL36 | RPL18 | sp\|Q9Y3U8\|RL36_HUMAN | tr\|J3QQ67\|J3QQ67_HUMAN | 999 |
| RPL36 | RPS3 | sp\|Q9Y3U8\|RL36_HUMAN | sp\|P23396\|RS3_HUMAN | 999 |
| RPL36 | RPS27A | sp\|Q9Y3U8\|RL36_HUMAN | tr\|B2RDW1\|B2RDW1_HUMAN | 999 |
| RPL36 | RAD23B | sp\|Q9Y3U8\|RL36_HUMAN | sp\|P54727\|RD23B_HUMAN | 443 |
| RPL36 | RPS5 | sp\|Q9Y3U8\|RL36_HUMAN | tr\|A0A024R4Q8\|A0A024R4Q8_HUMAN | 998 |
| RPL36 | RPLP2 | sp\|Q9Y3U8\|RL36_HUMAN | tr\|A0A024RCA7\|A0A024RCA7_HUMAN | 999 |
| RPL36 | RPL3 | sp\|Q9Y3U8\|RL36_HUMAN | sp\|P39023\|RL3_HUMAN | 999 |
| RPL36 | SRP14 | sp\|Q9Y3U8\|RL36_HUMAN | sp\|P37108\|SRP14_HUMAN | 903 |
| RPL36 | RPS6 | sp\|Q9Y3U8\|RL36_HUMAN | tr\|A2A3R6\|A2A3R6_HUMAN | 998 |
| RPL36 | RPL23A | sp\|Q9Y3U8\|RL36_HUMAN | sp\|P62750\|RL23A_HUMAN | 999 |
| RPL36 | RPS19 | sp\|Q9Y3U8\|RL36_HUMAN | tr\|Q8WVX7\|Q8WVX7_HUMAN | 998 |
| UROD | HEL-S-128m | sp\|P06132\|DCUP_HUMAN | tr\|V9HWC7\|V9HWC7_HUMAN | 904 |
| UROD | TPI1 | sp\|P06132\|DCUP_HUMAN | sp\|P60174\|TPIS_HUMAN | 504 |
| UROD | PRDX1 | sp\|P06132\|DCUP_HUMAN | sp\|Q06830\|PRDX1_HUMAN | 909 |
| UROD | HADHA | sp\|P06132\|DCUP_HUMAN | tr\|E9KL44\|E9KL44_HUMAN | 602 |
| UROD | HEL-S-2a | sp\|P06132\|DCUP_HUMAN | tr\|V9HW12\|V9HW12_HUMAN | 908 |
| UROD | HSPA9 | sp\|P06132\|DCUP_HUMAN | tr\|Q8N1C8\|Q8N1C8_HUMAN | 400 |
| UROD | IMPDH2 | sp\|P06132\|DCUP_HUMAN | tr\|H0Y4R1\|H0Y4R1_HUMAN | 539 |
| COPS8 | CAND1 | tr\|B2R8N1\|B2R8N1_HUMAN | tr\|A8K8U1\|A8K8U1_HUMAN | 640 |
| DNAJC17 | EL52 | sp\|Q9NVM6\|DJC17_HUMAN | tr\|K9JA46\|K9JA46_HUMAN | 423 |
| DNAJC17 | HSP90AB1 | sp\|Q9NVM6\|DJC17_HUMAN | tr\|A0A024RD80\|A0A024RD80_HUMAN | 410 |
| DNAJC17 | HSPH1 | sp\|Q9NVM6\|DJC17_HUMAN | tr\|A0A024RDQ0\|A0A024RDQ0_HUMAN | 718 |
| DNAJC17 | HEL-S-72p | sp\|Q9NVM6\|DJC17_HUMAN | tr\|V9HW22\|V9HW22_HUMAN | 742 |
| DNAJC17 | HEL-S-103 | sp\|Q9NVM6\|DJC17_HUMAN | tr\|A8K5I0\|A8K5I0_HUMAN | 742 |
| DNAJC17 | HSPA9 | sp\|Q9NVM6\|DJC17_HUMAN | tr\|Q8N1C8\|Q8N1C8_HUMAN | 718 |
| DNAJC17 | HEL-S-89n | sp\|Q9NVM6\|DJC17_HUMAN | tr\|V9HWB4\|V9HWB4_HUMAN | 742 |
| KPNA2 | RANBP1 | tr\|Q6NVW7\|Q6NVW7_HUMAN | tr\|F6WQW2\|F6WQW2_HUMAN | 987 |
| KPNA2 | KPNB1 | tr\|Q6NVW7\|Q6NVW7_HUMAN | sp\|Q14974\|IMB1_HUMAN | 999 |
| KPNA2 | HNRPA1 | tr\|Q6NVW7\|Q6NVW7_HUMAN | tr\|A0A024RAZ7\|A0A024RAZ7_HUMAN | 520 |
| RPL23A | hCG_24487 | sp\|P62750\|RL23A_HUMAN | tr\|A0A024R261\|A0A024R261_HUMAN | 999 |
| RPL23A | RPS3A | sp\|P62750\|RL23A_HUMAN | tr\|Q6NXR8\|Q6NXR8_HUMAN | 999 |
| RPL23A | IARS | sp\|P62750\|RL23A_HUMAN | sp\|P41252\|SYIC_HUMAN | 679 |
| RPL23A | RPL7 | sp\|P62750\|RL23A_HUMAN | tr\|A0A024R814\|A0A024R814_HUMAN | 999 |
| RPL23A | RPL13 | sp\|P62750\|RL23A_HUMAN | tr\|Q6NZ55\|Q6NZ55_HUMAN | 999 |
| RPL23A | RPL5 | sp\|P62750\|RL23A_HUMAN | tr\|A2RUM7\|A2RUM7_HUMAN | 999 |
| RPL23A | RPS18 | sp\|P62750\|RL23A_HUMAN | sp\|P62269\|RS18_HUMAN | 989 |
| RPL23A | RPL6 | sp\|P62750\|RL23A_HUMAN | tr\|Q8N5Z7\|Q8N5Z7_HUMAN | 999 |
| RPL23A | HNRPA1 | sp\|P62750\|RL23A_HUMAN | tr\|A0A024RAZ7\|A0A024RAZ7_HUMAN | 420 |
| RPL23A | HNRPU | sp\|P62750\|RL23A_HUMAN | tr\|B4DLR3\|B4DLR3_HUMAN | 778 |
| RPL23A | RPS5 | sp\|P62750\|RL23A_HUMAN | tr\|A0A024R4Q8\|A0A024R4Q8_HUMAN | 999 |
| RPL23A | SRP14 | sp\|P62750\|RL23A_HUMAN | sp\|P37108\|SRP14_HUMAN | 934 |
| RPL23A | RPS6 | sp\|P62750\|RL23A_HUMAN | tr\|A2A3R6\|A2A3R6_HUMAN | 999 |
| RPL23A | TUFM | sp\|P62750\|RL23A_HUMAN | sp\|P49411\|EFTU_HUMAN | 845 |
| RPL23A | RPS9 | sp\|P62750\|RL23A_HUMAN | tr\|A0A024R4M0\|A0A024R4M0_HUMAN | 986 |
| RPL23A | HEL-S-123m | sp\|P62750\|RL23A_HUMAN | tr\|V9HW26\|V9HW26_HUMAN | 621 |
| RPL23A | RPS8 | sp\|P62750\|RL23A_HUMAN | tr\|Q5JR94\|Q5JR94_HUMAN | 999 |
| RPL23A | RPL22 | sp\|P62750\|RL23A_HUMAN | sp\|P35268\|RL22_HUMAN | 999 |
| RPL23A | DDX17 | sp\|P62750\|RL23A_HUMAN | sp\|Q92841\|DDX17_HUMAN | 646 |
| RPL23A | HEL103 | sp\|P62750\|RL23A_HUMAN | tr\|V9HWK0\|V9HWK0_HUMAN | 931 |
| RPL23A | EEF1G | sp\|P62750\|RL23A_HUMAN | tr\|Q53YD7\|Q53YD7_HUMAN | 587 |
| RPL23A | GNB2L1 | sp\|P62750\|RL23A_HUMAN | tr\|E9KL35\|E9KL35_HUMAN | 974 |
| RPL23A | RPL26 | sp\|P62750\|RL23A_HUMAN | sp\|P61254\|RL26_HUMAN | 999 |
| RPL23A | RPL10A | sp\|P62750\|RL23A_HUMAN | sp\|P62906\|RL10A_HUMAN | 999 |
| RPL23A | RPS13 | sp\|P62750\|RL23A_HUMAN | sp\|P62277\|RS13_HUMAN | 999 |
| RPL23A | RPS7 | sp\|P62750\|RL23A_HUMAN | sp\|P62081\|RS7_HUMAN | 998 |
| RPL23A | RPS4X | sp\|P62750\|RL23A_HUMAN | tr\|B2R491\|B2R491_HUMAN | 999 |
| RPL23A | RPL19 | sp\|P62750\|RL23A_HUMAN | tr\|Q53G49\|Q53G49_HUMAN | 999 |
| RPL23A | RPL18 | sp\|P62750\|RL23A_HUMAN | tr\|J3QQ67\|J3QQ67_HUMAN | 999 |
| RPL23A | RPS3 | sp\|P62750\|RL23A_HUMAN | sp\|P23396\|RS3_HUMAN | 999 |
| RPL23A | RPS27A | sp\|P62750\|RL23A_HUMAN | tr\|B2RDW1\|B2RDW1_HUMAN | 999 |
| RPL23A | EL52 | sp\|P62750\|RL23A_HUMAN | tr\|K9JA46\|K9JA46_HUMAN | 640 |
| RPL23A | RAD23B | sp\|P62750\|RL23A_HUMAN | sp\|P54727\|RD23B_HUMAN | 507 |
| RPL23A | HEL-S-271 | sp\|P62750\|RL23A_HUMAN | tr\|V9HW31\|V9HW31_HUMAN | 402 |
| RPL23A | RPLP2 | sp\|P62750\|RL23A_HUMAN | tr\|A0A024RCA7\|A0A024RCA7_HUMAN | 999 |
| RPL23A | RPL3 | sp\|P62750\|RL23A_HUMAN | sp\|P39023\|RL3_HUMAN | 999 |
| RPL23A | ATP5O | sp\|P62750\|RL23A_HUMAN | sp\|P48047\|ATPO_HUMAN | 688 |
| RPL23A | RPS19 | sp\|P62750\|RL23A_HUMAN | tr\|Q8WVX7\|Q8WVX7_HUMAN | 998 |
| TUFM | hCG_24487 | sp\|P49411\|EFTU_HUMAN | tr\|A0A024R261\|A0A024R261_HUMAN | 951 |
| TUFM | GNB2 | sp\|P49411\|EFTU_HUMAN | tr\|Q6FHM2\|Q6FHM2_HUMAN | 515 |
| TUFM | RPL7 | sp\|P49411\|EFTU_HUMAN | tr\|A0A024R814\|A0A024R814_HUMAN | 839 |
| TUFM | PHB2 | sp\|P49411\|EFTU_HUMAN | sp\|Q99623\|PHB2_HUMAN | 732 |
| TUFM | RPL5 | sp\|P49411\|EFTU_HUMAN | tr\|A2RUM7\|A2RUM7_HUMAN | 729 |
| TUFM | RPS18 | sp\|P49411\|EFTU_HUMAN | sp\|P62269\|RS18_HUMAN | 827 |
| TUFM | TRAP1 | sp\|P49411\|EFTU_HUMAN | tr\|Q59EK6\|Q59EK6_HUMAN | 481 |
| TUFM | RPL6 | sp\|P49411\|EFTU_HUMAN | tr\|Q8N5Z7\|Q8N5Z7_HUMAN | 685 |
| TUFM | DLD | sp\|P49411\|EFTU_HUMAN | tr\|A0A024R713\|A0A024R713_HUMAN | 423 |
| TUFM | RPS5 | sp\|P49411\|EFTU_HUMAN | tr\|A0A024R4Q8\|A0A024R4Q8_HUMAN | 970 |
| TUFM | SNCG | sp\|P49411\|EFTU_HUMAN | tr\|Q6FHG5\|Q6FHG5_HUMAN | 644 |
| TUFM | RPS6 | sp\|P49411\|EFTU_HUMAN | tr\|A2A3R6\|A2A3R6_HUMAN | 731 |
| TUFM | HEL-S-100n | sp\|P49411\|EFTU_HUMAN | tr\|V9HW96\|V9HW96_HUMAN | 598 |
| TUFM | ERP70 | sp\|P49411\|EFTU_HUMAN | tr\|A0A090N8Y2\|A0A090N8Y2_HUMAN | 551 |
| TUFM | HSPA9 | sp\|P49411\|EFTU_HUMAN | tr\|Q8N1C8\|Q8N1C8_HUMAN | 537 |
| TUFM | RPS9 | sp\|P49411\|EFTU_HUMAN | tr\|A0A024R4M0\|A0A024R4M0_HUMAN | 846 |
| TUFM | HEL-S-162eP | sp\|P49411\|EFTU_HUMAN | tr\|V9HVZ4\|V9HVZ4_HUMAN | 611 |
| TUFM | HEL-S-123m | sp\|P49411\|EFTU_HUMAN | tr\|V9HW26\|V9HW26_HUMAN | 688 |
| TUFM | RPS8 | sp\|P49411\|EFTU_HUMAN | tr\|Q5JR94\|Q5JR94_HUMAN | 645 |
| TUFM | RPL22 | sp\|P49411\|EFTU_HUMAN | sp\|P35268\|RL22_HUMAN | 619 |
| TUFM | ENO1 | sp\|P49411\|EFTU_HUMAN | tr\|A0A024R4F1\|A0A024R4F1_HUMAN | 444 |
| TUFM | RPL26 | sp\|P49411\|EFTU_HUMAN | sp\|P61254\|RL26_HUMAN | 628 |
| TUFM | RPL10A | sp\|P49411\|EFTU_HUMAN | sp\|P62906\|RL10A_HUMAN | 828 |
| TUFM | RPS13 | sp\|P49411\|EFTU_HUMAN | sp\|P62277\|RS13_HUMAN | 905 |
| TUFM | RPL19 | sp\|P49411\|EFTU_HUMAN | tr\|Q53G49\|Q53G49_HUMAN | 640 |
| TUFM | EL52 | sp\|P49411\|EFTU_HUMAN | tr\|K9JA46\|K9JA46_HUMAN | 776 |
| TUFM | RPS3 | sp\|P49411\|EFTU_HUMAN | sp\|P23396\|RS3_HUMAN | 979 |
| TUFM | CCT4 | sp\|P49411\|EFTU_HUMAN | sp\|P50991\|TCPD_HUMAN | 607 |
| TUFM | HEL-S-271 | sp\|P49411\|EFTU_HUMAN | tr\|V9HW31\|V9HW31_HUMAN | 668 |
| TUFM | RPL3 | sp\|P49411\|EFTU_HUMAN | sp\|P39023\|RL3_HUMAN | 980 |
| TUFM | HSP90AB1 | sp\|P49411\|EFTU_HUMAN | tr\|A0A024RD80\|A0A024RD80_HUMAN | 481 |
| TUFM | ATP5O | sp\|P49411\|EFTU_HUMAN | sp\|P48047\|ATPO_HUMAN | 422 |
| ANXA3 | HEL-S-274 | sp\|P12429\|ANXA3_HUMAN | tr\|V9HW59\|V9HW59_HUMAN | 651 |
| RPL22 | DHX15 | sp\|P35268\|RL22_HUMAN | sp\|O43143\|DHX15_HUMAN | 580 |
| RPL22 | hCG_24487 | sp\|P35268\|RL22_HUMAN | tr\|A0A024R261\|A0A024R261_HUMAN | 999 |
| RPL22 | RPS9 | sp\|P35268\|RL22_HUMAN | tr\|A0A024R4M0\|A0A024R4M0_HUMAN | 748 |
| RPL22 | RPS3A | sp\|P35268\|RL22_HUMAN | tr\|Q6NXR8\|Q6NXR8_HUMAN | 997 |
| RPL22 | RPS8 | sp\|P35268\|RL22_HUMAN | tr\|Q5JR94\|Q5JR94_HUMAN | 998 |
| RPL22 | RPL7 | sp\|P35268\|RL22_HUMAN | tr\|A0A024R814\|A0A024R814_HUMAN | 999 |
| RPL22 | PHB2 | sp\|P35268\|RL22_HUMAN | sp\|Q99623\|PHB2_HUMAN | 638 |
| RPL22 | RPL13 | sp\|P35268\|RL22_HUMAN | tr\|Q6NZ55\|Q6NZ55_HUMAN | 999 |
| RPL22 | EEF1G | sp\|P35268\|RL22_HUMAN | tr\|Q53YD7\|Q53YD7_HUMAN | 553 |
| RPL22 | RPL5 | sp\|P35268\|RL22_HUMAN | tr\|A2RUM7\|A2RUM7_HUMAN | 999 |
| RPL22 | HEL103 | sp\|P35268\|RL22_HUMAN | tr\|V9HWK0\|V9HWK0_HUMAN | 900 |
| RPL22 | RPL26 | sp\|P35268\|RL22_HUMAN | sp\|P61254\|RL26_HUMAN | 999 |
| RPL22 | RPS18 | sp\|P35268\|RL22_HUMAN | sp\|P62269\|RS18_HUMAN | 705 |
| RPL22 | GNB2L1 | sp\|P35268\|RL22_HUMAN | tr\|E9KL35\|E9KL35_HUMAN | 838 |
| RPL22 | RPL10A | sp\|P35268\|RL22_HUMAN | sp\|P62906\|RL10A_HUMAN | 999 |
| RPL22 | RPL6 | sp\|P35268\|RL22_HUMAN | tr\|Q8N5Z7\|Q8N5Z7_HUMAN | 999 |
| RPL22 | RPS7 | sp\|P35268\|RL22_HUMAN | sp\|P62081\|RS7_HUMAN | 970 |
| RPL22 | RPS13 | sp\|P35268\|RL22_HUMAN | sp\|P62277\|RS13_HUMAN | 998 |
| RPL22 | RPS4X | sp\|P35268\|RL22_HUMAN | tr\|B2R491\|B2R491_HUMAN | 997 |
| RPL22 | RPL19 | sp\|P35268\|RL22_HUMAN | tr\|Q53G49\|Q53G49_HUMAN | 999 |
| RPL22 | RPL18 | sp\|P35268\|RL22_HUMAN | tr\|J3QQ67\|J3QQ67_HUMAN | 999 |
| RPL22 | RPS3 | sp\|P35268\|RL22_HUMAN | sp\|P23396\|RS3_HUMAN | 998 |
| RPL22 | RPS27A | sp\|P35268\|RL22_HUMAN | tr\|B2RDW1\|B2RDW1_HUMAN | 999 |
| RPL22 | EL52 | sp\|P35268\|RL22_HUMAN | tr\|K9JA46\|K9JA46_HUMAN | 598 |
| RPL22 | STRAP | sp\|P35268\|RL22_HUMAN | sp\|Q9Y3F4\|STRAP_HUMAN | 893 |
| RPL22 | RAD23B | sp\|P35268\|RL22_HUMAN | sp\|P54727\|RD23B_HUMAN | 419 |
| RPL22 | RPS5 | sp\|P35268\|RL22_HUMAN | tr\|A0A024R4Q8\|A0A024R4Q8_HUMAN | 974 |
| RPL22 | RPLP2 | sp\|P35268\|RL22_HUMAN | tr\|A0A024RCA7\|A0A024RCA7_HUMAN | 998 |
| RPL22 | RPL3 | sp\|P35268\|RL22_HUMAN | sp\|P39023\|RL3_HUMAN | 999 |
| RPL22 | SRP14 | sp\|P35268\|RL22_HUMAN | sp\|P37108\|SRP14_HUMAN | 921 |
| RPL22 | RPS6 | sp\|P35268\|RL22_HUMAN | tr\|A2A3R6\|A2A3R6_HUMAN | 998 |
| RPL22 | RPS19 | sp\|P35268\|RL22_HUMAN | tr\|Q8WVX7\|Q8WVX7_HUMAN | 998 |
| ENO1 | IARS | tr\|A0A024R4F1\|A0A024R4F1_HUMAN | sp\|P41252\|SYIC_HUMAN | 456 |
| ENO1 | VDAC2 | tr\|A0A024R4F1\|A0A024R4F1_HUMAN | tr\|A0A024QZN9\|A0A024QZN9_HUMAN | 487 |
| ENO1 | HEL-S-103 | tr\|A0A024R4F1\|A0A024R4F1_HUMAN | tr\|A8K5I0\|A8K5I0_HUMAN | 488 |
| ENO1 | HEL-S-89n | tr\|A0A024R4F1\|A0A024R4F1_HUMAN | tr\|V9HWB4\|V9HWB4_HUMAN | 606 |
| ENO1 | RPL5 | tr\|A0A024R4F1\|A0A024R4F1_HUMAN | tr\|A2RUM7\|A2RUM7_HUMAN | 401 |
| ENO1 | RPS18 | tr\|A0A024R4F1\|A0A024R4F1_HUMAN | sp\|P62269\|RS18_HUMAN | 491 |
| ENO1 | PRDX1 | tr\|A0A024R4F1\|A0A024R4F1_HUMAN | sp\|Q06830\|PRDX1_HUMAN | 563 |
| ENO1 | TRAP1 | tr\|A0A024R4F1\|A0A024R4F1_HUMAN | tr\|Q59EK6\|Q59EK6_HUMAN | 456 |
| ENO1 | IMPDH2 | tr\|A0A024R4F1\|A0A024R4F1_HUMAN | tr\|H0Y4R1\|H0Y4R1_HUMAN | 755 |
| ENO1 | TPI1 | tr\|A0A024R4F1\|A0A024R4F1_HUMAN | sp\|P60174\|TPIS_HUMAN | 991 |
| ENO1 | DLD | tr\|A0A024R4F1\|A0A024R4F1_HUMAN | tr\|A0A024R713\|A0A024R713_HUMAN | 770 |
| ENO1 | TARS | tr\|A0A024R4F1\|A0A024R4F1_HUMAN | tr\|Q53GX7\|Q53GX7_HUMAN | 475 |
| ENO1 | TALDO1 | tr\|A0A024R4F1\|A0A024R4F1_HUMAN | tr\|A0A140VK56\|A0A140VK56_HUMAN | 642 |
| ENO1 | TKT | tr\|A0A024R4F1\|A0A024R4F1_HUMAN | tr\|Q53EM5\|Q53EM5_HUMAN | 677 |
| ENO1 | P4HB | tr\|A0A024R4F1\|A0A024R4F1_HUMAN | tr\|A0A024R8S5\|A0A024R8S5_HUMAN | 664 |
| ENO1 | PYGB | tr\|A0A024R4F1\|A0A024R4F1_HUMAN | sp\|P11216\|PYGB_HUMAN | 794 |
| ENO1 | HEL-S-100n | tr\|A0A024R4F1\|A0A024R4F1_HUMAN | tr\|V9HW96\|V9HW96_HUMAN | 558 |
| ENO1 | HEL-S-2a | tr\|A0A024R4F1\|A0A024R4F1_HUMAN | tr\|V9HW12\|V9HW12_HUMAN | 498 |
| ENO1 | ERP70 | tr\|A0A024R4F1\|A0A024R4F1_HUMAN | tr\|A0A090N8Y2\|A0A090N8Y2_HUMAN | 409 |
| ENO1 | HSPA9 | tr\|A0A024R4F1\|A0A024R4F1_HUMAN | tr\|Q8N1C8\|Q8N1C8_HUMAN | 629 |
| ENO1 | HEL-S-133P | tr\|A0A024R4F1\|A0A024R4F1_HUMAN | tr\|V9HWB9\|V9HWB9_HUMAN | 907 |
| ENO1 | RPS9 | tr\|A0A024R4F1\|A0A024R4F1_HUMAN | tr\|A0A024R4M0\|A0A024R4M0_HUMAN | 483 |
| ENO1 | HEL-S-26 | tr\|A0A024R4F1\|A0A024R4F1_HUMAN | tr\|V9HWJ2\|V9HWJ2_HUMAN | 552 |
| ENO1 | HEL-S-72p | tr\|A0A024R4F1\|A0A024R4F1_HUMAN | tr\|V9HW22\|V9HW22_HUMAN | 676 |
| ENO1 | HEL-S-162eP | tr\|A0A024R4F1\|A0A024R4F1_HUMAN | tr\|V9HVZ4\|V9HVZ4_HUMAN | 999 |
| ENO1 | HADHA | tr\|A0A024R4F1\|A0A024R4F1_HUMAN | tr\|E9KL44\|E9KL44_HUMAN | 500 |
| ENO1 | HEL-S-123m | tr\|A0A024R4F1\|A0A024R4F1_HUMAN | tr\|V9HW26\|V9HW26_HUMAN | 699 |
| ENO1 | GLUD1 | tr\|A0A024R4F1\|A0A024R4F1_HUMAN | tr\|E9KL48\|E9KL48_HUMAN | 655 |
| ENO1 | HEL-S-53e | tr\|A0A024R4F1\|A0A024R4F1_HUMAN | tr\|V9HW83\|V9HW83_HUMAN | 493 |
| ENO1 | HADH | tr\|A0A024R4F1\|A0A024R4F1_HUMAN | tr\|B2RB06\|B2RB06_HUMAN | 475 |
| ENO1 | EEF1G | tr\|A0A024R4F1\|A0A024R4F1_HUMAN | tr\|Q53YD7\|Q53YD7_HUMAN | 797 |
| ENO1 | GNB2L1 | tr\|A0A024R4F1\|A0A024R4F1_HUMAN | tr\|E9KL35\|E9KL35_HUMAN | 532 |
| ENO1 | HSPH1 | tr\|A0A024R4F1\|A0A024R4F1_HUMAN | tr\|A0A024RDQ0\|A0A024RDQ0_HUMAN | 528 |
| ENO1 | VDAC1 | tr\|A0A024R4F1\|A0A024R4F1_HUMAN | sp\|P21796\|VDAC1_HUMAN | 473 |
| ENO1 | LDHB | tr\|A0A024R4F1\|A0A024R4F1_HUMAN | tr\|Q5U077\|Q5U077_HUMAN | 834 |
| ENO1 | RPL18 | tr\|A0A024R4F1\|A0A024R4F1_HUMAN | tr\|J3QQ67\|J3QQ67_HUMAN | 592 |
| ENO1 | RPS3 | tr\|A0A024R4F1\|A0A024R4F1_HUMAN | sp\|P23396\|RS3_HUMAN | 456 |
| ENO1 | EL52 | tr\|A0A024R4F1\|A0A024R4F1_HUMAN | tr\|K9JA46\|K9JA46_HUMAN | 743 |
| ENO1 | HEL-S-269 | tr\|A0A024R4F1\|A0A024R4F1_HUMAN | tr\|V9HVY3\|V9HVY3_HUMAN | 579 |
| ENO1 | PDIA6 | tr\|A0A024R4F1\|A0A024R4F1_HUMAN | sp\|Q15084\|PDIA6_HUMAN | 472 |
| ENO1 | HEL-S-15 | tr\|A0A024R4F1\|A0A024R4F1_HUMAN | tr\|V9HWI5\|V9HWI5_HUMAN | 619 |
| ENO1 | MDH2 | tr\|A0A024R4F1\|A0A024R4F1_HUMAN | tr\|Q6FHZ0\|Q6FHZ0_HUMAN | 880 |
| ENO1 | HEL-S-68p | tr\|A0A024R4F1\|A0A024R4F1_HUMAN | tr\|V9HWF4\|V9HWF4_HUMAN | 992 |
| ENO1 | HEL-S-271 | tr\|A0A024R4F1\|A0A024R4F1_HUMAN | tr\|V9HW31\|V9HW31_HUMAN | 576 |
| ENO1 | CCT4 | tr\|A0A024R4F1\|A0A024R4F1_HUMAN | sp\|P50991\|TCPD_HUMAN | 546 |
| ENO1 | RPL3 | tr\|A0A024R4F1\|A0A024R4F1_HUMAN | sp\|P39023\|RL3_HUMAN | 483 |
| ENO1 | HEL-S-128m | tr\|A0A024R4F1\|A0A024R4F1_HUMAN | tr\|V9HWC7\|V9HWC7_HUMAN | 448 |
| ENO1 | HSP90AB1 | tr\|A0A024R4F1\|A0A024R4F1_HUMAN | tr\|A0A024RD80\|A0A024RD80_HUMAN | 748 |
| ENO1 | RPS19 | tr\|A0A024R4F1\|A0A024R4F1_HUMAN | tr\|Q8WVX7\|Q8WVX7_HUMAN | 404 |
| DDX17 | DHX15 | sp\|Q92841\|DDX17_HUMAN | sp\|O43143\|DHX15_HUMAN | 659 |
| DDX17 | ANXA1 | sp\|Q92841\|DDX17_HUMAN | tr\|Q5TZZ9\|Q5TZZ9_HUMAN | 569 |
| DDX17 | SRSF1 | sp\|Q92841\|DDX17_HUMAN | sp\|Q07955\|SRSF1_HUMAN | 809 |
| DDX17 | SSB | sp\|Q92841\|DDX17_HUMAN | tr\|B5BUB5\|B5BUB5_HUMAN | 458 |
| DDX17 | HEL-S-72p | sp\|Q92841\|DDX17_HUMAN | tr\|V9HW22\|V9HW22_HUMAN | 471 |
| DDX17 | HNRNPA2B1 | sp\|Q92841\|DDX17_HUMAN | sp\|P22626\|ROA2_HUMAN | 515 |
| DDX17 | HNRNPAB | sp\|Q92841\|DDX17_HUMAN | tr\|D6RD18\|D6RD18_HUMAN | 411 |
| DDX17 | RPL3 | sp\|Q92841\|DDX17_HUMAN | sp\|P39023\|RL3_HUMAN | 550 |
| DDX17 | RPL6 | sp\|Q92841\|DDX17_HUMAN | tr\|Q8N5Z7\|Q8N5Z7_HUMAN | 540 |
| DDX17 | HEL-S-100n | sp\|Q92841\|DDX17_HUMAN | tr\|V9HW96\|V9HW96_HUMAN | 413 |
| DDX17 | HNRPA1 | sp\|Q92841\|DDX17_HUMAN | tr\|A0A024RAZ7\|A0A024RAZ7_HUMAN | 829 |
| DDX17 | HSPA9 | sp\|Q92841\|DDX17_HUMAN | tr\|Q8N1C8\|Q8N1C8_HUMAN | 405 |
| DDX17 | IMPDH2 | sp\|Q92841\|DDX17_HUMAN | tr\|H0Y4R1\|H0Y4R1_HUMAN | 461 |
| GNB2L1 | hCG_24487 | tr\|E9KL35\|E9KL35_HUMAN | tr\|A0A024R261\|A0A024R261_HUMAN | 970 |
| GNB2L1 | RPS3A | tr\|E9KL35\|E9KL35_HUMAN | tr\|Q6NXR8\|Q6NXR8_HUMAN | 999 |
| GNB2L1 | HEL-S-89n | tr\|E9KL35\|E9KL35_HUMAN | tr\|V9HWB4\|V9HWB4_HUMAN | 456 |
| GNB2L1 | RPL7 | tr\|E9KL35\|E9KL35_HUMAN | tr\|A0A024R814\|A0A024R814_HUMAN | 989 |
| GNB2L1 | RPL13 | tr\|E9KL35\|E9KL35_HUMAN | tr\|Q6NZ55\|Q6NZ55_HUMAN | 998 |
| GNB2L1 | RPL5 | tr\|E9KL35\|E9KL35_HUMAN | tr\|A2RUM7\|A2RUM7_HUMAN | 992 |
| GNB2L1 | RPS18 | tr\|E9KL35\|E9KL35_HUMAN | sp\|P62269\|RS18_HUMAN | 984 |
| GNB2L1 | RPL6 | tr\|E9KL35\|E9KL35_HUMAN | tr\|Q8N5Z7\|Q8N5Z7_HUMAN | 980 |
| GNB2L1 | CDH1 | tr\|E9KL35\|E9KL35_HUMAN | tr\|D3XNU5\|D3XNU5_HUMAN | 665 |
| GNB2L1 | IMPDH2 | tr\|E9KL35\|E9KL35_HUMAN | tr\|H0Y4R1\|H0Y4R1_HUMAN | 475 |
| GNB2L1 | CSTB | tr\|E9KL35\|E9KL35_HUMAN | tr\|Q76LA1\|Q76LA1_HUMAN | 416 |
| GNB2L1 | RPS5 | tr\|E9KL35\|E9KL35_HUMAN | tr\|A0A024R4Q8\|A0A024R4Q8_HUMAN | 999 |
| GNB2L1 | RPS6 | tr\|E9KL35\|E9KL35_HUMAN | tr\|A2A3R6\|A2A3R6_HUMAN | 999 |
| GNB2L1 | HEL-S-100n | tr\|E9KL35\|E9KL35_HUMAN | tr\|V9HW96\|V9HW96_HUMAN | 697 |
| GNB2L1 | GSPT1 | tr\|E9KL35\|E9KL35_HUMAN | tr\|B2RCT6\|B2RCT6_HUMAN | 606 |
| GNB2L1 | RPS9 | tr\|E9KL35\|E9KL35_HUMAN | tr\|A0A024R4M0\|A0A024R4M0_HUMAN | 999 |
| GNB2L1 | HSD17B4 | tr\|E9KL35\|E9KL35_HUMAN | tr\|B2R659\|B2R659_HUMAN | 644 |
| GNB2L1 | HEL-S-72p | tr\|E9KL35\|E9KL35_HUMAN | tr\|V9HW22\|V9HW22_HUMAN | 504 |
| GNB2L1 | HEL-S-162eP | tr\|E9KL35\|E9KL35_HUMAN | tr\|V9HVZ4\|V9HVZ4_HUMAN | 452 |
| GNB2L1 | RPS8 | tr\|E9KL35\|E9KL35_HUMAN | tr\|Q5JR94\|Q5JR94_HUMAN | 999 |
| GNB2L1 | EEF1G | tr\|E9KL35\|E9KL35_HUMAN | tr\|Q53YD7\|Q53YD7_HUMAN | 944 |
| GNB2L1 | HEL-S-94n | tr\|E9KL35\|E9KL35_HUMAN | tr\|V9HW72\|V9HW72_HUMAN | 486 |
| GNB2L1 | RPL26 | tr\|E9KL35\|E9KL35_HUMAN | sp\|P61254\|RL26_HUMAN | 963 |
| GNB2L1 | RPL10A | tr\|E9KL35\|E9KL35_HUMAN | sp\|P62906\|RL10A_HUMAN | 961 |
| GNB2L1 | RPS7 | tr\|E9KL35\|E9KL35_HUMAN | sp\|P62081\|RS7_HUMAN | 999 |
| GNB2L1 | RPS13 | tr\|E9KL35\|E9KL35_HUMAN | sp\|P62277\|RS13_HUMAN | 999 |
| GNB2L1 | RPS4X | tr\|E9KL35\|E9KL35_HUMAN | tr\|B2R491\|B2R491_HUMAN | 999 |
| GNB2L1 | RPL19 | tr\|E9KL35\|E9KL35_HUMAN | tr\|Q53G49\|Q53G49_HUMAN | 994 |
| GNB2L1 | RPL18 | tr\|E9KL35\|E9KL35_HUMAN | tr\|J3QQ67\|J3QQ67_HUMAN | 983 |
| GNB2L1 | RPS3 | tr\|E9KL35\|E9KL35_HUMAN | sp\|P23396\|RS3_HUMAN | 999 |
| GNB2L1 | RPS27A | tr\|E9KL35\|E9KL35_HUMAN | tr\|B2RDW1\|B2RDW1_HUMAN | 990 |
| GNB2L1 | EL52 | tr\|E9KL35\|E9KL35_HUMAN | tr\|K9JA46\|K9JA46_HUMAN | 957 |
| GNB2L1 | TRA1 | tr\|E9KL35\|E9KL35_HUMAN | tr\|Q5CAQ5\|Q5CAQ5_HUMAN | 699 |
| GNB2L1 | RAD23B | tr\|E9KL35\|E9KL35_HUMAN | sp\|P54727\|RD23B_HUMAN | 477 |
| GNB2L1 | CCT4 | tr\|E9KL35\|E9KL35_HUMAN | sp\|P50991\|TCPD_HUMAN | 492 |
| GNB2L1 | RPLP2 | tr\|E9KL35\|E9KL35_HUMAN | tr\|A0A024RCA7\|A0A024RCA7_HUMAN | 976 |
| GNB2L1 | RPL3 | tr\|E9KL35\|E9KL35_HUMAN | sp\|P39023\|RL3_HUMAN | 990 |
| GNB2L1 | HMGN2 | tr\|E9KL35\|E9KL35_HUMAN | sp\|P05204\|HMGN2_HUMAN | 489 |
| GNB2L1 | RPS19 | tr\|E9KL35\|E9KL35_HUMAN | tr\|Q8WVX7\|Q8WVX7_HUMAN | 999 |
| HSPH1 | IARS | tr\|A0A024RDQ0\|A0A024RDQ0_HUMAN | sp\|P41252\|SYIC_HUMAN | 402 |
| HSPH1 | HEL-S-103 | tr\|A0A024RDQ0\|A0A024RDQ0_HUMAN | tr\|A8K5I0\|A8K5I0_HUMAN | 565 |
| HSPH1 | HEL-S-89n | tr\|A0A024RDQ0\|A0A024RDQ0_HUMAN | tr\|V9HWB4\|V9HWB4_HUMAN | 553 |
| HSPH1 | TRAP1 | tr\|A0A024RDQ0\|A0A024RDQ0_HUMAN | tr\|Q59EK6\|Q59EK6_HUMAN | 773 |
| HSPH1 | PRDX1 | tr\|A0A024RDQ0\|A0A024RDQ0_HUMAN | sp\|Q06830\|PRDX1_HUMAN | 449 |
| HSPH1 | CANX | tr\|A0A024RDQ0\|A0A024RDQ0_HUMAN | sp\|P27824\|CALX_HUMAN | 547 |
| HSPH1 | IMPDH2 | tr\|A0A024RDQ0\|A0A024RDQ0_HUMAN | tr\|H0Y4R1\|H0Y4R1_HUMAN | 640 |
| HSPH1 | STUB1 | tr\|A0A024RDQ0\|A0A024RDQ0_HUMAN | sp\|Q9UNE7\|CHIP_HUMAN | 437 |
| HSPH1 | TARS | tr\|A0A024RDQ0\|A0A024RDQ0_HUMAN | tr\|Q53GX7\|Q53GX7_HUMAN | 407 |
| HSPH1 | TKT | tr\|A0A024RDQ0\|A0A024RDQ0_HUMAN | tr\|Q53EM5\|Q53EM5_HUMAN | 454 |
| HSPH1 | P4HB | tr\|A0A024RDQ0\|A0A024RDQ0_HUMAN | tr\|A0A024R8S5\|A0A024R8S5_HUMAN | 426 |
| HSPH1 | HEL-S-100n | tr\|A0A024RDQ0\|A0A024RDQ0_HUMAN | tr\|V9HW96\|V9HW96_HUMAN | 771 |
| HSPH1 | HEL-S-2a | tr\|A0A024RDQ0\|A0A024RDQ0_HUMAN | tr\|V9HW12\|V9HW12_HUMAN | 490 |
| HSPH1 | ERP70 | tr\|A0A024RDQ0\|A0A024RDQ0_HUMAN | tr\|A0A090N8Y2\|A0A090N8Y2_HUMAN | 471 |
| HSPH1 | GSPT1 | tr\|A0A024RDQ0\|A0A024RDQ0_HUMAN | tr\|B2RCT6\|B2RCT6_HUMAN | 605 |
| HSPH1 | HSPA9 | tr\|A0A024RDQ0\|A0A024RDQ0_HUMAN | tr\|Q8N1C8\|Q8N1C8_HUMAN | 776 |
| HSPH1 | RPS9 | tr\|A0A024RDQ0\|A0A024RDQ0_HUMAN | tr\|A0A024R4M0\|A0A024R4M0_HUMAN | 438 |
| HSPH1 | PAFAH1B1 | tr\|A0A024RDQ0\|A0A024RDQ0_HUMAN | sp\|P43034\|LIS1_HUMAN | 613 |
| HSPH1 | HEL-S-72p | tr\|A0A024RDQ0\|A0A024RDQ0_HUMAN | tr\|V9HW22\|V9HW22_HUMAN | 983 |
| HSPH1 | HEL-S-162eP | tr\|A0A024RDQ0\|A0A024RDQ0_HUMAN | tr\|V9HVZ4\|V9HVZ4_HUMAN | 512 |
| HSPH1 | HEL-S-94n | tr\|A0A024RDQ0\|A0A024RDQ0_HUMAN | tr\|V9HW72\|V9HW72_HUMAN | 997 |
| HSPH1 | EEF1G | tr\|A0A024RDQ0\|A0A024RDQ0_HUMAN | tr\|Q53YD7\|Q53YD7_HUMAN | 426 |
| HSPH1 | HEL2 | tr\|A0A024RDQ0\|A0A024RDQ0_HUMAN | tr\|V9HW98\|V9HW98_HUMAN | 921 |
| HSPH1 | HEL-S-70 | tr\|A0A024RDQ0\|A0A024RDQ0_HUMAN | tr\|V9HW80\|V9HW80_HUMAN | 566 |
| HSPH1 | RPS3 | tr\|A0A024RDQ0\|A0A024RDQ0_HUMAN | sp\|P23396\|RS3_HUMAN | 408 |
| HSPH1 | EL52 | tr\|A0A024RDQ0\|A0A024RDQ0_HUMAN | tr\|K9JA46\|K9JA46_HUMAN | 998 |
| HSPH1 | HEL-S-269 | tr\|A0A024RDQ0\|A0A024RDQ0_HUMAN | tr\|V9HVY3\|V9HVY3_HUMAN | 414 |
| HSPH1 | HEL-S-15 | tr\|A0A024RDQ0\|A0A024RDQ0_HUMAN | tr\|V9HWI5\|V9HWI5_HUMAN | 584 |
| HSPH1 | TRA1 | tr\|A0A024RDQ0\|A0A024RDQ0_HUMAN | tr\|Q5CAQ5\|Q5CAQ5_HUMAN | 920 |
| HSPH1 | PDIA6 | tr\|A0A024RDQ0\|A0A024RDQ0_HUMAN | sp\|Q15084\|PDIA6_HUMAN | 433 |
| HSPH1 | HEL-S-68p | tr\|A0A024RDQ0\|A0A024RDQ0_HUMAN | tr\|V9HWF4\|V9HWF4_HUMAN | 662 |
| HSPH1 | CCT4 | tr\|A0A024RDQ0\|A0A024RDQ0_HUMAN | sp\|P50991\|TCPD_HUMAN | 792 |
| HSPH1 | UBE1 | tr\|A0A024RDQ0\|A0A024RDQ0_HUMAN | tr\|A0A024R1A3\|A0A024R1A3_HUMAN | 735 |
| HSPH1 | RPL3 | tr\|A0A024RDQ0\|A0A024RDQ0_HUMAN | sp\|P39023\|RL3_HUMAN | 413 |
| HSPH1 | HEL-S-99n | tr\|A0A024RDQ0\|A0A024RDQ0_HUMAN | tr\|V9HW88\|V9HW88_HUMAN | 948 |
| HSPH1 | FARSB | tr\|A0A024RDQ0\|A0A024RDQ0_HUMAN | tr\|Q9BR63\|Q9BR63_HUMAN | 501 |
| HSPH1 | HEL-S-128m | tr\|A0A024RDQ0\|A0A024RDQ0_HUMAN | tr\|V9HWC7\|V9HWC7_HUMAN | 444 |
| HSPH1 | HSP90AB1 | tr\|A0A024RDQ0\|A0A024RDQ0_HUMAN | tr\|A0A024RD80\|A0A024RD80_HUMAN | 997 |
| RPL10A | hCG_24487 | sp\|P62906\|RL10A_HUMAN | tr\|A0A024R261\|A0A024R261_HUMAN | 999 |
| RPL10A | RPS3A | sp\|P62906\|RL10A_HUMAN | tr\|Q6NXR8\|Q6NXR8_HUMAN | 999 |
| RPL10A | HEL-S-103 | sp\|P62906\|RL10A_HUMAN | tr\|A8K5I0\|A8K5I0_HUMAN | 419 |
| RPL10A | HEL-S-89n | sp\|P62906\|RL10A_HUMAN | tr\|V9HWB4\|V9HWB4_HUMAN | 419 |
| RPL10A | RPL7 | sp\|P62906\|RL10A_HUMAN | tr\|A0A024R814\|A0A024R814_HUMAN | 999 |
| RPL10A | RPL13 | sp\|P62906\|RL10A_HUMAN | tr\|Q6NZ55\|Q6NZ55_HUMAN | 999 |
| RPL10A | RPL5 | sp\|P62906\|RL10A_HUMAN | tr\|A2RUM7\|A2RUM7_HUMAN | 999 |
| RPL10A | RPS18 | sp\|P62906\|RL10A_HUMAN | sp\|P62269\|RS18_HUMAN | 951 |
| RPL10A | RPL6 | sp\|P62906\|RL10A_HUMAN | tr\|Q8N5Z7\|Q8N5Z7_HUMAN | 999 |
| RPL10A | IMPDH2 | sp\|P62906\|RL10A_HUMAN | tr\|H0Y4R1\|H0Y4R1_HUMAN | 621 |
| RPL10A | RPS5 | sp\|P62906\|RL10A_HUMAN | tr\|A0A024R4Q8\|A0A024R4Q8_HUMAN | 999 |
| RPL10A | SRP14 | sp\|P62906\|RL10A_HUMAN | sp\|P37108\|SRP14_HUMAN | 900 |
| RPL10A | RPS6 | sp\|P62906\|RL10A_HUMAN | tr\|A2A3R6\|A2A3R6_HUMAN | 999 |
| RPL10A | HEL-S-100n | sp\|P62906\|RL10A_HUMAN | tr\|V9HW96\|V9HW96_HUMAN | 457 |
| RPL10A | RPS9 | sp\|P62906\|RL10A_HUMAN | tr\|A0A024R4M0\|A0A024R4M0_HUMAN | 968 |
| RPL10A | HEL-S-72p | sp\|P62906\|RL10A_HUMAN | tr\|V9HW22\|V9HW22_HUMAN | 478 |
| RPL10A | HEL-S-162eP | sp\|P62906\|RL10A_HUMAN | tr\|V9HVZ4\|V9HVZ4_HUMAN | 425 |
| RPL10A | HEL-S-123m | sp\|P62906\|RL10A_HUMAN | tr\|V9HW26\|V9HW26_HUMAN | 647 |
| RPL10A | RPS8 | sp\|P62906\|RL10A_HUMAN | tr\|Q5JR94\|Q5JR94_HUMAN | 999 |
| RPL10A | HEL103 | sp\|P62906\|RL10A_HUMAN | tr\|V9HWK0\|V9HWK0_HUMAN | 900 |
| RPL10A | EEF1G | sp\|P62906\|RL10A_HUMAN | tr\|Q53YD7\|Q53YD7_HUMAN | 692 |
| RPL10A | RPL26 | sp\|P62906\|RL10A_HUMAN | sp\|P61254\|RL26_HUMAN | 999 |
| RPL10A | RPS13 | sp\|P62906\|RL10A_HUMAN | sp\|P62277\|RS13_HUMAN | 999 |
| RPL10A | RPS7 | sp\|P62906\|RL10A_HUMAN | sp\|P62081\|RS7_HUMAN | 997 |
| RPL10A | RPS4X | sp\|P62906\|RL10A_HUMAN | tr\|B2R491\|B2R491_HUMAN | 999 |
| RPL10A | RPL19 | sp\|P62906\|RL10A_HUMAN | tr\|Q53G49\|Q53G49_HUMAN | 999 |
| RPL10A | RPL18 | sp\|P62906\|RL10A_HUMAN | tr\|J3QQ67\|J3QQ67_HUMAN | 999 |
| RPL10A | RPS27A | sp\|P62906\|RL10A_HUMAN | tr\|B2RDW1\|B2RDW1_HUMAN | 997 |
| RPL10A | RPS3 | sp\|P62906\|RL10A_HUMAN | sp\|P23396\|RS3_HUMAN | 999 |
| RPL10A | S100A10 | sp\|P62906\|RL10A_HUMAN | tr\|D3DV26\|D3DV26_HUMAN | 565 |
| RPL10A | RPLP2 | sp\|P62906\|RL10A_HUMAN | tr\|A0A024RCA7\|A0A024RCA7_HUMAN | 999 |
| RPL10A | RPL3 | sp\|P62906\|RL10A_HUMAN | sp\|P39023\|RL3_HUMAN | 999 |
| RPL10A | HSP90AB1 | sp\|P62906\|RL10A_HUMAN | tr\|A0A024RD80\|A0A024RD80_HUMAN | 635 |
| RPL10A | ATP5O | sp\|P62906\|RL10A_HUMAN | sp\|P48047\|ATPO_HUMAN | 532 |
| RPL10A | RPS19 | sp\|P62906\|RL10A_HUMAN | tr\|Q8WVX7\|Q8WVX7_HUMAN | 999 |
| VDAC1 | HEL-S-162eP | sp\|P21796\|VDAC1_HUMAN | tr\|V9HVZ4\|V9HVZ4_HUMAN | 635 |
| VDAC1 | HEL-S-123m | sp\|P21796\|VDAC1_HUMAN | tr\|V9HW26\|V9HW26_HUMAN | 666 |
| VDAC1 | VDAC2 | sp\|P21796\|VDAC1_HUMAN | tr\|A0A024QZN9\|A0A024QZN9_HUMAN | 905 |
| VDAC1 | PHB2 | sp\|P21796\|VDAC1_HUMAN | sp\|Q99623\|PHB2_HUMAN | 403 |
| VDAC1 | PRDX1 | sp\|P21796\|VDAC1_HUMAN | sp\|Q06830\|PRDX1_HUMAN | 509 |
| VDAC1 | ATP5H | sp\|P21796\|VDAC1_HUMAN | sp\|O75947\|ATP5H_HUMAN | 513 |
| VDAC1 | CANX | sp\|P21796\|VDAC1_HUMAN | sp\|P27824\|CALX_HUMAN | 756 |
| VDAC1 | EL52 | sp\|P21796\|VDAC1_HUMAN | tr\|K9JA46\|K9JA46_HUMAN | 668 |
| VDAC1 | RPS27A | sp\|P21796\|VDAC1_HUMAN | tr\|B2RDW1\|B2RDW1_HUMAN | 725 |
| VDAC1 | DLD | sp\|P21796\|VDAC1_HUMAN | tr\|A0A024R713\|A0A024R713_HUMAN | 448 |
| VDAC1 | TRA1 | sp\|P21796\|VDAC1_HUMAN | tr\|Q5CAQ5\|Q5CAQ5_HUMAN | 429 |
| VDAC1 | MDH2 | sp\|P21796\|VDAC1_HUMAN | tr\|Q6FHZ0\|Q6FHZ0_HUMAN | 655 |
| VDAC1 | RAB1A | sp\|P21796\|VDAC1_HUMAN | tr\|Q5U0I6\|Q5U0I6_HUMAN | 435 |
| VDAC1 | HEL-S-271 | sp\|P21796\|VDAC1_HUMAN | tr\|V9HW31\|V9HW31_HUMAN | 655 |
| VDAC1 | HSP90AB1 | sp\|P21796\|VDAC1_HUMAN | tr\|A0A024RD80\|A0A024RD80_HUMAN | 540 |
| VDAC1 | ATP5O | sp\|P21796\|VDAC1_HUMAN | sp\|P48047\|ATPO_HUMAN | 529 |
| VDAC1 | HEL-S-2a | sp\|P21796\|VDAC1_HUMAN | tr\|V9HW12\|V9HW12_HUMAN | 498 |
| VDAC1 | HSPA9 | sp\|P21796\|VDAC1_HUMAN | tr\|Q8N1C8\|Q8N1C8_HUMAN | 904 |
| PEBP1 | TKT | tr\|D9IAI1\|D9IAI1_HUMAN | tr\|Q53EM5\|Q53EM5_HUMAN | 462 |
| PEBP1 | PRDX1 | tr\|D9IAI1\|D9IAI1_HUMAN | sp\|Q06830\|PRDX1_HUMAN | 774 |
| PEBP1 | HEL-S-2a | tr\|D9IAI1\|D9IAI1_HUMAN | tr\|V9HW12\|V9HW12_HUMAN | 439 |
| LDHB | HEL-S-133P | tr\|Q5U077\|Q5U077_HUMAN | tr\|V9HWB9\|V9HWB9_HUMAN | 919 |
| LDHB | HEL-S-26 | tr\|Q5U077\|Q5U077_HUMAN | tr\|V9HWJ2\|V9HWJ2_HUMAN | 890 |
| LDHB | HADHA | tr\|Q5U077\|Q5U077_HUMAN | tr\|E9KL44\|E9KL44_HUMAN | 467 |
| LDHB | HEL-S-162eP | tr\|Q5U077\|Q5U077_HUMAN | tr\|V9HVZ4\|V9HVZ4_HUMAN | 854 |
| LDHB | HEL-S-72p | tr\|Q5U077\|Q5U077_HUMAN | tr\|V9HW22\|V9HW22_HUMAN | 485 |
| LDHB | HEL-S-123m | tr\|Q5U077\|Q5U077_HUMAN | tr\|V9HW26\|V9HW26_HUMAN | 460 |
| LDHB | GLUD1 | tr\|Q5U077\|Q5U077_HUMAN | tr\|E9KL48\|E9KL48_HUMAN | 585 |
| LDHB | HEL-S-53e | tr\|Q5U077\|Q5U077_HUMAN | tr\|V9HW83\|V9HW83_HUMAN | 415 |
| LDHB | HEL-S-89n | tr\|Q5U077\|Q5U077_HUMAN | tr\|V9HWB4\|V9HWB4_HUMAN | 427 |
| LDHB | PRDX1 | tr\|Q5U077\|Q5U077_HUMAN | sp\|Q06830\|PRDX1_HUMAN | 420 |
| LDHB | HEL-S-45 | tr\|Q5U077\|Q5U077_HUMAN | tr\|V9HWG3\|V9HWG3_HUMAN | 506 |
| LDHB | UBE2V1 | tr\|Q5U077\|Q5U077_HUMAN | sp\|Q13404\|UB2V1_HUMAN | 499 |
| LDHB | TPI1 | tr\|Q5U077\|Q5U077_HUMAN | sp\|P60174\|TPIS_HUMAN | 570 |
| LDHB | DLD | tr\|Q5U077\|Q5U077_HUMAN | tr\|A0A024R713\|A0A024R713_HUMAN | 596 |
| LDHB | HNRNPAB | tr\|Q5U077\|Q5U077_HUMAN | tr\|D6RD18\|D6RD18_HUMAN | 412 |
| LDHB | HNRNPA2B1 | tr\|Q5U077\|Q5U077_HUMAN | sp\|P22626\|ROA2_HUMAN | 427 |
| LDHB | HEL-S-68p | tr\|Q5U077\|Q5U077_HUMAN | tr\|V9HWF4\|V9HWF4_HUMAN | 511 |
| LDHB | HEL-S-271 | tr\|Q5U077\|Q5U077_HUMAN | tr\|V9HW31\|V9HW31_HUMAN | 500 |
| LDHB | CCT4 | tr\|Q5U077\|Q5U077_HUMAN | sp\|P50991\|TCPD_HUMAN | 533 |
| LDHB | TALDO1 | tr\|Q5U077\|Q5U077_HUMAN | tr\|A0A140VK56\|A0A140VK56_HUMAN | 876 |
| LDHB | FLJ00064 | tr\|Q5U077\|Q5U077_HUMAN | tr\|B4DV28\|B4DV28_HUMAN | 624 |
| LDHB | HEL-S-128m | tr\|Q5U077\|Q5U077_HUMAN | tr\|V9HWC7\|V9HWC7_HUMAN | 420 |
| LDHB | TKT | tr\|Q5U077\|Q5U077_HUMAN | tr\|Q53EM5\|Q53EM5_HUMAN | 896 |
| LDHB | PYGB | tr\|Q5U077\|Q5U077_HUMAN | sp\|P11216\|PYGB_HUMAN | 464 |
| LDHB | HEL-S-100n | tr\|Q5U077\|Q5U077_HUMAN | tr\|V9HW96\|V9HW96_HUMAN | 515 |
| LDHB | HEL-S-2a | tr\|Q5U077\|Q5U077_HUMAN | tr\|V9HW12\|V9HW12_HUMAN | 436 |
| LDHB | HSPA9 | tr\|Q5U077\|Q5U077_HUMAN | tr\|Q8N1C8\|Q8N1C8_HUMAN | 428 |
| RPS4X | hCG_24487 | tr\|B2R491\|B2R491_HUMAN | tr\|A0A024R261\|A0A024R261_HUMAN | 999 |
| RPS4X | RPS9 | tr\|B2R491\|B2R491_HUMAN | tr\|A0A024R4M0\|A0A024R4M0_HUMAN | 998 |
| RPS4X | RPS3A | tr\|B2R491\|B2R491_HUMAN | tr\|Q6NXR8\|Q6NXR8_HUMAN | 999 |
| RPS4X | HEL-S-72p | tr\|B2R491\|B2R491_HUMAN | tr\|V9HW22\|V9HW22_HUMAN | 645 |
| RPS4X | RPS8 | tr\|B2R491\|B2R491_HUMAN | tr\|Q5JR94\|Q5JR94_HUMAN | 999 |
| RPS4X | RPL7 | tr\|B2R491\|B2R491_HUMAN | tr\|A0A024R814\|A0A024R814_HUMAN | 999 |
| RPS4X | HEL-S-94n | tr\|B2R491\|B2R491_HUMAN | tr\|V9HW72\|V9HW72_HUMAN | 424 |
| RPS4X | RPL13 | tr\|B2R491\|B2R491_HUMAN | tr\|Q6NZ55\|Q6NZ55_HUMAN | 999 |
| RPS4X | EEF1G | tr\|B2R491\|B2R491_HUMAN | tr\|Q53YD7\|Q53YD7_HUMAN | 843 |
| RPS4X | RPL5 | tr\|B2R491\|B2R491_HUMAN | tr\|A2RUM7\|A2RUM7_HUMAN | 999 |
| RPS4X | HEL103 | tr\|B2R491\|B2R491_HUMAN | tr\|V9HWK0\|V9HWK0_HUMAN | 900 |
| RPS4X | RPL26 | tr\|B2R491\|B2R491_HUMAN | sp\|P61254\|RL26_HUMAN | 998 |
| RPS4X | RPS18 | tr\|B2R491\|B2R491_HUMAN | sp\|P62269\|RS18_HUMAN | 979 |
| RPS4X | RPL6 | tr\|B2R491\|B2R491_HUMAN | tr\|Q8N5Z7\|Q8N5Z7_HUMAN | 999 |
| RPS4X | HNRPU | tr\|B2R491\|B2R491_HUMAN | tr\|B4DLR3\|B4DLR3_HUMAN | 426 |
| RPS4X | RPS7 | tr\|B2R491\|B2R491_HUMAN | sp\|P62081\|RS7_HUMAN | 999 |
| RPS4X | RPS13 | tr\|B2R491\|B2R491_HUMAN | sp\|P62277\|RS13_HUMAN | 999 |
| RPS4X | IMPDH2 | tr\|B2R491\|B2R491_HUMAN | tr\|H0Y4R1\|H0Y4R1_HUMAN | 540 |
| RPS4X | RPL19 | tr\|B2R491\|B2R491_HUMAN | tr\|Q53G49\|Q53G49_HUMAN | 999 |
| RPS4X | RPL18 | tr\|B2R491\|B2R491_HUMAN | tr\|J3QQ67\|J3QQ67_HUMAN | 999 |
| RPS4X | RPS3 | tr\|B2R491\|B2R491_HUMAN | sp\|P23396\|RS3_HUMAN | 999 |
| RPS4X | RPS27A | tr\|B2R491\|B2R491_HUMAN | tr\|B2RDW1\|B2RDW1_HUMAN | 998 |
| RPS4X | NPM1 | tr\|B2R491\|B2R491_HUMAN | tr\|A0A0S2Z491\|A0A0S2Z491_HUMAN | 589 |
| RPS4X | RAD23B | tr\|B2R491\|B2R491_HUMAN | sp\|P54727\|RD23B_HUMAN | 433 |
| RPS4X | RPS5 | tr\|B2R491\|B2R491_HUMAN | tr\|A0A024R4Q8\|A0A024R4Q8_HUMAN | 999 |
| RPS4X | RPLP2 | tr\|B2R491\|B2R491_HUMAN | tr\|A0A024RCA7\|A0A024RCA7_HUMAN | 999 |
| RPS4X | UBE1 | tr\|B2R491\|B2R491_HUMAN | tr\|A0A024R1A3\|A0A024R1A3_HUMAN | 440 |
| RPS4X | RPL3 | tr\|B2R491\|B2R491_HUMAN | sp\|P39023\|RL3_HUMAN | 999 |
| RPS4X | SRP14 | tr\|B2R491\|B2R491_HUMAN | sp\|P37108\|SRP14_HUMAN | 976 |
| RPS4X | RPS6 | tr\|B2R491\|B2R491_HUMAN | tr\|A2A3R6\|A2A3R6_HUMAN | 999 |
| RPS4X | RPS19 | tr\|B2R491\|B2R491_HUMAN | tr\|Q8WVX7\|Q8WVX7_HUMAN | 999 |
| UGDH | TKT | sp\|O60701\|UGDH_HUMAN | tr\|Q53EM5\|Q53EM5_HUMAN | 718 |
| UGDH | UBE2V1 | sp\|O60701\|UGDH_HUMAN | sp\|Q13404\|UB2V1_HUMAN | 648 |
| UGDH | PDIA6 | sp\|O60701\|UGDH_HUMAN | sp\|Q15084\|PDIA6_HUMAN | 424 |
| NPM1 | SRSF1 | tr\|A0A0S2Z491\|A0A0S2Z491_HUMAN | sp\|Q07955\|SRSF1_HUMAN | 661 |
| NPM1 | HEL-S-26 | tr\|A0A0S2Z491\|A0A0S2Z491_HUMAN | tr\|V9HWJ2\|V9HWJ2_HUMAN | 644 |
| NPM1 | SNRPD2 | tr\|A0A0S2Z491\|A0A0S2Z491_HUMAN | sp\|P62316\|SMD2_HUMAN | 422 |
| NPM1 | HEL-S-162eP | tr\|A0A0S2Z491\|A0A0S2Z491_HUMAN | tr\|V9HVZ4\|V9HVZ4_HUMAN | 819 |
| NPM1 | LMNB1 | tr\|A0A0S2Z491\|A0A0S2Z491_HUMAN | sp\|P20700\|LMNB1_HUMAN | 854 |
| NPM1 | HEL-S-89n | tr\|A0A0S2Z491\|A0A0S2Z491_HUMAN | tr\|V9HWB4\|V9HWB4_HUMAN | 462 |
| NPM1 | RPL5 | tr\|A0A0S2Z491\|A0A0S2Z491_HUMAN | tr\|A2RUM7\|A2RUM7_HUMAN | 950 |
| NPM1 | RPL6 | tr\|A0A0S2Z491\|A0A0S2Z491_HUMAN | tr\|Q8N5Z7\|Q8N5Z7_HUMAN | 593 |
| NPM1 | HEL2 | tr\|A0A0S2Z491\|A0A0S2Z491_HUMAN | tr\|V9HW98\|V9HW98_HUMAN | 423 |
| NPM1 | HEL-S-70 | tr\|A0A0S2Z491\|A0A0S2Z491_HUMAN | tr\|V9HW80\|V9HW80_HUMAN | 448 |
| NPM1 | HNRPU | tr\|A0A0S2Z491\|A0A0S2Z491_HUMAN | tr\|B4DLR3\|B4DLR3_HUMAN | 783 |
| NPM1 | PTBP1 | tr\|A0A0S2Z491\|A0A0S2Z491_HUMAN | sp\|P26599\|PTBP1_HUMAN | 637 |
| NPM1 | EL52 | tr\|A0A0S2Z491\|A0A0S2Z491_HUMAN | tr\|K9JA46\|K9JA46_HUMAN | 602 |
| NPM1 | HEL-S-15 | tr\|A0A0S2Z491\|A0A0S2Z491_HUMAN | tr\|V9HWI5\|V9HWI5_HUMAN | 403 |
| NPM1 | RPS5 | tr\|A0A0S2Z491\|A0A0S2Z491_HUMAN | tr\|A0A024R4Q8\|A0A024R4Q8_HUMAN | 403 |
| NPM1 | SMARCA5 | tr\|A0A0S2Z491\|A0A0S2Z491_HUMAN | tr\|B7ZAX9\|B7ZAX9_HUMAN | 910 |
| NPM1 | NAP1L4b | tr\|A0A0S2Z491\|A0A0S2Z491_HUMAN | tr\|B4DS05\|B4DS05_HUMAN | 909 |
| NPM1 | HEL-S-128m | tr\|A0A0S2Z491\|A0A0S2Z491_HUMAN | tr\|V9HWC7\|V9HWC7_HUMAN | 430 |
| NPM1 | HSP90AB1 | tr\|A0A0S2Z491\|A0A0S2Z491_HUMAN | tr\|A0A024RD80\|A0A024RD80_HUMAN | 430 |
| NPM1 | RPS6 | tr\|A0A0S2Z491\|A0A0S2Z491_HUMAN | tr\|A2A3R6\|A2A3R6_HUMAN | 696 |
| NPM1 | KPNB1 | tr\|A0A0S2Z491\|A0A0S2Z491_HUMAN | sp\|Q14974\|IMB1_HUMAN | 917 |
| HEL-S-15 | ACTN4 | tr\|V9HWI5\|V9HWI5_HUMAN | tr\|A0A0S2Z3G9\|A0A0S2Z3G9_HUMAN | 523 |
| HEL-S-15 | RPS27A | tr\|V9HWI5\|V9HWI5_HUMAN | tr\|B2RDW1\|B2RDW1_HUMAN | 712 |
| HEL-S-15 | EL52 | tr\|V9HWI5\|V9HWI5_HUMAN | tr\|K9JA46\|K9JA46_HUMAN | 951 |
| HEL-S-15 | TPI1 | tr\|V9HWI5\|V9HWI5_HUMAN | sp\|P60174\|TPIS_HUMAN | 600 |
| HEL-S-15 | HEL-S-162eP | tr\|V9HWI5\|V9HWI5_HUMAN | tr\|V9HVZ4\|V9HVZ4_HUMAN | 400 |
| HEL-S-15 | TKT | tr\|V9HWI5\|V9HWI5_HUMAN | tr\|Q53EM5\|Q53EM5_HUMAN | 536 |
| HEL-S-15 | HSP90AB1 | tr\|V9HWI5\|V9HWI5_HUMAN | tr\|A0A024RD80\|A0A024RD80_HUMAN | 950 |
| HEL-S-15 | PRDX1 | tr\|V9HWI5\|V9HWI5_HUMAN | sp\|Q06830\|PRDX1_HUMAN | 457 |
| LPCAT1 | PAFAH1B1 | sp\|Q8NF37\|PCAT1_HUMAN | sp\|P43034\|LIS1_HUMAN | 904 |
| LPCAT1 | PTDSS1 | sp\|Q8NF37\|PCAT1_HUMAN | tr\|A8KAH1\|A8KAH1_HUMAN | 904 |
| HNRNPL | SRSF1 | sp\|P14866\|HNRPL_HUMAN | sp\|Q07955\|SRSF1_HUMAN | 949 |
| HNRNPL | SNRPD2 | sp\|P14866\|HNRPL_HUMAN | sp\|P62316\|SMD2_HUMAN | 900 |
| HNRNPL | HNRNPA2B1 | sp\|P14866\|HNRPL_HUMAN | sp\|P22626\|ROA2_HUMAN | 999 |
| HNRNPL | DYNC1H1 | sp\|P14866\|HNRPL_HUMAN | sp\|Q14204\|DYHC1_HUMAN | 644 |
| HNRNPL | HEL-S-89n | sp\|P14866\|HNRPL_HUMAN | tr\|V9HWB4\|V9HWB4_HUMAN | 629 |
| HNRNPL | PRIC295 | sp\|P14866\|HNRPL_HUMAN | tr\|E1NZA1\|E1NZA1_HUMAN | 653 |
| HNRNPL | SNRNP200 | sp\|P14866\|HNRPL_HUMAN | sp\|O75643\|U520_HUMAN | 909 |
| HNRNPL | HNRPU | sp\|P14866\|HNRPL_HUMAN | tr\|B4DLR3\|B4DLR3_HUMAN | 946 |
| HNRNPL | HNRPA1 | sp\|P14866\|HNRPL_HUMAN | tr\|A0A024RAZ7\|A0A024RAZ7_HUMAN | 998 |
| HNRNPL | PTBP1 | sp\|P14866\|HNRPL_HUMAN | sp\|P26599\|PTBP1_HUMAN | 998 |
| HEL-S-99n | HEL-S-162eP | tr\|V9HW88\|V9HW88_HUMAN | tr\|V9HVZ4\|V9HVZ4_HUMAN | 530 |
| HEL-S-99n | HEL-S-72p | tr\|V9HW88\|V9HW88_HUMAN | tr\|V9HW22\|V9HW22_HUMAN | 623 |
| HEL-S-99n | HEL-S-103 | tr\|V9HW88\|V9HW88_HUMAN | tr\|A8K5I0\|A8K5I0_HUMAN | 404 |
| HEL-S-99n | HEL-S-89n | tr\|V9HW88\|V9HW88_HUMAN | tr\|V9HWB4\|V9HWB4_HUMAN | 861 |
| HEL-S-99n | PPIB | tr\|V9HW88\|V9HW88_HUMAN | sp\|P23284\|PPIB_HUMAN | 498 |
| HEL-S-99n | TRAP1 | tr\|V9HW88\|V9HW88_HUMAN | tr\|Q59EK6\|Q59EK6_HUMAN | 423 |
| HEL-S-99n | HEL-S-70 | tr\|V9HW88\|V9HW88_HUMAN | tr\|V9HW80\|V9HW80_HUMAN | 469 |
| HEL-S-99n | CDH1 | tr\|V9HW88\|V9HW88_HUMAN | tr\|D3XNU5\|D3XNU5_HUMAN | 845 |
| HEL-S-99n | CANX | tr\|V9HW88\|V9HW88_HUMAN | sp\|P27824\|CALX_HUMAN | 966 |
| HEL-S-99n | EL52 | tr\|V9HW88\|V9HW88_HUMAN | tr\|K9JA46\|K9JA46_HUMAN | 984 |
| HEL-S-99n | HEL-S-130P | tr\|V9HW88\|V9HW88_HUMAN | tr\|V9HWI3\|V9HWI3_HUMAN | 404 |
| HEL-S-99n | HEL-S-269 | tr\|V9HW88\|V9HW88_HUMAN | tr\|V9HVY3\|V9HVY3_HUMAN | 999 |
| HEL-S-99n | TRA1 | tr\|V9HW88\|V9HW88_HUMAN | tr\|Q5CAQ5\|Q5CAQ5_HUMAN | 999 |
| HEL-S-99n | PDIA6 | tr\|V9HW88\|V9HW88_HUMAN | sp\|Q15084\|PDIA6_HUMAN | 888 |
| HEL-S-99n | P4HB | tr\|V9HW88\|V9HW88_HUMAN | tr\|A0A024R8S5\|A0A024R8S5_HUMAN | 993 |
| HEL-S-99n | HSP90AB1 | tr\|V9HW88\|V9HW88_HUMAN | tr\|A0A024RD80\|A0A024RD80_HUMAN | 721 |
| HEL-S-99n | LMAN2 | tr\|V9HW88\|V9HW88_HUMAN | tr\|A8K7T4\|A8K7T4_HUMAN | 492 |
| HEL-S-99n | ERP70 | tr\|V9HW88\|V9HW88_HUMAN | tr\|A0A090N8Y2\|A0A090N8Y2_HUMAN | 953 |
| HEL-S-99n | HSPA9 | tr\|V9HW88\|V9HW88_HUMAN | tr\|Q8N1C8\|Q8N1C8_HUMAN | 472 |
| PRDX5 | DLD | sp\|P30044\|PRDX5_HUMAN | tr\|A0A024R713\|A0A024R713_HUMAN | 509 |
| PRDX5 | HEL-S-269 | sp\|P30044\|PRDX5_HUMAN | tr\|V9HVY3\|V9HVY3_HUMAN | 497 |
| PRDX5 | PDIA6 | sp\|P30044\|PRDX5_HUMAN | sp\|Q15084\|PDIA6_HUMAN | 517 |
| PRDX5 | RAD23B | sp\|P30044\|PRDX5_HUMAN | sp\|P54727\|RD23B_HUMAN | 753 |
| PRDX5 | HEL-S-128m | sp\|P30044\|PRDX5_HUMAN | tr\|V9HWC7\|V9HWC7_HUMAN | 879 |
| PRDX5 | P4HB | sp\|P30044\|PRDX5_HUMAN | tr\|A0A024R8S5\|A0A024R8S5_HUMAN | 504 |
| PRDX5 | PRDX1 | sp\|P30044\|PRDX5_HUMAN | sp\|Q06830\|PRDX1_HUMAN | 968 |
| PRDX5 | ATP5O | sp\|P30044\|PRDX5_HUMAN | sp\|P48047\|ATPO_HUMAN | 455 |
| PRDX5 | HEL-S-2a | sp\|P30044\|PRDX5_HUMAN | tr\|V9HW12\|V9HW12_HUMAN | 972 |
| PRDX5 | ERP70 | sp\|P30044\|PRDX5_HUMAN | tr\|A0A090N8Y2\|A0A090N8Y2_HUMAN | 467 |
| HSP90AB1 | MYL6 | tr\|A0A024RD80\|A0A024RD80_HUMAN | tr\|G8JLA2\|G8JLA2_HUMAN | 698 |
| HSP90AB1 | hCG_24487 | tr\|A0A024RD80\|A0A024RD80_HUMAN | tr\|A0A024R261\|A0A024R261_HUMAN | 627 |
| HSP90AB1 | RPS3A | tr\|A0A024RD80\|A0A024RD80_HUMAN | tr\|Q6NXR8\|Q6NXR8_HUMAN | 589 |
| HSP90AB1 | IARS | tr\|A0A024RD80\|A0A024RD80_HUMAN | sp\|P41252\|SYIC_HUMAN | 641 |
| HSP90AB1 | VDAC2 | tr\|A0A024RD80\|A0A024RD80_HUMAN | tr\|A0A024QZN9\|A0A024QZN9_HUMAN | 486 |
| HSP90AB1 | HEL-S-103 | tr\|A0A024RD80\|A0A024RD80_HUMAN | tr\|A8K5I0\|A8K5I0_HUMAN | 980 |
| HSP90AB1 | HEL-S-89n | tr\|A0A024RD80\|A0A024RD80_HUMAN | tr\|V9HWB4\|V9HWB4_HUMAN | 988 |
| HSP90AB1 | TRAP1 | tr\|A0A024RD80\|A0A024RD80_HUMAN | tr\|Q59EK6\|Q59EK6_HUMAN | 729 |
| HSP90AB1 | PRDX1 | tr\|A0A024RD80\|A0A024RD80_HUMAN | sp\|Q06830\|PRDX1_HUMAN | 446 |
| HSP90AB1 | RPL6 | tr\|A0A024RD80\|A0A024RD80_HUMAN | tr\|Q8N5Z7\|Q8N5Z7_HUMAN | 656 |
| HSP90AB1 | HNRPA1 | tr\|A0A024RD80\|A0A024RD80_HUMAN | tr\|A0A024RAZ7\|A0A024RAZ7_HUMAN | 470 |
| HSP90AB1 | XRCC6 | tr\|A0A024RD80\|A0A024RD80_HUMAN | tr\|A0A024R1N4\|A0A024R1N4_HUMAN | 479 |
| HSP90AB1 | CANX | tr\|A0A024RD80\|A0A024RD80_HUMAN | sp\|P27824\|CALX_HUMAN | 893 |
| HSP90AB1 | STUB1 | tr\|A0A024RD80\|A0A024RD80_HUMAN | sp\|Q9UNE7\|CHIP_HUMAN | 896 |
| HSP90AB1 | P4HB | tr\|A0A024RD80\|A0A024RD80_HUMAN | tr\|A0A024R8S5\|A0A024R8S5_HUMAN | 647 |
| HSP90AB1 | RPS6 | tr\|A0A024RD80\|A0A024RD80_HUMAN | tr\|A2A3R6\|A2A3R6_HUMAN | 735 |
| HSP90AB1 | HEL-S-100n | tr\|A0A024RD80\|A0A024RD80_HUMAN | tr\|V9HW96\|V9HW96_HUMAN | 919 |
| HSP90AB1 | STOML2 | tr\|A0A024RD80\|A0A024RD80_HUMAN | sp\|Q9UJZ1\|STML2_HUMAN | 904 |
| HSP90AB1 | HEL-S-2a | tr\|A0A024RD80\|A0A024RD80_HUMAN | tr\|V9HW12\|V9HW12_HUMAN | 496 |
| HSP90AB1 | ERP70 | tr\|A0A024RD80\|A0A024RD80_HUMAN | tr\|A0A090N8Y2\|A0A090N8Y2_HUMAN | 721 |
| HSP90AB1 | GSPT1 | tr\|A0A024RD80\|A0A024RD80_HUMAN | tr\|B2RCT6\|B2RCT6_HUMAN | 616 |
| HSP90AB1 | HSPA9 | tr\|A0A024RD80\|A0A024RD80_HUMAN | tr\|Q8N1C8\|Q8N1C8_HUMAN | 928 |
| HSP90AB1 | HEL-S-133P | tr\|A0A024RD80\|A0A024RD80_HUMAN | tr\|V9HWB9\|V9HWB9_HUMAN | 403 |
| HSP90AB1 | HEL-S-72p | tr\|A0A024RD80\|A0A024RD80_HUMAN | tr\|V9HW22\|V9HW22_HUMAN | 997 |
| HSP90AB1 | HEL-S-162eP | tr\|A0A024RD80\|A0A024RD80_HUMAN | tr\|V9HVZ4\|V9HVZ4_HUMAN | 806 |
| HSP90AB1 | HEL-S-123m | tr\|A0A024RD80\|A0A024RD80_HUMAN | tr\|V9HW26\|V9HW26_HUMAN | 982 |
| HSP90AB1 | PRIC295 | tr\|A0A024RD80\|A0A024RD80_HUMAN | tr\|E1NZA1\|E1NZA1_HUMAN | 409 |
| HSP90AB1 | HEL-S-94n | tr\|A0A024RD80\|A0A024RD80_HUMAN | tr\|V9HW72\|V9HW72_HUMAN | 999 |
| HSP90AB1 | HEL103 | tr\|A0A024RD80\|A0A024RD80_HUMAN | tr\|V9HWK0\|V9HWK0_HUMAN | 733 |
| HSP90AB1 | PPIB | tr\|A0A024RD80\|A0A024RD80_HUMAN | sp\|P23284\|PPIB_HUMAN | 477 |
| HSP90AB1 | EEF1G | tr\|A0A024RD80\|A0A024RD80_HUMAN | tr\|Q53YD7\|Q53YD7_HUMAN | 870 |
| HSP90AB1 | HEL2 | tr\|A0A024RD80\|A0A024RD80_HUMAN | tr\|V9HW98\|V9HW98_HUMAN | 626 |
| HSP90AB1 | HEL-S-70 | tr\|A0A024RD80\|A0A024RD80_HUMAN | tr\|V9HW80\|V9HW80_HUMAN | 644 |
| HSP90AB1 | PSMD4 | tr\|A0A024RD80\|A0A024RD80_HUMAN | sp\|P55036\|PSMD4_HUMAN | 840 |
| HSP90AB1 | RPS27A | tr\|A0A024RD80\|A0A024RD80_HUMAN | tr\|B2RDW1\|B2RDW1_HUMAN | 413 |
| HSP90AB1 | EL52 | tr\|A0A024RD80\|A0A024RD80_HUMAN | tr\|K9JA46\|K9JA46_HUMAN | 999 |
| HSP90AB1 | RALY | tr\|A0A024RD80\|A0A024RD80_HUMAN | tr\|A8K4T9\|A8K4T9_HUMAN | 685 |
| HSP90AB1 | HEL-S-269 | tr\|A0A024RD80\|A0A024RD80_HUMAN | tr\|V9HVY3\|V9HVY3_HUMAN | 710 |
| HSP90AB1 | TRA1 | tr\|A0A024RD80\|A0A024RD80_HUMAN | tr\|Q5CAQ5\|Q5CAQ5_HUMAN | 596 |
| HSP90AB1 | PDIA6 | tr\|A0A024RD80\|A0A024RD80_HUMAN | sp\|Q15084\|PDIA6_HUMAN | 676 |
| HSP90AB1 | RAD23B | tr\|A0A024RD80\|A0A024RD80_HUMAN | sp\|P54727\|RD23B_HUMAN | 410 |
| HSP90AB1 | HEL-S-68p | tr\|A0A024RD80\|A0A024RD80_HUMAN | tr\|V9HWF4\|V9HWF4_HUMAN | 652 |
| HSP90AB1 | HEL-S-271 | tr\|A0A024RD80\|A0A024RD80_HUMAN | tr\|V9HW31\|V9HW31_HUMAN | 971 |
| HSP90AB1 | CCT4 | tr\|A0A024RD80\|A0A024RD80_HUMAN | sp\|P50991\|TCPD_HUMAN | 787 |
| HSP90AB1 | RPL3 | tr\|A0A024RD80\|A0A024RD80_HUMAN | sp\|P39023\|RL3_HUMAN | 890 |
| HSP90AB1 | MYH9 | tr\|A0A024RD80\|A0A024RD80_HUMAN | tr\|A0A024R1N1\|A0A024R1N1_HUMAN | 916 |
| HSP90AB1 | ATP5O | tr\|A0A024RD80\|A0A024RD80_HUMAN | sp\|P48047\|ATPO_HUMAN | 635 |
| CHCHD3 | ATP5O | tr\|C9JRZ6\|C9JRZ6_HUMAN | sp\|P48047\|ATPO_HUMAN | 415 |
| CHCHD3 | HEL-S-103 | tr\|C9JRZ6\|C9JRZ6_HUMAN | tr\|A8K5I0\|A8K5I0_HUMAN | 452 |
| SRI | TRAP1 | tr\|C9J0K6\|C9J0K6_HUMAN | tr\|Q59EK6\|Q59EK6_HUMAN | 816 |
| TOLLIP | DIABLO | tr\|Q6FIE9\|Q6FIE9_HUMAN | tr\|A0A0S2Z5U7\|A0A0S2Z5U7_HUMAN | 459 |
| HEL-S-274 | ANXA1 | tr\|V9HW59\|V9HW59_HUMAN | tr\|Q5TZZ9\|Q5TZZ9_HUMAN | 659 |
| LIMA1 | CDH1 | tr\|Q53GG0\|Q53GG0_HUMAN | tr\|D3XNU5\|D3XNU5_HUMAN | 970 |
| IARS | RPL18 | sp\|P41252\|SYIC_HUMAN | tr\|J3QQ67\|J3QQ67_HUMAN | 584 |
| IARS | EL52 | sp\|P41252\|SYIC_HUMAN | tr\|K9JA46\|K9JA46_HUMAN | 654 |
| IARS | TARS | sp\|P41252\|SYIC_HUMAN | tr\|Q53GX7\|Q53GX7_HUMAN | 938 |
| IARS | CCT4 | sp\|P41252\|SYIC_HUMAN | sp\|P50991\|TCPD_HUMAN | 459 |
| IARS | UBE1 | sp\|P41252\|SYIC_HUMAN | tr\|A0A024R1A3\|A0A024R1A3_HUMAN | 945 |
| IARS | FARSB | sp\|P41252\|SYIC_HUMAN | tr\|Q9BR63\|Q9BR63_HUMAN | 988 |
| IARS | AARS | sp\|P41252\|SYIC_HUMAN | sp\|P49588\|SYAC_HUMAN | 995 |
| IARS | RPL3 | sp\|P41252\|SYIC_HUMAN | sp\|P39023\|RL3_HUMAN | 569 |
| IARS | TRAP1 | sp\|P41252\|SYIC_HUMAN | tr\|Q59EK6\|Q59EK6_HUMAN | 504 |
| IARS | HEL-S-100n | sp\|P41252\|SYIC_HUMAN | tr\|V9HW96\|V9HW96_HUMAN | 431 |
| IARS | HSPA9 | sp\|P41252\|SYIC_HUMAN | tr\|Q8N1C8\|Q8N1C8_HUMAN | 431 |
| IARS | IMPDH2 | sp\|P41252\|SYIC_HUMAN | tr\|H0Y4R1\|H0Y4R1_HUMAN | 599 |
| GNB2 | ACTN4 | tr\|Q6FHM2\|Q6FHM2_HUMAN | tr\|A0A0S2Z3G9\|A0A0S2Z3G9_HUMAN | 593 |
| GNB2 | HEL-S-94n | tr\|Q6FHM2\|Q6FHM2_HUMAN | tr\|V9HW72\|V9HW72_HUMAN | 468 |
| GNB2 | HIST1H4L | tr\|Q6FHM2\|Q6FHM2_HUMAN | tr\|B2R4R0\|B2R4R0_HUMAN | 575 |
| COPB1 | COPB2 | sp\|P53618\|COPB_HUMAN | sp\|P35606\|COPB2_HUMAN | 999 |
| COPB1 | ARCN1 | sp\|P53618\|COPB_HUMAN | tr\|B0YIW6\|B0YIW6_HUMAN | 999 |
| RPL5 | hCG_24487 | tr\|A2RUM7\|A2RUM7_HUMAN | tr\|A0A024R261\|A0A024R261_HUMAN | 999 |
| RPL5 | RPS3A | tr\|A2RUM7\|A2RUM7_HUMAN | tr\|Q6NXR8\|Q6NXR8_HUMAN | 999 |
| RPL5 | SSB | tr\|A2RUM7\|A2RUM7_HUMAN | tr\|B5BUB5\|B5BUB5_HUMAN | 460 |
| RPL5 | RPL7 | tr\|A2RUM7\|A2RUM7_HUMAN | tr\|A0A024R814\|A0A024R814_HUMAN | 999 |
| RPL5 | RPL13 | tr\|A2RUM7\|A2RUM7_HUMAN | tr\|Q6NZ55\|Q6NZ55_HUMAN | 999 |
| RPL5 | RPS18 | tr\|A2RUM7\|A2RUM7_HUMAN | sp\|P62269\|RS18_HUMAN | 994 |
| RPL5 | RPL6 | tr\|A2RUM7\|A2RUM7_HUMAN | tr\|Q8N5Z7\|Q8N5Z7_HUMAN | 999 |
| RPL5 | HNRPA1 | tr\|A2RUM7\|A2RUM7_HUMAN | tr\|A0A024RAZ7\|A0A024RAZ7_HUMAN | 413 |
| RPL5 | IMPDH2 | tr\|A2RUM7\|A2RUM7_HUMAN | tr\|H0Y4R1\|H0Y4R1_HUMAN | 736 |
| RPL5 | TARS | tr\|A2RUM7\|A2RUM7_HUMAN | tr\|Q53GX7\|Q53GX7_HUMAN | 559 |
| RPL5 | RPS5 | tr\|A2RUM7\|A2RUM7_HUMAN | tr\|A0A024R4Q8\|A0A024R4Q8_HUMAN | 999 |
| RPL5 | SRP14 | tr\|A2RUM7\|A2RUM7_HUMAN | sp\|P37108\|SRP14_HUMAN | 900 |
| RPL5 | RPS6 | tr\|A2RUM7\|A2RUM7_HUMAN | tr\|A2A3R6\|A2A3R6_HUMAN | 999 |
| RPL5 | HEL-S-100n | tr\|A2RUM7\|A2RUM7_HUMAN | tr\|V9HW96\|V9HW96_HUMAN | 517 |
| RPL5 | RPS9 | tr\|A2RUM7\|A2RUM7_HUMAN | tr\|A0A024R4M0\|A0A024R4M0_HUMAN | 997 |
| RPL5 | HEL-S-162eP | tr\|A2RUM7\|A2RUM7_HUMAN | tr\|V9HVZ4\|V9HVZ4_HUMAN | 401 |
| RPL5 | HEL-S-123m | tr\|A2RUM7\|A2RUM7_HUMAN | tr\|V9HW26\|V9HW26_HUMAN | 774 |
| RPL5 | RPS8 | tr\|A2RUM7\|A2RUM7_HUMAN | tr\|Q5JR94\|Q5JR94_HUMAN | 999 |
| RPL5 | PRIC295 | tr\|A2RUM7\|A2RUM7_HUMAN | tr\|E1NZA1\|E1NZA1_HUMAN | 445 |
| RPL5 | HEL103 | tr\|A2RUM7\|A2RUM7_HUMAN | tr\|V9HWK0\|V9HWK0_HUMAN | 902 |
| RPL5 | EEF1G | tr\|A2RUM7\|A2RUM7_HUMAN | tr\|Q53YD7\|Q53YD7_HUMAN | 940 |
| RPL5 | RPL26 | tr\|A2RUM7\|A2RUM7_HUMAN | sp\|P61254\|RL26_HUMAN | 999 |
| RPL5 | RPS13 | tr\|A2RUM7\|A2RUM7_HUMAN | sp\|P62277\|RS13_HUMAN | 999 |
| RPL5 | RPS7 | tr\|A2RUM7\|A2RUM7_HUMAN | sp\|P62081\|RS7_HUMAN | 999 |
| RPL5 | RPL19 | tr\|A2RUM7\|A2RUM7_HUMAN | tr\|Q53G49\|Q53G49_HUMAN | 999 |
| RPL5 | RPL18 | tr\|A2RUM7\|A2RUM7_HUMAN | tr\|J3QQ67\|J3QQ67_HUMAN | 999 |
| RPL5 | RPS3 | tr\|A2RUM7\|A2RUM7_HUMAN | sp\|P23396\|RS3_HUMAN | 999 |
| RPL5 | EL52 | tr\|A2RUM7\|A2RUM7_HUMAN | tr\|K9JA46\|K9JA46_HUMAN | 723 |
| RPL5 | RPS27A | tr\|A2RUM7\|A2RUM7_HUMAN | tr\|B2RDW1\|B2RDW1_HUMAN | 999 |
| RPL5 | RAD23B | tr\|A2RUM7\|A2RUM7_HUMAN | sp\|P54727\|RD23B_HUMAN | 474 |
| RPL5 | HEL-S-271 | tr\|A2RUM7\|A2RUM7_HUMAN | tr\|V9HW31\|V9HW31_HUMAN | 774 |
| RPL5 | CCT4 | tr\|A2RUM7\|A2RUM7_HUMAN | sp\|P50991\|TCPD_HUMAN | 514 |
| RPL5 | RPLP2 | tr\|A2RUM7\|A2RUM7_HUMAN | tr\|A0A024RCA7\|A0A024RCA7_HUMAN | 999 |
| RPL5 | RPL3 | tr\|A2RUM7\|A2RUM7_HUMAN | sp\|P39023\|RL3_HUMAN | 999 |
| RPL5 | KPNB1 | tr\|A2RUM7\|A2RUM7_HUMAN | sp\|Q14974\|IMB1_HUMAN | 598 |
| RPL5 | ATP5O | tr\|A2RUM7\|A2RUM7_HUMAN | sp\|P48047\|ATPO_HUMAN | 683 |
| RPL5 | RPS19 | tr\|A2RUM7\|A2RUM7_HUMAN | tr\|Q8WVX7\|Q8WVX7_HUMAN | 999 |
| TRAP1 | HEL-S-162eP | tr\|Q59EK6\|Q59EK6_HUMAN | tr\|V9HVZ4\|V9HVZ4_HUMAN | 787 |
| TRAP1 | HEL-S-72p | tr\|Q59EK6\|Q59EK6_HUMAN | tr\|V9HW22\|V9HW22_HUMAN | 870 |
| TRAP1 | HEL-S-123m | tr\|Q59EK6\|Q59EK6_HUMAN | tr\|V9HW26\|V9HW26_HUMAN | 448 |
| TRAP1 | VDAC2 | tr\|Q59EK6\|Q59EK6_HUMAN | tr\|A0A024QZN9\|A0A024QZN9_HUMAN | 709 |
| TRAP1 | HEL-S-103 | tr\|Q59EK6\|Q59EK6_HUMAN | tr\|A8K5I0\|A8K5I0_HUMAN | 764 |
| TRAP1 | HEL-S-89n | tr\|Q59EK6\|Q59EK6_HUMAN | tr\|V9HWB4\|V9HWB4_HUMAN | 929 |
| TRAP1 | HEL-S-94n | tr\|Q59EK6\|Q59EK6_HUMAN | tr\|V9HW72\|V9HW72_HUMAN | 966 |
| TRAP1 | EEF1G | tr\|Q59EK6\|Q59EK6_HUMAN | tr\|Q53YD7\|Q53YD7_HUMAN | 794 |
| TRAP1 | RPS27A | tr\|Q59EK6\|Q59EK6_HUMAN | tr\|B2RDW1\|B2RDW1_HUMAN | 410 |
| TRAP1 | EL52 | tr\|Q59EK6\|Q59EK6_HUMAN | tr\|K9JA46\|K9JA46_HUMAN | 723 |
| TRAP1 | HEL-S-269 | tr\|Q59EK6\|Q59EK6_HUMAN | tr\|V9HVY3\|V9HVY3_HUMAN | 431 |
| TRAP1 | TRA1 | tr\|Q59EK6\|Q59EK6_HUMAN | tr\|Q5CAQ5\|Q5CAQ5_HUMAN | 664 |
| TRAP1 | PDIA6 | tr\|Q59EK6\|Q59EK6_HUMAN | sp\|Q15084\|PDIA6_HUMAN | 426 |
| TRAP1 | HEL-S-68p | tr\|Q59EK6\|Q59EK6_HUMAN | tr\|V9HWF4\|V9HWF4_HUMAN | 454 |
| TRAP1 | HEL-S-271 | tr\|Q59EK6\|Q59EK6_HUMAN | tr\|V9HW31\|V9HW31_HUMAN | 552 |
| TRAP1 | CCT4 | tr\|Q59EK6\|Q59EK6_HUMAN | sp\|P50991\|TCPD_HUMAN | 778 |
| TRAP1 | HEL-S-100n | tr\|Q59EK6\|Q59EK6_HUMAN | tr\|V9HW96\|V9HW96_HUMAN | 792 |
| TRAP1 | RPS19 | tr\|Q59EK6\|Q59EK6_HUMAN | tr\|Q8WVX7\|Q8WVX7_HUMAN | 596 |
| TRAP1 | HSPA9 | tr\|Q59EK6\|Q59EK6_HUMAN | tr\|Q8N1C8\|Q8N1C8_HUMAN | 827 |
| PRDX1 | HEL-S-162eP | sp\|Q06830\|PRDX1_HUMAN | tr\|V9HVZ4\|V9HVZ4_HUMAN | 746 |
| PRDX1 | HEL-S-72p | sp\|Q06830\|PRDX1_HUMAN | tr\|V9HW22\|V9HW22_HUMAN | 586 |
| PRDX1 | VDAC2 | sp\|Q06830\|PRDX1_HUMAN | tr\|A0A024QZN9\|A0A024QZN9_HUMAN | 413 |
| PRDX1 | HEL-S-103 | sp\|Q06830\|PRDX1_HUMAN | tr\|A8K5I0\|A8K5I0_HUMAN | 401 |
| PRDX1 | HEL-S-89n | sp\|Q06830\|PRDX1_HUMAN | tr\|V9HWB4\|V9HWB4_HUMAN | 579 |
| PRDX1 | EEF1G | sp\|Q06830\|PRDX1_HUMAN | tr\|Q53YD7\|Q53YD7_HUMAN | 546 |
| PRDX1 | IMPDH2 | sp\|Q06830\|PRDX1_HUMAN | tr\|H0Y4R1\|H0Y4R1_HUMAN | 570 |
| PRDX1 | ANXA1 | sp\|Q06830\|PRDX1_HUMAN | tr\|Q5TZZ9\|Q5TZZ9_HUMAN | 450 |
| PRDX1 | EL52 | sp\|Q06830\|PRDX1_HUMAN | tr\|K9JA46\|K9JA46_HUMAN | 506 |
| PRDX1 | DLD | sp\|Q06830\|PRDX1_HUMAN | tr\|A0A024R713\|A0A024R713_HUMAN | 429 |
| PRDX1 | TPI1 | sp\|Q06830\|PRDX1_HUMAN | sp\|P60174\|TPIS_HUMAN | 415 |
| PRDX1 | HEL-S-269 | sp\|Q06830\|PRDX1_HUMAN | tr\|V9HVY3\|V9HVY3_HUMAN | 732 |
| PRDX1 | TRA1 | sp\|Q06830\|PRDX1_HUMAN | tr\|Q5CAQ5\|Q5CAQ5_HUMAN | 447 |
| PRDX1 | PDIA6 | sp\|Q06830\|PRDX1_HUMAN | sp\|Q15084\|PDIA6_HUMAN | 699 |
| PRDX1 | TKT | sp\|Q06830\|PRDX1_HUMAN | tr\|Q53EM5\|Q53EM5_HUMAN | 780 |
| PRDX1 | HEL-S-128m | sp\|Q06830\|PRDX1_HUMAN | tr\|V9HWC7\|V9HWC7_HUMAN | 791 |
| PRDX1 | P4HB | sp\|Q06830\|PRDX1_HUMAN | tr\|A0A024R8S5\|A0A024R8S5_HUMAN | 653 |
| PRDX1 | HEL-S-100n | sp\|Q06830\|PRDX1_HUMAN | tr\|V9HW96\|V9HW96_HUMAN | 448 |
| PRDX1 | HEL-S-2a | sp\|Q06830\|PRDX1_HUMAN | tr\|V9HW12\|V9HW12_HUMAN | 975 |
| PRDX1 | ERP70 | sp\|Q06830\|PRDX1_HUMAN | tr\|A0A090N8Y2\|A0A090N8Y2_HUMAN | 671 |
| PRDX1 | HSPA9 | sp\|Q06830\|PRDX1_HUMAN | tr\|Q8N1C8\|Q8N1C8_HUMAN | 567 |
| HEL-S-45 | TMSB4X | tr\|V9HWG3\|V9HWG3_HUMAN | tr\|Q0P5N8\|Q0P5N8_HUMAN | 579 |
| HEL-S-45 | KRT19 | tr\|V9HWG3\|V9HWG3_HUMAN | sp\|P08727\|K1C19_HUMAN | 640 |
| HEL-S-45 | HEL-S-22 | tr\|V9HWG3\|V9HWG3_HUMAN | tr\|V9HWE9\|V9HWE9_HUMAN | 614 |
| HEL-S-45 | ANXA1 | tr\|V9HWG3\|V9HWG3_HUMAN | tr\|Q5TZZ9\|Q5TZZ9_HUMAN | 637 |
| HEL-S-45 | HEL-S-162eP | tr\|V9HWG3\|V9HWG3_HUMAN | tr\|V9HVZ4\|V9HVZ4_HUMAN | 505 |
| HEL-S-45 | C7orf24 | tr\|V9HWG3\|V9HWG3_HUMAN | tr\|A0A090N7V5\|A0A090N7V5_HUMAN | 844 |
| HEL-S-45 | UBE2V1 | tr\|V9HWG3\|V9HWG3_HUMAN | sp\|Q13404\|UB2V1_HUMAN | 538 |
| HEL-S-45 | CDH1 | tr\|V9HWG3\|V9HWG3_HUMAN | tr\|D3XNU5\|D3XNU5_HUMAN | 858 |
| HEL-S-45 | PSMD4 | tr\|V9HWG3\|V9HWG3_HUMAN | sp\|P55036\|PSMD4_HUMAN | 587 |
| RPL6 | hCG_24487 | tr\|Q8N5Z7\|Q8N5Z7_HUMAN | tr\|A0A024R261\|A0A024R261_HUMAN | 999 |
| RPL6 | RPS9 | tr\|Q8N5Z7\|Q8N5Z7_HUMAN | tr\|A0A024R4M0\|A0A024R4M0_HUMAN | 984 |
| RPL6 | RPS3A | tr\|Q8N5Z7\|Q8N5Z7_HUMAN | tr\|Q6NXR8\|Q6NXR8_HUMAN | 999 |
| RPL6 | RPS8 | tr\|Q8N5Z7\|Q8N5Z7_HUMAN | tr\|Q5JR94\|Q5JR94_HUMAN | 999 |
| RPL6 | RPL7 | tr\|Q8N5Z7\|Q8N5Z7_HUMAN | tr\|A0A024R814\|A0A024R814_HUMAN | 999 |
| RPL6 | RPL13 | tr\|Q8N5Z7\|Q8N5Z7_HUMAN | tr\|Q6NZ55\|Q6NZ55_HUMAN | 999 |
| RPL6 | EEF1G | tr\|Q8N5Z7\|Q8N5Z7_HUMAN | tr\|Q53YD7\|Q53YD7_HUMAN | 905 |
| RPL6 | HEL103 | tr\|Q8N5Z7\|Q8N5Z7_HUMAN | tr\|V9HWK0\|V9HWK0_HUMAN | 900 |
| RPL6 | RPL26 | tr\|Q8N5Z7\|Q8N5Z7_HUMAN | sp\|P61254\|RL26_HUMAN | 999 |
| RPL6 | RPS18 | tr\|Q8N5Z7\|Q8N5Z7_HUMAN | sp\|P62269\|RS18_HUMAN | 955 |
| RPL6 | RPS7 | tr\|Q8N5Z7\|Q8N5Z7_HUMAN | sp\|P62081\|RS7_HUMAN | 999 |
| RPL6 | RPS13 | tr\|Q8N5Z7\|Q8N5Z7_HUMAN | sp\|P62277\|RS13_HUMAN | 999 |
| RPL6 | RPL19 | tr\|Q8N5Z7\|Q8N5Z7_HUMAN | tr\|Q53G49\|Q53G49_HUMAN | 999 |
| RPL6 | RPL18 | tr\|Q8N5Z7\|Q8N5Z7_HUMAN | tr\|J3QQ67\|J3QQ67_HUMAN | 999 |
| RPL6 | EPS8 | tr\|Q8N5Z7\|Q8N5Z7_HUMAN | sp\|Q12929\|EPS8_HUMAN | 516 |
| RPL6 | RPS3 | tr\|Q8N5Z7\|Q8N5Z7_HUMAN | sp\|P23396\|RS3_HUMAN | 999 |
| RPL6 | RPS27A | tr\|Q8N5Z7\|Q8N5Z7_HUMAN | tr\|B2RDW1\|B2RDW1_HUMAN | 997 |
| RPL6 | RPS5 | tr\|Q8N5Z7\|Q8N5Z7_HUMAN | tr\|A0A024R4Q8\|A0A024R4Q8_HUMAN | 999 |
| RPL6 | RPLP2 | tr\|Q8N5Z7\|Q8N5Z7_HUMAN | tr\|A0A024RCA7\|A0A024RCA7_HUMAN | 999 |
| RPL6 | RPL3 | tr\|Q8N5Z7\|Q8N5Z7_HUMAN | sp\|P39023\|RL3_HUMAN | 999 |
| RPL6 | SRP14 | tr\|Q8N5Z7\|Q8N5Z7_HUMAN | sp\|P37108\|SRP14_HUMAN | 959 |
| RPL6 | RPS6 | tr\|Q8N5Z7\|Q8N5Z7_HUMAN | tr\|A2A3R6\|A2A3R6_HUMAN | 999 |
| RPL6 | HEL-S-100n | tr\|Q8N5Z7\|Q8N5Z7_HUMAN | tr\|V9HW96\|V9HW96_HUMAN | 567 |
| RPL6 | RPS19 | tr\|Q8N5Z7\|Q8N5Z7_HUMAN | tr\|Q8WVX7\|Q8WVX7_HUMAN | 998 |
| XRCC6 | EL52 | tr\|A0A024R1N4\|A0A024R1N4_HUMAN | tr\|K9JA46\|K9JA46_HUMAN | 647 |
| XRCC6 | HEL-S-162eP | tr\|A0A024R1N4\|A0A024R1N4_HUMAN | tr\|V9HVZ4\|V9HVZ4_HUMAN | 457 |
| XRCC6 | HNRPA1 | tr\|A0A024R1N4\|A0A024R1N4_HUMAN | tr\|A0A024RAZ7\|A0A024RAZ7_HUMAN | 444 |
| XRCC6 | RAD23B | tr\|A0A024R1N4\|A0A024R1N4_HUMAN | sp\|P54727\|RD23B_HUMAN | 925 |
| XRCC6 | HEL-S-89n | tr\|A0A024R1N4\|A0A024R1N4_HUMAN | tr\|V9HWB4\|V9HWB4_HUMAN | 701 |
| XRCC6 | FARSB | tr\|A0A024R1N4\|A0A024R1N4_HUMAN | tr\|Q9BR63\|Q9BR63_HUMAN | 532 |
| CDH1 | KRT19 | tr\|D3XNU5\|D3XNU5_HUMAN | sp\|P08727\|K1C19_HUMAN | 635 |
| CDH1 | HEL-S-22 | tr\|D3XNU5\|D3XNU5_HUMAN | tr\|V9HWE9\|V9HWE9_HUMAN | 581 |
| CDH1 | ITGB4 | tr\|D3XNU5\|D3XNU5_HUMAN | tr\|B7ZLD5\|B7ZLD5_HUMAN | 927 |
| CDH1 | EZR | tr\|D3XNU5\|D3XNU5_HUMAN | tr\|E7EQR4\|E7EQR4_HUMAN | 961 |
| CDH1 | EL52 | tr\|D3XNU5\|D3XNU5_HUMAN | tr\|K9JA46\|K9JA46_HUMAN | 452 |
| CDH1 | MKI67 | tr\|D3XNU5\|D3XNU5_HUMAN | tr\|A0A087WV66\|A0A087WV66_HUMAN | 831 |
| CDH1 | HEL-S-162eP | tr\|D3XNU5\|D3XNU5_HUMAN | tr\|V9HVZ4\|V9HVZ4_HUMAN | 749 |
| CDH1 | DPP4 | tr\|D3XNU5\|D3XNU5_HUMAN | sp\|P27487\|DPP4_HUMAN | 414 |
| CDH1 | HEL-S-130P | tr\|D3XNU5\|D3XNU5_HUMAN | tr\|V9HWI3\|V9HWI3_HUMAN | 414 |
| CDH1 | HEL-S-53e | tr\|D3XNU5\|D3XNU5_HUMAN | tr\|V9HW83\|V9HW83_HUMAN | 471 |
| CDH1 | HEL70 | tr\|D3XNU5\|D3XNU5_HUMAN | tr\|V9HWC0\|V9HWC0_HUMAN | 507 |
| IMPDH2 | RPS9 | tr\|H0Y4R1\|H0Y4R1_HUMAN | tr\|A0A024R4M0\|A0A024R4M0_HUMAN | 686 |
| IMPDH2 | RPS3A | tr\|H0Y4R1\|H0Y4R1_HUMAN | tr\|Q6NXR8\|Q6NXR8_HUMAN | 578 |
| IMPDH2 | HADHA | tr\|H0Y4R1\|H0Y4R1_HUMAN | tr\|E9KL44\|E9KL44_HUMAN | 585 |
| IMPDH2 | HEL-S-162eP | tr\|H0Y4R1\|H0Y4R1_HUMAN | tr\|V9HVZ4\|V9HVZ4_HUMAN | 690 |
| IMPDH2 | HEL-S-72p | tr\|H0Y4R1\|H0Y4R1_HUMAN | tr\|V9HW22\|V9HW22_HUMAN | 641 |
| IMPDH2 | HEL-S-123m | tr\|H0Y4R1\|H0Y4R1_HUMAN | tr\|V9HW26\|V9HW26_HUMAN | 574 |
| IMPDH2 | GLUD1 | tr\|H0Y4R1\|H0Y4R1_HUMAN | tr\|E9KL48\|E9KL48_HUMAN | 495 |
| IMPDH2 | RPS8 | tr\|H0Y4R1\|H0Y4R1_HUMAN | tr\|Q5JR94\|Q5JR94_HUMAN | 419 |
| IMPDH2 | HEL-S-103 | tr\|H0Y4R1\|H0Y4R1_HUMAN | tr\|A8K5I0\|A8K5I0_HUMAN | 610 |
| IMPDH2 | HEL-S-89n | tr\|H0Y4R1\|H0Y4R1_HUMAN | tr\|V9HWB4\|V9HWB4_HUMAN | 649 |
| IMPDH2 | RPL7 | tr\|H0Y4R1\|H0Y4R1_HUMAN | tr\|A0A024R814\|A0A024R814_HUMAN | 490 |
| IMPDH2 | RPL13 | tr\|H0Y4R1\|H0Y4R1_HUMAN | tr\|Q6NZ55\|Q6NZ55_HUMAN | 626 |
| IMPDH2 | RPS13 | tr\|H0Y4R1\|H0Y4R1_HUMAN | sp\|P62277\|RS13_HUMAN | 640 |
| IMPDH2 | CDIPT | tr\|H0Y4R1\|H0Y4R1_HUMAN | tr\|Q53HA5\|Q53HA5_HUMAN | 430 |
| IMPDH2 | RPS3 | tr\|H0Y4R1\|H0Y4R1_HUMAN | sp\|P23396\|RS3_HUMAN | 611 |
| IMPDH2 | DLD | tr\|H0Y4R1\|H0Y4R1_HUMAN | tr\|A0A024R713\|A0A024R713_HUMAN | 580 |
| IMPDH2 | TPI1 | tr\|H0Y4R1\|H0Y4R1_HUMAN | sp\|P60174\|TPIS_HUMAN | 643 |
| IMPDH2 | TARS | tr\|H0Y4R1\|H0Y4R1_HUMAN | tr\|Q53GX7\|Q53GX7_HUMAN | 494 |
| IMPDH2 | RAD23B | tr\|H0Y4R1\|H0Y4R1_HUMAN | sp\|P54727\|RD23B_HUMAN | 404 |
| IMPDH2 | HEL-S-271 | tr\|H0Y4R1\|H0Y4R1_HUMAN | tr\|V9HW31\|V9HW31_HUMAN | 619 |
| IMPDH2 | CCT4 | tr\|H0Y4R1\|H0Y4R1_HUMAN | sp\|P50991\|TCPD_HUMAN | 714 |
| IMPDH2 | RPL3 | tr\|H0Y4R1\|H0Y4R1_HUMAN | sp\|P39023\|RL3_HUMAN | 436 |
| IMPDH2 | HEL-S-128m | tr\|H0Y4R1\|H0Y4R1_HUMAN | tr\|V9HWC7\|V9HWC7_HUMAN | 538 |
| IMPDH2 | TKT | tr\|H0Y4R1\|H0Y4R1_HUMAN | tr\|Q53EM5\|Q53EM5_HUMAN | 547 |
| IMPDH2 | RPS6 | tr\|H0Y4R1\|H0Y4R1_HUMAN | tr\|A2A3R6\|A2A3R6_HUMAN | 711 |
| IMPDH2 | ATP5O | tr\|H0Y4R1\|H0Y4R1_HUMAN | sp\|P48047\|ATPO_HUMAN | 510 |
| IMPDH2 | HEL-S-100n | tr\|H0Y4R1\|H0Y4R1_HUMAN | tr\|V9HW96\|V9HW96_HUMAN | 733 |
| IMPDH2 | HEL-S-2a | tr\|H0Y4R1\|H0Y4R1_HUMAN | tr\|V9HW12\|V9HW12_HUMAN | 578 |
| IMPDH2 | HSPA9 | tr\|H0Y4R1\|H0Y4R1_HUMAN | tr\|Q8N1C8\|Q8N1C8_HUMAN | 663 |
| KRT19 | HEL-S-162eP | sp\|P08727\|K1C19_HUMAN | tr\|V9HVZ4\|V9HVZ4_HUMAN | 504 |
| KRT19 | DPP4 | sp\|P08727\|K1C19_HUMAN | sp\|P27487\|DPP4_HUMAN | 414 |
| HEL-S-22 | MYL6 | tr\|V9HWE9\|V9HWE9_HUMAN | tr\|G8JLA2\|G8JLA2_HUMAN | 610 |
| HEL-S-22 | HEL-S-128m | tr\|V9HWE9\|V9HWE9_HUMAN | tr\|V9HWC7\|V9HWC7_HUMAN | 967 |
| HEL-S-22 | TXNDC12 | tr\|V9HWE9\|V9HWE9_HUMAN | sp\|O95881\|TXD12_HUMAN | 901 |
| HEL-S-22 | HEL-S-162eP | tr\|V9HWE9\|V9HWE9_HUMAN | tr\|V9HVZ4\|V9HVZ4_HUMAN | 424 |
| ANXA1 | DHX15 | tr\|Q5TZZ9\|Q5TZZ9_HUMAN | sp\|O43143\|DHX15_HUMAN | 561 |
| ANXA1 | S100A10 | tr\|Q5TZZ9\|Q5TZZ9_HUMAN | tr\|D3DV26\|D3DV26_HUMAN | 575 |
| ANXA1 | HEL-S-162eP | tr\|Q5TZZ9\|Q5TZZ9_HUMAN | tr\|V9HVZ4\|V9HVZ4_HUMAN | 442 |
| ITGB4 | ACTN4 | tr\|B7ZLD5\|B7ZLD5_HUMAN | tr\|A0A0S2Z3G9\|A0A0S2Z3G9_HUMAN | 851 |
| ITGB4 | HEL2 | tr\|B7ZLD5\|B7ZLD5_HUMAN | tr\|V9HW98\|V9HW98_HUMAN | 900 |
| ITGB4 | HNRPU | tr\|B7ZLD5\|B7ZLD5_HUMAN | tr\|B4DLR3\|B4DLR3_HUMAN | 644 |
| CSTB | PHB2 | tr\|Q76LA1\|Q76LA1_HUMAN | sp\|Q99623\|PHB2_HUMAN | 418 |
| CSTB | SCARB2 | tr\|Q76LA1\|Q76LA1_HUMAN | tr\|A0A024RDG6\|A0A024RDG6_HUMAN | 664 |
| CSTB | HEL-S-130P | tr\|Q76LA1\|Q76LA1_HUMAN | tr\|V9HWI3\|V9HWI3_HUMAN | 694 |
| CSTB | HADH | tr\|Q76LA1\|Q76LA1_HUMAN | tr\|B2RB06\|B2RB06_HUMAN | 508 |
| CRABP2 | HEL-S-53e | sp\|P29373\|RABP2_HUMAN | tr\|V9HW83\|V9HW83_HUMAN | 874 |
| RPS5 | hCG_24487 | tr\|A0A024R4Q8\|A0A024R4Q8_HUMAN | tr\|A0A024R261\|A0A024R261_HUMAN | 999 |
| RPS5 | RPS3A | tr\|A0A024R4Q8\|A0A024R4Q8_HUMAN | tr\|Q6NXR8\|Q6NXR8_HUMAN | 999 |
| RPS5 | RPL7 | tr\|A0A024R4Q8\|A0A024R4Q8_HUMAN | tr\|A0A024R814\|A0A024R814_HUMAN | 999 |
| RPS5 | RPL13 | tr\|A0A024R4Q8\|A0A024R4Q8_HUMAN | tr\|Q6NZ55\|Q6NZ55_HUMAN | 999 |
| RPS5 | RPS18 | tr\|A0A024R4Q8\|A0A024R4Q8_HUMAN | sp\|P62269\|RS18_HUMAN | 992 |
| RPS5 | HNRPA1 | tr\|A0A024R4Q8\|A0A024R4Q8_HUMAN | tr\|A0A024RAZ7\|A0A024RAZ7_HUMAN | 659 |
| RPS5 | TARS | tr\|A0A024R4Q8\|A0A024R4Q8_HUMAN | tr\|Q53GX7\|Q53GX7_HUMAN | 516 |
| RPS5 | SRP14 | tr\|A0A024R4Q8\|A0A024R4Q8_HUMAN | sp\|P37108\|SRP14_HUMAN | 900 |
| RPS5 | RPS6 | tr\|A0A024R4Q8\|A0A024R4Q8_HUMAN | tr\|A2A3R6\|A2A3R6_HUMAN | 999 |
| RPS5 | HEL-S-100n | tr\|A0A024R4Q8\|A0A024R4Q8_HUMAN | tr\|V9HW96\|V9HW96_HUMAN | 478 |
| RPS5 | GSPT1 | tr\|A0A024R4Q8\|A0A024R4Q8_HUMAN | tr\|B2RCT6\|B2RCT6_HUMAN | 421 |
| RPS5 | HSPA9 | tr\|A0A024R4Q8\|A0A024R4Q8_HUMAN | tr\|Q8N1C8\|Q8N1C8_HUMAN | 406 |
| RPS5 | RPS9 | tr\|A0A024R4Q8\|A0A024R4Q8_HUMAN | tr\|A0A024R4M0\|A0A024R4M0_HUMAN | 999 |
| RPS5 | HEL-S-72p | tr\|A0A024R4Q8\|A0A024R4Q8_HUMAN | tr\|V9HW22\|V9HW22_HUMAN | 460 |
| RPS5 | HEL-S-123m | tr\|A0A024R4Q8\|A0A024R4Q8_HUMAN | tr\|V9HW26\|V9HW26_HUMAN | 583 |
| RPS5 | RPS8 | tr\|A0A024R4Q8\|A0A024R4Q8_HUMAN | tr\|Q5JR94\|Q5JR94_HUMAN | 999 |
| RPS5 | HEL103 | tr\|A0A024R4Q8\|A0A024R4Q8_HUMAN | tr\|V9HWK0\|V9HWK0_HUMAN | 900 |
| RPS5 | EEF1G | tr\|A0A024R4Q8\|A0A024R4Q8_HUMAN | tr\|Q53YD7\|Q53YD7_HUMAN | 667 |
| RPS5 | RPL26 | tr\|A0A024R4Q8\|A0A024R4Q8_HUMAN | sp\|P61254\|RL26_HUMAN | 999 |
| RPS5 | RPS13 | tr\|A0A024R4Q8\|A0A024R4Q8_HUMAN | sp\|P62277\|RS13_HUMAN | 999 |
| RPS5 | RPS7 | tr\|A0A024R4Q8\|A0A024R4Q8_HUMAN | sp\|P62081\|RS7_HUMAN | 999 |
| RPS5 | RPL19 | tr\|A0A024R4Q8\|A0A024R4Q8_HUMAN | tr\|Q53G49\|Q53G49_HUMAN | 999 |
| RPS5 | RPL18 | tr\|A0A024R4Q8\|A0A024R4Q8_HUMAN | tr\|J3QQ67\|J3QQ67_HUMAN | 999 |
| RPS5 | RPS27A | tr\|A0A024R4Q8\|A0A024R4Q8_HUMAN | tr\|B2RDW1\|B2RDW1_HUMAN | 994 |
| RPS5 | RPS3 | tr\|A0A024R4Q8\|A0A024R4Q8_HUMAN | sp\|P23396\|RS3_HUMAN | 999 |
| RPS5 | RAD23B | tr\|A0A024R4Q8\|A0A024R4Q8_HUMAN | sp\|P54727\|RD23B_HUMAN | 408 |
| RPS5 | HEL-S-68p | tr\|A0A024R4Q8\|A0A024R4Q8_HUMAN | tr\|V9HWF4\|V9HWF4_HUMAN | 489 |
| RPS5 | CCT4 | tr\|A0A024R4Q8\|A0A024R4Q8_HUMAN | sp\|P50991\|TCPD_HUMAN | 410 |
| RPS5 | HEL-S-271 | tr\|A0A024R4Q8\|A0A024R4Q8_HUMAN | tr\|V9HW31\|V9HW31_HUMAN | 405 |
| RPS5 | RPL3 | tr\|A0A024R4Q8\|A0A024R4Q8_HUMAN | sp\|P39023\|RL3_HUMAN | 999 |
| RPS5 | RPLP2 | tr\|A0A024R4Q8\|A0A024R4Q8_HUMAN | tr\|A0A024RCA7\|A0A024RCA7_HUMAN | 998 |
| RPS5 | ATP5O | tr\|A0A024R4Q8\|A0A024R4Q8_HUMAN | sp\|P48047\|ATPO_HUMAN | 489 |
| RPS5 | RPS19 | tr\|A0A024R4Q8\|A0A024R4Q8_HUMAN | tr\|Q8WVX7\|Q8WVX7_HUMAN | 999 |
| TALDO1 | TKT | tr\|A0A140VK56\|A0A140VK56_HUMAN | tr\|Q53EM5\|Q53EM5_HUMAN | 998 |
| TALDO1 | HEL-S-26 | tr\|A0A140VK56\|A0A140VK56_HUMAN | tr\|V9HWJ2\|V9HWJ2_HUMAN | 578 |
| TALDO1 | TPI1 | tr\|A0A140VK56\|A0A140VK56_HUMAN | sp\|P60174\|TPIS_HUMAN | 424 |
| TALDO1 | HEL-S-162eP | tr\|A0A140VK56\|A0A140VK56_HUMAN | tr\|V9HVZ4\|V9HVZ4_HUMAN | 878 |
| TALDO1 | MDH2 | tr\|A0A140VK56\|A0A140VK56_HUMAN | tr\|Q6FHZ0\|Q6FHZ0_HUMAN | 527 |
| SCP2 | HSD17B4 | tr\|B2R761\|B2R761_HUMAN | tr\|B2R659\|B2R659_HUMAN | 984 |
| SCP2 | VDAC2 | tr\|B2R761\|B2R761_HUMAN | tr\|A0A024QZN9\|A0A024QZN9_HUMAN | 644 |
| SCP2 | HEL-S-89n | tr\|B2R761\|B2R761_HUMAN | tr\|V9HWB4\|V9HWB4_HUMAN | 408 |
| S100A6 | STUB1 | sp\|P06703\|S10A6_HUMAN | sp\|Q9UNE7\|CHIP_HUMAN | 574 |
| S100A6 | MKI67 | sp\|P06703\|S10A6_HUMAN | tr\|A0A087WV66\|A0A087WV66_HUMAN | 506 |
| S100A6 | S100A10 | sp\|P06703\|S10A6_HUMAN | tr\|D3DV26\|D3DV26_HUMAN | 638 |
| S100A6 | HEL-S-162eP | sp\|P06703\|S10A6_HUMAN | tr\|V9HVZ4\|V9HVZ4_HUMAN | 625 |
| HEL-S-100n | hCG_24487 | tr\|V9HW96\|V9HW96_HUMAN | tr\|A0A024R261\|A0A024R261_HUMAN | 635 |
| HEL-S-100n | SSB | tr\|V9HW96\|V9HW96_HUMAN | tr\|B5BUB5\|B5BUB5_HUMAN | 424 |
| HEL-S-100n | HEL-S-103 | tr\|V9HW96\|V9HW96_HUMAN | tr\|A8K5I0\|A8K5I0_HUMAN | 757 |
| HEL-S-100n | HEL-S-89n | tr\|V9HW96\|V9HW96_HUMAN | tr\|V9HWB4\|V9HWB4_HUMAN | 776 |
| HEL-S-100n | RPL7 | tr\|V9HW96\|V9HW96_HUMAN | tr\|A0A024R814\|A0A024R814_HUMAN | 429 |
| HEL-S-100n | PHB2 | tr\|V9HW96\|V9HW96_HUMAN | sp\|Q99623\|PHB2_HUMAN | 419 |
| HEL-S-100n | RPL13 | tr\|V9HW96\|V9HW96_HUMAN | tr\|Q6NZ55\|Q6NZ55_HUMAN | 414 |
| HEL-S-100n | DLD | tr\|V9HW96\|V9HW96_HUMAN | tr\|A0A024R713\|A0A024R713_HUMAN | 403 |
| HEL-S-100n | TARS | tr\|V9HW96\|V9HW96_HUMAN | tr\|Q53GX7\|Q53GX7_HUMAN | 655 |
| HEL-S-100n | PFDN6 | tr\|V9HW96\|V9HW96_HUMAN | tr\|Q5STK2\|Q5STK2_HUMAN | 415 |
| HEL-S-100n | P4HB | tr\|V9HW96\|V9HW96_HUMAN | tr\|A0A024R8S5\|A0A024R8S5_HUMAN | 413 |
| HEL-S-100n | RPS6 | tr\|V9HW96\|V9HW96_HUMAN | tr\|A2A3R6\|A2A3R6_HUMAN | 470 |
| HEL-S-100n | HEL-S-2a | tr\|V9HW96\|V9HW96_HUMAN | tr\|V9HW12\|V9HW12_HUMAN | 469 |
| HEL-S-100n | GSPT1 | tr\|V9HW96\|V9HW96_HUMAN | tr\|B2RCT6\|B2RCT6_HUMAN | 775 |
| HEL-S-100n | ERP70 | tr\|V9HW96\|V9HW96_HUMAN | tr\|A0A090N8Y2\|A0A090N8Y2_HUMAN | 409 |
| HEL-S-100n | HSPA9 | tr\|V9HW96\|V9HW96_HUMAN | tr\|Q8N1C8\|Q8N1C8_HUMAN | 782 |
| HEL-S-100n | HEL-S-133P | tr\|V9HW96\|V9HW96_HUMAN | tr\|V9HWB9\|V9HWB9_HUMAN | 505 |
| HEL-S-100n | RPS9 | tr\|V9HW96\|V9HW96_HUMAN | tr\|A0A024R4M0\|A0A024R4M0_HUMAN | 487 |
| HEL-S-100n | HEL-S-72p | tr\|V9HW96\|V9HW96_HUMAN | tr\|V9HW22\|V9HW22_HUMAN | 789 |
| HEL-S-100n | HEL-S-162eP | tr\|V9HW96\|V9HW96_HUMAN | tr\|V9HVZ4\|V9HVZ4_HUMAN | 481 |
| HEL-S-100n | GLUD1 | tr\|V9HW96\|V9HW96_HUMAN | tr\|E9KL48\|E9KL48_HUMAN | 415 |
| HEL-S-100n | RPS8 | tr\|V9HW96\|V9HW96_HUMAN | tr\|Q5JR94\|Q5JR94_HUMAN | 419 |
| HEL-S-100n | EEF1G | tr\|V9HW96\|V9HW96_HUMAN | tr\|Q53YD7\|Q53YD7_HUMAN | 620 |
| HEL-S-100n | HEL-S-70 | tr\|V9HW96\|V9HW96_HUMAN | tr\|V9HW80\|V9HW80_HUMAN | 722 |
| HEL-S-100n | RPS13 | tr\|V9HW96\|V9HW96_HUMAN | sp\|P62277\|RS13_HUMAN | 624 |
| HEL-S-100n | RPL18 | tr\|V9HW96\|V9HW96_HUMAN | tr\|J3QQ67\|J3QQ67_HUMAN | 458 |
| HEL-S-100n | EL52 | tr\|V9HW96\|V9HW96_HUMAN | tr\|K9JA46\|K9JA46_HUMAN | 915 |
| HEL-S-100n | RPS3 | tr\|V9HW96\|V9HW96_HUMAN | sp\|P23396\|RS3_HUMAN | 754 |
| HEL-S-100n | HEL-S-269 | tr\|V9HW96\|V9HW96_HUMAN | tr\|V9HVY3\|V9HVY3_HUMAN | 489 |
| HEL-S-100n | TRA1 | tr\|V9HW96\|V9HW96_HUMAN | tr\|Q5CAQ5\|Q5CAQ5_HUMAN | 904 |
| HEL-S-100n | PDIA6 | tr\|V9HW96\|V9HW96_HUMAN | sp\|Q15084\|PDIA6_HUMAN | 470 |
| HEL-S-100n | MDH2 | tr\|V9HW96\|V9HW96_HUMAN | tr\|Q6FHZ0\|Q6FHZ0_HUMAN | 530 |
| HEL-S-100n | HEL-S-271 | tr\|V9HW96\|V9HW96_HUMAN | tr\|V9HW31\|V9HW31_HUMAN | 414 |
| HEL-S-100n | CCT4 | tr\|V9HW96\|V9HW96_HUMAN | sp\|P50991\|TCPD_HUMAN | 999 |
| HEL-S-100n | RPL3 | tr\|V9HW96\|V9HW96_HUMAN | sp\|P39023\|RL3_HUMAN | 572 |
| HEL-S-100n | HEL-S-128m | tr\|V9HW96\|V9HW96_HUMAN | tr\|V9HWC7\|V9HWC7_HUMAN | 465 |
| GSPT1 | EL52 | tr\|B2RCT6\|B2RCT6_HUMAN | tr\|K9JA46\|K9JA46_HUMAN | 579 |
| GSPT1 | RPS3A | tr\|B2RCT6\|B2RCT6_HUMAN | tr\|Q6NXR8\|Q6NXR8_HUMAN | 470 |
| GSPT1 | HEL-S-72p | tr\|B2RCT6\|B2RCT6_HUMAN | tr\|V9HW22\|V9HW22_HUMAN | 452 |
| GSPT1 | HEL-S-103 | tr\|B2RCT6\|B2RCT6_HUMAN | tr\|A8K5I0\|A8K5I0_HUMAN | 440 |
| GSPT1 | CCT4 | tr\|B2RCT6\|B2RCT6_HUMAN | sp\|P50991\|TCPD_HUMAN | 553 |
| GSPT1 | HEL-S-89n | tr\|B2RCT6\|B2RCT6_HUMAN | tr\|V9HWB4\|V9HWB4_HUMAN | 440 |
| GSPT1 | AARS | tr\|B2RCT6\|B2RCT6_HUMAN | sp\|P49588\|SYAC_HUMAN | 424 |
| GSPT1 | PRIC295 | tr\|B2RCT6\|B2RCT6_HUMAN | tr\|E1NZA1\|E1NZA1_HUMAN | 466 |
| GSPT1 | EEF1G | tr\|B2RCT6\|B2RCT6_HUMAN | tr\|Q53YD7\|Q53YD7_HUMAN | 883 |
| SUB1 | HEL-S-133P | tr\|Q59G24\|Q59G24_HUMAN | tr\|V9HWB9\|V9HWB9_HUMAN | 517 |
| SUB1 | RPS3A | tr\|Q59G24\|Q59G24_HUMAN | tr\|Q6NXR8\|Q6NXR8_HUMAN | 405 |
| SUB1 | LASP1 | tr\|Q59G24\|Q59G24_HUMAN | tr\|A0A024R1S8\|A0A024R1S8_HUMAN | 455 |
| HEL-S-72p | PSMC3 | tr\|V9HW22\|V9HW22_HUMAN | tr\|A0A140VK42\|A0A140VK42_HUMAN | 924 |
| HEL-S-72p | HEL-S-103 | tr\|V9HW22\|V9HW22_HUMAN | tr\|A8K5I0\|A8K5I0_HUMAN | 571 |
| HEL-S-72p | LMNB1 | tr\|V9HW22\|V9HW22_HUMAN | sp\|P20700\|LMNB1_HUMAN | 572 |
| HEL-S-72p | HEL-S-89n | tr\|V9HW22\|V9HW22_HUMAN | tr\|V9HWB4\|V9HWB4_HUMAN | 608 |
| HEL-S-72p | RPS18 | tr\|V9HW22\|V9HW22_HUMAN | sp\|P62269\|RS18_HUMAN | 435 |
| HEL-S-72p | CANX | tr\|V9HW22\|V9HW22_HUMAN | sp\|P27824\|CALX_HUMAN | 849 |
| HEL-S-72p | STUB1 | tr\|V9HW22\|V9HW22_HUMAN | sp\|Q9UNE7\|CHIP_HUMAN | 999 |
| HEL-S-72p | TPI1 | tr\|V9HW22\|V9HW22_HUMAN | sp\|P60174\|TPIS_HUMAN | 420 |
| HEL-S-72p | TARS | tr\|V9HW22\|V9HW22_HUMAN | tr\|Q53GX7\|Q53GX7_HUMAN | 422 |
| HEL-S-72p | LMNA | tr\|V9HW22\|V9HW22_HUMAN | sp\|P02545\|LMNA_HUMAN | 506 |
| HEL-S-72p | TKT | tr\|V9HW22\|V9HW22_HUMAN | tr\|Q53EM5\|Q53EM5_HUMAN | 510 |
| HEL-S-72p | P4HB | tr\|V9HW22\|V9HW22_HUMAN | tr\|A0A024R8S5\|A0A024R8S5_HUMAN | 638 |
| HEL-S-72p | HEL70 | tr\|V9HW22\|V9HW22_HUMAN | tr\|V9HWC0\|V9HWC0_HUMAN | 576 |
| HEL-S-72p | PSMA4 | tr\|V9HW22\|V9HW22_HUMAN | sp\|P25789\|PSA4_HUMAN | 912 |
| HEL-S-72p | HEL-S-2a | tr\|V9HW22\|V9HW22_HUMAN | tr\|V9HW12\|V9HW12_HUMAN | 540 |
| HEL-S-72p | ERP70 | tr\|V9HW22\|V9HW22_HUMAN | tr\|A0A090N8Y2\|A0A090N8Y2_HUMAN | 689 |
| HEL-S-72p | HEL-S-133P | tr\|V9HW22\|V9HW22_HUMAN | tr\|V9HWB9\|V9HWB9_HUMAN | 758 |
| HEL-S-72p | RPS9 | tr\|V9HW22\|V9HW22_HUMAN | tr\|A0A024R4M0\|A0A024R4M0_HUMAN | 461 |
| HEL-S-72p | HEL-S-162eP | tr\|V9HW22\|V9HW22_HUMAN | tr\|V9HVZ4\|V9HVZ4_HUMAN | 783 |
| HEL-S-72p | HEL-S-123m | tr\|V9HW22\|V9HW22_HUMAN | tr\|V9HW26\|V9HW26_HUMAN | 496 |
| HEL-S-72p | HEL-S-94n | tr\|V9HW22\|V9HW22_HUMAN | tr\|V9HW72\|V9HW72_HUMAN | 998 |
| HEL-S-72p | PPIB | tr\|V9HW22\|V9HW22_HUMAN | sp\|P23284\|PPIB_HUMAN | 422 |
| HEL-S-72p | EEF1G | tr\|V9HW22\|V9HW22_HUMAN | tr\|Q53YD7\|Q53YD7_HUMAN | 522 |
| HEL-S-72p | HEL2 | tr\|V9HW22\|V9HW22_HUMAN | tr\|V9HW98\|V9HW98_HUMAN | 668 |
| HEL-S-72p | HEL-S-70 | tr\|V9HW22\|V9HW22_HUMAN | tr\|V9HW80\|V9HW80_HUMAN | 937 |
| HEL-S-72p | RPS13 | tr\|V9HW22\|V9HW22_HUMAN | sp\|P62277\|RS13_HUMAN | 629 |
| HEL-S-72p | PSMD4 | tr\|V9HW22\|V9HW22_HUMAN | sp\|P55036\|PSMD4_HUMAN | 926 |
| HEL-S-72p | PTBP1 | tr\|V9HW22\|V9HW22_HUMAN | sp\|P26599\|PTBP1_HUMAN | 552 |
| HEL-S-72p | EPS8 | tr\|V9HW22\|V9HW22_HUMAN | sp\|Q12929\|EPS8_HUMAN | 804 |
| HEL-S-72p | RPS3 | tr\|V9HW22\|V9HW22_HUMAN | sp\|P23396\|RS3_HUMAN | 568 |
| HEL-S-72p | RPS27A | tr\|V9HW22\|V9HW22_HUMAN | tr\|B2RDW1\|B2RDW1_HUMAN | 923 |
| HEL-S-72p | EL52 | tr\|V9HW22\|V9HW22_HUMAN | tr\|K9JA46\|K9JA46_HUMAN | 999 |
| HEL-S-72p | EZR | tr\|V9HW22\|V9HW22_HUMAN | tr\|E7EQR4\|E7EQR4_HUMAN | 818 |
| HEL-S-72p | HEL-S-269 | tr\|V9HW22\|V9HW22_HUMAN | tr\|V9HVY3\|V9HVY3_HUMAN | 914 |
| HEL-S-72p | HEL-S-130P | tr\|V9HW22\|V9HW22_HUMAN | tr\|V9HWI3\|V9HWI3_HUMAN | 534 |
| HEL-S-72p | TRA1 | tr\|V9HW22\|V9HW22_HUMAN | tr\|Q5CAQ5\|Q5CAQ5_HUMAN | 925 |
| HEL-S-72p | HNRNPA2B1 | tr\|V9HW22\|V9HW22_HUMAN | sp\|P22626\|ROA2_HUMAN | 453 |
| HEL-S-72p | PDIA6 | tr\|V9HW22\|V9HW22_HUMAN | sp\|Q15084\|PDIA6_HUMAN | 568 |
| HEL-S-72p | RAB1A | tr\|V9HW22\|V9HW22_HUMAN | tr\|Q5U0I6\|Q5U0I6_HUMAN | 902 |
| HEL-S-72p | HEL-S-68p | tr\|V9HW22\|V9HW22_HUMAN | tr\|V9HWF4\|V9HWF4_HUMAN | 419 |
| HEL-S-72p | HEL-S-271 | tr\|V9HW22\|V9HW22_HUMAN | tr\|V9HW31\|V9HW31_HUMAN | 541 |
| HEL-S-72p | CCT4 | tr\|V9HW22\|V9HW22_HUMAN | sp\|P50991\|TCPD_HUMAN | 781 |
| HEL-S-72p | UBE1 | tr\|V9HW22\|V9HW22_HUMAN | tr\|A0A024R1A3\|A0A024R1A3_HUMAN | 468 |
| HEL-S-72p | RPL3 | tr\|V9HW22\|V9HW22_HUMAN | sp\|P39023\|RL3_HUMAN | 525 |
| HEL-S-72p | HEL-S-128m | tr\|V9HW22\|V9HW22_HUMAN | tr\|V9HWC7\|V9HWC7_HUMAN | 542 |
| HEL-S-53e | TKT | tr\|V9HW83\|V9HW83_HUMAN | tr\|Q53EM5\|Q53EM5_HUMAN | 615 |
| HEL-S-53e | HEL-S-26 | tr\|V9HW83\|V9HW83_HUMAN | tr\|V9HWJ2\|V9HWJ2_HUMAN | 498 |
| HEL-S-53e | HADHA | tr\|V9HW83\|V9HW83_HUMAN | tr\|E9KL44\|E9KL44_HUMAN | 482 |
| HEL-S-53e | HEL-S-162eP | tr\|V9HW83\|V9HW83_HUMAN | tr\|V9HVZ4\|V9HVZ4_HUMAN | 552 |
| HEL-S-53e | MDH2 | tr\|V9HW83\|V9HW83_HUMAN | tr\|Q6FHZ0\|Q6FHZ0_HUMAN | 778 |
| HEL103 | hCG_24487 | tr\|V9HWK0\|V9HWK0_HUMAN | tr\|A0A024R261\|A0A024R261_HUMAN | 900 |
| HEL103 | RPS3A | tr\|V9HWK0\|V9HWK0_HUMAN | tr\|Q6NXR8\|Q6NXR8_HUMAN | 900 |
| HEL103 | SSB | tr\|V9HWK0\|V9HWK0_HUMAN | tr\|B5BUB5\|B5BUB5_HUMAN | 957 |
| HEL103 | RPS8 | tr\|V9HWK0\|V9HWK0_HUMAN | tr\|Q5JR94\|Q5JR94_HUMAN | 900 |
| HEL103 | RPL7 | tr\|V9HWK0\|V9HWK0_HUMAN | tr\|A0A024R814\|A0A024R814_HUMAN | 900 |
| HEL103 | RPL13 | tr\|V9HWK0\|V9HWK0_HUMAN | tr\|Q6NZ55\|Q6NZ55_HUMAN | 900 |
| HEL103 | RPL26 | tr\|V9HWK0\|V9HWK0_HUMAN | sp\|P61254\|RL26_HUMAN | 906 |
| HEL103 | HEL-S-70 | tr\|V9HWK0\|V9HWK0_HUMAN | tr\|V9HW80\|V9HW80_HUMAN | 409 |
| HEL103 | RPS7 | tr\|V9HWK0\|V9HWK0_HUMAN | sp\|P62081\|RS7_HUMAN | 900 |
| HEL103 | RPS13 | tr\|V9HWK0\|V9HWK0_HUMAN | sp\|P62277\|RS13_HUMAN | 900 |
| HEL103 | RPL19 | tr\|V9HWK0\|V9HWK0_HUMAN | tr\|Q53G49\|Q53G49_HUMAN | 900 |
| HEL103 | RPL18 | tr\|V9HWK0\|V9HWK0_HUMAN | tr\|J3QQ67\|J3QQ67_HUMAN | 900 |
| HEL103 | RPS3 | tr\|V9HWK0\|V9HWK0_HUMAN | sp\|P23396\|RS3_HUMAN | 900 |
| HEL103 | EL52 | tr\|V9HWK0\|V9HWK0_HUMAN | tr\|K9JA46\|K9JA46_HUMAN | 733 |
| HEL103 | RPS27A | tr\|V9HWK0\|V9HWK0_HUMAN | tr\|B2RDW1\|B2RDW1_HUMAN | 905 |
| HEL103 | RPLP2 | tr\|V9HWK0\|V9HWK0_HUMAN | tr\|A0A024RCA7\|A0A024RCA7_HUMAN | 900 |
| HEL103 | RPL3 | tr\|V9HWK0\|V9HWK0_HUMAN | sp\|P39023\|RL3_HUMAN | 924 |
| HEL103 | SRP14 | tr\|V9HWK0\|V9HWK0_HUMAN | sp\|P37108\|SRP14_HUMAN | 999 |
| HEL103 | RPS6 | tr\|V9HWK0\|V9HWK0_HUMAN | tr\|A2A3R6\|A2A3R6_HUMAN | 900 |
| HEL103 | RPS19 | tr\|V9HWK0\|V9HWK0_HUMAN | tr\|Q8WVX7\|Q8WVX7_HUMAN | 902 |
| SCARB2 | HEL-S-130P | tr\|A0A024RDG6\|A0A024RDG6_HUMAN | tr\|V9HWI3\|V9HWI3_HUMAN | 416 |
| PPIB | EL52 | sp\|P23284\|PPIB_HUMAN | tr\|K9JA46\|K9JA46_HUMAN | 585 |
| PPIB | HEL-S-162eP | sp\|P23284\|PPIB_HUMAN | tr\|V9HVZ4\|V9HVZ4_HUMAN | 574 |
| PPIB | HEL-S-269 | sp\|P23284\|PPIB_HUMAN | tr\|V9HVY3\|V9HVY3_HUMAN | 978 |
| PPIB | PDIA6 | sp\|P23284\|PPIB_HUMAN | sp\|Q15084\|PDIA6_HUMAN | 532 |
| PPIB | TRA1 | sp\|P23284\|PPIB_HUMAN | tr\|Q5CAQ5\|Q5CAQ5_HUMAN | 819 |
| PPIB | HEL-S-89n | sp\|P23284\|PPIB_HUMAN | tr\|V9HWB4\|V9HWB4_HUMAN | 946 |
| PPIB | P4HB | sp\|P23284\|PPIB_HUMAN | tr\|A0A024R8S5\|A0A024R8S5_HUMAN | 862 |
| PPIB | ERP70 | sp\|P23284\|PPIB_HUMAN | tr\|A0A090N8Y2\|A0A090N8Y2_HUMAN | 951 |
| PPIB | CANX | sp\|P23284\|PPIB_HUMAN | sp\|P27824\|CALX_HUMAN | 431 |
| TAGLN2 | ACTN4 | sp\|P37802\|TAGL2_HUMAN | tr\|A0A0S2Z3G9\|A0A0S2Z3G9_HUMAN | 585 |
| TAGLN2 | SPTBN1 | sp\|P37802\|TAGL2_HUMAN | tr\|D6W5C0\|D6W5C0_HUMAN | 527 |
| UBE2V1 | RPS27A | sp\|Q13404\|UB2V1_HUMAN | tr\|B2RDW1\|B2RDW1_HUMAN | 427 |
| UBE2V1 | STUB1 | sp\|Q13404\|UB2V1_HUMAN | sp\|Q9UNE7\|CHIP_HUMAN | 991 |
| UBE2V1 | PSMA4 | sp\|Q13404\|UB2V1_HUMAN | sp\|P25789\|PSA4_HUMAN | 427 |
| UBE2V1 | UBE1 | sp\|Q13404\|UB2V1_HUMAN | tr\|A0A024R1A3\|A0A024R1A3_HUMAN | 498 |
| HEL2 | EL52 | tr\|V9HW98\|V9HW98_HUMAN | tr\|K9JA46\|K9JA46_HUMAN | 953 |
| HEL2 | PAFAH1B1 | tr\|V9HW98\|V9HW98_HUMAN | sp\|P43034\|LIS1_HUMAN | 974 |
| HEL2 | DYNC1H1 | tr\|V9HW98\|V9HW98_HUMAN | sp\|Q14204\|DYHC1_HUMAN | 903 |
| HEL2 | HEL-S-128m | tr\|V9HW98\|V9HW98_HUMAN | tr\|V9HWC7\|V9HWC7_HUMAN | 724 |
| HEL2 | EEF1G | tr\|V9HW98\|V9HW98_HUMAN | tr\|Q53YD7\|Q53YD7_HUMAN | 608 |
| HEL2 | TMOD3 | tr\|V9HW98\|V9HW98_HUMAN | sp\|Q9NYL9\|TMOD3_HUMAN | 422 |
| HEL2 | ERP70 | tr\|V9HW98\|V9HW98_HUMAN | tr\|A0A090N8Y2\|A0A090N8Y2_HUMAN | 628 |
| HEL2 | RPL19 | tr\|V9HW98\|V9HW98_HUMAN | tr\|Q53G49\|Q53G49_HUMAN | 584 |
| RPL19 | hCG_24487 | tr\|Q53G49\|Q53G49_HUMAN | tr\|A0A024R261\|A0A024R261_HUMAN | 999 |
| RPL19 | RPS9 | tr\|Q53G49\|Q53G49_HUMAN | tr\|A0A024R4M0\|A0A024R4M0_HUMAN | 984 |
| RPL19 | RPS3A | tr\|Q53G49\|Q53G49_HUMAN | tr\|Q6NXR8\|Q6NXR8_HUMAN | 999 |
| RPL19 | HEL-S-162eP | tr\|Q53G49\|Q53G49_HUMAN | tr\|V9HVZ4\|V9HVZ4_HUMAN | 519 |
| RPL19 | RPS8 | tr\|Q53G49\|Q53G49_HUMAN | tr\|Q5JR94\|Q5JR94_HUMAN | 999 |
| RPL19 | RPL7 | tr\|Q53G49\|Q53G49_HUMAN | tr\|A0A024R814\|A0A024R814_HUMAN | 999 |
| RPL19 | PRIC295 | tr\|Q53G49\|Q53G49_HUMAN | tr\|E1NZA1\|E1NZA1_HUMAN | 545 |
| RPL19 | RPL13 | tr\|Q53G49\|Q53G49_HUMAN | tr\|Q6NZ55\|Q6NZ55_HUMAN | 999 |
| RPL19 | EEF1G | tr\|Q53G49\|Q53G49_HUMAN | tr\|Q53YD7\|Q53YD7_HUMAN | 790 |
| RPL19 | RPL26 | tr\|Q53G49\|Q53G49_HUMAN | sp\|P61254\|RL26_HUMAN | 999 |
| RPL19 | RPS18 | tr\|Q53G49\|Q53G49_HUMAN | sp\|P62269\|RS18_HUMAN | 982 |
| RPL19 | RPS7 | tr\|Q53G49\|Q53G49_HUMAN | sp\|P62081\|RS7_HUMAN | 999 |
| RPL19 | RPS13 | tr\|Q53G49\|Q53G49_HUMAN | sp\|P62277\|RS13_HUMAN | 999 |
| RPL19 | RPL18 | tr\|Q53G49\|Q53G49_HUMAN | tr\|J3QQ67\|J3QQ67_HUMAN | 999 |
| RPL19 | RPS27A | tr\|Q53G49\|Q53G49_HUMAN | tr\|B2RDW1\|B2RDW1_HUMAN | 999 |
| RPL19 | RPS3 | tr\|Q53G49\|Q53G49_HUMAN | sp\|P23396\|RS3_HUMAN | 999 |
| RPL19 | RAD23B | tr\|Q53G49\|Q53G49_HUMAN | sp\|P54727\|RD23B_HUMAN | 477 |
| RPL19 | RPLP2 | tr\|Q53G49\|Q53G49_HUMAN | tr\|A0A024RCA7\|A0A024RCA7_HUMAN | 999 |
| RPL19 | RPL3 | tr\|Q53G49\|Q53G49_HUMAN | sp\|P39023\|RL3_HUMAN | 999 |
| RPL19 | SRP14 | tr\|Q53G49\|Q53G49_HUMAN | sp\|P37108\|SRP14_HUMAN | 954 |
| RPL19 | RPS6 | tr\|Q53G49\|Q53G49_HUMAN | tr\|A2A3R6\|A2A3R6_HUMAN | 999 |
| RPL19 | RPS19 | tr\|Q53G49\|Q53G49_HUMAN | tr\|Q8WVX7\|Q8WVX7_HUMAN | 999 |
| EPS8 | ACTN4 | sp\|Q12929\|EPS8_HUMAN | tr\|A0A0S2Z3G9\|A0A0S2Z3G9_HUMAN | 440 |
| SPTBN1 | ACTN4 | tr\|D6W5C0\|D6W5C0_HUMAN | tr\|A0A0S2Z3G9\|A0A0S2Z3G9_HUMAN | 904 |
| SPTBN1 | MYH9 | tr\|D6W5C0\|D6W5C0_HUMAN | tr\|A0A024R1N1\|A0A024R1N1_HUMAN | 497 |
| SPTBN1 | CANX | tr\|D6W5C0\|D6W5C0_HUMAN | sp\|P27824\|CALX_HUMAN | 440 |
| SPTBN1 | HEL-S-89n | tr\|D6W5C0\|D6W5C0_HUMAN | tr\|V9HWB4\|V9HWB4_HUMAN | 783 |
| EL52 | hCG_24487 | tr\|K9JA46\|K9JA46_HUMAN | tr\|A0A024R261\|A0A024R261_HUMAN | 804 |
| EL52 | RPS3A | tr\|K9JA46\|K9JA46_HUMAN | tr\|Q6NXR8\|Q6NXR8_HUMAN | 659 |
| EL52 | VDAC2 | tr\|K9JA46\|K9JA46_HUMAN | tr\|A0A024QZN9\|A0A024QZN9_HUMAN | 579 |
| EL52 | HEL-S-103 | tr\|K9JA46\|K9JA46_HUMAN | tr\|A8K5I0\|A8K5I0_HUMAN | 984 |
| EL52 | HEL-S-89n | tr\|K9JA46\|K9JA46_HUMAN | tr\|V9HWB4\|V9HWB4_HUMAN | 992 |
| EL52 | RPL7 | tr\|K9JA46\|K9JA46_HUMAN | tr\|A0A024R814\|A0A024R814_HUMAN | 760 |
| EL52 | HNRPA1 | tr\|K9JA46\|K9JA46_HUMAN | tr\|A0A024RAZ7\|A0A024RAZ7_HUMAN | 770 |
| EL52 | CANX | tr\|K9JA46\|K9JA46_HUMAN | sp\|P27824\|CALX_HUMAN | 941 |
| EL52 | STUB1 | tr\|K9JA46\|K9JA46_HUMAN | sp\|Q9UNE7\|CHIP_HUMAN | 999 |
| EL52 | P4HB | tr\|K9JA46\|K9JA46_HUMAN | tr\|A0A024R8S5\|A0A024R8S5_HUMAN | 666 |
| EL52 | RPS6 | tr\|K9JA46\|K9JA46_HUMAN | tr\|A2A3R6\|A2A3R6_HUMAN | 772 |
| EL52 | HEL-S-2a | tr\|K9JA46\|K9JA46_HUMAN | tr\|V9HW12\|V9HW12_HUMAN | 496 |
| EL52 | ERP70 | tr\|K9JA46\|K9JA46_HUMAN | tr\|A0A090N8Y2\|A0A090N8Y2_HUMAN | 757 |
| EL52 | HSPA9 | tr\|K9JA46\|K9JA46_HUMAN | tr\|Q8N1C8\|Q8N1C8_HUMAN | 989 |
| EL52 | HEL-S-133P | tr\|K9JA46\|K9JA46_HUMAN | tr\|V9HWB9\|V9HWB9_HUMAN | 431 |
| EL52 | PAFAH1B1 | tr\|K9JA46\|K9JA46_HUMAN | sp\|P43034\|LIS1_HUMAN | 998 |
| EL52 | HEL-S-162eP | tr\|K9JA46\|K9JA46_HUMAN | tr\|V9HVZ4\|V9HVZ4_HUMAN | 871 |
| EL52 | HEL-S-123m | tr\|K9JA46\|K9JA46_HUMAN | tr\|V9HW26\|V9HW26_HUMAN | 888 |
| EL52 | PRIC295 | tr\|K9JA46\|K9JA46_HUMAN | tr\|E1NZA1\|E1NZA1_HUMAN | 417 |
| EL52 | EEF1G | tr\|K9JA46\|K9JA46_HUMAN | tr\|Q53YD7\|Q53YD7_HUMAN | 872 |
| EL52 | HEL-S-94n | tr\|K9JA46\|K9JA46_HUMAN | tr\|V9HW72\|V9HW72_HUMAN | 999 |
| EL52 | HEL-S-70 | tr\|K9JA46\|K9JA46_HUMAN | tr\|V9HW80\|V9HW80_HUMAN | 888 |
| EL52 | PSMD4 | tr\|K9JA46\|K9JA46_HUMAN | sp\|P55036\|PSMD4_HUMAN | 877 |
| EL52 | RPS27A | tr\|K9JA46\|K9JA46_HUMAN | tr\|B2RDW1\|B2RDW1_HUMAN | 939 |
| EL52 | RPS3 | tr\|K9JA46\|K9JA46_HUMAN | sp\|P23396\|RS3_HUMAN | 934 |
| EL52 | HEL-S-130P | tr\|K9JA46\|K9JA46_HUMAN | tr\|V9HWI3\|V9HWI3_HUMAN | 414 |
| EL52 | HEL-S-269 | tr\|K9JA46\|K9JA46_HUMAN | tr\|V9HVY3\|V9HVY3_HUMAN | 784 |
| EL52 | TRA1 | tr\|K9JA46\|K9JA46_HUMAN | tr\|Q5CAQ5\|Q5CAQ5_HUMAN | 655 |
| EL52 | PDIA6 | tr\|K9JA46\|K9JA46_HUMAN | sp\|Q15084\|PDIA6_HUMAN | 621 |
| EL52 | HEL-S-68p | tr\|K9JA46\|K9JA46_HUMAN | tr\|V9HWF4\|V9HWF4_HUMAN | 659 |
| EL52 | DYNC1H1 | tr\|K9JA46\|K9JA46_HUMAN | sp\|Q14204\|DYHC1_HUMAN | 956 |
| EL52 | HEL-S-271 | tr\|K9JA46\|K9JA46_HUMAN | tr\|V9HW31\|V9HW31_HUMAN | 970 |
| EL52 | CCT4 | tr\|K9JA46\|K9JA46_HUMAN | sp\|P50991\|TCPD_HUMAN | 817 |
| EL52 | RPL3 | tr\|K9JA46\|K9JA46_HUMAN | sp\|P39023\|RL3_HUMAN | 904 |
| EL52 | UBE1 | tr\|K9JA46\|K9JA46_HUMAN | tr\|A0A024R1A3\|A0A024R1A3_HUMAN | 466 |
| EL52 | HEL-S-128m | tr\|K9JA46\|K9JA46_HUMAN | tr\|V9HWC7\|V9HWC7_HUMAN | 454 |
| EL52 | SNRNP200 | tr\|K9JA46\|K9JA46_HUMAN | sp\|O75643\|U520_HUMAN | 516 |
| EL52 | ATP5O | tr\|K9JA46\|K9JA46_HUMAN | sp\|P48047\|ATPO_HUMAN | 677 |
| EL52 | MYH9 | tr\|K9JA46\|K9JA46_HUMAN | tr\|A0A024R1N1\|A0A024R1N1_HUMAN | 789 |
| HEL-S-130P | DPP4 | tr\|V9HWI3\|V9HWI3_HUMAN | sp\|P27487\|DPP4_HUMAN | 447 |
| HEL-S-130P | HEL-S-162eP | tr\|V9HWI3\|V9HWI3_HUMAN | tr\|V9HVZ4\|V9HVZ4_HUMAN | 535 |
| HEL-S-130P | PDIA6 | tr\|V9HWI3\|V9HWI3_HUMAN | sp\|Q15084\|PDIA6_HUMAN | 448 |
| HEL-S-130P | HEL-S-103 | tr\|V9HWI3\|V9HWI3_HUMAN | tr\|A8K5I0\|A8K5I0_HUMAN | 401 |
| HEL-S-130P | CANX | tr\|V9HWI3\|V9HWI3_HUMAN | sp\|P27824\|CALX_HUMAN | 447 |
| HEL-S-130P | HEL-S-89n | tr\|V9HWI3\|V9HWI3_HUMAN | tr\|V9HWB4\|V9HWB4_HUMAN | 567 |
| HEL-S-269 | HEL-S-133P | tr\|V9HVY3\|V9HVY3_HUMAN | tr\|V9HWB9\|V9HWB9_HUMAN | 567 |
| HEL-S-269 | HEL-S-162eP | tr\|V9HVY3\|V9HVY3_HUMAN | tr\|V9HVZ4\|V9HVZ4_HUMAN | 628 |
| HEL-S-269 | HEL-S-103 | tr\|V9HVY3\|V9HVY3_HUMAN | tr\|A8K5I0\|A8K5I0_HUMAN | 620 |
| HEL-S-269 | HEL-S-89n | tr\|V9HVY3\|V9HVY3_HUMAN | tr\|V9HWB4\|V9HWB4_HUMAN | 955 |
| HEL-S-269 | HEL-S-94n | tr\|V9HVY3\|V9HVY3_HUMAN | tr\|V9HW72\|V9HW72_HUMAN | 458 |
| HEL-S-269 | EEF1G | tr\|V9HVY3\|V9HVY3_HUMAN | tr\|Q53YD7\|Q53YD7_HUMAN | 634 |
| HEL-S-269 | HEL-S-70 | tr\|V9HVY3\|V9HVY3_HUMAN | tr\|V9HW80\|V9HW80_HUMAN | 440 |
| HEL-S-269 | CANX | tr\|V9HVY3\|V9HVY3_HUMAN | sp\|P27824\|CALX_HUMAN | 999 |
| HEL-S-269 | ACTN4 | tr\|V9HVY3\|V9HVY3_HUMAN | tr\|A0A0S2Z3G9\|A0A0S2Z3G9_HUMAN | 535 |
| HEL-S-269 | DLD | tr\|V9HVY3\|V9HVY3_HUMAN | tr\|A0A024R713\|A0A024R713_HUMAN | 495 |
| HEL-S-269 | TPI1 | tr\|V9HVY3\|V9HVY3_HUMAN | sp\|P60174\|TPIS_HUMAN | 409 |
| HEL-S-269 | PDIA6 | tr\|V9HVY3\|V9HVY3_HUMAN | sp\|Q15084\|PDIA6_HUMAN | 688 |
| HEL-S-269 | TRA1 | tr\|V9HVY3\|V9HVY3_HUMAN | tr\|Q5CAQ5\|Q5CAQ5_HUMAN | 921 |
| HEL-S-269 | RAB1A | tr\|V9HVY3\|V9HVY3_HUMAN | tr\|Q5U0I6\|Q5U0I6_HUMAN | 556 |
| HEL-S-269 | HEL-S-271 | tr\|V9HVY3\|V9HVY3_HUMAN | tr\|V9HW31\|V9HW31_HUMAN | 473 |
| HEL-S-269 | CCT4 | tr\|V9HVY3\|V9HVY3_HUMAN | sp\|P50991\|TCPD_HUMAN | 427 |
| HEL-S-269 | HEL-S-128m | tr\|V9HVY3\|V9HVY3_HUMAN | tr\|V9HWC7\|V9HWC7_HUMAN | 708 |
| HEL-S-269 | FLJ00064 | tr\|V9HVY3\|V9HVY3_HUMAN | tr\|B4DV28\|B4DV28_HUMAN | 425 |
| HEL-S-269 | TXNDC12 | tr\|V9HVY3\|V9HVY3_HUMAN | sp\|O95881\|TXD12_HUMAN | 420 |
| HEL-S-269 | P4HB | tr\|V9HVY3\|V9HVY3_HUMAN | tr\|A0A024R8S5\|A0A024R8S5_HUMAN | 822 |
| HEL-S-269 | HEL-S-2a | tr\|V9HVY3\|V9HVY3_HUMAN | tr\|V9HW12\|V9HW12_HUMAN | 725 |
| HEL-S-269 | HSPA9 | tr\|V9HVY3\|V9HVY3_HUMAN | tr\|Q8N1C8\|Q8N1C8_HUMAN | 601 |
| RAB1A | GDI2 | tr\|Q5U0I6\|Q5U0I6_HUMAN | tr\|B4DLV7\|B4DLV7_HUMAN | 998 |
| RAB1A | VDAC2 | tr\|Q5U0I6\|Q5U0I6_HUMAN | tr\|A0A024QZN9\|A0A024QZN9_HUMAN | 423 |
| RAB1A | HEL-S-103 | tr\|Q5U0I6\|Q5U0I6_HUMAN | tr\|A8K5I0\|A8K5I0_HUMAN | 449 |
| RAB1A | CANX | tr\|Q5U0I6\|Q5U0I6_HUMAN | sp\|P27824\|CALX_HUMAN | 556 |
| RAB1A | PTBP1 | tr\|Q5U0I6\|Q5U0I6_HUMAN | sp\|P26599\|PTBP1_HUMAN | 644 |
| RAB1A | HEL-S-89n | tr\|Q5U0I6\|Q5U0I6_HUMAN | tr\|V9HWB4\|V9HWB4_HUMAN | 507 |
| RPL3 | hCG_24487 | sp\|P39023\|RL3_HUMAN | tr\|A0A024R261\|A0A024R261_HUMAN | 999 |
| RPL3 | RPS9 | sp\|P39023\|RL3_HUMAN | tr\|A0A024R4M0\|A0A024R4M0_HUMAN | 994 |
| RPL3 | RPS3A | sp\|P39023\|RL3_HUMAN | tr\|Q6NXR8\|Q6NXR8_HUMAN | 999 |
| RPL3 | HEL-S-162eP | sp\|P39023\|RL3_HUMAN | tr\|V9HVZ4\|V9HVZ4_HUMAN | 477 |
| RPL3 | HEL-S-123m | sp\|P39023\|RL3_HUMAN | tr\|V9HW26\|V9HW26_HUMAN | 693 |
| RPL3 | RPS8 | sp\|P39023\|RL3_HUMAN | tr\|Q5JR94\|Q5JR94_HUMAN | 999 |
| RPL3 | HEL-S-103 | sp\|P39023\|RL3_HUMAN | tr\|A8K5I0\|A8K5I0_HUMAN | 437 |
| RPL3 | HEL-S-89n | sp\|P39023\|RL3_HUMAN | tr\|V9HWB4\|V9HWB4_HUMAN | 479 |
| RPL3 | RPL7 | sp\|P39023\|RL3_HUMAN | tr\|A0A024R814\|A0A024R814_HUMAN | 999 |
| RPL3 | RPL13 | sp\|P39023\|RL3_HUMAN | tr\|Q6NZ55\|Q6NZ55_HUMAN | 999 |
| RPL3 | EEF1G | sp\|P39023\|RL3_HUMAN | tr\|Q53YD7\|Q53YD7_HUMAN | 869 |
| RPL3 | RPL26 | sp\|P39023\|RL3_HUMAN | sp\|P61254\|RL26_HUMAN | 999 |
| RPL3 | RPS18 | sp\|P39023\|RL3_HUMAN | sp\|P62269\|RS18_HUMAN | 974 |
| RPL3 | RPS7 | sp\|P39023\|RL3_HUMAN | sp\|P62081\|RS7_HUMAN | 999 |
| RPL3 | RPS13 | sp\|P39023\|RL3_HUMAN | sp\|P62277\|RS13_HUMAN | 999 |
| RPL3 | RPL18 | sp\|P39023\|RL3_HUMAN | tr\|J3QQ67\|J3QQ67_HUMAN | 999 |
| RPL3 | RPS3 | sp\|P39023\|RL3_HUMAN | sp\|P23396\|RS3_HUMAN | 999 |
| RPL3 | RPS27A | sp\|P39023\|RL3_HUMAN | tr\|B2RDW1\|B2RDW1_HUMAN | 997 |
| RPL3 | TARS | sp\|P39023\|RL3_HUMAN | tr\|Q53GX7\|Q53GX7_HUMAN | 527 |
| RPL3 | HEL-S-68p | sp\|P39023\|RL3_HUMAN | tr\|V9HWF4\|V9HWF4_HUMAN | 584 |
| RPL3 | HEL-S-271 | sp\|P39023\|RL3_HUMAN | tr\|V9HW31\|V9HW31_HUMAN | 412 |
| RPL3 | CCT4 | sp\|P39023\|RL3_HUMAN | sp\|P50991\|TCPD_HUMAN | 529 |
| RPL3 | RPLP2 | sp\|P39023\|RL3_HUMAN | tr\|A0A024RCA7\|A0A024RCA7_HUMAN | 998 |
| RPL3 | SRP14 | sp\|P39023\|RL3_HUMAN | sp\|P37108\|SRP14_HUMAN | 900 |
| RPL3 | RPS6 | sp\|P39023\|RL3_HUMAN | tr\|A2A3R6\|A2A3R6_HUMAN | 999 |
| RPL3 | ATP5O | sp\|P39023\|RL3_HUMAN | sp\|P48047\|ATPO_HUMAN | 532 |
| RPL3 | RPS19 | sp\|P39023\|RL3_HUMAN | tr\|Q8WVX7\|Q8WVX7_HUMAN | 997 |
| RPL3 | HSPA9 | sp\|P39023\|RL3_HUMAN | tr\|Q8N1C8\|Q8N1C8_HUMAN | 433 |
| HEL-S-128m | DLD | tr\|V9HWC7\|V9HWC7_HUMAN | tr\|A0A024R713\|A0A024R713_HUMAN | 488 |
| HEL-S-128m | HEL-S-162eP | tr\|V9HWC7\|V9HWC7_HUMAN | tr\|V9HVZ4\|V9HVZ4_HUMAN | 516 |
| HEL-S-128m | GLUD1 | tr\|V9HWC7\|V9HWC7_HUMAN | tr\|E9KL48\|E9KL48_HUMAN | 425 |
| HEL-S-128m | PDIA6 | tr\|V9HWC7\|V9HWC7_HUMAN | sp\|Q15084\|PDIA6_HUMAN | 644 |
| HEL-S-128m | CCT4 | tr\|V9HWC7\|V9HWC7_HUMAN | sp\|P50991\|TCPD_HUMAN | 404 |
| HEL-S-128m | HEL-S-89n | tr\|V9HWC7\|V9HWC7_HUMAN | tr\|V9HWB4\|V9HWB4_HUMAN | 512 |
| HEL-S-128m | P4HB | tr\|V9HWC7\|V9HWC7_HUMAN | tr\|A0A024R8S5\|A0A024R8S5_HUMAN | 624 |
| HEL-S-128m | HEL-S-2a | tr\|V9HWC7\|V9HWC7_HUMAN | tr\|V9HW12\|V9HW12_HUMAN | 940 |
| HEL-S-128m | ERP70 | tr\|V9HWC7\|V9HWC7_HUMAN | tr\|A0A090N8Y2\|A0A090N8Y2_HUMAN | 625 |
| HEL-S-128m | HSPA9 | tr\|V9HWC7\|V9HWC7_HUMAN | tr\|Q8N1C8\|Q8N1C8_HUMAN | 516 |
| C7orf24 | HEL-S-162eP | tr\|A0A090N7V5\|A0A090N7V5_HUMAN | tr\|V9HVZ4\|V9HVZ4_HUMAN | 540 |
| ATP5O | RPS3 | sp\|P48047\|ATPO_HUMAN | sp\|P23396\|RS3_HUMAN | 690 |
| ATP5O | hCG_24487 | sp\|P48047\|ATPO_HUMAN | tr\|A0A024R261\|A0A024R261_HUMAN | 658 |
| ATP5O | RPS9 | sp\|P48047\|ATPO_HUMAN | tr\|A0A024R4M0\|A0A024R4M0_HUMAN | 550 |
| ATP5O | HEL-S-123m | sp\|P48047\|ATPO_HUMAN | tr\|V9HW26\|V9HW26_HUMAN | 999 |
| ATP5O | VDAC2 | sp\|P48047\|ATPO_HUMAN | tr\|A0A024QZN9\|A0A024QZN9_HUMAN | 475 |
| ATP5O | MDH2 | sp\|P48047\|ATPO_HUMAN | tr\|Q6FHZ0\|Q6FHZ0_HUMAN | 623 |
| ATP5O | HEL-S-271 | sp\|P48047\|ATPO_HUMAN | tr\|V9HW31\|V9HW31_HUMAN | 999 |
| ATP5O | RPL7 | sp\|P48047\|ATPO_HUMAN | tr\|A0A024R814\|A0A024R814_HUMAN | 432 |
| ATP5O | RPS18 | sp\|P48047\|ATPO_HUMAN | sp\|P62269\|RS18_HUMAN | 694 |
| ATP5O | RPL26 | sp\|P48047\|ATPO_HUMAN | sp\|P61254\|RL26_HUMAN | 625 |
| ATP5O | ATP5H | sp\|P48047\|ATPO_HUMAN | sp\|O75947\|ATP5H_HUMAN | 999 |
| MYH9 | MYL6 | tr\|A0A024R1N1\|A0A024R1N1_HUMAN | tr\|G8JLA2\|G8JLA2_HUMAN | 996 |
| MYH9 | ACTN4 | tr\|A0A024R1N1\|A0A024R1N1_HUMAN | tr\|A0A0S2Z3G9\|A0A0S2Z3G9_HUMAN | 764 |
| MYH9 | HEL70 | tr\|A0A024R1N1\|A0A024R1N1_HUMAN | tr\|V9HWC0\|V9HWC0_HUMAN | 430 |
| MYH9 | HEL-S-123m | tr\|A0A024R1N1\|A0A024R1N1_HUMAN | tr\|V9HW26\|V9HW26_HUMAN | 659 |
| MYH9 | TRA1 | tr\|A0A024R1N1\|A0A024R1N1_HUMAN | tr\|Q5CAQ5\|Q5CAQ5_HUMAN | 424 |
| MYH9 | HNRPU | tr\|A0A024R1N1\|A0A024R1N1_HUMAN | tr\|B4DLR3\|B4DLR3_HUMAN | 600 |
| MYH9 | HEL-S-89n | tr\|A0A024R1N1\|A0A024R1N1_HUMAN | tr\|V9HWB4\|V9HWB4_HUMAN | 600 |
| RPS3A | hCG_24487 | tr\|Q6NXR8\|Q6NXR8_HUMAN | tr\|A0A024R261\|A0A024R261_HUMAN | 999 |
| RPS3A | RPS9 | tr\|Q6NXR8\|Q6NXR8_HUMAN | tr\|A0A024R4M0\|A0A024R4M0_HUMAN | 999 |
| RPS3A | HEL-S-162eP | tr\|Q6NXR8\|Q6NXR8_HUMAN | tr\|V9HVZ4\|V9HVZ4_HUMAN | 556 |
| RPS3A | RPS8 | tr\|Q6NXR8\|Q6NXR8_HUMAN | tr\|Q5JR94\|Q5JR94_HUMAN | 999 |
| RPS3A | RPL7 | tr\|Q6NXR8\|Q6NXR8_HUMAN | tr\|A0A024R814\|A0A024R814_HUMAN | 999 |
| RPS3A | RPL13 | tr\|Q6NXR8\|Q6NXR8_HUMAN | tr\|Q6NZ55\|Q6NZ55_HUMAN | 999 |
| RPS3A | EEF1G | tr\|Q6NXR8\|Q6NXR8_HUMAN | tr\|Q53YD7\|Q53YD7_HUMAN | 758 |
| RPS3A | RPL26 | tr\|Q6NXR8\|Q6NXR8_HUMAN | sp\|P61254\|RL26_HUMAN | 998 |
| RPS3A | RPS18 | tr\|Q6NXR8\|Q6NXR8_HUMAN | sp\|P62269\|RS18_HUMAN | 991 |
| RPS3A | HNRPA1 | tr\|Q6NXR8\|Q6NXR8_HUMAN | tr\|A0A024RAZ7\|A0A024RAZ7_HUMAN | 662 |
| RPS3A | RPS7 | tr\|Q6NXR8\|Q6NXR8_HUMAN | sp\|P62081\|RS7_HUMAN | 999 |
| RPS3A | RPS13 | tr\|Q6NXR8\|Q6NXR8_HUMAN | sp\|P62277\|RS13_HUMAN | 999 |
| RPS3A | PSMD4 | tr\|Q6NXR8\|Q6NXR8_HUMAN | sp\|P55036\|PSMD4_HUMAN | 764 |
| RPS3A | RPL18 | tr\|Q6NXR8\|Q6NXR8_HUMAN | tr\|J3QQ67\|J3QQ67_HUMAN | 999 |
| RPS3A | RPS3 | tr\|Q6NXR8\|Q6NXR8_HUMAN | sp\|P23396\|RS3_HUMAN | 999 |
| RPS3A | RPS27A | tr\|Q6NXR8\|Q6NXR8_HUMAN | tr\|B2RDW1\|B2RDW1_HUMAN | 999 |
| RPS3A | RAD23B | tr\|Q6NXR8\|Q6NXR8_HUMAN | sp\|P54727\|RD23B_HUMAN | 508 |
| RPS3A | HEL-S-271 | tr\|Q6NXR8\|Q6NXR8_HUMAN | tr\|V9HW31\|V9HW31_HUMAN | 717 |
| RPS3A | RPLP2 | tr\|Q6NXR8\|Q6NXR8_HUMAN | tr\|A0A024RCA7\|A0A024RCA7_HUMAN | 999 |
| RPS3A | SRP14 | tr\|Q6NXR8\|Q6NXR8_HUMAN | sp\|P37108\|SRP14_HUMAN | 900 |
| RPS3A | RPS6 | tr\|Q6NXR8\|Q6NXR8_HUMAN | tr\|A2A3R6\|A2A3R6_HUMAN | 999 |
| RPS3A | RPS19 | tr\|Q6NXR8\|Q6NXR8_HUMAN | tr\|Q8WVX7\|Q8WVX7_HUMAN | 999 |
| SSB | SRP14 | tr\|B5BUB5\|B5BUB5_HUMAN | sp\|P37108\|SRP14_HUMAN | 824 |
| SSB | RANBP1 | tr\|B5BUB5\|B5BUB5_HUMAN | tr\|F6WQW2\|F6WQW2_HUMAN | 526 |
| SSB | RPS7 | tr\|B5BUB5\|B5BUB5_HUMAN | sp\|P62081\|RS7_HUMAN | 401 |
| SSB | UBA2 | tr\|B5BUB5\|B5BUB5_HUMAN | tr\|B2RDF5\|B2RDF5_HUMAN | 465 |
| SSB | RPL7 | tr\|B5BUB5\|B5BUB5_HUMAN | tr\|A0A024R814\|A0A024R814_HUMAN | 524 |
| HEL-S-103 | RPS9 | tr\|A8K5I0\|A8K5I0_HUMAN | tr\|A0A024R4M0\|A0A024R4M0_HUMAN | 447 |
| HEL-S-103 | HEL-S-162eP | tr\|A8K5I0\|A8K5I0_HUMAN | tr\|V9HVZ4\|V9HVZ4_HUMAN | 607 |
| HEL-S-103 | LMNB1 | tr\|A8K5I0\|A8K5I0_HUMAN | sp\|P20700\|LMNB1_HUMAN | 488 |
| HEL-S-103 | HEL-S-94n | tr\|A8K5I0\|A8K5I0_HUMAN | tr\|V9HW72\|V9HW72_HUMAN | 957 |
| HEL-S-103 | EEF1G | tr\|A8K5I0\|A8K5I0_HUMAN | tr\|Q53YD7\|Q53YD7_HUMAN | 460 |
| HEL-S-103 | RPS13 | tr\|A8K5I0\|A8K5I0_HUMAN | sp\|P62277\|RS13_HUMAN | 405 |
| HEL-S-103 | CANX | tr\|A8K5I0\|A8K5I0_HUMAN | sp\|P27824\|CALX_HUMAN | 667 |
| HEL-S-103 | PTBP1 | tr\|A8K5I0\|A8K5I0_HUMAN | sp\|P26599\|PTBP1_HUMAN | 494 |
| HEL-S-103 | RPS3 | tr\|A8K5I0\|A8K5I0_HUMAN | sp\|P23396\|RS3_HUMAN | 435 |
| HEL-S-103 | STUB1 | tr\|A8K5I0\|A8K5I0_HUMAN | sp\|Q9UNE7\|CHIP_HUMAN | 787 |
| HEL-S-103 | TARS | tr\|A8K5I0\|A8K5I0_HUMAN | tr\|Q53GX7\|Q53GX7_HUMAN | 406 |
| HEL-S-103 | LMNA | tr\|A8K5I0\|A8K5I0_HUMAN | sp\|P02545\|LMNA_HUMAN | 580 |
| HEL-S-103 | PDIA6 | tr\|A8K5I0\|A8K5I0_HUMAN | sp\|Q15084\|PDIA6_HUMAN | 445 |
| HEL-S-103 | TRA1 | tr\|A8K5I0\|A8K5I0_HUMAN | tr\|Q5CAQ5\|Q5CAQ5_HUMAN | 848 |
| HEL-S-103 | CCT4 | tr\|A8K5I0\|A8K5I0_HUMAN | sp\|P50991\|TCPD_HUMAN | 757 |
| HEL-S-103 | TKT | tr\|A8K5I0\|A8K5I0_HUMAN | tr\|Q53EM5\|Q53EM5_HUMAN | 420 |
| HEL-S-103 | P4HB | tr\|A8K5I0\|A8K5I0_HUMAN | tr\|A0A024R8S5\|A0A024R8S5_HUMAN | 582 |
| HEL-S-103 | NQO1 | tr\|A8K5I0\|A8K5I0_HUMAN | sp\|P15559\|NQO1_HUMAN | 576 |
| HEL-S-103 | HEL-S-2a | tr\|A8K5I0\|A8K5I0_HUMAN | tr\|V9HW12\|V9HW12_HUMAN | 401 |
| HEL-S-103 | ERP70 | tr\|A8K5I0\|A8K5I0_HUMAN | tr\|A0A090N8Y2\|A0A090N8Y2_HUMAN | 582 |
| RPL7 | hCG_24487 | tr\|A0A024R814\|A0A024R814_HUMAN | tr\|A0A024R261\|A0A024R261_HUMAN | 999 |
| RPL7 | RPS9 | tr\|A0A024R814\|A0A024R814_HUMAN | tr\|A0A024R4M0\|A0A024R4M0_HUMAN | 980 |
| RPL7 | HEL-S-162eP | tr\|A0A024R814\|A0A024R814_HUMAN | tr\|V9HVZ4\|V9HVZ4_HUMAN | 427 |
| RPL7 | HEL-S-123m | tr\|A0A024R814\|A0A024R814_HUMAN | tr\|V9HW26\|V9HW26_HUMAN | 604 |
| RPL7 | RPS8 | tr\|A0A024R814\|A0A024R814_HUMAN | tr\|Q5JR94\|Q5JR94_HUMAN | 999 |
| RPL7 | RPL13 | tr\|A0A024R814\|A0A024R814_HUMAN | tr\|Q6NZ55\|Q6NZ55_HUMAN | 999 |
| RPL7 | EEF1G | tr\|A0A024R814\|A0A024R814_HUMAN | tr\|Q53YD7\|Q53YD7_HUMAN | 628 |
| RPL7 | RPS18 | tr\|A0A024R814\|A0A024R814_HUMAN | sp\|P62269\|RS18_HUMAN | 990 |
| RPL7 | RPL26 | tr\|A0A024R814\|A0A024R814_HUMAN | sp\|P61254\|RL26_HUMAN | 999 |
| RPL7 | RPS7 | tr\|A0A024R814\|A0A024R814_HUMAN | sp\|P62081\|RS7_HUMAN | 999 |
| RPL7 | RPS13 | tr\|A0A024R814\|A0A024R814_HUMAN | sp\|P62277\|RS13_HUMAN | 999 |
| RPL7 | CANX | tr\|A0A024R814\|A0A024R814_HUMAN | sp\|P27824\|CALX_HUMAN | 590 |
| RPL7 | RPL18 | tr\|A0A024R814\|A0A024R814_HUMAN | tr\|J3QQ67\|J3QQ67_HUMAN | 999 |
| RPL7 | RPS3 | tr\|A0A024R814\|A0A024R814_HUMAN | sp\|P23396\|RS3_HUMAN | 999 |
| RPL7 | RPS27A | tr\|A0A024R814\|A0A024R814_HUMAN | tr\|B2RDW1\|B2RDW1_HUMAN | 999 |
| RPL7 | RPLP2 | tr\|A0A024R814\|A0A024R814_HUMAN | tr\|A0A024RCA7\|A0A024RCA7_HUMAN | 999 |
| RPL7 | SRP14 | tr\|A0A024R814\|A0A024R814_HUMAN | sp\|P37108\|SRP14_HUMAN | 900 |
| RPL7 | RPS6 | tr\|A0A024R814\|A0A024R814_HUMAN | tr\|A2A3R6\|A2A3R6_HUMAN | 999 |
| RPL7 | RPS19 | tr\|A0A024R814\|A0A024R814_HUMAN | tr\|Q8WVX7\|Q8WVX7_HUMAN | 999 |
| PHB2 | DHCR7 | sp\|Q99623\|PHB2_HUMAN | tr\|X5DNI9\|X5DNI9_HUMAN | 472 |
| PHB2 | HADH | sp\|Q99623\|PHB2_HUMAN | tr\|B2RB06\|B2RB06_HUMAN | 467 |
| PHB2 | HSPA9 | sp\|Q99623\|PHB2_HUMAN | tr\|Q8N1C8\|Q8N1C8_HUMAN | 634 |
| PHB2 | HEL57 | sp\|Q99623\|PHB2_HUMAN | tr\|V9HWH1\|V9HWH1_HUMAN | 585 |
| RPS18 | hCG_24487 | sp\|P62269\|RS18_HUMAN | tr\|A0A024R261\|A0A024R261_HUMAN | 990 |
| RPS18 | RPS9 | sp\|P62269\|RS18_HUMAN | tr\|A0A024R4M0\|A0A024R4M0_HUMAN | 996 |
| RPS18 | HEL-S-162eP | sp\|P62269\|RS18_HUMAN | tr\|V9HVZ4\|V9HVZ4_HUMAN | 415 |
| RPS18 | HEL-S-123m | sp\|P62269\|RS18_HUMAN | tr\|V9HW26\|V9HW26_HUMAN | 596 |
| RPS18 | RPS8 | sp\|P62269\|RS18_HUMAN | tr\|Q5JR94\|Q5JR94_HUMAN | 997 |
| RPS18 | RPL13 | sp\|P62269\|RS18_HUMAN | tr\|Q6NZ55\|Q6NZ55_HUMAN | 984 |
| RPS18 | EEF1G | sp\|P62269\|RS18_HUMAN | tr\|Q53YD7\|Q53YD7_HUMAN | 562 |
| RPS18 | RPL26 | sp\|P62269\|RS18_HUMAN | sp\|P61254\|RL26_HUMAN | 993 |
| RPS18 | RPS7 | sp\|P62269\|RS18_HUMAN | sp\|P62081\|RS7_HUMAN | 992 |
| RPS18 | RPS13 | sp\|P62269\|RS18_HUMAN | sp\|P62277\|RS13_HUMAN | 996 |
| RPS18 | RPL18 | sp\|P62269\|RS18_HUMAN | tr\|J3QQ67\|J3QQ67_HUMAN | 985 |
| RPS18 | RPS27A | sp\|P62269\|RS18_HUMAN | tr\|B2RDW1\|B2RDW1_HUMAN | 990 |
| RPS18 | RPS3 | sp\|P62269\|RS18_HUMAN | sp\|P23396\|RS3_HUMAN | 997 |
| RPS18 | CCT4 | sp\|P62269\|RS18_HUMAN | sp\|P50991\|TCPD_HUMAN | 446 |
| RPS18 | HEL-S-271 | sp\|P62269\|RS18_HUMAN | tr\|V9HW31\|V9HW31_HUMAN | 507 |
| RPS18 | RPLP2 | sp\|P62269\|RS18_HUMAN | tr\|A0A024RCA7\|A0A024RCA7_HUMAN | 980 |
| RPS18 | RPS6 | sp\|P62269\|RS18_HUMAN | tr\|A2A3R6\|A2A3R6_HUMAN | 991 |
| RPS18 | RPS19 | sp\|P62269\|RS18_HUMAN | tr\|Q8WVX7\|Q8WVX7_HUMAN | 992 |
| HNRPU | RPS3 | tr\|B4DLR3\|B4DLR3_HUMAN | sp\|P23396\|RS3_HUMAN | 424 |
| HNRPU | SRSF1 | tr\|B4DLR3\|B4DLR3_HUMAN | sp\|Q07955\|SRSF1_HUMAN | 974 |
| HNRPU | RALY | tr\|B4DLR3\|B4DLR3_HUMAN | tr\|A8K4T9\|A8K4T9_HUMAN | 661 |
| HNRPU | SNRPD2 | tr\|B4DLR3\|B4DLR3_HUMAN | sp\|P62316\|SMD2_HUMAN | 916 |
| HNRPU | VDAC2 | tr\|B4DLR3\|B4DLR3_HUMAN | tr\|A0A024QZN9\|A0A024QZN9_HUMAN | 622 |
| HNRPU | HNRNPA2B1 | tr\|B4DLR3\|B4DLR3_HUMAN | sp\|P22626\|ROA2_HUMAN | 948 |
| HNRPU | RPS8 | tr\|B4DLR3\|B4DLR3_HUMAN | tr\|Q5JR94\|Q5JR94_HUMAN | 411 |
| HNRPU | RPLP2 | tr\|B4DLR3\|B4DLR3_HUMAN | tr\|A0A024RCA7\|A0A024RCA7_HUMAN | 612 |
| HNRPU | RPS6 | tr\|B4DLR3\|B4DLR3_HUMAN | tr\|A2A3R6\|A2A3R6_HUMAN | 611 |
| HNRPU | SNRNP200 | tr\|B4DLR3\|B4DLR3_HUMAN | sp\|O75643\|U520_HUMAN | 960 |
| HNRPU | HNRPA1 | tr\|B4DLR3\|B4DLR3_HUMAN | tr\|A0A024RAZ7\|A0A024RAZ7_HUMAN | 989 |
| HNRPU | PTBP1 | tr\|B4DLR3\|B4DLR3_HUMAN | sp\|P26599\|PTBP1_HUMAN | 982 |
| HNRPA1 | DHX15 | tr\|A0A024RAZ7\|A0A024RAZ7_HUMAN | sp\|O43143\|DHX15_HUMAN | 537 |
| HNRPA1 | SRSF1 | tr\|A0A024RAZ7\|A0A024RAZ7_HUMAN | sp\|Q07955\|SRSF1_HUMAN | 988 |
| HNRPA1 | SNRPD2 | tr\|A0A024RAZ7\|A0A024RAZ7_HUMAN | sp\|P62316\|SMD2_HUMAN | 953 |
| HNRPA1 | HEL-S-162eP | tr\|A0A024RAZ7\|A0A024RAZ7_HUMAN | tr\|V9HVZ4\|V9HVZ4_HUMAN | 456 |
| HNRPA1 | RPS8 | tr\|A0A024RAZ7\|A0A024RAZ7_HUMAN | tr\|Q5JR94\|Q5JR94_HUMAN | 666 |
| HNRPA1 | LMNB1 | tr\|A0A024RAZ7\|A0A024RAZ7_HUMAN | sp\|P20700\|LMNB1_HUMAN | 449 |
| HNRPA1 | HEL-S-89n | tr\|A0A024RAZ7\|A0A024RAZ7_HUMAN | tr\|V9HWB4\|V9HWB4_HUMAN | 491 |
| HNRPA1 | RANBP1 | tr\|A0A024RAZ7\|A0A024RAZ7_HUMAN | tr\|F6WQW2\|F6WQW2_HUMAN | 547 |
| HNRPA1 | PTBP1 | tr\|A0A024RAZ7\|A0A024RAZ7_HUMAN | sp\|P26599\|PTBP1_HUMAN | 999 |
| HNRPA1 | RPS3 | tr\|A0A024RAZ7\|A0A024RAZ7_HUMAN | sp\|P23396\|RS3_HUMAN | 666 |
| HNRPA1 | HNRNPAB | tr\|A0A024RAZ7\|A0A024RAZ7_HUMAN | tr\|D6RD18\|D6RD18_HUMAN | 414 |
| HNRPA1 | HNRNPA2B1 | tr\|A0A024RAZ7\|A0A024RAZ7_HUMAN | sp\|P22626\|ROA2_HUMAN | 984 |
| HNRPA1 | RPLP2 | tr\|A0A024RAZ7\|A0A024RAZ7_HUMAN | tr\|A0A024RCA7\|A0A024RCA7_HUMAN | 707 |
| HNRPA1 | KPNB1 | tr\|A0A024RAZ7\|A0A024RAZ7_HUMAN | sp\|Q14974\|IMB1_HUMAN | 411 |
| HNRPA1 | SNRNP200 | tr\|A0A024RAZ7\|A0A024RAZ7_HUMAN | sp\|O75643\|U520_HUMAN | 958 |
| HNRPA1 | RPS6 | tr\|A0A024RAZ7\|A0A024RAZ7_HUMAN | tr\|A2A3R6\|A2A3R6_HUMAN | 705 |
| HNRPA1 | HSPA9 | tr\|A0A024RAZ7\|A0A024RAZ7_HUMAN | tr\|Q8N1C8\|Q8N1C8_HUMAN | 450 |
| CDIPT | PI4K2A | tr\|Q53HA5\|Q53HA5_HUMAN | sp\|Q9BTU6\|P4K2A_HUMAN | 903 |
| TMSB4X | ACTN4 | tr\|Q0P5N8\|Q0P5N8_HUMAN | tr\|A0A0S2Z3G9\|A0A0S2Z3G9_HUMAN | 900 |
| TARS | RPS3 | tr\|Q53GX7\|Q53GX7_HUMAN | sp\|P23396\|RS3_HUMAN | 500 |
| TARS | hCG_24487 | tr\|Q53GX7\|Q53GX7_HUMAN | tr\|A0A024R261\|A0A024R261_HUMAN | 447 |
| TARS | RPS9 | tr\|Q53GX7\|Q53GX7_HUMAN | tr\|A0A024R4M0\|A0A024R4M0_HUMAN | 460 |
| TARS | CCT4 | tr\|Q53GX7\|Q53GX7_HUMAN | sp\|P50991\|TCPD_HUMAN | 580 |
| TARS | HEL-S-89n | tr\|Q53GX7\|Q53GX7_HUMAN | tr\|V9HWB4\|V9HWB4_HUMAN | 424 |
| TARS | FARSB | tr\|Q53GX7\|Q53GX7_HUMAN | tr\|Q9BR63\|Q9BR63_HUMAN | 923 |
| TARS | AARS | tr\|Q53GX7\|Q53GX7_HUMAN | sp\|P49588\|SYAC_HUMAN | 888 |
| TARS | RPS13 | tr\|Q53GX7\|Q53GX7_HUMAN | sp\|P62277\|RS13_HUMAN | 536 |
| TARS | HSPA9 | tr\|Q53GX7\|Q53GX7_HUMAN | tr\|Q8N1C8\|Q8N1C8_HUMAN | 408 |
| HNRNPAB | SRSF1 | tr\|D6RD18\|D6RD18_HUMAN | sp\|Q07955\|SRSF1_HUMAN | 447 |
| HNRNPAB | SNRPD2 | tr\|D6RD18\|D6RD18_HUMAN | sp\|P62316\|SMD2_HUMAN | 417 |
| HNRNPAB | HNRNPA2B1 | tr\|D6RD18\|D6RD18_HUMAN | sp\|P22626\|ROA2_HUMAN | 672 |
| HNRNPAB | PTBP1 | tr\|D6RD18\|D6RD18_HUMAN | sp\|P26599\|PTBP1_HUMAN | 953 |
| HNRNPAB | HEL-S-89n | tr\|D6RD18\|D6RD18_HUMAN | tr\|V9HWB4\|V9HWB4_HUMAN | 473 |
| LMNA | LMNB1 | sp\|P02545\|LMNA_HUMAN | sp\|P20700\|LMNB1_HUMAN | 992 |
| LMNA | HEL-S-89n | sp\|P02545\|LMNA_HUMAN | tr\|V9HWB4\|V9HWB4_HUMAN | 412 |
| AARS | TRMU | sp\|P49588\|SYAC_HUMAN | sp\|O75648\|MTU1_HUMAN | 577 |
| AARS | UBE1 | sp\|P49588\|SYAC_HUMAN | tr\|A0A024R1A3\|A0A024R1A3_HUMAN | 974 |
| AARS | HEL-S-89n | sp\|P49588\|SYAC_HUMAN | tr\|V9HWB4\|V9HWB4_HUMAN | 404 |
| AARS | FARSB | sp\|P49588\|SYAC_HUMAN | tr\|Q9BR63\|Q9BR63_HUMAN | 989 |
| SMARCA5 | HIST1H4L | tr\|B7ZAX9\|B7ZAX9_HUMAN | tr\|B2R4R0\|B2R4R0_HUMAN | 684 |
| SMARCA5 | NAP1L4b | tr\|B7ZAX9\|B7ZAX9_HUMAN | tr\|B4DS05\|B4DS05_HUMAN | 411 |
| SRP14 | RPL18 | sp\|P37108\|SRP14_HUMAN | tr\|J3QQ67\|J3QQ67_HUMAN | 902 |
| SRP14 | RPS3 | sp\|P37108\|SRP14_HUMAN | sp\|P23396\|RS3_HUMAN | 900 |
| SRP14 | RPS27A | sp\|P37108\|SRP14_HUMAN | tr\|B2RDW1\|B2RDW1_HUMAN | 910 |
| SRP14 | hCG_24487 | sp\|P37108\|SRP14_HUMAN | tr\|A0A024R261\|A0A024R261_HUMAN | 908 |
| SRP14 | UBA2 | sp\|P37108\|SRP14_HUMAN | tr\|B2RDF5\|B2RDF5_HUMAN | 549 |
| SRP14 | RPS8 | sp\|P37108\|SRP14_HUMAN | tr\|Q5JR94\|Q5JR94_HUMAN | 900 |
| SRP14 | RPLP2 | sp\|P37108\|SRP14_HUMAN | tr\|A0A024RCA7\|A0A024RCA7_HUMAN | 902 |
| SRP14 | RPL13 | sp\|P37108\|SRP14_HUMAN | tr\|Q6NZ55\|Q6NZ55_HUMAN | 900 |
| SRP14 | RPL26 | sp\|P37108\|SRP14_HUMAN | sp\|P61254\|RL26_HUMAN | 900 |
| SRP14 | RPS6 | sp\|P37108\|SRP14_HUMAN | tr\|A2A3R6\|A2A3R6_HUMAN | 900 |
| SRP14 | RPS13 | sp\|P37108\|SRP14_HUMAN | sp\|P62277\|RS13_HUMAN | 926 |
| SRP14 | RPS7 | sp\|P37108\|SRP14_HUMAN | sp\|P62081\|RS7_HUMAN | 900 |
| SRP14 | RPS19 | sp\|P37108\|SRP14_HUMAN | tr\|Q8WVX7\|Q8WVX7_HUMAN | 900 |
| PFDN6 | PAFAH1B1 | tr\|Q5STK2\|Q5STK2_HUMAN | sp\|P43034\|LIS1_HUMAN | 404 |
| TKT | HEL-S-133P | tr\|Q53EM5\|Q53EM5_HUMAN | tr\|V9HWB9\|V9HWB9_HUMAN | 548 |
| TKT | HEL-S-26 | tr\|Q53EM5\|Q53EM5_HUMAN | tr\|V9HWJ2\|V9HWJ2_HUMAN | 535 |
| TKT | DLD | tr\|Q53EM5\|Q53EM5_HUMAN | tr\|A0A024R713\|A0A024R713_HUMAN | 601 |
| TKT | TPI1 | tr\|Q53EM5\|Q53EM5_HUMAN | sp\|P60174\|TPIS_HUMAN | 455 |
| TKT | HEL-S-162eP | tr\|Q53EM5\|Q53EM5_HUMAN | tr\|V9HVZ4\|V9HVZ4_HUMAN | 964 |
| TKT | MDH2 | tr\|Q53EM5\|Q53EM5_HUMAN | tr\|Q6FHZ0\|Q6FHZ0_HUMAN | 403 |
| TKT | HEL-S-68p | tr\|Q53EM5\|Q53EM5_HUMAN | tr\|V9HWF4\|V9HWF4_HUMAN | 753 |
| TKT | HEL-S-89n | tr\|Q53EM5\|Q53EM5_HUMAN | tr\|V9HWB4\|V9HWB4_HUMAN | 488 |
| TKT | HEL-S-2a | tr\|Q53EM5\|Q53EM5_HUMAN | tr\|V9HW12\|V9HW12_HUMAN | 402 |
| TKT | HSPA9 | tr\|Q53EM5\|Q53EM5_HUMAN | tr\|Q8N1C8\|Q8N1C8_HUMAN | 454 |
| P4HB | HEL-S-162eP | tr\|A0A024R8S5\|A0A024R8S5_HUMAN | tr\|V9HVZ4\|V9HVZ4_HUMAN | 569 |
| P4HB | HEL-S-123m | tr\|A0A024R8S5\|A0A024R8S5_HUMAN | tr\|V9HW26\|V9HW26_HUMAN | 784 |
| P4HB | UBA2 | tr\|A0A024R8S5\|A0A024R8S5_HUMAN | tr\|B2RDF5\|B2RDF5_HUMAN | 474 |
| P4HB | HEL-S-89n | tr\|A0A024R8S5\|A0A024R8S5_HUMAN | tr\|V9HWB4\|V9HWB4_HUMAN | 961 |
| P4HB | EEF1G | tr\|A0A024R8S5\|A0A024R8S5_HUMAN | tr\|Q53YD7\|Q53YD7_HUMAN | 578 |
| P4HB | HEL-S-70 | tr\|A0A024R8S5\|A0A024R8S5_HUMAN | tr\|V9HW80\|V9HW80_HUMAN | 800 |
| P4HB | CANX | tr\|A0A024R8S5\|A0A024R8S5_HUMAN | sp\|P27824\|CALX_HUMAN | 880 |
| P4HB | ACTN4 | tr\|A0A024R8S5\|A0A024R8S5_HUMAN | tr\|A0A0S2Z3G9\|A0A0S2Z3G9_HUMAN | 687 |
| P4HB | DLD | tr\|A0A024R8S5\|A0A024R8S5_HUMAN | tr\|A0A024R713\|A0A024R713_HUMAN | 481 |
| P4HB | PDIA6 | tr\|A0A024R8S5\|A0A024R8S5_HUMAN | sp\|Q15084\|PDIA6_HUMAN | 992 |
| P4HB | TRA1 | tr\|A0A024R8S5\|A0A024R8S5_HUMAN | tr\|Q5CAQ5\|Q5CAQ5_HUMAN | 996 |
| P4HB | CCT4 | tr\|A0A024R8S5\|A0A024R8S5_HUMAN | sp\|P50991\|TCPD_HUMAN | 899 |
| P4HB | HEL-S-271 | tr\|A0A024R8S5\|A0A024R8S5_HUMAN | tr\|V9HW31\|V9HW31_HUMAN | 785 |
| P4HB | HEL-S-2a | tr\|A0A024R8S5\|A0A024R8S5_HUMAN | tr\|V9HW12\|V9HW12_HUMAN | 665 |
| P4HB | ERP70 | tr\|A0A024R8S5\|A0A024R8S5_HUMAN | tr\|A0A090N8Y2\|A0A090N8Y2_HUMAN | 826 |
| P4HB | HSPA9 | tr\|A0A024R8S5\|A0A024R8S5_HUMAN | tr\|Q8N1C8\|Q8N1C8_HUMAN | 516 |
| SNCG | FUBP1 | tr\|Q6FHG5\|Q6FHG5_HUMAN | tr\|B4E0X8\|B4E0X8_HUMAN | 504 |
| SNCG | UBA2 | tr\|Q6FHG5\|Q6FHG5_HUMAN | tr\|B2RDF5\|B2RDF5_HUMAN | 906 |
| LMAN2 | CANX | tr\|A8K7T4\|A8K7T4_HUMAN | sp\|P27824\|CALX_HUMAN | 505 |
| TMOD3 | DHX15 | sp\|Q9NYL9\|TMOD3_HUMAN | sp\|O43143\|DHX15_HUMAN | 524 |
| PYGB | HEL-S-133P | sp\|P11216\|PYGB_HUMAN | tr\|V9HWB9\|V9HWB9_HUMAN | 416 |
| PYGB | TPI1 | sp\|P11216\|PYGB_HUMAN | sp\|P60174\|TPIS_HUMAN | 650 |
| PYGB | HEL-S-162eP | sp\|P11216\|PYGB_HUMAN | tr\|V9HVZ4\|V9HVZ4_HUMAN | 564 |
| PYGB | MDH2 | sp\|P11216\|PYGB_HUMAN | tr\|Q6FHZ0\|Q6FHZ0_HUMAN | 460 |
| PYGB | HEL-S-68p | sp\|P11216\|PYGB_HUMAN | tr\|V9HWF4\|V9HWF4_HUMAN | 589 |
| RPS6 | RPL18 | tr\|A2A3R6\|A2A3R6_HUMAN | tr\|J3QQ67\|J3QQ67_HUMAN | 999 |
| RPS6 | RPS3 | tr\|A2A3R6\|A2A3R6_HUMAN | sp\|P23396\|RS3_HUMAN | 999 |
| RPS6 | RPS27A | tr\|A2A3R6\|A2A3R6_HUMAN | tr\|B2RDW1\|B2RDW1_HUMAN | 999 |
| RPS6 | hCG_24487 | tr\|A2A3R6\|A2A3R6_HUMAN | tr\|A0A024R261\|A0A024R261_HUMAN | 999 |
| RPS6 | RPS9 | tr\|A2A3R6\|A2A3R6_HUMAN | tr\|A0A024R4M0\|A0A024R4M0_HUMAN | 999 |
| RPS6 | HEL-S-162eP | tr\|A2A3R6\|A2A3R6_HUMAN | tr\|V9HVZ4\|V9HVZ4_HUMAN | 577 |
| RPS6 | RPS8 | tr\|A2A3R6\|A2A3R6_HUMAN | tr\|Q5JR94\|Q5JR94_HUMAN | 999 |
| RPS6 | RAD23B | tr\|A2A3R6\|A2A3R6_HUMAN | sp\|P54727\|RD23B_HUMAN | 453 |
| RPS6 | RPLP2 | tr\|A2A3R6\|A2A3R6_HUMAN | tr\|A0A024RCA7\|A0A024RCA7_HUMAN | 997 |
| RPS6 | NAP1L4b | tr\|A2A3R6\|A2A3R6_HUMAN | tr\|B4DS05\|B4DS05_HUMAN | 498 |
| RPS6 | EEF1G | tr\|A2A3R6\|A2A3R6_HUMAN | tr\|Q53YD7\|Q53YD7_HUMAN | 759 |
| RPS6 | RPL13 | tr\|A2A3R6\|A2A3R6_HUMAN | tr\|Q6NZ55\|Q6NZ55_HUMAN | 999 |
| RPS6 | RPL26 | tr\|A2A3R6\|A2A3R6_HUMAN | sp\|P61254\|RL26_HUMAN | 998 |
| RPS6 | RPS7 | tr\|A2A3R6\|A2A3R6_HUMAN | sp\|P62081\|RS7_HUMAN | 999 |
| RPS6 | RPS13 | tr\|A2A3R6\|A2A3R6_HUMAN | sp\|P62277\|RS13_HUMAN | 999 |
| RPS6 | RPS19 | tr\|A2A3R6\|A2A3R6_HUMAN | tr\|Q8WVX7\|Q8WVX7_HUMAN | 999 |
| HEL-S-2a | HEL-S-133P | tr\|V9HW12\|V9HW12_HUMAN | tr\|V9HWB9\|V9HWB9_HUMAN | 400 |
| HEL-S-2a | DLD | tr\|V9HW12\|V9HW12_HUMAN | tr\|A0A024R713\|A0A024R713_HUMAN | 541 |
| HEL-S-2a | TPI1 | tr\|V9HW12\|V9HW12_HUMAN | sp\|P60174\|TPIS_HUMAN | 409 |
| HEL-S-2a | HEL-S-162eP | tr\|V9HW12\|V9HW12_HUMAN | tr\|V9HVZ4\|V9HVZ4_HUMAN | 692 |
| HEL-S-2a | PDIA6 | tr\|V9HW12\|V9HW12_HUMAN | sp\|Q15084\|PDIA6_HUMAN | 645 |
| HEL-S-2a | TRA1 | tr\|V9HW12\|V9HW12_HUMAN | tr\|Q5CAQ5\|Q5CAQ5_HUMAN | 435 |
| HEL-S-2a | HEL-S-89n | tr\|V9HW12\|V9HW12_HUMAN | tr\|V9HWB4\|V9HWB4_HUMAN | 581 |
| HEL-S-2a | EEF1G | tr\|V9HW12\|V9HW12_HUMAN | tr\|Q53YD7\|Q53YD7_HUMAN | 556 |
| HEL-S-2a | ERP70 | tr\|V9HW12\|V9HW12_HUMAN | tr\|A0A090N8Y2\|A0A090N8Y2_HUMAN | 636 |
| HEL-S-2a | HSPA9 | tr\|V9HW12\|V9HW12_HUMAN | tr\|Q8N1C8\|Q8N1C8_HUMAN | 505 |
| ERP70 | ACTN4 | tr\|A0A090N8Y2\|A0A090N8Y2_HUMAN | tr\|A0A0S2Z3G9\|A0A0S2Z3G9_HUMAN | 528 |
| ERP70 | PAFAH1B1 | tr\|A0A090N8Y2\|A0A090N8Y2_HUMAN | sp\|P43034\|LIS1_HUMAN | 403 |
| ERP70 | DLD | tr\|A0A090N8Y2\|A0A090N8Y2_HUMAN | tr\|A0A024R713\|A0A024R713_HUMAN | 443 |
| ERP70 | HEL-S-162eP | tr\|A0A090N8Y2\|A0A090N8Y2_HUMAN | tr\|V9HVZ4\|V9HVZ4_HUMAN | 532 |
| ERP70 | PDIA6 | tr\|A0A090N8Y2\|A0A090N8Y2_HUMAN | sp\|Q15084\|PDIA6_HUMAN | 663 |
| ERP70 | TRA1 | tr\|A0A090N8Y2\|A0A090N8Y2_HUMAN | tr\|Q5CAQ5\|Q5CAQ5_HUMAN | 993 |
| ERP70 | CCT4 | tr\|A0A090N8Y2\|A0A090N8Y2_HUMAN | sp\|P50991\|TCPD_HUMAN | 460 |
| ERP70 | HEL-S-89n | tr\|A0A090N8Y2\|A0A090N8Y2_HUMAN | tr\|V9HWB4\|V9HWB4_HUMAN | 949 |
| ERP70 | EEF1G | tr\|A0A090N8Y2\|A0A090N8Y2_HUMAN | tr\|Q53YD7\|Q53YD7_HUMAN | 572 |
| ERP70 | HEL70 | tr\|A0A090N8Y2\|A0A090N8Y2_HUMAN | tr\|V9HWC0\|V9HWC0_HUMAN | 503 |
| ERP70 | CANX | tr\|A0A090N8Y2\|A0A090N8Y2_HUMAN | sp\|P27824\|CALX_HUMAN | 872 |
| ERP70 | HSPA9 | tr\|A0A090N8Y2\|A0A090N8Y2_HUMAN | tr\|Q8N1C8\|Q8N1C8_HUMAN | 491 |
| HEL-S-133P | HEL-S-26 | tr\|V9HWB9\|V9HWB9_HUMAN | tr\|V9HWJ2\|V9HWJ2_HUMAN | 893 |
| HEL-S-133P | DLD | tr\|V9HWB9\|V9HWB9_HUMAN | tr\|A0A024R713\|A0A024R713_HUMAN | 747 |
| HEL-S-133P | TPI1 | tr\|V9HWB9\|V9HWB9_HUMAN | sp\|P60174\|TPIS_HUMAN | 647 |
| HEL-S-133P | HADHA | tr\|V9HWB9\|V9HWB9_HUMAN | tr\|E9KL44\|E9KL44_HUMAN | 481 |
| HEL-S-133P | HEL-S-162eP | tr\|V9HWB9\|V9HWB9_HUMAN | tr\|V9HVZ4\|V9HVZ4_HUMAN | 751 |
| HEL-S-133P | HEL-S-123m | tr\|V9HWB9\|V9HWB9_HUMAN | tr\|V9HW26\|V9HW26_HUMAN | 463 |
| HEL-S-133P | GLUD1 | tr\|V9HWB9\|V9HWB9_HUMAN | tr\|E9KL48\|E9KL48_HUMAN | 579 |
| HEL-S-133P | HEL-S-68p | tr\|V9HWB9\|V9HWB9_HUMAN | tr\|V9HWF4\|V9HWF4_HUMAN | 668 |
| HEL-S-133P | HEL-S-271 | tr\|V9HWB9\|V9HWB9_HUMAN | tr\|V9HW31\|V9HW31_HUMAN | 513 |
| HEL-S-133P | CCT4 | tr\|V9HWB9\|V9HWB9_HUMAN | sp\|P50991\|TCPD_HUMAN | 527 |
| HEL-S-133P | HEL-S-89n | tr\|V9HWB9\|V9HWB9_HUMAN | tr\|V9HWB4\|V9HWB4_HUMAN | 476 |
| HEL-S-133P | FLJ00064 | tr\|V9HWB9\|V9HWB9_HUMAN | tr\|B4DV28\|B4DV28_HUMAN | 613 |
| HEL-S-133P | HSPA9 | tr\|V9HWB9\|V9HWB9_HUMAN | tr\|Q8N1C8\|Q8N1C8_HUMAN | 421 |
| RPS9 | hCG_24487 | tr\|A0A024R4M0\|A0A024R4M0_HUMAN | tr\|A0A024R261\|A0A024R261_HUMAN | 982 |
| RPS9 | HEL-S-162eP | tr\|A0A024R4M0\|A0A024R4M0_HUMAN | tr\|V9HVZ4\|V9HVZ4_HUMAN | 446 |
| RPS9 | HEL-S-123m | tr\|A0A024R4M0\|A0A024R4M0_HUMAN | tr\|V9HW26\|V9HW26_HUMAN | 607 |
| RPS9 | RPS8 | tr\|A0A024R4M0\|A0A024R4M0_HUMAN | tr\|Q5JR94\|Q5JR94_HUMAN | 999 |
| RPS9 | HEL-S-89n | tr\|A0A024R4M0\|A0A024R4M0_HUMAN | tr\|V9HWB4\|V9HWB4_HUMAN | 450 |
| RPS9 | EEF1G | tr\|A0A024R4M0\|A0A024R4M0_HUMAN | tr\|Q53YD7\|Q53YD7_HUMAN | 872 |
| RPS9 | RPL13 | tr\|A0A024R4M0\|A0A024R4M0_HUMAN | tr\|Q6NZ55\|Q6NZ55_HUMAN | 975 |
| RPS9 | RPL26 | tr\|A0A024R4M0\|A0A024R4M0_HUMAN | sp\|P61254\|RL26_HUMAN | 988 |
| RPS9 | RPS7 | tr\|A0A024R4M0\|A0A024R4M0_HUMAN | sp\|P62081\|RS7_HUMAN | 999 |
| RPS9 | RPS13 | tr\|A0A024R4M0\|A0A024R4M0_HUMAN | sp\|P62277\|RS13_HUMAN | 999 |
| RPS9 | RPL18 | tr\|A0A024R4M0\|A0A024R4M0_HUMAN | tr\|J3QQ67\|J3QQ67_HUMAN | 992 |
| RPS9 | RPS27A | tr\|A0A024R4M0\|A0A024R4M0_HUMAN | tr\|B2RDW1\|B2RDW1_HUMAN | 901 |
| RPS9 | RPS3 | tr\|A0A024R4M0\|A0A024R4M0_HUMAN | sp\|P23396\|RS3_HUMAN | 999 |
| RPS9 | CCT4 | tr\|A0A024R4M0\|A0A024R4M0_HUMAN | sp\|P50991\|TCPD_HUMAN | 412 |
| RPS9 | HEL-S-271 | tr\|A0A024R4M0\|A0A024R4M0_HUMAN | tr\|V9HW31\|V9HW31_HUMAN | 529 |
| RPS9 | RPLP2 | tr\|A0A024R4M0\|A0A024R4M0_HUMAN | tr\|A0A024RCA7\|A0A024RCA7_HUMAN | 961 |
| RPS9 | RPS19 | tr\|A0A024R4M0\|A0A024R4M0_HUMAN | tr\|Q8WVX7\|Q8WVX7_HUMAN | 999 |
| RPS9 | HSPA9 | tr\|A0A024R4M0\|A0A024R4M0_HUMAN | tr\|Q8N1C8\|Q8N1C8_HUMAN | 429 |
| HSD17B4 | HADHA | tr\|B2R659\|B2R659_HUMAN | tr\|E9KL44\|E9KL44_HUMAN | 796 |
| HSD17B4 | HADH | tr\|B2R659\|B2R659_HUMAN | tr\|B2RB06\|B2RB06_HUMAN | 705 |
| HADHA | HEL70 | tr\|E9KL44\|E9KL44_HUMAN | tr\|V9HWC0\|V9HWC0_HUMAN | 644 |
| HADHA | DLD | tr\|E9KL44\|E9KL44_HUMAN | tr\|A0A024R713\|A0A024R713_HUMAN | 453 |
| HADHA | MDH2 | tr\|E9KL44\|E9KL44_HUMAN | tr\|Q6FHZ0\|Q6FHZ0_HUMAN | 540 |
| HADHA | HADH | tr\|E9KL44\|E9KL44_HUMAN | tr\|B2RB06\|B2RB06_HUMAN | 960 |
| HADHA | HSPA9 | tr\|E9KL44\|E9KL44_HUMAN | tr\|Q8N1C8\|Q8N1C8_HUMAN | 403 |
| HEL-S-162eP | SRSF1 | tr\|V9HVZ4\|V9HVZ4_HUMAN | sp\|Q07955\|SRSF1_HUMAN | 414 |
| HEL-S-162eP | HEL-S-26 | tr\|V9HVZ4\|V9HVZ4_HUMAN | tr\|V9HWJ2\|V9HWJ2_HUMAN | 624 |
| HEL-S-162eP | HADHA | tr\|V9HVZ4\|V9HVZ4_HUMAN | tr\|E9KL44\|E9KL44_HUMAN | 459 |
| HEL-S-162eP | GLUD1 | tr\|V9HVZ4\|V9HVZ4_HUMAN | tr\|E9KL48\|E9KL48_HUMAN | 506 |
| HEL-S-162eP | VDAC2 | tr\|V9HVZ4\|V9HVZ4_HUMAN | tr\|A0A024QZN9\|A0A024QZN9_HUMAN | 428 |
| HEL-S-162eP | LMNB1 | tr\|V9HVZ4\|V9HVZ4_HUMAN | sp\|P20700\|LMNB1_HUMAN | 516 |
| HEL-S-162eP | HEL-S-89n | tr\|V9HVZ4\|V9HVZ4_HUMAN | tr\|V9HWB4\|V9HWB4_HUMAN | 823 |
| HEL-S-162eP | HEL-S-94n | tr\|V9HVZ4\|V9HVZ4_HUMAN | tr\|V9HW72\|V9HW72_HUMAN | 769 |
| HEL-S-162eP | RPS13 | tr\|V9HVZ4\|V9HVZ4_HUMAN | sp\|P62277\|RS13_HUMAN | 431 |
| HEL-S-162eP | PSMD4 | tr\|V9HVZ4\|V9HVZ4_HUMAN | sp\|P55036\|PSMD4_HUMAN | 431 |
| HEL-S-162eP | CANX | tr\|V9HVZ4\|V9HVZ4_HUMAN | sp\|P27824\|CALX_HUMAN | 599 |
| HEL-S-162eP | PTBP1 | tr\|V9HVZ4\|V9HVZ4_HUMAN | sp\|P26599\|PTBP1_HUMAN | 416 |
| HEL-S-162eP | RPS3 | tr\|V9HVZ4\|V9HVZ4_HUMAN | sp\|P23396\|RS3_HUMAN | 646 |
| HEL-S-162eP | EZR | tr\|V9HVZ4\|V9HVZ4_HUMAN | tr\|E7EQR4\|E7EQR4_HUMAN | 453 |
| HEL-S-162eP | STUB1 | tr\|V9HVZ4\|V9HVZ4_HUMAN | sp\|Q9UNE7\|CHIP_HUMAN | 531 |
| HEL-S-162eP | TPI1 | tr\|V9HVZ4\|V9HVZ4_HUMAN | sp\|P60174\|TPIS_HUMAN | 913 |
| HEL-S-162eP | DLD | tr\|V9HVZ4\|V9HVZ4_HUMAN | tr\|A0A024R713\|A0A024R713_HUMAN | 634 |
| HEL-S-162eP | TRA1 | tr\|V9HVZ4\|V9HVZ4_HUMAN | tr\|Q5CAQ5\|Q5CAQ5_HUMAN | 650 |
| HEL-S-162eP | PDIA6 | tr\|V9HVZ4\|V9HVZ4_HUMAN | sp\|Q15084\|PDIA6_HUMAN | 430 |
| HEL-S-162eP | MDH2 | tr\|V9HVZ4\|V9HVZ4_HUMAN | tr\|Q6FHZ0\|Q6FHZ0_HUMAN | 537 |
| HEL-S-162eP | HEL-S-68p | tr\|V9HVZ4\|V9HVZ4_HUMAN | tr\|V9HWF4\|V9HWF4_HUMAN | 988 |
| HEL-S-162eP | HEL-S-271 | tr\|V9HVZ4\|V9HVZ4_HUMAN | tr\|V9HW31\|V9HW31_HUMAN | 556 |
| HEL-S-162eP | CCT4 | tr\|V9HVZ4\|V9HVZ4_HUMAN | sp\|P50991\|TCPD_HUMAN | 478 |
| HEL-S-162eP | HEL70 | tr\|V9HVZ4\|V9HVZ4_HUMAN | tr\|V9HWC0\|V9HWC0_HUMAN | 407 |
| HEL-S-162eP | HSPA9 | tr\|V9HVZ4\|V9HVZ4_HUMAN | tr\|Q8N1C8\|Q8N1C8_HUMAN | 732 |
| DPP4 | DPP3 | sp\|P27487\|DPP4_HUMAN | sp\|Q9NY33\|DPP3_HUMAN | 448 |
| PRIC295 | SRSF1 | tr\|E1NZA1\|E1NZA1_HUMAN | sp\|Q07955\|SRSF1_HUMAN | 644 |
| PRIC295 | EEF1G | tr\|E1NZA1\|E1NZA1_HUMAN | tr\|Q53YD7\|Q53YD7_HUMAN | 772 |
| PRIC295 | RANBP1 | tr\|E1NZA1\|E1NZA1_HUMAN | tr\|F6WQW2\|F6WQW2_HUMAN | 405 |
| PRIC295 | CCT4 | tr\|E1NZA1\|E1NZA1_HUMAN | sp\|P50991\|TCPD_HUMAN | 877 |
| EEF1G | hCG_24487 | tr\|Q53YD7\|Q53YD7_HUMAN | tr\|A0A024R261\|A0A024R261_HUMAN | 750 |
| EEF1G | RPS8 | tr\|Q53YD7\|Q53YD7_HUMAN | tr\|Q5JR94\|Q5JR94_HUMAN | 919 |
| EEF1G | HEL-S-89n | tr\|Q53YD7\|Q53YD7_HUMAN | tr\|V9HWB4\|V9HWB4_HUMAN | 500 |
| EEF1G | RPL13 | tr\|Q53YD7\|Q53YD7_HUMAN | tr\|Q6NZ55\|Q6NZ55_HUMAN | 938 |
| EEF1G | RPL26 | tr\|Q53YD7\|Q53YD7_HUMAN | sp\|P61254\|RL26_HUMAN | 679 |
| EEF1G | RPS7 | tr\|Q53YD7\|Q53YD7_HUMAN | sp\|P62081\|RS7_HUMAN | 807 |
| EEF1G | RPS13 | tr\|Q53YD7\|Q53YD7_HUMAN | sp\|P62277\|RS13_HUMAN | 783 |
| EEF1G | RPL18 | tr\|Q53YD7\|Q53YD7_HUMAN | tr\|J3QQ67\|J3QQ67_HUMAN | 773 |
| EEF1G | COPB2 | tr\|Q53YD7\|Q53YD7_HUMAN | sp\|P35606\|COPB2_HUMAN | 472 |
| EEF1G | RPS27A | tr\|Q53YD7\|Q53YD7_HUMAN | tr\|B2RDW1\|B2RDW1_HUMAN | 470 |
| EEF1G | RPS3 | tr\|Q53YD7\|Q53YD7_HUMAN | sp\|P23396\|RS3_HUMAN | 959 |
| EEF1G | TRA1 | tr\|Q53YD7\|Q53YD7_HUMAN | tr\|Q5CAQ5\|Q5CAQ5_HUMAN | 483 |
| EEF1G | HEL-S-68p | tr\|Q53YD7\|Q53YD7_HUMAN | tr\|V9HWF4\|V9HWF4_HUMAN | 496 |
| EEF1G | CCT4 | tr\|Q53YD7\|Q53YD7_HUMAN | sp\|P50991\|TCPD_HUMAN | 483 |
| EEF1G | RPLP2 | tr\|Q53YD7\|Q53YD7_HUMAN | tr\|A0A024RCA7\|A0A024RCA7_HUMAN | 879 |
| EEF1G | FARSB | tr\|Q53YD7\|Q53YD7_HUMAN | tr\|Q9BR63\|Q9BR63_HUMAN | 441 |
| EEF1G | RPS19 | tr\|Q53YD7\|Q53YD7_HUMAN | tr\|Q8WVX7\|Q8WVX7_HUMAN | 784 |
| EEF1G | HSPA9 | tr\|Q53YD7\|Q53YD7_HUMAN | tr\|Q8N1C8\|Q8N1C8_HUMAN | 504 |
| RPL26 | RPL18 | sp\|P61254\|RL26_HUMAN | tr\|J3QQ67\|J3QQ67_HUMAN | 999 |
| RPL26 | RPS3 | sp\|P61254\|RL26_HUMAN | sp\|P23396\|RS3_HUMAN | 999 |
| RPL26 | RPS27A | sp\|P61254\|RL26_HUMAN | tr\|B2RDW1\|B2RDW1_HUMAN | 999 |
| RPL26 | hCG_24487 | sp\|P61254\|RL26_HUMAN | tr\|A0A024R261\|A0A024R261_HUMAN | 999 |
| RPL26 | HEL-S-123m | sp\|P61254\|RL26_HUMAN | tr\|V9HW26\|V9HW26_HUMAN | 484 |
| RPL26 | RPS8 | sp\|P61254\|RL26_HUMAN | tr\|Q5JR94\|Q5JR94_HUMAN | 998 |
| RPL26 | RAD23B | sp\|P61254\|RL26_HUMAN | sp\|P54727\|RD23B_HUMAN | 400 |
| RPL26 | RPLP2 | sp\|P61254\|RL26_HUMAN | tr\|A0A024RCA7\|A0A024RCA7_HUMAN | 999 |
| RPL26 | RPL13 | sp\|P61254\|RL26_HUMAN | tr\|Q6NZ55\|Q6NZ55_HUMAN | 999 |
| RPL26 | RPS13 | sp\|P61254\|RL26_HUMAN | sp\|P62277\|RS13_HUMAN | 998 |
| RPL26 | RPS7 | sp\|P61254\|RL26_HUMAN | sp\|P62081\|RS7_HUMAN | 998 |
| RPL26 | RPS19 | sp\|P61254\|RL26_HUMAN | tr\|Q8WVX7\|Q8WVX7_HUMAN | 999 |
| RPS13 | RPL18 | sp\|P62277\|RS13_HUMAN | tr\|J3QQ67\|J3QQ67_HUMAN | 999 |
| RPS13 | DHX15 | sp\|P62277\|RS13_HUMAN | sp\|O43143\|DHX15_HUMAN | 559 |
| RPS13 | RPS3 | sp\|P62277\|RS13_HUMAN | sp\|P23396\|RS3_HUMAN | 999 |
| RPS13 | RPS27A | sp\|P62277\|RS13_HUMAN | tr\|B2RDW1\|B2RDW1_HUMAN | 995 |
| RPS13 | hCG_24487 | sp\|P62277\|RS13_HUMAN | tr\|A0A024R261\|A0A024R261_HUMAN | 999 |
| RPS13 | TPI1 | sp\|P62277\|RS13_HUMAN | sp\|P60174\|TPIS_HUMAN | 625 |
| RPS13 | RPS8 | sp\|P62277\|RS13_HUMAN | tr\|Q5JR94\|Q5JR94_HUMAN | 999 |
| RPS13 | CCT4 | sp\|P62277\|RS13_HUMAN | sp\|P50991\|TCPD_HUMAN | 405 |
| RPS13 | RPLP2 | sp\|P62277\|RS13_HUMAN | tr\|A0A024RCA7\|A0A024RCA7_HUMAN | 999 |
| RPS13 | HEL-S-89n | sp\|P62277\|RS13_HUMAN | tr\|V9HWB4\|V9HWB4_HUMAN | 401 |
| RPS13 | RPL13 | sp\|P62277\|RS13_HUMAN | tr\|Q6NZ55\|Q6NZ55_HUMAN | 999 |
| RPS13 | RPS7 | sp\|P62277\|RS13_HUMAN | sp\|P62081\|RS7_HUMAN | 999 |
| RPS13 | RPS19 | sp\|P62277\|RS13_HUMAN | tr\|Q8WVX7\|Q8WVX7_HUMAN | 999 |
| RPS13 | HSPA9 | sp\|P62277\|RS13_HUMAN | tr\|Q8N1C8\|Q8N1C8_HUMAN | 447 |
| ARCN1 | COPB2 | tr\|B0YIW6\|B0YIW6_HUMAN | sp\|P35606\|COPB2_HUMAN | 999 |
| ACTN4 | EZR | tr\|A0A0S2Z3G9\|A0A0S2Z3G9_HUMAN | tr\|E7EQR4\|E7EQR4_HUMAN | 947 |
| ACTN4 | HEL70 | tr\|A0A0S2Z3G9\|A0A0S2Z3G9_HUMAN | tr\|V9HWC0\|V9HWC0_HUMAN | 429 |
| ACTN4 | HEL-S-70 | tr\|A0A0S2Z3G9\|A0A0S2Z3G9_HUMAN | tr\|V9HW80\|V9HW80_HUMAN | 503 |
| EZR | HEL70 | tr\|E7EQR4\|E7EQR4_HUMAN | tr\|V9HWC0\|V9HWC0_HUMAN | 936 |
| EZR | DRIP4 | tr\|E7EQR4\|E7EQR4_HUMAN | tr\|Q4W4Y1\|Q4W4Y1_HUMAN | 486 |
| EZR | UBE1 | tr\|E7EQR4\|E7EQR4_HUMAN | tr\|A0A024R1A3\|A0A024R1A3_HUMAN | 500 |
| RALY | SNRNP200 | tr\|A8K4T9\|A8K4T9_HUMAN | sp\|O75643\|U520_HUMAN | 407 |
| STRAP | SNRPD2 | sp\|Q9Y3F4\|STRAP_HUMAN | sp\|P62316\|SMD2_HUMAN | 964 |
| STRAP | PTBP1 | sp\|Q9Y3F4\|STRAP_HUMAN | sp\|P26599\|PTBP1_HUMAN | 840 |
| TRA1 | RPS27A | tr\|Q5CAQ5\|Q5CAQ5_HUMAN | tr\|B2RDW1\|B2RDW1_HUMAN | 417 |
| TRA1 | HEL-S-123m | tr\|Q5CAQ5\|Q5CAQ5_HUMAN | tr\|V9HW26\|V9HW26_HUMAN | 489 |
| TRA1 | PDIA6 | tr\|Q5CAQ5\|Q5CAQ5_HUMAN | sp\|Q15084\|PDIA6_HUMAN | 989 |
| TRA1 | HEL-S-271 | tr\|Q5CAQ5\|Q5CAQ5_HUMAN | tr\|V9HW31\|V9HW31_HUMAN | 676 |
| TRA1 | CCT4 | tr\|Q5CAQ5\|Q5CAQ5_HUMAN | sp\|P50991\|TCPD_HUMAN | 793 |
| TRA1 | HEL-S-89n | tr\|Q5CAQ5\|Q5CAQ5_HUMAN | tr\|V9HWB4\|V9HWB4_HUMAN | 999 |
| TRA1 | HEL-S-94n | tr\|Q5CAQ5\|Q5CAQ5_HUMAN | tr\|V9HW72\|V9HW72_HUMAN | 985 |
| TRA1 | HEL-S-70 | tr\|Q5CAQ5\|Q5CAQ5_HUMAN | tr\|V9HW80\|V9HW80_HUMAN | 559 |
| TRA1 | PSMD4 | tr\|Q5CAQ5\|Q5CAQ5_HUMAN | sp\|P55036\|PSMD4_HUMAN | 508 |
| TRA1 | CANX | tr\|Q5CAQ5\|Q5CAQ5_HUMAN | sp\|P27824\|CALX_HUMAN | 968 |
| TRA1 | HSPA9 | tr\|Q5CAQ5\|Q5CAQ5_HUMAN | tr\|Q8N1C8\|Q8N1C8_HUMAN | 985 |
| HNRNPA2B1 | RPS27A | sp\|P22626\|ROA2_HUMAN | tr\|B2RDW1\|B2RDW1_HUMAN | 660 |
| HNRNPA2B1 | SRSF1 | sp\|P22626\|ROA2_HUMAN | sp\|Q07955\|SRSF1_HUMAN | 960 |
| HNRNPA2B1 | SNRPD2 | sp\|P22626\|ROA2_HUMAN | sp\|P62316\|SMD2_HUMAN | 932 |
| HNRNPA2B1 | HEL-S-271 | sp\|P22626\|ROA2_HUMAN | tr\|V9HW31\|V9HW31_HUMAN | 737 |
| HNRNPA2B1 | HEL-S-89n | sp\|P22626\|ROA2_HUMAN | tr\|V9HWB4\|V9HWB4_HUMAN | 539 |
| HNRNPA2B1 | SNRNP200 | sp\|P22626\|ROA2_HUMAN | sp\|O75643\|U520_HUMAN | 939 |
| HNRNPA2B1 | RPS7 | sp\|P22626\|ROA2_HUMAN | sp\|P62081\|RS7_HUMAN | 608 |
| HNRNPA2B1 | PTBP1 | sp\|P22626\|ROA2_HUMAN | sp\|P26599\|PTBP1_HUMAN | 997 |
| HNRNPA2B1 | RPS19 | sp\|P22626\|ROA2_HUMAN | tr\|Q8WVX7\|Q8WVX7_HUMAN | 632 |
| PDIA6 | DLD | sp\|Q15084\|PDIA6_HUMAN | tr\|A0A024R713\|A0A024R713_HUMAN | 477 |
| PDIA6 | HEL-S-70 | sp\|Q15084\|PDIA6_HUMAN | tr\|V9HW80\|V9HW80_HUMAN | 513 |
| PDIA6 | HEL-S-271 | sp\|Q15084\|PDIA6_HUMAN | tr\|V9HW31\|V9HW31_HUMAN | 445 |
| PDIA6 | CANX | sp\|Q15084\|PDIA6_HUMAN | sp\|P27824\|CALX_HUMAN | 644 |
| PDIA6 | CCT4 | sp\|Q15084\|PDIA6_HUMAN | sp\|P50991\|TCPD_HUMAN | 412 |
| PDIA6 | HSPA9 | sp\|Q15084\|PDIA6_HUMAN | tr\|Q8N1C8\|Q8N1C8_HUMAN | 507 |
| PDIA6 | HEL-S-89n | sp\|Q15084\|PDIA6_HUMAN | tr\|V9HWB4\|V9HWB4_HUMAN | 933 |
| RAD23B | RPL18 | sp\|P54727\|RD23B_HUMAN | tr\|J3QQ67\|J3QQ67_HUMAN | 454 |
| RAD23B | RPS3 | sp\|P54727\|RD23B_HUMAN | sp\|P23396\|RS3_HUMAN | 525 |
| RAD23B | RPS27A | sp\|P54727\|RD23B_HUMAN | tr\|B2RDW1\|B2RDW1_HUMAN | 521 |
| RAD23B | STUB1 | sp\|P54727\|RD23B_HUMAN | sp\|Q9UNE7\|CHIP_HUMAN | 729 |
| RAD23B | PSMC3 | sp\|P54727\|RD23B_HUMAN | tr\|A0A140VK42\|A0A140VK42_HUMAN | 913 |
| RAD23B | UBA2 | sp\|P54727\|RD23B_HUMAN | tr\|B2RDF5\|B2RDF5_HUMAN | 897 |
| RAD23B | UBE1 | sp\|P54727\|RD23B_HUMAN | tr\|A0A024R1A3\|A0A024R1A3_HUMAN | 692 |
| RAD23B | RPL13 | sp\|P54727\|RD23B_HUMAN | tr\|Q6NZ55\|Q6NZ55_HUMAN | 450 |
| RAD23B | HEL-S-70 | sp\|P54727\|RD23B_HUMAN | tr\|V9HW80\|V9HW80_HUMAN | 891 |
| RAD23B | RPS7 | sp\|P54727\|RD23B_HUMAN | sp\|P62081\|RS7_HUMAN | 457 |
| RAD23B | PSMD4 | sp\|P54727\|RD23B_HUMAN | sp\|P55036\|PSMD4_HUMAN | 999 |
| RAD23B | RPS19 | sp\|P54727\|RD23B_HUMAN | tr\|Q8WVX7\|Q8WVX7_HUMAN | 453 |
| HEL-S-68p | TPI1 | tr\|V9HWF4\|V9HWF4_HUMAN | sp\|P60174\|TPIS_HUMAN | 997 |
| HEL-S-68p | DLD | tr\|V9HWF4\|V9HWF4_HUMAN | tr\|A0A024R713\|A0A024R713_HUMAN | 498 |
| HEL-S-68p | GLUD1 | tr\|V9HWF4\|V9HWF4_HUMAN | tr\|E9KL48\|E9KL48_HUMAN | 435 |
| HEL-S-68p | MDH2 | tr\|V9HWF4\|V9HWF4_HUMAN | tr\|Q6FHZ0\|Q6FHZ0_HUMAN | 520 |
| HEL-S-68p | HSPA9 | tr\|V9HWF4\|V9HWF4_HUMAN | tr\|Q8N1C8\|Q8N1C8_HUMAN | 459 |
| HEL-S-68p | HEL-S-89n | tr\|V9HWF4\|V9HWF4_HUMAN | tr\|V9HWB4\|V9HWB4_HUMAN | 417 |
| DYNC1H1 | PAFAH1B1 | sp\|Q14204\|DYHC1_HUMAN | sp\|P43034\|LIS1_HUMAN | 999 |
| CCT4 | RPS3 | sp\|P50991\|TCPD_HUMAN | sp\|P23396\|RS3_HUMAN | 451 |
| CCT4 | hCG_24487 | sp\|P50991\|TCPD_HUMAN | tr\|A0A024R261\|A0A024R261_HUMAN | 463 |
| CCT4 | MDH2 | sp\|P50991\|TCPD_HUMAN | tr\|Q6FHZ0\|Q6FHZ0_HUMAN | 494 |
| CCT4 | FARSB | sp\|P50991\|TCPD_HUMAN | tr\|Q9BR63\|Q9BR63_HUMAN | 463 |
| CCT4 | HEL-S-89n | sp\|P50991\|TCPD_HUMAN | tr\|V9HWB4\|V9HWB4_HUMAN | 785 |
| CCT4 | PSMA4 | sp\|P50991\|TCPD_HUMAN | sp\|P25789\|PSA4_HUMAN | 437 |
| CCT4 | HEL-S-70 | sp\|P50991\|TCPD_HUMAN | tr\|V9HW80\|V9HW80_HUMAN | 428 |
| CCT4 | PSMD4 | sp\|P50991\|TCPD_HUMAN | sp\|P55036\|PSMD4_HUMAN | 444 |
| CCT4 | HSPA9 | sp\|P50991\|TCPD_HUMAN | tr\|Q8N1C8\|Q8N1C8_HUMAN | 787 |
| FLJ00064 | DLD | tr\|B4DV28\|B4DV28_HUMAN | tr\|A0A024R713\|A0A024R713_HUMAN | 583 |
| FLJ00064 | RPLP2 | tr\|B4DV28\|B4DV28_HUMAN | tr\|A0A024RCA7\|A0A024RCA7_HUMAN | 495 |
| KPNB1 | RPS27A | sp\|Q14974\|IMB1_HUMAN | tr\|B2RDW1\|B2RDW1_HUMAN | 472 |
| KPNB1 | RANBP1 | sp\|Q14974\|IMB1_HUMAN | tr\|F6WQW2\|F6WQW2_HUMAN | 999 |
| KPNB1 | HEL-S-89n | sp\|Q14974\|IMB1_HUMAN | tr\|V9HWB4\|V9HWB4_HUMAN | 675 |
| SNRNP200 | DHX15 | sp\|O75643\|U520_HUMAN | sp\|O43143\|DHX15_HUMAN | 846 |
| SNRNP200 | SRSF1 | sp\|O75643\|U520_HUMAN | sp\|Q07955\|SRSF1_HUMAN | 970 |
| SNRNP200 | SNRPD2 | sp\|O75643\|U520_HUMAN | sp\|P62316\|SMD2_HUMAN | 997 |
| SNRNP200 | PTBP1 | sp\|O75643\|U520_HUMAN | sp\|P26599\|PTBP1_HUMAN | 910 |
| MYL6 | HEL-S-271 | tr\|G8JLA2\|G8JLA2_HUMAN | tr\|V9HW31\|V9HW31_HUMAN | 662 |
| hCG_24487 | RPL18 | tr\|A0A024R261\|A0A024R261_HUMAN | tr\|J3QQ67\|J3QQ67_HUMAN | 999 |
| hCG_24487 | RPS3 | tr\|A0A024R261\|A0A024R261_HUMAN | sp\|P23396\|RS3_HUMAN | 999 |
| hCG_24487 | RPS27A | tr\|A0A024R261\|A0A024R261_HUMAN | tr\|B2RDW1\|B2RDW1_HUMAN | 998 |
| hCG_24487 | HEL-S-123m | tr\|A0A024R261\|A0A024R261_HUMAN | tr\|V9HW26\|V9HW26_HUMAN | 713 |
| hCG_24487 | RPS8 | tr\|A0A024R261\|A0A024R261_HUMAN | tr\|Q5JR94\|Q5JR94_HUMAN | 999 |
| hCG_24487 | HEL-S-271 | tr\|A0A024R261\|A0A024R261_HUMAN | tr\|V9HW31\|V9HW31_HUMAN | 616 |
| hCG_24487 | RPLP2 | tr\|A0A024R261\|A0A024R261_HUMAN | tr\|A0A024RCA7\|A0A024RCA7_HUMAN | 993 |
| hCG_24487 | RPL13 | tr\|A0A024R261\|A0A024R261_HUMAN | tr\|Q6NZ55\|Q6NZ55_HUMAN | 999 |
| hCG_24487 | RPS7 | tr\|A0A024R261\|A0A024R261_HUMAN | sp\|P62081\|RS7_HUMAN | 998 |
| hCG_24487 | RPS19 | tr\|A0A024R261\|A0A024R261_HUMAN | tr\|Q8WVX7\|Q8WVX7_HUMAN | 999 |
| PSMC3 | RPS27A | tr\|A0A140VK42\|A0A140VK42_HUMAN | tr\|B2RDW1\|B2RDW1_HUMAN | 935 |
| PSMC3 | HEL-S-94n | tr\|A0A140VK42\|A0A140VK42_HUMAN | tr\|V9HW72\|V9HW72_HUMAN | 694 |
| PSMC3 | PSMA4 | tr\|A0A140VK42\|A0A140VK42_HUMAN | sp\|P25789\|PSA4_HUMAN | 998 |
| PSMC3 | HEL-S-271 | tr\|A0A140VK42\|A0A140VK42_HUMAN | tr\|V9HW31\|V9HW31_HUMAN | 449 |
| PSMC3 | PSMD4 | tr\|A0A140VK42\|A0A140VK42_HUMAN | sp\|P55036\|PSMD4_HUMAN | 999 |
| VDAC2 | RPS27A | tr\|A0A024QZN9\|A0A024QZN9_HUMAN | tr\|B2RDW1\|B2RDW1_HUMAN | 684 |
| VDAC2 | DLD | tr\|A0A024QZN9\|A0A024QZN9_HUMAN | tr\|A0A024R713\|A0A024R713_HUMAN | 414 |
| VDAC2 | HEL-S-123m | tr\|A0A024QZN9\|A0A024QZN9_HUMAN | tr\|V9HW26\|V9HW26_HUMAN | 616 |
| VDAC2 | MDH2 | tr\|A0A024QZN9\|A0A024QZN9_HUMAN | tr\|Q6FHZ0\|Q6FHZ0_HUMAN | 559 |
| VDAC2 | HEL-S-271 | tr\|A0A024QZN9\|A0A024QZN9_HUMAN | tr\|V9HW31\|V9HW31_HUMAN | 615 |
| VDAC2 | CANX | tr\|A0A024QZN9\|A0A024QZN9_HUMAN | sp\|P27824\|CALX_HUMAN | 597 |
| VDAC2 | ATP5H | tr\|A0A024QZN9\|A0A024QZN9_HUMAN | sp\|O75947\|ATP5H_HUMAN | 550 |
| HEL-S-89n | RPS3 | tr\|V9HWB4\|V9HWB4_HUMAN | sp\|P23396\|RS3_HUMAN | 787 |
| HEL-S-89n | STUB1 | tr\|V9HWB4\|V9HWB4_HUMAN | sp\|Q9UNE7\|CHIP_HUMAN | 524 |
| HEL-S-89n | TPI1 | tr\|V9HWB4\|V9HWB4_HUMAN | sp\|P60174\|TPIS_HUMAN | 413 |
| HEL-S-89n | HEL-S-123m | tr\|V9HWB4\|V9HWB4_HUMAN | tr\|V9HW26\|V9HW26_HUMAN | 747 |
| HEL-S-89n | HEL-S-271 | tr\|V9HWB4\|V9HWB4_HUMAN | tr\|V9HW31\|V9HW31_HUMAN | 496 |
| HEL-S-89n | LMNB1 | tr\|V9HWB4\|V9HWB4_HUMAN | sp\|P20700\|LMNB1_HUMAN | 496 |
| HEL-S-89n | HEL-S-94n | tr\|V9HWB4\|V9HWB4_HUMAN | tr\|V9HW72\|V9HW72_HUMAN | 866 |
| HEL-S-89n | HEL70 | tr\|V9HWB4\|V9HWB4_HUMAN | tr\|V9HWC0\|V9HWC0_HUMAN | 578 |
| HEL-S-89n | HEL-S-70 | tr\|V9HWB4\|V9HWB4_HUMAN | tr\|V9HW80\|V9HW80_HUMAN | 798 |
| HEL-S-89n | STOML2 | tr\|V9HWB4\|V9HWB4_HUMAN | sp\|Q9UJZ1\|STML2_HUMAN | 689 |
| HEL-S-89n | CANX | tr\|V9HWB4\|V9HWB4_HUMAN | sp\|P27824\|CALX_HUMAN | 937 |
| HEL-S-89n | PTBP1 | tr\|V9HWB4\|V9HWB4_HUMAN | sp\|P26599\|PTBP1_HUMAN | 950 |
| RPL13 | RPL18 | tr\|Q6NZ55\|Q6NZ55_HUMAN | tr\|J3QQ67\|J3QQ67_HUMAN | 999 |
| RPL13 | RPS3 | tr\|Q6NZ55\|Q6NZ55_HUMAN | sp\|P23396\|RS3_HUMAN | 999 |
| RPL13 | RPS27A | tr\|Q6NZ55\|Q6NZ55_HUMAN | tr\|B2RDW1\|B2RDW1_HUMAN | 999 |
| RPL13 | RPS7 | tr\|Q6NZ55\|Q6NZ55_HUMAN | sp\|P62081\|RS7_HUMAN | 999 |
| RPL13 | RPS8 | tr\|Q6NZ55\|Q6NZ55_HUMAN | tr\|Q5JR94\|Q5JR94_HUMAN | 999 |
| RPL13 | RPS19 | tr\|Q6NZ55\|Q6NZ55_HUMAN | tr\|Q8WVX7\|Q8WVX7_HUMAN | 996 |
| RPL13 | RPLP2 | tr\|Q6NZ55\|Q6NZ55_HUMAN | tr\|A0A024RCA7\|A0A024RCA7_HUMAN | 999 |
| RANBP1 | HEL-S-26 | tr\|F6WQW2\|F6WQW2_HUMAN | tr\|V9HWJ2\|V9HWJ2_HUMAN | 512 |
| RANBP1 | UBA2 | tr\|F6WQW2\|F6WQW2_HUMAN | tr\|B2RDF5\|B2RDF5_HUMAN | 664 |
| DRIP4 | DPP3 | tr\|Q4W4Y1\|Q4W4Y1_HUMAN | sp\|Q9NY33\|DPP3_HUMAN | 431 |
| DRIP4 | UBE1 | tr\|Q4W4Y1\|Q4W4Y1_HUMAN | tr\|A0A024R1A3\|A0A024R1A3_HUMAN | 540 |
| ATP5H | HEL-S-26 | sp\|O75947\|ATP5H_HUMAN | tr\|V9HWJ2\|V9HWJ2_HUMAN | 413 |
| ATP5H | DLD | sp\|O75947\|ATP5H_HUMAN | tr\|A0A024R713\|A0A024R713_HUMAN | 408 |
| ATP5H | HEL-S-123m | sp\|O75947\|ATP5H_HUMAN | tr\|V9HW26\|V9HW26_HUMAN | 998 |
| ATP5H | MDH2 | sp\|O75947\|ATP5H_HUMAN | tr\|Q6FHZ0\|Q6FHZ0_HUMAN | 741 |
| ATP5H | STOML2 | sp\|O75947\|ATP5H_HUMAN | sp\|Q9UJZ1\|STML2_HUMAN | 692 |
| ATP5H | HEL-S-271 | sp\|O75947\|ATP5H_HUMAN | tr\|V9HW31\|V9HW31_HUMAN | 999 |
| CANX | HEL-S-70 | sp\|P27824\|CALX_HUMAN | tr\|V9HW80\|V9HW80_HUMAN | 977 |
| CANX | HSPA9 | sp\|P27824\|CALX_HUMAN | tr\|Q8N1C8\|Q8N1C8_HUMAN | 813 |
| GDI2 | HEL-S-94n | tr\|B4DLV7\|B4DLV7_HUMAN | tr\|V9HW72\|V9HW72_HUMAN | 438 |
| GDI2 | SNRPD2 | tr\|B4DLV7\|B4DLV7_HUMAN | sp\|P62316\|SMD2_HUMAN | 419 |
| STUB1 | RPS27A | sp\|Q9UNE7\|CHIP_HUMAN | tr\|B2RDW1\|B2RDW1_HUMAN | 955 |
| STUB1 | HEL-S-70 | sp\|Q9UNE7\|CHIP_HUMAN | tr\|V9HW80\|V9HW80_HUMAN | 862 |
| STUB1 | NQO1 | sp\|Q9UNE7\|CHIP_HUMAN | sp\|P15559\|NQO1_HUMAN | 582 |
| STUB1 | PSMD4 | sp\|Q9UNE7\|CHIP_HUMAN | sp\|P55036\|PSMD4_HUMAN | 964 |
| DPP3 | DLD | sp\|Q9NY33\|DPP3_HUMAN | tr\|A0A024R713\|A0A024R713_HUMAN | 550 |
| TPI1 | RPS3 | sp\|P60174\|TPIS_HUMAN | sp\|P23396\|RS3_HUMAN | 478 |
| TPI1 | HEL-S-26 | sp\|P60174\|TPIS_HUMAN | tr\|V9HWJ2\|V9HWJ2_HUMAN | 420 |
| TPI1 | GLUD1 | sp\|P60174\|TPIS_HUMAN | tr\|E9KL48\|E9KL48_HUMAN | 585 |
| TPI1 | MDH2 | sp\|P60174\|TPIS_HUMAN | tr\|Q6FHZ0\|Q6FHZ0_HUMAN | 838 |
| TPI1 | HEL-S-271 | sp\|P60174\|TPIS_HUMAN | tr\|V9HW31\|V9HW31_HUMAN | 605 |
| TPI1 | HSPA9 | sp\|P60174\|TPIS_HUMAN | tr\|Q8N1C8\|Q8N1C8_HUMAN | 522 |
| DLD | GLUD1 | tr\|A0A024R713\|A0A024R713_HUMAN | tr\|E9KL48\|E9KL48_HUMAN | 447 |
| DLD | HEL-S-123m | tr\|A0A024R713\|A0A024R713_HUMAN | tr\|V9HW26\|V9HW26_HUMAN | 636 |
| DLD | MDH2 | tr\|A0A024R713\|A0A024R713_HUMAN | tr\|Q6FHZ0\|Q6FHZ0_HUMAN | 806 |
| DLD | HEL-S-271 | tr\|A0A024R713\|A0A024R713_HUMAN | tr\|V9HW31\|V9HW31_HUMAN | 540 |
| NAP1L4b | HSPA9 | tr\|B4DS05\|B4DS05_HUMAN | tr\|Q8N1C8\|Q8N1C8_HUMAN | 646 |
| PSMA4 | RPS27A | sp\|P25789\|PSA4_HUMAN | tr\|B2RDW1\|B2RDW1_HUMAN | 920 |
| PSMA4 | HEL-S-70 | sp\|P25789\|PSA4_HUMAN | tr\|V9HW80\|V9HW80_HUMAN | 790 |
| PSMA4 | PSMD4 | sp\|P25789\|PSA4_HUMAN | sp\|P55036\|PSMD4_HUMAN | 999 |
| HEL70 | DHX15 | tr\|V9HWC0\|V9HWC0_HUMAN | sp\|O43143\|DHX15_HUMAN | 426 |
| STOML2 | HEL-S-123m | sp\|Q9UJZ1\|STML2_HUMAN | tr\|V9HW26\|V9HW26_HUMAN | 919 |
| NQO1 | RPS19 | sp\|P15559\|NQO1_HUMAN | tr\|Q8WVX7\|Q8WVX7_HUMAN | 644 |
| HSPA9 | RPS3 | tr\|Q8N1C8\|Q8N1C8_HUMAN | sp\|P23396\|RS3_HUMAN | 416 |
| HSPA9 | HEL-S-94n | tr\|Q8N1C8\|Q8N1C8_HUMAN | tr\|V9HW72\|V9HW72_HUMAN | 774 |
| HSPA9 | HEL-S-70 | tr\|Q8N1C8\|Q8N1C8_HUMAN | tr\|V9HW80\|V9HW80_HUMAN | 411 |
| HSPA9 | HEL-S-123m | tr\|Q8N1C8\|Q8N1C8_HUMAN | tr\|V9HW26\|V9HW26_HUMAN | 477 |
| HSPA9 | MDH2 | tr\|Q8N1C8\|Q8N1C8_HUMAN | tr\|Q6FHZ0\|Q6FHZ0_HUMAN | 431 |
| HSPA9 | HEL-S-271 | tr\|Q8N1C8\|Q8N1C8_HUMAN | tr\|V9HW31\|V9HW31_HUMAN | 576 |
| DHX15 | SRSF1 | sp\|O43143\|DHX15_HUMAN | sp\|Q07955\|SRSF1_HUMAN | 752 |
| DHX15 | SNRPD2 | sp\|O43143\|DHX15_HUMAN | sp\|P62316\|SMD2_HUMAN | 603 |
| SRSF1 | SNRPD2 | sp\|Q07955\|SRSF1_HUMAN | sp\|P62316\|SMD2_HUMAN | 935 |
| SRSF1 | PTBP1 | sp\|Q07955\|SRSF1_HUMAN | sp\|P26599\|PTBP1_HUMAN | 980 |
| PAFAH1B1 | SNRPD2 | sp\|P43034\|LIS1_HUMAN | sp\|P62316\|SMD2_HUMAN | 537 |
| HEL-S-26 | FUBP1 | tr\|V9HWJ2\|V9HWJ2_HUMAN | tr\|B4E0X8\|B4E0X8_HUMAN | 471 |
| HEL-S-26 | GLUD1 | tr\|V9HWJ2\|V9HWJ2_HUMAN | tr\|E9KL48\|E9KL48_HUMAN | 944 |
| HEL-S-26 | MDH2 | tr\|V9HWJ2\|V9HWJ2_HUMAN | tr\|Q6FHZ0\|Q6FHZ0_HUMAN | 925 |
| SNRPD2 | PTBP1 | sp\|P62316\|SMD2_HUMAN | sp\|P26599\|PTBP1_HUMAN | 910 |
| MKI67 | KRT20 | tr\|A0A087WV66\|A0A087WV66_HUMAN | sp\|P35900\|K1C20_HUMAN | 410 |
| HEL-S-123m | RPL18 | tr\|V9HW26\|V9HW26_HUMAN | tr\|J3QQ67\|J3QQ67_HUMAN | 598 |
| HEL-S-123m | RPS3 | tr\|V9HW26\|V9HW26_HUMAN | sp\|P23396\|RS3_HUMAN | 893 |
| HEL-S-123m | MDH2 | tr\|V9HW26\|V9HW26_HUMAN | tr\|Q6FHZ0\|Q6FHZ0_HUMAN | 825 |
| HEL-S-123m | HEL-S-271 | tr\|V9HW26\|V9HW26_HUMAN | tr\|V9HW31\|V9HW31_HUMAN | 999 |
| GLUD1 | MDH2 | tr\|E9KL48\|E9KL48_HUMAN | tr\|Q6FHZ0\|Q6FHZ0_HUMAN | 906 |
| GLUD1 | HEL-S-271 | tr\|E9KL48\|E9KL48_HUMAN | tr\|V9HW31\|V9HW31_HUMAN | 456 |
| UBA2 | RPS27A | tr\|B2RDF5\|B2RDF5_HUMAN | tr\|B2RDW1\|B2RDW1_HUMAN | 783 |
| UBA2 | UBE1 | tr\|B2RDF5\|B2RDF5_HUMAN | tr\|A0A024R1A3\|A0A024R1A3_HUMAN | 866 |
| RPS8 | RPL18 | tr\|Q5JR94\|Q5JR94_HUMAN | tr\|J3QQ67\|J3QQ67_HUMAN | 999 |
| RPS8 | RPS3 | tr\|Q5JR94\|Q5JR94_HUMAN | sp\|P23396\|RS3_HUMAN | 999 |
| RPS8 | RPS27A | tr\|Q5JR94\|Q5JR94_HUMAN | tr\|B2RDW1\|B2RDW1_HUMAN | 996 |
| RPS8 | RPS7 | tr\|Q5JR94\|Q5JR94_HUMAN | sp\|P62081\|RS7_HUMAN | 999 |
| RPS8 | RPS19 | tr\|Q5JR94\|Q5JR94_HUMAN | tr\|Q8WVX7\|Q8WVX7_HUMAN | 999 |
| RPS8 | RPLP2 | tr\|Q5JR94\|Q5JR94_HUMAN | tr\|A0A024RCA7\|A0A024RCA7_HUMAN | 999 |
| HEL-S-94n | HEL-S-70 | tr\|V9HW72\|V9HW72_HUMAN | tr\|V9HW80\|V9HW80_HUMAN | 587 |
| HEL-S-94n | UBE1 | tr\|V9HW72\|V9HW72_HUMAN | tr\|A0A024R1A3\|A0A024R1A3_HUMAN | 437 |
| HEL-S-70 | PSMD4 | tr\|V9HW80\|V9HW80_HUMAN | sp\|P55036\|PSMD4_HUMAN | 845 |
| HEL-S-70 | UBE1 | tr\|V9HW80\|V9HW80_HUMAN | tr\|A0A024R1A3\|A0A024R1A3_HUMAN | 699 |
| RPS7 | RPL18 | sp\|P62081\|RS7_HUMAN | tr\|J3QQ67\|J3QQ67_HUMAN | 999 |
| RPS7 | RPS3 | sp\|P62081\|RS7_HUMAN | sp\|P23396\|RS3_HUMAN | 999 |
| RPS7 | RPS27A | sp\|P62081\|RS7_HUMAN | tr\|B2RDW1\|B2RDW1_HUMAN | 999 |
| RPS7 | PTBP1 | sp\|P62081\|RS7_HUMAN | sp\|P26599\|PTBP1_HUMAN | 561 |
| RPS7 | RPS19 | sp\|P62081\|RS7_HUMAN | tr\|Q8WVX7\|Q8WVX7_HUMAN | 999 |
| RPS7 | RPLP2 | sp\|P62081\|RS7_HUMAN | tr\|A0A024RCA7\|A0A024RCA7_HUMAN | 998 |
| PSMD4 | RPS27A | sp\|P55036\|PSMD4_HUMAN | tr\|B2RDW1\|B2RDW1_HUMAN | 997 |
| PSMD4 | UBE1 | sp\|P55036\|PSMD4_HUMAN | tr\|A0A024R1A3\|A0A024R1A3_HUMAN | 667 |
| RPL18 | RPS3 | tr\|J3QQ67\|J3QQ67_HUMAN | sp\|P23396\|RS3_HUMAN | 999 |
| RPL18 | RPS27A | tr\|J3QQ67\|J3QQ67_HUMAN | tr\|B2RDW1\|B2RDW1_HUMAN | 999 |
| RPL18 | HEL-S-271 | tr\|J3QQ67\|J3QQ67_HUMAN | tr\|V9HW31\|V9HW31_HUMAN | 655 |
| RPL18 | RPLP2 | tr\|J3QQ67\|J3QQ67_HUMAN | tr\|A0A024RCA7\|A0A024RCA7_HUMAN | 999 |
| RPL18 | RPS19 | tr\|J3QQ67\|J3QQ67_HUMAN | tr\|Q8WVX7\|Q8WVX7_HUMAN | 999 |
| RPS3 | RPS27A | sp\|P23396\|RS3_HUMAN | tr\|B2RDW1\|B2RDW1_HUMAN | 999 |
| RPS3 | HEL-S-271 | sp\|P23396\|RS3_HUMAN | tr\|V9HW31\|V9HW31_HUMAN | 739 |
| RPS3 | RPLP2 | sp\|P23396\|RS3_HUMAN | tr\|A0A024RCA7\|A0A024RCA7_HUMAN | 999 |
| RPS3 | RPS19 | sp\|P23396\|RS3_HUMAN | tr\|Q8WVX7\|Q8WVX7_HUMAN | 999 |
| RPS27A | RPLP2 | tr\|B2RDW1\|B2RDW1_HUMAN | tr\|A0A024RCA7\|A0A024RCA7_HUMAN | 998 |
| RPS27A | UBE1 | tr\|B2RDW1\|B2RDW1_HUMAN | tr\|A0A024R1A3\|A0A024R1A3_HUMAN | 815 |
| RPS27A | RPS19 | tr\|B2RDW1\|B2RDW1_HUMAN | tr\|Q8WVX7\|Q8WVX7_HUMAN | 999 |
| MDH2 | HEL-S-271 | tr\|Q6FHZ0\|Q6FHZ0_HUMAN | tr\|V9HW31\|V9HW31_HUMAN | 769 |
| RPLP2 | RPS19 | tr\|A0A024RCA7\|A0A024RCA7_HUMAN | tr\|Q8WVX7\|Q8WVX7_HUMAN | 998 |
|  |  |  |  |  |
