# Supplementary material for: EspF of Enterohemorrhagic Escherichia coli Enhances Apoptosis via Endoplasmic Reticulum Stress in Intestinal Epithelial Cells: An Isobaric Tags for Relative and Absolute Quantitation-Based Comparative Proteomic Analysis
Source: Front Microbiol. 2022 Jun 30;13:900919. doi: 10.3389/fmicb.2022.900919 (PMC9279134; doi:10.3389/fmicb.2022.900919)
Supplement: Supplementary file 5 [file Data_Sheet_1.docx]

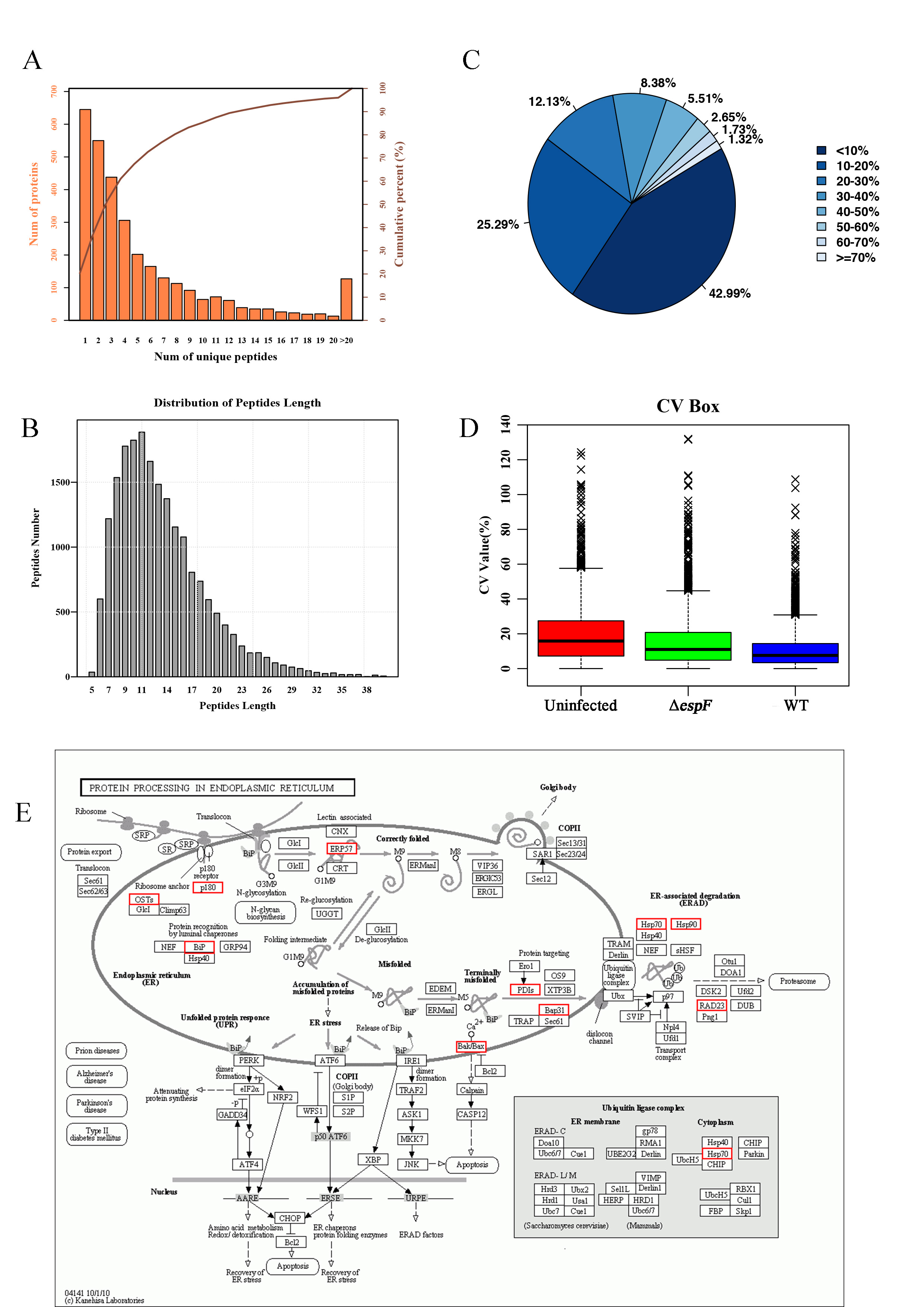


**Fig. S1a Distribution of unique peptides.** 3175 proteins were identified, of which 2530 (79.69%) proteins included at least two unique peptides. X-axis indicated the number of unique peptides. Left Y-axis indicated the number of proteins. Right Y-axis indicated the accumulative percent of proteins. **Fig. S1b Distribution of peptides length.** The peptide length of identified proteins mainly ranged from 7 aa to 20 aa. The most frequently occurring peptides were 11aa in length. X-axis showed the peptide length. Y-axis showed the number of peptides. **Fig.S1c Pie chart of proteins coverage percent (peptides with confidence≥95%).** The protein sequence coverage (95%) was estimated for specific proteins by the percentage of matching amino acids from the identified peptides having confidence higher than or equal to 95% divided by the total number of amino acids in the sequence, which have an average coverage of 25.15%.

**Fig.S1d The CV values** **of technical replicates.** The upper and lower sides of the box are the upper and lower quartile lines, and the black horizontal line in the middle of the box is the median of the data. **Fig.S1e The endoplasmic reticulum pathway from KEGG analysis within host cells.** The red box represented significantly up-regulated proteins after the WT infection, compared to the Δ*espF* group (*p*＜0.05).
